# N6-methyladenosine-modified ALDH9A1 modulates lipid accumulation and tumor progression in clear cell renal cell carcinoma through the NPM1/IQGAP2/AKT signaling pathway

**DOI:** 10.1038/s41419-024-06896-z

**Published:** 2024-07-22

**Authors:** Diaoyi Tan, Daojia Miao, Chuanyi Zhao, Jian Shi, Qingyang Lv, Feiyi Lu, Hailong Ruan, Zhiyong Xiong, Xiaoping Zhang

**Affiliations:** 1 Department of Urology, Union Hospital, Tongji Medical College, Huazhong University of Science and Technology, Wuhan, 430022 China; 2 Institute of Urology, Union Hospital, Tongji Medical College, Huazhong University of Science and Technology, Wuhan, 430022 China

**Keywords:** Renal cell carcinoma, Fatty acids, Lipid signalling

## Abstract

Aldehyde dehydrogenases superfamily (ALDHs), which are ubiquitously present in various organisms with diverse subcellular localizations, play a crucial role in regulating malignant tumor progression; Nevertheless, their involvement in clear cell renal cell carcinoma (ccRCC) has not been elucidated. In this study, we performed comprehensive bioinformatics analyses on the 19 ALDHs genes, and identified ALDH9A1 as a key contributor in ccRCC. Expression patterns and clinical relevance of ALDH9A1 were determined using bioinformatics analyses, real-time PCR, western blotting, and immunohistochemistry. To explore the underlying mechanism behind the tumor suppressor role of ALDH9A1, RNA sequencing, methylated RNA immunoprecipitation, luciferase reporter assay, mass spectroscopy, immunoprecipitation, mutational studies and immunofluorescence were employed. The impact of ALDH9A1 in ccRCC progression and metabolic programming was assessed through both in vitro and in vivo. Here, this study revealed ALDH9A1 as a tumor suppressor gene in ccRCC. The fat mass and obesity associated protein (FTO) was identified as a demethylase for *ALDH9A1* mRNA, resulting in its reduced stability and expression levels in ccRCC. Functional experiments demonstrated that the deficiency of ALDH9A1 in ccRCC promoted tumor proliferation, invasion, migration and lipid accumulation. Mechanistic insights illustrated that the diminished levels of ALDH9A1 resulted in the failure to sequester nucleophosmin 1 (NPM1) within cytoplasm, thereby suppressing the transcription of IQ motif containing the GTPase-activating protein 2 (IQGAP2), subsequently activating the AKT-mTOR signaling, ultimately fostering tumor progression and lipid accumulation. In conclusion, the present study highlights the robust prognostic significance of ALDH9A1 and delivers a comprehensive understanding of ALDH9A1-NPM1-IQGAP2-AKT axis in ccRCC. These findings established a solid research foundation for novel therapeutic strategies for ccRCC patients.

## Introduction

Kidney cancer is a prevalent malignancy in the urinary system, resulting in 179,368 global fatalities in 2020 [1]. Moreover, its incidence exhibits an annual upward trajectory [2]. Renal cell carcinoma (RCC) accounts for 90% of kidney cancers, with clear cell renal cell carcinoma (ccRCC) being the predominant subtype observed in around 80% of all RCC cases [3, 4]. At present, the treatment strategies for ccRCC patients primarily revolve around a combination of surgical intervention and medications such as targeted therapy and immune checkpoint inhibitor therapy [5]. However, progress in improving the disease-free survival rate remains relatively restricted, with advanced RCC posing a significant risk of mortality [5]. Hence, it is imperative to conduct comprehensive investigations into the molecular biology and genetics alternation of ccRCC, as well as elucidate the underlying mechanisms governing its occurrence and progression.

Metabolism reprogramming is widely acknowledged as a distinguishing feature of cancer [6]. In ccRCC, there exists a significant disruption in fatty acid (FA) metabolism [7], which results in the formation of lipid droplets and gives rise to a distinct histological phenotype [6]. Recent studies have revealed that lipid storage plays an essential role in tumor adaptation rather than being merely an incidental effect and altered metabolism is associated with poorer clinical prognosis in ccRCC patients since lipogenic genes trigger tumorigenesis [8]. Acquiring a deeper understanding of the mechanisms governing FA metabolism in ccRCC holds the potential to pave the way for innovative personalized treatment strategies.

Aldehyde dehydrogenase 9A1 (ALDH9A1) is one of the ALDHs gene superfamily, which consists of 19 putatively functional genes, oxidizing aldehydes to the corresponding carboxylic acids to maintain cellular homeostasis [9]. ALDHs have been discovered in almost all organisms with multiple subcellular localizations and have been involved in a series of cell functions, like proliferation, survival, and cellular response to oxidative stress [9]. It has been reported that the robust enzymatic activity exhibited by ALDHs represents a hallmark of cancer stem cells (CSC), with ALDH1 assuming a preeminent role [10]. The high expression of ALDHs in prostate cancers is associated with enhanced tumorigenicity and migratory potential [11]. In cholangiocarcinoma, ALDH3B2 has been identified as a key contributor to cell proliferation, migration, and tumor metastasis [12]. While there has been growing interest in understanding the involvement of ALDHs in tumorigenesis, limited studies have been conducted on the expression patterns between ccRCC tumors and peritumor tissues. Furthermore, the precise function of ALDHs in ccRCC is yet to be clarified.

The AKT and the mammalian target of rapamycin (mTOR) signaling pathways play a critical role in both physiological and pathological conditions. The two pathways are highly interconnected, to the extent that they could be considered as one unified pathway [13]. The signaling represents a sophisticated cellular response to various stimuli, such as nutrient intake, growth factor, and others, aiming to modulate downstream signaling events like promoting the translation necessary for cell growth and cell cycle [14]. Surprisingly, through its downstream effectors, P70S6 kinase (S6K), the AKT-mTOR pathway is involved in lipid metabolism by stimulating sterol regulatory element binding proteins (SREBP) isoforms and facilitating an SREBP-dependent rise in de novo lipogenesis and triacylglycerol (TG) synthesis [15]. Nowadays, very few drugs targeting the AKT-mTOR pathway have been approved for the treatment of human cancers in clinical trials. A comprehensive understanding of the AKT-mTOR pathway and associated lipid metabolism in ccRCC could enhance our knowledge of intricate cellular regulation, thereby establishing a robust foundation for novel therapeutic strategies.

In this study, we performed comprehensive bioinformatic investigations on the 19 ALDHs genes using the Cancer Genome Atlas Kidney Renal Clear Cell Carcinoma (TCGA-KIRC) and Clinical Proteomic Tumor Analysis Consortium (CPTAC)-PDC000127 cohorts and identified ALDH9A1 as a prominent player in ccRCC. Subsequently, experiments were conducted both in vitro and in vivo to confirm the efficacy of ALDH9A1 in ccRCC. Furthermore, overall investigations were carried out to elucidate the precise mechanism through which ALDH9A1 influenced the progression of ccRCC.

## Method

### Cell culture and tissue samples

All cell lines involved in this study, including human ccRCC cell lines (A498, 786-O, CAKI-1, and OSRC-2) and human embryonic kidney cell lines (HEK293 and HEK293T), were purchased from the American Type Culture Collection (ATCC, Manassas, VA, USA). Short tandem repeat profiling was conducted to authenticate the identity of these cell lines utilized in this study on Aug 14, 2021. The cells were cultured in Dulbecco’s modified eagle medium (DMEM, Gibco, Waltham, MA, USA), supplemented with 10% fetal bovine serum (Gibco) and 1% penicillin-streptomycin solution (#BL505A, Biosharp, Beijing, China), and maintained at a temperature of 37 °C in a standard humidified incubator with a CO_2_ concentration of 5%.

This research was conducted following the Declaration of Helsinki and approved by the Institutional Review Board of Huazhong University of Science and Technology (IEC-072). A total of 18 pairs of human ccRCC tissues and peritumor normal tissue were obtained from the Department of Urology at Wuhan Union Hospital (Wuhan, Hubei, China). The patients who underwent partial or total nephrectomy for ccRCC, or were diagnosed pathologically as ccRCC by two senior urological pathologists between January 2021 and January 2023 were included in this study. Patients with two or more primary malignancies or incomplete clinical information were excluded. All patients related to this study had written informed consent.

### RNA isolation and real-time PCR analysis

The Magzol reagent (#R4801, Magen Biotechnology Co., Ltd, Guangzhou, Guangdong, China) was used to extract total RNA from cells following the manufacturer’s instructions. The concentration and purity of the RNA solution were assessed by the NanoDrop 2000 spectrophotometer (NanoDrop Technologies, Wilmington, USA). One microgram of the isolated pure RNA was employed for the reverse transcription process via HiScript Il One-Step RT-PCR Kit (#R223, Vazyme BioTech, Nanjing, China), and SYBR qPCR master mix (#Q312, Vazyme BioTech) was conducted to perform quantitative PCR (qTOWER, Analytik Jena, Jena, Germany). Primer sequences are provided in (Supplementary Table 1).

### Western blotting

Proteins were isolated from ccRCC cell lines and patient tissues using RIPA buffer (#P0013C, Beyotime, Shanghai, China), supplemented with proteases and phosphatases inhibitors (#B14001, Beyotime). The protein samples were qualified using BCA assay kits (#23225, Thermo Fisher Scientific, MA, USA) and then subjected to heating at 100 °C for 10 minutes. A total of 30 μg proteins were employed for gel electrophoresis followed by transfer into polyvinylidene fluoride membranes (Roche, Basel, Switzerland). The membranes underwent primary antibody labeling and incubation in a blocking buffer containing secondary antibodies for 2 h before detection. Supplementary Table 1 provides the comprehensive list of antibodies utilized in this study.

### Immunohistochemistry

Fixation of patient-derived tissues and subcutaneous tumor models was performed with a 4% paraformaldehyde solution (#BL539A, Biosharp). Subsequently, the specimens underwent a series of steps including dehydration, paraffin embedding, sectioning, deparaffinization, and rehydration to facilitate antigen retrieval. The sections were subsequently treated with fetal bovine serum (Gibco) for 1 h to inhibit any non-specific binding. Following this, the tissue sections were exposed to primary antibodies overnight at a temperature of 4 degrees Celsius. The antibodies used in IHC are provided in Supplementary Table 1. The semiquantitative analysis of IHC was conducted by three pathologists who possessed extensive experience. They evaluated the staining intensity of the protein of interest using an IHC score, which ranged from 0 (indicating negative staining) to 3 (representing strong staining). Additionally, we assessed the percentage of positive cells, categorized as follows: 0% for a score of 0, 1–25% for a score of 1, 26–50% for a score of 2, and 51–75% for a score of 3. For scores above that range (76–100%), a value of 4 was assigned.

### Lentivirus and plasmid

The expression lentivirus for ALDH9A1 (NM_000696) along with its corresponding control vector was procured from Genechem (Shanghai, China). Specific IQGAP2 shRNA lentivirus along with its corresponding control vector were procured from GeneChem. A498 and CAKI-1 cells were transfected with lentivirus following guidelines outlined by manufacturers while puromycin (2 μg/mL, Sigma, Darmstadt, Germany) was used in established stable cell lines for further purification.

The FTO siRNA and ALDH9A1 siRNA were obtained from Genepharma Corporation (Shanghai, China). FTO, NPM1 and IQGAP2 overexpression plasmids were purchased from GeneChem Corporation. Cell lines were transfected according to the manufacturer’s instructions. The targeting sequences were provided in Supplementary Table S1. ALDH9A1-wt and ALDH9A1-mut (site873, site1330, and site1846) plasmids used in luciferase reporter assays were cloned into the GV306 vector (GeneChem).

### Colony formation assays

One well in 6-well plates was inoculated with a total of one thousand cells, which were subsequently incubated for 14 days. Following this, the colonies underwent multiple washes using phosphate-buffer saline (PBS), followed by fixation using methanol and staining with 0.05% crystal violet (#G1014, Servicebio, Wuhan, China). Finally, the stained colonies were utilized for visualization purposes.

### Cell viability assays

One well in 96-well plates was inoculated with a total of two thousand cells. CCK8 (#A311, Vazyme BioTech) with a concentration of 10% mixed with 100 μL DMEM (Gibco) was used to detect the cell viability. The absorbance value at 450 nm was measured using a spectrophotometer (NanoDrop Technologies) at time points: 0 h, 24 h, 48 h, 72 h and 96 h.

### Transwell assays

In the transwell migration and invasion assays, a density of 10^5^ ccRCC cells that were deprived of nutrients were introduced into the upper chamber (#REF3422, Corning Inc., NY, USA) either with (invasion assay) or without (migration assay) Matrigel (#356234, diluted 1:8, Corning Inc.), cultured with mediums lacking fetal bovine serum (Gibco). To attract the cells, 10% fetal bovine serum (Gibco) was supplemented to the lower chamber medium. After 24 h, cells that had invaded into the lower surface of the chamber membrane were fixed by methanol. Next, cells stained with 0.05% crystal violet (#G1014, Servicebio) were randomly photographed.

### Flow cytometry apoptosis assay

Cells were collected for flow cytometry analysis of cell apoptosis using Annexin V-Phycoerythrin (PE) and 7-amino-actinomycin D (7-AAD) staining (#A213-01, Vazyme BioTech). Different combinations of 7-AAD and Annexin V can identify apoptosis at different stages. The obtained data was processed with FlowJo software from Becton Dickinson, Franklin Lakes, NJ, USA.

### Animal models

All animal studies were approved by the Institutional Animal Use and Care Committee of Tongji Medical College (#3693). Male BALB/c nude mice aged 5 to 6 weeks were procured from Vital River Laboratories (Beijing, China) and kept in a pathogen-free setting. Each nude mouse was assigned a random number, and their placement into experimental groups was determined by the sequence of these random numbers.

For the subcutaneous tumor models, five mice were designated as one signal experimental group. A total of 2 × 10^6^ tumor CAKI-1 cells resuspended in 100 μl PBS were injected into one side of the posterior flanks of each nude mice. The growth of tumors was monitored every 4 days by measuring their length and width. Tumor volumes were calculated using the formula: volume = 0.52 × length × (width)^2^. On day 44 or when the tumor size exceeded 1.5 cm in diameter, mice were humanely euthanized using CO_2_ and cervical dislocation. The tumors were then dissected, photographed, and weighed.

For the metastasis model, three mice were designated as one signal experimental group. A total of 2 × 10^6^ tumor A498 cells suspended in 100 uL PBS were administered intravenously via the tail vein to assess the metastatic potential. Eight weeks later, imaging was performed using the LagoX system (Spectral Instruments Imaging, Tucson, AZ, USA) followed by the sacrifice of the mice. Subsequently, the liver specimens were excised, imaged, and paraffin-embedded.

In the animal study, blinding was used to reduce biases in data collection and analysis. The investigator was blind to the group assignments while conducting and evaluating the experiment.

### Oil red staining

Cells at a confluency level of 30–40% were seeded onto 12-well plates and fixed using 4% paraformaldehyde for 15 min. Subsequently, the plates underwent two rinses with PBS for 10 min, followed by staining with Oil red (#G1015, Servicebio) for approximately 2 hours. After staining, the plates were washed three times with water. Finally, random microscopic images of the cells in the 12-well plate were captured using a microscope (#DSZ2000, UOP Photo-electric Technology). For tissue analyses, tumor xenografts were snap-frozen in O.C.T. Compound (Sakura Finetek) and then sliced using a cryostat. The sections with a thickness of 10 µm were air-dried overnight at room temperature, followed by water washing and rinsing with 60% isopropanol. Subsequently, the sections were stained with Oil Red O solution for 15 min.

### Triglyceride (TG) detection

A total of 100 μL of 2% TritonX-100 solution (#P0096, Beyotime) was used to lyse the cell which was collected from a 6 cm dish with a confluency level of 90% or 0.1 g of the subcutaneous tumor was employed for this purpose for 40 min. Subsequently, 2.5 μL of the aforementioned product was added to a 250 μL working solution from the Triglyceride assay kit (#A110-1-1, Jiancheng, Nanjing, Jiangsu, China). The mixture was then incubated at 37 °C for 10 min. Simultaneously, 2.0 μL of the aforementioned product was added for protein quantification.

### Methylated RNA immunoprecipitation (MeRIP)-qPCR

Total RNA was extracted from tissue samples and ccRCC cells by the Magzol Reagent (#R4801, Magen Biotechnology Co., Ltd). Following mRNA purification via Mag-MK mRNA Purification Kit (#B518710-0010, Sangon Biotech, Shanghai, China), a small portion (approximately 1/10) was saved as input control. The remaining sample was incubated with anti-m^6^A antibody for 2 h and then mixed with pre-washed Protein A/G Magnetic Beads (#HY-K0202, MedChemExpress, Monmouth Junction, NJ, USA) in immunoprecipitation buffer at 4 °C overnight, supplemented with 100 U RNase inhibitor (#R0102, Beyotime). After washing four times with MeRIP buffer, the MeRIP portion was used to extract MeRIP mRNA by Magzol and ethanol. The relative mRNA expression was evaluated by the number of amplification cycles (Cq).

### RNA stability assays

ccRCC cells were seeded in 6-well plates at 50% confluence. After overnight incubation, the cells were exposed to Act D (#HY-17559, MedChemExpress) at a final concentration of 5 μg/mL for different durations of time (0, 2, 4, 6, 8, and 12 h), with Dimethyl sulfoxide (#196005, Aladdin, Shanghai, China) was used as the control treatment. Total RNA was extracted using Magzol Reagent (#R4801, Magen Biotechnology Co., Ltd), followed by RT-PCR analysis. The half-life of the mRNA was estimated based on the Cq value.

### Luciferase reporter assay

Luciferase assay was carried out on the third day post-transfection cell plasmid transfection, following the guidelines provided by the dual-luciferase reporter assay system (Promega, Madison, WI, USA). The cells were seeded into 24-cell plates, and wild-type and/or mutant reporter constructs were transfected in the HEK293T, A498, and CAKI-1 cells using lipofectamine 8000 (#C0533, Beyotime). The luciferase activity was measured using a multimode plate reader (EnSpire, Perkin Elmer, Waltham, MA, USA). By utilizing renilla luciferase activity as an internal reference, the ratio between firefly luciferase activity and renilla luciferase activity can serve as an indicator of reporter gene activation.

### RNA-sequencing (RNA-seq) analysis

Techniques and methods for whole-transcriptome sequencing after ALDH9A1 stable overexpression were provided by Bioyi Biotechnology Co., Ltd (Wuhan, Hubei, China). RNA samples were isolated from 3 groups of ALDH9A1-overexpression CAKI-1 cells and CAKI-1 cells carried vector lentivirus as control using Magzol Reagent Magzol Reagent (#R4801, Magen Biotechnology Co., Ltd). Then RNA samples were qualified and quantified for mRNA purification. Following fragmentation using divalent cations, PCR was employed to amplify the mRNA. The resulting PCR products were utilized for constructing a library. Ultimately, the sequencing library was sequenced on DNBSEQ-T7 (Making Great Innovation, Shenzhen, Guangdong, China) with the PE150 model as per the manufacturer’s protocol.

### Co-immunoprecipitation (Co-IP)

The cell lysate obtained from ccRCC cells was subjected to incubation in Co-IP buffer (#HY-K0202, MedChemExpress) containing primary antibodies or IgG overnight at 4 °C with agitation, in which the immune complexes were exposed to protein A/G magnetic beads. Subsequently, a wash step using RIPA (#P0013C, Beyotime) was performed to remove unbound immune complexes. The dissociated immune complexes bound to the beads were utilized for conducting western blotting assays.

### Shotgun liquid chromatography-mass spectrometry/mass spectrometry (LC-MS/MS)

The lysate of HEK293T cells, which were stably transfected with ALDH9A1-flag lentivirus and vector virus, was incubated with Co-IP buffer (#HY-K0202, MedChemExpress) overnight at 4°C with shaking, along with primary anti-flag antibodies. Subsequently, a wash step using RIPA (#P0013C, Beyotime) was performed to remove unbound immune complexes. The bound immune complexes were then subjected to shotgun liquid chromatography-mass spectrometry/mass spectrometry (LC-MS/MS) analysis by Genechem. The contract number is M-GSGC0378683. Database searches were performed using Proteome Discoverer 2.2 (Thermo Fisher Scientific) and MASCOT 2.6 (Matrix Science) software tools.

### Protein-protein docking

To decifer the interaction domain between ALDH9A1 and NPM1, the docking of the NPM1 domain with ALDH9A1 was carried out. The prediction of NPM1-ALDH9A1 interaction was carried out using ZDOCK software with default parameters [16]. ALDH9A1 protein data bank files (UniProt ID: A0A7K6F5U8) were input as receptors, and NPM1 protein data bank files (UniProt ID: A0A851XE84) were input as ligands. The top-ranked prediction was downloaded and then applied to PDBePISA [17] for exploration of the macromolecular interface and pymol software for visualization.

### Subcellular fractionation

The nuclear and cytoplasmic protein extraction kit (#P0028, Beyotime) was utilized to isolate the cytosolic fraction and nuclear fraction using the guidelines provided by the manufacturer.

### Chromatin immunoprecipitation assay (ChIP)

The ChIP assay was performed with the SimpleChIP Enzymatic Chromatin IP Kit (Agarose Beads) from Cell Signaling Technology (#9002, Boston, USA), following the manufacturer’s instructions. Immunoprecipitation was conducted using anti-NPM1 antibody (Proteintech, #60096-1-lg, Hubei, China) or normal IgG (#2729, Cell Signaling Technology). Quantitative PCR (qPCR) was employed to investigate the potential DNA-binding sites of NPM1 on *IQGAP2* promoter. Enrichment levels were normalized against the IgG control. Details of the primer sequences and antibodies are provided in Supplementary Tables 1, 2.

### Bioinformatics and statistical analyses

In this study, R version 4.2.3 (Bell Laboratories, Lucent Technologies) and GraphPad Prism 9.0 (Boston, USA) were used for bioinformatics and statistical analyses.

The TCGA-KIRC cohort and their matching clinical characteristics were retrieved from the TCGA database. The FPKM format of the TCGA-KIRC data was subsequently transformed into TPM due to the similarity between TPM values and microarray values [18]. Proteomics datasets were downloaded via the CPTAC data portal (https://pdc.cancer.gov/pdc/browse). Differential expression analyses were conducted using the TCGA-KIRC cohort and in-house RNA-Seq data via the “edgeR” package [19]. Differentially expressed genes were identified based on criteria of *P*-value < 0.05 and |Log fold-change | > 1. Univariate and multivariant Cox regression analyses were performed via “survival” package based on the TCGA-KIRC cohort to identify significant genes associated with overall survival. The “survminer” package was applied to perform Kaplan-Meier analyses to assess the survival differences between the high-expression and low-expression subgroups based on the TCGA-KIRC cohort. Correlation analyses were performed and visualized using the “corrplot” package, with the “spearman” method specified. The GSVA function from “GSVA” R package [20], along with the “KEGG_RENAL_CELL_CARCINOMA” gene set from the Molecular Signatures Database (MSigDB), was used to assess the variation in gene set enrichment across the samples in the TCGA-KIRC cohort. In conjunction with correlation analysis, the goal here is to identify the gene most closely related with the characteristics of renal cell carcinoma. Functional enrichment analysis was conducted via Gene Set Enrichment Analysis (GSEA) software using TCGA-KIRC cohort and in-house RNA-seq data following the default parameters. The reference gene sets “h.all.v7.2.symbols.gmt” and “c2.cp.kegg.v6.2.symbols.gmt” were used. Additionally, the cDNA sequence of ALDH9A1 was analyzed via SRAMP (www.cuilab.cn/sramp) to predict potential m^6^A modification sites based on sequence information [21]. The Venn diagrams presented in this paper were created using the online tool available at www.ehbio.com/test/venn website.

Continuous variables were organized using the mean and standard error (SD). To compare two subgroups of normally distributed variables, the student’s t-test was employed, while for non-normally distributed variables, the Mann-Whitney U test was used. For comparing means among three or more groups, one-way analysis of variance (ANOVA) with Tukey’s multiple comparisons test was utilized. A two-sided statistical test was consistently conducted, with a minimum sample size of *n* ≥ 3 was considered. The log-rank test was applied to examine potential survival differences among two or more groups in the Kaplan-Meier analysis. All results involved represented at least three independent experiments (*P < 0.05, **P < 0.01, ***P < 0.001).

## Result

### ALDH9A1, identified as a prognostic factor of ccRCC, was downregulated in ccRCC

**Fig. 1:**
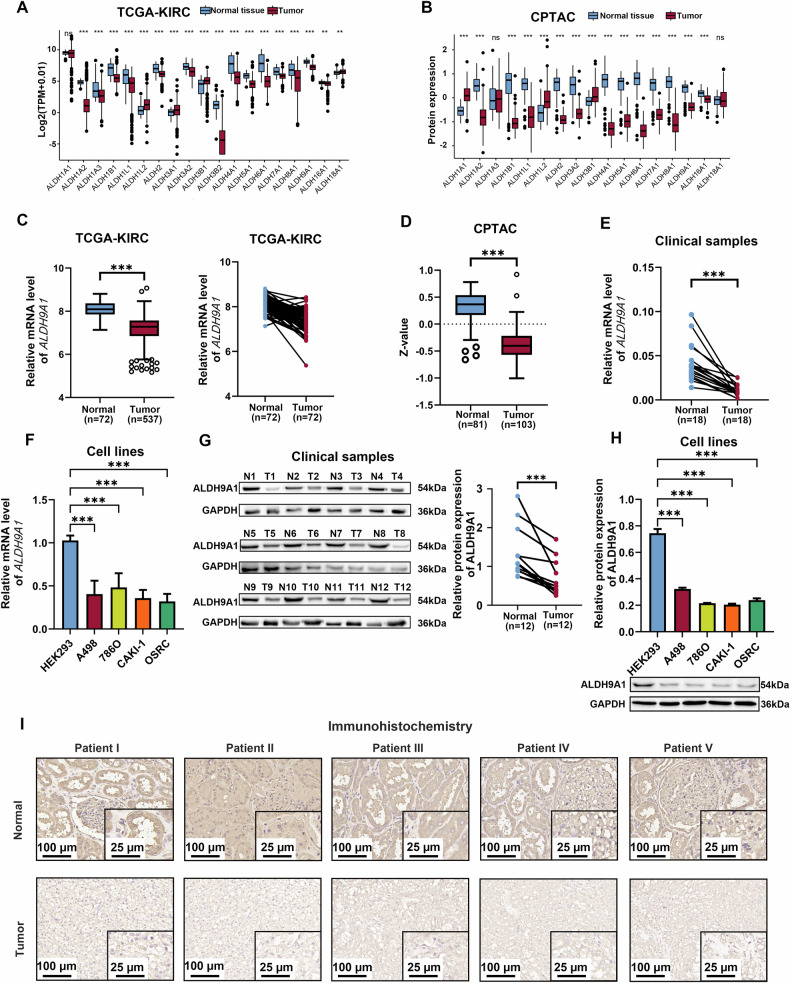
ALDH9A1 was downregulated in ccRCC and identified as a prognostic factor. **A** The mRNA levels of 19 genes from ALDHs family in ccRCC tissues and normal tissues based on TCGA-KIRC cohort (independent-samples *t*-test for statistics). The gene-level transcription estimates were shown in a form of log2 (TPM + 0.01). **B** The protein expression of 17 genes from ALDHs family in ccRCC tissues and normal tissues based on CPTAC-PDC000127 cohort (independent-samples *t*-test for statistics). ALDH3A1 and ALDH3B2 was not shown in the Fig. due to their absence in CPTAC-PDC000127 cohort. **C** The mRNA levels of *ALDH9A1* in ccRCC tissues and normal tissues based on TCGA-KIRC cohort (independent-samples *t*-test and paired-samples *t-*test for analysis). **D** The protein expression of ALDH9A1 in ccRCC tissues and normal tissues based on CPTAC-PDC000127 cohort (independent-samples *t*-test for statistics). **E** The mRNA levels of *ALDH9A1* in 18 pairs ccRCC tissues and corresponding normal tissues (paired-samples *t-*test for statistics). **F** The mRNA levels of *ALDH9A1* in ccRCC cell lines and a normal cell line (*n* = 3) (ANOVA for statistics). **G** The protein expression of ALDH9A1 in 12 pairs ccRCC tissues and corresponding normal tissues (paired-samples *t-*test for statistics). **H** The protein expression of ALDH9A1 in ccRCC cell lines and a normal cell line (*n* = 3) (ANOVA for statistics). **I** Representative IHC staining for ALDH9A1 in 5 pairs of ccRCC tissues and peritumor normal tissues. The tissues were obtained from randomly selected tissue samples, ensuring an equal number of paired tumor tissue and adjacent tissue derived from the same patient. Results represented at least three independent experiments (*P < 0.05, **P < 0.01, ***P < 0.001).

By mining the data from TCGA-KIRC and CPTAC-PDC000127 cohorts, we discovered the differential expression patterns among the 19 ALDHs genes, only ALDH1L2, ALDH3A1 and ALDH3B1 exhibited upregulation in ccRCC, while other 13 molecules, namely ALDH1A2, ALDH1B1, ALDH1L1, ALDH2, ALDH3A2, ALDH3B2, ALDH4A1, ALDH5A1, ALDH6A1, ALDH7A1, ALDH8A1, ALDH9A1 and ALDH16A were found to be significantly downregulated (Fig. 1A, B and Supplementary Fig. 1A, B). To screen out genes with greater clinical significance, we meticulously examine the association between these 16 genes and survival outcomes using both univariant Cox regression and Kaplan-Meier analysis (Table 1). According to the findings, a total of 7 molecules, namely ALDH1L1, ALDH1L2, ALDH3A2, ALDH6A1, ALDH7A1, ALDH9A1, and ALDH16A1 met the criterion of *P* lower than 0.01 and demonstrated statistical significance in relation to overall survival (OS) among ccRCC patients. To further investigate the relationship between ALDHs and renal cell carcinoma, we applied the gene set variation analysis (GSVA) algorithm to calculate the abundance scores of the KEGG_RENAL_CELL_CARCINOMA pathway based on the TCGA-KIRC cohort and utilized them with the aforementioned 7 ALDHs molecules for correlation analyses. As illustrated in Supplementary Fig. 1C, ALDH9A1 exhibits the strongest correlation with renal cell carcinoma among the 7 prognostically significant ALDHs. Notably, Henrion et al. proposed that ALDH9A1 was implicated in the development of RCC through comprehensive genome-wide meta-analyses [22]. Therefore, ALDH9A1 was proposed as the core gene to investigate the role of the ALDHs in ccRCC.

**Table 1:** Univariate Cox regression and Kaplan-Meier analyses were performed on a panel of 16 ALDH genes to assess their association with overall survival.

Gene ID	univariant COX analyses					Kaplan-Meier analyses		
	HR	lower	upper	95%CI	*p*-valule	mean value	HR	*p*-valule
ALDH16A1	1.0338	1.01563	1.0523	1.01563–1.0523	0.00024	25.5131328	1.781021	0.000152
ALDH1A2	0.9965	0.98797	1.0051	0.98797–1.0051	0.42349	7.99563726	1.190099	0.411831
ALDH1B1	0.99795	0.99468	1.00124	0.99468–1.00124	0.2216	62.7421378	0.597825	0.005473
ALDH1L1	0.98462	0.97789	0.9914	0.97789–0.9914	0.00001	31.4740116	0.47813	9.02E-06
ALDH1L2	1.04627	1.02988	1.06293	1.02988–1.06293	< 0.00001	3.7179477	1.757524	0.000336
ALDH2	0.9953	0.99075	0.99987	0.99075–0.99987	0.04401	77.0450267	0.659116	0.007678
ALDH3A2	0.99099	0.9881	0.99388	0.9881–0.99388	< 0.00001	106.798136	0.423692	1.48E-07
ALDH3B1	1.00401	0.99615	1.01192	0.99615–1.01192	0.31834	37.2037745	0.938727	0.681019
ALDH4A1	0.99901	0.99684	1.00119	0.99684–1.00119	0.37481	72.0128257	1.069066	0.677833
ALDH5A1	0.99002	0.9789	1.00126	0.9789–1.00126	0.0816	27.3960977	0.75332	0.078265
ALDH6A1	0.98114	0.97456	0.98776	0.97456–0.98776	< 0.00001	41.7626197	0.358698	2.20E-08
ALDH7A1	0.98202	0.97473	0.98937	0.97473–0.98937	< 0.00001	59.5580922	0.487562	6.38E-06
ALDH8A1	0.99585	0.99322	0.99848	0.99322–0.99848	0.00201	67.1854366	0.651409	0.010118
ALDH9A1	0.99485	0.99208	0.99764	0.99208–0.99764	0.00029	158.146479	0.61061	0.001469
ALDH3A1	1.065278	1.005857	1.128208	1.00585–1.12820	0.030819	1.83945508	1.375073	0.03882
ALDH3B2	1.127907	0.93488	1.360788	0.93488–1.36078	0.208814	0.18875657	1.609035	0.006821

(A) The analyses were based on the expression file derived from the TCGA-KIRC cohort and corresponding patient survival data.

(B) *P*-values lower than 0.01 was considered statistically significant. Log-rank test was conducted for hypothesis testing in Kaplan-Meier analyses.

(C) The hazard ratio was calculated using the Cox proportional hazard regression model.

(D) The confidence interval (CI) presents the estimated HR.

The expression of ALDH9A1 was remarkably diminished in ccRCC samples compared with normal tissues at both mRNA level and protein expression, as we mentioned before (Fig. 1C, D). The proteomics data from CPTAC further revealed a pronounced decrease in ALDH9A1 expression in ccRCC compared to other tumor types (Supplementary Fig. 1D). The expression of *ALDH9A1* at mRNA levels was validated in clinical ccRCC samples and ccRCC cell lines using the quantitative real-time polymerase chain reaction (qRT-PCR) analysis (Fig. 1E, F). Western blotting was further conducted to confirm the reduced protein expression of ALDH9A1 in ccRCC utilizing 12 pairs of clinical ccRCC samples as well as their corresponding peritumor normal kidney tissues, and a panel of ccRCC cell lines (Fig. 1G, H). Immunohistochemistry (IHC) assays provided further evidence for the decreased expression of ALDH9A1 in ccRCC (Fig. 1I). The bioinformatics analyses revealed a significant correlation between the downregulation of ALDH9A1 and higher TNM stage as well as more advanced histologic grades in ccRCC (Table 2 and Supplementary Fig. 1E–H). The multivariate Cox analyses revealed a strong association between ALDH9A1 and the inferior prognosis, and after adjusting for gender, age, stage, and grade, ALDH9A1 remained an accurate and independent predictor of outcomes in patients with ccRCC (Table 3). In conclusion, the expression of ALDH9A1 was declined in ccRCC, and decreased ALDH9A1 was positively related with poor survival in ccRCC patients.

**Table 2:** Correlation between *ALDH9A1* mRNA expression and clinicopathological parameters of patients with ccRCC.

Parameter	Number	*ALDH9A1* mRNA expression		*P-* value
		Low (n = 262)	High (*n* = 262)	
Age (years)				
< 65	329 (62.79)	164 (62.60)	165 (62.98)	1
≥ 65	195 (37.21)	98 (37.40)	97 (37.02)	
Gender				
Male	342 (65.27)	151 (57.63)	191 (72.90)	0.0003
Female	182 (34.73)	111 (42.37)	71 (27.10)	
TNM stage				
I + II	317 (60.50)	177 (67.56)	140 (53.44)	0.0021
III + IV	205 (39.12)	85 (32.44)	120 (45.80)	
Unknown	2 (0.38)	0 (0.00)	2 (0.76)	
M stage				
M0	416 (79.39)	217 (82.82)	199 (75.95)	0.1477
M1	78 (14.89)	32 (12.21)	46 (17.56)	
Unknown	30 (5.73)	13 (4.96)	17 (6.49)	
N stage				
N0	239 (45.61)	121 (46.18)	118 (45.04)	0.0394
N1	16 (3.05)	3 (1.15)	13 (4.96)	
Unknown	269 (51.34)	138 (52.67)	131 (50.00)	
T stage				
T1 + T2	335 (63.93)	183 (69.85)	152 (58.02)	0.0063
T3 + T4	189 (36.07)	79 (30.15)	110 (41.98)	
G grade				
G1 + G2	237 (45.23)	131 (50.00)	106 (40.46)	0.0807
G3 + G4	279 (53.24)	128 (48.85)	151 (57.63)	
Unknown	8 (1.53)	3 (1.15)	5 (1.91)	
Overall Survival (OS)				
Alive	351 (66.98)	192 (73.28)	159 (60.69)	0.003
Dead	173 (33.02)	70 (26.72)	103 (39.31)	
Disease-Specific Survival (DSS)				
NO	405 (77.29)	220 (83.97)	185 (70.61)	0.001
YES	108 (20.61)	37 (14.12)	71 (27.10)	
Unknown	11 (2.10)	5 (1.91)	6 (2.29)	
Progression-Free Interval (PFI)				
NO	365 (69.66)	198 (75.57)	167 (63.74)	0.0044
YES	159 (30.34)	64 (24.43)	95 (36.26)	

(A) Overall survival (OS) refers to the duration from diagnosis (or initiation of treatment) until death, while disease-specific survival (DSS) is defined as death specifically caused by the diagnosed cancer type. Progression-free interval (PFI), on the other hand, denotes the time between diagnosis and recurrence or progression of the disease.

(B) The ALDH9A1 expression file and clinical information were obtained from the TCGA-KIRC cohort.

(C) The Chi-square test was utilized for analyses.

**Table 3:** Univariate and multivariate Cox regression analysis of *ALDH9A1* for overall survival based on the TCGA-KIRC cohort.

Variables	Univariate analyses			Multivariate analyses		
	HR	95%CI	*P*-value	HR	95%CI	*P*-value
Age (years)						
< 65						
≥ 65	1.66	1.23–2.23	0.001	1.33	0.88–2.01	0.173
Gender						
Female						
Male	0.95	0.7–1.3	0.758			
TNM stage						
I + II						
III + IV	3.83	2.79–5.26	< 0.001	1.19	0.47–2.99	0.718
M stage						
M0						
M1	4.29	3.14–5.87	< 0.001	2.64	1.56–4.45	< 0.001
N stage						
N0						
N1	3.43	1.82–6.46	< 0.001	1.36	0.68–2.75	0.385
T stage						
T1 + T2						
T3 + T4	3.14	2.32–4.26	< 0.001	1.51	0.66–3.43	0.328
G grade						
G1 + G2						
G3 + G4	2.63	1.87–3.7	< 0.001	1.64	0.99–2.7	0.054
ALDH9A1						
Low	1.71	1.26–2.32	0.001	2.04	1.3–3.2	0.002
High						

(A) Overall survival (OS) refers to the duration from diagnosis (or initiation of treatment) until death.

(B) Variables with *P*-values below 0.05 in univariate Cox regression were included in further multivariate Cox regression analyses.

(C) Hazard ratios were estimated using the Cox proportional hazards model.

(D) Clinical information was obtained from the TCGA-KIRC cohort.

(E) CI represents the confidence interval of the estimated hazard ratio.

### The downregulation of ALDH9A1 promoted the progression and lipid accumulation of ccRCC in vitro and in vivo

**Fig. 2:**
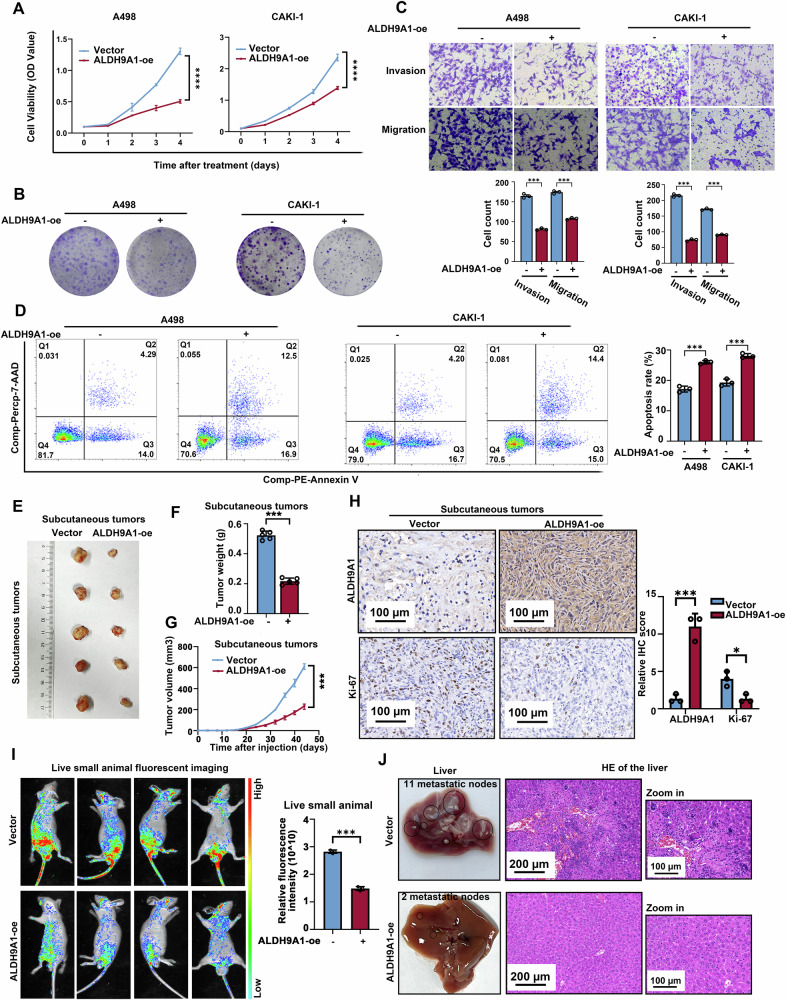
ALDH9A1 inhibited the progression of ccRCC in vitro and in vivo. **A** Cell proliferation curves of CCK8 assays for ALDH9A1-overexpression cells compared with the control group (*n* = 3) (independent-samples *t*-test for statistics). **B** Colony formation assays for ALDH9A1-overexpression cells compared with the control group (*n* = 3). **C** Transwell assay of the migration and invasion for ALDH9A1-overexpression cells compared with the control group (*n* = 3) (independent-samples *t*-test for statistics). **D** Flow cytometry assay determining the proportion of apoptotic cells in ALDH9A1-overexpressed ccRCC cells compared with control cells (*n* = 3) (independent-samples *t*-test for statistics). Comp-PE-Annexin V means that Annexin V was compensated by negative control and single positive control, and Comp-Percp-7-AAD means that 7-AAD was compensated by negative control and single positive control. **E**–**G** ALDH9A1-overexpression CAKI-1 cells were implanted into BALB/c nude mice for in vivo studies. The tumor size was monitored at 4-day intervals, with the last measurement was performed on day 44. Tumors were harvested and weighed after mice were euthanized (*n* = 5) (independent-samples *t*-test for statistics). **H** IHC staining and IHC score for ALDH9A1 and Ki67 in the isolated tumor xenografts from the ALDH9A1-overexpressed group compared with the control group (*n* = 3) (independent-samples *t*-test for statistics). **I** Live small animal fluorescent images of vein tail metastasis models in the ALDH9A1-overexpressed group compared with control group (*n* = 3) (independent-samples *t*-test for statistics). **J** H&E staining of the liver tissue from vein tail metastasis models in the ALDH9A1-overexpressed group compared with control groups (*n* = 3). Results represented at least three independent experiments (*P < 0.05, **P < 0.01, ***P < 0.001).

Given that the expression of ALDH9A1 notably impacted the outcomes of patients with ccRCC, this study then delved into the unseen mechanism of ALDH9A1 in ccRCC. Lentivirus and siRNAs were applied to construct ALDH9A1-overexpression and ALDH9A1-knockdown models in two regular ccRCC cell lines, A498 and CAKI-1. The efficiency of overexpression and knockdown of ALDH9A1 was confirmed via qRT-PCR and western blotting assays (Supplementary Fig. 2A–D). CCK8 assays demonstrated that the exogenous increased ALDH9A1 notably damped cell proliferation (Fig. 2A), in accordance with the results from the colony formation assays (Fig. 2B and Supplementary Fig. 2F), while the deficiency of ALDH9A1 remarkably enhanced the proliferation of ccRCC cells (Supplementary Fig. 2E). The migration and invasion abilities were damped upon forced expressing of ALDH9A1 in ccRCC cells (Fig. 2C), whereas ALDH9A1-deficient ccRCC cells displayed the opposite effect (Supplementary Fig. 2G–H). In addition, there was a significant increase in apoptotic cells observed in cells overexpressing ALDH9A1 (Fig. 2D), while a decrease in apoptotic cells was observed in cells with ALDH9A1 knockdown (Supplementary Fig. 2I, J). Aiming to assess the tumor suppressor role of ALDH9A1 in ccRCC in vivo, this study constructed subcutaneous tumor xenografts and tail vein metastasis models in BALB/c nude mice, with ALDH9A1-overexpressed CAKI-1 and A498 cells, respectively. The weight and volume of tumor xenografts generated by ALDH9A1-overexpression CAKI-1 cells were noticeably smaller than those generated by CAKI-1 cells bearing the empty vector (Fig. 2E–G). Immunohistochemical staining demonstrated that Ki-67, the index of cellular proliferation, was declined in the tumor xenografts induced by cells with overexpression of ALDH9A1 (Fig. 2H). Besides, the live small animal imaging manifested that increased ALDH9A1 significantly deadened tumor metastases (Fig. 2I), and the number of metastatic nodes in the liver from the ALDH9A1-overexpressed group was less than the control group (Fig. 2J and Supplementary Fig. 2K). Taken together, this study proposes ALDH9A1 as an ideal biomarker in ccRCC.

**Fig. 3:**
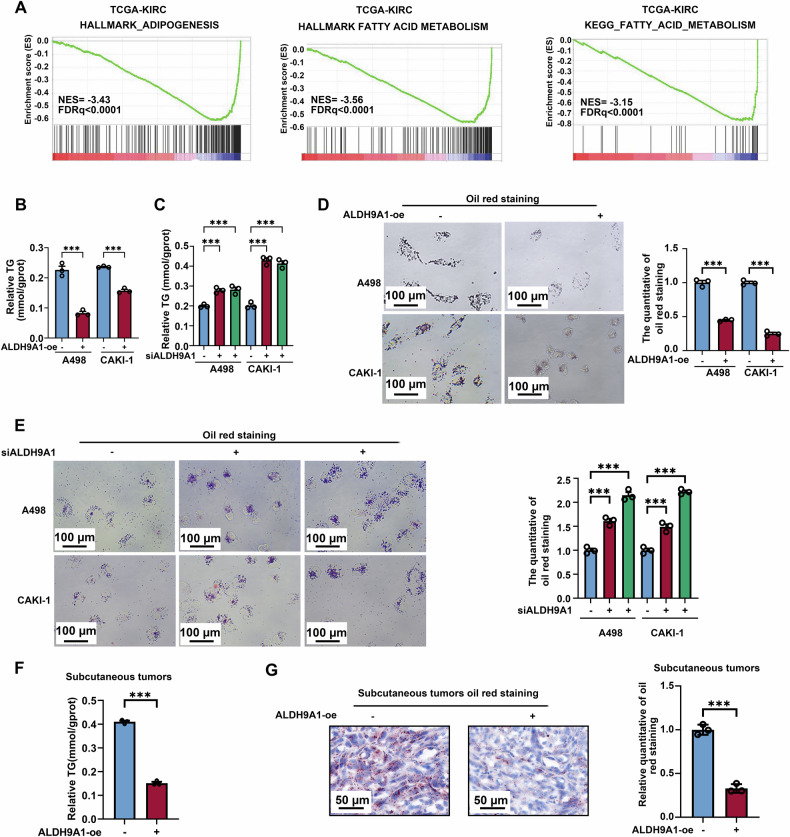
ALDH9A1 declined lipid accumulation in ccRCC. **A** Results from GSEA revealed the association between lipid metabolism and ALDH9A1 based on TCGA-KIRC cohort. Statistical significance was determined at FDRq < 25%. **B** Relative TG (mmol/gprot) levels in ALDH9A1-overexpressing cells compared with the control group (*n* = 3) (independent-samples *t*-test for statistics). **C** Relative TG (mmol/gprot) levels in ALDH9A1-deficienct cells (*n* = 3) compared with the control group (ANOVA for statistics). **D** Photomicrographs of Oil red staining of ALDH9A1-overexpressing cells compared with the control group (*n* = 3) (independent-samples *t*-test for statistics). **E** Photomicrographs of Oil red staining of ALDH9A1-deficient cells compared with the control group (*n* = 3) (ANOVA for statistics). **F** Relative TG (mmol/gprot) levels in isolated tumor xenografts from the ALDH9A1-overexpressed group compared with the control group (*n* = 3) (independent-samples *t*-test for statistics). **G** Photomicrographs of Oil red staining in isolated tumor xenografts from the ALDH9A1-overexpressed group compared with the control group (independent-samples *t*-test for statistics). Results represented at least three independent experiments (*P < 0.05, **P < 0.01, ***P < 0.001).

It is widely recognized that lipid droplets were the hallmark of ccRCC, thus this study then investigated if ALDH9A1 played a role in lipid metabolic reprogramming. Interestingly, the Gene Set Enrichment Analysis (GSEA) depicted that ALDH9A1 was highly related to adipogenesis and fatty acid metabolism (Fig. 3A). Triglyceride concentration detection indicated that A498 and CAKI-1 cells overexpressing ALDH9A1 showed lower TG levels compared with the control groups (Fig. 3B), while ALDH9A1-deficient A498 and CAKI-1 cells displayed the opposite results (Fig. 3C). Consistently, the oil red staining assays revealed that the lipid drop levels declined upon forced expression of ALDH9A1 (Fig. 3D), whereas the ALDH9A1 deficiency resulted in elevated lipid accumulation (Fig. 3E). Notably, the triglyceride concentration levels in the tumor xenografts from ALDH9A1-overexpressed groups were significantly mitigated compared to those from control groups (Fig. 3F), and the oil red staining demonstrated that the tumor xenografts from ALDH9A1-overexpressed groups owned less lipid accumulation compared with those from the control group (Fig. 3G). To sum up, these findings denoted that ALDH9A1 served as a potential regulator in lipid accumulation.

### FTO-mediated m^6^A demethylation was implicated in ALDH9A1 downregulation

To unveil the nonnegligible responsibility for the downregulation of ALDH9A1 in ccRCC, epigenetic modifications were taken into consideration, given their substantial significance in biomedical research [23]. DNA methylation is an essential aspect of epigenetic modification in genes [24]. Through the analyses of the promoter methylation profile derived from the ualcan website [25], we observed that both normal and tumor tissues exhibited extreme hypo-methylation in the promoters of ALDH9A1 (Supplementary Fig. 3A). Subsequently, the demethylating agent 5-AZA was added to the cells to clarify the impact of DNA methylation on the expression of ALDH9A1. The result indicated that 5-AZA did not affect ALDH9A1 in either normal and ccRCC cell lines, at both mRNA and protein levels (Supplementary Fig. 3B–D). Thus, it can be concluded that the downregulation of ALDH9A1 in ccRCC was independent of DNA methylation.

**Fig. 4:**
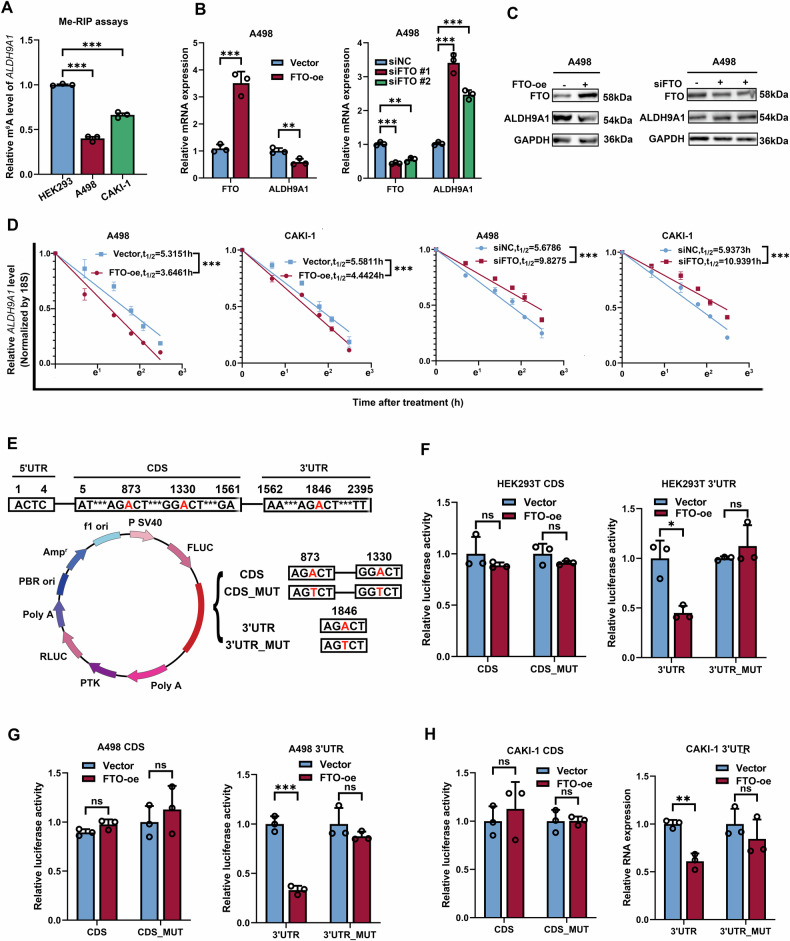
FTO-mediated m^6^A demethylation was implicated in ALDH9A1 downregulation in ccRCC. **A** The m^6^A modification levels of the mRNA of *ALDH9A1* in ccRCC cell lines and a normal cell line (*n* = 3) (ANOVA for statistics). **B** The mRNA levels of *ALDH9A1* in FTO-overexpression and FTO-deficient ccRCC cells, respectively, compared with the control group (*n* = 3) (independent-samples *t*-test and ANOVA for statistics). **C** The protein expression of ALDH9A1 in FTO-overexpression and FTO-deficient ccRCC cells, respectively, compared with the control group (*n* = 3). **D** The decay rate of *ALDH9A1* mRNA following the treatment with actinomycin D in FTO-overexpression and FTO-deficient ccRCC cells (*n* = 3) (independent-samples *t*-test for statistics). **E** A schematic diagram depicts wild-type *ALDH9A1* CDS, wild-type *ALDH9A1* 3′-UTR, as well as mutations of both *ALDH9A1* CDS and *ALDH9A1* 3′-UTR at the m^6^A modification sites. **F**–**H** Relative luciferase activity of the wild-type and mutant *ALDH9A1* CDS reporter vectors, as well as the wild-type and mutant *ALDH9A1* 3′-UTR reporter vectors in FTO-overexpression HEK293T, A498 and CAKI-1 cells, compared with the control group (*n* = 3) (independent-samples *t*-test for statistics). Results represented at least three independent experiments (*P < 0.05, **P < 0.01, ***P < 0.001).

The m^6^A modification, a newly predominant epigenetic modification these days, could control the expression of tumor-related genes to preserve the malignancy of the tumors [26]. Hence, we are intrigued as to whether the m^6^A modification played a role in the deficiency of ALDH9A1 in ccRCC. The presence of m^6^A modification in the mRNA of *ALDH9A1* was significantly lower in A498 and CAKI-1 cells compared to HEK293 cells through Me-RIP assays (Fig. 4A). The results also delineated that the m^6^A modification level of *ALDH9A1* from ccRCC tissues was significantly downregulated compared with adjacent normal tissues (Supplementary Fig. 4A). According to the prediction from the R2MTarget website [27], ALDH9A1 was a potential target gene of FTO in ccRCC (Supplementary Fig. 4B). FTO, the first m^6^A demethylase to be discovered, has been reported to function as an oncogene in ccRCC [28]. To validate the regulatory relationship between FTO and ALDH9A1, we forced and silenced the expression of FTO in A498 and CAKI-1 cells, respectively. The mRNA levels of *ALDH9A1* were upregulated upon the depletion of FTO and declined upon the overexpress of FTO (Fig. 4B and Supplementary Fig. 4C). Correspondingly, the protein expression of ALDH9A1 demonstrated a similar alteration pattern (Fig. 4C and Supplementary Fig. 4D, E). The RNA stability measurements exhibited that the half-time of *ALDH9A1* transcripts was weakened upon the reintroduction of FTO, while the deficiency of FTO resulted in prolonged half-time of *ALDH9A1* transcripts (Fig. 4D). The SRAMP server, which predicts mammalian m^6^A sites based on cDNA sequence features [21], has identified three credible m^6^A sites in *ALDH9A1* transcripts: site 873, site 1330, and site 1846 (Fig. 4E). Both site 873 and site 1330 were located within the coding sequence (CDS) of *ALDH9A1* transcripts, whereas site 1846 was situated in the 3’-untranslated region (UTR) (Fig. 4E). To screen out the precise position through which FTO affected the stability of *ALDH9A1* transcripts, mutant ALDH9A1 plasmids were established and applied for luciferase reporter assays (Fig. 4E). The results exhibited that FTO controls the stability of *ALDH9A1* mRNA via the site 1846 from 3’-UTR rather than site 873 and site 1330 from CDS, in HEK293T, A498, and CAKI-1 cells (Fig. 4F–H and Supplementary Fig. 4F–H). In summary, these findings indicated that FTO-mediated m^6^A demethylation lowered the expression of ALDH9A1 in ccRCC.

### The downregulation of ALDH9A1 activated AKT-mTOR signaling, accelerating tumor progression and lipid accumulation in ccRCC

**Fig. 5:**
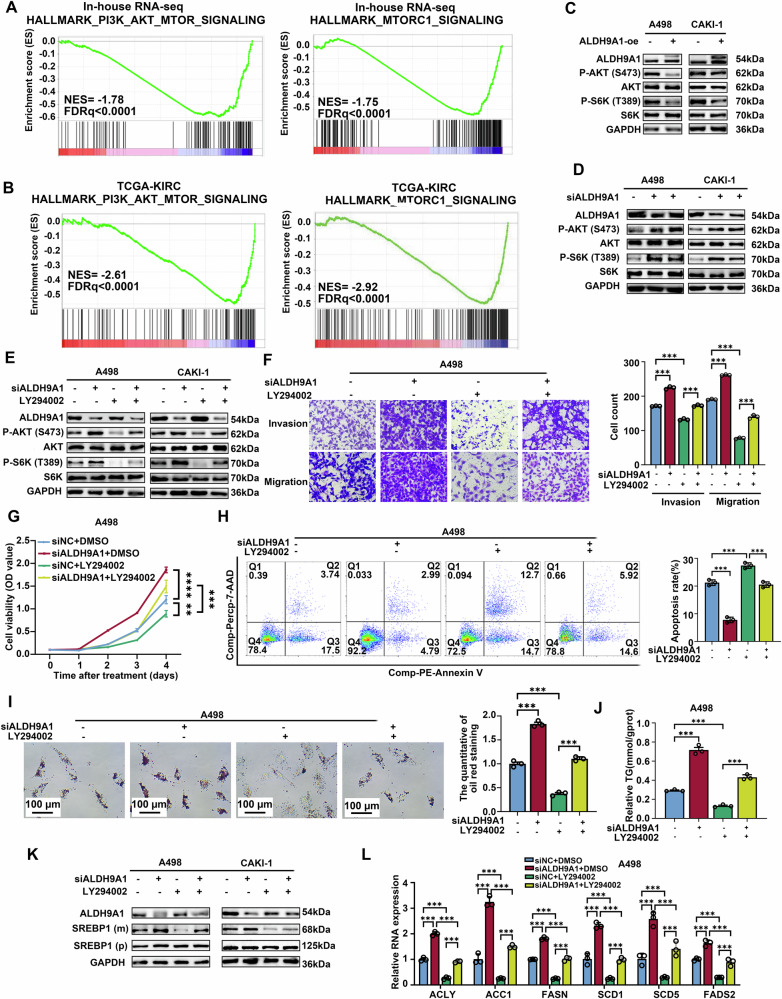
ALDH9A1 repressed the activation of PI3K-AKT-mTOR pathway to inhibited the progression and lipid accumulation in ccRCC. **A** Results from GSEA revealed the association between PI3K-AKT-mTOR pathway and ALDH9A1 based on in-house RNA-sequencing data. Statistical significance was determined at FDRq < 25%. **B** Results from GSEA revealed the association between PI3K-AKT-mTOR pathway and ALDH9A1 based on TCGA-KIRC cohort. Statistical significance was determined at FDRq < 25%. **C** The protein expression of phosphorylated AKT, S6K, as well as total AKT and S6K in ALDH9A1-overexpression ccRCC cells and control ccRCC cells (*n* = 3). **D** The protein expression of phosphorylated AKT, S6K, as well as total AKT and S6K in ALDH9A1-deficient ccRCC cells and control ccRCC cells (*n* = 3). **E** The protein expression of phosphorylated AKT, S6K, as well as total AKT and S6K were assessed in the indicated cell lines (*n* = 3). ccRCC cells with or without ALDH9A1-deficient were treated with 20 μmol/L LY294002 (an inhibitor of PI3K-AKT pathway) or DMSO. **F** Transwell assays were performed to evaluate the migratory and invasive capacity of the indicated ccRCC cells (*n* = 3) (ANOVA for statistics). ccRCC cells with or without ALDH9A1-deficient were treated with 20 μmol/L LY294002 (an inhibitor of PI3K-AKT pathway) or DMSO. **G** Cell proliferation curves were generated using CCK8 assays for the indicated ccRCC cells (*n* = 3) (ANOVA for statistics). ccRCC cells with or without ALDH9A1-deficient were treated with 20 μmol/L LY294002 (an inhibitor of PI3K-AKT pathway) or DMSO. **H** Flow cytometry assay showed the proportion of apoptotic cells for the indicated cell lines (*n* = 3) (ANOVA for statistics). ccRCC cells with or without ALDH9A1-deficient were treated with 20 μmol/L LY294002 (an inhibitor of PI3K-AKT pathway) or DMSO. Comp-PE-Annexin V means that Annexin V was compensated by negative control and single positive control, and Comp-Percp-7-AAD means that 7-AAD was compensated by negative control and single positive control. **I** Photomicrographs of Oil red staining were performed in indicated cell lines (*n* = 3) (ANOVA for statistics). ccRCC cells with or without ALDH9A1-deficient were treated with 20 μmol/L LY294002 (an inhibitor of PI3K-AKT pathway) or DMSO. **J** Relative TG (mmol/gprot) levels were assessed in indicated cell lines (*n* = 3) (ANOVA for statistics). ccRCC cells with or without ALDH9A1-deficient were treated with 20 μmol/L LY294002 (an inhibitor of PI3K-AKT pathway) or DMSO. **K** The protein expression of precursor SREBP1 and mature SREBP1 in the indicated cell lines (*n* = 3). ccRCC cells with or without ALDH9A1-deficient were treated with 20 μmol/L LY294002 (an inhibitor of PI3K-AKT pathway) or DMSO. **L** The mRNA levels of SREBP1 target genes, including *ACLY*, *ACC1*, *FASN*, *SCD1*, *SCD5* and *FADS2* were assessed in the indicated cell lines (*n* = 3) (ANOVA for statistics). ccRCC cells with or without ALDH9A1-deficient were treated with 20 μmol/L LY294002 (an inhibitor of PI3K-AKT pathway) or DMSO. Results represented at least three independent experiments (*P < 0.05, **P < 0.01, ***P < 0.001).

To identify the potential mechanism of ALDH9A1 modulating the progression of ccRCC, CAKI-1 cells overexpressing ALDH9A1 were applied for RNA sequencing. The study then subjected the in-house sequencing data to GSEA analyses, revealing a significant association between ALDH9A1 and the AKT-mTOR pathway (Fig. 5A). This finding was further validated through GSEA analysis using TCGA-KIRC RNA-seq data (Fig. 5B).

To confirm the correlation between ALDH9A1 and PI3K-AKT-mTOR pathway, we investigated the expression of AKT, a serine/threonine kinase playing a pivotal role in Pl3K signaling cascade, as well as ribosomal protein S6 kinase (S6K), which served as a robust downstream effector of mTORC1. Based on the western blotting assay, the level of phosphorated AKT declined in ALDH9A1 overexpressing cells but increased in ALDH9A1-deficient cells (Fig. 5C, D and Supplementary Fig. 5A, B). The level of phosphorated S6K showed a similar trend (Fig. 5C, D and Supplementary Fig. 5A, B). These findings indicated that the lack of ALDH9A1 triggered the activation of AKT-mTOR cascade in ccRCC. To identify weather ALDH9A1 exerted its tumor suppressor role via the AKT-mTOR network, we administrated ALDH9A1-deficient cells with LY294002, a PI3K inhibitor. Firstly, we authenticated that the expression of ALDH9A1 was not affected by the administration of LY294002 (Fig. 5E and Supplementary Fig. 5C). According to the western blotting results, the upregulated expression of phosphorated AKT and phosphorated S6K in ALDH9A1-deficient cells was neutralized by additionally treated with LY294002 (Fig. 5E and Supplementary Fig. 5C). Then, it is reported that the treatment of LY294002 partly neutralized the ascendant invasions and migrations abilities of ALDH9A1-depleting cells (Fig. 5F and Supplementary Fig. 5D). Furthermore, the accelerating proliferation abilities and declined fraction of apoptosis in ALDH9A1-deficient cells were partly reversed by additional treatment of LY294002 (Fig. 5G, H and Supplementary Fig. 5E, F). These results marked that the AKT-mTOR network was involved in the tumor-suppressor role of ALDH9A1 in ccRCC.

It has been acknowledged that the PI3K-AKT axis remarkably regulated metabolism disorders as a central link role. The treatment of LY294002 indeed reduced the level of TG content and lipid droplets in ccRCC cells (Fig. 5I, J). Therefore, we hypothesized that ALDH9A1-mediated lipid metabolism was dependent on the PI3K-AKT pathway. As evidenced by oil red staining and TG concentration detection assays, the enhanced accumulation of lipids in ALDH9A1-depleting cells was abolished upon administrating with LY294002 (Fig. 5I, J and Supplementary Fig. 7A-B). Given that AKT could promote lipogenesis via prompting SREBP1 translocating into the nuclear to stimulate specific genes in an mTOR-dependent manner [29], we then investigated the relationship between the effect of ALDH9A1 on lipid metabolism and AKT-mTOR-SREBP1 cascade. According to the western blotting results, treatment of LY294002 damped the maturation of SREBP1 in ccRCC cells (Supplementary Fig. 6A). The mRNA levels of several SREBP1 target genes, including ACLY, ACC1, FASN, SCD1, SCD5 and FADS2 were decreased upon the treatment of LY294002 (Supplementary Fig. 6B, C). Additionally, ALDH9A1 overexpression led to a reduction in the maturation level of SREBP1 and a decrease in the mRNA levels of its target genes (Supplementary Fig. 6D, E). Conversely, knocking down ALDH9A1 resulted in opposite effects (Supplementary Fig. 6F–I). We then identified that the treatment of LY294002 neutralized the boosted mature SREBP1 in ALDH9A1-absent cells and efficiently attenuated the elevated mRNA levels of SREBP1 target genes involved in lipogenesis in cells lacking ALDH9A1, which pointed out the indispensable role of AKT-mTOR-SREBP1 in ALDH9A1-mediated metabolism reprogramming (Fig. 5K, L and Supplementary Fig. 7C, D).

Taken together, these experiments supported the notion that the classical PI3K-AKT-mTOR participated in the ALDH9A1-mediated tumor-suppressor and metabolic reprogramming roles in ccRCC.

### The deficiency of ALDH9A1 triggered the AKT-mTOR pathway via downregulating the expression of IQGAP2 in ccRCC

**Fig. 6:**
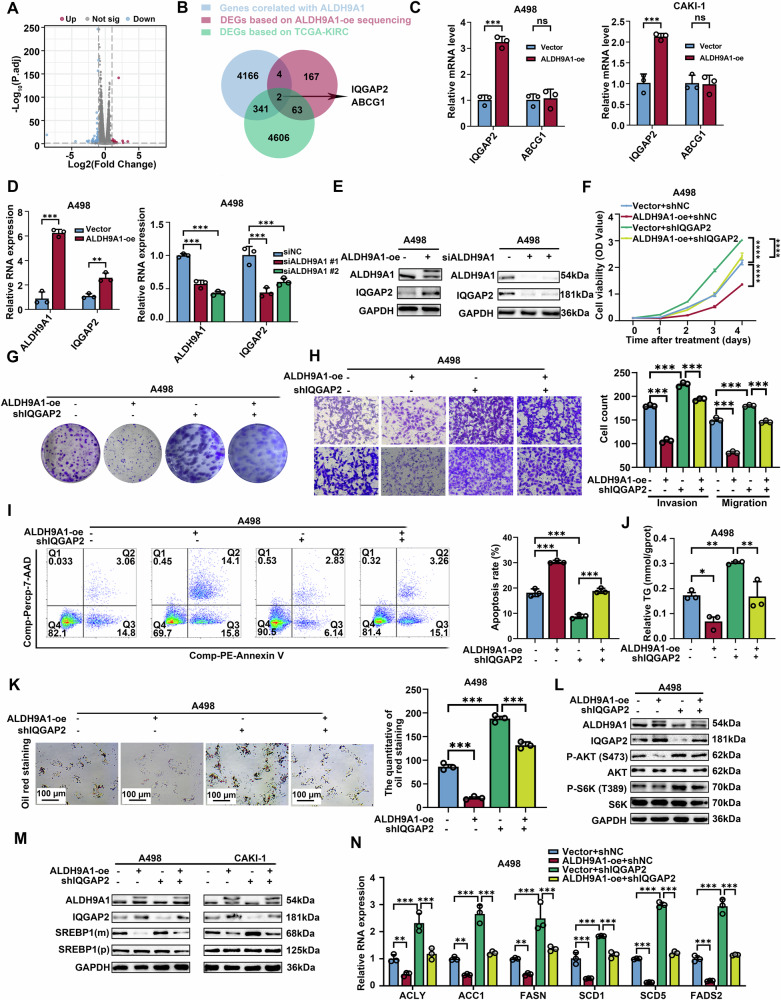
ALDH9A1 repressed the activation of PI3K-AKT-mTOR pathway in IQGAP2-dependent manner. **A** The volcano plot depicts the differentially expressed genes between ALDH9A1-oe group and vector group in CAKI-1 cells. **B** Venn diagram of genes differentially expressed based on in-house sequencing data, genes differentially expressed between ccRCC tumors and normal tissues based on TCGA-KIRC cohort and genes tightly corelated with ALDH9A1 based on TCGA-KIRC cohort. **C** The mRNA expression of *IQGAP2* and *ABCG1* in ALDH9A1-overexpressing ccRCC cells compared with the control group (*n* = 3) (independent-samples *t*-test for statistics). **D** The mRNA expression of *IQGAP2* both in ALDH9A1-overexpressing and ALDH9A1-deficient ccRCC cells compared with the control group (*n* = 3) (independent-samples *t*-test and ANOVA for statistics). **E** The protein expression of IQGAP2 both in ALDH9A1-overexpressing and ALDH9A1-deficient ccRCC cells compared with the control group (*n* = 3). **F** Cell proliferation curves were generated using CCK8 assays for the indicated ccRCC cells (*n* = 3) (ANOVA for statistics). ccRCC cells with or without ALDH9A1-overexpressing were transfected with IQGAP2 knockdown lentivirus or vector. **G** Colony formation assays were performed in the indicated ccRCC cells (*n* = 3). ccRCC cells with or without ALDH9A1-overexpressing were transfected with IQGAP2 knockdown lentivirus or vector. **H** Transwell assays were performed to evaluate the migratory and invasive capacity of the indicated ccRCC cells (*n* = 3) (ANOVA for statistics). ccRCC cells with or without ALDH9A1-overexpressing were transfected with IQGAP2 knockdown lentivirus or vector. **I** Flow cytometry assay showed the proportion of apoptotic cells for the indicated cell lines (*n* = 3) (ANOVA for statistics). ccRCC cells with or without ALDH9A1-overexpressing were transfected with IQGAP2 knockdown lentivirus or vector. Comp-PE-Annexin V means that Annexin V was compensated by negative control and single positive control, and Comp-Percp-7-AAD means that 7-AAD was compensated by negative control and single positive control. **J** Relative TG (mmol/gprot) levels were assessed in indicated cell lines (*n* = 3) (ANOVA for statistics). ccRCC cells with or without ALDH9A1-overexpressing were transfected with IQGAP2 knockdown lentivirus or vector. **K** Photomicrographs of Oil red staining were performed in indicated cell lines (*n* = 3) (ANOVA for statistics). ccRCC cells with or without ALDH9A1-overexpressing were transfected with IQGAP2 knockdown lentivirus or vector. **L** The protein expression of phosphorylated AKT, S6K, as well as total AKT and S6K were assessed in the indicated cell lines (*n* = 3). ccRCC cells with or without ALDH9A1-overexpressing were transfected with IQGAP2 knockdown lentivirus or vector. **M** The protein expression of precursor SREBP1 and mature SREBP1 in the indicated cell lines (*n* = 3). ccRCC cells with or without ALDH9A1-overexpressing were transfected with IQGAP2 knockdown lentivirus or vector. **N** The mRNA levels of SREBP1 target genes, including *ACLY*, *ACC1*, *FASN*, *SCD1*, *SCD5* and *FADS2* were assessed in the indicated cell lines (*n* = 3) (ANOVA for statistics). ccRCC cells with or without ALDH9A1-overexpressing were transfected with IQGAP2 knockdown lentivirus or vector. Results represented at least three independent experiments (*P < 0.05, **P < 0.01, ***P < 0.001).

To better understand the molecular mechanism of the tumor suppressor role of ALDH9A1 in ccRCC, we reanalysis the in-house transcriptome sequencing data, conducted by CAKI-1 cells overexpressing ALDH9A1. A total of 236 genes were identified as differentially expressed in ALDH9A1-overexpressing CAKI-1 cells, with 44 upregulated and 192 downregulated (Log_2_|FC| greater than 1 and a *P*-value lower than 0.05) (Fig. 6A). On that basis, we incorporated a gene set consisting of differentially expressed genes between ccRCC tissues and peritumor normal tissues (Log_2_|FC| greater than 1 and a *P*-value lower than 0.05), as well as sets of genes exhibiting strong correlation with ALDH9A1 (correlation coefficient greater than 0.5) based on the TCGA-KIRC cohort, to identify potential downstream of ALDH9A1 in ccRCC (Fig. 6B). As depicted in the Venn diagram, only IQGAP2 and ABCG1 met these criteria (Fig. 6B and Supplementary Table 3). IQGAP2 exhibited a stronger association with ALDH9A1 and superior predictive value in determining outcomes for ccRCC patients (Supplementary Fig. 8A, B). Furthermore, only the mRNA level of IQGAP2 ascended following the overexpression of ALDH9A1 (Fig. 6C), thus prompting our subsequent experiments to focus on investigating IQGAP2. The positive regulatory relationship between ALDH9A1 and IQGAP2 was confirmed in ccRCC cell lines at both the RNA and protein levels (Fig. 6D, E and Supplementary Fig. 8C–F).

Evidence has reported that IQGAP2 functioned as a tumor suppressor in malignancies [30]. In ccRCC, the mRNA levels of *IQGAP2* were significantly reduced in ccRCC samples compared to normal tissues (Supplementary Fig. 9A). The proteomics data from CPTAC also confirmed a pronounced decrease in IQGAP2 expression in ccRCC (Supplementary Fig. 9B). The migration and invasion capacities were attenuated upon forced expressing of IQGAP2 in ccRCC cells (Supplementary Fig. 9C). CCK8 assays revealed that the upregulation of IQGAP2 notably suppressed cell proliferation in ccRCC cells (Supplementary Fig. 9D). Furthermore, evident increase in apoptotic cells was observed in IQGAP2-overexpressed cells (Supplementary Fig. 9E). These experiments validated the tumor suppressor role of IQGAP2 in ccRCC. Interestingly, accumulating evidence has demonstrated that IQGAP2 inactivates the canonical AKT signaling pathway to exert the tumor suppressor roles [31, 32]. The GSEA investigations indicated a potential involvement of IQGAP2 in lipogenesis (Supplementary Fig. 10A). Thus, we then assessed the association between IQGAP2 and PI3K-AKT pathway as well as lipid metabolism. According to the western blotting assay, the level of phosphorated AKT and phosphorated S6K, as well as the maturation of SREBP1, declined in IQGAP2-overexpressed cells (Supplementary Fig. 10B, C). Triglyceride concentration detection indicated that ccRCC cells overexpressing IQGAP2 showed lower TG levels compared with the control groups (Supplementary Fig. 10D). Consistently, the oil red staining assays revealed that the lipid drop levels declined upon forced expression of IQGAP2 (Supplementary Fig. 10E). Collectively, these findings suggested that the downregulation of IQGAP2 in ccRCC promoted disease progression and elevated lipid accumulation.

To further elucidate the functional significance of IQGAP2 in the tumor suppressor role of ALDH9A1 in ccRCC, we introduced shRNA to achieve stable silencing of IQGAP2 in A498 and CAKI-1 cell lines (Supplementary Fig. 11A–C), accompanied by transfection with ALDH9A1 overexpression lentivirus or control. As demonstrated by CCK8 and colony formation assays, the knockdown of IQGAP2 accelerated the cell proliferation and counteracted the impact of forced expression of ALDH9A1 in ccRCC cells (Fig. 6F, G and Supplementary Fig. 11D–G). Besides, the ablation of IQGAP2 effectively mitigated the compromised migratory and invasive capabilities observed in ccRCC cell lines overexpressing ALDH9A1 (Fig. 6H and Supplementary Fig. 11H). Meanwhile, the heightened rate of apoptosis observed in ALDH9A1-overexpressing cells was effectively attenuated upon IQGAP2 ablation (Fig. 6I and Supplementary Fig. 11I). This finding suggested that the downregulation of ALDH9A1 promoted the proliferation and migration through repressing the expression of IQGAP2 in ccRCC.

Then, we proceeded to examine whether there is any association between IQGAP2 and ALDH9A1-mediated metabolic reprogramming. The oil red staining and TG concentration detection assays revealed that the reduction in lipid accumulation observed in ALDH9A1-overexpressed cells was neutralized upon further suppression of IQGAP2, suggesting the significance of IQGAP2 in ALDH9A1-mediated metabolic reprogramming (Fig. 6J, K and Supplementary Fig. 12A, B). Given the validated regulatory relationship between ALDH9A1 and AKT-mTOR signaling in our previous research, we subsequently investigated whether the ALDH9A1-mediated abnormal activation of the AKT-mTOR pathway involved IQGAP2 participation. The western blotting exhibited that the knockdown of IQGAP2 effectively promotes the phosphorylating of AKT and S6K, which were offset by overexpression of ALDH9A1 (Fig. 6L and Supplementary Fig. 12C–E). Additionally, the knockdown of IQGAP2 forced the maturation of SREBP1 and promoted the mRNA levels of its target genes (Fig. 6M, N and Supplementary Fig. 12F–H). This effect effectively offset the reduced maturation of SREBP1 and defective expression of its target genes we observed in cells overexpression ALDH9A1 (Fig. 6M, N and Supplementary Fig. 12F–H). Taken together, these results validated that ALDH9A1 exerted tumor suppressor role and metabolic reprogramming role by repressing the expression of IQGAP2 and activating the AKT-mTOR-SREBP1 pathway in ccRCC.

### ALDH9A1 interacted with NPM1 and influenced the transcription of IQGAP2 in ccRCC

**Fig. 7:**
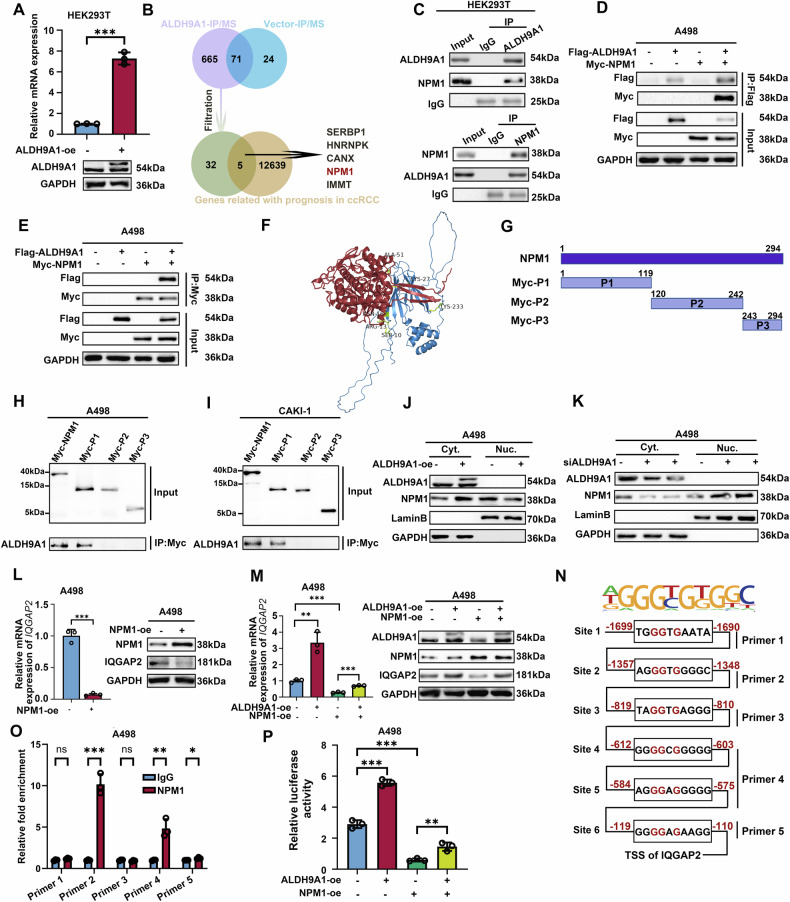
ALDH9A1 interacted with NPM1 and affected the transcription of IQGAP2. **A** The HEK293T cell lines were engineered to overexpress ALDH9A1 through the introduction of Flag-ALDH9A1 lentivirus. The overexpression of ALDH9A1 was verified at the mRNA expression and protein level (*n* = 3) (independent-samples *t*-test for statistics). **B** Venn diagram illustrated molecules pulled down through Flag-ALDH9A1 group, but not pulled down through vector group and the molecules exhibiting strong correlation with ccRCC patient prognosis at mRNA levels based on the TCGA-KIRC cohort. **C** The endogenous ALDH9A1-NPM1 interaction was determined by Co-IP assays in HEK293T cells (*n* = 3). **D**, **E** The exogenous ALDH9A1-NPM1 interaction was determined by Co-IP assays in A498 cells overexpressed Flag-ALDH9A1 and Myc-NPM1 (*n* = 3). **F** The protein-protein complex interaction prediction between ALDH9A1 and NPM1 is depicted based on the 3D structure of ALDH9A1 represented in red and the 3D structure of NPM1 represented in blue. The binding site prediction is based on the amino acid sequence of NPM1. **G** The diagrams show wild-type NPM1 (full length, 1-294) and its three truncations. **H**, **I** A498 and CAKI-1 cells were transfected with the indicated truncated plasmids, followed by Co-IP assays and western blotting to assess the interaction between ALDH9A1 and NPM1 truncations (*n* = 3). **J** Western blotting shows subcellular localization of NPM1 after overexpression of ALDH9A1 in A498 cells (*n* = 3). **K** Western blotting shows subcellular localization of NPM1 after knockdown of ALDH9A1in A498 cells (*n* = 3). **L** The mRNA levels and protein expression of IQGAP2 in NPM-1-overexpressing ccRCC cells (*n* = 3) (independent-samples *t*-test for statistics). **M** The mRNA levels and protein expression of IQGAP2 in indicated ccRCC cells (*n* = 3) (ANOVA for statistics). ccRCC cells with or without ALDH9A1-overexpressing were transfected with NPM1 overexpressing plasmid or vector. **N** Schematic representation of NPM1 binding motif (the upper panel) and the predicted NPM1 binding sites within the 2000-bp upstream the *IQGAP2* transcription start site (the lower panel). **O** ChIP-PCR assays of the NPM1 binding sites in the promoter of *IQGAP2* in ccRCC cells (*n* = 3) (independent-samples *t*-test for statistics). **P** Relative luciferase activity of the IQGAP2 luciferase reporter vector in indicated ccRCC cells (*n* = 3) (ANOVA for statistics). ccRCC cells with or without ALDH9A1-overexpressing were transfected with NPM1 overexpressing plasmid or vector. Results represented at least three independent experiments (*P < 0.05, **P < 0.01, ***P < 0.001).

To elucidate how ALDH9A1 influenced the expression of IQGAP2, we immunoprecipitated the ALDH9A1 protein using an anti-flag antibody in HEK293T cells transfected with flag-ALDH9A1 lentivirus and discerned the molecules that bound with ALDH9A1 via shotgun LC-MS/MS (Fig. 7A and Supplementary Fig. 13A). We integrated proteins isolated from cells transfected with Flag-ALDH9A1 lentivirus, but not from cells transfected with the vector, and set coverage higher than 20% and unique peptides higher than 5 as the threshold to select more reliable proteins. Along with molecules exhibiting significant prognostic value for ccRCC patients, we identified five potential candidates, namely HNRNPK, IMMT, CANX, NPM1, and SERBP1 (Fig. 7B and Supplementary Tables 4, 5). Subsequently, we used anti-flag antibodies to immunoprecipitated endogenous compounds to validate the accuracy of our findings obtained via shotgun LC-MS/MS. Notably, only NPM1 demonstrated explicit interaction with Flag-ALDH9A1 in A498 and CAKI-1 cells (Supplementary Fig. 13B, C). A series of more profound Co-IP assays were performed to verify the interaction between ALDH9A1 and NPM1. Endogenous ALDH9A1 was immunoprecipitated from cell lysates obtained from HEK293T cells, followed by successful immunoblotting for NPM1 (Fig. 7C). Also, endogenously immunoprecipitated NPM1 also efficiently immunoblot for ALDH9A1 (Fig. 7C). Subsequently, endogenous Co-IP assays were repeated in A498 and CAKI-1 cells (Supplementary Fig. 13D, E). To further validate the interaction, Flag-ALDH9A1 and Myc-NPM1 were forced expressed in A498 and CAKI-1 cells, followed by Flag immunoprecipitation and Myc immunoprecipitation (Fig. 7D, E and Supplementary Fig. 13F, G). The data demonstrated a reciprocal co-immunoprecipitation between Flag-ALDH9A1 and Myc-NPM1. We then devolved into the precise mechanism underlying the interaction between ALDH9A1 and NPM1. Initially, the ZDOCK server was conducted to predict interaction based on the 3D structure of protein complexes, which was further refined by electrostatics, desolvation-free energy, and shape complementarity [16]. The top prediction was further analyzed in the PDBePISA server and visualized via pymol software (Fig. 7F). According to the PDBePISA analyses, this interface buried ~1738.5 Å^2^ of surface area with a ΔG of −14.9 kcal/mol, and the region interacted with ALDH9A1 was mainly localized in the N-terminus domain in NPM1. To further decipher the binding domain of NPM1 with ALDH9A1, we truncated the full-length NPM1 into three domains according to the distribution of its functional domain [33] (Fig. 7G). Subsequently, the three Myc-tagged NPM1 parts were cloned and then transfected into A498 and CAKI-1 cells. Among the forementioned three truncated parts, only Myc-P1, representing the N-terminus domain of NPM1, exhibited interaction with ALDH9A1, which was consistent with previous predictions (Fig. 7H, I).

When investigating biological function of the interaction between ALDH9A1 and NPM1,the initial hypothesis is that protein binding may influence the stability of NPM1. We tested the protein levels of NPM1 in A498 and CAKI-1 cells with ALDH9A1 overexpression and deficiency. The results showed that ALDH9A1 does not influence the total amount of NPM1 protein (Supplementary Fig. 14A–D). Interstingly, it has been reported that NPM1 constantly shuttled between the nucleolus and cytoplasm [34]. Thus, we reasonably speculated if the combination of ALDH9A1 and NPM1 affected the distribution of NPM1 between cytoplasm and nucleolus. The western blotting of nucleo-cytoplasmic fractionation exhibited that the enforced expression of ALDH9A1 led to a notable enhancement of accumulation of NPM1 in cytoplasm while damping NPM1 nuclear localization. Inversely, the knockdown of ALDH9A1 accelerated the NPM1 translocating into nuclear (Fig. 7J, K and Supplementary Fig. 14E–J). The immunofluorescence assays have yielded similar conclusions that in the cells overexpressing ALDH9A1, the accumulation of NPM1 in the cytoplasm was enhanced (Supplementary Fig. 14K, L).

NPM1 plays diverse roles in cellular processes including transcription [34, 35]. Consequently, we assessed if ALDH9A1 regulated the expression of IQGAP2 in an NPM1-dependent manner. The overexpression of NPM1 resulted in a decline of both mRNA and protein levels of IQGAP2 (Fig. 7L and Supplementary Fig. 15A–D), effectively compensating for the elevated expression observed in ALDH9A1-overexpressing cells, which verified the involving of NPM1 in ALDH9A1-mediated regulation of IQGAP2 (Fig. 7M and Supplementary Fig. 15E-H). Given the known function of NPM1 as a transcription regulator, we hypothesized that NPM1 might interact with the *IQGAP2* promoter to regulate transcription [36–38]. Zhang et al. identified a specific binding motif within the *TSC1* promoter where NPM1 bound to suppresse *TSC1* expression [36]. Based on this NPM1 binding motif, six binding sites were screened out within the 2000-bp upstream the *IQGAP2* transcription start site (Fig. 7N). For sites 4 and 5, a single primer was designed to amplify a contiguous DNA segment containing the two sites due to their close proximity within the promoter region, while primers for the other sites were individually designed (Fig. 7N and Supplementary Table 2). ChIP results exhibited the regions around the six predicted binding sites showed varying binding efficiency with the NPM1, with site 2, site 4 and site 5 exhibiting the highest efficiency (Fig. 7O and Supplementary Fig. 15I). To further confirm the link between NPM1 and the promoter of the *IQGAP2*, a luciferase report assay was carried out by constructing a plasmid carrying the luciferase gene controlled by *IQGAP2* promoter and introducing it into ccRCC cell lines. The results demonstrated that NPM1-overexpressing cells exhibited a decrease in luciferase activity, which offset the increased luciferase activity observed in cells overexpressing ALDH9A1 (Fig. 7P and Supplementary Fig. 15J). In conclusion, these experiments illustrated that ALDH9A1 influenced the distribution of NPM1 within cells, consequently leading to reduced expression of IQGAP2 at the gene transcription levels in ccRCC.

### The ALDH9A1-IQGAP2 axis in ccRCC modulated tumor progression and lipid accumulation in vivo

**Fig. 8:**
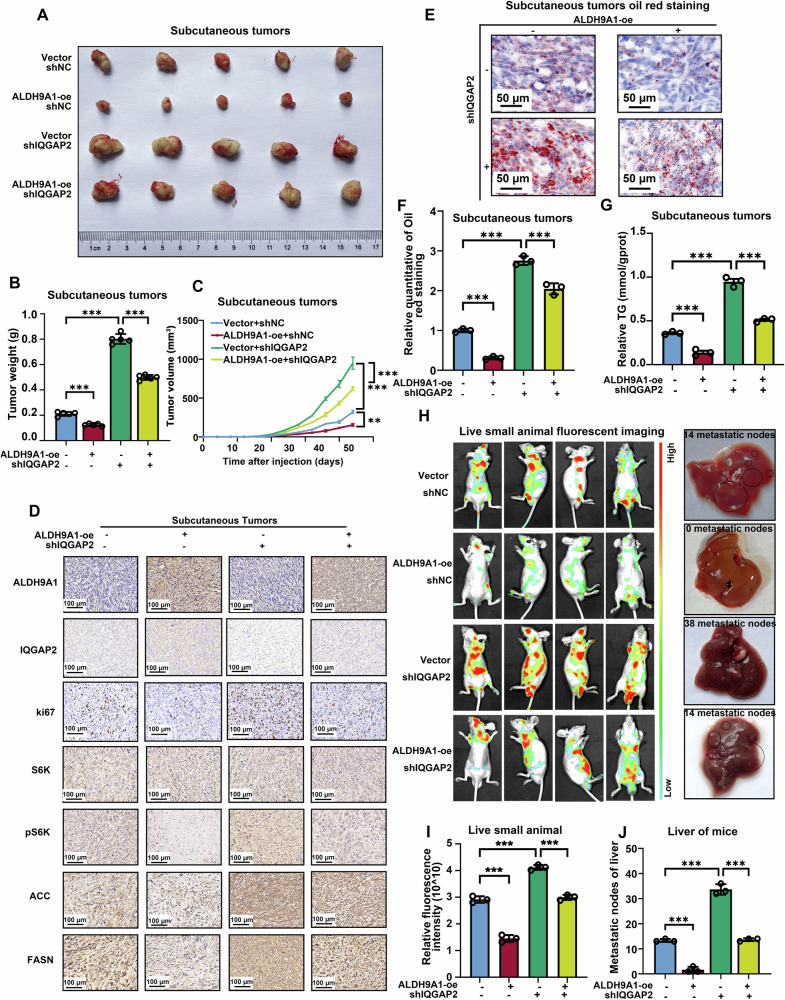
The ALDH9A1-IQGAP2 axis modulated tumor progression and lipid accumulation in vivo. **A** Representative images depicting isolated subcutaneous tumors of the Vector + shNC, ALDH9A1-oe + shNC, Vector + shIQGAP2, and ALDH9A1-oe + shIQGAP2 groups from BALB/c nude mice. **B**, **C** CAKI-1 cells with or without ALDH9A1 overexpression, were transfected with IQGAP2 knockdown lentivirus or vector and subsequently implanted into BALB/c nude mice for in vivo studies. The tumor size was monitored at 4-day intervals, with the last measurement performed on day 44. Tumors were harvested and weighed after mice were euthanized (*n* = 5) (ANOVA for statistics). **D** IHC staining for ALDH9A1, IQGAP2, ki-67, S6K, phosphorylated S6K, ACC and FASN in the isolated tumor xenografts of the Vector + shNC, ALDH9A1-oe +shNC, Vector + shIQGAP2, and ALDH9A1-oe + shIQGAP2 groups (*n* = 3). **E**, **F** Photomicrographs of Oil red staining in isolated tumor xenografts of the Vector + shNC, ALDH9A1-oe + shNC, Vector + shIQGAP2, and ALDH9A1-oe + shIQGAP2 groups (*n* = 3) (ANOVA for statistics). **G** Relative TG (mmol/gprot) levels in isolated tumor xenografts of the Vector + shNC, ALDH9A1-oe + shNC, Vector + shIQGAP2, and ALDH9A1-oe + shIQGAP2 groups (*n* = 3) (ANOVA for statistics). **H**, **I** Live small animal fluorescent images of the Vector + shNC, ALDH9A1-oe + shNC, Vector + shIQGAP2, and ALDH9A1-oe + shIQGAP2 metastasis groups (*n* = 3) (ANOVA for statistics). **J** The number of metastatic nodes was counted on all sides of the liver (*n* = 3) (ANOVA for statistics). Results represent at least three independent experiments (*P < 0.05, **P < 0.01, ***P < 0.001).

Next, subcutaneous and metastatic tumor models were employed to study the role of the ALDH9A1-IQGAP2-AKT axis in vivo. Our subcutaneous tumor xenografts studies revealed that the enforced expression of ALDH9A1 remarkably restrained the tumor growth in comparisons with the vector group, whereas the knockdown of IQGAP2 reversed the impact of ALDH9A1 overexpression on the tumor growth (Fig. 8A). The tumor weight and tumor volume in the ALDH9A1-overexpressing group were significantly decreased, while the additional knockdown of IQGAP2 reversed this inhibition caused by ALDH9A1 overexpression (Fig. 8B, C). Then, tumor tissues isolated from subcutaneous xenografts were subjected to IHC staining (Fig. 8D and Supplementary Fig. 16A). In the ALDH9A1-overexpression group, we observed declined expression of ki67, phosphorylated S6K, FASN and ACC1, which was restored by additional knockdown of IQGAP2 (Fig. 8D and Supplementary Fig. 16A). Notably, the triglyceride concentration detection and oil red staining assays revealed a significant reduction in lipid accumulation in the tumor xenografts from ALDH9A1-overexpressing groups, thereby counteracting the observed upregulation of lipid accumulation in the IQGAP2-knockdown group (Fig. 8E–G). Live small animal fluorescent imaging assays showed that the knockdown of IQGAP2 abrogated suppressive impact on tumor metastasis caused by ALDH9A1 overexpression (Fig. 8H, I). Besides, in the ALDH9A1-overexpression group, we observed inconspicuous metastasis nodes in the liver, which were subverted upon the depletion of IQGAP2 (Fig. 8H, J). These findings suggested that the ALDH9A1-IQGAP2-AKT axis plays a irreplaceable role in tumor progression and metabolic reprogramming role in vivo in ccRCC.

## Discussion

The aldehyde dehydrogenase (ALDHs) superfamily, comprising 19 protein-coding phase I oxidizing enzymes responsible for the detoxification process converting aldehydes into corresponding carboxylic acids, also exhibits substantial potential in the realm of cancer research [39]. Nevertheless, there has been a dearth of comprehensive investigations into the involvement of ALDHs in ccRCC [12]. In this study, we conducted multi-dimensional bioinformatics analyses on the 19 ALDH genes and identified ALDH9A1 as a prominent player in ccRCC. We unveiled a significant association between ALDH9A1 and tumor progression, as well as a linkage between ALDH9A1 and lipid accumulation in ccRCC. The downregulation of ALDH9A1 regulated by FTO-mediated m^6^A modification resulted in increased nuclear re-shuttling of NPM1, leading to a decrease in IQGAP2 expression and activation of the AKT-mTOR signaling pathway. Functional experiments validated the ALDH9A1-NPM1-IQGAP2-AKT irreplaceable roles in tumor progression and lipid accumulation, both in vivo and in vitro.

RNA modification represented a highly efficient way of regulating RNA stability, splicing, and translation, and the m^6^A modification is wildly confirmed as the most robust RNA modification [26]. m^6^A modification is distinguished by its dynamic and reversible nature and necessitates the collaborative efforts of m^6^A methyltransferases, demethylases, and the m^6^A recognition proteins. Among these components, FTO, as the pioneering identified m^6^A demethylases, catalyzes the demethylation of m^6^A and modifies RNA stability [40]. Recently, upregulated FTO has been validated in ccRCC and correlates with poor outcomes of patients [28]. In detail, FTO modulates the m^6^A levels in the mRNA of *SIK2* and attenuates its mRNA stability, thereby promoting proliferation and metastasis in ccRCC [28]. In our previous study, we also identified that FTO-mediated OGDHL demethylation led to damaged RNA stability and inhibited its expression in ccRCC [41]. ALDHs are frequently overexpressed in tumors [10], whereas ALDH9A1 exhibits downregulation in ccRCC. Thus, we were compelled to investigate the underlying cause of reduced ALDH9A1 expression, with m^6^A modification emerging as our primary focus. Through data mining and experiment validation, we demonstrated that FTO facilitated the demethylation of ALDH9A1 transcripts at site 1846 within 3’-UTR and impaired its RNA stability leading to the suppression of ALDH9A1. Small-molecule inhibitors targeting FTO, such as CS1 and CS2, have been investigated for years, but none have been tested in ccRCC patients [42]. Our team has multiply validated the oncogene role of FTO in ccRCC, suggesting that applying FTO inhibitors could be a potential therapeutic strategy for these patients. Given the nonspecific nature of compounds, siRNA-based therapeutics can be employed to selectively silence FTO and subsequently evaluate its antitumor efficacy [43].

The metabolism-reprogramming nature of ccRCC has been extensively elucidated, with the cytoplasmic lipid deposits being characterized as its defining histological feature [6]. Altered metabolism of fatty acids (FA) and the accumulation of lipid droplets contribute to intrinsic antioxidant activity within tumors, whereas maintaining cell membrane fluidity, plays a crucial ccRCC tumorigenesis [6]. Previously, we have identified a phenomenon termed the “slimming” effect, wherein restoration of PLCL1 hinders tumor progression and reduces lipid accumulation [44]. Additionally, DBT, the constituent of the branched-chain alpha-keto acid dehydrogenase complex, has been observed to mitigate lipid accumulation through the Hippo pathway [45]. Here, we demonstrated that the restoration of ALDH9A1 efficiently moderated the volume of TG and the abnormal lipid accumulation through an enzyme activity-independent mechanism. SREBP1, the master regulators of lipid metabolism, functioned as the upstream transcription factor for several key players in lipid metabolism, including FASN, ACLY, ACC, and SCD1, whose coordinated enzymatic activities are essential for de novo lipogenesis and TG synthesis [46, 47]. In the present study, we demonstrated that restoration of ALDH9A1 effectively attenuated the maturation of SREBP1 and mitigated the mRNA levels of SREBP1 target genes. The silencing, deficiency, or mutation of tumor suppressor genes leads to tumor initiation and progression. The CRISPR/Cas9 system has revolutionized cancer research by allowing rapid reinforcement of these genes in vitro and in vivo [48]. In this study, we have validated ALDH9A1 as a tumor suppressor gene specially in ccRCC, where its downregulation enhances tumor progression and lipid accumulation. Therefore, CRISPR/Cas9 technology can be utilized therapeutically to restore ALDH9A1 expression, to slow tumor progression and achieve tumor “slimming” clinically.

The AKT and mTOR pathways are two crucial intracellular signaling, which exhibit such a high degree of interconnectivity that they are often regarded as a unified pathway [13]. These pathways are activated by diverse cellular stimuli and play a pivotal role in regulating fundamental cellular processes [14]. The disruption in the functioning of the AKT-mTOR pathway is associated with various malignancies, highlighting its significance as a target for antitumor therapeutics [13]. Interestingly, SREBP1 functions as the downstream effector of the AKT-mTOR signaling [49]. Here, we demonstrated that the attenuation of ALDH9A1 in ccRCC triggers aberrant abnormal activation of the AKT-mTOR pathway, which is responsible for the tumor progression and lipid accumulation mediated by SREBP1. The activation of AKT-mTOR signaling triggered by the deficiency of ALDH9A1 necessitates two additional key molecules, namely NPM1 and IQGAP2. NPM1, a phosphoprotein primarily localized in the nucleus with nucleocytoplasmic shuttling capability, plays diverse roles in cellular processes including DNA repair, transcription, and ribosome biogenesis [34, 35]. In the present study, we validated that ALDH9A1 interacted with NPM1 through its N terminus domain and affected the re-shuttle of NPM1 from the cytoplasm back to the nucleus to play a role in transcription regulation. The conserved N terminus domain of NPM1 owned two nuclear exporter signals (NES) and it has been reported that the high binding affinity of the NES might lead to the cytoplasmic accumulation [33, 50], which partly explained the mechanism by which ALDH9A1 affected the cellular distribution of NPM1. Through in-house RNA-seq analysis and experimental validation, we have demonstrated that IQGAP2 is downstream of ALDH9A1. Besides, our data showed the forced expression of NPM1 could offset the elevated mRNA and protein levels of IQGAP2 observed in ALDH9A1-overexpression ccRCC cells. Thus, we hypothesized that NPM1 may influence the transcription of IQGAP2, which has subsequently been experimentally validated via ChIP and luciferase reporter assays. Notably, NPM1 facilitated the activation of AKT signaling in colorectal cancer, while IQGAP2 could also influence the activation of the AKT pathway [31, 32, 51]. Thus, here we supposed that in ccRCC, the attenuation of ALDH9A1 fails to retain NPM1 in the cytoplasm and promote its accumulation in the nuclear, leading to inhibition of IQGAP2 and subsequent activation of AKT-mTOR signaling to support tumor progression and lipid accumulation. Extensive effort has been dedicated to identifying drugs targeting the AKT-mTOR pathway; however, only a few are being assessed in clinical trials [13]. Here, we further emphasized the close relationship between the AKT pathway and dysregulated lipid metabolism in ccRCC. Therefore, the combined usage of AKT inhibitors and small-molecule inhibitors targeting fatty acid metabolism might represent a novel therapeutic strategy to improve clinical outcomes in ccRCC.

Certain aspects require additional clarification. Firstly, ALDH9A1 is a member of the ALDHs family, which primarily functions in the dehydrogenation of aldehyde compounds [9]. Our findings suggest that ALDH9A1 holds significant potential for research purposes. However, as an enzyme, the exclusion of ALDH9A1 enzymic activity in affecting gene expression requires further elucidation.

## Conclusion

**Fig. 9:**
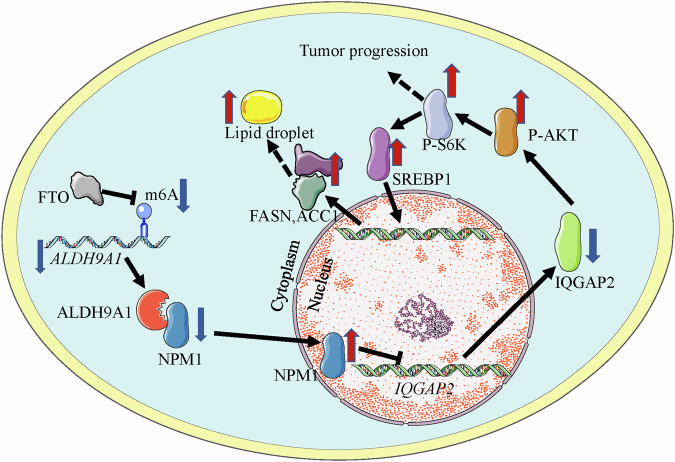
The mechanism scheme of ALDH9A1 in ccRCC. The mechanism scheme of ALDH9A1 in ccRCC. FTO-mediated demethylated *ALDH9A1* mRNA leads to the decrease of ALDH9A1 in ccRCC. Consequently, the reduction in ALDH9A1 fails to sequester NPM1 within the cytoplasm, causing its accumulation in the nuclear and subsequent suppression of IQGAP2. This ultimately activates the AKT-mTOR signaling pathway. Activation of the AKT-mTOR pathway boosts the maturation of SREBP1 and elevates the mRNA expression of genes involving lipogenesis, leading to lipid accumulation. The Figs. are portrayed with the help of the website Servier Medical Art (smart.servier.com).

This study elucidated how ALDH9A1 exerted its tumor suppressor roles via the AKT-mTOR pathway in ccRCC. FTO demethylated the mRNA of *ALDH9A1* and attenuated the RNA stability, leading to the decrease of ALDH9A1 in ccRCC. The reduction in ALDH9A1 fails to retain NPM1 within the cytoplasm, causing its accumulation in the nuclear, resulting in suppression of IQGAP2 and subsequent activation of AKT-mTOR signaling. The activation of the AKT-mTOR pathway boosts the maturation of SREBP1 and elevates the mRNA expression of genes involving lipogenesis. These findings exhibit that ALDH9A1 can be regarded as a reliable prognostic marker and deliver comprehensive insights into the regulations and functions of the AKT-mTOR pathway in ccRCC, thereby establishing a solid research foundation of new therapeutic approaches for ccRCC patients (Fig. 9).

### Supplementary information

supplementary Figs.

supplementary table 1

supplementary table 2

supplementary table 3

supplementary table 4

supplementary table 5

Original data

## Data Availability

The datasets used in this article were derived from the TCGA database (https://portal.gdc.cancer.gov/) and the CPTAC project (https://pdc.cancer.gov/pdc/browse). The in-house sequencing dataset has been stored in a recommended data repository (Science Data Bank, http://www.scidb.cn/). The private link of the datasets is https://www.scidb.cn/en/s/7vU3Qf.
