# Publisher Correction: N6-methyladenosine-modified ALDH9A1 modulates lipid accumulation and tumor progression in clear cell renal cell carcinoma through the NPM1/IQGAP2/AKT signaling pathway

**DOI:** 10.1038/s41419-024-07019-4

**Published:** 2024-08-29

**Authors:** Diaoyi Tan, Daojia Miao, Chuanyi Zhao, Jian Shi, Qingyang Lv, Feiyi Lu, Hailong Ruan, Zhiyong Xiong, Xiaoping Zhang

**Affiliations:** 1 Department of Urology, Union Hospital, Tongji Medical College, Huazhong University of Science and Technology, Wuhan, 430022 China; 2 Institute of Urology, Union Hospital, Tongji Medical College, Huazhong University of Science and Technology, Wuhan, 430022 China

**Keywords:** Renal cell carcinoma, Fatty acids, Lipid signalling

Correction to: *Cell Death & Disease* 10.1038/s41419-024-06896-z, published online 22 July 2024

In this article the wrong ESM file has been process. This has been corrected.

The original article has been corrected.

### Supplementary information

full length uncropped original western blots corrected version

Supplementary figures correct version

