# Supplementary material for: N6-methyladenosine-modified ALDH9A1 modulates lipid accumulation and tumor progression in clear cell renal cell carcinoma through the NPM1/IQGAP2/AKT signaling pathway
Source: Cell Death Dis. 2024 Jul 22;15(7):520. doi: 10.1038/s41419-024-06896-z (PMC11263707; doi:10.1038/s41419-024-06896-z)
Supplement: Supplementary file 1 — supplementary Figs. [file 41419_2024_6896_MOESM1_ESM.pdf]

Supplementary figure 1: ALDH9A1 functioned as the core gene of the ALDH family in ccRCC.

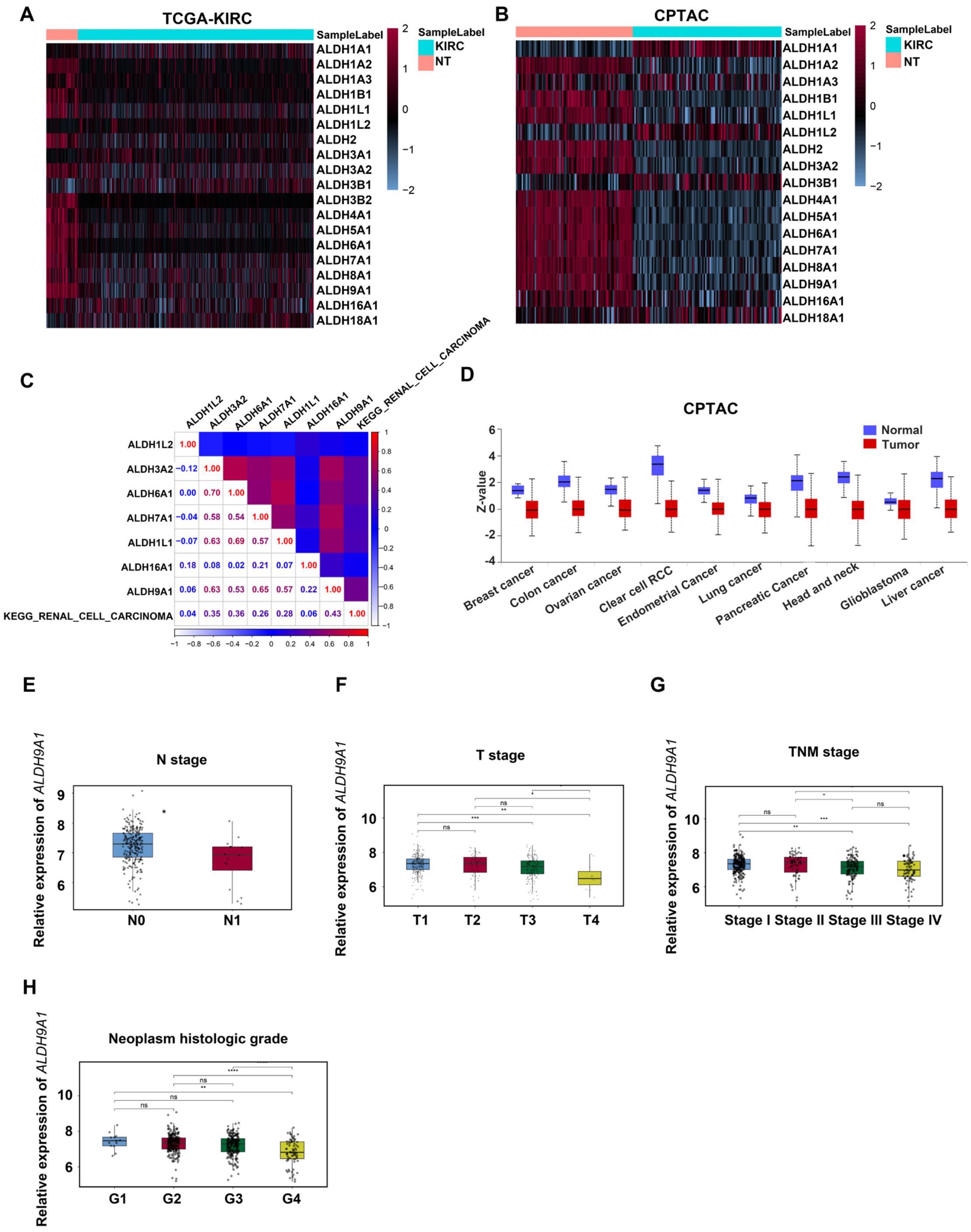

(A) The heatmap demonstrating the mRNA levels of 19 genes from the ALDHs family in ccRCC tissues and normal tissues based on the TCGA-

KIRC cohort.

(B) The heatmap demonstrating the protein expression of 17 genes from the ALDHs family in ccRCC tissues and normal tissues based on the CPTAC-PDC000127 cohort. ALDH3A1 and ALDH3B2 were not shown in the figure due to their absence in the CPTAC-PDC000127 cohort.

(C) Results from GSVA revealed the association between KEGG_RENAL_CELL_CARCINOMA pathway in ccRCC and the mRNA level of the ALDHs family based on the TCGA-KIRC cohort.

(D) The relative protein expression of ALDH9A1 in tumor tissues compared to normal tissues was assessed across 10 common tumor types using the CPTAC cohorts.

(E-H) The plots showed the association of ALDH9A1 with the N stage, T stage, TNM stage, and neoplasm histologic grade based on the TCGA-KIRC cohort.

Supplementary figure 2: ALDH9A1 inhibited the progression of ccRCC in vitro and in vivo.

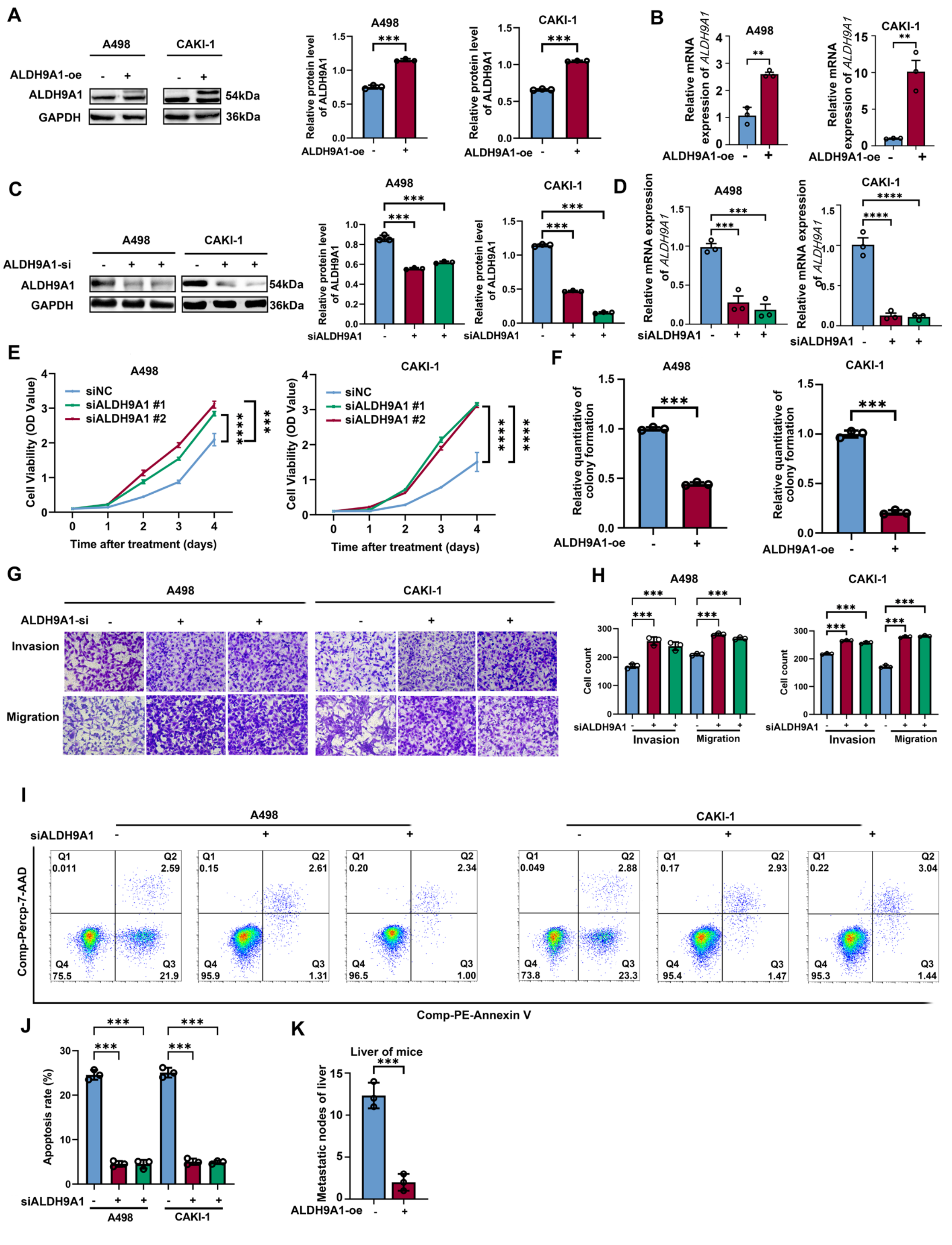

(A) The protein expression of ALDH9A1 in ALDH9A1-overexpression cells compared with control groups (n = 3) (independent-samples t-test

for statistics).

(B) The mRNA levels of *ALDH9A1* in ALDH9A1-overexpression cells compared with the control group (*n* = 3) (independent-samples *t*-test for statistics).

(C) The protein expression of ALDH9A1 in ALDH9A1-deficient ccRCC cells compared with control ccRCC groups (*n* = 3) (ANOVA for statistics).

(D) The mRNA levels of *ALDH9A1* in ALDH9A1-deficient ccRCC cells compared with control ccRCC groups (*n* = 3) (ANOVA for statistics).

(E) Cell proliferation curves of CCK8 assays for ALDH9A1-deficient cells compared with the control group (*n* = 3) (ANOVA for statistics).

(F) The quantitative result of colony formation assays for ALDH9A1-overexpression cells compared with the control group (*n* = 3) (independent-samples *t*-test for statistics).

(G-H) Transwell assay of the migration and invasion for ALDH9A1-deficient cells compared with the control group (*n* = 3) (ANOVA for statistics).

(I-J) Flow cytometry assay determining the proportion of apoptotic cells in ALDH9A1-deficient ccRCC cells compared to control cells (*n* = 3) (ANOVA for statistics). Comp-PE-Annexin V means that Annexin V was compensated by negative control and single positive control, and Comp-Percp-7-AAD means that 7-AAD was compensated by negative control

and single positive control.

(K) The number of metastatic nodes in the liver tissue from vein tail metastasis models in the ALDH9A1-overexpressed group compared with control groups ($n = 3$) (independent-samples t-test for statistics).

Results represent at least three independent experiments ($*P < 0.05$, $**P < 0.01$, $***P < 0.001$).

Supplementary figure 3: Low expression of ALDH9A1 in ccRCC was independent of DNA methylation.

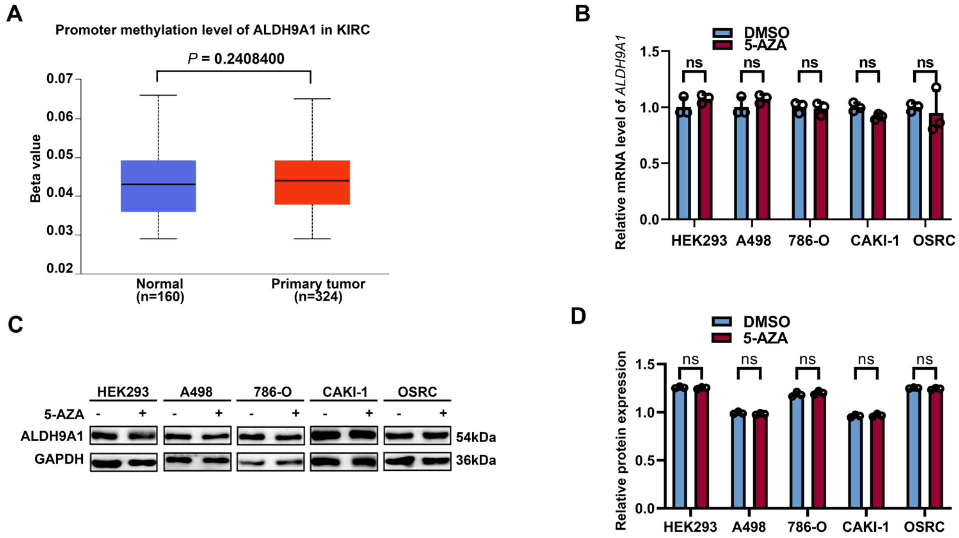

(A) Promoter methylation level of ALDH9A1 in ccRCC tumor compared with normal tissues based on TCGA-KIRC cohort (https://ualcan.path.uab.edu). Different beta value cut-offs have been considered to indicate hyper-methylation [Beta value:0.7-0.5] or hypo-methylation [Beta-value:0.3-0.25].

(B) The mRNA level of *ALDH9A1* in ccRCC cell lines after 5-AZA treatment (independent-samples *t*-test for statistics).

(C-D) The protein expression of ALDH9A1 in ccRCC cell lines after 5-AZA treatment (independent-samples *t*-test for statistics).

Supplementary figure 4: Low expression of ALDH9A1 in ccRCC is caused by FTO-mediated m^{6}A modification.

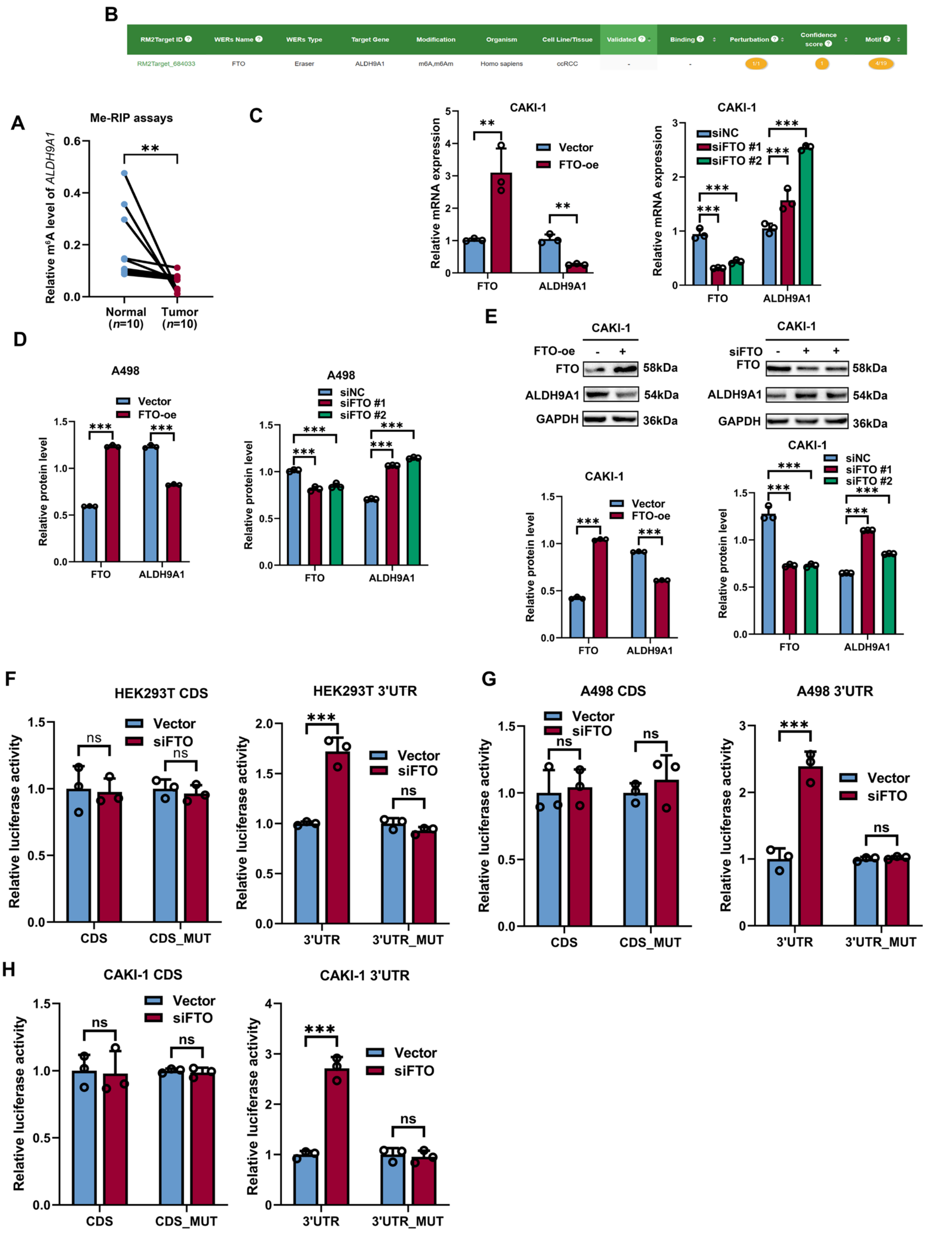

(A) The m^{6}A modification levels of the mRNA of *ALDH9A1* in 10 pairs of ccRCC tissues and corresponding normal tissue (paired-samples *t*-test for

statistics).

(B) The prediction of the upstream m^6A modification molecule of ALDH9A1 in ccRCC based on the R2MTarget website (rm2target.canceromics.org/#/home).

(C) The mRNA levels of *ALDH9A1* in FTO-overexpression and FTO-deficient CAKI-1 cells, respectively, compared with the control group (n = 3) (independent-samples t-test and ANOVA for statistics).

(D) The protein expression of ALDH9A1 in FTO-overexpression and FTO-deficient A498 cells, respectively, compared with the control group (n = 3) (independent-samples t-test and ANOVA for statistics).

(E) The protein expression of ALDH9A1 in FTO-overexpression and FTO-deficient CAKI-1 cells, respectively, compared with the control group (n = 3) (independent-samples t-test and ANOVA for statistics).

(F-H) Relative luciferase activity of the wild-type and mutant *ALDH9A1* CDS reporter vectors, as well as the wild-type and mutant *ALDH9A1* 3′-UTR reporter vectors in FTO-deficient HEK293T, A498, and CAKI-1 cells, compared with the control group (n = 3) (independent-samples t-test for statistics).

Results represented at least three independent experiments (*P < 0.05, **P < 0.01, ***P < 0.001).

Supplementary figure 5: ALDH9A1 repressed the activation of the AKT-mTOR pathway to restrain the proliferation, and invasion, and promote the apoptosis in ccRCC.

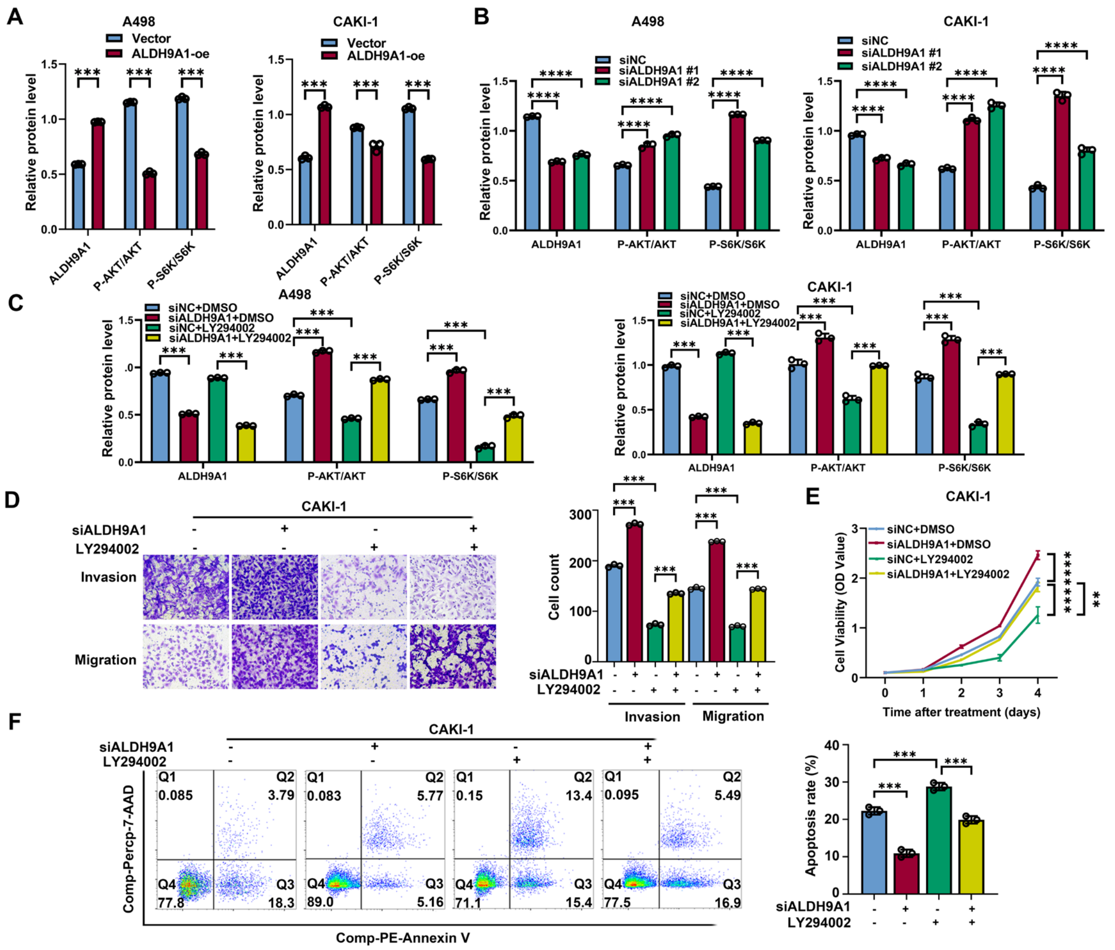

(A-B) The protein expression of phosphorylated AKT, S6K, as well as

total AKT and S6K in ALDH9A1-overexpression and ALDH9A1-deficiency ccRCC cells and control ccRCC cells (n = 3) (independent-samples t-test and ANOVA for statistics).

(C) The protein expression of phosphorylated AKT, S6K, as well as total AKT and S6K were assessed in the indicated cell lines (n = 3) (ANOVA for statistics). ccRCC cells with or without ALDH9A1-deficient were treated with 20 μmol/L LY294002 (an inhibitor of PI3K-AKT pathway) or DMSO.

(D) Transwell assays were performed to evaluate the migratory and invasive capacity of the indicated CAKI-1 cells (n = 3) (ANOVA for statistics). ccRCC cells with or without ALDH9A1-deficient were treated with 20 μmol/L LY294002 (an inhibitor of PI3K-AKT pathway) or DMSO.

(E) Cell proliferation curves were generated using CCK8 assays for the indicated CAKI-1 cells (n = 3) (ANOVA for statistics). ccRCC cells with or without ALDH9A1-deficient were treated with 20 μmol/L LY294002 (an inhibitor of PI3K-AKT pathway) or DMSO.

(F) Flow cytometry assay showed the proportion of apoptotic cells for the indicated CAKI-1 cells (n = 3) (ANOVA for statistics). ccRCC cells with or without ALDH9A1-deficient were treated with 20 μmol/L LY294002 (an inhibitor of PI3K-AKT pathway) or DMSO. Comp-PE-Annexin V means that Annexin V was compensated by negative control and single

positive control, and Comp-Percp-7-AAD means that 7-AAD was compensated by negative control and single positive control.

Results represented at least three independent experiments (**P* < 0.05, ***P* < 0.01, ****P* < 0.001).

Supplementary figure 6: ALDH9A1 repressed the maturation of SREBP1 and downregulated the mRNA levels of SREBP1 target genes.

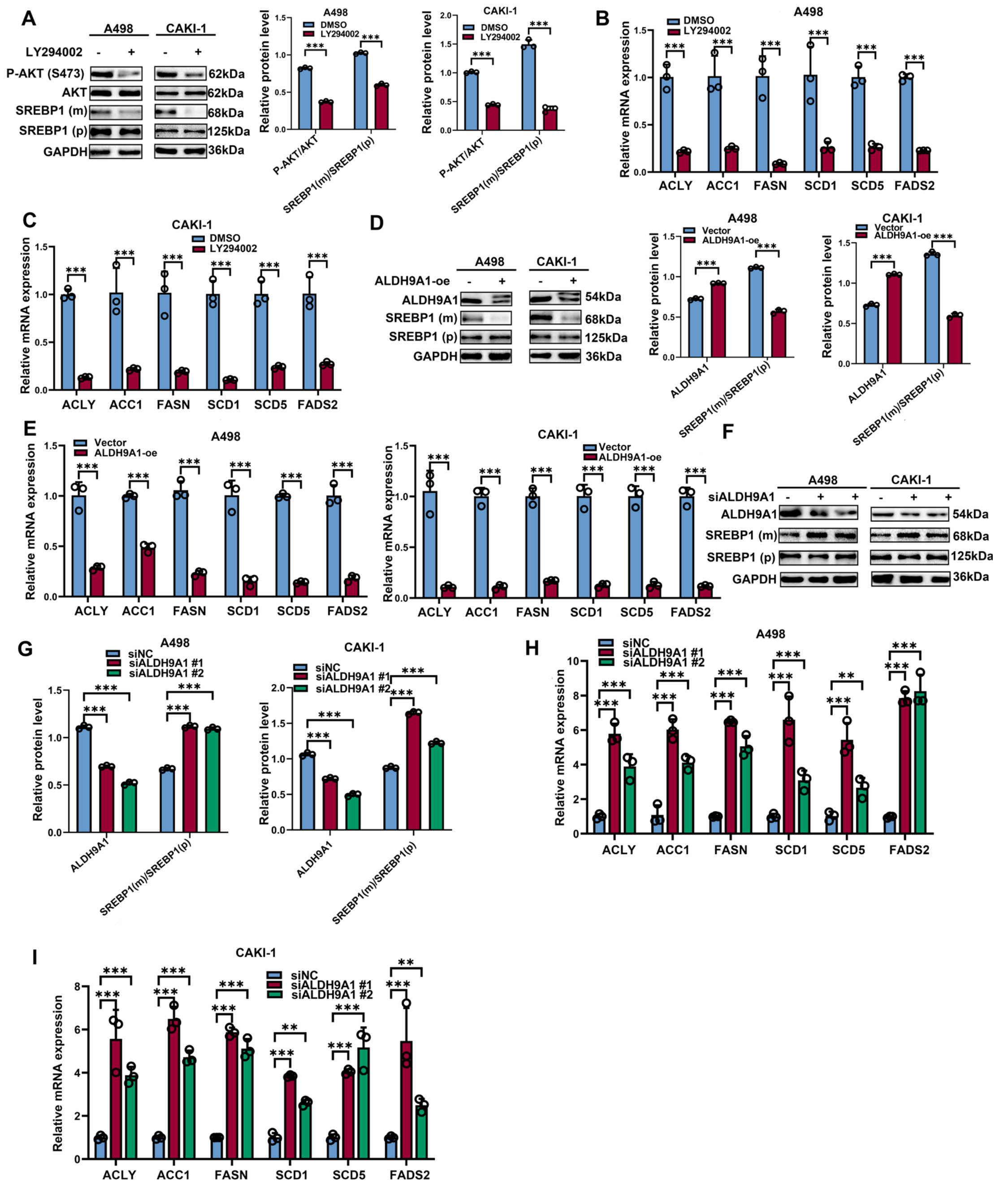

(A) The protein expression of precursor SREBP1 and mature SREBP1 in ccRCC cells treated with 20 μmol/L LY294002 (an inhibitor of PI3K-

AKT pathway) or DMSO (n = 3) (independent-samples t-test for statistics).

(B-C) The mRNA levels of SREBP1 target genes, including *ACLY*, *ACC1*, *FASN*, *SCD1*, *SCD5* and *FADS2* were assessed in ccRCC cells treated with 20 μmol/L LY294002 (an inhibitor of PI3K-AKT pathway) or DMSO (n = 3) (independent-samples t-test for statistics).

(D) The protein expression of precursor SREBP1 and mature SREBP1 in ALDH9A1-overexpressing ccRCC cells (independent-samples t-test for statistics).

(E) The mRNA levels of SREBP1 target genes, including *ACLY*, *ACC1*, *FASN*, *SCD1*, *SCD5* and *FADS2* were assessed in ALDH9A1-overexpressing ccRCC cells (n = 3) (independent-samples t-test for statistics).

(F-G) The protein expression of precursor SREBP1 and mature SREBP1 in ALDH9A1-deficient ccRCC cells (ANOVA for statistics).

(H-I) The mRNA levels of SREBP1 target genes, including *ACLY*, *ACC1*, *FASN*, *SCD1*, *SCD5* and *FADS2* were assessed in ALDH9A1- deficient ccRCC cells (n = 3) (ANOVA for statistics).

Results represented at least three independent experiments (*$P < 0.05$, **$P < 0.01$, ***$P < 0.001$).

Supplementary figure 7: ALDH9A1 declined the accumulation of lipid through repressing the activation of AKT-mTOR-SREBP1 pathway.

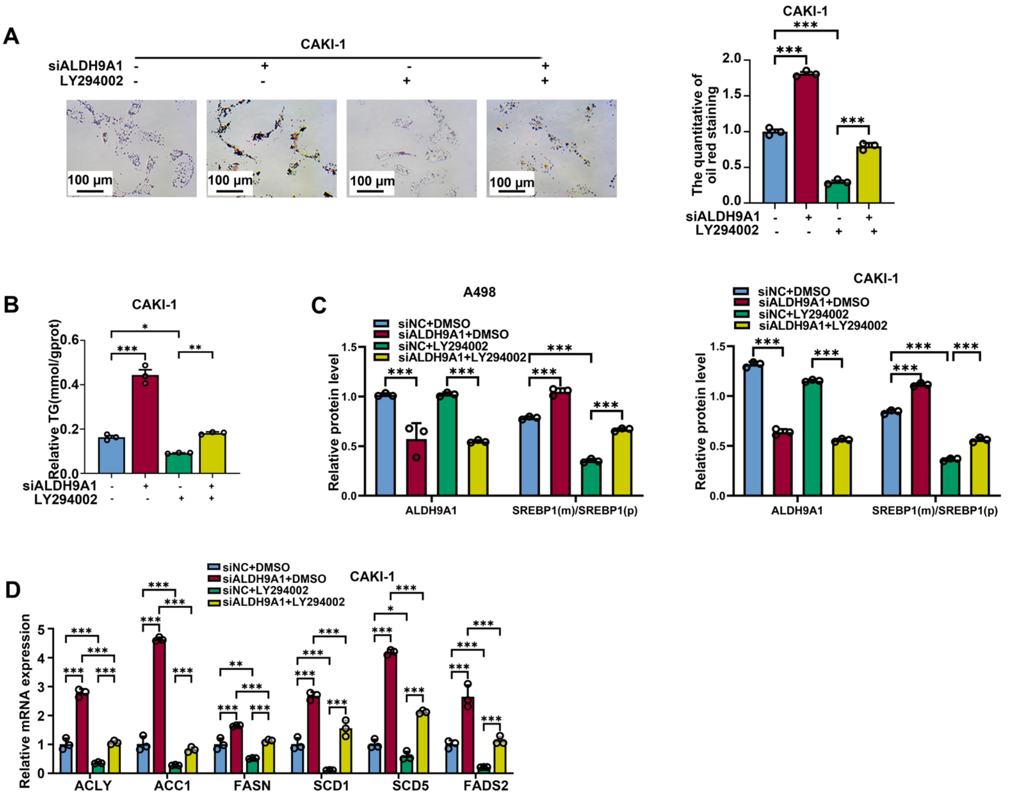

(A) Photomicrographs of Oil red staining were performed in indicated cell lines (*n* = 3) (ANOVA for statistics). ccRCC cells with or without ALDH9A1-deficient were treated with 20 μmol/L LY294002 (an inhibitor of PI3K-AKT pathway) or DMSO.

(B) Relative TG (mmol/gprot) levels were assessed in indicated cell lines (*n* = 3) (ANOVA for statistics). ccRCC cells with or without ALDH9A1-deficient were treated with 20 μmol/L LY294002 (an inhibitor of PI3K-AKT pathway) or DMSO.

(C) The protein expression of precursor SREBP1 and mature SREBP1in

the indicated cell lines (*n* = 3) (ANOVA for statistics). ccRCC cells with or without ALDH9A1-deficient were treated with 20 μmol/L LY294002 (an inhibitor of PI3K-AKT pathway) or DMSO.

(D) The mRNA levels of SREBP1 target genes, including *ACLY*, *ACC1*, *FASN*, *SCD1*, *SCD5* and *FADS2* were asses sed in the indicated cell lines (*n* = 3) (ANOVA for statistics). ccRCC cells with or without ALDH9A1-deficient were treated with 20 μmol/L LY294002 (an inhibitor of PI3K-AKT pathway) or DMSO.

Results represented at least three independent experiments ($*P < 0.05$, $**P < 0.01$, $***P < 0.001$).

Supplementary figure 8: ALDH9A1 upregulated the mRNA expression of IQGAP2, thereby enhancing protein abundance.

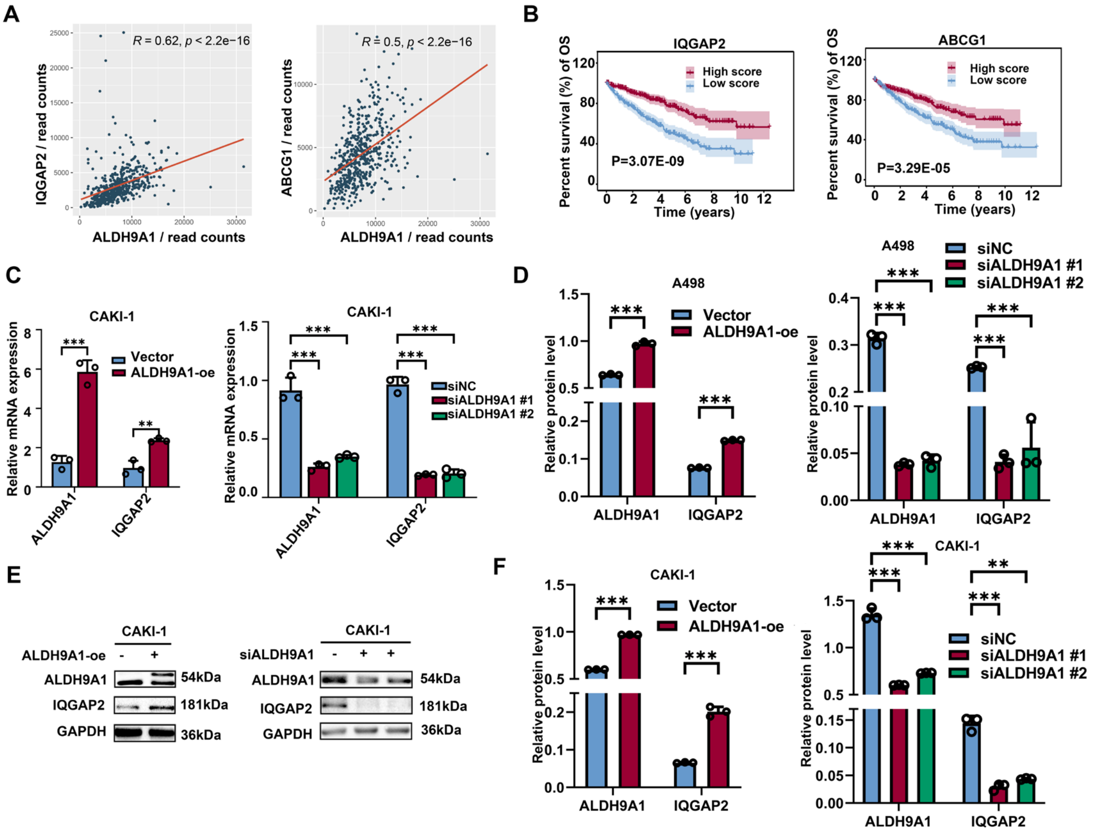

(A) The scatter plot depicting the correlation analyses between *ALDH9A1* and *IQGAP2*, *ABCG1* based on the count data of TCGA-KIRC cohort (Pearson correlation coefficient for statistics).

(B) The Kaplan-Meier curves of IQGAP2 and ABCG1 in patients with ccRCC for determining overall survival (OS) (log-rank for statistics).

(C) The mRNA levels of *IQGAP2* in ALDH9A1-overexpressing and ALDH9A1-deficient CAKI-1 cells compared with the control group (n = 3) (independent-samples t-test and ANOVA for statistics).

(D) The protein expression of IQGAP2 in ALDH9A1-overexpressing and

ALDH9A1-deficient A498 cells compared with the control group (n=3) (independent-samples *t*-test and ANOVA for statistics).

(E-F) The protein expression of IQGAP2 in ALDH9A1-overexpressing and ALDH9A1-deficient CAKI-1 cells compared with the control group ($n = 3$) (independent-samples *t*-test and ANOVA for statistics).

Results represented at least three independent experiments (*$P < 0.05$, **$P < 0.01$, ***$P < 0.001$).

Supplementary figure 9: IQGAP2 repressed the proliferation, invasion and promote the apoptosis of ccRCC cells.

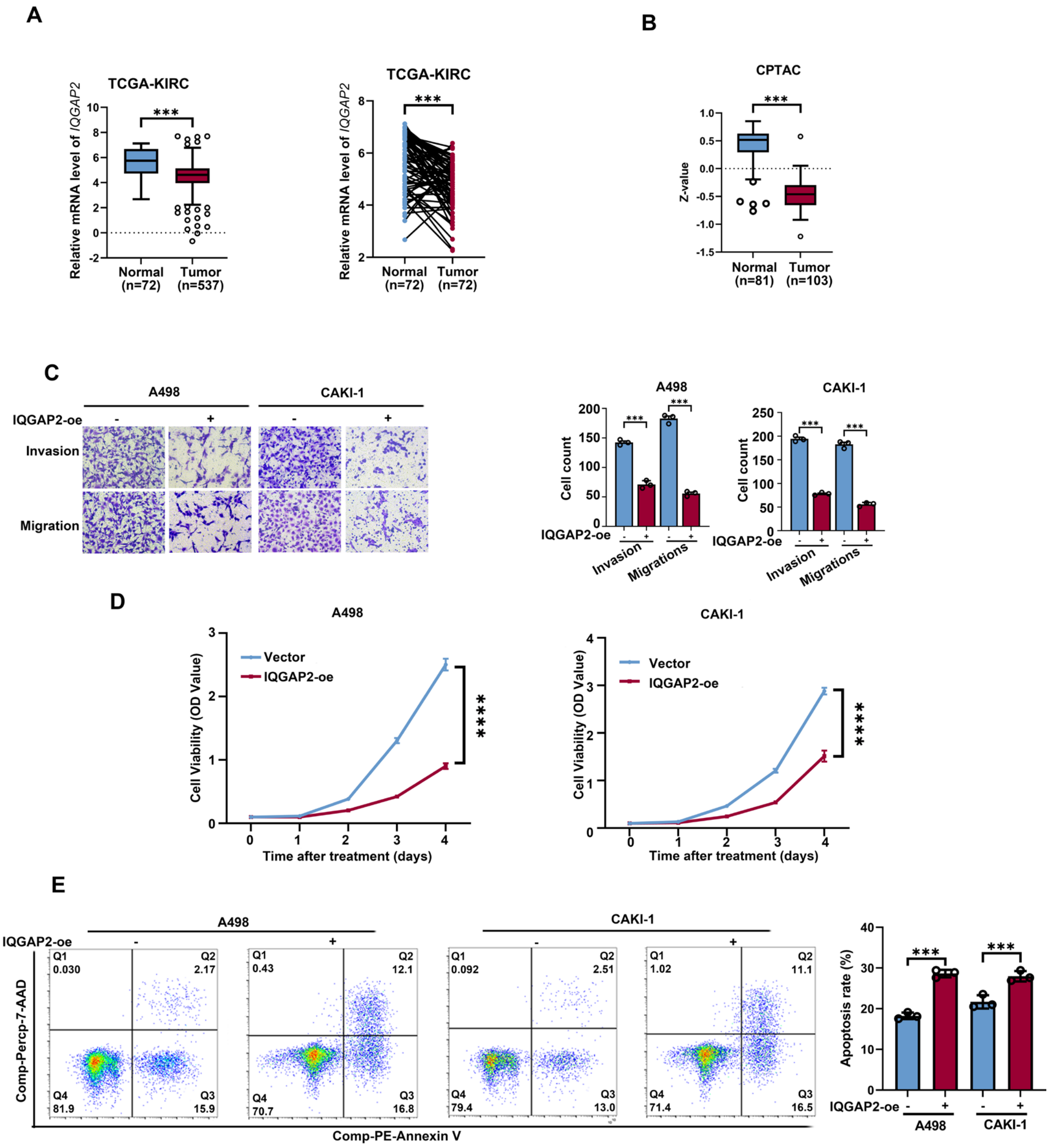

(A) The mRNA levels of *IQGAP2* in ccRCC tissues and normal tissues based on TCGA-KIRC cohort (independent-samples *t*-test and paired-

samples *t*-test for analysis).

(B) The protein expression of IQGAP2 in ccRCC tissues and normal tissues based on CPTAC-PDC000127 cohort (independent-samples *t*-test for statistics).

(C) Transwell assays were performed to evaluate the migratory and invasive capacity of IQGAP2-overexpressing ccRCC cells ($n = 3$) (independent-samples *t*-test for statistics).

(D) Cell proliferation curves were generated using CCK8 assays in IQGAP2-overexpressing ccRCC cells ($n = 3$) (independent-samples *t*-test for statistics).

(E) Flow cytometry assay determining the proportion of apoptotic cells in IQGAP2-overexpressed ccRCC cells compared to control cells ($n = 3$) (independent-samples *t*-test for statistics for statistics). Comp-PE-Annexin V means that Annexin V was compensated by negative control and single positive control, and Comp-Percp-7-AAD means that 7-AAD was compensated by negative control and single positive control.

Results represented at least three independent experiments (*$P < 0.05$, **$P < 0.01$, ***$P < 0.001$).

Supplementary figure 10: IQGAP2 repressed the lipid accumulation of ccRCC cells.

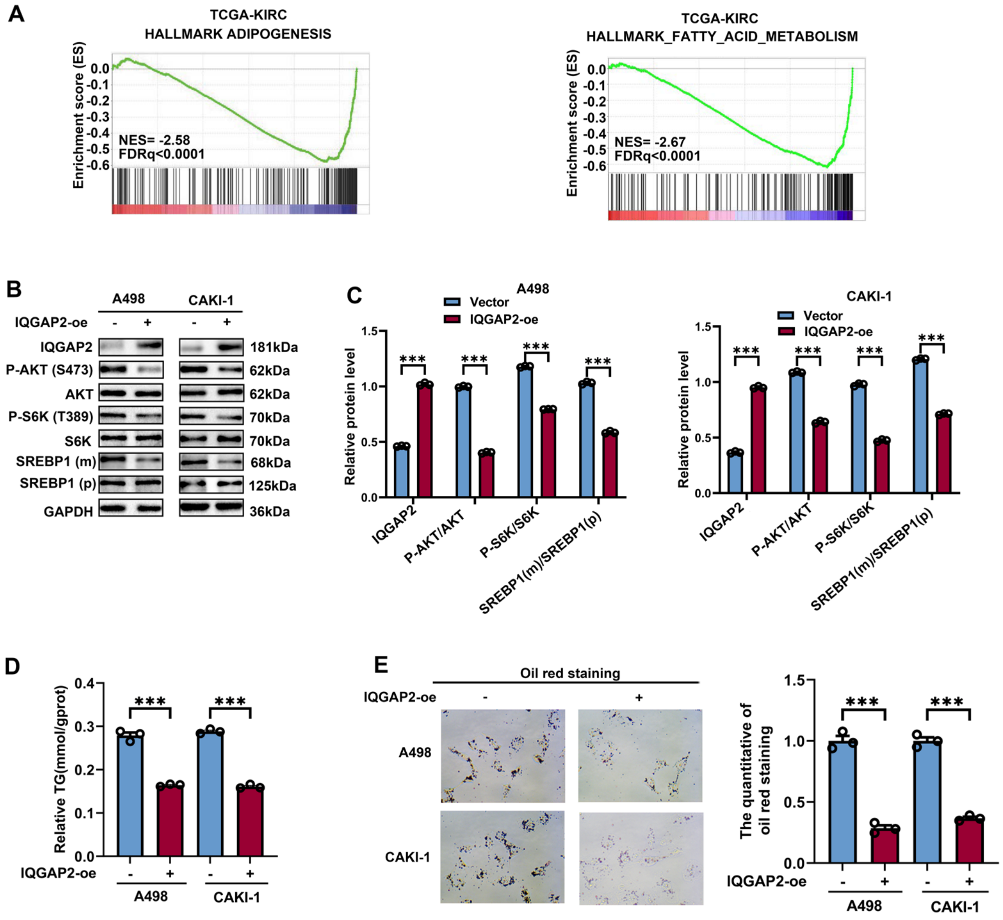

(A) Results from GSEA revealed the association between lipid metabolism in ccRCC and the mRNA level of IQGAP2 based on TCGA-KIRC cohort. Statistical significance was determined at FDRq < 25%.

(B-C) The protein expression of phosphorylated AKT, phosphorylated S6K, total AKT and S6K as well as precursor SREBP1, mature SREBP were assessed in IQGAP2-overexpressing ccRCC cells (n = 3) (independent-samples t-test for statistics for statistics).

(D) Relative TG (mmol/gprot) levels in IQGAP2-overexpressed group compared with the control group (*n* =3) (independent-samples *t*-test for statistics).

(E) Photomicrographs of Oil red staining in IQGAP2- overexpressed cells compared with the control group (*n* = 3) (independent-samples *t*-test for statistics).

Results represented at least three independent experiments (*$P < 0.05$, **$P < 0.01$, ***$P < 0.001$).

Supplementary figure 11: ALDH9A1 inhibited the proliferation, invasiveness and promoted the apoptosis of ccRCC in IQGAP2-dependent manner.

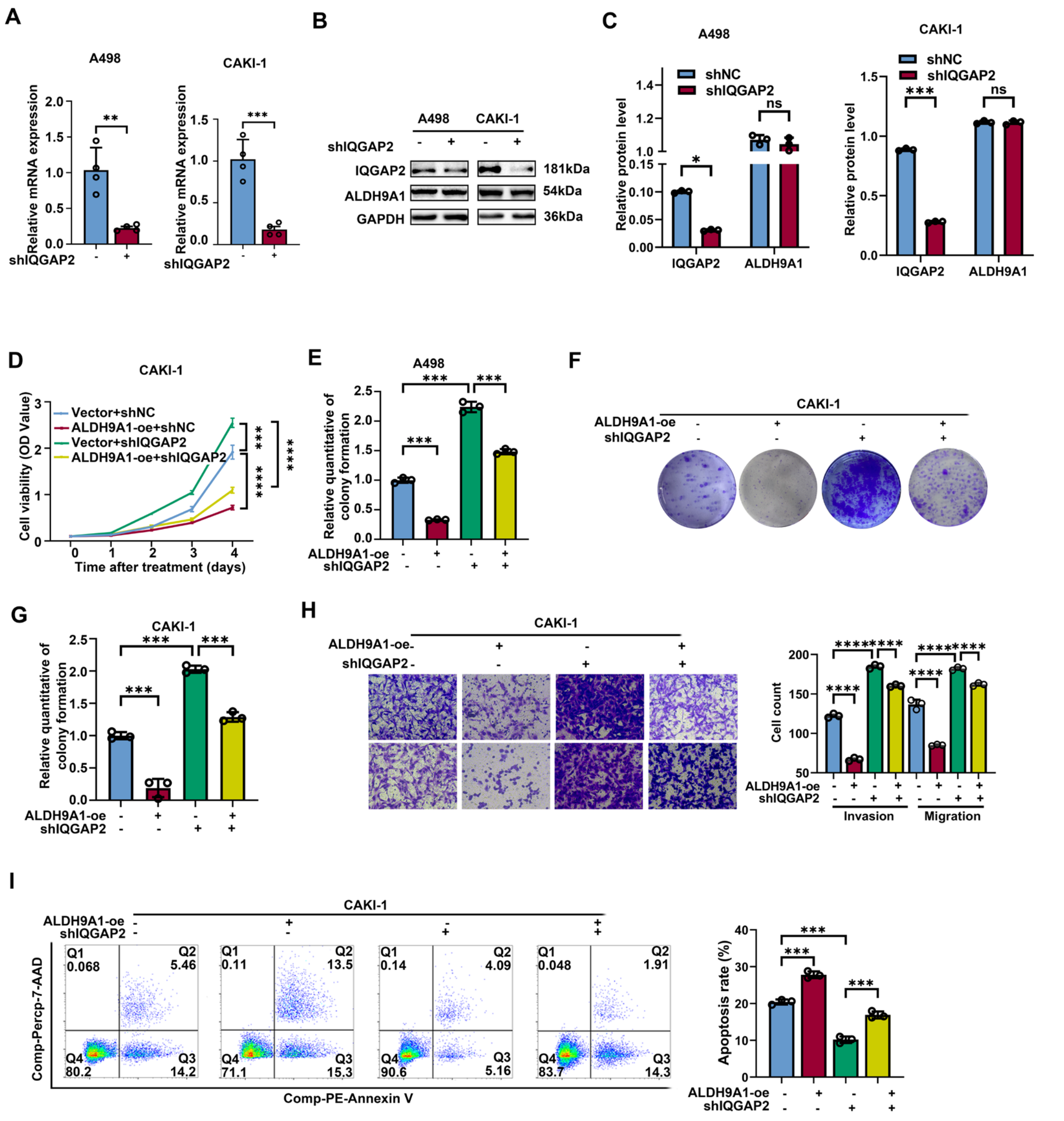

(A-C) ccRCC cells with IQGAP2 knockdown were constructed by lentivirus containing shRNA. The knockdown of IQGAP2 were verified at the protein and mRNA levels using Western blotting and qPCR, respectively (*n* = 3) (independent-samples *t*-test for statistics).

(D) Cell proliferation curves were generated using CCK8 assays for the indicated ccRCC cells (*n* = 3) (ANOVA for statistics). ccRCC cells with or without ALDH9A1-overexpressing were transfected with IQGAP2 knockdown lentivirus or vector.

(E) The quantitative result of colony formation assays in the indicated A498 cells (*n* = 3) (ANOVA for statistics). A498 cells with or without ALDH9A1-overexpressing were transfected with IQGAP2 knockdown lentivirus or vector.

(F-G) The colony formation assays in the indicated CAKI-1 cells (*n* = 3) (ANOVA for statistics). CAKI-1 cells with or without ALDH9A1-overexpressing were transfected with IQGAP2 knockdown lentivirus or vector.

(H) Transwell assays were performed to evaluate the migratory and invasive capacity of the indicated CAKI-1 cells (*n* = 3) (ANOVA for statistics). CAKI-1 cells with or without ALDH9A1-overexpressing were transfected with IQGAP2 knockdown lentivirus or vector.

(I) Flow cytometry assay showed the proportion of apoptotic cells for the indicated CAKI-1 cells (*n* = 3) (ANOVA for statistics). CAKI-1 cells with or without ALDH9A1-overexpressing were transfected with IQGAP2 knockdown lentivirus or vector. Comp-PE-Annexin V means that Annexin V was compensated by negative control and single positive control, and Comp-Percp-7-AAD means that 7-AAD was compensated

by negative control and single positive control.

Results represented at least three independent experiments (*$P < 0.05$, **$P < 0.01$, ***$P < 0.001$).

Supplementary figure 12: ALDH9A1 inhibited the lipid accumulation of ccRCC in IQGAP2-dependent manner.

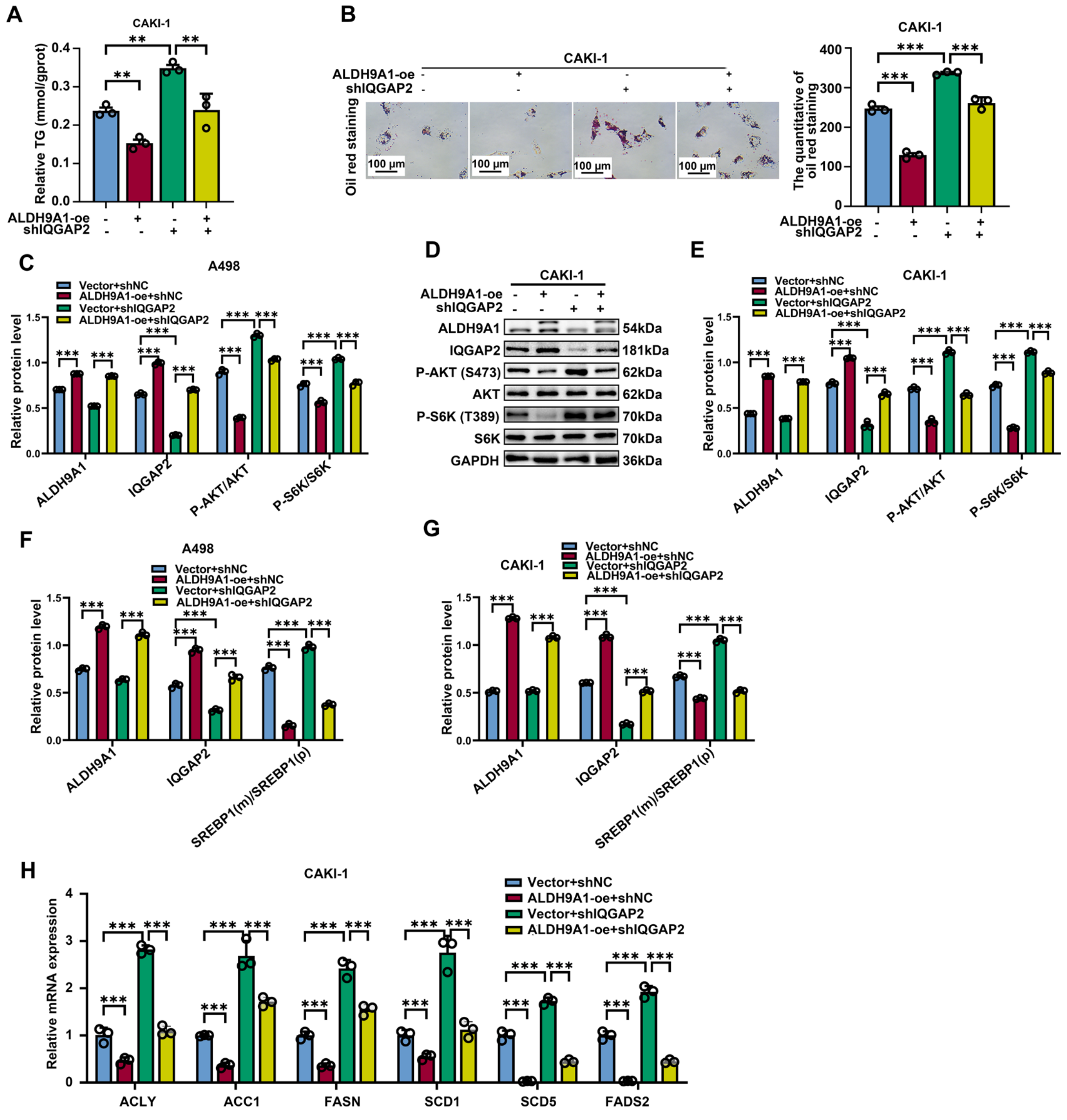

(A) Relative TG (mmol/gprot) levels were assessed in indicated CAKI-1 (n = 3) (ANOVA for statistics). CAKI-1 cells with or without ALDH9A1-overexpressing were transfected with IQGAP2 knockdown lentivirus or vector.

(B) Photomicrographs of Oil red staining were performed in indicated

CAKI-1 lines (n = 3) (ANOVA for statistics). CAKI-1 cells with or without ALDH9A1-overexpressing were transfected with IQGAP2 knockdown lentivirus or vector.

(C) The protein expression of phosphorylated AKT, S6K, as well as total AKT and S6K were assessed in the indicated A498 lines (n = 3) (ANOVA for statistics). A498 cells with or without ALDH9A1-overexpressing were transfected with IQGAP2 knockdown lentivirus or vector.

(D-E) The protein expression of phosphorylated AKT, S6K, as well as total AKT and S6K were assessed in the indicated CAKI-1 lines (n = 3) (ANOVA for statistics). CAKI-1cells with or without ALDH9A1-overexpressing were transfected with IQGAP2 knockdown lentivirus or vector.

(F-G) The protein expression of precursor SREBP1 and mature SREBP1 in the indicated cell lines (n = 3) (ANOVA for statistics). ccRCC cells with or without ALDH9A1-overexpressing were transfected with IQGAP2 knockdown lentivirus or vector.

(H) The mRNA levels of SREBP1 target genes, including *ACLY*, *ACC1*, *FASN*, *SCD1*, *SCD5* and *FADS2* were assessed in the indicated CAKI-1 lines (n = 3) (ANOVA for statistics). CAKI-1cells with or without ALDH9A1-overexpressing were transfected with IQGAP2 knockdown lentivirus or vector.

Results represented at least three independent experiments ($*P < 0.05$, $**P < 0.01$, $***P < 0.001$).

Supplementary figure 13: ALDH9A1 interacted with NPM1.

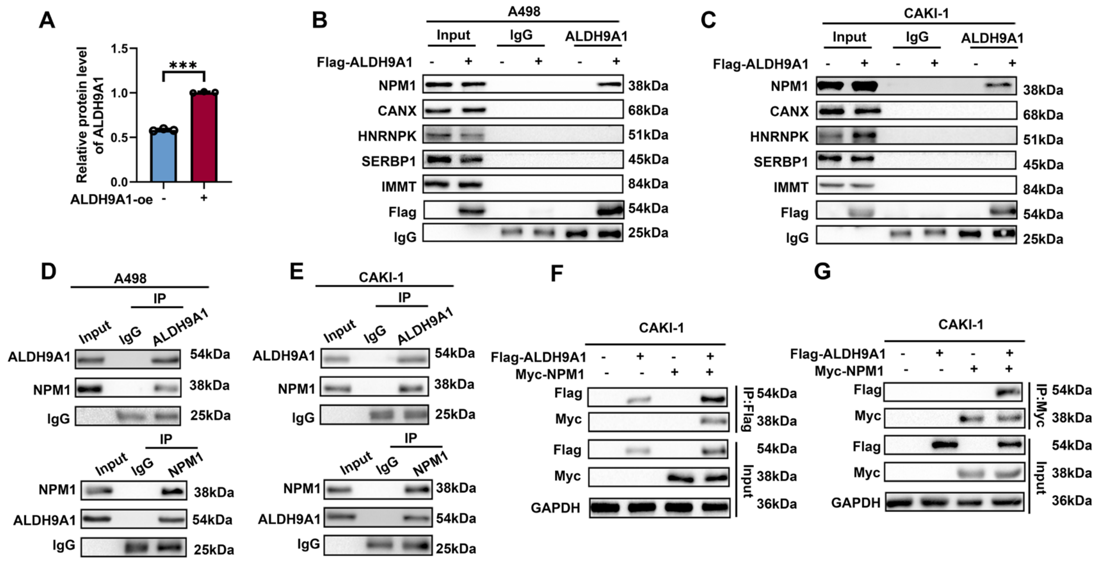

(A) ALDH9A1-overexpressed cell lines were constructed in HEK293T by Flag- ALDH9A1 lentivirus. The overexpression of ALDH9A1 was verified at the protein level using Western blotting (n = 3) (independent-samples t-test for statistics).

(B-C) The ALDH9A1-NPM1 interaction was determined by Co-IP assays in A498 and CAKI-1 cells with flag- ALDH9A1 overexpression (n = 3).

(D-E) The endogenous ALDH9A1-NPM1 interaction was determined by Co-IP assays in A498 and CAKI-1 cells (n = 3).

(F-G) The exogenous ALDH9A1-NPM1 interaction was determined by Co-IP assays in CAKI-1 cells overexpressed Flag-ALDH9A1 and/or Myc-NPM1 (n = 3).

Results represented at least three independent experiments (*P < 0.05, **P < 0.01, ***P < 0.001).

Supplementary figure 14: ALDH9A1 altered the cellular localization of NPM1.

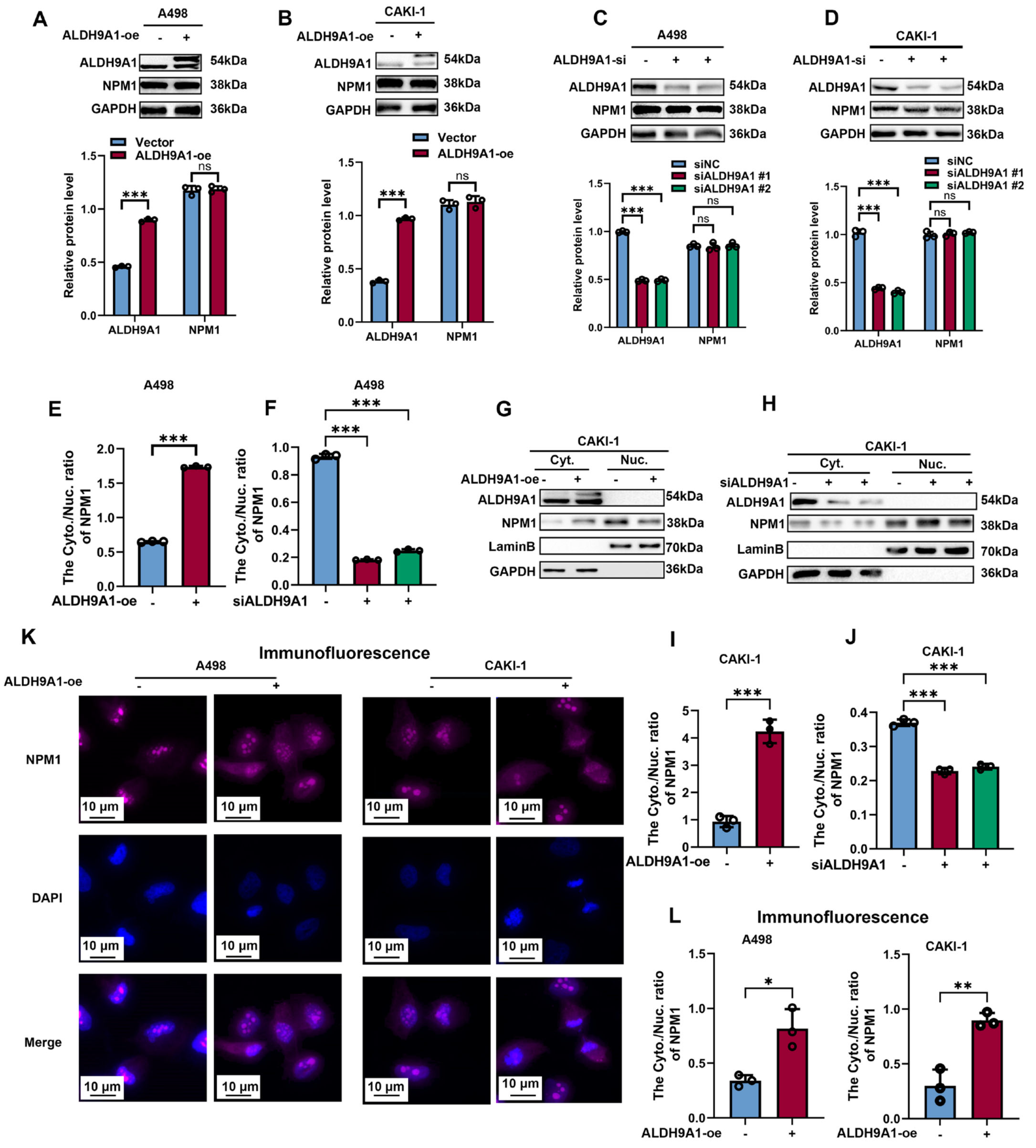

(A-B) The protein expression of NPM1 in ALDH9A1-overexpressing ccRCC cell lines compared with the control group (*n* = 3) (independent-samples *t*-test for statistics).

(C-D) The protein expression of NPM1 in ALDH9A1-deficient ccRCC cell lines compared with the control group ($n = 3$) (ANOVA for statistics).

(E) Quantification of the cytoplasm/nuclear ratio of NPM1 in A498 cells with ALDH9A1 overexpression ($n = 3$) (independent-samples t-test for statistics).

(F) Quantification of the cytoplasm/nuclear ratio of NPM1 in A498 cells with ALDH9A1 deficiency ($n = 3$) (ANOVA for statistics).

(G) The cytoplasm/nuclear ratio of NPM1 in CAKI-1 cells with ALDH9A1 overexpression ($n = 3$).

(H) The cytoplasm/nuclear ratio of NPM1 in CAKI-1 cells with ALDH9A1 deficiency ($n = 3$).

(I) Quantification of the cytoplasm/nuclear ratio of NPM1 in CAKI-1 cells with ALDH9A1 overexpression ($n = 3$) (independent-samples t-test for statistics).

(J) Quantification of the cytoplasm/nuclear ratio of NPM1 in CAKI-1 cells with ALDH9A1 deficiency ($n = 3$) (ANOVA for statistics).

(K) Immunofluorescence assays display subcellular localization of NPM1 after ALDH9A1 overexpression ($n = 3$).

(L) Quantification of Immunofluorescence assays display subcellular localization of NPM1 after ALDH9A1 overexpression ($n = 3$) (independent-samples t-test for statistics).

Results represented at least three independent experiments (*$P < 0.05$, **$P < 0.01$, ***$P < 0.001$).

Supplementary figure 15: ALDH9A1 upregulated the expression of IQGAP2 in NPM1-dependent manner.

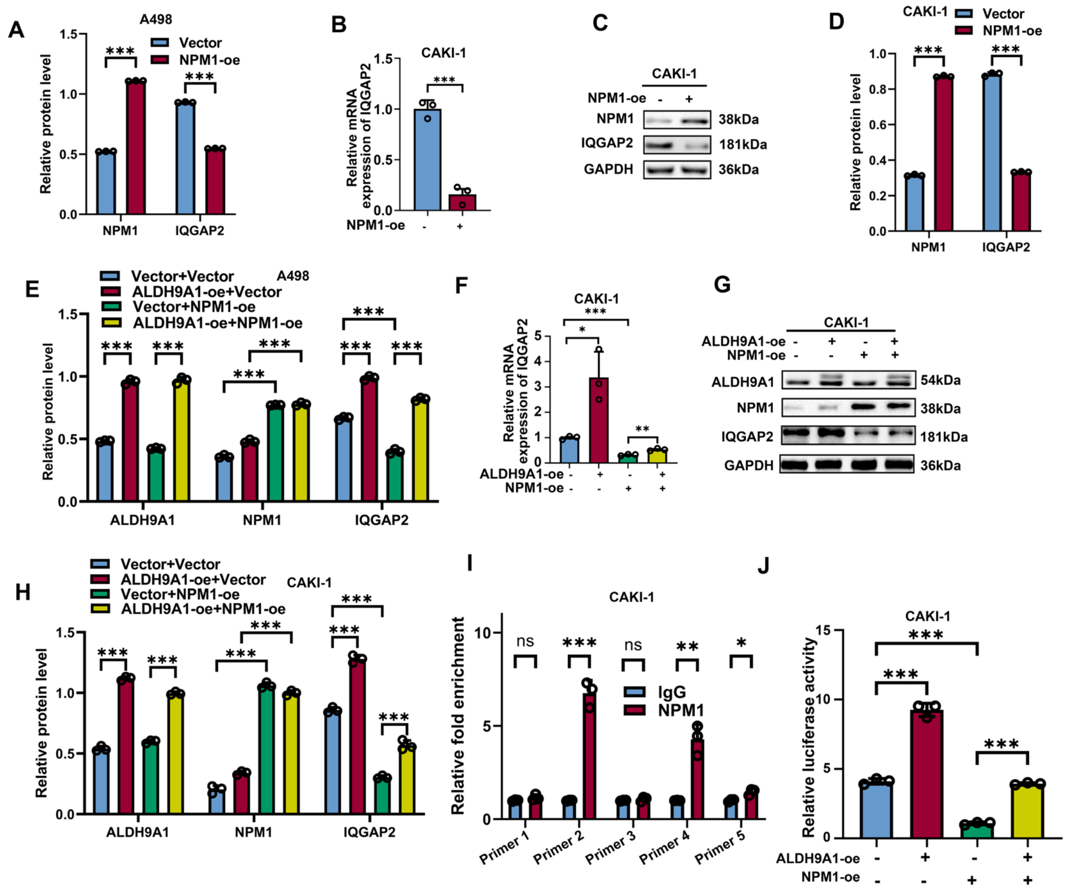

(A) The protein expression of IQGAP2 in NPM1-overexpressing A498 cells (n = 3) (independent-samples t-test for statistics).

(B) The mRNA levels of *IQGAP2* in NPM1-overexpressing CAKI-1 cells (n = 3) (independent-samples t-test for statistics).

(C-D) The protein expression of IQGAP2 in NPM1-overexpressing CAKI-1 cells (n = 3) (independent-samples t-test for statistics).

(E) The protein expression of IQGAP2 in indicated ccRCC cells (n = 3) (ANOVA for statistics). A498 cells with or without ALDH9A1-

overexpressing were transfected with NPM1 overexpressing plasmid or vector.

(F-H) The mRNA levels and protein expression of IQGAP2 in indicated ccRCC cells (*n* = 3) (ANOVA for statistics). CAKI-1 cells with or without ALDH9A1-overexpressing were transfected with NPM1 overexpressing plasmid or vector.

(I) ChIP-PCR assays of the NPM1 binding sites in the promoter of *IQGAP2* in ccRCC cells (*n* = 3) (independent-samples *t*-test for statistics).

(J) Relative luciferase activity of the IQGAP2 luciferase reporter vector in indicated ccRCC cells (*n* = 3) (ANOVA for statistics). ccRCC cells with or without ALDH9A1-overexpressing were transfected with NPM1 overexpressing plasmid or vector.

Results represented at least three independent experiments (*P<0.05, **P<0.01, ***P<0.001).

Supplementary figure 16: The ALDH9A1-IQGAP2 axis suppressed tumor progression and lipid accumulation in vivo.

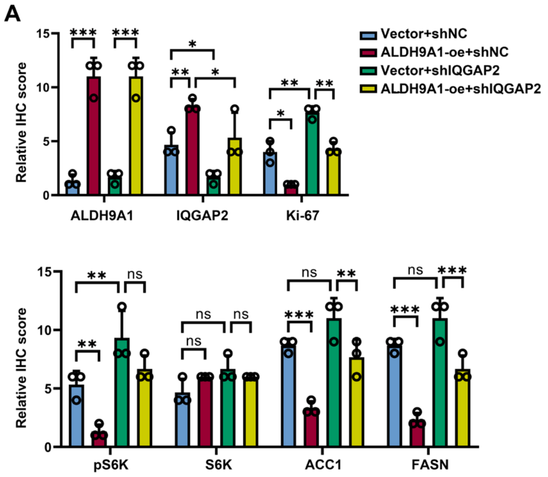

(A) IHC staining for ALDH9A1, IQGAP2, ki-67, phosphorylated S6K, S6K, ACC and FASN in the isolated tumor xenograft of the Vector + shNC, ALDH9A1-oe + shNC, Vector + shIQGAP2, and ALDH9A1-oe + shIQGAP2 groups (n = 3) (ANOVA for statistics).
