# Supplementary material for: N6-methyladenosine-modified ALDH9A1 modulates lipid accumulation and tumor progression in clear cell renal cell carcinoma through the NPM1/IQGAP2/AKT signaling pathway
Source: Cell Death Dis. 2024 Jul 22;15(7):520. doi: 10.1038/s41419-024-06896-z (PMC11263707; doi:10.1038/s41419-024-06896-z)
Supplement: Supplementary file 2 — supplementary table 1 [file 41419_2024_6896_MOESM2_ESM.pdf]

Gene		Sequences (5'--3')
GAPDH	Forward	GAGTCAACGGATTTGGTCGT
	Reverse	GACAAGCTTCCCGTTCTCAG
ALDH9A1	Forward	TTGGAACTTGGAGGCAAATC
	Reverse	GTGTGGTCGGTTGATGAGTG
IQGAP2	Forward	TCCAGAGGAATCTTCGGACG
	Reverse	GGTCTGCTGAAGTTTTGCCA
FTO	Forward	GCCTTTCTCACACTGCACAA
	Reverse	TAGACCCTTTCACTGGCCAG
18S rRNA	Forward	GTAACCCGTTGAACCCCATT
	Reverse	CCATCCAATCGGTAGTAGCG
ACC1	Forward	ATGTCTGGCTTGCACCTAGTA
	Reverse	CCCCAAAGCGAGTAACAAATTCT
FASN	Forward	CCGAGACACTCGTGGGCTA
	Reverse	CTTCAGCAGGACATTGATGCC
SCD-1	Forward	TCTAGCTCCTATACCACCACCA
	Reverse	TCGTCTCCAACTTATCTCCTCC
SCD-5	Forward	TGGCTGTTTGTTCGCAAGC
	Reverse	GGACCACAGGATCAGCAAGC
FADS2	Forward	GACCACGGCAAGAACTCAAAG
	Reverse	GAGGGTAGGAATCCAGCCATT
ACLY	Forward	ATCGGTTCAAGTATGCTCGGG
	Reverse	GACCAAGTTTTCCACGACGTT
NPM1	Forward	GGTGGTTCTCTTCCCAAAGTGGA
	Reverse	GACTTCCTCCACTGCCAGAGATC

Antibodies and reagents	Vendors	Cat#	Working concentration (application)
**Antibodies**			
ALDH9A1	ABclonal	A18098	1:1000 (WB), 1:100 (IHC)
ALDH9A1	SANTA CRUZ	sc-398054	1:50 (IP)
IQGAP2	ABclonal	A20956	1:500 (WB), 1:100 (IHC)
NPM1	proteintech	60096-1-Ig	1:1000 (WB), 1:100 (IP),1:20 (ChIP)
P-AKT (S473)	ABclonal	AP0637	1:1000 (WB)
AKT	ABclonal	A18120	1:1000 (WB)
P-S6K (T389)	ABclonal	A16658	1:1000 (WB), 1:100 (IHC)
S6K	ABclonal	AP0564	1:1000 (WB), 1:100 (IHC)
SREBP1	SANTA CRUZ	sc-13551	1:500 (WB)
FTO	ABclonal	A3861	1:1000 (WB)
FASN	ABclonal	A0461	1:100 (IHC)
ACC1	ABclonal	A15606	1:100 (IHC)
GAPDH	ABclonal	AC001	1:5000 (WB)
Lamin B1	ABclonal	A11495	1:1000 (WB)
DDDDK-Tag	ABclonal	AE063	1:1000 (WB), 1:100 (IP)
SERBP1	ABclonal	A14870	1:1000 (WB)
HNRNPK	ABclonal	A0772	1:1000 (WB)
CANK	ABclonal	A4846	1:1000 (WB)
IMMT	ABclonal	A2751	1:1000 (WB)
Flag	ABclonal	AE005	1:1000 (WB), 1:100 (IP)
Myc	Cell Signaling Technology	#2276S	1:1000 (WB), 1:100 (IP)
N6-Methyladenosine	Beyotime	AF7407	1:100 (MeRIP)
Ki-67	Cell Signaling Technology	#9449	1:100 (IHC)
IgG	Cell Signaling Technology	#2729	1:100 (ChIP)
Anti-Mouse	ABclonal	AS003	1:2000 (WB)
Anti-Rabbit	ABclonal	AS014	1:2000 (WB)
**reagents**			
actinomycin D	MCE	HY-17559	5 μg/mL (mRNA stability assays)
Decitabine (5-AZA)	MCE	HY-A0004	2.5μM (DNA methylation)
LY294002	MCE	HY-10108	20 μmol/L (AKT inhibiton assays)

Gene	Traanscripts/Sequences (5'- -3')
siALDH9A<br>1#1	5'-GCAUGGAGCGUUGCCGAAUTT-<br>3'
siALDH9A<br>1#2	5'-GCUCCCAGGUGGAUCGUUUTT-<br>3'
shIQGAP2	5'-GCTCCTACCTACTGCGAAT-3'
siFTO#1	5'-GCAGAATGTCTGTGACGAT-3'
siFTO#2	5'-CCTGAACACCAGGCTCTTT-3'
