# Supplementary material for: N6-methyladenosine-modified ALDH9A1 modulates lipid accumulation and tumor progression in clear cell renal cell carcinoma through the NPM1/IQGAP2/AKT signaling pathway
Source: Cell Death Dis. 2024 Jul 22;15(7):520. doi: 10.1038/s41419-024-06896-z (PMC11263707; doi:10.1038/s41419-024-06896-z)
Supplement: Supplementary file 3 — supplementary table 2 [file 41419_2024_6896_MOESM3_ESM.pdf]

Supplementary table 2

Prediction of NPM1 protein binding with 6 sites within *IQGAP2* gene promoters

>hg38_knownGene_ENST00000274364.11 range=chr5:76401285-76403284 5'pad=0 3'pad=0 strand=+ repeatMasking=none

TTTCTTTCCCTTGGATGCTCTCCATCCAGCTTTTACCACACTTCCACTTCAAGCCACACT
TTCAAACCCTGTCACCTTCCTGGACCATTTTAGTTCAGAGTGATTTTTTCACCCACCTCT
AAATATATAGACTACTCATTTATTTGGGCTCTAAGTCATACACTCATACGAAAATTGTTAT
GTGTAAATACTTAATTGTTAACACTTGACAAATGCAACAACAGAAATGGAATGAACTTT
AGACATCATTTAATTCAGCATCTCCTGACATGCACGAAAATCCCCTTCCACATTTCTCCT
TTGGGTGAATATCAATTGACAGGGTGCCGAGCCTCAAATGTGGCCATTTCGCCTCTGAA
TTGTTCTTAGACTGAGAGGCATCTGCTTTTTAAGAAAGGAAAACTCCATTCATCCCATT
CCAAACAGGTGAGAATCTAATGCCATCAGGGAAAAAAAAAAAATATTTTGCTATCCCG
TCTGAATTGAGAGTTCCCTGTGTACAGTTTACGTCCCTGTGAGCATCTACCACTCACAG
AGCCACCCTAAGCACGCTCTGCAGTGTCAGCATCACCTGGCAGTATGCTAGAAATTAG
AAATGCAAATTCATGAGCACCAGCCCAGACCCATTGAGTCGGAAATTCTGAGGGTGGG
GCCCAAGAATGTGAGTTTCAACAAGTTTGAGAAGGACTGCGTCTCCTATATGAAATGG
AAAACAGCTGCTCATCCTCTGTATCCTTGCATAATAAAAAATGATTAGTGTCACTATGCC
ACCATTTCCTCAGCGAAATAATTTCCATCCCCCCACCGCCTCCGAGGGTCTTGCACTCT
CACCCAGGCTGGAGTGCACTGGTGCAATCACAACCCACAGCAGCCTTGTCCTCTCGGG
CTCAAGCAATCCTCCCACCTCAGCTTCCTGAGTAGCTTGGGACTGCAGGCGCAACCAC
CACGCCCGGCTAATACGGCTAATATTATTTTTTCATTTTTTGGAGAGACGGGGTCTCGCT
ATGTTGCCCAGGCTGGTCTGGAACTCTTGGGCTCAAGGGATCCTTGTGCCTCGGCTTC
ACAAAGTGCTGGGATTACAGGCGTGAGCCGCCGCGCCCGGCTCTAAATAATTTCTTATC
GCCCTCCCTCAGTTTGTTTTTCCTGTTATCGGGTCACCCACACATTCCACAATTGTCAA
ATAGGTGAGGGCACAGGTACGGAAGCGTCCCGCATTCCCTCCAGATTTTATCACGCTG
AGACACAAAGAGAAAAATAGCTCCTGGCGACCGCATGAAAACCCCGATTTCAAAACA
GCCACATGCAGTTTAATTTCCTTAAAAGCTTAGCCGGAAAAGGTTTCTGTGGCTTGCGA
CCCCAGCTGGGCTTGAGAGAAGGCTCTGCACACTGGGGCGGGGGCGGGCACAAGGT
GATCGGAGGGAGGGGGATGACTCGCAGTTGTGTTTTGAGTGGTTGCCTTGGGTTATCT
TGTTCCTCCAGCACACACACACTCAGTTTCTTGTCATAAGGGGAACCCTGTTATTTTGT
AGACAAAAAGTGACAGAAGCAACTTTCGAAGCAAAAAAAAAAAAAAAAACAAAAAA
AAACAAAACCAAAAAGACGAAGAAGAAGAACAAAAGTCAACGTGAAATCTCCCCAG
AGGAAAAGGAACCGCGCTGTTTGTCCCTCAGTAACTGAGCAACACCCCGGACACGAA
GGGCTCCCTGTGACTTCTGCGGTTTTTCTTGCACTTCTCCGCGTTGGTTCACTTCCACC
CTCGAAACTCTCCAGAAGCGAGAAATAAGGCGGGGTGGCCGCCACCCCCAGGCCACC
AGCTGTGCGGGGGCCCCAGCTGCAGGGGGTCCCGGCCCAGCGTCCCTCTCGGCCCCT
TCCCTCTCCCCCGGGGGAGAAGGGGCGGGTCCACGCGGCCTGGGCGCTGCGCCCGAC
ACGCGGTAGGGGTCGCTGCGTCTCGGCGCGCTCCCACCGCGGTGAATAACGGGGC
CTGGCTGGAGGAGAAAGG

Red highlights denote potential NPM1 binding sites within the IQGAP2 promoter
Green highlights indicate reference sequences used for primer design.

primers list:

Gene Name	Forward Sequence (5’-3’)	Reverse Sequence (5’-3’)
**primer 1 for binding site 1**	GCACGAAAATCCCCTTCCAC	TGCCTCTCAGTCTAAGAACAATT
**primer 2 for binding site 2**	CAGCCCAGACCCATTGAGT	TGAGCAGCTGTTTTCCATTTCA
**primer 3 for binding site 3**	CTTATCGCCCTCCCTCAGTT	AAAATCTGGAGGGAATGCGG
**primer 4 for binding site 4&5**	AGCCGGAAAAGGTTTCTGTG	CCACTCAAAACACAACTGCG
**primer 5 for binding site 6**	TCTCGGCCCCTTCCCTCT	AGCCAGGCCCCGTTATTC
