# Supplementary material for: N6-methyladenosine-modified ALDH9A1 modulates lipid accumulation and tumor progression in clear cell renal cell carcinoma through the NPM1/IQGAP2/AKT signaling pathway
Source: Cell Death Dis. 2024 Jul 22;15(7):520. doi: 10.1038/s41419-024-06896-z (PMC11263707; doi:10.1038/s41419-024-06896-z)
Supplement: Supplementary file 4 — supplementary table 3 [file 41419_2024_6896_MOESM4_ESM.pdf]

DEG_TCGA					DEG_seq					Correlation		
gene_id	g2FoldCha	log2CPM	PValue	FDR	gene_id	g2FoldCha	log2CPM	PValue	FDR	gene_id	correlation	pvalue
TMEM238	-7.09152	0.407622	0	0	ALDH9A1	1.989297	6.625178	0	0	SCYL3	0.56287464	2.75E-46
ACP3	-5.5724	2.887831	0	0	IL6	-1.11247	6.747399	5.99E-251	5.13E-247	FUCA2	0.59372749	1.49E-52
MFSD4A	-5.25046	6.030317	0	0	COL7A1	-1.01262	6.714157	2.04E-183	4.38E-180	GCLC	0.65945904	1.95E-68
SIM2	-4.50525	2.684384	0	0	ATF3	-1.08273	5.847647	9.50E-161	1.48E-157	NFYA	0.53242477	1.02E-40
ESRRB	-5.93392	2.996033	055278649216e-	6.44716297817554e-311	CXCL8	-1.28218	4.140501	\	1.02E-67	STPG1	0.60563397	3.70E-55
GPC5	-5.83285	1.009863	1.34E-298	3.78E-295	EID3	-1.17642	3.914193	1.09E-56	6.31E-55	NIPAL3	0.53474401	4.04E-41
KCNJ10	-6.1867	4.185559	3.10E-288	7.48E-285	OC1019291	-1.23634	3.905517	1.62E-46	6.12E-45	ENPP4	0.63388893	8.43E-62
ADGRF3	-3.5395	0.779622	1.24E-281	2.62E-278	AGAP6	-1.19098	4.573365	4.97E-34	1.00E-32	ANKIB1	0.62106478	1.06E-58
MYLK3	-4.0327	0.711422	1.09E-279	2.04E-276	CLEC4E	-1.11519	3.286989	1.06E-27	1.56E-26	LAP3	0.51230097	2.47E-37
ATP1A1	-2.76695	10.25547	3.90E-277	6.60E-274	P3H3	-1.05421	3.093762	2.64E-27	3.81E-26	LASP1	0.53626751	2.18E-41
ELF5	-7.85135	2.629778	8.57E-277	1.32E-273	PTX3	-1.16306	2.814513	1.42E-23	1.70E-22	SNX11	0.59810595	1.69E-53
DDN	-6.34873	2.096838	6.45E-275	9.09E-272	IL4I1	-1.05391	2.727288	2.17E-21	2.32E-20	M6PR	0.5665179	5.41E-47
HSPA2	-3.90802	5.702339	7.00E-272	8.53E-269	Y75-CD30	-8.7206	-0.67128	9.48E-21	9.82E-20	KLHL13	0.50253121	9.03E-36
CALB1	-7.52312	5.594941	7.06E-272	8.53E-269	MIR17HG	-1.02166	2.879657	1.39E-19	1.34E-18	ALS2	0.62301232	3.66E-59
IRX2	-5.02822	2.065748	6.49E-268	7.32E-265	OC1053745	-1.02915	2.469353	9.22E-19	8.45E-18	SARM1	0.5113783	3.49E-37
ENPP6	-5.03577	2.275352	9.31E-264	9.85E-261	OC1249021	-1.6225	1.089626	2.44E-18	2.17E-17	POLDIP2	0.60053648	4.97E-54
MTURN	-3.04876	5.997246	1.08E-243	1.08E-240	IR29B2CH	-1.1705	2.055485	1.37E-16	1.09E-15	AK2	0.58299455	2.68E-50
FECH	-1.95841	5.287222	4.22E-239	3.96E-236	OC1249027	-1.22261	1.795048	9.64E-16	7.25E-15	KDM1A	0.50989149	6.07E-37
SLC12A1	-8.28093	8.578405	2.51E-235	2.23E-232	RGPD2	-1.00048	2.16706	4.21E-14	2.84E-13	RECQL	0.55534279	7.43E-45
SLC4A11	-4.44179	3.076226	1.99E-232	1.68E-229	C5AR1	-1.16192	1.729033	1.16E-12	7.06E-12	VPS50	0.54938604	9.50E-44
TRPV6	-4.87098	1.209031	2.94E-228	2.37E-225	HP3-ACAI	-1.80028	2.516388	2.21E-12	1.32E-11	SLC25A13	0.70546885	3.54E-82
CLDN16	-5.90702	3.850385	4.47E-228	3.44E-225	MUC20	-1.08593	1.91966	2.02E-11	1.12E-10	ST7	0.62133344	9.15E-59
FGF1	-4.21263	4.556714	7.48E-228	5.50E-225	SRRM5	-1.15823	1.174383	8.36E-11	4.46E-10	CDC27	0.56515982	9.94E-47
GSTM3	-3.32009	5.854168	1.12E-227	7.91E-225	PSORS1C3	1.128967	1.226844	1.47E-10	7.70E-10	HCCS	0.56154573	4.95E-46
HS6ST2	-6.25432	2.776456	2.66E-220	1.80E-217	FOXJ1	-1.8711	-0.56628	6.05E-10	3.01E-09	UPF1	0.55039615	6.19E-44
DUSP9	-6.83237	4.02504	8.52E-219	5.54E-216	CCL5	-1.04692	1.506749	8.02E-10	3.96E-09	SKAP2	0.64436361	1.92E-64
UNCX	-7.02609	-2.17786	2.03E-210	1.27E-207	OC1122681	-1.2565	0.978699	8.87E-09	4.04E-08	DHX33	0.54941128	9.40E-44
ARMH4	-3.54812	3.445207	4.76E-206	2.88E-203	SEMA3B	-1.04553	1.440889	1.48E-08	6.60E-08	LIG3	0.56033124	8.45E-46
ATP12A	-7.22719	1.266108	3.20E-205	1.87E-202	FCAB13-D	-1.02266	1.013019	1.93E-08	8.51E-08	RPAP3	0.56785772	2.96E-47
HS6ST1	-2.16139	5.573737	6.67E-203	3.76E-200	LAT2	-1.03016	1.2156	1.94E-08	8.53E-08	ACSM3	0.50967537	6.58E-37
WNK4	-4.07998	4.074266	9.36E-203	5.11E-200	OC1249022	-1.38203	0.004822	2.07E-08	9.09E-08	CIAPIN1	0.58553556	7.98E-51
CLUL1	-3.44933	0.608371	5.96E-200	3.15E-197	OC1249017	-1.35155	0.439927	2.63E-08	1.14E-07	CREBBP	0.59061464	6.84E-52
EGF	-5.42006	5.980644	2.78E-199	1.42E-196	36A-HNRN	-1.35098	4.075371	4.76E-08	2.01E-07	GCFC2	0.58696766	4.01E-51
ACSF2	-3.27329	5.024818	5.28E-198	2.63E-195	OC1249017	-1.93496	-0.96816	7.27E-08	3.02E-07	CROT	0.53963564	5.53E-42
SLC4A8	-2.71367	1.893249	7.11E-195	3.44E-192	IARCA5-A	1.388895	0.134543	9.34E-08	3.84E-07	KMT2E	0.52161817	7.16E-39
SLC14A2	-6.44407	3.025176	3.00E-193	1.41E-190	DCN	-2.4489	-1.66514	1.02E-07	4.18E-07	IBTK	0.64559202	9.28E-65
CPNE6	-6.52054	0.63685	1.08E-191	4.94E-189	CDRT4	-1.32709	4.064707	1.03E-07	4.20E-07	FBXL3	0.62139527	8.84E-59
KLHL14	-3.85205	2.172283	3.30E-189	1.47E-186	TMEM86A	-1.34942	-0.1416	1.50E-07	6.02E-07	PDK2	0.75934262	4.22E-102
RANBP3L	-5.19981	3.178568	1.15E-188	4.98E-186	BE2F-SCL	2.577929	-1.01656	1.68E-07	6.71E-07	ZFX	0.50959413	6.78E-37
PLA2R1	-3.08898	4.563025	2.65E-186	1.12E-183	SCG5	-1.30816	0.568473	3.87E-07	1.49E-06	LAMP2	0.60565807	3.65E-55
RBP2	-3.96932	-1.5906	3.64E-186	1.50E-183	CAVIN4	-1.06094	0.874874	6.74E-07	2.53E-06	AP2B1	0.64793578	2.29E-65
ERMP1	-2.45739	6.339994	7.90E-186	3.18E-183	TBX20	1.071132	0.744472	7.73E-07	2.89E-06	ZNF263	0.52469416	2.17E-39
CCNP	-4.80893	0.231609	1.79E-185	7.03E-183	UTP14C	-2.71069	0.916743	8.30E-07	3.09E-06	CX3CL1	0.65078654	4.09E-66
HYKK	-2.0019	1.853514	2.11E-185	8.10E-183	CACNG8	-1.00344	0.824909	9.52E-07	3.52E-06	FARP2	0.5737992	1.98E-48
SFRP1	-5.27965	6.057749	2.79E-185	1.05E-182	OC1053755	-1.47383	-0.54255	9.70E-07	3.58E-06	USH1C	0.63638771	2.02E-62
WNT8B	-3.88649	-1.84498	7.43E-185	2.73E-182	OC1249080	-1.31834	0.265901	1.33E-06	4.82E-06	COX10	0.59575634	5.45E-53
SIAH3	-5.22202	0.317898	2.55E-184	9.19E-182	OC1249035	-1.99294	-1.21407	1.44E-06	5.23E-06	PAF1	0.53064792	2.08E-40
NOS1	-4.42576	1.760198	2.94E-181	1.03E-178	SWIM8-AS	-1.02691	0.48475	1.50E-06	5.43E-06	VPS41	0.52123401	8.31E-39
KCNJ1	-6.36805	5.943942	3.97E-178	1.37E-175	LRRC39	-1.13542	0.277277	1.53E-06	5.54E-06	ARSD	0.59512905	7.43E-53
CLDN19	-5.73541	3.929967	7.88E-176	2.67E-173	AKR1C8	-1.05207	0.60847	1.55E-06	5.58E-06	PNPLA4	0.60793617	1.13E-55
SOST	-6.814	1.330563	1.10E-175	3.66E-173	OC1249042	-1.12864	0.25639	2.23E-06	7.92E-06	ADIPOR2	0.52627535	1.17E-39
WNT9B	-4.13915	0.733064	1.21E-175	3.93E-173	SLFNL1	-1.03615	0.381737	2.24E-06	7.94E-06	PAFAH1B1	0.6437201	2.81E-64
SH3GL3	-5.64299	-0.12366	3.73E-173	1.19E-170	OC1001307	-4.44597	1.124196	2.52E-06	8.89E-06	KIAA0100	0.55186827	3.31E-44
P11-467J1	-4.01187	2.304296	7.98E-172	2.50E-169	IQGAP2	1.019037	0.569257	2.57E-06	9.04E-06	SLC13A2	0.50504697	3.61E-36
TMEM45E	-3.91617	3.290161	1.64E-171	5.05E-169	SNRK-AS1	-1.17564	0.655142	3.78E-06	1.31E-05	ST7L	0.5445214	7.34E-43
USP44	-3.0227	-0.22123	2.07E-171	6.24E-169	LINC0117	-1.0184	0.775403	4.37E-06	1.50E-05	PAX6	0.55118189	4.43E-44
GP2	-6.91181	0.639461	4.64E-171	1.38E-168	C1B-GAL	3.349939	-0.00214	4.68E-06	1.60E-05	DNAJC11	0.65632165	1.38E-67
ALDH6A1	-3.02298	6.876105	5.60E-171	1.63E-168	OC1249032	1.113321	0.176534	5.20E-06	1.77E-05	MYLIP	0.65460171	3.98E-67
NHLRC4	-3.21391	1.559716	6.68E-171	1.91E-168	OC1249024	-1.14979	-0.13581	6.02E-06	2.03E-05	CDKL5	0.57306239	2.77E-48
LGI2	-3.28937	3.085293	8.51E-171	2.40E-168	LOC28563	-1.47963	-0.81966	6.24E-06	2.11E-05	SYPL1	0.64225678	6.66E-64
PROX1	-4.33324	3.232525	6.94E-167	1.92E-164	ENDOU	-1.29223	0.368467	7.50E-06	2.50E-05	CYB561	0.52297848	4.23E-39
TCF21	-3.51941	3.190817	6.47E-166	1.76E-163	LY6K	-1.03871	0.693362	9.28E-06	3.06E-05	SPAG9	0.70730549	8.82E-83
CLIC5	-3.79605	5.242814	7.41E-166	1.99E-163	OC1079854	-1.17585	-0.17232	9.43E-06	3.11E-05	CRY1	0.54283948	1.48E-42
ABCA4	-4.72035	2.775177	1.05E-164	2.77E-162	ACTA1	-1.80396	-1.477	9.77E-06	3.22E-05	MED24	0.55989707	1.02E-45
AKAP3	-2.32754	0.77018	5.91E-164	1.54E-161	PNLIPRP3	-1.41749	-0.61096	1.09E-05	3.58E-05	RHOBTB2	0.52938502	3.44E-40
RALBP1	-1.46093	6.411468	1.09E-163	2.79E-161	CTXND1	-1.18719	-0.39869	1.59E-05	5.10E-05	HEATR5B	0.6383999	6.32E-63
DBT	-1.53621	5.299195	4.02E-163	1.02E-160	CLCNKA	-1.73055	-1.52598	1.80E-05	5.75E-05	SEC62	0.68028766	2.41E-74
NT5C1A	-4.12052	-2.94811	4.16E-163	1.03E-160	COL11A2	-1.05877	0.997034	1.81E-05	5.80E-05	CSDE1	0.68120476	1.29E-74
NPHS1	-5.94573	3.364267	2.43E-162	5.96E-160	ADHFE1	-1.20771	0.028221	2.03E-05	6.46E-05	UBE3C	0.51007122	5.68E-37
UPP2	-4.30516	2.22622	7.63E-162	1.84E-159	OC1053713	-1.03974	0.237425	2.75E-05	8.66E-05	FAM76A	0.57267008	3.32E-48
RVMER34	-4.7027	1.485583	8.39E-162	2.00E-159	SEPTIN5	1.207852	0.269527	2.96E-05	9.28E-05	POMT2	0.54596	4.02E-43
CHP2	-5.24913	-1.45778	2.33E-161	5.48E-159	FKBP6	-1.51793	-1.16193	3.62E-05	0.000112	VTA1	0.63507227	4.29E-62
COBLL1	-2.39057	6.705969	2.39E-160	5.54E-158	RBM46	-1.13839	-0.03795	3.75E-05	0.000116	BAZ1B	0.53373432	6.06E-41
RNF212B	-4.10876	-0.19988	3.07E-159	7.03E-157	OC1053786	-1.29561	-0.88115	4.84E-05	0.000147	RANBP9	0.60316645	1.31E-54
UXS1	-1.44416	5.931824	1.00E-156	2.26E-154	SPDYA	-1.0835	0.155268	5.37E-05	0.000163	SPRTN	0.66712668	1.48E-70
PRR15	-3.9437	2.317048	2.11E-154	4.70E-152	RFPL2	-1.86225	-1.88253	6.47E-05	0.000194	EEF1AKNMT	0.65863911	3.25E-68
HCRTR2	-5.59448	-3.12795	4.83E-154	1.06E-151	22D4-C7O	-1.84684	-0.43625	8.41E-05	0.000249	ZNF207	0.50038098	1.96E-35
SLC16A5	-3.05003	3.894688	1.55E-151	3.36E-149	OC1249046	-1.59965	-1.42992	9.33E-05	0.000275	STARD3NL	0.68827947	9.61E-77
KCNE1B	-5.07412	-2.32364	1.93E-150	4.14E-148	ADCY10	-1.03106	-0.36888	0.000106	0.000308	SLC6A13	0.50787787	1.28E-36
XPNPEP2	-5.37635	4.937357	1.09E-149	2.30E-147	ARTN	-1.10123	-0.39963	0.00011	0.000321	ZNF200	0.54773969	1.90E-43
FAM171A	-2.16569	5.397016	2.39E-149	4.95E-147	RIM59-IFT	-1.27975	2.892994	0.000113	0.000329	HFE	0.55197234	3.16E-44
CASR	-5.51712	4.185843	2.40E-149	4.95E-147	OC1079870	1.104477	-0.21853	0.000114	0.000332	HIVEP2	0.51779444	3.11E-38
ABAT	-3.03202	5.536297	8.37E-149	1.71E-146	OC1225131	-2.56024	-0.47325	0.000118	0.000343	FMO1	0.6860583	4.54E-76
SRGAP3	-2.45706	3.072489	2.41E-148	4.86E-146	VWA5B2	-1.54375	-1.64789	0.000121	0.000349	ELOA	0.59689107	3.10E-53
CRHBP	-4.05543	2.766994	6.50E-148	1.29E-145	OC1249052	-1.83634	-1.96928	0.000121	0.00035	CLCN6	0.51799025	2.88E-38
SFXN2	-2.35276	4.453784	2.83E-147	5.57E-145	OC1249022	-1.7979	-1.66486	0.000123	0.000355	TSPAN9	0.50636761	2.23E-36
PRDM16	-3.69675	3.062345	7.53E-147	1.46E-144	OC1249042	1.352109	-1.01821	0.000127	0.000367	BTBD7	0.59489547	8.35E-53
PRKAR2E	-2.23433	3.265229	3.60E-146	6.92E-144	PPM1E	-1.03827	0.504496	0.000128	0.00037	ABHD5	0.60700855	1.82E-55
TMEM52E	-5.03684	5.075405	1.53E-144	2.92E-142	ABCC9	-1.00207	-0.04275	0.000129	0.000372	UTP18	0.63827926	6.78E-63
PTPRO	-3.17923	4.291816	2.03E-143	3.82E-141	ASAP1-IT2	-1.78111	-2.01579	0.00013	0.000376	TTC19	0.66838813	6.54E-71
DDB2	2.038685	5.627527	2.43E-142	4.52E-140	LINC0273	-1.04282	-0.01822	0.000139	0.0004	PTBP1	0.5388837	7.52E-42
CACNA2D	-2.28513	1.639223	6.81E-142	1.25E-139	OC1249013	1.394257	-0.92883	0.00014	0.000402	LARS2	0.51569649	6.89E-38
IRX1	-4.57481	1.427462	9.81E-142	1.79E-139	07-LOC100	-1.84012	-1.29621	0.000141	0.000404	PIK3C2A	0.55389184	1.39E-44
CCNI2	-2.93993	0.252901	1.23E-141	2.22E-139	OC1053724	-1.26758	-0.88172	0.000161	0.000457	RABGAP1	0.57906068	1.72E-49
TMEM164	-1.49295	4.998307	2.13E-141	3.79E-139	RSPO3	-1.63241	-1.2681	0.000162	0.000461	PPP5C	0.53360268	6.39E-41
EHF	-5.29609	3.572428	3.48E-141	6.13E-139	ACSBG1	-1.00018	-0.34656	0.000163	0.000464	CEP68	0.60315027	1.32E-54
TPPP2	-4.07767	-2.06098	3.73E-141	6.51E-139	OC1053728	-1.26315	-1.11117	0.000163	0.000464	MAP4K3	0.51438577	1.13E-37
SLC7A8	-3.56926	6.082189	1.28E-140	2.21E-138	OC1249020	-1.16349	-0.83963	0.000166	0.000471	TMEM159	0.59568372	5.65E-53
SEMA6D	-2.93143	4.241909	2.24E-140	3.82E-138	SNX22	-1.15501	3.154387	0.000169	0.000479	MBTPS2	0.60698838	1.84E-55
CYFIP2	-2.25103	8.129267	8.23E-140	1.39E-137	OC1053753	-1.332	-0.46086	0.000177	0.000502	EXTL3	0.5623557	3.46E-46
TCF24	-4.98564	-2.22003	2.70E-139	4.52E-137	OC1249050	-1.10422	0.668951	0.000188	0.00053	NR1H4	0.53687249	1.71E-41
CPAMD8	-2.78425	3.859036	2.95E-139	4.89E-137	LINC0062	-1.29171	-1.16149	0.000203	0.00057	ELOVL5	0.53548713	2.99E-41
EFHD1	-2.61732	6.282567	3.06E-139	5.03E-137	LIMS3	1.249459	1.210322	0.000213	0.000595	UBR7	0.67757365	1.51E-73
EYA4	-3.48991	2.250018	1.51E-138	2.45E-136	OC1249024	-1.74049	-1.511	0.000229	0.000639	MVP	0.50052526	1.86E-35
KCNK13	-2.62954	1.754556	1.94E-138	3.13E-136	OC1249050	-1.24748	-1.00245	0.000243	0.000673	PGM3	0.60437835	7.04E-55
TMEM178A	-3.21842	2.532407	2.36E-138	3.77E-136	TAF7L	-1.27593	-0.839	0.000272	0.000748	RWDD2A	0.55409639	1.27E-44
SLC20A2	-1.38028	5.18995	3.80E-138	6.01E-136	IX2-CTNN	-1.31086	1.289097	0.000283	0.000776	POLR3B	0.53260405	9.54E-41
SEMG2	-7.30602	-1.98558	7.62E-138	1.19E-135	NF341-AS	-1.2727	-1.09808	0.000311	0.000848	RNF14	0.60197399	2.40E-54
TRPV5	-5.10158	-0.65271	1.02E-137	1.58E-135	OC1249039	1.116835	-0.67693	0.000317	0.000865	DNASE1L1	0.51493949	9.17E-38
AIF1L	-3.2217	8.027222	1.77E-137	2.72E-135	OC1249016	-1.24524	-1.12397	0.000324	0.000882	UFL1	0.67997029	2.99E-74
C4orf54	-6.74624	0.362362	5.14E-137	7.83E-135	OC1053726	-1.05934	-0.57734	0.000339	0.000919	CAPN1	0.50859269	9.82E-37
FLRT1	-4.09547	0.606869	1.83E-135	2.76E-133	SLC22A1	-1.01724	-0.09774	0.000345	0.000935	MDH1	0.58482588	1.12E-50
AQP2	-8.94564	8.646635	4.04E-134	6.05E-132	DLEC1	-1.13368	-0.65474	0.000346	0.000937	SLC30A9	0.63755068	1.03E-62
SLC28A2	-3.5135	-0.2487	5.26E-134	7.81E-132	FRZB	-1.1745	-0.78802	0.000355	0.00096	COX15	0.68565613	6.01E-76
SLC5A2	-4.69671	2.528191	6.74E-134	9.91E-132	OC1019297	1.203858	-0.81303	0.000359	0.00097	ZMYND11	0.66912202	4.06E-71
SLC5A7	-4.2844	-2.16057	1.11E-133	1.61E-131	BAAT	-1.25014	-1.11081	0.00036	0.000972	NUDCD3	0.52118664	8.46E-39
RASL11B	-4.26833	2.688284	3.32E-133	4.80E-131	C1orf100	-1.16905	-0.23934	0.000434	0.001155	CHDH	0.58017658	1.02E-49
HADH	-1.76322	5.952393	6.76E-133	9.69E-131	OC1249021	1.418106	-1.25661	0.000475	0.001256	ATP2C1	0.55985867	1.04E-45
FAM81A	-2.55928	2.434254	1.19E-132	1.69E-130	SEC16B	-1.50498	0.406481	0.000479	0.001266	RALBP1	0.59149718	4.45E-52
TNNT2	-4.84632	1.345934	4.38E-132	6.18E-130	ZNF135	-1.19796	-0.85079	0.000482	0.001274	WWTR1	0.59784172	1.93E-53
PRR35	-7.006	0.697742	1.16E-131	1.61E-129	LDN14-AS	1.09991	-0.63351	0.000489	0.00129	AGPS	0.74291717	1.69E-95
CALML3	-5.66288	0.460114	1.32E-131	1.82E-129	SPHO2-KL	1.341363	0.89843	0.000517	0.001358	STEEP1	0.62503376	1.21E-59
PLCL1	-2.63655	5.079605	1.61E-131	2.21E-129	SLC38A11	-1.00191	-0.31734	0.000565	0.001476	TTC27	0.64641219	5.69E-65
SLC15A2	-3.07973	3.079088	2.45E-131	3.34E-129	OC1249012	-1.38881	-1.39915	0.000567	0.001481	ZRANB1	0.58961269	1.11E-51
PIP4K2C	-1.40167	5.610966	5.85E-131	7.92E-129	CERS6-AS	-1.25088	-0.85979	0.00057	0.001487	ZFP64	0.57622385	6.44E-49
EMX1	-2.60684	4.346579	4.97E-130	6.68E-128	SMIM18	-1.05881	-0.3841	0.000613	0.001593	MNAT1	0.5117534	3.03E-37
MECOM	-2.55517	5.972346	6.81E-130	9.07E-128	OC1053744	-1.10931	-0.99155	0.000619	0.001608	SERPINB1	0.50326144	6.93E-36
FAM167A	-3.53915	2.777244	9.11E-130	1.20E-127	OC1053715	-1.18941	-1.14895	0.000631	0.001637	SLC7A9	0.57580399	7.83E-49
FOXC1	-2.17064	4.675297	9.20E-130	1.21E-127	OC1122680	-1.42406	-1.26795	0.000637	0.00165	SPAST	0.55673751	4.06E-45
CNP	-1.30568	6.597548	1.64E-129	2.13E-127	TEX13B	1.619688	-1.76149	0.000656	0.001695	AQR	0.67019907	2.01E-71
MUC15	-7.27356	3.515954	3.33E-129	4.30E-127	OC1053709	-1.31073	-1.51022	0.000674	0.001738	RTF2	0.65508981	2.95E-67
RNF150	-3.10814	4.360469	3.85E-129	4.93E-127	MRC1	-1.45104	-1.76027	0.000762	0.001949	ZDHHC6	0.64068627	1.67E-63
NUDT4	-1.79669	5.755825	3.93E-129	4.99E-127	OC1249004	-1.10285	-0.73113	0.000812	0.002066	RNH1	0.60852585	8.29E-56
EDDM3A	-4.81827	-2.74162	4.57E-129	5.77E-127	PNCK	-1.24297	-1.22751	0.000854	0.002168	NDUFS1	0.71262171	1.48E-84
CYP27B1	-3.59311	2.026502	8.65E-129	1.08E-126	OC1019270	-1.53892	-1.44464	0.000925	0.002335	ERP44	0.59941201	8.76E-54
WNK1	-1.51639	7.726106	1.18E-128	1.47E-126	CYYR1-AS	1.708546	-1.99427	0.00093	0.002347	ALAS1	0.56418031	1.54E-46
MTOR	-1.16601	5.855388	1.83E-128	2.27E-126	OC1249029	-1.14133	-1.01501	0.000956	0.002405	AKAP11	0.56495498	1.09E-46
COL4A6	-4.37484	1.918189	4.92E-128	6.03E-126	TREX2	-1.28054	-0.62797	0.001018	0.002549	STRAP	0.52934836	3.49E-40
UMOD	-8.56251	10.13738	5.59E-128	6.81E-126	RFPL3S	-1.07591	-0.65571	0.001106	0.002756	GCLM	0.61610746	1.53E-57
NOL3	3.435697	6.700289	2.27E-127	2.74E-125	MSTN	-1.25842	-0.87052	0.00112	0.002785	UBR2	0.60401791	8.47E-55
PAQR7	-1.59336	5.035736	3.40E-127	4.08E-125	OC1053788	-1.01498	-0.85083	0.001154	0.002864	CCDC28A	0.64391254	2.51E-64
CARMIL1	-1.4628	4.775599	7.00E-127	8.33E-125	OC1053739	-1.02933	-0.47698	0.001179	0.002925	RRAGD	0.54305984	1.35E-42
MRO	-2.97045	3.169949	3.24E-125	3.83E-123	OC1249002	-1.01488	-0.13609	0.001252	0.003092	PHF20	0.5119277	2.84E-37
CCDC181	-3.05743	0.911689	3.40E-125	4.00E-123	OC1053776	-1.14356	-0.87125	0.001309	0.003224	SEC63	0.64838679	1.74E-65
IYD	-4.25666	2.984902	9.06E-125	1.06E-122	GALR2	-1.14437	-1.09858	0.001415	0.003463	KPNA6	0.669832	2.56E-71
ERICH4	-3.58767	0.040422	3.02E-124	3.50E-122	SERPINA6	-1.12889	-0.24131	0.001458	0.003562	FAS	0.5123426	2.43E-37
ASS1	-2.70308	7.892044	4.51E-124	5.18E-122	ABCG1	-1.14227	-1.13591	0.001483	0.003616	MIPEP	0.59988198	6.91E-54
ACAA1	-1.60246	5.919595	6.83E-124	7.81E-122	OC1019273	-1.03935	-0.88103	0.001586	0.003846	VRK2	0.51388606	1.36E-37
ABHD17C	-2.03773	3.51863	9.26E-124	1.05E-121	CDHR5	1.455367	-1.70447	0.001728	0.004167	SNX1	0.72757961	8.96E-90
SERPINA5	-4.88899	5.713936	9.92E-124	1.12E-121	GREM1	-1.02963	-0.94688	0.001768	0.004252	BCLAF1	0.59066042	6.69E-52
LMO3	-3.37208	1.615344	1.18E-123	1.32E-121	TF2A1-AS	1.364622	-1.18884	0.001866	0.004472	SLC39A9	0.6602182	1.21E-68
PCARE	-5.69086	-1.94043	2.58E-123	2.88E-121	ANKRD1	-1.13852	-1.21435	0.00187	0.004481	RABEP1	0.54563986	4.60E-43
TMEM207	-8.1697	0.916116	3.42E-123	3.78E-121	RAMP1	-1.33437	-1.33897	0.001905	0.004557	NUP160	0.6529039	1.13E-66
SLC22A8	-6.11369	5.420994	6.11E-123	6.72E-121	NPC1L1	-1.25194	-1.59486	0.001912	0.004573	ARHGAP31	0.5965856	3.61E-53
EPB41L5	-1.75131	5.329565	2.99E-122	3.26E-120	OC1079859	1.034034	-0.84123	0.002047	0.004874	SARS1	0.59839344	1.46E-53
SAP30	2.493537	4.124183	1.30E-121	1.41E-119	ESRP2	-1.01443	-0.99214	0.002114	0.005019	ARID4A	0.58254526	3.32E-50
AGBL4	-2.45651	0.071517	2.62E-121	2.82E-119	EGR3	-1.1698	-1.41555	0.002117	0.005024	IFT88	0.64585531	7.93E-65
PTPN13	-1.8389	5.853621	3.19E-121	3.41E-119	BIRC6-AS1	-1.43	-1.90355	0.002118	0.005025	ALG1	0.56657287	5.28E-47
SPAG4	3.898979	5.456397	4.90E-121	5.22E-119	SNHG31	1.061544	-0.91684	0.002151	0.005098	ATP6V0A1	0.6820267	7.35E-75
GADL1	-4.40372	-0.76839	2.42E-120	2.55E-118	OC1249078	-1.10939	-0.81943	0.002233	0.005278	PIAS1	0.58943766	1.21E-51
CADPS2	-1.5813	5.539852	2.95E-120	3.10E-118	SCN3A	-1.15434	-1.36958	0.002315	0.005453	ASTE1	0.57242522	3.71E-48
HPD	-4.85877	5.328079	1.56E-119	1.63E-117	ZNF804A	1.606453	-1.94902	0.002617	0.006112	PEX3	0.6190257	3.19E-58
KCNK10	-3.31888	0.34201	3.04E-119	3.15E-117	SLC5A12	-1.0859	-1.02748	0.002633	0.006145	GABARAPL2	0.63069868	5.13E-61
BTG2	-2.10233	7.413441	3.19E-119	3.29E-117	SYT5	-1.28844	-1.44615	0.002689	0.006266	SH3YL1	0.52983328	2.88E-40
AVPR2	-3.41854	1.805848	6.32E-119	6.48E-117	HIF1A-AS	1.083277	0.251399	0.002744	0.006385	ADSS2	0.53931687	6.30E-42
AK3	-1.454	6.355777	8.20E-119	8.36E-117	OC1249017	-1.68675	-1.28412	0.002852	0.006625	RFC1	0.62375974	2.43E-59
TFAP2B	-5.81403	2.418061	9.02E-119	9.13E-117	RNF212	-1.07079	-1.21402	0.003148	0.00727	TBC1D23	0.51723691	3.84E-38
MYOZ2	-3.10328	-1.82867	3.89E-118	3.92E-116	OGARAM	-1.20319	-1.50995	0.003155	0.007283	CUL3	0.73438819	2.88E-92
PCK2	-2.27839	6.074661	5.05E-118	5.06E-116	OC1122684	-1.12977	-1.26942	0.003164	0.007302	ZZZ3	0.54569252	4.50E-43
ANXA9	-1.90849	3.789494	1.81E-117	1.80E-115	PTN	-1.05127	-0.63984	0.003216	0.007416	FBXO42	0.5692557	1.57E-47
ACADSB	-1.9993	6.095177	5.54E-117	5.48E-115	GRPR	-1.16179	-1.25512	0.003279	0.007547	MFAP3	0.59170537	4.02E-52
DIO1	-4.77315	3.286131	9.28E-117	9.13E-115	OC1249028	-1.00191	-1.16259	0.003418	0.007838	AGA	0.63615233	2.31E-62
EGLN3	4.259293	9.001138	1.03E-116	1.00E-114	CYP2E1	-1.2309	-1.44522	0.003619	0.008247	PI4K2B	0.6257845	7.96E-60
SLC47A2	-3.90152	3.266898	3.46E-116	3.37E-114	C10orf82	-1.07482	-1.36945	0.003654	0.008318	MAT2B	0.6396054	3.14E-63
ZNF503	-1.9181	4.462881	4.08E-116	3.95E-114	GRM8	-1.06218	-0.75922	0.003831	0.008697	TLL1	0.55984678	1.05E-45
DNAJC11	-1.31844	5.248989	1.21E-115	1.16E-113	PRRG2	-1.05404	-1.08653	0.003859	0.008755	MTREX	0.6581494	4.42E-68
TAFA4	-4.30611	-3.27879	2.78E-115	2.66E-113	GLUD2	1.344698	-1.44893	0.003979	0.009014	ZFYVE16	0.53846487	8.92E-42
PPP2R2B	-2.28386	1.901673	8.53E-115	8.11E-113	ATP2B3	-1.27242	-1.86128	0.004006	0.00907	RIPOR1	0.54014581	4.48E-42
PRMT6	-1.27989	4.160071	9.59E-115	9.06E-113	IGSF5	1.375279	-1.24353	0.004024	0.009107	RAI14	0.5053141	3.28E-36
NIPAL1	-3.21798	2.578397	1.12E-114	1.06E-112	OC1053710	-1.30984	-1.64715	0.004025	0.009108	STAU2	0.56144405	5.17E-46
EPM2A	-1.37475	3.175265	1.99E-114	1.86E-112	TERB1	-1.13736	-1.61248	0.004204	0.009475	CTNS	0.58530213	8.92E-51
PTH1R	-3.23428	5.474438	3.07E-114	2.85E-112	PLA2G4B	2.67178	0.239198	0.004237	0.009545	PHF23	0.60789936	1.15E-55
KLRG2	-5.06421	0.84441	4.36E-114	4.03E-112	RO7-PAM	1.604209	0.756782	0.004426	0.009952	LSG1	0.51315299	1.80E-37
HOXD8	-1.76701	5.312763	7.36E-114	6.77E-112	NHBA-AS	-1.09851	-0.93583	0.0047	0.01053	TDP1	0.51412043	1.25E-37
USP46	-1.30377	4.170601	2.48E-113	2.27E-111	IRLET7BH	-1.13576	1.676061	0.004754	0.010639	SPATA7	0.55170789	3.54E-44
DCAF11	-1.13264	6.268008	6.17E-113	5.61E-111	CCER2	-1.34236	-1.37021	0.004881	0.010886	MED17	0.65753864	6.46E-68
TRPM6	-2.98158	1.300174	7.48E-113	6.77E-111	CORT	-1.12881	-1.36911	0.005113	0.01137	RETSAT	0.6584968	3.56E-68
C1orf226	-2.76493	2.648147	1.10E-112	9.86E-111	OC1249002	-1.00032	-1.11101	0.005422	0.012017	BARX2	0.51638103	5.32E-38

DEG_TCGA | DEG_seq | Correlation										
gene_id	g2FoldCha	log2CPM	PValue	FDR	g2FoldCha	log2CPM	PValue	FDR	correlation	pvalue
IQGAP2	-1.07484	5.886204	2.14E-22	8.89E-22	1.019037	0.569257	2.57E-06	9.04E-06	0.619634	2.30E-58
ABCG1	1.301151	6.113234	9.26E-44	1.03E-42	-1.14227	-1.13591	0.001483	0.003616	0.503314	6.80E-36

PTGER1	-4.92343	0.923024	3.06E-112	2.74E-110
COL26A1	-4.68228	0.996385	4.07E-112	3.62E-110
SUCLG1	-1.73815	6.75964	6.56E-112	5.81E-110
RAB11FIP	-1.95006	4.370181	7.66E-112	6.75E-110
ACY1	-2.17225	3.217928	1.39E-111	1.22E-109
EPCAM	-2.85566	6.457958	1.52E-111	1.33E-109
REEP6	-2.96896	2.44643	6.65E-111	5.77E-109
NAT8L	-4.6134	3.30005	9.01E-111	7.78E-109
OPHN1	-1.66819	3.450724	9.31E-111	7.99E-109
TNNI1	-4.04148	1.314627	2.56E-110	2.19E-108
SLC1A4	1.955224	5.130455	9.71E-110	8.26E-108
PAPPA	-3.12881	3.520414	1.14E-109	9.61E-108
ARHGEF3	2.732177	2.545506	2.09E-109	1.76E-107
NOS1AP	-2.77871	0.557645	2.71E-109	2.27E-107
STRA6	-4.03485	1.75072	3.09E-109	2.58E-107
CASZ1	-2.02294	2.777586	1.65E-108	1.37E-106
SPTB	-2.64136	1.793271	2.24E-108	1.85E-106
HELT	-5.23508	-3.14715	2.29E-108	1.88E-106
TYRO3	-2.2331	3.612112	2.33E-108	1.90E-106
SUSD4	-3.6833	3.15215	3.75E-108	3.05E-106
VIM	2.588376	11.49324	6.90E-108	5.58E-106
NRK	-4.77779	2.984993	1.50E-107	1.21E-105
DEGS2	-2.99878	2.745868	1.94E-107	1.56E-105
PCDH9	-3.25524	2.471031	2.49E-107	1.99E-105
PNP	-1.46385	6.306392	3.43E-107	2.72E-105
ASB15	-5.2256	0.197202	3.72E-107	2.94E-105
IRF2BPL	-1.46701	5.382143	3.98E-107	3.13E-105
GPC3	-3.64452	4.619458	1.02E-106	8.02E-105
MPP7	-2.7853	3.948112	4.32E-106	3.37E-104
DPEP1	-4.34758	5.291553	1.18E-105	9.13E-104
KLHL13	-2.15953	4.054068	1.26E-105	9.74E-104
MLKL	1.941726	4.555402	1.86E-105	1.43E-103
SLC48A1	-1.42604	5.323405	2.03E-105	1.56E-103
S100A2	-3.50152	3.774574	4.80E-105	3.66E-103
AFAP1L2	-1.85887	5.148112	1.07E-104	8.14E-103
SLC14A1	-2.98975	4.219479	2.26E-104	1.71E-102
HECW1	-3.69731	2.044714	1.60E-103	1.20E-101
AMPH	-3.15272	1.113331	1.91E-103	1.43E-101
GABRD	5.141228	3.878571	3.34E-103	2.49E-101
HILPDA	4.677203	7.682429	5.47E-103	4.06E-101
NETO2	3.315946	6.074646	1.62E-102	1.20E-100
TYRP1	-5.45683	1.47354	2.08E-102	1.53E-100
DTX1	-2.4574	2.837826	2.41E-102	1.76E-100
VEGFA	3.485096	10.61736	2.43E-102	1.77E-100
FAM222A	-2.74012	2.333312	4.35E-102	3.16E-100
SLC16A3	3.050556	8.008134	1.73E-101	1.25E-99
LILRB1	2.936744	3.871198	1.82E-101	1.31E-99
MAP6	-2.38718	2.613809	3.75E-101	2.69E-99
CLDN14	-2.95161	1.257249	5.44E-101	3.88E-99
SPTBN2	-4.32341	5.576367	2.12E-100	1.51E-98
SORD	-2.16591	4.790458	2.58E-100	1.83E-98
ZFHX2	-1.74173	1.80962	1.04E-99	7.31E-98
SLC25A3	-1.71791	2.742919	1.29E-99	9.04E-98
SLC26A4	-3.25112	1.985235	1.40E-99	9.78E-98
ARHGAP2	-1.62058	6.332227	3.67E-99	2.55E-97
LDHB	-1.53861	9.08132	4.53E-99	3.14E-97
SLC15A4	1.860969	6.469195	6.37E-99	4.40E-97
KCTD8	-4.02719	-1.86478	7.42E-99	5.10E-97
MRLN	-4.45326	-1.5507	7.81E-99	5.35E-97
AFM	-4.65343	2.302771	1.86E-98	1.27E-96
ARHGAP2	2.774743	2.907408	2.16E-98	1.46E-96
NKD1	-2.90916	0.999443	2.25E-98	1.52E-96
AGAP2	2.712917	2.773796	2.35E-98	1.59E-96
NAV2	-1.60426	5.191836	4.42E-98	2.97E-96
LZTS3	-2.29038	4.557803	4.53E-98	3.03E-96
ZNF488	-3.50464	-0.72536	6.31E-98	4.20E-96
PEG3	-2.35545	2.945056	7.26E-98	4.82E-96
PDE1A	-2.81184	5.287309	8.98E-98	5.94E-96
RHBDF2	1.96391	5.674018	1.22E-97	8.01E-96
OLFM3	-5.3067	-2.41803	3.10E-97	2.04E-95
ALAD	-1.26802	6.015999	4.30E-97	2.81E-95
PEPD	-1.85474	7.047508	7.23E-97	4.70E-95
RNF43	-2.54424	2.87845	1.87E-96	1.21E-94
VSIG8	-2.83382	-0.99232	3.00E-96	1.94E-94
RASSF8	-1.50101	5.227666	3.78E-96	2.43E-94
CDKN2A	4.949164	2.922432	4.77E-96	3.06E-94
P2RX7	2.69231	4.191017	5.06E-96	3.23E-94
AREL1	-1.17972	5.192648	6.26E-96	3.98E-94
CXCR4	2.795754	7.564563	7.28E-96	4.61E-94
COLGALT	1.73264	7.030898	8.24E-96	5.20E-94
ST8SIA4	3.572059	5.829193	1.05E-95	6.58E-94
FAM151A	-3.94059	4.415842	1.41E-95	8.83E-94
NYAP1	-2.60639	-0.81366	2.04E-95	1.27E-93
STAMBPL	2.490047	3.856167	5.49E-95	3.41E-93
TRPV2	1.975934	4.5218	8.59E-95	5.32E-93
NPHS2	-7.09304	4.064897	1.56E-94	9.66E-93
MT1G	-4.43438	6.050916	1.58E-94	9.69E-93
HOMER1	-2.19531	3.123137	2.82E-94	1.73E-92
ST6GAL1	-2.17385	7.459778	3.69E-94	2.25E-92
PARD3B	-1.41214	4.50445	3.85E-94	2.35E-92
ACSL4	-1.49954	6.675583	8.01E-94	4.86E-92
ZNF425	-1.21407	1.533421	8.96E-94	5.41E-92
DEPTOR	-1.55326	5.107397	1.17E-93	7.05E-92
COL5A3	3.558518	5.232825	1.25E-93	7.48E-92
F11	-4.23438	2.362798	1.93E-93	1.15E-91
ZNF395	2.645448	8.321258	3.06E-93	1.82E-91
CA8	-3.25743	2.578066	3.13E-93	1.86E-91
GATA3	-3.12888	4.156657	3.72E-93	2.20E-91
CTH	-1.85319	2.313733	3.85E-93	2.27E-91
EDA2R	2.306296	4.120777	4.27E-93	2.51E-91
CD40	1.843196	5.69503	7.69E-93	4.50E-91
SCARB1	3.808541	7.902253	9.26E-93	5.40E-91
SLC9A4	-6.08793	2.894592	1.36E-92	7.93E-91
KLK7	-5.7934	0.448544	1.74E-92	1.01E-90
CACNA2D	-2.09526	1.509615	2.92E-92	1.69E-90
C2orf15	-1.84911	1.18732	3.54E-92	2.04E-90
P11-190A1	-3.82759	-3.38485	6.05E-92	3.47E-90
FMO5	-2.43312	3.630043	1.03E-91	5.91E-90
H1-0	-1.89835	7.22062	2.00E-91	1.14E-89
PROZ	-4.14439	0.367097	2.41E-91	1.37E-89
PPM1K	-1.51996	4.727797	1.16E-90	6.59E-89
NDUFA4L	5.457299	10.51994	1.25E-90	7.02E-89
CROT	-1.24672	4.368829	1.94E-90	1.09E-88
CFAP74	3.988883	0.120005	2.17E-90	1.22E-88
NAALADL	-1.87925	3.173065	2.22E-90	1.24E-88
MARVELD	-2.36956	4.034307	2.46E-90	1.37E-88
SLC52A3	-2.96963	2.625162	2.57E-90	1.42E-88
FAM78A	2.384071	3.886691	1.25E-89	6.93E-88
MAGI2	-1.59278	3.592935	1.50E-89	8.28E-88
RALYL	-5.73211	1.208992	1.94E-89	1.06E-87
CLCNKA	-4.96671	5.157131	2.60E-89	1.42E-87
SLC13A3	-4.68149	6.133672	3.06E-89	1.67E-87
PAFAH2	-1.04237	4.843007	7.46E-89	4.04E-87
ENTPD3	-3.2257	-0.3793	7.69E-89	4.16E-87
DOK3	2.641829	3.749841	9.18E-89	4.95E-87
SLC25A2	-1.87094	4.449623	1.88E-88	1.01E-86
TREM2	4.078543	4.539319	2.35E-88	1.26E-86
NELL1	-5.80024	2.654923	2.45E-88	1.30E-86
CDH16	-2.359	8.508113	2.45E-88	1.30E-86
ANGPTL4	4.956689	9.579216	2.86E-88	1.52E-86
ZNF44	-1.32029	4.265157	3.32E-88	1.76E-86
HPCAL4	-3.96757	1.280506	8.41E-88	4.43E-86
SORT1	-1.51787	7.126185	9.45E-88	4.96E-86
MRPL33	-1.07093	4.835608	1.53E-87	8.00E-86
PML	1.225929	6.81158	2.46E-87	1.29E-85
ALDOB	-4.71196	9.329284	2.67E-87	1.39E-85
MTCP1	3.265239	3.091165	3.05E-87	1.58E-85
TGFBR3	-1.602	5.831052	3.12E-87	1.62E-85
TNNC1	-3.3343	0.474716	6.08E-87	3.14E-85
ARRB2	1.518375	5.859208	8.27E-87	4.25E-85
RASAL3	2.684914	4.057524	1.11E-86	5.67E-85
ZHX3	-1.29024	6.304746	1.37E-86	7.02E-85
PRDM1	2.29045	5.138124	1.68E-86	8.54E-85
PTPRE	1.685001	5.261098	1.84E-86	9.34E-85
ITLN1	-3.64038	-0.46732	2.04E-86	1.03E-84
PHKA2	2.195859	7.081885	2.59E-86	1.31E-84
HLA-B	1.945017	11.57874	3.00E-86	1.51E-84
PTPN4	-1.24784	4.720528	4.10E-86	2.06E-84
SELENBP	-1.71133	5.546516	5.24E-86	2.62E-84
CD1D	2.287299	3.082703	7.40E-86	3.70E-84
KNG1	-6.17719	7.131714	8.61E-86	4.28E-84
STC2	3.930792	6.99537	1.27E-85	6.28E-84
HSF4	5.732523	6.18824	1.34E-85	6.61E-84
BPHL	-1.57303	4.692694	1.75E-85	8.62E-84
MTMR10	-1.14464	6.081898	2.00E-85	9.82E-84
POLR3B	-1.20889	3.477044	2.75E-85	1.35E-83
HLA-F	2.465525	7.193891	3.83E-85	1.87E-83
OVCH2	-4.41528	0.448847	4.26E-85	2.08E-83
SHBG	-2.3061	-0.68386	4.44E-85	2.16E-83
C6orf52	-1.85189	-1.12924	5.02E-85	2.43E-83
ADGRV1	-2.73257	2.288939	6.02E-85	2.91E-83
FUT11	1.571577	5.936144	6.21E-85	2.99E-83
PRRG2	-2.81891	1.603257	6.73E-85	3.24E-83
HK2	3.450301	6.525654	7.86E-85	3.76E-83
TMEM74E	3.73206	2.546267	8.91E-85	4.26E-83
GGT6	-4.74217	3.822121	1.49E-84	7.08E-83
ACOT12	-4.25751	-1.16797	1.79E-84	8.49E-83
PIK3R5	2.82144	4.09668	3.01E-84	1.42E-82
SEMA5B	3.707339	7.243327	4.35E-84	2.05E-82
KSR2	-3.22081	1.804126	4.84E-84	2.28E-82
CLDN10	-2.29024	5.259614	5.58E-84	2.61E-82
KRBA1	2.811199	5.924231	1.06E-83	4.93E-82
ODF3B	3.550474	4.601071	1.08E-83	5.05E-82
NTNG1	-4.49256	1.525981	1.57E-83	7.31E-82
DGKD	2.125021	5.548903	1.59E-83	7.39E-82
TAPBP	1.61773	8.612934	1.74E-83	8.05E-82
VGLL1	-6.18553	0.20614	1.78E-83	8.21E-82
FAM3B	-3.88557	2.132367	2.92E-83	1.34E-81
PDHB	-1.14725	5.735022	3.33E-83	1.52E-81
NUAK2	-2.10715	4.99581	4.59E-83	2.10E-81
MRAP2	-3.36731	0.394161	4.86E-83	2.21E-81
PARVG	2.889085	4.280448	5.93E-83	2.70E-81
DAG1	-1.13988	6.894858	6.05E-83	2.74E-81
SREBF2	-1.24227	6.714248	8.92E-83	4.03E-81
CAV2	2.012934	7.281108	1.35E-82	6.08E-81
ESRRG	-3.04984	4.61106	2.09E-82	9.39E-81
MYO3B	-3.79464	2.903802	2.63E-82	1.18E-80
DACH1	-2.35876	3.359021	3.18E-82	1.42E-80
CCSER1	-2.46762	1.762657	3.23E-82	1.44E-80
LCP2	2.230588	5.619313	3.25E-82	1.45E-80

RIPPLY3	1.044723	-1.10084	0.005785	0.012751
3A7-CYP3	-1.81162	-2.24126	0.006192	0.013574
B3GNT7	-1.1347	-1.40066	0.006213	0.013616
FGF18	-1.01718	-1.32568	0.006392	0.013971
OC1249044	-1.05388	-1.16354	0.006702	0.014616
NKAIN4	1.25066	-1.68533	0.007168	0.015533
OC124902	1.262966	-1.74208	0.007343	0.015876
RAMP2	-1.06881	-1.28203	0.007453	0.016101
PPP1R27	-1.22022	-1.81954	0.007981	0.017143
MFNG	-1.04539	-1.04958	0.00821	0.017594
CD38	-1.04296	-1.20168	0.008429	0.018033
OC1249078	-1.21915	-1.88182	0.008875	0.018892
RAD21-AS	1.082903	-1.579	0.009156	0.019435
YPEL1	-1.02567	-1.25536	0.009406	0.01993
OC1249021	1.051519	-1.54453	0.009521	0.020154
OC105373	1.11119	-1.51172	0.009871	0.020829
OC124902	-1.21598	-1.70259	0.009889	0.020858
OC1249013	-1.10653	-1.61136	0.00995	0.020974
PRDM8-AS	-1.02059	-1.52638	0.011004	0.023007
OC1005065	-1.07664	-1.51074	0.011188	0.023358
KTI12	1.084443	2.169025	0.012274	0.025435
COL5A3	-1.05878	-1.5427	0.012287	0.025456
D52-MRP	1.331597	-0.57086	0.012441	0.025729
RAMP2-AS	-1.13748	-1.64762	0.01283	0.026476
OC1079851	-1.06995	-1.74085	0.013187	0.027134
OC1249011	-1.07546	-1.64849	0.013292	0.027325
OC1249013	-1.0961	-1.66532	0.014085	0.02876
HURC1-FN	-1.37091	1.400335	0.01489	0.030228
OC1249047	-1.04058	-0.99054	0.015008	0.030449
KLF-CMT	-1.09035	-1.20075	0.015152	0.030713
LGALS2	-1.10108	-1.61176	0.015261	0.030912
OC1053724	-1.1358	-1.99225	0.015594	0.031527
OC1249077	1.199548	-1.68665	0.015774	0.031834
AANAT	-1.02172	-1.63064	0.016663	0.033438
KCNH2	-1.21842	-1.66634	0.016949	0.033966
EIF1B-AS	1.012535	-1.56199	0.017236	0.034487
OC1053719	1.066127	-1.59558	0.017436	0.03484
DTX1	-1.04752	-1.59424	0.017711	0.035352
OC1053772	-1.00058	-1.84107	0.017804	0.035524
PLCXD2	1.051025	-1.35707	0.018414	0.036648
FAM87B	1.040963	-1.68521	0.019758	0.039048
STPG2	1.256727	-1.88436	0.020008	0.039504
LINC0021	-1.10424	-1.7216	0.025221	0.048559
KLHL41	-1.03757	-1.64743	0.026457	0.050645
OC1249094	-1.19829	-1.90333	0.0275	0.052407
LINC0232	-1.04228	-1.47676	0.029547	0.055744
5C1B-RD	-1.07391	-0.81059	0.031035	0.05825
LAIR1	-1.02497	-1.88249	0.034576	0.064166

DCUN1D1	0.50855814	9.95E-37
JADE2	0.53693708	1.66E-41
CUL7	0.50018149	2.11E-35
CTNNA1	0.6991153	4.01E-80
CNTLN	0.5788402	1.90E-49
HSPA5	0.58589426	6.72E-51
DSG2	0.5044822	4.44E-36
YTHDC2	0.51380263	1.41E-37
CTPS2	0.55234353	2.70E-44
ATP6V1H	0.50810014	1.18E-36
POLR2B	0.65920873	2.28E-68
FAM214A	0.52996754	2.73E-40
TPR	0.57266007	3.33E-48
DTNBP1	0.62324266	3.23E-59
MAP4	0.50451928	4.38E-36
GOPC	0.52491451	1.99E-39
USP28	0.51392766	1.34E-37
CC2D2A	0.62376622	2.42E-59
ZNF800	0.5106881	4.51E-37
SNX29	0.73439589	2.87E-92
MRPS10	0.6037925	9.50E-55
RSF1	0.57658443	5.45E-49
VPS13D	0.52964205	3.10E-40
FAM120A	0.64889927	1.28E-65
R3HDM1	0.54132937	2.75E-42
ERCC8	0.52744061	7.41E-40
VAMP3	0.69022619	2.43E-77
ARID1B	0.53824708	9.75E-42
CLPTM1L	0.61341856	6.38E-57
HEXB	0.5485814	1.34E-43
PTCD2	0.50758029	1.43E-36
JKAMP	0.68647474	3.40E-76
MCUR1	0.72404421	1.65E-88
MAPK9	0.61687572	1.01E-57
HERPUD1	0.58329328	2.33E-50
PIK3CB	0.62489682	1.30E-59
SIKE1	0.64269481	5.15E-64
FNIP2	0.57486013	1.21E-48
FOXN3	0.52054142	1.08E-38
METTL24	0.59892427	1.12E-53
AKR7A2	0.65141461	2.80E-66
AP5M1	0.58638827	5.30E-51
TRAPPC3	0.58719988	3.58E-51
THRAP3	0.65155195	2.57E-66
ARID4B	0.5332444	7.38E-41
SDCCAG8	0.58282677	2.90E-50
HHAT	0.66837067	6.62E-71
KIF1B	0.66195475	4.04E-69
FAM168A	0.55622685	5.07E-45
GALC	0.62160673	7.88E-59
SZRD1	0.52982082	2.89E-40
FAM114A2	0.68362746	2.45E-75
CYFIP2	0.57619512	6.53E-49
TAB2	0.53240848	1.03E-40
GINM1	0.65253556	1.41E-66
EIF2AK2	0.56850633	2.21E-47
CCDC85A	0.55826087	2.09E-45
PUM2	0.60779796	1.21E-55
HPF1	0.59827229	1.55E-53
ZFR	0.65244684	1.49E-66
RC3H2	0.52318843	3.90E-39
PKP2	0.55569192	6.39E-45
GDI2	0.64782525	2.44E-65
ATG5	0.55234787	2.69E-44
TMCC3	0.62340202	2.96E-59
PITHD1	0.51277229	2.07E-37
ATP11B	0.58897682	1.52E-51
CDK14	0.51554947	7.28E-38
ATP2B4	0.51584461	6.51E-38
ZC3H11A	0.52451921	2.33E-39
RIOK2	0.59406838	1.26E-52
YIPF1	0.53505876	3.56E-41
NDC1	0.53401363	5.42E-41
GATB	0.55588542	5.88E-45
DNAJC25	0.53763888	1.25E-41
PIGV	0.6647311	6.90E-70
SNRNP40	0.62968839	9.05E-61
QSER1	0.52665662	1.01E-39
NCKAP1	0.72334743	2.91E-88
MRPS35	0.59846389	1.41E-53
GPBP1	0.594808	8.72E-53
ZNF112	0.50500902	3.67E-36
CS	0.53032782	2.36E-40
WAPL	0.5855321	7.99E-51
APPBP2	0.65431432	4.75E-67
EIF4B	0.5524159	2.62E-44
MED29	0.65758815	6.27E-68
RNF4	0.56910731	1.68E-47
INTS13	0.60782448	1.19E-55
TM7SF3	0.74478765	3.17E-96
SPA17	0.6235822	2.68E-59
CDON	0.51602383	6.09E-38
TAF2	0.53738552	1.38E-41
HIPK2	0.61847177	4.30E-58
TNPO3	0.55520433	7.89E-45
DDX20	0.7008409	1.12E-80
BTBD1	0.58764691	2.89E-51
FAR2	0.55022813	6.65E-44
CALCRL	0.53111548	1.73E-40
TAF11	0.59558756	5.92E-53
ANKS1A	0.546535	3.16E-43
AP3D1	0.61426529	4.08E-57
GNAI3	0.59321427	1.91E-52
IPO5	0.5880148	2.42E-51
WDR3	0.62405102	2.07E-59
PKN2	0.62843443	1.83E-60
KARS1	0.54738796	2.21E-43
ADAT1	0.50681415	1.89E-36
TBC1D22B	0.66519187	5.14E-70
SPEN	0.51952262	1.60E-38
ZC3H15	0.6783071	9.22E-74
MAP2K4	0.66972642	2.74E-71
SLK	0.65530906	2.57E-67
FAM107B	0.54014248	4.49E-42
FOXJ2	0.59295819	2.17E-52
ELAVL1	0.58561525	7.68E-51
DIP2B	0.62802239	2.30E-60
SMARCD1	0.5344258	4.59E-41
KDM4A	0.58838951	2.02E-51
NFYC	0.55275753	2.26E-44
ELOVL1	0.53466637	4.17E-41
ZBTB11	0.54734622	2.25E-43
GOLGA5	0.62866791	1.60E-60
LRRC40	0.6838219	2.14E-75
ISOC1	0.66164222	4.92E-69
EML1	0.59536135	6.63E-53
THUMPD1	0.60917369	5.92E-56
MSANTD3	0.50496157	3.73E-36
ZFAT	0.64136584	1.12E-63
MTFR1	0.64168304	9.33E-64
FECH	0.68465426	1.20E-75
MYO9A	0.62218016	5.77E-59
PFKP	0.50811369	1.17E-36
IDI1	0.63811297	7.46E-63
PLPP1	0.55177046	3.45E-44
NEO1	0.63744938	1.09E-62
PKM	0.54852592	1.37E-43
DHX29	0.64939276	9.51E-66
DNTTIP2	0.65794424	5.02E-68
TP53BP1	0.50262207	8.74E-36
RRP15	0.63735478	1.16E-62
RHOA	0.57438134	1.51E-48
DHX8	0.62225665	5.53E-59
PRKCZ	0.55278816	2.23E-44
IARS2	0.7492413	5.57E-98
ROCK1	0.51567457	6.95E-38
PDK3	0.52012031	1.28E-38
COASY	0.51917254	1.83E-38
MEF2A	0.60616842	2.81E-55
OTUD5	0.50620681	2.36E-36
TFE3	0.50988812	6.08E-37
TBC1D25	0.51901502	1.95E-38
INPP5A	0.55978613	1.07E-45
GPKOW	0.6412901	1.17E-63
ATP11A	0.61834624	4.60E-58
POLR1A	0.54647687	3.24E-43
LAPTM4A	0.67726378	1.86E-73
SRBD1	0.72405314	1.63E-88
SIRT2	0.54091068	3.27E-42
ERLEC1	0.58063159	8.20E-50
MAST4	0.62197142	6.46E-59
NUP133	0.68586058	5.21E-76
NUCKS1	0.68260938	4.93E-75
VPS35	0.70034411	1.62E-80
DNAJA2	0.66443286	8.35E-70
MAOB	0.7415229	5.81E-95
RORA	0.5410393	3.10E-42
TGFBR3	0.63544794	3.46E-62
NEDD4	0.59489963	8.33E-53
PIGB	0.6045907	6.32E-55
MAPK6	0.551372	4.09E-44
GNB5	0.62004993	1.84E-58
HDHD5	0.53214022	1.15E-40
LRP6	0.6155955	2.01E-57
ELP1	0.58562753	7.63E-51
PTPN3	0.55083518	5.14E-44
SLC44A1	0.60360264	1.05E-54
SMG6	0.5813725	5.78E-50
EXOC5	0.65214869	1.79E-66
DGCR2	0.5627671	2.88E-46
ZXDC	0.522948	4.28E-39
WIPI1	0.55785621	2.50E-45
NDST1	0.50194949	1.11E-35
AP3M2	0.5772963	3.91E-49
TESK2	0.50194813	1.11E-35
CFAP20	0.60819149	9.86E-56
EIF2B3	0.58468516	1.20E-50
CDC42	0.69786093	1.00E-79
NCK2	0.52929638	3.56E-40
MGAT4A	0.60909614	6.16E-56

MYBPH	-3.18656	-1.6198	5.12E-82	2.27E-80
LIN52	-1.09294	3.438259	5.18E-82	2.29E-80
CCDC186	-1.27901	5.000393	5.32E-82	2.35E-80
LAMA4	2.673069	7.396913	6.58E-82	2.90E-80
SPTSSB	-3.48951	-0.68952	6.81E-82	2.99E-80
AMFR	-1.39133	7.309186	1.12E-81	4.90E-80
SHMT2	1.959725	7.683718	1.86E-81	8.12E-80
SPHK2	-1.18453	3.768764	2.99E-81	1.30E-79
ENO2	3.208227	7.515161	3.69E-81	1.60E-79
TYMS	2.080103	4.773779	3.77E-81	1.63E-79
GAL3ST3	-4.33991	0.374003	3.95E-81	1.71E-79
LRRC2	-3.41244	2.012436	5.32E-81	2.28E-79
RELT	2.098919	3.01004	5.45E-81	2.33E-79
SLC2A12	-3.29504	1.741989	5.84E-81	2.49E-79
COL23A1	4.860123	7.606576	1.29E-80	5.48E-79
SLC9A3	-3.48303	4.971109	2.40E-80	1.02E-78
TNFAIP6	5.932118	5.605267	3.54E-80	1.50E-78
TAP1	1.939051	7.283897	3.78E-80	1.60E-78
ARHGAP2	1.773584	4.956205	6.67E-80	2.82E-78
KCNJ12	-2.32774	1.695499	7.52E-80	3.16E-78
NFRSF10	1.519737	6.62723	1.22E-79	5.14E-78
CDCA2	4.28575	4.352396	2.65E-79	1.11E-77
TCAIM	-1.04823	4.97032	3.88E-79	1.62E-77
CYP4F2	-4.92136	2.627137	4.09E-79	1.70E-77
MPP5	-1.36418	5.61211	4.57E-79	1.90E-77
ADK	-1.1468	4.284121	5.00E-79	2.07E-77
CA9	5.91902	7.663171	5.15E-79	2.12E-77
FXYD4	-6.2759	4.906365	5.34E-79	2.20E-77
RNF149	1.335767	6.517769	7.73E-79	3.18E-77
ERBB2	-1.45973	7.31821	8.12E-79	3.33E-77
CD300A	2.99259	4.410908	8.44E-79	3.45E-77
TYMP	2.962377	6.638373	9.38E-79	3.82E-77
COL4A4	-2.11317	5.148997	9.91E-79	4.03E-77
GAL3ST4	2.905208	4.061026	1.28E-78	5.19E-77
CD300LF	3.028505	2.351531	1.43E-78	5.78E-77
BHLHE41	2.763833	7.069166	1.80E-78	7.28E-77
STAC3	2.293705	2.044201	2.23E-78	8.99E-77
SCCPDH	-1.32969	5.950536	2.40E-78	9.66E-77
RASD1	-2.5333	5.83892	3.01E-78	1.21E-76
KLK6	-5.41658	0.981386	3.48E-78	1.39E-76
BCL9	-1.04892	4.535971	3.87E-78	1.55E-76
ESM1	3.594875	7.940915	4.13E-78	1.64E-76
RIPPLY1	-2.82367	-1.03438	4.48E-78	1.78E-76
SLC25A3	-1.56969	2.473678	5.23E-78	2.07E-76
ITGAX	3.306029	5.038016	6.53E-78	2.58E-76
KIAA1522	-1.39737	6.199058	7.82E-78	3.08E-76
GRIK2	-2.79563	-0.5664	1.17E-77	4.62E-76
TMEM72	-3.00648	5.475382	1.42E-77	5.58E-76
ENOX1	-1.90755	1.282231	1.85E-77	7.23E-76
NKRD34	-3.89855	-1.44168	2.01E-77	7.87E-76
VASH1	2.187029	5.724706	3.02E-77	1.18E-75
TRADD	1.466386	5.280387	3.04E-77	1.18E-75
MYO1F	2.521105	5.155871	3.05E-77	1.18E-75
PLK2	2.233289	6.749411	3.60E-77	1.39E-75
CTSV	-2.8436	1.617942	4.71E-77	1.82E-75
ADAP2	1.864051	4.626834	4.85E-77	1.87E-75
AQP3	-2.20244	7.342755	5.43E-77	2.09E-75
TSPAN6	-1.24975	5.906097	6.18E-77	2.37E-75
RGS19	1.733351	3.906226	8.12E-77	3.11E-75
ACAD8	-1.0772	4.823838	8.93E-77	3.41E-75
LIPH	-3.60473	2.928322	1.28E-76	4.88E-75
GJC1	2.758139	5.240094	1.51E-76	5.75E-75
SCN7A	-3.99227	-0.0611	1.54E-76	5.82E-75
SSC4D	-2.23557	1.411253	1.63E-76	6.18E-75
HOXB6	-2.09311	3.806438	1.75E-76	6.59E-75
SLFN13	2.177209	5.394481	1.79E-76	6.73E-75
CAB39L	-1.61224	4.250241	2.21E-76	8.31E-75
TMCC1	1.799444	6.60487	2.56E-76	9.59E-75
PALD1	1.972398	5.061365	2.83E-76	1.06E-74
PADI2	-2.48459	4.536351	3.06E-76	1.14E-74
TNFRSF4	3.305303	3.104461	3.51E-76	1.31E-74
PIPOX	-2.95789	4.734062	3.81E-76	1.42E-74
TKFC	-1.22101	5.631923	4.54E-76	1.69E-74
PDILT	-2.99391	-3.19537	5.20E-76	1.93E-74
GRIK3	5.037392	4.776909	5.23E-76	1.93E-74
BMP6	-2.11771	3.653622	8.27E-76	3.05E-74
NCCRP1	-2.94742	-0.25977	1.08E-75	3.99E-74
PLXDC1	2.885636	5.587909	1.55E-75	5.69E-74
RASSF10	-3.08927	1.015059	1.63E-75	5.96E-74
ARHGDIB	1.670317	7.9459	1.78E-75	6.49E-74
IGSF10	-2.72796	0.025247	1.83E-75	6.67E-74
POMGNT	-1.12048	4.112106	3.97E-75	1.44E-73
GGACT	-2.34963	3.590971	4.49E-75	1.63E-73
GALNT3	-2.56613	3.366747	5.44E-75	1.97E-73
PSMB8	1.640491	7.072338	5.94E-75	2.15E-73
PLAT	-1.77168	6.010732	6.99E-75	2.52E-73
NLRC5	2.262162	5.750281	8.29E-75	2.98E-73
IGFBP3	3.487238	11.1666	1.09E-74	3.92E-73
HOXB9	-3.29991	3.668326	1.11E-74	3.97E-73
DCXR	-2.07004	5.381355	1.29E-74	4.61E-73
IL4R	1.478713	6.195992	1.40E-74	4.98E-73
DPEP2	2.295845	2.501878	1.78E-74	6.34E-73
DOC2A	6.137304	4.939677	2.00E-74	7.08E-73
PDK1	1.92385	6.074458	2.12E-74	7.51E-73
TMEM91	3.744065	5.448447	2.41E-74	8.52E-73
SLC5A11	-3.06353	0.352598	2.89E-74	1.02E-72
NUDT10	-2.66619	-0.20615	2.93E-74	1.03E-72
MOGAT2	-4.2458	-1.21341	3.11E-74	1.09E-72
SNRPN	-1.20191	6.161929	3.58E-74	1.25E-72
NR1I3	-2.4591	0.233627	4.61E-74	1.61E-72
LGALS9	2.193694	5.63225	4.78E-74	1.67E-72
TRPM2	2.774144	3.845838	5.92E-74	2.06E-72
RAET1E	-1.8648	0.019586	6.70E-74	2.32E-72
SRL	-1.88485	1.546617	8.99E-74	3.11E-72
SUOX	-1.05353	5.401892	9.21E-74	3.18E-72
LAPTM5	2.553098	8.229429	1.11E-73	3.84E-72
FBP1	-2.3219	5.974767	1.15E-73	3.96E-72
LPCAT1	2.555944	7.9569	1.52E-73	5.21E-72
CGN	-2.43727	4.654889	1.52E-73	5.21E-72
HSPB7	-3.25778	3.556251	1.63E-73	5.58E-72
CHL1	-3.32126	3.94276	1.78E-73	6.07E-72
TNFRSF1	2.153202	7.028889	2.13E-73	7.24E-72
GAS2L3	3.130358	5.3125	2.32E-73	7.85E-72
FAM219A	1.081617	5.131252	2.36E-73	7.97E-72
CPEB3	-1.34125	3.581405	2.66E-73	8.98E-72
NUSAP1	2.160424	4.222458	2.80E-73	9.44E-72
TBXAS1	1.815794	4.849846	3.11E-73	1.05E-71
TTC36	-2.76279	-0.24819	4.91E-73	1.64E-71
MYO6	-1.22469	6.883153	6.92E-73	2.31E-71
ADM	2.726463	7.632176	7.23E-73	2.41E-71
TSPAN8	-3.93401	3.463545	8.34E-73	2.78E-71
TMIGD3	2.939043	3.95392	8.84E-73	2.94E-71
NICN1	-1.06666	4.072457	9.65E-73	3.20E-71
IGSF6	2.700463	4.314512	1.03E-72	3.40E-71
APOC1	5.093034	6.662987	1.16E-72	3.82E-71
INHBB	3.834141	6.165322	1.20E-72	3.97E-71
CCDC160	-2.37802	1.330483	1.22E-72	4.00E-71
CKM	-2.88639	-1.39558	1.45E-72	4.78E-71
PDP2	-1.49645	3.946213	1.55E-72	5.07E-71
SLC34A3	-3.38439	1.908531	1.72E-72	5.62E-71
TACSTD2	-3.52381	6.310307	1.76E-72	5.74E-71
NDST3	-3.04868	-1.08321	1.99E-72	6.49E-71
FCGR3A	3.349637	7.116544	2.12E-72	6.89E-71
MBOAT2	-1.55533	4.581144	2.24E-72	7.26E-71
ERBB4	-3.89922	3.550571	2.71E-72	8.77E-71
BCAM	-1.68217	8.201779	3.05E-72	9.86E-71
PLXNB1	-1.4113	6.383614	3.39E-72	1.09E-70
ARL4D	-2.69003	3.751776	7.07E-72	2.28E-70
LDHA	1.687495	10.6745	9.47E-72	3.04E-70
PAQR5	-1.7647	5.85568	9.77E-72	3.14E-70
PSMB9	2.239045	6.109318	1.19E-71	3.80E-70
RFC2	1.116381	4.732708	1.32E-71	4.21E-70
RAP1GAP	-2.55448	6.371731	1.49E-71	4.76E-70
IKBIP	1.830429	5.730204	1.54E-71	4.90E-70
IL2RB	3.018766	4.699862	1.86E-71	5.90E-70
SLC34A1	-4.40167	4.88252	1.92E-71	6.10E-70
DISC1	1.669269	3.601114	2.18E-71	6.91E-70
EHD2	2.2605	8.33366	2.53E-71	8.01E-70
KCTD1	-1.95754	4.001527	2.69E-71	8.50E-70
LAT2	2.172079	4.002157	3.92E-71	1.24E-69
N4BP2L1	1.363024	4.516003	4.73E-71	1.49E-69
PLAU	-2.1695	6.043899	5.29E-71	1.66E-69
MAL2	-2.73624	6.311098	5.38E-71	1.68E-69
AEN	1.411704	5.166536	5.59E-71	1.75E-69
ELMO1	1.81321	5.468752	5.61E-71	1.75E-69
BTN3A1	1.890057	6.288808	6.12E-71	1.91E-69
FATE1	3.534409	0.908402	6.19E-71	1.93E-69
PFKFB4	2.427595	4.80599	6.77E-71	2.10E-69
HLA-A	1.711203	11.03386	7.59E-71	2.35E-69
RAPGEF3	-1.73474	5.107158	8.42E-71	2.60E-69
FABP1	-4.40931	3.466815	9.43E-71	2.91E-69
P2RX2	-3.46741	-3.45249	1.34E-70	4.13E-69
LAIR1	2.659385	5.344342	1.42E-70	4.38E-69
SUCLG2	-1.2676	6.329111	2.01E-70	6.17E-69
TYROBP	2.674535	6.311265	2.49E-70	7.61E-69
AHNAK2	4.107453	6.971694	3.10E-70	9.48E-69
NAPEPLD	-1.17067	4.991335	3.88E-70	1.18E-68
HCLS1	1.921029	6.256172	3.96E-70	1.20E-68
WIPF1	1.419007	6.643179	4.08E-70	1.24E-68
CAND2	-1.80665	1.897931	5.40E-70	1.63E-68
PPP1R18	1.671379	6.332568	5.51E-70	1.66E-68
ATG12	1.019449	5.376245	5.69E-70	1.72E-68
TOM1L2	-1.04541	6.160802	6.16E-70	1.85E-68
EZH2	1.876682	2.910612	6.40E-70	1.92E-68
ACTRT3	-1.7649	0.799559	7.71E-70	2.31E-68
COL4A5	-2.31362	3.987218	8.05E-70	2.41E-68
SMIM43	5.099558	0.780262	1.23E-69	3.66E-68
SULT2B1	-3.17383	0.892605	1.33E-69	3.96E-68
SYT7	-3.35946	3.018132	1.37E-69	4.09E-68
ADAMTS	2.456434	3.697322	1.65E-69	4.92E-68
TENT5B	-2.57696	1.911431	2.00E-69	5.93E-68
ACKR2	-2.27237	0.598305	3.01E-69	8.91E-68
AKIRIN1	-1.08281	5.581543	3.08E-69	9.12E-68
MAGI3	-1.78144	4.009766	4.40E-69	1.30E-67
LILRB2	2.301462	3.828355	5.13E-69	1.51E-67
PCCB	-1.64224	5.274309	5.66E-69	1.66E-67
SLC13A2	-4.67359	3.265758	6.10E-69	1.79E-67
CASP1	1.752763	4.825669	6.30E-69	1.85E-67
FDX1	-1.10905	4.834582	7.32E-69	2.14E-67
FMNL1	2.255706	5.069088	7.76E-69	2.26E-67

SNX13	0.52251769	5.06E-39
ING3	0.5598927	1.02E-45
SEL1L	0.52913013	3.80E-40
ATP6AP1	0.52543714	1.63E-39
RDH11	0.59201218	3.45E-52
ZFYVE26	0.52637357	1.13E-39
EPN2	0.53382849	5.84E-41
ALDH3A2	0.6977702	1.07E-79
AFF4	0.55128919	4.23E-44
MPP5	0.64568621	8.77E-65
RHOBTB1	0.55248141	2.54E-44
SMC1A	0.64756018	2.86E-65
MARK2	0.5410985	3.03E-42
TRHDE	0.58854811	1.87E-51
NFATC3	0.61965067	2.28E-58
FBXW11	0.63520332	3.98E-62
EVC	0.54876374	1.24E-43
DERL2	0.50138233	1.37E-35
NDE1	0.50560532	2.95E-36
PDE8A	0.57099314	7.14E-48
CLCN4	0.72298359	3.91E-88
SMARCE1	0.65600905	1.67E-67
FERMT2	0.50953835	6.92E-37
MAP3K13	0.69995399	2.16E-80
PICALM	0.62655779	5.19E-60
NSF	0.67588705	4.68E-73
CLASP1	0.53521523	3.34E-41
TSG101	0.6749238	8.91E-73
NTN4	0.60054976	4.94E-54
DPP8	0.6041107	8.08E-55
LMAN1	0.59751197	2.27E-53
HACD3	0.60037511	5.39E-54
ENO1	0.58690261	4.14E-51
ACTR6	0.64642763	5.64E-65
TIPIN	0.61089892	2.40E-56
EIF4G3	0.53253053	9.82E-41
TTC38	0.61724715	8.31E-58
ACAT1	0.68214352	6.79E-75
ZNF638	0.56115831	5.87E-46
TIMM21	0.63957395	3.20E-63
FRYL	0.53737954	1.39E-41
TMEM131	0.60844691	8.64E-56
PLD1	0.64119826	1.24E-63
DLG1	0.56498596	1.07E-46
RAB7A	0.59954486	8.19E-54
BCAP29	0.55560431	6.64E-45
SART3	0.58203709	4.22E-50
KIFAP3	0.7724222	9.45E-108
REXO2	0.56913614	1.66E-47
RBM7	0.53542101	3.07E-41
RBMS2	0.52237743	5.34E-39
MLH1	0.50592467	2.62E-36
UNG	0.54602055	3.92E-43
FMO4	0.55919015	1.39E-45
KLHL20	0.67846276	8.30E-74
SLC46A1	0.62324305	3.22E-59
PLXNA2	0.53794786	1.10E-41
GPATCH1	0.52540799	1.64E-39
TM9SF3	0.57906327	1.71E-49
DNAJC10	0.55550075	6.94E-45
GTF3C1	0.55089339	5.01E-44
USP33	0.68134653	1.17E-74
DYNC1I2	0.75154295	6.68E-99
POLD3	0.52722077	8.08E-40
GPR137B	0.58987968	9.79E-52
SLC25A43	0.59792768	1.84E-53
UBE2A	0.51384715	1.38E-37
ARAF	0.5614444	5.17E-46
MCCC1	0.61971343	2.20E-58
NEBL	0.53130865	1.60E-40
UBE2K	0.60886461	6.95E-56
PIK3C3	0.63239173	1.97E-61
TULP3	0.57057428	8.65E-48
PPP2R5C	0.5450394	5.91E-43
GNB1	0.67105226	1.15E-71
MLLT10	0.53324644	7.37E-41
VDAC3	0.55429582	1.17E-44
PCM1	0.56053446	7.73E-46
ITCH	0.59508175	7.61E-53
TP53INP2	0.54014991	4.47E-42
TOLLIP	0.70435049	8.22E-82
XRCC5	0.67312434	2.94E-72
LXN	0.5658677	7.24E-47
TNS1	0.57672644	5.10E-49
SAR1A	0.53668121	1.84E-41
CEACAM1	0.5551686	8.01E-45
SENP1	0.51479882	9.67E-38
FDFT1	0.59569083	5.63E-53
OPHN1	0.5184431	2.42E-38
SCGN	0.53339625	6.94E-41
CARMIL1	0.54954367	8.89E-44
PGM1	0.51558778	7.18E-38
DDX1	0.76845467	5.35E-106
STX7	0.64325382	3.70E-64
KEAP1	0.58618197	5.85E-51
RIF1	0.55105176	4.68E-44
RAB21	0.73018347	1.02E-90
SLC4A4	0.66585373	3.36E-70
SMARCA2	0.63048626	5.78E-61
MID2	0.61548357	2.13E-57
CNOT4	0.59524222	7.03E-53
PSEN1	0.62465364	1.49E-59
HSP90AA1	0.50381814	5.66E-36
RSBN1	0.63576994	2.88E-62
OSTM1	0.55440394	1.11E-44
PCNP	0.54765552	1.97E-43
EXD2	0.58613201	5.99E-51
UBA5	0.52382118	3.05E-39
CDC14B	0.66260135	2.68E-69
ZNF510	0.55140911	4.02E-44
LRP2	0.69347402	2.41E-78
JMJD4	0.51504008	8.83E-38
DUSP12	0.53860672	8.42E-42
DELE1	0.51623779	5.61E-38
SLC13A1	0.54128617	2.80E-42
CADPS2	0.57015511	1.05E-47
WDR70	0.50397479	5.34E-36
STRADB	0.57077998	7.87E-48
BZW1	0.58163562	5.11E-50
C5orf22	0.58401789	1.65E-50
GEMIN5	0.59718993	2.67E-53
NFE2L1	0.63564761	3.09E-62
GSK3B	0.55310875	1.94E-44
ERC1	0.59064616	6.74E-52
RNF13	0.69915238	3.90E-80
TRPM3	0.62007316	1.81E-58
PALB2	0.52898137	4.03E-40
LYRM2	0.59258856	2.60E-52
BCKDHB	0.57756132	3.46E-49
KAT6A	0.56098924	6.32E-46
ULK2	0.51379573	1.41E-37
TNPO1	0.54606415	3.85E-43
DIS3	0.63856316	5.75E-63
PIBF1	0.54337598	1.18E-42
NUFIP1	0.63881429	4.97E-63
PDS5B	0.5603663	8.32E-46
OXCT1	0.57100429	7.11E-48
CYLD	0.51795945	2.92E-38
ZNF324	0.50608753	2.47E-36
ZNF416	0.55821508	2.13E-45
ZNF264	0.50835992	1.07E-36
YTHDC1	0.64676014	4.62E-65
CHMP2B	0.54568151	4.52E-43
ZMPSTE24	0.72010129	4.02E-87
STARD7	0.67532458	6.82E-73
NOA1	0.5311936	1.68E-40
REST	0.5499349	7.53E-44
WBP11	0.56818501	2.55E-47
NCOA1	0.64741512	3.12E-65
AGBL5	0.53568585	2.76E-41
RAB10	0.67987943	3.18E-74
HADHA	0.78397197	4.60E-113
CD59	0.57968406	1.28E-49
BCORL1	0.55727853	3.21E-45
ATRX	0.53599138	2.44E-41
AK6	0.54335723	1.19E-42
MYNN	0.59327628	1.86E-52
SCAMP1	0.51726126	3.80E-38
PREP	0.52303938	4.13E-39
HACE1	0.50032808	2.00E-35
SEH1L	0.61412766	4.38E-57
WDR47	0.68132382	1.19E-74
WDFY1	0.62754098	3.01E-60
SLC25A24	0.59536439	6.62E-53
CPNE3	0.61427046	4.06E-57
RRN3	0.59806326	1.72E-53
MTIF2	0.57253795	3.53E-48
DDHD2	0.5579626	2.38E-45
EPS15	0.59029878	7.98E-52
CHERP	0.53167738	1.38E-40
DNAJA1	0.6494926	8.96E-66
CHMP5	0.67337292	2.49E-72
NFX1	0.61854149	4.15E-58
IPO11	0.56382819	1.80E-46
SEPHS1	0.65974565	1.63E-68
RBM22	0.58988356	9.77E-52
TMED2	0.50495941	3.73E-36
ERO1B	0.66428405	9.19E-70
ZFAND6	0.62739826	3.25E-60
TXLNG	0.56713305	4.10E-47
HUWE1	0.57177141	5.01E-48
ZW10	0.6513317	2.94E-66
ALG9	0.57872718	2.01E-49
MTMR2	0.65116316	3.26E-66
NLK	0.65090163	3.82E-66
PIGS	0.60836185	9.03E-56
NOP14	0.59288924	2.25E-52
ADD1	0.69823884	7.62E-80
L2HGDH	0.57638694	5.97E-49
TXNDC16	0.60096628	4.00E-54

PFKP	1.974972	8.91797	1.60E-68	4.66E-67
GPR182	-2.42849	-0.28905	1.69E-68	4.92E-67
SCNN1B	-3.99475	4.249197	1.91E-68	5.53E-67
SCD	3.029393	8.773076	2.46E-68	7.14E-67
ENPP3	4.593893	8.060394	3.59E-68	1.04E-66
CYP2B6	-4.50501	1.451299	4.45E-68	1.29E-66
C1orf127	3.116661	-0.90216	5.44E-68	1.57E-66
FMN2	-3.76612	0.241289	6.97E-68	2.01E-66
DHRS11	-1.42169	3.11943	7.13E-68	2.05E-66
GPSM3	1.943778	4.972955	8.06E-68	2.31E-66
CSPG4	2.749764	7.16568	9.06E-68	2.59E-66
PSORS1C	2.974915	2.63854	9.31E-68	2.66E-66
SLC37A2	2.756358	4.221195	1.01E-67	2.88E-66
PYGL	2.02582	6.130487	1.04E-67	2.96E-66
P4HA1	1.711682	7.244021	1.69E-67	4.80E-66
RPS6KA6	-2.97014	3.283319	2.04E-67	5.79E-66
PDLIM1	1.687513	7.513433	2.07E-67	5.86E-66
CAV1	2.246902	7.980357	2.08E-67	5.86E-66
CMTM4	-1.85773	6.344681	2.16E-67	6.08E-66
SGPP1	-1.25846	5.260062	2.44E-67	6.88E-66
ABCA13	-3.52367	-0.22311	2.76E-67	7.74E-66
SLC6A17	-2.74449	1.158564	3.36E-67	9.40E-66
SH3BP2	1.691667	7.396703	3.92E-67	1.09E-65
PRELID2	2.084561	3.112075	5.71E-67	1.59E-65
ACADM	-1.51033	6.620557	7.10E-67	1.98E-65
CHGB	-3.62464	2.674018	7.42E-67	2.06E-65
FCER1G	2.550257	5.5321	7.82E-67	2.17E-65
NNMT	3.936538	9.484094	7.84E-67	2.17E-65
C7	-3.30122	7.734352	8.43E-67	2.33E-65
KLRD1	2.346767	2.768241	9.72E-67	2.68E-65
TCIM	-2.17073	6.106849	9.96E-67	2.74E-65
BTN3A2	2.004929	6.894609	1.06E-66	2.90E-65
DLX5	3.425043	1.33069	1.13E-66	3.11E-65
GMFG	1.858975	5.070189	1.35E-66	3.70E-65
ANGPT2	3.123277	6.864101	1.62E-66	4.41E-65
YBX3	1.527127	8.137222	1.78E-66	4.86E-65
C1QTNF6	2.294264	4.438853	1.83E-66	4.98E-65
SPI1	2.371808	4.961912	1.85E-66	5.03E-65
GRAMD1	1.321022	6.083886	1.94E-66	5.27E-65
SLC12A3	-4.85843	6.114986	2.04E-66	5.51E-65
SNX33	1.33044	6.688144	2.70E-66	7.29E-65
BIN2	2.023899	4.025579	5.50E-66	1.48E-64
DACH2	-3.16102	-1.33971	5.58E-66	1.50E-64
SIGLEC8	4.344848	2.991651	5.73E-66	1.54E-64
CLEC2D	2.56898	3.900962	6.93E-66	1.85E-64
CLDN11	-2.47037	1.890153	7.58E-66	2.03E-64
ACOX2	-1.87864	4.013355	7.62E-66	2.03E-64
MYO9B	1.178625	6.435366	9.08E-66	2.42E-64
FREM1	-3.30143	3.411344	9.24E-66	2.46E-64
USP53	-1.37566	5.352507	9.66E-66	2.57E-64
APOBEC3	2.344128	4.799488	9.98E-66	2.65E-64
SIGLEC1	2.988226	3.938552	1.18E-65	3.14E-64
CGNL1	-2.10146	7.174246	1.23E-65	3.26E-64
MYH10	-1.73076	6.738265	1.34E-65	3.53E-64
PIK3R6	3.391405	2.195512	1.36E-65	3.58E-64
OSCAR	2.59298	2.270227	1.41E-65	3.71E-64
SASH3	2.494578	4.599781	1.46E-65	3.85E-64
ARHGEF3	-1.06397	5.309328	1.51E-65	3.96E-64
GJA3	-2.97601	-0.21672	1.77E-65	4.63E-64
GPM6B	-2.11758	2.739907	1.90E-65	4.96E-64
TAF1D	1.518339	5.890167	1.95E-65	5.08E-64
C9orf135	-3.46699	-1.82703	2.09E-65	5.44E-64
RASSF2	2.208195	5.703338	2.16E-65	5.63E-64
PAG1	1.748047	5.596833	2.30E-65	5.98E-64
SLC36A2	-4.64092	3.55583	3.02E-65	7.81E-64
NFAM1	2.128908	3.931386	3.28E-65	8.46E-64
IPO13	-1.03861	5.415583	3.34E-65	8.62E-64
AP1M2	-2.46058	4.488005	3.43E-65	8.82E-64
OR2A4	6.679109	0.223621	3.98E-65	1.02E-63
LTB4R	2.545546	3.601495	4.56E-65	1.17E-63
EVL	1.696347	6.291499	5.15E-65	1.32E-63
NPY5R	-2.41194	0.489886	6.63E-65	1.70E-63
OAT	-1.18998	5.861744	7.87E-65	2.01E-63
P2RY1	2.450009	3.872633	8.42E-65	2.15E-63
IL12RB1	2.602803	2.728205	1.12E-64	2.86E-63
KLHL6	2.630055	3.690117	1.14E-64	2.91E-63
FAM217A	-2.33855	-2.3979	1.19E-64	3.03E-63
LPIN2	-1.02751	6.270129	1.27E-64	3.23E-63
CCND1	2.312968	9.616634	1.68E-64	4.26E-63
C1orf162	2.358987	4.894357	2.48E-64	6.26E-63
ALDH3B2	-3.90018	-1.10659	2.78E-64	7.02E-63
ISG20	2.37061	3.83436	2.97E-64	7.50E-63
RDH10	-1.62881	5.746129	2.99E-64	7.54E-63
CDK18	2.380072	8.220223	3.23E-64	8.12E-63
ANXA4	1.864102	9.477089	3.40E-64	8.53E-63
CD68	3.175471	1.985605	3.61E-64	9.04E-63
MT1H	-4.33503	3.518153	5.30E-64	1.32E-62
CASP4	1.263303	5.811913	5.32E-64	1.33E-62
STK10	1.202586	5.787701	8.88E-64	2.21E-62
INPP5D	1.699915	5.654648	9.30E-64	2.31E-62
ITGAD	4.769672	1.212393	9.66E-64	2.40E-62
PTPRQ	-3.94135	-1.19135	9.90E-64	2.45E-62
KCNQ1	-1.84806	5.034161	1.06E-63	2.61E-62
LY86	2.499503	3.601932	1.07E-63	2.64E-62
MYORG	-1.50999	4.658557	1.19E-63	2.94E-62
CNTN1	-3.67571	2.888426	1.32E-63	3.25E-62
GAB3	1.632103	3.075648	1.32E-63	3.25E-62
SOX11	5.40057	2.508052	1.37E-63	3.36E-62
RASD2	2.90702	3.807761	1.38E-63	3.38E-62
LILRB4	3.137052	4.484572	1.45E-63	3.54E-62
TSPYL4	-1.04905	5.198293	1.47E-63	3.58E-62
NDUFS1	-1.17705	6.991185	1.51E-63	3.69E-62
TUBB2B	-2.59023	1.967529	1.65E-63	4.03E-62
DGKZ	1.295757	6.25413	1.68E-63	4.09E-62
ARRDC2	1.991246	6.384336	1.68E-63	4.09E-62
CXorf58	-1.8608	-2.90946	2.35E-63	5.71E-62
BAX	1.247358	5.612573	2.60E-63	6.31E-62
VWA2	-2.91311	-0.19389	2.71E-63	6.55E-62
MPPED2	-2.44784	2.91093	2.96E-63	7.15E-62
OTOGL	-2.47795	1.657791	2.97E-63	7.17E-62
SLC16A1	2.057299	6.45843	3.33E-63	8.04E-62
ACOT11	-1.9688	3.791003	4.48E-63	1.08E-61
FCGR1B	2.857794	0.642529	4.86E-63	1.17E-61
STX4	1.183922	5.815325	5.19E-63	1.24E-61
ADAMTS1	-2.64522	2.345047	5.28E-63	1.26E-61
FAM169A	-3.0825	1.962699	5.33E-63	1.27E-61
MAGEE2	-2.6799	-3.00943	5.36E-63	1.28E-61
ATP1B1	-1.35004	10.14751	5.64E-63	1.34E-61
PM20D1	-2.83169	0.430829	6.14E-63	1.46E-61
TRAM1L1	-1.49706	2.590799	6.17E-63	1.47E-61
RHEBL1	1.917995	-0.01129	6.40E-63	1.52E-61
TMSB10	2.144185	10.21182	6.54E-63	1.55E-61
MYO3A	3.332525	3.41465	8.51E-63	2.01E-61
GRM1	-3.35313	0.628321	8.56E-63	2.02E-61
CCDC88B	2.590588	4.091762	9.68E-63	2.28E-61
SIGLEC9	2.324822	2.298264	1.12E-62	2.64E-61
LARS2	-1.38109	4.389435	1.20E-62	2.81E-61
SDS	4.497295	3.956348	1.35E-62	3.17E-61
ECHS1	-1.30959	7.489638	1.39E-62	3.26E-61
HLX	2.553386	4.480681	1.41E-62	3.30E-61
SCN1B	2.016267	4.194015	1.50E-62	3.50E-61
SYNJ2BP	-1.2216	5.850288	1.58E-62	3.70E-61
INSRR	-3.58944	0.111508	1.99E-62	4.64E-61
SQOR	1.286171	5.806842	2.12E-62	4.93E-61
IFNGR2	1.121658	6.784268	2.26E-62	5.26E-61
FABP6	5.67504	4.414608	2.51E-62	5.83E-61
BRCC3	-1.05538	3.994867	2.55E-62	5.92E-61
CSF3R	2.768331	4.223974	2.76E-62	6.40E-61
TMEM14	1.434152	6.828715	2.86E-62	6.62E-61
MS4A7	2.438843	5.89736	2.94E-62	6.80E-61
CYP2J2	5.150346	7.11119	3.23E-62	7.46E-61
WASF3	-1.54376	3.486043	3.34E-62	7.69E-61
DPP9	1.436287	6.393084	3.37E-62	7.74E-61
SCNN1G	-4.75149	5.050866	5.28E-62	1.21E-60
OLFML2A	2.697444	7.218949	5.82E-62	1.33E-60
LILRB3	2.470319	1.834717	6.53E-62	1.49E-60
FBXL8	1.921929	2.94559	7.19E-62	1.64E-60
SFXN3	1.236562	6.382581	7.81E-62	1.78E-60
APOBEC3	2.905258	0.862644	8.49E-62	1.94E-60
ARL11	1.949618	1.595672	8.88E-62	2.02E-60
ZDHHC2	-1.79913	5.516519	9.78E-62	2.22E-60
ITGB2	2.405642	7.201914	1.15E-61	2.61E-60
RGS6	-2.30968	-0.07335	1.23E-61	2.80E-60
PEBP1	-1.19034	9.217738	1.26E-61	2.85E-60
RAB42	4.878948	4.8281	1.31E-61	2.95E-60
FCGR1A	3.104414	3.041049	1.57E-61	3.53E-60
MBOAT4	2.366337	-1.06188	1.61E-61	3.62E-60
E2F1	2.201202	2.640072	1.70E-61	3.83E-60
C1QB	2.944102	7.9981	1.75E-61	3.92E-60
RUNX3	2.698193	4.571606	2.06E-61	4.62E-60
LZTS1	2.403075	5.40032	2.20E-61	4.92E-60
CDKL1	-1.54483	2.974253	2.48E-61	5.55E-60
POU5F1	3.426977	4.979344	2.76E-61	6.17E-60
PCCA	-1.45241	5.762781	3.14E-61	7.00E-60
ARHGAP	2.568971	3.765418	3.16E-61	7.05E-60
PPP1R1B	-3.74622	0.428315	3.57E-61	7.95E-60
HTRA4	4.805909	0.546014	3.67E-61	8.15E-60
PPP1R3G	2.148586	3.162283	4.47E-61	9.93E-60
EPN3	-3.76564	2.12701	4.84E-61	1.07E-59
FERMT3	2.121049	4.870788	6.35E-61	1.41E-59
MYH7	-3.06227	-3.00272	6.72E-61	1.49E-59
TBX21	2.478364	1.263799	7.49E-61	1.65E-59
PHYKPL	1.646294	6.177804	7.56E-61	1.67E-59
PAQR8	-1.3391	4.165429	8.44E-61	1.86E-59
FRG2C	-4.08978	-0.4509	8.66E-61	1.90E-59
TMPRSS4	-3.85354	2.488706	9.01E-61	1.98E-59
TBX3	-1.82682	3.75888	1.05E-60	2.31E-59
PALM	-1.60261	4.94759	1.07E-60	2.34E-59
HOXB5	-1.94349	2.361045	1.15E-60	2.52E-59
IFI16	1.625021	6.691989	1.37E-60	2.99E-59
GMPR	-2.28893	3.645436	1.38E-60	3.00E-59
C4orf47	2.527992	2.698187	1.40E-60	3.06E-59
EHBP1L1	1.450373	5.894398	1.54E-60	3.35E-59
BTK	2.203892	3.367303	1.54E-60	3.35E-59
FGD2	2.525138	3.822092	1.64E-60	3.56E-59
IFFO1	1.786994	4.575494	2.56E-60	5.55E-59
CD247	2.598435	3.459567	2.72E-60	5.89E-59
CNGA1	-2.49429	1.601523	2.79E-60	6.03E-59
SSTR5	-3.75701	-1.22598	2.86E-60	6.17E-59
VAV1	2.326479	3.779134	2.90E-60	6.27E-59

GMCL1	0.53021014	2.48E-40
SF3B2	0.64868339	1.46E-65
METTL2A	0.61839125	4.49E-58
ALG6	0.58573869	7.24E-51
PTPN4	0.57959978	1.33E-49
DDX18	0.69307681	3.20E-78
KHSRP	0.54722614	2.36E-43
GNA11	0.52940879	3.41E-40
EPB41L1	0.5685489	2.17E-47
TGDS	0.66511973	5.38E-70
ARHGAP28	0.56730701	3.79E-47
PPP1R13B	0.54639719	3.35E-43
ATRN	0.54439297	7.74E-43
NSFL1C	0.56956015	1.37E-47
C20orf194	0.53763025	1.25E-41
XRN2	0.52540785	1.64E-39
SNX5	0.69756553	1.25E-79
ESF1	0.53210794	1.16E-40
RBBP9	0.71203814	2.33E-84
GCN1	0.52688058	9.23E-40
KIF16B	0.59062402	6.81E-52
PEBP1	0.6314115	3.43E-61
BRAP	0.57981696	1.20E-49
GANAB	0.56985819	1.20E-47
DDX24	0.56503614	1.05E-46
NECAP1	0.6859451	4.91E-76
DHX32	0.51801	2.86E-38
RCOR1	0.5698694	1.19E-47
SPTLC1	0.63534888	3.66E-62
PAPOLA	0.57433219	1.55E-48
CCNK	0.510141	5.53E-37
MUL1	0.70141512	7.34E-81
PDCD7	0.56435957	1.42E-46
SPG21	0.67206729	5.90E-72
ZNF268	0.51446062	1.10E-37
USP48	0.54108962	3.04E-42
AARS1	0.57664355	5.30E-49
GLG1	0.59830262	1.53E-53
EXOC1	0.59328809	1.85E-52
RBM27	0.54257309	1.65E-42
OSBPL8	0.6276291	2.86E-60
DLD	0.61793009	5.76E-58
WDR7	0.6299543	7.80E-61
TXNL1	0.56593269	7.03E-47
ITGA6	0.66989832	2.45E-71
FH	0.62494915	1.26E-59
SEL1L3	0.51019081	5.43E-37
CDV3	0.50204402	1.08E-35
ALKBH5	0.67303958	3.11E-72
SCFD1	0.64932872	9.89E-66
HNRNPC	0.68682291	2.67E-76
SUPT16H	0.60461307	6.24E-55
TOX4	0.62646885	5.45E-60
TINF2	0.57310139	2.72E-48
TRPM7	0.52722532	8.06E-40
WDR76	0.50807635	1.19E-36
SNAP23	0.61080916	2.52E-56
EZR	0.67295532	3.29E-72
AGO1	0.63925419	3.85E-63
RFFL	0.61306618	7.69E-57
MFSD11	0.6246224	1.51E-59
DPYSL2	0.63923928	3.88E-63
GPATCH2	0.51927401	1.76E-38
NUP50	0.55048029	5.97E-44
ECHDC1	0.64822383	1.92E-65
SEC22C	0.50388485	5.52E-36
XYLB	0.6509909	3.62E-66
UPRT	0.66501477	5.76E-70
CDC23	0.68088859	1.60E-74
CBX5	0.53304419	8.00E-41
MSH2	0.62822497	2.05E-60
ARCN1	0.65391245	6.08E-67
TMEM38B	0.69185393	7.66E-78
PSMD5	0.57406245	1.75E-48
NUP188	0.55783653	2.52E-45
CRAT	0.62436268	1.74E-59
IKZF5	0.57662743	5.34E-49
WAC	0.70116741	8.82E-81
BRPF3	0.65766224	5.98E-68
MRPS18A	0.66799705	8.43E-71
TMEM14A	0.72051141	2.89E-87
HSP90AB1	0.53082564	1.94E-40
CDC5L	0.6615181	5.33E-69
ZNF184	0.6382498	6.89E-63
SIRT1	0.62906876	1.28E-60
HNRNPH3	0.59844898	1.42E-53
IFT74	0.51087832	4.21E-37
ABL1	0.51911015	1.88E-38
SH3GLB1	0.63492738	4.66E-62
SYDE2	0.68442595	1.41E-75
PCSK5	0.52382523	3.05E-39
TMED1	0.51067479	4.54E-37
ABLIM1	0.62668042	4.85E-60
RAB18	0.70029693	1.68E-80
NRP1	0.54618023	3.67E-43
WASHC2A	0.62130896	9.27E-59
HNRNPM	0.65512257	2.89E-67
MTAP	0.59472805	9.07E-53
POLR2E	0.55626404	4.99E-45
KLHL22	0.55878425	1.66E-45
SNAP29	0.73873247	6.75E-94
CRKL	0.65843554	3.69E-68
SMARCB1	0.61485695	2.98E-57
BCL2L13	0.65212821	1.81E-66
SF3A1	0.6936476	2.12E-78
SPECC1L	0.55999622	9.79E-46
UPB1	0.56362356	1.97E-46
SNRPD3	0.59341604	1.73E-52
PES1	0.5948877	8.38E-53
MAPK1	0.77553422	3.76E-109
GGT1	0.59164013	4.15E-52
HIRA	0.55513229	8.14E-45
SRRD	0.60029776	5.61E-54
PATZ1	0.58519926	9.37E-51
TFIP11	0.56077503	6.95E-46
EIF3L	0.56261182	3.09E-46
TTC28	0.61134545	1.90E-56
SLC5A1	0.52251411	5.07E-39
TCF20	0.58024504	9.84E-50
TOMM22	0.5357812	2.66E-41
RTCB	0.62413136	1.98E-59
JOSD1	0.56362786	1.97E-46
FBXO7	0.66017209	1.24E-68
POLDIP3	0.63077113	4.93E-61
RAB36	0.53148728	1.49E-40
TIMP3	0.57399936	1.80E-48
SUN2	0.5443925	7.74E-43
DNAL4	0.52129627	8.11E-39
PACSIN2	0.57083372	7.68E-48
HMGXB4	0.57276271	3.18E-48
MCAT	0.50677295	1.92E-36
THOC5	0.52091655	9.39E-39
CBX7	0.58964498	1.10E-51
RBFOX2	0.51696479	4.26E-38
TAB1	0.60853106	8.27E-56
MIEF1	0.70103328	9.74E-81
SAMM50	0.72285271	4.35E-88
SLC25A17	0.68497462	9.64E-76
ST13	0.73359118	5.70E-92
EP300	0.61441263	3.77E-57
L3MBTL2	0.72726653	1.16E-89
ZC3H7B	0.58094617	7.07E-50
PHF5A	0.55283303	2.19E-44
ACO2	0.69232655	5.47E-78
POLR3H	0.52014749	1.26E-38
PMM1	0.59332155	1.82E-52
CERK	0.52924998	3.63E-40
RBM23	0.602178	2.16E-54
PRMT5	0.65699335	9.07E-68
AP4S1	0.50844139	1.04E-36
SOS2	0.56727398	3.85E-47
CDKL1	0.62606764	6.81E-60
PSMC6	0.51118748	3.75E-37
CNIH1	0.58606506	6.19E-51
CGRRF1	0.59366076	1.54E-52
ATP6V1D	0.62828261	1.99E-60
PIGH	0.5854231	8.42E-51
VTI1B	0.59951275	8.33E-54
KIAA0586	0.55692064	3.75E-45
TMED8	0.68020017	2.56E-74
SAMD15	0.586005	6.37E-51
SPTLC2	0.55988867	1.03E-45
ALKBH1	0.58362137	1.99E-50
SNW1	0.61641884	1.29E-57
DHRS7	0.5023506	9.64E-36
PPM1A	0.63323547	1.22E-61
SUSD6	0.57789615	2.96E-49
EIF5	0.58668997	4.58E-51
MTHFD1	0.65959949	1.78E-68
ZC3H14	0.58423931	1.48E-50
PCNX1	0.54689374	2.72E-43
PSMC1	0.63498418	4.51E-62
PPP4R3A	0.51873998	2.16E-38
YY1	0.6403485	2.04E-63
TRIP11	0.55175806	3.47E-44
APEX1	0.52171476	6.90E-39
ARHGAP5	0.54488694	6.30E-43
SRP54	0.71296096	1.14E-84
CHD8	0.58371805	1.90E-50
PCK2	0.59090553	5.94E-52
PRORP	0.60185858	2.54E-54
DCAF11	0.64969464	7.93E-66
PSMA6	0.55072491	5.38E-44
BRMS1L	0.58653183	4.94E-51
TM9SF1	0.64600464	7.26E-65
SEC23A	0.53243691	1.02E-40
GMPR2	0.62664138	4.95E-60
PCIF1	0.55220364	2.86E-44
IFT52	0.52551706	1.58E-39
NDRG3	0.58435548	1.40E-50

CHFR	1.130913	4.511466	3.02E-60	6.51E-59
MS4A14	3.211021	1.985853	3.11E-60	6.70E-59
TMC8	2.536103	4.203779	3.13E-60	6.73E-59
LST1	2.269267	4.159861	3.17E-60	6.81E-59
YPEL4	2.667938	1.283218	3.18E-60	6.81E-59
ETV7	2.706866	3.173281	3.32E-60	7.12E-59
PCGF1	1.048441	3.877676	3.56E-60	7.62E-59
GBP2	1.998127	7.129856	4.56E-60	9.75E-59
TINCR	-2.32588	-0.60104	5.14E-60	1.10E-58
DAO	-3.21734	3.45375	6.30E-60	1.34E-58
GNLY	3.134662	3.562301	6.31E-60	1.34E-58
PRAM1	2.472677	2.121674	6.42E-60	1.36E-58
MAL	-3.2846	6.62849	7.73E-60	1.64E-58
MFSD6L	-3.40364	0.199146	8.00E-60	1.70E-58
PRF1	2.653542	4.596032	8.04E-60	1.70E-58
RNASET2	3.361318	7.870593	8.84E-60	1.87E-58
TNFSF9	3.489936	3.195221	1.24E-59	2.62E-58
GMIP	1.61244	4.280154	1.30E-59	2.73E-58
PLPPR1	-4.18492	2.357447	1.39E-59	2.92E-58
SAT2	-1.13012	5.738965	1.72E-59	3.60E-58
TNFAIP8L	2.270844	2.778139	2.04E-59	4.27E-58
C1QC	2.767945	7.69449	2.11E-59	4.41E-58
BICDL1	-2.01015	5.763205	2.40E-59	5.01E-58
NR3C2	-1.98642	4.637415	2.45E-59	5.12E-58
CTNNAL1	-1.39543	4.878271	2.93E-59	6.10E-58
PDHA1	-1.14167	6.478499	3.71E-59	7.72E-58
HCK	2.060796	4.580033	3.74E-59	7.78E-58
RNF166	1.301424	4.351012	3.80E-59	7.89E-58
CR2	-3.86127	1.228915	5.37E-59	1.11E-57
HVCN1	1.407962	3.377751	5.61E-59	1.16E-57
OTOA	2.923247	-0.25332	7.26E-59	1.50E-57
JPH4	-1.6144	1.295503	7.69E-59	1.59E-57
PLOD3	1.400744	6.895502	8.21E-59	1.69E-57
THEMIS2	2.077725	5.199766	8.95E-59	1.84E-57
THRB	-1.88057	4.258447	1.05E-58	2.17E-57
MFAP3L	-1.69648	5.839133	1.55E-58	3.19E-57
ITPRIPL1	1.800312	1.311594	1.57E-58	3.22E-57
IL10RB	1.03923	5.538343	1.66E-58	3.41E-57
AGMAT	-2.42982	5.667204	2.39E-58	4.89E-57
SELPLG	2.067682	4.89003	2.54E-58	5.20E-57
TMEM131	1.126916	4.426782	2.55E-58	5.22E-57
MCAM	2.084671	8.722793	2.62E-58	5.34E-57
RALGPS1	-1.91034	3.300132	2.70E-58	5.50E-57
DTX2	1.320368	3.752206	3.30E-58	6.71E-57
TRIM9	2.85859	3.998307	3.35E-58	6.81E-57
NXPH4	4.210275	4.496153	3.82E-58	7.74E-57
APOBEC3	2.031804	2.774704	4.01E-58	8.12E-57
SFXN5	-1.24793	4.255546	5.03E-58	1.02E-56
B4GALNT	-3.59984	3.317192	5.44E-58	1.10E-56
FMO4	-1.36559	3.960422	5.79E-58	1.17E-56
MEIOC	-1.6657	-0.54033	5.98E-58	1.21E-56
PROM2	-3.54015	5.079794	6.50E-58	1.31E-56
GGTA1	1.930611	4.415877	6.59E-58	1.33E-56
GATM	-2.16277	8.845605	6.97E-58	1.40E-56
VSIG1	3.964402	1.942588	7.03E-58	1.41E-56
ASPDH	-2.72408	2.459834	9.31E-58	1.87E-56
ITGB6	-2.58485	5.221735	9.93E-58	1.99E-56
GLDC	-2.34179	4.541954	1.06E-57	2.12E-56
GNAI1	-1.21233	5.714869	1.13E-57	2.25E-56
FAM163A	3.219877	0.57067	1.22E-57	2.43E-56
SCN2A	-3.06647	1.459607	1.26E-57	2.50E-56
CA10	-4.7934	1.325615	1.38E-57	2.73E-56
NHSL1	-1.24179	4.585506	1.50E-57	2.97E-56
TMC1	-2.10128	-1.06557	1.51E-57	2.98E-56
RGS1	3.127617	6.883792	1.51E-57	2.99E-56
APEH	-1.2127	6.300883	1.71E-57	3.38E-56
NPNT	-2.0041	6.248691	1.86E-57	3.68E-56
MAN1C1	-2.1815	4.468052	1.94E-57	3.81E-56
NKG7	3.501722	4.809629	2.27E-57	4.46E-56
RIN3	1.430101	5.230551	2.60E-57	5.11E-56
KCNE3	2.142896	6.376807	2.78E-57	5.45E-56
OLFML2B	2.696226	5.97478	2.78E-57	5.45E-56
SMIM5	-3.13219	3.134302	2.82E-57	5.52E-56
LRBA	-1.17734	6.656985	2.89E-57	5.65E-56
LURAP1	-1.77135	1.382139	3.21E-57	6.26E-56
ECI2	-1.05132	6.252089	3.31E-57	6.46E-56
CD37	2.156958	4.77378	3.85E-57	7.48E-56
DEPP1	2.275566	8.453474	3.97E-57	7.70E-56
BPI	-2.4067	-0.41484	4.03E-57	7.81E-56
APOBEC3	2.117072	5.974545	4.24E-57	8.22E-56
CD72	2.726647	2.857704	6.35E-57	1.23E-55
EPB41L4E	-3.36996	2.569479	6.66E-57	1.29E-55
CSRNP3	-1.59844	2.716829	7.20E-57	1.39E-55
DLGAP3	-1.58895	-0.42476	7.61E-57	1.47E-55
CYS1	-2.08308	6.886623	1.00E-56	1.93E-55
CORO1A	2.44614	6.006775	1.05E-56	2.03E-55
GCDH	-1.0401	4.245302	1.09E-56	2.09E-55
DEF6	2.28636	3.726963	1.12E-56	2.15E-55
PAK6	-3.34328	-1.96325	1.18E-56	2.27E-55
SPARC	1.840505	11.28622	1.24E-56	2.37E-55
WLS	-1.44896	6.428335	1.28E-56	2.45E-55
GABRA2	-4.94723	1.992552	1.28E-56	2.45E-55
M1AP	-1.73819	0.089379	1.36E-56	2.59E-55
MYO5C	-1.27305	4.317513	1.41E-56	2.69E-55
ILDR1	-1.99727	3.428466	1.50E-56	2.86E-55
STAT4	1.893789	2.482388	1.58E-56	3.01E-55
MYO1G	2.659078	4.083404	1.62E-56	3.08E-55
ADH6	-2.60181	3.536893	1.64E-56	3.11E-55
CRACR2A	2.181832	2.163365	1.67E-56	3.15E-55
BMP1	1.875912	5.73753	1.96E-56	3.70E-55
NME8	2.796321	-0.5889	2.05E-56	3.87E-55
DNASE1L	-2.16482	3.360985	2.28E-56	4.30E-55
KIF21B	2.148958	3.156793	2.50E-56	4.72E-55
TMEM86A	-1.39696	4.056988	2.62E-56	4.93E-55
SOWAHA	-3.06728	1.948569	2.66E-56	5.00E-55
TCAF2	1.680059	3.554179	2.69E-56	5.05E-55
VAV3	-1.38138	6.490475	3.45E-56	6.47E-55
MOB3A	1.101091	6.254597	3.66E-56	6.85E-55
DLL4	2.439345	6.465086	3.80E-56	7.11E-55
TBC1D4	-1.44288	6.096277	4.27E-56	7.97E-55
BID	1.11756	4.633645	4.28E-56	7.99E-55
HK3	2.60397	2.43605	4.34E-56	8.08E-55
BBC3	1.721176	3.34722	4.34E-56	8.08E-55
GALM	-1.16779	5.892274	4.48E-56	8.34E-55
GZMH	2.999961	2.755618	4.55E-56	8.45E-55
PLEKHG2	1.757465	5.691604	4.64E-56	8.62E-55
IL16	1.875928	4.577455	4.76E-56	8.83E-55
CHST11	1.794505	5.410512	5.04E-56	9.34E-55
COL9A2	-2.19715	3.126307	5.91E-56	1.09E-54
WNT7B	-3.86206	1.78667	6.00E-56	1.11E-54
CSNK1E	1.121864	6.670895	6.03E-56	1.11E-54
GNG7	-1.39279	4.069554	6.14E-56	1.13E-54
PLEKHB1	-2.30578	3.28583	6.45E-56	1.19E-54
PLA2G7	3.683141	3.099995	6.76E-56	1.24E-54
RASA3	1.413793	5.285633	8.01E-56	1.47E-54
MAP4K4	1.217562	7.212522	1.02E-55	1.87E-54
CHSY3	2.22365	2.434922	1.08E-55	1.98E-54
TSPYL5	-1.60942	4.502513	1.08E-55	1.98E-54
CCNE2	1.577943	0.82817	1.37E-55	2.51E-54
PATL2	2.85536	1.562173	1.46E-55	2.66E-54
SLA2	2.926495	2.385187	1.82E-55	3.33E-54
LMBR1L	1.408349	4.939373	1.91E-55	3.49E-54
RAB37	1.916549	3.66572	2.21E-55	4.02E-54
IFNAR2	1.119073	4.616068	2.27E-55	4.13E-54
MAPK4	-4.0615	1.821734	2.46E-55	4.48E-54
EFNA3	2.41262	2.183852	2.76E-55	5.02E-54
PRDX4	1.436387	6.55301	3.26E-55	5.91E-54
VWA8	-1.12462	5.177812	3.49E-55	6.32E-54
LMX1B	-4.09441	0.8606	3.55E-55	6.42E-54
PRRG1	-1.25781	4.520169	3.64E-55	6.58E-54
MYEOV	7.109116	4.631243	3.66E-55	6.60E-54
WAS	2.192434	4.119098	3.83E-55	6.90E-54
HSD11B2	-2.76726	7.098674	4.56E-55	8.21E-54
CTDSPL	-1.18686	6.163753	4.66E-55	8.39E-54
PRKCQ	-1.68	3.428056	4.94E-55	8.88E-54
ITGAL	2.534712	5.356932	5.00E-55	8.99E-54
TBC1D24	-1.34225	4.394347	5.10E-55	9.15E-54
PTP4A3	1.894963	5.779609	5.57E-55	9.98E-54
INTS6L	1.783326	3.616375	5.71E-55	1.02E-53
MAP3K15	-2.85546	0.978148	7.36E-55	1.32E-53
CLEC2B	2.310417	4.189237	1.09E-54	1.94E-53
ARHGAP3	2.040784	5.16168	1.23E-54	2.18E-53
RASGRP4	1.757763	1.809133	1.26E-54	2.23E-53
CTSH	-1.26518	7.450488	1.31E-54	2.33E-53
CNNM2	-1.03211	4.608688	1.53E-54	2.71E-53
FMNL3	1.286827	6.530764	1.60E-54	2.83E-53
SYP	-1.55427	1.682034	1.65E-54	2.92E-53
SCPEP1	-1.17483	6.171669	1.69E-54	2.99E-53
MCM5	1.071386	5.594563	1.83E-54	3.23E-53
YEATS2	1.10157	5.816538	1.97E-54	3.47E-53
B2M	1.349832	11.89886	1.98E-54	3.48E-53
SPACA9	-1.13077	3.107335	2.23E-54	3.92E-53
CAMKK1	1.475482	3.950403	2.30E-54	4.04E-53
BAZ1A	1.205285	5.105746	2.37E-54	4.16E-53
DOK1	1.261371	3.816466	2.40E-54	4.21E-53
MS4A6A	2.265196	6.266274	2.56E-54	4.49E-53
RAB7B	2.072914	2.565477	2.64E-54	4.62E-53
JAK3	2.446103	4.928681	3.16E-54	5.53E-53
ZAP70	2.910963	3.054206	3.56E-54	6.20E-53
ZMYND1	1.739935	2.494796	3.60E-54	6.26E-53
SH3BGR	-1.14343	2.25941	3.69E-54	6.43E-53
COL4A3	-1.95816	4.870122	3.99E-54	6.94E-53
LILRA6	2.529538	1.13384	4.53E-54	7.86E-53
C1QA	2.626697	7.758024	4.64E-54	8.05E-53
MAB21L4	-3.80103	1.478814	5.24E-54	9.08E-53
NEK6	1.998928	6.931516	5.43E-54	9.40E-53
CYTH4	1.975643	4.619551	6.12E-54	1.06E-52
MRPS6	-1.58825	5.889553	6.45E-54	1.11E-52
UNC13D	2.188358	3.834009	7.12E-54	1.23E-52
CAMK2A	-2.74714	-0.68622	7.47E-54	1.29E-52
TREML1	2.935281	-0.63503	7.64E-54	1.31E-52
ADAMTS1	-4.29178	-1.81412	7.99E-54	1.37E-52
CD276	1.214322	6.165314	9.87E-54	1.69E-52
CAVIN3	2.314351	5.611711	1.10E-53	1.88E-52
HSPB8	2.183793	7.831175	1.35E-53	2.30E-52
PLIN2	2.937871	9.885769	1.38E-53	2.36E-52
FASLG	3.495059	1.436564	1.41E-53	2.41E-52
BARX2	2.650093	5.468916	1.46E-53	2.49E-52
GRIK5	-3.03765	2.1564	1.46E-53	2.49E-52
SIRPB2	2.147213	2.596513	1.49E-53	2.53E-52

ADNP	0.53513942	3.44E-41
CSTF1	0.68421091	1.64E-75
NELFCD	0.52931402	3.54E-40
PRPF6	0.52319678	3.89E-39
PRELID3B	0.50723734	1.62E-36
DIDO1	0.50994625	5.95E-37
GID8	0.55949919	1.22E-45
CSNK2A1	0.5343137	4.80E-41
CDS2	0.58569877	7.38E-51
SEC23B	0.66623769	2.63E-70
CRNKL1	0.64041619	1.96E-63
KIF3B	0.62192862	6.61E-59
IDH3B	0.53930098	6.34E-42
MAPRE1	0.58104541	6.75E-50
JAG1	0.50223051	1.01E-35
TTI1	0.55356533	1.60E-44
RPRD1B	0.59820896	1.60E-53
ADNP2	0.57484278	1.22E-48
USP14	0.55955125	1.19E-45
VAPA	0.55490028	8.99E-45
LPIN2	0.51525063	8.15E-38
RNMT	0.54863097	1.31E-43
RIOK3	0.66466203	7.22E-70
CSTF2	0.63546987	3.42E-62
PSMD10	0.61303407	7.82E-57
ATG4A	0.59921795	9.66E-54
STS	0.52890012	4.16E-40
TBL1X	0.66771134	1.01E-70
PGRMC1	0.73666633	4.06E-93
POLA1	0.5474575	2.15E-43
MID1	0.50548906	3.08E-36
NKAP	0.50086104	1.65E-35
NXT2	0.57753691	3.50E-49
AMMECR1	0.50774136	1.34E-36
XIAP	0.59174418	3.94E-52
STAG2	0.62767855	2.78E-60
ATP11C	0.60140388	3.20E-54
SMARCA1	0.56973555	1.27E-47
ASB9	0.62363782	2.60E-59
RBBP7	0.54722629	2.36E-43
PGK1	0.61682449	1.04E-57
MAGT1	0.65603772	1.64E-67
SMS	0.51204955	2.72E-37
CD99L2	0.62622238	6.25E-60
RP2	0.61217361	1.23E-56
JADE3	0.5652192	9.68E-47
HTATSF1	0.66030444	1.14E-68
ARMCX3	0.63822065	7.01E-63
NDFIP2	0.66566664	3.79E-70
FNDC3A	0.59032051	7.90E-52
CDADC1	0.71487529	2.55E-85
CAB39L	0.50877923	9.17E-37
STK24	0.60846342	8.57E-56
DNAJC3	0.68791168	1.24E-76
ARHGEF7	0.52610452	1.25E-39
PARP4	0.54476803	6.62E-43
MRPS31	0.57468577	1.31E-48
SLC25A15	0.50335507	6.70E-36
KPNA3	0.55151969	3.84E-44
FLT1	0.58152283	5.39E-50
VWA8	0.65908096	2.47E-68
KATNAL1	0.64417844	2.15E-64
INTS6	0.5728866	3.01E-48
TSC22D1	0.57270282	3.27E-48
CLN5	0.63946622	3.40E-63
MAPK3	0.61986825	2.03E-58
PHKB	0.63840277	6.31E-63
LONP2	0.58466317	1.21E-50
N4BP1	0.55629415	4.92E-45
ARL2BP	0.64724055	3.47E-65
CTCF	0.7036851	1.35E-81
POLR2C	0.73145656	3.49E-91
ZNF821	0.59043987	7.45E-52
CYB5B	0.59729843	2.53E-53
TANGO6	0.6909639	1.44E-77
COG4	0.50754925	1.44E-36
SLC7A6OS	0.57384236	1.94E-48
MON1B	0.55966245	1.13E-45
MLYCD	0.54174349	2.32E-42
HSDL1	0.61939381	2.62E-58
USP10	0.70221164	4.06E-81
STUB1	0.53572506	2.72E-41
NUBP1	0.71677044	5.72E-86
CRYM	0.57246262	3.65E-48
EEF2K	0.5539283	1.37E-44
GSPT1	0.53004527	2.65E-40
ZNF174	0.66983023	2.56E-71
CLUAP1	0.61744149	7.49E-58
UBFD1	0.51544871	7.57E-38
EARS2	0.64752837	2.92E-65
GGA2	0.50731552	1.57E-36
CPPED1	0.65149614	2.66E-66
DNAJA3	0.59682532	3.20E-53
BFAR	0.50727251	1.60E-36
SALL1	0.64933086	9.88E-66
RBL2	0.54337591	1.18E-42
MAZ	0.51024689	5.32E-37
CDIPT	0.53884199	7.65E-42
CCP110	0.52653189	1.06E-39
VPS35L	0.73594269	7.59E-93
RNF40	0.66956712	3.04E-71
IOCH	0.5296117	3.14E-40
HERC1	0.51159042	3.22E-37
TRIP4	0.80556972	6.19E-124
MTFMT	0.63225264	2.13E-61
RAB11A	0.64366948	2.90E-64
RPAP1	0.56085182	6.72E-46
EHD4	0.53705458	1.58E-41
TMEM87A	0.57505294	1.11E-48
ZNF106	0.58331927	2.30E-50
TJP1	0.58012976	1.04E-49
EIF3J	0.69614985	3.49E-79
SPG11	0.61521407	2.46E-57
VPS18	0.54012726	4.52E-42
SLC30A4	0.67884001	6.43E-74
BLOC1S6	0.69030803	2.30E-77
MYEF2	0.51843493	2.43E-38
SGK3	0.52223392	5.65E-39
BRF2	0.51257358	2.23E-37
TRIM35	0.54830629	1.50E-43
ZFAND1	0.539417	6.04E-42
CA2	0.60921639	5.79E-56
INTS9	0.54468052	6.86E-43
CPQ	0.58385612	1.78E-50
DECR1	0.50442296	4.54E-36
UBE2W	0.5393841	6.13E-42
POP1	0.5597555	1.09E-45
STK3	0.57056005	8.70E-48
GDAP1	0.54400908	9.08E-43
RAB2A	0.52353878	3.41E-39
EMC2	0.63377823	8.98E-62
NDRG1	0.54737208	2.22E-43
ARMC1	0.60056296	4.90E-54
SPAG1	0.56653642	5.37E-47
MTMR9	0.5363561	2.10E-41
LEPROTL1	0.67927099	4.80E-74
DCTN6	0.6906795	1.77E-77
R3HCC1	0.58153297	5.36E-50
TNFRSF10A	0.54339523	1.17E-42
UBXN8	0.59955689	8.14E-54
PPP2CB	0.6442187	2.10E-64
ARHGEF10	0.50026019	2.05E-35
KCTD9	0.60245926	1.88E-54
ASAH1	0.71785355	2.42E-86
BNIP3L	0.62904313	1.30E-60
NUCB1	0.5845714	1.27E-50
HNRNPL	0.54308939	1.33E-42
CLPTM1	0.5768395	4.84E-49
FCGRT	0.54222551	1.90E-42
ERCC2	0.60889848	6.83E-56
VRK3	0.69979716	2.43E-80
FAM32A	0.54287383	1.45E-42
ILVBL	0.57567814	8.30E-49
POP4	0.51432362	1.16E-37
URI1	0.63310149	1.32E-61
ANKRD27	0.62703272	3.99E-60
PIAS4	0.55997029	9.90E-46
HNRNPUL1	0.52874969	4.42E-40
DMAC2	0.51693303	4.31E-38
NAPA	0.63793915	8.25E-63
GRWD1	0.57374034	2.03E-48
PLA2G4C	0.51890853	2.03E-38
RAB3D	0.56750127	3.48E-47
PPP2R1A	0.54759584	2.02E-43
GCDH	0.50302511	7.55E-36
ARMC6	0.50313745	7.25E-36
ZNF14	0.55403339	1.31E-44
GSK3A	0.52960953	3.15E-40
ZNF574	0.51575107	6.75E-38
POLR1F	0.54781042	1.85E-43
HBP1	0.61126293	1.99E-56
CBLL1	0.54428898	8.08E-43
MTPN	0.51400876	1.30E-37
MPP6	0.56734388	3.73E-47
ZC3HAV1	0.54088814	3.30E-42
TTC26	0.55703683	3.57E-45
OGDH	0.60114305	3.66E-54
TFEC	0.52602742	1.29E-39
H2AZ2	0.63755655	1.03E-62
LMBR1	0.53077115	1.98E-40
TSPAN12	0.61982099	2.08E-58
HIBADH	0.59582676	5.26E-53
TAX1BP1	0.62943269	1.05E-60
GRB10	0.5196306	1.54E-38
TAF6	0.50205817	1.07E-35
WASL	0.64330869	3.59E-64
TMEM106B	0.52273589	4.65E-39
ANKMY2	0.54133979	2.74E-42
CHCHD3	0.52288763	4.38E-39
TMEM176B	0.56936372	1.50E-47

DNAH11	4.632112	5.245988	1.49E-53	2.54E-52
CHN2	-1.56741	3.168922	1.50E-53	2.55E-52
INO80E	1.235094	5.500702	1.51E-53	2.57E-52
GPAT3	-2.44622	4.994545	1.65E-53	2.80E-52
GDF3	-2.14507	-0.22507	1.80E-53	3.04E-52
NDC80	2.064215	2.085682	1.96E-53	3.31E-52
BHLHB9	-1.06115	2.83366	1.97E-53	3.32E-52
PLXND1	1.612367	7.964114	1.98E-53	3.34E-52
EVPL	-1.54998	4.279356	2.26E-53	3.80E-52
E2F2	2.372338	1.010006	2.34E-53	3.94E-52
C10orf82	-2.78073	-1.42335	3.00E-53	5.04E-52
TACR3	-3.88051	-2.38604	3.01E-53	5.06E-52
EFHD2	1.4529	6.10762	3.09E-53	5.18E-52
S1PR5	2.411533	2.016739	3.21E-53	5.38E-52
GAPDH	1.476317	11.97639	3.35E-53	5.60E-52
SLC30A2	-3.37076	2.626999	3.59E-53	6.01E-52
SOX30	-1.96995	-1.37551	3.86E-53	6.45E-52
SLC7A13	-4.95166	1.426572	4.68E-53	7.80E-52
CRB2	-2.88248	2.408251	5.26E-53	8.76E-52
ITGA5	1.976021	7.664254	5.98E-53	9.95E-52
GZMA	3.146258	4.118832	6.26E-53	1.04E-51
DNAJC5B	3.745557	0.477464	6.27E-53	1.04E-51
AJM1	2.087845	2.869904	7.20E-53	1.19E-51
P11-872D1	3.034044	-1.67985	7.96E-53	1.32E-51
CORO7	1.560353	3.758427	8.50E-53	1.40E-51
VWA7	-1.56824	2.862526	8.64E-53	1.42E-51
CENPU	1.85315	2.634901	8.67E-53	1.43E-51
LTB4R2	2.207222	1.207153	8.78E-53	1.45E-51
DDIT4	2.180079	8.076969	9.30E-53	1.53E-51
CD244	2.323238	1.470186	9.39E-53	1.54E-51
TMEM30E	-2.84672	4.168933	9.95E-53	1.63E-51
GOLGA7E	3.863931	2.260098	1.03E-52	1.69E-51
SAMD3	2.347895	1.402636	1.26E-52	2.06E-51
NCKAP1L	2.329354	5.648692	1.28E-52	2.09E-51
GINS2	1.919484	2.552641	1.33E-52	2.16E-51
PREX1	1.370138	6.556292	1.33E-52	2.16E-51
MXD3	2.572252	2.217992	1.39E-52	2.26E-51
ADAMTS1	-1.95449	3.590703	1.49E-52	2.42E-51
ESRP2	-1.51599	3.937415	1.53E-52	2.47E-51
FKBP10	2.808914	7.653537	1.58E-52	2.55E-51
BTBD16	3.578806	1.759354	1.59E-52	2.58E-51
APBB1IP	2.443827	5.780947	1.64E-52	2.65E-51
HLA-DPB1	2.081926	8.876311	1.89E-52	3.04E-51
PSTPIP1	2.796333	2.894511	1.97E-52	3.17E-51
CLEC7A	2.366612	3.845876	2.12E-52	3.41E-51
ASF1B	2.176053	2.732204	2.20E-52	3.53E-51
CCDC127	1.063536	5.397623	2.30E-52	3.69E-51
IL10RA	2.247893	5.629922	2.36E-52	3.79E-51
LDLRAD3	1.493049	4.382787	2.40E-52	3.84E-51
DARS1	1.072245	7.580608	2.56E-52	4.09E-51
FHOD1	1.273865	5.148078	2.71E-52	4.33E-51
SLFN11	1.711085	5.775825	2.89E-52	4.61E-51
THSD4	-1.93653	4.832623	3.02E-52	4.80E-51
MTCL1	2.26748	5.173646	3.05E-52	4.85E-51
CDT1	2.297033	2.477271	3.14E-52	4.99E-51
SLCO1C1	2.510008	0.831628	3.52E-52	5.59E-51
HMX2	-5.31222	-2.34575	3.59E-52	5.70E-51
RFX8	3.437186	0.527161	4.19E-52	6.64E-51
HPCAL1	1.850404	7.745826	4.78E-52	7.57E-51
NTN4	-1.64151	7.204419	4.79E-52	7.58E-51
CCL5	3.241801	6.126963	4.85E-52	7.67E-51
TJP3	-2.76099	2.314432	5.07E-52	8.00E-51
MAN1A1	-1.39624	7.041642	5.92E-52	9.33E-51
PNPLA1	-2.4547	0.407805	5.96E-52	9.38E-51
NCF4	2.030123	3.49674	6.54E-52	1.03E-50
MCCD1	-4.23465	1.821409	6.67E-52	1.05E-50
PLEKHO1	1.798121	5.646823	6.82E-52	1.07E-50
GM2A	1.374994	6.873608	7.67E-52	1.20E-50
GAL3ST1	2.942634	7.196295	1.15E-51	1.80E-50
SLC1A3	2.748739	4.444643	1.21E-51	1.89E-50
RXFP4	-2.37818	-3.24754	1.26E-51	1.97E-50
CD33	1.958122	2.582252	1.40E-51	2.17E-50
ATP6V1H	-1.1453	5.837611	1.42E-51	2.21E-50
P11-644F5.	2.03009	1.847798	1.47E-51	2.28E-50
TBC1D10C	2.552914	3.393978	1.48E-51	2.29E-50
IGSF11	-2.87376	0.919867	1.53E-51	2.38E-50
CTSW	3.039854	3.793518	1.56E-51	2.42E-50
ERO1A	1.492823	7.009866	1.57E-51	2.43E-50
CLEC4A	1.708207	1.972198	2.07E-51	3.20E-50
APLN	2.4796	6.615791	2.42E-51	3.74E-50
G6PC1	-3.48563	4.370892	2.55E-51	3.94E-50
SLC5A3	-1.90786	7.257199	2.67E-51	4.11E-50
CD200	1.684087	5.301629	2.68E-51	4.13E-50
NMUR2	-3.35671	-2.53586	2.76E-51	4.25E-50
HMGN5	-1.08556	2.905414	2.96E-51	4.55E-50
HACD3	-1.37962	6.750815	3.31E-51	5.09E-50
NLRC4	1.803422	2.142177	3.52E-51	5.39E-50
ACKR3	2.294799	6.439436	3.63E-51	5.55E-50
SLC24A4	1.744341	0.618995	3.74E-51	5.72E-50
AK7	-1.51282	2.43226	3.83E-51	5.86E-50
CLMN	-1.37287	6.121127	3.84E-51	5.86E-50
EPHA1	-1.54308	2.456889	3.90E-51	5.95E-50
RIMBP2	-2.92094	0.624028	3.96E-51	6.05E-50
DTL	2.157686	2.594102	4.06E-51	6.18E-50
CDKL2	-1.58188	2.789364	4.41E-51	6.71E-50
CD84	2.803721	4.957203	5.33E-51	8.11E-50
CKLF	1.108622	2.666484	5.78E-51	8.78E-50
LPAR5	2.175977	2.861888	6.14E-51	9.31E-50
CYRIA	1.576511	4.279638	6.56E-51	9.95E-50
LIG1	1.046723	4.737606	6.62E-51	1.00E-49
DPP6	-3.3613	1.880282	7.11E-51	1.08E-49
PYHIN1	2.98359	2.100096	7.63E-51	1.15E-49
FCRL6	2.513239	1.435772	8.33E-51	1.26E-49
L2HGDH	-1.39984	3.8716	8.83E-51	1.33E-49
LSP1	2.268921	5.638584	8.92E-51	1.34E-49
HLA-E	1.075542	10.41271	9.89E-51	1.49E-49
FAM111B	2.224755	2.060101	1.07E-50	1.60E-49
PCLAF	2.242036	2.398818	1.15E-50	1.73E-49
C11orf21	2.653488	1.144664	1.17E-50	1.75E-49
LHFPL2	2.024312	5.844094	1.27E-50	1.91E-49
EMILIN2	1.693149	4.434408	1.32E-50	1.98E-49
ANKRD2	-3.21956	1.699892	1.49E-50	2.23E-49
CDKN2C	1.563006	4.57883	1.98E-50	2.95E-49
PTCRA	2.883269	-1.87835	2.03E-50	3.02E-49
CAPN12	3.163893	4.778256	2.06E-50	3.06E-49
TTPA	-3.29078	-1.58406	2.13E-50	3.17E-49
HS3ST2	4.90076	2.604635	2.46E-50	3.66E-49
JAKMIP1	3.565927	0.89305	3.06E-50	4.54E-49
CST7	3.137952	3.954512	3.10E-50	4.59E-49
CALHM6	2.977985	3.689181	3.72E-50	5.50E-49
PKMYT1	2.629776	1.271553	4.44E-50	6.56E-49
PTGR1	-1.30134	6.509076	4.73E-50	6.98E-49
LPXN	1.330243	4.495274	4.83E-50	7.12E-49
FCGR2A	1.891494	5.717093	5.44E-50	8.00E-49
TWSG1	-1.15231	6.100664	5.57E-50	8.20E-49
NINL	-1.2049	4.333379	5.62E-50	8.26E-49
ARRDC5	3.715536	-0.94769	6.27E-50	9.19E-49
BATF	3.031654	1.77211	6.27E-50	9.19E-49
CCM2	1.133453	5.662095	6.61E-50	9.68E-49
FRK	-1.27059	5.494587	7.07E-50	1.03E-48
CAMK1D	1.294705	4.86481	7.33E-50	1.07E-48
HAPLN3	2.403172	3.317665	7.41E-50	1.08E-48
CPA1	-2.85676	-2.4686	7.68E-50	1.12E-48
GLOD5	-2.3867	0.07789	7.70E-50	1.12E-48
TBC1D30	-1.08967	2.546872	7.99E-50	1.16E-48
SUCLA2	-1.02076	5.587741	8.22E-50	1.20E-48
CAPN15	1.318386	5.65554	9.17E-50	1.33E-48
DCLK3	3.296945	-0.01894	9.68E-50	1.41E-48
CENPK	1.952369	1.451374	1.09E-49	1.58E-48
MYMK	-3.1759	-2.90922	1.12E-49	1.62E-48
TTYH3	1.950802	8.006955	1.23E-49	1.77E-48
GIT2	1.31562	5.975085	1.32E-49	1.91E-48
LDHD	-2.24158	4.353667	1.33E-49	1.92E-48
DLGAP2	-2.6258	-0.67044	1.41E-49	2.04E-48
MATK	2.269178	2.453705	1.42E-49	2.05E-48
MILR1	2.003911	2.281575	1.46E-49	2.10E-48
PLCB2	2.142923	4.556337	1.48E-49	2.12E-48
RALGDS	1.135949	5.89563	1.50E-49	2.15E-48
PRTG	-1.9979	1.545133	1.54E-49	2.21E-48
APOL5	3.830779	-2.0086	1.63E-49	2.34E-48
FBXO2	-2.49768	3.015447	1.71E-49	2.45E-48
ALDH4A1	-2.04956	6.440578	1.74E-49	2.49E-48
LAD1	-2.67735	4.368717	1.87E-49	2.68E-48
CD300C	2.303148	1.597671	1.90E-49	2.73E-48
SH2B3	1.30508	6.516217	1.92E-49	2.74E-48
CD4	1.804991	6.588298	1.95E-49	2.78E-48
FAM181B	-2.22614	-0.78404	1.96E-49	2.79E-48
APOBEC3I	1.118901	2.998172	2.00E-49	2.85E-48
PPARA	-1.04219	5.980424	2.28E-49	3.24E-48
GLRX5	-1.08098	5.062644	2.32E-49	3.30E-48
HMGCR	-1.01029	5.251873	2.49E-49	3.53E-48
PPP1R36	-2.43952	0.355648	2.54E-49	3.59E-48
SLC6A3	6.082806	7.357097	2.59E-49	3.66E-48
CCR5	2.90678	4.257718	2.63E-49	3.72E-48
BTN3A3	1.470252	5.731592	3.20E-49	4.52E-48
BTN2A2	1.196882	4.758739	3.65E-49	5.15E-48
SLC15A3	1.664948	5.41102	3.66E-49	5.16E-48
EBI3	2.313078	2.513645	3.91E-49	5.50E-48
MAPK10	-1.07663	4.219279	4.00E-49	5.63E-48
MYDGF	1.255804	6.412969	4.21E-49	5.91E-48
CBLN2	-3.07204	-0.49324	4.24E-49	5.96E-48
HIBCH	-1.24265	5.523631	4.31E-49	6.04E-48
HLA-C	1.352388	10.8035	4.58E-49	6.41E-48
SNX20	2.465925	3.284595	4.60E-49	6.44E-48
OVOL1	-2.3274	2.012357	4.79E-49	6.70E-48
GBP5	3.522294	5.059914	4.80E-49	6.71E-48
GPBAR1	-1.69502	0.979272	4.94E-49	6.90E-48
PRSS53	3.528112	1.656616	5.68E-49	7.93E-48
SYN2	-2.30056	0.253055	5.77E-49	8.04E-48
IL21R	2.61953	2.455181	5.96E-49	8.30E-48
MCCC1	-1.06163	5.286647	6.66E-49	9.27E-48
ARHGAP4.	1.507885	5.45423	6.68E-49	9.29E-48
MISP3	-1.66949	2.982715	6.82E-49	9.47E-48
EOMES	3.520814	2.749612	6.88E-49	9.55E-48
ARHGAP1.	1.634689	3.904466	7.13E-49	9.89E-48
TRIB3	3.135188	5.84445	7.35E-49	1.02E-47
SCARF1	1.547848	4.976634	7.91E-49	1.10E-47
CD2	3.03281	4.480447	8.45E-49	1.17E-47
BRCA2	1.734905	2.255727	8.71E-49	1.20E-47
RUFY4	4.888454	0.100869	8.77E-49	1.21E-47

BLVRA	0.54372874	1.02E-42
EIF4H	0.64263477	5.34E-64
SLC1A1	0.69667558	2.38E-79
FKTN	0.59110346	5.39E-52
SPIN1	0.64175379	8.95E-64
NMRK1	0.55900148	1.51E-45
TMEM245	0.58319851	2.43E-50
PRUNE2	0.68522375	8.11E-76
MEGF9	0.63177283	2.80E-61
TRIM14	0.53071635	2.03E-40
PTGR1	0.57479805	1.25E-48
CDC37L1	0.5774914	3.57E-49
TBC1D13	0.65362121	7.27E-67
DOCK8	0.56391259	1.73E-46
KANK1	0.51030482	5.21E-37
FUBP3	0.66264547	2.61E-69
CREB3	0.59440118	1.07E-52
RGP1	0.63155835	3.16E-61
MPDZ	0.52767506	6.75E-40
DDX58	0.64855788	1.57E-65
GLIS3	0.52381101	3.06E-39
BAG1	0.54357524	1.09E-42
RAPGEF1	0.59032459	7.88E-52
SETX	0.56734697	3.73E-47
UBE2R2	0.62270143	4.34E-59
ABHD17B	0.68957185	3.87E-77
EXOSC3	0.52598529	1.31E-39
PHYH	0.67199789	6.18E-72
RAB11FIP2	0.63281241	1.55E-61
ERLIN1	0.6143722	3.85E-57
EIF3A	0.63868998	5.34E-63
CUBN	0.64298332	4.35E-64
TRDMT1	0.56702566	4.31E-47
DDX50	0.59617228	4.43E-53
MAPK8	0.64834511	1.79E-65
SEC23IP	0.61505675	2.68E-57
ATE1	0.72983973	1.36E-90
PLEKHA1	0.56093579	6.47E-46
MICU1	0.75219139	3.66E-99
CCSER2	0.5770353	4.42E-49
BMPR1A	0.54370548	1.03E-42
MINPP1	0.62084234	1.19E-58
LIPA	0.53353324	6.57E-41
TNKS2	0.54717485	2.42E-43
GBF1	0.55774729	2.62E-45
SUFU	0.60643676	2.45E-55
ACBD5	0.73202076	2.16E-91
GTPBP4	0.57370604	2.06E-48
BCCIP	0.61619439	1.46E-57
MTPAP	0.639937	2.59E-63
STN1	0.5298739	2.83E-40
XPNPEP1	0.6007414	4.48E-54
SMC3	0.64661287	5.05E-65
SHOC2	0.66298874	2.10E-69
TFAM	0.63233304	2.04E-61
CUL2	0.59415007	1.21E-52
CCNY	0.62593522	7.32E-60
PBLD	0.63120216	3.86E-61
TSPAN14	0.50579437	2.75E-36
NUFIP2	0.51759455	3.35E-38
FBXL20	0.52743178	7.43E-40
UBTF	0.52560522	1.52E-39
PSMD3	0.58934981	1.27E-51
CASC3	0.6081604	1.00E-55
ASPA	0.51567367	6.95E-38
RAD51C	0.52491631	1.99E-39
TRIM37	0.57631359	6.18E-49
DHX40	0.68916818	5.14E-77
TUBD1	0.59589607	5.08E-53
KPNB1	0.59543116	6.40E-53
GOSR2	0.59002353	9.13E-52
PNPO	0.65751149	6.57E-68
RPS6KB1	0.54075352	3.49E-42
CBX1	0.6442216	2.09E-64
INTS2	0.58516273	9.54E-51
MED13	0.57648756	5.70E-49
RNF167	0.64796715	2.24E-65
SLC25A11	0.59562743	5.81E-53
NUP88	0.57418765	1.65E-48
C1QBP	0.56743933	3.57E-47
BLMH	0.70912156	2.21E-83
GOSR1	0.74811681	1.56E-97
CCDC47	0.74057588	1.34E-94
DRG2	0.53941025	6.06E-42
FTSJ3	0.59520629	7.15E-53
AKAP10	0.61099226	2.29E-56
UTP6	0.54754071	2.07E-43
C17orf75	0.60884391	7.03E-56
PSMD11	0.62190825	6.69E-59
PEX12	0.64239699	6.14E-64
RAB5C	0.6940942	1.54E-78
NAGLU	0.53610048	2.33E-41
MLX	0.67071941	1.43E-71
EZH1	0.50115565	1.48E-35
VAT1	0.65137757	2.86E-66
LRRC59	0.54808513	1.65E-43
HDAC5	0.50882172	9.02E-37
SMURF2	0.52455554	2.29E-39
DUSP3	0.73036534	8.74E-91
EFTUD2	0.53953329	5.76E-42
HLF	0.58265968	3.14E-50
PRKAR1A	0.69391283	1.76E-78
YWHAE	0.68935718	4.50E-77
SLC9A3R1	0.65551338	2.27E-67
TNFAIP1	0.62114689	1.01E-58
SUPT6H	0.65090971	3.80E-66
TMEM33	0.56023289	8.82E-46
SLAIN2	0.64674567	4.66E-65
OCIAD1	0.67161157	7.97E-72
DCUN1D4	0.50497168	3.72E-36
USP46	0.63800632	7.94E-63
LAMTOR3	0.62982227	8.40E-61
NFKB1	0.67931832	4.65E-74
MANBA	0.52255839	4.98E-39
UBE2D3	0.68496087	9.74E-76
MAPK10	0.51417097	1.23E-37
ZNF330	0.62490556	1.29E-59
GAB1	0.60594924	3.14E-55
GRPEL1	0.62490369	1.29E-59
FRG1	0.51511323	8.59E-38
CLCN3	0.68662718	3.06E-76
DHX15	0.6494087	9.42E-66
SEPSECS	0.62202206	6.28E-59
SLC2A9	0.5186133	2.27E-38
TBC1D19	0.68290458	4.02E-75
SH3D19	0.69485777	8.89E-79
STIM2	0.50394345	5.41E-36
RAPGEF2	0.61026814	3.34E-56
SNX25	0.50731769	1.57E-36
UFSP2	0.75371089	8.86E-100
KLF3	0.58060987	8.29E-50
KLHL5	0.5665626	5.30E-47
FAM149A	0.62212947	5.93E-59
ELP4	0.7671519	1.98E-105
ZPR1	0.55669118	4.14E-45
MTCH2	0.69952497	2.96E-80
SC5D	0.55054798	5.80E-44
HSPA8	0.53935479	6.20E-42
SIAE	0.56630766	5.95E-47
EHD1	0.54364668	1.06E-42
OSBP	0.72183145	9.95E-88
PUS3	0.5645604	1.30E-46
KMT5B	0.63635782	2.05E-62
PPP6R3	0.58114367	6.44E-50
NRXN2	0.5119075	2.86E-37
CPT1A	0.63988193	2.67E-63
CCND1	0.61247244	1.05E-56
CCDC86	0.50452081	4.38E-36
PRPF19	0.61730831	8.04E-58
TMEM109	0.6317166	2.89E-61
RNF141	0.62894968	1.37E-60
EIF4G2	0.6396696	3.02E-63
GALNT18	0.56563391	8.04E-47
BIRC2	0.58851738	1.90E-51
UBE4A	0.54934732	9.66E-44
DDX6	0.5771997	4.09E-49
CBL	0.51279315	2.06E-37
HIPK3	0.6136208	5.73E-57
FBXO3	0.7810111	1.14E-111
PDHX	0.60095362	4.02E-54
COMMD9	0.51860264	2.28E-38
AMBRA1	0.59407721	1.25E-52
MADD	0.54310257	1.32E-42
SOX6	0.56733592	3.75E-47
C11orf58	0.64406244	2.30E-64
NUP98	0.57859384	2.14E-49
GTF2H1	0.5483148	1.49E-43
VWF	0.52230826	5.49E-39
PSMD9	0.52481998	2.07E-39
PPFIBP1	0.61512991	2.57E-57
PRDM4	0.5456429	4.59E-43
COQ5	0.6483859	1.74E-65
CORO1C	0.55627888	4.95E-45
KCTD10	0.53606753	2.36E-41
MLEC	0.55373523	1.49E-44
ATP5F1B	0.6094339	5.17E-56
PTGES3	0.56202484	4.00E-46
BCL7A	0.51837842	2.49E-38
LIN7A	0.52859217	4.70E-40
METAP2	0.61529949	2.35E-57
LTA4H	0.58091756	7.17E-50
GPN3	0.62076858	1.24E-58
VPS29	0.50496292	3.73E-36
MANSC1	0.54831797	1.49E-43
DUSP16	0.57428393	1.58E-48
CREBL2	0.70165286	6.15E-81
ALDH2	0.52474417	2.13E-39
CDKN1B	0.62090563	1.15E-58
GTF2H3	0.55607901	5.40E-45
SPRING1	0.69113211	1.28E-77

GAS1	-2.08149	2.584635	8.97E-49	1.24E-47
CLEC12A	2.824655	2.032762	9.18E-49	1.27E-47
TGFB1	1.583881	7.029264	9.68E-49	1.33E-47
DLX3	-2.23031	-1.33718	9.92E-49	1.36E-47
DUSP26	-2.66909	-0.29142	1.08E-48	1.49E-47
DMAC2L	-1.01041	5.158889	1.21E-48	1.65E-47
GMNC	-3.53995	0.242634	1.27E-48	1.74E-47
ATG16L2	2.333422	4.713987	1.48E-48	2.02E-47
LGALS1	2.161008	8.02163	1.53E-48	2.10E-47
LNX1	-1.50072	4.093691	1.54E-48	2.10E-47
MPC1	-1.52924	5.795432	1.64E-48	2.25E-47
SEPTIN1	1.848638	3.667109	1.69E-48	2.30E-47
CD6	2.376554	3.401158	1.78E-48	2.43E-47
AGFG2	-1.17046	4.805039	1.79E-48	2.43E-47
DLX1	3.200241	-0.15528	1.81E-48	2.46E-47
EMP3	1.716883	5.932787	1.97E-48	2.68E-47
UHRF1	2.534423	2.189076	1.97E-48	2.68E-47
NANOS1	-1.59459	1.22172	1.97E-48	2.68E-47
ATP5F1A	-1.21836	9.02022	2.36E-48	3.20E-47
KDF1	-2.03518	2.352141	2.45E-48	3.31E-47
DDX25	-2.44371	-0.24938	2.53E-48	3.42E-47
HUNK	-1.72162	3.883052	2.55E-48	3.45E-47
GNRH1	2.822748	1.011102	2.58E-48	3.49E-47
BTC	-1.78645	2.0977	2.69E-48	3.63E-47
SYNE2	-1.16808	7.858874	2.83E-48	3.82E-47
GOT2	-1.06547	6.815938	2.87E-48	3.87E-47
PPP1R3C	1.972495	6.111418	2.96E-48	3.99E-47
CAPSL	-2.82023	-2.35233	3.05E-48	4.10E-47
MMUT	-1.05883	5.816478	3.14E-48	4.22E-47
ANXA2R	2.044721	2.080385	3.17E-48	4.26E-47
CD86	2.158206	4.07349	3.94E-48	5.29E-47
IL11	-3.31156	-0.362	4.72E-48	6.34E-47
ZNF469	2.08307	2.427491	4.73E-48	6.34E-47
SLC25A4	-2.3836	-2.38657	5.10E-48	6.83E-47
ACLY	1.3696	8.719489	5.11E-48	6.83E-47
CSF2RA	1.950269	3.654371	5.36E-48	7.17E-47
CSF2RA.1	1.950269	3.654371	5.36E-48	7.17E-47
HOXB8	-2.24011	3.43616	5.79E-48	7.74E-47
FGF10	-3.40879	-2.07973	5.82E-48	7.77E-47
MAP4K1	2.405517	3.274955	6.04E-48	8.04E-47
FXYD5	1.779134	6.353776	6.38E-48	8.49E-47
SERPINB	1.372379	5.076995	7.12E-48	9.46E-47
SERPINH	1.568597	7.854817	7.15E-48	9.50E-47
LRRC37B	1.056649	2.769131	7.24E-48	9.60E-47
ALX1	-3.07911	-0.87755	7.84E-48	1.04E-46
FOXJ1	-3.16366	0.55709	8.71E-48	1.15E-46
CGREF1	3.068336	4.827771	8.84E-48	1.17E-46
HMOX1	2.348268	8.144552	8.95E-48	1.18E-46
IDO1	3.299546	5.164255	8.98E-48	1.18E-46
PPM1H	-1.87329	4.590581	9.22E-48	1.22E-46
NCF1	2.306355	1.974912	1.00E-47	1.32E-46
LRRK1	1.655352	5.092503	1.08E-47	1.43E-46
BEST1	1.908401	2.891631	1.34E-47	1.76E-46
TSPAN33	-1.54795	6.4914	1.34E-47	1.76E-46
CNKSR1	-2.5852	1.593373	1.36E-47	1.79E-46
NEXMIF	-2.93848	1.184434	1.44E-47	1.88E-46
SLC27A3	1.506637	5.122283	1.47E-47	1.93E-46
VWF	2.652394	10.06699	1.52E-47	1.99E-46
LLGL2	-1.23368	5.704986	1.53E-47	2.00E-46
CSTA	2.348086	1.847168	1.62E-47	2.12E-46
VWA3B	-2.25696	-2.03142	1.80E-47	2.35E-46
ABCB6	1.769947	3.404613	1.85E-47	2.40E-46
SUCNR1	-2.13062	4.540834	1.85E-47	2.41E-46
PDIA5	1.767113	6.119566	1.87E-47	2.43E-46
CCL4	2.665176	3.975585	1.95E-47	2.53E-46
KCNK9	4.559344	2.131608	1.96E-47	2.55E-46
PELI2	-1.22761	3.989667	2.02E-47	2.63E-46
LYPD6	-2.68955	0.613047	2.16E-47	2.80E-46
TNFRSF1	1.433368	6.2636	2.27E-47	2.93E-46
PLEKHN1	2.478827	1.604944	2.35E-47	3.04E-46
AXDND1	-2.0877	-1.36022	2.40E-47	3.11E-46
MUC6	-2.64219	0.474245	2.78E-47	3.59E-46
ZNF683	3.385228	1.379935	2.81E-47	3.63E-46
CXCR6	2.720804	2.788109	2.85E-47	3.68E-46
CEMIP2	1.249771	6.958936	2.87E-47	3.69E-46
DIRAS2	3.013143	4.828047	3.28E-47	4.22E-46
KSR1	1.926998	6.769301	3.87E-47	4.96E-46
MYCN	-1.90071	0.357598	3.98E-47	5.10E-46
CDH8	3.474628	1.100756	4.14E-47	5.31E-46
RACK1	1.221313	10.60492	4.18E-47	5.36E-46
VDR	-1.70222	4.622318	4.25E-47	5.44E-46
ARHGEF	1.177703	5.557535	4.36E-47	5.58E-46
SKA3	2.105476	1.165492	4.59E-47	5.87E-46
LRIT3	-2.07975	-0.31849	4.92E-47	6.28E-46
GPR4	1.948623	5.740268	5.10E-47	6.51E-46
FCHO1	2.64284	2.540086	5.35E-47	6.83E-46
ITM2C	-1.39903	7.362641	6.34E-47	8.08E-46
AOAH	2.197205	4.402356	6.55E-47	8.35E-46
C5orf58	2.920882	-0.92244	6.85E-47	8.72E-46
EBF2	2.778093	3.765462	7.04E-47	8.96E-46
MUC3A	3.434905	4.430828	7.12E-47	9.05E-46
NOD2	2.465731	2.239723	8.09E-47	1.03E-45
ABCC3	2.334257	7.481646	8.10E-47	1.03E-45
CAT	-1.25402	7.662846	8.26E-47	1.05E-45
RPS2	1.483646	9.495431	8.38E-47	1.06E-45
CLK4	1.51321	5.241214	9.13E-47	1.15E-45
CD53	1.817361	5.952089	9.28E-47	1.17E-45
MICALL2	2.065588	4.72344	9.84E-47	1.24E-45
KAZN	-1.08086	5.04419	1.01E-46	1.28E-45
AP5M1	-1.04692	5.531045	1.04E-46	1.31E-45
CRTAM	3.051043	1.499236	1.10E-46	1.38E-45
TMEM24	-1.0693	7.130999	1.11E-46	1.40E-45
CGAS	1.408324	2.546497	1.24E-46	1.56E-45
SLC35G2	1.430975	3.426178	1.32E-46	1.66E-45
NCR1	2.608564	-0.84592	1.34E-46	1.68E-45
CASP5	2.923863	-1.14169	1.54E-46	1.94E-45
ACSL6	-2.13245	1.90084	1.64E-46	2.05E-45
IL18BP	1.5296	5.051092	1.69E-46	2.12E-45
SEMA4A	-1.57051	4.625952	1.70E-46	2.13E-45
PARP15	2.687215	1.610208	1.86E-46	2.32E-45
C1S5-TX	-1.81908	-0.15705	2.05E-46	2.56E-45
ENTPD1	1.008939	6.569912	2.12E-46	2.65E-45
CDON	2.26035	4.828128	2.15E-46	2.68E-45
FSTL4	-2.51998	1.061824	2.18E-46	2.71E-45
TNFSF13	2.525002	3.974405	2.35E-46	2.93E-45
CENPH	1.386033	2.133232	2.48E-46	3.08E-45
FANCI	1.299974	3.686448	2.48E-46	3.08E-45
KITLG	-1.61765	5.691399	2.50E-46	3.11E-45
SH3BP1	1.664602	3.567839	2.58E-46	3.20E-45
GZMB	2.579832	2.535164	2.63E-46	3.26E-45
ALOX15B	4.29244	2.045343	2.72E-46	3.37E-45
PAPPA2	-3.3854	3.975798	2.80E-46	3.46E-45
PCED1B	1.706429	3.192675	3.03E-46	3.74E-45
P4HB	1.41513	9.979436	3.08E-46	3.81E-45
AP3S1	1.047227	5.717476	3.09E-46	3.81E-45
ADGRF1	-4.01887	4.504256	3.31E-46	4.08E-45
ABARAP	-1.50273	7.213728	3.44E-46	4.24E-45
BIRC7	7.053311	3.698656	3.85E-46	4.74E-45
GPRIN1	2.192344	2.403749	4.22E-46	5.18E-45
KLHL21	-1.16543	5.831648	4.27E-46	5.24E-45
PLEK	2.265497	5.162028	4.28E-46	5.24E-45
TNFRSF9	4.082219	3.15122	4.65E-46	5.69E-45
DOK2	1.896366	3.297945	4.84E-46	5.92E-45
GRHL2	-3.7504	2.340648	4.92E-46	6.01E-45
PTGDR	2.223757	0.709837	5.71E-46	6.97E-45
FBXO21	-1.04199	6.37759	5.74E-46	7.01E-45
GPR19	2.215232	-0.81039	5.89E-46	7.19E-45
OR51E1	2.509907	3.011007	6.14E-46	7.49E-45
HROB	1.552742	1.076048	6.71E-46	8.17E-45
SIGLEC1	2.555143	4.526486	6.94E-46	8.45E-45
SHC1	1.087006	7.233871	6.98E-46	8.48E-45
STRA8	3.341427	1.36955	6.99E-46	8.49E-45
BATF3	2.142698	1.897373	7.06E-46	8.57E-45
VTCN1	-3.5146	3.306778	7.07E-46	8.58E-45
HIF1A	-1.17011	7.632734	8.14E-46	9.85E-45
CENPM	2.215496	1.55403	8.28E-46	1.00E-44
VSIR	1.207559	6.336299	8.98E-46	1.08E-44
CORO1C	1.077716	7.366451	9.94E-46	1.20E-44
CYGB	2.03538	5.43791	1.01E-45	1.22E-44
ANK2	-2.12225	6.664541	1.07E-45	1.29E-44
RBM47	-1.07774	7.213121	1.10E-45	1.33E-44
FBXO17	1.558064	6.81292	1.16E-45	1.40E-44
C3orf85	-3.29876	-0.04568	1.17E-45	1.41E-44
CD8A	3.606392	5.159876	1.20E-45	1.44E-44
PHYHD1	-2.22867	3.587475	1.29E-45	1.55E-44
NRL	-1.1548	0.867989	1.33E-45	1.59E-44
HPCA	2.80166	0.646774	1.38E-45	1.65E-44
MS4A4E	3.147683	0.06477	1.51E-45	1.81E-44
KCNE4	2.05939	5.139402	1.70E-45	2.03E-44
SAMD14	1.631015	3.021812	1.75E-45	2.09E-44
TET3	1.031586	4.547207	1.79E-45	2.13E-44
DOCK2	2.271757	5.150645	1.94E-45	2.31E-44
BCL7A	-1.01744	4.796757	2.00E-45	2.38E-44
MAMDC2	-1.9708	1.825053	2.05E-45	2.43E-44
VWA5A	-1.16079	4.878433	2.14E-45	2.54E-44
JMY	-1.17802	4.500346	2.21E-45	2.63E-44
CD160	1.86894	0.398098	2.25E-45	2.66E-44
OCLN	-2.06403	3.474557	2.33E-45	2.76E-44
UCN	2.596671	-0.0253	2.59E-45	3.06E-44
TBX19	1.587926	2.307381	2.95E-45	3.49E-44
CXCL10	3.413426	5.180501	2.97E-45	3.52E-44
PSORS1C	3.675751	-1.22935	3.04E-45	3.60E-44
PILRA	1.659131	3.550182	3.37E-45	3.98E-44
GTSE1	2.359284	1.776696	3.42E-45	4.04E-44
RASSF4	1.705512	7.539358	3.44E-45	4.06E-44
OGFR	1.062291	5.791212	3.78E-45	4.45E-44
SIRPA	1.551036	7.726265	3.92E-45	4.61E-44
RGS5	2.606575	11.07502	3.97E-45	4.66E-44
MCM10	2.279128	0.990622	4.20E-45	4.93E-44
RAP2B	1.193755	5.537909	4.28E-45	5.02E-44
HLA-DOB	2.312685	8.303398	4.30E-45	5.05E-44
CD96	2.489113	3.516005	4.77E-45	5.59E-44
NAP1L2	-2.22694	2.609593	4.83E-45	5.65E-44
SEMA6A	1.931694	6.754035	4.94E-45	5.78E-44
FFAR4	2.774915	0.801736	5.69E-45	6.66E-44
RAB24	1.672104	3.782795	5.87E-45	6.85E-44
AKNA	1.474653	5.501828	6.48E-45	7.56E-44
IQGAP3	2.503575	3.145193	6.94E-45	8.10E-44
FAM184A	-1.54398	2.003314	7.00E-45	8.16E-44
MRGPRF	-2.20064	2.267323	7.38E-45	8.59E-44

CAND1	0.59022181	8.29E-52
RAB5B	0.66284445	2.30E-69
MDM1	0.50628206	2.30E-36
CNOT2	0.51757939	3.37E-38
KRR1	0.53502392	3.61E-41
CHD4	0.59790841	1.86E-53
UHRF1BP1L	0.51145734	3.39E-37
CHPT1	0.53915239	6.73E-42
USP5	0.54315625	1.29E-42
GNPTAB	0.51703199	4.15E-38
LPCAT3	0.5990507	1.05E-53
PRKAB1	0.54240688	1.76E-42
HCFC2	0.54472459	6.74E-43
RAB35	0.55696776	3.67E-45
RIC8B	0.6585338	3.47E-68
FGFR1OP2	0.53491766	3.76E-41
TDP2	0.67644458	3.22E-73
FRK	0.50678733	1.91E-36
TMEM14C	0.5559897	5.62E-45
PAK1IP1	0.53631228	2.14E-41
GCNT2	0.61950861	2.46E-58
MCM9	0.5395347	5.76E-42
RNGTT	0.54412182	8.66E-43
SERINC1	0.72050024	2.91E-87
HINT3	0.65324059	9.18E-67
NCOA7	0.53953886	5.75E-42
SASH1	0.56800974	2.76E-47
MTRF1L	0.61585855	1.75E-57
MAPK14	0.62857006	1.69E-60
KCTD20	0.6124586	1.06E-56
SRSF3	0.62658385	5.11E-60
MCM3	0.53516653	3.40E-41
RNF8	0.55259311	2.42E-44
CILK1	0.61604069	1.58E-57
FBXO9	0.62521167	1.09E-59
CAP2	0.67320115	2.79E-72
FBXL4	0.59098419	5.71E-52
CCNC	0.53193292	1.25E-40
PTP4A1	0.60557179	3.82E-55
MED23	0.54655995	3.13E-43
WASF1	0.5347564	4.02E-41
ALDH5A1	0.72488523	8.27E-89
ACOT13	0.64696853	4.08E-65
SMAP1	0.57522273	1.02E-48
C6orf62	0.63041149	6.03E-61
SNX3	0.65242673	1.51E-66
HBS1L	0.60110233	3.73E-54
PEX7	0.54746199	2.14E-43
ZBTB24	0.5157691	6.70E-38
FIG4	0.5698453	1.20E-47
ADGRG6	0.50662292	2.03E-36
PHACTR2	0.52588865	1.36E-39
EPM2A	0.65318365	9.50E-67
SLC39A7	0.597425	2.37E-53
SLC22A2	0.61085367	2.46E-56
CUTA	0.52581555	1.40E-39
QKI	0.53800854	1.07E-41
FAM120B	0.60364386	1.03E-54
BICRAL	0.57342451	2.35E-48
PPP2R5D	0.67274376	3.78E-72
SRF	0.5026701	8.58E-36
DUSP22	0.54676518	2.87E-43
EXOC2	0.6529562	1.09E-66
TMEM30A	0.55656822	4.37E-45
PRPF4B	0.54377278	1.00E-42
ENPP5	0.5751725	1.05E-48
ERBIN	0.54095784	3.21E-42
NUDT12	0.53622295	2.22E-41
SEMA5A	0.57981703	1.20E-49
GHR	0.61883024	3.55E-58
HMGCS1	0.63119277	3.89E-61
DAP	0.51702652	4.16E-38
NNT	0.54225985	1.88E-42
MRPS30	0.51103595	3.97E-37
HSPA9	0.6297168	8.91E-61
MRPS27	0.52364841	3.26E-39
IK	0.55309333	1.96E-44
HMGCR	0.50982819	6.21E-37
CERT1	0.60700011	1.83E-55
FAF2	0.65249987	1.44E-66
RNF130	0.58190784	4.49E-50
ARSB	0.67428979	1.36E-72
CLINT1	0.58968078	1.08E-51
CNOT6	0.58896582	1.53E-51
TTC1	0.51406329	1.28E-37
MSH3	0.58956454	1.14E-51
CCNG1	0.55931919	1.32E-45
GOLPH3	0.58037694	9.25E-50
SUB1	0.54310881	1.32E-42
NPR3	0.63386673	8.54E-62
FAM172A	0.56278543	2.86E-46
TARS1	0.547692	1.94E-43
RAD1	0.61239583	1.09E-56
AGXT2	0.5992839	9.34E-54
SLC12A7	0.52798975	5.97E-40
RAD50	0.5554919	6.97E-45
GNPDA1	0.59596883	4.90E-53
SKP1	0.62160093	7.91E-59
NUP155	0.5288824	4.19E-40
PPP2CA	0.60533883	4.30E-55
C5orf15	0.58402897	1.64E-50
LIFR	0.61439454	3.80E-57
TRIM23	0.64225055	6.69E-64
TRAPPC13	0.60891147	6.79E-56
SEC24A	0.51592913	6.31E-38
TXNDC15	0.63402512	7.80E-62
TTC33	0.57833879	2.41E-49
WWC1	0.50329955	6.83E-36
SMAD5	0.60459776	6.29E-55
CSNK1A1	0.55053613	5.83E-44
CPEB4	0.53187024	1.28E-40
HRH2	0.57923166	1.58E-49
ZNF346	0.55507334	8.35E-45
EHHADH	0.7386241	7.42E-94
TBCCD1	0.62577053	8.02E-60
HGD	0.50966122	6.61E-37
ARL6	0.56722348	3.94E-47
KPNA1	0.57352925	2.24E-48
UBE3A	0.65792675	5.07E-68
ARMC8	0.59099779	5.68E-52
KAT2B	0.56322064	2.36E-46
PDCD10	0.50956911	6.84E-37
PRKAR2A	0.56962089	1.33E-47
ACAP2	0.5544123	1.11E-44
FXR1	0.51701416	4.18E-38
BBX	0.5137755	1.42E-37
IFT57	0.53844529	8.99E-42
UMPS	0.62149345	8.38E-59
NCBP2	0.50713424	1.68E-36
SNX4	0.75794621	1.61E-101
FRMD4B	0.60303425	1.40E-54
ATP6V1A	0.61446282	3.67E-57
NEK11	0.52971155	3.02E-40
PLSCR4	0.55347444	1.66E-44
WDR48	0.5140101	1.30E-37
COMMD2	0.65478192	3.56E-67
EIF4G1	0.5230289	4.15E-39
NEK4	0.54547081	4.94E-43
MOB1A	0.59557215	5.97E-53
LMAN2L	0.63554401	3.27E-62
PIKFYVE	0.53501496	3.62E-41
NCL	0.63996084	2.55E-63
ACTR1B	0.63859147	5.66E-63
SLC35F5	0.63581055	2.81E-62
ACTR3	0.58772059	2.79E-51
EPB41L5	0.62778548	2.62E-60
SF3B6	0.53034194	2.35E-40
DNAJC27	0.56760243	3.32E-47
STAM2	0.67870822	7.03E-74
GPD2	0.59401431	1.29E-52
ACVR1	0.6530143	1.05E-66
TANC1	0.53538182	3.12E-41
GTF3C2	0.60007925	6.26E-54
SNX17	0.67006422	2.20E-71
IFIH1	0.65376803	6.64E-67
GCA	0.57799637	2.82E-49
SPTBN1	0.63815157	7.30E-63
RTN4	0.66920767	3.84E-71
HTRA2	0.63555418	3.26E-62
ACADL	0.54087495	3.32E-42
MRPL19	0.64886268	1.31E-65
LANCL1	0.65444676	4.38E-67
GLS	0.55840831	1.96E-45
PAPOLG	0.55683154	3.90E-45
PECR	0.59477086	8.88E-53
UNC50	0.66004135	1.35E-68
CCT4	0.58871485	1.72E-51
GGCX	0.64420564	2.11E-64
EHBP1	0.71171734	2.99E-84
TXNDC9	0.63623236	2.21E-62
COQ10B	0.53605494	2.38E-41
SF3B1	0.60144419	3.14E-54
PDCL3	0.59016056	8.54E-52
MOB4	0.64878966	1.37E-65
CHMP3	0.69949832	3.02E-80
ZNF142	0.52841513	5.04E-40
CNPPD1	0.64073889	1.62E-63
UXS1	0.61505558	2.68E-57
STK16	0.61415766	4.31E-57
HDLBP	0.56716956	4.04E-47
PPP1R7	0.58788097	2.58E-51
STK25	0.52525827	1.74E-39
TAF1B	0.62962068	9.41E-61
ODC1	0.65269491	1.28E-66
BIRC6	0.54844429	1.42E-43
NOL10	0.65065477	4.43E-66
PLEKHB2	0.56832449	2.40E-47
GORASP2	0.56284899	2.78E-46
STRN	0.59919323	9.78E-54
CEBPZ	0.64899354	1.21E-65

RASSF5	1.721888	4.859714	7.80E-45	9.07E-44
SLAMF8	2.714456	4.257215	9.05E-45	1.05E-43
BCKDHB	-1.25423	4.411971	9.31E-45	1.08E-43
MMP25	2.231425	1.509021	9.74E-45	1.13E-43
TLCD3A	1.59913	4.793245	9.88E-45	1.15E-43
CORO2B	-1.99466	2.82559	1.07E-44	1.23E-43
MARCHF	1.452373	2.030735	1.13E-44	1.30E-43
UBASH3A	2.804767	1.597408	1.16E-44	1.34E-43
MLC1	2.213219	0.000138	1.27E-44	1.47E-43
CDC45	2.30846	1.348396	1.29E-44	1.49E-43
KIF4A	2.240747	2.327979	1.30E-44	1.50E-43
KIAA0895	2.069483	4.711115	1.40E-44	1.61E-43
MICAL1	1.485358	5.387154	1.42E-44	1.64E-43
PHYHIP	-1.81578	0.827969	1.44E-44	1.65E-43
FKBP11	1.971074	4.588213	1.46E-44	1.68E-43
BNIP3	1.552127	7.922773	1.83E-44	2.10E-43
DIPK2B	1.900637	6.035326	1.96E-44	2.24E-43
TMEM44	1.815137	4.442909	1.98E-44	2.27E-43
OVOL2	-3.48299	-0.83966	2.14E-44	2.45E-43
RIMKLA	2.350763	4.919115	2.24E-44	2.56E-43
VEPH1	-1.4659	4.985679	2.35E-44	2.68E-43
LOXL2	2.692045	6.883401	2.56E-44	2.92E-43
RIOX2	-1.20452	3.783918	2.64E-44	3.01E-43
SAMD12	-1.83331	3.626182	2.75E-44	3.14E-43
NT5DC3	2.280148	5.394893	2.76E-44	3.15E-43
HLA-DMA	1.505943	7.077046	2.79E-44	3.18E-43
KCNN1	4.302173	1.765673	2.89E-44	3.29E-43
DOK7	-2.3759	0.558542	3.04E-44	3.45E-43
CORIN	-1.53489	0.690581	3.32E-44	3.77E-43
SLC12A5	2.419099	0.321913	3.49E-44	3.96E-43
TSPAN32	2.024559	1.119787	3.51E-44	3.98E-43
ADA	2.236405	3.642406	3.63E-44	4.12E-43
SCNN1A	-2.87529	6.598153	3.75E-44	4.24E-43
ICOS	3.369253	0.758431	4.08E-44	4.62E-43
HLA-G	3.861267	5.260953	4.23E-44	4.78E-43
CES4A	3.683959	4.942507	4.24E-44	4.79E-43
KRT36	3.506597	-2.69666	4.53E-44	5.11E-43
PRRT3	-1.26069	1.382309	4.56E-44	5.14E-43
SLC17A9	3.36095	3.358809	5.48E-44	6.17E-43
IKZF1	2.090276	4.628656	5.91E-44	6.66E-43
PPP1R13L	1.612492	5.597899	6.21E-44	6.97E-43
LAT	3.358579	0.11662	6.21E-44	6.97E-43
PLPPR5	6.385665	2.757439	6.21E-44	6.97E-43
L1CAM	-3.20968	5.0743	6.22E-44	6.98E-43
EME1	2.1046	0.602291	7.41E-44	8.31E-43
STAB1	1.709616	7.151218	7.69E-44	8.62E-43
CXCL9	3.599366	6.282986	7.98E-44	8.93E-43
TNFSF14	3.798141	2.762996	8.22E-44	9.19E-43
TNFRSF1	2.933536	0.560897	8.27E-44	9.25E-43
ACOT6	-2.1877	-2.04753	8.34E-44	9.32E-43
CHST6	-2.67968	-0.30611	8.81E-44	9.84E-43
SH2D2A	2.74419	2.553998	9.05E-44	1.01E-42
ABCG1	1.301151	6.113234	9.26E-44	1.03E-42
UQCRFS1	-1.21546	6.01539	9.51E-44	1.06E-42
CXCR3	3.179323	2.491202	9.58E-44	1.07E-42
SLC11A1	2.397924	3.650824	1.00E-43	1.11E-42
MTFP1	1.646913	3.733147	1.03E-43	1.14E-42
HIBADH	-1.05984	6.8013	1.03E-43	1.14E-42
ABCA1	1.582665	6.858803	1.04E-43	1.15E-42
ACAP1	1.855489	3.981927	1.06E-43	1.18E-42
SHROOM	-1.8583	5.120378	1.10E-43	1.22E-42
GFI1	2.490536	1.467956	1.11E-43	1.23E-42
UBE2L6	1.118415	6.786431	1.21E-43	1.34E-42
ARHGEF1	1.187714	6.398706	1.25E-43	1.39E-42
TRIM22	1.383953	6.614799	1.29E-43	1.43E-42
AVPR1B	3.745146	1.862153	1.31E-43	1.45E-42
HOGA1	-1.89053	4.995889	1.33E-43	1.47E-42
P2RY8	1.85641	4.93015	1.41E-43	1.55E-42
P2RY8.1	1.85641	4.93015	1.41E-43	1.55E-42
ARNT2	-1.76432	5.550906	1.47E-43	1.61E-42
SMPDL3A	1.82413	6.648339	1.50E-43	1.64E-42
ADGRE1	2.742346	1.928613	1.55E-43	1.70E-42
CTSS	1.948151	7.310489	1.55E-43	1.70E-42
SLFN12L	2.699215	1.279292	1.56E-43	1.72E-42
SPN	2.148892	4.32993	1.64E-43	1.79E-42
PLAAT2	-2.03749	0.478587	1.70E-43	1.86E-42
ARC	-2.09622	1.95543	1.72E-43	1.89E-42
IL15RA	1.479514	4.290443	1.73E-43	1.89E-42
RGS10	1.626314	4.423024	1.85E-43	2.02E-42
PPFIA4	3.176115	4.882705	1.86E-43	2.03E-42
VPS13D	-1.00288	6.247879	2.07E-43	2.26E-42
P3H1	1.337514	5.345328	2.24E-43	2.44E-42
COL4A1	1.885398	10.33982	2.27E-43	2.47E-42
FUT2	-1.87769	0.173913	2.36E-43	2.57E-42
C1orf116	-2.90861	3.072689	2.39E-43	2.60E-42
DNAJC28	-1.13343	1.030746	2.41E-43	2.62E-42
S100Z	2.254337	-1.51155	2.63E-43	2.85E-42
LIMD2	2.015157	4.447895	2.73E-43	2.96E-42
MT1F	-2.14647	4.891122	2.99E-43	3.24E-42
PCDHB1	-2.84239	-0.92474	3.01E-43	3.25E-42
EVI2B	1.961351	4.605232	3.23E-43	3.49E-42
PITPNM3	-1.49531	1.945325	3.48E-43	3.75E-42
SIPA1	1.10997	5.751106	3.59E-43	3.87E-42
MSC	3.100943	5.256161	3.62E-43	3.90E-42
KIFC1	1.976036	2.455746	3.96E-43	4.27E-42
SLC2A1	1.997928	8.148982	4.08E-43	4.40E-42
ACSBG2	-1.80122	-1.19957	4.10E-43	4.42E-42
CD74	1.764018	12.01252	4.21E-43	4.52E-42
SOD3	-1.54026	5.479449	4.32E-43	4.64E-42
ASPHD1	2.681568	3.594751	4.77E-43	5.12E-42
SH3GL1	1.055984	6.48429	4.84E-43	5.20E-42
C11orf16	-2.0223	-2.12402	4.88E-43	5.24E-42
TBL1Y	-3.37524	-1.3541	4.99E-43	5.35E-42
WNT7A	-2.99707	-1.17702	5.02E-43	5.38E-42
TC-479C5.	2.119457	1.425916	5.18E-43	5.55E-42
CDKN2B	1.699067	3.684897	5.81E-43	6.21E-42
UGT2A1	-4.6407	-0.12622	5.93E-43	6.34E-42
SLC30A8	-3.30914	0.013669	6.15E-43	6.57E-42
SLC10A6	2.620778	0.283995	6.17E-43	6.59E-42
C3	3.38985	10.79366	6.19E-43	6.61E-42
TAP2	1.158707	5.394218	6.24E-43	6.65E-42
TAGAP	1.991153	3.55976	6.38E-43	6.80E-42
CDC25C	2.740126	0.361246	6.44E-43	6.86E-42
AICDA	4.83219	0.084478	6.91E-43	7.35E-42
L1-TNFRS	2.490453	1.789419	7.26E-43	7.72E-42
RHOG	1.0214	5.710625	7.29E-43	7.74E-42
SAMD13	-1.46569	-0.18042	7.40E-43	7.86E-42
GIMAP4	1.319684	6.356877	7.74E-43	8.21E-42
TRIM59	1.18221	2.698814	7.87E-43	8.35E-42
TRAF2	1.051237	4.354656	8.25E-43	8.72E-42
CDC6	2.01999	2.324869	8.63E-43	9.12E-42
DNASE1	-2.19777	4.869213	1.01E-42	1.07E-41
CAPS	-1.77372	3.728445	1.02E-42	1.08E-41
MELK	2.408047	1.884792	1.04E-42	1.09E-41
PTTG1	2.384555	2.799692	1.10E-42	1.16E-41
BAG1	-1.21366	6.144332	1.15E-42	1.21E-41
C20orf204	2.1259	0.146656	1.15E-42	1.22E-41
TP73	3.064172	0.589322	1.23E-42	1.30E-41
OIP5	1.580536	0.007312	1.25E-42	1.32E-41
BTBD19	1.930122	3.547238	1.29E-42	1.35E-41
RNF175	2.422349	0.835578	1.38E-42	1.45E-41
CCNA2	2.009997	2.604737	1.43E-42	1.50E-41
KCNJ16	-1.56089	7.783796	1.52E-42	1.59E-41
ITGAM	2.019271	4.634842	1.57E-42	1.65E-41
PRKD1	-1.05138	4.582703	1.60E-42	1.67E-41
SPC24	2.326949	1.899343	1.73E-42	1.80E-41
PCDH15	-3.93443	-1.53474	1.78E-42	1.85E-41
DNM3	-1.20237	3.019461	1.79E-42	1.87E-41
LBX2	2.060387	0.172075	1.82E-42	1.90E-41
BRINP3	-4.30043	-0.77662	1.90E-42	1.98E-41
LILRA2	1.9169	2.223145	1.97E-42	2.05E-41
PRSS35	-2.17525	1.757314	1.97E-42	2.05E-41
PKHD1L1	-2.4584	-0.8635	2.07E-42	2.15E-41
FOLR3	-3.20852	0.259259	2.22E-42	2.30E-41
NRG3	1.974154	2.543445	2.27E-42	2.35E-41
PDXK	-1.05943	7.123068	2.58E-42	2.67E-41
NADSYN1	1.075683	6.009772	2.59E-42	2.69E-41
SEL1L3	1.68156	7.248836	2.71E-42	2.81E-41
PIEZO2	2.169649	4.937593	2.93E-42	3.03E-41
PCDHB1	1.729235	2.64194	3.01E-42	3.11E-41
HLA-DQA	2.222426	8.023793	3.25E-42	3.36E-41
PLXNA4	-2.43323	2.205951	3.33E-42	3.44E-41
PGF	3.944888	7.29906	3.42E-42	3.53E-41
ZNF331	-1.18921	4.813367	3.43E-42	3.54E-41
MAP3K9	-1.65558	2.663742	3.44E-42	3.55E-41
TBC1D9	-1.13652	5.873167	3.46E-42	3.57E-41
AFAP1L1	1.499523	5.403588	3.47E-42	3.57E-41
CXCL11	3.518046	3.085291	3.54E-42	3.64E-41
TMEM23	2.098326	2.88954	3.54E-42	3.64E-41
FDXR	1.414672	4.715357	3.63E-42	3.73E-41
CDCA7L	1.785126	4.300273	3.63E-42	3.73E-41
QRFPR	2.949426	3.695934	3.68E-42	3.77E-41
TNK2	1.398868	5.217063	4.02E-42	4.12E-41
ATP8B1	-1.23601	5.034087	4.03E-42	4.13E-41
ALDOA	1.113025	10.93623	4.52E-42	4.62E-41
SCIMP	1.975424	2.868772	4.58E-42	4.69E-41
SLC41A2	1.232074	5.302456	4.82E-42	4.92E-41
TRMT1	1.182523	4.829647	4.82E-42	4.92E-41
CD99	1.286085	7.388878	4.85E-42	4.94E-41
CD99.1	1.286085	7.388878	4.85E-42	4.94E-41
MICB	1.309296	2.60667	4.93E-42	5.02E-41
BIRC3	2.335033	7.437499	5.26E-42	5.35E-41
GK	-1.47782	4.588224	5.29E-42	5.38E-41
CDH26	1.641259	-0.02767	5.37E-42	5.45E-41
PDE1B	1.659539	4.08301	5.72E-42	5.80E-41
FPR3	2.22177	5.133214	5.72E-42	5.80E-41
JAG2	1.677197	5.512088	5.91E-42	5.99E-41
ZBP1	2.870961	1.328449	5.93E-42	6.01E-41
GPR65	1.921621	3.268198	6.25E-42	6.33E-41
FAM193B	2.116218	5.482003	6.31E-42	6.38E-41
OAS1	1.454048	5.937912	6.45E-42	6.52E-41
NMI	1.033217	4.628259	6.77E-42	6.83E-41
LGI4	4.601065	6.474485	6.77E-42	6.83E-41
PCK1	-2.81884	8.034213	7.20E-42	7.26E-41
TRAF3IP2	1.074081	5.407974	7.51E-42	7.57E-41
CDKN1C	-1.50691	4.845928	7.96E-42	8.01E-41
RGL4	1.937761	0.70151	8.05E-42	8.11E-41
CD3D	2.89998	3.329937	8.20E-42	8.25E-41
NBPF15	1.257134	3.880962	9.06E-42	9.09E-41
HSD3B7	2.240802	6.194166	9.25E-42	9.28E-41

PRKD3	0.50736439	1.55E-36
DCAF17	0.52411303	2.72E-39
RAB3GAP1	0.68478376	1.10E-75
SLC25A12	0.52425741	2.58E-39
RMDN2	0.62843134	1.83E-60
DARS1	0.68621508	4.07E-76
PLCL1	0.53619566	2.25E-41
SLC1A4	0.56592426	7.06E-47
SOS1	0.57159862	5.42E-48
ORC2	0.50431091	4.73E-36
COX7A2L	0.57620934	6.49E-49
PNO1	0.59304377	2.08E-52
ORC4	0.66751943	1.15E-70
ATF2	0.68059249	1.96E-74
THADA	0.53920946	6.58E-42
TRAK2	0.58945052	1.21E-51
C2orf42	0.60096356	4.00E-54
PCYOX1	0.70253792	3.19E-81
EPAS1	0.52355592	3.38E-39
SUMO1	0.6779972	1.14E-73
NFE2L2	0.6517531	2.28E-66
MSH6	0.58205641	4.18E-50
PLEKHA3	0.6217421	7.32E-59
EPHA4	0.60833165	9.17E-56
FARSB	0.60025604	5.72E-54
ALMS1	0.51215212	2.61E-37
BCL9	0.58261131	3.22E-50
DNAJC16	0.75570305	1.36E-100
CACYBP	0.62125575	9.54E-59
SCP2	0.77786573	3.25E-110
CEP104	0.70707653	1.05E-82
FAM20B	0.61691294	9.94E-58
TCEANC2	0.50693387	1.81E-36
TMEM59	0.72316271	3.38E-88
LRRC42	0.61457937	3.45E-57
MRPL37	0.55094325	4.91E-44
ICMT	0.58974745	1.04E-51
RPL22	0.53432809	4.77E-41
STXBP3	0.68215001	6.76E-75
EDEM3	0.57475373	1.27E-48
ATP5PB	0.69364339	2.13E-78
RAP1A	0.59578411	5.37E-53
RNF19B	0.68032752	2.34E-74
SCAMP3	0.60505637	4.97E-55
ASH1L	0.61000107	3.84E-56
RHOU	0.5764734	5.74E-49
GON4L	0.50047679	1.90E-35
MEF2D	0.51104924	3.95E-37
DOCK7	0.525095	1.86E-39
SWT1	0.71260809	1.50E-84
LEPR	0.51304864	1.87E-37
IVNS1ABP	0.64348476	3.23E-64
MFN2	0.69030138	2.31E-77
SMG7	0.66050805	1.01E-68
SLC35D1	0.62255607	4.70E-59
WLS	0.56102576	6.22E-46
RO60	0.62553514	9.14E-60
UCHL5	0.72697504	1.48E-89
BCAS2	0.72661039	2.00E-89
AGMAT	0.67442392	1.24E-72
CRYZ	0.66205739	3.79E-69
TMEM9	0.54084778	3.36E-42
WARS2	0.64697459	4.07E-65
HAO2	0.6240076	2.12E-59
OSCP1	0.57700358	4.49E-49
EXOC8	0.69449712	1.15E-78
GNPAT	0.67206834	5.90E-72
TSNAX	0.71179276	2.82E-84
C1orf109	0.6124722	1.05E-56
RRAGC	0.63659236	1.79E-62
LGALS8	0.61522005	2.45E-57
MTR	0.5141221	1.25E-37
AKT3	0.58142306	5.65E-50
ETV3	0.61012155	3.61E-56
ACADM	0.74399057	6.48E-96
ADGRL2	0.57433853	1.54E-48
SDHB	0.67513137	7.76E-73
RPF1	0.6283943	1.87E-60
KDM5B	0.55854672	1.85E-45
CTBS	0.62664673	4.94E-60
KLHL12	0.70518532	4.39E-82
SSX2IP	0.60775984	1.23E-55
ZNHIT6	0.57199292	4.53E-48
RBBP5	0.74674908	5.41E-97
GPR89A	0.54700535	2.59E-43
CDK18	0.51619767	5.70E-38
RAB29	0.72960614	1.65E-90
HMGCL	0.6346655	5.41E-62
CD46	0.64103228	1.37E-63
APH1A	0.68580296	5.43E-76
EBNA1BP2	0.63264271	1.71E-61
MPL	0.54166601	2.40E-42
AKR1A1	0.50188266	1.14E-35
PIK3R3	0.52965474	3.09E-40
BLZF1	0.7068826	1.22E-82
NSUN4	0.63883794	4.90E-63
TMED5	0.65383813	6.36E-67
DR1	0.61464559	3.33E-57
CNN3	0.62555315	9.05E-60
PRRC2C	0.50218859	1.02E-35
ABCD3	0.74232691	2.85E-95
DPH5	0.54723981	2.35E-43
DARS2	0.67083471	1.33E-71
UTP25	0.64434856	1.94E-64
SYF2	0.5571825	3.35E-45
SLC35A3	0.69182505	7.82E-78
RCOR3	0.53129404	1.61E-40
DHDDS	0.71537191	1.72E-85
NSL1	0.75491359	2.86E-100
ARID1A	0.52837504	5.13E-40
RPA2	0.56982213	1.22E-47
PPP1R8	0.68661731	3.08E-76
STX12	0.70849587	3.56E-83
MTARC2	0.69796257	9.32E-80
OSBPL9	0.68162279	9.69E-75
MESD	0.66804859	8.15E-71
RCN2	0.52751455	7.19E-40
STAG1	0.58971327	1.06E-51
IFT46	0.63507154	4.29E-62
DDX59	0.64501603	1.31E-64
CAMSAP2	0.50474206	4.04E-36
ATF6	0.66741665	1.23E-70
FASTKD2	0.66500532	5.79E-70
CREB1	0.59134777	4.78E-52
SPCS2	0.59796932	1.81E-53
ANKRD13C	0.6606727	9.08E-69
PHF3	0.59556622	5.98E-53
AKAP7	0.62602764	6.96E-60
SGK1	0.52810086	5.71E-40
RNF146	0.57559325	8.63E-49
FBXL5	0.75534632	1.90E-100
FOXO3	0.55855757	1.84E-45
PKD2	0.65153372	2.60E-66
ABCG2	0.55652434	4.45E-45
STBD1	0.56043957	8.05E-46
CCNI	0.54814253	1.61E-43
MFSD1	0.50440596	4.57E-36
RAB3GAP2	0.66186416	4.28E-69
EEF2KMT	0.58643567	5.18E-51
UBN1	0.56680294	4.76E-47
KLF12	0.55349658	1.65E-44
HS1BP3	0.59026212	8.13E-52
LDAH	0.68381057	2.16E-75
WDR35	0.59365641	1.54E-52
CYP20A1	0.56120497	5.75E-46
NDUFB3	0.54048302	3.90E-42
GTF3C3	0.59207665	3.35E-52
SATB2	0.62011506	1.77E-58
UBE2B	0.50204617	1.08E-35
KLF9	0.50351295	6.32E-36
TJP2	0.65595627	1.72E-67
CPSF3	0.55530184	7.56E-45
HEATR1	0.54337565	1.18E-42
RAD23B	0.65564789	2.09E-67
FKBP15	0.54083149	3.38E-42
ABITRAM	0.58723847	3.52E-51
SET	0.63091042	4.56E-61
GLE1	0.6841805	1.67E-75
RAB14	0.64295661	4.41E-64
TRIM32	0.57038774	9.41E-48
FBXW2	0.58656381	4.87E-51
NEK6	0.55339283	1.72E-44
PPP6C	0.68550832	6.66E-76
RBM18	0.65005099	6.39E-66
HSDL2	0.68973907	3.44E-77
MAPKAP1	0.51924566	1.78E-38
INVS	0.6155926	2.01E-57
GALNT12	0.53858301	8.50E-42
DENND1A	0.60443615	6.84E-55
ALG2	0.6923289	5.46E-78
KDSR	0.6632117	1.82E-69
VPS4B	0.68907152	5.50E-77
YLPM1	0.56034067	8.41E-46
FCF1	0.61461801	3.38E-57
BBOF1	0.54763711	1.99E-43
NEK9	0.5421067	2.00E-42
DNAL1	0.67135615	9.42E-72
ACOT2	0.52413973	2.70E-39
TTLL5	0.53496878	3.69E-41
DLST	0.60589323	3.24E-55
ZC2HC1C	0.51907098	1.91E-38
ALDH6A1	0.65925074	2.22E-68
EIF2B2	0.54582076	4.26E-43
COQ6	0.50876652	9.21E-37
ATL2	0.65604825	1.63E-67
YPEL5	0.59296576	2.16E-52
FAM98A	0.57496115	1.16E-48
YIPF4	0.61451628	3.57E-57
AFTPH	0.72879344	3.26E-90
LGALSL	0.66250955	2.84E-69

HES4	2.317793	3.20445	9.70E-42	9.73E-41
SLC22A1	-2.86802	1.480816	9.89E-42	9.91E-41
TROAP	2.837483	1.610445	1.05E-41	1.05E-40
FAM107A	-1.52126	5.310806	1.06E-41	1.06E-40
EIF4EBP1	2.131304	5.382323	1.09E-41	1.09E-40
MARCHF	2.787347	0.566562	1.13E-41	1.13E-40
SEC61G	1.131652	5.733982	1.18E-41	1.18E-40
EACAM2	1.86856	1.101267	1.25E-41	1.25E-40
NCF2	1.765075	4.220672	1.37E-41	1.37E-40
UNC5B	1.775211	6.775177	1.46E-41	1.46E-40
PLEKHG3	-1.01296	5.587674	1.51E-41	1.51E-40
NOP16	1.138595	4.101389	1.57E-41	1.57E-40
TSPAN4	1.217164	6.546822	1.68E-41	1.67E-40
RAD51	1.608267	1.227887	1.69E-41	1.68E-40
S1PR4	1.829813	1.991145	1.69E-41	1.68E-40
TC-435M1	2.697585	-2.43084	1.69E-41	1.68E-40
SUSD1	-1.40181	4.273631	1.71E-41	1.69E-40
ERCC5	1.003997	5.100645	1.72E-41	1.70E-40
TESPA1	2.466393	2.302374	1.82E-41	1.81E-40
SIRPG	3.383927	2.114132	1.86E-41	1.84E-40
WEE2	-1.72872	-2.16085	1.90E-41	1.88E-40
NFASC	-1.6993	5.604766	2.00E-41	1.97E-40
S100A14	-2.09628	2.393082	2.03E-41	2.00E-40
RELB	1.130522	4.659507	2.05E-41	2.02E-40
PAQR4	1.751378	2.735852	2.08E-41	2.05E-40
FAM110A	1.139157	3.484601	2.08E-41	2.05E-40
GDF6	3.541921	3.888253	2.10E-41	2.07E-40
ACSM3	-1.41377	5.10643	2.17E-41	2.14E-40
LMO7	-1.2068	6.276813	2.23E-41	2.19E-40
TCIRG1	1.495897	5.743845	2.36E-41	2.32E-40
FGR	1.528984	4.192569	2.47E-41	2.43E-40
TNIP1	1.058851	8.683862	2.50E-41	2.45E-40
CHD3	-1.0109	6.773167	2.81E-41	2.76E-40
HLA-DRA	1.736983	10.38182	2.98E-41	2.93E-40
GRB10	1.464578	7.420984	3.01E-41	2.95E-40
PPL	-1.53054	5.561439	3.24E-41	3.18E-40
FOXM1	2.456079	3.372318	3.79E-41	3.71E-40
ETNK2	-1.69416	5.22482	3.82E-41	3.74E-40
CTAGE9	4.035288	-0.44293	3.87E-41	3.78E-40
GNA15	1.760659	2.802468	4.09E-41	3.99E-40
MUC12	4.328877	1.388324	4.09E-41	3.99E-40
SIM1	-3.01313	4.392719	4.33E-41	4.22E-40
NRP2	1.716425	6.283756	4.50E-41	4.38E-40
PXDC1	1.19078	5.95751	4.90E-41	4.77E-40
NLRC3	1.326237	3.418155	5.55E-41	5.41E-40
FJX1	1.52284	3.878533	5.93E-41	5.77E-40
TACC3	1.468739	4.122219	5.96E-41	5.80E-40
B3GNTL1	1.35026	2.233102	6.12E-41	5.94E-40
IDNK	-1.16592	3.189514	6.81E-41	6.60E-40
ANGPTL1	-2.49352	2.787317	6.92E-41	6.71E-40
C3AR1	1.902629	4.60531	6.98E-41	6.75E-40
PROCR	1.610744	5.174184	7.22E-41	6.98E-40
KIF6	-1.5322	0.64611	8.06E-41	7.79E-40
RAC2	2.099753	5.30809	8.08E-41	7.81E-40
RAD54L	2.516557	1.508991	8.50E-41	8.20E-40
ACO1	-1.08028	6.702412	9.19E-41	8.86E-40
PTPN22	2.084622	2.490516	1.05E-40	1.01E-39
MMP11	2.044516	4.121596	1.07E-40	1.03E-39
PGGHG	3.587538	6.809159	1.16E-40	1.11E-39
GRAMD1	-1.37729	4.496378	1.19E-40	1.14E-39
GATA2	-1.50992	4.116065	1.40E-40	1.35E-39
HLA-DPA	1.860745	9.294217	1.58E-40	1.51E-39
TRIP13	1.79675	2.369176	1.59E-40	1.52E-39
GRK5	1.273867	4.776691	1.63E-40	1.56E-39
FLI1	1.222897	5.492945	1.76E-40	1.69E-39
LGR6	-1.58065	0.256154	1.80E-40	1.72E-39
PCSK6	2.986042	6.514009	2.00E-40	1.91E-39
E2F8	2.672443	1.001732	2.02E-40	1.93E-39
LRRC25	2.317283	4.162458	2.07E-40	1.98E-39
ELOVL2	3.208044	2.30357	2.12E-40	2.02E-39
SEPTIN5	1.727447	4.191214	2.13E-40	2.03E-39
TSGA10	-1.04394	2.123811	2.23E-40	2.13E-39
ABCA8	-1.95575	3.300575	2.29E-40	2.18E-39
ASAP1	1.056374	6.050135	2.37E-40	2.25E-39
SLAMF6	2.540314	3.170213	2.46E-40	2.34E-39
ABHD17A	1.002936	5.072322	2.50E-40	2.37E-39
DDX41	1.049711	6.758044	2.54E-40	2.41E-39
PYCARD	2.073117	3.950533	2.54E-40	2.41E-39
AXL	1.605163	6.871351	2.67E-40	2.54E-39
CD80	2.468768	-0.02642	2.68E-40	2.54E-39
EPHX2	-1.3591	6.298884	2.93E-40	2.78E-39
TRIM2	-1.73248	6.557311	2.95E-40	2.79E-39
C16orf89	-2.84192	3.071765	3.06E-40	2.89E-39
SIRPD	3.020418	-2.52928	3.22E-40	3.05E-39
CD8B	3.423617	2.795713	3.30E-40	3.12E-39
TNNI2	2.439632	-0.45174	3.67E-40	3.46E-39
BMERB1	-1.0543	3.929095	3.82E-40	3.61E-39
SLC6A1	2.296345	3.000946	3.83E-40	3.62E-39
MOXD1	-2.28302	4.429816	4.03E-40	3.80E-39
EHD3	-1.26013	5.320743	4.19E-40	3.95E-39
AANAT	2.599315	-1.47266	4.38E-40	4.13E-39
ALKBH6	1.705621	1.432043	4.41E-40	4.15E-39
EVI2A	1.89324	3.806391	4.70E-40	4.42E-39
IFI44	1.56831	4.88534	4.72E-40	4.43E-39
MFHAS1	-1.12471	4.969391	5.12E-40	4.81E-39
GRAMD4	1.777203	6.396383	5.24E-40	4.91E-39
CMTM3	1.527879	5.468704	5.33E-40	5.00E-39
IMPG2	-1.37738	-0.25638	5.39E-40	5.05E-39
MAPK8IP	2.103667	6.052739	5.42E-40	5.08E-39
NCAPH	1.952899	1.567608	5.42E-40	5.08E-39
CEP55	2.384791	2.37548	5.66E-40	5.30E-39
RPS19	1.487044	9.47085	5.80E-40	5.43E-39
HS3ST3B1	-1.27748	2.37794	5.81E-40	5.43E-39
CD3E	2.665195	4.830082	6.04E-40	5.64E-39
PCYT2	-1.29113	4.95208	6.45E-40	6.01E-39
KLRK1	3.383192	-0.19162	6.62E-40	6.18E-39
HPSE2	-2.36892	0.187899	6.95E-40	6.47E-39
MTNR1A	-2.96922	-2.06072	7.02E-40	6.53E-39
CHRNA1	5.366327	0.143316	7.50E-40	6.97E-39
SLC22A7	-3.13417	3.173903	7.50E-40	6.97E-39
EXOSC5	1.328069	4.333108	7.76E-40	7.21E-39
RHOH	2.237934	2.713894	9.42E-40	8.73E-39
RIN1	2.052073	3.258045	9.51E-40	8.81E-39
JUP	-1.02271	7.989081	1.06E-39	9.81E-39
IFNG	4.419576	-0.26164	1.07E-39	9.90E-39
ACBD7	-1.62316	-2.02609	1.07E-39	9.91E-39
ANO4	3.751425	3.182033	1.10E-39	1.01E-38
ANKZF1	1.467214	5.484641	1.21E-39	1.11E-38
HAPLN1	3.555795	4.119619	1.25E-39	1.16E-38
NDRG2	-1.31236	6.906306	1.28E-39	1.18E-38
TFCP2L1	-3.10846	6.308462	1.28E-39	1.18E-38
REEP4	1.153629	3.841196	1.37E-39	1.26E-38
UGT8	-1.70704	5.480723	1.52E-39	1.40E-38
RIT1	1.114886	6.272474	1.60E-39	1.47E-38
ICAM3	1.991146	1.438018	1.63E-39	1.49E-38
FAS	1.21608	5.100569	1.63E-39	1.49E-38
ZMYND1	-1.31716	2.14273	1.77E-39	1.63E-38
IL3RA	1.453824	4.426772	1.79E-39	1.65E-38
IL3RA.1	1.453824	4.426772	1.79E-39	1.65E-38
MCHR1	5.719793	3.583957	1.94E-39	1.77E-38
CD27	3.316166	3.199468	1.97E-39	1.80E-38
SLAMF7	2.831527	4.327328	2.02E-39	1.85E-38
PLEKHO2	1.048708	6.057602	2.06E-39	1.88E-38
OPN4	5.452946	-1.23395	2.06E-39	1.88E-38
POPDC2	1.587835	3.225147	2.25E-39	2.05E-38
DLX6	2.571919	-1.0497	2.29E-39	2.09E-38
CCL18	5.706436	3.948726	2.30E-39	2.09E-38
DNMT3L	-2.92278	-3.15728	2.37E-39	2.16E-38
DYSF	1.492348	7.207218	2.44E-39	2.23E-38
GZMM	2.094255	1.156267	2.45E-39	2.23E-38
OR2I1P	3.838309	4.903658	2.48E-39	2.26E-38
GUCY1B1	1.204961	6.073771	2.50E-39	2.27E-38
TAGLN3	-3.15398	-0.07558	2.57E-39	2.34E-38
FRY	-1.15584	5.633781	2.78E-39	2.53E-38
PLAC8L1	2.617648	-1.51968	2.79E-39	2.53E-38
CBLN3	1.648989	3.077832	2.89E-39	2.63E-38
PIMREG	2.325891	1.013793	2.99E-39	2.71E-38
SLC29A4	2.201607	5.487143	3.27E-39	2.96E-38
IRF7	1.773424	4.725509	3.32E-39	3.01E-38
LIN37	1.021413	1.84578	3.34E-39	3.03E-38
FNDC11	-2.00631	-1.13522	3.45E-39	3.12E-38
CPA6	2.5171	-0.01265	3.54E-39	3.20E-38
CLCN5	-1.40479	6.657112	3.60E-39	3.25E-38
COX4I2	2.850827	3.597528	4.02E-39	3.63E-38
PNMA2	2.488407	6.262108	4.04E-39	3.64E-38
PRC1	1.36566	3.680297	4.10E-39	3.69E-38
CCDC142	1.092386	2.838424	4.33E-39	3.90E-38
MCUB	1.652756	3.46118	4.38E-39	3.94E-38
AMZ1	3.964085	1.264364	4.40E-39	3.96E-38
HLA-DRB	1.639707	9.573651	4.41E-39	3.96E-38
GCH1	-1.10674	3.700171	4.80E-39	4.32E-38
DOCK10	1.435479	5.121888	4.97E-39	4.47E-38
AURKB	2.687046	1.593817	5.03E-39	4.52E-38
RAPGEFL	1.786021	2.556799	5.05E-39	4.53E-38
PRR7	2.391852	1.53476	5.20E-39	4.67E-38
DUSP4	1.930934	4.124583	5.59E-39	5.01E-38
SLC43A3	1.611386	5.044157	5.82E-39	5.21E-38
IKZF3	2.58488	4.144545	5.88E-39	5.26E-38
TMPRSS2	-3.49483	4.659032	6.09E-39	5.44E-38
SAMHD1	1.435073	6.86666	6.15E-39	5.48E-38
MAP3K7C	2.125075	4.945389	6.16E-39	5.49E-38
HJURP	2.628418	1.845337	6.19E-39	5.51E-38
KIF5A	-2.13124	-0.1475	6.27E-39	5.58E-38
KCTD13	1.043339	3.772913	6.31E-39	5.61E-38
TLR5	-1.17915	3.317833	6.32E-39	5.62E-38
PPM1F	1.04565	6.087103	6.75E-39	6.00E-38
PSAT1	-2.48882	4.828252	6.79E-39	6.04E-38
RAB25	-3.48081	3.020864	7.09E-39	6.30E-38
B3GALT9	-1.25777	0.714724	7.29E-39	6.47E-38
ABCD1	1.050028	4.742012	7.33E-39	6.51E-38
CD3G	2.567342	2.855355	7.36E-39	6.53E-38
PLVAP	1.954596	9.543893	7.40E-39	6.57E-38
KISS1R	5.147573	3.186161	7.49E-39	6.64E-38
MAP3K12	1.357405	4.045416	7.53E-39	6.67E-38
HS3ST5	-3.17457	-2.17965	7.86E-39	6.96E-38
CD52	2.119786	4.493804	8.35E-39	7.38E-38
UBD	3.194221	4.651952	8.50E-39	7.51E-38
BIRC5	2.501262	2.916918	8.99E-39	7.94E-38
PCDH17	2.012182	5.582183	9.12E-39	8.05E-38
TIGIT	2.899943	2.719128	9.23E-39	8.14E-38

CRIPT	0.62639512	5.68E-60
SLC17A5	0.7035161	1.54E-81
IDE	0.58306127	2.60E-50
IFIT3	0.56128284	5.56E-46
MXI1	0.60292398	1.48E-54
SMNDC1	0.60691174	1.91E-55
C10orf88	0.55355069	1.61E-44
TCTN3	0.62182818	6.99E-59
DENND10	0.60545664	4.05E-55
WDR11	0.59256	2.64E-52
ARMH3	0.61904342	3.16E-58
GOT1	0.60779065	1.21E-55
GNA13	0.60355557	1.07E-54
PANK3	0.53590757	2.52E-41
TEK	0.51580847	6.60E-38
RCL1	0.5372784	1.45E-41
MOB3B	0.6923475	5.39E-78
NUP43	0.50571423	2.83E-36
LRP11	0.51351238	1.57E-37
PCMT1	0.63361093	9.88E-62
MYCT1	0.51426643	1.18E-37
MRPS14	0.76997221	1.15E-106
TCP1	0.5850653	9.99E-51
SNX19	0.58434605	1.41E-50
SLC10A7	0.6391918	3.99E-63
NUDCD1	0.50871661	9.38E-37
ENY2	0.54768554	1.95E-43
KIAA1217	0.54751053	2.10E-43
EPC1	0.55505569	8.41E-45
PROSER1	0.56524491	9.57E-47
UFM1	0.67254183	4.32E-72
WBP4	0.64791666	2.31E-65
ELF1	0.59646287	3.83E-53
KBTBD7	0.69543501	5.86E-79
ALG5	0.63518749	4.02E-62
ETF1	0.5583599	2.00E-45
FAM53C	0.53936308	6.18E-42
PAIP2	0.75318569	1.45E-99
KDM3B	0.60853474	8.25E-56
SERP1	0.50272347	8.42E-36
PLS1	0.68600021	4.73E-76
ARL1	0.66114387	6.75E-69
EPHX2	0.58571354	7.32E-51
RNF170	0.59290695	2.23E-52
UBIAD1	0.67250258	4.43E-72
TARDBP	0.63411821	7.40E-62
AKAP1	0.5675222	3.44E-47
COIL	0.67824239	9.63E-74
SPOP	0.66451777	7.91E-70
SLC35B1	0.54595689	4.03E-43
TRIM6	0.65028727	5.54E-66
TENT4B	0.59429412	1.12E-52
PYROXD1	0.5847168	1.18E-50
PSPC1	0.57698467	4.52E-49
PDZRN3	0.54838584	1.45E-43
RNF2	0.62164809	7.71E-59
TRMT1L	0.77897437	1.00E-110
NAA50	0.55176321	3.46E-44
DESI2	0.5735661	2.20E-48
MAPK8IP1	0.53158174	1.44E-40
CRY2	0.67746653	1.62E-73
CAT	0.74959545	4.03E-98
TBC1D15	0.67416512	1.47E-72
ZCCHC17	0.57393186	1.86E-48
KHDRBS1	0.66534128	4.67E-70
POLR3GL	0.66852481	5.99E-71
ZNF639	0.52923793	3.64E-40
PIK3CA	0.55730408	3.17E-45
PDS5A	0.57751957	3.53E-49
CLCC1	0.65170627	2.34E-66
ACVR2A	0.59404012	1.27E-52
POLK	0.59866088	1.28E-53
FYTTD1	0.59482594	8.64E-53
MTERF4	0.5714941	5.69E-48
OCRL	0.52434946	2.48E-39
MRPS2	0.53149478	1.49E-40
KIAA1191	0.53512466	3.46E-41
COPA	0.73029926	9.24E-91
ZC3H7A	0.56084452	6.74E-46
SERAC1	0.53556532	2.90E-41
SHLD2	0.71384065	5.73E-85
PRXL2A	0.6534317	8.16E-67
ZNF644	0.55651164	4.48E-45
BBS9	0.62346857	2.85E-59
PMS2	0.56575317	7.62E-47
SEPTIN7	0.54048369	3.90E-42
KLHL7	0.56241409	3.37E-46
HERPUD2	0.59943774	8.65E-54
MRM2	0.59424286	1.15E-52
SMU1	0.68582668	5.34E-76
SLC25A51	0.5782997	2.45E-49
RECK	0.57379749	1.98E-48
ACO1	0.63658069	1.81E-62
DCAF10	0.59969844	7.58E-54
KIAA1549	0.66730987	1.31E-70
TRIM24	0.51266945	2.15E-37
ECD	0.68942975	4.28E-77
P4HA1	0.52712043	8.40E-40
VPS26A	0.66774714	9.91E-71
MED13L	0.51807938	2.79E-38
RNF11	0.64439195	1.89E-64
RASSF8	0.55159638	3.71E-44
WWP1	0.64313908	3.96E-64
SPRYD7	0.71397866	5.14E-85
ATP7B	0.66788444	9.07E-71
ZC3H13	0.61830945	4.70E-58
NLN	0.53493175	3.74E-41
OPTN	0.70050451	1.44E-80
ATF1	0.59860014	1.32E-53
EEF1AKMT3	0.50569908	2.85E-36
KBTBD4	0.65953477	1.86E-68
ATPAF1	0.63382826	8.73E-62
AMD1	0.58104421	6.75E-50
SERPINA7	0.50578231	2.76E-36
MORF4L2	0.55673575	4.06E-45
RAB9A	0.60665477	2.19E-55
TTC21B	0.6073489	1.53E-55
SLC36A1	0.50468312	4.13E-36
LPGAT1	0.55700836	3.61E-45
PLA2G12A	0.52708743	8.51E-40
ACSL3	0.53777801	1.18E-41
DNPEP	0.62085985	1.18E-58
SLC12A4	0.57414918	1.68E-48
NCOA3	0.5087425	9.29E-37
NCOA5	0.56156069	4.92E-46
VAPB	0.62005807	1.83E-58
ARFGEF2	0.53783307	1.15E-41
ZNFX1	0.53372442	6.09E-41
CSE1L	0.57837746	2.36E-49
STAU1	0.63068672	5.17E-61
MOCS3	0.60474191	5.85E-55
RNF114	0.5647976	1.17E-46
MTRR	0.62332926	3.08E-59
FASTKD3	0.64188661	8.28E-64
PEPD	0.58024345	9.85E-50
VAMP7	0.61073345	2.62E-56
VAMP7	0.61073345	2.62E-56
STAMBP	0.6495081	8.87E-66
MCEE	0.50359339	6.14E-36
PAIP2B	0.6745855	1.12E-72
SNRNP27	0.64756473	2.86E-65
USP22	0.58467574	1.20E-50
ZNF576	0.60853152	8.27E-56
ZNF45	0.63043121	5.97E-61
USP9X	0.54167751	2.39E-42
BTN2A2	0.50800728	1.22E-36
SIRT5	0.54100495	3.15E-42
MRS2	0.61459384	3.42E-57
WRNIP1	0.59889116	1.14E-53
RRP36	0.52413733	2.70E-39
SERPINB6	0.52340947	3.58E-39
XPO5	0.56766309	3.23E-47
OARD1	0.58079673	7.59E-50
AARS2	0.54113545	2.98E-42
MED20	0.56894115	1.81E-47
TBCC	0.50398795	5.32E-36
MAD2L1BP	0.65951606	1.88E-68
KLHDC3	0.5515639	3.76E-44
GLO1	0.65021862	5.78E-66
RREB1	0.5431133	1.32E-42
SSR1	0.67649203	3.12E-73
RIOK1	0.57905848	1.72E-49
SLC35B3	0.59889595	1.14E-53
NUP153	0.61596297	1.65E-57
DEK	0.62999131	7.64E-61
LRRFIP1	0.50902633	8.37E-37
CNOT1	0.59436307	1.09E-52
BBS2	0.54391792	9.43E-43
C16orf70	0.65015938	5.99E-66
GOT2	0.52627934	1.17E-39
CLYBL	0.51987255	1.40E-38
RAP2A	0.62434008	1.77E-59
SLC10A2	0.54428153	8.11E-43
ABCC4	0.56891668	1.83E-47
EFNB2	0.52486757	2.03E-39
TM9SF2	0.64159496	9.82E-64
DMAC2L	0.55297202	2.06E-44
MRPS7	0.59918773	9.80E-54
MIF4GD	0.55253368	2.49E-44
TTF1	0.56739804	3.64E-47
GTF3C4	0.62461914	1.51E-59
DDX31	0.54755166	2.06E-43
INSIG2	0.55180542	3.40E-44
POLR1B	0.67703397	2.17E-73
SLC25A23	0.60252586	1.81E-54
GTF2F1	0.54336946	1.18E-42
THOC2	0.50930368	7.55E-37
MED1	0.58959393	1.12E-51
ATG4C	0.68277732	4.39E-75
GPR108	0.64542502	1.02E-64

SH2D5	2.492425	1.134493	9.26E-39	8.16E-38
SLA	1.798012	5.21483	9.51E-39	8.38E-38
RTP4	1.751442	3.988341	9.59E-39	8.44E-38
PRKCA	-1.20403	5.548564	1.04E-38	9.13E-38
BRIP1	1.836985	1.647215	1.15E-38	1.01E-37
FOXN1	-2.52911	-2.38818	1.16E-38	1.02E-37
RGN	-1.41434	4.238675	1.22E-38	1.07E-37
PNCK	5.12166	6.82358	1.22E-38	1.07E-37
POLE2	1.355916	1.051851	1.22E-38	1.07E-37
ADAMTS1	2.184736	4.072035	1.23E-38	1.08E-37
SIGLEC7	2.131983	1.684148	1.26E-38	1.11E-37
ADAMTSL	2.130721	4.249554	1.55E-38	1.36E-37
NCAPG	2.156866	2.055882	1.57E-38	1.38E-37
RTP2	5.017444	-1.53346	1.60E-38	1.40E-37
SIRPB1	2.679201	2.602266	1.68E-38	1.47E-37
EXOC3L1	1.534076	3.99632	1.69E-38	1.48E-37
GALNT6	-1.17352	3.197835	1.74E-38	1.52E-37
IGSF3	-1.14517	5.681343	1.81E-38	1.58E-37
COL5A2	2.081708	6.761058	1.81E-38	1.58E-37
TUBAL3	-3.79627	-0.24987	1.96E-38	1.71E-37
AFMID	-1.06426	5.127449	2.05E-38	1.78E-37
CLEC12B	3.630976	-1.91753	2.08E-38	1.81E-37
PIP5K1B	-1.66894	2.696389	2.25E-38	1.96E-37
C3orf52	-1.92493	2.293781	2.39E-38	2.08E-37
TRPA1	3.186297	2.728133	2.41E-38	2.10E-37
MSR1	2.09328	5.888693	2.42E-38	2.11E-37
LAG3	3.552276	3.339675	2.68E-38	2.32E-37
NEIL3	2.756739	0.175085	2.69E-38	2.33E-37
KIF18B	2.764835	1.546858	2.79E-38	2.42E-37
CLEC1B	2.752869	-2.09917	2.93E-38	2.54E-37
TPX2	2.30401	3.828202	3.09E-38	2.67E-37
PBX1	-1.39883	6.074118	3.38E-38	2.93E-37
LCP1	1.83688	7.258375	3.40E-38	2.94E-37
SLC29A2	-1.88601	2.756689	3.42E-38	2.95E-37
FLT1	1.899861	8.992504	3.67E-38	3.17E-37
TPI1	1.004248	9.906093	3.80E-38	3.28E-37
GPR141	2.321092	0.440055	4.01E-38	3.45E-37
WDR72	-1.85797	6.362566	4.13E-38	3.56E-37
POLQ	2.168654	0.86523	4.64E-38	3.99E-37
EPX	-1.4571	-1.58261	4.86E-38	4.18E-37
DCN	-2.5387	7.01548	4.87E-38	4.19E-37
HSD17B7	1.659371	3.727742	5.11E-38	4.39E-37
NTRK2	-2.06561	5.66717	5.12E-38	4.40E-37
LTA	2.639054	-0.11791	5.93E-38	5.09E-37
PLEKHD1	-2.48507	0.140773	6.29E-38	5.39E-37
KHDRBS2	-2.26042	-2.26333	6.40E-38	5.49E-37
KIF13B	-1.30522	6.320071	6.55E-38	5.61E-37
GPR176	1.445889	4.973813	6.80E-38	5.82E-37
PTHLH	5.514876	5.452043	7.28E-38	6.22E-37
MARCKS	1.110918	7.588326	7.32E-38	6.26E-37
EIF4A1	2.146419	3.177632	7.45E-38	6.37E-37
ATP2B2	3.442503	3.94726	7.81E-38	6.66E-37
TNIP3	3.742685	0.582244	8.05E-38	6.87E-37
PRELID3A	1.626472	0.862194	8.36E-38	7.13E-37
HES5	3.755099	-1.36593	9.11E-38	7.75E-37
MPP2	-1.94527	1.178033	9.25E-38	7.87E-37
RCSD1	1.248509	5.009953	9.69E-38	8.23E-37
MYBL1	1.771887	2.937319	1.01E-37	8.56E-37
PLA2G4A	-1.53868	2.937628	1.10E-37	9.36E-37
TLR2	1.61405	4.815448	1.11E-37	9.39E-37
DIAPH2	1.140741	5.507655	1.11E-37	9.40E-37
SLC9A5	1.781411	0.974164	1.18E-37	1.00E-36
HTATIP2	1.217601	5.568964	1.31E-37	1.10E-36
TBX15	3.121387	2.633097	1.33E-37	1.12E-36
THOC6	1.112169	4.669634	1.35E-37	1.14E-36
PLD4	2.282223	3.530785	1.50E-37	1.26E-36
ORAI3	1.301159	5.034668	1.51E-37	1.27E-36
PSMB10	1.394209	5.039939	1.51E-37	1.27E-36
ALDOC	2.295582	6.443164	1.52E-37	1.28E-36
B4GALNT	-2.21004	3.369881	1.69E-37	1.42E-36
CHEK2	1.232374	2.68296	1.74E-37	1.46E-36
TLR6	1.746726	2.045538	1.75E-37	1.47E-36
SIT1	2.719709	2.075452	1.84E-37	1.54E-36
ADAMDEC	4.291612	2.530947	1.90E-37	1.59E-36
NEBL	-1.48773	6.288852	1.93E-37	1.61E-36
BCL2A1	2.579341	2.09966	2.05E-37	1.72E-36
LINGO3	2.494229	-1.17956	2.16E-37	1.81E-36
ITGA4	1.766983	4.95603	2.23E-37	1.87E-36
CD180	2.14301	2.871489	2.27E-37	1.89E-36
IQCH	-1.15279	1.498881	2.27E-37	1.90E-36
SPATA18	1.704701	5.514478	2.42E-37	2.02E-36
RAB29	-1.12941	5.777849	2.59E-37	2.16E-36
GZMK	3.35374	4.009413	2.62E-37	2.18E-36
MS4A4A	1.935581	4.514672	2.68E-37	2.23E-36
P11-514O1	2.21028	-1.89829	2.72E-37	2.27E-36
PLXNA3	1.364351	5.3158	2.91E-37	2.43E-36
PSKH2	-3.92861	-3.59677	2.93E-37	2.44E-36
GABRE	2.952024	4.678803	2.95E-37	2.46E-36
TENM1	2.480488	4.568212	3.05E-37	2.53E-36
GGH	-1.41747	4.379233	3.37E-37	2.80E-36
POLG2	1.120169	3.158619	3.45E-37	2.86E-36
UBAP1L	2.193332	2.463111	3.47E-37	2.88E-36
MFNG	1.215548	4.57355	3.49E-37	2.89E-36
RPL10	1.060016	10.34445	3.50E-37	2.90E-36
C10orf95	-1.36385	-1.30755	3.74E-37	3.10E-36
NUDT1	1.252919	3.158783	3.79E-37	3.13E-36
PNMA8A	-1.85806	4.133092	3.82E-37	3.16E-36
NR1H3	1.04728	5.228951	4.14E-37	3.42E-36
MELTF	-2.51854	3.994058	4.44E-37	3.67E-36
ADAMTS1	3.397334	1.699434	4.52E-37	3.73E-36
MMP14	1.469798	8.016448	4.57E-37	3.77E-36
PALM3	-1.84351	3.820723	4.73E-37	3.90E-36
GJA1	1.781523	7.368574	4.83E-37	3.98E-36
PLSCR3	1.581617	-0.37812	4.85E-37	4.00E-36
RASGRP3	1.060526	5.531455	4.93E-37	4.06E-36
FABP5	1.933883	3.302487	6.17E-37	5.06E-36
TEK	-1.4167	5.588053	6.28E-37	5.15E-36
HEY1	1.594546	4.304196	6.28E-37	5.15E-36
TTC29	-2.3571	-2.79504	7.17E-37	5.87E-36
CXorf65	3.384129	-1.37367	7.35E-37	6.01E-36
MNDA	1.816209	3.990899	7.62E-37	6.24E-36
ARID5A	1.413379	4.966709	7.69E-37	6.28E-36
TNFSF8	1.855862	2.19054	9.15E-37	7.47E-36
CDS1	-1.3815	4.91512	9.63E-37	7.85E-36
KCNJ9	-1.71701	-2.27897	9.64E-37	7.86E-36
CYP26B1	-1.63103	3.269568	9.81E-37	7.99E-36
INF2	1.317443	7.222608	9.89E-37	8.06E-36
DTHD1	3.234349	0.310435	1.02E-36	8.31E-36
CTSZ	1.283074	8.320324	1.05E-36	8.54E-36
TPD52L1	-1.71181	4.427353	1.05E-36	8.55E-36
ANO1	1.847798	5.659772	1.07E-36	8.70E-36
OSMR	1.530954	7.456181	1.12E-36	9.12E-36
SPC25	1.867163	1.042595	1.19E-36	9.68E-36
PRRT2	2.438203	1.895933	1.21E-36	9.81E-36
CLECL1	2.307479	-0.12741	1.28E-36	1.04E-35
COL4A2	1.54299	10.17421	1.29E-36	1.05E-35
CFAP221	-1.40005	3.386038	1.32E-36	1.07E-35
COL6A2	1.984827	8.80399	1.39E-36	1.12E-35
CD7	3.091835	3.315599	1.55E-36	1.25E-35
CLCN1	-1.82492	-2.20209	1.59E-36	1.28E-35
SEZ6L2	2.309139	6.265758	1.70E-36	1.37E-35
SOSTDC1	-2.89643	3.2081	1.76E-36	1.42E-35
ZNF205	1.057928	4.075002	1.78E-36	1.44E-35
ATF7-NPF	2.325787	0.864029	1.96E-36	1.57E-35
NPTX2	5.056974	7.519825	1.97E-36	1.59E-35
SLC9A9	1.818921	4.575854	2.04E-36	1.64E-35
ABTB2	-1.5971	4.230833	2.44E-36	1.95E-35
KCTD11	1.001494	5.00971	2.50E-36	2.00E-35
FSCN1	1.564642	6.52973	2.52E-36	2.02E-35
IL32	1.996287	7.97238	2.54E-36	2.04E-35
STMN3	2.166459	6.147263	2.56E-36	2.05E-35
RBM11	-2.52583	-0.54317	2.64E-36	2.12E-35
LOX	3.446303	8.320516	2.87E-36	2.29E-35
JAML	1.691493	4.151425	2.95E-36	2.35E-35
SLC7A14	-2.95341	-2.24184	2.98E-36	2.38E-35
DUSP10	1.403235	3.154561	3.10E-36	2.47E-35
FANCA	1.658541	2.530641	3.44E-36	2.74E-35
RPRM	-3.27893	-1.1049	3.58E-36	2.85E-35
CHAC1	-1.91902	1.666437	3.61E-36	2.87E-35
IDH2	-1.07941	7.649777	3.85E-36	3.06E-35
CD14	1.762059	6.777261	3.93E-36	3.12E-35
HAMP	3.77318	0.652193	3.97E-36	3.15E-35
PDGFRA	-2.3601	4.378978	4.01E-36	3.18E-35
PPIL6	-1.0156	2.255454	4.02E-36	3.19E-35
CD5	2.123039	2.982416	4.04E-36	3.20E-35
NLGN1	2.043139	5.040821	4.05E-36	3.21E-35
XCL2	2.56974	0.054564	4.19E-36	3.32E-35
SKA1	2.151702	0.644791	4.24E-36	3.35E-35
MYH15	2.398815	0.410359	4.25E-36	3.36E-35
GRAP2	1.701223	1.922338	4.29E-36	3.39E-35
GRB7	-1.21277	4.380117	4.46E-36	3.52E-35
ACROH2	-1.2121	3.530082	4.57E-36	3.60E-35
DDX39A	1.028221	5.430525	4.60E-36	3.62E-35
TNFAIP3	1.296954	6.144325	4.74E-36	3.73E-35
CADM1	-1.34977	5.693871	4.89E-36	3.85E-35
AGER	2.095397	2.575096	5.18E-36	4.07E-35
POU2F3	-2.11037	-0.26671	5.36E-36	4.21E-35
KIF20A	2.381353	2.811788	5.47E-36	4.29E-35
AGRP	-1.82405	-2.46373	5.53E-36	4.34E-35
ASAP2	-1.1596	5.017103	5.55E-36	4.35E-35
APOBR	1.699293	3.625494	5.57E-36	4.36E-35
CCL23	-1.46249	-1.29677	5.70E-36	4.47E-35
MKI67	2.24853	4.426727	5.70E-36	4.47E-35
CTLA4	2.964413	0.770109	5.90E-36	4.62E-35
TMEM255	1.526746	4.218656	5.93E-36	4.64E-35
C21orf62	-2.22194	2.8441	5.94E-36	4.65E-35
HLA-DOB	2.827324	4.959929	6.00E-36	4.69E-35
TMEM25	-1.21408	4.372831	6.19E-36	4.83E-35
SIX1	2.473375	1.203523	6.32E-36	4.93E-35
KIR2DL3	2.52072	-2.09492	6.88E-36	5.36E-35
LINGO1	1.968273	4.172552	6.98E-36	5.44E-35
CATSPER	2.002462	-0.64937	7.09E-36	5.52E-35
SBK1	-1.44793	1.655515	7.17E-36	5.58E-35
CDH4	4.087967	4.799613	7.68E-36	5.97E-35
SCGN	4.706092	5.266988	8.35E-36	6.48E-35
PHKG1	1.966149	0.331049	8.43E-36	6.54E-35
NPIPB4	2.001006	1.558149	8.56E-36	6.64E-35
NFKBID	2.195454	2.985358	8.66E-36	6.71E-35
ATP4B	-2.70768	-2.2436	9.34E-36	7.23E-35
ZNF692	1.830948	4.263676	9.66E-36	7.47E-35

OPA3	0.6218435	6.93E-59
PANK2	0.5128808	1.99E-37
GZF1	0.50781447	1.31E-36
PSMF1	0.6140699	4.52E-57
MKKS	0.5839415	1.71E-50
DSTN	0.50689434	1.84E-36
HNRNPR	0.64568046	8.80E-65
MAX	0.51074024	4.43E-37
ARMCX5	0.58627301	5.60E-51
AMOT	0.70150004	6.89E-81
HECTD3	0.55671971	4.09E-45
TUBGCP3	0.64199461	7.77E-64
UBA2	0.54365325	1.05E-42
THRA	0.65511541	2.90E-67
SBDS	0.57861662	2.11E-49
BECN1	0.63210147	2.33E-61
TRAP1	0.64510691	1.24E-64
NSRP1	0.57722571	4.04E-49
DNAJC8	0.66814972	7.63E-71
ZNF384	0.51629798	5.49E-38
KTN1	0.56770247	3.18E-47
TRMT5	0.57036803	9.50E-48
SGPP1	0.62154149	8.17E-59
RHOT1	0.63296471	1.43E-61
NUP214	0.60973766	4.41E-56
HNRNPH2	0.68816865	1.04E-76
ARMCX1	0.52782602	6.36E-40
TIMM8A	0.52243345	5.23E-39
CANX	0.61138409	1.86E-56
IPPK	0.58216435	3.98E-50
ZNF484	0.52593779	1.34E-39
PPCS	0.58977847	1.03E-51
MASP1	0.53898884	7.20E-42
RAP1B	0.55345375	1.68E-44
RAB3IP	0.6626549	2.59E-69
PTPRB	0.52258841	4.92E-39
PIN1	0.52874163	4.43E-40
EMC1	0.61289129	8.43E-57
UBR4	0.51511635	8.58E-38
HP1BP3	0.52829627	5.29E-40
SLC35E1	0.63961553	3.12E-63
EPS15L1	0.60586799	3.28E-55
SMARCA4	0.51800471	2.87E-38
TICAM1	0.57626861	6.31E-49
METTL16	0.56735347	3.72E-47
VIL1	0.50364699	6.02E-36
AAMP	0.67005574	2.21E-71
TNFRSF19	0.61365439	5.63E-57
RNF6	0.64783896	2.42E-65
ECHS1	0.52318808	3.90E-39
HIP1	0.53574696	2.69E-41
FGL2	0.50607117	2.48E-36
PEX1	0.53511309	3.48E-41
CASD1	0.51057541	4.71E-37
PAICS	0.55209304	3.00E-44
KDR	0.59580223	5.32E-53
PPAT	0.60912013	6.09E-56
ADM2	0.55631749	4.87E-45
YWHAH	0.64479202	1.49E-64
A4GALT	0.5010645	1.53E-35
EMC4	0.54670026	2.95E-43
POT1	0.54298304	1.39E-42
PODXL	0.53441032	4.62E-41
MKLN1	0.51648384	5.11E-38
DNAJB9	0.63716806	1.29E-62
NDUFA5	0.539697	5.39E-42
MTX2	0.5943194	1.11E-52
OSGEPL1	0.5207643	9.96E-39
HAT1	0.71027097	9.12E-84
HERC2	0.50774451	1.34E-36
SNRPN	0.53492354	3.76E-41
EIF2AK4	0.54672504	2.92E-43
CCDC32	0.5320443	1.19E-40
INO80	0.60062679	4.75E-54
ICE2	0.54155662	2.51E-42
IVD	0.66470154	7.04E-70
CLN6	0.53042066	2.28E-40
ARPP19	0.63765642	9.71E-63
CALML4	0.50563574	2.91E-36
THAP10	0.61174574	1.54E-56
ANAPC13	0.56232807	3.50E-46
COPB1	0.66938381	3.42E-71
SUMF2	0.57627484	6.29E-49
SPCS3	0.52696216	8.94E-40
BBOX1	0.60602091	3.03E-55
DCTD	0.60807252	1.05E-55
FXR2	0.62357423	2.69E-59
KIF1C	0.5460114	3.94E-43
MPDU1	0.53142952	1.53E-40
CCNT1	0.53867324	8.19E-42
SLC44A2	0.50807589	1.19E-36
BCL2L2	0.54164991	2.41E-42
AJUBA	0.58233989	3.66E-50
DTD2	0.58492498	1.07E-50
HEATR5A	0.51946811	1.64E-38
SNX6	0.52118587	8.46E-39
EAPP	0.58328582	2.33E-50
DAD1	0.60502404	5.06E-55
EPB41L4A	0.61770542	6.50E-58
REEP5	0.56019797	8.96E-46
ITFG1	0.64397708	2.42E-64
SEC14L1	0.59391219	1.36E-52
ASH2L	0.71551956	1.53E-85
TTI2	0.63480801	4.99E-62
PUDP	0.56126857	5.59E-46
STARD8	0.579291	1.54E-49
GNL3L	0.53207551	1.18E-40
MOSPD2	0.54747358	2.13E-43
CDC16	0.54574457	4.40E-43
THEM6	0.50941268	7.25E-37
XPO7	0.62028908	1.61E-58
ACE2	0.61570648	1.89E-57
TULP4	0.5117215	3.07E-37
SNX9	0.67151918	8.47E-72
RTN4IP1	0.50736314	1.55E-36
QRSL1	0.57349548	2.27E-48
RSPH3	0.57508931	1.09E-48
ACTN4	0.52451375	2.33E-39
NDUFA10	0.61630146	1.38E-57
PGPEP1	0.57876702	1.97E-49
CRB3	0.56222625	3.66E-46
CAMSAP1	0.5941802	1.19E-52
ATXN10	0.72592508	3.52E-89
TUBGCP2	0.5141603	1.23E-37
EXOSC2	0.58211374	4.07E-50
POMT1	0.59364202	1.55E-52
EIF2S3	0.57300057	2.85E-48
LRRC47	0.66655068	2.15E-70
MED18	0.56688834	4.58E-47
ZNF317	0.56739021	3.65E-47
DKC1	0.50145428	1.33E-35
NOL11	0.71939938	7.05E-87
UBE4B	0.6272043	3.62E-60
HABP4	0.60534815	4.28E-55
SLC35D2	0.67238094	4.80E-72
PRRG1	0.50213433	1.04E-35
UBA1	0.56689997	4.56E-47
RGN	0.54221012	1.91E-42
PPIL4	0.54725783	2.33E-43
SYNE1	0.52152478	7.43E-39
LATS1	0.52064732	1.04E-38
AAR2	0.61743399	7.52E-58
ARHGEF9	0.59972231	7.49E-54
ATP6V1E1	0.61603795	1.59E-57
NFATC1	0.50415517	5.00E-36
CAP1	0.61722066	8.43E-58
PPT1	0.66241884	3.01E-69
RLIM	0.55740617	3.04E-45
ABCB7	0.55912701	1.43E-45
SH3BP5	0.57166554	5.26E-48
HACL1	0.51288288	1.99E-37
TBC1D5	0.54629627	3.49E-43
CAPN7	0.52620795	1.20E-39
RBSN	0.50573664	2.81E-36
GALNT15	0.50523832	3.37E-36
KIF3A	0.535134	3.45E-41
PSME3	0.59512625	7.44E-53
ACLY	0.67811651	1.05E-73
VPS25	0.53704927	1.59E-41
G6PC1	0.52446314	2.38E-39
DIAPH1	0.60488966	5.42E-55
NDFIP1	0.59594039	4.97E-53
EXOC4	0.57978486	1.22E-49
PPFIA1	0.59911687	1.02E-53
WDR44	0.54974657	8.16E-44
CHD1L	0.51831357	2.55E-38
PEX11B	0.77489141	7.35E-109
RAI2	0.51751735	3.45E-38
MCCC2	0.61192021	1.41E-56
ZNF304	0.52788844	6.21E-40
THAP1	0.6605283	9.94E-69
RHPN2	0.66630497	2.51E-70
C19orf12	0.57384905	1.93E-48
ACTR10	0.64681536	4.47E-65
TRIM21	0.52749758	7.24E-40
SPATA6	0.611638	1.63E-56
PPARG	0.55823342	2.12E-45
ARFIP2	0.5280909	5.73E-40
TRIM5	0.5806716	8.05E-50
TIMM10B	0.58572943	7.27E-51
IMMT	0.66300696	2.07E-69
MRPL35	0.63274441	1.62E-61
PRKAA1	0.59229374	3.01E-52
CLUH	0.51228616	2.49E-37
INPP5K	0.60296344	1.45E-54
RPA1	0.67735152	1.75E-73
UBE2G1	0.62350971	2.79E-59
TBC1D14	0.51270423	2.13E-37
TMEM128	0.58210218	4.09E-50
LANCL2	0.60191452	2.47E-54

SEMA3G	-1.61455	5.194236	9.72E-36	7.51E-35
PDGFD	1.862012	6.516789	1.01E-35	7.80E-35
SH2D1A	2.487422	2.23036	1.03E-35	7.96E-35
CD48	1.966887	4.48875	1.03E-35	7.98E-35
DIRAS3	-1.83154	1.679642	1.11E-35	8.55E-35
CCDC102I	1.455529	3.751463	1.16E-35	8.94E-35
TDRD5	-2.81702	-0.45421	1.22E-35	9.36E-35
PRR11	1.915838	2.57568	1.30E-35	9.98E-35
KIR2DL4	2.39826	-0.6684	1.33E-35	1.02E-34
TOX2	1.730052	3.200145	1.39E-35	1.07E-34
MEF2C	1.30429	6.49753	1.49E-35	1.15E-34
FUT3	-2.28248	1.505416	1.53E-35	1.18E-34
TMEM45A	3.077121	5.459255	1.54E-35	1.18E-34
DOCK6	1.190413	6.552641	1.68E-35	1.29E-34
POU2F2	1.960689	3.518945	1.79E-35	1.37E-34
PHLDA3	1.660886	5.785863	1.79E-35	1.37E-34
EN1-CDK	2.024977	0.674131	1.85E-35	1.41E-34
BSPRY	-2.07441	3.16675	1.87E-35	1.43E-34
DDX47	1.648626	1.109446	1.90E-35	1.45E-34
MTMR11	1.623623	5.759422	1.93E-35	1.48E-34
NMB	2.58645	4.627569	2.06E-35	1.57E-34
MAP3K14	1.384835	5.396136	2.09E-35	1.60E-34
LILRA1	1.540723	1.619878	2.11E-35	1.61E-34
C2orf92	2.059238	1.448779	2.23E-35	1.70E-34
CARMIL2	2.524377	1.819961	2.26E-35	1.72E-34
SEMA6B	1.638135	5.09508	2.30E-35	1.75E-34
HSPA6	2.085298	2.571812	2.33E-35	1.77E-34
GPRC5B	-1.1919	6.323308	2.41E-35	1.83E-34
IFI30	2.320026	1.125951	2.48E-35	1.88E-34
PPP1R3B	1.333392	6.885187	2.52E-35	1.91E-34
DOCK8	1.207304	6.591166	2.59E-35	1.96E-34
ITK	2.120531	3.144959	2.60E-35	1.98E-34
LRRC71	2.975422	-1.14906	2.61E-35	1.98E-34
AIM2	3.06417	1.237263	2.74E-35	2.08E-34
ZNF83	1.493139	6.218476	2.87E-35	2.17E-34
TTC24	3.69648	-1.25194	2.91E-35	2.20E-34
FOXA3	-2.8779	-0.13023	2.96E-35	2.24E-34
PODXL	-1.34251	8.578017	3.02E-35	2.28E-34
PLEKHG4	2.139757	4.020185	3.03E-35	2.29E-34
PMCH	4.571511	-1.98999	3.06E-35	2.31E-34
CD70	4.314623	5.671014	3.23E-35	2.44E-34
PTPN3	-1.24237	6.03236	3.23E-35	2.44E-34
HAO2	-2.46131	5.149357	3.34E-35	2.52E-34
PLAAT3	1.071448	6.959139	3.37E-35	2.54E-34
METTL26	1.591784	6.114541	3.46E-35	2.61E-34
OTOF	2.576084	-1.3669	3.76E-35	2.83E-34
JHY	-1.02879	2.802858	3.90E-35	2.93E-34
ABLIM3	1.997438	6.892275	3.96E-35	2.97E-34
DEGS1	1.278972	7.109937	4.05E-35	3.04E-34
UBE2C	2.966899	2.821709	4.07E-35	3.05E-34
SLC2A3	2.043662	6.996541	4.76E-35	3.57E-34
SHISA2	-2.33292	1.745033	5.02E-35	3.76E-34
TGFBR3L	-1.90633	-1.57389	5.13E-35	3.84E-34
TAZ	1.114135	4.579393	5.15E-35	3.86E-34
IL4I1	2.130352	3.971685	5.23E-35	3.91E-34
FBLN5	-1.72129	6.288587	5.45E-35	4.07E-34
FADS3	1.344267	4.974489	5.52E-35	4.11E-34
NFKB2	1.231518	6.214346	5.54E-35	4.13E-34
USP2	-1.8318	5.150271	5.68E-35	4.23E-34
DNAJB13	3.74132	1.875203	5.70E-35	4.24E-34
CAPN5	-1.06269	5.07515	5.95E-35	4.43E-34
WDR90	1.347508	4.810187	6.01E-35	4.47E-34
CR1	-1.98103	2.148901	6.23E-35	4.63E-34
ADH1B	-2.61151	5.094167	6.57E-35	4.88E-34
PIF1	2.277009	0.455604	6.66E-35	4.94E-34
XAF1	1.982199	3.843501	6.85E-35	5.08E-34
STYK1	-1.74795	-0.51621	7.30E-35	5.40E-34
XRCC3	1.466675	2.784472	7.43E-35	5.50E-34
SLFNL1	2.211154	-0.39742	7.53E-35	5.57E-34
CENPA	2.472983	0.369184	7.62E-35	5.63E-34
TRAF3IP3	1.870144	3.219243	8.11E-35	5.98E-34
NGF	2.13823	2.85265	8.11E-35	5.98E-34
OLFM4	-3.72972	1.895576	8.56E-35	6.31E-34
TREH	-2.30296	2.198535	8.87E-35	6.54E-34
RFX2	1.498546	3.778377	8.89E-35	6.55E-34
GIMAP2	1.293351	3.933737	9.36E-35	6.89E-34
TMEM262	1.569417	0.227737	1.06E-34	7.84E-34
ZNF726	-1.44673	0.449502	1.08E-34	7.96E-34
SNAI3	1.315801	0.78538	1.09E-34	8.00E-34
ARHGAP3	2.121392	3.471553	1.12E-34	8.22E-34
PKD1L1	1.853448	0.596285	1.22E-34	8.93E-34
PRSS8	-1.69008	5.70716	1.22E-34	8.94E-34
DNAJC6	-1.42165	3.521071	1.22E-34	8.96E-34
RBM46	3.587926	-1.14996	1.28E-34	9.41E-34
ARL4A	1.136159	4.165076	1.29E-34	9.44E-34
LRRN2	-2.31856	3.418685	1.42E-34	1.04E-33
MEF2B	2.016885	-1.01598	1.44E-34	1.05E-33
GDPD5	1.399099	3.592867	1.53E-34	1.12E-33
PDCD1	3.332169	2.306224	1.53E-34	1.12E-33
PFKFB3	-1.257	8.033454	1.57E-34	1.15E-33
IL17RE	-1.44652	2.41695	1.66E-34	1.21E-33
AGBL3	1.551549	3.400514	1.70E-34	1.24E-33
DENND1E	-1.05143	4.400838	1.72E-34	1.25E-33
LHX1	-3.49321	1.545879	1.74E-34	1.27E-33
TMC4	-2.07673	4.739789	1.81E-34	1.32E-33
SIPA1L2	1.164826	5.664345	1.85E-34	1.35E-33
INSR	1.281419	8.508524	1.98E-34	1.44E-33
ABI3	1.077897	4.727424	2.01E-34	1.46E-33
DLK2	2.349968	1.051712	2.18E-34	1.58E-33
KIAA1210	-2.13611	-1.78001	2.25E-34	1.63E-33
GSAP	1.207511	5.192898	2.25E-34	1.63E-33
SCG2	4.046287	3.035158	2.31E-34	1.68E-33
NDRG1	1.352864	11.08606	2.45E-34	1.78E-33
GABRG3	-2.68613	-2.24873	2.52E-34	1.83E-33
SH2B2	1.309287	2.647304	2.61E-34	1.89E-33
CPVL	-1.38839	6.519153	2.82E-34	2.04E-33
KNTC1	1.203454	4.083593	3.00E-34	2.16E-33
STARD9	1.529664	4.343229	3.20E-34	2.31E-33
RGCC	1.607318	6.34579	3.60E-34	2.60E-33
AUH	-1.1033	4.82477	3.74E-34	2.69E-33
MMP16	2.882094	2.638106	3.83E-34	2.76E-33
MYL4	1.596783	-0.31384	3.84E-34	2.77E-33
PRSS22	-3.23252	0.308853	4.05E-34	2.92E-33
ANXA1	1.347231	7.498814	4.22E-34	3.04E-33
BUB1	2.158979	2.550493	4.24E-34	3.04E-33
ZNF276	1.311716	4.106012	4.35E-34	3.12E-33
RAB33A	2.163137	0.865036	4.35E-34	3.12E-33
CNTNAP1	1.655033	3.728579	4.35E-34	3.12E-33
ZNF831	2.268364	1.585997	4.59E-34	3.29E-33
LY6G5B	2.381282	1.360028	4.85E-34	3.47E-33
SYNE4	-2.20514	0.940835	4.99E-34	3.57E-33
HDAC10	1.532169	3.01832	5.04E-34	3.61E-33
TMPRSS9	2.710705	-0.72149	5.07E-34	3.62E-33
HCST	2.023077	2.501407	5.18E-34	3.71E-33
C16orf54	1.786982	2.266796	5.58E-34	3.98E-33
PANK1	-1.448	4.770535	5.62E-34	4.01E-33
KIAA1549I	-2.06429	1.303706	5.69E-34	4.06E-33
APOO	-1.1619	3.7014	5.75E-34	4.10E-33
TSHR	2.384808	0.293926	6.10E-34	4.34E-33
LRP1B	-2.55811	0.100631	6.23E-34	4.43E-33
CEP43	1.384945	4.896721	6.52E-34	4.64E-33
CNIH2	1.962018	-0.81995	6.70E-34	4.76E-33
NFKBIE	1.227641	5.209081	6.89E-34	4.89E-33
P4HA3	2.848764	2.945434	7.22E-34	5.13E-33
ERFL	2.788897	-2.53361	7.75E-34	5.49E-33
C20orf202	1.347217	0.121021	8.55E-34	6.06E-33
NPIPB5	2.953055	2.200043	8.72E-34	6.18E-33
ADSS1	2.485605	6.181686	8.87E-34	6.28E-33
ADAMTS2	1.939405	4.878672	9.15E-34	6.47E-33
ADCY7	1.940809	3.240304	9.96E-34	7.04E-33
TCEAL2	-3.11524	1.311188	9.99E-34	7.06E-33
UNC5A	3.427958	1.243599	1.03E-33	7.24E-33
PRR16	1.764263	1.700023	1.07E-33	7.51E-33
PIK3C2G	-3.83662	1.766783	1.08E-33	7.60E-33
HDAC11	-1.03764	4.705073	1.11E-33	7.80E-33
GPD1L	-1.4672	5.31204	1.18E-33	8.31E-33
LY9	2.110997	1.649872	1.26E-33	8.85E-33
LRRC19	-1.98342	4.457088	1.32E-33	9.26E-33
RUNX1	1.786471	5.987028	1.37E-33	9.64E-33
CFAP20DC	1.670645	2.247079	1.39E-33	9.72E-33
MND1	1.55419	0.06349	1.40E-33	9.80E-33
GAPT	2.108286	2.60198	1.42E-33	9.95E-33
ATP8B3	3.411843	3.459536	1.43E-33	1.01E-32
SLC39A4	-1.5262	3.99871	1.47E-33	1.03E-32
NCR3	1.843466	-0.25476	1.51E-33	1.06E-32
TMIGD2	2.173417	-0.87255	1.60E-33	1.12E-32
MAPRE2	1.024742	6.937228	1.74E-33	1.21E-32
PAQR6	2.615049	1.683063	1.93E-33	1.35E-32
GBP1	1.833589	6.601281	2.10E-33	1.46E-32
VENTX	1.936454	0.830761	2.19E-33	1.53E-32
MSH5	2.201824	2.081053	2.28E-33	1.59E-32
CDKN3	1.913313	1.369838	2.39E-33	1.66E-32
PLOD1	1.341417	8.139441	2.49E-33	1.73E-32
ECRG4	-2.03554	2.498059	2.54E-33	1.77E-32
CCDC141	2.133535	1.058207	2.59E-33	1.80E-32
ADGRE5	1.129917	6.51714	2.71E-33	1.88E-32
CSF1R	1.623884	6.754849	3.02E-33	2.09E-32
SHC3	-1.53672	1.340039	3.02E-33	2.09E-32
ADGRE2	1.845032	3.548531	3.03E-33	2.10E-32
PTPRC	1.857344	6.345569	3.03E-33	2.10E-32
PLPP4	-2.46988	0.969041	3.24E-33	2.24E-32
ADAM8	2.145874	3.475147	3.25E-33	2.25E-32
NLGN3	-1.26588	0.304043	3.42E-33	2.36E-32
KCNJ2	1.870795	4.320107	3.42E-33	2.36E-32
KIR2DL1	2.406428	-2.13728	3.69E-33	2.55E-32
TLR3	1.525127	6.610606	3.79E-33	2.61E-32
RRM2	2.190463	3.705558	3.92E-33	2.70E-32
ATP6V0E2	-1.27407	5.111408	3.97E-33	2.73E-32
CENATAC	1.760708	3.925895	4.06E-33	2.79E-32
RPL36A	1.275535	6.059363	4.20E-33	2.89E-32
SBNO2	1.018445	6.006163	4.26E-33	2.93E-32
NOTCH4	1.582516	6.901907	4.38E-33	3.01E-32
ANKHD1	1.026412	2.943589	4.42E-33	3.04E-32
IL27	2.389702	-2.19738	4.48E-33	3.08E-32
NPIPB3	2.168117	0.953377	4.55E-33	3.12E-32
SACS	1.323576	4.81139	4.76E-33	3.27E-32
LCAT	1.805328	3.509787	4.78E-33	3.28E-32
MSH4	2.902439	-0.98105	4.88E-33	3.35E-32
TEX15	5.774433	2.617798	5.39E-33	3.69E-32
THBS3	1.250001	4.841017	5.50E-33	3.77E-32

DDC	0.65611503	1.56E-67
GRSF1	0.56501334	1.06E-46
ENAM	0.55682173	3.91E-45
ANKRD17	0.57086858	7.56E-48
UTP3	0.60516899	4.70E-55
WBP2	0.57601938	7.08E-49
H3-3B	0.53196138	1.23E-40
RIDA	0.5743461	1.54E-48
VPS13B	0.5562477	5.02E-45
SDF2	0.62703474	3.98E-60
FLOT2	0.61893395	3.36E-58
ERAL1	0.61211938	1.27E-56
NIP7	0.55708705	3.49E-45
TERF2	0.69748696	1.32E-79
VPS4A	0.50356752	6.20E-36
ANKEF1	0.50669749	1.97E-36
BTBD3	0.52415405	2.68E-39
POLR3F	0.5443343	7.93E-43
RIN2	0.58463213	1.23E-50
PTPRA	0.60857975	8.06E-56
DAP3	0.52108808	8.79E-39
MMACHC	0.52184469	6.56E-39
TOE1	0.51334159	1.67E-37
CTNNBL1	0.52038239	1.15E-38
SERINC3	0.5282808	5.32E-40
PPP1R3D	0.62254929	4.71E-59
DMGDH	0.6817285	9.02E-75
BHMT2	0.58320941	2.42E-50
AP3B1	0.64587093	7.86E-65
ZBED3	0.51131757	3.57E-37
PATJ	0.65735693	7.24E-68
CPLANE2	0.60603537	3.01E-55
DCTN4	0.59999492	6.53E-54
XPO4	0.57495607	1.16E-48
SCO1	0.62751616	3.05E-60
PIK3C2B	0.54475488	6.66E-43
DSTYK	0.64582226	8.09E-65
SLC41A1	0.74589528	1.17E-96
COG6	0.66458936	7.56E-70
SPART	0.71133002	4.03E-84
RFXAP	0.52102586	9.00E-39
GPALPP1	0.5751872	1.04E-48
KL	0.65986515	1.51E-68
RFC3	0.57768209	3.27E-49
FAM104A	0.55085166	5.10E-44
CNDP2	0.65843716	3.69E-68
RTN3	0.65369144	6.96E-67
CEP20	0.66228938	3.27E-69
GIMAP6	0.55622344	5.08E-45
MKRN1	0.52752274	7.17E-40
C12orf29	0.53018017	2.51E-40
TMEM254	0.5428902	1.44E-42
KRAS	0.60292715	1.48E-54
IPO8	0.60252852	1.81E-54
LARS1	0.56913026	1.67E-47
IMPA1	0.52039922	1.15E-38
SWAP70	0.61262399	9.71E-57
SBF2	0.59359033	1.59E-52
RRAS2	0.59820403	1.61E-53
HSD17B4	0.65038755	5.21E-66
SARAF	0.71860489	1.33E-86
DPF2	0.58894453	1.54E-51
DGLUCY	0.50086581	1.65E-35
NUMB	0.67593655	4.53E-73
COX16	0.54389191	9.53E-43
EIF2S1	0.6202134	1.68E-58
ELP3	0.59854498	1.35E-53
MBD2	0.58678185	4.38E-51
IER3IP1	0.57893332	1.82E-49
MRPS36	0.50547527	3.09E-36
IRAK2	0.52326999	3.78E-39
CAMK1	0.56226548	3.60E-46
THUMPD3	0.53880534	7.76E-42
DPH6	0.51819674	2.66E-38
EMC7	0.51317214	1.78E-37
VAV3	0.55801368	2.33E-45
LAMTOR5	0.56369038	1.91E-46
CEPT1	0.59082879	6.16E-52
SPIRE1	0.55054877	5.80E-44
ARF3	0.64585864	7.91E-65
YWHAQ	0.59197936	3.51E-52
KIDINS220	0.66673923	1.90E-70
ROCK2	0.53927948	6.39E-42
CMPK2	0.51705274	4.12E-38
LDHA	0.53891809	7.41E-42
IL6ST	0.57615753	6.65E-49
CDC73	0.70932957	1.88E-83
TIMM17A	0.6698377	2.55E-71
NARS1	0.61026248	3.35E-56
RELCH	0.51050155	4.84E-37
FBH1	0.56784505	2.98E-47
ECHDC3	0.63767124	9.63E-63
TMEM241	0.57721073	4.07E-49
CABLES1	0.67168879	7.57E-72
PUM1	0.67362093	2.12E-72
HOOK1	0.67894572	5.99E-74
BTF3L4	0.55510507	8.23E-45
TUT4	0.512381	2.40E-37
PRPF38A	0.63210371	2.32E-61
DSC2	0.52916314	3.75E-40
RNF138	0.5219564	6.29E-39
ELP2	0.53404384	5.35E-41
CLOCK	0.59184235	3.75E-52
CLDN10	0.63384913	8.62E-62
UBAC2	0.51594158	6.28E-38
TPP2	0.55944064	1.25E-45
ARHGAP32	0.64820452	1.95E-65
STT3A	0.61893104	3.36E-58
ETS1	0.56455173	1.30E-46
TMED7	0.5946046	9.64E-53
APC	0.63209018	2.34E-61
OSTF1	0.52784731	6.31E-40
UBQLN1	0.59999909	6.52E-54
NAA35	0.58335362	2.26E-50
CTSL	0.62713346	3.77E-60
AGTPBP1	0.51613386	5.84E-38
ISCA1	0.61332619	6.70E-57
TAOK3	0.55480855	9.36E-45
USP30	0.66303822	2.03E-69
HNF1A	0.6322045	2.19E-61
FBXO21	0.57739054	3.75E-49
CD36	0.54014123	4.49E-42
UGT2A3	0.57329782	2.49E-48
PNPLA8	0.56016103	9.10E-46
MTO1	0.69142305	1.04E-77
ANKRD6	0.57740369	3.72E-49
SYNCRIP	0.63048427	5.79E-61
SNX14	0.56177477	4.47E-46
ORC3	0.55270858	2.31E-44
LCA5	0.50438172	4.61E-36
NAT10	0.60609612	2.91E-55
PRRG4	0.52915299	3.77E-40
CAPRIN1	0.6599791	1.40E-68
DNAJC14	0.58184295	4.63E-50
TFCP2	0.5726397	3.37E-48
ZC3H10	0.62792144	2.43E-60
HNRNPA1	0.50804186	1.20E-36
ACVR1B	0.50576186	2.78E-36
OS9	0.55673972	4.06E-45
MAP7	0.72352755	2.51E-88
CD164	0.69687797	2.06E-79
AFG1L	0.58891304	1.57E-51
REPS1	0.55927233	1.34E-45
CCT7	0.61029571	3.30E-56
RAB11FIP5	0.64129762	1.17E-63
SMYD5	0.50495542	3.74E-36
DYSF	0.61872398	3.76E-58
GNS	0.54981968	7.91E-44
DYNC1LI2	0.58366913	1.95E-50
URB2	0.52927968	3.58E-40
EGLN1	0.75096538	1.14E-98
COG2	0.70848598	3.59E-83
ABCB10	0.73902765	5.22E-94
NTPCR	0.54394428	9.33E-43
TAF5L	0.60081438	4.32E-54
STX6	0.55279917	2.22E-44
RNASEL	0.66000442	1.38E-68
DHX9	0.7560219	1.00E-100
KIAA1614	0.57129493	6.23E-48
CEP350	0.53051893	2.19E-40
PIGC	0.63334984	1.15E-61
LAMC1	0.53901364	7.13E-42
RC3H1	0.55536727	7.35E-45
MRPL44	0.75740055	2.71E-101
USP37	0.56110705	6.00E-46
TMBIM1	0.58997863	9.33E-52
CYP27A1	0.52638359	1.12E-39
EIF4E2	0.63816295	7.25E-63
CAB39	0.61650309	1.24E-57
REV1	0.50846142	1.03E-36
MFSD9	0.60394815	8.78E-55
TMEM127	0.67006348	2.20E-71
TGFBRAP1	0.60485382	5.52E-55
GCC2	0.52230358	5.50E-39
MRPS9	0.65235	1.58E-66
EPC2	0.60068921	4.60E-54
APPL2	0.66618137	2.72E-70
PWP1	0.6106606	2.72E-56
DRAM1	0.63060079	5.42E-61
WASHC4	0.5581445	2.20E-45
SLC41A2	0.54646353	3.26E-43
VPS36	0.67294531	3.31E-72
TBC1D4	0.52341618	3.57E-39
LRCH1	0.62359125	2.66E-59
SUCLA2	0.74777027	2.14E-97
RCBTB1	0.64626937	6.20E-65
MED4	0.56601661	6.77E-47
COG3	0.60255157	1.79E-54
ITM2B	0.61062866	2.77E-56
NUDT15	0.61218417	1.22E-56

RASL10A	2.044835	-0.34583	5.57E-33	3.81E-32
FHOD3	-1.22245	5.015055	5.60E-33	3.83E-32
NHLH1	2.052344	-1.90274	5.66E-33	3.87E-32
PRODH2	-2.24038	4.948337	5.66E-33	3.87E-32
OAS2	1.329506	5.78078	5.80E-33	3.96E-32
ADAM11	2.09793	0.05502	5.85E-33	3.99E-32
ANKRD37	1.334391	3.532949	6.10E-33	4.16E-32
LYZ	2.463635	7.486589	6.48E-33	4.42E-32
RNASE6	1.549349	4.298074	6.56E-33	4.47E-32
OXNAD1	-1.10888	3.422441	6.65E-33	4.53E-32
TLN2	-1.12373	6.66372	6.75E-33	4.59E-32
CLSTN2	-2.27637	4.282976	6.91E-33	4.70E-32
LMNB1	1.35707	4.029204	7.13E-33	4.85E-32
TUBB6	1.17532	6.043163	7.56E-33	5.14E-32
LRRC36	1.845735	1.213429	7.78E-33	5.28E-32
GDF7	-1.49842	3.223891	7.90E-33	5.37E-32
IGSF5	-2.23207	-1.94566	8.05E-33	5.46E-32
ALDH5A1	-1.06133	5.299767	8.17E-33	5.54E-32
ASPM	2.291811	2.672451	8.34E-33	5.65E-32
OR10Q1	4.903827	-2.92566	8.37E-33	5.67E-32
FBXO39	2.293764	-1.34092	8.63E-33	5.84E-32
TNS4	-2.2583	0.662046	9.47E-33	6.40E-32
XRCC2	1.621381	0.940647	9.71E-33	6.55E-32
TUBA1B	1.096337	7.628872	1.02E-32	6.90E-32
CHST15	1.689531	6.577135	1.03E-32	6.94E-32
SEC16B	1.731765	-0.33828	1.06E-32	7.17E-32
TIMP1	2.075338	8.721515	1.14E-32	7.71E-32
DCSTAMI	3.180031	-0.46805	1.15E-32	7.77E-32
KCNE1	-1.80325	-0.97699	1.16E-32	7.83E-32
TICRR	1.991304	0.928804	1.23E-32	8.31E-32
LMNTD2	2.00128	2.688426	1.28E-32	8.61E-32
SYT1	-2.22714	1.050847	1.29E-32	8.69E-32
ABCB1	-1.68226	5.669158	1.31E-32	8.81E-32
COL27A1	1.779035	6.193016	1.44E-32	9.67E-32
LRRIQ3	-1.27636	0.51816	1.46E-32	9.77E-32
PMFBP1	2.110485	-0.00548	1.55E-32	1.04E-31
IL7	1.193621	2.348711	1.59E-32	1.06E-31
ZGRF1	1.285065	1.719759	1.60E-32	1.07E-31
MYOZ1	-1.42503	0.811771	1.66E-32	1.11E-31
LAPTM4E	-1.0347	6.68958	1.67E-32	1.12E-31
KCNMA1	2.313105	5.912775	1.74E-32	1.16E-31
CREB5	1.506845	5.371735	1.78E-32	1.19E-31
KCNH3	-1.97513	0.08868	1.79E-32	1.19E-31
ZNF337	1.304769	1.945257	1.81E-32	1.21E-31
TAS2R5	1.931467	-0.49017	1.85E-32	1.23E-31
ALOX12	1.230139	1.294171	1.88E-32	1.26E-31
CSF2RB	1.363721	4.006354	1.90E-32	1.26E-31
CEL	-2.67505	1.491058	1.90E-32	1.26E-31
CCNB2	1.76118	2.290144	1.99E-32	1.32E-31
KLK5	-3.50151	-2.22225	2.04E-32	1.35E-31
PADI1	5.81296	3.891133	2.05E-32	1.36E-31
FAT2	-1.7942	1.046205	2.08E-32	1.38E-31
CDK3	2.407979	-0.73421	2.28E-32	1.51E-31
RPA4	2.741953	-2.67505	2.37E-32	1.57E-31
HASPIN	1.734613	-0.42365	2.57E-32	1.70E-31
KPNA7	3.224863	-2.50477	2.69E-32	1.78E-31
SGK2	-1.51917	4.687456	2.69E-32	1.78E-31
NUF2	1.998041	1.19843	2.81E-32	1.86E-31
HEATR9	3.334686	-2.03751	2.87E-32	1.89E-31
BNIPL	-1.59953	0.012617	2.91E-32	1.92E-31
SLC8A1	-1.3409	5.453996	3.07E-32	2.03E-31
PCED1A	1.014439	5.430681	3.17E-32	2.09E-31
ATAD5	1.276019	1.996268	3.23E-32	2.13E-31
GUCA2B	5.891856	0.968034	3.26E-32	2.14E-31
KIAA0319	2.835825	1.254122	3.60E-32	2.36E-31
CDH1	-1.45354	7.14792	3.77E-32	2.48E-31
COL8A1	1.997993	6.73189	4.28E-32	2.81E-31
PXDN	1.691913	7.070335	4.43E-32	2.90E-31
SHISAL2A	1.804793	0.037357	4.48E-32	2.93E-31
NKAIN1	3.358814	1.306911	4.98E-32	3.26E-31
FAM72A	1.45912	-1.26227	5.02E-32	3.29E-31
POGLUT2	1.239986	4.023094	5.06E-32	3.31E-31
HOXD3	-1.09534	1.524645	5.29E-32	3.46E-31
IGFALS	-1.70535	-0.82024	5.39E-32	3.53E-31
GPR27	-1.82528	1.686245	5.85E-32	3.83E-31
RHOBTB3	-1.55835	5.718377	6.18E-32	4.04E-31
BCL2L15	-1.92017	0.581983	6.22E-32	4.07E-31
CENPI	1.621982	0.612454	6.25E-32	4.08E-31
SLC43A2	-1.06312	6.745438	6.28E-32	4.10E-31
DCDC2	-1.65532	5.538945	6.36E-32	4.15E-31
NKPD1	2.932381	-1.67114	6.65E-32	4.33E-31
ALOX5	2.104805	5.408337	6.85E-32	4.46E-31
SYNGR1	-1.34131	4.524627	7.06E-32	4.59E-31
GATA5	-2.16185	-0.43245	7.14E-32	4.64E-31
PRKX	-1.00543	5.346558	7.57E-32	4.92E-31
P11-407P1	-1.76583	-2.21668	7.60E-32	4.94E-31
GSTO2	-1.78039	3.424143	7.65E-32	4.97E-31
CTHRC1	3.162541	4.734777	8.54E-32	5.54E-31
VAMP5	1.218621	5.701694	8.62E-32	5.59E-31
LOXL3	1.609395	3.046194	8.69E-32	5.64E-31
TMEM145	4.306498	1.051915	8.80E-32	5.70E-31
FCHSD1	1.314869	4.010825	8.85E-32	5.74E-31
HSD17B3	3.051316	0.461976	8.89E-32	5.76E-31
CLSPN	1.642302	1.131597	8.97E-32	5.81E-31
APBB3	1.490762	4.274498	9.05E-32	5.86E-31
RSPO1	-2.96335	-2.38145	9.52E-32	6.16E-31
PKD2L1	3.219902	-0.00179	9.81E-32	6.34E-31
GRHL1	-1.32411	1.939378	9.99E-32	6.45E-31
MYBL2	2.75101	2.715225	1.03E-31	6.68E-31
ZNF581	1.084782	4.291635	1.06E-31	6.84E-31
FZD1	1.285661	7.276642	1.08E-31	6.94E-31
PLK1	2.065729	2.433667	1.15E-31	7.40E-31
GASK1A	-1.48979	0.240086	1.15E-31	7.43E-31
CRIP1	1.899609	1.641152	1.23E-31	7.89E-31
PLOD2	1.780904	8.310067	1.27E-31	8.18E-31
RHGAP11	1.414467	2.779464	1.29E-31	8.27E-31
P2RY2	-1.40819	0.558835	1.33E-31	8.56E-31
TDRD6	1.892482	1.299647	1.51E-31	9.65E-31
DOC2B	-1.2858	4.001589	1.54E-31	9.89E-31
ATG9B	3.019816	2.376101	1.76E-31	1.13E-30
CPNE4	-2.58602	-0.83805	1.79E-31	1.15E-30
SLC25A4	-1.10948	6.126875	1.81E-31	1.15E-30
TLR7	2.102499	3.724491	1.84E-31	1.17E-30
PLB1	1.334796	2.278211	1.84E-31	1.18E-30
MAP9	-1.27023	4.132724	2.01E-31	1.28E-30
CKAP2L	1.994322	1.555998	2.11E-31	1.34E-30
FAM83B	-3.47696	0.930282	2.28E-31	1.45E-30
EMB	1.583761	4.527756	2.32E-31	1.47E-30
TFR2	4.078085	1.884156	2.35E-31	1.50E-30
EGFR	1.475598	8.38407	2.62E-31	1.66E-30
NRARP	1.646416	3.707048	2.76E-31	1.75E-30
ANKLE1	2.056548	-0.76293	2.76E-31	1.75E-30
FOXJ3	1.649882	7.34379	2.79E-31	1.77E-30
RBCK1	1.207451	6.947543	2.80E-31	1.77E-30
MSRA	-1.26133	5.336926	2.83E-31	1.79E-30
PC	-1.40271	5.193721	2.91E-31	1.84E-30
MEFV	1.938992	0.99453	2.91E-31	1.84E-30
CD36	2.192636	7.209212	2.98E-31	1.89E-30
MIOX	-2.29153	6.852736	3.08E-31	1.95E-30
ACAN	2.62961	5.433112	3.15E-31	1.99E-30
HTR6	5.029292	0.904602	3.21E-31	2.03E-30
ATP2A1	2.38546	0.84679	3.37E-31	2.12E-30
ANO7	1.155208	1.846889	3.42E-31	2.16E-30
MMP9	4.172499	4.943156	3.45E-31	2.17E-30
PTGER3	-2.08709	6.828858	3.46E-31	2.18E-30
PARD6A	-1.22338	2.260793	3.63E-31	2.29E-30
MTFR2	1.502836	0.195883	3.82E-31	2.41E-30
PMEPA1	1.618942	7.056427	3.94E-31	2.48E-30
CCNL2	1.816949	6.452155	4.40E-31	2.77E-30
CRYGS	2.363408	1.592281	4.49E-31	2.82E-30
RNASE2	2.443307	0.504237	4.54E-31	2.85E-30
ZNF354B	1.132486	3.322565	4.92E-31	3.09E-30
KIF18A	1.562688	1.37085	5.41E-31	3.39E-30
NCS1	-1.20957	4.823433	5.54E-31	3.47E-30
NPAP1	-2.57741	-2.25105	5.65E-31	3.54E-30
ADAM19	1.596139	4.110742	5.72E-31	3.59E-30
SEMA3F	1.152839	6.78987	5.85E-31	3.67E-30
TAS2R20	1.980982	-0.97732	5.93E-31	3.71E-30
PIWIL4	1.324379	1.883333	5.95E-31	3.73E-30
CDHR1	4.223948	4.759159	6.01E-31	3.76E-30
TMSB15B	1.542715	-1.69724	6.13E-31	3.83E-30
ARHGAP2	1.052427	5.813953	6.27E-31	3.92E-30
SCGB3A2	5.267378	-0.52672	6.46E-31	4.04E-30
RNASE10	2.275108	-2.41026	6.62E-31	4.13E-30
WDR49	-2.0099	-2.58336	6.74E-31	4.21E-30
RPS8	1.042299	9.902276	6.86E-31	4.28E-30
SYNM	1.086401	5.434677	7.19E-31	4.48E-30
ENHO	-1.53417	-1.51137	7.27E-31	4.53E-30
PPEF1	2.67217	-0.07848	7.53E-31	4.69E-30
NINJ2	1.753531	1.334032	7.65E-31	4.76E-30
SDC3	1.080977	6.836846	7.91E-31	4.92E-30
ITPKA	3.974466	1.529529	7.97E-31	4.96E-30
PHACTR1	-1.08525	4.220048	8.17E-31	5.08E-30
SELE	-1.82812	2.721078	8.84E-31	5.49E-30
PLXNC1	1.517095	4.971052	8.84E-31	5.49E-30
GCNT4	-1.58687	2.965182	8.87E-31	5.50E-30
XCL1	2.375251	0.355325	9.35E-31	5.80E-30
SCNN1D	2.408039	2.010335	9.54E-31	5.92E-30
AMT	-1.08573	4.301984	9.66E-31	5.99E-30
SHROOM	1.145404	4.169754	9.69E-31	6.00E-30
LPIN3	1.566896	5.375858	1.01E-30	6.28E-30
ALS2CL	-1.69631	4.083489	1.02E-30	6.29E-30
THSD7A	-1.62306	5.46806	1.11E-30	6.84E-30
HOXA7	-1.48348	1.81631	1.13E-30	6.96E-30
DCLK1	2.161884	4.849306	1.13E-30	6.99E-30
CHTF18	1.554365	2.919112	1.14E-30	7.02E-30
RAP1GAP	-1.23634	4.431581	1.15E-30	7.13E-30
BVES	-1.26812	2.091837	1.17E-30	7.23E-30
LSMEM1	1.87406	1.086305	1.18E-30	7.29E-30
SYPL2	-1.31563	3.452256	1.21E-30	7.44E-30
RGS14	1.381025	5.411218	1.22E-30	7.50E-30
NID1	1.384057	7.864996	1.22E-30	7.52E-30
RPL37	1.049114	9.21978	1.25E-30	7.67E-30
TMEM121	1.492698	0.290161	1.26E-30	7.78E-30
ADHFE1	-1.15628	3.126256	1.34E-30	8.23E-30
NCOA7	-1.13325	6.436506	1.36E-30	8.35E-30
NFATC2	1.177575	4.735901	1.37E-30	8.43E-30
FGFR2	-1.26087	5.406057	1.39E-30	8.55E-30
RPL18A	1.140307	8.081543	1.52E-30	9.29E-30
SPATA17	-1.1501	1.433605	1.53E-30	9.36E-30

EDNRB	0.54999938	7.33E-44
RCBTB2	0.67930374	4.70E-74
TNS3	0.58446673	1.33E-50
RAPGEF5	0.50835869	1.07E-36
ZDHHC4	0.5257869	1.42E-39
CIDEB	0.55516733	8.02E-45
TTC5	0.64975482	7.65E-66
MTHFS	0.66483998	6.44E-70
IREB2	0.62609075	6.72E-60
CALCOCO2	0.67875142	6.83E-74
RSAD1	0.53846517	8.92E-42
NMT1	0.57038219	9.44E-48
SRSF1	0.54594948	4.04E-43
VEZF1	0.63148007	3.30E-61
TACO1	0.5340251	5.39E-41
DCAF7	0.67265168	4.02E-72
KAT7	0.64169362	9.27E-64
ACTL6A	0.60244559	1.89E-54
TRA2B	0.61177379	1.52E-56
MARCHF7	0.64530768	1.10E-64
TANK	0.58880995	1.65E-51
EPRS1	0.6546296	3.91E-67
VPS45	0.5780027	2.82E-49
KCTD3	0.51533944	7.89E-38
RPS6KC1	0.56891813	1.83E-47
CBWD2	0.68113772	1.35E-74
WDR33	0.62875476	1.53E-60
CCDC115	0.66362013	1.40E-69
SAP130	0.65597004	1.71E-67
IMP4	0.54251328	1.69E-42
UGGT1	0.55221598	2.85E-44
STAM	0.69381687	1.88E-78
ABI1	0.60304063	1.39E-54
YME1L1	0.72568832	4.28E-89
DNAJC1	0.64941966	9.36E-66
NIPSNAP3A	0.58890644	1.57E-51
LRRC8A	0.63567727	3.03E-62
ECPAS	0.59108155	5.45E-52
TOR1B	0.61430009	4.00E-57
C9orf78	0.59633306	4.09E-53
SMC2	0.55063247	5.60E-44
TOR1A	0.59300074	2.13E-52
STXBP1	0.63923852	3.89E-63
CDK5RAP2	0.55162614	3.67E-44
SLC31A1	0.574075	1.74E-48
ZNF189	0.63359951	9.94E-62
STX17	0.58254558	3.32E-50
PRPF4	0.64252036	5.71E-64
ATP6V1G1	0.53225186	1.10E-40
TEX10	0.61188064	1.44E-56
MRPL50	0.65980311	1.57E-68
PSMB7	0.61047824	3.00E-56
TRMO	0.59134724	4.78E-52
RABEPK	0.55849822	1.89E-45
GOLGA1	0.56870833	2.02E-47
XPA	0.57785908	3.01E-49
NCBP1	0.5675935	3.34E-47
PDCL	0.59459542	9.68E-53
RANBP6	0.59701739	2.91E-53
PLAA	0.61517278	2.52E-57
UBAP2	0.58997668	9.34E-52
RNF38	0.6406178	1.74E-63
TLN1	0.55934657	1.30E-45
DCTN3	0.61271163	9.27E-57
GRHPR	0.6682035	7.37E-71
ALDH1B1	0.58729542	3.42E-51
DENND4C	0.59241747	2.83E-52
PPIL1	0.61149887	1.75E-56
KLC4	0.56630396	5.96E-47
KIF13A	0.58357712	2.03E-50
CMTR1	0.59667905	3.44E-53
YIPF3	0.67345417	2.36E-72
TMEM14B	0.61507962	2.64E-57
TMEM63B	0.52543751	1.63E-39
TINAG	0.59395935	1.33E-52
BPHL	0.72946418	1.86E-90
RIPK1	0.65650851	1.23E-67
TPMT	0.69773081	1.10E-79
MTCH1	0.60449874	6.62E-55
TAF8	0.50624908	2.33E-36
FAM8A1	0.74520708	2.17E-96
FCHSD2	0.54559909	4.68E-43
ARRB1	0.5631073	2.48E-46
ANKRD42	0.61210815	1.27E-56
CCDC90B	0.57290611	2.98E-48
PRCP	0.59813754	1.66E-53
NARS2	0.66260541	2.68E-69
RNF121	0.67831002	9.20E-74
TGS1	0.54231755	1.83E-42
SDCBP	0.53550621	2.97E-41
NEK1	0.5947502	8.97E-53
DDX60	0.59431336	1.11E-52
BUD13	0.60351222	1.10E-54
YAP1	0.50263823	8.68E-36
SLC37A4	0.54097404	3.19E-42
RDX	0.76097248	8.73E-103
PPP2R1B	0.60179109	2.63E-54
FDX1	0.60041417	5.29E-54
C11orf1	0.50203997	1.08E-35
ALKBH8	0.59100997	5.64E-52
MAP2K5	0.5981698	1.63E-53
CTDSPL2	0.62027812	1.62E-58
SLTM	0.54737164	2.22E-43
NDUFAF1	0.57293266	2.94E-48
HAUS2	0.62246693	4.93E-59
RTF1	0.56032146	8.48E-46
PAQR5	0.63174335	2.85E-61
LRRC49	0.5289181	4.14E-40
TUBGCP4	0.62360085	2.65E-59
RMDN3	0.7281271	5.68E-90
TMEM62	0.54055036	3.80E-42
ADAM10	0.56826512	2.46E-47
ZNF280D	0.54488946	6.29E-43
BCAR3	0.60864238	7.80E-56
FNBP1L	0.67837038	8.83E-74
KYAT3	0.52328292	3.76E-39
GTF2B	0.65066787	4.40E-66
RABGGTB	0.53072509	2.02E-40
GIPC2	0.70598104	2.41E-82
ARHGAP29	0.54518706	5.56E-43
DBT	0.68584145	5.28E-76
RTCA	0.62777136	2.64E-60
SELENOI	0.57820978	2.56E-49
HADHB	0.77078723	5.04E-107
KHK	0.56858901	2.13E-47
PPM1B	0.54940556	9.42E-44
PNPT1	0.59703569	2.88E-53
DYNC2LI1	0.55881239	1.64E-45
RAB1A	0.72258796	5.39E-88
ACTR2	0.5962654	4.23E-53
PREB	0.50875018	9.27E-37
PREPL	0.72631683	2.55E-89
SLC3A1	0.72053794	2.83E-87
FBXO11	0.59068878	6.60E-52
LRPPRC	0.62818685	2.10E-60
ACTR1A	0.5149991	8.97E-38
ATAD1	0.58295195	2.74E-50
TACC2	0.58418874	1.52E-50
ARL3	0.66452922	7.86E-70
EXOC6	0.59551136	6.15E-53
DBR1	0.62555855	9.02E-60
DNAJC13	0.56958759	1.35E-47
ANXA7	0.73489417	1.87E-92
FAM149B1	0.61599116	1.63E-57
ASCC1	0.62249089	4.87E-59
SMARCAL1	0.61727738	8.18E-58
ASNSD1	0.70693431	1.17E-82
SSB	0.6487702	1.38E-65
NAB1	0.5918016	3.83E-52
PPIG	0.62552051	9.21E-60
OLA1	0.59656526	3.64E-53
ITPRID2	0.71737208	3.55E-86
FAM117B	0.5845809	1.26E-50
WDR12	0.68139384	1.13E-74
ABI2	0.58752683	3.06E-51
ITGAV	0.52856947	4.75E-40
SLC40A1	0.61305565	7.73E-57
SLC35A5	0.64210582	7.28E-64
SLC49A4	0.53567087	2.78E-41
USP8	0.66346811	1.55E-69
SECISBP2L	0.60190855	2.48E-54
TMOD3	0.62668136	4.84E-60
SPPL2A	0.60142625	3.17E-54
GLCE	0.64159355	9.83E-64
INTS14	0.67326289	2.68E-72
PARP16	0.55655096	4.40E-45
ARHGAP24	0.72626798	2.65E-89
HERC3	0.57168041	5.22E-48
HERC5	0.51925422	1.78E-38
AP1AR	0.70871871	3.00E-83
COPS4	0.70933561	1.87E-83
HNRNPD	0.56375316	1.86E-46
SEC31A	0.71466579	3.00E-85
GPAT3	0.52755685	7.08E-40
BBS7	0.6751686	7.57E-73
RAP1GDS1	0.53369697	6.15E-41
LARP1B	0.5416216	2.44E-42
NAAA	0.5586995	1.73E-45
NUP54	0.5840369	1.63E-50
G3BP2	0.62486332	1.32E-59
SEPTIN11	0.66934575	3.51E-71
FRAS1	0.57840511	2.33E-49
SCARB2	0.65569525	2.03E-67
CNOT6L	0.53233494	1.06E-40
USO1	0.64274284	5.01E-64
CDKL2	0.54124379	2.85E-42
PPA2	0.55210844	2.98E-44
GSTCD	0.59797906	1.80E-53
INTS12	0.62477975	1.39E-59
ENPEP	0.68425592	1.59E-75

RARA	1.063198	6.071865	1.55E-30	9.49E-30
ANKK1	2.196379	-0.49684	1.56E-30	9.53E-30
CDHR3	1.438198	3.104512	1.62E-30	9.92E-30
RPLP0	1.067155	10.31776	1.70E-30	1.04E-29
C16orf74	3.078623	3.067548	1.70E-30	1.04E-29
SMTNL1	2.924646	-0.66601	1.85E-30	1.13E-29
RFTN1	1.003756	6.044418	1.92E-30	1.17E-29
TRAT1	2.371677	1.359309	1.92E-30	1.17E-29
TRIM52	1.110064	4.318794	1.94E-30	1.18E-29
GYPC	1.04517	5.936689	1.94E-30	1.18E-29
SCN4B	1.972907	5.139461	1.97E-30	1.20E-29
ATP11A	1.505015	8.158396	2.01E-30	1.23E-29
RAD51AP	1.323219	1.85141	2.22E-30	1.35E-29
CACNA1S	-2.04574	-1.46095	2.26E-30	1.37E-29
ANXA3	-2.00711	3.848424	2.27E-30	1.38E-29
TDGF1	-3.23834	2.355766	2.39E-30	1.45E-29
CCDC73	1.703172	-0.37247	2.47E-30	1.50E-29
GPR18	2.176944	0.227707	2.53E-30	1.54E-29
RPL38	1.029003	8.270223	2.64E-30	1.60E-29
DDX39B	1.594647	6.169411	2.85E-30	1.73E-29
EIF4EBP3	1.300696	3.415214	2.90E-30	1.76E-29
TMCC2	-1.04707	1.985155	3.01E-30	1.83E-29
RPL18	1.166197	9.232374	3.04E-30	1.84E-29
TASL	1.657251	1.854264	3.07E-30	1.86E-29
KL	-1.60135	6.835103	3.19E-30	1.93E-29
GPR82	2.068419	0.664738	3.20E-30	1.94E-29
AATK	1.393576	2.409222	3.21E-30	1.94E-29
NPDC1	1.402071	5.642871	3.28E-30	1.98E-29
MCTP2	1.326412	4.661001	3.41E-30	2.06E-29
LAIR2	4.022905	-0.32987	3.44E-30	2.08E-29
NPIPA1	1.746499	1.940259	3.46E-30	2.09E-29
APELA	-3.21395	-0.92121	3.59E-30	2.17E-29
CHDH	-1.29408	5.939961	3.74E-30	2.25E-29
FCRL3	2.828227	1.660081	3.87E-30	2.33E-29
ESRP1	-3.10723	3.017263	3.93E-30	2.37E-29
PLA2G5	2.792136	-0.25493	4.17E-30	2.51E-29
AKAP5	-1.31428	2.278782	4.31E-30	2.59E-29
ARHGAP4	1.283021	5.469817	4.38E-30	2.63E-29
EFCAB3	3.369358	-3.24994	4.45E-30	2.67E-29
CD300LB	1.745862	1.01507	4.48E-30	2.70E-29
CD200R1	2.272806	1.972098	4.83E-30	2.90E-29
CCDC13	-1.40682	0.353382	5.05E-30	3.03E-29
P4HA2	1.293674	6.821052	5.10E-30	3.06E-29
NLRP12	1.698547	-0.13921	5.22E-30	3.13E-29
PFKFB2	-1.70768	5.020329	5.73E-30	3.43E-29
CDCA5	1.601319	2.342896	5.79E-30	3.47E-29
DRD4	1.997074	-0.14412	5.93E-30	3.55E-29
HRH2	2.875727	3.991372	6.05E-30	3.62E-29
MYL5	1.22901	3.564068	6.16E-30	3.68E-29
BTNL9	2.183971	5.860801	6.65E-30	3.97E-29
SLC17A4	3.633214	5.638402	6.69E-30	3.99E-29
CLASRP	1.220284	4.756417	6.77E-30	4.03E-29
DHRS4	-1.01973	4.192613	7.11E-30	4.23E-29
CYP39A1	-1.65721	2.291548	7.13E-30	4.24E-29
ICAM1	1.416992	6.677277	7.16E-30	4.26E-29
NPHP3	1.169743	3.469199	7.58E-30	4.51E-29
DLX4	2.665488	0.013779	7.86E-30	4.67E-29
SLC6A4	-1.819	-0.50144	7.95E-30	4.72E-29
RNF223	-2.78219	-2.28388	8.50E-30	5.05E-29
GPX3	-1.59038	11.61694	8.62E-30	5.12E-29
PIDD1	1.405587	3.687357	8.80E-30	5.22E-29
FCMR	1.492928	3.115749	9.17E-30	5.43E-29
CENPT	1.184457	4.727346	9.52E-30	5.64E-29
CPNE5	1.777489	3.236872	9.60E-30	5.68E-29
CD101	1.474051	1.221182	9.71E-30	5.74E-29
SLC16A8	2.075998	-0.21502	9.87E-30	5.83E-29
HOXD13	3.317975	-0.86871	9.93E-30	5.86E-29
PARP10	1.005494	6.528896	9.95E-30	5.87E-29
FABP7	5.857122	7.666662	1.02E-29	6.03E-29
HORMAD	3.101272	-2.52307	1.03E-29	6.08E-29
CD248	1.797965	6.137469	1.09E-29	6.44E-29
CACNG8	2.19709	-0.10877	1.11E-29	6.53E-29
C3orf70	1.523313	3.500326	1.12E-29	6.62E-29
HHLA2	3.150028	5.992773	1.13E-29	6.67E-29
SH2D4A	-1.13534	4.242153	1.16E-29	6.84E-29
CARD9	1.513527	2.196447	1.17E-29	6.90E-29
OR52N4	2.523935	-1.27818	1.18E-29	6.96E-29
IL18RAP	1.77325	0.941369	1.20E-29	7.03E-29
KLF5	-1.48626	4.092104	1.24E-29	7.30E-29
PCDHGC	2.163631	-0.57063	1.28E-29	7.50E-29
MIM10L2	-1.46703	1.849113	1.29E-29	7.56E-29
NRM	1.094586	4.285577	1.33E-29	7.83E-29
P2RY13	1.706952	3.113444	1.35E-29	7.94E-29
EXO1	1.704254	0.988422	1.36E-29	7.97E-29
TLR8	2.145044	2.789362	1.36E-29	7.99E-29
ASAP3	-1.1023	4.278306	1.42E-29	8.32E-29
EVC	-1.19712	6.315306	1.45E-29	8.48E-29
CTXN3	-3.36091	2.477141	1.54E-29	9.01E-29
LPAR6	1.218112	5.639003	1.56E-29	9.13E-29
EFNA1	1.235721	6.895151	1.59E-29	9.26E-29
MZT2A	1.612035	5.293458	1.62E-29	9.43E-29
WDFY4	1.767663	4.00462	1.62E-29	9.44E-29
ZBTB32	1.799811	-0.52347	1.72E-29	1.00E-28
CHIT1	5.082522	4.149351	1.74E-29	1.02E-28
ADAMTS2	5.0889	-1.73334	1.86E-29	1.08E-28
DSCC1	1.011685	1.549071	1.88E-29	1.09E-28
STING1	1.049782	6.093498	1.96E-29	1.14E-28
RPS14	1.094305	9.6602	2.01E-29	1.17E-28
PAQR9	3.58544	-1.41917	2.05E-29	1.19E-28
CDH13	1.599602	6.561518	2.13E-29	1.24E-28
LY96	1.712002	2.912643	2.19E-29	1.27E-28
MAPK11	1.211613	4.030807	2.19E-29	1.27E-28
P11-434D1	1.985797	-1.34075	2.21E-29	1.28E-28
GLIPR1	1.524783	4.956774	2.26E-29	1.31E-28
B3GALT2	-1.59523	-0.05073	2.39E-29	1.39E-28
MME	-1.74943	7.399668	2.43E-29	1.41E-28
RPL22L1	1.411782	4.345364	2.60E-29	1.50E-28
GCNT3	-1.75157	4.328793	2.63E-29	1.52E-28
PLEKHG4	-2.13156	1.696537	2.66E-29	1.54E-28
SSBP4	1.002063	5.092189	2.69E-29	1.55E-28
IL2RG	1.884519	5.125365	2.96E-29	1.71E-28
MSI2	-1.00786	6.69334	3.25E-29	1.87E-28
IBSP	5.208855	0.236133	3.29E-29	1.90E-28
CHRNA6	2.984364	-0.7357	3.47E-29	2.00E-28
CDH3	-2.29586	2.978734	3.56E-29	2.05E-28
SAPCD1	2.982996	-0.23904	3.59E-29	2.06E-28
SH3PXD2	1.55431	5.108611	3.64E-29	2.09E-28
MLIP	2.168242	1.091935	3.70E-29	2.13E-28
F2RL3	2.365003	4.921051	3.76E-29	2.16E-28
SLC9A2	-2.98622	2.523293	3.80E-29	2.19E-28
KIRREL2	-2.41975	-1.31743	3.93E-29	2.26E-28
DPT	-2.18932	2.445828	3.96E-29	2.28E-28
UQCRC1	-1.0258	7.232595	4.02E-29	2.30E-28
SPRY4	1.286823	6.91483	4.06E-29	2.33E-28
MATN1	1.649681	-1.46417	4.06E-29	2.33E-28
BGLAP	2.311592	-0.51149	4.10E-29	2.35E-28
KLHL31	1.688276	0.781859	4.17E-29	2.39E-28
CDH24	1.113631	2.937746	4.54E-29	2.60E-28
OR51E2	2.22553	1.662087	4.83E-29	2.76E-28
TOP2A	1.957863	4.482284	5.04E-29	2.88E-28
LGSN	-2.05024	-0.29824	5.15E-29	2.94E-28
ACRBP	1.278694	2.099468	5.26E-29	3.00E-28
C5orf47	-1.80435	-2.62624	5.39E-29	3.07E-28
CENPF	1.836968	3.717718	5.40E-29	3.08E-28
TMPRSS1	-1.72066	0.254084	5.42E-29	3.09E-28
RASSF9	-1.44091	2.546554	5.52E-29	3.14E-28
KCTD19	2.443315	-0.81695	5.52E-29	3.15E-28
ADAMTSL	-1.30541	4.432296	5.56E-29	3.16E-28
CP	3.679074	8.790526	5.97E-29	3.40E-28
FBLN7	2.212795	3.167789	6.06E-29	3.45E-28
KCNK7	1.68718	-1.20663	6.41E-29	3.64E-28
PER2	1.154603	5.491136	6.49E-29	3.69E-28
TEPSIN	1.154905	3.238679	6.73E-29	3.82E-28
NRP1	1.088474	8.777477	6.90E-29	3.92E-28
TMEM27	1.340194	3.815353	7.12E-29	4.04E-28
CDCA7	2.036844	1.921459	7.30E-29	4.14E-28
FYB1	1.700869	5.56044	7.32E-29	4.15E-28
SLC44A4	-1.91054	5.137568	7.62E-29	4.32E-28
RUBCNL	1.508029	2.043573	8.02E-29	4.54E-28
FCGR2B	2.008182	3.32327	8.12E-29	4.59E-28
MSX1	1.376668	2.146012	8.29E-29	4.69E-28
SUSD2	-1.49194	5.145778	8.58E-29	4.85E-28
THEMIS	2.236998	2.150487	8.64E-29	4.88E-28
PAH	-2.8645	6.076038	8.72E-29	4.93E-28
BMP7	-3.09988	2.017183	8.87E-29	5.00E-28
EME2	1.905057	3.670895	8.89E-29	5.02E-28
IGFBP2	-1.81228	5.444657	9.00E-29	5.08E-28
C3orf22	2.861912	-3.1943	9.25E-29	5.22E-28
C17orf49	1.016738	0.620098	9.71E-29	5.47E-28
AXIN2	-1.10791	1.539078	9.99E-29	5.62E-28
MYC	1.540904	6.603861	1.00E-28	5.63E-28
CLEC5A	2.083902	2.398646	1.10E-28	6.18E-28
RPL36	1.267627	8.457701	1.10E-28	6.19E-28
LRRC66	1.959917	1.295875	1.10E-28	6.19E-28
APOL2	1.38038	6.624133	1.11E-28	6.22E-28
KRT81	3.532079	-0.37493	1.11E-28	6.25E-28
PERM1	3.104205	2.81547	1.15E-28	6.45E-28
GABRA4	-2.87968	-2.37797	1.17E-28	6.55E-28
RFLNB	1.46939	5.757213	1.19E-28	6.64E-28
SH3D21	1.472708	3.925042	1.25E-28	6.99E-28
TPRG1	2.656849	1.806077	1.37E-28	7.67E-28
CRYBB1	1.478796	-0.41377	1.37E-28	7.68E-28
VMP1	1.297654	7.6822	1.44E-28	8.03E-28
C9orf153	1.800977	-1.95345	1.57E-28	8.78E-28
NNT	-1.22238	6.908135	1.62E-28	9.04E-28
SLC17A2	3.315813	2.069234	1.65E-28	9.21E-28
CDH23	2.377012	3.185214	1.68E-28	9.34E-28
P2RX3	2.829588	-2.09759	1.71E-28	9.52E-28
ATP6V1G	-1.10875	0.194242	1.72E-28	9.56E-28
PCDHB14	1.406955	3.780894	1.72E-28	9.59E-28
HMGCS2	-2.62757	6.192875	1.76E-28	9.76E-28
IL15	1.035926	3.26439	1.77E-28	9.84E-28
CLK2	1.058776	5.164031	1.80E-28	9.98E-28
SLC22A6	-2.87218	5.770179	1.80E-28	9.99E-28
TUFT1	-1.01359	4.8185	1.81E-28	1.01E-27
PNMT	-2.45378	-1.68107	1.88E-28	1.04E-27
TLCD3B	5.125019	0.081387	1.90E-28	1.05E-27
CRYL1	-1.05562	7.353238	1.90E-28	1.05E-27
PKNOX2	-1.5674	1.102521	1.99E-28	1.10E-27
TXLNB	1.874891	0.750436	2.04E-28	1.13E-27

HADH	0.63264538	1.71E-61
PAPSS1	0.5024749	9.21E-36
SEC24B	0.59958506	8.03E-54
PPP3CA	0.50726972	1.60E-36
SLC39A8	0.61911565	3.04E-58
GUCD1	0.60390388	8.98E-55
RNF185	0.74634802	7.77E-97
KIF21A	0.5297054	3.03E-40
CPNE8	0.60084095	4.26E-54
ALG10	0.51652608	5.03E-38
AEBP2	0.52100938	9.06E-39
ETFBKMT	0.5763164	6.17E-49
PEX5	0.64921179	1.06E-65
SCAF11	0.55106487	4.66E-44
LLPH	0.516157	5.79E-38
LRIG3	0.5383244	9.45E-42
TMEM19	0.57709216	4.30E-49
POC1B	0.5494548	9.23E-44
TMTC3	0.58874582	1.70E-51
AMDHD1	0.59771345	2.05E-53
NEDD1	0.6255002	9.32E-60
GAS2L3	0.50205115	1.07E-35
RITA1	0.5573389	3.13E-45
GIT2	0.61390469	4.93E-57
NUP58	0.61169331	1.58E-56
MTMR6	0.66948701	3.20E-71
LNX2	0.65864982	3.23E-68
SUOX	0.60453064	6.51E-55
SMARCC2	0.58069496	7.96E-50
ESYT1	0.66037134	1.10E-68
TMBIM6	0.66016221	1.25E-68
ZNF740	0.504124	5.06E-36
ESD	0.67285453	3.51E-72
RB1	0.67969167	3.61E-74
SBNO1	0.53336252	7.04E-41
VPS33A	0.5867628	4.42E-51
DENR	0.50786649	1.28E-36
MBNL2	0.56372166	1.89E-46
ABHD13	0.60523595	4.54E-55
RAB20	0.56188278	4.26E-46
CUL4A	0.64961908	8.30E-66
TMX1	0.6279529	2.39E-60
NAA30	0.54089067	3.30E-42
WDR89	0.63595108	2.59E-62
EFCAB11	0.5772962	3.91E-49
AK7	0.52366485	3.24E-39
WDR20	0.57350624	2.26E-48
NIPA2	0.56197946	4.09E-46
SLC12A6	0.62049249	1.44E-58
MFAP1	0.72931402	2.11E-90
SORD	0.57679618	4.94E-49
ZSCAN29	0.50991737	6.01E-37
SLC27A2	0.67257057	4.24E-72
GTF2A2	0.50975245	6.39E-37
SRP14	0.51815226	2.71E-38
BAHD1	0.58333797	2.28E-50
TLE3	0.52053353	1.09E-38
ANP32A	0.62911422	1.25E-60
ETFA	0.70083117	1.13E-80
HMG20A	0.60890835	6.80E-56
SCAPER	0.57788613	2.97E-49
TSPAN3	0.60120466	3.54E-54
WDR61	0.65849929	3.55E-68
NCOA2	0.53690997	1.68E-41
IGF1R	0.54737488	2.22E-43
USP3	0.50685349	1.86E-36
SCAMP2	0.69427747	1.35E-78
ABHD2	0.54241228	1.76E-42
DET1	0.54391925	9.43E-43
UNC45A	0.62104798	1.07E-58
IQGAP1	0.57129748	6.22E-48
CRTC3	0.59125666	5.00E-52
EFL1	0.64189683	8.23E-64
SEC11A	0.57534984	9.66E-49
GLYR1	0.70158784	6.46E-81
RUSF1	0.62141265	8.76E-59
FTO	0.66356431	1.45E-69
UQCRC2	0.6126285	9.68E-57
DHX38	0.5559496	5.71E-45
NUDT7	0.61944087	2.55E-58
GCSH	0.56326756	2.31E-46
MAP1LC3B	0.51367232	1.48E-37
MBTPS1	0.60556191	3.84E-55
CDH13	0.52841318	5.05E-40
ZCCHC14	0.50518091	3.44E-36
PDPK1	0.64252205	5.70E-64
GAS8	0.51179342	2.99E-37
MED9	0.56254605	3.18E-46
NCOR1	0.62326033	3.19E-59
COPS3	0.61398648	4.72E-57
GID4	0.69801998	8.94E-80
ZNF287	0.51681919	4.50E-38
KSR1	0.58496535	1.05E-50
UTP4	0.7016926	5.97E-81
GFOD2	0.58790255	2.55E-51
PRPSAP2	0.74866389	9.46E-98
PCTP	0.52892425	4.13E-40
TOM1L1	0.71453463	3.33E-85
C17orf80	0.69523109	6.79E-79
TOB1	0.58105225	6.73E-50
CLTC	0.70067436	1.27E-80
BCAS3	0.64318054	3.87E-64
SS18	0.54687386	2.74E-43
TAF4B	0.54460722	7.08E-43
AFG3L2	0.66056926	9.69E-69
IMPA2	0.65180038	2.21E-66
RPRD1A	0.65391721	6.06E-67
GAREM1	0.70982299	1.29E-83
OSBPL1A	0.67968609	3.63E-74
RMC1	0.53573172	2.71E-41
ARHGDIA	0.52431503	2.52E-39
EIF4A3	0.58910098	1.43E-51
FN3KRP	0.64684989	4.38E-65
WDR45B	0.66581261	3.45E-70
DYM	0.62964478	9.28E-61
ELAC1	0.62920118	1.19E-60
MBD1	0.51489749	9.32E-38
SMAD4	0.59569267	5.62E-53
ZCCHC2	0.52431691	2.52E-39
RETREG3	0.51757955	3.37E-38
PFKL	0.56114604	5.90E-46
CCDC97	0.59367286	1.53E-52
SIRT3	0.65437451	4.58E-67
IFNAR1	0.67544484	6.29E-73
SOD1	0.53090122	1.88E-40
APP	0.67730939	1.81E-73
SAE1	0.59795107	1.82E-53
SLC47A1	0.65580175	1.90E-67
ZNF473	0.54610963	3.78E-43
RERE	0.54894446	1.15E-43
KIAA0319L	0.63467731	5.38E-62
WDTC1	0.57433098	1.55E-48
SERBP1	0.64893524	1.25E-65
BCL10	0.57704241	4.40E-49
PRKACB	0.57323329	2.56E-48
PIGK	0.66618106	2.72E-70
MOB3C	0.53053462	2.18E-40
SLC44A3	0.60683194	1.99E-55
ZNF697	0.69722303	1.60E-79
CTTNBP2NL	0.58714673	3.68E-51
ALDH9A1	1	0
ATP1B1	0.51231992	2.46E-37
TIPRL	0.67748937	1.60E-73
NME7	0.70634512	1.83E-82
POGK	0.65535878	2.50E-67
MPC2	0.65501819	3.08E-67
CREG1	0.6683123	6.87E-71
DCAF6	0.64171881	9.14E-64
TMCO1	0.75110011	1.01E-98
ILDR2	0.59904995	1.05E-53
MGST3	0.64119332	1.24E-63
COP1	0.59817438	1.63E-53
UFC1	0.5765775	5.47E-49
SDHC	0.76616446	5.30E-105
PRCC	0.51003009	5.77E-37
PIGM	0.72511259	6.86E-89
ISG20L2	0.662564	2.75E-69
HDGF	0.66771972	1.01E-70
XPR1	0.75882279	6.95E-102
TOR1AIP1	0.71797275	2.20E-86
RGL1	0.78096224	1.20E-111
LYPLAL1	0.60424336	7.55E-55
PRUNE1	0.63803435	7.81E-63
RORC	0.56861013	2.11E-47
TUFT1	0.63005287	7.38E-61
ZNF687	0.68027646	2.43E-74
TARS2	0.5324028	1.03E-40
SNX27	0.71914747	8.62E-87
SETDB1	0.5386932	8.13E-42
RFX5	0.54159349	2.47E-42
PI4KB	0.61903123	3.18E-58
PIP5K1A	0.51304219	1.87E-37
ANP32E	0.6628458	2.30E-69
MINDY1	0.51957291	1.57E-38
CERS2	0.70082526	1.14E-80
ENSA	0.62237461	5.18E-59
MRPL9	0.59748875	2.30E-53
ARNT	0.69757739	1.23E-79
GOLPH3L	0.71227975	1.93E-84
DYRK3	0.67202286	6.08E-72
EIF2D	0.50261916	8.74E-36
INTS7	0.56637086	5.78E-47
TAF1A	0.5149319	9.20E-38
SMYD2	0.55550361	6.93E-45
TP53BP2	0.5523469	2.69E-44
TPM3	0.61604095	1.58E-57
SNAPIN	0.57332743	2.45E-48
UBAP2L	0.60666698	2.17E-55
SLC39A1	0.6633653	1.65E-69
HAX1	0.54075692	3.49E-42

GRIN2D	1.96997	0.882872	2.06E-28	1.14E-27
WDR54	1.163472	4.900211	2.14E-28	1.18E-27
GOLGA8A	2.862762	4.504852	2.37E-28	1.30E-27
QTRT1	1.193271	5.298947	2.42E-28	1.33E-27
POLR2F	1.679554	-1.57278	2.45E-28	1.35E-27
FAM72C	2.286046	-2.76519	2.56E-28	1.41E-27
KMT5C	1.286178	2.741827	2.73E-28	1.50E-27
SCIN	-2.01018	6.484748	2.75E-28	1.51E-27
P11-203J2	2.148956	-1.12339	2.80E-28	1.54E-27
CIITA	1.462013	5.454351	2.83E-28	1.56E-27
SLCO2B1	1.586179	6.761803	2.87E-28	1.57E-27
TAFA1	-1.32398	-2.17986	2.91E-28	1.60E-27
IRF9	1.706465	2.682462	3.02E-28	1.66E-27
PSD2	2.426697	0.231383	3.34E-28	1.83E-27
SPOCK2	-1.05163	7.199983	3.45E-28	1.89E-27
WDR97	2.442065	0.658306	3.57E-28	1.96E-27
WT1	-2.49473	2.706811	3.67E-28	2.01E-27
ETS1	1.161832	8.221703	3.87E-28	2.11E-27
EXOC3L4	2.074449	3.660005	3.97E-28	2.17E-27
CHRNA4	-3.165	-0.19533	4.10E-28	2.24E-27
CHKB	1.725967	2.369294	4.17E-28	2.28E-27
ARL10	1.331834	4.537825	4.28E-28	2.34E-27
KRTAP5-1	2.70184	-1.67847	4.46E-28	2.43E-27
HYAL1	-1.05597	5.416039	4.52E-28	2.46E-27
CNTN5	-2.51769	-1.04141	4.67E-28	2.54E-27
IGFLR1	1.657862	1.233101	4.81E-28	2.62E-27
SHMT1	-1.02608	7.458859	5.01E-28	2.72E-27
TNFSF15	-1.23226	2.76703	5.02E-28	2.73E-27
CCL4L2	2.575933	2.936632	5.13E-28	2.79E-27
TRPM5	-2.07563	-2.68588	5.29E-28	2.87E-27
CSF3	-3.176	-1.18017	5.33E-28	2.89E-27
ADORA2A	1.411514	0.159667	5.38E-28	2.92E-27
PECAM1	1.245622	9.343317	5.39E-28	2.93E-27
APOLD1	1.974211	7.948498	5.46E-28	2.96E-27
INSC	-1.77123	-1.77409	5.61E-28	3.04E-27
WDR62	1.526525	1.49556	5.69E-28	3.09E-27
UBXN11	1.335576	4.972826	6.06E-28	3.29E-27
VWCE	2.776909	3.829455	6.17E-28	3.34E-27
DLGAP5	2.057132	1.723799	6.19E-28	3.35E-27
MARVELD	-1.43156	3.321896	6.26E-28	3.39E-27
PGM5	-1.23933	4.658313	6.34E-28	3.43E-27
PCDHB9	1.609291	2.199883	6.37E-28	3.44E-27
SCART1	2.252162	1.300927	6.47E-28	3.50E-27
PYGO1	-1.61562	1.838136	6.62E-28	3.58E-27
CARD11	2.120029	4.102251	7.23E-28	3.91E-27
SYTL4	-1.15717	3.819232	7.66E-28	4.13E-27
CABYR	1.496176	0.609825	7.96E-28	4.29E-27
CCDC178	-1.47127	1.18112	8.52E-28	4.59E-27
GABRP	-2.23469	1.622998	8.54E-28	4.60E-27
APOL6	1.075389	6.870591	9.46E-28	5.09E-27
ZC3H12D	1.551591	1.699484	9.65E-28	5.19E-27
NFE2L3	1.383601	5.238047	9.68E-28	5.21E-27
IDUA	1.254381	3.750346	9.74E-28	5.24E-27
STBD1	1.010477	2.105304	9.75E-28	5.24E-27
LY6H	3.47786	1.106061	9.89E-28	5.31E-27
KIR3DL1	2.19028	-1.67762	1.00E-27	5.37E-27
GPR55	1.839507	-0.30436	1.02E-27	5.46E-27
LIPA	1.187001	7.678641	1.03E-27	5.50E-27
ALPK2	1.978832	6.365739	1.03E-27	5.51E-27
BLNK	-1.11245	4.17954	1.05E-27	5.61E-27
RGS18	1.632632	2.464661	1.08E-27	5.76E-27
ARHGEF2	-1.40135	1.835446	1.08E-27	5.76E-27
DLX2	2.560378	-2.04083	1.09E-27	5.84E-27
PRG2	2.924315	-2.88032	1.10E-27	5.89E-27
PARM1	-1.12091	6.39927	1.11E-27	5.93E-27
RUNX2	1.47703	3.374292	1.12E-27	6.01E-27
HEYL	1.640849	6.19955	1.17E-27	6.24E-27
ASB9	-1.05942	2.717098	1.22E-27	6.51E-27
C1orf54	1.085373	3.739122	1.31E-27	6.97E-27
AFP	-2.30065	-0.39797	1.34E-27	7.13E-27
ADH1C	-2.99155	2.704854	1.40E-27	7.46E-27
FAM177B	1.911121	0.055565	1.51E-27	8.02E-27
GPC2	1.983273	-0.77262	1.52E-27	8.09E-27
CDK5R1	1.097244	1.890596	1.54E-27	8.17E-27
CDCA3	1.703238	1.94828	1.66E-27	8.79E-27
ARL6IP4	1.661825	1.174593	1.70E-27	9.03E-27
IL9R	2.115862	-1.41444	1.75E-27	9.29E-27
IL9R.1	2.115862	-1.41444	1.75E-27	9.29E-27
RTEL1	1.161781	0.616453	1.77E-27	9.40E-27
KCNT1	2.362083	-1.41684	1.80E-27	9.52E-27
STX1B	1.755675	1.798947	1.80E-27	9.53E-27
MUC13	-2.52049	2.897017	1.80E-27	9.55E-27
C1QTNF7	-1.60608	2.098332	1.84E-27	9.74E-27
COL1A1	2.82422	9.987572	2.00E-27	1.06E-26
NRBP2	1.501238	5.682553	2.09E-27	1.10E-26
TNFRSF2	1.867016	3.105192	2.11E-27	1.11E-26
DCST2	2.004121	-0.42437	2.34E-27	1.24E-26
ANKAR	1.063487	1.923005	2.51E-27	1.32E-26
CD82	-1.37483	5.547678	2.59E-27	1.37E-26
CIART	1.361467	3.310675	2.71E-27	1.42E-26
TGFA	1.671791	7.213121	2.74E-27	1.44E-26
CCDC14	1.156316	5.061396	2.89E-27	1.52E-26
C6orf132	-1.45021	3.159565	2.93E-27	1.54E-26
CXCL13	4.195432	2.657808	3.03E-27	1.59E-26
MYO1A	2.813742	-0.74132	3.10E-27	1.63E-26
ANG	1.72487	4.55771	3.18E-27	1.67E-26
SLC4A9	-3.07216	2.11937	3.34E-27	1.75E-26
ANLN	2.074355	3.524505	3.35E-27	1.75E-26
T6GALNA	-1.19209	3.726474	3.64E-27	1.90E-26
DNASE2E	2.90464	-1.27522	3.77E-27	1.97E-26
KCNV2	2.347089	-2.46262	4.00E-27	2.09E-26
PBK	1.848421	1.390214	4.03E-27	2.10E-26
SLC25A1	-1.0791	2.142579	4.03E-27	2.10E-26
LILRA4	2.54304	0.463925	4.06E-27	2.12E-26
PLG	-3.1864	5.397845	4.18E-27	2.18E-26
COL21A1	2.234323	3.687706	4.46E-27	2.32E-26
FSTL1	1.384898	8.978419	4.60E-27	2.40E-26
CTXND1	-2.09649	2.093859	4.72E-27	2.45E-26
MET	1.189208	8.260047	4.79E-27	2.49E-26
KIF19	1.796214	-0.18566	5.18E-27	2.69E-26
SP140	1.796573	2.121635	5.33E-27	2.77E-26
GPR174	2.469699	0.580652	5.34E-27	2.77E-26
KLHL33	-1.50169	-2.20642	5.71E-27	2.96E-26
PAM16	1.051445	2.769739	5.84E-27	3.02E-26
SPINK13	4.009722	2.648223	6.10E-27	3.15E-26
DMP1	4.188173	-1.78432	6.13E-27	3.17E-26
TUBA1A	1.036227	7.316464	6.16E-27	3.18E-26
PHYH	-1.11624	6.087417	6.20E-27	3.20E-26
DNHD1	1.588144	3.547932	6.34E-27	3.28E-26
CADM4	-1.65428	3.971013	6.40E-27	3.30E-26
ZNF878	-1.21659	-1.6955	6.68E-27	3.45E-26
SMIM10	1.073558	3.682325	6.70E-27	3.46E-26
NLRP1	1.20526	4.954352	6.86E-27	3.54E-26
SLC6A8	1.657296	8.71041	6.88E-27	3.55E-26
RDM1	2.000294	-1.40221	7.42E-27	3.83E-26
FER1L6	-2.67179	0.885035	8.05E-27	4.15E-26
DEPDC1	2.027251	1.398431	8.18E-27	4.21E-26
ATP10B	-2.06731	-0.86815	8.24E-27	4.24E-26
ABCA12	2.471126	3.062573	8.32E-27	4.29E-26
ANK3	-1.31023	6.458014	8.40E-27	4.32E-26
RDH12	-2.05076	1.738833	8.63E-27	4.44E-26
FGFBP2	1.89147	1.268173	8.72E-27	4.49E-26
MARCHF	1.625666	4.368024	8.81E-27	4.53E-26
GIMAP5	1.358897	2.634563	8.82E-27	4.54E-26
PEAK3	1.420255	0.212798	8.90E-27	4.58E-26
H3C10	2.596098	1.641334	8.91E-27	4.58E-26
NPIPB11	2.350738	0.451718	9.02E-27	4.64E-26
RPL28	1.117046	9.022049	9.15E-27	4.70E-26
GUCA1C	-3.12377	-2.9185	9.17E-27	4.71E-26
SPIC	3.649261	-2.35076	9.24E-27	4.74E-26
RPL13	1.150768	9.93012	9.38E-27	4.81E-26
ACCS	1.283465	4.314449	9.43E-27	4.83E-26
RASL11A	-1.04604	4.304203	9.57E-27	4.90E-26
VKORC1	1.167399	5.943492	9.64E-27	4.94E-26
CLEC1A	1.272388	3.17344	1.00E-26	5.11E-26
VWA1	1.320101	7.334782	1.02E-26	5.23E-26
CCDC18	1.272182	1.700052	1.04E-26	5.32E-26
DDX11	1.280417	4.083653	1.04E-26	5.33E-26
KRT32	3.540076	-2.72177	1.05E-26	5.36E-26
C19orf84	1.963833	-2.0631	1.06E-26	5.41E-26
SLC39A14	1.726037	8.358958	1.07E-26	5.48E-26
CCR10	1.699553	0.624103	1.11E-26	5.65E-26
VAT1L	-2.40293	2.892065	1.12E-26	5.71E-26
SCTR	-1.42243	2.838024	1.16E-26	5.89E-26
E2F7	2.12568	0.939884	1.16E-26	5.90E-26
ANKRD9	-1.17092	4.099276	1.18E-26	5.99E-26
SHISA3	-2.14331	4.130954	1.20E-26	6.09E-26
PPARGC1	-1.67364	5.941944	1.24E-26	6.32E-26
FREM2	-1.66715	5.020657	1.26E-26	6.39E-26
NPIPB13	3.072934	-1.78455	1.26E-26	6.41E-26
GLIPR2	1.051717	4.432682	1.26E-26	6.41E-26
H2BC15	1.126221	1.147246	1.30E-26	6.58E-26
DNA2	1.489453	1.889876	1.32E-26	6.68E-26
LRAT	2.284916	2.18303	1.35E-26	6.83E-26
EPHA3	1.768116	4.132926	1.35E-26	6.83E-26
ABCB4	2.133417	2.553775	1.41E-26	7.16E-26
PREX2	2.077946	5.833752	1.41E-26	7.17E-26
LBHD1	1.046214	1.651428	1.43E-26	7.26E-26
TTK	1.596805	1.344717	1.44E-26	7.28E-26
ANKRD2	1.611405	-0.13809	1.45E-26	7.36E-26
BLM	1.261224	1.345922	1.52E-26	7.70E-26
DAND5	2.892137	-1.55366	1.57E-26	7.95E-26
ANKRD13	1.45948	2.405688	1.60E-26	8.08E-26
C1orf87	-2.71968	-2.95662	1.62E-26	8.19E-26
KIR3DL2	2.315463	-2.46984	1.65E-26	8.33E-26
C5AR1	1.390295	4.681813	1.65E-26	8.35E-26
RPS20	1.072574	9.619147	1.66E-26	8.39E-26
MC1R	1.604803	2.040378	1.76E-26	8.86E-26
PABPC1L	2.232167	4.267409	1.76E-26	8.88E-26
GPR84	2.118145	0.2663	1.77E-26	8.89E-26
STOX1	-1.69476	1.399288	1.86E-26	9.35E-26
BDH1	-1.79349	3.592151	1.87E-26	9.42E-26
OPRD1	1.91103	-0.60781	2.01E-26	1.01E-25
MEIG1	-1.24247	-2.91297	2.03E-26	1.02E-25
KREMEN	2.872455	-1.22132	2.11E-26	1.06E-25
KCNH1	-1.83185	-1.83543	2.12E-26	1.06E-25
PDE7A	1.173254	5.353731	2.14E-26	1.08E-25
TIFAB	2.438236	-0.39201	2.22E-26	1.12E-25
ACRV1	1.872174	-2.5919	2.25E-26	1.13E-25
OGDHL	-1.89815	7.0502	2.41E-26	1.21E-25

CREB3L4	0.50354908	6.24E-36
KCNN3	0.50503357	3.63E-36
C1orf43	0.75811257	1.37E-101
GATAD2B	0.542694	1.57E-42
ILF2	0.64114695	1.28E-63
RIT1	0.75190116	4.79E-99
PKLR	0.58456156	1.27E-50
SCCPDH	0.5827645	2.99E-50
ACP1	0.71858602	1.35E-86
SNAP47	0.62320423	3.29E-59
SRP9	0.69816035	8.07E-80
SDE2	0.70818529	4.51E-83
FBXO28	0.69271902	4.13E-78
ARF1	0.60661428	2.23E-55
CDC42BPA	0.64152624	1.02E-63
PARP1	0.60895771	6.62E-56
PSEN2	0.59533774	6.70E-53
LBR	0.5765176	5.62E-49
EPHX1	0.59524845	7.01E-53
SOX13	0.52303424	4.14E-39
PLEKHA6	0.59562854	5.80E-53
ARL8A	0.6487823	1.37E-65
PDIA6	0.59391211	1.36E-52
HNRNPLL	0.57268852	3.29E-48
GALM	0.55156221	3.77E-44
CAMKMT	0.51197396	2.79E-37
EML4	0.61456239	3.48E-57
WDPCP	0.60828033	9.42E-56
VPS54	0.68339668	2.87E-75
ASXL2	0.59815847	1.64E-53
ETAA1	0.5536133	1.57E-44
CIAO1	0.68082173	1.67E-74
SNRNP200	0.64768311	2.66E-65
MRPS5	0.53041071	2.29E-40
TPRKB	0.53662762	1.88E-41
NAT8	0.57210579	4.30E-48
EXOC6B	0.66995663	2.36E-71
TEX261	0.70773857	6.34E-83
DUSP11	0.73355187	5.89E-92
RALB	0.75822866	1.23E-101
TMEM177	0.526321	1.15E-39
AFF3	0.59342356	1.73E-52
UBXN4	0.69006723	2.72E-77
SPOPL	0.65088906	3.85E-66
POLR2D	0.63595224	2.59E-62
AMMECR1L	0.58056772	8.45E-50
PKP4	0.62040487	1.51E-58
SCRN3	0.70546397	3.55E-82
LNPK	0.56140299	5.27E-46
UBR3	0.64192379	8.10E-64
PHOSPHO2	0.61125311	2.00E-56
HSPD1	0.61959936	2.34E-58
METTL21A	0.53209711	1.17E-40
KANSL1L	0.60114224	3.66E-54
SPAG16	0.62904009	1.30E-60
RHBDD1	0.65956133	1.83E-68
RAB5A	0.51728368	3.77E-38
RETREG2	0.69333918	2.65E-78
CTDSP1	0.55564359	6.52E-45
EAF1	0.5126727	2.15E-37
DYNC1LI1	0.57945147	1.43E-49
CTDSPL	0.51627142	5.54E-38
IL17RD	0.60481344	5.64E-55
PHLDB2	0.51789443	2.99E-38
ABHD10	0.5396361	5.52E-42
RABL3	0.63639168	2.01E-62
ADPRH	0.57644232	5.82E-49
ATG3	0.60547003	4.02E-55
EIF2A	0.56233774	3.49E-46
ALDH1L1	0.59156237	4.31E-52
OSBPL11	0.51797834	2.90E-38
NCEH1	0.50566605	2.88E-36
TCTA	0.69007861	2.70E-77
DCAF1	0.50643022	2.18E-36
FIP1L1	0.62180685	7.07E-59
ATP10D	0.52060467	1.06E-38
OCIAD2	0.51565083	7.01E-38
ENOPH1	0.67136393	9.37E-72
TRMT10A	0.51950934	1.61E-38
KLHL8	0.64323454	3.75E-64
TBCK	0.63733885	1.17E-62
CAMK2D	0.60712977	1.71E-55
CISD2	0.70864826	3.17E-83
TIFA	0.61249179	1.04E-56
SPATA5	0.50747465	1.48E-36
METTL14	0.7008026	1.16E-80
SETD7	0.63838761	6.36E-63
NAF1	0.61480562	3.06E-57
CBR4	0.59160174	4.22E-52
CYP4V2	0.64044769	1.92E-63
MARCHF6	0.57553149	8.88E-49
PIK3R1	0.54579698	4.31E-43
BHMT	0.62417901	1.93E-59
IQGAP2	0.61963434	2.30E-58
RASA1	0.50031084	2.01E-35
GIN1	0.62037653	1.54E-58
PPIP5K2	0.5096162	6.72E-37
PAM	0.51697268	4.25E-38
SLC30A5	0.5901236	8.69E-52
FBXL17	0.57929967	1.53E-49
COMMD10	0.54284151	1.47E-42
YIPF5	0.58078315	7.64E-50
ARHGAP26	0.54248929	1.71E-42
SLC25A48	0.51360546	1.52E-37
DDX46	0.63472935	5.22E-62
FBXO38	0.57513379	1.07E-48
TNIP1	0.51694516	4.29E-38
G3BP1	0.56015737	9.12E-46
RMND5B	0.51966215	1.52E-38
FAM50B	0.67907192	5.50E-74
FARS2	0.68689063	2.54E-76
CDKAL1	0.62875385	1.53E-60
ZMAT2	0.58981102	1.01E-51
TNFRSF21	0.53524079	3.30E-41
MMUT	0.7164431	7.40E-86
ABT1	0.55300968	2.03E-44
RPL7L1	0.65159797	2.50E-66
IRAK1BP1	0.5735253	2.24E-48
RARS2	0.55432581	1.15E-44
SHPRH	0.50859492	9.81E-37
AIG1	0.58374065	1.88E-50
WTAP	0.50023075	2.07E-35
ZMYM4	0.5076134	1.41E-36
ARMT1	0.65631794	1.38E-67
PURB	0.54348871	1.13E-42
CCT6A	0.51665309	4.79E-38
PSPH	0.51978878	1.45E-38
ATXN7L1	0.53011529	2.57E-40
TMEM168	0.56018651	9.00E-46
TRIM4	0.52605855	1.27E-39
MEPCE	0.51894558	2.00E-38
TLK2	0.63731609	1.18E-62
CLTRN	0.64464148	1.63E-64
CASK	0.53686064	1.71E-41
KDM6A	0.55920261	1.39E-45
MSN	0.60504529	5.00E-55
ZNF41	0.51430901	1.16E-37
NONO	0.5831801	2.46E-50
SNX12	0.63203889	2.41E-61
DIAPH2	0.5661254	6.45E-47
PRPS1	0.71890127	1.05E-86
RBMX	0.57669578	5.18E-49
MCPH1	0.51980161	1.44E-38
NSDHL	0.54245558	1.73E-42
ZNF185	0.50277277	8.27E-36
ATP6V1B2	0.68311886	3.47E-75
CCDC25	0.66788779	9.05E-71
CHMP7	0.64527239	1.12E-64
PLPBP	0.71909084	9.02E-87
ERLIN2	0.66417061	9.88E-70
TACC1	0.53188293	1.27E-40
GOLGA7	0.66734847	1.28E-70
MRPS28	0.53339748	6.94E-41
TERF1	0.63225023	2.14E-61
DPYS	0.5598425	1.05E-45
MTDH	0.54854398	1.36E-43
EBAG9	0.5992156	9.67E-54
UTP23	0.51230554	2.47E-37
AK3	0.58730183	3.41E-51
NFIB	0.58021282	9.99E-50
PLIN2	0.59349526	1.67E-52
IDNK	0.62126396	9.50E-59
AUH	0.60868762	7.62E-56
MFSD14B	0.50194398	1.12E-35
ZNF462	0.59679206	3.25E-53
INIP	0.6851966	8.27E-76
SNX30	0.69251345	4.78E-78
STOM	0.70524084	4.21E-82
ALAD	0.72264524	5.15E-88
WDR31	0.66278481	2.39E-69
POLE3	0.63710529	1.34E-62
SURF4	0.56363532	1.96E-46
SURF6	0.55609167	5.37E-45
MED22	0.58716166	3.65E-51
REXO4	0.52762701	6.88E-40
CIZ1	0.53787425	1.14E-41
GPR107	0.60254485	1.80E-54
SEC16A	0.55683041	3.90E-45
NACC2	0.50851086	1.01E-36
USP6NL	0.57967786	1.28E-49
FAM171A1	0.55063429	5.59E-44
MINDY3	0.6333568	1.14E-61
RSU1	0.52021188	1.23E-38
PARD3	0.52479559	2.09E-39
NRBF2	0.50241807	9.41E-36
A1CF	0.64701572	3.97E-65
POLR3A	0.53390162	5.67E-41
GLUD1	0.61829995	4.72E-58
ADD3	0.73013237	1.06E-90
EIF4EBP2	0.69091839	1.49E-77

ENTPD8	-1.71204	0.685544	2.44E-26	1.22E-25
C19orf67	3.81624	0.008703	2.52E-26	1.26E-25
SLC5A4	1.790831	0.943418	2.71E-26	1.36E-25
HPX	4.438373	1.763765	2.78E-26	1.39E-25
GBX2	3.151484	-2.79212	2.98E-26	1.49E-25
CD5L	4.886798	1.364424	2.99E-26	1.49E-25
VCAM1	1.825215	9.143208	3.09E-26	1.54E-25
CYBB	1.824673	6.396059	3.22E-26	1.61E-25
CFAP73	-1.45302	-1.38267	3.27E-26	1.63E-25
RAET1L	-2.26431	-3.00806	3.60E-26	1.79E-25
CSF1	1.025013	6.190365	3.81E-26	1.90E-25
FIBCD1	4.385502	1.681602	3.85E-26	1.91E-25
GSG1L2	10.124	2.603684	3.93E-26	1.95E-25
C21orf58	1.283477	1.369383	4.12E-26	2.05E-25
LAYN	1.269628	4.052365	4.17E-26	2.07E-25
RAB44	1.704161	-1.88018	4.17E-26	2.07E-25
CBLC	-1.95741	1.164231	4.51E-26	2.24E-25
SMC1B	2.283186	-1.41638	4.52E-26	2.25E-25
CDCA8	1.490137	2.060083	4.54E-26	2.25E-25
BHLHA15	2.430377	-0.14386	4.58E-26	2.28E-25
CEACAM	-1.1521	4.742221	4.63E-26	2.30E-25
MARVELD	1.121398	5.370265	4.64E-26	2.30E-25
C19orf38	1.262116	1.567953	4.79E-26	2.37E-25
DCST1	2.452157	-1.67006	5.08E-26	2.51E-25
HLA-DOB	2.19018	2.285423	5.50E-26	2.72E-25
KIF14	1.92714	1.045901	5.56E-26	2.74E-25
TOX3	-2.18708	3.349447	5.87E-26	2.90E-25
LENG8	1.579166	7.148242	5.87E-26	2.90E-25
ZNF80	2.556548	-2.01351	5.88E-26	2.90E-25
ZNF98	-2.13012	-0.97371	6.21E-26	3.06E-25
ADD2	2.723856	1.991911	6.27E-26	3.09E-25
SH3RF3	1.174184	4.069502	6.30E-26	3.10E-25
FN1	1.818787	10.80738	6.48E-26	3.19E-25
TEX11	3.485854	2.544826	6.52E-26	3.21E-25
CACNA1F	2.271884	0.919789	6.75E-26	3.32E-25
ZNF320	1.142871	5.949575	6.78E-26	3.34E-25
RTP5	3.01386	-2.16981	6.79E-26	3.34E-25
CENPE	1.642538	1.899375	6.90E-26	3.39E-25
DLEU7	1.705594	-0.0054	7.02E-26	3.44E-25
ELOVL3	-1.44186	-1.92975	7.13E-26	3.50E-25
HOXD10	-1.32995	3.729668	7.20E-26	3.53E-25
CCRL2	1.550395	2.84749	7.22E-26	3.54E-25
WSCD2	-2.23845	0.665191	7.25E-26	3.55E-25
MYO15B	1.641219	7.005081	7.41E-26	3.63E-25
ZNF296	1.576399	0.651127	7.41E-26	3.63E-25
LCN1	5.959227	-1.78519	7.79E-26	3.81E-25
TMPRSS5	1.839299	-0.8697	7.87E-26	3.85E-25
NLRP3	1.370553	2.685801	8.01E-26	3.92E-25
LRRC17	1.638487	3.11336	8.16E-26	3.99E-25
ZC3HAV1	1.199013	2.919499	8.72E-26	4.26E-25
FILIP1	1.410622	4.402666	8.99E-26	4.39E-25
FBXL6	1.133043	3.515541	9.00E-26	4.39E-25
ZNF841	1.094814	3.595725	9.01E-26	4.40E-25
SLITRK5	3.436597	2.790104	9.15E-26	4.46E-25
HOXA4	1.255741	3.263121	9.40E-26	4.58E-25
BCL11B	1.610155	2.875339	1.03E-25	5.00E-25
ITPR2	-1.19629	5.981791	1.03E-25	5.02E-25
B4GALNT	3.77206	4.234964	1.06E-25	5.15E-25
TBX5	7.179722	-0.0544	1.08E-25	5.26E-25
SVEP1	-1.56296	4.412855	1.11E-25	5.38E-25
GJA4	1.450756	5.892136	1.12E-25	5.43E-25
CHRND	3.299384	-2.47022	1.13E-25	5.50E-25
SSPN	1.18955	5.543993	1.13E-25	5.50E-25
CLK1	1.174008	6.487129	1.14E-25	5.55E-25
ALDH1B1	-1.252	5.697472	1.15E-25	5.61E-25
GIMAP7	1.200331	5.208658	1.18E-25	5.73E-25
ADGRA2	1.352472	6.102518	1.18E-25	5.73E-25
PCDHGC4	1.858062	-1.01493	1.20E-25	5.81E-25
KLF8	1.225209	4.391413	1.21E-25	5.87E-25
GBGT1	1.011122	3.244061	1.21E-25	5.88E-25
BAIAP3	-1.11627	3.408718	1.23E-25	5.97E-25
FRMD7	-3.62253	-0.11966	1.26E-25	6.12E-25
CCL11	-2.42779	-0.47922	1.32E-25	6.39E-25
OMG	4.897793	1.949364	1.33E-25	6.42E-25
SCD5	-1.73629	5.632954	1.36E-25	6.59E-25
INO80C	1.024068	4.388497	1.60E-25	7.70E-25
KCNJ14	1.326305	0.755447	1.60E-25	7.71E-25
GP6	2.248249	-0.31722	1.66E-25	8.01E-25
GPS2	1.487978	2.389991	1.77E-25	8.53E-25
BST2	1.333486	7.153015	1.78E-25	8.57E-25
ASIC3	2.019949	0.22009	1.81E-25	8.69E-25
IL17REL	2.612361	-2.42157	1.85E-25	8.87E-25
CCDC188	1.738042	-0.515	1.86E-25	8.93E-25
LAX1	2.038989	1.413333	2.10E-25	1.01E-24
CCR8	2.765581	-1.59923	2.16E-25	1.03E-24
KRT72	3.542753	-2.98391	2.17E-25	1.04E-24
SRARP	-2.93383	-1.04586	2.19E-25	1.05E-24
PPP1R1A	-2.68399	5.653471	2.23E-25	1.07E-24
MRNIP	1.214274	4.659663	2.24E-25	1.07E-24
ATP2C2	-1.91805	0.655669	2.33E-25	1.12E-24
11-302B1	1.779929	-2.80318	2.43E-25	1.16E-24
CCND2	1.208334	6.170659	2.45E-25	1.17E-24
C19orf33	2.526379	4.830027	2.51E-25	1.20E-24
C4orf50	2.417151	-2.37978	2.53E-25	1.21E-24
FBXO43	1.74883	-0.87151	2.55E-25	1.21E-24
PLEKHG7	-1.45819	-1.426	2.57E-25	1.22E-24
ECSCR	1.281315	3.347245	2.57E-25	1.23E-24
CYP21A2	2.520692	0.82721	2.58E-25	1.23E-24
GPHN	-1.12706	4.602201	2.58E-25	1.23E-24
GAREM2	1.259113	3.230721	2.62E-25	1.25E-24
FBXO41	1.988025	3.102909	2.81E-25	1.34E-24
PLA2G4D	4.393464	-0.83291	2.94E-25	1.40E-24
KCNMB2	-2.071	0.446022	2.97E-25	1.41E-24
AGAP6	1.61453	2.325411	2.97E-25	1.41E-24
SNX10	1.259349	6.778775	3.05E-25	1.45E-24
DGKA	1.136533	4.06687	3.06E-25	1.45E-24
RPE65	2.615866	-2.95849	3.08E-25	1.46E-24
AGAP5	1.733788	-1.5592	3.15E-25	1.49E-24
NOL12	1.085503	2.058669	3.20E-25	1.52E-24
LINGO2	-2.40476	-1.51391	3.24E-25	1.53E-24
CLCNKB	-3.1662	6.034956	3.26E-25	1.55E-24
ZNF157	-1.24668	-1.78324	3.30E-25	1.56E-24
FUT7	1.81371	-1.03852	3.40E-25	1.61E-24
INHBE	3.453104	1.766646	3.57E-25	1.69E-24
ADAMTS4	2.011156	6.387057	3.58E-25	1.69E-24
HYPK	1.403882	1.357325	3.61E-25	1.70E-24
CYP51A1	-1.13582	1.923713	3.65E-25	1.72E-24
C20orf203	2.887774	-1.97488	3.75E-25	1.77E-24
PLA1A	1.649424	5.573576	3.81E-25	1.80E-24
RHEX	1.990396	5.796522	3.98E-25	1.88E-24
H2BC21	-1.19337	4.047725	4.00E-25	1.89E-24
AGBL2	1.496803	1.428637	4.24E-25	1.99E-24
PDE6C	1.730284	-0.88192	4.31E-25	2.03E-24
SLC16A7	-1.91875	5.56845	4.31E-25	2.03E-24
CARD14	2.644031	1.686223	4.40E-25	2.07E-24
CCDC74A	1.512461	3.506033	4.51E-25	2.12E-24
MCF2L	1.167079	6.125288	4.59E-25	2.16E-24
IGFL2	4.289506	-1.20941	4.72E-25	2.22E-24
CCDC194	3.028365	-2.71355	4.78E-25	2.25E-24
TNFAIP2	1.308586	6.543158	4.89E-25	2.29E-24
EDN1	1.785869	5.958266	5.03E-25	2.36E-24
GREM1	-2.4328	2.994702	5.54E-25	2.60E-24
SPRY1	1.350681	6.867882	5.97E-25	2.79E-24
SNAP25	2.027627	2.990441	6.08E-25	2.84E-24
GRIP1	-1.28662	2.038302	6.16E-25	2.88E-24
MAMDC4	1.786457	3.883447	6.28E-25	2.93E-24
ADGRB3	-1.69055	0.054517	6.52E-25	3.04E-24
SLC43A1	-1.28462	3.12573	7.03E-25	3.27E-24
PSTPIP2	1.118546	3.200347	8.11E-25	3.77E-24
PLA2G4C	1.044045	4.461175	8.30E-25	3.86E-24
S100P	-1.92207	-0.72079	8.51E-25	3.96E-24
ZNRF3	-1.27263	-1.11496	8.56E-25	3.98E-24
IL5RA	-1.65725	-1.36329	9.24E-25	4.29E-24
SHANK3	1.241706	6.360037	9.56E-25	4.43E-24
MYO10	-1.08957	6.277072	9.70E-25	4.50E-24
ARPC1B	1.034022	7.001188	1.05E-24	4.86E-24
OR9Q1	3.135222	-2.61555	1.07E-24	4.94E-24
RPGRIP1	1.042969	-0.20424	1.07E-24	4.94E-24
SLC22A15	-1.3009	2.484171	1.13E-24	5.25E-24
KLRB1	1.575058	2.554974	1.18E-24	5.45E-24
CBARP	-1.42147	-0.21803	1.18E-24	5.47E-24
TM4SF19	3.393676	-0.8551	1.19E-24	5.49E-24
LGR5	-2.57881	0.06186	1.20E-24	5.53E-24
NR5A2	1.289796	3.080241	1.24E-24	5.73E-24
16c-431H	2.187587	-1.86317	1.28E-24	5.91E-24
NKAPL	-1.07261	-0.19052	1.31E-24	6.05E-24
EVA1C	1.162463	4.483652	1.35E-24	6.22E-24
DLK1	-2.9768	-1.444	1.36E-24	6.28E-24
NPIPB12	2.117944	-1.37244	1.43E-24	6.59E-24
GIPC3	1.276482	4.289308	1.44E-24	6.65E-24
HTR7	1.774424	0.199598	1.48E-24	6.80E-24
RAB15	-1.01351	3.788828	1.49E-24	6.87E-24
CCER2	2.613445	-1.12036	1.50E-24	6.90E-24
ADAM28	1.312053	4.35468	1.53E-24	7.05E-24
MASP1	2.011983	5.582646	1.55E-24	7.12E-24
CDH6	1.818323	8.283147	1.56E-24	7.16E-24
SPARCL1	1.634961	9.075524	1.58E-24	7.26E-24
TSPAN18	1.345512	6.838251	1.62E-24	7.42E-24
KCNH6	-1.97693	1.821719	1.63E-24	7.49E-24
STAG3	1.300977	2.325973	1.70E-24	7.78E-24
RC4-KLR	2.264668	-1.78039	1.73E-24	7.91E-24
HOXA11	-1.43207	1.208512	1.75E-24	8.01E-24
OPRL1	1.008649	1.488928	1.77E-24	8.09E-24
GLIS1	1.896221	3.627108	1.81E-24	8.27E-24
NCMAP	-1.98713	0.803411	1.89E-24	8.66E-24
CTF1	-1.15988	2.701334	1.94E-24	8.88E-24
GCNA	1.551141	0.395125	1.96E-24	8.96E-24
GLYCTK	-1.0504	3.76125	1.99E-24	9.08E-24
SLC4A1	-3.1979	5.783916	1.99E-24	9.10E-24
CFP	1.32723	1.890389	2.01E-24	9.19E-24
DPEP3	2.23821	-2.16646	2.07E-24	9.47E-24
PTCH2	1.482314	1.264092	2.14E-24	9.75E-24
PLAC8	1.883636	1.982859	2.22E-24	1.01E-23
TLCD4	-1.12356	3.966452	2.27E-24	1.03E-23
C1QL1	3.69368	5.008074	2.29E-24	1.04E-23
PTPN7	1.758076	3.616303	2.46E-24	1.12E-23
TVP23A	1.342568	0.916937	2.56E-24	1.17E-23
H2AC17	2.660404	-2.10899	2.63E-24	1.20E-23
PILRB	2.441056	3.197035	2.69E-24	1.23E-23
FSTL3	1.781152	6.681458	2.74E-24	1.25E-23

TCF7L2	0.51843416	2.43E-38
PAOX	0.5018399	1.16E-35
CNNM2	0.52556253	1.55E-39
PDCD11	0.51311617	1.82E-37
BTBD10	0.55751278	2.90E-45
LIN7C	0.59288091	2.26E-52
PGAP2	0.50175265	1.20E-35
ZNF214	0.52001198	1.33E-38
HSD17B12	0.50749786	1.47E-36
APIP	0.64265284	5.28E-64
EIF3M	0.52435267	2.48E-39
GLYAT	0.56461019	1.27E-46
SSRP1	0.52848136	4.91E-40
ARFGAP2	0.64981616	7.37E-66
CELF1	0.52086906	9.56E-39
ENDOD1	0.61285089	8.61E-57
CAPN5	0.51449968	1.08E-37
INTS4	0.55935525	1.30E-45
PAK1	0.61271915	9.23E-57
ZC3H12C	0.57818579	2.58E-49
TTC12	0.53443353	4.58E-41
NPAT	0.53922502	6.54E-42
AASDHPPT	0.743309	1.19E-95
LAMTOR1	0.55364884	1.54E-44
HYOU1	0.51798173	2.89E-38
KAT14	0.52296286	4.26E-39
TKFC	0.59882092	1.18E-53
CPSF7	0.5130306	1.88E-37
EI24	0.65694335	9.36E-68
LSM14B	0.58142765	5.63E-50
YTHDF1	0.56520669	9.73E-47
MRPL49	0.6965376	2.63E-79
TAOK2	0.54011716	4.54E-42
ARID5B	0.50823844	1.12E-36
TMCO3	0.56553692	8.40E-47
TMEM218	0.57994965	1.13E-49
TIRAP	0.62817759	2.11E-60
LATS2	0.56611113	6.49E-47
SAP18	0.65289621	1.13E-66
HNMT	0.67816847	1.01E-73
PDCD4	0.56909739	1.69E-47
WDR17	0.50873502	9.32E-37
MTMR12	0.65760212	6.21E-68
CCT5	0.54103616	3.11E-42
ATPSCKMT	0.55874453	1.69E-45
DOCK1	0.61685914	1.02E-57
DIXDC1	0.56329665	2.28E-46
DLAT	0.64887051	1.30E-65
NKAPD1	0.63245845	1.90E-61
PIP4K2A	0.53315895	7.64E-41
FREM2	0.65120129	3.18E-66
CRIM1	0.57723848	4.02E-49
RILPL2	0.526687	9.96E-40
UEVLD	0.62740356	3.24E-60
UBE3B	0.56403232	1.64E-46
ANK3	0.64438876	1.89E-64
SLC2A13	0.54826045	1.53E-43
GXYLT1	0.59266187	2.51E-52
DIP2C	0.69926087	3.60E-80
EIF4E	0.57467538	1.32E-48
SRFBP1	0.5706176	8.48E-48
FAM177A1	0.65164904	2.42E-66
EXT2	0.52449561	2.35E-39
TMEM18	0.52989987	2.80E-40
KCTD14	0.54998399	7.37E-44
NUBPL	0.60550856	3.94E-55
NEK7	0.60212788	2.22E-54
VIPAS39	0.60805143	1.06E-55
UPF2	0.54324576	1.25E-42
CDC123	0.54375038	1.01E-42
EPS8	0.58771117	2.80E-51
ACAD8	0.56779701	3.04E-47
VPS26B	0.57253121	3.54E-48
VTI1A	0.54255701	1.66E-42
QDPR	0.644929	1.38E-64
FAM160B1	0.50853076	1.00E-36
TEX9	0.61470064	3.23E-57
QTRT2	0.52733222	7.73E-40
MMAA	0.63802371	7.86E-63
ZNF827	0.52049251	1.11E-38
PIGF	0.68160452	9.81E-75
MFSD6	0.51211196	2.65E-37
FLI1	0.51823493	2.63E-38
WWC2	0.59834395	1.50E-53
ACSL1	0.62853213	1.73E-60
SLC25A4	0.6499444	6.82E-66
SAV1	0.50940369	7.27E-37
NBAS	0.57267497	3.31E-48
GUF1	0.52816721	5.56E-40
BAG3	0.57757275	3.44E-49
FAM168B	0.68908943	5.44E-77
MGAT5	0.60009826	6.20E-54
GPATCH11	0.61981953	2.08E-58
HSPB8	0.51787928	3.01E-38
OBI1	0.58360276	2.01E-50
ARL14EP	0.6258934	7.50E-60
ATP5F1A	0.64524128	1.14E-64
C18orf25	0.52137611	7.87E-39
PDK1	0.5525583	2.46E-44
TGOLN2	0.65248787	1.45E-66
UHMK1	0.56250307	3.24E-46
TADA1	0.72236255	6.47E-88
CWF19L2	0.60836947	8.99E-56
XRCC4	0.52588732	1.36E-39
ZNF776	0.60049485	5.07E-54
SUV39H2	0.52974419	2.98E-40
NMT2	0.595729	5.52E-53
USP12	0.62463118	1.50E-59
CCDC50	0.54820178	1.57E-43
TMEM123	0.53051059	2.20E-40
NADK2	0.60006284	6.31E-54
SLC30A6	0.59841115	1.45E-53
PELO	0.65292217	1.11E-66
RASGRP3	0.53477783	3.98E-41
SAR1B	0.6620689	3.76E-69
GPR180	0.54963672	8.55E-44
FARP1	0.65840333	3.77E-68
IFIT5	0.64885187	1.32E-65
SLC16A12	0.5485077	1.38E-43
PANK1	0.6790338	5.64E-74
HNRNPDL	0.50445986	4.48E-36
PTPRK	0.62140797	8.78E-59
GGPS1	0.64110199	1.31E-63
RAD17	0.66381864	1.24E-69
MED21	0.66371222	1.32E-69
STK32B	0.67087681	1.29E-71
CWC27	0.60173049	2.71E-54
CDYL	0.52931873	3.53E-40
BANK1	0.54082283	3.39E-42
TXNDC11	0.62329558	3.13E-59
DAB2	0.7120844	2.25E-84
ACMSD	0.62818547	2.10E-60
ANAPC1	0.5325043	9.93E-41
SCOC	0.5652149	9.70E-47
CETN3	0.50082401	1.67E-35
SMARCA5	0.62394241	2.20E-59
HNRNPU	0.72154942	1.25E-87
RANBP2	0.56020794	8.92E-46
AHCTF1	0.61116622	2.09E-56
TRAPPC8	0.65163249	2.45E-66
TMEM251	0.55209987	2.99E-44
RMND5A	0.73221599	1.83E-91
RPIA	0.52953745	3.24E-40
CNKSR3	0.57670149	5.16E-49
GTF2E1	0.56827505	2.45E-47
CFDP1	0.59534561	6.68E-53
KCNJ16	0.50556398	2.99E-36
TRIP12	0.58978561	1.02E-51
FBXO36	0.61138758	1.86E-56
DDAH1	0.77039641	7.50E-107
HS2ST1	0.6418027	8.70E-64
NUS1	0.66609894	2.87E-70
PPP2R5E	0.57005567	1.09E-47
SLC5A10	0.5693083	1.54E-47
IMPACT	0.68742455	1.75E-76
TOMM70	0.58715229	3.67E-51
PITPNC1	0.65465614	3.85E-67
C4orf19	0.53618055	2.26E-41
MIA3	0.68999379	2.87E-77
DISP1	0.68019506	2.57E-74
TNIK	0.54221897	1.91E-42
SH3RF1	0.61071196	2.65E-56
BUB3	0.54524144	5.43E-43
ATP5MC3	0.50948638	7.05E-37
SORBS2	0.64609304	6.88E-65
MRPL39	0.58006591	1.07E-49
GABPA	0.62594008	7.31E-60
XPC	0.59473515	9.04E-53
DPH3	0.5127009	2.13E-37
PLCL2	0.59597458	4.89E-53
MPPE1	0.63870361	5.30E-63
ANKRD40	0.71066183	6.75E-84
APOOL	0.62940819	1.06E-60
KLF10	0.58462803	1.23E-50
ATP6V1C1	0.6636174	1.41E-69
CDK19	0.53267852	9.26E-41
AGPAT5	0.60681341	2.01E-55
HSPA13	0.55348392	1.65E-44
USP25	0.6011924	3.57E-54
GRAMD2B	0.55657697	4.35E-45
ZCCHC10	0.59773696	2.03E-53
C16orf87	0.63844772	6.15E-63
LARP1	0.57331327	2.47E-48
CNOT8	0.58215444	3.99E-50
MIER3	0.56386776	1.77E-46
NUP205	0.54749744	2.11E-43
ZKSCAN2	0.52119268	8.44E-39
C9orf85	0.57271693	3.25E-48
KDM8	0.5380861	1.04E-41
KCTD18	0.72291731	4.13E-88

ADCY10	1.878853	-0.31223	2.81E-24	1.27E-23
LY6E	1.700727	8.066735	2.87E-24	1.30E-23
TCF19	1.326893	4.763963	2.93E-24	1.33E-23
CDH5	1.255424	7.328696	2.94E-24	1.33E-23
MNS1	-1.04586	3.008863	2.97E-24	1.35E-23
INKA1	1.346069	2.269114	2.98E-24	1.35E-23
ARMC12	2.016857	-0.98367	3.05E-24	1.38E-23
PBLD	-1.21763	6.647024	3.07E-24	1.39E-23
C10orf99	6.038104	4.163275	3.10E-24	1.40E-23
AF011889.	1.935666	0.353035	3.11E-24	1.41E-23
HOATZ	1.610131	-2.16144	3.11E-24	1.41E-23
TRIM14	1.127382	5.741731	3.14E-24	1.42E-23
CFAP77	-1.65647	-3.19316	3.25E-24	1.47E-23
ANKRD36	1.729798	2.08981	3.31E-24	1.49E-23
GPR34	1.642497	3.982618	3.31E-24	1.50E-23
CELF3	-1.78896	-3.0468	3.75E-24	1.70E-23
MATN2	-1.11987	5.706729	3.85E-24	1.74E-23
KIF2C	1.492921	2.29765	3.92E-24	1.77E-23
STX19	-1.68378	-2.30909	4.02E-24	1.81E-23
COBL	-1.19282	4.976341	4.06E-24	1.83E-23
ARHGAP4	1.110623	5.342738	4.10E-24	1.85E-23
LRRC75B	1.46682	5.186453	4.22E-24	1.90E-23
ADGRL1	-1.00419	4.487374	4.76E-24	2.14E-23
TRNP1	-1.89992	2.838148	4.90E-24	2.20E-23
ISG15	1.63648	5.192943	4.93E-24	2.21E-23
NTN5	1.892928	-1.02532	5.00E-24	2.25E-23
C5orf46	4.909752	3.938902	5.08E-24	2.28E-23
CCR6	2.571947	-1.01201	5.16E-24	2.32E-23
CACNA2D	1.729088	2.812181	5.21E-24	2.34E-23
TFAP2A	-2.07346	2.865207	5.23E-24	2.34E-23
NGFR	1.876342	4.999431	5.29E-24	2.37E-23
SLC2A7	2.224862	-2.70778	5.37E-24	2.41E-23
HPGD	-1.91288	4.446916	5.40E-24	2.42E-23
INO80B	1.097514	1.78854	5.51E-24	2.47E-23
PDE8B	-1.03749	3.662208	5.60E-24	2.50E-23
CYP3A5	2.121603	5.052086	5.63E-24	2.52E-23
SYTL3	1.213544	3.75332	6.12E-24	2.74E-23
HIGD1B	1.682622	2.372886	6.22E-24	2.78E-23
PTAFR	1.427174	4.515515	6.26E-24	2.80E-23
ESCO2	1.515863	0.650726	6.34E-24	2.83E-23
IGSF21	1.566989	1.563044	6.46E-24	2.88E-23
SEC14L3	4.094915	-2.8403	6.51E-24	2.90E-23
MUC17	8.867918	1.370772	6.51E-24	2.90E-23
CYBA	1.243738	7.02389	6.56E-24	2.93E-23
ZNF728	-2.4436	-1.55537	7.68E-24	3.42E-23
FAIM2	-1.56739	0.640832	7.88E-24	3.51E-23
GPR155	-1.10749	4.730718	7.90E-24	3.51E-23
LILRB5	1.732334	2.776263	8.54E-24	3.80E-23
SH2D3C	1.105878	5.528632	8.58E-24	3.81E-23
KCNAB1	1.47495	3.916994	8.61E-24	3.83E-23
IL1RL1	-2.05448	4.002056	8.79E-24	3.91E-23
LYL1	1.101434	2.823173	8.80E-24	3.91E-23
DNER	-2.52464	3.350802	8.96E-24	3.98E-23
DNAH10	-1.02722	0.874765	8.97E-24	3.98E-23
ERMN	2.055611	-0.70113	8.97E-24	3.98E-23
PLAG1	-1.61451	1.304212	9.09E-24	4.03E-23
NEURL1B	1.148998	5.954555	9.16E-24	4.06E-23
BCL6B	1.339164	5.589544	9.20E-24	4.08E-23
H2AC13	3.207987	-0.76437	9.21E-24	4.08E-23
DNAI3	-1.32863	-0.30077	9.32E-24	4.13E-23
ADRA1B	1.425015	3.214643	9.47E-24	4.19E-23
SH3GL2	-2.69006	2.273621	9.59E-24	4.24E-23
FAM43B	-1.59958	0.294951	9.68E-24	4.28E-23
LYNX1	-1.17889	3.837061	9.89E-24	4.37E-23
RNASE3	2.430949	-2.6944	9.93E-24	4.39E-23
C7orf61	1.825289	-1.70116	9.99E-24	4.41E-23
RP1	-1.59608	-1.40122	1.00E-23	4.43E-23
TTYH2	1.116784	3.34648	1.04E-23	4.60E-23
NOTCH3	1.341808	8.317734	1.06E-23	4.65E-23
TCF4	1.197273	6.963877	1.06E-23	4.69E-23
BEST4	2.680864	1.344754	1.09E-23	4.79E-23
CCDC78	2.852445	1.078923	1.11E-23	4.90E-23
DGKB	-1.4407	-0.07476	1.15E-23	5.06E-23
ARG2	-1.90126	4.976311	1.16E-23	5.12E-23
KCNG2	-1.45768	-2.98441	1.21E-23	5.32E-23
FAM186A	1.771512	-0.57359	1.32E-23	5.80E-23
UPK1A	-2.47614	-1.32422	1.32E-23	5.81E-23
XK	-1.86105	0.872925	1.33E-23	5.83E-23
SLC9B2	1.115504	2.709773	1.34E-23	5.88E-23
KLRC1	1.912598	-0.73025	1.36E-23	5.99E-23
LONRF2	-1.8645	2.848168	1.42E-23	6.22E-23
PRSS36	1.080293	1.001213	1.50E-23	6.60E-23
PCSK2	-2.34425	-2.31089	1.51E-23	6.64E-23
GRAMD1I	-1.25345	3.977613	1.54E-23	6.76E-23
CSAD	1.55812	4.248674	1.61E-23	7.05E-23
CDSN	2.750669	-3.15163	1.63E-23	7.15E-23
PROSER2	-1.12644	3.501481	1.78E-23	7.77E-23
KRT85	-1.91806	-3.46324	1.82E-23	7.94E-23
EAF2	-1.0766	2.351078	1.88E-23	8.20E-23
PGBD5	1.784928	4.549529	1.90E-23	8.27E-23
LGALS12	3.512641	2.309065	1.93E-23	8.40E-23
LINC0051	1.89842	-1.6032	1.94E-23	8.45E-23
PLAGL1	1.243449	5.132163	2.10E-23	9.14E-23
NEK2	1.803656	1.272252	2.10E-23	9.14E-23
CLEC14A	1.356126	6.355874	2.11E-23	9.17E-23
WNT4	-1.85526	0.359969	2.34E-23	1.01E-22
GJB1	-1.45693	4.361865	2.46E-23	1.07E-22
BDNF	2.151557	2.127939	2.56E-23	1.11E-22
SPDYA	1.676548	-0.32745	2.83E-23	1.23E-22
RSPH9	1.168062	1.308343	2.91E-23	1.26E-22
RARRES2	1.646076	7.869156	3.16E-23	1.37E-22
GSDMB	1.884981	2.828787	3.30E-23	1.43E-22
ATAD3B	1.440784	3.384039	3.58E-23	1.55E-22
LYZL1	4.171405	-2.55655	3.85E-23	1.66E-22
TTLL3	1.607953	4.093784	4.13E-23	1.78E-22
CEACAM	1.839149	-0.52667	4.20E-23	1.81E-22
TLCD1	-1.24899	2.066212	4.24E-23	1.82E-22
CD38	1.865469	3.074741	4.24E-23	1.82E-22
CCNF	1.008981	2.367004	4.36E-23	1.87E-22
GPR171	1.69501	1.150376	4.43E-23	1.90E-22
LYPD6B	-2.75105	1.712794	4.46E-23	1.92E-22
RASL12	1.260523	4.168891	4.49E-23	1.93E-22
IRX3	1.513587	6.040387	4.49E-23	1.93E-22
FAM153A	3.26051	2.194152	4.50E-23	1.93E-22
PPM1J	-1.16017	0.057399	4.57E-23	1.96E-22
SIX4	-2.20674	0.913748	4.79E-23	2.05E-22
HEY2	1.300647	3.69282	4.93E-23	2.11E-22
PCDHA12	-2.02615	-0.1914	5.01E-23	2.15E-22
PLCXD3	-1.83861	2.95899	5.03E-23	2.15E-22
PNPLA3	-1.5975	0.611459	5.11E-23	2.19E-22
P11-468E2	1.649081	-0.85547	5.25E-23	2.25E-22
ENAM	-1.70954	3.365739	5.46E-23	2.34E-22
TMEM156	1.443723	1.012624	5.76E-23	2.46E-22
MSS51	1.472715	0.46098	5.80E-23	2.48E-22
IRF6	-1.55878	5.06436	6.29E-23	2.68E-22
SLC7A11	1.800717	1.784469	6.42E-23	2.74E-22
IL18R1	1.286054	3.389671	6.48E-23	2.77E-22
SLC51B	-1.51179	1.771864	6.54E-23	2.79E-22
GPR35	2.434266	2.917087	6.69E-23	2.85E-22
ITIH1	6.408883	2.266143	7.01E-23	2.99E-22
KLHL3	-1.61667	3.694471	7.06E-23	3.00E-22
SLFN14	2.448614	-2.93238	7.30E-23	3.11E-22
RGS13	1.846787	-1.0966	7.60E-23	3.23E-22
CACNB4	-1.60328	1.426466	7.73E-23	3.28E-22
FBXO6	1.005993	3.85777	7.76E-23	3.29E-22
NAP1L3	-1.29305	1.778109	7.88E-23	3.34E-22
CCDC116	1.67798	-1.75591	7.95E-23	3.37E-22
SLC16A1(	-1.49394	3.188311	8.39E-23	3.55E-22
HP	7.584149	7.108137	8.44E-23	3.57E-22
DERL3	2.172388	3.362953	8.51E-23	3.60E-22
GOT1	-1.00671	6.846828	8.74E-23	3.70E-22
ORC6	1.365944	1.075624	8.82E-23	3.73E-22
KAT2A	1.141095	5.37156	8.85E-23	3.75E-22
MUC1	-1.52928	6.930409	8.93E-23	3.78E-22
ADRA2C	-1.4236	2.981729	8.98E-23	3.80E-22
TC-360G5	2.631986	-3.35122	9.18E-23	3.88E-22
CDC20	1.718744	2.561335	9.25E-23	3.91E-22
T6GALNA(	-1.45305	3.053773	9.46E-23	4.00E-22
DSP	-1.4334	6.960665	9.97E-23	4.21E-22
DNAH1	1.386734	3.802407	1.00E-22	4.22E-22
APOL1	2.065774	8.438985	1.01E-22	4.24E-22
MPEG1	1.328241	5.952407	1.02E-22	4.29E-22
HX1-C8orf	1.050082	-0.78672	1.08E-22	4.56E-22
ERICH6B	1.701745	-1.70482	1.16E-22	4.88E-22
H2BC13	2.764068	-2.52027	1.16E-22	4.89E-22
IL2RA	2.226176	2.199102	1.33E-22	5.59E-22
CACNB1	1.310222	2.185286	1.37E-22	5.76E-22
EGFL8	1.889437	0.865608	1.39E-22	5.82E-22
SKIDA1	-1.25832	1.056131	1.40E-22	5.87E-22
MYH14	-1.75778	5.122502	1.40E-22	5.89E-22
CXCL12	-1.23027	6.80094	1.41E-22	5.91E-22
RGPD3	-1.53984	-1.93899	1.41E-22	5.91E-22
LRFN1	1.204771	0.957615	1.44E-22	6.04E-22
CHST13	2.019012	3.383126	1.54E-22	6.45E-22
SARDH	-1.22559	3.723819	1.56E-22	6.52E-22
AP1G2	1.15888	4.739069	1.56E-22	6.53E-22
KCNJ5	-1.39533	4.017802	1.60E-22	6.70E-22
KCNIP2	1.73136	0.877351	1.64E-22	6.83E-22
PHF21B	-2.24817	-1.17529	1.71E-22	7.13E-22
IFI27	1.48144	7.046652	1.75E-22	7.30E-22
SNRNP70	1.002359	6.998089	1.76E-22	7.34E-22
LPL	-1.2187	5.303258	1.79E-22	7.45E-22
ZP3	1.562216	2.333267	1.89E-22	7.85E-22
KCNH4	1.572992	-1.10147	1.96E-22	8.14E-22
SOWAHB	-1.30096	3.406239	2.00E-22	8.30E-22
LINC0067	1.1698	0.239831	2.06E-22	8.55E-22
IQGAP2	-1.07484	5.886204	2.14E-22	8.89E-22
DHRS9	1.962474	0.936289	2.15E-22	8.91E-22
WNT1	2.376101	-3.24567	2.15E-22	8.93E-22
LAMA2	-1.50493	3.6435	2.20E-22	9.14E-22
FFAR2	1.772145	-0.8843	2.25E-22	9.31E-22
X16-NPEF	2.004462	0.214194	2.29E-22	9.50E-22
RPS15	1.014405	8.005022	2.34E-22	9.68E-22
GPR183	1.481269	3.899102	2.36E-22	9.78E-22
GPR62	-1.56457	-1.93221	2.41E-22	9.98E-22
CD163	1.878946	6.532651	2.42E-22	1.00E-21
OASL	1.575034	3.366658	2.47E-22	1.02E-21
CXCL5	4.736652	3.586761	2.49E-22	1.03E-21
BMX	1.536891	1.499622	2.52E-22	1.04E-21
P2RY10	1.955699	1.398892	2.59E-22	1.07E-21

FAM126B	0.5587217	1.71E-45
RNF20	0.70165223	6.16E-81
SLC26A2	0.51406417	1.28E-37
MED7	0.52924848	3.63E-40
RRAGA	0.63623067	2.21E-62
RMND1	0.53494392	3.72E-41
VBP1	0.62506542	1.18E-59
VPS37A	0.58411253	1.58E-50
CARNMT1	0.62912729	1.24E-60
MCU	0.5137185	1.45E-37
FAM161B	0.65230032	1.63E-66
GNAQ	0.53572465	2.72E-41
ADK	0.52874108	4.44E-40
DCK	0.50659928	2.05E-36
DPY19L4	0.52331413	3.72E-39
C8orf37	0.64636163	5.86E-65
SLC28A1	0.52851604	4.85E-40
RWDD2B	0.557858	2.49E-45
USP16	0.56499843	1.07E-46
CCT8	0.59465071	9.42E-53
SCAF4	0.54188212	2.19E-42
CDK20	0.5896994	1.07E-51
SFXN2	0.64363673	2.96E-64
FUT6	0.57389447	1.89E-48
SUPV3L1	0.54768126	1.95E-43
EEF1A1	0.56452549	1.32E-46
ZDHHC5	0.65789914	5.16E-68
ZFAND3	0.66564934	3.83E-70
NPTN	0.54554226	4.79E-43
KAT6B	0.52496906	1.95E-39
SAMD8	0.5449693	6.09E-43
AIFM1	0.56348021	2.10E-46
BAG4	0.5144439	1.11E-37
ZNF689	0.66405632	1.06E-69
MFSD14A	0.67535192	6.69E-73
VPS8	0.5243575	2.48E-39
GALK2	0.77162937	2.13E-107
PDE6D	0.53532226	3.20E-41
NTAN1	0.6106886	2.68E-56
ZFYVE9	0.72258567	5.40E-88
FCHO2	0.61714858	8.76E-58
TMEM171	0.54831464	1.49E-43
ODR4	0.68702344	2.32E-76
CPT2	0.74946187	4.55E-98
NECAP2	0.59250301	2.72E-52
STEAP2	0.51499659	8.98E-38
CLDN12	0.52494244	1.97E-39
FZD1	0.64799734	2.20E-65
DHRS4	0.59378634	1.44E-52
DDX19B	0.55163861	3.65E-44
FCSK	0.51460081	1.04E-37
AASDH	0.59413271	1.22E-52
RNF111	0.63334059	1.15E-61
APPL1	0.58370499	1.91E-50
VPS26C	0.75447338	4.33E-100
DYRK1A	0.52153426	7.40E-39
KCNJ15	0.58093685	7.10E-50
ETS2	0.5365472	1.95E-41
TSPAN18	0.61153564	1.72E-56
SLC35B2	0.55170433	3.55E-44
C2CD2	0.50622354	2.35E-36
TAB3	0.53485361	3.86E-41
BRAF	0.55274042	2.28E-44
AP3S2	0.63573333	2.94E-62
RAB28	0.5322493	1.10E-40
PRXL2B	0.53425155	4.92E-41
PANK4	0.5191906	1.82E-38
C12orf43	0.57799259	2.83E-49
PEX10	0.61851852	4.20E-58
RER1	0.57443041	1.48E-48
AGAP1	0.61715835	8.72E-58
PAFAH2	0.68173871	8.95E-75
BABAM2	0.5433527	1.19E-42
NCK1	0.62870648	1.57E-60
TPRG1L	0.70391644	1.14E-81
PRXL2C	0.58785322	2.61E-51
CNNM4	0.5302296	2.46E-40
EYA3	0.5750454	1.11E-48
FANCC	0.69179096	8.01E-78
WASF2	0.54174795	2.32E-42
CUL4B	0.55692331	3.75E-45
GPRASP2	0.61483623	3.01E-57
SHROOM4	0.54386288	9.65E-43
H4C8	0.52211355	5.92E-39
B4GALT5	0.56834074	2.38E-47
SPATA2	0.54260013	1.63E-42
TSR2	0.51604998	6.03E-38
ZC3H18	0.52537399	1.67E-39
TMED4	0.52865614	4.59E-40
PPP1R15B	0.67285567	3.51E-72
EMSY	0.50938684	7.32E-37
GPAT4	0.50268934	8.52E-36
ELK4	0.50171075	1.21E-35
F11R	0.59254771	2.66E-52
NIT1	0.54335829	1.19E-42
DEDD	0.59348904	1.67E-52
PINK1	0.66944651	3.29E-71
B4GALT3	0.54797621	1.72E-43
NDUFS2	0.68794034	1.22E-76
TOMM40L	0.57229595	3.94E-48
CCAR2	0.53374775	6.03E-41
CDC42SE2	0.6593551	2.08E-68
MIS18A	0.51640471	5.27E-38
ALG8	0.56821652	2.52E-47
SYNJ1	0.62668876	4.83E-60
MRPL10	0.54830215	1.50E-43
GART	0.60528451	4.42E-55
SON	0.57975375	1.24E-49
RCAN1	0.519864	1.41E-38
UBE2Z	0.55439766	1.12E-44
HLCS	0.51815794	2.70E-38
ADIPOR1	0.7275131	9.47E-90
CYB5R1	0.74290614	1.70E-95
THEM4	0.51366663	1.48E-37
UBR1	0.59203175	3.42E-52
MED8	0.5549312	8.88E-45
RSPRY1	0.57420302	1.64E-48
GPBP1L1	0.66065501	9.18E-69
NAE1	0.5915983	4.23E-52
EFCAB14	0.66079897	8.39E-69
ATP6V0D1	0.51529686	8.01E-38
CCDC117	0.56636439	5.80E-47
ZNF230	0.51665275	4.79E-38
GNE	0.70476583	6.01E-82
ARHGAP35	0.5516611	3.61E-44
CALM3	0.65799573	4.86E-68
DFFA	0.63123455	3.80E-61
BSDC1	0.66981955	2.58E-71
SSU72	0.51735392	3.67E-38
VMA21	0.53584293	2.59E-41
ABCG1	0.50331388	6.80E-36
SLC37A1	0.50191248	1.13E-35
PKNOX1	0.51717217	3.94E-38
AGPAT3	0.66846302	6.23E-71
LSS	0.5110089	4.01E-37
MCM3AP	0.57707947	4.33E-49
PRMT2	0.54235402	1.80E-42
ZER1	0.59000059	9.23E-52
TAOK1	0.53609007	2.34E-41
SIK3	0.59604097	4.73E-53
CHTOP	0.51268691	2.14E-37
ZBTB7B	0.61014428	3.57E-56
VPS11	0.73804845	1.23E-93
NLRX1	0.56072888	7.09E-46
ADAR	0.65467728	3.80E-67
IL6R	0.65365671	7.11E-67
UBE2Q1	0.66660867	2.07E-70
FDPS	0.72075367	2.38E-87
SLC25A44	0.75616003	8.82E-101
UBQLN4	0.61591505	1.69E-57
GPATCH4	0.56562799	8.06E-47
SQSTM1	0.53163126	1.41E-40
NAPEPLD	0.65761544	6.16E-68
PSMC2	0.5337835	5.94E-41
ABCF3	0.5150341	8.85E-38
PCYT1A	0.58277585	2.98E-50
PGAP3	0.56245045	3.32E-46
ACOX1	0.72449635	1.14E-88
PRPSAP1	0.63444618	6.14E-62
RAVER1	0.53391692	5.63E-41
TNFSF13	0.63972164	2.93E-63
SENP3	0.56130012	5.51E-46
ADCY9	0.72351706	2.53E-88
CLPB	0.54049284	3.89E-42
CYB561A3	0.5823712	3.60E-50
ASRGL1	0.54861706	1.32E-43
STX5	0.61317187	7.27E-57
LRP5	0.62156549	8.06E-59
TPCN2	0.53054907	2.17E-40
CYP4A22	0.51457247	1.05E-37
CMPK1	0.62912914	1.24E-60
COA7	0.71286066	1.23E-84
ZYG11B	0.61857042	4.08E-58
PARS2	0.62132568	9.18E-59
USP24	0.54986268	7.76E-44
PLPP3	0.70342359	1.65E-81
NOL9	0.50764881	1.39E-36
PRKAA2	0.65963967	1.74E-68
ZSWIM5	0.65146962	2.70E-66
GMEB1	0.54016686	4.44E-42
AK4	0.62047076	1.46E-58
JAK1	0.69417198	1.46E-78
RAVER2	0.65496763	3.18E-67
LZIC	0.70480856	5.82E-82
PEF1	0.59122269	5.08E-52
RBBP4	0.6900306	2.80E-77
NFIA	0.631908	2.59E-61
TM2D1	0.60542299	4.12E-55
USP1	0.57407714	1.74E-48
FUBP1	0.58720686	3.57E-51

TRIM73	2.024205	-1.84937	2.63E-22	1.09E-21
TP53I11	1.092993	7.051997	2.64E-22	1.09E-21
FBLN1	-1.53994	5.597383	2.70E-22	1.11E-21
ANGPTL2	1.434495	6.542339	2.77E-22	1.14E-21
FGF20	2.081986	-2.05318	2.82E-22	1.16E-21
LRRC39	2.128272	0.696159	2.89E-22	1.19E-21
NPEPL1	1.504418	4.507472	2.97E-22	1.22E-21
SEPTIN4	1.153608	5.181991	3.09E-22	1.27E-21
SMOX	1.12894	4.30802	3.12E-22	1.28E-21
MEIS3	1.459245	2.83077	3.16E-22	1.30E-21
SKOR1	1.214096	-0.84935	3.32E-22	1.36E-21
PRR22	1.437236	1.121307	3.32E-22	1.36E-21
YJEFN3	2.114796	1.677482	3.49E-22	1.43E-21
HLA-DME	1.097029	6.34809	3.55E-22	1.45E-21
FAM72D	1.759443	-2.39798	3.60E-22	1.47E-21
IRF1	1.123164	6.424507	3.62E-22	1.48E-21
ANK1	2.248034	3.500766	3.81E-22	1.56E-21
NODAL	1.579897	-2.10494	3.83E-22	1.57E-21
RPL35	1.134229	8.515483	3.83E-22	1.57E-21
TLR10	1.842629	0.997181	3.86E-22	1.58E-21
GPR150	2.204428	-1.57836	3.91E-22	1.60E-21
SLC28A1	1.921495	7.107337	3.98E-22	1.62E-21
CLDN4	-1.19113	6.880211	4.08E-22	1.67E-21
PMP22	1.011663	6.372941	4.09E-22	1.67E-21
RPS5	1.011583	9.031509	4.30E-22	1.75E-21
CYSLTR1	1.156027	2.47584	4.33E-22	1.77E-21
OXGR1	-2.62527	0.932801	4.42E-22	1.80E-21
CGB7	2.306898	-2.64994	4.58E-22	1.87E-21
ACAT1	-1.18766	7.564365	4.66E-22	1.90E-21
CCDC154	2.304419	-0.09643	4.70E-22	1.91E-21
SFTA2	-2.50447	-0.82864	4.80E-22	1.95E-21
PAEP	9.258668	3.206053	4.81E-22	1.96E-21
MALRD1	-1.8832	-0.53263	5.24E-22	2.13E-21
PLP1	-2.07968	-0.8469	5.25E-22	2.13E-21
SH3BGRL	1.132566	7.292679	5.38E-22	2.19E-21
FAM83E	-2.36812	-1.30555	5.39E-22	2.19E-21
CEBPA	1.491761	3.383533	5.47E-22	2.22E-21
ELAVL4	1.730703	-1.63719	5.67E-22	2.30E-21
PRLR	-1.78733	4.70018	5.85E-22	2.37E-21
C9orf152	-1.77919	-2.12542	5.94E-22	2.41E-21
CCDC192	1.200774	-1.41662	6.08E-22	2.46E-21
VNN2	1.799848	3.200237	6.09E-22	2.46E-21
PRSS37	2.599589	-2.32026	6.17E-22	2.50E-21
OBP2A	3.965982	-2.96008	6.47E-22	2.62E-21
PNPLA7	1.569636	3.962773	6.52E-22	2.64E-21
IPCEF1	-1.00842	2.963151	6.66E-22	2.69E-21
LHX2	4.705909	-0.79242	6.66E-22	2.69E-21
MGAT4C	-2.00997	-1.74118	6.77E-22	2.73E-21
CAPN11	2.164665	1.087483	6.85E-22	2.76E-21
JD7-PLA2	1.555328	1.896478	7.37E-22	2.97E-21
MFSD3	-1.05156	3.594404	7.77E-22	3.13E-21
NOVA2	1.170374	3.986472	7.78E-22	3.14E-21
AKR7A3	-1.51836	4.409691	7.81E-22	3.15E-21
GSC	1.876074	-1.81949	7.82E-22	3.15E-21
DPP7	1.001899	7.143024	8.15E-22	3.28E-21
TMEM116	-1.13159	4.186622	8.25E-22	3.32E-21
RS1	-1.30011	-1.9668	8.28E-22	3.33E-21
KLRC2	2.444148	-2.65403	8.30E-22	3.34E-21
RBBP8NL	-3.23054	-1.66438	8.35E-22	3.36E-21
SHISA6	-1.66843	1.483118	8.41E-22	3.38E-21
PLA2G2D	3.486322	1.545832	8.68E-22	3.49E-21
KIAA0408	2.143934	-2.22684	8.77E-22	3.52E-21
PABPC4L	1.295923	3.296544	8.84E-22	3.55E-21
FYB2	-2.08431	1.868286	8.84E-22	3.55E-21
BCL11A	1.553154	1.790643	8.96E-22	3.60E-21
GOLGA8M	1.937406	-0.40626	9.02E-22	3.62E-21
ZNF385D	-1.30541	2.161712	9.26E-22	3.71E-21
TSPYL2	1.180625	5.422688	9.31E-22	3.73E-21
AMY2B	1.772792	3.255681	9.35E-22	3.75E-21
RETN	2.616741	-1.88047	9.69E-22	3.88E-21
RPS28	1.018935	7.817279	9.70E-22	3.88E-21
SLC2A14	2.379735	-0.35451	9.93E-22	3.97E-21
FOXF1	1.392733	2.943937	1.00E-21	4.00E-21
HAUS7	1.983035	2.737678	1.02E-21	4.07E-21
RRAD	1.869167	6.080906	1.02E-21	4.07E-21
C11orf54	-1.05688	7.618349	1.04E-21	4.16E-21
ALOX12B	1.945953	-0.16179	1.06E-21	4.24E-21
SPDYE5	1.642132	-0.66428	1.10E-21	4.38E-21
FAM118A	1.0983	3.979877	1.10E-21	4.40E-21
GPR85	1.508294	1.955481	1.11E-21	4.43E-21
U2AF1L4	1.149243	2.96393	1.12E-21	4.45E-21
VEGFC	1.14081	3.979746	1.14E-21	4.54E-21
OTOG	2.200478	-1.25697	1.17E-21	4.66E-21
HECW2	1.446205	5.496312	1.23E-21	4.90E-21
NOX4	-1.29354	5.415035	1.27E-21	5.04E-21
AFF3	1.480254	4.353664	1.27E-21	5.07E-21
LHX8	5.780251	-0.52193	1.32E-21	5.25E-21
EDNRA	1.255121	5.119209	1.34E-21	5.32E-21
ERC2	-1.63245	-0.60588	1.35E-21	5.35E-21
CYP4B1	-1.57245	0.328549	1.45E-21	5.74E-21
CRYBB2	1.760895	-1.80008	1.48E-21	5.88E-21
KLC3	-1.99839	0.432466	1.56E-21	6.20E-21
BAAT	5.505636	3.707139	1.61E-21	6.36E-21
COL5A1	2.051868	6.950042	1.64E-21	6.48E-21
PLAUR	1.562422	4.496756	1.64E-21	6.48E-21
MEX3B	1.190836	1.587622	1.68E-21	6.63E-21
VNN3	2.565578	-1.3513	1.68E-21	6.66E-21
CCL3	1.843602	2.947634	1.75E-21	6.93E-21
PPP1R35	1.044215	3.530573	1.76E-21	6.94E-21
STEAP1B	2.26136	-0.62465	1.91E-21	7.54E-21
PRND	3.288366	2.552051	1.91E-21	7.55E-21
ERCC6L	1.554085	0.016951	2.04E-21	8.03E-21
FXYD7	1.691357	-2.18557	2.18E-21	8.59E-21
TTC16	1.657284	0.16794	2.26E-21	8.89E-21
GLB1L	1.164982	5.671065	2.48E-21	9.75E-21
CEBPE	1.489293	-2.69169	2.60E-21	1.02E-20
SORCS1	-2.22283	0.906989	2.64E-21	1.04E-20
PROC	-1.92813	2.267201	2.78E-21	1.09E-20
CLVS2	3.547148	-0.44894	2.81E-21	1.10E-20
BACH2	-1.08101	1.356884	2.93E-21	1.15E-20
REELD1	2.313591	-2.03716	2.95E-21	1.16E-20
SNX22	1.256202	1.987989	2.96E-21	1.16E-20
SIGLEC14	1.636586	2.057452	3.07E-21	1.20E-20
GCM1	-1.99437	-0.60268	3.11E-21	1.22E-20
ZDHHC15	-1.28216	1.997242	3.13E-21	1.23E-20
GOLGA8E	2.02099	4.1689	3.18E-21	1.25E-20
AMH	2.703137	-0.09032	3.19E-21	1.25E-20
POU5F2	3.961443	-1.26619	3.23E-21	1.26E-20
ENGASE	1.062985	4.438613	3.25E-21	1.27E-20
RASSF6	1.26484	5.574609	3.26E-21	1.27E-20
TRIM46	1.933758	1.06669	3.29E-21	1.29E-20
GRAP	1.08028	1.612535	3.31E-21	1.29E-20
SLC25A37	1.025107	5.707927	3.33E-21	1.30E-20
CRP	6.728741	2.975201	3.38E-21	1.32E-20
P11-164J1	1.753992	1.178587	3.53E-21	1.38E-20
RCN3	1.515515	4.601211	3.60E-21	1.40E-20
CRLF2	2.39673	-2.28538	3.69E-21	1.44E-20
CRLF2.1	2.39673	-2.28538	3.69E-21	1.44E-20
ATP6V1A	-1.12906	7.615877	3.78E-21	1.47E-20
TMEM174	-2.22532	4.583309	3.80E-21	1.48E-20
CEBPB	1.408847	5.495546	3.89E-21	1.52E-20
KLRC4	2.266509	-2.55932	4.05E-21	1.58E-20
GP9	2.253967	-2.66518	4.07E-21	1.58E-20
FAM9C	2.066079	-2.35754	4.32E-21	1.68E-20
TEDC1	1.13798	2.320962	4.56E-21	1.77E-20
IRAG2	1.248923	4.540802	4.68E-21	1.82E-20
AC138969.	1.711107	-0.95493	4.69E-21	1.82E-20
SLC5A5	2.726576	-2.21563	4.73E-21	1.84E-20
CX3CL1	1.006331	7.839444	4.74E-21	1.84E-20
SYN3	-1.09543	-0.27711	4.87E-21	1.89E-20
TUBA8	1.419029	-1.58292	4.88E-21	1.89E-20
LOXL4	-1.14392	3.917105	5.10E-21	1.98E-20
SERPINE3	1.419328	-1.49716	5.14E-21	1.99E-20
TBC1D3B	2.201127	-2.954	5.18E-21	2.01E-20
JPH2	1.682514	4.073126	5.21E-21	2.02E-20
EGFLAM	1.488282	3.795417	5.52E-21	2.14E-20
SCN2B	-2.25975	-0.08137	5.54E-21	2.15E-20
PRSS51	1.92066	-0.89562	5.66E-21	2.19E-20
CCDC81	1.356144	-0.05748	5.71E-21	2.21E-20
MALL	1.657505	2.191798	5.82E-21	2.25E-20
KCNQ5	1.799527	-0.04258	5.89E-21	2.28E-20
NPY1R	-1.18949	4.964902	6.06E-21	2.34E-20
HAVCR2	1.858351	6.349251	6.22E-21	2.40E-20
CUZD1	1.345634	0.476199	6.33E-21	2.44E-20
SHC2	1.329748	4.47156	6.46E-21	2.49E-20
XYLB	-1.0517	3.093603	6.92E-21	2.67E-20
PT2-EGFL	1.199622	0.244871	7.29E-21	2.81E-20
TMEM179	3.475941	1.994981	7.65E-21	2.95E-20
UTS2	2.741933	-1.48016	7.95E-21	3.06E-20
EPSTI1	1.112689	4.82632	8.09E-21	3.11E-20
VCAN	1.90794	8.414142	8.11E-21	3.12E-20
RERGL	-1.55373	1.904759	8.11E-21	3.12E-20
PTGFR	-1.6828	2.403115	8.25E-21	3.17E-20
IFI44L	1.427618	5.67574	8.53E-21	3.28E-20
IL20RB	3.950299	4.574765	8.62E-21	3.31E-20
OSBPL6	-1.373	1.129293	8.70E-21	3.34E-20
KCNJ15	-1.33723	7.367281	9.02E-21	3.46E-20
EBF3	1.679419	2.257926	9.16E-21	3.51E-20
LGALS9C	1.719023	-3.00986	9.20E-21	3.53E-20
ADGRD1	-1.30086	1.475174	9.56E-21	3.66E-20
A3GALT2	2.197554	-2.96042	9.61E-21	3.68E-20
VIP	2.098065	-0.61012	9.95E-21	3.81E-20
CCN2	-1.18727	7.809671	1.03E-20	3.93E-20
HIGD1A	-1.20061	5.551653	1.11E-20	4.25E-20
SDR42E1	-1.26573	3.0005	1.12E-20	4.28E-20
ZNF804B	-2.81181	-1.28961	1.14E-20	4.34E-20
SAXO2	-1.02498	-0.03476	1.20E-20	4.56E-20
SPHK1	1.688421	2.965647	1.26E-20	4.79E-20
BCAN	2.057403	1.57126	1.27E-20	4.84E-20
DIRAS1	-2.19818	1.565766	1.31E-20	4.99E-20
ANKRD33	3.192324	-2.27592	1.34E-20	5.12E-20
WDR27	1.334826	3.706441	1.37E-20	5.22E-20
CRISP2	-3.33163	-2.57348	1.42E-20	5.42E-20
IL24	1.153577	0.706627	1.44E-20	5.47E-20
TTN	1.790505	4.014146	1.46E-20	5.56E-20
PHGDH	-1.39077	5.323262	1.49E-20	5.67E-20
C2	2.198846	5.528657	1.55E-20	5.89E-20
PI16	-2.27039	0.910626	1.59E-20	6.03E-20
FOXS1	1.589803	2.810747	1.69E-20	6.41E-20
EN1	3.040032	-0.97885	1.69E-20	6.42E-20

DNAJB4	0.62977597	8.62E-61
ADGRL4	0.51475883	9.82E-38
TYW3	0.69455706	1.10E-78
SNX7	0.62516032	1.12E-59
AGL	0.59623064	4.30E-53
VCAM1	0.54379011	9.95E-43
EXTL2	0.60650954	2.36E-55
SLC30A7	0.59140026	4.66E-52
ZNF281	0.52328056	3.77E-39
ZNF496	0.56334569	2.23E-46
PEA15	0.55711613	3.44E-45
PEX19	0.75564231	1.44E-100
NCSTN	0.60280373	1.57E-54
C1orf74	0.67693868	2.32E-73
RBM15	0.55460306	1.02E-44
BPNT1	0.77944923	6.05E-111
C1orf115	0.70559731	3.21E-82
BROX	0.69390134	1.77E-78
TFB2M	0.63116762	3.94E-61
CNST	0.66378101	1.27E-69
PPP1R21	0.73998653	2.25E-94
MAPKAPK2	0.62039205	1.52E-58
FCAMR	0.52597126	1.32E-39
WDR26	0.56502363	1.06E-46
PEX13	0.68821546	1.01E-76
MAIP1	0.76062252	1.23E-102
SLC66A3	0.56778726	3.06E-47
ARL5A	0.57402242	1.78E-48
KCNJ3	0.57470971	1.30E-48
DUSP19	0.54801383	1.70E-43
CFAP36	0.54776049	1.89E-43
NUP35	0.62695749	4.16E-60
WDCP	0.59368286	1.52E-52
SMC6	0.51677907	4.57E-38
H3-3A	0.52324638	3.82E-39
COQ8A	0.59778524	1.98E-53
SGCB	0.76726947	1.76E-105
SPATA18	0.66229773	3.25E-69
NOSTRIN	0.53941617	6.05E-42
SGPP2	0.51055447	4.74E-37
SMARCAD1	0.61251684	1.03E-56
RPRD2	0.72683286	1.66E-89
PACRGL	0.51025322	5.31E-37
LYSMD1	0.59867436	1.27E-53
VPS72	0.62442131	1.69E-59
IWS1	0.5511661	4.46E-44
DHX57	0.66236003	3.13E-69
CCNYL1	0.72187	9.65E-88
FZD5	0.66312452	1.92E-69
GNPDA2	0.5826014	3.23E-50
ANTXR2	0.54232702	1.82E-42
HELQ	0.61318145	7.23E-57
MRPS18C	0.63298054	1.41E-61
CGGBP1	0.51448296	1.09E-37
ABRAXAS1	0.53669068	1.84E-41
PMVK	0.5721997	4.12E-48
PBXIP1	0.58577536	7.11E-51
PYGO2	0.53008965	2.60E-40
HIPK1	0.57898936	1.77E-49
YY1AP1	0.58940658	1.23E-51
EIF4E3	0.60911058	6.12E-56
LRRC58	0.61600447	1.62E-57
TMEM183A	0.66567742	3.76E-70
CCT3	0.64719627	3.56E-65
CWC22	0.68228404	6.16E-75
AZI2	0.51577545	6.69E-38
TGFBR2	0.57917646	1.63E-49
GLB1L	0.59339243	1.75E-52
CHCHD4	0.53795188	1.10E-41
CLASP2	0.54915837	1.05E-43
SUCLG1	0.63612572	2.35E-62
PRKCI	0.51677825	4.57E-38
SLC2A2	0.57406423	1.75E-48
RYBP	0.54519507	5.54E-43
NEPRO	0.52865687	4.59E-40
CDS1	0.58156928	5.27E-50
WDFY3	0.65336666	8.49E-67
COX18	0.50511156	3.53E-36
PTPN13	0.54226716	1.87E-42
SMIM14	0.5574609	2.96E-45
ABHD6	0.66931261	3.59E-71
RBM47	0.65124743	3.09E-66
RCHY1	0.67388644	1.77E-72
TM4SF18	0.5540676	1.29E-44
SNRK	0.58098215	6.95E-50
SLC4A1AP	0.66862014	5.63E-71
KIAA1143	0.50013795	2.14E-35
WDR43	0.57586497	7.61E-49
LZTFL1	0.55866382	1.75E-45
ZNF148	0.58888529	1.59E-51
SMIM12	0.63494709	4.61E-62
MEAF6	0.5829648	2.72E-50
SNIP1	0.62859876	1.67E-60
SENP2	0.56782202	3.01E-47
IFT122	0.54046054	3.94E-42
SFMBT1	0.50725521	1.61E-36
LRPAP1	0.51087153	4.22E-37
UBXN7	0.506027	2.53E-36
PIGX	0.61729966	8.08E-58
EXO5	0.60460626	6.27E-55
C1orf50	0.55382109	1.43E-44
ERMAP	0.51737983	3.64E-38
AIMP1	0.64285947	4.67E-64
SGMS2	0.55604528	5.48E-45
METAP1	0.60285769	1.53E-54
DNAJB14	0.58134414	5.86E-50
EMCN	0.51474899	9.85E-38
BDH2	0.57776303	3.15E-49
PGRMC2	0.5230624	4.10E-39
RNF123	0.53585469	2.58E-41
HSPA4L	0.61603889	1.59E-57
MFSD8	0.65281012	1.19E-66
C4orf3	0.68582292	5.35E-76
ANXA5	0.5006034	1.81E-35
GUCY1A1	0.51330856	1.69E-37
FBXO8	0.65876759	3.00E-68
TMEM144	0.54141063	2.66E-42
NAA15	0.65655406	1.19E-67
FAM160A1	0.50557265	2.98E-36
ARFIP1	0.62121811	9.74E-59
ANAPC10	0.58480181	1.13E-50
ABCE1	0.63207229	2.36E-61
LSM6	0.55730542	3.17E-45
TMEM184C	0.6393076	3.73E-63
PRMT9	0.62932293	1.11E-60
MOCS2	0.57313622	2.68E-48
LMBRD2	0.53781955	1.16E-41
SLC25A46	0.57969058	1.28E-49
PGGT1B	0.58545231	8.30E-51
CMBL	0.7204755	2.97E-87
PRRC1	0.64056044	1.80E-63
F2RL1	0.53137491	1.56E-40
AGGF1	0.65671683	1.08E-67
NDUFS4	0.55343709	1.69E-44
ARSK	0.54884041	1.20E-43
TIGD6	0.54192578	2.15E-42
SERINC5	0.55065014	5.56E-44
PRIMPOL	0.5155928	7.16E-38
CFAP97	0.65288662	1.14E-66
TMEM174	0.52537414	1.67E-39
UBLCP1	0.52628041	1.17E-39
TLR3	0.63974802	2.89E-63
GFM2	0.61852938	4.17E-58
CREBRF	0.519279	1.76E-38
SFXN1	0.67515648	7.63E-73
TMEM200A	0.68662241	3.07E-76
PDSS2	0.68446398	1.37E-75
RAET1E	0.51506502	8.75E-38
KIAA0895	0.50996246	5.91E-37
SAP30L	0.64929324	1.01E-65
COG5	0.54233117	1.82E-42
BMT2	0.52607401	1.27E-39
SLU7	0.59285743	2.28E-52
KCNK5	0.58691368	4.11E-51
ZNF12	0.51913567	1.86E-38
STEAP1	0.50378345	5.73E-36
ZNF704	0.6332603	1.21E-61
CHMP4C	0.51248891	2.30E-37
LMTK2	0.60043157	5.24E-54
PEX2	0.54102829	3.12E-42
RAD21	0.62950704	1.00E-60
DNAAF5	0.5410833	3.05E-42
GPR146	0.50551736	3.04E-36
SLC4A2	0.54476107	6.64E-43
PHAX	0.66238768	3.07E-69
ALDH7A1	0.73470742	2.19E-92
SLC25A32	0.52473127	2.14E-39
DCAF13	0.51894836	2.00E-38
VIRMA	0.52893609	4.11E-40
WASHC5	0.62524783	1.07E-59
SNAPC3	0.63188557	2.63E-61
MYORG	0.5755177	8.94E-49
PSIP1	0.60129216	3.39E-54
UBAP1	0.56658805	5.24E-47
DIRAS2	0.67531128	6.88E-73
FXN	0.50972862	6.45E-37
ALDH1A1	0.55798828	2.36E-45
KDM1B	0.60454258	6.47E-55
RASEF	0.58327449	2.35E-50
C9orf64	0.70954914	1.59E-83
HNRNPK	0.75824436	1.21E-101
ANKS6	0.52797833	5.99E-40
FBP1	0.5604732	7.94E-46
PGAP4	0.67069044	1.46E-71
ZHX1	0.61916653	2.96E-58
DYNLT3	0.50826757	1.11E-36
KIAA1958	0.67299237	3.21E-72
STRBP	0.51917756	1.83E-38
GAPVD1	0.6161503	1.49E-57
ATP7A	0.5314794	1.50E-40

NETO1	2.551357	-0.43443	1.71E-20	6.50E-20
PCDHGC3	1.130559	4.955529	1.71E-20	6.50E-20
GTSF1L	3.451411	-3.04465	1.72E-20	6.51E-20
SLC18A3	8.708931	1.618944	1.74E-20	6.58E-20
LRRC41	1.126336	7.373727	1.75E-20	6.64E-20
TPBGL	2.357839	0.623459	1.76E-20	6.66E-20
FAM209A	1.931149	-2.93945	1.77E-20	6.69E-20
SORL1	-1.0614	7.567191	1.79E-20	6.76E-20
SLC16A6	2.032299	3.033641	1.90E-20	7.16E-20
CYP4F3	-2.16405	3.462983	1.92E-20	7.24E-20
CCDC185	-2.72363	-3.46717	2.07E-20	7.81E-20
SLC23A1	-1.5768	4.016897	2.10E-20	7.92E-20
P11-444E1	1.783417	-1.96496	2.17E-20	8.18E-20
FHL1	1.728435	8.327659	2.22E-20	8.36E-20
ASCL5	1.809331	-2.05093	2.24E-20	8.43E-20
TAC1	-2.87905	-1.25723	2.29E-20	8.63E-20
CA2	-1.25613	7.297848	2.41E-20	9.07E-20
GPA33	1.705869	-1.51516	2.42E-20	9.10E-20
LYPD3	-1.3015	0.524699	2.51E-20	9.45E-20
DNAJC22	1.122457	5.665836	2.52E-20	9.46E-20
MGARP	3.005057	2.879777	2.52E-20	9.49E-20
ANKRD36	1.701626	1.36137	2.58E-20	9.67E-20
KIF4B	2.301177	-2.95377	2.65E-20	9.94E-20
KCNA4	-2.47047	-2.75278	2.74E-20	1.03E-19
PPP1R42	-1.65641	-2.04702	2.75E-20	1.03E-19
ZDHHC19	1.930037	-2.07253	2.75E-20	1.03E-19
VSIG4	1.61387	5.180201	2.79E-20	1.04E-19
RIMBP3	1.345033	-1.7022	2.79E-20	1.05E-19
ZBTB8B	-1.58082	-2.86052	2.80E-20	1.05E-19
GOLGA6L	6.191465	-1.42306	2.84E-20	1.07E-19
ZP1	3.734627	-0.33978	3.19E-20	1.19E-19
TIAF1	1.190569	1.536039	3.29E-20	1.23E-19
LRIT2	3.956295	-2.46947	3.33E-20	1.24E-19
RASGRP2	1.074403	3.427966	3.34E-20	1.25E-19
BUB1B	1.322406	1.963386	3.39E-20	1.27E-19
SIGLEC12	2.592094	-0.32753	3.48E-20	1.30E-19
ATP6V1B	-2.65909	5.239725	3.49E-20	1.30E-19
LRRC43	-1.47144	0.660687	3.56E-20	1.33E-19
P11-411B6	1.789157	-2.67182	3.61E-20	1.34E-19
TMEM176	1.304153	8.990778	3.76E-20	1.40E-19
GBP4	1.245985	7.085028	3.87E-20	1.44E-19
NPPA	1.987971	-2.47609	3.88E-20	1.44E-19
TLL1	1.563086	4.085705	3.95E-20	1.47E-19
TMEM61	-2.80748	1.120746	3.96E-20	1.47E-19
P2RY12	1.733392	1.453113	4.19E-20	1.56E-19
DSCAML	1.326984	4.005746	4.25E-20	1.58E-19
H4C11	2.122045	-3.15714	4.31E-20	1.60E-19
CYP2A6	5.99696	1.431263	4.32E-20	1.60E-19
SCGB2A1	-2.17436	-1.24318	4.34E-20	1.61E-19
EPYC	4.965165	-1.249	4.41E-20	1.63E-19
P11-849F2	-1.21478	-2.4981	4.68E-20	1.73E-19
PRPH2	-1.2474	-0.02224	4.69E-20	1.74E-19
GRM8	2.039695	3.102214	4.75E-20	1.76E-19
VSX1	3.173507	-0.94151	4.77E-20	1.77E-19
SAP25	2.314397	-2.33178	4.78E-20	1.77E-19
IGDCC3	-1.72163	-1.35778	5.00E-20	1.85E-19
CCR7	1.64278	1.896682	5.08E-20	1.88E-19
P11-561B1	1.98844	-2.29804	5.40E-20	2.00E-19
PRR15L	-2.1172	3.390718	5.47E-20	2.02E-19
CD40LG	1.505584	0.849067	5.72E-20	2.11E-19
TACR1	-1.42106	0.978681	5.82E-20	2.15E-19
CYTL1	1.649328	0.865177	5.91E-20	2.18E-19
ATRNL1	-1.91062	2.268736	5.95E-20	2.20E-19
ASMT	1.986983	-2.90711	6.22E-20	2.29E-19
ASMT.1	1.986983	-2.90711	6.22E-20	2.29E-19
SLAMF1	1.513318	1.502709	6.43E-20	2.37E-19
WNT5A	-1.267	3.231295	6.54E-20	2.41E-19
STRC	2.683378	-2.53955	6.59E-20	2.42E-19
MZT2B	1.16822	5.836795	6.80E-20	2.50E-19
UCHL1	-1.90037	4.773674	6.85E-20	2.52E-19
FLT4	1.148327	6.31885	6.88E-20	2.53E-19
CDRT4	1.509262	-0.63068	6.92E-20	2.55E-19
P3-403A15	1.629028	-1.11134	7.06E-20	2.60E-19
HLA-DOA	1.403848	6.380623	7.51E-20	2.76E-19
PCDH12	1.178948	6.199782	7.86E-20	2.89E-19
MAPK15	1.938514	3.442365	8.00E-20	2.94E-19
NECTIN4	-1.93636	1.353454	8.15E-20	2.99E-19
SPATA21	2.841845	-2.36758	8.31E-20	3.04E-19
TMEM200	1.32745	5.582199	8.39E-20	3.07E-19
PGLYRP2	4.037575	-0.81065	8.73E-20	3.20E-19
ARL4C	1.586444	6.49802	8.91E-20	3.26E-19
CETP	1.257727	3.420882	9.15E-20	3.34E-19
GHRHR	-1.56806	-2.95693	9.55E-20	3.49E-19
B3GNT8	-1.27009	1.679474	9.86E-20	3.60E-19
MAP6D1	1.108138	1.000403	9.88E-20	3.61E-19
CELF2	1.007906	5.166479	1.05E-19	3.83E-19
AD000671.	1.755269	-3.18257	1.06E-19	3.86E-19
NPFFR1	2.502034	-0.42148	1.08E-19	3.92E-19
BCO1	1.567438	3.284945	1.08E-19	3.93E-19
CD28	1.438732	2.293822	1.10E-19	4.01E-19
HLA-DQA	2.47335	5.752439	1.11E-19	4.03E-19
FOLR2	1.40891	4.503459	1.16E-19	4.24E-19
OSM	1.901406	1.913711	1.18E-19	4.30E-19
STRIT1	-2.18784	-1.5632	1.22E-19	4.42E-19
RAB3B	-2.09957	1.393725	1.22E-19	4.43E-19
ROPN1L	1.26107	0.193549	1.23E-19	4.48E-19
C4B	1.726475	6.244525	1.25E-19	4.53E-19
HDC	1.837049	0.309662	1.25E-19	4.54E-19
IL2	2.390141	-3.39931	1.28E-19	4.63E-19
TMEM86E	1.272941	0.826586	1.28E-19	4.64E-19
SH2D1B	1.238832	0.574587	1.30E-19	4.72E-19
CH25H	-1.2951	1.136402	1.31E-19	4.76E-19
TMEM200	1.209345	3.770165	1.34E-19	4.85E-19
DDC	-1.69349	6.359879	1.40E-19	5.06E-19
NABP1	1.17778	4.503397	1.42E-19	5.13E-19
BMP8A	1.426094	-0.12421	1.42E-19	5.15E-19
CCN5	3.472329	2.759918	1.46E-19	5.29E-19
GOLGA6L	1.343644	1.006523	1.51E-19	5.44E-19
TIMD4	1.805039	2.868938	1.59E-19	5.74E-19
LPA	-2.14068	-1.37577	1.61E-19	5.80E-19
CBFA2T3	1.058775	1.994187	1.63E-19	5.89E-19
SPTA1	2.26971	-0.81293	1.67E-19	6.01E-19
GRIA4	3.661831	3.97024	1.69E-19	6.11E-19
PBX4	1.490805	1.200313	1.70E-19	6.13E-19
CASS4	1.253539	2.513524	1.81E-19	6.53E-19
ETNPPL	-2.43631	0.155257	1.99E-19	7.14E-19
FAM78B	1.383867	2.014163	1.99E-19	7.17E-19
DLGAP1	-1.71676	1.037241	2.27E-19	8.17E-19
TSGA10IP	2.062731	-2.84362	2.28E-19	8.17E-19
MGAM2	2.097824	0.545533	2.35E-19	8.42E-19
CBR3	1.247552	1.786023	2.36E-19	8.46E-19
MYH13	5.048856	-0.65467	2.40E-19	8.62E-19
RBM44	1.430387	-0.66896	2.40E-19	8.62E-19
FRAS1	-1.25751	5.875715	2.42E-19	8.68E-19
SELP	-1.10893	3.702165	2.56E-19	9.16E-19
CCDC113	-1.18267	2.348994	2.75E-19	9.85E-19
BCAT1	1.448539	4.700885	2.83E-19	1.01E-18
SEC31B	1.659215	2.932914	2.86E-19	1.02E-18
CPT1B	1.871627	1.358444	2.87E-19	1.03E-18
DCDC2B	1.83805	-1.32327	2.91E-19	1.04E-18
AQP5	-2.47306	-0.71655	2.97E-19	1.06E-18
SOD2	1.224012	10.65211	3.05E-19	1.09E-18
NR2E1	4.299564	1.271516	3.13E-19	1.11E-18
COL15A1	1.437909	7.086764	3.15E-19	1.12E-18
GPAT2	1.393446	1.231823	3.21E-19	1.14E-18
FPR1	1.580083	3.705253	3.25E-19	1.16E-18
TMEM92	2.782392	3.028615	3.30E-19	1.18E-18
LARGE2	-1.38586	3.602059	3.43E-19	1.22E-18
GPD1	-1.64815	6.15877	3.44E-19	1.23E-18
LUC7L	1.050016	5.001345	3.46E-19	1.23E-18
RGS20	2.457184	-0.34956	3.53E-19	1.26E-18
KLHL17	1.13881	2.492647	3.55E-19	1.26E-18
TST	-1.0444	5.443535	3.56E-19	1.27E-18
FAM102A	-1.0559	6.094138	3.61E-19	1.28E-18
KCNK3	2.168214	6.419158	3.63E-19	1.29E-18
CYP1A1	-2.89928	-0.40508	3.64E-19	1.29E-18
CATSPER	1.053976	0.251402	3.99E-19	1.41E-18
PTX4	2.073293	-3.14581	4.00E-19	1.42E-18
NME3	1.047622	5.2353	4.25E-19	1.51E-18
TRIM36	1.279107	1.178693	4.35E-19	1.54E-18
IRF4	1.990775	2.025637	4.35E-19	1.54E-18
PLA2G4B	1.852004	-0.85694	4.39E-19	1.55E-18
C10orf105	1.497609	-2.37944	4.43E-19	1.56E-18
PRSS27	1.172014	0.639212	4.49E-19	1.59E-18
SNORC	1.735596	1.284693	4.50E-19	1.59E-18
ENKUR	1.540328	1.082115	4.52E-19	1.60E-18
SEMA3B	-1.32404	5.010344	4.67E-19	1.65E-18
SERPINE1	2.262512	8.501085	4.77E-19	1.68E-18
HLA-DRB	1.370399	7.935467	4.80E-19	1.69E-18
ADPRHL1	-1.06762	-0.16794	4.95E-19	1.74E-18
FOXP3	1.690614	1.526321	4.97E-19	1.75E-18
ST3GAL4	-1.16917	5.122832	5.03E-19	1.77E-18
AZGP1	-1.7504	5.506366	5.09E-19	1.79E-18
MESP1	1.272358	0.962112	5.34E-19	1.88E-18
SYNPO	1.134671	8.844289	5.45E-19	1.92E-18
MYH3	1.448334	1.508628	5.51E-19	1.94E-18
ITIH4	2.485547	-0.03366	5.53E-19	1.94E-18
GBP6	1.710276	-1.87909	5.53E-19	1.94E-18
SPATA46	-1.59072	-3.10758	5.74E-19	2.01E-18
ANKRD45	-1.27964	0.729335	5.75E-19	2.02E-18
TRIM74	2.140525	-2.63603	5.79E-19	2.03E-18
FAM72B	1.354744	-1.50451	5.81E-19	2.04E-18
NAT2	-1.69706	-1.02064	6.07E-19	2.13E-18
SULT1A3	2.256656	-3.32309	6.10E-19	2.14E-18
CYTIP	1.25257	3.981594	6.10E-19	2.14E-18
PODNL1	2.578201	1.558251	6.43E-19	2.25E-18
LDLRAD2	1.170829	2.006993	6.46E-19	2.26E-18
CHI3L2	2.319039	2.694588	6.77E-19	2.37E-18
LRRC14B	3.596976	0.414423	6.83E-19	2.39E-18
SCML4	1.341357	0.812931	7.11E-19	2.48E-18
PARVB	1.10174	5.407587	7.21E-19	2.52E-18
RSPH4A	-1.02304	-0.338	7.39E-19	2.58E-18
B3GNT4	1.784583	2.860727	7.53E-19	2.63E-18
GASK1B	1.224823	6.624999	7.65E-19	2.67E-18
CD44	1.421686	7.49576	7.76E-19	2.71E-18
SCRN1	-1.02601	6.585596	7.91E-19	2.76E-18
PVRIG	1.592079	-1.52841	7.91E-19	2.76E-18
NDNF	-2.43853	4.563039	7.92E-19	2.76E-18
COL1A2	1.75999	9.730476	8.67E-19	3.02E-18
AGAP4	1.098445	0.718891	8.87E-19	3.08E-18

ZNF367	0.56370666	1.90E-46
HDX	0.55423603	1.20E-44
NDUFB6	0.54088049	3.31E-42
VCP	0.62285952	3.98E-59
PIGO	0.58366563	1.95E-50
STOML2	0.59482954	8.62E-53
OTUD1	0.56954337	1.38E-47
ARHGAP12	0.52490078	2.00E-39
MARCHF8	0.71335412	8.37E-85
CFL2	0.5330264	8.05E-41
SUGT1	0.6683629	6.65E-71
GTF2A1	0.58258726	3.25E-50
PHYHIPL	0.6412723	1.19E-63
SLC16A9	0.59605266	4.70E-53
CRYL1	0.71225	1.98E-84
REEP3	0.53156883	1.44E-40
MICU2	0.66825888	7.11E-71
DNAAF2	0.61003966	3.77E-56
KLHDC2	0.60912985	6.06E-56
RPUSD4	0.56253315	3.20E-46
ARF6	0.50342253	6.53E-36
TTC8	0.5525411	2.48E-44
KBTBD6	0.66619797	2.69E-70
BEND7	0.54244473	1.74E-42
ATP5F1C	0.52457978	2.27E-39
PRPF18	0.7342312	3.30E-92
TAF3	0.6042919	7.36E-55
VDAC2	0.55264583	2.37E-44
PDZD8	0.53208614	1.17E-40
ABRAXAS2	0.7103596	8.52E-84
QSOX2	0.54135346	2.73E-42
FAM204A	0.59722926	2.62E-53
NSD1	0.51590341	6.37E-38
PRDX3	0.72744652	1.00E-89
GHITM	0.65030935	5.47E-66
DDX21	0.5426934	1.57E-42
BMS1	0.5843855	1.38E-50
NDRG2	0.50014865	2.13E-35
ARHGEF40	0.56433654	1.44E-46
CASP7	0.60667288	2.16E-55
CCDC186	0.59531495	6.78E-53
SALL2	0.5160282	6.08E-38
TRUB1	0.56844684	2.27E-47
ZFYVE1	0.54531489	5.27E-43
ARHGAP42	0.62763021	2.86E-60
ISCA2	0.56243216	3.34E-46
OTOGL	0.50357454	6.18E-36
TTC7B	0.53053921	2.17E-40
CPSF2	0.52117853	8.49E-39
MOAP1	0.70423572	8.95E-82
ARL5B	0.51575802	6.73E-38
AMOTL1	0.55219889	2.87E-44
SPRED1	0.51591847	6.33E-38
RAB8B	0.5092349	7.74E-37
RPUSD2	0.51375319	1.43E-37
HIF1AN	0.5521414	2.94E-44
BRD7	0.5790806	1.70E-49
BTRC	0.65571722	2.00E-67
BAG5	0.54097737	3.18E-42
API5	0.65768678	5.89E-68
ZNF319	0.52700866	8.78E-40
HPS6	0.60376164	9.65E-55
SENP8	0.6385266	5.87E-63
NOLC1	0.59120939	5.12E-52
COPS2	0.58503694	1.01E-50
GABRB3	0.55954617	1.19E-45
SGPL1	0.52324436	3.82E-39
FRS2	0.59910136	1.02E-53
CCT2	0.61565547	1.95E-57
ARIH1	0.57019233	1.03E-47
COX11	0.67619407	3.81E-73
FAM227B	0.54212982	1.98E-42
STXBP4	0.60776197	1.23E-55
CYYR1	0.52372284	3.17E-39
CUL5	0.63254187	1.81E-61
WBP1L	0.51141132	3.45E-37
BORCS7	0.65771505	5.79E-68
ANAPC16	0.60777815	1.22E-55
APBB1	0.65444925	4.37E-67
TRIM44	0.6573343	7.34E-68
ILK	0.66288384	2.24E-69
TPP1	0.64713531	3.69E-65
CYB5A	0.65871233	3.11E-68
RAG1	0.61016543	3.53E-56
IFTAP	0.5061416	2.42E-36
ATP9B	0.55553167	6.85E-45
PPFIBP2	0.50754626	1.44E-36
GARRE1	0.53860133	8.44E-42
IDH3A	0.58626139	5.63E-51
WDR72	0.57756709	3.45E-49
CDYL2	0.56003802	9.61E-46
ATMIN	0.67510698	7.88E-73
C16orf46	0.51346646	1.60E-37
LEO1	0.74173178	4.83E-95
ZNF143	0.63073477	5.03E-61
ZSCAN21	0.52777746	6.49E-40
SEC11C	0.5101255	5.56E-37
TMEM135	0.69125761	1.17E-77
COG1	0.54448544	7.45E-43
PLEKHA7	0.60812374	1.02E-55
B2M	0.53115666	1.70E-40
ZNF592	0.64597402	7.39E-65
GOLM2	0.62457488	1.55E-59
AP1G1	0.69372539	2.01E-78
MARF1	0.63300621	1.39E-61
CIAO2A	0.50262235	8.73E-36
PEX11A	0.72750563	9.52E-90
TMEM170A	0.64593834	7.55E-65
GLYATL1	0.63589322	2.68E-62
DCTN5	0.58269441	3.09E-50
TERF2IP	0.53452927	4.40E-41
CLPX	0.66735288	1.28E-70
ZBTB39	0.52918615	3.72E-40
VPS39	0.55652325	4.46E-45
PATL1	0.60771662	1.26E-55
STX3	0.65924016	2.23E-68
PIP4K2C	0.5455735	4.73E-43
MTMR10	0.56603813	6.71E-47
YWHAB	0.62061064	1.35E-58
DIS3L	0.636802	1.59E-62
CCNDBP1	0.64284118	4.72E-64
SMAD3	0.5014903	1.32E-35
AKTIP	0.58669995	4.56E-51
MAPRE2	0.57637454	6.01E-49
NUDT21	0.56362829	1.97E-46
TEF	0.50114445	1.49E-35
PHB	0.60932153	5.48E-56
SNRPD1	0.51406157	1.28E-37
GOLGA2	0.51275479	2.09E-37
TRUB2	0.59291683	2.22E-52
UGT1A6	0.50248941	9.17E-36
SP2	0.64738911	3.17E-65
COQ7	0.61714005	8.80E-58
CRK	0.67783364	1.27E-73
HDHD2	0.70939343	1.79E-83
RNF214	0.55581546	6.06E-45
CDK12	0.58071926	7.87E-50
ACAA2	0.79275069	2.52E-117
RRM1	0.56025781	8.72E-46
TRIM68	0.51663494	4.83E-38
SMG8	0.69604678	3.76E-79
RAB8A	0.55312358	1.93E-44
ZNF641	0.54833918	1.48E-43
DHRS13	0.51100897	4.01E-37
ZNF526	0.52059664	1.06E-38
TRAPPC9	0.53971552	5.35E-42
ZNF146	0.54003439	4.69E-42
GLOD4	0.74705521	4.09E-97
SRR	0.58703559	3.88E-51
TSR1	0.65446747	4.32E-67
CYB5D2	0.65423374	4.99E-67
SPRYD3	0.55893629	1.56E-45
SRP68	0.69185019	7.68E-78
GHDC	0.59272443	2.44E-52
ZNF597	0.54752029	2.09E-43
DDB1	0.66466578	7.20E-70
VPS37C	0.59270717	2.46E-52
FTH1	0.53665117	1.87E-41
BSCL2	0.56815716	2.59E-47
CTNNB1	0.6089388	6.69E-56
FADD	0.549217	1.02E-43
SLC22A11	0.60686572	1.96E-55
SF1	0.5814255	5.64E-50
PAFAH1B2	0.57636047	6.05E-49
KIAA1586	0.59476796	8.89E-53
RAB4A	0.74289585	1.72E-95
RNF187	0.62261001	4.56E-59
MAPK1IP1L	0.55139249	4.05E-44
LMBRD1	0.71319592	9.47E-85
ZCCHC4	0.52967732	3.06E-40
UBTD2	0.62394038	2.20E-59
DNAJC7	0.58419286	1.52E-50
IRF2BP2	0.65015891	5.99E-66
MGAT2	0.50977646	6.33E-37
BMI1	0.53565306	2.80E-41
THAP11	0.65228468	1.65E-66
MMADHC	0.74635873	7.70E-97
PDHB	0.50213402	1.04E-35
ACOX2	0.53037248	2.32E-40
IRF2	0.52623537	1.19E-39
SEPTIN2	0.68462868	1.23E-75
RFWD3	0.55260223	2.42E-44
COG7	0.59605625	4.69E-53
CDC40	0.65624635	1.44E-67
STIP1	0.5046478	4.18E-36
FEN1	0.51384282	1.39E-37
CAVIN2	0.55641742	4.67E-45
TRAPPC11	0.62305851	3.57E-59
ING2	0.53542589	3.07E-41
CDKN2AIP	0.68877856	6.77E-77

TGFBI	2.837135	10.63122	9.50E-19	3.30E-18
WFDC10E	3.482248	-2.60611	9.96E-19	3.46E-18
EFCAB8	2.46789	-2.35789	9.97E-19	3.46E-18
UGT2B7	-1.28168	8.083537	1.05E-18	3.65E-18
ATP6V1C	-1.84593	3.162877	1.05E-18	3.65E-18
AIF1	1.062991	5.197382	1.06E-18	3.68E-18
HKB-CPT	1.734647	0.189625	1.10E-18	3.81E-18
BMP3	-1.97154	-0.56667	1.15E-18	4.00E-18
SGCG	-1.35853	-1.64349	1.20E-18	4.16E-18
HAL	2.598276	0.244131	1.20E-18	4.16E-18
ELAPOR1	1.829011	1.914963	1.20E-18	4.16E-18
CX3CR1	1.680287	4.452527	1.21E-18	4.18E-18
CCDC85B	1.493097	4.396719	1.21E-18	4.19E-18
PLXNB3	2.027657	1.675231	1.24E-18	4.29E-18
C4A	1.728807	6.329483	1.32E-18	4.57E-18
TTC34	1.352983	1.131655	1.33E-18	4.61E-18
ANKRD36	1.366702	0.818162	1.45E-18	4.98E-18
GPR63	-1.00645	-0.03648	1.51E-18	5.19E-18
KIF26B	-1.26477	3.25145	1.51E-18	5.20E-18
CCR9	-1.4784	-1.96192	1.57E-18	5.39E-18
ATP1A3	2.419393	1.376573	1.58E-18	5.45E-18
MTHFD2	1.022804	4.81474	1.61E-18	5.54E-18
P11-87C1	1.876136	-2.31511	1.62E-18	5.55E-18
FAM124A	-1.11789	3.012817	1.64E-18	5.64E-18
MCEMP1	2.1748	-0.71221	1.65E-18	5.68E-18
MEP1A	2.646176	-2.59017	1.66E-18	5.71E-18
CLIC3	1.632789	0.870931	1.71E-18	5.89E-18
TGM2	1.286578	8.985755	1.80E-18	6.16E-18
CREG2	2.02516	1.650545	1.94E-18	6.66E-18
SPSB1	1.313298	5.530975	2.03E-18	6.96E-18
ANO5	-1.9616	2.772211	2.08E-18	7.10E-18
FSCN3	1.609204	-2.57406	2.09E-18	7.16E-18
GCAT	-1.063	3.33798	2.12E-18	7.26E-18
TREM1	2.222155	2.074813	2.13E-18	7.27E-18
C1orf210	-1.40479	3.627519	2.17E-18	7.43E-18
LILRA5	1.263567	1.603428	2.26E-18	7.71E-18
APOC2	2.434523	-0.75328	2.29E-18	7.82E-18
RIPOR2	1.020398	4.257316	2.32E-18	7.92E-18
GPT2	-1.09998	4.692565	2.40E-18	8.18E-18
HMMR	1.39127	1.965169	2.41E-18	8.22E-18
FGF9	-2.87574	3.315626	2.60E-18	8.85E-18
ZGLP1	1.278281	-0.25805	2.60E-18	8.85E-18
EMCN	-1.09608	6.411286	2.72E-18	9.26E-18
ELF3	-1.10672	6.333431	2.73E-18	9.29E-18
CDH2	1.235467	6.605089	2.82E-18	9.59E-18
ALDH1A3	-1.39768	4.137524	2.84E-18	9.66E-18
KCNN4	1.580533	1.50638	2.93E-18	9.94E-18
TBC1D3L	2.236598	-0.57956	2.93E-18	9.94E-18
PZP	1.567433	-0.34432	3.10E-18	1.05E-17
WNT10B	2.175619	-1.26145	3.11E-18	1.05E-17
RLN1	-1.1115	-2.49351	3.17E-18	1.08E-17
LAMP3	1.18608	2.587673	3.18E-18	1.08E-17
COL6A1	1.117492	8.214815	3.18E-18	1.08E-17
UGT1A1	2.749493	-0.82112	3.18E-18	1.08E-17
LHFPL5	3.118416	-2.95483	3.35E-18	1.13E-17
CEACAM	1.782025	-1.76635	3.61E-18	1.22E-17
HAVCR1	2.214396	5.718676	3.76E-18	1.27E-17
CBWD3	1.069679	-0.47246	3.78E-18	1.28E-17
ZNF804A	-1.2247	-0.21946	3.84E-18	1.30E-17
JSRP1	2.437915	0.454539	3.86E-18	1.30E-17
PRIMA1	3.061671	5.226172	3.87E-18	1.31E-17
CCDC168	1.662696	-1.57332	3.92E-18	1.32E-17
ADCY2	2.606849	2.455917	4.10E-18	1.38E-17
EMILIN3	-1.21393	-1.59648	4.21E-18	1.42E-17
CCL28	1.541641	4.905161	4.23E-18	1.43E-17
C14orf132	-1.42731	3.747823	4.28E-18	1.44E-17
C1QL4	3.053214	2.930716	4.39E-18	1.48E-17
EPOP	1.152343	1.77733	4.52E-18	1.52E-17
LURAP1L	1.027921	3.981793	4.58E-18	1.54E-17
TAS2R4	1.458315	-0.55731	5.01E-18	1.68E-17
H4C5	3.560069	-0.14553	5.20E-18	1.75E-17
CCL25	2.872275	-3.23226	5.26E-18	1.77E-17
MT3	3.340229	3.944438	5.66E-18	1.90E-17
ASIC2	-1.75177	-0.04792	5.69E-18	1.91E-17
PLAAT4	1.295305	6.673406	5.74E-18	1.93E-17
HSPB2	1.285187	2.461529	5.78E-18	1.94E-17
CCDC57	1.046434	4.486187	5.82E-18	1.95E-17
PIK3CG	1.247022	3.301491	5.84E-18	1.96E-17
FBXL16	1.593275	5.58501	5.93E-18	1.99E-17
ELFN1	-1.00596	2.910996	6.06E-18	2.03E-17
CORO6	2.16376	0.716035	6.27E-18	2.10E-17
RNF152	-1.17621	6.86314	6.55E-18	2.19E-17
NIM1K	1.565686	0.719447	6.56E-18	2.19E-17
HOXD1	-1.2097	-0.07228	6.69E-18	2.24E-17
PCDHB6	1.820747	2.489895	6.74E-18	2.25E-17
HMCN1	1.247453	5.114939	6.77E-18	2.26E-17
LYG2	1.840914	-2.15948	7.08E-18	2.36E-17
GALNT14	1.121032	7.773814	7.19E-18	2.40E-17
KRT25	4.88375	-1.72504	7.27E-18	2.43E-17
GLYAT	-1.4871	6.247617	7.35E-18	2.45E-17
KIF15	1.230842	1.005831	7.37E-18	2.46E-17
CD69	1.233023	3.097487	7.46E-18	2.49E-17
LSMEM2	-1.27521	-2.8947	7.47E-18	2.49E-17
IGFN1	5.522955	3.315863	7.47E-18	2.49E-17
C17orf107	1.15737	2.894067	7.70E-18	2.56E-17
HBE1	-2.16767	-1.6854	7.88E-18	2.62E-17
B4GALNT	3.191276	1.562877	7.92E-18	2.63E-17
NXPH2	-3.48263	-0.73884	8.10E-18	2.69E-17
GFOD1	1.072801	4.208938	8.11E-18	2.69E-17
ALOX5AP	1.207595	3.80478	8.39E-18	2.78E-17
PTPRR	1.547489	0.910603	8.56E-18	2.84E-17
NTNG2	1.71224	1.656925	8.74E-18	2.90E-17
CELSR3	1.500866	1.38685	9.09E-18	3.01E-17
ADAM18	4.025587	0.952127	9.16E-18	3.03E-17
UMODL1	-2.19005	-1.67978	9.54E-18	3.16E-17
THSD1	1.017201	2.991566	9.60E-18	3.18E-17
P2RX5	1.906005	-0.3792	9.76E-18	3.23E-17
FOXF2	1.712274	-0.35703	9.78E-18	3.23E-17
FAHD2B	-1.04367	1.981448	9.96E-18	3.29E-17
CLDN9	-1.47255	-0.47681	1.02E-17	3.39E-17
GXYLT2	1.972855	3.114181	1.07E-17	3.55E-17
PDLIM7	1.026788	5.360887	1.09E-17	3.60E-17
EYA2	-1.27932	2.148975	1.11E-17	3.68E-17
TMEM132	-2.00289	-0.79114	1.17E-17	3.86E-17
CAMK2N	1.916579	-0.9694	1.19E-17	3.94E-17
PRSS57	2.177495	-2.36682	1.22E-17	4.01E-17
ANO9	1.705766	4.186855	1.30E-17	4.27E-17
TTC39A	1.200522	5.547434	1.35E-17	4.45E-17
P11-45M2	1.584134	-2.90303	1.36E-17	4.48E-17
MIF	1.252879	5.844999	1.37E-17	4.49E-17
SEZ6L	3.385663	0.311895	1.49E-17	4.88E-17
NPIPA3	2.430291	-1.70591	1.50E-17	4.93E-17
PLEKHF1	1.021781	2.861289	1.54E-17	5.07E-17
MEGF6	1.331797	4.4637	1.56E-17	5.14E-17
VXN	1.691287	2.498954	1.58E-17	5.19E-17
LGALS3	1.438012	7.915759	1.64E-17	5.38E-17
CD93	1.07012	8.120188	1.65E-17	5.41E-17
FOXH1	1.955289	-2.63482	1.66E-17	5.45E-17
CREB3L3	2.700872	4.334116	1.69E-17	5.53E-17
EML5	-1.33467	-0.27979	1.70E-17	5.55E-17
KCNJ13	-2.30619	1.526024	1.74E-17	5.69E-17
RTL4	-1.90233	-1.35114	1.75E-17	5.73E-17
LMTK3	-1.4903	1.658163	1.76E-17	5.74E-17
GPR25	2.057601	-3.18526	1.78E-17	5.81E-17
FRMD1	-1.97854	1.409107	1.82E-17	5.95E-17
PDZRN3	-1.16165	3.590995	1.83E-17	5.97E-17
NEDD4L	-1.01004	6.889285	1.88E-17	6.14E-17
ALK	1.875164	-1.20584	1.91E-17	6.23E-17
WNK3	-1.40897	1.963923	1.92E-17	6.26E-17
SSUH2	-1.20961	-0.74062	1.94E-17	6.31E-17
TCP11	-1.28204	-1.86912	1.95E-17	6.34E-17
PROS1	1.154764	7.284914	1.95E-17	6.37E-17
C15orf48	1.564291	2.449586	2.04E-17	6.66E-17
RDH5	1.255271	2.563062	2.05E-17	6.67E-17
CIB4	2.523653	0.168495	2.17E-17	7.05E-17
C2CD4C	1.441953	1.117986	2.19E-17	7.11E-17
PPP4R4	-1.75294	-1.24927	2.20E-17	7.14E-17
NLRP2	-1.63665	1.668374	2.23E-17	7.23E-17
BCL2L10	-1.63864	0.471081	2.28E-17	7.41E-17
KRT86	1.528979	0.644231	2.37E-17	7.69E-17
HRH1	1.039611	4.078294	2.37E-17	7.70E-17
SLC25A1	-1.02548	3.82416	2.40E-17	7.81E-17
MCF2	-1.2643	-0.32598	2.65E-17	8.59E-17
WSB1	1.025403	7.065528	2.70E-17	8.76E-17
PRCD	1.058963	0.058738	2.89E-17	9.36E-17
HOXC12	2.40755	-2.08855	2.90E-17	9.38E-17
CD163L1	1.530279	2.790308	2.91E-17	9.41E-17
ARID3C	2.201142	-2.05917	2.92E-17	9.44E-17
SLC6A12	-1.14862	6.036316	2.95E-17	9.54E-17
LRRC55	-1.22623	-0.09155	3.01E-17	9.73E-17
SMTNL2	-1.19974	4.309736	3.13E-17	1.01E-16
DPYS	-1.44036	6.044492	3.18E-17	1.03E-16
CADM2	-2.05046	-1.93708	3.35E-17	1.08E-16
EPS8L1	-1.5786	3.323556	3.35E-17	1.08E-16
PIM2	1.270311	4.856333	3.48E-17	1.12E-16
SLC22A1	2.229	-1.54613	3.60E-17	1.16E-16
APOD	-1.4322	3.388163	3.63E-17	1.17E-16
CBLN4	2.089126	2.172488	3.66E-17	1.18E-16
SYNGR4	2.064172	-2.57009	3.72E-17	1.20E-16
FOXQ1	-1.19351	3.001723	3.77E-17	1.21E-16
WIF1	-1.96413	-2.51977	4.13E-17	1.33E-16
SCG3	4.599952	2.224155	4.18E-17	1.34E-16
DNAJC12	-1.33554	2.278579	4.28E-17	1.38E-16
PLA2G6	1.271899	3.859428	4.30E-17	1.38E-16
SPON2	1.795489	6.386084	4.49E-17	1.44E-16
KIFC2	1.234541	3.471316	4.50E-17	1.44E-16
ANKRD2	1.121756	3.933784	4.74E-17	1.52E-16
IHO1	1.351992	-0.68732	4.82E-17	1.55E-16
EBLN2	1.25597	-0.49144	5.06E-17	1.62E-16
TPO	1.853563	-2.8308	5.21E-17	1.67E-16
GPR31	2.198975	-2.64111	5.33E-17	1.71E-16
ASGR1	1.671715	0.97925	5.43E-17	1.74E-16
SAA2	5.083681	4.01624	5.56E-17	1.78E-16
FABP2	-1.83788	-3.04761	5.64E-17	1.80E-16
NRXN1	-1.99972	-1.01071	5.67E-17	1.81E-16
KCNMB1	1.191935	3.198916	5.68E-17	1.81E-16
ME1-NME	1.055252	-0.96059	5.78E-17	1.85E-16
NAIP	1.377124	0.859984	5.79E-17	1.85E-16
COL11A1	3.450462	3.119588	5.81E-17	1.85E-16
ZMAT1	1.158004	4.833847	5.81E-17	1.85E-16

SNRNP48	0.55602414	5.53E-45
TMUB2	0.57723063	4.04E-49
STAT3	0.61362733	5.71E-57
ADAM9	0.50643507	2.17E-36
LRATD2	0.60299599	1.43E-54
SLC16A4	0.69226895	5.70E-78
AHCYL1	0.6723567	4.88E-72
DNAJC21	0.59628233	4.19E-53
CNNM3	0.72851496	4.11E-90
TCTN2	0.50200188	1.09E-35
ABHD15	0.62093621	1.13E-58
ZBTB5	0.6003213	5.54E-54
CHTF8	0.67525086	7.16E-73
ADAL	0.55459628	1.03E-44
LCMT2	0.69858063	5.93E-80
ZNF507	0.5187149	2.19E-38
GFM1	0.63673782	1.65E-62
DDX19A	0.6706198	1.53E-71
LRRC28	0.64471869	1.56E-64
MAT2A	0.64992935	6.88E-66
CEP120	0.5226228	4.86E-39
MFF	0.58483667	1.11E-50
E2F6	0.58806222	2.36E-51
FEM1B	0.64339828	3.40E-64
COMMD8	0.56108253	6.07E-46
UQCRFS1	0.59618488	4.40E-53
MAP2K1	0.56228632	3.57E-46
AR	0.56077838	6.94E-46
CSNK1G1	0.60006039	6.32E-54
UBE2V2	0.58214812	4.01E-50
CD2BP2	0.52837767	5.12E-40
NMD3	0.56372541	1.88E-46
B3GALNT1	0.69702249	1.85E-79
MRPL1	0.54747936	2.13E-43
SHE	0.52783608	6.34E-40
PGM2	0.54744132	2.16E-43
SLC33A1	0.68840337	8.81E-77
SNUPN	0.58109211	6.60E-50
CRADD	0.63296149	1.43E-61
SIN3A	0.6364759	1.92E-62
ARL13B	0.55413578	1.25E-44
PTK2	0.61575199	1.85E-57
PTPN9	0.62230586	5.38E-59
RASSF6	0.53908292	6.93E-42
SDC2	0.51458831	1.05E-37
MMGT1	0.57463372	1.35E-48
TM2D2	0.64578069	8.29E-65
PLEKHA2	0.62540646	9.81E-60
CLIC4	0.74026547	1.76E-94
METTL15	0.63740802	1.12E-62
GJB1	0.64361165	3.00E-64
PCBP1	0.77956038	5.37E-111
DTWD2	0.65889044	2.78E-68
NFU1	0.67147266	8.73E-72
RAMAC	0.51958272	1.57E-38
APLF	0.59907042	1.04E-53
LUZP1	0.60659145	2.26E-55
CNBP	0.60136397	3.27E-54
LDB2	0.52047805	1.11E-38
NLGN1	0.50029032	2.03E-35
TAPT1	0.7062627	1.94E-82
UGP2	0.51610092	5.91E-38
HNRNPF	0.66239874	3.05E-69
BTD	0.58551688	8.05E-51
SYAP1	0.54245302	1.73E-42
TOR1AIP2	0.5406905	3.58E-42
BRD3	0.52581628	1.40E-39
ZNF764	0.5984841	1.40E-53
ZNF747	0.58704238	3.87E-51
ZNF768	0.51354114	1.55E-37
MAP3K2	0.55475556	9.57E-45
UBE2E3	0.66233072	3.18E-69
TMEM192	0.73662496	4.21E-93
ZNF778	0.55542277	7.18E-45
NIPA1	0.54085665	3.35E-42
HNRNPA3	0.61367041	5.58E-57
SIK2	0.6120326	1.33E-56
USP38	0.61988625	2.01E-58
NANP	0.56959791	1.35E-47
PDCD6IP	0.53385996	5.76E-41
GON7	0.61152967	1.73E-56
ELP5	0.54163097	2.43E-42
CMTM8	0.51571607	6.84E-38
GABARAP	0.59871617	1.24E-53
UBB	0.62561398	8.75E-60
PRDM10	0.53158851	1.43E-40
B3GNT2	0.64093375	1.45E-63
TMED10	0.58165971	5.05E-50
EMX2	0.54091939	3.26E-42
SLC30A1	0.620639	1.33E-58
TMEM182	0.53100561	1.81E-40
NFXL1	0.53010801	2.58E-40
DENND5B	0.58436315	1.40E-50
NUDT9	0.59923516	9.57E-54
ARL6IP1	0.51421648	1.20E-37
CDH2	0.59486032	8.49E-53
NUDCD2	0.60131756	3.35E-54
HSPA4	0.52258565	4.93E-39
ARMC10	0.54522866	5.46E-43
ATF7	0.57133433	6.12E-48
SOCS6	0.60053682	4.97E-54
POLH	0.55709555	3.48E-45
SYT9	0.53764102	1.25E-41
KIF5B	0.58662831	4.72E-51
USP32	0.60840503	8.83E-56
PPM1D	0.64159879	9.80E-64
KBTBD2	0.52230796	5.49E-39
KIAA0232	0.6060269	3.02E-55
MTSS1	0.56522974	9.63E-47
RNF139	0.61527505	2.38E-57
GSTA4	0.65164198	2.43E-66
MSANTD4	0.63110467	4.08E-61
PKHD1	0.51043449	4.96E-37
DNAJC24	0.5141332	1.24E-37
PDGFD	0.61654163	1.21E-57
S1PR1	0.53668142	1.84E-41
FEZ2	0.61593629	1.68E-57
MTM1	0.70046982	1.48E-80
TRMT61B	0.6679679	8.59E-71
INSR	0.55999094	9.81E-46
MFN1	0.50612935	2.43E-36
GIMAP8	0.51787506	3.01E-38
PRKCE	0.66259656	2.69E-69
SOCS5	0.5646909	1.23E-46
C1GALT1C1	0.62620881	6.30E-60
ZNF672	0.57004202	1.10E-47
NAIF1	0.59018079	8.45E-52
TMEM126B	0.61359793	5.80E-57
TMEM37	0.55879401	1.66E-45
FAM98B	0.7148957	2.51E-85
PGAM1	0.65861506	3.30E-68
CLCN5	0.7189608	1.00E-86
PDE7B	0.55676552	4.01E-45
NAT1	0.52415466	2.68E-39
DSEL	0.50019608	2.10E-35
ZNF318	0.6269274	4.23E-60
ZNF561	0.56820499	2.53E-47
WIPF2	0.71414863	4.50E-85
RSL1D1	0.5449283	6.19E-43
LRRC8D	0.5443224	7.97E-43
PPID	0.61992682	1.96E-58
ETFDH	0.70121142	8.54E-81
TBCA	0.50036094	1.98E-35
PLRG1	0.73874787	6.66E-94
ZNF584	0.54768339	1.95E-43
CLSTN1	0.61827294	4.79E-58
BPTF	0.53125419	1.64E-40
HDAC3	0.53679115	1.76E-41
GPHN	0.54350346	1.12E-42
CAMTA1	0.5497507	8.14E-44
SPATA5L1	0.65043978	5.05E-66
GATM	0.70212722	4.33E-81
BCL2	0.61110512	2.16E-56
METTL18	0.59234072	2.94E-52
FBXL14	0.6184123	4.44E-58
EXOSC10	0.5113398	3.54E-37
ZNF570	0.51251508	2.28E-37
MRM3	0.61927184	2.80E-58
PTEN	0.5482025	1.57E-43
RNASEH1	0.52929239	3.57E-40
TLN2	0.66601132	3.04E-70
TVP23B	0.63582382	2.79E-62
ATPAF2	0.54572863	4.43E-43
JMJD1C	0.53769618	1.22E-41
RAB33B	0.60836126	9.03E-56
USP19	0.54697294	2.63E-43
KLF11	0.71048801	7.72E-84
EIF2AK3	0.52381226	3.06E-39
FRMD3	0.6796151	3.80E-74
TEFM	0.53733663	1.41E-41
PAIP1	0.63010471	7.17E-61
DPAGT1	0.62772771	2.71E-60
SPTLC3	0.55087748	5.04E-44
BPGM	0.64029352	2.10E-63
ALG14	0.66178544	4.50E-69
SUCLG2	0.56499794	1.07E-46
GNG12	0.60559446	3.77E-55
CLP1	0.67174935	7.28E-72
FGGY	0.59149835	4.44E-52
TCEAL1	0.56531052	9.29E-47
ZNF24	0.62650667	5.34E-60
MANEA	0.66606132	2.94E-70
AFF1	0.60737298	1.51E-55
FIBP	0.52147095	7.58E-39
HCFC1	0.50716807	1.66E-36
SMPDL3A	0.5725134	3.57E-48
WASHC2C	0.59670495	3.40E-53
ZFAND4	0.56140526	5.26E-46
FUT10	0.58309772	2.55E-50
TMCC1	0.50849857	1.02E-36

CC2D2B	1.576423	-1.99456	5.82E-17	1.86E-16
PLP2	1.150686	6.417845	5.87E-17	1.87E-16
NLRP2B	1.546152	-2.81426	6.12E-17	1.95E-16
H2BC9	2.457873	-1.77014	6.19E-17	1.97E-16
MOV10L1	1.435946	-0.3763	6.27E-17	2.00E-16
ENP3-EIF4	1.676788	-3.1602	6.37E-17	2.03E-16
BCAS1	-1.99908	1.614933	6.60E-17	2.10E-16
MYRFL	1.957625	1.481228	6.67E-17	2.12E-16
SPINK5	2.150404	1.92627	6.70E-17	2.13E-16
EPHB3	-1.09482	2.767443	6.79E-17	2.16E-16
NFRSF11	-1.12935	5.461166	7.75E-17	2.46E-16
ANKRD61	1.221626	-1.13155	7.78E-17	2.47E-16
KLK14	1.955289	-2.21651	7.99E-17	2.53E-16
CYP2S1	-1.05196	1.927082	8.06E-17	2.56E-16
LDLR	-1.25124	4.591247	8.19E-17	2.60E-16
TD-2370N	1.64659	-3.06098	8.33E-17	2.64E-16
ARHGEF3	-1.44335	1.906799	8.52E-17	2.70E-16
TMEM132	1.209409	5.129017	8.78E-17	2.78E-16
EGFL7	1.164854	6.061831	8.92E-17	2.82E-16
TFAP2C	-2.11845	0.738508	9.24E-17	2.92E-16
IRS1	-1.03171	4.788409	1.03E-16	3.26E-16
KLRF2	3.10956	-3.25205	1.07E-16	3.37E-16
SCGB3A1	1.685006	-0.45026	1.07E-16	3.37E-16
GCNT7	1.69336	-1.79894	1.07E-16	3.37E-16
TEX29	1.808663	-1.60221	1.10E-16	3.47E-16
CFB	1.350923	4.59655	1.15E-16	3.61E-16
HTR1F	1.626059	0.928516	1.16E-16	3.63E-16
PYCR1	1.730017	3.210357	1.21E-16	3.81E-16
ANGPTL8	3.990426	1.899281	1.24E-16	3.89E-16
NTRK1	-1.27366	-0.70326	1.28E-16	4.00E-16
TNNT3	3.734074	1.536064	1.28E-16	4.00E-16
ALDH1A2	-1.69479	4.374538	1.29E-16	4.04E-16
P5-966M1	1.76426	0.159007	1.29E-16	4.05E-16
TERT	4.554705	-0.926	1.32E-16	4.14E-16
TP63	-1.15132	-0.01604	1.37E-16	4.29E-16
PCDH7	-1.3803	2.803732	1.43E-16	4.48E-16
HD14A-A	-1.07557	-1.31303	1.44E-16	4.49E-16
POU4F1	4.647874	-0.50498	1.48E-16	4.62E-16
ACR	1.305466	-0.69522	1.53E-16	4.77E-16
TSC22D3	1.136389	8.434586	1.53E-16	4.78E-16
MARCO	2.385611	2.311922	1.56E-16	4.85E-16
ASB2	1.45601	1.964631	1.61E-16	5.02E-16
SYTL1	1.53359	2.472262	1.63E-16	5.07E-16
TOX	-1.22723	3.212745	1.74E-16	5.42E-16
TPSAB1	1.682842	2.816521	1.76E-16	5.47E-16
ADGRL4	1.02767	6.660143	1.85E-16	5.75E-16
KLHDC8A	1.698772	1.497903	1.86E-16	5.77E-16
LHFPL3	-1.41858	-0.58945	1.87E-16	5.79E-16
NMU	2.979557	-0.0855	1.90E-16	5.89E-16
SH2D6	-1.47443	-1.40829	1.96E-16	6.07E-16
RNF165	1.401645	2.806724	2.07E-16	6.42E-16
IGFBP1	4.331963	5.041121	2.11E-16	6.56E-16
FAM171A	-1.05491	0.925627	2.14E-16	6.63E-16
CHGA	-2.08815	-1.03488	2.19E-16	6.80E-16
IL1RAP	1.027493	4.125766	2.21E-16	6.85E-16
PRR4	1.045329	0.233916	2.24E-16	6.92E-16
IGSF9	-1.56873	-0.04331	2.26E-16	6.98E-16
TMEM26	1.650274	-3.07995	2.42E-16	7.47E-16
HP3-ACAI	1.601295	-3.22113	2.46E-16	7.60E-16
IGF2BP3	3.0909	1.353546	2.49E-16	7.69E-16
SLC25A5	-1.00752	8.048292	2.52E-16	7.79E-16
NUPR1	1.460198	7.320203	2.54E-16	7.84E-16
GLYATL1	-1.40653	6.197438	2.63E-16	8.10E-16
ADAMTS	-1.22064	0.796205	2.65E-16	8.16E-16
SAA1	4.999125	6.167277	2.65E-16	8.18E-16
TF	4.36466	5.26463	2.81E-16	8.65E-16
CD1A	2.77969	-0.88598	2.94E-16	9.04E-16
MMEL1	-1.41371	-0.7735	2.96E-16	9.09E-16
S100A8	1.541874	3.326436	2.98E-16	9.16E-16
FGFBP1	-2.66387	-1.6998	3.05E-16	9.37E-16
REC8	-1.02283	3.113178	3.15E-16	9.67E-16
PDCD1LG	1.108095	2.310437	3.21E-16	9.84E-16
HHATL	6.841243	3.531472	3.24E-16	9.94E-16
ENPP2	1.587207	8.750167	3.46E-16	1.06E-15
TM6SF1	1.016084	2.361222	3.47E-16	1.06E-15
IRF8	1.069421	4.751689	3.57E-16	1.09E-15
EPS8L3	3.554857	2.274154	3.57E-16	1.09E-15
ADTRP	-1.60057	2.588303	3.61E-16	1.10E-15
ARG1	2.655349	-0.56859	3.72E-16	1.14E-15
IFITM5	3.57035	-3.11648	4.07E-16	1.24E-15
ASIC4	2.117855	-2.61311	4.09E-16	1.25E-15
NEU4	-1.72844	0.138973	4.16E-16	1.27E-15
ACSM4	1.891267	-2.1452	4.19E-16	1.28E-15
CRX	3.647051	-2.5195	4.43E-16	1.35E-15
LCK	1.293575	3.914324	4.70E-16	1.43E-15
PLCXD2	-1.38582	1.980292	4.79E-16	1.46E-15
NPIPA9	1.759336	-3.11845	4.85E-16	1.48E-15
ONECUT	3.005049	-2.66194	5.05E-16	1.54E-15
GOLGA8N	1.167692	1.283414	5.18E-16	1.57E-15
ZNF189	1.000637	5.731261	5.20E-16	1.58E-15
CCR4	1.442828	1.550991	5.34E-16	1.62E-15
RPS6KL1	1.189919	2.334967	5.41E-16	1.64E-15
LAMC2	-1.57674	4.640457	5.52E-16	1.67E-15
MDK	1.737729	5.616269	5.71E-16	1.73E-15
GRM2	1.306689	-0.9944	5.86E-16	1.77E-15
CRACR2E	1.211063	3.882035	6.01E-16	1.82E-15
ADAMTS1	1.520678	1.243016	6.01E-16	1.82E-15
DNM1	1.465242	4.90276	6.27E-16	1.89E-15
IL34	1.292657	3.239295	6.37E-16	1.93E-15
PLAC9	1.359594	3.453426	6.52E-16	1.97E-15
STK33	-1.61753	2.303559	6.52E-16	1.97E-15
AOX1	-1.45141	6.559908	6.55E-16	1.98E-15
GRID1	1.491847	1.899368	6.74E-16	2.04E-15
DUSP23	1.043316	5.808114	6.84E-16	2.06E-15
ERVFRD-	-1.5127	-0.61599	6.92E-16	2.09E-15
ADAM29	2.228291	-3.30777	7.03E-16	2.12E-15
SLC35G3	4.220395	-3.09377	7.03E-16	2.12E-15
CCR2	1.354347	3.194346	7.32E-16	2.21E-15
ULBP2	1.046068	0.573523	7.36E-16	2.22E-15
SERPINA	1.712477	10.6134	7.49E-16	2.26E-15
GOLGA6L1	1.473572	-1.09658	7.57E-16	2.28E-15
CPNE7	2.379921	1.78781	7.73E-16	2.33E-15
AHRR	1.487438	2.018396	8.44E-16	2.54E-15
STPG2	-1.09096	-0.65729	8.60E-16	2.59E-15
RIPPLY3	-1.34793	-1.59168	8.64E-16	2.60E-15
PDE6G	-1.06495	0.630266	8.74E-16	2.63E-15
CDH9	-2.20217	0.040451	9.04E-16	2.72E-15
CHODL	-1.65691	1.003495	9.19E-16	2.76E-15
MOB1B	-1.02096	6.062559	9.31E-16	2.79E-15
GOLGA6L	4.170745	-0.49079	9.88E-16	2.96E-15
CYP3A43	1.764397	-2.40028	9.91E-16	2.97E-15
NKX2-5	4.823725	-2.42291	1.00E-15	3.01E-15
ADGRG3	1.721226	0.253173	1.05E-15	3.14E-15
TNXB	-1.1154	4.181296	1.05E-15	3.15E-15
RAB3C	1.430812	1.262016	1.08E-15	3.22E-15
SLC45A2	2.104178	-0.34757	1.08E-15	3.23E-15
CLEC18A	2.093826	3.695503	1.08E-15	3.24E-15
SLC7A9	-1.36713	4.345769	1.11E-15	3.33E-15
CCL20	2.618515	4.47024	1.13E-15	3.36E-15
SCN4A	1.656114	2.796725	1.13E-15	3.37E-15
11-196G1	1.35032	-2.89684	1.20E-15	3.59E-15
GVQW3	1.036266	2.129743	1.21E-15	3.62E-15
KCNAB3	1.406136	0.656509	1.24E-15	3.71E-15
KCNA3	1.37351	0.320242	1.35E-15	4.01E-15
TAS2R19	1.578353	-2.01408	1.37E-15	4.07E-15
GJC2	1.374172	1.721303	1.37E-15	4.07E-15
CLEC9A	1.44899	0.83656	1.38E-15	4.10E-15
IL12RB2	2.637343	2.101617	1.46E-15	4.34E-15
11-574K11	1.346323	-2.07528	1.48E-15	4.40E-15
LGALS4	3.610642	5.337478	1.48E-15	4.41E-15
PLIN5	-1.42933	1.625178	1.53E-15	4.54E-15
SLC2A5	1.745833	6.827096	1.55E-15	4.60E-15
SLC22A1	-2.00919	6.189853	1.56E-15	4.64E-15
NUTM2G	1.351061	-2.01226	1.62E-15	4.82E-15
KCNK17	3.197696	0.298515	1.67E-15	4.95E-15
SAG	2.63659	-2.86708	1.70E-15	5.04E-15
NMUR1	1.074455	1.9066	1.71E-15	5.07E-15
PRUNE2	1.319899	7.890508	1.71E-15	5.07E-15
BTLA	1.436466	0.225214	1.73E-15	5.13E-15
TGFB3	1.005382	4.484766	1.75E-15	5.19E-15
SYCE1L	1.57718	1.037872	1.76E-15	5.21E-15
ORM2	5.817832	1.55809	1.80E-15	5.34E-15
GABRR1	1.69881	-1.53395	1.82E-15	5.39E-15
ABCB5	4.519723	0.997791	1.84E-15	5.45E-15
GDF15	-1.14519	6.412373	1.84E-15	5.45E-15
ENPP1	-1.06008	3.680326	1.85E-15	5.46E-15
B3GAT2	1.128496	-0.31639	1.95E-15	5.77E-15
NRXN2	1.536644	5.032927	1.98E-15	5.84E-15
MUC22	2.505676	-3.08096	1.98E-15	5.85E-15
SVOPL	-2.12236	0.137918	2.11E-15	6.23E-15
PKHD1	-1.23958	6.277788	2.21E-15	6.51E-15
HOXC13	1.898332	-1.62877	2.24E-15	6.60E-15
QPRT	-1.15112	6.243232	2.29E-15	6.75E-15
SLC19A2	-1.0863	4.164318	2.40E-15	7.07E-15
LBP	4.649058	5.818391	2.43E-15	7.15E-15
COL14A1	-1.21163	6.075913	2.47E-15	7.26E-15
IL22RA1	1.5837	2.773569	2.60E-15	7.63E-15
CATSPER	1.190177	1.797307	2.60E-15	7.64E-15
U2AF1	1.608591	0.136736	2.75E-15	8.06E-15
HOXA13	2.449316	0.903795	3.03E-15	8.88E-15
THBS4	2.428937	3.569467	3.25E-15	9.51E-15
GOLGA6L	1.675351	-2.27885	3.29E-15	9.63E-15
TAT	4.236123	1.806512	3.30E-15	9.64E-15
SLC10A1	3.388331	-1.27476	3.31E-15	9.67E-15
ROPN1B	-1.28583	-2.99257	3.32E-15	9.69E-15
REG1B	4.915979	1.335746	3.39E-15	9.92E-15
MAP7D2	2.396493	5.131487	3.41E-15	9.97E-15
IFITM10	1.413279	3.622493	3.41E-15	9.97E-15
MMP17	2.042242	0.610982	3.43E-15	1.00E-14
TAS2R10	1.429087	-2.64972	3.43E-15	1.00E-14
PDC	1.771114	-3.14344	3.48E-15	1.02E-14
C9orf131	2.377483	-2.47798	3.62E-15	1.05E-14
PTPRN	3.039708	3.181207	3.67E-15	1.07E-14
PPEF2	1.968378	-3.05938	3.79E-15	1.10E-14
11-903H1	1.176019	-0.54906	3.82E-15	1.11E-14
GPNMB	1.76337	8.294177	3.91E-15	1.14E-14
GLYATL2	1.382585	0.508613	4.00E-15	1.16E-14
CDO1	-1.50118	0.738013	4.15E-15	1.21E-14
PDE6A	1.77201	-2.18949	4.20E-15	1.22E-14
LRRD1	1.630653	-3.07309	4.28E-15	1.24E-14

CBWD1	0.56034894	8.38E-46
CYP7B1	0.63055893	5.55E-61
CES2	0.53060446	2.12E-40
PDP2	0.53122666	1.65E-40
SP3	0.62878651	1.50E-60
DMXL1	0.55890023	1.58E-45
MYD88	0.50829346	1.10E-36
OXSR1	0.60887829	6.90E-56
SLC22A13	0.50276339	8.30E-36
LCLAT1	0.68979922	3.29E-77
ADH6	0.52532429	1.70E-39
KAT5	0.67943605	4.30E-74
TADA2B	0.63536011	3.64E-62
RELA	0.53434156	4.75E-41
FAM222B	0.6225517	4.71E-59
KDM2A	0.53100374	1.81E-40
NOC3L	0.61114508	2.11E-56
COMMD1	0.58198778	4.32E-50
MTX1	0.51539848	7.71E-38
ADCY5	0.52173103	6.86E-39
CKS1B	0.52826812	5.35E-40
VANGL1	0.63931347	3.72E-63
GOLGB1	0.51825314	2.61E-38
MMRN2	0.54776838	1.88E-43
ZBTB21	0.54083978	3.37E-42
STOX2	0.51885733	2.07E-38
MAP3K11	0.52564247	1.50E-39
ARV1	0.6220368	6.23E-59
NAA20	0.51629632	5.49E-38
THAP2	0.51221248	2.56E-37
RNF26	0.61012772	3.60E-56
PTPRM	0.53429959	4.83E-41
ZNF622	0.52685835	9.31E-40
NMNAT1	0.61725046	8.30E-58
EIF1AX	0.59698015	2.96E-53
PSMD1	0.62973431	8.82E-61
TOMM20	0.64787726	2.37E-65
AGFG1	0.51286765	2.00E-37
STAT5B	0.64962119	8.29E-66
JUP	0.50389904	5.49E-36
EIF1	0.53539787	3.10E-41
NET1	0.50261838	8.75E-36
ZNF791	0.50749057	1.47E-36
GPR160	0.5644082	1.39E-46
GOLIM4	0.67737458	1.73E-73
SLCO4C1	0.65359578	7.38E-67
RBM4	0.58350923	2.10E-50
PIFO	0.51359915	1.52E-37
UBXN2A	0.52154076	7.38E-39
CEP19	0.51740405	3.60E-38
KLHL15	0.54139675	2.68E-42
FBXO45	0.5367825	1.77E-41
SLC25A30	0.66187366	4.25E-69
CD34	0.52010133	1.28E-38
LEMD3	0.59215808	3.22E-52
C16orf91	0.50632886	2.26E-36
FAM174A	0.5147218	9.96E-38
TRMT10C	0.52206892	6.02E-39
MGA	0.51086011	4.23E-37
C12orf66	0.54485592	6.38E-43
PRPF8	0.62105225	1.07E-58
PITPNA	0.63501988	4.42E-62
DDX23	0.57310761	2.72E-48
ZBTB4	0.60239259	1.94E-54
TNK1	0.60635138	2.56E-55
ZHX3	0.54161689	2.45E-42
SLC16A13	0.57054376	8.77E-48
RALGAPA1	0.52072457	1.01E-38
ATP2A2	0.55423125	1.20E-44
DENND4A	0.56308294	2.51E-46
SLC26A9	0.52027933	1.20E-38
MFSD4A	0.56501404	1.06E-46
AKIRIN1	0.54272624	1.55E-42
MSL2	0.55890722	1.58E-45
TRAM1L1	0.60567936	3.61E-55
ANGEL2	0.5489024	1.17E-43
IQCK	0.62607492	6.78E-60
B4GAT1	0.68896107	5.95E-77
TMEM167A	0.50180198	1.18E-35
LARP7	0.57408031	1.74E-48
SRP72	0.64734005	3.27E-65
THAP6	0.55162563	3.67E-44
FZD4	0.58274016	3.03E-50
PDZK1	0.73601744	7.12E-93
DENND6A	0.54157478	2.49E-42
PDE12	0.52347434	3.49E-39
NLRP6	0.5111989	3.73E-37
RSRC1	0.55886385	1.61E-45
RAB1B	0.54397322	9.22E-43
C3orf33	0.59299606	2.13E-52
DHX36	0.53417021	5.09E-41
CTBP2	0.65027942	5.57E-66
PDIK1L	0.62425153	1.85E-59
TRAF6	0.59589067	5.09E-53
MRPS22	0.5653424	9.16E-47
SH3BP5L	0.52741017	7.50E-40
YPEL2	0.56407016	1.62E-46
PSMD2	0.53159718	1.43E-40
PCCA	0.68584214	5.28E-76
DCTN2	0.5169417	4.30E-38
CTDSP2	0.54684927	2.77E-43
CKAP5	0.52347011	3.50E-39
MED16	0.52684574	9.36E-40
ATG13	0.60815095	1.01E-55
DOLK	0.65533368	2.53E-67
ANKS4B	0.60964996	4.62E-56
TMEM9B	0.51971676	1.49E-38
EIF1AD	0.5406369	3.66E-42
SMAD2	0.52058235	1.07E-38
PPP2R2D	0.54509597	5.77E-43
LONRF3	0.52953898	3.23E-40
C11orf68	0.58995081	9.46E-52
PAAF1	0.62422246	1.88E-59
RAB6A	0.65154116	2.59E-66
ERCC4	0.64281227	4.81E-64
SUGCT	0.61206661	1.30E-56
TOM1L2	0.6057042	3.57E-55
TTLL11	0.59054249	7.09E-52
TOMM5	0.50928941	7.59E-37
MSRA	0.66784321	9.31E-71
CTDNEP1	0.52765499	6.81E-40
PLEKHF2	0.63472609	5.23E-62
A2M	0.50545819	3.11E-36
UNC119B	0.58406655	1.61E-50
LYSMD3	0.64037763	2.00E-63
ZNF613	0.53548368	3.00E-41
MBLAC2	0.58083362	7.46E-50
SLC35A4	0.62915601	1.22E-60
IP6K1	0.62449464	1.62E-59
CSTF3	0.54839363	1.45E-43
YES1	0.62842564	1.84E-60
CHMP6	0.590416	7.54E-52
TCP11L1	0.55188411	3.28E-44
BNIP3	0.54468141	6.86E-43
SMIM19	0.50373181	5.84E-36
ZSCAN2	0.68515785	8.49E-76
CDC26	0.60133396	3.32E-54
RNPEP	0.67339772	2.45E-72
EID2	0.5961644	4.45E-53
DMRTA1	0.69278659	3.94E-78
KCMF1	0.53326462	7.32E-41
SPRYD4	0.61616755	1.48E-57
SLCO3A1	0.53472048	4.08E-41
WDR25	0.53621629	2.23E-41
USF3	0.53608251	2.35E-41
KBTBD11	0.58941969	1.22E-51
RMDN1	0.61815581	5.10E-58
CCDC121	0.6617873	4.49E-69
NCKAP5	0.51305566	1.86E-37
RUFY1	0.53123241	1.65E-40
VSIG10	0.56491847	1.11E-46
FAM91A1	0.56658611	5.25E-47
WSB2	0.52710107	8.47E-40
TYMS	0.59986166	6.98E-54
PXMP2	0.62508913	1.17E-59
TCEANC	0.54360346	1.07E-42
PNMA1	0.53883649	7.66E-42
SHMT1	0.73280022	1.12E-91
SEC24C	0.62598602	7.12E-60
SMCR8	0.59856046	1.34E-53
MTHFR	0.51149273	3.34E-37
ACER2	0.55384027	1.42E-44
SCN4B	0.50834882	1.07E-36
ANO6	0.60179995	2.62E-54
FAM210A	0.60982248	4.22E-56
RIMKLA	0.55321913	1.85E-44
RPS6KA3	0.56489936	1.12E-46
CHD9	0.5983329	1.51E-53
TOP3A	0.52479706	2.09E-39
CASKIN2	0.52654921	1.05E-39
TIMM22	0.64359464	3.03E-64
MIEF2	0.52145938	7.62E-39
ERICH5	0.56916016	1.64E-47
ZBTB33	0.5234996	3.46E-39
TBL1XR1	0.54562568	4.63E-43
C18orf32	0.55188238	3.29E-44
CSTF2T	0.72282458	4.45E-88
GBA	0.69308675	3.17E-78
ACAD9	0.55428512	1.17E-44
THAP5	0.61697685	9.61E-58
NECTIN3	0.59875841	1.22E-53
FLII	0.50348051	6.40E-36
HNRNPA0	0.53324515	7.38E-41
GRB2	0.58706587	3.82E-51
ZBTB41	0.56439509	1.40E-46
UBE2N	0.59090989	5.92E-52
BET1L	0.5629025	2.71E-46
RIC8A	0.59632829	4.10E-53
IMP3	0.56346049	2.12E-46
ASB8	0.70488196	5.51E-82

CRYM	-1.183	4.113026	4.32E-15	1.25E-14
TMEM213	-2.83306	6.331151	4.33E-15	1.25E-14
CACNA1I	1.799915	-1.36843	4.43E-15	1.28E-14
MAP1B	1.00142	6.729669	4.43E-15	1.28E-14
MROH2A	3.051109	1.283622	4.45E-15	1.29E-14
CD109	1.075232	6.49198	4.47E-15	1.30E-14
MOGAT3	2.2599	2.010301	4.58E-15	1.33E-14
TBX4	1.777768	-2.42377	4.62E-15	1.34E-14
RHOXF1	1.644405	-1.57037	4.66E-15	1.35E-14
SST	4.474505	4.834512	4.91E-15	1.42E-14
ZSCAN4	1.631384	-2.2745	4.92E-15	1.42E-14
SORCS3	3.207092	4.094718	4.99E-15	1.44E-14
HAP1	-1.58946	-0.58608	5.00E-15	1.44E-14
SERPINA4	-2.23686	1.416104	5.09E-15	1.47E-14
TDO2	2.187501	1.704181	5.10E-15	1.47E-14
PGA5	2.359547	0.285628	5.14E-15	1.48E-14
TRPC7	-1.64121	-0.8593	5.46E-15	1.57E-14
CHAT	6.435192	-1.48151	5.73E-15	1.65E-14
SHOX2	2.46507	-0.59526	5.87E-15	1.69E-14
MYO1H	1.738949	-0.45351	6.17E-15	1.77E-14
PCDHGA1	1.357544	1.053712	6.46E-15	1.86E-14
RDH8	-2.81812	-1.27734	6.47E-15	1.86E-14
TDRD10	1.173508	0.993181	6.50E-15	1.87E-14
FREM3	-1.47245	-2.15465	6.63E-15	1.90E-14
SLC6A19	-2.16035	6.254424	7.03E-15	2.02E-14
NTM	1.755322	3.814738	7.34E-15	2.11E-14
GRB14	-1.28518	3.93325	7.34E-15	2.11E-14
SMIM24	-1.26728	7.020565	7.35E-15	2.11E-14
NOG	2.698686	1.426981	7.64E-15	2.19E-14
P11-986E7	-1.93866	-2.09231	8.47E-15	2.42E-14
NUMBL	1.004935	3.547465	8.68E-15	2.48E-14
BSND	-3.26583	3.478346	8.72E-15	2.49E-14
CCDC180	1.339941	0.835184	8.86E-15	2.53E-14
SPDYE1	1.312524	-1.50172	9.00E-15	2.57E-14
SLC13A4	1.304382	0.788689	9.22E-15	2.63E-14
CATSPER(	1.633222	2.156297	9.27E-15	2.65E-14
FAM183A	1.785378	0.317679	9.35E-15	2.67E-14
GRM4	2.5018	-1.57437	9.77E-15	2.79E-14
RSPO4	2.614916	-0.16569	1.01E-14	2.87E-14
VGLL3	-1.30423	3.190994	1.03E-14	2.92E-14
MEI1	1.382027	1.445831	1.03E-14	2.93E-14
BIK	-1.30095	-0.22816	1.04E-14	2.96E-14
KCNH7	-1.1969	-2.73031	1.07E-14	3.04E-14
RAD54B	1.084431	1.477999	1.07E-14	3.06E-14
ST14	-1.22505	6.294343	1.12E-14	3.19E-14
ALDH1L2	1.332791	2.778677	1.13E-14	3.21E-14
ODF3L2	1.174647	-1.6799	1.14E-14	3.24E-14
FAM184B	-1.3576	-0.00031	1.19E-14	3.37E-14
COL9A3	-1.32504	0.282529	1.20E-14	3.39E-14
CCDC8	-1.30569	4.313501	1.22E-14	3.47E-14
PROX2	1.289671	-1.24106	1.24E-14	3.51E-14
H2BC7	2.834313	-1.97937	1.27E-14	3.60E-14
KDR	1.154946	7.933067	1.29E-14	3.64E-14
P2RY14	1.12349	2.441572	1.30E-14	3.67E-14
11-314N13	1.24834	-1.4645	1.30E-14	3.67E-14
RGS17	1.551368	0.877279	1.32E-14	3.74E-14
LSAMP	-2.1487	2.005804	1.33E-14	3.76E-14
PF4V1	2.131347	0.610101	1.34E-14	3.78E-14
FA2H	-1.68266	2.034224	1.36E-14	3.85E-14
TAC4	1.274978	-1.93358	1.37E-14	3.87E-14
LRRC7	1.501223	1.735564	1.38E-14	3.89E-14
ADGRA1	-2.41839	-2.04437	1.40E-14	3.95E-14
SCARA5	-1.9169	0.470348	1.41E-14	3.97E-14
RTN4R	1.102549	1.65577	1.41E-14	3.99E-14
SH3TC2	1.002321	0.332835	1.44E-14	4.08E-14
SERPINA1	3.986001	-2.21696	1.48E-14	4.17E-14
TRABD2A	1.685787	1.683585	1.49E-14	4.22E-14
CECR2	-1.46231	0.619112	1.56E-14	4.41E-14
NLRP7	2.098694	-2.21366	1.59E-14	4.48E-14
ANOS1	1.023048	3.247767	1.65E-14	4.64E-14
SLCO1A2	-2.22576	0.34646	1.65E-14	4.64E-14
TCTE1	1.480849	-2.21535	1.73E-14	4.86E-14
UROC1	1.9893	-2.54899	1.80E-14	5.07E-14
MUC16	2.942943	0.605609	1.82E-14	5.12E-14
51-393G12	2.298678	-3.32622	1.85E-14	5.20E-14
GDNF	-1.84485	-0.41846	2.04E-14	5.71E-14
MPRSS11	2.826669	-2.12339	2.05E-14	5.73E-14
FAM229A	1.15013	1.381297	2.06E-14	5.78E-14
SLC18A2	2.157235	1.328217	2.09E-14	5.86E-14
HRH4	1.189932	-2.7426	2.10E-14	5.87E-14
MAFA	-1.85731	-2.28619	2.14E-14	6.00E-14
LIPG	-1.32259	3.878716	2.21E-14	6.18E-14
FOXC2	1.260892	3.765007	2.24E-14	6.26E-14
SLC6A7	1.900053	-3.11714	2.24E-14	6.26E-14
RBFOX1	-1.99648	-1.76257	2.35E-14	6.56E-14
MAGEB17	2.274996	-2.43168	2.36E-14	6.58E-14
MNX1	2.477476	-0.77443	2.37E-14	6.62E-14
RDH16	2.105414	-0.20132	2.41E-14	6.72E-14
HACD1	-1.07401	1.055783	2.46E-14	6.87E-14
BTBD11	-1.45573	2.960964	2.47E-14	6.88E-14
LRRK2	1.074145	7.3262	2.48E-14	6.92E-14
CLDN7	-1.16379	5.449562	2.51E-14	7.00E-14
KAAG1	-1.077	-0.7311	2.53E-14	7.06E-14
L3MBTL1	1.016052	2.738241	2.59E-14	7.22E-14
CNR1	1.705318	1.202261	2.71E-14	7.54E-14
CA4	-1.45472	3.531588	2.80E-14	7.79E-14
TREX2	1.909715	1.687499	2.82E-14	7.84E-14
BHMT	-1.27454	8.184772	2.98E-14	8.27E-14
SYT12	1.975735	2.618276	3.01E-14	8.34E-14
AKR1D1	3.845016	-1.23666	3.07E-14	8.52E-14
DEFB1	-1.71178	7.306057	3.15E-14	8.74E-14
NR4A3	-1.257	4.56598	3.18E-14	8.80E-14
ADAM32	1.494741	0.062304	3.26E-14	9.03E-14
IZUMO1	1.586458	-1.52759	3.31E-14	9.15E-14
CADM3	2.701605	4.190406	3.33E-14	9.22E-14
GABRR2	1.320784	-0.39699	3.40E-14	9.41E-14
DMRT3	4.259194	-2.34039	3.42E-14	9.47E-14
TPSB2	1.703521	3.152666	3.51E-14	9.70E-14
H4C9	1.151911	2.395828	3.52E-14	9.72E-14
GUCA1B	1.137675	-0.74289	3.58E-14	9.88E-14
RAB39A	1.427034	0.009847	3.60E-14	9.93E-14
SMCO2	1.674376	-2.09125	3.66E-14	1.01E-13
TSKS	1.411618	-2.10795	3.68E-14	1.02E-13
AJAP1	-1.16452	3.672281	3.77E-14	1.04E-13
ACP4	1.476577	-3.12752	3.77E-14	1.04E-13
SCN1A	2.104133	-0.57719	3.87E-14	1.07E-13
PKD1L3	1.417866	-1.98612	3.96E-14	1.09E-13
RNF207	1.157495	3.531413	3.96E-14	1.09E-13
CALML6	2.074791	-2.53483	4.00E-14	1.10E-13
TRPC5OS	-1.29846	-2.08845	4.02E-14	1.11E-13
TAFA5	1.39302	2.302589	4.19E-14	1.15E-13
SYCE2	1.031083	-0.57659	4.27E-14	1.17E-13
ADAM12	1.909828	3.94073	4.41E-14	1.21E-13
RNASE4	1.039942	2.334881	4.67E-14	1.28E-13
SCGB1D2	-1.47009	0.615503	4.97E-14	1.36E-13
APOM	-1.24638	5.048789	4.98E-14	1.37E-13
DLG2	-1.15238	2.138153	5.02E-14	1.38E-13
ACE	1.112785	5.393179	5.13E-14	1.41E-13
PRAME	2.803049	3.780261	5.23E-14	1.43E-13
SERPINA9	3.239147	-3.22794	5.26E-14	1.44E-13
ISL2	2.448472	-2.84428	5.31E-14	1.45E-13
CALCRL	1.005729	6.849365	5.33E-14	1.46E-13
ATF3	-1.20936	6.565545	5.37E-14	1.47E-13
TMIGD1	-1.34981	-0.70206	5.39E-14	1.48E-13
CALB2	1.946769	-0.69829	5.42E-14	1.48E-13
AZU1	2.050583	-2.41431	5.53E-14	1.51E-13
GSTM5	-1.23652	1.336216	5.63E-14	1.54E-13
H2AC11	1.780992	0.549178	5.75E-14	1.57E-13
AGAP9	1.23569	2.04149	5.77E-14	1.57E-13
HCAR1	-1.52245	1.425747	5.87E-14	1.60E-13
APLNR	1.35547	6.106653	6.02E-14	1.64E-13
SYT5	2.263191	-1.56171	6.23E-14	1.70E-13
TGM3	1.786413	-1.97045	6.28E-14	1.71E-13
CACNA1A	1.191116	0.861298	6.32E-14	1.72E-13
BLACAT1	-1.49056	-1.00232	6.43E-14	1.75E-13
GABBR1	1.139147	4.306839	6.51E-14	1.77E-13
SLC46A2	1.428233	-1.64246	6.82E-14	1.86E-13
GRIK1	2.349184	-0.04597	6.89E-14	1.87E-13
SOWAHD	1.06833	0.719618	6.95E-14	1.89E-13
CES3	1.772958	6.003649	7.34E-14	1.99E-13
FLRT2	-1.12797	2.807313	7.46E-14	2.03E-13
C18orf63	1.922662	-2.40681	7.72E-14	2.09E-13
CCR1	1.139235	4.062496	7.87E-14	2.13E-13
PAX9	2.763261	0.158491	7.87E-14	2.13E-13
CLEC18B	1.703148	4.575602	8.04E-14	2.18E-13
CBY2	-1.91611	-2.98152	8.19E-14	2.22E-13
AFF2	1.21025	1.693387	8.24E-14	2.23E-13
RASAL1	-1.05015	4.631589	8.48E-14	2.29E-13
TMEM249	2.253685	-3.16808	8.95E-14	2.42E-13
GNGT1	2.254175	0.448549	9.13E-14	2.47E-13
NXF3	-1.16097	-1.79528	9.50E-14	2.56E-13
ADAMTS1	1.377582	2.996993	9.72E-14	2.62E-13
GRK7	1.323809	-2.94836	9.73E-14	2.62E-13
ZNF750	-1.2116	-0.73373	9.81E-14	2.65E-13
ARMS2	1.452546	-3.14438	9.94E-14	2.68E-13
FOXD4	1.134882	-0.44121	9.96E-14	2.68E-13
AGTR2	-2.57444	-2.64459	1.01E-13	2.71E-13
ZNF732	1.208439	-2.07272	1.02E-13	2.74E-13
CRACD	-1.25462	0.855021	1.03E-13	2.78E-13
BARX1	3.997465	-1.35794	1.05E-13	2.84E-13
RSRP1	1.051466	5.388312	1.07E-13	2.87E-13
IL10	1.327138	-0.08049	1.07E-13	2.87E-13
CYP2C8	1.875546	1.889214	1.07E-13	2.87E-13
CLVS1	1.873879	-1.67606	1.07E-13	2.88E-13
OCM	1.453303	-3.08433	1.09E-13	2.94E-13
DISP3	1.624517	-1.00443	1.12E-13	3.01E-13
ZDHHC11	1.43964	1.540694	1.14E-13	3.05E-13
NR1I2	1.507421	-0.84292	1.16E-13	3.11E-13
SGO1	1.060905	-0.0088	1.22E-13	3.28E-13
CLEC4E	1.400165	2.4462	1.23E-13	3.31E-13
RP11-33O4	1.468886	-0.36956	1.33E-13	3.56E-13
PTPRT	-1.58903	-1.03304	1.34E-13	3.58E-13
HTR3B	-2.65118	-2.86546	1.35E-13	3.60E-13
CCDC146	1.99073	6.230512	1.35E-13	3.61E-13
CD22	1.261054	2.228132	1.37E-13	3.66E-13
HOXB1	-2.19172	-3.23303	1.40E-13	3.75E-13
GOLGA8S	1.493284	-3.0556	1.45E-13	3.87E-13
SAA4	4.50852	-0.98211	1.46E-13	3.89E-13
MEST	-1.16419	4.230323	1.50E-13	4.00E-13
SUMO4	1.526389	-2.50886	1.52E-13	4.04E-13

C2orf69	0.54162034	2.44E-42
DDX10	0.66360196	1.42E-69
ZNF366	0.54572208	4.44E-43
PARD6G	0.5196726	1.51E-38
POGLUT3	0.53174844	1.34E-40
ZNF543	0.51558798	7.18E-38
GALNT11	0.60891352	6.78E-56
PLEKHM3	0.53814464	1.02E-41
DNAJC22	0.63413523	7.33E-62
NT5DC1	0.54414645	8.58E-43
KLHL11	0.55066284	5.53E-44
SLC25A20	0.69466114	1.02E-78
MAF	0.62439813	1.71E-59
SUZ12	0.59510608	7.52E-53
DHFR2	0.66573628	3.62E-70
FAM219B	0.67310577	2.98E-72
MPI	0.66607775	2.91E-70
APOLD1	0.50039043	1.96E-35
TAF7	0.58331932	2.30E-50
PFAS	0.53465424	4.19E-41
RMI1	0.53807022	1.05E-41
FBXO34	0.59616475	4.45E-53
MRFAP1L1	0.6330917	1.33E-61
SNX18	0.51015238	5.51E-37
MRFAP1	0.68571272	5.78E-76
KLHDC7A	0.53551503	2.96E-41
TMEM107	0.5781047	2.68E-49
CCDC89	0.62853327	1.73E-60
TMTC2	0.60000892	6.48E-54
FARSA	0.5957316	5.51E-53
SPTY2D1	0.62878733	1.50E-60
EDC3	0.54157828	2.49E-42
TCAIM	0.53978523	5.20E-42
FUCA1	0.51671762	4.68E-38
ZNF664	0.58387258	1.77E-50
RAD23A	0.56273084	2.93E-46
PTPN11	0.56915128	1.65E-47
ELMOD2	0.64450114	1.77E-64
GEMIN4	0.60279778	1.58E-54
ZBTB18	0.62215637	5.84E-59
SLITRK4	0.50670944	1.97E-36
GCC1	0.57337734	2.40E-48
CDC42EP4	0.55977509	1.08E-45
LACC1	0.56556762	8.28E-47
SERTAD2	0.66992581	2.40E-71
PDXDC1	0.64958241	8.49E-66
SEPHS2	0.71725774	3.88E-86
C14orf119	0.52694156	9.01E-40
BBS10	0.59350704	1.66E-52
DCTPP1	0.59495944	8.09E-53
TSHZ1	0.68331984	3.02E-75
ZADH2	0.59454351	9.93E-53
ZNF48	0.51097244	4.06E-37
EXOC3	0.52456643	2.28E-39
MED14	0.5123702	2.41E-37
FAHD1	0.66775803	9.84E-71
PRKRA	0.56432252	1.44E-46
ZNRF2	0.66662764	2.04E-70
OAZ2	0.59014871	8.59E-52
CCDC43	0.73377356	4.88E-92
TIGD2	0.55094293	4.91E-44
PAK2	0.63212124	2.30E-61
MCFD2	0.67140076	9.15E-72
MIGA1	0.69353573	2.30E-78
NRIP1	0.55681674	3.92E-45
H2AC6	0.60966186	4.59E-56
MB21D2	0.67532795	6.80E-73
PCGF5	0.56278192	2.86E-46
YOD1	0.54279485	1.50E-42
TMEM64	0.56191042	4.21E-46
ZFP3	0.55263423	2.38E-44
HOXC10	0.60633313	2.58E-55
ZNF792	0.51519943	8.31E-38
KCTD2	0.64761336	2.77E-65
CMTR2	0.57948992	1.40E-49
PITPNB	0.54813284	1.61E-43
TCEAL8	0.62054993	1.40E-58
LRRC57	0.58210112	4.10E-50
BBS12	0.59020322	8.36E-52
SLC25A42	0.5670719	4.22E-47
PJA1	0.62415557	1.95E-59
DHTKD1	0.60976031	4.36E-56
POLR2A	0.63113892	4.01E-61
TMEM102	0.51491179	9.27E-38
ZNF322	0.59333776	1.80E-52
ZBTB2	0.54124525	2.85E-42
RNF135	0.56203189	3.99E-46
MRPS23	0.51543024	7.62E-38
YIPF6	0.67655535	2.99E-73
DIPK2A	0.53873062	8.00E-42
AMIGO1	0.52865094	4.60E-40
TMEM252	0.5051929	3.43E-36
SIAH2	0.56249068	3.26E-46
RELL1	0.5387632	7.90E-42
RNF41	0.64818482	1.97E-65
IBA57	0.61520184	2.48E-57
ZNF329	0.59812468	1.67E-53
C5orf24	0.66343626	1.58E-69
ADO	0.63192776	2.57E-61
PRKAG1	0.61091561	2.38E-56
CCDC149	0.59079895	6.25E-52
SNRPE	0.54142633	2.65E-42
GET1	0.68854506	7.97E-77
TDRKH	0.53044834	2.25E-40
IST1	0.65044282	5.04E-66
CREB3L2	0.55510402	8.24E-45
P2RY8	0.50212917	1.04E-35
P2RY8	0.50212917	1.04E-35
MRPS16	0.59300183	2.13E-52
ATP6AP2	0.53476385	4.01E-41
CYB5D1	0.65807494	4.63E-68
SYNM	0.62851398	1.75E-60
TMIGD1	0.53537317	3.13E-41
ZSCAN22	0.50980692	6.26E-37
KBTBD3	0.65003985	6.44E-66
EXOC7	0.64662538	5.01E-65
CEP97	0.54636254	3.40E-43
ADI1	0.52032354	1.18E-38
RWDD4	0.6531331	9.80E-67
PLCB1	0.54085135	3.35E-42
SKA2	0.56831323	2.41E-47
IGIP	0.518859	2.07E-38
PAQR7	0.51594322	6.27E-38
NGRN	0.59029604	7.99E-52
CCDC87	0.61882149	3.56E-58
DDX28	0.53832509	9.44E-42
ACBD3	0.66625484	2.60E-70
C16orf72	0.62773542	2.70E-60
ALG12	0.61246805	1.05E-56
GLUD2	0.74258983	2.26E-95
C11orf54	0.70274844	2.72E-81
SRPRA	0.65914157	2.38E-68
HMGN4	0.68894281	6.03E-77
AP2A2	0.67236093	4.86E-72
SLC25A21	0.5597286	1.10E-45
ABAT	0.57088635	7.50E-48
AFMID	0.54069789	3.57E-42
ARHGEF37	0.57540952	9.40E-49
RABIF	0.64921752	1.06E-65
DAZAP2	0.66400635	1.10E-69
SELENOF	0.57762419	3.36E-49
ZNF623	0.50866536	9.56E-37
BCOR	0.62975028	8.75E-61
JRKL	0.51268793	2.14E-37
RIPK4	0.52180775	6.66E-39
SF3A3	0.62255824	4.69E-59
GTF2H2C	0.54154011	2.52E-42
ASB7	0.670496	1.66E-71
COA5	0.54241631	1.76E-42
PRR14L	0.5220441	6.08E-39
ACSM5	0.52338344	3.62E-39
SETD3	0.72199303	8.73E-88
ZNRF3	0.52827279	5.34E-40
TRMT12	0.64034894	2.04E-63
TMEM50A	0.64899196	1.21E-65
TBK1	0.5536573	1.54E-44
CBX6	0.52909206	3.86E-40
LIN9	0.50905451	8.28E-37
BTBD9	0.60844451	8.65E-56
TOB2	0.66057571	9.65E-69
ST6GALNAC3	0.54083516	3.38E-42
DENND5A	0.54022781	4.33E-42
DIABLO	0.56302511	2.57E-46
EIF3C	0.56106012	6.13E-46
NIPSNAP1	0.67878588	6.67E-74
SCFD2	0.588139	2.28E-51
PPP1R2	0.55512389	8.17E-45
TM2D3	0.58398447	1.67E-50
PRKD1	0.666439	2.31E-70
MAML2	0.66554948	4.09E-70
COPB2	0.59059876	6.90E-52
LRRC19	0.50583396	2.71E-36
FOXO4	0.57984487	1.19E-49
ZFP1	0.64231635	6.43E-64
SNN	0.55663447	4.25E-45
EIF4ENIF1	0.63780803	8.90E-63
ATL3	0.55441631	1.11E-44
TMEM186	0.6430928	4.07E-64
RBM43	0.52554163	1.56E-39
SUMO3	0.60660582	2.24E-55
IMMP2L	0.50431238	4.73E-36
USP18	0.62322339	3.26E-59
TMEM106A	0.66325499	1.77E-69
BRI3BP	0.51351271	1.57E-37
AP3M1	0.66368487	1.35E-69
INTS5	0.68647711	3.39E-76
FAF1	0.68040624	2.22E-74
NSMCE3	0.60797751	1.10E-55
C6orf120	0.58955402	1.15E-51
PURA	0.62696966	4.13E-60

CCL3L1	1.719931	1.799389	1.55E-13	4.13E-13
CLEC4F	1.551307	-0.2506	1.60E-13	4.27E-13
CSAG1	2.93229	-1.62899	1.67E-13	4.44E-13
CTSE	2.597851	1.940392	1.71E-13	4.54E-13
TRIM7	1.471926	2.133057	1.72E-13	4.57E-13
DOX1	2.80336	-1.95773	1.72E-13	4.57E-13
NR4A2	-1.1619	4.626449	1.72E-13	4.58E-13
C8G	1.90451	0.336535	1.74E-13	4.63E-13
NRXN3	1.396927	3.597144	1.79E-13	4.76E-13
LIPN	1.564077	-2.71432	1.82E-13	4.84E-13
ARNTL2	1.018763	2.885568	1.88E-13	4.99E-13
LRRC4B	1.030338	1.653383	1.94E-13	5.14E-13
CABP7	1.09885	-1.75747	1.94E-13	5.15E-13
PPM1N	1.13067	0.025234	1.96E-13	5.18E-13
MAPK12	1.296883	4.080825	1.97E-13	5.22E-13
SERPINE	1.316847	7.164989	2.01E-13	5.31E-13
H2BC12	1.118751	3.572559	2.09E-13	5.54E-13
BMP8B	1.221786	2.805866	2.17E-13	5.75E-13
CILP2	2.713511	0.704261	2.28E-13	6.01E-13
RSPO3	-1.27487	1.769539	2.29E-13	6.04E-13
PHEX	1.299337	1.223539	2.29E-13	6.05E-13
ADCY8	3.243172	2.422595	2.31E-13	6.11E-13
SCARA3	-1.20316	4.793256	2.33E-13	6.14E-13
FNDC5	-1.26965	0.279208	2.38E-13	6.28E-13
TMPRSS6	2.263346	0.348032	2.44E-13	6.43E-13
MKRN2O	-1.13888	-1.33916	2.44E-13	6.44E-13
SLC22A4	1.063363	3.841451	2.56E-13	6.74E-13
CCDC17	1.232429	0.276572	2.60E-13	6.85E-13
UPP1	1.148162	5.358147	2.70E-13	7.09E-13
PMP2	-2.39213	-2.4874	2.72E-13	7.14E-13
LRRTM4	2.760534	-0.24731	2.73E-13	7.17E-13
SLC1A6	4.509627	0.194204	2.89E-13	7.59E-13
KLK4	6.14696	2.691323	2.91E-13	7.64E-13
TNFSF11	2.13482	-0.65096	2.94E-13	7.71E-13
MYH7B	1.182378	1.53599	2.98E-13	7.81E-13
ZDHHC11	1.658596	2.218547	3.01E-13	7.89E-13
INHA	3.175246	2.650561	3.04E-13	7.97E-13
TRPV1	1.382413	-1.68582	3.25E-13	8.51E-13
C1QTNF1	1.19853	5.93816	3.29E-13	8.61E-13
SPDYE2	1.392458	-1.8686	3.30E-13	8.65E-13
GRPR	1.943365	-0.9759	3.42E-13	8.95E-13
AQP4	-1.32696	2.270451	3.43E-13	8.97E-13
PCP4	-2.38416	2.515435	3.46E-13	9.05E-13
PRR36	-1.41385	1.180542	3.51E-13	9.18E-13
PPP1R1C	-1.63334	-0.81992	3.52E-13	9.20E-13
SCUBE3	-1.1116	2.737693	3.61E-13	9.44E-13
NELL2	1.388468	2.018225	3.66E-13	9.56E-13
PRTN3	2.646565	-1.79968	3.71E-13	9.68E-13
TDGF1P3	-2.62218	-2.58571	3.78E-13	9.86E-13
SYTL5	-1.54498	0.839672	3.88E-13	1.01E-12
CCDC74B	1.264462	2.415419	3.88E-13	1.01E-12
DNAI2	1.331235	-2.52718	3.92E-13	1.02E-12
ENTPD2	1.261438	3.687403	3.93E-13	1.03E-12
ANGPTL3	-1.7487	3.836218	4.07E-13	1.06E-12
ORM1	5.523213	2.294464	4.11E-13	1.07E-12
ENTHD1	2.111347	-2.96312	4.22E-13	1.10E-12
P5-864K1	1.164663	-2.67668	4.32E-13	1.12E-12
P11-691N	1.260424	-1.66633	4.34E-13	1.13E-12
P11-316F1	1.682811	-0.72973	4.74E-13	1.23E-12
CHST1	1.466152	3.675952	4.78E-13	1.24E-12
GFRA3	-1.59514	-1.9011	4.88E-13	1.27E-12
ZAN	3.474642	-1.58572	4.95E-13	1.29E-12
TDRD1	-1.90658	-0.06133	4.97E-13	1.29E-12
MISP	-1.64023	2.771591	5.07E-13	1.31E-12
MARCHF1	-1.86872	-0.01776	5.07E-13	1.32E-12
AQP9	2.253074	3.704604	5.25E-13	1.36E-12
CYP2D6	1.570337	0.356979	5.50E-13	1.43E-12
PRKCG	2.703817	-2.68922	5.61E-13	1.45E-12
C6orf163	1.076799	-0.31999	5.83E-13	1.51E-12
STEAP4	1.30321	5.699357	5.88E-13	1.52E-12
TEDC2	1.025191	0.261942	5.91E-13	1.53E-12
BEX2	-1.01752	4.195301	6.07E-13	1.57E-12
M23B-AG	1.257049	-2.10237	6.31E-13	1.63E-12
CAVIN4	1.062354	-1.08233	6.86E-13	1.77E-12
TREML4	2.455716	-3.03964	6.90E-13	1.78E-12
SRD5A2	-1.62117	-1.68699	7.00E-13	1.80E-12
COL3A1	1.334761	9.466888	7.18E-13	1.85E-12
DISP2	1.290245	0.714714	7.24E-13	1.86E-12
SPDEF	2.63262	-0.48819	7.27E-13	1.87E-12
RPH3A	1.540053	-1.12961	7.30E-13	1.88E-12
CST2	2.905791	-1.49418	7.35E-13	1.89E-12
NXNL2	1.125248	2.639636	7.36E-13	1.89E-12
F12	1.779443	2.559854	7.56E-13	1.94E-12
FERMT1	-1.31765	2.086545	8.14E-13	2.09E-12
KLKB1	1.454971	2.307953	8.20E-13	2.10E-12
GSG1L	-1.69142	-3.12966	8.29E-13	2.12E-12
CFD	1.275306	3.924877	8.30E-13	2.13E-12
CCDC60	1.822583	-2.1871	8.34E-13	2.13E-12
CALCB	-1.36578	-2.11011	8.49E-13	2.17E-12
ITIH3	2.494416	3.429845	8.57E-13	2.19E-12
PRMT8	-1.6363	-2.02062	8.74E-13	2.23E-12
FSD2	1.807797	-1.57016	8.76E-13	2.24E-12
MMP13	4.977893	0.299445	9.56E-13	2.44E-12
STPG4	1.736776	-3.08812	9.68E-13	2.47E-12
ZNF705E	1.156899	-2.50376	9.95E-13	2.54E-12
TPSG1	2.58551	1.889623	9.96E-13	2.54E-12
SLC7A4	-1.72037	-1.5931	9.99E-13	2.55E-12
FDCSP	6.971049	2.15532	1.01E-12	2.59E-12
PCDHGB	1.650388	2.500827	1.04E-12	2.66E-12
ARX	3.945374	0.199349	1.07E-12	2.73E-12
SLC7A7	-1.15348	6.516513	1.09E-12	2.76E-12
ALKAL2	1.691467	4.013247	1.11E-12	2.81E-12
MMP3	-2.18054	-1.10185	1.12E-12	2.84E-12
CARMIL3	1.252029	1.45613	1.15E-12	2.93E-12
GALNT16	-1.01656	3.410603	1.16E-12	2.94E-12
BMPR1B	-2.05285	3.815158	1.17E-12	2.98E-12
PLA2G10	1.140558	-2.71591	1.18E-12	2.99E-12
BGN	1.095602	9.944598	1.19E-12	3.01E-12
PIP	5.589681	1.026793	1.22E-12	3.09E-12
CITED4	1.049786	4.356212	1.24E-12	3.15E-12
STMN2	3.856577	0.481998	1.26E-12	3.19E-12
MYBPC3	1.53999	-1.86572	1.27E-12	3.22E-12
CD300E	1.109837	2.136689	1.27E-12	3.22E-12
SOX1	4.254092	-2.90443	1.27E-12	3.23E-12
WNT10A	-1.25273	1.051834	1.29E-12	3.26E-12
MAGEC3	3.569108	-1.96653	1.29E-12	3.28E-12
CBLN1	2.101798	0.079313	1.30E-12	3.30E-12
SERPIND1	3.718272	1.930889	1.32E-12	3.34E-12
EGR2	-1.16619	4.168496	1.32E-12	3.35E-12
DPYSL4	2.311949	1.150662	1.33E-12	3.36E-12
FAM71F2	1.276944	0.876332	1.35E-12	3.42E-12
L2L2-PAB	1.019213	-1.88702	1.39E-12	3.51E-12
MYOT	1.446665	0.818745	1.40E-12	3.55E-12
F2	3.70909	2.800782	1.43E-12	3.62E-12
EPHB2	-1.01244	3.192944	1.45E-12	3.67E-12
DCYAP1F	1.817544	-0.83019	1.46E-12	3.68E-12
PTPRH	2.639649	1.675207	1.46E-12	3.70E-12
PCDH11Y	-1.74984	-1.95607	1.50E-12	3.78E-12
SLC4A5	1.503055	0.488832	1.50E-12	3.78E-12
FRZB	1.624236	7.043957	1.52E-12	3.84E-12
POSTN	1.574095	6.893733	1.54E-12	3.90E-12
GABBR2	1.849861	-0.73697	1.61E-12	4.07E-12
NUGGC	-1.30811	2.005674	1.66E-12	4.18E-12
ZNF90	1.057018	1.998536	1.67E-12	4.20E-12
S100A3	1.060989	1.230237	1.69E-12	4.26E-12
RNF182	-1.26339	1.385846	1.72E-12	4.33E-12
CLRN3	1.492534	5.299042	1.85E-12	4.66E-12
MSTN	1.398402	-0.28922	1.87E-12	4.71E-12
H3C8	1.927016	-1.20419	1.88E-12	4.72E-12
TUBA3D	3.028153	3.653159	1.90E-12	4.77E-12
EXTL1	-1.13348	-1.13609	1.97E-12	4.95E-12
AA2-SAA	5.556356	1.80523	1.98E-12	4.97E-12
PFKFB1	-1.0101	-1.39891	2.00E-12	5.01E-12
C6orf141	2.131309	0.394988	2.04E-12	5.11E-12
ADAD2	-1.34779	-2.84196	2.07E-12	5.17E-12
MUC5B	2.478514	-0.25306	2.18E-12	5.46E-12
DPYSL3	1.150034	6.479932	2.27E-12	5.69E-12
APOBEC3	1.200269	0.018596	2.31E-12	5.78E-12
M47E-STE	-1.00377	-3.00289	2.33E-12	5.83E-12
CXCL2	1.652148	3.429943	2.35E-12	5.86E-12
MBL2	5.072197	-1.67701	2.39E-12	5.96E-12
CTRL	1.444956	-3.26502	2.44E-12	6.10E-12
VSNL1	-1.81542	-0.3436	2.46E-12	6.14E-12
TRPC5	-1.48968	-3.03648	2.51E-12	6.27E-12
CPA4	2.99308	2.391169	2.52E-12	6.27E-12
BTBD18	1.537684	-2.33661	2.52E-12	6.29E-12
SOX21	2.546705	-3.01753	2.53E-12	6.30E-12
ROS1	-2.41996	-0.06219	2.54E-12	6.32E-12
CDK5R2	3.03682	-1.76247	2.66E-12	6.63E-12
METTL27	1.082252	3.109919	2.67E-12	6.65E-12
RDH13	1.005253	5.045713	2.68E-12	6.66E-12
INSL3	1.437548	-1.28492	2.68E-12	6.66E-12
CYP8B1	-1.45398	3.501172	2.69E-12	6.68E-12
H2AW	1.657886	1.644772	2.69E-12	6.69E-12
SULF1	1.109178	6.856111	2.71E-12	6.73E-12
ALDH1L1	-1.16058	6.843801	2.74E-12	6.80E-12
FCN1	1.088145	3.560716	2.74E-12	6.80E-12
H2BC6	1.806419	-1.50228	2.82E-12	7.00E-12
PDIA2	3.237347	-0.483	3.03E-12	7.51E-12
FRMPD3	1.477176	-1.07382	3.09E-12	7.67E-12
ACTN3	1.507868	-2.83045	3.11E-12	7.71E-12
MGAT3	1.297069	4.839128	3.12E-12	7.73E-12
ADORA2E	1.012886	1.378789	3.16E-12	7.83E-12
EGR4	-1.70922	-2.61208	3.23E-12	7.99E-12
NR0B2	-2.72193	1.912661	3.32E-12	8.22E-12
GPR20	1.296432	0.028408	3.34E-12	8.26E-12
KRBOX1	-1.22157	-1.24502	3.38E-12	8.36E-12
GLTPD2	-1.07752	1.965686	3.46E-12	8.55E-12
IGLL5	2.709249	5.421723	3.46E-12	8.55E-12
A1BG	1.350416	-1.50139	3.52E-12	8.69E-12
CMTM2	1.208922	-1.19801	3.55E-12	8.77E-12
GABRQ	1.487593	1.47013	3.70E-12	9.12E-12
CALY	3.01853	-1.30146	3.76E-12	9.28E-12
CYP4A22	-1.82474	3.618641	3.91E-12	9.64E-12
EEF1G	1.182102	-0.9953	3.96E-12	9.76E-12
LHX9	4.946311	0.859718	4.39E-12	1.08E-11
CLDN8	-3.10221	5.002671	4.59E-12	1.13E-11
AC004076.	1.433309	-3.03626	4.74E-12	1.17E-11
H3C6	1.174835	-0.26876	4.74E-12	1.17E-11
CCNE1	1.065626	1.006982	4.95E-12	1.21E-11

PPIL6	0.50852094	1.01E-36
KIAA0825	0.58782909	2.65E-51
TCN2	0.63308628	1.33E-61
RAD51D	0.50069643	1.75E-35
MRPL30	0.66349355	1.52E-69
TARS3	0.57131814	6.16E-48
METTL7A	0.62274081	4.24E-59
SP1	0.53837002	9.27E-42
PBX1	0.54027753	4.25E-42
SYN3	0.51883528	2.09E-38
MOSMO	0.55992687	1.01E-45
DRG1	0.56450424	1.33E-46
ANKFY1	0.64348398	3.23E-64
YTHDF3	0.58629394	5.54E-51
SRL	0.515126	8.55E-38
IFIT1	0.61807382	5.33E-58
CXorf38	0.56881661	1.92E-47
MORF4L1	0.69056433	1.92E-77
PIGP	0.63554511	3.27E-62
THNSL1	0.64561665	9.14E-65
TRIM69	0.56573894	7.67E-47
LAMP1	0.6270355	3.98E-60
BICD2	0.54267821	1.58E-42
TMLHE	0.65420457	5.08E-67
TAL2	0.62352785	2.76E-59
AIDA	0.66410516	1.03E-69
CDIN1	0.51056492	4.73E-37
ANKRD46	0.67968836	3.62E-74
ZBTB6	0.56726876	3.86E-47
POLR3C	0.66919402	3.87E-71
WWOX	0.64538228	1.05E-64
MTARC1	0.59159546	4.24E-52
SOWAHB	0.64472927	1.55E-64
BLOC1S4	0.50699767	1.77E-36
KLHL32	0.59306011	2.06E-52
MRTFB	0.55905048	1.48E-45
KDM4D	0.6104082	3.11E-56
PPP1CC	0.52074521	1.00E-38
NKRF	0.54268685	1.57E-42
KPNA4	0.54981534	7.92E-44
ZNF396	0.55173652	3.50E-44
SEPTIN10	0.68345357	2.76E-75
GPATCH8	0.50940799	7.26E-37
KATNA1	0.64315722	3.92E-64
ZFP91	0.65147425	2.70E-66
BCDIN3D	0.57006912	1.09E-47
LYRM7	0.60842624	8.73E-56
BCR	0.50384168	5.61E-36
SPIN4	0.52734797	7.68E-40
HEXIM1	0.57127669	6.28E-48
QRFPR	0.50321958	7.03E-36
MAPT	0.62860002	1.67E-60
TMEM17	0.59033849	7.83E-52
PPARA	0.59847571	1.40E-53
CYP4A11	0.558778	1.67E-45
TEAD1	0.60761955	1.33E-55
ENTPD5	0.66550251	4.21E-70
SHTN1	0.54328418	1.23E-42
TSPYL4	0.56022187	8.86E-46
SESTD1	0.6339524	8.13E-62
DYNC2H1	0.60852448	8.30E-56
TAF9B	0.62877127	1.51E-60
CHP1	0.56239042	3.41E-46
HSPA14	0.5448029	6.52E-43
AGMO	0.54482366	6.47E-43
USP7	0.58017454	1.02E-49
MAGEH1	0.56313375	2.45E-46
B3GLCT	0.54801842	1.69E-43
TMEM203	0.52813183	5.64E-40
TCEA1	0.54466608	6.91E-43
NHEJ1	0.64178224	8.80E-64
TMEM220	0.63965901	3.04E-63
PABIR1	0.61388796	4.98E-57
UBQLN2	0.62697238	4.12E-60
SMTNL2	0.61831583	4.68E-58
NCR3LG1	0.529975	2.72E-40
ZNF669	0.57908332	1.70E-49
GTF2F2	0.50943204	7.20E-37
FOCAD	0.56578995	7.50E-47
CHM	0.62335708	3.03E-59
BLOC1S5	0.7287918	3.27E-90
SRSF10	0.51613262	5.84E-38
NBR1	0.77144556	2.57E-107
SUMO2	0.50657541	2.07E-36
RTL6	0.64379036	2.70E-64
PTAR1	0.59710527	2.78E-53
ZDHHC9	0.67842439	8.52E-74
SMIM15	0.59648495	3.79E-53
MTF1	0.59955074	8.17E-54
NHLRC3	0.50566592	2.88E-36
LRRK2	0.58758946	2.97E-51
TRMT2B	0.58486346	1.10E-50
NYX	0.50001216	2.24E-35
FAM120AOS	0.58000462	1.10E-49
NOC2L	0.52806354	5.80E-40
NELFB	0.54972386	8.24E-44
KCTD21	0.57080601	7.78E-48
RNFT1	0.53560087	2.86E-41
ARID2	0.5441615	8.52E-43
SF3B3	0.58093072	7.12E-50
ZNF33A	0.50278071	8.25E-36
C15orf61	0.51789455	2.99E-38
TSPYL1	0.67820458	9.88E-74
PNRC2	0.67615309	3.92E-73
LIN54	0.63690954	1.49E-62
FAM53B	0.51673497	4.65E-38
KAZN	0.59156199	4.31E-52
NEMP2	0.63705524	1.37E-62
GSPT2	0.51211619	2.65E-37
HMGB1	0.61504616	2.69E-57
TDRD7	0.68920185	5.02E-77
SPATS2L	0.6801822	2.59E-74
WDSUB1	0.65314895	9.71E-67
ZNF79	0.5161458	5.81E-38
FAT4	0.59254207	2.66E-52
ACADSB	0.67120124	1.04E-71
STK40	0.6061822	2.79E-55
MPHOSPH8	0.52988313	2.82E-40
NIF3L1	0.58237288	3.60E-50
IARS1	0.57336158	2.42E-48
ZBTB44	0.61303658	7.81E-57
WDR5	0.58242384	3.52E-50
SRGAP2B	0.5689905	1.77E-47
ASB13	0.55924923	1.36E-45
ZNF124	0.51853875	2.34E-38
XRCC6	0.6715533	8.28E-72
PPP1R26	0.50993478	5.97E-37
ZNF569	0.53690042	1.69E-41
YRDC	0.55183051	3.36E-44
PIK3R4	0.67421321	1.43E-72
PRPF40A	0.62265108	4.46E-59
GDAP2	0.53953139	5.77E-42
TPK1	0.52141961	7.74E-39
MYO18A	0.59605044	4.71E-53
MME	0.55117088	4.45E-44
PLXNB2	0.51866536	2.23E-38
MYO6	0.67650529	3.10E-73
HDAC2	0.5951938	7.20E-53
ZNF136	0.5454376	5.00E-43
TRAPPC4	0.62069132	1.30E-58
TTC30B	0.67066763	1.48E-71
TECPR2	0.61702948	9.34E-58
ERI2	0.53759745	1.27E-41
ZNF33B	0.54907019	1.09E-43
AMZ2	0.58114914	6.43E-50
NF1	0.56278104	2.86E-46
MAML3	0.51411327	1.25E-37
STRN3	0.53540026	3.10E-41
ILRUN	0.6336694	9.55E-62
PPTC7	0.52602721	1.29E-39
NHLRC2	0.52609536	1.26E-39
ARHGEF12	0.65901149	2.58E-68
TMEM26	0.54057529	3.76E-42
NOP9	0.60603432	3.01E-55
FUT11	0.55379108	1.45E-44
ANXA4	0.54390187	9.49E-43
METTL9	0.75492654	2.83E-100
ZNF398	0.61882862	3.55E-58
ZSCAN25	0.52516454	1.81E-39
GMFB	0.62649396	5.38E-60
ZNF420	0.57341777	2.36E-48
ZSCAN26	0.53782368	1.16E-41
KIAA1671	0.66228351	3.28E-69
IGF2R	0.60351361	1.10E-54
DYNC1H1	0.5572564	3.24E-45
PCBP2	0.5748431	1.22E-48
PSMD12	0.62340693	2.95E-59
SLC22A4	0.52457567	2.28E-39
C1D	0.67224075	5.27E-72
TBC1D9B	0.53774377	1.20E-41
FITM2	0.64130193	1.17E-63
DDI2	0.58198709	4.32E-50
SVIL	0.53202594	1.20E-40
TRIM33	0.56186693	4.29E-46
LRP10	0.54063775	3.66E-42
PELI1	0.52347911	3.49E-39
HTT	0.51413852	1.24E-37
VEPH1	0.57884105	1.90E-49
IPP	0.67102577	1.17E-71
RPF2	0.53145619	1.51E-40
SIPA1L1	0.60738997	1.49E-55
TTC30A	0.67895421	5.95E-74
PIGN	0.61657449	1.19E-57
ZNF624	0.51070898	4.48E-37
HOXA4	0.52920725	3.69E-40
TOPORS	0.67596348	4.45E-73
DPP4	0.61512067	2.59E-57
SLC22A24	0.58854649	1.87E-51
SPTAN1	0.54325896	1.24E-42
PARVA	0.53693686	1.66E-41

ADGRG6	1.096746	6.05341	5.02E-12	1.23E-11
LYPD2	1.594555	-3.19843	5.10E-12	1.25E-11
H2AC8	1.657816	0.642121	5.12E-12	1.25E-11
NOTUM	-1.78628	-1.48537	5.35E-12	1.31E-11
SLC5A1	2.143847	5.726483	5.38E-12	1.32E-11
TTYH1	1.418947	0.157877	5.44E-12	1.33E-11
DCAF4L1	1.156704	-1.39648	5.52E-12	1.35E-11
EDIL3	1.169978	6.992767	5.66E-12	1.38E-11
NCAM2	-1.38714	-0.50041	5.73E-12	1.40E-11
FCAMR	-1.50619	3.973827	5.84E-12	1.43E-11
COL6A3	1.429995	7.769845	5.96E-12	1.46E-11
CRYAB	1.050382	9.927625	6.03E-12	1.47E-11
ACAD11	1.159912	3.116909	6.19E-12	1.51E-11
TFPI	1.08603	7.796758	6.23E-12	1.52E-11
NID2	1.018328	5.392036	6.40E-12	1.56E-11
LAMB4	-1.35241	0.963755	6.47E-12	1.57E-11
FOXL1	1.085873	2.231534	6.51E-12	1.58E-11
C19orf18	1.015807	-0.65544	6.83E-12	1.66E-11
ODAM	-2.87179	-1.36809	6.83E-12	1.66E-11
KCNJ4	1.691681	-0.35007	6.94E-12	1.69E-11
NLRP6	1.410831	2.334913	7.04E-12	1.71E-11
POBEC3I	1.011389	1.429878	7.15E-12	1.74E-11
ADGRE3	1.216414	-0.93344	7.18E-12	1.74E-11
CTRC	1.959518	-2.71226	7.66E-12	1.86E-11
SHOX	2.430757	-3.20312	7.68E-12	1.86E-11
SHOX.1	2.430757	-3.20312	7.68E-12	1.86E-11
CNDP1	-1.20442	0.984298	8.22E-12	1.99E-11
ITGA10	1.168825	2.72621	8.23E-12	1.99E-11
H3C4	1.551106	-0.80577	8.27E-12	2.00E-11
MYL3	-1.25356	2.630412	8.42E-12	2.04E-11
CALHM4	-1.20234	-3.1958	8.64E-12	2.09E-11
WNT11	-1.4148	0.527935	8.65E-12	2.09E-11
LPAR4	1.344583	-1.2076	8.70E-12	2.10E-11
CPB2	3.518572	-0.61885	9.34E-12	2.25E-11
RNF113B	2.360025	-2.96843	9.40E-12	2.27E-11
VSTM1	1.515589	-2.42409	9.49E-12	2.29E-11
XKR4	-1.59532	-0.47927	9.50E-12	2.29E-11
GAL3ST2	-1.26817	-2.76196	9.78E-12	2.36E-11
NCAN	1.752812	-3.12824	9.89E-12	2.38E-11
TNC	-1.04363	7.322616	1.05E-11	2.54E-11
NEUROD	2.117584	-2.78085	1.07E-11	2.57E-11
CSMD1	-1.30238	1.380872	1.08E-11	2.60E-11
FUT6	-1.27639	4.552924	1.10E-11	2.63E-11
PITX1	3.247884	0.645675	1.10E-11	2.64E-11
CXXC4	1.003492	2.162439	1.18E-11	2.83E-11
TCF7	1.005721	3.381352	1.18E-11	2.83E-11
PRR18	-1.65455	-2.53077	1.19E-11	2.85E-11
REG1A	2.863524	5.934515	1.19E-11	2.85E-11
FXYD1	1.846051	1.998995	1.20E-11	2.87E-11
CDH10	-1.72362	-2.7442	1.22E-11	2.93E-11
CPA3	1.342948	3.445058	1.24E-11	2.98E-11
OLIG2	3.67197	0.110142	1.26E-11	3.01E-11
C1S	1.352791	8.541481	1.28E-11	3.05E-11
TSLP	-1.10777	-0.43261	1.30E-11	3.10E-11
TGM5	3.040973	-0.6261	1.31E-11	3.14E-11
FSIP2	1.732906	1.758371	1.31E-11	3.14E-11
UST	-1.19216	2.890556	1.34E-11	3.20E-11
SLC5A12	-1.53215	6.964824	1.39E-11	3.31E-11
C17orf64	1.572052	-2.29808	1.43E-11	3.41E-11
ANXA13	1.84562	4.402683	1.46E-11	3.48E-11
SPATA32	1.002873	-1.93822	1.47E-11	3.50E-11
BPIFB1	3.080845	-2.04716	1.50E-11	3.57E-11
KCNC1	1.382519	-2.36403	1.61E-11	3.82E-11
SCN9A	1.360652	4.025982	1.62E-11	3.86E-11
CFAP46	-1.05179	1.254046	1.73E-11	4.10E-11
ETV4	-1.31548	0.850827	1.73E-11	4.11E-11
RGS9BP	-1.09874	-1.86755	1.79E-11	4.24E-11
ZYG11A	-1.27462	0.542154	1.87E-11	4.43E-11
H2BU1	1.74711	-1.35647	1.90E-11	4.50E-11
KCNS2	-1.35083	-1.56142	1.91E-11	4.52E-11
PCSK1N	-1.55086	3.692261	1.97E-11	4.67E-11
LIX1	-1.74864	3.024911	2.00E-11	4.73E-11
KCNF1	1.546547	1.235626	2.03E-11	4.80E-11
MEP1B	1.56112	-2.9567	2.06E-11	4.88E-11
AGXT	-1.76415	2.704136	2.09E-11	4.94E-11
KNDC1	1.6683	3.024093	2.14E-11	5.04E-11
CACNA1E	3.087968	-0.59975	2.17E-11	5.11E-11
STPG3	1.74195	0.045018	2.17E-11	5.12E-11
CERS1	2.074584	-0.7755	2.18E-11	5.14E-11
PROKR2	-1.86509	-3.13772	2.21E-11	5.20E-11
TRPM8	2.174101	0.135352	2.23E-11	5.25E-11
ZNF556	-1.16192	-0.41882	2.27E-11	5.34E-11
LDHC	-1.464	-0.82864	2.27E-11	5.35E-11
L928654.	1.602575	-3.43729	2.28E-11	5.36E-11
CPB1	2.332612	-0.34754	2.48E-11	5.84E-11
MIP	1.398858	-2.63983	2.62E-11	6.16E-11
CHRNA5	1.135928	-0.61379	2.64E-11	6.19E-11
POU3F1	1.402029	-0.72472	2.65E-11	6.23E-11
SLC30A1	3.529498	-0.81323	2.68E-11	6.29E-11
PKIB	1.074907	2.320299	2.73E-11	6.39E-11
PKP1	2.121481	3.278009	2.77E-11	6.49E-11
OXCT1	-1.20181	6.018782	2.78E-11	6.52E-11
GPR22	-1.88195	-1.85154	2.80E-11	6.57E-11
DCHS2	1.530109	1.571115	2.84E-11	6.65E-11
NKD2	1.846992	3.15697	2.86E-11	6.71E-11
COL11A2	1.12258	0.847243	2.96E-11	6.92E-11
FPR2	1.320678	0.500245	3.02E-11	7.06E-11
MS4A2	1.307449	1.174917	3.06E-11	7.15E-11
SALL4	1.866303	-0.51391	3.12E-11	7.30E-11
BTN1A1	1.101625	-2.71575	3.25E-11	7.57E-11
1QTNF9	1.970127	-2.24418	3.30E-11	7.69E-11
CWH43	-2.06874	2.501702	3.30E-11	7.70E-11
GLDN	2.102756	2.497136	3.42E-11	7.98E-11
CLEC17A	1.489217	-1.02064	3.44E-11	8.01E-11
CYP17A1	-1.92812	4.093253	3.56E-11	8.29E-11
KRT7	-1.62215	5.833313	3.71E-11	8.65E-11
ARMH1	1.006292	0.077702	3.94E-11	9.16E-11
FAXC	-1.6223	0.846114	3.94E-11	9.16E-11
CCBE1	-1.70189	2.165918	3.98E-11	9.24E-11
JMJD7	1.03171	-2.38706	4.00E-11	9.28E-11
PLPPR3	1.981568	-2.51103	4.05E-11	9.40E-11
HAGHL	1.795593	2.974083	4.09E-11	9.48E-11
A4GNT	1.403139	-0.08803	4.25E-11	9.84E-11
AQP7	-1.02873	3.674877	4.27E-11	9.89E-11
CSMD2	1.028911	1.415382	4.34E-11	1.00E-10
TRPM3	-1.12087	4.452963	4.51E-11	1.04E-10
SLC16A1	-1.13243	6.869895	4.76E-11	1.10E-10
MXRA8	1.268212	6.185909	4.96E-11	1.14E-10
EZHIP	1.851601	-2.9117	5.01E-11	1.16E-10
NPIPB15	1.768002	1.097033	5.06E-11	1.17E-10
RFPL2	1.58582	-2.06098	5.37E-11	1.24E-10
CDHR4	2.213832	-2.07247	5.38E-11	1.24E-10
ATE-MU	1.060655	-1.12461	5.42E-11	1.25E-10
CDH19	-2.05347	-1.17642	5.51E-11	1.27E-10
EPO	3.186676	3.449726	5.79E-11	1.33E-10
TCN1	3.313349	0.088476	5.81E-11	1.34E-10
TAS2R31	1.305179	-2.01629	6.15E-11	1.41E-10
GSDMA	1.43225	0.011315	6.20E-11	1.43E-10
TAS1R1	-1.08277	-2.42876	6.27E-11	1.44E-10
TMEM27	1.140223	-2.21958	6.28E-11	1.44E-10
IL7R	1.179226	4.849314	6.28E-11	1.44E-10
FGF11	1.670771	1.337106	6.32E-11	1.45E-10
SHISA7	1.297618	-3.30436	6.50E-11	1.49E-10
FCRL5	2.16464	1.508233	6.56E-11	1.51E-10
SPTBN5	1.267656	2.362231	6.75E-11	1.55E-10
BDKRB2	-1.1648	4.83495	6.95E-11	1.59E-10
SCN3A	-1.55838	0.761256	7.19E-11	1.64E-10
TWIST1	1.249483	0.366768	7.43E-11	1.70E-10
ERVW-1	1.482324	-1.17476	7.51E-11	1.72E-10
CYP3A4	-1.41687	0.0989	7.57E-11	1.73E-10
FLRT3	-1.1516	5.413053	7.70E-11	1.76E-10
H4C8	1.13821	1.430031	7.77E-11	1.77E-10
IGLL1	2.740678	-2.42773	7.81E-11	1.78E-10
GFAP	1.401936	1.131068	8.03E-11	1.83E-10
BRICD5	1.108953	1.126197	8.15E-11	1.86E-10
DYNLT4	1.069114	-2.15466	8.31E-11	1.89E-10
NKAIN2	-1.4729	-1.69145	8.50E-11	1.94E-10
SLIT1	1.143389	-0.57602	8.74E-11	1.99E-10
KIAA2012	1.670084	-2.71885	8.77E-11	2.00E-10
PPM1E	-1.42659	1.461593	9.10E-11	2.07E-10
SULT4A1	2.526297	-0.2474	9.62E-11	2.18E-10
GRIK4	1.70156	1.040763	9.87E-11	2.24E-10
KIF25	1.145207	-1.23466	1.00E-10	2.28E-10
IL12B	1.810534	-1.23187	1.00E-10	2.28E-10
PLCB4	1.105081	4.812725	1.02E-10	2.32E-10
FCGBP	-1.16125	5.347783	1.09E-10	2.46E-10
CPLX1	1.024841	1.943628	1.09E-10	2.46E-10
ABCG2	1.002203	4.319528	1.10E-10	2.50E-10
FOXN4	-1.46116	-2.40774	1.13E-10	2.56E-10
TMSB4Y	-1.61147	0.306764	1.14E-10	2.58E-10
GABRB2	-1.43737	0.807206	1.19E-10	2.69E-10
GTSF1	1.479636	-0.69187	1.20E-10	2.71E-10
CDC144	2.468143	0.786239	1.20E-10	2.71E-10
GPR15	2.124462	-1.10321	1.29E-10	2.91E-10
SCG5	1.628886	2.060319	1.29E-10	2.91E-10
MTTP	-1.72071	2.294861	1.29E-10	2.91E-10
FAM166A	1.784261	-2.51063	1.31E-10	2.95E-10
HAPLN2	1.168732	-0.61959	1.31E-10	2.95E-10
RHD	1.335522	-0.18624	1.34E-10	3.03E-10
STAB2	1.36905	0.050291	1.35E-10	3.03E-10
CASQ1	1.004339	-1.6468	1.39E-10	3.12E-10
GDAP1L1	1.649476	-2.13283	1.39E-10	3.13E-10
APBA2	1.25321	1.845242	1.41E-10	3.16E-10
TRIM72	2.606438	-0.52582	1.41E-10	3.17E-10
AKR1C2	-1.15436	3.319262	1.44E-10	3.24E-10
NBL1	1.187219	5.328892	1.45E-10	3.27E-10
MN1	1.011153	3.452546	1.46E-10	3.28E-10
ODF3L1	1.02376	-0.77216	1.50E-10	3.37E-10
PPP1R14	2.102895	2.726256	1.51E-10	3.40E-10
PPP2R2C	2.226975	1.610911	1.54E-10	3.46E-10
CDH18	-2.1378	-2.16476	1.55E-10	3.47E-10
BAIAP2L	1.195573	4.641938	1.56E-10	3.49E-10
SMIM3	1.163576	6.121649	1.56E-10	3.50E-10
PLCZ1	-1.60635	-2.81678	1.57E-10	3.52E-10
SLC25A2	1.62036	-3.34332	1.58E-10	3.54E-10
AGR2	-2.21319	1.947197	1.60E-10	3.59E-10
BTNL8	1.097527	-1.2521	1.66E-10	3.72E-10
P11-20I23	1.209364	-2.69358	1.67E-10	3.73E-10
SYN1	1.192687	1.218365	1.67E-10	3.74E-10
CHRDL1	-1.80032	3.468845	1.70E-10	3.80E-10

RPE	0.62537192	1.00E-59
PHF2	0.50755927	1.44E-36
TXNRD3	0.53214433	1.15E-40
MCMBP	0.64342658	3.35E-64
TAF13	0.53759942	1.27E-41
FAM118B	0.54664513	3.02E-43
ZNF181	0.56442342	1.38E-46
SGTB	0.55049256	5.94E-44
MYO1C	0.57919467	1.61E-49
NKIRAS1	0.6036406	1.03E-54
SLC22A12	0.55311301	1.94E-44
ADH5	0.78995362	6.04E-116
SLC22A6	0.50401773	5.26E-36
ZNF71	0.51053799	4.77E-37
VPS13A	0.52905416	3.92E-40
MBP	0.55811032	2.23E-45
MRPL42	0.59790623	1.86E-53
MAK16	0.50947713	7.08E-37
AVPR1B	0.62646567	5.46E-60
PRIM1	0.55100114	4.79E-44
MARCHF5	0.61802953	5.46E-58
SULT1C4	0.58450464	1.31E-50
ZBTB14	0.63318985	1.25E-61
CD2AP	0.63793664	8.26E-63
ZNF649	0.53041883	2.28E-40
HIBCH	0.72239729	6.29E-88
SOWAHC	0.66889443	4.71E-71
ZNF770	0.62210983	5.99E-59
MIER1	0.53859708	8.45E-42
MAN1A2	0.64889318	1.29E-65
TFDP1	0.61515408	2.54E-57
HSD17B11	0.69858539	5.91E-80
ZXDA	0.64736752	3.21E-65
DDX42	0.57718743	4.12E-49
HELZ	0.53078772	1.97E-40
SDAD1	0.58764587	2.89E-51
ZKSCAN8	0.53128566	1.62E-40
HYLS1	0.53305232	7.97E-41
GET3	0.59157339	4.28E-52
ASPH	0.52083746	9.68E-39
SPRED2	0.55725554	3.24E-45
WWP2	0.56111607	5.98E-46
GFPT1	0.62433669	1.77E-59
UVRAG	0.65800709	4.83E-68
ITSN2	0.63399112	7.95E-62
TCAF1	0.5139945	1.31E-37
ZXDB	0.60481475	5.63E-55
ZNF480	0.50873682	9.31E-37
SH3BGRL2	0.67470523	1.03E-72
YTHDF2	0.70574144	2.88E-82
TMA16	0.51887953	2.05E-38
ATL1	0.54754988	2.06E-43
GPN1	0.6260707	6.80E-60
ZNF28	0.58470485	1.19E-50
ZNF627	0.55020293	6.72E-44
CTNND1	0.69338871	2.56E-78
TLK1	0.54066775	3.62E-42
LRBA	0.60808855	1.04E-55
COPS8	0.66772344	1.01E-70
KLHL9	0.67777337	1.32E-73
NCOA6	0.52406102	2.78E-39
C6orf89	0.6716388	7.83E-72
CALM1	0.61611407	1.52E-57
TTC37	0.63515757	4.09E-62
TUSC1	0.51668036	4.74E-38
SLC9A6	0.64697807	4.06E-65
FAN1	0.50208898	1.06E-35
IPO9	0.69034614	2.24E-77
GLMP	0.53184106	1.29E-40
TOGARAM1	0.56214532	3.80E-46
ECI2	0.6539594	5.91E-67
UNC13B	0.62829755	1.97E-60
CTR9	0.69718303	1.65E-79
ZNF652	0.50275722	8.32E-36
GPATCH3	0.62923668	1.17E-60
CDC42BPB	0.54829678	1.51E-43
ZNF830	0.62275239	4.22E-59
CNOT7	0.5545188	1.06E-44
MTOR	0.55572972	6.29E-45
PNP	0.6455554	9.48E-65
GK	0.54430973	8.01E-43
SFT2D1	0.50272405	8.42E-36
CHAMP1	0.72215207	7.68E-88
INPP5F	0.58855963	1.86E-51
HMGN2	0.61486438	2.96E-57
UBE2J1	0.57268583	3.30E-48
OPA1	0.57418349	1.66E-48
KTI12	0.55748194	2.94E-45
SELENOT	0.54369553	1.03E-42
OSTC	0.52150869	7.47E-39
LTN1	0.52330278	3.73E-39
RUNDC1	0.71305478	1.06E-84
TYW1	0.5194158	1.67E-38
DCAF12	0.67682016	2.51E-73
PRMT6	0.61084735	2.47E-56
CIPC	0.54815743	1.60E-43
CAPZA2	0.57220879	4.10E-48
TOP1	0.59745278	2.34E-53
BHLHB9	0.61866265	3.88E-58
MAP3K3	0.50930466	7.55E-37
SREBF2	0.50735371	1.55E-36
POU3F3	0.57268179	3.30E-48
DZIP3	0.57120028	6.50E-48
DCLRE1A	0.63043176	5.96E-61
ATG9A	0.55745306	2.98E-45
MAGEE1	0.55642351	4.65E-45
MFAP3L	0.61057518	2.85E-56
NAGA	0.51884928	2.08E-38
SMG5	0.59954566	8.19E-54
KIFBP	0.66064181	9.26E-69
ARMCX6	0.53832287	9.45E-42
PJA2	0.63576174	2.89E-62
SGMS1	0.59344472	1.71E-52
COX20	0.58457171	1.27E-50
CHML	0.53596007	2.47E-41
TATDN3	0.64246518	5.89E-64
SAMD5	0.59162041	4.19E-52
EEF1AKMT2	0.50088532	1.64E-35
DDO	0.63048317	5.79E-61
SYS1	0.58461194	1.24E-50
TRAF3IP1	0.64766798	2.68E-65
CHIC1	0.6093384	5.43E-56
GIGYF2	0.71527694	1.86E-85
PHACTR4	0.56837121	2.35E-47
ASAH2B	0.65087419	3.88E-66
MACO1	0.7445155	4.05E-96
DAXX	0.51530216	8.00E-38
BMPR2	0.55158105	3.74E-44
TCEA3	0.50843892	1.04E-36
HSD17B8	0.54120507	2.90E-42
BRD2	0.54297788	1.39E-42
PBX2	0.5038849	5.52E-36
RNF5	0.71556377	1.48E-85
AGPAT1	0.69901325	4.32E-80
FKBPL	0.50299321	7.64E-36
SDHD	0.68348293	2.70E-75
NEU1	0.53235898	1.05E-40
HSPA1L	0.63584828	2.75E-62
VARS1	0.53017317	2.51E-40
MBD5	0.60257158	1.77E-54
GPANK1	0.62764854	2.83E-60
C6orf47	0.6741544	1.49E-72
BAG6	0.63535503	3.65E-62
PRRC2A	0.51580342	6.62E-38
MRPS18B	0.72839624	4.54E-90
PPP1R10	0.5323521	1.06E-40
ABCF1	0.67819189	9.96E-74
GNL1	0.50730241	1.58E-36
HLA-E	0.57345911	2.31E-48
TRIM39	0.53341544	6.89E-41
ZNF468	0.51879545	2.12E-38
TRIM10	0.62585941	7.64E-60
PPP1R11	0.63712277	1.32E-62
INSYN2B	0.50874791	9.27E-37
DCTN1	0.72568294	4.30E-89
ZNF155	0.52631783	1.15E-39
FBXO48	0.65446356	4.33E-67
ZNF425	0.52860329	4.68E-40
TRIM13	0.65694499	9.35E-68
SLC35B4	0.64974779	7.68E-66
TRIQK	0.5332587	7.34E-41
ZBTB10	0.52585558	1.38E-39
LGR4	0.60163633	2.85E-54
TMEM170B	0.61815498	5.10E-58
SNX2	0.68592432	4.99E-76
IPO7	0.50650597	2.12E-36
LIN52	0.57674804	5.05E-49
ETFRF1	0.55484562	9.21E-45
DENND1C	0.55697646	3.66E-45
CRYZL1	0.60342634	1.15E-54
C5orf51	0.57248168	3.62E-48
CYS1	0.5576831	2.69E-45
PLPP6	0.56210233	3.87E-46
JPT2	0.53013999	2.55E-40
RAB12	0.58520628	9.34E-51
HACD2	0.54860079	1.32E-43
LNP1	0.52683593	9.40E-40
SACM1L	0.52164577	7.09E-39
TSN	0.70538171	3.78E-82
NUP62	0.52243288	5.23E-39
SFT2D2	0.67535468	6.68E-73
SCAF8	0.58927633	1.31E-51
SUPT4H1	0.60178056	2.65E-54
NRAS	0.68189588	8.04E-75
CHUK	0.66154276	5.24E-69
COG8	0.61957156	2.38E-58
ARHGAP19	0.57653282	5.58E-49
SYNJ2BP	0.56054511	7.69E-46
RBMXL1	0.65257629	1.38E-66
VDAC1	0.54937898	9.53E-44

NMNAT2	1.628334	2.34205	1.76E-10	3.93E-10
WFIKKN1	1.295313	1.841528	1.81E-10	4.04E-10
SORCS2	-1.32359	4.388674	1.87E-10	4.18E-10
PRPH	1.942171	0.534133	1.98E-10	4.42E-10
C007040.1	1.79229	-2.07786	2.00E-10	4.45E-10
TMEM255	2.164821	4.146165	2.00E-10	4.46E-10
MCOLN3	-1.40055	1.915414	2.02E-10	4.50E-10
SLCO5A1	1.235037	-0.48751	2.04E-10	4.55E-10
CCDC68	1.032759	3.115888	2.08E-10	4.63E-10
P1-130H16	1.109774	-2.55761	2.09E-10	4.65E-10
C14orf180	2.47863	1.575845	2.17E-10	4.83E-10
EGR3	-1.13051	4.007521	2.19E-10	4.88E-10
B3GALT1	1.308646	-1.18246	2.21E-10	4.90E-10
CYP26A1	2.006361	-1.79073	2.21E-10	4.90E-10
ROBO2	-1.01958	1.936433	2.26E-10	5.02E-10
THBS2	1.43747	7.057378	2.28E-10	5.06E-10
COL6A5	-1.65398	-2.95356	2.37E-10	5.25E-10
PCDHGB7	1.293607	4.149277	2.37E-10	5.26E-10
NPIPA5	1.295354	0.223267	2.47E-10	5.46E-10
MZB1	2.14544	3.386342	2.57E-10	5.68E-10
AGXT2	-1.19766	6.077761	2.66E-10	5.88E-10
CCDC177	2.34548	-3.1579	2.73E-10	6.04E-10
PTPRD	-1.14019	4.912151	2.79E-10	6.17E-10
SRPK3	1.364697	-0.30342	2.86E-10	6.31E-10
HTRA3	1.096793	3.321404	2.86E-10	6.32E-10
PTCHD4	1.121993	2.355535	2.86E-10	6.32E-10
ACHE	1.369997	2.452517	2.90E-10	6.40E-10
DPF1	1.166286	-1.66507	2.93E-10	6.47E-10
TMC2	1.311132	-2.98528	3.05E-10	6.73E-10
CELF6	1.516392	-3.40069	3.07E-10	6.76E-10
KASH5	2.141922	-3.04541	3.09E-10	6.80E-10
NPM2	-1.00351	0.981638	3.12E-10	6.86E-10
MSLNL	3.484754	-2.34892	3.16E-10	6.96E-10
F8	1.053963	6.559165	3.24E-10	7.12E-10
JPH3	1.659271	-1.16988	3.42E-10	7.51E-10
TSPAN1	-1.113	7.52152	3.46E-10	7.60E-10
S100A9	1.084405	4.416557	3.46E-10	7.61E-10
RTN4RL2	1.055049	2.231837	3.49E-10	7.66E-10
ASIP	1.553448	-1.23864	3.57E-10	7.84E-10
NKX2-3	3.17302	-1.82876	3.62E-10	7.93E-10
XKR3	2.015579	-3.05113	3.65E-10	8.01E-10
CDH7	4.050197	0.372673	3.66E-10	8.02E-10
GATA4	3.999147	-0.90563	3.79E-10	8.30E-10
VWC2L	1.367355	-2.23011	3.79E-10	8.30E-10
SCX	1.445717	-0.52588	4.01E-10	8.77E-10
STEAP3	1.409937	5.453105	4.04E-10	8.85E-10
CCDC150	1.144954	2.769258	4.10E-10	8.98E-10
SLC27A2	-1.05998	5.602641	4.21E-10	9.21E-10
SPOCD1	1.40625	0.314619	4.23E-10	9.26E-10
CRHR2	1.446277	-0.03421	4.40E-10	9.62E-10
H2BC4	1.080177	2.15434	4.42E-10	9.67E-10
UGT2B17	2.009979	-1.25042	4.50E-10	9.83E-10
LYPD8	-1.42472	0.284774	4.75E-10	1.04E-09
CD207	1.499997	0.61121	4.85E-10	1.06E-09
TRIM55	1.039719	4.434625	4.89E-10	1.07E-09
ADORA1	-1.10927	2.953176	5.04E-10	1.10E-09
DGKI	1.640369	4.113649	5.06E-10	1.10E-09
GSDMC	2.472075	-1.2737	5.10E-10	1.11E-09
TUBB3	1.610453	0.1149	5.18E-10	1.13E-09
COL19A1	-1.23475	0.229108	5.25E-10	1.14E-09
INPP5J	-1.72871	4.54445	5.30E-10	1.15E-09
CYP4A11	-1.62102	6.652519	5.48E-10	1.19E-09
PCDHB16	1.048231	3.927294	5.51E-10	1.20E-09
GPR37	1.726873	2.242341	5.70E-10	1.24E-09
CD177	2.824927	1.93958	5.76E-10	1.25E-09
HOXB13	3.347539	0.198349	5.90E-10	1.28E-09
FCGR3B	1.480523	1.492259	6.09E-10	1.32E-09
AKR1C1	-1.03538	5.314513	6.22E-10	1.35E-09
SLAMF9	2.452876	-2.58119	6.27E-10	1.36E-09
ELAVL3	1.516881	-0.77496	6.37E-10	1.38E-09
BRINP2	2.570357	-1.06058	6.86E-10	1.48E-09
LTB	1.100516	3.198762	6.91E-10	1.49E-09
LY6K	-1.37453	0.0792	7.12E-10	1.54E-09
S100A12	1.315738	-0.2717	7.12E-10	1.54E-09
LRRC3B	1.857205	-1.60535	7.14E-10	1.54E-09
WNT2B	1.344151	3.460667	7.19E-10	1.55E-09
GPR45	1.454766	-2.73248	7.25E-10	1.56E-09
VAX2	1.245554	-1.35404	7.43E-10	1.60E-09
PON3	-1.18625	0.572209	7.64E-10	1.64E-09
XKR9	1.120978	0.587439	7.67E-10	1.65E-09
MYB	1.36267	-0.08774	7.83E-10	1.68E-09
DNAH3	1.251347	-0.96553	8.31E-10	1.78E-09
KRT20	4.107982	0.435052	8.39E-10	1.80E-09
H2AC7	1.712188	-1.34161	8.40E-10	1.80E-09
DHDH	-1.19745	2.474215	8.42E-10	1.81E-09
FAP	1.213712	2.700261	8.44E-10	1.81E-09
ALPI	2.706513	3.654025	9.66E-10	2.07E-09
CNTN3	-1.69844	1.90677	9.72E-10	2.08E-09
SALL3	-2.42847	0.496166	1.01E-09	2.15E-09
P11-106M3	1.162345	-1.11234	1.02E-09	2.17E-09
CYP3A7	-1.01829	1.515336	1.03E-09	2.21E-09
TRPV3	1.26019	-0.05872	1.07E-09	2.28E-09
ESYT3	-1.01172	0.837613	1.07E-09	2.29E-09
PNMA3	1.442239	3.022601	1.11E-09	2.37E-09
GUCY2D	1.509421	-0.82231	1.12E-09	2.38E-09
H2BC11	1.165059	-0.53862	1.15E-09	2.44E-09
NMRK2	4.715939	3.567486	1.19E-09	2.54E-09
C4BPA	3.11482	1.427387	1.20E-09	2.56E-09
DRC1	-1.15534	-2.12206	1.28E-09	2.72E-09
LECT2	1.951001	-1.25332	1.30E-09	2.76E-09
CD79A	1.798059	2.644597	1.32E-09	2.81E-09
CLEC18C	1.739373	3.410444	1.42E-09	3.00E-09
CST1	2.671998	-2.39451	1.45E-09	3.06E-09
BRSK2	1.248983	0.013105	1.49E-09	3.15E-09
MCIDAS	-1.72573	-2.8087	1.50E-09	3.17E-09
YBX2	1.361558	1.647868	1.54E-09	3.26E-09
PCDH10	1.67049	4.648963	1.56E-09	3.29E-09
ZG16B	1.134924	-0.58758	1.76E-09	3.70E-09
GRIA1	1.993918	-0.02194	1.77E-09	3.74E-09
STK32A	-1.32519	2.467335	1.82E-09	3.82E-09
PLD5	-1.37194	-0.4762	1.96E-09	4.13E-09
H2AC20	1.789163	-0.61282	2.05E-09	4.30E-09
NFRSF13	1.190571	0.262956	2.09E-09	4.38E-09
OR13A1	1.77829	-2.65477	2.21E-09	4.63E-09
RASGEF1	1.051385	3.18482	2.22E-09	4.65E-09
GNB3	1.432376	-0.38288	2.24E-09	4.70E-09
BMPER	1.470977	1.145495	2.28E-09	4.77E-09
MAST1	1.373106	-0.71598	2.30E-09	4.82E-09
SLC25A27	1.073031	2.816856	2.38E-09	4.97E-09
SF12-TNF	1.021643	-2.97567	2.61E-09	5.44E-09
SLC38A5	1.928214	3.856259	2.71E-09	5.64E-09
GLRB	-1.22075	2.189309	2.79E-09	5.81E-09
GCGR	-2.28219	2.396542	2.83E-09	5.89E-09
XCR1	1.33028	1.424375	2.89E-09	6.02E-09
TRIM15	1.254786	3.47616	3.07E-09	6.39E-09
SMIM22	-1.61499	1.301975	3.21E-09	6.66E-09
ATP6V1FN	1.260449	2.541157	3.22E-09	6.68E-09
P3-370M2	1.064014	-2.47541	3.25E-09	6.74E-09
SLC25A41	1.05761	-1.88128	3.27E-09	6.78E-09
ISM2	-1.48372	0.282048	3.30E-09	6.84E-09
IL13RA2	-1.12922	1.338922	3.35E-09	6.94E-09
LVRN	1.181132	-1.33972	3.35E-09	6.95E-09
TNNI3	3.184554	-1.56866	3.49E-09	7.24E-09
S100A5	-1.20523	-1.34296	3.71E-09	7.67E-09
GJB2	1.323063	4.924272	3.82E-09	7.90E-09
KLK13	2.215924	-2.55158	3.83E-09	7.92E-09
CCL22	1.115325	0.916748	3.85E-09	7.95E-09
ZNF114	1.953318	1.939379	3.89E-09	8.04E-09
C1R	1.115217	8.294125	3.94E-09	8.14E-09
TNR	1.961205	-0.06456	3.95E-09	8.16E-09
NSG1	-1.23463	1.155856	3.99E-09	8.23E-09
RYR2	1.586129	1.990909	4.08E-09	8.42E-09
MIXL1	1.612099	-2.02624	4.14E-09	8.53E-09
SPINK7	2.4442	-0.84058	4.28E-09	8.82E-09
CYP1B1	-1.06696	6.188933	4.31E-09	8.87E-09
AGR3	-2.02389	-0.92936	4.35E-09	8.95E-09
AKR1C8P	1.639159	-1.9965	4.38E-09	9.01E-09
ANXA8L1	1.870407	-1.67085	4.61E-09	9.48E-09
FGA	3.262993	6.759828	5.06E-09	1.04E-08
LUM	-1.26897	6.662138	5.10E-09	1.05E-08
CA1	2.439926	0.403405	5.13E-09	1.05E-08
RASGEF1	1.715893	-0.29887	5.29E-09	1.08E-08
SLC38A4	-1.48106	2.784781	5.40E-09	1.11E-08
SLC17A3	1.65631	7.768081	5.51E-09	1.13E-08
FGF17	1.346992	-1.8641	5.52E-09	1.13E-08
FGF14	1.275954	2.1647	5.58E-09	1.14E-08
PRRX2	1.220134	0.376472	5.60E-09	1.15E-08
LEF1	1.161244	3.928122	5.84E-09	1.19E-08
CIBAR2	2.015909	-2.39886	6.09E-09	1.24E-08
SLIT2	-1.12751	4.46875	6.33E-09	1.29E-08
ADAM20	1.414751	-2.49582	6.35E-09	1.29E-08
CELF5	1.88826	0.477499	6.35E-09	1.30E-08
NLRP4	1.579017	-3.45657	6.44E-09	1.31E-08
TXNDC2	1.149906	-2.68805	6.45E-09	1.31E-08
PLA2G4F	-2.23667	3.512513	6.46E-09	1.32E-08
SPIB	1.328758	0.166825	6.49E-09	1.32E-08
LRRN1	-1.37666	0.333574	6.54E-09	1.33E-08
CLGN	1.781296	1.569728	6.55E-09	1.33E-08
SLC17A1	-1.14129	5.238011	6.70E-09	1.36E-08
GCKR	2.214279	-0.86297	6.93E-09	1.41E-08
SLC26A7	-1.85445	5.184607	7.11E-09	1.44E-08
PDZRN4	-1.32374	0.385044	7.34E-09	1.49E-08
KLHL35	1.324721	-0.27713	7.70E-09	1.56E-08
TRIM31	1.027765	-1.51699	8.01E-09	1.62E-08
TB-102L5	-1.01227	-2.37473	8.07E-09	1.64E-08
CCDC27	1.345814	-2.24385	8.41E-09	1.70E-08
BTNL3	1.388396	-2.56119	8.49E-09	1.72E-08
VIT	-1.49712	-1.85975	8.82E-09	1.78E-08
CDC20B	-1.24011	-0.68539	9.16E-09	1.85E-08
HSF5	1.10394	-2.8367	9.26E-09	1.87E-08
SLC10A4	1.241731	-2.52922	9.50E-09	1.92E-08
TMEM10(	1.360943	2.60821	9.75E-09	1.97E-08
PCDHB2	1.161409	2.288494	9.75E-09	1.97E-08
GGT2	1.552687	-0.09067	1.01E-08	2.03E-08
IGLON5	2.513829	2.663481	1.03E-08	2.07E-08
SLC35G5	1.233934	-3.10458	1.11E-08	2.23E-08
SYT8	2.828519	0.703769	1.16E-08	2.32E-08
NRN1	1.139121	4.443365	1.21E-08	2.44E-08
WDR38	1.098506	-2.13379	1.22E-08	2.45E-08
PADI3	3.264823	1.656495	1.22E-08	2.45E-08

TMX2	0.60921479	5.79E-56
LEPROT	0.6171804	8.61E-58
PPP1CB	0.57701147	4.47E-49
SLC35F6	0.57381084	1.97E-48
CLIC1	0.51232191	2.45E-37
ZNF134	0.51937401	1.70E-38
ZNF845	0.51576959	6.70E-38
MDP1	0.50451656	4.39E-36
ITGA1	0.51128536	3.61E-37
SMIM7	0.55899117	1.52E-45
SMIM30	0.50506534	3.59E-36
AS3MT	0.54083847	3.37E-42
B3GALT9	0.55614075	5.26E-45
ZBED1	0.58172141	4.90E-50
ZBED1	0.58172141	4.90E-50
HNRNPUL2	0.53165871	1.39E-40
ZSWIM7	0.56703876	4.28E-47
ARHGEF28	0.55074657	5.33E-44
UBXN2B	0.63485094	4.87E-62
PEX26	0.58939064	1.24E-51
FASTKD5	0.68870063	7.15E-77
HOMEZ	0.60353073	1.09E-54
DDX3X	0.64281354	4.80E-64
ZNF407	0.55424786	1.19E-44
TMEM242	0.57444575	1.47E-48
TMEM167B	0.6583635	3.86E-68
NBPF1	0.54142788	2.64E-42
UMAD1	0.56776301	3.09E-47
VAMP2	0.55325535	1.83E-44
PPP3R1	0.59484677	8.55E-53
PPP2R2A	0.592829	2.31E-52
FAM185A	0.53480154	3.94E-41
VPS52	0.62600237	7.06E-60
ATXN1L	0.69031161	2.29E-77
SMIM13	0.51651252	5.06E-38
NOL7	0.63392251	8.27E-62
TMEM185B	0.59835864	1.49E-53
WDR46	0.51751608	3.45E-38
KLLN	0.58367257	1.94E-50
PARG	0.58520506	9.34E-51
DHFR	0.53083847	1.93E-40
ACBD6	0.5458987	4.13E-43
PET117	0.51136232	3.51E-37
TRIM26	0.60453809	6.49E-55
HNRNPUL2-BSC	0.50090171	1.63E-35
BRD3OS	0.59860215	1.32E-53
ZBTB22	0.5944053	1.06E-52
B3GNT9	0.53906744	6.97E-42
C2orf74	0.52206436	6.03E-39
TMEM250	0.50315717	7.19E-36
TXNDC5	0.5290889	3.87E-40
RNF103	0.70834124	4.01E-83
RBM14	0.52146254	7.61E-39
ADSL	0.66502918	5.70E-70
PCDHGC3	0.57253971	3.52E-48
ACAD11	0.65718566	8.05E-68
AQP1	0.54768696	1.95E-43
PNMA2	0.52273014	4.66E-39
RDH14	0.73330261	7.29E-92
UGT1A9	0.52804912	5.83E-40
CRCP	0.53777131	1.18E-41
PDXP	0.50012751	2.15E-35
PI4KA	0.54178467	2.28E-42
AMACR	0.54620869	3.62E-43
ARFGAP3	0.67818369	1.00E-73
C22orf39	0.64585376	7.94E-65
ARPIN	0.60655874	2.30E-55
GNG10	0.5721205	4.27E-48
MICAL3	0.54676351	2.87E-43
STON1	0.50842569	1.04E-36
TTC4	0.60952906	4.92E-56
TMEM35B	0.63613664	2.33E-62
NFS1	0.52401866	2.83E-39
TMEM199	0.66662192	2.05E-70
RBM12	0.63481198	4.98E-62
C4A	0.50074914	1.72E-35
ZCCHC3	0.58350025	2.11E-50
MARS2	0.59891734	1.12E-53
USP51	0.55750069	2.91E-45
BCKDHA	0.60316977	1.31E-54
TMEM150C	0.58964591	1.10E-51
ZNF324B	0.50841926	1.05E-36
ARPIN-AP3S2	0.52019857	1.24E-38
SELENOP	0.59524153	7.03E-53
CENPS-CORT	0.5055149	3.05E-36
C1orf210	0.59866117	1.28E-53
ALG11	0.58259441	3.24E-50
ATXN7L3B	0.68590209	5.06E-76
UTP14C	0.7215151	1.29E-87
ZNF260	0.59973875	7.43E-54
AP5B1	0.54778052	1.87E-43
PABPC4L	0.50525963	3.34E-36
FPGT	0.70520142	4.33E-82
BRK1	0.50289275	7.92E-36
CHMP1B	0.64886729	1.31E-65
EID1	0.69137072	1.08E-77
POLR2M	0.51751751	3.45E-38
TRIL	0.53344106	6.82E-41
CTSO	0.64588576	7.79E-65
ZNF225	0.50050183	1.88E-35
ZNF253	0.52771454	6.65E-40
SLC5A8	0.57532097	9.79E-49
DENND11	0.55232276	2.72E-44
LSM14A	0.69997235	2.13E-80
GATC	0.53358042	6.45E-41
MGAM	0.5366154	1.89E-41
GALNT4	0.54776073	1.89E-43
CUX1	0.537368	1.39E-41
P2RX5-TAX1BP	0.55031957	6.40E-44
CHURC1	0.57993154	1.14E-49
RP11-903H12.5	0.57491114	1.18E-48
THTPA	0.64102738	1.37E-63
MRPL46	0.58133494	5.89E-50
RP11-303E16.8	0.51037646	5.07E-37
CCPG1	0.55510668	8.23E-45
PECAM1	0.57557845	8.69E-49
GTF2I	0.58157569	5.25E-50
SRSF8	0.73179639	2.62E-91
DYNLL2	0.66737672	1.26E-70
OTUD7B	0.68843073	8.64E-77
RBM8A	0.74578956	1.29E-96
RNF115	0.62088294	1.17E-58
SEC22B	0.64573751	8.51E-65
TXNIP	0.57104783	6.97E-48
NCOA4	0.7036298	1.41E-81
LIX1L	0.55276388	2.25E-44
NUDT3	0.57494825	1.16E-48
ZNF595	0.53003222	2.66E-40
DCP1A	0.51490032	9.31E-38
CWC25	0.58499656	1.03E-50
ZNHIT3	0.55074972	5.33E-44
CYFIP1	0.61170246	1.58E-56
USP27X	0.61221066	1.21E-56
TAF9	0.50872112	9.37E-37
SOCS7	0.58952826	1.16E-51
ZNF658	0.65731394	7.43E-68
PPP4R3B	0.71730678	3.74E-86
SYNRG	0.60269706	1.66E-54
ZNF2	0.59997536	6.59E-54
PRAG1	0.51352151	1.56E-37
AATF	0.51521063	8.28E-38
PIP4K2B	0.61325377	6.96E-57
PIGW	0.53698925	1.63E-41
ZNF670	0.5663301	5.89E-47
CISD3	0.58081987	7.51E-50
DDX52	0.6065216	2.34E-55
GGNBP2	0.70255917	3.14E-81
MRPL45	0.66313289	1.91E-69

GAP43	1.463436	-0.9819	1.22E-08	2.46E-08
CELF4	1.057047	0.597548	1.23E-08	2.48E-08
DRD1	-1.02758	-0.43856	1.24E-08	2.49E-08
PLA2G3	-1.98776	-2.86279	1.25E-08	2.50E-08
CHRM4	1.31813	-2.00302	1.25E-08	2.50E-08
GRIN2A	1.629112	3.93107	1.26E-08	2.52E-08
KCNV1	1.692464	0.596939	1.26E-08	2.52E-08
WNT6	1.172722	-0.27727	1.29E-08	2.59E-08
SMCO3	-1.14546	0.776219	1.30E-08	2.60E-08
SFRP2	1.95189	5.580415	1.30E-08	2.60E-08
TRIM54	1.975467	2.452629	1.32E-08	2.64E-08
C11orf86	2.407514	1.193848	1.36E-08	2.71E-08
PCDHGA5	1.048645	1.177367	1.36E-08	2.71E-08
H4C12	1.344301	-3.06675	1.37E-08	2.74E-08
DOCK3	-1.08202	0.611319	1.40E-08	2.79E-08
KRT6B	3.216896	-2.64302	1.43E-08	2.86E-08
TNNT1	2.46121	1.279001	1.45E-08	2.90E-08
SH3RF2	1.323103	2.682502	1.49E-08	2.98E-08
COL17A1	2.003515	1.982226	1.51E-08	3.01E-08
PYDC1	2.396587	-2.55499	1.53E-08	3.06E-08
STXBP5L	3.185467	-0.1505	1.62E-08	3.22E-08
AGT	1.101747	6.799325	1.88E-08	3.73E-08
CSDC2	-1.34575	3.830102	1.94E-08	3.86E-08
PCDHB8	1.088681	0.865251	1.98E-08	3.93E-08
CST6	-1.31709	-0.56552	2.01E-08	3.99E-08
WNT9A	1.074923	1.721031	2.07E-08	4.10E-08
ACTN2	1.488596	1.222848	2.08E-08	4.12E-08
ATP2B3	-1.73508	-0.8331	2.23E-08	4.40E-08
TRDN	-1.57342	-2.1545	2.27E-08	4.48E-08
SIGLEC6	1.086537	-1.38305	2.28E-08	4.50E-08
DMBX1	2.939447	-2.1478	2.44E-08	4.80E-08
SERTM2	3.498158	2.620905	2.49E-08	4.89E-08
SPATA12	1.128204	-2.37671	2.51E-08	4.95E-08
MORN5	-1.00566	-1.24022	2.58E-08	5.07E-08
KCNIP1	-1.38621	0.70562	2.58E-08	5.08E-08
KISS1	1.878547	-1.12471	2.66E-08	5.22E-08
SLC18A1	1.150416	-2.87629	2.68E-08	5.27E-08
CCR3	1.104071	-2.40212	2.68E-08	5.27E-08
PLA2G2C	1.333975	-2.70282	2.68E-08	5.27E-08
CRIP3	1.082704	0.828008	2.68E-08	5.27E-08
CAMP	1.511255	-2.52277	2.71E-08	5.32E-08
FLG2	1.355948	-2.68074	2.75E-08	5.39E-08
HAS2	-1.01429	0.918434	2.79E-08	5.48E-08
GDPD4	1.095443	-1.74786	2.82E-08	5.53E-08
SLC25A48	-1.06876	2.221821	2.97E-08	5.82E-08
MSLN	2.51116	5.263907	2.97E-08	5.83E-08
RFLNA	1.72686	2.011423	3.18E-08	6.22E-08
PDGFRL	1.265152	2.085609	3.24E-08	6.33E-08
EDAR	-1.16317	0.345963	3.29E-08	6.43E-08
LRRN4CL	1.629927	1.697872	3.34E-08	6.52E-08
MB	1.254369	-0.84109	3.37E-08	6.57E-08
FOXG1	2.184737	-2.2408	3.40E-08	6.62E-08
MEOX2	1.384637	1.610831	3.41E-08	6.64E-08
UGT1A7	1.980013	-0.52327	3.41E-08	6.66E-08
DES	-1.47303	4.447261	3.43E-08	6.68E-08
TRIM63	2.796424	2.612518	3.43E-08	6.69E-08
NAPSA	-1.13223	4.948168	3.53E-08	6.87E-08
C9orf50	1.086029	-0.45273	3.59E-08	6.98E-08
SLC16A9	-1.04959	6.658396	3.60E-08	7.01E-08
SPHKAP	-1.72023	-3.15333	3.69E-08	7.17E-08
NLRP11	1.059115	0.426198	3.76E-08	7.30E-08
MMP12	2.702842	1.089728	3.82E-08	7.42E-08
PLA2G1B	1.161443	-1.24493	3.95E-08	7.65E-08
KRT17	2.222019	4.182241	4.15E-08	8.04E-08
KRT15	1.916763	-0.13542	4.20E-08	8.14E-08
PGLYRP1	1.2335	-2.73417	4.21E-08	8.15E-08
TNFRSF17	1.584368	-0.46614	4.32E-08	8.35E-08
GALR1	1.487799	0.636946	4.36E-08	8.44E-08
TMEM40	-1.23165	-2.81166	4.54E-08	8.77E-08
GLRA3	-1.5223	-2.83063	4.65E-08	8.98E-08
ADAM7	3.122908	0.471793	4.66E-08	8.99E-08
C4orf51	1.536136	-2.51946	4.74E-08	9.15E-08
UNC13A	1.207365	0.709162	4.90E-08	9.43E-08
PTGS2	-1.09839	3.941158	4.98E-08	9.59E-08
CNTFR	-1.31086	0.264206	5.34E-08	1.03E-07
STOML3	1.678967	-2.52083	5.37E-08	1.03E-07
PIGR	-1.44106	7.910493	5.55E-08	1.07E-07
SLPI	-1.62828	5.260246	5.55E-08	1.07E-07
SHISAL1	1.541274	1.761905	5.59E-08	1.07E-07
VWA5B1	-1.69496	0.83654	5.64E-08	1.08E-07
TCHH	1.658297	1.096954	6.01E-08	1.15E-07
ELAVL2	1.419245	0.849785	6.04E-08	1.16E-07
KCND2	1.596318	-0.31784	6.05E-08	1.16E-07
C6	1.558229	3.627585	6.17E-08	1.18E-07
GBP7	1.436019	-0.84041	6.17E-08	1.18E-07
ITGA2B	1.340953	1.116692	6.29E-08	1.20E-07
ZNF365	1.397085	1.519128	6.30E-08	1.21E-07
PRELP	-1.00188	5.71353	6.31E-08	1.21E-07
CDCP2	1.026371	-3.21648	6.39E-08	1.22E-07
SLC4A10	1.326878	-0.98741	6.65E-08	1.27E-07
CBS	-1.14014	-2.14932	7.00E-08	1.34E-07
SERPINA7	2.350307	0.471449	7.04E-08	1.34E-07
MPRSS11	4.125089	1.116852	7.06E-08	1.35E-07
GPRC5A	-1.25384	4.901372	7.40E-08	1.41E-07
CPE	1.003608	8.145741	7.76E-08	1.48E-07
NEB	1.590116	4.186229	8.08E-08	1.53E-07
SNCG	1.011704	4.552062	8.11E-08	1.54E-07
ERICH3	1.253557	-0.8058	8.70E-08	1.65E-07
PADI4	1.127684	-1.31055	8.77E-08	1.66E-07
AKR1B10	2.724689	3.674319	8.91E-08	1.69E-07
HSPB6	-1.01328	3.73448	8.95E-08	1.70E-07
CXCR1	1.103326	0.156451	9.04E-08	1.71E-07
VWA3A	1.471935	-0.55811	9.09E-08	1.72E-07
NKRD40C	1.747028	-2.66445	9.55E-08	1.81E-07
PCDHA9	1.016548	-2.05937	9.98E-08	1.89E-07
ZIC5	2.817977	-2.17605	1.04E-07	1.96E-07
SLC8A3	1.652441	-0.37836	1.05E-07	1.98E-07
PROKR1	1.046401	-3.14855	1.06E-07	2.00E-07
GALNT5	1.823264	2.215709	1.09E-07	2.06E-07
UGT3A1	-1.30969	5.620736	1.12E-07	2.10E-07
PCDH20	-1.31	-3.25612	1.12E-07	2.11E-07
NPIPB6	1.209357	-2.83628	1.12E-07	2.11E-07
SERPINF1	1.081855	5.827385	1.17E-07	2.20E-07
AKR1C4	1.675625	-0.43052	1.18E-07	2.22E-07
ATP6V0A4	-2.00127	6.204272	1.20E-07	2.26E-07
POU2AF1	1.47272	2.361409	1.20E-07	2.27E-07
CBSL	-1.3518	-3.19261	1.22E-07	2.29E-07
EPHA6	1.390367	1.049431	1.26E-07	2.36E-07
SMIM32	-1.25496	3.654401	1.27E-07	2.38E-07
STUM	-1.08757	3.760308	1.30E-07	2.44E-07
DSG3	2.226263	-3.19984	1.30E-07	2.44E-07
CACNA1C	1.60461	-1.60677	1.32E-07	2.47E-07
LRRC52	-2.22514	-1.91566	1.33E-07	2.50E-07
SLN	2.652988	0.662747	1.33E-07	2.50E-07
SERPINF2	1.037727	7.767375	1.37E-07	2.56E-07
EYA1	1.827246	1.094964	1.39E-07	2.61E-07
CAPN6	-1.29603	4.534532	1.41E-07	2.63E-07
CCIN	1.059031	-2.2902	1.45E-07	2.71E-07
FCAR	1.112896	-1.1659	1.50E-07	2.80E-07
CHST9	1.213159	4.387089	1.55E-07	2.89E-07
FTCD	-1.23586	5.100977	1.65E-07	3.08E-07
CTSK	1.442961	5.489776	1.75E-07	3.26E-07
SERPINB7	-1.2987	-1.77629	1.78E-07	3.31E-07
SLC30A3	1.175322	-1.30166	1.78E-07	3.32E-07
WFDC2	-1.10075	6.35626	1.85E-07	3.44E-07
HCN4	-1.08885	-0.98762	1.98E-07	3.67E-07
ERAP2	1.081188	6.468137	2.11E-07	3.92E-07
GYG2	-1.22786	1.21942	2.14E-07	3.97E-07
UGT1A10	2.751644	0.461721	2.32E-07	4.29E-07
SEZ6	1.272227	-2.31147	2.37E-07	4.37E-07
TUBA3C	2.955055	-1.96154	2.38E-07	4.40E-07
GCOM1	-1.40778	-0.78883	2.46E-07	4.54E-07
KRTAP5-1	1.486603	-2.31227	2.46E-07	4.54E-07
LRG1	1.227718	4.057165	2.51E-07	4.64E-07
SLC35F3	1.267855	1.285997	2.56E-07	4.71E-07
ODAPH	1.500398	-2.34794	2.63E-07	4.85E-07
SLC13A5	-1.30029	-1.23309	2.68E-07	4.93E-07
GABRB1	-1.1699	-1.64278	2.77E-07	5.09E-07
SERPINC1	2.64842	2.712189	2.80E-07	5.15E-07
WFDC3	1.05289	-0.06763	2.80E-07	5.15E-07
C1orf167	1.046096	-3.01857	3.09E-07	5.67E-07
SSTR3	-1.00163	-2.33008	3.16E-07	5.79E-07
XIRP1	1.409535	-0.83981	3.22E-07	5.90E-07
TM6SF2	1.163499	1.944702	3.34E-07	6.11E-07
CNTNAP5	2.09577	1.650724	3.37E-07	6.18E-07
BMP5	-1.37648	0.668207	3.39E-07	6.21E-07
IL31RA	1.379084	-1.23145	3.49E-07	6.38E-07
SRRM3	1.185708	1.834297	3.55E-07	6.50E-07
KCTD4	1.394016	-0.27192	3.61E-07	6.60E-07
CYSLTR2	1.602304	4.7419	3.69E-07	6.74E-07
NTS	-1.09197	0.593467	3.69E-07	6.74E-07
U2AF1L5	1.354099	-1.6301	3.89E-07	7.10E-07
MYZAP	-1.22306	1.443011	3.94E-07	7.18E-07
TMEM82	-1.14063	2.409135	4.11E-07	7.49E-07
TPTE2	1.224937	-2.63133	4.29E-07	7.81E-07
POU3F4	-2.30282	-0.07477	4.33E-07	7.89E-07
CYP2C9	1.737642	1.197045	4.37E-07	7.95E-07
DRICH1	1.047347	-1.029	4.43E-07	8.06E-07
SLC25A52	1.155099	-3.20326	4.80E-07	8.72E-07
IGDCC4	1.436273	2.721229	4.99E-07	9.06E-07
HJV	2.061876	-0.66353	5.06E-07	9.18E-07
CASP14	5.131813	4.317475	5.12E-07	9.28E-07
PPDPFL	2.780894	3.198692	5.14E-07	9.32E-07
DIPK1C	1.695752	-0.69223	5.16E-07	9.35E-07
DACT2	-1.27338	1.568969	5.18E-07	9.39E-07
CAPN8	-1.3661	0.59402	5.20E-07	9.42E-07
GYPA	-1.1744	-0.46261	5.28E-07	9.57E-07
UGT3A2	-1.36952	0.493019	5.55E-07	1.00E-06
SEMA3A	1.145899	1.862486	5.61E-07	1.01E-06
XKR7	1.971974	-1.04465	5.73E-07	1.04E-06
PRAP1	-1.16124	5.024886	5.93E-07	1.07E-06
UGT2B4	3.241482	0.534018	6.23E-07	1.12E-06
UGT2B11	1.103997	-0.57835	6.46E-07	1.17E-06
TDRD9	-1.08045	2.017168	6.54E-07	1.18E-06
TRARG1	-1.34807	-1.78644	6.57E-07	1.18E-06
PCDHA11	-1.07596	0.293687	6.75E-07	1.22E-06
SLC13A1	-1.26783	5.432265	6.89E-07	1.24E-06
P11-219A1	1.856047	-1.23929	7.13E-07	1.28E-06
SLC5A8	1.541656	5.19507	7.31E-07	1.31E-06
WFIKKN2	-1.3968	-1.99632	7.47E-07	1.34E-06

NUPR2	-1.91954	0.668211	7.66E-07	1.37E-06
ZMAT4	1.899147	2.359081	7.76E-07	1.39E-06
CYP2W1	1.523065	-1.24517	7.98E-07	1.43E-06
IL23R	1.0931	-2.43854	8.32E-07	1.49E-06
PTGDS	-1.21318	5.476311	8.42E-07	1.51E-06
ADGRB1	1.448787	2.072368	8.80E-07	1.57E-06
TMEM196	2.254082	-1.41205	8.89E-07	1.59E-06
SLC6A13	1.07386	7.312766	9.08E-07	1.62E-06
STYXL2	1.047972	-1.67292	9.14E-07	1.63E-06
FOXB1	2.021719	-2.45464	9.29E-07	1.66E-06
TUBB4A	1.396251	3.42848	9.81E-07	1.75E-06
SLITRK2	1.442718	2.469657	1.03E-06	1.84E-06
CCNO	-1.16412	0.332553	1.03E-06	1.84E-06
OTX1	1.732011	-2.06949	1.06E-06	1.89E-06
ADRB1	-1.18126	0.204809	1.07E-06	1.90E-06
CDH15	1.50084	-0.6872	1.09E-06	1.93E-06
FGG	2.820716	6.84495	1.17E-06	2.07E-06
EPB42	1.184682	-1.82899	1.22E-06	2.17E-06
FCRL2	1.393921	-1.36496	1.25E-06	2.21E-06
CFTR	2.11045	4.378795	1.27E-06	2.25E-06
OMD	-1.00775	0.898772	1.31E-06	2.32E-06
ADH1A	2.774368	0.229357	1.33E-06	2.35E-06
KCNG3	-1.57175	-2.14746	1.36E-06	2.40E-06
CNTN6	1.739627	0.890717	1.47E-06	2.60E-06
KRTAP5-9	1.044816	-1.83111	1.49E-06	2.63E-06
F10	1.22135	3.440185	1.53E-06	2.70E-06
TEX101	2.257747	-2.62773	1.58E-06	2.78E-06
C6orf118	-1.13338	-3.33335	1.62E-06	2.85E-06
KCNH8	1.272818	-1.21012	1.62E-06	2.86E-06
G6PC2	1.28804	-2.23103	1.64E-06	2.89E-06
CXCL3	1.009413	0.221367	1.66E-06	2.92E-06
DSCAM	1.379218	-1.21555	1.66E-06	2.92E-06
FAM180B	-1.12191	-2.9576	1.66E-06	2.93E-06
STAR	1.0537	-2.2845	1.68E-06	2.96E-06
ZIC4	1.9695	-2.73694	1.73E-06	3.04E-06
ZIC2	2.302223	-1.34211	1.76E-06	3.10E-06
VSTM2L	1.791132	1.26053	1.79E-06	3.15E-06
IGF2BP2	-1.00329	2.938601	1.80E-06	3.15E-06
CALCA	-2.04669	2.58629	1.81E-06	3.17E-06
PCSK9	-1.31461	-1.67717	1.83E-06	3.21E-06
SLC7A10	-1.2515	-1.32383	1.90E-06	3.33E-06
PROM1	-1.22184	6.431492	1.91E-06	3.34E-06
RTL3	1.541068	-3.12077	1.93E-06	3.38E-06
C7orf57	1.169029	-0.80658	2.04E-06	3.56E-06
RIC3	-1.05795	2.359823	2.13E-06	3.71E-06
CD19	1.115242	-0.38346	2.16E-06	3.76E-06
TBC1D3D	2.77199	-3.32383	2.32E-06	4.04E-06
NKRD20A	1.183726	-2.67274	2.33E-06	4.06E-06
CCL16	1.164383	-1.47932	2.48E-06	4.30E-06
MYT1L	1.402113	-3.24185	2.69E-06	4.67E-06
PF4	1.00732	-1.96833	2.70E-06	4.69E-06
GLP1R	1.27391	-0.75191	2.70E-06	4.69E-06
EEF1A2	1.807044	3.48936	2.78E-06	4.82E-06
TMEM211	1.014494	-2.95138	2.84E-06	4.92E-06
PITX2	1.908165	1.337943	2.88E-06	4.98E-06
CDHR5	1.064221	7.40989	3.06E-06	5.29E-06
HABP2	1.744634	6.118609	3.12E-06	5.39E-06
GPX2	-1.07473	0.660736	3.28E-06	5.65E-06
ZNF676	-1.16974	0.238422	3.37E-06	5.80E-06
IQSEC3	1.244152	3.973879	3.41E-06	5.87E-06
CTNNA2	2.414269	0.036733	3.48E-06	5.99E-06
KLF1	1.404669	-2.82185	3.51E-06	6.03E-06
RFPL4A	1.195373	-3.26485	3.52E-06	6.04E-06
CYP24A1	-1.32432	3.769728	3.56E-06	6.11E-06
PTK6	1.022332	1.384237	3.59E-06	6.17E-06
INSYN2B	1.129275	1.372327	3.94E-06	6.76E-06
C4BPB	1.620412	-0.33505	4.11E-06	7.04E-06
PTCHD1	-1.17151	-1.08757	4.13E-06	7.07E-06
MLANA	1.385896	0.945919	4.18E-06	7.16E-06
MTRNR2L	1.428199	-1.23588	4.25E-06	7.26E-06
HV3OR16	1.986003	-2.60798	4.31E-06	7.36E-06
TRIM29	-1.07951	1.985805	4.33E-06	7.41E-06
CALCR	1.19356	2.721194	4.45E-06	7.61E-06
XDH	1.783734	0.401772	4.51E-06	7.69E-06
MYPN	1.357056	-2.33891	4.55E-06	7.76E-06
NCAM1	1.354598	4.459234	4.98E-06	8.49E-06
UGT1A6	1.104175	5.227551	5.23E-06	8.89E-06
FGB	2.331444	7.493634	5.35E-06	9.09E-06
TPSD1	1.304435	-0.10468	5.35E-06	9.09E-06
TTLL6	1.032088	2.320682	5.44E-06	9.23E-06
TMEM130	1.353147	5.343896	5.45E-06	9.26E-06
NT5DC4	1.234126	-2.15767	5.57E-06	9.46E-06
NTSR1	1.430673	0.93633	5.98E-06	1.01E-05
ELSPBP1	3.016186	-1.56247	6.51E-06	1.10E-05
JCHAIN	1.283699	7.007781	6.59E-06	1.11E-05
RIMS4	-1.27891	-2.09399	6.81E-06	1.15E-05
TUBA3E	2.27031	0.934436	7.46E-06	1.26E-05
HMGA2	2.085464	1.536764	7.55E-06	1.27E-05
TM4SF4	1.724777	1.592999	8.03E-06	1.35E-05
PGPEP1L	-1.09601	-2.20757	9.16E-06	1.53E-05
GALNTL6	1.484217	-0.93629	9.55E-06	1.60E-05
LHCGR	2.494082	-0.36384	9.92E-06	1.66E-05
CXCL1	1.289837	3.421778	1.02E-05	1.70E-05
OGN	-1.13479	2.625207	1.04E-05	1.73E-05
FRRS1L	-1.196	-2.31015	1.04E-05	1.74E-05
HHIPL2	1.274309	-2.38959	1.08E-05	1.81E-05
CHST8	1.009496	-2.37756	1.12E-05	1.86E-05
A2ML1	1.076539	-1.83053	1.14E-05	1.90E-05
MEGF11	1.125799	2.995432	1.17E-05	1.94E-05
KRT14	1.633005	-0.55909	1.26E-05	2.09E-05
CHRDL2	1.330982	1.613652	1.30E-05	2.16E-05
SERTM1	-1.21627	-2.38947	1.36E-05	2.25E-05
ELANE	1.262545	-2.28407	1.45E-05	2.40E-05
CDH17	1.51926	1.35557	1.47E-05	2.43E-05
KCNE5	1.518561	-0.76814	1.49E-05	2.46E-05
COL20A1	1.261702	-2.09573	1.49E-05	2.47E-05
IFNE	1.465026	-1.62886	1.52E-05	2.52E-05
P11-404P2	-1.11799	-3.31235	1.55E-05	2.56E-05
EDN3	-1.37792	-1.67097	1.60E-05	2.64E-05
FGL1	2.563395	0.652399	1.61E-05	2.65E-05
TBX10	1.016277	-3.22722	1.68E-05	2.76E-05
RIMS1	1.71192	-0.67582	1.68E-05	2.77E-05
TMEM132I	1.194923	0.123208	1.86E-05	3.05E-05
FGF7	-1.01674	2.665925	1.97E-05	3.22E-05
HTR3A	1.514687	-1.94556	1.97E-05	3.23E-05
INSYN1	-1.06202	2.880218	2.00E-05	3.28E-05
DCX	1.031252	-1.23355	2.02E-05	3.31E-05
SLC9C2	1.298888	-1.25574	2.02E-05	3.31E-05
AIRE	1.06568	-2.37251	2.03E-05	3.33E-05
KCNA2	1.171826	-1.4553	2.15E-05	3.51E-05
SLC8A2	1.21202	-1.93582	2.20E-05	3.59E-05
SLC38A3	-1.32868	1.490881	2.24E-05	3.66E-05
DLL3	1.409235	-2.86812	2.26E-05	3.68E-05
KRT4	1.941281	-2.34804	2.26E-05	3.68E-05
KCNA1	-1.19298	-2.95116	2.36E-05	3.84E-05
TEKT4	1.078636	-2.9778	2.37E-05	3.87E-05
HES7	1.034501	-2.52996	2.39E-05	3.89E-05
EPHA10	1.334808	0.624561	2.52E-05	4.10E-05
SFTPB	2.201022	2.582906	2.62E-05	4.26E-05
CCDC187	-1.36287	-2.34182	2.79E-05	4.52E-05
AKR1B15	2.233367	-1.39194	2.80E-05	4.53E-05
CRHR1	1.946141	0.104934	3.23E-05	5.20E-05
DPP10	-1.47912	-0.71817	3.56E-05	5.73E-05
LPAR3	1.675703	-0.51932	3.84E-05	6.16E-05
RGPD1	1.047748	-3.4522	3.92E-05	6.29E-05
S100A1	1.008641	6.183328	4.15E-05	6.65E-05
ADGRF4	1.570249	-0.119	4.22E-05	6.75E-05
SNTG1	-1.45879	-1.96604	4.45E-05	7.11E-05
ADH4	2.443555	1.386868	4.54E-05	7.25E-05
DSG1	1.104069	-3.16844	4.77E-05	7.59E-05
ACTL8	2.513024	-2.68494	4.88E-05	7.76E-05
PRG4	1.337243	1.102862	4.96E-05	7.89E-05
NFRSF13	1.043946	-1.6312	5.36E-05	8.50E-05
DAPL1	2.023205	1.385194	5.64E-05	8.93E-05
AHSP	1.594157	-1.75285	6.73E-05	0.000105773
DMRT2	-1.63292	3.788505	6.90E-05	0.000108405
DPYSL5	1.728361	-1.0059	7.27E-05	0.000114041
VTN	1.662014	3.396483	7.35E-05	0.000115222
CYP26C1	1.128062	-1.7421	8.58E-05	0.000134003
NKX3-2	1.096514	-2.71492	9.25E-05	0.000143997
PCSK1	1.017687	0.960282	9.65E-05	0.000150104
PMEL	1.10225	3.070511	9.70E-05	0.000150925
LCN2	-1.26001	2.658162	9.73E-05	0.000151406
PLEKHS1	-1.05836	0.390808	9.76E-05	0.00015171
CNGB1	1.580247	-0.85509	9.77E-05	0.000151954
GRIN1	1.233289	-2.59966	9.80E-05	0.000152317
LRFN5	1.40895	0.799595	0.000102258	0.000158691
FAM83F	-1.02305	3.307513	0.000107351	0.000166108
MMP1	1.301551	3.581414	0.000107399	0.000166152
MS4A10	-1.30411	-2.05883	0.000108348	0.000167575
ADIPOQ	-1.77812	-0.35939	0.000108748	0.000168162
APOA1	1.816255	1.770203	0.000109579	0.000169339
KLK3	2.340887	-0.12661	0.000109719	0.000169524
NPPC	1.435608	-0.70051	0.000117444	0.000181062
HAO1	-1.61182	-1.56588	0.000128134	0.000197112
COL10A1	1.193387	1.32559	0.000128202	0.000197198
SLC1A7	1.07719	1.139439	0.000130227	0.000200131
GPR26	1.045979	-3.3573	0.000130697	0.000200799
NPFFR2	-1.04295	-2.83279	0.000131047	0.000201301
CEACAM7	-1.49221	-2.88588	0.000131874	0.000202534
SLC6A2	-1.40679	-1.62533	0.000135013	0.000207166
NIPAL4	1.283331	-0.5467	0.000139498	0.000213816
LGI1	1.711661	-1.71632	0.000140773	0.000215711
AMER2	-1.2662	-2.59443	0.000140809	0.000215727
UGT1A8	1.656664	-0.51978	0.000143821	0.000220122
SLC26A3	1.270957	-1.516	0.000145599	0.000222784
SERPINA1	1.55641	-0.633	0.00014864	0.000227375
MYH2	1.595604	-2.86607	0.000168108	0.000256031
EN2	1.262136	-0.90362	0.000174942	0.000266046
SPATA22	1.165421	-1.94811	0.000179163	0.000272293
CFAP47	1.222183	0.36246	0.000189334	0.000287288
KLK15	2.945225	0.670902	0.000197545	0.000299531
MMP8	1.454735	-2.5922	0.000202701	0.000307267
ANGPTL7	-1.11226	-0.87867	0.000208183	0.00031521
CHIA	-1.04472	-2.60216	0.000214799	0.000324993
KLB	1.207156	1.402156	0.000221298	0.000334408
IL1R2	1.16354	4.673761	0.000229124	0.000346017
ZFR2	1.041127	-1.53602	0.000230072	0.000347387
LGI3	1.434027	-0.12419	0.000234671	0.000354015
ARPP21	1.232871	0.721786	0.000235816	0.00035568

RORB	-1.06239	0.483576	0.000239149	0.000360449
GLB1L3	1.349592	0.886289	0.000241101	0.000363166
APOA2	2.111566	1.119236	0.000242487	0.00036509
H1-3	1.562002	-0.83328	0.000255794	0.000384407
CCKBR	1.024067	-3.00762	0.000329329	0.000490471
ABCG8	-1.12883	-1.43964	0.00034975	0.000520197
TFF1	-1.30973	-1.22493	0.000355723	0.000528663
ASB11	1.050097	-2.36493	0.000370922	0.000550429
MYH4	1.497093	-2.4696	0.000380347	0.000564068
HRG	-1.56379	4.532892	0.000388626	0.000575993
ASB4	1.166526	-0.1166	0.000426953	0.000630863
RHBG	-1.3948	3.079868	0.000445854	0.000657742
TFF2	1.426658	-1.44551	0.000459601	0.000677389
LRRTM1	2.432141	0.607338	0.000511925	0.000752083
FAM9B	1.110175	-2.23353	0.000541331	0.000793837
CIDEC	1.527515	0.800761	0.00054696	0.000801952
FADS6	-1.06879	-0.87904	0.000581614	0.000850844
OOEP	1.225743	-1.64821	0.000586423	0.000857582
HTR1D	1.012125	-1.24417	0.00059001	0.000862679
RGS22	1.094955	0.065247	0.000642505	0.000937166
COL2A1	1.019444	-2.33715	0.000658925	0.000960124
FAM133A	1.337723	-1.44815	0.000806545	0.001168178
IL6	1.094313	3.292872	0.000833707	0.001206074
ADGRG7	-1.22902	-1.49011	0.000839081	0.001213523
NRAP	1.187417	0.390602	0.000852318	0.001231733
TTR	1.90484	2.722977	0.000866329	0.001251446
CPN2	-1.17363	3.391672	0.000968259	0.001393571
HRK	1.209913	0.253888	0.001042753	0.001495951
ADCYAP1	1.00335	1.485203	0.001057637	0.001516275
LRRC15	1.262942	2.106358	0.001059379	0.001518643
C10orf71	-1.42661	-1.68027	0.001118326	0.001599215
ZFP57	1.066169	-0.14834	0.001201497	0.001713949
TTC6	1.108413	-3.17597	0.001218452	0.001737696
APOC3	2.134174	3.584805	0.001270942	0.001809656
ATP6V0D2	-1.20219	5.999292	0.001462445	0.002072042
ANKRD30I	1.344241	-2.20919	0.001528076	0.002160868
UPK3BL1	1.171183	-0.98951	0.001559623	0.002203452
TMEM151	1.051175	-1.29482	0.00157977	0.002230985
UNC93A	-1.07319	0.334843	0.001594685	0.002250921
HEMGN	1.05464	-1.64767	0.00187668	0.002634891
CACNG5	1.085277	-2.42742	0.001910592	0.002680724
SCGB2A2	-1.16547	-2.38127	0.001945021	0.002727221
KRT13	-1.17469	0.856086	0.001947266	0.002730142
CEACAM5	-1.12413	-2.42452	0.001954009	0.002738916
ANXA8	1.494126	-1.61232	0.002066364	0.002889937
PSCA	-1.04728	1.281892	0.002068376	0.002892512
H1-4	1.15201	0.473706	0.002672468	0.003708181
MS4A8	1.295742	-2.4399	0.002720822	0.003771872
PAK5	-1.22596	-1.1142	0.003271671	0.004508175
KRT16	1.079158	-1.39003	0.003374481	0.004646057
ADGRG4	1.140756	-2.34901	0.003722962	0.005105906
ITIH2	1.095076	2.242509	0.003726223	0.005109964
GJB6	1.246692	-0.87105	0.0038762	0.005304026
APOB	1.065952	5.356565	0.003953774	0.005405803
IGF2	1.142684	8.143893	0.004109088	0.00560909
KRT6A	1.521574	-0.76365	0.00412685	0.005632881
FUT9	-1.01904	0.035886	0.004347519	0.00592261
SLC6A15	1.558439	0.541964	0.00437826	0.005962568
TEX55	-1.08403	-3.25416	0.00457603	0.006220885
WFDC5	1.619805	0.090329	0.005594119	0.007538591
PON1	1.226966	-0.02997	0.005712875	0.007690968
CXCL6	1.038876	3.348179	0.005792717	0.007790397
REG3G	1.507903	1.202972	0.005951888	0.00799556
ZIC1	1.154069	-2.23508	0.006377943	0.008541455
SMOC1	1.040455	3.608929	0.007161406	0.009540522
SNAP91	1.198508	-1.25916	0.007568537	0.010061051
SP8	1.643445	-1.59297	0.009300664	0.012250026
STAC2	1.072125	4.891462	0.012502887	0.016261346
GABRA3	1.010471	-1.3295	0.014543467	0.018763867
FOXI1	-1.11218	4.641138	0.027361113	0.0344063
ATP6V1G3	-1.14169	2.824932	0.038862968	0.048101026
GPR87	1.035906	-0.81805	0.041675582	0.051446718
CYP1A2	1.048179	-0.75002	0.046690293	0.057385911
ERVV-2	1.058655	-0.57702	0.047053835	0.057794941
