# Supplementary material for: N6-methyladenosine-modified ALDH9A1 modulates lipid accumulation and tumor progression in clear cell renal cell carcinoma through the NPM1/IQGAP2/AKT signaling pathway
Source: Cell Death Dis. 2024 Jul 22;15(7):520. doi: 10.1038/s41419-024-06896-z (PMC11263707; doi:10.1038/s41419-024-06896-z)
Supplement: Supplementary file 5 — supplementary table 4 [file 41419_2024_6896_MOESM5_ESM.pdf]

ALDH9A1-IP/MS							
Gene Nar	Coverage	# Peptide	# PSMs	# Unique	# AAs	MW [kDa	calc. pI
VAMP1	6	1	1	1	118	12.9	6.65
TXNDC1	4	1	1	1	172	19.2	5.4
IGKV2D-	11	1	4	1	121	13.3	4.61
CBX3	15	4	4	4	183	20.8	5.33
ALDOA	25	8	9	8	364	39.4	8.09
YWHAZ	22	5	5	3	245	27.7	4.79
EEF1A2	12	5	5	1	463	50.4	9.03
KLHL42	2	1	1	1	505	56.8	5.74
GRWD1	2	1	2	1	446	49.4	4.92
CASP14	7	2	2	2	242	27.7	5.58
HNRNPA	18	6	7	5	332	36.2	8.21
SUCLG1	4	1	1	1	346	36.2	8.79
STT3A	1	1	1	1	705	80.5	8.07
RAB10	9	2	2	2	200	22.5	8.38
CNBP	17	3	3	3	177	19.5	7.71
SETDB1	1	1	1	1	1291	143.1	6.02
NUCKS1	4	1	1	1	243	27.3	5.08
DIABLO	4	1	1	1	239	27.1	5.9
HADH	5	2	2	2	314	34.3	8.85
HBA1	11	2	2	2	142	15.2	8.68
LAMP2	4	2	2	2	410	44.9	5.63
RPL3	8	3	3	3	403	46.1	10.18
EEF1D	3	1	1	1	281	31.1	5.01
LAP3	2	1	1	1	519	56.1	7.93
NDUFA5	22	2	2	2	116	13.5	5.99
RCC1	18	5	5	5	421	44.9	7.52
ATP5PB	12	3	3	3	256	28.9	9.36
ANXA6	10	7	7	7	673	75.8	5.6
RPL13	23	5	5	5	211	24.2	11.65
ILF3	3	3	3	3	894	95.3	8.76
ME2	3	2	2	2	584	65.4	7.61
SDHB	7	3	3	3	280	31.6	8.76
XRCC5	2	1	1	1	732	82.7	5.81
SNRNP7(	10	5	5	5	437	51.5	9.94
AGPS	2	1	1	1	658	72.9	7.34
NME1	23	3	4	3	152	17.1	6.19
RPLP0	3	1	1	1	317	34.3	5.97
TBCA	9	1	1	1	108	12.8	5.29
HSP90B1	28	21	27	20	803	92.4	4.84
FUBP1	7	5	5	3	644	67.5	7.61
GNL3	1	1	1	1	549	62	9.16
GATAD2	2	1	1	1	633	68	9.94
SAFB	6	5	5	4	915	102.6	5.47
MAP4	3	3	3	3	1152	120.9	5.43
RPL12	19	2	2	2	165	17.8	9.42
HACD3	4	1	1	1	362	43.1	8.94
PYCR2	9	2	2	2	320	33.6	7.77
CHCHD2	9	1	1	1	151	15.5	9.89
IGF2BP1	3	2	2	2	577	63.4	9.2
EWSR1	4	2	2	2	656	68.4	9.33
RBBP7	12	6	6	2	425	47.8	5.05
TGM3	3	2	2	2	693	76.6	5.86
MRPL53	9	1	1	1	112	12.1	8.76
ACIN1	1	1	1	1	1341	151.8	6.43
LMAN1	6	3	3	3	510	57.5	6.77
CCT7	1	1	1	1	543	59.3	7.65
HNRNPA	24	7	10	7	378	39.6	9.01
NDUFS1	6	4	4	4	727	79.4	6.23
RUVBL2	3	1	1	1	463	51.1	5.64
COX7A2	12	1	1	1	83	9.4	9.76
COX17	25	1	1	1	63	6.9	7.24
NCBP2	7	1	1	1	156	18	8.21
CISD1	20	2	2	2	108	12.2	9.09
DDX21	1	1	1	1	783	87.3	9.28
NDUFS4	5	1	1	1	175	20.1	10.3
AHSG	2	1	1	1	367	39.3	5.72
DDX3X	8	4	5	3	662	73.2	7.18
PTPN1	6	2	2	2	435	49.9	6.27
CADM1	4	2	2	2	442	48.5	5.07
TGM1	2	2	2	2	817	89.7	6.04
PDIA4	14	8	10	8	645	72.9	5.07
HMGB3	7	1	1	1	200	23	8.37
GTPBP4	1	1	1	1	634	73.9	9.5
SNRNP4(	3	1	1	1	357	39.3	8.1
MIF	17	2	2	2	115	12.5	7.88
RANBP1	4	1	1	1	201	23.3	5.29
HMGB2	22	4	4	3	209	24	7.81
KPNB1	2	2	2	2	876	97.1	4.78
RTRAF	13	3	3	3	244	28.1	6.65
CCT4	3	2	2	2	539	57.9	7.83
SSBP1	22	3	4	3	148	17.2	9.6
LGALS1	9	1	1	1	135	14.7	5.5
VIM	55	25	30	21	466	53.6	5.12
SNRPA	6	2	2	2	282	31.3	9.83
RPL14	6	1	1	1	215	23.4	10.93
HSP90A	15	10	12	4	732	84.6	5.02
ALB	8	5	5	5	609	69.3	6.28
RPS21	12	1	1	1	83	9.1	8.5
YWHAB	12	3	3	1	246	28.1	4.83
TMX1	4	1	1	1	280	31.8	4.98
CMC1	9	1	1	1	106	12.5	8.63
TMEM9E	4	1	1	1	198	22.5	8.06
SET	14	3	3	3	290	33.5	4.32
VCP	8	6	6	6	806	89.3	5.26
KRT73	7	6	8	1	540	58.9	7.23
SLC1A5	2	1	1	1	541	56.6	5.48
NVL	1	1	1	1	856	95	6.48
EBNA1B	3	1	1	1	306	34.8	10.1
OPA1	1	1	1	1	960	111.6	7.87
MRPS22	6	2	3	2	360	41.3	7.9
H2AZ1	15	2	2	1	128	13.5	10.58
SNRPD3	25	2	2	2	126	13.9	10.32
NUCB2	8	3	3	3	420	50.2	5.12
KRT2	53	30	43	21	639	65.4	8
POLDIP2	2	1	1	1	368	42	8.63
GALNT2	1	1	1	1	571	64.7	8.35
LYRM7	10	1	1	1	104	11.9	9.66
RPS19	26	4	4	4	145	16.1	10.32
G3BP1	4	2	2	2	466	52.1	5.52
DDOST	2	1	1	1	456	50.8	6.55
RPL10A	4	1	1	1	217	24.8	9.94
FASN	0	1	1	1	2511	273.3	6.44
PRSS3	4	1	1	1	304	32.5	7.49
MRPL49	16	2	2	2	166	19.2	9.45
HNRNPA	18	3	3	3	305	30.8	9.29
PPIF	12	3	3	3	207	22	9.38
MANF	8	1	1	1	182	20.7	8.69
FDX1	18	4	4	4	184	19.4	5.83
KRT14	40	19	23	7	472	51.5	5.16
CLPB	1	1	1	1	707	78.7	9.01
LSM14A	3	1	2	1	463	50.5	9.52
PDHA1	4	3	3	3	390	43.3	8.06
CTTN	1	1	1	1	550	61.5	5.4
GCN1	0	1	1	1	2671	292.5	7.43
HRNR	19	16	17	16	2850	282.2	10.04
PHF5A	7	1	1	1	110	12.4	8.41
RPS10	5	1	1	1	165	18.9	10.15
MACROI	3	1	1	1	325	35.5	9.51
RPL6	3	1	1	1	288	32.7	10.58
PCMT1	4	1	1	1	227	24.6	7.21
CSDE1	2	2	2	2	798	88.8	6.25
MRPL42	11	1	1	1	142	16.7	8.35
DLST	7	3	3	3	453	48.7	8.95

Vector-IP/MS							
Gene Nar	Coverage [%]	# Peptide	# PSMs	# Unique	# AAs	MW [kDa	calc. pI
MEF2C	4	1	1	1	473	51.2	8.03
NUCKS1	4	1	1	1	243	27.3	5.08
RPL13	4	1	1	1	211	24.2	11.65
ILF3	1	1	1	1	894	95.3	8.76
HSP90B1	1	1	1	1	803	92.4	4.84
KRT77	6	4	6	1	578	61.9	5.99
TGM3	2	1	1	1	693	76.6	5.86
HNRNPA	5	2	2	2	378	39.6	9.01
TGM1	1	1	1	1	817	89.7	6.04
VIM	6	3	3	3	466	53.6	5.12
HSP90A	1	1	1	1	732	84.6	5.02
ALB	10	6	6	6	609	69.3	6.28
H2AZ1	15	2	2	1	128	13.5	10.58
KRT2	34	20	24	13	639	65.4	8
BIRC5	7	1	1	1	142	16.4	5.9
KRT14	26	13	14	2	472	51.5	5.16
HRNR	10	6	6	6	2850	282.2	10.04
KRT31	8	3	4	2	416	47.2	4.88
KRT19	15	8	9	2	400	44.1	5.14
HNRNPR	2	1	1	1	633	70.9	8.13
S100A7	7	1	1	1	101	11.5	6.77
KRT10	44	23	33	19	584	58.8	5.21
CUL3	2	1	2	1	768	88.9	8.48
RPS27A	10	1	1	1	156	18	9.64
KRT1	38	28	45	22	644	66	8.12
KRT6B	28	16	20	1	564	60	8
KRT6A	27	16	20	1	564	60	8
KPRP	3	2	2	2	579	64.1	8.27
PHB2	3	1	1	1	299	33.3	9.83
KRT80	4	2	2	1	452	50.5	5.67
SLC25A	3	1	1	1	298	32.8	9.69
PIP	8	1	1	1	146	16.6	8.05
DSG1	6	6	6	6	1049	113.7	5.03
CIRBP	6	1	1	1	172	18.6	9.51
KRT85	5	3	3	3	507	55.8	6.55
KRT18	6	3	4	1	430	48	5.45
DSP	2	7	7	7	2871	331.6	6.81
SLC4A8	2	1	1	1	1093	122.9	6.68
MKI67	0	1	1	1	3256	358.5	9.45
HSPA8	4	3	3	2	646	70.9	5.52
H2BC12	29	4	4	1	126	13.9	10.32
KRT34	4	2	2	2	436	49.4	5.06
EEF1A1	6	3	3	3	462	50.1	9.01
ACTB	19	6	7	6	375	41.7	5.48
HNRNPU	7	5	6	5	825	90.5	6
PKP1	3	2	2	2	747	82.8	9.13
OFD1	1	1	1	1	1012	116.6	6.1
ANXA2	6	2	2	2	339	38.6	7.75
H2BC13	29	4	4	1	126	13.9	10.32
LMNA	2	1	1	1	664	74.1	7.02
ILF2	3	1	1	1	390	43	5.26
HSPA5	3	2	2	1	654	72.3	5.16
SERPINE	4	2	2	2	405	46.2	5.53
JUP	4	2	2	2	745	81.7	6.14
KRT15	10	6	7	1	456	49.2	4.77
HNRNPA	17	6	6	5	353	37.4	8.95
	2	1	1	1	449	49.3	8.72
IGKV2D-	17	2	2	2	120	13.1	7.12
H4C1	50	5	6	5	103	11.4	11.36
LYZ	11	2	2	2	148	16.5	9.16
ANO8	1	1	1	1	1232	135.9	5.82
PPIA	5	1	1	1	165	18	7.81
ATP5F1A	5	3	3	3	553	59.7	9.13
MYH9	0	1	1	1	1960	226.4	5.6
H1-2	23	5	5	5	213	21.4	10.93
RPS4X	3	1	1	1	263	29.6	10.15
BCLAF1	1	1	1	1	920	106.1	9.98
NCL	1	1	1	1	710	76.6	4.7
TAF15	6	1	1	1	592	61.8	8.02
H2AX	11	2	2	1	143	15.1	10.74
HNRNPD	2	1	1	1	420	46.4	9.57
TUBA1B	2	1	1	1	451	50.1	5.06
RPL15	4	1	1	1	204	24.1	11.62
LTF	3	2	2	2	710	78.1	8.12
RPL7	8	2	2	2	248	29.2	10.65
ENO1	2	1	1	1	434	47.1	7.39
KRT17	16	8	9	2	432	48.1	5.02
TXN	9	1	1	1	105	11.7	4.92
THADA	1	1	1	1	1953	219.5	6.06
TMPRSS	1	1	1	1	586	63.1	8.63
DEFA1	10	1	1	1	94	10.2	6.99
GSDMA	2	1	1	1	445	49.3	5.29
KRT78	5	3	3	2	520	56.8	6.02
FABP5	7	1	1	1	135	15.2	7.01
KRT4	8	4	4	1	520	56.1	6.61
YLPM1	1	1	1	1	2146	241.5	6.55
GBP1	2	1	1	1	592	67.9	6.32
KRT9	44	23	35	22	623	62	5.24
KRT16	19	10	11	2	473	51.2	5.05
PTMA	34	3	3	3	111	12.2	3.78
DSC1	2	1	1	1	894	99.9	5.43
YWHAE	3	1	1	1	255	29.2	4.74
CTSD	2	1	1	1	412	44.5	6.54
HNRNPA	9	3	4	2	372	38.7	9.13
KRT5	34	22	26	12	590	62.3	7.74
RBM3	6	1	1	1	157	17.2	8.91

In ALDH9A1-IP/MS not Vector-IP/MS							
Gene Nar	Coverage	# Peptide	# PSMs	# Unique	# AAs	MW [kDa	calc. pI
VAMP1	6	1	1	1	118	12.9	6.65
TXNDC1:	4	1	1	1	172	19.2	5.4
IGKV2D-4	11	1	4	1	121	13.3	4.61
CBX3	15	4	4	4	183	20.8	5.33
ALDOA	25	8	9	8	364	39.4	8.09
YWHAZ	22	5	5	3	245	27.7	4.79
EEF1A2	12	5	5	1	463	50.4	9.03
KLHL42	2	1	1	1	505	56.8	5.74
GRWD1	2	1	2	1	446	49.4	4.92
CASP14	7	2	2	2	242	27.7	5.58
HNRNPA	18	6	7	5	332	36.2	8.21
SUCLG1	4	1	1	1	346	36.2	8.79
STT3A	1	1	1	1	705	80.5	8.07
RAB10	9	2	2	2	200	22.5	8.38
CNBP	17	3	3	3	177	19.5	7.71
SETDB1	1	1	1	1	1291	143.1	6.02
DIABLO	4	1	1	1	239	27.1	5.9
HADH	5	2	2	2	314	34.3	8.85
HBA1	11	2	2	2	142	15.2	8.68
LAMP2	4	2	2	2	410	44.9	5.63
RPL3	8	3	3	3	403	46.1	10.18
EEF1D	3	1	1	1	281	31.1	5.01
LAP3	2	1	1	1	519	56.1	7.93
NDUFA5	22	2	2	2	116	13.5	5.99
RCC1	18	5	5	5	421	44.9	7.52
ATP5PB	12	3	3	3	256	28.9	9.36
ANXA6	10	7	7	7	673	75.8	5.6
ME2	3	2	2	2	584	65.4	7.61
SDHB	7	3	3	3	280	31.6	8.76
XRCC5	2	1	1	1	732	82.7	5.81
SNRNP70	10	5	5	5	437	51.5	9.94
AGPS	2	1	1	1	658	72.9	7.34
NME1	23	3	4	3	152	17.1	6.19
RPLP0	3	1	1	1	317	34.3	5.97
TBCA	9	1	1	1	108	12.8	5.29
FUBP1	7	5	5	3	644	67.5	7.61
GNL3	1	1	1	1	549	62	9.16
GATAD2	2	1	1	1	633	68	9.94
SAFB	6	5	5	4	915	102.6	5.47
MAP4	3	3	3	3	1152	120.9	5.43
RPL12	19	2	2	2	165	17.8	9.42
HACD3	4	1	1	1	362	43.1	8.94
PYCR2	9	2	2	2	320	33.6	7.77
CHCHD2	9	1	1	1	151	15.5	9.89
IGF2BP1	3	2	2	2	577	63.4	9.2
EWSR1	4	2	2	2	656	68.4	9.33
RBBP7	12	6	6	2	425	47.8	5.05
MRPL53	9	1	1	1	112	12.1	8.76
ACIN1	1	1	1	1	1341	151.8	6.43
LMAN1	6	3	3	3	510	57.5	6.77
CCT7	1	1	1	1	543	59.3	7.65
NDUFS1	6	4	4	4	727	79.4	6.23
RUVBL2	3	1	1	1	463	51.1	5.64
COX7A2	12	1	1	1	83	9.4	9.76
COX17	25	1	1	1	63	6.9	7.24
NCBP2	7	1	1	1	156	18	8.21
CISD1	20	2	2	2	108	12.2	9.09
DDX21	1	1	1	1	783	87.3	9.28
NDUFS4	5	1	1	1	175	20.1	10.3
AHSG	2	1	1	1	367	39.3	5.72
DDX3X	8	4	5	3	662	73.2	7.18
PTPN1	6	2	2	2	435	49.9	6.27
CADM1	4	2	2	2	442	48.5	5.07
PDIA4	14	8	10	8	645	72.9	5.07
HMGB3	7	1	1	1	200	23	8.37
GTPBP4	1	1	1	1	634	73.9	9.5
SNRNP40	3	1	1	1	357	39.3	8.1
MIF	17	2	2	2	115	12.5	7.88
RANBP1	4	1	1	1	201	23.3	5.29
HMGB2	22	4	4	3	209	24	7.81
KPNB1	2	2	2	2	876	97.1	4.78
RTRAF	13	3	3	3	244	28.1	6.65
CCT4	3	2	2	2	539	57.9	7.83
SSBP1	22	3	4	3	148	17.2	9.6
LGALS1	9	1	1	1	135	14.7	5.5
SNRPA	6	2	2	2	282	31.3	9.83
RPL14	6	1	1	1	215	23.4	10.93
RPS21	12	1	1	1	83	9.1	8.5
YWHAB	12	3	3	1	246	28.1	4.83
TMX1	4	1	1	1	280	31.8	4.98
CMC1	9	1	1	1	106	12.5	8.63
TMEM9B	4	1	1	1	198	22.5	8.06
SET	14	3	3	3	290	33.5	4.32
VCP	8	6	6	6	806	89.3	5.26
KRT73	7	6	8	1	540	58.9	7.23
SLC1A5	2	1	1	1	541	56.6	5.48
NVL	1	1	1	1	856	95	6.48
EBNA1BI	3	1	1	1	306	34.8	10.1
OPA1	1	1	1	1	960	111.6	7.87
MRPS22	6	2	3	2	360	41.3	7.9
SNRPD3	25	2	2	2	126	13.9	10.32
NUCB2	8	3	3	3	420	50.2	5.12
POLDIP2	2	1	1	1	368	42	8.63
GALNT2	1	1	1	1	571	64.7	8.35
LYRM7	10	1	1	1	104	11.9	9.66
RPS19	26	4	4	4	145	16.1	10.32
G3BP1	4	2	2	2	466	52.1	5.52
DDOST	2	1	1	1	456	50.8	6.55
RPL10A	4	1	1	1	217	24.8	9.94
FASN	0	1	1	1	2511	273.3	6.44
PRSS3	4	1	1	1	304	32.5	7.49
MRPL49	16	2	2	2	166	19.2	9.45
HNRNPA(	18	3	3	3	305	30.8	9.29
PPIF	12	3	3	3	207	22	9.38
MANF	8	1	1	1	182	20.7	8.69
FDX1	18	4	4	4	184	19.4	5.83
CLPB	1	1	1	1	707	78.7	9.01
LSM14A	3	1	2	1	463	50.5	9.52
PDHA1	4	3	3	3	390	43.3	8.06
CTTN	1	1	1	1	550	61.5	5.4
GCN1	0	1	1	1	2671	292.5	7.43
PHF5A	7	1	1	1	110	12.4	8.41
RPS10	5	1	1	1	165	18.9	10.15
MACROD	3	1	1	1	325	35.5	9.51
RPL6	3	1	1	1	288	32.7	10.58
PCMT1	4	1	1	1	227	24.6	7.21
CSDE1	2	2	2	2	798	88.8	6.25
MRPL42	11	1	1	1	142	16.7	8.35
DLST	7	3	3	3	453	48.7	8.95
G3BP2	3	1	1	1	482	54.1	5.55
KRT28	8	5	8	1	464	50.5	5.47
SLTM	2	1	1	1	1034	117.1	7.87
CS	2	1	2	1	466	51.7	8.32
SRRM1	1	1	1	1	904	102.3	11.84
AIFM1	3	2	2	2	613	66.9	8.95
SRSF7	16	4	6	4	238	27.4	11.82
AMOT	12	14	14	14	1084	118	7.64
RPS20	9	1	1	1	119	13.4	9.94
RPL34	15	2	2	2	117	13.3	11.47
ERP29	5	1	1	1	261	29	7.31
ATP5PF	8	1	1	1	108	12.6	9.52
RBM14	7	5	5	5	669	69.4	9.67
TIMM44	4	2	2	2	452	51.3	8.32

G3BP2	3	1	1	1	482	54.1	5.55
KRT28	8	5	8	1	464	50.5	5.47
SLTM	2	1	1	1	1034	117.1	7.87
CS	2	1	2	1	466	51.7	8.32
SRRM1	1	1	1	1	904	102.3	11.84
AIFM1	3	2	2	2	613	66.9	8.95
SRSF7	16	4	6	4	238	27.4	11.82
AMOT	12	14	14	14	1084	118	7.64
RPS20	9	1	1	1	119	13.4	9.94
RPL34	15	2	2	2	117	13.3	11.47
ERP29	5	1	1	1	261	29	7.31
ATP5PF	8	1	1	1	108	12.6	9.52
RBM14	7	5	5	5	669	69.4	9.67
TIMM44	4	2	2	2	452	51.3	8.32
CAPRIN	2	2	2	2	709	78.3	5.25
POLDIP3	3	1	1	1	421	46.1	9.99
VAPA	5	1	1	1	249	27.9	8.62
LAMB1	1	1	1	1	1786	197.9	4.94
PHGDH	8	4	4	4	533	56.6	6.71
ECI1	14	4	5	4	302	32.8	8.54
HYOU1	9	9	10	9	999	111.3	5.22
IGHG2	5	2	2	2	326	35.9	7.59
TRMT10	3	1	1	1	403	47.3	9.36
LBR	3	2	2	2	615	70.7	9.36
HNRNPR	12	8	8	3	633	70.9	8.13
ANXA11	2	1	1	1	505	54.4	7.65
PSMB6	5	1	1	1	239	25.3	4.92
EMG1	4	1	1	1	244	26.7	9.17
S100A7	7	1	1	1	101	11.5	6.77
SNRPD2	16	2	2	2	118	13.5	9.91
GCSH	6	1	1	1	173	18.9	4.88
PSMB1	4	1	1	1	241	26.5	8.13
ESYT1	1	1	1	1	1104	122.8	5.83
CDK1	3	1	1	1	297	34.1	8.4
RBMX	23	10	12	10	391	42.3	10.05
MYH10	5	9	9	7	1976	228.9	5.54
SHMT2	22	10	11	10	504	56	8.53
RPL36	10	1	1	1	105	12.2	11.59
COQ9	3	1	1	1	318	35.5	5.94
MT-CO2	4	1	1	1	227	25.5	4.82
EMD	4	1	1	1	254	29	5.5
TUFM	29	11	12	11	455	49.8	7.61
RPS8	24	4	4	4	208	24.2	10.32
RBM8A	11	2	3	2	174	19.9	5.72
TRA2A	6	2	2	1	282	32.7	11.27
TOMM2	8	1	1	1	142	15.5	4.34
NAP1L1	5	2	2	2	391	45.3	4.46
PYCR1	13	3	3	3	319	33.3	7.61
NOL12	6	1	1	1	213	24.6	10.23
KRT10	51	25	43	20	584	58.8	5.21
TRPM4	1	1	1	1	1214	134.2	8.15
S100A8	31	3	3	3	93	10.8	7.03
RPS27A	37	4	5	4	156	18	9.64
KRT1	46	31	71	25	644	66	8.12
RPL4	4	2	2	2	427	47.7	11.06
NUP54	3	1	1	1	507	55.4	7.02
ALDH18	6	5	6	5	795	87.2	7.12
PNPT1	2	2	2	2	783	85.9	7.77
ZNF326	2	1	1	1	582	65.6	5.15
MTDH	3	2	2	2	582	63.8	9.32
RPS18	6	1	1	1	152	17.7	10.99
POR	2	1	1	1	677	76.6	5.58
GLUD1	9	4	4	4	558	61.4	7.8
PSPC1	2	1	1	1	523	58.7	6.67
KRT6B	33	21	28	1	564	60	8
KRT6A	37	23	28	3	564	60	8
TMED10	3	1	1	1	219	25	7.44
KPRP	7	5	5	5	579	64.1	8.27
LRRC59	6	2	2	2	307	34.9	9.57
GLA	3	1	1	1	429	48.7	5.6
MIA3	2	4	4	4	1907	213.6	4.84
SCAMP3	5	1	1	1	347	38.3	7.64
SRP9	9	1	1	1	86	10.1	7.97
RPS6	8	2	2	2	249	28.7	10.84
OCIAD1	4	1	1	1	245	27.6	7.49
MRPL38	5	2	2	2	380	44.6	7.53
UQCRFS	3	1	1	1	283	30.8	8.87
DARS2	3	2	2	2	645	73.5	8.02
PLEC	1	5	5	5	4684	531.5	5.96
IKBIP	3	1	1	1	350	39.3	9.17
HMGN2	13	1	1	1	90	9.4	9.99
COX5B	9	2	2	2	129	13.7	8.81
DAZAP1	3	1	1	1	407	43.4	8.56
SNRPC	8	1	1	1	159	17.4	9.67
GANAB	7	7	7	7	944	106.8	6.14
PSMA5	4	1	1	1	241	26.4	4.79
FUBP3	14	7	7	5	572	61.6	8.38
NDUFV1	6	3	3	3	464	50.8	8.21
SSR4	14	2	3	2	173	19	6.15
SCO2	4	1	1	1	266	29.8	8.85
RPSA2	14	3	3	3	295	32.9	4.87
SLC7A5	3	1	1	1	507	55	7.72
RHOT2	1	1	1	1	618	68.1	5.86
RPL23	7	1	1	1	140	14.9	10.51
MRPL58	6	1	1	1	206	23.6	10.07
NDUFS6	10	1	1	1	124	13.7	8.28
RTCB	13	6	7	6	505	55.2	7.23
GRSF1	6	3	3	3	480	53.1	6.19
NASP	2	2	2	2	788	85.2	4.3
RPN2	5	2	2	2	631	69.2	5.69
P4HB	30	16	17	16	508	57.1	4.87
PSIP1	6	3	3	3	530	60.1	9.13
NOP16	4	1	1	1	178	21.2	9.94
PHB2	33	10	10	10	299	33.3	9.83
NAPA	7	2	2	2	295	33.2	5.36
CCDC86	4	1	1	1	360	40.2	10.33
IARS2	2	2	2	2	1012	113.7	7.2
VDAC2	21	5	5	5	294	31.5	7.56
LETM1	3	2	2	2	739	83.3	6.7
SERBP1	24	8	11	8	408	44.9	8.65
MRRF	3	1	1	1	262	29.3	9.79
KRT80	7	3	3	2	452	50.5	5.67
PRDX5	5	1	2	1	214	22.1	8.7
MDH2	45	12	15	12	338	35.5	8.68
SLC25A	23	7	9	7	298	32.8	9.69
HSP90AI	22	13	16	6	724	83.2	5.03
TKT	1	1	1	1	623	67.8	7.66
TIMM8A	11	1	1	1	97	11	5.16
IK	2	1	1	1	557	65.6	6.64
PABPC4	2	2	2	2	644	70.7	9.26
ATP1A1	5	5	5	5	1023	112.8	5.49
PIP	8	1	1	1	146	16.6	8.05
FLG2	1	1	1	1	2391	247.9	8.31
TPX2	1	1	1	1	747	85.6	9.23
NDUFB1	7	1	1	1	153	17.3	5.22
NACA	3	5	5	5	2078	205.3	9.58
TRIM28	3	2	2	2	835	88.5	5.77
DSG1	11	9	9	9	1049	113.7	5.03
RPL19	5	1	1	1	196	23.5	11.47
GFM1	4	3	3	3	751	83.4	7.01
RANGAI	2	1	1	1	587	63.5	4.68
CIRBP	22	4	5	4	172	18.6	9.51
KRT18	6	3	5	2	430	48	5.45
ZC3H15	2	1	1	1	426	48.6	5.31
GNB1	8	3	3	1	340	37.4	6

CAPRIN1	2	2	2	2	709	78.3	5.25
POLDIP3	3	1	1	1	421	46.1	9.99
VAPA	5	1	1	1	249	27.9	8.62
LAMB1	1	1	1	1	1786	197.9	4.94
PHGDH	8	4	4	4	533	56.6	6.71
ECI1	14	4	5	4	302	32.8	8.54
HYOU1	9	9	10	9	999	111.3	5.22
IGHG2	5	2	2	2	326	35.9	7.59
TRMT10C	3	1	1	1	403	47.3	9.36
LBR	3	2	2	2	615	70.7	9.36
ANXA11	2	1	1	1	505	54.4	7.65
PSMB6	5	1	1	1	239	25.3	4.92
EMG1	4	1	1	1	244	26.7	9.17
SNRPD2	16	2	2	2	118	13.5	9.91
GCSH	6	1	1	1	173	18.9	4.88
PSMB1	4	1	1	1	241	26.5	8.13
ESYT1	1	1	1	1	1104	122.8	5.83
CDK1	3	1	1	1	297	34.1	8.4
RBMX	23	10	12	10	391	42.3	10.05
MYH10	5	9	9	7	1976	228.9	5.54
SHMT2	22	10	11	10	504	56	8.53
RPL36	10	1	1	1	105	12.2	11.59
COQ9	3	1	1	1	318	35.5	5.94
MT-CO2	4	1	1	1	227	25.5	4.82
EMD	4	1	1	1	254	29	5.5
TUFM	29	11	12	11	455	49.8	7.61
RPS8	24	4	4	4	208	24.2	10.32
RBM8A	11	2	3	2	174	19.9	5.72
TRA2A	6	2	2	1	282	32.7	11.27
TOMM22	8	1	1	1	142	15.5	4.34
NAP1L1	5	2	2	2	391	45.3	4.46
PYCR1	13	3	3	3	319	33.3	7.61
NOL12	6	1	1	1	213	24.6	10.23
TRPM4	1	1	1	1	1214	134.2	8.15
S100A8	31	3	3	3	93	10.8	7.03
RPL4	4	2	2	2	427	47.7	11.06
NUP54	3	1	1	1	507	55.4	7.02
ALDH18A	6	5	6	5	795	87.2	7.12
PNPT1	2	2	2	2	783	85.9	7.77
ZNF326	2	1	1	1	582	65.6	5.15
MTDH	3	2	2	2	582	63.8	9.32
RPS18	6	1	1	1	152	17.7	10.99
POR	2	1	1	1	677	76.6	5.58
GLUD1	9	4	4	4	558	61.4	7.8
PSPC1	2	1	1	1	523	58.7	6.67
TMED10	3	1	1	1	219	25	7.44
LRRC59	6	2	2	2	307	34.9	9.57
GLA	3	1	1	1	429	48.7	5.6
MIA3	2	4	4	4	1907	213.6	4.84
SCAMP3	5	1	1	1	347	38.3	7.64
SRP9	9	1	1	1	86	10.1	7.97
RPS6	8	2	2	2	249	28.7	10.84
OCIAD1	4	1	1	1	245	27.6	7.49
MRPL38	5	2	2	2	380	44.6	7.53
UQCRFS1	3	1	1	1	283	30.8	8.87
DARS2	3	2	2	2	645	73.5	8.02
PLEC	1	5	5	5	4684	531.5	5.96
IKBIP	3	1	1	1	350	39.3	9.17
HMGN2	13	1	1	1	90	9.4	9.99
COX5B	9	2	2	2	129	13.7	8.81
DAZAP1	3	1	1	1	407	43.4	8.56
SNRPC	8	1	1	1	159	17.4	9.67
GANAB	7	7	7	7	944	106.8	6.14
PSMA5	4	1	1	1	241	26.4	4.79
FUBP3	14	7	7	5	572	61.6	8.38
NDUFV1	6	3	3	3	464	50.8	8.21
SSR4	14	2	3	2	173	19	6.15
SCO2	4	1	1	1	266	29.8	8.85
RPSA2	14	3	3	3	295	32.9	4.87
SLC7A5	3	1	1	1	507	55	7.72
RHOT2	1	1	1	1	618	68.1	5.86
RPL23	7	1	1	1	140	14.9	10.51
MRPL58	6	1	1	1	206	23.6	10.07
NDUFS6	10	1	1	1	124	13.7	8.28
RTCB	13	6	7	6	505	55.2	7.23
GRSF1	6	3	3	3	480	53.1	6.19
NASP	2	2	2	2	788	85.2	4.3
RPN2	5	2	2	2	631	69.2	5.69
P4HB	30	16	17	16	508	57.1	4.87
PSIP1	6	3	3	3	530	60.1	9.13
NOP16	4	1	1	1	178	21.2	9.94
NAPA	7	2	2	2	295	33.2	5.36
CCDC86	4	1	1	1	360	40.2	10.33
IARS2	2	2	2	2	1012	113.7	7.2
VDAC2	21	5	5	5	294	31.5	7.56
LETM1	3	2	2	2	739	83.3	6.7
SERBP1	24	8	11	8	408	44.9	8.65
MRRF	3	1	1	1	262	29.3	9.79
PRDX5	5	1	2	1	214	22.1	8.7
MDH2	45	12	15	12	338	35.5	8.68
HSP90AB	22	13	16	6	724	83.2	5.03
TKT	1	1	1	1	623	67.8	7.66
TIMM8A	11	1	1	1	97	11	5.16
IK	2	1	1	1	557	65.6	6.64
PABPC4	2	2	2	2	644	70.7	9.26
ATP1A1	5	5	5	5	1023	112.8	5.49
FLG2	1	1	1	1	2391	247.9	8.31
TPX2	1	1	1	1	747	85.6	9.23
NDUFB11	7	1	1	1	153	17.3	5.22
NACA	3	5	5	5	2078	205.3	9.58
TRIM28	3	2	2	2	835	88.5	5.77
RPL19	5	1	1	1	196	23.5	11.47
GFM1	4	3	3	3	751	83.4	7.01
RANGAP	2	1	1	1	587	63.5	4.68
ZC3H15	2	1	1	1	426	48.6	5.31
GNB1	8	3	3	1	340	37.4	6
RBM15	1	1	1	1	977	107.1	10.08
RPS11	15	4	4	4	158	18.4	10.3
EIF4A1	4	1	2	1	406	46.1	5.48
COX5A	6	1	1	1	150	16.8	6.79
CFL1	28	4	4	4	166	18.5	8.09
TRAP1	17	12	12	11	704	80.1	8.21
HSPE1	68	7	9	7	102	10.9	8.92
RPS27	24	3	3	3	84	9.5	9.45
PPA2	7	2	2	2	334	37.9	7.39
PPP1CC	8	3	3	3	323	37	6.54
ATP5PD	11	2	2	2	161	18.5	5.3
MRPL2	4	1	1	1	305	33.3	11.3
RCN2	3	1	1	1	317	36.9	4.4
RPL13A	4	1	1	1	203	23.6	10.93
PGK1	5	2	2	2	417	44.6	8.1
HSD17B4	6	4	4	4	736	79.6	8.84
ALDH9A	2	1	1	1	494	53.8	5.87
LRPPRC	2	3	3	3	1394	157.8	6.13
HMGN1	34	3	3	3	100	10.7	9.6
CAPZB	3	1	1	1	272	30.6	6
VDAC1	16	3	3	3	283	30.8	8.54
HNRNPH	24	8	9	8	449	49.2	6.3
CCT8	2	1	1	1	548	59.6	5.6
PRDX3	14	4	5	4	256	27.7	7.78
FAM162A	7	1	1	1	154	17.3	9.77
PARP1	11	9	10	9	1014	113	8.88
SLC3A2	10	6	6	6	630	68	5.01
AZGP1	7	2	2	2	298	34.2	6.05
THRAP3	12	11	11	11	955	108.6	10.15

RBM15	1	1	1	1	977	107.1	10.08
RPS11	15	4	4	4	158	18.4	10.3
EIF4A1	4	1	2	1	406	46.1	5.48
COX5A	6	1	1	1	150	16.8	6.79
CFL1	28	4	4	4	166	18.5	8.09
TRAP1	17	12	12	11	704	80.1	8.21
DSP	7	22	23	22	2871	331.6	6.81
HSPE1	68	7	9	7	102	10.9	8.92
RPS27	24	3	3	3	84	9.5	9.45
PPA2	7	2	2	2	334	37.9	7.39
PPP1CC	8	3	3	3	323	37	6.54
ATP5PD	11	2	2	2	161	18.5	5.3
MRPL2	4	1	1	1	305	33.3	11.3
RCN2	3	1	1	1	317	36.9	4.4
RPL13A	4	1	1	1	203	23.6	10.93
PGK1	5	2	2	2	417	44.6	8.1
HSD17B4	6	4	4	4	736	79.6	8.84
ALDH9A	2	1	1	1	494	53.8	5.87
LRPPRC	2	3	3	3	1394	157.8	6.13
HMGN1	34	3	3	3	100	10.7	9.6
CAPZB	3	1	1	1	272	30.6	6
VDAC1	16	3	3	3	283	30.8	8.54
HNRNPH	24	8	9	8	449	49.2	6.3
CCT8	2	1	1	1	548	59.6	5.6
PRDX3	14	4	5	4	256	27.7	7.78
FAM162	7	1	1	1	154	17.3	9.77
PARP1	11	9	10	9	1014	113	8.88
SLC3A2	10	6	6	6	630	68	5.01
AZGP1	7	2	2	2	298	34.2	6.05
MKI67	1	1	1	1	3256	358.5	9.45
THRAP3	12	11	11	11	955	108.6	10.15
HSPA8	30	17	23	11	646	70.9	5.52
ERAL1	2	1	1	1	437	48.3	8.84
GSN	1	1	1	1	782	85.6	6.28
GOLGB1	0	1	1	1	3259	375.8	5
SEC22B	11	2	2	2	215	24.7	8.51
RPL9	5	1	1	1	192	21.9	9.95
MRPL9	3	1	1	1	267	30.2	10.08
H2BC12	23	3	3	3	126	13.9	10.32
HSPA4	3	2	2	2	840	94.3	5.19
HMGA1	23	2	2	2	107	11.7	10.32
RPL28	5	1	1	1	137	15.7	12.02
CSTF2T	2	1	1	1	616	64.4	7.25
RPL18	13	2	2	2	188	21.6	11.72
SYNCRIP	16	9	9	4	623	69.6	8.59
PEBP1	4	1	1	1	187	21	7.53
SNU13	9	1	1	1	128	14.2	8.46
EEF1A1	18	6	7	2	462	50.1	9.01
MRPL16	4	1	1	1	251	28.4	10.13
NDUFS2	3	2	2	2	463	52.5	7.55
GOLM1	4	2	2	2	401	45.3	4.97
PSMA3	5	1	1	1	255	28.4	5.33
HNRNPU	22	18	26	18	825	90.5	6
LDHB	9	3	3	2	334	36.6	6.05
MCCC2	2	1	1	1	563	61.3	7.68
PLD3	2	1	1	1	490	54.7	6.47
SEC61B	10	1	1	1	96	10	11.56
PKP1	2	1	1	1	747	82.8	9.13
SRSF8	5	2	2	2	282	32.3	11.72
PRMT1	4	1	1	1	371	42.4	5.35
SUB1	9	1	1	1	127	14.4	9.6
DDX1	8	6	6	6	740	82.4	7.23
SPTAN1	1	2	2	2	2472	284.4	5.35
RAB7A	9	2	2	2	207	23.5	6.7
GSTK1	4	1	1	1	226	25.5	8.41
MYDGF	9	2	2	2	173	18.8	6.68
BRD4	1	1	1	1	1362	152.1	9.19
UHRF1	1	1	1	1	793	89.8	7.56
MAZ	2	1	1	1	477	48.6	8.95
PHB1	35	9	10	9	272	29.8	5.76
NDUFS3	5	1	1	1	264	30.2	7.5
HSP90AB	4	2	2	1	505	58.2	4.73
EMC8	5	1	1	1	210	23.8	6.4
SDHA	9	6	6	6	664	72.6	7.39
NUP35	4	1	1	1	326	34.8	9.09
MRPL12	11	2	2	2	198	21.3	8.87
RPS3	30	8	8	8	243	26.7	9.66
SSR1	3	1	1	1	286	32.2	4.49
PCYOX1	2	1	1	1	505	56.6	6.18
FUS	11	7	9	5	526	53.4	9.36
CCDC124	9	2	3	2	223	25.8	9.54
SRSF9	10	2	3	2	221	25.5	8.65
ANXA2	31	10	10	10	339	38.6	7.75
RBM25	2	2	2	2	843	100.1	6.32
GPATCH	2	1	1	1	446	50.4	9.63
SF3A3	2	1	1	1	501	58.8	5.38
PMPCA	5	2	2	2	525	58.2	6.92
HADHA	13	9	9	9	763	82.9	9.04
MRPS7	5	1	1	1	242	28.1	9.99
PCBP1	7	2	2	2	356	37.5	7.09
LMNA	27	17	18	16	664	74.1	7.02
YBX1	13	3	4	3	324	35.9	9.88
GATC	8	1	1	1	136	15.1	5.05
KHSRP	15	8	8	8	711	73.1	7.3
CWC15	8	2	2	2	229	26.6	5.71
SNW1	2	1	1	1	536	61.5	9.52
TMPO	8	3	3	3	454	50.6	9.38
GAPDH	19	6	6	6	335	36	8.46
YWHAG	11	3	3	1	247	28.3	4.89
HMGN4	10	1	1	1	90	9.5	10.48
ILF2	3	1	1	1	390	43	5.26
DBN1	10	4	4	4	649	71.4	4.45
UQCRC2	12	4	5	4	453	48.4	8.63
HSPA5	41	24	31	22	654	72.3	5.16
SARNP	9	2	2	2	210	23.7	6.42
MRPS26	9	2	2	2	205	24.2	10.39
DAD1	11	1	1	1	113	12.5	7.08
HNRNPC	32	10	12	10	306	33.7	5.08
SERPINE	2	1	1	1	405	46.2	5.53
JUP	10	7	7	7	745	81.7	6.14
MTHFD1	1	1	1	1	978	105.7	8.06
GLS	3	2	2	2	669	73.4	7.77
TXNDC5	16	6	6	6	432	47.6	5.97
RPL11	8	1	2	1	178	20.2	9.6
MATR3	14	11	11	11	847	94.6	6.25
MRPS28	5	1	1	1	187	20.8	9.1
RUVBL1	3	1	1	1	456	50.2	6.42
SAFB2	1	2	2	1	953	107.4	6.16
PGRMC1	9	2	2	2	195	21.7	4.7
EIF2S3	6	2	2	2	472	51.1	8.4
TIMM9	12	1	1	1	89	10.4	7.21
EIF4B	1	1	1	1	611	69.1	5.73
RPL38	50	4	4	4	70	8.2	10.1
HNRNPA	50	16	20	15	353	37.4	8.95
PTGES3	5	1	1	1	160	18.7	4.54
ECH1	8	3	3	3	328	35.8	8
FAM136	6	1	1	1	138	15.6	7.61
RPL27	28	3	4	3	136	15.8	10.56
CBX5	6	1	1	1	191	22.2	5.86
SEC63	1	1	1	1	760	87.9	5.31
ALDH2	10	5	5	4	517	56.3	7.05
TOMM70	6	4	4	4	608	67.4	7.12
RPL7A	15	4	4	4	266	30	10.61
ERH	17	2	3	2	104	12.3	5.92
POFUT1	3	1	1	1	388	43.9	8.53

ERAL1	2	1	1	1	437	48.3	8.84
GSN	1	1	1	1	782	85.6	6.28
GOLGB1	0	1	1	1	3259	375.8	5
SEC22B	11	2	2	2	215	24.7	8.51
RPL9	5	1	1	1	192	21.9	9.95
MRPL9	3	1	1	1	267	30.2	10.08
HSPA4	3	2	2	2	840	94.3	5.19
HMGA1	23	2	2	2	107	11.7	10.32
RPL28	5	1	1	1	137	15.7	12.02
CSTF2T	2	1	1	1	616	64.4	7.25
RPL18	13	2	2	2	188	21.6	11.72
SYNCRIP	16	9	9	4	623	69.6	8.59
PEBP1	4	1	1	1	187	21	7.53
SNU13	9	1	1	1	128	14.2	8.46
MRPL16	4	1	1	1	251	28.4	10.13
NDUFS2	3	2	2	2	463	52.5	7.55
GOLM1	4	2	2	2	401	45.3	4.97
PSMA3	5	1	1	1	255	28.4	5.33
LDHB	9	3	3	2	334	36.6	6.05
MCCC2	2	1	1	1	563	61.3	7.68
PLD3	2	1	1	1	490	54.7	6.47
SEC61B	10	1	1	1	96	10	11.56
SRSF8	5	2	2	2	282	32.3	11.72
PRMT1	4	1	1	1	371	42.4	5.35
SUB1	9	1	1	1	127	14.4	9.6
DDX1	8	6	6	6	740	82.4	7.23
SPTAN1	1	2	2	2	2472	284.4	5.35
RAB7A	9	2	2	2	207	23.5	6.7
GSTK1	4	1	1	1	226	25.5	8.41
MYDGF	9	2	2	2	173	18.8	6.68
BRD4	1	1	1	1	1362	152.1	9.19
UHRF1	1	1	1	1	793	89.8	7.56
MAZ	2	1	1	1	477	48.6	8.95
PHB1	35	9	10	9	272	29.8	5.76
NDUFS3	5	1	1	1	264	30.2	7.5
HSP90AB	4	2	2	1	505	58.2	4.73
EMC8	5	1	1	1	210	23.8	6.4
SDHA	9	6	6	6	664	72.6	7.39
NUP35	4	1	1	1	326	34.8	9.09
MRPL12	11	2	2	2	198	21.3	8.87
RPS3	30	8	8	8	243	26.7	9.66
SSR1	3	1	1	1	286	32.2	4.49
PCYOX1	2	1	1	1	505	56.6	6.18
FUS	11	7	9	5	526	53.4	9.36
CCDC124	9	2	3	2	223	25.8	9.54
SRSF9	10	2	3	2	221	25.5	8.65
RBM25	2	2	2	2	843	100.1	6.32
GPATCH4	2	1	1	1	446	50.4	9.63
SF3A3	2	1	1	1	501	58.8	5.38
PMPCA	5	2	2	2	525	58.2	6.92
HADHA	13	9	9	9	763	82.9	9.04
MRPS7	5	1	1	1	242	28.1	9.99
PCBP1	7	2	2	2	356	37.5	7.09
YBX1	13	3	4	3	324	35.9	9.88
GATC	8	1	1	1	136	15.1	5.05
KHSRP	15	8	8	8	711	73.1	7.3
CWC15	8	2	2	2	229	26.6	5.71
SNW1	2	1	1	1	536	61.5	9.52
TMPO	8	3	3	3	454	50.6	9.38
GAPDH	19	6	6	6	335	36	8.46
YWHAG	11	3	3	1	247	28.3	4.89
HMGN4	10	1	1	1	90	9.5	10.48
DBN1	10	4	4	4	649	71.4	4.45
UQCRC2	12	4	5	4	453	48.4	8.63
SARNP	9	2	2	2	210	23.7	6.42
MRPS26	9	2	2	2	205	24.2	10.39
DAD1	11	1	1	1	113	12.5	7.08
HNRNPC	32	10	12	10	306	33.7	5.08
MTHFD1L	1	1	1	1	978	105.7	8.06
GLS	3	2	2	2	669	73.4	7.77
TXNDC5	16	6	6	6	432	47.6	5.97
RPL11	8	1	2	1	178	20.2	9.6
MATR3	14	11	11	11	847	94.6	6.25
MRPS28	5	1	1	1	187	20.8	9.1
RUVBL1	3	1	1	1	456	50.2	6.42
SAFB2	1	2	2	1	953	107.4	6.16
PGRMC1	9	2	2	2	195	21.7	4.7
EIF2S3	6	2	2	2	472	51.1	8.4
TIMM9	12	1	1	1	89	10.4	7.21
EIF4B	1	1	1	1	611	69.1	5.73
RPL38	50	4	4	4	70	8.2	10.1
PTGES3	5	1	1	1	160	18.7	4.54
ECH1	8	3	3	3	328	35.8	8
FAM136A	6	1	1	1	138	15.6	7.61
RPL27	28	3	4	3	136	15.8	10.56
CBX5	6	1	1	1	191	22.2	5.86
SEC63	1	1	1	1	760	87.9	5.31
ALDH2	10	5	5	4	517	56.3	7.05
TOMM70	6	4	4	4	608	67.4	7.12
RPL7A	15	4	4	4	266	30	10.61
ERH	17	2	3	2	104	12.3	5.92
POFUT1	3	1	1	1	388	43.9	8.53
ERLIN2	12	4	4	4	339	37.8	5.62
LMNB2	24	15	17	13	620	69.9	5.59
ATP2A2	3	3	3	3	1042	114.7	5.34
RCC2	3	1	1	1	522	56	8.78
ARG1	6	2	2	2	322	34.7	7.21
SF1	2	1	1	1	639	68.3	8.98
CACYBP	3	1	1	1	228	26.2	8.25
PFN1	17	2	2	2	140	15	8.27
EIF2S1	8	3	3	3	315	36.1	5.08
PDIA3	31	14	17	14	505	56.7	6.35
TFAM	7	2	2	2	246	29.1	9.72
HNRNPH	10	3	3	3	346	36.9	6.87
HNRNPK	33	13	18	13	463	50.9	5.54
EEF1B2	10	2	2	2	225	24.7	4.67
WDR18	2	1	1	1	432	47.4	6.7
FAU	8	1	1	1	133	14.4	10.17
NDUFAF3	6	1	1	1	184	20.3	8.22
HIBCH	2	1	1	1	386	43.5	8.19
KHDRBS1	7	3	4	3	443	48.2	8.66
NOLC1	5	4	4	4	699	73.6	9.47
RPL30	27	2	2	2	115	12.8	9.63
SNRPB	12	3	4	3	240	24.6	11.19
UQCRH	16	2	2	2	91	10.7	4.44
MXRA7	9	2	2	2	204	21.5	4.26
DPM1	4	1	1	1	260	29.6	9.57
C1QBP	22	4	6	4	282	31.3	4.84
NEFM	4	4	4	3	916	102.4	4.91
RPL17	20	3	3	3	184	21.4	10.17
COX4I1	19	3	4	3	169	19.6	9.51
SUCLG2	3	1	1	1	432	46.5	6.39
SFPQ	22	15	19	14	707	76.1	9.44
SRSF6	9	4	4	4	344	39.6	11.43
RAB11B	9	2	2	2	218	24.5	5.94
ACAT1	12	4	4	4	427	45.2	8.85
DDX17	16	11	12	7	729	80.2	8.27
ZRANB2	3	1	1	1	330	37.4	10.01
RPL32	10	1	1	1	135	15.9	11.33
PRDX4	25	5	7	5	271	30.5	6.29
CALML5	5	1	1	1	146	15.9	4.44
EIF5A	18	3	4	3	154	16.8	5.24
XRCC6	9	5	5	5	609	69.8	6.64
HNRNPF	4	1	1	1	415	45.6	5.58
RPL21	9	1	1	1	160	18.6	10.49

ERLIN2	12	4	4	4	339	37.8	5.62
LMNB2	24	15	17	13	620	69.9	5.59
ATP2A2	3	3	3	3	1042	114.7	5.34
RCC2	3	1	1	1	522	56	8.78
ARG1	6	2	2	2	322	34.7	7.21
SF1	2	1	1	1	639	68.3	8.98
CACYBF	3	1	1	1	228	26.2	8.25
PFN1	17	2	2	2	140	15	8.27
EIF2S1	8	3	3	3	315	36.1	5.08
PDIA3	31	14	17	14	505	56.7	6.35
TFAM	7	2	2	2	246	29.1	9.72
HNRNPF	10	3	3	3	346	36.9	6.87
H4C1	41	4	4	4	103	11.4	11.36
HNRNPK	33	13	18	13	463	50.9	5.54
EEF1B2	10	2	2	2	225	24.7	4.67
WDR18	2	1	1	1	432	47.4	6.7
FAU	8	1	1	1	133	14.4	10.17
NDUFAF	6	1	1	1	184	20.3	8.22
HIBCH	2	1	1	1	386	43.5	8.19
KHDRBS	7	3	4	3	443	48.2	8.66
NOLC1	5	4	4	4	699	73.6	9.47
RPL30	27	2	2	2	115	12.8	9.63
SNRPB	12	3	4	3	240	24.6	11.19
UQCRH	16	2	2	2	91	10.7	4.44
MXRA7	9	2	2	2	204	21.5	4.26
DPM1	4	1	1	1	260	29.6	9.57
C1QBP	22	4	6	4	282	31.3	4.84
NEFM	4	4	4	3	916	102.4	4.91
RPL17	20	3	3	3	184	21.4	10.17
COX4I1	19	3	4	3	169	19.6	9.51
SUCLG2	3	1	1	1	432	46.5	6.39
SFPQ	22	15	19	14	707	76.1	9.44
SRSF6	9	4	4	4	344	39.6	11.43
RAB11B	9	2	2	2	218	24.5	5.94
ACAT1	12	4	4	4	427	45.2	8.85
DDX17	16	11	12	7	729	80.2	8.27
LYZ	5	1	1	1	148	16.5	9.16
ZRANB2	3	1	1	1	330	37.4	10.01
RPL32	10	1	1	1	135	15.9	11.33
PRDX4	25	5	7	5	271	30.5	6.29
CALML5	5	1	1	1	146	15.9	4.44
EIF5A	18	3	4	3	154	16.8	5.24
XRCC6	9	5	5	5	609	69.8	6.64
HNRNPF	4	1	1	1	415	45.6	5.58
PPIA	14	2	3	2	165	18	7.81
RPL21	9	1	1	1	160	18.6	10.49
RPL22	10	1	1	1	128	14.8	9.19
GTF2I	2	2	2	2	998	112.3	6.39
BCAP31	8	2	2	2	246	28	8.44
ATP5F1A	29	16	22	16	553	59.7	9.13
NDUFA1	6	1	1	1	144	16.7	8.43
UBA1	1	1	1	1	1058	117.8	5.76
H3C1	5	1	2	1	136	15.4	11.12
CANX	22	12	13	12	592	67.5	4.6
RPN1	13	7	7	7	607	68.5	6.38
VRK1	2	1	1	1	396	45.4	8.91
HNRNPL	6	3	4	3	589	64.1	8.22
ERO1A	3	1	1	1	468	54.4	5.68
ETFB	18	5	5	5	255	27.8	8.1
MRPS30	2	1	1	1	439	50.3	7.97
LDHA	7	2	2	1	332	36.7	8.27
PRKAR2	8	3	3	3	404	45.5	5.07
HSPA1A	24	14	16	10	641	70	5.66
COX6C	9	1	1	1	75	8.8	10.39
ALYREF	26	5	8	5	257	26.9	11.15
MRPL44	2	1	1	1	332	37.5	8.4
IRS4	2	2	2	2	1257	133.7	8.44
EMC1	1	1	1	1	993	111.7	7.66
TPI1	19	4	5	4	249	26.7	6.9
HSD17B	22	4	5	4	261	26.9	7.78
H1-4	26	6	7	6	219	21.9	11.03
MYH9	2	4	4	2	1960	226.4	5.6
SPCS2	12	3	3	3	226	25	8.47
CCT2	5	2	2	2	535	57.5	6.46
CALM1	28	4	4	4	149	16.8	4.22
RPL5	3	1	1	1	297	34.3	9.72
ETFA	9	2	2	2	333	35.1	8.38
NUP62	2	1	1	1	522	53.2	5.31
SACM1L	1	1	1	1	587	66.9	7.12
RPS4X	11	3	3	3	263	29.6	10.15
BCLAF1	13	11	12	11	920	106.1	9.98
HEXB	1	1	1	1	556	63.1	6.76
CLPX	6	3	3	3	633	69.2	7.58
ACADM	16	6	6	6	421	46.6	8.37
FLG	0	1	1	1	4061	434.9	9.25
MGME1	3	1	1	1	344	39.4	7.68
RPS14	15	2	2	2	151	16.3	10.05
U2AF2	6	2	2	2	475	53.5	9.09
CCT6A	1	1	1	1	531	58	6.68
SFXN1	14	4	4	4	322	35.6	9.07
RPS25	7	1	1	1	125	13.7	10.11
ATAD3B	8	7	7	2	648	72.5	9.2
DHCR24	2	1	1	1	516	60.1	8.16
RPL24	25	4	4	4	157	17.8	11.25
MRM3	2	1	1	1	420	47	8.73
DLAT	4	3	3	3	647	69	7.84
MRPL57	13	1	1	1	102	12.3	11.44
IDH2	4	2	2	2	452	50.9	8.69
RTN3	1	1	1	1	1032	112.5	4.96
ACTN4	5	5	5	5	911	104.8	5.44
OXA1L	2	1	1	1	435	48.5	9.45
CYB5A	10	1	1	1	134	15.3	4.96
PTBP1	3	2	2	2	557	59.6	9.16
TSFM	2	1	1	1	325	35.4	8.38
SNRPF	9	1	1	1	86	9.7	4.67
NDUFAF	2	1	1	1	327	37.7	7.64
HMGB1	31	7	10	6	215	24.9	5.74
NCL	17	14	14	14	710	76.6	4.7
PLCH1	0	1	1	1	1693	189.1	7.74
TMEM1(	3	1	1	1	274	31.1	6.99
ATP5F1C	11	3	4	3	298	33	9.22
ATP5F1E	16	1	1	1	51	5.8	9.92
TAF15	20	8	11	6	592	61.8	8.02
EEF2	3	3	3	2	858	95.3	6.83
CHCHD3	11	3	3	3	227	26.1	8.28
RPS2	3	1	1	1	293	31.3	10.24
STIP1	8	5	5	5	543	62.6	6.8
MYL6	19	3	3	3	151	16.9	4.65
PA2G4	9	3	3	3	394	43.8	6.55
H2AX	11	2	2	1	143	15.1	10.74
RPS3A	17	4	4	4	264	29.9	9.73
RPL26	17	4	4	4	145	17.2	10.55
HNRNPD	15	5	6	4	355	38.4	7.81
HLA-A	2	1	1	1	365	40.8	6
TIMM50	2	1	1	1	353	39.6	8.37
PRDX1	21	4	4	4	199	22.1	8.13
MRPS36	12	1	1	1	103	11.5	9.99
PDHB	13	4	4	4	359	39.2	6.65
HADHB	10	5	5	5	474	51.3	9.41
RPS5	4	1	1	1	204	22.9	9.72
SRSF1	17	4	6	4	248	27.7	10.36
HNRNPD	14	6	8	5	420	46.4	9.57
HDAC2	2	1	1	1	488	55.3	5.91
LIN7C	6	1	1	1	197	21.8	8.43
ADAR	1	1	1	1	1226	136	8.65

RPL22	10	1	1	1	128	14.8	9.19
GTF2I	2	2	2	2	998	112.3	6.39
BCAP31	8	2	2	2	246	28	8.44
NDUFA13	6	1	1	1	144	16.7	8.43
UBA1	1	1	1	1	1058	117.8	5.76
H3C1	5	1	2	1	136	15.4	11.12
CANX	22	12	13	12	592	67.5	4.6
RPN1	13	7	7	7	607	68.5	6.38
VRK1	2	1	1	1	396	45.4	8.91
HNRNPL	6	3	4	3	589	64.1	8.22
ERO1A	3	1	1	1	468	54.4	5.68
ETFB	18	5	5	5	255	27.8	8.1
MRPS30	2	1	1	1	439	50.3	7.97
LDHA	7	2	2	1	332	36.7	8.27
PRKAR2A	8	3	3	3	404	45.5	5.07
HSPA1A	24	14	16	10	641	70	5.66
COX6C	9	1	1	1	75	8.8	10.39
ALYREF	26	5	8	5	257	26.9	11.15
MRPL44	2	1	1	1	332	37.5	8.4
IRS4	2	2	2	2	1257	133.7	8.44
EMC1	1	1	1	1	993	111.7	7.66
TPI1	19	4	5	4	249	26.7	6.9
HSD17B1	22	4	5	4	261	26.9	7.78
H1-4	26	6	7	6	219	21.9	11.03
SPCS2	12	3	3	3	226	25	8.47
CCT2	5	2	2	2	535	57.5	6.46
CALM1	28	4	4	4	149	16.8	4.22
RPL5	3	1	1	1	297	34.3	9.72
ETFA	9	2	2	2	333	35.1	8.38
NUP62	2	1	1	1	522	53.2	5.31
SACM1L	1	1	1	1	587	66.9	7.12
HEXB	1	1	1	1	556	63.1	6.76
CLPX	6	3	3	3	633	69.2	7.58
ACADM	16	6	6	6	421	46.6	8.37
FLG	0	1	1	1	4061	434.9	9.25
MGME1	3	1	1	1	344	39.4	7.68
RPS14	15	2	2	2	151	16.3	10.05
U2AF2	6	2	2	2	475	53.5	9.09
CCT6A	1	1	1	1	531	58	6.68
SFXN1	14	4	4	4	322	35.6	9.07
RPS25	7	1	1	1	125	13.7	10.11
ATAD3B	8	7	7	2	648	72.5	9.2
DHCR24	2	1	1	1	516	60.1	8.16
RPL24	25	4	4	4	157	17.8	11.25
MRM3	2	1	1	1	420	47	8.73
DLAT	4	3	3	3	647	69	7.84
MRPL57	13	1	1	1	102	12.3	11.44
IDH2	4	2	2	2	452	50.9	8.69
RTN3	1	1	1	1	1032	112.5	4.96
ACTN4	5	5	5	5	911	104.8	5.44
OXA1L	2	1	1	1	435	48.5	9.45
CYB5A	10	1	1	1	134	15.3	4.96
PTBP1	3	2	2	2	557	59.6	9.16
TSFM	2	1	1	1	325	35.4	8.38
SNRPF	9	1	1	1	86	9.7	4.67
NDUFAF1	2	1	1	1	327	37.7	7.64
HMGB1	31	7	10	6	215	24.9	5.74
PLCH1	0	1	1	1	1693	189.1	7.74
TMEM10(	3	1	1	1	274	31.1	6.99
ATP5F1C	11	3	4	3	298	33	9.22
ATP5F1E	16	1	1	1	51	5.8	9.92
EEF2	3	3	3	2	858	95.3	6.83
CHCHD3	11	3	3	3	227	26.1	8.28
RPS2	3	1	1	1	293	31.3	10.24
STIP1	8	5	5	5	543	62.6	6.8
MYL6	19	3	3	3	151	16.9	4.65
PA2G4	9	3	3	3	394	43.8	6.55
RPS3A	17	4	4	4	264	29.9	9.73
RPL26	17	4	4	4	145	17.2	10.55
HNRNPD	15	5	6	4	355	38.4	7.81
HLA-A	2	1	1	1	365	40.8	6
TIMM50	2	1	1	1	353	39.6	8.37
PRDX1	21	4	4	4	199	22.1	8.13
MRPS36	12	1	1	1	103	11.5	9.99
PDHB	13	4	4	4	359	39.2	6.65
HADHB	10	5	5	5	474	51.3	9.41
RPS5	4	1	1	1	204	22.9	9.72
SRSF1	17	4	6	4	248	27.7	10.36
HDAC2	2	1	1	1	488	55.3	5.91
LIN7C	6	1	1	1	197	21.8	8.43
ADAR	1	1	1	1	1226	136	8.65
KCTD5	7	1	1	1	234	26.1	6.24
STOM	7	2	2	2	288	31.7	7.88
TACO1	4	1	1	1	297	32.5	8.13
TMA7B	14	1	1	1	64	7.1	9.99
EPB41L2	1	1	1	1	1005	112.5	5.44
RPL22L1	10	1	1	1	122	14.6	9.38
APMAP	2	1	1	1	416	46.5	6.16
CLPP	3	1	1	1	277	30.2	8.09
VDAC3	7	2	2	2	283	30.6	8.66
YARS1	2	1	1	1	528	59.1	7.05
ERP44	7	3	3	3	406	46.9	5.26
AHCY	3	1	1	1	432	47.7	6.34
MTFR1	3	1	1	1	333	37	9.01
MRPS2	3	1	1	1	296	33.2	9.26
UQCRC1	17	7	8	6	480	52.6	6.37
ATP5IF1	17	2	2	2	106	12.2	9.35
DARS1	4	2	2	2	501	57.1	6.55
NUP153	1	2	2	2	1475	153.8	8.73
ATP5PO	10	2	2	2	213	23.3	9.96
NPM1	23	5	8	5	294	32.6	4.78
CLTC	1	2	2	2	1675	191.5	5.69
C1orf167	1	1	1	1	1468	162.3	10.7
MMTAG2	3	1	1	1	263	29.4	10.02
DCD	10	1	1	1	110	11.3	6.54
SRSF10	13	3	3	3	262	31.3	11.27
GOLIM4	2	1	1	1	696	81.8	4.77
MRPS31	2	1	1	1	395	45.3	9.29
CKMT1A	9	4	4	4	417	47	8.34
EIF4A3	5	2	2	2	411	46.8	6.73
SLIRP	17	2	2	2	109	12.3	10.24
NDUFB4	8	1	1	1	129	15.2	9.85
NDUFB10	20	3	3	3	172	20.8	8.48
CRELD2	2	1	1	1	353	38.2	4.59
PDCL2	4	1	1	1	241	28.1	4.87
FDXR	2	1	1	1	491	53.8	8.44
DDX5	23	14	16	10	614	69.1	8.92
SCP2	1	1	1	1	547	59	6.89
GRPEL1	17	3	3	3	217	24.3	8.12
PABPN1	9	2	2	2	306	32.7	5.06
NUDT21	4	1	1	1	227	26.2	8.82
FH	8	3	3	3	510	54.6	8.76
HSPA9	31	21	26	20	679	73.6	6.16
HSPD1	47	21	32	21	573	61	5.87
HDGFL2	3	2	2	2	671	74.3	7.49
SOD2	16	3	3	3	222	24.7	8.25
NENF	11	2	2	2	172	18.8	5.69
U2AF1	5	1	1	1	240	27.9	8.81
PGAM5	12	3	3	3	289	32	8.68
CYC1	4	1	2	1	325	35.4	9
CHMP1A	4	1	1	1	196	21.7	8.06
RPL23A	13	2	2	2	156	17.7	10.45
PNN	2	2	2	2	717	81.6	7.14
CYB5R3	8	2	2	2	301	34.2	7.59
PTGES2	3	1	1	1	377	41.9	9.16

KCTD5	7	1	1	1	234	26.1	6.24
STOM	7	2	2	2	288	31.7	7.88
TACO1	4	1	1	1	297	32.5	8.13
TUBA1B	27	9	10	9	451	50.1	5.06
TMA7B	14	1	1	1	64	7.1	9.99
EPB41L2	1	1	1	1	1005	112.5	5.44
RPL22L1	10	1	1	1	122	14.6	9.38
APMAP	2	1	1	1	416	46.5	6.16
CLPP	3	1	1	1	277	30.2	8.09
VDAC3	7	2	2	2	283	30.6	8.66
YARS1	2	1	1	1	528	59.1	7.05
ERP44	7	3	3	3	406	46.9	5.26
AHCY	3	1	1	1	432	47.7	6.34
MTFR1	3	1	1	1	333	37	9.01
MRPS2	3	1	1	1	296	33.2	9.26
UQCRC1	17	7	8	6	480	52.6	6.37
ATP5IF1	17	2	2	2	106	12.2	9.35
DARS1	4	2	2	2	501	57.1	6.55
NUP153	1	2	2	2	1475	153.8	8.73
ATP5PO	10	2	2	2	213	23.3	9.96
NPM1	23	5	8	5	294	32.6	4.78
CLTC	1	2	2	2	1675	191.5	5.69
C1orf167	1	1	1	1	1468	162.3	10.7
MMTAG	3	1	1	1	263	29.4	10.02
DCD	10	1	1	1	110	11.3	6.54
SRSF10	13	3	3	3	262	31.3	11.27
GOLIM4	2	1	1	1	696	81.8	4.77
MRPS31	2	1	1	1	395	45.3	9.29
CKMT1A	9	4	4	4	417	47	8.34
LTF	3	2	2	2	710	78.1	8.12
EIF4A3	5	2	2	2	411	46.8	6.73
SLIRP	17	2	2	2	109	12.3	10.24
NDUFB4	8	1	1	1	129	15.2	9.85
NDUFB1	20	3	3	3	172	20.8	8.48
CRELD2	2	1	1	1	353	38.2	4.59
PDCL2	4	1	1	1	241	28.1	4.87
FDXR	2	1	1	1	491	53.8	8.44
DDX5	23	14	16	10	614	69.1	8.92
SCP2	1	1	1	1	547	59	6.89
GRPEL1	17	3	3	3	217	24.3	8.12
PABPN1	9	2	2	2	306	32.7	5.06
NUDT21	4	1	1	1	227	26.2	8.82
FH	8	3	3	3	510	54.6	8.76
HSPA9	31	21	26	20	679	73.6	6.16
ENO1	18	6	7	6	434	47.1	7.39
HSPD1	47	21	32	21	573	61	5.87
HDGFL2	3	2	2	2	671	74.3	7.49
SOD2	16	3	3	3	222	24.7	8.25
KRT17	28	13	15	5	432	48.1	5.02
NENF	11	2	2	2	172	18.8	5.69
U2AF1	5	1	1	1	240	27.9	8.81
PGAM5	12	3	3	3	289	32	8.68
CYC1	4	1	2	1	325	35.4	9
CHMP1A	4	1	1	1	196	21.7	8.06
RPL23A	13	2	2	2	156	17.7	10.45
PNN	2	2	2	2	717	81.6	7.14
CYB5R3	8	2	2	2	301	34.2	7.59
PTGES2	3	1	1	1	377	41.9	9.16
PDIA6	22	8	8	8	440	48.1	5.08
XP32	3	1	1	1	250	26.2	7.97
RPS12	33	3	5	3	132	14.5	7.21
PRKDC	1	3	3	3	4128	468.8	7.12
TXN	21	2	2	2	105	11.7	4.92
SART1	1	1	1	1	800	90.2	6.13
CPOX	2	1	1	1	454	50.1	8.25
CALU	5	1	1	1	315	37.1	4.64
BSG	11	3	5	3	385	42.2	5.66
HM13	3	1	1	1	377	41.5	6.43
GOT2	22	8	9	8	430	47.5	9.01
PITRM1	2	2	2	2	1037	117.3	6.92
LSM7	8	1	1	1	103	11.6	5.27
APOO	6	1	1	1	198	22.3	9.13
UBTF	1	1	1	1	764	89.4	5.81
AGK	5	2	2	2	422	47.1	8.09
TM9SF4	1	1	1	1	642	74.5	6.54
MRPS27	2	1	1	1	414	47.6	6.18
RBM4	5	2	2	2	364	40.3	7.08
RPL36A	16	2	2	2	106	12.4	10.58
EMC2	3	1	1	1	297	34.8	6.57
NIPSNAI	3	1	1	1	284	33.3	9.31
TUBB	39	13	21	1	444	49.6	4.89
PKM	11	6	6	6	531	57.9	7.84
RBBP4	14	6	6	2	425	47.6	4.89
IMPDH2	4	2	2	2	514	55.8	6.9
RALY	5	2	2	2	306	32.4	9.17
RNPS1	5	1	1	1	305	34.2	11.84
CHD4	0	1	1	1	1912	217.9	5.86
DEK	15	5	5	5	375	42.6	8.56
RAN	19	4	4	4	216	24.4	7.49
TUBB4B	38	13	20	2	445	49.8	4.89
CHCHD1	9	1	1	1	118	13.5	10.21
ACO2	6	4	4	4	780	85.4	7.61
SERPINI	3	1	1	1	418	46.4	8.69
DDX39B	8	4	4	4	428	49	5.67
ACTC1	28	9	16	2	377	42	5.39
SRRT	2	2	2	2	876	100.6	5.96
MRPS18.	4	1	1	1	196	22.2	10.33
BRD2	3	2	2	2	801	88	9.09
EIF2S2	4	1	1	1	333	38.4	5.8
CCT3	3	2	2	2	545	60.5	6.49
ACTG1	43	13	22	6	375	41.8	5.48
PABPC1	10	6	6	6	636	70.6	9.5
GSDMA	2	1	1	1	445	49.3	5.29
DHX9	3	4	4	4	1270	140.9	6.84
ANXA5	7	2	2	2	320	35.9	5.05
KRT8	19	13	14	4	483	53.7	5.59
RPL10	4	1	1	1	214	24.6	10.08
NONO	33	16	18	15	471	54.2	8.95
APPL2	1	1	1	1	664	74.4	4.94
KRT78	14	8	8	5	520	56.8	6.02
CHTOP	10	2	2	2	248	26.4	12.23
FKBP1A	12	1	1	1	108	11.9	8.16
MRPS18	7	2	2	2	258	29.4	9.38
RPS15A	7	1	1	1	130	14.8	10.13
PMPCB	6	3	3	2	489	54.3	6.83
SF3B2	2	2	2	2	895	100.2	5.67
TFRC	2	1	1	1	760	84.8	6.61
PRPF40A	1	1	1	1	957	108.7	7.56
CCT5	3	2	2	2	541	59.6	5.66
FABP5	20	3	3	3	135	15.2	7.01
TPR	3	7	7	7	2363	267.1	5.02
PIN4	9	1	1	1	131	13.8	9.77
RACK1	22	5	5	5	317	35.1	7.69
MRPS23	11	2	2	2	190	21.8	8.9
PRKCSH	19	9	11	9	528	59.4	4.41
SND1	8	8	8	8	910	101.9	7.17
TPM3	3	1	1	1	285	32.9	4.72
PTMS	11	1	1	1	102	11.5	4.16
ITGB1	1	1	1	1	798	88.4	5.39
ATP5F1I	14	2	2	2	168	17.5	5.49
ABHD10	3	1	1	1	306	33.9	8.57
HNRNPU	11	7	8	7	747	85.1	4.91
KRT9	62	29	50	29	623	62	5.24
KRT16	43	19	23	10	473	51.2	5.05
PTMA	34	3	3	3	111	12.2	3.78
PDIA6	22	8	8	8	440	48.1	5.08
XP32	3	1	1	1	250	26.2	7.97
RPS12	33	3	5	3	132	14.5	7.21
PRKDC	1	3	3	3	4128	468.8	7.12
SART1	1	1	1	1	800	90.2	6.13
CPOX	2	1	1	1	454	50.1	8.25
CALU	5	1	1	1	315	37.1	4.64
BSG	11	3	5	3	385	42.2	5.66
HM13	3	1	1	1	377	41.5	6.43
GOT2	22	8	9	8	430	47.5	9.01
PITRM1	2	2	2	2	1037	117.3	6.92
LSM7	8	1	1	1	103	11.6	5.27
APOO	6	1	1	1	198	22.3	9.13
UBTF	1	1	1	1	764	89.4	5.81
AGK	5	2	2	2	422	47.1	8.09
TM9SF4	1	1	1	1	642	74.5	6.54
MRPS27	2	1	1	1	414	47.6	6.18
RBM4	5	2	2	2	364	40.3	7.08
RPL36A	16	2	2	2	106	12.4	10.58
EMC2	3	1	1	1	297	34.8	6.57
NIPSNAP	3	1	1	1	284	33.3	9.31
TUBB	39	13	21	1	444	49.6	4.89
PKM	11	6	6	6	531	57.9	7.84
RBBP4	14	6	6	2	425	47.6	4.89
IMPDH2	4	2	2	2	514	55.8	6.9
RALY	5	2	2	2	306	32.4	9.17
RNPS1	5	1	1	1	305	34.2	11.84
CHD4	0	1	1	1	1912	217.9	5.86
DEK	15	5	5	5	375	42.6	8.56
RAN	19	4	4	4	216	24.4	7.49
TUBB4B	38	13	20	2	445	49.8	4.89
CHCHD1	9	1	1	1	118	13.5	10.21
ACO2	6	4	4	4	780	85.4	7.61
SERPINH	3	1	1	1	418	46.4	8.69
DDX39B	8	4	4	4	428	49	5.67
ACTC1	28	9	16	2	377	42	5.39
SRRT	2	2	2	2	876	100.6	5.96
MRPS18A	4	1	1	1	196	22.2	10.33
BRD2	3	2	2	2	801	88	9.09
EIF2S2	4	1	1	1	333	38.4	5.8
CCT3	3	2	2	2	545	60.5	6.49
ACTG1	43	13	22	6	375	41.8	5.48
PABPC1	10	6	6	6	636	70.6	9.5
DHX9	3	4	4	4	1270	140.9	6.84
ANXA5	7	2	2	2	320	35.9	5.05
KRT8	19	13	14	4	483	53.7	5.59
RPL10	4	1	1	1	214	24.6	10.08
NONO	33	16	18	15	471	54.2	8.95
APPL2	1	1	1	1	664	74.4	4.94
CHTOP	10	2	2	2	248	26.4	12.23
FKBP1A	12	1	1	1	108	11.9	8.16
MRPS18B	7	2	2	2	258	29.4	9.38
RPS15A	7	1	1	1	130	14.8	10.13
PMPCB	6	3	3	2	489	54.3	6.83
SF3B2	2	2	2	2	895	100.2	5.67
TFRC	2	1	1	1	760	84.8	6.61
PRPF40A	1	1	1	1	957	108.7	7.56
CCT5	3	2	2	2	541	59.6	5.66
TPR	3	7	7	7	2363	267.1	5.02
PIN4	9	1	1	1	131	13.8	9.77
RACK1	22	5	5	5	317	35.1	7.69
MRPS23	11	2	2	2	190	21.8	8.9
PRKCSH	19	9	11	9	528	59.4	4.41
SND1	8	8	8	8	910	101.9	7.17
TPM3	3	1	1	1	285	32.9	4.72
PTMS	11	1	1	1	102	11.5	4.16
ITGB1	1	1	1	1	798	88.4	5.39
ATP5F1D	14	2	2	2	168	17.5	5.49
ABHD10	3	1	1	1	306	33.9	8.57
HNRNPU	11	7	8	7	747	85.1	4.91
MARCKS	22	5	5	5	332	31.5	4.45
ALDH1B1	10	6	6	5	517	57.2	6.99
YWHAQ	13	3	3	1	245	27.7	4.78
ZFP91	2	1	1	1	570	63.4	7.36
RCN1	8	2	2	2	331	38.9	5
RPLP2	22	2	2	2	115	11.7	4.54
M6PR	5	1	1	1	277	31	5.83
ATAD3A	10	7	7	2	634	71.3	8.98
GNB2	11	4	4	2	340	37.3	6
MRPL46	3	1	1	1	279	31.7	7.05
HMGN5	3	1	1	1	282	31.5	4.55
ATP5ME	25	2	2	2	69	7.9	9.35
CAT	6	3	3	3	527	59.7	7.39
LONP1	3	3	3	3	959	106.4	6.39
CRYAA	6	1	1	1	173	19.9	6.2
PSAP	5	3	3	3	524	58.1	5.17
EFTUD2	2	2	2	1	972	109.4	5
DHRS4L1	3	1	1	1	281	30.6	9.77
TMEM10	5	1	1	1	243	26.2	10.48
FBL	16	5	5	5	321	33.8	10.18
TAGLN2	6	1	1	1	199	22.4	8.25
TCERG1	1	1	1	1	1098	123.8	8.65
HIC2	2	1	1	1	615	66.1	6.38
RPS16	5	1	1	1	146	16.4	10.21
PARK7	7	1	1	1	189	19.9	6.79
OAT	8	3	3	3	439	48.5	7.03
SLC25A3	6	2	2	2	362	40.1	9.38
CALR	24	8	9	8	417	48.1	4.44
VN1R5	2	1	1	1	357	40.8	9.2
ELAVL1	17	6	6	6	326	36.1	9.17
RPS28	29	2	4	2	69	7.8	10.7
COX19	11	1	1	1	90	10.4	8.72
ACOT13	9	1	1	1	140	15	9.14
MRPL43	5	1	1	1	215	23.4	8.65
IDH3A	10	4	4	4	366	39.6	6.92
KTN1	1	1	1	1	1357	156.2	5.64
CLTA	6	2	2	2	248	27.1	4.51
FAM98A	2	1	1	1	518	55.2	8.95
RPL27A	20	3	3	3	148	16.6	11
EIF4H	5	1	1	1	248	27.4	7.23
DLD	18	9	9	9	509	54.1	7.85
NCOA5	2	1	1	1	579	65.5	9.6
STOML2	16	4	4	4	356	38.5	7.39
TRA2B	6	2	2	1	288	33.6	11.25
TCOF1	2	2	2	2	1488	152	9.04
BCAS2	5	1	1	1	225	26.1	5.66
TOMM40	3	1	1	1	361	37.9	7.25
IMMT	24	16	16	16	758	83.6	6.48
CHMP4C	3	1	1	1	233	26.4	6.07
SRSF5	3	1	1	1	272	31.2	11.59
TARDBP	2	1	1	1	414	44.7	6.19
MRPL37	2	1	1	1	423	48.1	8.59
RPL29	5	1	1	1	159	17.7	11.66
CYCS	18	2	2	2	105	11.7	9.57
ECHS1	13	4	4	4	290	31.4	8.07
PSMA4	3	1	1	1	261	29.5	7.72
NUCB1	2	1	1	1	461	53.8	5.25

MARCKS	22	5	5	5	332	31.5	4.45
ALDH1B	10	6	6	5	517	57.2	6.99
YWHAQ	13	3	3	1	245	27.7	4.78
ZFP91	2	1	1	1	570	63.4	7.36
RCN1	8	2	2	2	331	38.9	5
DSC1	4	3	3	3	894	99.9	5.43
RPLP2	22	2	2	2	115	11.7	4.54
M6PR	5	1	1	1	277	31	5.83
ATAD3A	10	7	7	2	634	71.3	8.98
GNB2	11	4	4	2	340	37.3	6
MRPL46	3	1	1	1	279	31.7	7.05
YWHAE	24	6	6	4	255	29.2	4.74
HMGN5	3	1	1	1	282	31.5	4.55
CTSD	2	1	1	1	412	44.5	6.54
ATP5ME	25	2	2	2	69	7.9	9.35
CAT	6	3	3	3	527	59.7	7.39
HNRNPA	41	14	21	13	372	38.7	9.13
LONP1	3	3	3	3	959	106.4	6.39
CRYAA	6	1	1	1	173	19.9	6.2
PSAP	5	3	3	3	524	58.1	5.17
EFTUD2	2	2	2	1	972	109.4	5
DHRS4L	3	1	1	1	281	30.6	9.77
TMEM10	5	1	1	1	243	26.2	10.48
FBL	16	5	5	5	321	33.8	10.18
TAGLN2	6	1	1	1	199	22.4	8.25
TCERG1	1	1	1	1	1098	123.8	8.65
HIC2	2	1	1	1	615	66.1	6.38
RPS16	5	1	1	1	146	16.4	10.21
PARK7	7	1	1	1	189	19.9	6.79
OAT	8	3	3	3	439	48.5	7.03
SLC25A3	6	2	2	2	362	40.1	9.38
CALR	24	8	9	8	417	48.1	4.44
VN1R5	2	1	1	1	357	40.8	9.2
ELAVL1	17	6	6	6	326	36.1	9.17
RPS28	29	2	4	2	69	7.8	10.7
COX19	11	1	1	1	90	10.4	8.72
ACOT13	9	1	1	1	140	15	9.14
MRPL43	5	1	1	1	215	23.4	8.65
IDH3A	10	4	4	4	366	39.6	6.92
KTN1	1	1	1	1	1357	156.2	5.64
KRT5	44	28	34	16	590	62.3	7.74
CLTA	6	2	2	2	248	27.1	4.51
FAM98A	2	1	1	1	518	55.2	8.95
RPL27A	20	3	3	3	148	16.6	11
EIF4H	5	1	1	1	248	27.4	7.23
DLD	18	9	9	9	509	54.1	7.85
NCOA5	2	1	1	1	579	65.5	9.6
STOML2	16	4	4	4	356	38.5	7.39
TRA2B	6	2	2	1	288	33.6	11.25
TCOF1	2	2	2	2	1488	152	9.04
BCAS2	5	1	1	1	225	26.1	5.66
RBM3	22	3	5	3	157	17.2	8.91
TOMM40	3	1	1	1	361	37.9	7.25
IMMT	24	16	16	16	758	83.6	6.48
CHMP4C	3	1	1	1	233	26.4	6.07
SRSF5	3	1	1	1	272	31.2	11.59
TARDBP	2	1	1	1	414	44.7	6.19
MRPL37	2	1	1	1	423	48.1	8.59
RPL29	5	1	1	1	159	17.7	11.66
CYCS	18	2	2	2	105	11.7	9.57
ECHS1	13	4	4	4	290	31.4	8.07
PSMA4	3	1	1	1	261	29.5	7.72
NUCB1	2	1	1	1	461	53.8	5.25
