# Supplementary material for: N6-methyladenosine-modified ALDH9A1 modulates lipid accumulation and tumor progression in clear cell renal cell carcinoma through the NPM1/IQGAP2/AKT signaling pathway
Source: Cell Death Dis. 2024 Jul 22;15(7):520. doi: 10.1038/s41419-024-06896-z (PMC11263707; doi:10.1038/s41419-024-06896-z)
Supplement: Supplementary file 6 — supplementary table 5 [file 41419_2024_6896_MOESM6_ESM.pdf]

Restricted IP/MS								
Gene Name	Descriptic	Coverage	# Peptide:	# PSMs	# Unique	# AAs	MW [kD:	calc. pI
ALDOA	Fructose-l	25	8	9	8	364	39.4	8.09
RBMX	RNA-binc	23	10	12	10	391	42.3	10.05
SHMT2	Serine hy	22	10	11	10	504	56	8.53
TUFM	Elongatio	29	11	12	11	455	49.8	7.61
P4HB	Protein di	30	16	17	16	508	57.1	4.87
VDAC2	Voltage-d	21	5	5	5	294	31.5	7.56
SERBP1	Plasminog	24	8	11	8	408	44.9	8.65
MDH2	Malate de	45	12	15	12	338	35.5	8.68
HSP90AB1	Heat shoc	22	13	16	6	724	83.2	5.03
HSPE1	10 kDa h	68	7	9	7	102	10.9	8.92
HNRNPH1	Heterogen	24	8	9	8	449	49.2	6.3
PHB1	Prohibitin	35	9	10	9	272	29.8	5.76
RPS3	40S ribos	30	8	8	8	243	26.7	9.66
HNRNPC	Heterogen	32	10	12	10	306	33.7	5.08
LMNB2	Lamin-B2	24	15	17	13	620	69.9	5.59
PDIA3	Protein di	31	14	17	14	505	56.7	6.35
HNRNPK	Heterogen	33	13	18	13	463	50.9	5.54
SFPQ	Splicing f	22	15	19	14	707	76.1	9.44
PRDX4	Peroxired	25	5	7	5	271	30.5	6.29
CANX	Calnexin	22	12	13	12	592	67.5	4.6
HSPA1A	Heat shoc	24	14	16	10	641	70	5.66
ALYREF	THO com	26	5	8	5	257	26.9	11.15
H1-4	Histone H	26	6	7	6	219	21.9	11.03
HMGB1	High mob	31	7	10	6	215	24.9	5.74
NPM1	Nucleoph	23	5	8	5	294	32.6	4.78
DDX5	Probable	23	14	16	10	614	69.1	8.92
HSPA9	Stress-70	31	21	26	20	679	73.6	6.16
HSPD1	60 kDa h	47	21	32	21	573	61	5.87
PDIA6	Protein di	22	8	8	8	440	48.1	5.08
GOT2	Aspartate	22	8	9	8	430	47.5	9.01
ACTG1	Actin, cyt	43	13	22	6	375	41.8	5.48
NONO	Non-POU	33	16	18	15	471	54.2	8.95
RACK1	Receptor	22	5	5	5	317	35.1	7.69
MARCKS	Myristoyl	22	5	5	5	332	31.5	4.45
CALR	Calreticul	24	8	9	8	417	48.1	4.44
IMMT	MICOS c	24	16	16	16	758	83.6	6.48

Geneset with prognosis value in ccRCC			
Gene Name	Mean	p_value	HR
A1BG	0.343682391	0.004606615	1.557019068
A1CF	18.95073182	1.29E-06	0.442160131
A2M	1104.128577	0.000991836	0.596144883
A2ML1	0.143280092	3.31E-05	1.950846801
A3GALT2	0.4480688	0.000799076	1.662000724
AAAS	38.18112207	6.06E-06	1.974133526
AAMP	143.4621757	0.000139437	0.561005733
AANAT	0.708117783	0.000117716	1.786601449
AARSD1	5.487436454	0.00017269	1.752651006
AASDH	10.19154365	0.000166795	0.546739603
AASDHPPT	35.23361438	0.004968985	0.64344871
ABAT	11.08920278	0.00202634	0.57976241
ABCA11P	1.589858708	0.001585442	1.605971669
ABCA13	0.075838251	0.000931362	1.754361466
ABCA17P	1.668184208	0.001924864	1.65789581
ABCA7	6.709564878	0.000249206	1.737073706
ABCB1	30.33081773	3.32E-05	0.494911966
ABCB10	23.88470829	0.001894478	0.615556836
ABCB7	35.96924205	2.26E-07	0.435531922
ABCB8	12.48558051	0.004213897	1.542405937
ABCB9	1.779838675	1.25E-05	1.929929298
ABCC10	8.655132983	0.000869177	1.655604314
ABCC12	0.016079382	0.002669559	1.752814439
ABCC13	0.040900623	0.000410021	1.77545008
ABCC4	41.22494484	2.29E-06	0.44755205
ABCC6	21.70677448	0.00405773	0.634033554
ABCD3	39.72100616	5.02E-05	0.51487361
ABCF1	60.64777883	0.003406786	0.63475478
ABCG1	51.82341733	0.000134026	0.545141509
ABCG2	19.26499343	5.92E-05	0.497393749
ABHD10	63.72631706	0.000407856	0.56918484
ABHD14A	19.77760913	0.004268313	1.543474687
ABHD16B	0	0.004606615	1.557019068
ABHD17AP	0.142516462	0.002169561	1.596576725
ABHD17AP	0	0.004606615	1.557019068
ABHD17B	24.37842581	1.16E-06	0.454070183
ABHD2	59.82044488	0.000301474	0.529276794
ABHD3	12.69060108	0.001515393	1.677883125
ABHD6	38.59809033	4.16E-05	0.517007838
ABI1	62.23493571	0.001605111	0.613469584
ABI1P1	0.055728882	0.000592977	1.708502567
ABLIM1	71.83860929	7.82E-05	0.529794089
ABRAXAS1	7.688810067	1.89E-08	0.373997288
ABRAXAS2	31.82858906	0.000233947	0.565955207
AC000068.2	1.138681449	0.001994464	1.596593591
AC000068.3	0	0.004606615	1.557019068
AC000095.1	0	0.004606615	1.557019068
AC000123.2	6.205184633	1.24E-05	1.928226139
AC001226.1	0.716258279	0.002006901	1.602483116
AC002044.1	2.124115075	0.003524883	1.572742159
AC002044.2	0.761770292	0.000246801	1.751026097
AC002059.1	2.175994541	0.000832774	1.65053269
AC002070.1	8.667499109	5.41E-05	0.517808283
AC002094.2	0	0.004606615	1.557019068
AC002094.5	0.607792051	0.000361176	1.71436918
AC002115.1	0.040832692	0.00084064	1.750393773
AC002128.1	2.744631488	0.000838957	1.648329074
AC002306.1	0.241979531	0.000429673	1.726069462
AC002310.3	0.284475954	0.004306335	1.54456852
AC002350.1	1.63375287	0.003983065	1.546144682
AC002351.1	0.300147914	2.46E-06	2.552673947
AC002386.1	0.007511442	0.000110637	3.453267907
AC002398.1	5.956326565	1.79E-10	2.560458546
AC002401.4	3.331503602	0.001880028	1.657513587
AC002451.1	2.1248291	2.14E-06	0.403362352
AC002454.1	0.837425831	6.37E-08	2.315552681
AC002456.1	1.675880611	1.12E-07	2.226540618
AC002463.1	0.019249681	0.001645802	1.798595653
AC002464.1	0.05398139	7.25E-05	1.830773295
AC002480.1	0.994569152	0.000227008	1.978949141
AC002511.2	2.959156291	0.001404334	1.688026717
AC002551.1	0.168939065	0.000343271	1.741227003
AC002553.1	5.769279396	9.78E-06	1.930871319
AC002553.2	4.116431623	1.57E-05	1.902940816
AC002558.1	0.485604508	2.71E-05	1.875714337
AC002558.2	0.206249658	0.001341407	1.635753259
AC002558.3	0.324301029	0.000918134	1.656822697
AC003001.2	0.042057389	0.002388909	1.656614905
AC003072.1	12.95000501	1.12E-08	2.327631731
AC003087.1	0.138718431	0.000556995	1.764120132
AC003092.1	4.059413268	9.02E-07	2.480541473
AC003092.2	0.308937016	1.27E-12	3.027604944
AC003101.2	0.98384386	0.000872743	1.671669273
AC003101.3	0	0.004606615	1.557019068
AC003102.1	3.717992438	1.59E-06	2.037376891
AC003659.1	0.055232608	0.000436909	1.895024489
AC003665.1	8.188345433	0.000397754	1.702022448
AC003965.1	1.13246227	3.46E-05	1.874160045
AC003975.1	0.021541392	0.000347295	1.904711465
AC003989.1	0.379099559	0.000850322	1.815595995
AC004000.1	1.210398191	1.57E-05	1.907583264
AC004012.1	0.28224548	7.44E-10	2.486051652
AC004014.2	0.026467359	0.000883829	1.712171412
AC004034.1	1.215878306	5.90E-06	1.957500824
AC004053.1	0.952893705	0.003053293	0.594551547
AC004066.2	0.118232075	0.000298569	0.491017284
AC004067.1	2.46311519	0.00086311	1.657437284
AC004076.2	1.119645819	0.004413161	1.533293599
AC004080.1	0.125272877	0.000759447	1.882305752
AC004080.2	0.347469073	0.000163835	1.815664871
AC004112.1	1.703732664	0.001151623	0.573009545
AC004129.2	0.113371809	0.004749865	1.558548295
AC004148.2	8.011447898	0.000962883	1.637387424
AC004223.2	0.461020887	1.79E-05	1.924473107
AC004232.1	0	0.004606615	1.557019068
AC004232.2	0	0.004606615	1.557019068
AC004241.1	0.772367569	0.00023423	1.73334066
AC004253.1	3.503389007	0.000250873	1.724486362
AC004264.1	4.150686011	0.000388731	1.770690019
AC004408.2	0.026186956	0.000182148	1.769824352
AC004448.1	0.016071051	0.001478895	2.028612862
AC004461.1	0.524871524	0.000201763	1.75709858
AC004466.1	0.765072152	8.04E-05	1.799683539
AC004477.1	0.980364144	7.68E-07	2.081883585
AC004477.2	0	0.004606615	1.557019068
AC004490.1	0.084882858	0.000533753	1.714283404
AC004494.2	0	0.004606615	1.557019068
AC004528.1	0.115987597	0.002040297	1.619322319
AC004528.2	0	0.004606615	1.557019068
AC004542.1	0	0.004606615	1.557019068
AC004542.2	0	0.004606615	1.557019068
AC004584.1	0.746715673	0.000179818	1.75333308
AC004584.3	0.132954763	0.000500346	1.691343233
AC004623.1	0.645055933	0.001065617	1.63479891
AC004672.2	0.139620923	0.000187582	1.789010134
AC004678.1	0.430443444	0.000579657	1.679045086
AC004692.2	0.01274948	0.000226922	2.0752305
AC004754.1	0.946334617	0.000325062	1.720437903
AC004771.1	2.265613212	1.61E-09	2.429834033
AC004775.1	0.118108349	0.000941379	1.660100606
AC004777.1	0.525792183	2.28E-10	2.526443805
AC004792.1	0	0.004606615	1.557019068
AC004797.1	0.119559191	0.0006068	1.688396213
AC004801.2	1.525580096	1.99E-06	2.018258369
AC004801.4	0.029754927	7.38E-05	1.84165089
AC004801.6	1.796086159	0.000487554	1.700422238
AC004801.7	0.019009667	0.001452263	1.639010601
AC004805.3	0.274610354	0.000140658	1.783638705
AC004832.1	0.104534614	4.63E-05	1.880951406
AC004832.4	1.48597184	0.0007495	1.662999313
AC004837.1	0	0.004606615	1.557019068
AC004837.2	1.032728164	0.002078169	1.596927533
AC004846.2	1.663523243	0.002237277	1.592465609
AC004852.1	0	0.004606615	1.557019068
AC004852.3	0.034364118	0.004498336	1.89003363
AC004854.1	0.918645535	1.55E-05	1.924612148
AC004870.1	0.296796967	0.003545871	0.557413712
AC004882.2	0	0.004606615	1.557019068
AC004888.1	0.109517451	0.000191446	1.771390529
AC004890.2	2.64764168	0.000691955	1.667883923
AC004893.1	0.456481667	1.26E-05	1.91503064
AC004895.1	0.475862017	0.002065559	1.603391375
AC004906.1	0.238074352	0.001499255	1.633459543
AC004908.1	5.432048151	0.000868608	1.652544919
AC004908.2	4.326656267	0.00319614	1.557382081
AC004908.3	3.046762872	5.93E-07	2.097788453
AC004917.1	0.104809632	0.000377854	1.72307422
AC004923.4	2.479726648	1.98E-06	2.02687294

Restricted IP/MS													
IP/MS											KM analyses		
Gene Name	Accession	Descriptio	Coverage [	# Peptides	# PSMs	# Unique I	# AAs	MW [kDa	calc. pI	Mean	p_value	HR
CANX	P27824	Calnexin C	22	12	13	12	592	67.5	4.6	878.8982	0.001599	0.60529
HNRNPK	P61978	Heterogen	33	13	18	13	463	50.9	5.54	440.2147	5.82E-06	0.498346
IMMT	Q16891	MICOS cc	24	16	16	16	758	83.6	6.48	95.7439	0.001837	0.608072
NPM1	P06748	Nucleopho	23	5	8	5	294	32.6	4.78	739.4235	0.003395	0.624764
SERBP1	Q8NC51	Plasminog	24	8	11	8	408	44.9	8.65	128.6644	0.000193	0.562825

AC004941.1	0.032303739	0.00135781	1.651048547
AC004943.2	4.243982222	0.000338882	0.560981312
AC004943.3	0.258826096	3.81E-05	1.855957836
AC004951.1	0.655633802	0.000398472	1.700308381
AC004951.3	1.439817239	2.60E-05	1.874327153
AC004951.4	0.654144491	8.17E-05	1.799815894
AC004965.1	0.119974381	0.004004942	1.7023352
AC004967.1	20.25487656	2.98E-07	2.137805979
AC004990.1	0.056749038	0.000612629	1.779544433
AC004997.1	0.310943109	0.00360602	1.558534126
AC005005.4	0.033279209	5.61E-05	1.861836218
AC005008.1	0.008741186	0.003116726	2.434917821
AC005014.1	0.091313627	0.001417164	1.663087002
AC005014.2	1.72036161	8.06E-06	1.951565539
AC005042.1	0.418876743	0.001895587	1.615807974
AC005046.1	6.644111488	1.91E-05	1.895690749
AC005077.4	0.290851887	2.31E-07	2.323645976
AC005086.1	0.290109034	0.000922373	1.656269139
AC005091.1	0.173705826	0.003670986	1.556790043
AC005104.1	5.222773606	7.86E-05	1.802771461
AC005154.1	1.616709752	0.00060306	1.681005637
AC005154.3	0	0.004606615	1.557019068
AC005154.5	0.285223422	4.86E-06	1.989389086
AC005156.1	0.169742981	0.003921156	1.548668167
AC005186.1	0.30892353	0.004434743	1.580224315
AC005189.1	0.248811358	0.000444925	1.714131652
AC005224.2	0.020161761	0.002301833	1.629319368
AC005224.3	0.170145183	6.60E-07	2.126000673
AC005244.1	0.520526207	5.38E-05	1.842765463
AC005253.1	3.483474394	0.002954244	1.56235974
AC005253.2	0	0.004606615	1.557019068
AC005261.1	27.03150758	0.0008614	1.64968574
AC005261.3	12.99645589	1.34E-10	2.594109654
AC005262.2	0	0.004606615	1.557019068
AC005277.1	0	0.004606615	1.557019068
AC005291.2	0.533883321	8.93E-06	3.467297713
AC005304.1	0.147173731	0.003313461	1.559156262
AC005306.1	1.568711797	0.000104882	1.78445731
AC005307.1	0.144073277	0.001435785	1.707518669
AC005324.3	0.049702841	0.000419722	1.703358405
AC005324.5	0.747333426	1.01E-05	1.936145786
AC005329.2	0	0.004606615	1.557019068
AC005332.1	1.122843746	8.20E-06	1.941086762
AC005332.3	24.47531058	0.000172916	1.76056863
AC005332.5	8.134231613	6.78E-05	1.813135767
AC005332.6	56.26394105	0.000509009	0.587801558
AC005339.1	0	0.004606615	1.557019068
AC005363.1	0	0.004606615	1.557019068
AC005363.2	0.542644656	0.004692306	1.5413795
AC005387.1	1.957329699	2.40E-07	2.14416792
AC005387.2	2.073304768	4.63E-07	2.109946234
AC005391.1	0.613304852	0.000466139	1.717489982
AC005392.3	0.115295639	0.000130892	1.866966664
AC005393.1	1.861385882	5.51E-06	1.969926741
AC005410.1	0.09203865	0.001189818	1.640907186
AC005479.2	1.618020126	0.004136138	1.546506594
AC005519.1	3.116060472	0.000636207	1.669281981
AC005520.5	0	0.004606615	1.557019068
AC005523.2	0	0.004606615	1.557019068
AC005532.1	0.694331106	0.000887089	1.67524031
AC005534.1	0.681082428	3.68E-05	1.861040041
AC005534.2	1.343625016	0.000730173	1.677198069
AC005538.2	0	0.004606615	1.557019068
AC005544.2	0	0.004606615	1.557019068
AC005562.1	1.701852298	0.001869648	0.617360291
AC005580.1	0.038363974	0.000168924	1.83677574
AC005586.2	2.374494683	2.11E-07	2.180998266
AC005597.1	0.121702524	6.65E-05	1.877776325
AC005606.1	0	0.004606615	1.557019068
AC005609.2	0	0.004606615	1.557019068
AC005609.3	0	0.004606615	1.557019068
AC005609.4	0	0.004606615	1.557019068
AC005618.2	0	0.004606615	1.557019068
AC005618.3	0	0.004606615	1.557019068
AC005625.1	0.479637287	0.004031797	1.62074179
AC005670.1	0.386671786	0.000798796	1.673191622
AC005696.2	0	0.004606615	1.557019068
AC005696.3	1.122184222	0.004076305	1.570746762
AC005703.5	0.136078692	0.001709519	1.665641936
AC005725.1	0.486552667	0.002861017	0.562422835
AC005726.5	0.080477387	0.000896058	1.654057517
AC005730.3	0.71791441	0.001443255	1.623743126
AC005740.3	0.998993554	0.000226685	1.736171296
AC005775.1	0.509461787	2.51E-06	2.019432495
AC005776.1	0.944699837	3.49E-05	1.856338491
AC005776.2	1.317388229	1.61E-06	2.044585533
AC005785.1	2.583080427	1.96E-09	2.409741836
AC005785.2	3.003713545	0.00018572	1.748595374
AC005786.3	0	0.004606615	1.557019068
AC005789.1	0	0.004606615	1.557019068
AC005796.1	0.099938366	4.11E-05	1.851709122
AC005828.4	0.400500212	1.74E-07	2.169074584
AC005828.5	0.329832882	0.002776264	1.590361999
AC005829.1	0.823229292	5.56E-06	1.981828357
AC005837.3	1.305989466	0.001860016	1.647250161
AC005838.1	0	0.004606615	1.557019068
AC005840.2	3.389215723	2.47E-06	2.022028478
AC005841.1	0.665610907	6.46E-05	1.820881251
AC005865.1	0.525580487	5.32E-05	1.868093216
AC005865.2	0.390361727	0.003009696	1.636330651
AC005899.5	0.579932188	0.003533138	1.561728256
AC005899.6	4.727683194	0.001794869	1.599025976
AC005899.7	1.440193954	3.15E-05	1.856513076
AC005906.2	0.153777576	0.000505075	1.923548403
AC005911.1	2.032890242	0.002063763	1.59590853
AC005921.2	0	0.004606615	1.557019068
AC005943.2	0	0.004606615	1.557019068
AC005944.1	0	0.004606615	1.557019068
AC005962.1	1.234219151	0.000313688	1.72053844
AC005972.2	0.101099521	3.95E-06	2.049500249
AC005996.1	0.168710261	2.80E-05	1.918918933
AC006001.1	0.361904748	0.000290589	1.725923017
AC006001.3	5.04776789	0.000463596	1.689044256
AC006001.4	49.74614241	0.000353654	1.718168214
AC006011.1	0	0.004606615	1.557019068
AC006012.1	0.989841645	4.86E-06	1.992478894
AC006015.1	0.017062914	0.000925538	1.996174691
AC006017.1	1.905862982	0.002770868	1.573728484
AC006027.1	0	0.004606615	1.557019068
AC006037.1	0.026062033	0.001407893	2.015447221
AC006058.1	0.647192462	6.10E-09	2.788499411
AC006058.2	0.067060788	0.001607257	1.909690341
AC006058.3	0.308721697	0.004687391	1.79197264
AC006058.4	0.437302437	3.06E-07	2.510475234
AC006064.1	0.929971077	0.004091787	1.545367033
AC006064.4	0	0.004606615	1.557019068
AC006064.5	0	0.004606615	1.557019068
AC006065.3	0.046770368	0.001416324	1.720966681
AC006065.4	0.182458628	0.00164451	1.649442891
AC006077.2	3.219386439	3.21E-05	1.880411967
AC006111.1	0	0.004606615	1.557019068
AC006111.3	0.642353698	0.000761869	1.66790428
AC006116.7	0	0.004606615	1.557019068
AC006128.1	7.243424221	8.69E-05	1.797419874
AC006133.1	0.009293202	9.76E-05	3.706623038
AC006141.1	0.095957053	0.003620782	1.600171852
AC006145.1	0.085826449	2.01E-06	2.310285847
AC006146.1	0	0.004606615	1.557019068
AC006148.1	0.023857208	0.000522498	1.705180972
AC006153.1	0	0.004606615	1.557019068
AC006158.1	0	0.004606615	1.557019068
AC006160.1	1.392234864	4.71E-05	1.839483121
AC006210.2	0.091901616	0.000438899	0.562420051
AC006237.1	0.256273919	0.003946929	1.561227468
AC006254.1	0.80536981	3.64E-07	2.122444268
AC006254.3	0.079398711	0.002388858	1.6308265
AC006262.2	0.799477498	0.000180653	1.931121542
AC006270.1	0.9602984	2.05E-06	2.103819461
AC006270.3	0.033475948	0.002884511	1.68944187
AC006272.1	1.052259787	2.07E-06	2.024721045
AC006277.1	1.136555847	7.22E-05	1.811709672
AC006335.2	0	0.004606615	1.557019068
AC006338.2	0	0.004606615	1.557019068
AC006378.1	3.166297288	0.001673526	1.606512318
AC006378.2	0.952384234	7.10E-06	1.953606424
AC006380.1	0.128950725	1.64E-07	2.434450732
AC006387.1	0.087259429	0.000160647	1.798887827
AC006435.1	0	0.004606615	1.557019068
AC006435.2	3.999363658	0.001355982	1.61699279
AC006435.3	0	0.004606615	1.557019068

AC006441.3	0	0.004606615	1.557019068
AC006449.6	4.116572656	0.001953636	1.600207431
AC006449.7	0	0.004606615	1.557019068
AC006450.2	0.153498315	0.000801622	1.749289151
AC006450.3	0.014722921	0.000479735	1.845785412
AC006455.1	0	0.004606615	1.557019068
AC006455.2	0	0.004606615	1.557019068
AC006480.2	2.458265399	0.003312445	1.558996624
AC006483.2	0.176311539	1.72E-10	2.55494789
AC006504.8	5.58412646	7.59E-06	1.954369236
AC006511.1	0.049959973	0.00255198	1.788538179
AC006512.1	0.291495205	0.001825486	1.613200061
AC006547.1	5.517795547	0.000373309	1.704988057
AC006547.2	0	0.004606615	1.557019068
AC006547.3	0	0.004606615	1.557019068
AC006548.2	0	0.004606615	1.557019068
AC006557.1	0.334112915	0.000532599	1.68023142
AC006557.3	0	0.004606615	1.557019068
AC006557.4	0	0.004606615	1.557019068
AC006600.2	0.024964338	1.66E-06	2.065802194
AC006967.3	0.107416908	0.003713831	1.670225983
AC006971.1	0.166754648	0.001565738	0.593743955
AC006986.1	0	0.004606615	1.557019068
AC007014.2	0.937091645	0.000267651	1.779496458
AC007036.3	0	0.004606615	1.557019068
AC007038.2	5.695707293	1.18E-05	1.921005476
AC007040.2	0.157333339	2.88E-07	2.184872277
AC007064.1	0.062327112	0.001119253	1.804579867
AC007068.1	0	0.004606615	1.557019068
AC007115.1	0.009175756	0.000239504	3.273081127
AC007163.1	0.074711387	0.000678765	1.670952993
AC007182.3	0	0.004606615	1.557019068
AC007192.2	0	0.004606615	1.557019068
AC007193.1	0	0.004606615	1.557019068
AC007193.3	0	0.004606615	1.557019068
AC007204.2	0.084357279	1.42E-05	1.950548296
AC007216.1	1.898527576	0.002321837	1.643747801
AC007216.2	0.780836548	0.00164473	1.613731229
AC007216.3	0.963161201	0.002942703	1.565581369
AC007224.2	1.207717957	2.68E-05	1.874012628
AC007272.1	0.43878391	0.0046021	1.543452136
AC007283.1	0	0.004606615	1.557019068
AC007298.2	0.180525955	0.004920706	1.559256894
AC007308.1	0	0.004606615	1.557019068
AC007322.1	0	0.004606615	1.557019068
AC007322.3	0	0.004606615	1.557019068
AC007336.1	0.410462067	9.29E-05	1.806015339
AC007342.6	0.540667296	0.003519685	1.550268061
AC007347.2	0	0.004606615	1.557019068
AC007349.2	0.265632945	0.000403173	0.533725547
AC007382.1	4.022573228	0.001443321	0.597506456
AC007383.3	0	0.004606615	1.557019068
AC007391.1	0.261642793	5.72E-05	1.827147983
AC007394.1	0.026569233	0.001848363	1.836231833
AC007402.1	0.051836395	1.18E-05	2.080707328
AC007405.1	0.422589894	6.63E-05	1.826081625
AC007422.2	0.385460053	0.000104515	1.843929071
AC007423.1	0.095291576	0.001247577	1.759763218
AC007448.1	0.186735189	0.000981907	1.664606843
AC007448.3	1.667042943	1.32E-07	2.189326141
AC007490.1	2.40943018	0.000244194	0.494535135
AC007497.1	1.115958035	3.31E-05	1.864422784
AC007527.2	0	0.004606615	1.557019068
AC007529.1	0.015789502	0.003144182	1.60513644
AC007540.1	0.053646847	0.000243482	1.849838744
AC007546.1	2.388883247	0.000990807	1.650241113
AC007546.2	0.211824169	0.000494643	1.696962337
AC007569.1	0.60954297	1.61E-07	2.181987177
AC007601.1	0.415509668	0.001824681	1.618705395
AC007608.4	0.101020317	1.62E-05	1.966726262
AC007610.3	0.023979481	0.000115209	1.975807544
AC007610.4	0.281273861	0.000944405	1.646340849
AC007619.1	1.567243305	0.001951522	1.593631715
AC007619.2	0.18506533	0.00047161	1.711849227
AC007620.2	0	0.004606615	1.557019068
AC007620.3	0.297745038	6.21E-05	1.823928516
AC007622.2	0.949410494	0.003407311	1.554127576
AC007637.1	4.498127938	0.000150542	0.513830704
AC007638.1	0.068677843	0.002216044	1.63712959
AC007663.3	1.175142287	0.004874904	1.538503881
AC007666.1	0.246707626	0.000435102	1.706094769
AC007686.1	0.554561737	0.000235389	1.919481817
AC007686.2	0.08121779	0.002808053	1.612281333
AC007687.1	0.42570944	1.75E-07	2.181706744
AC007690.1	0.108929783	0.001238924	0.588904167
AC007743.1	4.151910284	0.000585216	0.555314362
AC007750.1	0.198060232	2.66E-05	1.891845258
AC007773.1	1.269210968	2.19E-05	1.892167826
AC007785.1	1.498910435	3.68E-08	3.404096318
AC007823.1	0.528873236	0.000592094	1.679515502
AC007842.1	3.512322525	0.002273397	1.585915813
AC007879.3	0.592133487	2.31E-10	2.536833508
AC007899.1	0.161687077	0.000554033	1.704673236
AC007907.1	0.129600192	0.000174671	1.775045756
AC007919.1	2.2764762	0.001591994	1.627238741
AC007923.2	0.013825284	0.000866532	1.721070993
AC007923.3	0.217549411	0.000941358	1.754242725
AC007923.4	0.240892694	1.65E-05	1.906937659
AC007938.2	0.081303572	0.004638159	1.544289583
AC007938.3	1.821975491	1.15E-08	2.326377956
AC007991.2	4.98005132	0.003715311	1.581288625
AC008011.2	1.261379749	2.28E-05	1.978302645
AC008013.1	1.102015443	0.000759915	1.807377662
AC008026.3	0.213929903	0.000357841	1.719767569
AC008040.4	0	0.004606615	1.557019068
AC008050.1	1.221309413	8.28E-05	0.489069547
AC008073.2	0.347909425	6.90E-05	1.814320696
AC008079.1	0.293632845	0.000986711	1.639862081
AC008080.1	0.293779417	0.002092428	1.600623877
AC008105.1	0.647155184	5.84E-09	2.359473226
AC008105.3	3.424722259	2.06E-07	2.151895486
AC008115.1	0.331855985	9.02E-05	1.803823907
AC008115.2	0.179465069	0.000271288	1.741704607
AC008127.1	0.129239947	0.000131084	1.83854452
AC008127.2	0.053018702	4.32E-08	2.298450665
AC008132.1	0.012452871	0.004294102	1.587699985
AC008147.3	1.047602226	0.000379345	1.739721209
AC008162.1	0	0.004606615	1.557019068
AC008162.2	0	0.004606615	1.557019068
AC008163.1	0.622956334	0.003017611	1.738304062
AC008243.1	0.30351954	2.87E-05	1.875548704
AC008264.1	0.210415194	0.002910794	1.58732566
AC008267.1	0.133448415	0.000294112	1.743078709
AC008267.2	2.638622363	0.004654733	1.532681089
AC008267.3	3.899472467	0.000708082	1.661304697
AC008267.5	10.81734809	0.000837518	1.658940883
AC008268.1	0.10409555	2.60E-06	2.160216046
AC008277.1	0.195351493	4.83E-05	1.906525557
AC008280.2	0	0.004606615	1.557019068
AC008403.4	0.313552898	2.72E-05	1.923175447
AC008406.3	0.027608108	0.002568771	1.637020127
AC008434.1	0.430480662	1.44E-05	1.912647202
AC008438.2	0.229041364	0.000161141	1.826340585
AC008440.2	0	0.004606615	1.557019068
AC008443.2	0	0.004606615	1.557019068
AC008446.1	0	0.004606615	1.557019068
AC008453.1	0	0.004606615	1.557019068
AC008464.1	0.052297037	0.003267894	1.682446319
AC008467.1	0.498364879	0.00018852	1.750319556
AC008522.1	0.064061522	3.06E-07	2.136458905
AC008525.2	0	0.004606615	1.557019068
AC008543.1	0.924327006	0.000576052	0.563431979
AC008551.1	0.358754145	0.001602248	1.635241702
AC008554.1	11.14740276	0.001065825	0.585657086
AC008555.1	4.106244437	0.000218474	0.544696903
AC008569.1	0	0.004606615	1.557019068
AC008581.1	0	0.004606615	1.557019068
AC008588.1	0.075658616	0.001183017	1.673664457
AC008591.1	0.054857989	4.91E-07	2.109848434
AC008610.1	7.582016165	6.54E-09	2.348823502
AC008649.1	0.307187243	0.004808062	1.545660026
AC008655.1	0	0.004606615	1.557019068
AC008662.1	0.054710548	0.002565825	1.627641015
AC008663.1	0.555022413	1.68E-07	2.323572277
AC008663.3	0.707436895	1.27E-06	2.070269839
AC008669.1	7.860110845	0.001449024	0.601441417
AC008670.1	0	0.004606615	1.557019068
AC008680.1	0.301805232	1.82E-05	1.930724646
AC008708.2	0.068058299	0.000145716	1.881866618
AC008725.1	0.035128599	0.000816878	1.712205742

AC008731.1	1.54567425	0.003301181	1.555691591
AC008734.1	0.545077711	0.003503686	1.557869182
AC008735.2	14.19517669	3.74E-05	1.847985756
AC008735.5	0.12835754	0.00012346	1.809617924
AC008736.2	4.820965215	0.000195813	1.754638515
AC008738.3	0	0.004606615	1.557019068
AC008738.5	0	0.004606615	1.557019068
AC008738.7	0	0.004606615	1.557019068
AC008739.2	0.575526219	0.001559175	1.617881007
AC008741.1	0	0.004606615	1.557019068
AC008743.1	0.061824343	0.000493873	1.695499386
AC008749.1	0	0.004606615	1.557019068
AC008750.2	0.211913725	7.16E-06	1.954017191
AC008750.3	0.168433262	0.001283505	1.640316491
AC008751.1	0.250156449	8.52E-06	1.993900722
AC008758.3	0	0.004606615	1.557019068
AC008760.1	3.736361738	0.001423918	1.617089754
AC008761.1	0	0.004606615	1.557019068
AC008764.3	0	0.004606615	1.557019068
AC008764.5	0	0.004606615	1.557019068
AC008764.6	1.921697633	4.05E-06	1.985983547
AC008764.7	0	0.004606615	1.557019068
AC008764.8	2.158083986	2.12E-07	2.154436977
AC008785.1	0.079468666	7.12E-06	2.039326209
AC008799.2	0.06203699	0.000428126	1.753405522
AC008808.2	0.017320149	1.59E-07	3.04642182
AC008813.1	0.36469589	6.84E-05	1.828801552
AC008870.1	0	0.004606615	1.557019068
AC008870.2	2.561809071	1.64E-06	2.040884264
AC008870.3	0.150730124	0.000402709	1.707032256
AC008871.1	0.205008632	0.001392151	1.661101616
AC008875.1	1.310153513	4.70E-07	2.119507303
AC008878.3	0.036686923	6.84E-06	0.450337478
AC008878.4	0	0.004606615	1.557019068
AC008894.3	0.523130585	8.42E-06	1.940730179
AC008906.1	4.077990777	0.001665041	1.605857671
AC008914.1	0	0.004606615	1.557019068
AC008915.1	0.290163015	0.000300676	1.726011463
AC008945.1	0	0.004606615	1.557019068
AC008962.1	0.047291612	0.001384077	1.948830503
AC008985.1	0.119881759	9.31E-05	1.816145905
AC009022.1	3.389418787	5.30E-05	1.826325395
AC009033.1	0.074293779	0.000163464	1.905512096
AC009041.3	0	0.004606615	1.557019068
AC009053.3	15.5979247	0.000484829	0.548263745
AC009061.2	2.268456011	0.004385114	1.5396727
AC009065.2	2.479445755	4.78E-05	1.839676552
AC009065.4	2.26661931	5.79E-05	1.828282356
AC009065.5	2.560403254	3.00E-06	2.010455338
AC009065.6	0.216881595	0.000112254	1.799680331
AC009065.7	0	0.004606615	1.557019068
AC009065.8	5.877202901	0.001925073	1.599970014
AC009078.3	0.989450083	0.00397297	1.548457447
AC009084.3	0	0.004606615	1.557019068
AC009087.1	0.924797624	0.002526504	1.576642502
AC009088.3	0	0.004606615	1.557019068
AC009093.2	0.571243722	1.67E-05	1.903866274
AC009093.3	0.165550749	0.001434329	1.651567583
AC009093.6	1.057375608	0.0002564	1.738453684
AC009113.2	0	0.004606615	1.557019068
AC009120.2	7.306893742	0.000194927	1.745715508
AC009120.5	0.31637313	2.21E-05	1.892986798
AC009121.1	0.209630608	0.00132287	1.631949615
AC009121.2	0.165878495	0.001045501	1.644653126
AC009126.1	1.90764579	0.0032771	1.565067187
AC009127.2	0.194449658	0.000311428	1.729102811
AC009133.4	0	0.004606615	1.557019068
AC009135.2	0.048700382	0.001884235	1.655342172
AC009139.1	0.311769731	0.001685638	1.626763469
AC009142.1	0.087405366	0.004524645	0.590890866
AC009145.2	0.184535445	0.003235446	1.592429708
AC009145.4	0.262468775	0.000386329	1.72389667
AC009148.1	0.52843379	2.94E-05	1.87032625
AC009169.1	0.041620911	0.000123006	1.863316851
AC009171.1	0.06507873	0.000213448	1.936263603
AC009220.1	1.124831451	1.09E-05	1.933160169
AC009229.3	0.53250105	4.74E-10	2.576036797
AC009237.1	0	0.004606615	1.557019068
AC009237.7	0	0.004606615	1.557019068
AC009237.9	0.006704949	7.60E-06	5.255844275
AC009238.2	0.006119761	0.000629637	3.446253276
AC009238.3	0	0.004606615	1.557019068
AC009262.1	0.018718722	0.001777202	2.06868665
AC009264.1	0.024620268	0.00021383	1.824210881
AC009269.1	0.070098383	1.98E-05	2.02947589
AC009269.2	0.453341108	0.003014098	1.58842853
AC009269.5	1.18197304	0.000119206	1.792976993
AC009275.1	2.187024953	9.06E-05	1.883323617
AC009283.1	15.15760525	0.000169151	1.759127581
AC009292.2	0.235022365	0.001599147	1.625769421
AC009299.1	0.087055504	9.19E-05	1.898172977
AC009299.2	7.923891187	0.00415814	0.619126614
AC009318.1	1.281473958	0.00366483	1.555233335
AC009318.4	1.770126514	0.00019409	1.763730614
AC009403.1	10.84311414	0.000733399	1.664597336
AC009404.1	1.843440993	3.43E-05	1.853645639
AC009446.1	0.046518083	3.23E-05	1.916993448
AC009474.1	0.021744664	0.000124018	1.877390064
AC009487.1	0	0.004606615	1.557019068
AC009487.4	0.022833714	0.00164484	1.713974372
AC009502.1	0.056626704	3.29E-09	2.453465452
AC009506.2	0	0.004606615	1.557019068
AC009511.1	0	0.004606615	1.557019068
AC009518.1	0.1327773	1.35E-08	2.508447195
AC009518.2	0.064892789	4.24E-05	1.882930441
AC009533.1	5.583885827	0.002454581	1.580378271
AC009549.1	5.942839355	2.88E-05	1.91318196
AC009563.1	0.14862425	3.09E-05	1.866883754
AC009630.3	0	0.004606615	1.557019068
AC009652.2	0.157036984	0.000122104	1.816016385
AC009654.1	0.040625691	0.000131116	1.945820042
AC009686.2	2.870162085	0.000775104	1.663377301
AC009690.2	1.976194798	0.003851504	1.545113887
AC009690.3	0.003131131	0.000284181	2.392921749
AC009803.1	0.095599475	0.000224123	1.786699968
AC009804.1	0.093679086	0.000933876	2.016275118
AC009812.4	4.641361829	0.000359066	1.713738609
AC009884.1	0.061472233	0.000135465	1.97415692
AC009908.1	0.032047047	0.00037416	1.948278809
AC009947.2	0	0.004606615	1.557019068
AC009948.1	0.882030863	0.001221577	1.629209245
AC009948.2	0.680773612	8.46E-07	2.084173688
AC009948.3	1.887741662	0.002385487	1.584069343
AC009951.1	0.487169398	0.000296898	1.772394029
AC009955.2	0.078064124	0.000325557	1.863121439
AC009961.2	0.533183117	8.59E-05	1.819053795
AC009963.1	0.070105268	0.002412077	1.970579917
AC009965.1	0.014523874	0.001366614	2.212509522
AC009988.1	0.074876705	9.89E-05	2.027082502
AC009996.1	1.2757379	1.90E-05	1.890882804
AC009997.1	0.519947345	0.001172207	1.632006481
AC010086.2	0	0.004606615	1.557019068
AC010132.2	0	0.004606615	1.557019068
AC010132.4	0.430644473	0.000485764	1.687858899
AC010141.1	0	0.004606615	1.557019068
AC010141.2	0	0.004606615	1.557019068
AC010153.1	0	0.004606615	1.557019068
AC010154.1	0	0.004606615	1.557019068
AC010168.2	3.690192918	0.003730712	1.548262833
AC010173.1	1.567574308	0.000568265	1.683324152
AC010201.1	2.875511857	6.26E-05	1.820626061
AC010245.1	0	0.004606615	1.557019068
AC010245.2	3.965396411	0.000799397	1.65726947
AC010255.3	0.051849733	0.003981329	1.564921654
AC010266.2	0.208964544	0.001356178	1.635187256
AC010307.3	0.021979558	0.001171782	1.690684732
AC010319.2	0	0.004606615	1.557019068
AC010319.3	0	0.004606615	1.557019068
AC010320.3	1.106313047	0.003787139	1.552116601
AC010323.2	0.626657995	7.76E-05	1.805126349
AC010326.3	14.241201	7.71E-06	1.949724631
AC010326.4	10.51886216	1.34E-07	2.187375553
AC010327.2	0.556253226	0.002608074	1.581507338
AC010327.3	0.085599294	4.49E-08	2.421059528
AC010327.4	0.399201306	4.80E-06	1.983570432
AC010327.5	1.464364442	0.000668731	1.665292844
AC010329.2	0.083480014	2.72E-05	1.912403626
AC010329.5	0.083508578	1.03E-06	2.100462362
AC010331.1	1.301740382	9.83E-06	1.944923319
AC010333.3	0.269431775	0.001470585	1.643565687

AC010335.2	0	0.004606615	1.557019068
AC010336.3	0	0.004606615	1.557019068
AC010336.4	0	0.004606615	1.557019068
AC010336.6	0	0.004606615	1.557019068
AC010342.1	0.260248488	1.76E-05	0.477586497
AC010422.2	1.045742115	1.15E-05	1.924828416
AC010451.1	0.06040046	1.08E-06	2.146188491
AC010457.1	1.91596188	0.001533966	1.683515606
AC010463.3	1.280550356	2.50E-05	1.872263837
AC010468.3	0.041448848	2.29E-05	2.197689967
AC010478.1	0.016496598	0.003707815	1.70169686
AC010491.1	0.805542977	7.04E-09	2.346126119
AC010503.1	0	0.004606615	1.557019068
AC010503.2	0	0.004606615	1.557019068
AC010503.3	0.046102135	0.000926609	1.753276734
AC010525.1	0.837056593	1.29E-05	1.915731733
AC010525.2	0	0.004606615	1.557019068
AC010531.6	2.302652772	0.000638257	1.695462419
AC010531.7	0	0.004606615	1.557019068
AC010533.1	0.162826187	0.003369676	1.574706554
AC010536.1	0.688493513	2.39E-05	1.884944784
AC010542.2	0	0.004606615	1.557019068
AC010542.5	8.023516669	0.000147864	1.76298413
AC010547.2	0.144757889	5.63E-05	1.867934273
AC010551.1	0.026841252	0.002516005	1.921022621
AC010551.3	0.131191315	0.001174747	1.734430941
AC010601.1	0.058684242	2.00E-05	1.949353936
AC010616.1	0.927858752	3.36E-08	2.268389665
AC010618.1	75.13415917	4.08E-05	1.852412149
AC010618.3	1.661082352	0.000115612	1.780139332
AC010618.4	2.402261486	0.000236718	1.731826999
AC010619.2	0.188799591	7.25E-05	1.821516799
AC010653.2	0.084287423	0.000148069	1.77871815
AC010655.3	0	0.004606615	1.557019068
AC010719.1	2.662962218	4.80E-06	1.981589943
AC010733.2	0	0.004606615	1.557019068
AC010737.1	4.757542039	0.000513655	0.547416959
AC010746.1	0.086679588	5.77E-05	1.840109607
AC010754.1	0.467816	0.002225713	1.611377345
AC010761.2	4.444013279	0.000142825	1.765803197
AC010761.6	0	0.004606615	1.557019068
AC010768.1	0.059300376	0.000458844	1.759698203
AC010809.2	1.769885157	0.00051052	1.686175942
AC010834.1	0	0.004606615	1.557019068
AC010864.1	1.224399733	1.67E-07	2.170163152
AC010883.1	6.503277434	0.000279557	1.734540829
AC010900.1	0.421655597	0.000587694	1.681118519
AC010904.2	0.397043685	0.003418446	1.578479538
AC010973.2	3.376761946	8.63E-08	2.207040556
AC010976.1	0.924534099	0.000147577	1.764558174
AC010980.1	0.668569844	1.57E-06	2.089193511
AC010980.2	1.510448179	4.59E-06	2.046839013
AC010997.5	0.401179427	4.30E-06	1.992762827
AC010999.1	1.048539262	4.06E-06	1.981890617
AC011005.4	1.921909563	1.27E-06	2.055165343
AC011092.1	0	0.004606615	1.557019068
AC011120.1	0.136442551	0.002889718	1.610837569
AC011131.1	0.083860774	1.57E-05	2.13646044
AC011195.1	0.089574055	0.000685302	1.988483585
AC011243.1	0.049801646	0.001369026	1.813204815
AC011287.1	0.074182771	0.001629981	1.657991459
AC011290.2	2.914264544	4.43E-07	2.111611982
AC011337.1	0.753630798	0.000982343	1.642132402
AC011369.1	0.087491885	0.000727018	1.761036713
AC011369.2	0.053289866	4.89E-07	2.182931793
AC011373.1	0.165830801	0.001796802	1.635202419
AC011374.2	6.394427928	0.000225868	1.744323446
AC011379.1	0.287865272	0.002201703	1.639662474
AC011389.2	1.021946385	0.000723435	1.699736305
AC011405.1	0.181241466	0.002912212	1.606840643
AC011445.2	4.237261619	4.12E-06	2.006708285
AC011446.2	0	0.004606615	1.557019068
AC011447.1	0	0.004606615	1.557019068
AC011447.6	0.230828243	0.002995225	1.56931341
AC011447.7	2.479661217	0.000363134	1.711946255
AC011448.1	1.782908066	7.32E-05	1.813894911
AC011453.1	0.090430841	0.001122797	1.660318605
AC011453.2	0.055858868	5.36E-05	1.968572477
AC011461.1	1.138222208	0.000127642	1.773702742
AC011462.3	0	0.004606615	1.557019068
AC011462.4	0.418619386	9.15E-05	1.847432514
AC011462.5	7.064002454	0.000118051	1.77885528
AC011465.1	0.684100829	0.001333128	1.62933997
AC011466.3	0.445800455	0.001027713	1.63744457
AC011466.4	0.267792446	2.28E-05	1.883506956
AC011467.1	0.106595519	0.00383231	1.605495501
AC011468.1	6.845269397	0.00039173	1.708286652
AC011471.1	0	0.004606615	1.557019068
AC011472.1	9.218153879	0.000572291	1.679564832
AC011472.2	1.626580415	0.00057704	1.690746055
AC011472.3	0	0.004606615	1.557019068
AC011473.2	0	0.004606615	1.557019068
AC011473.3	0.083947346	0.002356172	1.679171007
AC011474.1	0.022860223	0.000106892	1.84701028
AC011476.2	0.339062618	3.88E-05	1.854609479
AC011479.1	1.061635204	0.004174409	1.55849003
AC011479.2	1.1054131	1.39E-05	1.911025807
AC011481.1	50.68788912	0.001434754	1.621602637
AC011481.2	3.169498568	0.000613305	1.673153621
AC011487.2	0.112481767	0.000164797	1.837308307
AC011495.2	0	0.004606615	1.557019068
AC011495.3	0	0.004606615	1.557019068
AC011497.1	0	0.004606615	1.557019068
AC011497.2	4.90347385	4.39E-06	0.400172163
AC011498.2	0	0.004606615	1.557019068
AC011498.3	0.189069052	0.003896112	1.562656921
AC011498.7	2.518008429	0.001053949	1.634138396
AC011503.2	1.203315273	5.06E-06	1.97684816
AC011510.1	1.638277072	0.00021927	1.747270883
AC011511.1	0.463749488	0.000136776	1.770463841
AC011511.4	0.027648971	0.004193739	1.542741758
AC011511.5	0.431361627	0.001638045	1.617489183
AC011551.1	0.20327856	0.002397669	1.609951969
AC011558.1	0	0.004606615	1.557019068
AC011595.1	0.190668678	0.001102924	1.657511468
AC011603.3	0	0.004606615	1.557019068
AC011611.4	0	0.004606615	1.557019068
AC011611.6	0.048213057	0.003277955	0.360843264
AC011700.1	0.054658469	6.08E-06	2.241423756
AC011742.3	0.510634263	4.05E-05	1.864531725
AC011752.1	0.322595118	0.000660086	0.501704628
AC011773.4	0.232735764	6.37E-10	2.476577454
AC011816.1	0.131156075	0.000657674	1.700371375
AC011840.4	0	0.004606615	1.557019068
AC011891.1	0	0.004606615	1.557019068
AC011933.3	0.296859597	0.002114211	1.598934577
AC011939.1	0.508375558	8.23E-07	2.079774918
AC011939.3	0	0.004606615	1.557019068
AC011944.1	0.290437316	0.000929118	1.650700014
AC011978.2	1.685463799	3.38E-05	1.868247919
AC011979.2	0.09727908	1.69E-06	2.116935769
AC012020.1	0.47232219	7.18E-05	1.805429463
AC012065.2	0.971474128	0.003409235	0.619333903
AC012065.4	0	0.004606615	1.557019068
AC012066.1	0.873083419	3.34E-06	2.008519405
AC012073.1	2.14979884	3.80E-07	2.12969798
AC012074.1	0.548907163	0.00341334	1.555610248
AC012085.1	1.257029517	0.003166975	0.6137179
AC012146.1	2.015354044	1.30E-05	1.927744328
AC012157.1	0.596972699	0.000477579	1.69561568
AC012158.1	0.432571081	0.000820991	1.659175306
AC012158.2	0	0.004606615	1.557019068
AC012170.2	3.138825282	7.18E-06	1.953934919
AC012170.3	1.465783199	0.0001667	1.757675568
AC012184.1	0	0.004606615	1.557019068
AC012186.2	1.789575111	8.31E-06	1.944999587
AC012186.3	0.130922242	0.000362604	1.718572231
AC012213.2	0.144551246	0.003571636	1.561689002
AC012213.3	0.831450696	5.48E-07	2.110050462
AC012236.1	1.572250629	1.78E-11	2.745144643
AC012254.5	1.887166691	0.004254379	1.546633515
AC012313.1	0.647227831	0.004880478	1.532764633
AC012313.3	0.405607124	0.001320634	1.618864461
AC012313.7	0	0.004606615	1.557019068
AC012321.1	0	0.004606615	1.557019068
AC012339.1	0.020680144	0.000721626	1.822982229
AC012355.1	0.065901688	0.00207616	1.669253988
AC012358.2	2.705274846	1.44E-10	2.573188303
AC012360.3	2.86267472	0.000457896	1.691041969
AC012370.2	0.089897116	0.000554951	1.698305284

AC012379.1	1.292721643	0.000737255	0.52974343
AC012400.1	0.185939061	1.66E-05	1.99284797
AC012414.2	0	0.004606615	1.557019068
AC012414.3	0	0.004606615	1.557019068
AC012435.1	0.390988378	1.59E-06	2.041944133
AC012441.2	0.171405269	0.002816282	1.586287799
AC012451.1	0.054078601	6.70E-05	2.122496892
AC012462.1	0.177814614	0.001527563	1.64738201
AC012468.1	0.1184787	0.001040759	1.720997778
AC012486.1	0.354184657	0.002206666	1.59132842
AC012493.1	0.039185291	0.000458286	1.733218523
AC012507.1	0	0.004606615	1.557019068
AC012510.1	6.137375528	0.001327392	1.633964293
AC012512.1	1.071128317	1.05E-07	2.401914531
AC012588.1	0	0.004606615	1.557019068
AC012615.1	6.056678416	3.31E-05	1.858951715
AC012615.2	0	0.004606615	1.557019068
AC012615.5	0	0.004606615	1.557019068
AC012617.1	0.798556071	0.001052182	1.642111324
AC012618.1	2.630662691	0.001219074	1.633287727
AC012618.3	2.069881382	8.58E-07	0.409525462
AC012636.1	0.509680222	0.001901949	1.657855312
AC012640.1	0.334402248	5.31E-08	2.24782845
AC012645.4	1.451715106	5.27E-06	1.963087125
AC012651.1	2.342887572	0.002639156	1.57176239
AC012668.3	0.054457654	0.000828015	1.676042878
AC012676.4	0.493475679	0.001215403	1.630366721
AC013268.3	0.107061781	0.000451593	1.722344096
AC013269.1	0.000539223	0.002695885	4.842216482
AC013270.1	0.318877562	0.000121721	1.786981686
AC013356.4	0	0.004606615	1.557019068
AC013391.3	0.389959044	2.91E-06	2.104845813
AC013394.1	0.6703328	1.74E-05	1.906487771
AC013401.1	0.014797392	0.000775495	2.627413648
AC013452.2	0.308377307	0.00375829	1.548963946
AC013468.1	1.046364482	0.000414264	1.702205404
AC013470.1	0.150239547	0.000136978	1.793185912
AC013472.3	0.584384147	0.003876121	1.546497795
AC013489.3	0.371674658	1.29E-06	2.054265748
AC013549.1	0.434200741	5.92E-05	1.839225296
AC013553.4	0.418787036	0.002887235	1.577916763
AC013724.1	0.622552884	0.002838845	1.576385614
AC013726.1	0.096283968	1.39E-05	1.934370968
AC013731.1	1.105642324	3.69E-06	2.000974535
AC015674.1	0	0.004606615	1.557019068
AC015688.6	0	0.004606615	1.557019068
AC015689.2	0.016614861	0.004547835	0.591248363
AC015712.1	1.795055468	0.000209597	1.749925419
AC015712.2	2.017154399	0.000100959	1.793491599
AC015712.4	0.079784409	0.000600488	1.75454126
AC015712.6	0.581347347	0.000942347	1.663552012
AC015712.7	0.399607313	0.00051472	1.694272404
AC015743.1	0.493158643	0.001049721	1.649672798
AC015799.1	0.527835724	2.19E-05	1.90722971
AC015802.3	2.105323862	8.83E-05	1.79644083
AC015802.4	1.017331515	4.38E-05	1.841497411
AC015802.5	1.352891802	0.000178781	1.759554529
AC015813.1	7.860361605	5.45E-05	1.826147712
AC015813.2	0.063826785	0.000497624	1.693949524
AC015813.4	0.592346443	0.000243938	1.761220141
AC015813.5	2.519778019	0.001528143	1.611471079
AC015818.2	0.452701155	8.89E-10	2.506883312
AC015818.6	0.250265834	0.000606682	1.698428797
AC015818.7	0.007686289	0.003115697	6.245690327
AC015849.1	0.483501188	3.50E-07	2.135975754
AC015849.5	0.165347092	0.002187572	1.589798863
AC015853.1	0.561649321	0.000800187	1.664606405
AC015871.4	0.13583136	0.003473659	1.599619647
AC015878.1	0.024965276	0.000152956	2.118571432
AC015878.2	0.10429212	6.56E-05	1.851394506
AC015883.1	0.382053634	0.000419527	1.76142409
AC015908.3	2.40642098	0.001620167	0.598975255
AC015908.5	0.024197825	0.004962421	0.266669414
AC015909.1	0.2459142	0.004202158	1.56782328
AC015909.4	0.097960736	1.77E-05	1.902729142
AC015912.1	0	0.004606615	1.557019068
AC015912.3	2.452942544	2.26E-09	2.420355867
AC015917.1	0.083152765	0.000152332	1.826104856
AC015982.1	4.112745131	0.004416138	1.539384596
AC015987.1	1.152690356	0.001590349	1.623247895
AC016026.1	1.012429383	0.002851227	1.56917033
AC016027.4	0	0.004606615	1.557019068
AC016182.1	0.011120696	0.004301587	2.289802967
AC016252.1	0.901427114	0.000707409	1.688168423
AC016266.1	0.032261429	0.001049686	1.790497433
AC016394.1	4.678628361	0.001711767	1.607746863
AC016394.2	2.766214047	0.000133631	1.770094181
AC016526.2	0.599025824	3.97E-05	1.849020409
AC016542.2	0.121848996	0.000835174	1.708196381
AC016542.3	0.105657322	0.00102168	1.674490854
AC016575.1	0.393147262	9.04E-05	1.812018247
AC016576.1	0.10245612	4.11E-05	2.004085562
AC016582.2	0.750384366	0.001544985	0.606892515
AC016588.1	0	0.004606615	1.557019068
AC016595.1	0	0.004606615	1.557019068
AC016629.1	0	0.004606615	1.557019068
AC016642.1	0.102455435	0.000216275	1.835829825
AC016694.1	0	0.004606615	1.557019068
AC016716.2	0.145727394	0.000424647	1.807585696
AC016717.2	0.042966759	7.63E-05	1.860367244
AC016717.3	0.019156478	0.001484717	1.90499415
AC016730.1	0.106636946	0.000494701	1.938369648
AC016737.1	1.478688082	1.09E-06	2.061276996
AC016738.1	0.569223893	0.000604308	1.69282468
AC016738.2	0.158024782	0.001590915	1.611935443
AC016745.1	0.159541539	1.51E-06	2.083500935
AC016745.2	0.021881036	8.96E-05	1.806066792
AC016746.1	0.14524827	0.002514987	1.595889684
AC016773.1	2.889270926	1.50E-09	2.425455638
AC016820.1	0.028742449	0.004930106	1.81888912
AC016831.1	1.015287382	5.73E-07	2.285428903
AC016831.3	0.053041682	0.000164414	1.774951085
AC016831.6	0.06474723	9.43E-05	1.817856993
AC016876.1	17.21636541	0.000351745	1.738951117
AC016894.1	0.573737716	1.92E-05	1.909272367
AC016903.2	0.07109445	4.84E-05	2.0867147
AC016907.2	0.154030226	0.000646371	1.677418201
AC016911.1	0	0.004606615	1.557019068
AC016949.1	1.269894963	0.0006926	1.669642731
AC016954.1	0.187097069	0.002480724	1.617455071
AC016993.1	0	0.004606615	1.557019068
AC017002.2	0.101221348	8.71E-05	1.807167697
AC017002.5	0.087782282	6.51E-06	2.002161535
AC017006.2	0.332236328	4.19E-05	1.866358423
AC017007.2	0.055306409	3.80E-07	2.151403506
AC017015.1	0.117503057	2.96E-06	2.060993682
AC017015.2	0.257436499	0.001530855	1.620007766
AC017033.1	0.235133546	0.000304493	1.822865964
AC017035.1	0.068359285	0.000166199	1.785359241
AC017071.1	0.467079395	0.000617853	1.680890093
AC017074.1	3.001467883	0.000796514	0.576999898
AC017079.1	0.095320276	0.001364062	1.634383875
AC017083.1	1.207497643	3.44E-07	2.126194264
AC017083.2	0	0.004606615	1.557019068
AC017100.1	2.647963419	8.76E-05	0.505904077
AC017100.2	0	0.004606615	1.557019068
AC017116.1	0	0.004606615	1.557019068
AC018362.1	0	0.004606615	1.557019068
AC018362.2	0	0.004606615	1.557019068
AC018448.2	0.014593986	0.000375665	2.768944622
AC018450.1	1.537227213	0.000405674	1.706737282
AC018464.1	0.05211561	0.000131935	1.826385529
AC018470.1	0.006178422	0.001559318	1.790070887
AC018512.1	0	0.004606615	1.557019068
AC018521.2	0	0.004606615	1.557019068
AC018521.5	2.349234239	0.003356472	0.610367361
AC018529.2	0.827816895	0.00180388	1.602064222
AC018541.1	0.647956338	0.000611028	0.452207338
AC018552.2	0.216074279	0.00094578	1.662048702
AC018554.1	0.009699931	0.002613435	1.985581862
AC018557.1	0	0.004606615	1.557019068
AC018557.2	0.357636321	0.000277394	1.760474711
AC018558.3	0.065420232	0.000512243	1.760300218
AC018628.1	1.351151999	0.001364566	1.625294833
AC018630.2	0.431074157	5.74E-05	1.831774056
AC018634.1	0.17612094	0.000966813	1.685897439
AC018635.1	0	0.004606615	1.557019068
AC018635.2	0.19378796	7.78E-07	2.09401654
AC018638.1	12.958102	3.00E-05	1.863640134
AC018638.2	51.28831981	0.000256843	1.72871223

AC018638.5	38.36026679	2.94E-05	1.864981453
AC018638.6	1.365578032	1.25E-05	1.937948867
AC018638.7	3.076783861	0.000255888	1.72982222
AC018641.1	0.070680569	0.002337562	1.624880215
AC018644.1	1.031714207	4.97E-05	1.833624314
AC018645.2	22.86327562	0.000873944	1.653145362
AC018647.2	12.77890164	0.000853097	0.588596365
AC018648.1	1.060707787	2.04E-07	2.161664546
AC018653.3	10.14025611	0.00011979	1.782726781
AC018682.1	1.123007426	2.98E-05	1.863251531
AC018682.2	0.151025961	4.67E-05	1.844169548
AC018685.2	0.624923655	0.004089114	0.53700841
AC018690.1	3.515786988	5.79E-05	1.825769705
AC018693.2	0.097210285	0.004744731	1.55339809
AC018695.6	2.549017861	2.30E-05	1.926930078
AC018737.2	0.195984804	0.004843184	1.53751749
AC018742.1	7.806475786	0.000217394	0.48345894
AC018752.1	5.825062457	0.001341327	0.59381387
AC018761.2	0	0.004606615	1.557019068
AC018761.4	0	0.004606615	1.557019068
AC018809.1	1.311404049	5.78E-05	1.817972541
AC018816.1	4.548108728	0.000314144	1.727304945
AC018865.2	0.00334866	0.004297392	5.948876154
AC018866.1	0.061850952	0.002495811	1.625063471
AC018892.2	0.029424278	0.000833965	1.793725463
AC018892.3	0.068801366	9.00E-06	1.965598016
AC018904.1	15.50570162	0.00015846	1.767563681
AC018926.1	1.657842758	0.002085532	1.591062051
AC018926.2	1.963638402	2.82E-05	1.879471238
AC018926.3	2.870094791	0.004910459	1.531032121
AC019080.3	1.863352526	1.73E-06	2.038164658
AC019118.1	0.180210367	0.0040374	1.554402098
AC019129.1	0.286947962	0.001917125	1.621587767
AC019155.1	0.574913503	0.000559688	1.696232795
AC019205.1	1.095505579	0.000100991	1.793697883
AC019211.1	0.025502426	0.000307797	1.929734048
AC019226.1	1.474043601	0.001536561	1.621240812
AC019257.1	10.35340289	5.15E-05	1.861909336
AC019294.1	0.02471004	0.000236254	1.7456249
AC019322.1	0	0.004606615	1.557019068
AC019349.1	0	0.004606615	1.557019068
AC020550.1	0.48625082	0.000112705	1.808970633
AC020551.1	0.006837697	0.000614367	3.74889068
AC020558.2	2.45633104	0.000105023	1.782596034
AC020558.3	0.126117613	8.31E-07	2.110167346
AC020594.1	2.524964573	1.00E-08	2.324627477
AC020604.1	0.629492616	2.46E-07	2.148770523
AC020612.1	1.111958179	0.00029358	1.719901136
AC020629.1	0.058449619	0.002939324	1.637792633
AC020651.1	0	0.004606615	1.557019068
AC020656.1	0	0.004606615	1.557019068
AC020658.4	0.101836676	0.001522362	1.619695485
AC020658.5	1.786032737	0.000319234	1.721863141
AC020658.6	0.040385572	0.002492537	1.604434301
AC020663.3	0.546684783	8.34E-07	2.080655517
AC020741.1	0.026160643	0.00099451	1.657322419
AC020743.1	0.056502211	0.002463284	1.614284127
AC020763.2	0	0.004606615	1.557019068
AC020763.4	0.499279699	0.000113111	1.777907824
AC020765.2	1.994231799	0.00013894	1.778694338
AC020765.4	0.194578008	0.001983299	1.615333046
AC020779.2	1.34216062	0.000867958	0.569907225
AC020891.2	0.031608422	0.002250153	1.737259452
AC020904.3	0	0.004606615	1.557019068
AC020907.4	4.761869323	1.16E-08	2.320426516
AC020907.5	0.451656448	0.002479137	1.657180578
AC020908.1	0.276345773	0.003659838	1.563566393
AC020909.2	0	0.004606615	1.557019068
AC020909.3	0	0.004606615	1.557019068
AC020910.4	6.946617008	0.002121812	1.593343842
AC020913.2	0.25937906	6.65E-06	1.959993914
AC020913.3	1.821142524	0.003951462	1.551500594
AC020915.2	3.803157694	0.000230857	1.735967507
AC020917.2	0.372078131	6.52E-05	1.851570977
AC020917.4	4.675790934	0.000477557	1.68558461
AC020922.1	0.006209997	0.00466432	1.717077455
AC020922.2	0	0.004606615	1.557019068
AC020922.4	0.203979689	0.002144561	1.622746341
AC020931.1	0.756416456	0.001378234	1.620058621
AC020934.1	0.15382369	0.000220596	2.0858123
AC020978.3	2.224134565	0.001165385	1.632662229
AC020978.8	0	0.004606615	1.557019068
AC020978.9	1.234245724	0.000513571	1.68252093
AC021074.3	0.085866524	0.001505665	2.353644332
AC021078.1	12.26495809	0.003135538	1.559111427
AC021087.1	2.632512299	7.68E-05	0.510084884
AC021087.3	1.351774321	0.00078078	1.6641752
AC021097.1	0	0.004606615	1.557019068
AC021106.3	2.995978546	0.004079927	0.626588667
AC021192.1	0.364769556	2.89E-06	2.341592858
AC021218.1	12.49461635	0.001721712	0.572400535
AC021321.1	1.225665554	0.001972679	1.599383026
AC021483.2	1.721167709	1.53E-06	0.457395197
AC021491.2	0.360366199	8.09E-06	1.972767204
AC021534.1	0.144052798	2.55E-05	3.421887687
AC021594.1	0.029247066	0.000535457	1.941245563
AC021594.2	0.051599574	0.000202916	1.835052622
AC021683.3	0.024732681	0.000119791	1.862133479
AC021739.5	0.59443132	0.000478504	1.694035386
AC021851.1	1.303219261	0.000500021	1.687016792
AC021876.1	0.076640189	5.41E-08	2.441489303
AC021915.2	0.052561309	0.002394642	1.636194176
AC021945.1	0.283467679	4.29E-07	2.110828155
AC022007.1	1.901938376	1.49E-05	1.938388001
AC022035.1	0.300297677	1.28E-06	2.070353617
AC022079.2	2.784539647	4.13E-05	1.850902706
AC022080.2	0.198606384	0.004146614	1.598122493
AC022087.1	0	0.004606615	1.557019068
AC022092.1	0.374615325	4.78E-05	1.875752724
AC022107.1	7.187419375	0.00385834	0.628643775
AC022133.1	0	0.004606615	1.557019068
AC022137.1	0	0.004606615	1.557019068
AC022137.2	0	0.004606615	1.557019068
AC022146.1	0.037434266	0.003745454	1.637359224
AC022150.3	0.298600119	0.001202425	0.585950972
AC022167.1	0	0.004606615	1.557019068
AC022167.2	2.815842538	0.000423396	1.696795605
AC022167.3	0.352647531	0.000492713	1.691675167
AC022201.2	0	0.004606615	1.557019068
AC022211.1	0.946573686	0.001993574	1.59379406
AC022211.3	1.553864638	5.39E-11	2.623454122
AC022294.1	0.02826322	0.000268594	1.835613882
AC022296.3	0.451266136	1.47E-05	1.924284921
AC022306.3	2.279810352	0.002171772	1.592106244
AC022336.2	0	0.004606615	1.557019068
AC022364.2	0.350090544	0.000432223	1.888800919
AC022392.1	0.073597854	0.004722389	1.535906593
AC022400.1	2.203399885	0.000347473	1.7078398
AC022400.5	0.77223099	0.000633711	1.670225849
AC022400.6	2.394422554	0.003480614	1.550916076
AC022400.8	11.89617985	2.25E-06	2.014189684
AC022432.1	2.730779392	9.03E-07	2.089894974
AC022441.3	0.01752812	0.000818814	4.051793416
AC022445.1	0.343261089	5.51E-05	1.832027345
AC022532.1	1.301218732	0.001889301	1.601894629
AC022535.1	0.059896187	1.81E-05	1.960956768
AC022558.3	1.520045845	0.000105	1.786741582
AC022662.1	0.12485045	0.000737751	1.754448222
AC022690.1	0.093284811	0.000146605	1.861725369
AC022730.1	0.146769118	6.41E-05	1.833819517
AC022748.2	0.050548825	0.002167513	1.731832267
AC022762.2	2.355979608	0.002388666	1.58484715
AC022809.1	0.104400273	0.000303775	1.760686892
AC022816.1	0.082101249	1.96E-05	1.923007663
AC022819.1	0.21577727	8.88E-07	2.073924055
AC022858.1	0.129758868	0.00381281	1.569505986
AC022872.1	0.27585546	0.003426946	1.578318806
AC022878.1	0.086863825	0.003382321	1.881407809
AC022905.1	0.698840778	0.002000906	1.623000367
AC022966.1	0	0.004606615	1.557019068
AC022968.1	0.764437903	0.002268068	0.588302844
AC023034.1	0.099077799	0.003686285	1.579110588
AC023043.1	7.541843458	6.05E-08	2.238135382
AC023043.2	0.1542246	1.94E-07	2.201003877
AC023043.3	0.318210097	1.30E-08	2.364005259
AC023043.4	2.784305667	5.58E-12	2.741781339
AC023050.1	0.075517728	0.002389048	1.660409102
AC023051.1	0.393754704	0.002053787	1.687917329
AC023090.1	0.232447639	1.06E-05	2.076969868
AC023194.3	0	0.004606615	1.557019068

AC023300.1	0	0.004606615	1.557019068
AC023300.2	0.052334426	0.000764823	1.696562766
AC023300.3	0.008816268	0.000264089	1.790197327
AC023302.1	0.402676044	1.53E-07	2.184399783
AC023310.1	0.026772573	0.001878002	1.734426581
AC023355.1	0.484910359	0.002604206	1.575788457
AC023389.2	0.178969068	0.000955933	1.672848495
AC023449.2	0.76911323	0.001921636	1.6067415
AC023469.2	0.041345738	0.004628804	0.516454644
AC023509.4	0.264500346	0.001779188	1.64873462
AC023590.1	0.279128038	7.28E-05	1.851059295
AC023632.1	0.4679029	0.000328812	1.716032887
AC023632.5	0.27019523	0.001444144	1.624536354
AC023794.6	0.497348286	0.001928375	1.611324623
AC023813.3	0.689449177	3.09E-05	1.870294568
AC023818.1	1.459853528	0.00125896	1.622633187
AC023824.2	0	0.004606615	1.557019068
AC023825.1	0.121657132	0.004878436	1.577811413
AC023825.2	0.917495139	4.22E-05	1.849936767
AC023906.3	0	0.004606615	1.557019068
AC023906.5	2.02583354	0.002250299	1.590462766
AC023908.3	0.955124446	0.000181901	1.752402773
AC023946.1	0	0.004606615	1.557019068
AC023983.2	0.287293384	0.000341612	1.718802885
AC024023.1	0.011804789	0.001272523	2.00314088
AC024060.1	10.56486194	4.11E-07	2.121000536
AC024067.1	0	0.004606615	1.557019068
AC024082.1	0.089427427	1.74E-05	1.968325137
AC024084.1	0.216281735	0.004929864	1.541029365
AC024132.3	0.071155519	1.19E-05	2.026809692
AC024145.1	1.801575942	9.68E-06	1.940646113
AC024230.1	0.25210435	0.001066947	1.877515311
AC024243.1	0.277024979	5.65E-05	1.832285318
AC024257.3	0.47694941	0.001999225	1.597400852
AC024257.4	0.833892324	0.000607954	1.677387394
AC024270.3	0	0.004606615	1.557019068
AC024361.3	1.531697239	0.000715743	1.664267419
AC024451.3	0.156998795	0.00060438	1.681462257
AC024451.4	0.403808317	1.00E-09	2.449137673
AC024475.3	0.028969209	5.75E-06	2.382044673
AC024475.4	0.041171853	0.0049327	1.597655032
AC024559.1	0.125068854	0.003171235	1.57089875
AC024560.2	2.059167222	1.19E-05	1.925958009
AC024681.1	0	0.004606615	1.557019068
AC024681.2	0.069730684	2.76E-05	2.20217631
AC024884.2	0.13710183	3.95E-10	2.49633411
AC024940.2	0.113759835	1.57E-06	2.06587412
AC025031.3	0.489216601	3.28E-07	2.164891176
AC025031.4	0.486118884	0.000172086	1.769231429
AC025031.5	0.165117411	9.19E-09	2.343420199
AC025034.2	0	0.004606615	1.557019068
AC025048.1	0.469626918	0.003022523	0.589356735
AC025048.4	1.28502606	3.81E-05	1.857947486
AC025154.1	0	0.004606615	1.557019068
AC025159.1	6.130074803	4.21E-07	2.12406912
AC025162.1	0	0.004606615	1.557019068
AC025162.2	0.877164907	0.000480328	1.698817027
AC025165.1	0.776240246	9.94E-08	2.193635645
AC025165.2	0	0.004606615	1.557019068
AC025165.5	5.134698927	0.000319651	1.718572383
AC025171.3	1.16107844	0.00182379	1.603216029
AC025171.4	4.031433653	1.45E-09	2.435207163
AC025171.5	1.905878512	0.000181825	1.754210283
AC025176.1	0.773859353	0.000574007	1.68662517
AC025178.1	1.370814279	0.002185288	1.583232392
AC025186.1	0.08900124	0.0041189	1.561126329
AC025252.2	0.308449971	0.002478757	1.806339671
AC025254.1	0.109017542	6.19E-05	2.06516496
AC025257.1	0	0.004606615	1.557019068
AC025262.3	0	0.004606615	1.557019068
AC025263.1	0.385829873	0.00206272	0.597729352
AC025265.1	5.342965293	0.000335791	1.755880811
AC025271.1	0.304149587	0.001256121	0.529841653
AC025287.2	0.359728987	0.001779903	1.613742235
AC025370.1	0	0.004606615	1.557019068
AC025419.1	0.0839971	2.05E-08	2.380574336
AC025423.2	0	0.004606615	1.557019068
AC025423.4	0	0.004606615	1.557019068
AC025434.1	0.505945955	4.04E-05	1.892466858
AC025459.1	1.156341354	0.003280232	0.601401681
AC025576.1	0	0.004606615	1.557019068
AC025576.2	0.353417023	0.002840883	1.580682896
AC025578.1	0.02825536	0.002941891	1.751907419
AC025580.2	0.191855129	0.000373687	1.719419856
AC025588.1	0.462389555	0.004150323	1.555760834
AC025594.1	0.076769494	0.00059078	1.767046351
AC025682.1	0.901854461	0.004200139	1.541432303
AC025750.1	0.092858453	2.53E-05	1.937025019
AC025766.1	2.262046766	0.000362514	1.710653531
AC025809.2	0.310016887	2.85E-06	2.013377781
AC025857.2	12.31845348	8.29E-06	1.943924849
AC026124.1	0.059391592	0.0007619	1.703273993
AC026254.2	0.921972296	0.002173863	1.59125435
AC026310.2	0.188232207	3.61E-05	2.008948393
AC026333.4	1.096384993	0.000676858	1.674238875
AC026336.5	0	0.004606615	1.557019068
AC026355.1	0.256363039	0.000260579	1.736691761
AC026356.2	3.518723147	1.86E-07	2.169468835
AC026362.1	0.419847294	1.57E-08	2.305810342
AC026367.2	3.067688182	0.004347379	1.540752017
AC026367.3	4.632326842	0.002068056	1.591740802
AC026401.1	0.205535024	0.000128861	1.802224459
AC026401.2	0.248395933	5.19E-05	1.840955783
AC026401.3	15.20614272	4.83E-08	2.266176138
AC026414.1	0.017628305	0.000330491	2.217649169
AC026444.1	0.093442535	7.18E-05	1.898586548
AC026461.1	0	0.004606615	1.557019068
AC026462.1	0.481525594	0.00103365	0.577084394
AC026462.3	1.819390114	6.02E-06	0.434913333
AC026464.2	0	0.004606615	1.557019068
AC026464.3	0	0.004606615	1.557019068
AC026464.6	0	0.004606615	1.557019068
AC026470.1	27.23399842	0.000183509	0.557511256
AC026470.4	0	0.004606615	1.557019068
AC026471.4	15.74739596	2.71E-05	1.872897582
AC026765.1	0.099533334	0.000144508	1.834801145
AC026765.2	0.069685922	0.002491296	1.617027082
AC026780.1	0.374610754	7.19E-06	0.434728058
AC026780.2	1.351441299	0.000498651	0.51964465
AC026782.2	0.160752557	5.76E-05	1.855017207
AC026785.1	0.095312089	0.003910684	1.599768303
AC026785.3	0.128501338	0.000297296	1.865615405
AC026787.1	0.058921022	0.003229968	1.675616358
AC026803.1	1.557998926	0.001414504	1.618905789
AC026954.2	0.032743754	0.001042066	1.646041918
AC026954.3	0	0.004606615	1.557019068
AC026979.1	0.457582508	0.000682239	1.671520904
AC026992.2	2.480651527	0.002148219	0.598968824
AC027088.2	0.10242494	0.000425129	2.034150199
AC027104.1	0.412530524	0.002190603	1.631957059
AC027228.2	0.31532577	1.46E-07	2.187603314
AC027228.3	0.151637506	0.002245939	1.59058664
AC027243.1	0.033375025	0.000424978	1.838450422
AC027271.1	1.694739868	2.91E-06	2.015649886
AC027277.1	0	0.004606615	1.557019068
AC027279.1	0.649802656	0.000592424	1.685478024
AC027279.2	0.302196626	0.00051693	1.701552744
AC027288.2	1.028246121	0.000627538	1.695818414
AC027290.1	0	0.004606615	1.557019068
AC027312.1	0.060707274	0.004924184	1.564047463
AC027313.1	0.045634769	0.004867947	1.584822171
AC027319.1	0.66603566	1.24E-05	1.928392455
AC027369.4	0.000122209	0.002661197	10.97238137
AC027373.1	0.841644964	1.00E-07	2.196309821
AC027449.1	0.456620755	0.004326915	1.556764055
AC027451.1	0	0.004606615	1.557019068
AC027514.1	0.149659489	0.001409747	1.677637358
AC027544.3	0.093225311	4.58E-05	1.871068595
AC027575.1	0.070520378	8.97E-07	2.0986182
AC027601.1	1.226990344	9.78E-07	2.068053298
AC027601.2	2.881599559	0.000770322	0.564860756
AC027601.4	0	0.004606615	1.557019068
AC027607.1	2.008512493	0.003191558	1.559124247
AC027612.1	0.684967185	0.001882326	0.59510216
AC027682.2	0	0.004606615	1.557019068
AC027682.3	0	0.004606615	1.557019068
AC027682.4	0.709835378	5.47E-07	2.098899845
AC027701.1	0.010207777	0.004133455	1.875405204
AC027702.1	3.962008305	0.000368304	0.556263031
AC027796.2	1.078756286	8.57E-05	1.799740844

AC027796.4	4.587122562	5.73E-08	2.230048447
AC027808.2	0.339451592	0.001264998	1.622221045
AC032044.1	0.903442603	0.002738754	1.568720923
AC032044.2	1.894880313	6.54E-05	1.817967034
AC034102.4	0	0.004606615	1.557019068
AC034102.5	0.700749665	0.000280206	1.733974313
AC034154.1	0.101191385	0.004814654	1.580431271
AC034198.1	0	0.004606615	1.557019068
AC034206.1	3.563285255	0.000132343	0.525665649
AC034213.1	1.041129288	3.27E-08	2.325622762
AC034229.3	0	0.004606615	1.557019068
AC034229.4	0.982929179	1.61E-05	1.914542049
AC034236.2	5.035966	0.000859588	1.654406859
AC034236.3	0.51922111	7.10E-06	1.954225295
AC034244.2	0.053061313	0.00249658	1.647921526
AC035139.1	0.536878299	0.001126833	1.643803021
AC035140.1	0.596069482	0.003772883	0.620057146
AC036108.3	1.157737307	0.002881573	1.567259402
AC036176.3	0.01642507	0.002628624	2.116336238
AC036222.2	0.119773046	0.003550598	1.5989569
AC037198.1	3.776413798	0.001563476	1.624819463
AC037486.1	0.034915849	5.53E-05	1.902266238
AC037487.2	0.377682453	0.000343054	1.831072947
AC040160.1	2.209977517	0.00423858	1.542537479
AC040162.1	2.202532496	2.34E-05	1.877771179
AC040162.3	1.702452011	1.50E-05	1.904103856
AC040169.3	0.860766927	0.001949852	1.600582187
AC040934.1	0.680488224	3.45E-06	1.99676864
AC040970.1	8.426906636	0.00124941	1.626114489
AC044784.1	0.04567305	0.003172126	1.717751852
AC044802.2	0.216237422	0.002755801	1.58901034
AC044836.1	0.382127197	0.000225441	1.849317007
AC046143.2	3.766185457	2.71E-07	2.152610973
AC046144.1	0.057884313	7.05E-07	2.17485675
AC046185.2	6.281641174	0.000699382	0.58605467
AC048341.1	2.735156639	2.49E-06	2.008514523
AC048341.2	10.56978361	1.23E-09	2.435813284
AC048344.3	0.037096561	0.004904728	1.954927495
AC048344.4	2.789615958	6.53E-05	1.825807492
AC048380.2	0.39170494	3.13E-05	1.868217028
AC048382.1	0.470491558	0.002719721	1.5780117
AC048382.2	2.704815644	7.51E-05	1.802124774
AC048382.6	0	0.004606615	1.557019068
AC055713.1	1.650000048	3.19E-05	1.860671442
AC055717.1	0.025249725	0.00372614	1.579782896
AC055758.2	0.098945583	1.77E-06	2.098562263
AC055813.1	0.112993331	0.001978176	1.605057728
AC055872.1	0.120411544	0.004785615	1.685020062
AC055876.2	0.524317671	4.44E-05	1.841402167
AC055876.4	0.605678205	0.00070734	1.669298545
AC058791.1	0.700652222	0.000722694	1.975072261
AC060766.4	10.4845859	0.00046953	1.693747794
AC061709.1	0.014933935	0.003861746	3.875684858
AC061709.3	0.043342197	5.59E-05	1.859774107
AC061961.1	8.056098572	1.08E-05	0.432829572
AC061975.1	0.027367146	0.000425851	1.723232817
AC061975.4	0	0.004606615	1.557019068
AC061992.1	1.881624989	1.67E-09	2.437081132
AC061999.1	3.206744156	0.000334387	0.56059877
AC062037.2	4.053056623	0.00351512	1.554609467
AC063919.1	6.872477686	0.003527398	0.617180164
AC063923.1	0.017642575	0.004509411	1.666260078
AC063948.1	3.400173607	5.71E-06	1.970534208
AC063949.1	0.038151917	0.00036316	1.745051223
AC063949.2	4.88E-05	0.000247096	15.42408839
AC063965.1	2.036311884	0.002621716	1.574637092
AC063977.1	0.477412497	0.002309173	1.59924566
AC063977.6	0.277711893	7.86E-05	1.8081296
AC064807.1	5.087239366	0.0001646	0.538305785
AC064834.1	0.061054203	0.003220336	1.742669913
AC064847.1	0.456952673	1.35E-06	2.066242206
AC064850.1	0.199369724	0.000690659	1.670465503
AC064862.3	0.137546915	3.28E-06	2.1054884
AC064862.6	0.032397858	0.002005155	1.683958235
AC066616.1	0.27798234	0.0001014	1.85614432
AC066616.2	0.014123507	0.003068983	1.678332616
AC067750.1	0.931451207	0.002346072	1.585245886
AC067805.1	0.161814364	0.002020163	1.654828655
AC067838.1	5.796455962	0.00436338	1.539079192
AC067930.1	0	0.004606615	1.557019068
AC067930.2	0.717962027	5.35E-05	1.832993802
AC067930.3	2.84812258	0.000420238	1.704291985
AC067930.5	0.823064023	1.28E-06	2.059905874
AC067931.1	0.929295554	0.000716349	1.686115345
AC067942.2	0.100517945	0.002297151	0.592792706
AC067945.1	0.514570078	4.21E-05	1.880412541
AC067960.1	0.019120266	8.93E-05	2.372231493
AC067968.2	0.040079758	0.004641452	1.642271979
AC068025.1	0.658265625	0.001406871	1.624696176
AC068050.1	0.34607764	0.001189632	1.656495836
AC068051.1	1.404747443	9.82E-05	0.515671843
AC068135.2	0.046954881	0.001768941	1.690997099
AC068152.1	7.081297704	0.003570628	0.617863775
AC068196.1	0.673659966	0.000615226	1.687598979
AC068234.2	5.401270221	0.000399489	1.722123737
AC068385.1	0	0.004606615	1.557019068
AC068389.3	0.12744386	0.0001989	1.789544369
AC068418.1	0	0.004606615	1.557019068
AC068473.4	0.141325909	0.00054655	1.717935282
AC068489.1	0.099325171	0.003160396	1.677554879
AC068491.3	0.278040072	0.000426638	1.71333365
AC068491.4	0.255629801	1.19E-08	2.317407022
AC068492.1	0.699284572	0.000560909	1.711188495
AC068531.1	0.211547663	0.001155156	1.668872453
AC068594.2	0.182883169	0.003720126	1.581110789
AC068620.2	2.062103947	1.61E-05	1.906815664
AC068631.1	0.067692545	0.000227983	1.778323624
AC068669.1	0.178360816	0.000182173	1.757198099
AC068722.2	1.169493375	8.40E-09	2.343754183
AC068724.1	0.248540454	0.000190506	1.756020954
AC068726.1	0.163031652	0.000119668	1.780518607
AC068769.1	0.033260885	0.004762743	1.579340941
AC068789.1	0	0.004606615	1.557019068
AC068831.1	0.814147428	2.05E-07	2.15312134
AC068831.2	0	0.004606615	1.557019068
AC068831.4	0	0.004606615	1.557019068
AC068870.3	0.066392932	0.003276576	1.579701304
AC068944.2	0.028598553	4.11E-07	2.720296216
AC068985.1	0.134194969	6.00E-05	2.207708803
AC068987.2	0	0.004606615	1.557019068
AC069061.2	0.078832304	2.84E-05	1.949690876
AC069148.1	0.501064237	0.000499261	1.711286002
AC069200.1	2.124799861	1.20E-06	2.080267932
AC069222.1	0.279861568	1.16E-06	2.063673633
AC069234.2	0	0.004606615	1.557019068
AC069234.3	0	0.004606615	1.557019068
AC069234.4	1.888696077	0.000957434	1.647325498
AC069257.2	1.717535465	0.000165013	1.758457029
AC069281.2	3.30037076	3.13E-07	2.135318474
AC069285.2	0.037730419	0.004330188	1.89672493
AC069287.2	0	0.004606615	1.557019068
AC069287.3	0.016783109	0.004290738	1.774130799
AC069431.2	0.140604419	0.00105237	1.701494974
AC069437.1	0.275435943	0.000561448	1.731119323
AC069439.2	0.016420521	0.000449322	1.958770527
AC069499.2	0.356477856	0.002155314	1.60343356
AC069503.2	0.438311552	3.35E-08	2.263778219
AC069528.2	4.093342382	0.000623661	1.675920149
AC069542.2	0.142454244	8.05E-05	1.840396527
AC069549.1	1.593954487	2.09E-09	2.412941342
AC072022.1	0.799285562	0.002419184	1.595938292
AC073052.1	0.813000927	0.000504191	1.701237486
AC073052.2	0.177776325	2.30E-07	2.158525053
AC073062.1	0.187644429	4.57E-06	2.283338457
AC073065.1	0.036784895	0.000365003	1.763275537
AC073072.1	0.840233854	1.12E-07	2.383160944
AC073073.1	1.629733585	0.002113853	1.603010194
AC073111.1	0.759299066	1.28E-05	1.919794269
AC073136.2	0.027007705	0.002252517	0.505014726
AC073150.1	0.384839006	0.001342435	1.630942796
AC073188.1	0	0.004606615	1.557019068
AC073195.1	1.796237194	5.21E-07	2.110605187
AC073210.2	0	0.004606615	1.557019068
AC073288.1	0.032790231	0.000140001	2.146332271
AC073288.2	0.416133565	0.000344265	1.707378246
AC073316.1	0.213908959	0.000339967	1.750639822
AC073320.1	0.257717095	5.03E-05	1.843771762
AC073324.1	0.14644936	5.79E-06	2.09058095
AC073326.1	0.421430157	0.00013881	1.782486933
AC073343.2	0.466408256	0.003442498	1.557090122

AC073346.1	7.300030546	0.000475357	0.550426804
AC073347.1	0.015967777	0.00044755	1.732089055
AC073352.1	0.702516013	1.56E-08	2.3068957
AC073359.1	0.025640961	0.002094118	2.013866635
AC073359.2	0.037077844	0.000666642	1.949666035
AC073365.1	0.330317018	0.004125657	1.980396441
AC073389.2	0.092501198	0.00187659	1.607586733
AC073389.3	1.960757353	0.00020635	1.750120213
AC073410.1	1.146487777	2.01E-09	2.423725088
AC073410.2	0.20063741	0.002365552	1.609167343
AC073415.1	1.785904208	0.001820429	0.599058
AC073439.1	0.37977511	2.84E-05	1.868430117
AC073465.2	0.042197571	0.001335703	1.867257266
AC073476.2	0	0.004606615	1.557019068
AC073487.1	2.411195406	0.001045631	1.633394123
AC073508.1	0.102511066	6.14E-07	2.17757557
AC073517.1	0.683227181	0.00109802	1.635438247
AC073525.1	0.012185477	9.12E-06	1.97962058
AC073534.1	1.994743838	0.004818029	1.532023779
AC073534.2	1.284052429	1.12E-06	2.072224408
AC073539.1	0.012166812	0.000137427	1.87393478
AC073548.1	3.886439638	0.000292863	1.719514906
AC073571.1	0.05336037	3.77E-06	2.075967484
AC073573.1	0.272799021	8.53E-05	1.80811889
AC073575.2	1.710188275	8.00E-05	1.805910879
AC073585.1	0.561223807	0.000509975	1.697197098
AC073592.1	0.628354739	0.000186511	1.756575227
AC073592.1	0.031273757	0.00371786	1.682417986
AC073592.2	0.035689007	0.000243837	1.782218741
AC073610.1	2.001770835	1.82E-05	1.905545051
AC073610.3	0.550435922	2.10E-06	2.036811937
AC073611.2	6.062837926	6.42E-12	2.772515895
AC073636.1	1.196817584	0.004903136	0.632248935
AC073651.2	0.042918682	0.004030842	1.76804156
AC073655.2	2.163833254	0.000265711	1.728772558
AC073657.1	0	0.004606615	1.557019068
AC073842.2	2.039164806	0.001504795	1.615717676
AC073857.1	12.71029472	1.11E-07	2.196532306
AC073862.1	0	0.004606615	1.557019068
AC073862.3	0.037806632	0.00472313	1.545630866
AC073864.1	0	0.004606615	1.557019068
AC073869.1	19.66595166	3.15E-06	1.998589754
AC073869.3	1.008379424	0.0008321	1.706448461
AC073896.2	6.20282	7.06E-06	1.960317295
AC073896.4	33.25869945	3.31E-09	2.389086083
AC073901.1	0.076229728	0.002008884	1.698026325
AC073909.1	0.124161896	0.000742192	1.670861743
AC073957.2	0	0.004606615	1.557019068
AC073987.1	0.076633246	0.000972812	1.670852021
AC074011.1	0.629263813	0.003524847	1.555708997
AC074050.3	0	0.004606615	1.557019068
AC074117.1	4.552221165	2.25E-05	1.885133662
AC074117.2	0.673565654	0.002352642	1.580858363
AC074121.1	0.126881637	0.000534461	1.699772248
AC074135.2	0.187649737	0.000732224	1.77417585
AC074183.1	0	0.004606615	1.557019068
AC074212.1	1.279012955	1.66E-09	2.429750673
AC074254.1	0.040584894	1.23E-05	2.201844412
AC074281.1	0.035316686	0.000171681	1.81515847
AC076968.1	0.056741077	0.002176998	1.757999123
AC077690.1	0.114206648	0.000475638	1.708946086
AC078788.1	0.46888678	0.000202218	1.879471266
AC078795.2	0	0.004606615	1.557019068
AC078795.3	0	0.004606615	1.557019068
AC078809.1	0.084909436	0.002745927	1.908290486
AC078814.1	0	0.004606615	1.557019068
AC078819.1	1.387406856	0.00169438	0.603465031
AC078845.1	0.547795721	0.00065043	1.78501802
AC078852.1	0.275898019	0.001588892	1.648398873
AC078857.1	0.005564168	0.001482185	2.818233262
AC078880.3	0.271197848	0.001193747	1.684618622
AC078899.1	0.502811417	0.003382549	0.624903446
AC078899.5	0.495215903	9.61E-05	1.802541166
AC078906.1	1.399508331	0.000226307	1.744574043
AC078909.2	0.714140347	8.71E-06	1.953944846
AC078925.3	0.020362215	0.004877874	1.542251375
AC078962.1	0.460455089	0.000213597	1.760027039
AC078980.1	0.114519245	0.00054592	1.790771729
AC079035.1	0.277440452	4.47E-05	1.892454736
AC079061.1	0.180228339	0.000601094	1.824466776
AC079080.1	0.043827374	0.002769294	1.639338105
AC079089.1	0.111262663	1.86E-05	1.926293926
AC079140.3	1.42933334	0.004297166	1.540148479
AC079148.1	2.05843832	0.002473286	1.58037665
AC079160.1	0.046661058	0.000157449	1.927457869
AC079168.1	0.017046412	0.000901421	2.235449186
AC079174.2	1.487310063	9.41E-09	2.329914009
AC079178.2	0.024178116	2.63E-05	1.933434249
AC079203.1	1.721710397	2.15E-06	2.022653533
AC079210.1	1.032525166	2.45E-11	2.649867191
AC079226.2	0.02145506	0.0005501	2.027953109
AC079248.2	0.003811798	0.002558423	4.077693635
AC079313.1	1.509063739	0.001739221	1.606515798
AC079322.1	1.619030027	0.000526312	1.680801795
AC079328.2	0	0.004606615	1.557019068
AC079329.1	0	0.004606615	1.557019068
AC079336.2	0.230053009	0.003034569	1.567422628
AC079341.2	0.026681968	0.002639337	1.6159791
AC079354.2	0.22609922	0.000409483	1.704388455
AC079360.1	0.205432272	0.000757407	1.700239477
AC079385.2	0.092764693	0.003431958	1.562351109
AC079395.3	0.063048391	0.001098807	1.754369112
AC079414.3	4.362729575	2.41E-05	1.88423611
AC079416.3	0.170180671	1.25E-05	1.952045185
AC079467.1	0.022530775	0.000672063	1.918815748
AC079598.2	0.017207875	0.004194458	4.549533235
AC079598.4	0.17221531	0.00231204	1.74587381
AC079600.3	0.161482894	0.000813195	1.70188373
AC079601.1	0.105195614	0.002696798	1.582981741
AC079684.1	2.391490267	0.00121683	1.631386905
AC079741.1	0.540886367	0.000471946	1.708873936
AC079742.1	0	0.004606615	1.557019068
AC079793.1	0.467096722	0.000715832	1.695339062
AC079848.1	1.656070484	0.001807013	0.591715985
AC079907.1	1.781463814	2.78E-05	1.866213628
AC079917.1	0.01197641	0.000100559	2.291332387
AC079922.2	2.590234917	1.44E-06	2.043164243
AC080011.1	0.04880169	4.38E-09	2.409025297
AC080013.1	0.684931539	2.55E-07	2.155864045
AC080013.2	0.391779221	1.47E-05	1.92932983
AC080013.3	1.188010898	2.55E-13	2.897228057
AC080038.1	37.75730203	0.001894278	1.622632982
AC080038.2	1.020995666	2.68E-09	2.445000085
AC080038.3	0.27985537	0.001195173	1.632725467
AC080038.4	0.770677107	9.41E-07	2.127176297
AC080100.1	0.129971759	0.000110839	1.847006431
AC080128.1	0.075054892	9.64E-05	2.264924385
AC080129.1	0.090489475	0.000988711	1.719557396
AC080129.2	0.77702373	8.68E-05	1.803992499
AC082651.1	0.171231961	0.003234345	1.602583699
AC082651.3	0.12235927	2.23E-06	2.047564706
AC082651.4	0.033286601	1.59E-05	1.957183825
AC083799.1	29.08385593	0.000720411	1.675181812
AC083800.1	0	0.004606615	1.557019068
AC083801.2	0.050068382	0.000194912	1.773793468
AC083805.1	0.088867042	6.37E-05	1.847962529
AC083806.2	0.162113751	0.000410267	1.709858275
AC083809.1	0.498410973	0.001402708	1.66653265
AC083822.1	0.504943453	0.00012909	1.808845286
AC083829.1	0.789615503	8.55E-06	1.957810582
AC083841.1	0.208152717	0.000197708	1.797661956
AC083864.1	0.039570619	0.00081604	1.689588156
AC083873.1	0.956061114	4.06E-06	0.463800294
AC083880.1	2.000522687	0.000532862	1.701207331
AC083899.2	0.043812343	0.004329029	1.589672294
AC083900.1	1.411699176	0.004758481	1.613591144
AC083906.1	0.103014823	9.68E-05	1.805128297
AC083906.2	0.095760691	0.001541849	1.733284043
AC083906.3	0.152296522	1.18E-05	1.938815636
AC083982.1	0.242273472	0.00293027	1.597595032
AC084018.1	12.25372269	0.000243156	1.727124064
AC084018.2	1.134103232	0.001754384	1.602833411
AC084024.1	0.122817569	0.00045202	1.703820714
AC084026.2	0.626259244	1.28E-05	1.921988685
AC084030.1	0.076216455	0.004399474	1.636849903
AC084036.1	16.55475359	1.94E-06	2.037144521
AC084083.1	0.19989332	0.001338781	1.632390526
AC084117.1	9.009065186	2.90E-07	2.135180752
AC084125.1	0	0.004606615	1.557019068
AC084125.2	2.346385017	0.00223538	1.588882362

AC084357.2	1.523792449	0.003256819	1.559261501
AC084756.1	0	0.004606615	1.557019068
AC084759.3	1.0195031	0.001808113	0.359776608
AC084768.1	0.007857341	6.53E-05	2.545380608
AC084781.1	0.93891896	0.000297335	1.740366188
AC084816.1	0.032401947	0.00041627	1.726819856
AC084819.1	0.334715858	0.000993546	1.732861971
AC084824.3	2.185640878	0.002913366	1.563283118
AC084824.4	6.613682919	4.41E-09	2.379727518
AC084824.5	6.215888738	0.00012631	1.776681081
AC084864.1	0.276130964	3.84E-05	1.919432094
AC084876.1	2.418753686	4.16E-08	2.25347146
AC087163.3	1.172881248	0.000274156	1.719376335
AC087190.1	0	0.004606615	1.557019068
AC087190.2	0.09925007	0.000133054	1.793693634
AC087190.3	0	0.004606615	1.557019068
AC087239.1	1.821791305	0.000373551	1.72490986
AC087272.1	0.025232223	0.002383957	1.94830559
AC087273.2	0.484322633	1.33E-06	2.060812016
AC087276.3	0.191948897	8.52E-06	1.994995976
AC087284.1	0.669600015	0.003979426	1.589120074
AC087286.3	0	0.004606615	1.557019068
AC087289.1	54.63356743	0.002445676	1.581222703
AC087289.2	0.62534068	0.003485954	1.551791927
AC087289.3	1.253711834	1.75E-05	1.897961738
AC087289.4	0.054457527	1.42E-07	2.18009871
AC087289.5	0	0.004606615	1.557019068
AC087289.6	1.265728177	2.25E-05	1.890955002
AC087292.1	0	0.004606615	1.557019068
AC087301.1	1.376708334	0.000463904	1.692080226
AC087359.1	0.121971448	3.78E-06	2.016398564
AC087386.1	0.009824228	0.003021651	2.058311902
AC087392.1	0.454345768	5.68E-07	2.233558043
AC087392.3	0.580718675	1.01E-05	1.931348064
AC087393.2	0	0.004606615	1.557019068
AC087393.3	0	0.004606615	1.557019068
AC087430.1	0.020381969	0.002324813	1.629163076
AC087442.1	1.001787051	0.002412379	1.615670307
AC087481.3	11.40407489	0.000598882	1.671398282
AC087491.1	0.297632718	3.38E-06	2.010312852
AC087499.2	0.067744808	0.001379367	1.655175408
AC087499.5	0.025795186	0.004376643	1.676333011
AC087516.2	0.020017229	0.000399516	1.752418604
AC087588.2	1.176374327	3.49E-08	2.283448222
AC087612.1	0.083852304	0.000687268	1.677781889
AC087623.1	2.878637612	0.002801682	1.581118697
AC087623.2	1.167779893	0.001167769	1.634405649
AC087639.1	0.198902559	4.31E-06	1.999720394
AC087645.2	1.472981581	4.05E-05	1.853912026
AC087664.1	0.063652159	1.91E-05	2.175302344
AC087672.2	0.581403879	0.000248933	1.734644496
AC087721.1	0.105421505	0.002017124	1.596535996
AC087741.1	5.023917977	6.72E-05	1.816408248
AC087742.1	0.794703041	4.01E-06	1.996663083
AC087854.1	0.880395483	3.40E-06	2.008678879
AC087878.1	0.632455271	0.001113595	1.685227836
AC087893.1	0.259794765	0.000734003	1.679145042
AC087893.2	0	0.004606615	1.557019068
AC087894.1	0.046024739	2.64E-05	2.095758802
AC087894.2	0.062523145	0.00410868	1.603661858
AC089983.1	0.221168216	4.15E-08	2.301811395
AC089984.1	0	0.004606615	1.557019068
AC089984.2	0.377920653	8.29E-06	1.954668244
AC089987.1	0.04777219	1.97E-05	1.918937432
AC089999.1	0	0.004606615	1.557019068
AC089999.2	1.052870469	0.0004448	1.701965188
AC090004.1	1.315237691	0.003294783	1.559562631
AC090023.1	0.169150227	1.34E-07	2.47985504
AC090061.1	0.459713289	0.000494011	1.691516357
AC090079.1	0.068260515	7.53E-05	1.883144771
AC090092.1	0.423790253	0.004717041	1.555781909
AC090095.1	0.01796985	0.000616423	1.930399461
AC090099.1	0.023559482	0.001717694	1.672295486
AC090142.2	0.063190394	1.45E-05	2.053474953
AC090150.2	0.17835331	0.002651939	1.630423857
AC090164.2	1.187435106	0.001877962	1.68104251
AC090164.3	0.218927328	0.002613479	1.582763909
AC090181.1	0	0.004606615	1.557019068
AC090192.2	0.269512986	0.00027423	1.860578225
AC090193.1	0.036179008	6.02E-05	1.888579758
AC090236.1	0	0.004606615	1.557019068
AC090260.1	0.333057522	0.001309417	1.625800639
AC090286.1	1.455636753	3.31E-05	0.50338578
AC090312.1	0.022044708	0.004549658	1.937973481
AC090337.1	0.426934732	0.000470531	1.703894902
AC090340.1	0.020182003	9.15E-05	1.93727601
AC090377.1	0.044432622	8.14E-06	2.015804615
AC090403.1	0.109061859	0.001797468	1.689344955
AC090415.1	0.038595015	0.000628186	1.876915605
AC090458.1	0.022008218	0.000241176	2.095458064
AC090510.1	0	0.004606615	1.557019068
AC090510.3	0	0.004606615	1.557019068
AC090527.1	0.159516701	0.002918337	1.609464292
AC090527.3	0.936137001	0.004391711	1.543169292
AC090559.2	0.342535889	0.000745572	1.680333493
AC090578.1	1.103567644	0.002425161	2.091108857
AC090589.3	5.7798364	0.000818348	1.651148327
AC090592.1	0.047725426	0.000994935	1.714554828
AC090602.1	0.755902054	0.001087853	0.581649818
AC090607.4	0	0.004606615	1.557019068
AC090607.5	0.035378783	0.002742037	1.931161554
AC090616.6	1.523590845	0.000205243	1.743183405
AC090617.3	0	0.004606615	1.557019068
AC090617.6	0.5698672	0.00391048	1.549411489
AC090618.1	0.418157249	0.001172864	1.635255063
AC090643.1	0.018501619	0.002351682	1.597096481
AC090673.1	0.116290631	0.002167757	1.743859396
AC090739.1	1.890381148	0.00096231	1.645706781
AC090772.4	5.83175156	3.84E-07	2.138917362
AC090774.2	0.07942745	0.000170368	1.834321212
AC090791.1	0.060474591	0.000427038	1.784791789
AC090825.2	0	0.004606615	1.557019068
AC090826.1	0.064895888	0.000975476	1.78801203
AC090833.1	0.060023693	2.36E-05	1.913837729
AC090844.3	0.197481925	0.001586997	1.685498499
AC090877.1	0.589924481	2.43E-07	2.168681086
AC090888.1	0.013108574	0.000450264	1.932014251
AC090907.1	2.090553245	0.001045846	1.647447188
AC090907.2	0.658407558	0.001801333	1.602120609
AC090912.3	0.397762288	1.65E-07	2.17821854
AC090970.1	0.469237575	0.003892528	1.5827781
AC090971.1	0	0.004606615	1.557019068
AC090971.3	1.165926985	0.001747097	1.60553051
AC090983.1	0.031604718	0.000160277	1.81830949
AC090984.1	0	0.004606615	1.557019068
AC090987.1	0.030113124	8.61E-05	1.857275781
AC091027.2	0.075560887	0.000973583	1.901954634
AC091053.1	0	0.004606615	1.557019068
AC091053.2	0.277518779	0.00252841	1.582245268
AC091057.1	1.239421314	6.44E-06	1.958669909
AC091057.3	0.518043975	6.86E-05	1.828271732
AC091062.1	0	0.004606615	1.557019068
AC091100.1	0.187836025	0.000400892	1.715813751
AC091117.1	0	0.004606615	1.557019068
AC091132.2	0.518378836	0.000744575	1.6669951
AC091133.1	0	0.004606615	1.557019068
AC091133.3	0.419507695	0.00012526	1.787154011
AC091133.5	1.463841076	0.003732697	1.555140695
AC091151.1	0.175936306	0.000185675	1.947092277
AC091152.3	0.101476047	2.04E-05	2.021524409
AC091153.2	0.31762167	9.37E-07	2.08002675
AC091153.3	0.990869987	1.74E-06	2.04405523
AC091163.3	0.024945259	5.88E-05	2.009484631
AC091167.1	0	0.004606615	1.557019068
AC091167.4	0.459132506	0.003568015	1.553422234
AC091173.1	0.028865883	0.000116522	2.091463307
AC091180.1	0.112656219	8.97E-05	1.838047296
AC091180.3	0	0.004606615	1.557019068
AC091180.4	0.795077916	0.000508139	1.689516077
AC091180.5	0.661899461	0.000152986	1.768107309
AC091182.2	0.468453109	2.05E-07	2.277643918
AC091230.1	0.011161131	5.24E-06	2.006558705
AC091304.6	0	0.004606615	1.557019068
AC091435.2	0.640570513	0.001438736	1.625006021
AC091544.4	0.150382135	6.50E-06	2.009445503
AC091551.1	0.213925103	0.000249377	0.539719312
AC091564.1	0.928936242	0.000417716	1.708075749
AC091564.3	0	0.004606615	1.557019068
AC091564.4	0	0.004606615	1.557019068
AC091588.2	0.096840934	5.59E-05	1.892298149

AC091588.3	0.434728652	0.004413543	1.545466108
AC091729.3	10.4198662	0.000191323	1.751527251
AC091736.1	0	0.004606615	1.557019068
AC091805.1	0.207592714	0.002269758	1.608653169
AC091812.1	0.504690407	2.45E-07	2.611545387
AC091839.1	0.127446763	0.002532127	1.607998824
AC091932.1	0.041675726	1.96E-07	2.605202385
AC091946.1	0.640710117	0.004091825	1.611280366
AC091946.2	0.590981028	7.12E-06	1.963430406
AC091951.1	0.060580657	2.74E-06	2.145446617
AC091959.1	0.810421002	0.004985833	1.546929407
AC091959.3	0.122403422	0.002715517	0.616893951
AC091980.2	0.025243618	1.29E-06	2.144605136
AC091987.1	0.014016854	0.00044682	2.012415799
AC092045.1	0.399663619	7.27E-07	2.087519496
AC092068.1	0.262434679	0.003542003	1.581536314
AC092068.3	0.074364408	0.000196577	1.774493268
AC092069.1	0	0.004606615	1.557019068
AC092070.1	0	0.004606615	1.557019068
AC092070.3	0.260836335	0.000111136	0.504593205
AC092071.1	0.130629627	0.000481445	1.746972413
AC092111.1	1.523526784	0.003989099	1.547085675
AC092112.1	0.86385106	0.001201843	1.642890485
AC092115.1	0.754482696	0.000134729	0.526140154
AC092115.2	0	0.004606615	1.557019068
AC092115.3	0.494266901	0.002058261	1.600976975
AC092118.2	2.444462559	0.002964744	1.565529056
AC092119.2	2.828814092	5.91E-06	1.963570799
AC092132.1	0.067491628	0.00078732	1.836380041
AC092139.1	0	0.004606615	1.557019068
AC092143.1	0.012631302	5.21E-07	2.169393077
AC092143.3	0.929511954	0.000152969	1.762805093
AC092168.2	0.418655869	1.32E-05	1.921451134
AC092170.1	0.058591545	0.000694098	1.809338897
AC092171.4	3.027347579	6.90E-07	2.091048154
AC092198.1	0.040915374	0.004394156	1.545554376
AC092295.2	3.173080085	0.003372675	0.624673479
AC092296.1	0.823327654	8.08E-05	0.503446827
AC092301.1	1.004848744	0.000173158	1.754503854
AC092326.1	0.043203749	0.001602765	1.730012573
AC092327.2	0.018505121	0.000658507	2.009817207
AC092332.1	0.135174232	9.37E-06	1.971517857
AC092335.1	0.035664542	0.000771975	1.762206887
AC092338.2	1.447633566	0.002153664	1.586037693
AC092354.2	3.038403492	0.000743716	0.582528945
AC092423.1	0.176089721	1.36E-05	2.068437046
AC092436.2	0.211542689	0.000967488	1.789183304
AC092447.1	0.007559261	0.0005775	2.343507488
AC092447.3	0.028807713	7.05E-05	2.084406124
AC092447.7	0.0325091	0.000180119	1.843467066
AC092451.2	0.036038505	5.21E-06	2.142862483
AC092470.1	0.070738909	0.000803852	2.165621667
AC092484.1	0.226612783	1.71E-07	2.467398887
AC092535.3	0.454468167	0.001749138	0.550601593
AC092535.4	20.53354433	2.10E-06	2.05214236
AC092570.1	0.053943857	0.000298937	1.838195899
AC092580.3	0.250872746	0.003806477	1.56842306
AC092590.1	0.02400435	0.000257988	2.412459057
AC092593.2	0.034443471	0.000375696	1.812607377
AC092620.1	0.178982896	0.002504651	1.617933466
AC092651.2	6.399417235	1.42E-07	0.407902522
AC092673.1	0.025048177	0.003182004	2.048621228
AC092675.1	0.187683882	1.48E-10	2.614076453
AC092681.1	3.833478583	0.001002033	1.64343462
AC092683.1	0.388970541	8.24E-06	1.945529869
AC092685.1	0.048124424	0.000157499	1.787777944
AC092687.2	0.050183361	0.002926084	1.654187428
AC092687.3	5.844110524	0.00017308	1.761481603
AC092701.1	0.048599861	0.000867023	1.712122778
AC092718.4	14.97868333	0.000108111	1.793811038
AC092718.5	1.556530622	0.002146015	1.592140848
AC092725.1	0	0.004606615	1.557019068
AC092747.3	0.066224923	0.003288608	1.712777971
AC092754.2	0.028628022	0.001023228	1.926160975
AC092755.2	0.680749456	0.000417819	1.710822156
AC092756.1	0.873938806	0.000461524	1.697644269
AC092757.1	0.314908256	1.95E-07	2.211082166
AC092757.2	1.900251033	5.61E-09	2.352984669
AC092757.3	1.547086661	9.83E-09	2.333087778
AC092794.1	3.087378794	0.002246208	1.583496742
AC092794.2	0.533640927	8.71E-06	1.967427444
AC092809.4	1.446198602	1.96E-05	1.894386643
AC092810.3	0.032136755	0.000696581	1.918236099
AC092813.1	0.281635384	0.000180386	1.872649211
AC092818.1	0.09293005	0.000232117	1.745171631
AC092821.1	0.232870292	0.004160268	1.546532342
AC092828.1	0.439873542	1.25E-06	2.096206582
AC092894.1	3.491358636	0.002438684	0.596651589
AC092902.1	1.51048227	0.000810877	1.659610246
AC092953.2	3.353526555	7.80E-07	2.109706836
AC092954.1	0.529465096	0.002389981	1.607488296
AC092957.1	0.078704723	0.003770477	1.593557491
AC092958.1	0.018050641	0.000146158	1.814951513
AC092964.1	0.698690561	5.70E-12	2.749054503
AC093001.1	2.996797989	0.000178287	1.871530383
AC093023.1	0	0.004606615	1.557019068
AC093027.1	0.106580607	0.00060869	1.774197998
AC093117.1	0.035923166	0.002383357	1.602405093
AC093152.1	0.213345879	0.000980015	0.572809601
AC093166.3	0.195452767	0.000129516	1.800698577
AC093206.1	0.303858064	0.001164439	1.654318555
AC093278.2	23.9424162	0.000989842	0.585693511
AC093281.1	0.238340736	9.50E-07	2.13477192
AC093281.2	0.913085626	1.79E-06	2.094222438
AC093330.1	0.031847018	0.000583313	1.688273188
AC093382.1	0.694324555	7.60E-05	1.816013814
AC093425.1	0.303682643	0.00049652	1.698376493
AC093426.1	0.030354542	0.000386175	1.777704755
AC093458.1	0.987061843	0.000660388	1.673534979
AC093458.2	2.876021994	7.34E-05	1.822991924
AC093462.1	0.565696869	0.004438694	1.539000494
AC093484.3	0	0.004606615	1.557019068
AC093484.4	2.257929924	3.00E-05	1.862808143
AC093503.2	0	0.004606615	1.557019068
AC093503.3	0.328342445	0.001890687	1.628669301
AC093510.1	0.486882771	3.17E-05	0.482837088
AC093512.1	0	0.004606615	1.557019068
AC093520.1	0.243005161	4.79E-07	2.15310945
AC093520.2	1.031862885	0.001624051	1.640354126
AC093534.1	0.061877291	0.001808365	2.096930806
AC093534.2	0.0955811	0.000226689	1.780188546
AC093535.1	2.176383289	0.00302432	1.567936984
AC093536.2	0.082713991	0.000448829	1.793159317
AC093578.1	0.137815905	0.000206779	1.767720472
AC093582.1	0.990228842	0.00012574	1.784586584
AC093599.1	0.140821579	0.00028055	1.788763825
AC093627.1	0.153806349	3.09E-07	2.74598463
AC093673.1	45.3323319	7.76E-06	1.96343027
AC093689.1	0.032233655	0.000370114	2.11817479
AC093690.1	0.94950905	0.000357475	1.710189207
AC093702.1	0.65809935	8.08E-06	2.050643512
AC093726.2	5.880569615	8.43E-05	1.803282115
AC093730.1	0.04972162	0.001736081	1.746811145
AC093763.1	0.073077272	0.001017991	1.761465621
AC093788.1	2.28305527	2.15E-07	2.153394252
AC093801.2	0.040736872	0.000125513	1.847908717
AC093802.1	0.043019881	0.000494437	1.733846883
AC093816.1	0.225014511	0.000204125	0.537225944
AC093827.3	2.987014882	8.20E-06	1.946784283
AC093852.1	0.044385051	0.001724428	2.011823051
AC093857.1	0.036429129	0.002855517	1.667211311
AC093895.1	0.330410119	8.62E-05	1.865463877
AC093904.3	0.168934128	0.000810009	1.753149711
AC093909.1	1.627462308	0.000214013	0.550791609
AC095038.1	0.019845089	0.000219825	1.839423564
AC095038.2	0.038149245	0.000351495	1.805838441
AC095055.1	2.854566992	2.91E-05	0.504582945
AC095057.3	2.804857796	4.09E-08	2.24332915
AC096537.1	0.380251061	1.91E-06	2.271991347
AC096543.2	0.021244658	0.001714388	1.781774939
AC096586.1	0	0.004606615	1.557019068
AC096633.1	0.08739565	6.27E-05	1.842486869
AC096636.1	0	0.004606615	1.557019068
AC096637.2	0.165941427	0.000916041	1.656190353
AC096642.1	1.186660705	0.000146032	1.763633903
AC096649.2	0	0.004606615	1.557019068
AC096656.1	0.054449147	0.000106887	1.833755364
AC096677.1	2.900774086	0.002219432	1.59734304
AC096677.2	0	0.004606615	1.557019068
AC096708.1	0.097882846	0.004538874	1.556656495

AC096708.3	0.734082768	2.26E-08	2.287821073
AC096720.2	0.982731406	1.01E-11	2.758720238
AC096772.1	0	0.004606615	1.557019068
AC096947.1	0.412979631	0.004689801	1.564016496
AC097059.1	1.213238129	0.000223099	1.751196196
AC097065.1	0.281037654	8.36E-07	2.133483682
AC097347.1	0.359476252	5.46E-05	1.830917153
AC097358.2	0.063661631	2.94E-05	1.888287248
AC097359.1	0.246422399	0.004367319	1.554499072
AC097359.3	0.069090359	0.000379601	1.722046467
AC097372.2	1.304149828	0.00035629	1.712430333
AC097375.3	0.031119852	0.000161548	1.809282104
AC097460.3	0.243512454	0.002061598	1.612179737
AC097468.2	0.066556294	0.00183744	1.667846689
AC097480.1	0.024780354	0.000943124	1.838430482
AC097522.1	0.032617633	0.000109119	1.9408254
AC097535.2	0.296053049	0.001913201	1.693909237
AC097634.1	0.48039511	0.000251618	1.743457529
AC097637.2	0.158108514	0.002014347	1.594884338
AC097639.1	2.185816647	9.85E-05	0.512947587
AC097641.2	1.846513905	3.78E-08	2.259139519
AC097714.1	0.050222854	7.12E-06	2.000287017
AC097717.1	0.053185264	0.000291153	1.734093352
AC097721.1	0.056121877	0.00194081	1.664460284
AC097724.1	0.21819711	0.000203754	1.752935378
AC098484.1	7.495613509	3.96E-07	0.430494523
AC098484.2	1.024537566	1.60E-05	1.902543032
AC098613.1	4.706376567	0.001296207	1.627810671
AC098614.2	0.041682481	0.000297933	1.886886293
AC098657.2	0.013713075	0.002010575	1.689049986
AC098679.1	0	0.004606615	1.557019068
AC098679.2	0.422800084	0.00062415	1.673807081
AC098799.1	0.025628008	0.003101886	1.694551122
AC098818.2	0.097822452	0.000123232	1.792937081
AC098826.2	0.121036796	3.99E-05	2.120331798
AC098831.1	0	0.004606615	1.557019068
AC098847.1	0.181137007	0.004220879	1.547390476
AC098850.4	0.014706172	0.001504929	1.638080855
AC098851.1	2.047642985	0.001838294	1.602365348
AC098934.4	0.077979978	0.000495082	1.706968357
AC098936.1	0.032602437	0.003918084	1.550662663
AC099063.1	0	0.004606615	1.557019068
AC099066.1	0.14724995	0.000604286	1.690145849
AC099066.2	0.487957558	0.004721826	1.577902433
AC099335.1	0.323767183	0.003493525	0.599427673
AC099343.2	1.583029481	5.69E-05	1.849493486
AC099398.1	0.027315539	0.000158579	1.805167811
AC099487.1	0.139466418	0.000247153	1.794538398
AC099489.1	0.565011667	3.35E-09	2.421731813
AC099489.2	1.243537024	0.000457781	1.732927411
AC099489.3	0.064766929	0.000655179	1.705901218
AC099508.1	0	0.004606615	1.557019068
AC099513.1	2.11083227	9.33E-07	0.438231964
AC099518.5	0	0.004606615	1.557019068
AC099518.6	1.734508835	0.000948032	1.648650291
AC099520.1	0.019355272	3.73E-07	2.187880351
AC099520.2	0.018450417	0.003083745	2.783853602
AC099522.2	2.605610648	6.41E-05	1.823569459
AC099524.1	0.647103063	2.65E-06	2.069060742
AC099552.1	0.039183026	0.00318552	1.639975639
AC099552.2	0.091659629	0.000864989	0.560900623
AC099552.4	0.042385883	1.35E-07	2.218452672
AC099566.1	0.009732887	1.26E-05	2.173626971
AC099654.1	0.041131264	0.000550074	1.782056713
AC099677.3	0.143529636	0.000511846	1.705822492
AC099681.1	0.151185973	0.003943851	1.565300029
AC099681.3	0.124261024	6.40E-07	2.096614414
AC099687.1	1.106023935	0.000100684	0.502356883
AC099689.1	0.117820772	7.68E-05	1.92326931
AC099791.1	0.091712888	0.000866747	1.676591236
AC099791.2	0.949372316	1.34E-07	2.186023153
AC099796.2	0.098283518	0.00417052	1.878377855
AC099799.1	0.23383737	5.45E-07	2.200390461
AC099811.1	0.406243957	0.003843328	1.560923842
AC099811.4	0.655040522	0.002237274	1.598578947
AC099811.5	0.456352063	0.00311743	1.56933552
AC099850.2	0	0.004606615	1.557019068
AC100756.1	0	0.004606615	1.557019068
AC100756.2	0.073639439	3.61E-05	1.919665546
AC100768.1	0.027124258	0.001444645	1.92567576
AC100784.1	0.274508184	0.001428525	1.683345467
AC100800.1	0.051328668	9.22E-05	2.061486306
AC100803.3	2.197052046	3.95E-06	1.992438136
AC100812.1	1.080769972	0.000198619	1.85377455
AC100814.1	7.133772212	1.01E-06	2.076899439
AC100821.1	0.683907067	0.004201022	1.543262542
AC100827.2	0.497008259	0.002696067	1.575707092
AC100827.3	0	0.004606615	1.557019068
AC100827.5	0.254234615	7.17E-05	1.839998657
AC100830.3	0	0.004606615	1.557019068
AC100832.2	0.094449689	1.34E-06	2.091215133
AC100835.2	0	0.004606615	1.557019068
AC100854.1	4.661591478	0.000636661	1.672959252
AC100861.2	0	0.004606615	1.557019068
AC100872.1	1.469054257	0.00011508	1.911984306
AC102797.1	0.075122985	2.59E-05	1.956441404
AC102953.2	2.891484866	0.00060687	1.680182676
AC103409.1	0.108375714	1.29E-06	2.185582695
AC103591.2	3.445862666	0.002203267	0.610552454
AC103591.3	4.020843917	0.000491963	1.69485878
AC103691.1	5.657157129	0.000192434	1.762814666
AC103702.2	8.47486619	2.03E-06	2.061340268
AC103706.1	3.214985399	3.22E-12	2.757714967
AC103746.1	2.269681872	0.004252539	0.630475342
AC103769.1	0.486649207	1.82E-05	1.934412126
AC103770.1	0.474059207	4.17E-05	2.018612992
AC103808.1	0.046786522	0.001540417	1.660518435
AC103810.1	1.122161216	0.000535603	1.679788382
AC103810.3	10.61425192	7.02E-06	1.959536381
AC103810.8	0	0.004606615	1.557019068
AC103925.1	0.280959469	9.77E-08	2.895439507
AC103987.1	0.190467351	3.97E-05	1.864992791
AC104010.1	0.124778544	0.000378284	1.923834097
AC104042.1	0.068761703	0.004866711	1.573655964
AC104046.1	1.0034294	5.13E-11	2.624396657
AC104051.1	0.050441351	0.000475451	1.804300059
AC104073.1	0.075304767	0.001264244	1.93983544
AC104078.1	0.13003675	0.001667487	1.623728818
AC104116.1	0	0.004606615	1.557019068
AC104117.2	0	0.004606615	1.557019068
AC104170.2	0.419063957	0.000191246	1.779134613
AC104211.2	3.935993732	0.000946662	0.557972195
AC104248.1	0.223851481	3.08E-05	1.878274468
AC104333.1	0	0.004606615	1.557019068
AC104365.1	0.243862889	0.00194517	1.61237435
AC104365.2	0.522052128	0.001316644	1.659100028
AC104365.3	0	0.004606615	1.557019068
AC104423.1	0	0.004606615	1.557019068
AC104435.2	0.141124851	4.00E-05	1.863509947
AC104506.1	0	0.004606615	1.557019068
AC104564.2	0.082279783	0.00014468	1.78541594
AC104564.3	3.327173377	2.32E-05	1.879842
AC104564.5	0.792413534	4.93E-06	1.975334692
AC104574.1	0.030145938	0.001324863	1.721103338
AC104574.2	0.023947109	0.001472844	1.650459533
AC104590.1	0.213232539	0.000457354	1.699724799
AC104596.1	1.505190759	0.003553902	0.622088575
AC104619.3	1.314830599	0.0017625	0.575502035
AC104655.1	1.694200474	0.000354176	1.709122676
AC104695.2	0.541491437	7.38E-05	1.853281317
AC104695.3	6.42958843	3.25E-05	1.867969241
AC104695.4	12.78548898	2.15E-07	2.233351464
AC104758.2	1.396596734	1.39E-07	2.185298349
AC104758.5	0.040364018	0.002978537	1.762216749
AC104763.1	0.609245883	0.002220152	1.597762971
AC104763.3	0.133499918	0.000350661	1.769852004
AC104777.2	0.022262971	0.000676331	1.989383205
AC104791.2	0.133023551	0.000642197	1.687110006
AC104803.1	0.007655539	0.004454697	2.445987366
AC104852.1	0.017322761	0.000406556	1.845540855
AC104958.1	0.126110746	0.003109056	1.642407012
AC104982.2	0.101562638	0.003726705	1.60733952
AC104984.1	0.180230924	2.82E-05	1.886224413
AC104984.5	0	0.004606615	1.557019068
AC104986.2	7.1730004	0.003585937	1.555358774
AC104996.3	0	0.004606615	1.557019068
AC105020.1	5.430474	0.000230305	1.742130119
AC105020.2	0.278249897	0.004827342	1.538225185
AC105020.4	0	0.004606615	1.557019068
AC105020.5	4.096466794	0.004415428	1.543022223

AC105031.2	0.042046901	0.000126024	1.881989056
AC105052.2	1.857940707	4.16E-05	1.852703837
AC105053.1	0.304899332	0.000115552	0.513647674
AC105105.3	1.19844639	3.17E-06	2.010649432
AC105114.1	0.03406295	0.002852399	1.598962014
AC105118.1	1.148158787	1.38E-07	2.316919283
AC105137.2	0.751081267	0.001523255	1.622970793
AC105177.1	0.051345998	1.08E-07	2.418210435
AC105219.1	0.962942466	2.71E-07	2.137903969
AC105235.1	0.7075726	1.37E-05	1.914463108
AC105254.1	0.119172425	0.001541142	1.655086838
AC105275.1	0	0.004606615	1.557019068
AC105285.1	1.060436783	0.000633175	1.674868844
AC105339.1	0.058992258	7.78E-08	2.215155014
AC105339.2	0.558505233	0.000399049	1.701043077
AC105339.5	0.960823392	0.004286793	1.541305304
AC105384.2	0.551518778	2.88E-06	2.042957861
AC105391.1	0	0.004606615	1.557019068
AC105411.1	0.796494966	0.000762087	0.562335366
AC105429.1	0.572948327	0.002677347	1.585535686
AC105446.1	6.250421562	4.21E-09	2.414004612
AC105749.1	0.765225919	0.004582445	1.535485418
AC105758.1	0.085922683	0.000276112	1.903137758
AC105935.2	0.156656085	3.61E-05	1.93054756
AC106028.2	0.680003829	0.002821173	1.579835806
AC106028.3	1.109430139	0.000745538	1.663759265
AC106028.4	0.387815985	0.000129743	1.775942637
AC106037.1	0.792324344	0.004163656	1.547023809
AC106038.2	0.129846269	0.002474279	1.623028501
AC106053.1	0.130986955	5.48E-05	1.855062338
AC106760.1	0	0.004606615	1.557019068
AC106795.1	6.047088812	0.004870334	1.532890149
AC106796.1	0.486377013	0.000124963	0.491272686
AC106801.1	0.145310207	7.93E-08	2.225813305
AC106820.2	0.086982947	4.19E-06	1.991120277
AC106820.3	2.467772324	0.003444968	1.568968003
AC106820.4	0.422871454	2.93E-06	2.03981214
AC106822.1	0.165035075	0.001105938	1.702187283
AC106865.1	1.035166406	0.000485562	1.752063862
AC106870.1	0.151159452	0.000413528	1.796708135
AC106872.8	0.025170279	0.003815709	1.621016037
AC106882.1	0.106573018	4.09E-05	1.944438658
AC106886.1	0.39452158	0.003077142	1.566795038
AC106886.2	0.604199608	5.51E-06	1.978782624
AC106886.3	0.572841007	2.33E-05	1.892114606
AC106895.1	0.15260989	0.002497772	1.740424799
AC106895.2	0.069541475	4.05E-07	2.345676179
AC107016.1	0.02405894	0.001259518	2.455555285
AC107016.2	0.091115481	0.000332005	1.958711505
AC107021.2	10.24367625	4.07E-06	1.993979502
AC107027.3	3.832553313	0.004140302	0.63687642
AC107029.2	0.04798136	0.000747746	1.734869824
AC107081.2	1.229678219	1.58E-09	2.414289351
AC107081.3	0.146962881	2.30E-06	2.048370557
AC107204.1	0.125425302	5.67E-06	1.98385118
AC107294.1	0.604992445	2.57E-05	1.88262235
AC107308.1	0.318232822	1.37E-05	2.64516981
AC107373.1	0.327939279	0.002340737	1.590015426
AC107373.2	0	0.004606615	1.557019068
AC107398.2	1.208145169	7.73E-05	1.823109465
AC107419.1	0.062901505	0.000663031	1.733542877
AC107464.1	0.383583805	0.001645231	1.650265631
AC107958.3	0.195835895	0.001110532	1.680384673
AC107976.1	0.041228449	0.000331915	1.888484004
AC107993.1	0.717278312	0.000181563	1.754425364
AC108002.1	0	0.004606615	1.557019068
AC108025.2	0.24312804	3.74E-05	1.863243731
AC108067.1	0.068680032	0.001931185	1.844306809
AC108114.1	0.284751917	0.003855679	1.551872643
AC108134.1	0.271332174	0.004136337	1.572668919
AC108134.4	2.717868919	1.23E-07	2.188884744
AC108136.1	0.519742949	0.000733393	1.962432996
AC108145.1	0.239875896	2.12E-08	2.53171312
AC108448.2	0.760205391	0.001324867	1.630609885
AC108449.2	12.87935716	8.36E-05	0.51627864
AC108449.3	0	0.004606615	1.557019068
AC108462.1	0.020768462	0.004873053	1.722638356
AC108463.3	1.790212295	0.003783614	1.550924006
AC108472.1	0.676316164	0.00096701	0.568376336
AC108479.1	0.950676239	0.003206325	0.633518382
AC108482.1	0.103878782	0.000151955	1.808165181
AC108517.1	0.013198959	0.000253812	2.74601533
AC108673.2	3.87170626	0.000610575	1.676234608
AC108673.3	9.772329173	1.84E-08	2.301842939
AC108693.2	0.784595139	0.002363247	0.594152504
AC108727.1	1.783765561	0.000102609	1.791400695
AC108733.1	0.023624702	0.000374808	2.112240508
AC108747.1	0	0.004606615	1.557019068
AC108751.4	0.106508826	0.003291448	1.613514954
AC108863.1	0	0.004606615	1.557019068
AC108865.1	0.467243715	0.001529101	1.644290745
AC108865.2	0.255813292	0.000546528	1.717856004
AC109322.1	2.370835204	2.48E-05	1.888547706
AC109347.2	2.39162	0.001784807	1.606144652
AC109361.1	0.168400759	0.004075161	1.545975919
AC109446.3	0.721742936	0.003852086	1.554668404
AC109460.1	1.474617296	0.00040798	1.697011903
AC109460.3	0.859706171	5.03E-05	1.831024403
AC109460.4	0	0.004606615	1.557019068
AC109462.2	0.286573917	0.001603597	1.698484862
AC109466.1	0.058923741	0.00340838	1.625881882
AC109471.1	0.05419611	8.73E-08	2.319403566
AC109486.1	0.234274593	0.000543705	1.809156642
AC109492.1	0.073630698	0.004622219	1.577911393
AC109597.2	0.318528779	0.000435663	1.748988876
AC109780.1	0.019835291	0.001276335	1.746795957
AC109809.1	0.237831465	0.004772929	1.537898697
AC109927.2	0.093352891	0.001040767	1.781234692
AC109992.1	0	0.004606615	1.557019068
AC109992.2	0.2563215	0.000536599	1.686292773
AC110011.1	0.036212009	5.27E-05	2.079159921
AC110015.1	1.071705453	9.28E-05	1.796865568
AC110048.2	0.099389951	0.001757806	1.629111412
AC110285.2	7.877153998	0.001587988	1.621544449
AC110285.3	0.165381788	6.23E-05	1.900077989
AC110285.4	0	0.004606615	1.557019068
AC110285.6	1.132166735	4.53E-05	1.885351217
AC110597.3	0	0.004606615	1.557019068
AC110619.1	0.224678241	0.001390665	1.654835412
AC110771.1	1.149739052	0.003116333	1.567830593
AC110772.1	0.130249657	0.000187445	1.76582607
AC110772.2	1.233022194	0.000373725	1.797349146
AC111170.3	0.771365019	0.001724634	1.613454382
AC111188.2	0.078212619	0.004620405	1.583025153
AC111200.1	0.118816475	0.000774719	1.733620784
AC112184.1	2.945051945	0.004756111	1.543980939
AC112200.2	0.077178379	7.47E-07	2.160835809
AC112211.1	0	0.004606615	1.557019068
AC112220.2	4.916992373	5.65E-08	0.361016253
AC112242.1	0.007147476	0.00475295	3.037907231
AC112484.1	1.417875163	0.001870801	1.602596989
AC112484.3	1.558965841	0.00212096	1.611975784
AC112492.2	0.017713754	1.74E-05	2.391166092
AC112493.1	0.17279765	0.000947008	1.715007185
AC112497.1	10.07992677	5.20E-06	1.979514294
AC112498.1	0.019073841	0.003384566	1.980108557
AC112503.1	0	0.004606615	1.557019068
AC112512.1	0.148861419	0.001056326	1.677207694
AC112693.3	0.044970848	0.000879792	1.71727124
AC112694.1	0.796554346	0.004076779	1.541539448
AC112694.2	1.185656369	6.42E-05	1.812072571
AC112715.1	0.581677499	1.11E-16	3.335700067
AC112721.1	0.344316632	0.000240588	1.92868119
AC112721.2	0.582887201	4.04E-06	2.192662558
AC113139.1	1.536259875	0.000440019	1.701949518
AC113143.1	0.609695194	0.001148743	1.668336697
AC113143.2	0.18837807	0.001167862	1.637222367
AC113189.1	0	0.004606615	1.557019068
AC113189.3	0	0.004606615	1.557019068
AC113194.1	0.126511086	5.38E-06	2.014993142
AC113208.3	0	0.004606615	1.557019068
AC113346.1	1.589824799	3.31E-07	2.276387761
AC113346.2	0.035565785	0.000589901	1.896164909
AC113367.3	0.063245571	0.000924077	1.735773016
AC113368.1	0.761389717	0.003915419	0.619124775
AC113410.3	0.917084198	2.82E-07	2.152317357
AC113418.1	0.04636397	0.000259381	2.187515203
AC113608.1	1.744903375	0.000464502	0.54028469
AC114316.1	1.099635305	0.001036444	0.549057608
AC114321.1	0.193999687	0.000132317	1.862939731

AC114401.1	0.029989592	0.001980234	1.637110056
AC114402.1	1.538539108	0.000165803	1.759304066
AC114402.2	0.299109308	0.003183141	1.602487628
AC114488.2	0.384028395	5.52E-05	1.832735713
AC114489.1	0.126182543	0.004389098	1.562287071
AC114495.2	2.534775459	0.002850748	1.565501453
AC114498.2	0	0.004606615	1.557019068
AC114546.3	0	0.004606615	1.557019068
AC114689.1	0.03386136	1.58E-05	1.992382696
AC114730.3	2.159250942	0.000436706	1.695895622
AC114737.2	0	0.004606615	1.557019068
AC114755.2	0.177089839	1.18E-05	2.015521078
AC114755.5	0.038896958	0.000224701	1.837999694
AC114797.1	0	0.004606615	1.557019068
AC114814.2	0.016257382	3.64E-05	2.571853216
AC114878.2	0.069137925	0.000622187	1.759986656
AC114940.1	0.005292261	0.00080604	2.393200197
AC114956.1	0.830766514	4.68E-05	1.843664918
AC114956.2	0.72730112	0.003412353	1.566332334
AC114980.1	1.036746872	0.003769802	1.551894501
AC115102.1	0.385292522	0.001603841	1.608786674
AC115618.3	43.22545977	0.000268843	1.733310641
AC115676.1	0	0.004606615	1.557019068
AC115837.1	24.94206923	0.004395711	0.639435855
AC116003.1	0.121130809	3.42E-09	2.479237941
AC116021.1	0.357267589	7.40E-11	2.640420474
AC116049.2	0.75901107	3.19E-05	1.938232701
AC116345.2	0.06390754	0.000179649	0.483525796
AC116345.3	11.04736831	0.003089912	0.595349654
AC116366.2	0.296748544	0.002405585	1.613537462
AC116407.2	3.644790461	2.18E-07	2.153157888
AC116407.3	0.564298632	8.72E-05	1.805694745
AC116407.4	1.175048774	6.64E-06	1.953133163
AC116447.1	1.393696839	0.002378645	1.579487974
AC116553.1	0.086768985	0.00169479	1.678375424
AC116563.2	0	0.004606615	1.557019068
AC116913.1	3.320337189	1.48E-05	1.92395272
AC116914.1	0.255297127	0.001757548	1.628922178
AC116914.2	5.688603425	1.29E-06	2.048490149
AC117373.1	0.093557941	1.98E-06	2.123288732
AC117382.2	0.061945675	0.00376158	1.555455071
AC117489.1	0.480243495	0.002031959	0.595137991
AC117490.2	0.716254586	0.00091781	1.649645944
AC117498.1	0	0.004606615	1.557019068
AC117498.3	0.494612135	5.29E-06	1.985541809
AC117503.1	0.417660992	0.001040698	1.641444632
AC117503.4	1.691458347	0.001300697	1.618871856
AC117529.2	0.025848649	0.004554639	1.580737646
AC118465.1	0.146556896	0.000345414	1.728735596
AC118469.1	0.08442273	0.003219709	1.612201917
AC118755.1	0.776547338	0.000468418	1.736106511
AC119044.1	3.599259326	0.001869852	1.602511357
AC119396.2	1.553917396	0.00123198	1.627133516
AC119403.1	0.807355243	0.000314786	1.722376971
AC119751.4	0.198396189	0.000883281	1.768263287
AC119751.5	0.006866353	0.001686648	2.333766248
AC119868.1	0.125315933	7.81E-07	2.10460751
AC119868.2	0.273130481	0.002128602	1.619192331
AC120036.1	0.051058457	4.09E-05	1.895494375
AC120053.1	12.41106019	0.000535747	1.684052719
AC120057.3	0.532648591	0.001473307	1.617194237
AC120057.4	2.904619033	9.97E-06	1.927139393
AC120114.2	0	0.004606615	1.557019068
AC120114.3	3.802836294	0.000169554	1.754623323
AC120349.1	1.656789543	2.47E-07	2.179537081
AC120498.1	2.21776512	8.47E-06	2.050781882
AC120498.9	0.270821349	2.33E-05	1.883430804
AC121251.1	0.581342877	3.66E-06	2.007592721
AC121338.2	3.77163289	4.84E-07	0.443613702
AC121758.1	0.03201721	0.000286109	1.76608457
AC121761.2	2.502714945	6.45E-05	1.821187307
AC122108.1	0	0.004606615	1.557019068
AC122108.2	0.090381713	0.000173628	1.773111918
AC122129.2	0	0.004606615	1.557019068
AC122688.3	1.218546183	2.19E-05	1.882525798
AC122688.4	0.042508789	0.000457067	1.749963507
AC122710.2	0.425070173	8.17E-08	2.219779375
AC122713.1	0.058062119	0.000814046	1.70456963
AC122713.2	0.212008812	0.004356786	0.616842236
AC123768.1	0.08628267	1.33E-05	1.923817004
AC123768.2	1.043024083	2.94E-05	1.867445747
AC123786.2	0.009029524	9.65E-05	3.148303812
AC123904.3	0.007244997	0.000270556	2.35923155
AC123912.5	0.25073769	4.56E-06	2.018484702
AC123912.6	0	0.004606615	1.557019068
AC124016.1	1.358030954	1.14E-07	2.191713328
AC124057.1	0	0.004606615	1.557019068
AC124066.1	0	0.004606615	1.557019068
AC124067.2	1.178210979	3.84E-05	1.888763433
AC124067.3	0.071441746	2.01E-06	2.09562005
AC124067.4	1.10023807	1.18E-05	2.061826559
AC124068.1	0	0.004606615	1.557019068
AC124242.1	1.35670164	0.00333695	0.62005083
AC124276.1	0.133253224	1.60E-05	1.918970192
AC124283.1	0	0.004606615	1.557019068
AC124283.3	0.637532788	0.00050508	1.690958703
AC124283.5	0.3089047	4.98E-05	1.833396006
AC124290.1	0.256387517	0.000441548	2.015557242
AC124319.2	1.791837834	2.59E-05	1.881745524
AC124854.1	24.74466812	9.39E-08	0.38521503
AC124893.1	0.044496369	3.87E-05	1.991822012
AC124944.3	4.496410297	0.002137704	1.599710602
AC125257.2	0	0.004606615	1.557019068
AC125388.1	0	0.004606615	1.557019068
AC125494.1	0.450846694	1.13E-05	1.923465165
AC125604.1	0.158943911	0.00182926	1.617152425
AC125611.1	1.632478572	4.99E-08	2.244532173
AC125611.2	0.592774754	3.18E-06	2.005743086
AC125793.1	0.032137677	0.001665052	1.723194332
AC126118.1	1.389693086	4.16E-05	1.844953158
AC126177.6	0.295011328	0.001957151	1.682660018
AC126615.2	0.223191941	0.003826327	1.559999101
AC126696.1	0.119935083	4.67E-08	2.286337587
AC126696.2	0.185742053	1.56E-05	1.937872521
AC126696.3	0.139363228	1.72E-05	1.938535601
AC126768.1	0.068656114	0.000851792	1.727335852
AC126773.1	0.677486233	3.45E-07	2.13533128
AC127024.1	0.321589433	0.002521481	1.587089238
AC127024.2	0.404187052	1.90E-06	2.027645811
AC127024.4	4.093481146	0.000174762	1.75018787
AC127024.5	6.325689099	0.000844178	1.651826679
AC127024.6	1.531093738	0.000290949	1.726428661
AC127024.8	0.448928873	0.002557012	1.583802621
AC127070.1	0.930452263	0.000473602	1.699979465
AC127070.3	0.319379428	0.003954597	1.576201706
AC127164.1	0.194791679	0.000286634	1.743004763
AC127455.1	0.166047877	0.000141627	1.894659769
AC127459.1	0	0.004606615	1.557019068
AC127459.3	0	0.004606615	1.557019068
AC127496.2	0.536072379	0.003534457	1.576086943
AC127496.4	0.131398373	0.000618194	1.721454374
AC127496.6	0	0.004606615	1.557019068
AC127502.1	1.4447035	0.000401167	1.703157456
AC127502.2	9.386860245	2.79E-05	1.870784821
AC127522.1	0.036901742	0.000834592	1.745721583
AC128657.1	0.041160315	0.002717677	2.073172197
AC128685.1	0.367422331	1.24E-05	2.492040751
AC128709.2	0.146853406	6.95E-10	2.486853779
AC129492.8	0	0.004606615	1.557019068
AC129507.1	1.941924928	0.001064668	0.536165026
AC129510.1	4.439791947	2.09E-06	2.02380603
AC130304.1	0	0.004606615	1.557019068
AC130324.3	0	0.004606615	1.557019068
AC130343.1	0	0.004606615	1.557019068
AC130371.1	0.148975321	5.62E-06	1.978166758
AC130415.1	0.036082321	3.97E-06	2.181433384
AC130448.1	0.032732763	0.000197738	1.752460845
AC130456.2	0.019448128	0.002728828	1.670686093
AC130456.5	0	0.004606615	1.557019068
AC130456.6	0	0.004606615	1.557019068
AC130462.1	0.420604542	0.000900921	1.648619396
AC130469.1	2.138231263	8.35E-08	2.200597315
AC130650.1	0.41883459	3.76E-06	2.026747314
AC130650.2	1.069619694	3.51E-06	1.998604315
AC131009.1	2.263816861	0.000933394	1.657874162
AC131009.2	0.157830183	0.000265435	1.762208661
AC131009.3	5.155835017	1.37E-05	1.912377958
AC131025.1	0.470263446	7.65E-06	1.954306537
AC131025.2	0	0.004606615	1.557019068
AC131097.3	0.382836314	2.19E-05	1.905004322

AC131649.1	0.068477472	0.000132801	1.833081995
AC131902.1	0.113491544	1.22E-05	1.940524046
AC131902.2	0.029516932	0.004104335	1.558078386
AC131935.2	0.025670631	1.08E-06	2.147361489
AC132008.2	17.01107568	0.002234326	1.585142026
AC132186.1	0.041451037	0.001579569	1.814171817
AC132192.1	0.705915003	0.000147937	1.76749287
AC132192.2	3.628901367	0.000487446	1.693304678
AC132217.1	0	0.004606615	1.557019068
AC132812.1	24.87426906	0.004817969	1.530880588
AC132872.1	13.87453036	0.000150396	1.759270971
AC132872.2	0.65040239	1.18E-05	1.926331624
AC132872.3	8.794172842	8.92E-06	1.932893419
AC132938.2	0.294286065	0.00101919	1.644334133
AC132938.4	0	0.004606615	1.557019068
AC132938.5	1.585677126	0.001982599	1.593075568
AC133065.3	11.16241763	0.000214396	1.741022457
AC133104.1	0	0.004606615	1.557019068
AC133106.1	0.108302459	0.003801379	1.573185678
AC133435.1	2.022191845	0.001454331	1.618770993
AC133485.3	0	0.004606615	1.557019068
AC133540.1	0.065943643	0.000217813	1.797279331
AC133552.1	0.075838222	0.000322231	1.725351239
AC133565.1	0.436122678	0.000171262	1.799455373
AC133785.1	0.058424771	0.002243258	1.741468597
AC133865.1	0.01491098	0.000489067	2.42271305
AC133963.1	0.083520866	9.95E-05	1.812859572
AC134312.5	0.339522155	1.60E-05	1.967886775
AC134407.1	0.530398737	9.97E-06	1.933966962
AC134407.2	0.241826419	0.000612046	1.672317689
AC135048.1	0.741520681	3.39E-06	2.005314296
AC135048.2	0	0.004606615	1.557019068
AC135048.4	5.197906377	0.000375562	1.709846296
AC135050.1	0.457821545	3.18E-05	1.866350426
AC135050.3	6.266214769	5.96E-05	1.82645965
AC135050.7	0.749891558	0.000812361	1.656028667
AC135068.2	1.153914227	0.004311343	1.616633712
AC135068.4	0	0.004606615	1.557019068
AC135178.5	2.965249139	4.02E-08	2.256411261
AC135279.1	0.938153503	0.001421944	1.613352271
AC135457.1	0	0.004606615	1.557019068
AC135507.1	1.739837423	4.29E-05	1.885787307
AC135584.1	2.911913184	0.001427801	0.476800995
AC135586.1	0.280654908	0.00053434	1.693055576
AC135721.1	7.659841185	0.001900523	1.595590834
AC135776.2	0	0.004606615	1.557019068
AC135776.3	0	0.004606615	1.557019068
AC135782.1	0.019451996	0.002650134	1.599157753
AC135895.1	0.016178946	0.000335581	1.929695498
AC135983.1	0.127385169	0.000679884	1.676264488
AC135999.1	0.064623784	0.001145056	1.798840582
AC136469.1	0.415545738	0.000358814	1.718095373
AC136619.2	0.034404924	0.000230633	1.920288776
AC136944.5	0	0.004606615	1.557019068
AC137055.1	0.71358741	2.13E-05	1.909288686
AC137579.1	0.097964276	9.23E-07	2.158800655
AC137590.1	0	0.004606615	1.557019068
AC137630.2	0.563107019	0.003080204	1.564053413
AC137630.4	0	0.004606615	1.557019068
AC137695.1	0.225868647	0.000294894	1.760235197
AC137695.2	0.188963238	0.000556019	1.700153308
AC137767.1	1.653181188	1.62E-05	1.911832475
AC137932.3	1.533330443	0.000395384	1.698816572
AC138028.4	5.430349254	0.004470318	1.535085031
AC138123.1	0.86273825	0.002922985	0.604028721
AC138150.2	2.127708319	0.000177783	1.766344958
AC138207.1	0.81973646	0.001958299	1.59192371
AC138207.4	5.918716691	0.001069124	1.63282569
AC138230.1	1.871946891	5.92E-05	1.818562913
AC138304.1	0.017097768	0.001330355	2.021652203
AC138305.2	0.137674488	8.91E-05	1.919300898
AC138356.2	4.164719939	0.000393474	0.552800157
AC138393.2	0.509966709	0.000178919	1.7689363
AC138393.3	1.416308844	0.000730067	1.669138111
AC138430.2	0	0.004606615	1.557019068
AC138466.2	0	0.004606615	1.557019068
AC138466.5	0.420997067	0.000708672	1.664379587
AC138470.1	0.972475639	7.19E-05	1.812696451
AC138625.2	0.018340515	0.001311125	1.993840201
AC138649.2	1.747129544	4.55E-10	2.513393082
AC138749.5	0	0.004606615	1.557019068
AC138869.1	0	0.004606615	1.557019068
AC138869.2	0	0.004606615	1.557019068
AC138869.3	8.88E-05	7.70E-06	22.03573191
AC138904.1	0.13270605	0.001975242	1.609536385
AC138907.3	0	0.004606615	1.557019068
AC138907.4	0	0.004606615	1.557019068
AC138907.6	0	0.004606615	1.557019068
AC138915.2	0.004806698	4.86E-05	2.53425985
AC138932.2	0.116681631	0.00221541	1.589993054
AC138932.3	2.044832067	0.000875004	1.650287987
AC138932.5	2.326713885	1.16E-06	2.060770208
AC138969.1	0.291021183	4.70E-06	1.983427074
AC139100.2	4.241927628	0.000282708	1.733993135
AC139256.1	0.296558375	0.001825463	1.600529677
AC139256.2	2.002194427	0.002527923	1.573992873
AC139495.3	0.91346626	0.001163537	1.632073575
AC139530.4	0	0.004606615	1.557019068
AC139700.1	0.058572571	0.000230574	1.784185893
AC139712.3	0.008172192	0.003311924	2.338650406
AC139713.1	0	0.004606615	1.557019068
AC139769.1	1.802921168	2.38E-05	0.482898201
AC139769.3	0.506778596	0.004834417	0.609411917
AC139783.1	0.475611522	0.003733537	1.564339214
AC140479.2	0.320573888	0.002241035	1.59858328
AC140481.2	0	0.004606615	1.557019068
AC140658.6	0	0.004606615	1.557019068
AC140912.1	0.128183164	2.77E-06	2.049138377
AC141257.1	0	0.004606615	1.557019068
AC141424.1	0.113046941	0.004061015	1.545469725
AC141557.2	1.540565069	0.004902825	1.547387764
AC141586.3	0	0.004606615	1.557019068
AC144548.1	1.055659595	0.003185052	1.559672696
AC144652.1	1.841362736	0.000128596	1.845988614
AC145098.2	0.565910662	4.57E-06	2.005318304
AC145124.1	1.868178038	0.000141775	0.527343554
AC145207.2	0	0.004606615	1.557019068
AC145207.3	0	0.004606615	1.557019068
AC145207.4	0	0.004606615	1.557019068
AC145207.6	0	0.004606615	1.557019068
AC145207.7	0.081197477	0.0005282	1.735715985
AC145207.9	4.003589517	3.27E-06	1.99228685
AC145285.1	0.970997067	0.000153795	1.765644065
AC145285.2	1.645174799	0.00053407	1.684483509
AC145285.3	0.915426006	3.43E-06	1.988129271
AC145285.6	1.285361674	3.82E-06	1.988128127
AC145343.1	0.590306347	7.07E-07	2.093585072
AC145350.1	0	0.004606615	1.557019068
AC145422.1	1.381618162	9.37E-05	1.797186633
AC145423.2	3.5029233	1.37E-06	2.060729011
AC147651.2	48.57620833	0.000389146	0.561672589
AC147651.4	12.21479239	7.30E-06	1.95990179
AC148477.5	0	0.004606615	1.557019068
AC148477.7	0.017285244	0.002522564	2.020808925
AC156455.1	8.24242994	8.48E-06	1.951176384
AC159540.1	0.000439117	0.004571528	5.893547691
AC159540.2	0.027258166	6.15E-05	1.826945262
AC174065.1	0.054854392	1.24E-05	1.965085104
AC174071.1	0	0.004606615	1.557019068
AC187653.1	0.059982748	9.74E-05	1.97705235
AC209007.1	0.453305822	0.000599521	0.54115531
AC211429.1	0	0.004606615	1.557019068
AC211476.2	0.659509742	1.19E-05	1.920898883
AC211476.3	0	0.004606615	1.557019068
AC211476.4	0	0.004606615	1.557019068
AC211486.1	0.084226267	0.003391148	1.564841521
AC211486.4	0	0.004606615	1.557019068
AC226118.1	2.080799169	0.00171558	0.595766082
AC226119.2	0.124862116	8.89E-06	2.045780112
AC232271.1	3.847499992	0.000255009	1.729083389
AC232323.1	0	0.004606615	1.557019068
AC233266.2	1.076435755	0.001538052	1.633700348
AC233280.1	0.455859471	5.10E-06	1.986501063
AC233280.2	1.144759928	0.000833115	1.67333363
AC233724.3	0	0.004606615	1.557019068
AC233724.6	0.24792285	1.57E-06	2.114253594
AC233724.7	0	0.004606615	1.557019068
AC233728.1	2.18865773	0.000227332	1.738532612
AC233992.1	0	0.004606615	1.557019068
AC233992.2	0.20719332	4.02E-07	2.137707139

AC234031.1	0.019151567	0.001602378	1.614518214
AC234582.1	3.310661669	5.29E-05	1.831085426
AC234582.2	0	0.004606615	1.557019068
AC234772.2	0.705340682	2.03E-08	2.559306645
AC234775.3	3.284723648	0.001784708	0.60303989
AC234781.4	0.154705517	0.00010188	1.813839424
AC234782.2	0.252231448	0.003254397	1.563579538
AC234782.3	0.388173141	0.000374405	1.709933055
AC234917.1	0.478851964	0.000802701	1.662419145
AC235097.1	0.011405342	0.002264721	2.282625911
AC236972.3	0.016381227	0.004799249	1.694126435
AC236972.4	0	0.004606615	1.557019068
AC239727.1	0.021192901	0.00285705	1.946173266
AC239802.2	0.05357423	0.001847119	1.657658682
AC239803.2	0.377882077	0.002394817	1.590529896
AC239803.3	1.315769622	0.001241695	1.650790056
AC239868.1	7.805587693	0.002045558	1.593675743
AC241644.3	0	0.004606615	1.557019068
AC241952.1	3.843224226	7.52E-05	1.808877363
AC242376.2	6.307993263	0.000665966	1.668195179
AC243547.2	0	0.004606615	1.557019068
AC243562.2	3.108528348	1.59E-08	2.303388554
AC243571.2	0.218605848	0.000614353	1.684574922
AC243585.2	0	0.004606615	1.557019068
AC243654.2	0.467085386	0.000283053	1.72905185
AC243772.2	0.190676701	0.000776034	1.667455941
AC243829.6	0	0.004606615	1.557019068
AC243830.2	0.680930532	0.000264185	1.731311182
AC243960.2	0.490115192	7.07E-05	0.510798286
AC243960.4	0.130145336	0.00070512	1.806955174
AC243964.3	13.48557479	2.73E-06	2.013787392
AC243967.1	0	0.004606615	1.557019068
AC243967.3	0.071300831	5.58E-05	1.91397897
AC244034.2	0.503727827	3.95E-05	1.855429158
AC244090.3	0.230972406	0.00020519	1.765124348
AC244093.1	0.147945416	0.000841484	1.656377365
AC244093.2	0.226728028	0.001153886	1.674342965
AC244093.4	0.41180304	6.54E-05	1.817821878
AC244093.5	0.608950594	1.95E-05	1.88649222
AC244097.1	0	0.004606615	1.557019068
AC244107.2	0	0.004606615	1.557019068
AC244197.2	2.822328024	2.72E-05	1.8926862
AC244197.3	1.838821873	0.000934328	1.64818805
AC244205.1	0.31179733	0.000546445	1.773466514
AC244394.1	0.052505967	0.004944719	1.577858603
AC244505.3	0	0.004606615	1.557019068
AC244505.5	0.00063657	1.43E-13	56.8714362
AC244505.6	0.000616286	2.24E-05	19.99731918
AC244517.1	0.272280434	0.004959929	1.54286536
AC244517.3	0.343285318	4.02E-06	1.992794717
AC244636.2	0.03055032	0.000837022	1.678883111
AC244669.1	12.39284566	0.003618086	1.559168331
AC245008.1	0.063370346	0.001464516	1.673176907
AC245033.2	0.09767012	0.000198036	1.782631695
AC245033.3	1.588607292	0.004241223	1.548898928
AC245036.5	0	0.004606615	1.557019068
AC245041.1	0.52388595	8.79E-05	2.038508363
AC245041.2	0.34882271	7.81E-05	1.988664081
AC245052.3	0.321835334	0.000287446	1.731926612
AC245052.4	1.111278943	0.002255447	1.593283668
AC245052.7	0.102938514	0.001201123	1.656886852
AC245056.1	0	0.004606615	1.557019068
AC245060.3	0.023182146	0.000143329	1.938972707
AC245060.5	2.106495762	0.001083994	1.63252601
AC245060.6	0.839722782	0.000177092	1.751225664
AC245096.1	0	0.004606615	1.557019068
AC245100.1	0.259645637	4.57E-05	1.849618947
AC245100.2	1.078799937	0.000673633	1.667070246
AC245100.6	0.171906559	0.002802792	2.308209045
AC245100.7	1.369969155	0.000252932	1.73492924
AC245123.1	0.360328115	0.002115117	0.569451917
AC245128.3	1.295721271	0.002245363	1.616293617
AC245884.1	0.878456668	1.20E-05	1.920507709
AC245884.1	0.169051321	0.000238461	1.737581315
AC245884.8	5.439916258	0.000363895	1.707663435
AC245884.9	0.436350589	3.91E-06	1.993908993
AC246785.2	0.228100252	0.003817987	1.554309035
AC246793.1	0.068946105	0.000601882	1.728233234
AC253536.6	3.248022366	0.002006897	1.596783258
AC253576.1	0.111208194	0.001399913	1.763472736
AC253576.2	2.023656546	6.80E-05	1.820782513
AC254629.1	0.485693579	6.22E-08	2.299901962
ACAA2	203.9503699	1.47E-06	0.457166409
ACAD11	7.228362102	3.83E-05	0.488981216
ACAD9	43.87409647	0.003648068	0.640763123
ACADL	43.80624853	0.002174036	0.61396475
ACADM	73.27186198	6.33E-09	0.378051052
ACADSB	35.12385825	2.08E-06	0.450906185
ACAP1	9.83773636	0.000175345	1.76025528
ACAP2	21.89615916	0.002980879	0.625403204
ACAT1	118.6093941	2.48E-05	0.478519926
ACBD3-AS1	1.672416763	0.000798674	1.655418599
ACBD4	34.67285418	0.001916783	0.611569164
ACBD5	52.63372944	0.000177579	0.547054233
ACE	23.46515555	0.002076918	0.593762979
ACE2	98.62421808	9.28E-05	0.516157137
ACER2	8.818011529	2.13E-05	0.505489046
ACHE	6.491345364	0.002663358	1.607327226
ACLY	380.9607893	1.88E-05	0.5004732
ACMSD	86.42764811	0.000817037	0.595527973
ACO1	51.6256968	0.000255071	0.542519569
ACO2	174.9227852	4.71E-06	0.468053557
ACOD1	0.148970222	0.002736311	1.678326017
ACOT13	30.16025046	0.003254201	0.628780031
ACOT4	17.54767907	0.000116781	0.542227438
ACOT8	16.49060725	0.001184523	1.633919973
ACOX1	36.63860411	7.52E-05	0.532182368
ACOX2	13.82403848	0.001634307	0.600564297
ACP1	80.95751984	0.002974762	0.626532112
ACP4	0.275988918	0.00466912	1.538799223
ACP7	0.112748354	0.000987857	1.676819122
ACRV1	0.275436933	0.000140366	1.774882431
ACSL1	182.6106463	0.000143329	0.533737207
ACSS3	16.01728784	0.000321561	0.528090103
ACTB	5157.232588	0.000856171	1.667456321
ACTG1P17	0.977862732	1.36E-08	2.300250686
ACTG1P22	0.128693023	2.57E-06	2.038892992
ACTG1P24	0.3405125	0.00442285	1.542595005
ACTG1P3	1.367521469	0.004500116	1.535859312
ACTG1P4	0	0.004606615	1.557019068
ACTN1-AS1	0.35755883	4.41E-06	2.010103479
ACTN2	1.781389652	0.000620609	1.763094657
ACTN3	0.141368586	0.000587756	1.693449722
ACTN4	398.3116091	0.000127224	0.546742744
ACTP1	0.259936858	0.000341477	1.731315461
ACTR10	53.05285143	8.26E-06	0.494366823
ACTR6	24.1077966	0.003018467	0.630426516
ACTR8	19.48822657	5.87E-05	0.515432163
ACVR2A	10.06668026	2.43E-08	0.397895849
ACVR2B	4.187778366	0.000523333	0.557379751
ACVR2B-AS	3.641633784	1.11E-05	0.469983322
ACVRL1	66.19428204	0.000219276	0.551171553
AD000671.2	0.192314291	0.000983482	1.643028303
AD000813.1	0.076122808	0.002821478	1.593653882
AD000864.2	1.40931353	7.15E-05	1.819090333
AD001527.1	2.085892451	0.000512498	1.691154349
AD001527.2	0	0.004606615	1.557019068
ADA	20.00841216	8.74E-06	1.968712231
ADAM11	0.991615636	1.36E-05	1.917114166
ADAM12	7.120970434	3.53E-06	2.027165805
ADAM1A	0	0.004606615	1.557019068
ADAM32	0.394853025	1.08E-05	1.942652167
ADAM5	0.025313588	0.00023976	1.785707437
ADAM8	10.67846372	3.02E-06	2.031722452
ADAMTS10	14.12750369	0.003244996	1.563187678
ADAMTS13	2.378843183	0.000565525	1.681642882
ADAMTS14	2.822649366	1.18E-10	2.597258343
ADAMTSL3	14.25772323	0.000732543	0.563206172
ADAMTSL4	0.730877309	0.000132444	1.781794341
ADAMTSL5	1.35748548	0.003736446	1.578307966
ADARB2-AS	0.055183632	0.001553796	1.695303949
ADAT2	3.15200096	0.000673206	1.662035741
ADCK5	9.517425823	7.53E-05	1.808081545
ADCY1	3.169633339	0.000985929	0.534228078
ADCY10	0.578232492	0.001604674	1.621477037
ADCY10P1	3.138767409	0.000838952	1.657711864
ADCY5	35.50000759	0.000184429	0.551762685
ADCY9	25.66894284	1.84E-07	0.423281881
ADD1	94.5994963	2.09E-08	0.418222433
ADD2	1.443194384	0.000692007	1.723741093

ADD3	93.36471195	5.99E-06	0.477634182
ADGRB1	2.703676951	2.23E-10	3.109790565
ADGRB2	2.153184933	0.002161137	1.613159177
ADGRD2	0.027403228	0.001477465	1.658360813
ADGRE1	4.709807859	0.004852838	1.556909928
ADGRF5	157.171907	1.24E-05	0.463174796
ADGRG1	180.329294	0.000884453	0.578132251
ADGRG3	1.603603898	1.88E-05	1.988819875
ADGRL2	62.06321158	9.25E-05	0.536013216
ADGRL3	2.813152846	0.000704001	0.538681121
ADGRV1	0.458939858	3.45E-06	0.423234454
ADH5	132.7286693	2.45E-10	0.362074339
ADH5P4	0.579838088	0.001041442	0.580707694
ADH6	7.074501431	0.000766841	0.544042292
ADI1P1	0.035582899	0.001580099	1.80022935
ADIPOR1	170.2939934	0.003147999	0.635651432
ADM5	1.85740198	2.26E-09	2.409361495
ADNP2	20.79198401	0.00017733	0.544856644
ADORA1	6.828765371	0.004058248	1.562232527
ADORA2BF	0.382936129	0.001196184	1.675664278
ADPGK	44.62250081	0.001774794	1.608449071
ADPRH	23.17379212	0.000904801	0.598341342
ADPRHL1	1.357123042	0.004253645	1.603167237
ADRA2A	5.282686437	0.001449063	1.666051741
ADRB2	3.620396345	0.000465039	0.549904636
ADRB3	0.085445676	0.000519756	1.702896931
ADRM1	135.0166537	2.72E-08	2.31209584
AF038458.2	0.218354172	0.000216054	1.759448298
AF111169.1	0	0.004606615	1.557019068
AF117829.1	2.743443685	5.23E-06	1.97389806
AF117829.2	0.11111783	0.000591322	1.722372159
AF121898.1	0.06174978	0.003916736	1.705709457
AF127577.1	0	0.004606615	1.557019068
AF127577.2	0.285436089	0.001146705	1.68742994
AF127936.2	0.362942913	9.91E-05	1.803171983
AF129408.1	0	0.004606615	1.557019068
AF131215.4	0.275003552	0.00455248	1.574812597
AF165147.1	0.254883534	0.00314985	1.562579874
AF186192.3	0.234340665	4.62E-05	1.861710455
AF228730.3	0.00162022	2.73E-05	7.820734454
AF230666.1	0.744715265	0.000369399	1.708211969
AF230666.2	0.205017115	1.11E-08	2.325382694
AF235103.2	0	0.004606615	1.557019068
AF240627.1	0.042354492	2.62E-05	1.97579007
AF254983.1	0.002583467	0.000862492	2.24045789
AF254983.2	0.094024293	0.001434717	2.361771409
AFAP1-AS1	1.689849964	0.000278384	2.026404182
AFAP1L1	42.7066302	0.001882565	0.596719908
AFF1	83.72959542	0.000259509	0.553256522
AFF3	7.655708517	3.70E-05	0.510882351
AFF4	64.7527742	0.000130354	0.543064773
AFG1L	3.552883721	0.000597433	0.572889363
AFG3L1P	4.912192333	0.00029878	1.717989339
AFG3L2	54.13619187	1.26E-08	0.394173232
AFTPH	42.18274145	1.39E-06	0.46217938
AGAP1	18.97419052	0.000920236	0.579351916
AGAP10P	0.42088265	0.000306269	1.726705679
AGAP13P	2.493537614	2.18E-05	1.89493449
AGAP14P	0.436411473	0.000784731	1.678971251
AGAP2-AS1	22.876895	5.00E-05	1.840316537
AGAP3	19.9785563	0.002418522	1.57993733
AGAP4	2.170842996	0.000702718	1.663716489
AGAP5	0.473669829	0.000629128	1.670069764
AGAP6	7.509991602	2.81E-07	2.138726855
AGAP7P	0.644939258	0.004304115	1.587897143
AGAP9	6.762479391	3.78E-05	1.850272284
AGBL1-AS1	0.027834178	0.00167069	1.672804201
AGBL2	2.249010941	2.80E-05	1.867260414
AGER	13.89267234	0.000156006	1.758708026
AGGF1	34.68756854	3.30E-06	0.48152765
AGGF1P1	0.145707788	0.000715324	1.673916601
AGGF1P2	0.71048253	7.59E-05	1.820269312
AGGF1P3	0.359042614	0.003068306	1.581461756
AGL	18.89513177	4.89E-05	0.507975076
AGMAT	58.15641235	2.74E-05	0.493154405
AGO1	15.59223676	1.84E-06	0.46345168
AGPAT3	56.35878628	0.000619546	0.558929628
AGPAT5	47.81068677	0.000574254	0.573410332
AGPS	42.60963457	3.71E-05	0.520764897
AGR3	1.153269028	0.002540697	1.720621895
AGRP	0.58634885	0.001617367	1.613853583
AGTR1	19.04253523	0.000248853	0.522142358
AGXT2	64.63588406	6.99E-05	0.517160353
AHCYP8	0	0.004606615	1.557019068
AHI1	6.953640486	0.000788181	1.653087817
AHNAK	323.948666	0.000295698	0.54541752
AHR	69.1868385	0.000553356	0.57412375
AHSA2P	25.75400757	5.43E-05	1.824025948
AIDA	33.20989784	0.00010243	0.547933702
AIFM1	112.5421572	0.004461702	0.630074607
AIFM3	1.175919886	6.98E-05	1.821047595
AIG1	68.11649848	0.002552684	0.627477936
AIM2	5.392298494	0.00071148	1.686388771
AIMP1	59.42289014	0.000817084	0.592028168
AIRE	0.161592231	1.07E-05	2.016603463
AJ003147.1	0.094890965	0.00057248	1.731736449
AJ003147.2	0.097817176	4.31E-06	2.073095544
AJ003147.3	0.884152022	0.000190235	1.746003208
AJ011931.1	0.034418868	0.000294839	1.919754704
AJ239318.1	0.448530104	0.00486299	1.903845478
AJ239322.1	0.038611214	2.09E-05	1.958869584
AJ239328.1	0.115320321	6.65E-06	2.151122509
AJAP1	3.963074273	4.61E-07	0.381875305
AK3	64.61887351	0.000260387	0.546790542
AK4	114.0265212	0.00343271	0.637543041
AK4P1	5.167158532	1.40E-05	0.491550947
AK6P1	0.07981102	0.003271558	1.621903297
AK7	3.846948466	0.000476788	0.569336973
AK8	6.437865956	0.002373054	0.613161775
AKAP1	44.3873384	0.001600221	0.600115973
AKAP10	29.89074744	0.003547003	0.636156132
AKAP11	32.97504239	6.78E-05	0.523596286
AKAP12	108.9464887	0.0025328	0.591433909
AKAP13	35.2514421	0.000117673	0.536380303
AKAP17A	43.61652318	0.001396786	1.617285382
AKAP2	1.068107801	4.57E-05	0.49981615
AKAP6	3.372133756	8.60E-06	0.475100986
AKAP7	11.81061158	9.88E-05	0.534814614
AKAP8L	31.36856036	0.000187503	1.749708614
AKAP9	17.79090123	0.00243996	0.598578319
AKIRIN1P2	0.122009334	0.002463392	1.646600462
AKNAD1	0.110585096	9.59E-08	2.230157801
AKR1B15	0.458588228	0.0010363	2.078840934
AKR1C1	16.29120186	0.001187527	0.546684953
AKR1D1P1	0.051776124	0.001927797	1.598175466
AKR7A2	152.3250163	9.61E-05	0.536756047
AKR7A3	42.09567064	0.001631984	0.587263527
AKT1S1	38.11858167	0.000174448	1.766847416
AKT3	37.09318849	5.29E-07	0.434451724
AKTIP	28.73000898	5.14E-05	0.518357441
AL008582.1	2.382255708	3.90E-06	1.984322498
AL008635.1	1.341107477	0.001731466	1.610129683
AL008638.6	0.033241032	6.74E-05	1.835260676
AL008718.3	0.731641625	0.000777672	1.662404832
AL008726.1	1.123832247	4.27E-05	1.880313265
AL008727.1	0.094202189	0.000166442	1.758982936
AL008729.1	1.613319098	0.000180454	1.747557796
AL008729.2	0.506761518	8.41E-05	1.804586829
AL009050.1	0.068855963	0.001521297	1.727206217
AL009181.1	0.27595029	0.001001245	1.643848554
AL020996.3	0	0.004606615	1.557019068
AL020997.2	1.013159403	4.12E-05	1.878824099
AL021153.1	0.117591588	0.003122229	1.65224635
AL021154.1	1.824172686	0.001102654	1.640818865
AL021368.1	0.506028184	0.001147549	1.643320289
AL021368.3	0.865043957	0.000370324	1.713832928
AL021391.1	0.14193167	0.000591451	1.694557424
AL021392.1	1.913498104	3.15E-05	1.866855166
AL021393.1	0	0.004606615	1.557019068
AL021395.1	0.010646307	1.40E-05	2.109832449
AL021707.2	1.940174826	0.001899604	1.591017842
AL021707.6	8.074343931	1.11E-05	1.917732707
AL021707.8	6.281934926	1.58E-05	1.895309952
AL021807.1	1.39762335	0.000121366	1.788345258
AL021877.2	0.010205168	0.003496064	1.594371669
AL022067.1	1.128748444	1.18E-06	2.070316738
AL022237.1	0.120388203	0.000256088	1.737431427
AL022238.2	0.230914125	0.00312799	1.579997493
AL022238.3	1.609007996	0.000263037	1.728801861
AL022310.1	0.118855719	0.002891478	1.575296267

AL022311.1	0.539634692	9.03E-06	1.935064246
AL022318.2	0.199851947	0.00249786	1.59139575
AL022322.1	5.931548047	0.000851819	1.65266566
AL022322.2	0.08793618	0.001224057	1.634316235
AL022323.1	2.733250613	1.34E-05	0.467576495
AL022326.1	0.158299226	0.004204583	1.576109008
AL022328.3	3.249547456	9.58E-05	1.792953917
AL022328.4	2.947382671	0.003234291	1.562395601
AL022334.1	0.109793283	5.06E-05	1.861668113
AL022341.1	2.002832537	0.004723512	1.534291332
AL022341.2	0.442488182	2.05E-12	2.799139415
AL022400.1	0.078327251	0.002426623	1.608649167
AL022724.1	0.739555837	0.004774579	1.580176645
AL023584.2	0.532133523	4.10E-05	1.905302071
AL023653.1	2.257628378	0.000230441	1.741983564
AL023754.1	0.054700612	1.03E-06	2.1179186
AL023803.2	0.61630048	2.25E-07	2.163771592
AL023803.3	0.204064723	0.000121727	1.801577119
AL023806.2	0	0.004606615	1.557019068
AL023806.3	0.244795673	0.000317962	1.720640091
AL024474.1	0.126001535	2.15E-06	2.05943177
AL031186.1	2.610759067	4.81E-05	1.834112129
AL031280.1	0.166027299	2.48E-08	2.289569458
AL031283.3	0.304066266	0.000519723	1.692674964
AL031386.1	0	0.004606615	1.557019068
AL031575.1	0.05012453	0.000762279	1.77655567
AL031577.1	1.160819777	0.000114276	1.806795927
AL031577.2	0.221317514	0.000899313	1.673832579
AL031587.1	0.191854255	0.001879409	1.605560925
AL031587.3	0	0.004606615	1.557019068
AL031595.2	0.07042039	4.08E-05	1.858680655
AL031595.3	0.261658893	0.001799823	1.608158747
AL031600.1	3.496141142	0.000660953	1.666946147
AL031600.2	0.248217733	0.001796633	1.615416427
AL031600.3	0	0.004606615	1.557019068
AL031602.1	1.073133276	0.004279963	1.554539828
AL031651.1	0.116810368	2.36E-05	1.892157956
AL031659.1	0.131473985	0.002138907	1.604833237
AL031674.1	0.05137659	4.67E-06	2.066111405
AL031705.1	0.936706867	0.000558706	1.675660098
AL031710.2	0.660870513	0.001862342	1.618344999
AL031714.1	3.431905547	0.002279059	1.579999878
AL031727.1	12.04071139	2.70E-05	1.877571791
AL031728.1	0.472007666	8.96E-06	1.938029202
AL031733.2	0.492744087	0.000126264	1.790417923
AL031736.2	0.201483237	0.004028747	1.551229952
AL031767.1	0.072740023	0.001779935	1.732732975
AL031768.1	1.070562858	0.002800906	1.572942685
AL031770.1	0.211408944	0.000236549	1.753873752
AL031777.1	1.724022071	0.000113651	1.818869967
AL031848.2	0	0.004606615	1.557019068
AL031864.1	0.543045767	0.002643935	1.580882756
AL031963.1	0	0.004606615	1.557019068
AL032819.2	0.506066893	0.001556847	1.61975803
AL033379.1	0.008616613	0.004547418	2.277955958
AL033380.1	0.14017471	0.002853295	1.605597531
AL033397.2	1.153792024	0.000525156	1.687143524
AL033527.2	0.093186346	0.001226845	1.639052889
AL033527.3	0.550677239	8.34E-06	1.961543387
AL033529.1	0.015198075	1.87E-05	1.912447858
AL034345.2	0.116674658	7.34E-05	1.826924205
AL034346.1	0.075421801	2.92E-05	2.076480254
AL034369.1	0.005812797	9.68E-05	3.490535331
AL034380.1	0	0.004606615	1.557019068
AL034397.1	0.279339675	1.93E-07	0.414812988
AL034397.3	3.991474109	0.00241108	1.590399845
AL035071.1	14.81733517	2.65E-05	1.878844933
AL035250.1	0.120204873	0.000376807	1.711580399
AL035252.3	1.227368654	1.61E-05	1.903431297
AL035405.1	0.503701018	0.004861485	1.603949398
AL035411.1	1.703809732	0.000150769	0.529504378
AL035413.1	14.09883056	8.05E-06	0.473951505
AL035417.2	0.097935111	3.56E-05	1.872428677
AL035446.1	2.926318665	0.004309674	1.567439042
AL035460.1	0.013399071	2.69E-05	2.061097737
AL035461.2	4.212324626	2.23E-11	2.665305574
AL035706.1	0.301014743	0.000859697	1.682936449
AL049539.1	0.680691664	5.49E-06	2.000053926
AL049612.1	1.42441358	0.002884697	1.580321595
AL049634.1	0.228951224	0.002929619	1.56921399
AL049712.1	0.262797147	0.000264577	1.731892522
AL049747.1	0.159450709	0.001706377	1.68576517
AL049749.1	0.070247214	0.000507746	1.782761686
AL049776.1	1.699117334	0.002451782	1.576464173
AL049780.1	2.372721673	0.001684346	1.60885183
AL049794.1	0.357619427	0.000118395	1.785013207
AL049795.2	0	0.004606615	1.557019068
AL049829.2	0	0.004606615	1.557019068
AL049830.3	0.096568276	0.004886972	1.603951816
AL049833.2	0.016348949	0.000702588	1.768444064
AL049836.1	0.315457966	2.99E-05	1.906457038
AL049836.2	0.265987922	0.004691618	1.764371609
AL049839.2	0.199843914	0.000102419	1.921366879
AL050303.1	0	0.004606615	1.557019068
AL050303.3	0	0.004606615	1.557019068
AL050320.1	0.372912011	0.002300661	1.607676075
AL050327.1	0.292032227	5.13E-07	2.231894645
AL050331.2	0	0.004606615	1.557019068
AL050335.1	0.063901284	0.00413225	1.5913891
AL078581.1	5.866273459	0.000621554	0.563403292
AL078587.1	1.084393289	0.00013532	1.776446511
AL078595.2	0.04889459	0.000111692	1.87127947
AL078603.1	0.039272446	0.000111027	2.134006362
AL078604.2	1.463281578	1.86E-08	2.317470684
AL078604.3	0.784953152	9.10E-05	1.811954521
AL078605.1	0.359535294	0.000947858	1.672941193
AL078612.1	0	0.004606615	1.557019068
AL078623.1	0.04352893	0.000278141	1.808890598
AL078624.2	0.032753398	1.21E-05	2.322763019
AL078644.1	0.195870604	0.000110446	1.79738936
AL078644.2	2.27124501	0.000311722	0.559133783
AL080248.1	8.471546734	0.000121771	0.517775856
AL080317.1	45.47782416	0.00340276	1.57350834
AL080317.2	2.213860718	1.87E-06	2.044047336
AL096701.1	1.590558072	5.01E-09	2.37101553
AL096701.2	0.183437972	0.000471627	1.743224235
AL096701.3	1.258763171	2.17E-05	1.885252477
AL096711.1	0.053589368	4.02E-05	1.872365499
AL096803.2	0.451213816	2.01E-05	1.899752932
AL096803.3	0.23584002	0.001098987	1.651269397
AL096814.1	0	0.004606615	1.557019068
AL096840.1	0.116084401	0.002325765	1.856288921
AL096855.1	0.479390672	0.000136673	1.782552598
AL096865.1	1.693018747	4.61E-09	2.371673206
AL109610.1	0.013403162	0.000193008	2.507104464
AL109613.1	8.820615634	0.004493049	0.630970513
AL109653.2	0.044792421	1.67E-05	1.972797074
AL109659.3	0.260115742	0.002887447	1.576373351
AL109741.1	9.224996688	0.001172952	0.590226089
AL109761.1	0.378831841	2.54E-05	1.884098557
AL109811.1	0.405118633	2.83E-06	2.004896625
AL109811.2	10.34527055	0.002742612	1.571384246
AL109827.1	0.006653152	0.004329395	0.326539336
AL109918.3	0.253570003	0.003526262	1.570911096
AL109923.1	0.246779271	0.001243503	1.662099298
AL109933.4	0.332667963	0.00442572	0.576789692
AL109983.1	0.297546991	7.48E-06	1.965803507
AL110115.2	0.666888804	2.45E-05	1.882638379
AL110504.1	0	0.004606615	1.557019068
AL117190.2	0	0.004606615	1.557019068
AL117209.1	2.634678051	0.002813728	1.575230427
AL117328.2	0.052619701	2.84E-06	2.01381971
AL117329.1	0.031073416	0.004452434	1.744459574
AL117332.1	2.605988728	0.001024682	1.64456615
AL117336.1	6.010474496	6.74E-06	1.957289843
AL117339.1	0.126463797	9.12E-05	1.798461799
AL117339.2	0.432653036	1.41E-05	1.916136707
AL117339.4	0.516005025	2.26E-06	2.010654834
AL117344.1	1.031252462	5.95E-06	1.960724183
AL117379.1	4.608642094	8.49E-05	1.806717003
AL117381.1	1.251687286	0.00383142	1.583654296
AL117692.1	0.064211927	0.001339875	1.624827582
AL118508.1	0.273146824	0.000795484	1.75908913
AL118508.2	0.131502195	0.000153074	1.775622946
AL118511.1	0.439312499	0.004779078	1.539676519
AL118516.1	16.35547488	0.001630415	1.612779603
AL121594.1	0.215260337	5.87E-06	1.969624549
AL121601.2	0.288545927	1.70E-05	1.913420194
AL121652.1	0.296051463	0.003355062	1.569834619
AL121672.3	0.494568599	0.004343068	1.542922452

AL121748.1	1.280231305	0.002192444	1.596788126
AL121749.1	0.057627753	9.27E-05	1.814348517
AL121772.1	0.677810154	1.80E-10	2.553915175
AL121782.1	1.07536574	6.00E-07	2.10277163
AL121832.3	0.572947417	0.000106397	1.783911537
AL121835.2	0.045799225	0.000895434	1.666232587
AL121839.1	0.066294845	8.24E-05	1.854706945
AL121845.1	0	0.004606615	1.557019068
AL121845.3	1.102407425	6.53E-06	1.962098227
AL121852.1	0.55183873	8.33E-05	1.806804447
AL121890.1	0.082081759	0.001141659	1.914112517
AL121890.2	0.399951603	0.000179212	1.765218812
AL121890.5	0.957891948	5.02E-06	1.975717378
AL121894.2	2.430556192	0.000674152	1.673878698
AL121906.2	0.677461023	0.000742637	1.672378779
AL121917.2	0.444686499	0.00026582	1.754846365
AL121928.1	0	0.004606615	1.557019068
AL121949.2	0.083711172	0.003733057	1.585374622
AL121952.1	0.290969877	0.002333835	1.669988067
AL121989.1	0.261443686	7.66E-05	1.869243477
AL121992.2	0.39002206	0.001323071	1.648531757
AL132655.2	0.522985532	1.02E-06	2.09990717
AL132655.3	0.006654881	0.000197849	2.134566124
AL132656.1	0	0.004606615	1.557019068
AL132709.1	0.136618038	4.82E-06	2.719617827
AL132765.2	0.415307957	6.22E-07	2.109704429
AL132780.3	0	0.004606615	1.557019068
AL132780.5	0.594549508	0.000743657	1.659047186
AL132796.2	0.032431174	0.002173069	1.627743142
AL132800.1	2.243783322	4.42E-05	0.503924089
AL133153.1	0.247670909	0.002010812	1.597485305
AL133215.2	0.474270544	5.05E-07	2.116071419
AL133216.2	5.241883903	7.86E-06	1.955915009
AL133230.1	1.201896621	9.52E-05	1.807622835
AL133243.3	1.133709495	5.47E-08	2.229888453
AL133245.1	0.222360065	5.84E-05	1.825881168
AL133255.1	0.735594681	9.15E-06	1.945763423
AL133325.3	0.151725614	4.04E-06	2.244392641
AL133330.1	0.879812471	4.70E-05	1.877213183
AL133352.1	0.280220997	0.004228784	1.539043859
AL133355.1	9.0271557	4.94E-05	0.525321814
AL133372.2	0.012354057	0.003251367	1.69794533
AL133373.1	0.180401694	0.002269041	1.757732214
AL133373.2	0.14019355	0.000267234	1.769319377
AL133375.1	0	0.004606615	1.557019068
AL133387.2	0.077601992	0.00015325	1.881514851
AL133387.3	0.045206785	0.001727637	1.719246991
AL133396.2	0.238376385	7.04E-06	1.964767296
AL133406.2	1.277344342	0.000388543	1.702621609
AL133410.1	2.205837553	0.000323697	1.71669246
AL133410.2	0	0.004606615	1.557019068
AL133445.2	0.904083311	0.001805578	1.682033463
AL133453.1	0	0.004606615	1.557019068
AL133465.1	0.018996462	0.000240062	2.167744372
AL133492.1	0.054527596	1.27E-06	2.107849414
AL133517.1	2.745250371	1.78E-06	2.043881008
AL135746.1	0.049742704	0.000273827	1.868041912
AL135786.1	1.872675776	0.001228632	0.58312148
AL135818.2	0.721397947	5.33E-05	1.836622016
AL135923.2	0.13121434	3.46E-06	2.06667908
AL135999.1	3.856991069	3.82E-05	1.848659428
AL136038.2	0	0.004606615	1.557019068
AL136084.2	0.105815678	0.002383983	1.585999861
AL136099.1	0.099614451	5.94E-06	1.973161601
AL136115.1	0.556120972	5.59E-06	1.968116764
AL136115.2	0.854689205	2.00E-10	2.521804277
AL136131.3	0	0.004606615	1.557019068
AL136228.1	0.08197725	0.002050215	1.738071336
AL136295.2	2.685492546	0.002620152	1.57528257
AL136295.5	0.504412339	0.001801056	1.600607231
AL136295.6	5.305121857	0.001401136	1.617839069
AL136360.1	0.047193475	0.000443563	1.707109981
AL136368.1	2.372656905	0.004202049	1.540355494
AL136380.1	2.370330005	0.000202966	1.752365829
AL136528.1	1.51185996	0.00067625	1.860802064
AL136529.1	0.44629584	0.002937843	2.447200872
AL136981.2	4.59308931	0.003816604	0.613106531
AL136982.4	0	0.004606615	1.557019068
AL136982.7	0	0.004606615	1.557019068
AL136987.1	0.311701286	0.003142448	2.018579858
AL136988.1	0	0.004606615	1.557019068
AL137005.1	0.06513833	5.57E-05	1.85978499
AL137022.1	0.049666768	0.00023385	2.038228146
AL137025.1	0.202948414	0.000123335	0.509561989
AL137060.1	0.425970153	0.002158093	1.596826077
AL137072.1	0.035122879	0.001515637	1.93938303
AL137127.1	1.724163236	4.50E-08	2.249206835
AL137186.2	3.816935767	9.25E-05	1.798330589
AL137230.2	0	0.004606615	1.557019068
AL137779.3	0.131972656	0.001131037	1.693587676
AL137847.1	2.097379515	0.004164992	0.628503533
AL137856.1	0.217325684	0.000128761	1.782178984
AL137918.1	0.484085846	0.001754161	1.605632593
AL138478.1	0	0.004606615	1.557019068
AL138688.1	0.033356889	0.002778662	1.575806235
AL138724.1	3.74214143	0.000406369	1.70567048
AL138733.1	0.187373084	0.000347898	1.729228768
AL138760.1	0.169287422	3.84E-07	2.158235246
AL138767.1	0.058090163	0.004399794	1.552796663
AL138820.1	0.339132322	0.000225729	1.743996151
AL138826.1	20.73311304	0.001378971	0.554214453
AL138828.1	0.186702618	0.000522051	1.71464673
AL138831.3	2.263761953	9.10E-09	2.331193374
AL138847.1	0.238472252	1.11E-05	1.932370948
AL138847.2	1.649973293	0.003425233	0.472240671
AL138880.1	0.069881727	0.004180031	1.581287373
AL138889.1	0.020004145	2.03E-05	1.903061171
AL138916.1	0.110329316	3.19E-05	1.878558238
AL138921.1	1.076121987	9.35E-05	1.796086311
AL138960.1	1.118747867	4.61E-05	1.868157277
AL138960.2	0	0.004606615	1.557019068
AL138962.1	0.283217462	3.88E-05	1.951098924
AL138963.3	0	0.004606615	1.557019068
AL139041.1	0.83955221	0.000913399	1.648932377
AL139089.1	2.907668633	0.000340794	1.716645265
AL139099.1	0	0.004606615	1.557019068
AL139123.1	1.533756384	4.33E-06	1.980733116
AL139132.1	0.369081691	0.003952582	1.549590494
AL139220.1	0.076714116	0.000172818	1.793305464
AL139220.2	0.205089222	0	3.432918367
AL139260.3	0.16587128	0.001066442	1.638586744
AL139274.2	0.576010063	0.004791918	1.53671154
AL139286.2	1.535459673	4.15E-07	2.116107403
AL139287.1	23.62232084	0.002656946	1.575372126
AL139289.1	1.956600157	4.25E-05	1.83868979
AL139317.3	0	0.004606615	1.557019068
AL139327.1	0.223274963	8.59E-05	2.383001161
AL139327.2	0.054864712	0.00116999	1.869376254
AL139349.1	7.097780527	5.33E-05	1.84489034
AL139351.1	2.650497595	3.80E-06	2.006687022
AL139352.1	0.858603714	7.37E-05	1.82274159
AL139354.1	0.16461847	0.003510406	1.597372934
AL139384.2	1.578909615	0.002539617	0.612014692
AL139397.2	0.106230825	0.000295586	1.734025713
AL139412.1	0.238284812	0.002765454	1.591556826
AL139415.1	0.383325428	0.001354176	1.638450174
AL139420.1	0.234542219	0.002402708	1.628268017
AL139421.1	1.547786338	0.000535583	1.683998641
AL157378.1	0.073010237	0.003420557	1.770532436
AL157385.1	0.33983508	0.000164447	1.792422549
AL157387.1	0.073426413	0.000144422	1.995731135
AL157392.4	2.220450304	0.002398035	1.582987961
AL157394.1	4.302076514	9.24E-05	1.805472226
AL157400.5	0	0.004606615	1.557019068
AL157402.2	0.226763277	0.001849292	1.738655172
AL157756.1	0.435099079	0.000898387	1.665390238
AL157762.1	0.682540667	0.000536636	1.680867401
AL157791.2	1.865176135	0.000255534	1.739465659
AL157823.1	0.129152706	0.004336746	1.599421308
AL157823.2	0.728668327	8.17E-05	1.819976293
AL157838.1	2.176204662	0.000420719	1.70119097
AL157871.1	0	0.004606615	1.557019068
AL157871.2	2.072205544	0.000164665	1.761505531
AL157871.4	0	0.004606615	1.557019068
AL157884.1	0.037233912	0.004423748	1.674194467
AL157896.1	0.363231179	0.000511718	1.732009065
AL157935.1	1.574273455	0.004311651	1.723616565
AL157935.2	1.78508925	9.81E-06	1.930054231
AL157937.1	0.15333891	0.002521822	1.605561959
AL157938.3	0.251955423	7.98E-05	1.815433837

AL158032.2	0	0.004606615	1.557019068
AL158063.1	1.354769813	0.000213515	1.747568601
AL158147.1	0	0.004606615	1.557019068
AL158151.3	0	0.004606615	1.557019068
AL158151.4	1.317345639	8.26E-12	2.7109017
AL158152.1	0.761919344	0.003382526	0.591714313
AL158154.1	0	0.004606615	1.557019068
AL158154.2	0.082116348	0.00179142	1.610116755
AL158196.1	0.987025384	8.66E-05	1.801237
AL158201.1	0.827949518	0.000129344	0.515862271
AL158209.1	0.040345507	0.000338388	2.214817512
AL158211.2	0	0.004606615	1.557019068
AL158212.2	1.866430809	1.27E-06	2.056112121
AL158825.1	0.104801057	7.64E-05	1.895928829
AL158825.2	0.623500789	0.000715029	1.67491906
AL158834.2	1.175560594	8.71E-08	2.207033697
AL159163.1	1.7796405	5.82E-05	1.834880353
AL159169.2	1.739958358	0.000150597	1.756362173
AL159169.3	0.477148738	7.96E-05	1.809924235
AL159990.1	0	0.004606615	1.557019068
AL160004.1	0	0.004606615	1.557019068
AL160236.1	0	0.004606615	1.557019068
AL160275.1	0.128668293	0.00067748	1.682729753
AL160275.2	0.077820488	4.81E-06	2.089469097
AL160281.1	0.224631441	6.68E-07	2.149126709
AL160290.1	0.173993414	5.12E-05	1.836889778
AL160314.2	0.539301937	0.002211915	1.588763192
AL160408.2	0	0.004606615	1.557019068
AL160411.1	0.030895686	0.000638427	2.056746947
AL161431.1	1.534873131	7.33E-08	2.921558829
AL161452.1	1.065212919	4.79E-06	1.974122989
AL161618.1	0.020081658	0.000431194	0.377025106
AL161638.2	0.00734558	0.004177531	2.294955862
AL161668.2	0	0.004606615	1.557019068
AL161668.3	0.838343297	0.000243921	1.734973951
AL161669.2	7.074364197	0.000841606	1.691182973
AL161669.3	9.367750046	0.00170766	1.624032128
AL161733.1	0.148035959	0.000311249	1.815267267
AL161747.2	0	0.004606615	1.557019068
AL161756.1	0.278730857	3.33E-06	1.996108259
AL161757.5	0.046895695	0.000662423	1.718104756
AL161782.1	2.06359793	6.62E-10	0.336681718
AL161908.1	0.09740553	0.004390922	1.678028287
AL161935.1	1.228899743	0.000816568	1.65626106
AL161937.1	0.107473349	0.002345626	1.584027377
AL162151.1	0.04716649	0.000370209	1.834842212
AL162151.2	13.93242978	0.000134855	0.514662404
AL162171.1	12.72549246	0.000142698	0.527433296
AL162231.2	9.503907521	0.000304943	0.551857384
AL162233.1	0.043552122	0.004621156	1.570100257
AL162293.1	0.070334487	3.72E-05	1.924089002
AL162377.1	2.559280933	0.000376513	0.563299425
AL162390.1	0.268692322	0.003849689	1.570246655
AL162413.1	0.592889527	9.99E-06	2.418036876
AL162430.2	1.078867828	0.004446988	1.537013037
AL162457.2	0.141509814	0.000315222	1.901967389
AL162586.1	5.814755141	3.89E-05	1.848631988
AL162591.2	0	0.004606615	1.557019068
AL162741.1	0.967814541	0.000139167	1.789414185
AL162742.1	0	0.004606615	1.557019068
AL163051.1	0.95181297	0.000130917	1.807295138
AL163636.2	2.04440156	5.34E-05	0.517680175
AL163952.1	0.194190507	8.96E-05	1.816872792
AL352979.2	0	0.004606615	1.557019068
AL353132.1	0.014541056	0.002507377	1.664609707
AL353150.1	1.196399269	8.37E-09	2.414188992
AL353194.1	4.288255497	0.000819647	1.654789631
AL353588.1	0.301889845	0.000178994	1.761588176
AL353612.1	0.031945386	0.000243616	2.037449846
AL353621.1	1.670627698	0.001779838	0.567541256
AL353637.1	0.475503712	0.000254477	0.437178384
AL353662.1	0.268897018	0.002708082	1.635590944
AL353689.2	0	0.004606615	1.557019068
AL353708.3	1.283428627	0.000115299	1.78565659
AL353729.1	0	0.004606615	1.557019068
AL353742.1	0.008893817	0.003083031	3.512439578
AL353763.2	5.553115164	0.000381624	1.704097271
AL353764.1	1.361435384	0.000419293	1.706888616
AL353770.1	0.00034262	5.40E-08	31.65564918
AL353770.2	0	0.004606615	1.557019068
AL353801.3	1.636658144	0.000239199	1.743049353
AL353804.2	0.960148691	7.69E-06	1.953507596
AL353807.3	0.753112447	7.60E-05	1.811889436
AL353807.4	3.127630899	0.00022771	1.735980262
AL353813.1	0	0.004606615	1.557019068
AL353898.2	0	0.004606615	1.557019068
AL353898.3	1.49194671	1.06E-05	1.927893857
AL353997.1	0	0.004606615	1.557019068
AL353997.3	0	0.004606615	1.557019068
AL353997.4	0.006270593	0.004750655	3.341192196
AL354692.1	0.140241668	0.000527248	1.762110464
AL354714.3	0.20934871	0.001752171	1.986800029
AL354740.1	0.140032443	0.001982119	1.595507369
AL354751.1	0.568732315	1.72E-05	2.006643649
AL354751.2	1.157002503	0.00338704	1.601097094
AL354760.1	1.606664871	8.70E-06	1.946099432
AL354766.1	0.029351011	0.002615456	1.915896832
AL354798.1	1.57583608	7.51E-08	2.213950648
AL354813.1	0.185133898	0.000130242	1.798153332
AL354836.1	20.9083492	0.000949345	1.649601151
AL354877.1	1.037679004	0.000627888	1.681493374
AL354892.3	1.521437542	0.000500659	1.679945465
AL354919.2	0.225528123	1.42E-05	1.931239493
AL354920.1	8.390190623	0.001027638	0.594558091
AL354943.1	0.116288691	0.002754115	1.649202475
AL354950.2	0	0.004606615	1.557019068
AL354956.1	0	0.004606615	1.557019068
AL355075.2	2.988938997	0.003727539	1.546863802
AL355075.3	0	0.004606615	1.557019068
AL355076.2	0.10655398	0.001043177	1.665178991
AL355096.1	0.130750118	2.44E-06	2.161148392
AL355140.1	0	0.004606615	1.557019068
AL355306.1	0.032439544	0.003546947	1.766930198
AL355309.1	2.184503156	0.004788608	1.543443456
AL355310.1	0	0.004606615	1.557019068
AL355310.2	0	0.004606615	1.557019068
AL355310.3	0	0.004606615	1.557019068
AL355355.2	5.261560449	0.002280528	0.620328146
AL355375.1	0.151063375	0.002177697	1.62684632
AL355385.1	1.344004645	0.00010518	1.793669413
AL355388.2	1.324677741	6.53E-10	2.468449749
AL355472.3	1.091211249	0.001511755	1.641216661
AL355483.1	0.086816638	3.80E-06	2.216825642
AL355488.1	7.615719901	0.001063677	1.634113885
AL355490.2	0.237071636	3.93E-07	2.130102655
AL355598.1	0.1834433	0.000227852	1.835835744
AL355601.1	0.047047646	0.000397519	1.882616995
AL355607.2	0.080632587	0.000326507	1.730419469
AL355674.1	0.002363845	0.004715405	3.048934122
AL355796.1	1.546534318	6.59E-08	2.301368128
AL355802.1	2.629386507	9.09E-05	1.803438093
AL355802.2	1.574207157	0.000810494	1.659578449
AL355816.1	0	0.004606615	1.557019068
AL355816.2	0	0.004606615	1.557019068
AL355836.1	0.026746747	0.000597924	1.901155043
AL355838.1	0.026214464	0.000171522	2.128882647
AL355864.2	0.659383918	0.000593005	1.687962589
AL355922.1	0.83345466	0.002221662	1.593702865
AL355987.5	0.433566425	8.56E-07	2.085234583
AL355994.3	0	0.004606615	1.557019068
AL356010.1	0.075969936	0.004928304	1.879073022
AL356017.1	0	0.004606615	1.557019068
AL356055.1	0.453750376	6.84E-08	2.232539942
AL356056.1	0.030480714	7.19E-06	1.970760231
AL356094.1	0.024710005	0.001489659	1.702783566
AL356095.2	0	0.004606615	1.557019068
AL356124.2	0.715097989	0.000682048	1.674628325
AL356215.1	0.416411374	3.12E-09	2.553697742
AL356272.1	0.123691226	0.001271068	1.650272352
AL356273.3	5.89859297	1.06E-05	0.471821727
AL356277.1	0.070542802	0.000170033	2.092804839
AL356277.2	0.149567563	6.04E-05	2.172219511
AL356277.3	0.056087616	1.64E-08	2.457421336
AL356292.1	0.239688923	0.000929776	1.656990084
AL356299.2	1.109559565	2.56E-05	1.87758448
AL356356.1	3.384963883	3.71E-06	1.984019236
AL356414.1	0.093729712	1.32E-08	2.391190816
AL356417.1	0.148028617	0.001001096	1.656966993
AL356417.2	0.358181712	0.004111489	1.563796936
AL356473.1	0.037704447	0.000104317	1.986972042

AL356488.1	0.479722437	0.00498802	1.560713082
AL356515.1	0.472750314	2.64E-05	2.031332643
AL356750.1	0.335375083	0.000205075	1.754361971
AL356967.1	0.006777556	0.002405708	2.846300561
AL357055.3	0.98101383	0.003700548	1.550498319
AL357078.1	0.59062583	0.004289034	0.621594733
AL357140.1	0	0.004606615	1.557019068
AL357140.2	2.440682189	2.62E-07	0.43194393
AL357140.3	0.111155922	2.52E-05	1.911960982
AL357153.1	0.027589431	0.001568475	1.646481576
AL357153.4	0.120569379	0.004499994	1.627603633
AL357497.1	0.447024733	9.16E-05	1.806910364
AL357507.1	0.929974643	3.43E-06	2.709132732
AL357556.1	0.126922125	0.000299072	1.781721823
AL357992.1	1.693333038	3.90E-08	2.269178997
AL358072.1	0.959787128	0.000330686	1.714230702
AL358154.1	0.048406426	0.000106135	1.962557827
AL358215.1	0	0.004606615	1.557019068
AL358333.3	0	0.004606615	1.557019068
AL358334.3	0.683379666	0.001059687	1.639002402
AL358472.3	4.431442975	7.05E-06	1.958517865
AL358613.2	0.157188333	5.22E-07	2.215017219
AL358781.1	0	0.004606615	1.557019068
AL358876.2	0.098817626	0.004302087	1.547884693
AL359091.2	0.167439817	4.27E-07	2.127696649
AL359091.3	0	0.004606615	1.557019068
AL359091.5	3.054631219	0.000381489	1.714835129
AL359092.1	0.53117975	0.004293847	1.558324405
AL359094.1	0.106196328	8.68E-07	2.086336389
AL359094.2	0	0.004606615	1.557019068
AL359237.1	0.076428069	0.003712831	1.603783295
AL359313.1	0.17338968	3.78E-06	2.143194702
AL359397.2	0	0.004606615	1.557019068
AL359504.2	2.513713692	0.000149993	1.769581226
AL359510.2	1.02616881	2.75E-05	1.871038303
AL359532.1	1.088629945	0.003354923	1.558822396
AL359538.1	0.140136296	1.72E-08	2.329443766
AL359547.2	0.051418455	0.002154009	1.771708538
AL359636.2	0.25599962	0.001384464	1.70450407
AL359693.1	0.023666809	0.001782951	1.62205054
AL359694.1	0.132832892	0.00148871	1.627316453
AL359715.2	0.745007016	0.000360682	1.710790685
AL359740.1	0.505816893	0.000425576	1.708204338
AL359745.1	0.030648401	0.001009856	1.754160533
AL359757.2	0.041727727	0.004842003	1.904524143
AL359771.1	0.040515715	0.000232549	1.979922505
AL359878.2	0.557402297	1.12E-05	1.933114361
AL359881.1	3.46966476	0.000618175	1.693405491
AL359881.3	0.216772972	0.000805079	1.674133541
AL359915.1	0.878976848	0.001575352	0.585904442
AL359921.1	2.346283497	0.000737216	1.659965199
AL359955.2	0	0.004606615	1.557019068
AL359962.2	2.534586369	0.002523421	1.582465701
AL360012.1	0	0.004606615	1.557019068
AL360175.1	0.205321775	0.000393274	2.111567993
AL360181.2	9.326129516	0.001179837	1.628754676
AL365181.2	4.418002834	0.001554557	1.620351408
AL365203.2	8.842963452	0.000168313	1.762883144
AL365204.2	0.046084725	0.001753691	1.713771703
AL365205.2	0	0.004606615	1.557019068
AL365209.1	0.124571596	0.000602104	1.711264103
AL365214.2	0.300904761	0.000560471	1.684396685
AL365232.1	0.017172688	0.000198909	1.803686215
AL365318.1	0.099650144	0.000252761	1.75932143
AL365330.1	6.450600029	0.004723642	1.532439588
AL365475.1	0.437066113	0.000816992	1.655658923
AL389885.1	0.026611912	0.001483774	1.957535616
AL390037.1	0.254771896	4.83E-09	2.371048092
AL390115.1	0.630267274	1.97E-06	2.069450051
AL390205.1	0.128808424	0.002965324	0.588470982
AL390719.1	3.829102643	1.05E-05	1.949293941
AL390726.2	0	0.004606615	1.557019068
AL390726.4	0	0.004606615	1.557019068
AL390728.2	0.92970193	0.001936082	1.597828866
AL390728.6	25.74817926	8.58E-05	1.799607676
AL390729.1	0.763014662	0.004309474	1.547843172
AL390760.1	0.318677411	4.73E-05	1.861657698
AL390835.1	0.005608369	0.001182575	2.336714678
AL390838.1	0.036766151	0.002932499	1.604383708
AL390879.1	3.359917179	6.36E-08	2.217964863
AL391058.1	0.627985689	0.001903943	0.587282036
AL391069.1	0	0.004606615	1.557019068
AL391095.1	0.629059487	7.97E-06	1.954922038
AL391095.2	2.488081048	6.69E-05	2.140973052
AL391095.3	0.546371801	5.12E-05	1.84293571
AL391097.2	0.247565633	4.47E-06	2.030537636
AL391099.1	0	0.004606615	1.557019068
AL391244.2	4.687138001	1.65E-05	1.90258679
AL391262.1	0.123837212	0.000138574	1.781257607
AL391280.1	0.996758347	0.000748467	1.660188712
AL391684.1	2.640943064	0.004937166	1.530889489
AL391704.1	0.00850553	0.001267654	1.713962308
AL391825.1	0.843565172	1.27E-09	2.446604517
AL391832.1	0.173238964	0.003990124	1.555799136
AL391863.1	0.14110406	0.000887037	1.688337679
AL392086.1	0.094510925	0.001927108	1.643054824
AL441883.1	0	0.004606615	1.557019068
AL442125.1	0.981780161	3.52E-05	1.852088957
AL442128.2	0.937584536	2.61E-05	1.87289686
AL442663.4	3.027802786	0.000479571	1.694883111
AL445183.1	0	0.004606615	1.557019068
AL445183.2	0.148401859	0.000448835	1.712750992
AL445213.1	0.156115907	0.003996837	1.565208433
AL445222.1	3.041234756	0.00182592	1.605895077
AL445228.2	0.55144879	1.16E-07	2.197705797
AL445288.1	0	0.004606615	1.557019068
AL445471.1	0.566474928	0.000591113	1.683089193
AL445472.1	6.004032017	0.000438416	0.578472991
AL445483.1	1.644933934	0.000814982	1.676193572
AL445524.1	8.595003148	1.20E-05	1.93145265
AL445645.1	1.728631468	0.001746813	1.607284343
AL445649.1	0.19487564	2.00E-05	1.961879968
AL445686.1	0.111945278	0.00021504	1.783982869
AL445687.2	0.033340804	0.000623817	1.793379522
AL445933.1	0.205742593	0.000173714	1.767020706
AL449043.1	0.039425119	0.000739514	1.715686124
AL449212.1	5.60122839	0.000461684	1.688815237
AL449283.1	0.091723226	8.19E-05	1.834918254
AL449403.2	0.066779582	2.62E-05	1.948082831
AL450306.1	0.717532	0.002279133	1.612021142
AL450322.1	0.748888657	0.001304862	0.558526919
AL450326.2	0.519832147	2.73E-07	2.143551999
AL450332.1	0.380375807	0.002982305	0.618103867
AL450345.1	0.018028544	3.52E-06	2.064694485
AL450384.2	4.270345233	0.001202884	1.626111438
AL450472.1	0.18569775	1.90E-05	1.917093656
AL450487.2	0.033366827	1.39E-06	2.465762266
AL450992.1	0.070613307	9.68E-07	2.072177245
AL450998.3	1.329876062	0.000968192	1.640719031
AL451000.1	0.050650205	0.001981003	1.628478716
AL451047.1	0.109068114	0.000316815	1.774558304
AL451048.1	0.19169776	0.001044689	1.697796759
AL451050.2	1.058650294	7.75E-06	1.947249615
AL451054.1	0.120229917	0.000261683	1.745957471
AL451062.1	0.217156964	0.001692008	1.619093419
AL451069.3	0.114605494	0.003305306	1.579110398
AL451074.1	0.21436698	0.000504768	1.696339699
AL451139.1	0.235392209	0.003637001	1.572196564
AL451164.1	0.031849717	9.70E-07	2.297807581
AL451164.2	0.514398499	0.004471208	0.62531044
AL499627.1	0.056483396	3.22E-06	2.11560303
AL512288.2	1.244707915	1.43E-06	2.043437809
AL512306.2	1.403777394	9.83E-05	1.79514375
AL512310.9	0.002076504	4.54E-10	7.640877784
AL512353.2	0.033630516	0.002742529	1.577413633
AL512378.1	0.133747404	0.00023975	1.776269695
AL512422.1	0.631125421	0.000105294	1.827527946
AL512430.2	0.18324707	0.004817888	1.572021243
AL512506.1	0.720910151	0.004257983	1.554407182
AL512624.2	0.00274079	0.00012055	2.465917599
AL512652.1	1.059786894	0.000218654	1.742466362
AL512662.1	0	0.004606615	1.557019068
AL512770.1	1.582401053	4.49E-06	1.979817472
AL512785.1	0.151952851	4.90E-05	1.967284427
AL512844.1	0.097827026	0.001133737	0.584976033
AL513165.1	20.71082851	2.66E-05	0.500611406
AL513174.1	0.13243483	0.001177075	1.645529724
AL513217.1	0.304156929	0.000496305	1.69090243
AL513218.1	2.539835019	8.06E-08	2.215363472
AL513320.1	4.122920661	0.000234692	1.747479146

AL513325.1	0.25284557	0.001051535	1.648360612
AL513327.1	4.159051979	0.000153574	1.759252478
AL513329.1	0.963944757	5.81E-08	2.228328765
AL513366.1	0.202089032	0.004954004	1.550140889
AL513412.1	0.144759962	0.000155468	1.841495229
AL513477.2	3.414408912	3.29E-05	1.859329456
AL513478.1	0	0.004606615	1.557019068
AL513479.1	0.054193702	2.46E-05	1.992161758
AL513480.1	0.102094296	0.002395323	1.638301976
AL513497.1	0.688985054	3.24E-06	2.007151614
AL513534.1	9.004449137	0.002101881	1.589482652
AL513548.1	0.019234175	0.003107506	1.626302179
AL583722.2	0	0.004606615	1.557019068
AL583810.2	0.155720608	0.000333994	1.712308798
AL583810.3	0.097218474	0.003918974	1.558366896
AL589182.1	0.024021952	0.00034852	2.081073617
AL589647.1	0.146026788	0.002349359	0.601319025
AL589740.1	0.027913661	0.000176011	1.809594156
AL589743.1	0.271910074	5.42E-05	1.853202449
AL589743.6	0.630230683	0.000244924	1.746790246
AL589763.1	0	0.004606615	1.557019068
AL589765.2	0	0.004606615	1.557019068
AL589765.3	0.197755648	0.002441615	1.642660544
AL589880.1	13.81576134	0.000704577	0.581672334
AL589935.1	0.978278274	0.001448628	1.619317966
AL589935.2	0.63951454	0.00069346	1.681448839
AL589986.2	0.50485509	0.000393762	1.855135659
AL590065.1	0.014313077	0.001122913	1.667556162
AL590093.1	0.431028399	0.00367007	1.557675718
AL590133.2	0	0.004606615	1.557019068
AL590143.1	0.124260267	0.001187554	1.787222087
AL590226.1	3.980517325	0.00433319	0.625343341
AL590226.2	0.448474456	0.000433396	1.714497088
AL590302.2	0.082758352	0.000605564	1.721641374
AL590326.1	2.124914268	0.001475908	1.632819639
AL590327.1	19.54945093	0.001321425	0.588501005
AL590385.2	0.361388589	0.004640723	1.545422532
AL590396.1	0	0.004606615	1.557019068
AL590399.6	0.001520984	9.37E-05	7.024280846
AL590426.1	0.081028388	0.001044123	1.666770789
AL590428.1	1.59935695	0.000255447	0.54017035
AL590440.1	0	0.004606615	1.557019068
AL590491.3	0	0.004606615	1.557019068
AL590502.1	0.052108018	0.004718402	1.595642306
AL590556.1	1.076072777	4.46E-05	0.508392735
AL590560.2	7.528569005	6.24E-06	1.976136951
AL590560.3	0.537770518	0.000811847	1.669931641
AL590617.2	5.110957873	8.75E-05	1.802198173
AL590640.1	0.129483175	0.000230969	1.78713642
AL590644.1	6.437181369	2.68E-07	2.253267082
AL590683.1	0.047255156	0.004736286	1.577567664
AL590704.1	0.064248489	0.000211309	1.806499656
AL590762.1	1.48216999	0.002024556	1.590138782
AL590762.2	0.385405717	0.000435199	1.70207661
AL590999.1	2.871582341	0.000968485	1.702479909
AL591135.1	0.238207481	0.001887217	0.610299383
AL591212.1	0.072209546	0.00143296	1.632515881
AL591222.1	0.020157206	0.00013013	1.808049831
AL591419.1	0.266760053	4.25E-06	2.062145462
AL591499.1	0.056167987	0.00083306	1.698182211
AL591742.1	0.090758611	0.000149963	1.807024744
AL591806.1	0.059526755	2.48E-08	2.382000369
AL591845.1	10.80764566	1.67E-09	2.421936748
AL591848.2	0.281799949	0.002884776	1.606377286
AL591848.3	0.446823771	0.004310224	1.537255213
AL591885.1	0.132064695	0.004370622	1.548032875
AL592071.1	0.62018731	0.000234338	1.751444539
AL592078.1	0	0.004606615	1.557019068
AL592114.3	1.224735381	0.002325048	0.569557311
AL592146.1	0	0.004606615	1.557019068
AL592148.1	0.42651121	0.000532471	1.68638242
AL592211.1	1.029121138	0.00011073	1.784252213
AL592293.2	0.117758687	0.001381064	0.591564372
AL592463.1	0.071488879	5.62E-05	1.883533499
AL592486.1	0.104977517	0.003892806	1.584340891
AL592494.1	0.459894215	7.19E-05	1.830532684
AL592494.2	1.002619208	0.00072337	1.667464583
AL596223.1	0.121734589	5.02E-07	2.138832653
AL596223.2	1.129025183	0.000148635	1.790919608
AL596330.1	0.062568784	1.76E-05	2.005605601
AL603650.1	0.177227379	0.000218785	1.840445676
AL603832.1	1.88166881	0.000321794	1.718866766
AL603832.3	0.085364301	0.002404812	1.591010489
AL603839.4	0.49189933	8.39E-09	2.334087167
AL603914.1	0.412611261	0.000232589	1.773927539
AL603962.1	0	0.004606615	1.557019068
AL606462.1	0	0.004606615	1.557019068
AL606489.1	5.178155214	0.000451294	0.559452118
AL606490.6	0.016269891	0.003157601	1.989285873
AL606490.7	0	0.004606615	1.557019068
AL606490.8	0.052621165	4.99E-05	2.374014658
AL606519.1	0.065338022	0.001024855	1.76176233
AL606534.1	0.938836334	0.00013396	1.769482081
AL606662.1	0.081969477	3.12E-05	1.941281312
AL606748.1	0.07594793	0.002341986	1.602315729
AL606845.1	0.063957506	3.97E-05	2.030649707
AL627309.3	0	0.004606615	1.557019068
AL627309.6	3.921461224	0.000152868	1.788233776
AL627389.1	0.948601179	0.003883811	1.547715741
AL627443.1	0.042616927	0.000814743	1.682763762
AL645568.1	2.039733086	0.000573637	1.67326087
AL645568.2	0.109399268	2.22E-07	2.186303948
AL645608.5	0	0.004606615	1.557019068
AL645608.6	0.781451354	0.000339859	1.730203493
AL645608.8	0.920360958	0.002779934	1.593350841
AL645634.1	0.022368526	0.001326593	2.019184725
AL645730.1	0.047560976	0.001152879	1.741606941
AL645924.1	0.775578128	0.001475678	1.638654505
AL645939.1	2.302835433	0.000406645	1.703926784
AL645939.4	2.060259743	0.002878634	1.5900827
AL645940.1	1.360730287	3.80E-09	2.382785263
AL645941.3	0	0.004606615	1.557019068
AL662797.1	1.661983347	1.08E-07	2.197320817
AL662884.1	0.742631653	0.000759467	1.660979175
AL663070.1	1.375398851	0.000450712	1.733503889
AL663074.2	0.111225559	0.00277024	1.621143599
AL669818.1	0.182177609	0.004934283	1.578942838
AL669831.5	0.452216663	6.74E-07	2.084681838
AL669970.1	0.066456936	4.01E-06	2.17495394
AL669970.3	0.117163994	0.003580324	1.623372572
AL670379.1	0	0.004606615	1.557019068
AL670379.1	0	0.004606615	1.557019068
AL670379.1	0	0.004606615	1.557019068
AL670379.1	0	0.004606615	1.557019068
AL670379.2	0	0.004606615	1.557019068
AL670379.3	0	0.004606615	1.557019068
AL670379.4	0	0.004606615	1.557019068
AL670379.5	0	0.004606615	1.557019068
AL670379.6	0	0.004606615	1.557019068
AL670379.7	0	0.004606615	1.557019068
AL670379.8	0	0.004606615	1.557019068
AL670379.9	0	0.004606615	1.557019068
AL670729.2	0	0.004606615	1.557019068
AL670729.3	0.201069815	0.000173801	1.75901936
AL671710.1	1.068132843	2.55E-07	2.153444066
AL672207.1	1.341777011	8.81E-05	0.499581354
AL683807.1	2.440405284	0.000186895	1.763016563
AL683813.1	2.162702599	0.002296083	1.57947016
AL683887.1	0.058806268	0.000196963	2.354171445
AL691426.1	0.007913597	0.002061048	2.363944586
AL691432.1	2.939324655	0.00278563	1.572210409
AL691459.1	0	0.004606615	1.557019068
AL691482.1	0.161883747	6.59E-06	2.331104583
AL691482.3	2.272360224	0.002457092	1.624154782
AL691497.1	0.04601787	0.003871302	1.734965627
AL713923.1	0.022597608	0.001537204	2.003508747
AL731532.1	0.00752162	0.002278203	1.650994497
AL731556.1	0.415791346	0.000214259	1.86854451
AL731556.2	0.166589036	0.000158223	1.82950286
AL731567.1	3.490736736	1.63E-06	2.043215431
AL731568.1	0.700702188	0.003199571	1.629620282
AL731569.1	1.532008592	0.000206672	1.742178791
AL731577.1	0.185555275	0.001839254	1.640934665
AL731577.2	1.822694215	3.08E-06	0.449126028
AL731684.1	0.134223321	0.001597165	1.632389871
AL732292.1	0.077456792	0.000358105	1.832167568
AL732372.1	0	0.004606615	1.557019068
AL732437.1	0	0.004606615	1.557019068
AL732618.1	0.035614273	0.003636437	1.896954805
AL772161.1	0	0.004606615	1.557019068

AL772337.1	3.815319045	0.002054166	0.595667812
AL773545.2	0	0.004606615	1.557019068
AL807752.1	0.54223642	1.57E-06	2.046956106
AL807752.3	0	0.004606615	1.557019068
AL807757.2	0.850902907	7.05E-05	1.812276889
AL845552.1	0.379019102	3.82E-05	1.861124016
AL928654.1	3.779166349	0.002872954	0.612579066
AL928654.2	9.328466317	4.35E-05	1.840760699
AL928711.1	0.618409571	0.000789885	1.658830534
AL929288.2	0.016381007	0.002179216	2.188816495
AL929472.2	0	0.004606615	1.557019068
AL929472.3	0	0.004606615	1.557019068
AL929554.1	0	0.004606615	1.557019068
ALAD	48.39806714	3.71E-06	0.473761016
ALCAM	38.76791084	0.000733263	0.563494763
ALDH16A1	25.51313283	0.000152417	1.781021382
ALDH18A1	97.22669173	0.00328513	1.560916221
ALDH1L1	31.47401162	9.02E-06	0.478129988
ALDH1L1-A	3.43823851	0.003180719	0.617161571
ALDH1L2	3.717947697	0.000335502	1.757524167
ALDH3A2	106.7981356	1.48E-07	0.423692012
ALDH6A1	41.76261969	2.20E-08	0.358697872
ALDH7A1	59.55809225	6.38E-06	0.487561551
ALDH7A1P	0.41992085	0.001168469	0.581729576
ALDH9A1	158.1464788	0.001469311	0.610609677
ALG11	4.31938956	5.39E-05	0.519346739
ALG13-AS1	4.113368257	0.002162403	1.586185284
ALG1L13P	0.991827022	0.002583474	1.57895264
ALG1L2	0.508019619	0.002379805	1.584161867
ALG1L7P	0.347212257	0.001592125	1.623555211
ALG2	60.71270249	1.93E-06	0.461445161
ALG3	37.83960027	8.14E-08	2.246349783
ALG5	65.04665043	0.000105981	0.547772925
ALKBH1	16.58786703	0.000447328	0.576426973
ALKBH6	2.922135515	2.76E-05	1.866714531
ALKBH8	9.254045254	0.002567179	0.622977722
ALLC	0.364242612	0.0048058	1.929083253
ALMS1	8.470963778	0.002980606	0.620621208
ALMS1-IT1	1.02546907	0.000106393	1.795514183
ALOX12P2	0.952220404	0.004915871	1.556693053
ALOXE3	0.178757824	0.00291391	1.660660262
ALPP	0.027241832	0.000806926	1.754122393
ALS2CL	6.182157378	0.002419215	1.616185446
AMD1	77.24495456	8.32E-07	0.405750521
AMD1P4	0.227473754	0.000260766	1.823613522
AMDHD1	8.11344131	0.001227555	0.595894861
AMER1	4.213329278	0.000355787	0.549715061
AMH	1.691485686	5.54E-09	2.399874536
AMMECR1-	0.082988765	0.004129979	1.563407648
AMN	52.38098954	0.000338096	0.549128494
AMOT	25.87208459	2.11E-08	0.386506289
AMOTL1	30.39093232	0.000117655	0.523112698
AMPD2	33.23915178	0.00062428	1.691206239
AMY1C	0.000790152	0.002471238	3.269927726
AMZ1	1.52914276	0.003277062	1.649052584
AMZ2P1	9.71130298	0.001697591	1.60437383
ANAPC10P	0.130091263	0.000592459	1.69834183
ANAPC11	40.01001585	0.00082583	1.65828229
ANAPC4	13.9463985	0.001870341	1.599008019
ANAPC5	35.89098282	9.33E-05	1.799446088
ANAPC7	25.88532422	0.000167467	1.757278299
ANG	74.55659404	0.000734456	0.581074374
ANGPT1	9.671013755	0.002425941	0.589854541
ANGPTL3	14.33596247	0.001115172	0.444788196
ANGPTL5	0.142497084	0.000799385	1.713846947
ANGPTL6	0.779332199	7.32E-08	2.218835003
ANGPTL8	12.85894201	0.000156278	2.311418767
ANK3	12.91213961	3.07E-07	0.412305331
ANKFN1	0.166797896	6.10E-05	2.069366723
ANKH	26.57268316	0.00019661	0.548754488
ANKIB1	52.86672558	0.000960199	0.585540881
ANKK1	1.251954534	0.001145346	1.636926591
ANKLE1	0.740775744	1.09E-07	2.194584008
ANKRD10	77.58422432	0.000114754	1.777906725
ANKRD10-I	31.31298833	0.000342168	1.70909514
ANKRD12	18.72835516	0.003744401	0.62209447
ANKRD13B	6.165509871	2.48E-05	1.881391629
ANKRD13D	14.50116377	1.21E-09	2.448246422
ANKRD17	36.89693912	0.003329114	0.626508771
ANKRD18B	0.121794246	6.52E-08	2.538696707
ANKRD18E	8.159746188	6.97E-06	0.483509745
ANKRD20A	0.045833918	0.000141722	1.829267708
ANKRD20A	0.179980789	0.001854358	1.607864996
ANKRD20A	0.545661723	9.68E-05	1.804027895
ANKRD20A	0.049486583	0.00497232	1.651374859
ANKRD23	1.006184185	0.002405971	1.579249221
ANKRD26P	0.021455519	0.002417155	1.668880645
ANKRD27	17.63003757	7.38E-05	0.542112457
ANKRD30A	0.063981743	0.002023835	1.704378987
ANKRD30B	0.14066093	1.24E-05	2.298031253
ANKRD30B	0.030664486	7.88E-08	2.877367259
ANKRD31	0.540324017	0.00051005	0.558428592
ANKRD33	0.216193432	1.81E-06	2.130091879
ANKRD35	1.112228452	0.004280225	1.545987079
ANKRD36B	0	0.004606615	1.557019068
ANKRD36B	0.982543697	6.27E-07	2.210929668
ANKRD40	54.73090618	5.36E-05	0.526785352
ANKRD46	20.09277116	7.47E-05	0.530664168
ANKRD54	20.24650121	0.001678698	1.609150836
ANKRD6	10.4851303	0.001846133	0.608057755
ANKRD60	0.070471712	1.17E-06	2.504427787
ANKS4B	16.87025113	8.78E-08	0.41066684
ANKS6	14.10538206	4.15E-05	0.506823677
ANKUB1	0.040430789	6.13E-05	1.840360732
ANLN	8.97430338	1.65E-07	2.209549411
ANO1-AS1	0	0.004606615	1.557019068
ANO3	2.854325456	0.000209943	0.535852363
ANP32AP1	0.317432857	0.002284645	1.589408966
ANP32BP3	0.175842085	0.000276148	1.8156389
ANXA2	241.2606768	7.92E-05	1.811955046
ANXA7	204.796529	4.42E-06	0.487760673
ANXA8	0.243504205	2.02E-08	2.856176334
ANXA8L1	0.169168442	5.64E-07	2.336394902
AOAH-IT1	0.298857375	0.000360199	1.72468024
AP000221.1	0.097759843	0.003416482	1.614181161
AP000229.1	1.531073136	0.000608195	0.558629701
AP000238.1	1.36525224	1.31E-05	1.919888743
AP000240.1	3.743728309	3.39E-09	2.393100448
AP000254.1	12.02068323	2.18E-05	1.892474524
AP000350.5	0.9942846	0.001649055	1.611996047
AP000426.1	0	0.004606615	1.557019068
AP000432.2	0.070868732	0.000176487	1.831460123
AP000439.2	51.54063152	3.19E-06	0.409363585
AP000442.1	0.49873653	8.39E-07	2.085025723
AP000442.2	3.719000236	2.38E-06	2.021598387
AP000446.1	0.161896165	1.61E-05	1.903437338
AP000459.1	0.083596748	0.000722407	1.781067345
AP000462.3	0.219384412	0.000184913	1.779717435
AP000470.1	0.006766709	0.00053476	2.605977075
AP000522.1	0	0.004606615	1.557019068
AP000523.1	1.105321759	6.86E-05	1.824183153
AP000525.1	1.324360997	2.12E-07	2.171218945
AP000527.1	0.039593468	0.002288834	1.641751617
AP000533.3	0	0.004606615	1.557019068
AP000553.2	1.767980755	0.000103435	1.796026762
AP000561.1	0.112771508	1.33E-06	2.120488462
AP000577.1	0.608398652	6.76E-10	2.477960618
AP000580.1	3.101355191	0.001538392	1.615961685
AP000593.3	0.886501221	0.000340821	1.711917518
AP000619.1	0.057285929	0.000135171	1.837827885
AP000648.2	0.093621285	2.19E-05	2.058527341
AP000688.3	0.10573368	0.000385069	1.786874959
AP000692.1	2.94471486	0.000994175	1.638106109
AP000692.2	0.930242908	0.003824135	1.568716143
AP000695.2	4.249191109	1.87E-07	2.172670851
AP000695.3	0.583585025	0.001108801	1.656688499
AP000697.1	0.099945536	8.44E-07	2.243136093
AP000753.1	0.139249653	0.001175607	1.647042764
AP000755.1	0.096278605	0.004495315	0.611839099
AP000763.4	0	0.004606615	1.557019068
AP000769.1	11.96569899	0.000574863	1.681450375
AP000769.2	0	0.004606615	1.557019068
AP000769.3	0.064034956	0.001102907	1.681901161
AP000781.1	1.07641558	0.00075954	1.660868581
AP000797.3	1.034252961	0.000826661	1.720747405
AP000821.1	0.040823255	0.002165168	1.633537201
AP000844.2	2.358956461	0.000104823	1.884442171
AP000866.3	2.368254311	0.000503134	1.690213158
AP000866.6	1.937141581	0.000302249	1.724823941
AP000867.2	0.448592509	1.37E-06	2.049533541

AP000873.1	0	0.004606615	1.557019068
AP000873.4	2.02093594	0.002392616	1.578044564
AP000879.1	0	0.004606615	1.557019068
AP000892.2	1.218274068	0.001108069	1.636249225
AP000893.1	0	0.004606615	1.557019068
AP000893.2	0.110168155	0.000701836	1.711405684
AP000894.2	0.315558321	0.00314722	0.469183787
AP000897.2	1.865733358	0.001408308	0.563662738
AP000904.1	0.239637918	0.003837143	1.591285147
AP000919.3	0.495804416	0.004215169	1.578654614
AP000926.1	0.128439803	0.000775812	1.705243125
AP000942.2	0	0.004606615	1.557019068
AP000942.4	0.101303813	0.001082092	1.742822271
AP000962.1	0.176408348	0.004759133	1.583483471
AP000974.1	0.483857786	0.000701871	1.678792503
AP000997.1	0.179780567	0.004363809	1.588844718
AP001001.1	1.323463001	0.003694032	1.547586336
AP001002.1	0.024117105	0.004455177	1.604005155
AP001007.1	0.042098288	0.003533984	1.633625693
AP001020.1	0	0.004606615	1.557019068
AP001029.1	2.25851608	1.93E-05	1.891777057
AP001029.2	0.473229609	0.001068443	1.636154617
AP001029.3	0.754531855	0.000988281	1.642069652
AP001034.1	0.025171832	0.000290568	1.806040733
AP001052.1	6.572089243	0.000195582	1.758600331
AP001062.3	0	0.004606615	1.557019068
AP001063.1	0.13982845	0.000256258	1.753665471
AP001065.1	0.795980896	0.000867727	1.700680786
AP001099.1	0.393211006	0.001694872	1.623964985
AP001107.2	0	0.004606615	1.557019068
AP001107.3	0.413399453	0.001572467	1.624597355
AP001120.2	0.108824959	5.28E-06	1.992350761
AP001120.3	0.097095071	2.20E-05	1.960346577
AP001160.1	2.770291344	1.59E-06	2.040780042
AP001160.3	7.913700967	0.001772304	0.611720656
AP001160.4	2.080657993	0.001325042	1.627126889
AP001264.1	0.399806007	0.003691302	1.554627776
AP001266.1	0	0.004606615	1.557019068
AP001269.4	0	0.004606615	1.557019068
AP001273.1	2.615653991	0.004504623	1.535324624
AP001347.1	0.565949468	3.39E-05	1.858996875
AP001362.1	0.332507118	0.00044092	1.772912743
AP001372.2	13.54460099	2.31E-07	0.439395765
AP001372.3	0	0.004606615	1.557019068
AP001381.1	0.664573552	0.00470521	1.556719354
AP001412.1	0.792846391	3.98E-05	1.855035797
AP001453.2	5.576379375	0.000753291	1.70108878
AP001453.4	2.484245733	1.20E-08	2.313107382
AP001464.1	0.136671798	0.002006592	1.621289089
AP001469.2	1.305689734	0.000768713	1.657072685
AP001469.3	1.412833759	0.000666558	1.671000261
AP001471.1	0.351452416	0.001419477	1.648898038
AP001476.3	0.018888819	0.00029299	2.307198263
AP001486.2	3.926463602	0.004508653	0.64085331
AP001496.3	0	0.004606615	1.557019068
AP001505.1	13.88458212	3.71E-05	1.8611306
AP001506.1	0.000595332	5.10E-06	22.83636517
AP001527.1	0.066404642	0.000663448	1.701013413
AP001527.2	1.075795567	0.000443769	1.706263258
AP001528.2	13.60160407	0.000232209	0.549272776
AP001528.3	16.10251424	0.000111802	0.53023818
AP001542.1	2.065855221	0.000678059	1.702429801
AP001604.1	0.057913627	0.000286866	1.767559991
AP001610.1	0.471489218	0.001652102	1.612875174
AP001615.1	0	0.004606615	1.557019068
AP001619.2	0.145625862	0.000116024	1.788185429
AP001628.1	1.35055429	8.19E-08	2.210434107
AP001767.3	2.39406517	0.000190322	1.756051268
AP001783.1	0.338248344	7.02E-05	2.031971569
AP001830.2	0.033927736	0.000854322	1.78346281
AP001893.3	0.54921239	0.002793612	1.57526161
AP001922.6	3.009083463	0.004307286	0.634831955
AP001970.1	0.02515398	0.000110583	2.093447424
AP001972.2	0	0.004606615	1.557019068
AP001972.4	0.501160202	0.000873917	1.767650998
AP001992.1	3.366748208	0.000674684	1.672735334
AP002336.1	0.258439816	0.000478481	1.717478785
AP002360.3	5.826403261	3.96E-06	1.992006697
AP002364.1	0.364637225	7.75E-06	1.949723773
AP002383.1	0.029183511	0.000512564	1.881668498
AP002409.1	0.036363403	0.004557344	1.560757862
AP002478.1	0.056464886	0.002384617	1.664999193
AP002490.1	1.255651727	9.96E-05	1.788590814
AP002761.1	0.310008099	4.18E-07	2.136011263
AP002784.1	0.295080084	0.00140379	1.634995904
AP002791.1	0.177685865	0.001454818	1.684969684
AP002807.1	3.621632472	3.08E-07	2.135720857
AP002812.1	1.533036218	0.002915151	1.565111019
AP002812.5	1.333153195	0.001478609	1.613882033
AP002833.1	0.031607164	0.000507605	2.040220248
AP002833.3	0.05318073	0.000722985	1.745102616
AP002852.1	0.29629971	3.20E-13	3.141449567
AP002856.4	0.009788367	0.000528997	2.40679752
AP002884.4	0.124388426	0.001884488	1.601942393
AP002893.1	0	0.004606615	1.557019068
AP002907.1	2.212746554	7.18E-06	1.956352462
AP002981.1	0.092450059	3.35E-08	2.264836916
AP003025.1	0.034868065	3.63E-05	1.937164037
AP003032.2	0	0.004606615	1.557019068
AP003037.1	0	0.004606615	1.557019068
AP003062.1	0.352882393	2.34E-05	2.683755604
AP003064.1	0	0.004606615	1.557019068
AP003068.2	7.218474366	0.000129489	0.521988622
AP003068.3	0	0.004606615	1.557019068
AP003072.2	0.814414133	0.000631761	1.667143065
AP003080.1	0.254463718	0.000240267	0.542855058
AP003119.1	0.435127522	5.03E-05	1.988817441
AP003119.2	2.07616393	4.73E-05	1.855511791
AP003119.3	2.482651885	0.000721775	1.735930162
AP003122.2	0.07156436	3.53E-07	2.258770997
AP003170.2	0	0.004606615	1.557019068
AP003170.3	1.525097279	0.004090737	1.549671955
AP003170.5	0.201773162	0.0030093	1.57103797
AP003304.1	0	0.004606615	1.557019068
AP003327.1	0.262115572	0.00065074	1.780303245
AP003352.1	5.599508253	5.35E-11	2.623897061
AP003354.1	0.322801596	0.000936633	1.644933382
AP003390.1	1.218744734	0.00018744	1.768706354
AP003392.3	0.606124146	0.001661774	1.621380951
AP003392.4	3.672260264	0.000296276	0.557811887
AP003392.6	1.647898977	0.000631907	1.673679467
AP003396.1	0	0.004606615	1.557019068
AP003396.5	0	0.004606615	1.557019068
AP003400.6	0.030507282	0.002877871	1.776560292
AP003419.3	2.303379441	1.10E-05	1.925995883
AP003469.3	0.260598476	0.002982052	1.571542886
AP003469.4	9.298057279	0.000477807	1.692959987
AP003472.2	0.169180887	0.001352125	1.646026623
AP003478.1	0.041565278	2.59E-05	1.944610915
AP003499.2	0.02437941	0.002191532	1.876300474
AP003501.1	0	0.004606615	1.557019068
AP003555.2	1.563544848	0.004529853	1.538491248
AP003680.1	0.067652891	0.002272617	1.582161108
AP003692.1	0	0.004606615	1.557019068
AP003716.1	3.502721021	1.79E-05	0.475140253
AP003717.2	0.114615436	0.001031417	1.640851255
AP003721.1	0.192890535	0.000568812	1.706615421
AP003721.4	1.532671244	5.94E-05	0.522724502
AP003733.1	0.185087704	0.003218883	1.566840359
AP003733.4	1.469770952	0.000675117	1.674197267
AP003774.4	1.221648144	0.000235212	1.744535959
AP004147.1	0.034381259	0.000689888	1.723063266
AP004290.1	0.092390685	0.000399946	1.731776123
AP004607.1	0	0.004606615	1.557019068
AP004607.4	0.001253516	0.000943385	3.320437263
AP004607.9	0.000400075	0.003067808	10.70957424
AP004609.1	0.201490427	3.30E-05	1.86984539
AP004782.1	0.423088968	0.001220537	0.576662765
AP005120.1	0.074106477	0.002304772	1.631651923
AP005137.2	0.084664804	0.003909255	1.570972821
AP005139.1	0.015315104	0.001756819	1.735742429
AP005203.1	0.217359385	0.000296997	1.771437089
AP005212.2	1.085121338	0.002005643	1.600968538
AP005233.2	16.72807137	0.003737207	1.601757759
AP005242.1	0.064929694	0.00207093	1.714599417
AP005264.5	0.090883093	0.000149128	1.829209995
AP005264.7	0.056026594	0.000142462	1.810915653
AP005273.1	0.012143443	0.002122376	2.147557806
AP005328.2	0.173145181	6.25E-05	1.896048564
AP005329.1	1.519388442	5.50E-05	1.826983454

AP005329.3	0	0.004606615	1.557019068
AP005433.1	0.017105736	3.24E-05	2.152466383
AP005597.4	0	0.004606615	1.557019068
AP005901.2	0.014027606	0.001373408	2.133041536
AP006245.1	0	0.004606615	1.557019068
AP006248.2	0.151447154	0.000188693	1.791343651
AP006248.3	0.16720663	0.000635517	1.733711187
AP006248.5	0	0.004606615	1.557019068
AP006284.1	17.73930952	0.002030574	1.601524948
AP006289.1	0.880339092	0.001880358	1.608180011
AP006545.1	0.607230637	0.000180248	1.754848891
AP006545.2	0.845867509	5.12E-10	2.488734563
AP006621.2	7.81242562	8.11E-05	1.808108651
AP006621.3	12.33561882	0.000331804	1.719949787
AP1AR	37.97398889	1.01E-07	0.429994722
AP1G1	48.23198396	1.37E-06	0.455712104
AP1G2	18.16403523	0.001053184	1.637989643
AP2B1	145.0530631	8.27E-05	0.533745145
AP2S1	140.8022599	0.002532562	1.580342657
AP3B1	39.60495332	0.001687472	0.614957868
AP3D1	69.39110576	0.002973928	0.628193193
AP3S2	15.12888273	4.40E-05	0.51556509
AP4S1	5.996953634	0.001328674	0.601570675
AP5B1	17.73102594	0.003843166	0.633255874
AP5M1	13.07764908	0.000228302	0.530892935
AP5Z1	20.56996685	1.13E-05	1.929322182
APBA1	3.092263212	0.00014771	0.532286178
APBA2	2.28246103	1.55E-05	1.978919859
APBB1	38.33076782	2.66E-07	0.445687705
APBB3	19.11857198	0.000331877	1.70992529
APC	15.55365001	5.60E-07	0.422495796
APCDD1L	5.584990125	0.000627004	1.962742212
APCDD1L-I	1.324900631	1.51E-07	2.683211715
APEX1	278.6374059	0.000374376	0.574386883
APH1A	215.3369609	0.001399165	0.611925513
APH1B	18.48142538	0.001423656	0.594032558
API5	78.51219833	0.000431352	0.576530981
APLNR	88.72939306	0.000824819	0.56411829
APLP1	5.16098618	0.001835314	1.725312979
APOA4	2.443977737	5.94E-05	3.418193419
APOBEC3B	6.232301258	0.000864074	1.654989162
APOBEC3D	12.62316282	0.000123396	1.783191565
APOC2	1.886545085	8.26E-05	1.84944767
APOC4-APC	0.472320981	0.001479843	1.632574664
APOL1	448.5628088	0.001202008	1.697126792
APOL2	127.9784945	8.43E-05	1.835468361
APOL3	42.47990463	0.002636313	0.620596042
APOLD1	166.8262764	3.15E-05	0.473498859
APOOL	22.14407033	2.99E-06	0.454050808
APP	1074.573973	1.41E-06	0.461636564
APPBP2	22.24946603	0.001110688	0.598787151
APPL1	32.77346363	5.45E-07	0.445104688
APRT	181.4133762	0.004584929	1.536769096
AQP1	928.2468165	2.10E-06	0.457952482
AQP9	17.51874153	0.004561778	1.584076195
AQR	17.42208131	1.40E-05	0.503040322
AR	16.92533106	1.67E-06	0.457106268
ARAF	81.83466264	0.000319418	0.565368391
ARC	4.026200453	0.002624703	0.555332543
ARCN1	190.2498058	0.000421099	0.581612459
ARF3	197.1431828	0.002003283	0.620058064
ARF4-AS1	0.931502381	0.000416458	1.703559118
ARFGAP1	44.32342687	9.55E-11	2.589044795
ARFGAP3	98.13204737	0.000596815	0.583377834
ARFGEF1	26.61116121	0.0003002	0.553209315
ARFGEF2	35.43202391	0.000322724	0.553286488
ARFIP1	38.22782914	4.90E-05	0.529334787
ARHGAP10	8.966667846	3.54E-08	0.412272663
ARHGAP12	30.13410582	0.00152534	0.594116092
ARHGAP22	5.184782231	0.0003311	1.716163086
ARHGAP24	35.74270789	1.46E-05	0.49918457
ARHGAP26	19.54330518	8.80E-05	0.530504162
ARHGAP27	5.843475436	0.004662582	1.532973728
ARHGAP28	5.858173711	4.68E-05	0.516725316
ARHGAP31	32.031076	1.90E-05	0.500279438
ARHGAP32	14.43150475	0.000376037	0.570038264
ARHGAP33	10.30176138	4.45E-05	1.840164248
ARHGAP35	46.99930699	8.20E-07	0.435464024
ARHGAP36	0.121398966	1.61E-05	2.138948828
ARHGAP4	32.88827479	4.98E-05	1.837689717
ARHGAP42	30.13606814	3.32E-06	0.472451773
ARHGAP5	38.06588719	7.11E-06	0.478497758
ARHGAP5-/	8.040052968	0.001883796	0.609977803
ARHGAP6	11.40224948	8.21E-05	0.485620634
ARHGAP9	12.61414868	0.004148604	1.547300609
ARHGDIG	0.12874551	1.17E-05	2.08009695
ARHGEF1	45.60828039	0.003202485	1.559241063
ARHGEF10	23.284862	0.000211167	0.54879754
ARHGEF12	59.44879799	2.56E-07	0.43530461
ARHGEF16	30.79607323	0.000388687	0.566981742
ARHGEF19-	0	0.004606615	1.557019068
ARHGEF28	23.6087515	3.35E-06	0.474049511
ARHGEF3	23.04453692	0.000108263	0.534931372
ARHGEF37	38.36048363	6.78E-05	0.507350052
ARHGEF39	4.277480833	0.001338707	1.619177626
ARHGEF7-/	0.162546851	3.80E-07	0.417527848
ARHGEF9	17.68622881	2.23E-06	0.467964008
ARHGEF9-I	0.171726618	0.000153643	1.784624694
ARID1A	39.69097879	0.002830577	0.628246402
ARID1B	17.17339109	0.00012124	0.5402414
ARID3C	0.644529266	0.002688844	1.598146576
ARID4A	18.18943236	0.003106766	0.619438387
ARID5B	57.05074105	5.19E-05	0.504855094
ARL10	9.498014185	0.000822241	0.571844813
ARL14EP	20.50125026	7.82E-05	0.528160984
ARL14EPL	1.213404981	0.000522362	4.239815972
ARL2	72.06907093	0.001841737	1.602851367
ARL2BPP1	0.055513326	4.65E-05	2.062104631
ARL2BPP1(	0	0.004606615	1.557019068
ARL2BPP6	0.160405794	1.23E-06	2.146200426
ARL3	35.65891248	8.92E-05	0.544038888
ARL4C	103.075767	0.000772995	1.671138199
ARL5A	43.47353582	0.002153036	0.617104405
ARL5C	0.049346032	0.000136914	1.782380329
ARL6	14.67402999	4.17E-06	0.462218634
ARL6IP4	3.666265906	2.45E-09	2.400452884
ARL9	0.87508727	2.86E-06	2.096175803
ARMC12	1.539686507	1.09E-10	2.574423922
ARMCX3	78.67628998	1.46E-07	0.408143419
ARMH1	1.445354044	7.88E-11	2.585744708
ARNT	42.67478635	0.004124925	0.639010551
ARNTL2	4.73762825	0.000344071	1.725627037
ARPC1B	142.5955922	3.96E-05	1.856366782
ARPC3	254.1321521	1.95E-08	2.369878386
ARPIN	23.32263021	0.001202272	0.596400401
ARPP19	114.77038	9.15E-07	0.452042082
ARR3	0.921803756	0.004498305	1.537202614
ARRB1	20.78949475	0.004850728	0.638649521
ARRDC1-AS	13.24174694	0.000644314	1.673174366
ARRDC5	1.345317416	0.00091752	1.655111656
ARSB	38.70873678	0.001020034	0.590974049
ARSD	57.90467352	0.002598676	0.611131826
ARSD-AS1	0	0.004606615	1.557019068
ARSI	2.055686574	2.68E-07	2.435017551
ARSK	13.27664108	0.001408626	0.603086595
ARTN	1.924283885	4.99E-07	2.246460383
AS3MT	1.259106723	0.000112385	0.519977367
ASAH1	257.6863837	0.000643306	0.557879381
ASAH2B	3.718668585	0.004023057	0.628587933
ASAP1	37.62968367	0.00188751	0.600289403
ASAP1-IT2	0.702638945	3.98E-06	1.987549614
ASAP2	17.40701117	3.66E-06	0.451772773
ASB13	103.4908592	0.004117385	0.630721813
ASB16	2.35939201	0.000441992	1.695736618
ASB16-AS1	11.71317	3.87E-05	1.848423551
ASB17	0.492229011	0.002484561	0.593151363
ASB7	16.26977647	0.000101689	0.546827973
ASB8	41.52549133	6.15E-05	0.543613751
ASB9P1	1.58739251	0.002711334	1.590238446
ASCC1	23.45521101	0.001169673	0.604964468
ASGR1	2.158154452	2.46E-07	2.188486703
ASH1L	26.9021756	2.45E-05	0.50468341
ASH2L	33.10271483	0.00105029	0.601976652
ASIC1	1.460341239	0.000206177	1.928433027
ASIC3	1.980252407	1.19E-09	2.44310437
ASIC5	0.049358598	1.60E-06	2.263822058
ASIP	2.08353252	0.001060504	1.684603272
ASMTL	41.0330374	0.000481689	0.580227222
ASMTL-AS	14.45224351	0.000344247	1.721268179
ASNS	23.2739087	8.34E-08	2.215942283

ASNSD1	65.07669969	2.25E-05	0.516839742
ASNSP1	0.044595401	7.96E-06	2.02659412
ASNSP3	0.094955169	0.002975857	1.578979979
ASPHD2	6.227033741	0.00127044	1.662313113
ASPM	2.751450326	1.64E-08	2.310904452
ASPSCR1	7.048278715	1.75E-07	2.173882211
ASRGL1	32.24925038	0.000828068	0.566533104
ASTL	0.213303118	0.001655104	1.623322204
ASTN2	8.428654267	7.10E-07	0.387824049
ASXL2	19.29011378	6.65E-05	0.517770869
ATAD1	43.49248699	5.98E-07	0.444270256
ATAD3A	18.76569277	4.77E-05	1.843818441
ATAD3B	6.292400759	1.55E-06	2.043067895
ATAD5	2.540840657	0.001339962	1.619095118
ATAT1	9.28925414	0.001600602	1.605477987
ATE1	29.09252665	8.59E-07	0.45355294
ATF1	31.99833578	0.001811002	0.617478758
ATF2	48.54620122	0.000316107	0.562208995
ATF5	67.99037819	7.69E-07	2.098820939
ATG16L2	14.93429468	0.000389895	1.700001361
ATG3	29.91256429	0.003463513	0.639966958
ATG4B	28.25664112	0.001037683	1.638969133
ATG4C	21.4302396	0.000395153	0.578461731
ATL1	8.314292529	0.003693897	0.632909442
ATL2	38.94629195	0.002878257	0.624673039
ATL3	73.02548712	0.001645553	0.616319499
ATMIN	46.46320472	0.003958585	0.640970606
ATP10D	25.86977196	0.000313936	0.544639985
ATP11A	101.268496	5.81E-07	0.42307433
ATP13A1	32.70307175	1.82E-06	2.061586433
ATP13A4	2.088053232	0.002763019	0.548860256
ATP1A1	425.5598232	0.004339702	0.59547259
ATP1A1-AS	5.157111044	0.000150546	0.538346273
ATP1A3	2.213642429	0.000629354	1.826802175
ATP2A1	1.856648641	0.00012586	1.782168813
ATP2A1-AS	0.974342968	2.14E-07	2.19364336
ATP2B3	0.224299219	0.000290454	2.147746258
ATP2C1	60.55932236	8.36E-05	0.536183586
ATP2C2	0.564159531	0.000558615	1.841291519
ATP2C2-AS	0.485869714	8.37E-05	1.807151931
ATP5F1A	169.229217	0.001307311	0.564714577
ATP5F1AP2	0.434583233	0.002717669	1.624436516
ATP5F1B	1114.060662	0.000590153	0.55418486
ATP5F1E	132.0974244	2.84E-06	2.01617836
ATP5MC1	78.81981396	0.001451133	1.621968097
ATP5MC1P	1.346237748	7.03E-07	2.110824246
ATP5MC1P	0.192404953	0.000115758	1.865327539
ATP5MF	136.3152977	0.000456241	1.69902678
ATP5MFP1	0.102808883	0.003362846	1.736546772
ATP5MFP4	0.947658695	5.20E-06	1.974845351
ATP5MFP5	0.514596932	0.000678632	1.695170262
ATP5PB	159.9825735	7.26E-06	0.494446116
ATP5PBP5	4.29792124	0.000901701	0.580031298
ATP6AP1	168.1345265	0.000328437	0.550246137
ATP6AP1L	3.50190799	0.002919247	1.570115177
ATP6V0A1	40.90619104	0.003958074	0.639342301
ATP6V0E1	0.471334882	4.34E-05	1.849906458
ATP6V1A	134.2898406	0.000444085	0.497224677
ATP6V1B1-	0.184415143	0.000145155	1.872736454
ATP6V1B2	85.78985785	0.000109364	0.542564043
ATP6V1C1	71.91522549	0.000300332	0.556718935
ATP6V1D	66.85109709	0.000170083	0.508569723
ATP6V1FN	7.374642258	3.08E-05	1.934143769
ATP6V1G1	0.208283141	0.000799565	1.680995447
ATP6V1H	40.38159905	0.001503574	0.553254975
ATP7A	13.8434555	0.000284962	0.557111785
ATP8A1	12.54859558	4.71E-05	0.487118102
ATP8B3	7.329100921	0.000134414	1.875953543
ATP8B5P	0.185238105	0.000207104	1.757180677
ATPAF1	43.98029257	0.002459871	0.618045902
ATRN	40.77983459	7.61E-05	0.527594461
ATRX	19.65382142	5.50E-05	0.51402952
ATXN1	14.39934726	0.000758825	0.583122133
ATXN1L	36.66166627	4.11E-09	0.377568435
ATXN2-AS	1.407308454	0.000273121	1.734909118
ATXN2L	69.78540994	0.000470068	1.70672204
ATXN7L2	4.84321203	8.38E-13	2.828508595
ATXN7L3B	72.43979128	7.80E-07	0.460916928
AUH	46.06027294	5.98E-06	0.465103574
AUNIP	0.989371946	0.001016294	1.657052448
AUP1	169.0484127	6.96E-09	2.385805696
AURKA	9.246129901	4.11E-05	1.878910483
AURKB	6.19564761	4.31E-13	2.876422719
AURKC	2.435410398	5.37E-05	1.826559267
AUTS2	24.54299332	0.000568836	0.577265571
AVEN	20.31843883	0.00014882	1.773852867
AVIL	3.695853966	0.002371113	1.580334414
AVPR1B	7.986416329	0.002017602	0.581953166
AWAT2	0.047302444	0.001014986	1.670205549
AXIN1	18.22711107	0.004784969	1.53317501
AZI2	10.34473032	6.75E-05	0.535065814
B3GALNT1	37.63594648	6.52E-06	0.468709266
B3GALNT1	0.146206725	0.00021964	1.799510269
B3GALNT2	4.831924156	0.004099217	0.632001389
B3GALNT2	0.041552559	0.002819426	1.686431321
B3GALT5	1.938618772	0.000368301	1.797327597
B3GAT2	0.532518817	0.002681591	1.569621056
B3GAT3	65.24234102	1.09E-08	2.344825937
B3GNT10	4.287046015	8.04E-08	0.404159184
B3GNT2	79.63170815	2.59E-05	0.505808608
B3GNT8	5.756675602	0.00013199	1.829807709
B3GNTL1	3.092298226	1.51E-09	2.460953923
B4GALNT2	0.046253604	0.003235253	1.866887551
B4GALNT4	3.638446216	3.43E-05	1.949771062
B4GALT2	39.08991949	0.001784986	1.605588421
B4GALT7	30.85197228	0.000207537	1.751243082
B4GAT1	121.9919654	0.001376372	0.611961549
BABAM1	55.61663018	0.000168635	1.762297138
BACE1-AS	17.89967062	0.000730351	1.660205963
BACE2	44.37711539	0.00013895	1.798559922
BACE2-IT1	0	0.004606615	1.557019068
BACH1-IT1	1.494540132	0.000677726	1.666820665
BACH1-IT2	0.626368277	3.56E-05	1.862656797
BAG1	34.84791497	8.45E-10	0.328439457
BAG5	43.0350757	0.000659549	0.579830503
BAHD1	28.78668104	2.19E-06	0.468232154
BAIAP2L1	14.26418451	0.000338112	1.734099784
BAK1	40.92507526	0.001837363	1.602800718
BANF1	183.2727697	3.14E-06	2.015085519
BANF1P1	0.304811933	0.001723285	1.622486264
BARX1	0.853085428	3.36E-07	2.737526559
BARX1-DT	0.036399176	4.94E-06	2.124600331
BASP1	41.59273684	2.73E-08	2.30390578
BASP1-AS1	0.065443356	0.000213078	1.768009252
BBOX1	288.5294414	3.13E-06	0.472566527
BBS10	26.65174237	0.001521854	0.608327739
BBS12	18.48880361	1.31E-05	0.490449963
BBS2	22.54217791	0.003167579	0.628363027
BBS7	14.20440345	0.001354576	0.605064939
BBS9	12.9626327	6.00E-05	0.532776674
BBX	28.78897152	0.001143395	0.587923522
BCAR3	18.56812762	2.79E-05	0.506976294
BCAS2	94.26641953	2.52E-06	0.479426352
BCAS3	10.59551293	3.30E-06	0.452801711
BCDIN3D	5.385159987	0.001732915	0.611799079
BCKDHA	9.650296023	6.27E-07	0.450779682
BCKDHB	18.64398697	0.003142974	0.604416042
BCL10	34.9111585	0.000116247	0.537839584
BCL2	60.52048284	1.57E-06	0.461914576
BCL2L12	11.19436886	1.54E-09	2.46008847
BCL2L12P1	0.140746131	0.000377199	1.726121205
BCL2L13	46.55767245	0.000616287	0.58257594
BCL2L2	50.39235356	0.000706995	0.578870928
BCL2L2-PA	0.712871724	6.55E-08	2.226531877
BCL3	58.22357248	1.63E-08	2.349927057
BCL7C	36.33166596	0.002816479	1.572551804
BCL9	14.04452438	0.00099412	0.585716663
BCLAF1P2	0.110042963	0.001137438	0.581074706
BCO2	1.483824536	0.002270187	1.641055112
BCOR	20.2796923	0.002553869	0.622222271
BCORL1	11.83170606	0.000682864	0.586721869
BCRP2	0.019423865	0.000475384	1.707301324
BCRP3	4.427916199	2.70E-05	1.876335838
BCRP9	0.056432799	0.003616265	1.97142828
BDH2	87.40737135	0.000251655	0.563207685
BDKRB1	1.770061101	0.004673833	1.625623754
BEAN1-AS1	0.396726458	0.000215806	1.748677307
BECN2	0.125661305	5.83E-08	2.299316312
BEND6	1.482634426	0.000949085	1.665228287
BEST4	5.751223603	0.004605112	1.578828265

BET1	23.56736499	0.002212388	1.591068631
BEX2	58.75481956	8.16E-05	0.522090295
BEX4	142.8156927	2.70E-05	0.508017069
BEX5	26.14097392	0.000594877	0.562424215
BFSP2	0.284500185	0.002713793	1.611637576
BGLAP	2.727424508	0.000468635	1.690338979
BHLHB9	4.981265957	4.54E-06	0.462652198
BHMG1	0.097141498	0.000925101	1.749622863
BHMT	310.1389727	9.52E-05	0.529082224
BICRA	6.947928502	0.000630155	1.677255869
BICRAL	20.76818318	8.42E-05	0.531145514
BID	24.88560368	6.31E-05	1.840058658
BIRC5	8.999956529	4.79E-09	2.383661662
BIRC6	23.42195046	7.48E-05	0.523882625
BIRC6-AS2	0.59634622	3.73E-05	1.857359751
BLACAT1	0.540807544	9.61E-06	1.982048058
BLCAP	35.55252017	0.002702665	1.57647121
BLM	1.731178194	0.001304378	1.628585558
BLMH	40.73274969	0.000374203	0.577083085
BLOC1S5	42.38599092	0.000177038	0.561367005
BLZF1	31.77649609	0.000337796	0.566990092
BMF-AS1	0	0.004606615	1.557019068
BMP1	33.4696085	8.16E-08	2.239401565
BMP2K	8.084741025	0.001348973	0.596825409
BMP5	1.494059669	0.000757674	0.495403201
BMP7-AS1	0.011937088	0.004023634	2.133849254
BMPER	1.7026884	0.001738469	1.842749818
BMPR2	56.54829875	8.45E-06	0.474891008
BMS1P22	0.100883848	4.98E-05	1.851161326
BMS1P4	0.770573716	5.75E-05	1.820882185
BNC2-AS1	9.18371081	0.001687168	1.611840427
BNIP3	232.8417392	0.001254968	0.600061554
BNIP3P10	0.637950556	0.001678896	1.612169804
BNIP3P11	2.518486632	2.61E-05	1.880161753
BNIP3P13	0.017471722	0.002316261	1.857259586
BNIP3P24	0.229059967	0.000983878	1.651838535
BNIP3P27	1.11948411	1.70E-05	1.903342858
BNIP3P35	0.020326506	0.001875172	2.091278627
BNIP3P8	0.143493514	3.33E-06	2.047463732
BNIPL	1.081095403	1.95E-07	2.16302057
BOC	7.946792345	0.000508968	0.567502988
BOD1	96.54128607	0.000793655	0.583116729
BOD1L1	16.73562871	0.00017959	0.54516306
BOLA2	0	0.004606615	1.557019068
BOLA3	17.92728661	0.001414583	1.621424614
BOLA3P1	0.077907794	3.74E-08	2.386084164
BOLA3P2	0.23828196	9.60E-09	2.387023346
BOP1	35.0321743	1.26E-07	2.196293363
BORA	3.22844843	0.002121866	1.59062677
BORCS7	55.90478799	0.000321695	0.558479306
BPGM	55.35232196	0.000248032	0.545160489
BPHL	15.77397135	0.000565983	0.571681356
BPIFA4P	0.021067219	0.00140699	1.701629224
BPIFB4	0.076239094	0.000851195	1.815634453
BPIFC	0.058682019	0.001137304	1.665162128
BPNT1	36.33643565	1.01E-05	0.497740037
BPTF	22.00424065	0.000962102	0.591658132
BPY2B	0	0.004606615	1.557019068
BRAF	16.65720712	0.001149964	0.597531929
BRD3	15.85260489	0.003597196	0.632219345
BRD3OS	20.04678377	1.93E-06	0.471182901
BRD9	14.36188223	0.000148509	1.766667826
BRDT	0.043931304	0.000340916	1.737142497
BRF2	10.93869383	0.003716719	0.638187299
BRICD5	4.796893158	0.000138334	1.769937201
BRMS1L	10.17714492	5.36E-06	0.473971605
BRPF3	34.44878471	0.000136013	0.548102397
BRS3	0.752739144	0.001030076	2.079685356
BRSK1	2.761778772	0.00014114	1.77651652
BRSK2	0.622043417	0.000126226	1.853814535
BRWD1P1	0.081772469	6.22E-05	1.949400496
BRWD1P3	0.041153035	0.002041054	1.786517817
BSDC1	59.93152614	0.000556082	0.589030062
BSPRY	11.14875442	5.02E-06	0.411559335
BST1	14.25892948	0.002817944	0.62675604
BTBD10P2	0.656394589	3.10E-07	2.130894375
BTBD11	3.287490607	1.11E-06	2.133400872
BTBD19	16.10349508	0.000335178	1.711433329
BTBD7	20.10072004	4.63E-05	0.519631177
BTBD8	1.754940836	0.000100968	0.505260233
BTBD9	10.74915834	1.53E-06	0.455338703
BTBD9-AS1	0.249072898	2.65E-05	1.923573812
BTD	25.03995499	2.14E-05	0.497254337
BTF3P10	0.360253122	0.000304442	0.543092615
BTG3	34.82095582	0.000377781	1.709612459
BTG4	0.051200896	8.53E-06	1.983627208
BTN1A1	0.183074045	0.003295829	1.564362043
BTNL10	0.564289167	0.002346232	1.585909297
BTRC	15.97812942	0.000453411	0.574413632
BUB1	4.477633698	3.75E-07	2.146935369
BUB1B	3.954200171	1.40E-09	2.442059563
BUD23	31.73835751	5.87E-06	1.976999654
BUD31	74.13294846	0.000123487	1.792800789
BX005040.2	0	0.004606615	1.557019068
BX005195.1	0	0.004606615	1.557019068
BX119917.1	1.39170109	6.55E-05	0.501956124
BX276092.1	0	0.004606615	1.557019068
BX284668.2	0.437748736	0.00014824	0.474933042
BX322234.2	0.497327517	9.57E-06	2.039021473
BX322650.1	0.810305545	3.23E-05	1.882594065
BX510359.3	0.019820819	4.21E-06	2.900600289
BX510359.6	0	0.004606615	1.557019068
BX537318.2	0.180086941	5.86E-05	1.840017165
BX546450.1	0.046755821	0.000354655	1.798869407
BX546450.2	0.22051088	6.57E-08	2.227655976
BX927359.1	0	0.004606615	1.557019068
BYSL	28.18419131	0.001581942	1.613140099
BZW1P2	1.626460538	0.002530806	0.604315466
C10orf105	0.151194344	0.002420166	0.605963441
C10orf113	0.159508918	0.00296559	1.63641594
C10orf120	0.037474061	0.001487518	1.709122391
C10orf82	0.335234423	0.003581126	1.623355143
C10orf90	0.106255268	2.96E-07	2.266632016
C10orf91	0.176450504	2.34E-09	2.779210184
C11orf24	41.35138641	8.13E-05	1.812044157
C11orf40	0.058142924	3.43E-06	2.146975989
C11orf45	3.334612438	3.07E-05	1.869483282
C11orf54	120.7073787	2.68E-08	0.389459054
C11orf58	64.86346802	0.000621461	0.589887756
C11orf91	0.764812071	0.002591722	1.596899746
C11orf98	12.25719388	2.97E-06	2.022225981
C12orf45	19.09538274	5.35E-05	1.839539123
C12orf49	34.26517699	1.77E-05	0.489637202
C12orf50	0.045464646	6.75E-05	1.834825019
C12orf73	10.56673438	0.001765094	1.60631615
C16orf72	16.3801326	3.62E-05	0.52472824
C16orf74	16.7738502	0.001215138	1.648537779
C16orf82	0.023652023	0.000115299	1.83063807
C16orf86	7.705972091	0.004583512	0.635378315
C16orf90	0.135221473	0.004505618	1.54348429
C16orf95	0.995552264	0.000850038	1.655938042
C17orf107	10.11521801	0.00027695	0.54801105
C17orf47	0.155417774	1.01E-05	1.934289496
C17orf49	2.599387817	4.81E-06	1.986595645
C17orf50	0.178372332	0.000571102	1.715054996
C17orf53	3.163554841	1.37E-06	2.057583152
C17orf64	0.624590857	0.001412547	1.658185271
C17orf67	2.16886711	0.000895357	1.648227459
C17orf80	22.85255425	0.001946336	0.617723098
C18orf25	15.1172385	0.002888412	0.623772484
C19orf18	3.027474798	0.000568972	1.680700193
C19orf38	8.426607007	0.003365649	1.558909017
C19orf48	42.14009191	0.00127345	1.635409864
C19orf53	249.1964516	0.00350816	1.56017577
C19orf66	29.11932853	0.0002258	1.741612927
C19orf73	1.573082784	0.000828437	1.663827312
C19orf81	1.149674001	0.000111287	1.868779158
C1D	18.43219079	0.000139113	0.549943355
C1DP1	5.064970868	0.000221152	0.510324786
C1DP3	0.007567994	0.000284604	2.935203906
C1GALT1C	60.29283946	0.000373967	0.563492792
C1GALT1P1	0.412059651	1.48E-05	1.914526682
C1GALT1P2	0.310399131	0.001344839	1.64865011
C1orf100	0.294701352	0.003351273	1.559656385
C1orf109	12.86749652	0.001893061	0.617751129
C1orf115	83.24741531	0.000177979	0.556210213
C1orf122	36.18158237	8.99E-05	1.803357351
C1orf131	6.658545412	0.001409919	1.620988545
C1orf143	0.179090272	0.000835565	1.686873782
C1orf146	0.199407648	8.00E-05	1.811981688

C1orf147	0.353692811	3.68E-05	1.855318147
C1orf159	5.368879701	1.07E-06	2.060240555
C1orf174	17.99483081	0.001418355	1.618222207
C1orf194	0.387339589	1.89E-05	1.918185538
C1orf210	29.68681485	7.41E-09	0.3735934
C1orf216	18.2588356	0.004031902	1.549275787
C1orf220	0.945527985	0.00286342	1.574061584
C1orf35	12.44860828	0.000113381	1.793212418
C1orf43	377.1612483	0.000189572	0.554255884
C1orf53	7.83139114	2.59E-08	2.30394881
C1orf61	0.090420466	0.00246392	1.750345628
C1orf74	4.15356639	5.29E-05	0.515287667
C1orf94	0.046185255	0.001889118	1.809283909
C1QA	777.0799121	0.001096176	1.638863868
C1QBPP2	0.337265049	2.27E-05	1.88360768
C1QL1	110.5183855	0.001106041	1.66695535
C1QTNF1	56.39097937	7.33E-05	1.819677163
C1QTNF1-A	1.968633823	4.52E-06	2.020231787
C1QTNF5	0	0.004606615	1.557019068
C1QTNF6	14.40342698	1.39E-06	2.056174851
C1R	238.9750193	4.04E-06	1.994311169
C1RL	53.73170579	1.23E-07	2.204190221
C1S	252.3798144	7.30E-06	1.959613132
C2-AS1	0.687438348	0.000167901	1.781828753
C20orf141	0.17537405	5.45E-08	2.395595688
C20orf144	0.272162867	6.32E-05	1.82687191
C20orf203	0.197150657	5.13E-07	2.123697596
C20orf204	0.943498614	0.001983491	1.594250979
C21orf58	2.231869813	1.64E-05	1.902809804
C21orf91	12.57328681	0.000476242	0.565828047
C22orf31	0	0.004606615	1.557019068
C2CD2L	6.269628299	0.003188971	1.568690681
C2CD6	0.514641242	0.000623013	1.687212943
C2orf27A	7.532750961	0.000467482	1.695024669
C2orf40	10.45197977	0.001237583	0.532428828
C2orf66	0.281776429	7.58E-06	1.95414979
C2orf91	0.128955644	0.000646733	1.682929667
C2orf92	1.997843352	0.003376153	1.55439809
C3orf35	0.805631594	0.000237855	1.732260105
C3orf38	20.17635049	8.94E-05	0.532554445
C3orf70	9.122841976	0.000404796	0.565029497
C3orf85	0.757938772	0.003657967	0.544315444
C3P1	0.246066238	0.000376052	2.172019842
C4BPA	4.165148151	0.001674987	2.074096468
C4orf19	14.33972307	6.01E-06	0.469952019
C4orf3	256.3121839	6.86E-09	0.382620482
C4orf48	9.943185637	3.21E-06	2.03461315
C5orf24	69.7399403	0.000736005	0.591497199
C5orf34	2.513410641	0.000111775	1.782832361
C5orf38	2.036836633	0.003309816	1.661095825
C5orf58	0.733034158	0.002296299	1.590866433
C5orf60	0.068724727	0.003361195	1.571126233
C5orf66	0.400737199	1.07E-06	2.054988401
C6orf118	0.107065179	3.03E-07	2.158178713
C6orf141	2.007124735	1.18E-10	2.644370127
C6orf223	65.03554217	0.004206054	0.613624916
C6orf226	21.946758	0.002399937	1.583660865
C6orf47	49.93034334	3.99E-06	0.496163073
C6orf47-AS1	0	0.004606615	1.557019068
C6orf89	73.59324259	1.57E-05	0.508755571
C7orf43	15.97124866	2.47E-06	2.040917806
C7orf61	1.039953808	1.71E-12	2.791174492
C8G	4.878085204	3.05E-07	2.294701157
C8orf31	2.138364765	0.004250225	1.595966783
C8orf44	6.464319743	0.0001666	1.75473515
C8orf49	0.041378008	0.004646393	1.827680198
C8orf59	39.4374468	4.31E-09	2.435264448
C8orf74	0.024923085	0.003065967	1.611102372
C8orf76	7.571876069	1.38E-07	2.198470818
C9	1.575104195	8.03E-05	1.869749824
C9orf139	0.810260141	0.000142422	1.768413891
C9orf16	56.34961015	0.00164819	1.618674451
C9orf163	0.611460518	2.84E-06	2.018561745
C9orf170	0.206577692	0.003471454	1.587782541
C9orf64	42.31358818	0.001122465	0.604122814
C9orf66	14.8607082	0.002623663	0.612011326
C9orf85	6.622403451	0.000404097	0.575214925
CA15P1	0.77499368	0.001098309	1.640804334
CA4	17.46305995	0.000694985	0.538241422
CA5B	5.336361412	2.85E-06	0.440130332
CA6	0.068086855	0.002018851	1.600261659
CAAP1	29.33525766	0.002321929	0.606939515
CAB39	71.88101992	0.00366889	0.636257155
CAB39L	12.73920861	0.0016398	0.578303729
CABLES1	26.13895013	3.13E-05	0.510874511
CABLES2	7.150651988	1.93E-05	1.902406933
CABP5	0.068286527	2.26E-05	1.948634465
CABP7	0.367164427	8.37E-07	2.081184134
CABYR	1.900581367	0.000453607	1.698663144
CACHD1	8.777864507	0.003640975	0.624374112
CACNA1B	0.260857286	0.004992662	2.336115022
CACNA1G	0.137167062	0.002804691	1.950155066
CACNA1G-.	0.189471941	0.000642842	1.703164987
CACNB1	2.981754165	8.63E-05	1.800815613
CACNB3	8.921074499	0.004143305	1.55450921
CACNG2	0.050389428	0.001485629	1.728433368
CACNG6	0.348206073	0.00011866	2.575930574
CACYBPP1	0.21324606	3.26E-05	1.888094555
CADPS2	25.29481539	1.60E-05	0.497777293
CAGE1	0.050182961	0.000311785	1.720128109
CAHM	2.447809578	5.59E-08	2.225761607
CALB2	1.559107532	0.001210366	1.726476654
CALCOCO2	72.4247003	0.000139957	0.550868596
CALCRL	87.96857362	3.95E-06	0.452995375
CALD1	249.9464567	0.001433335	0.603469889
CALHM4	0.146921906	0.000107754	0.49491822
CALHM5	5.708576721	0.000671993	0.575861058
CALM1	246.3419348	9.91E-06	0.49100736
CALM3	440.6015436	0.002998626	0.63532451
CALML5	0.096393865	0.000453011	1.873390534
CALML6	0.288735795	5.37E-06	1.967352853
CALR3	0.094948914	0.004252361	1.548668143
CALR4P	0.191453442	0.000173167	1.772021106
CAMK1	17.38746995	0.000110976	0.536947297
CAMK2N1	193.9277868	0.002896377	0.626624555
CAMK2N2	1.593967298	0.000315719	1.739999528
CAMSAP1	19.22989859	2.87E-06	0.466610334
CAMTA1-D	1.526737445	0.001449986	1.624630571
CAND1	37.06707074	0.00145768	0.608871463
CANX	878.898187	0.001599164	0.605289797
CAP1	375.5139027	0.000311662	0.579742007
CAP2	29.16630945	0.003914973	0.631777236
CAPN10	8.6610432	0.001147344	1.630660282
CAPN10-DT	2.317486008	4.20E-06	1.984893648
CAPN3	3.301749567	0.000855457	1.698932415
CAPN8	0.954101943	0.004076129	1.881198151
CAPRIN2	9.816561735	0.002625159	1.57053516
CAPS	9.344859316	1.39E-05	1.919234573
CAPZA1	136.9473828	6.51E-06	1.980401175
CARD11	17.03620902	0.000218801	1.764772862
CARD14	1.811909827	6.62E-06	2.018887518
CARD19	25.94320319	0.001066935	1.640787162
CARD9	3.143866753	0.004926258	1.532119665
CARMIL1	14.48484463	0.00128157	0.595550834
CARMIL2	2.146653329	0.001146275	1.645654995
CARNMT1	20.54777721	0.000728643	0.586509374
CARNS1	0.933935129	0.002853632	1.580348356
CARS	19.91119022	3.99E-08	2.26924943
CARS2	18.6904477	1.95E-06	2.047131245
CARSP2	0.012091051	0.003933413	3.41628326
CASC10	5.813856509	0.002669012	0.613213756
CASC11	0.115774841	0.000421472	1.703776647
CASC17	0.016383207	0.000453181	1.783423964
CASC18	0.676361406	0.000123758	1.798569687
CASC20	0.079257018	0.000296052	1.807928555
CASC4	106.4918888	0.002133233	0.618000268
CASK-AS1	0.579617097	0.000127544	1.776498108
CASKIN2	47.20893835	5.21E-06	0.479370192
CASP4	35.22625114	0.000612725	1.687920923
CASP5	1.246879197	0.003026775	1.570455562
CASP9	12.3632548	0.003977222	1.547734161
CASR	2.250990407	9.89E-05	0.423206097
CAT	217.9321911	2.05E-05	0.504691701
CATIP-AS1	0.643821282	0.002699808	1.576433321
CATSPER1	1.013368274	1.49E-08	2.315920385
CATSPERB	0.462368015	0.002674852	1.604848505
CATSPERD	0.111864109	0.000168446	1.823273062
CAVIN2	130.5443815	3.43E-06	0.43307926
CAVIN4	0.783292625	1.40E-05	2.025207966
CBLL1	23.98563974	0.004866706	0.641608874

CBR3	12.82638697	0.002545133	1.585799812
CBR4	22.0198454	0.0024241	0.613288662
CBS	0.104588429	7.01E-06	2.087611948
CBWD1	3.487251157	0.001272416	0.601389077
CBWD4P	1.891352921	0.001671298	1.614295637
CBX3P3	0.15467523	1.78E-05	1.935190439
CBX3P4	0.612482502	0.000744916	1.670982077
CBX4	26.25391531	4.59E-07	2.155309069
CBX7	50.12500865	0.001844701	0.615474843
CBX8	6.306818707	7.91E-08	2.23014098
CC2D2A	17.12509686	4.94E-07	0.439708982
CCAT2	0.116210476	0.001812285	0.573866164
CCDC110	5.750987036	0.003431525	0.625114896
CCDC114	0.641942548	0.000824816	1.677296065
CCDC115	61.63588619	0.00422473	0.64568187
CCDC117	3.190752246	0.002989918	0.631532491
CCDC121	7.156125272	1.68E-09	0.367190087
CCDC130	30.0370428	0.000122637	1.778071267
CCDC134	13.17485284	0.000681564	0.567467789
CCDC136	1.063102385	1.33E-05	1.963995003
CCDC137	26.98463698	1.08E-08	2.345833047
CCDC14	13.61782524	1.74E-05	1.899528553
CCDC140	0.009308363	0.001215885	1.825214952
CCDC149	8.43880663	4.07E-06	0.471543424
CCDC154	1.608755983	1.05E-06	2.06850335
CCDC155	0.065529454	0.001989341	1.660778606
CCDC158	1.311759289	0.001411244	0.551066059
CCDC162P	0.522062226	0.003147183	1.635670086
CCDC167	56.24980815	0.000213966	1.748860356
CCDC17	1.670360803	0.000114008	1.782285844
CCDC171	0.965437067	0.000217034	0.5494195
CCDC18	2.554067332	3.06E-05	1.868619237
CCDC18-AS	10.60041729	0.001051594	1.635512409
CCDC186	13.1516917	3.02E-06	0.465283292
CCDC189	1.439738181	8.95E-06	1.940467877
CCDC194	0.689714709	4.98E-05	1.842784408
CCDC197	0.065984035	0.001957745	1.769890628
CCDC24	11.53462918	2.62E-05	1.879518401
CCDC25	39.17025221	5.03E-06	0.489713641
CCDC28A	94.36642968	0.000151253	0.551653734
CCDC28B	8.714452842	1.16E-07	2.199440986
CCDC34	11.64410967	1.38E-05	1.919342313
CCDC34P1	0.05096617	0.002484016	1.730963011
CCDC40	4.479672212	0.000150253	1.767084676
CCDC42	0.31734984	0.004314602	1.541383686
CCDC43	35.61230152	2.11E-05	0.518344921
CCDC47	123.8266778	0.00022455	0.564836219
CCDC57	8.33082758	1.37E-05	1.914719616
CCDC6	45.04241481	2.73E-05	0.497326744
CCDC63	0.053846923	0.000283067	1.788028563
CCDC71L	22.95130152	0.000137403	1.782492606
CCDC74A	16.78999559	2.26E-06	2.028305719
CCDC74B	5.43960796	9.28E-05	1.800790684
CCDC78	2.286669083	0.000141159	1.83366294
CCDC8	22.88474848	0.004164397	0.618960309
CCDC83	0.058116259	0.000700027	1.717773562
CCDC84	14.09971146	1.86E-05	1.884508888
CCDC85A	3.4985071	7.34E-05	0.513779104
CCDC85C	5.38324836	0.002085609	0.599823896
CCDC87	1.434071014	0.002653324	0.618863486
CCDC88B	9.508522788	0.000169607	1.764352021
CCDC89	8.753756427	6.05E-05	0.523646124
CCDC91	28.32040237	0.001230066	1.826936867
CCDC9B	7.427375015	0.002463882	1.592619516
CCER2	1.978908525	1.85E-07	2.179569105
CCL11	1.709032828	0.000465184	1.730895756
CCL13	3.432173769	0.000156197	1.815836055
CCL25	0.319825893	0.001870789	1.617761603
CCL7	0.66268847	2.01E-05	2.042311136
CCM2	27.59005299	1.01E-07	2.242030899
CCNA1	1.220358849	6.33E-07	2.370403203
CCNA2	10.16665426	3.23E-08	2.272593181
CCNB1	20.76576909	0.000158327	1.790180706
CCNB2	7.954684998	3.96E-10	2.514116576
CCNB3P1	0.043484838	0.00026304	1.793823625
CCND1	790.1037157	0.000477053	0.558729655
CCNDBP1	36.50806849	3.23E-07	0.45122946
CCNE1	3.258227628	3.17E-12	2.777233093
CCNF	3.661107109	4.66E-15	3.107770491
CCNG1	278.9877213	4.96E-05	0.519655021
CCNG2	53.30592314	0.002010289	0.598505789
CCNI	534.1518056	9.07E-05	0.539567234
CCNL2	61.26504539	1.10E-05	1.921409486
CCNQP1	0.038665126	9.65E-06	2.053708716
CCNY	82.17147611	0.00463004	0.637969163
CCNYL1	25.13339409	4.58E-05	0.512568667
CCNYL2	2.595413638	1.12E-07	2.21356415
CCR10	2.672018025	0.001208839	1.633290582
CCRL1P1	0.041435058	0.000895162	1.669164276
CCSER1	0.851352425	0.000110771	0.512754584
CCSER2	28.97044606	0.000415693	0.568532873
CCT4P2	0.079980012	0.000436211	1.747974381
CCT5P1	0.136630047	0.000193318	1.789217537
CCT6P1	10.35978716	0.003076133	1.562969099
CCT6P3	3.240907476	0.000891199	1.650129371
CD14	264.6135652	0.00277076	1.573609114
CD164	224.137141	0.000157138	0.551215074
CD19	1.129478389	0.001807341	1.6686774
CD1E	2.578998002	0.002659042	0.579472966
CD27-AS1	27.00864784	0.000469136	1.690021271
CD2AP	56.85451884	0.000247194	0.560752348
CD300E	5.356416303	0.003692825	1.560253901
CD302	11.11390274	0.001229274	0.593083202
CD34	123.0565437	0.001336197	0.601585758
CD44	80.32690219	8.06E-07	2.108014822
CD44-AS1	1.158219347	1.02E-07	2.274723695
CD46	135.6308799	0.000205633	0.556721594
CD59	462.4137017	0.001772625	0.60012048
CD72	10.02771674	0.001015618	1.648007335
CD82	38.34821689	0.000360247	1.722851678
CD93	196.5274063	0.002004313	0.601796671
CDADC1	13.75397869	2.63E-05	0.509248064
CDC14B	13.66387726	5.65E-07	0.4459563
CDC20	13.07578964	1.35E-10	2.590173009
CDC23	36.75888451	0.00021141	0.556093143
CDC25A	1.494901428	0.000102819	1.801397919
CDC25B	45.03906897	2.69E-06	2.018236784
CDC25C	1.881416571	9.38E-07	2.087249
CDC37L1	20.20082825	0.00087579	0.581290315
CDC37P1	0.335380208	0.000135651	1.78483188
CDC37P2	0.465119393	5.88E-07	2.108618133
CDC40	16.86731923	0.00239721	0.625192188
CDC42	271.3166916	0.000110458	0.550346354
CDC42BPA	32.54795802	4.22E-05	0.516189688
CDC42BPB	77.75964024	4.45E-05	0.518812917
CDC42BPG	8.391989128	8.63E-05	0.516799862
CDC42P2	0.514276278	2.00E-06	2.571793154
CDC42P5	0.515485019	0.002220998	1.594737814
CDC42P6	2.821106327	8.24E-05	0.466563717
CDC42SE2	104.3935374	2.49E-05	0.513859275
CDC45	4.136479792	3.26E-06	2.016331123
CDC6	4.527812838	0.001856343	1.61076613
CDC7	6.324339731	3.13E-08	2.267186745
CDC73	43.08143533	2.35E-05	0.516351986
CDCA2	25.42907085	0.000889382	0.584349038
CDCA3	3.10031625	1.41E-14	3.048476032
CDCA4P3	0.054451249	0.000290551	1.847172697
CDCA5	5.912139163	1.02E-08	2.338353384
CDCA7	4.49992256	2.49E-07	2.157064704
CDCA8	7.677315136	4.80E-12	2.747056994
CDH1	80.80539834	0.002520464	0.600044575
CDH13	40.8236396	0.000766192	0.575846948
CDH15	0.54744416	0.000832897	1.811913851
CDH16	278.7987355	0.002033071	0.598808154
CDH18	0.095057717	8.11E-05	2.208367737
CDHR2	22.34187188	0.002174603	0.560744874
CDHR4	0.284513157	3.20E-09	2.518678152
CDK1	9.892958356	8.87E-07	2.083894302
CDK11A	4.831540202	9.08E-06	1.938261699
CDK16	56.00903876	0.002284232	1.598043563
CDK19	31.4317241	0.002556328	0.616323172
CDK2AP2P1	0	0.004606615	1.557019068
CDK3	1.548933771	7.79E-06	1.939536528
CDK5R2	0.473253273	1.64E-07	2.438217652
CDK5RAP1	17.76345376	5.68E-05	1.829921492
CDK5RAP3	61.99203773	1.07E-05	1.923995325
CDK8P2	0.090616295	0.002106685	1.617973814
CDKAL1	17.66939995	0.000281515	0.565759296
CDKL1	4.514529882	1.34E-05	0.47015954
CDKL2	6.435486448	5.08E-06	0.459685955

CDKL4	0.215384	0.003036347	1.635689199
CDKL5	9.054780123	0.000245537	0.545081235
CDKN1B	110.6504831	0.002635707	0.623722257
CDKN2AIP	30.53664703	0.000583936	0.586042092
CDKN2B	15.11158982	0.004577006	0.628645726
CDKN2B-A	0.693825815	5.87E-06	1.969732994
CDKN3	6.359171575	6.18E-08	2.244596939
CDRT15	0.292095664	5.61E-08	2.236664902
CDRT15P1	1.512968015	0.000276499	1.727736887
CDRT4	0.878850318	0.002190428	1.583232928
CDRT7	0.109207865	2.26E-05	1.984137348
CDS1	23.70709904	3.39E-06	0.443299694
CDS2	40.74802313	0.002760813	0.618775838
CDY12P	0	0.004606615	1.557019068
CDY14P	0	0.004606615	1.557019068
CDY15P	0	0.004606615	1.557019068
CDY17P	0	0.004606615	1.557019068
CDY18P	0	0.004606615	1.557019068
CDY19P	0	0.004606615	1.557019068
CDY23P	0	0.004606615	1.557019068
CDYL	16.70451682	0.001385507	0.602695211
CDYLP1	0.019442147	0.000105763	1.900653063
CEACAM1	18.41168442	6.79E-05	0.522811904
CEACAM16	0.089424917	0.000767904	1.692230629
CEACAM19	3.939382941	0.000382881	1.705958147
CEACAM22	0.216036895	1.14E-10	2.5829142
CEACAM4	2.243978195	3.47E-05	1.871304894
CEACAMP1	0.077957649	0.000304881	1.884307197
CEACAMP9	0.006515812	0.001321564	2.446293249
CEBPB	101.3217168	1.55E-08	2.313878531
CEBPD	215.6798243	0.00228371	1.587629636
CEBPZ	60.68437714	6.47E-07	0.452019638
CELA2A	0.125148151	0.000797639	1.669486626
CELF3	0.064289063	0.000695771	1.685613398
CELSR3	0.867783411	2.74E-05	1.9270524
CEMP1	0	0.004606615	1.557019068
CENPA	2.938501918	5.76E-10	2.504078668
CENPBD1	6.850710922	2.47E-05	0.514203335
CENPCP1	0.086333054	0.004318375	0.616382971
CENPE	1.794319098	2.05E-07	2.177765067
CENPF	5.206306531	3.78E-07	2.142971086
CENPH	8.846944295	0.000574804	1.682716622
CENPI	1.605984174	0.00368681	1.564361998
CENPK	2.930925817	3.28E-05	1.867416266
CENPM	7.289832867	1.90E-05	1.902216818
CENPO	5.585621235	0.001541986	1.612749581
CENPT	22.26976441	1.86E-06	2.024723172
CENPW	11.62171617	2.64E-11	2.671708058
CEP104	21.31357628	3.07E-06	0.469197597
CEP120	25.03808276	0.000662392	0.582513504
CEP131	13.47572033	0.002662078	1.573188846
CEP164	8.753882585	0.004949767	1.52674877
CEP290	10.9578646	0.003885924	1.547724096
CEP350	16.47775394	0.001595026	0.602770959
CEP55	7.378735643	1.37E-09	2.452627362
CEP68	21.33302867	1.92E-06	0.454213746
CEP83	5.110824255	0.000141325	1.775897897
CEPT1	15.56240206	0.001550614	0.609683532
CERCAM	35.64458204	2.61E-06	2.030039196
CERNA1	1.041236209	0.002568803	0.609219262
CERS6-AS1	0.046173099	0.000276917	1.731812399
CES2	191.2856858	0.000113876	0.534793704
CES5AP1	0.202860547	0.000951101	1.780515316
CFAP100	0.153138381	0.000646828	1.67868117
CFAP126	0	0.004606615	1.557019068
CFAP161	0.475207574	0.000114788	0.518242761
CFAP221	3.819576854	0.00189453	1.600362547
CFAP298	22.50898286	0.000651874	1.673695111
CFAP300	3.606964899	2.65E-05	1.884930309
CFAP44	2.773966443	0.002195264	1.585140669
CFAP44-AS	0.115286932	8.98E-05	1.806640781
CFAP45	1.096172372	1.64E-08	2.304893717
CFAP47	0.466291743	0.000157016	1.822880292
CFAP54	0.243045761	0.000536121	1.690124403
CFAP73	0.71967969	0.000143112	1.773505452
CFAP97	38.22731688	0.001089251	0.602137924
CFB	21.33461399	2.04E-08	2.305104422
CFHR3	0.452048777	3.00E-06	2.093208033
CFL1P1	0.774350977	0.001534525	1.613824001
CFL1P6	0.418333624	5.37E-05	1.832718721
CGB2	0.022895224	0.000220497	2.029096891
CGB5	0.023798745	0.001253069	1.825809593
CGGBP1	62.18774186	0.000254677	0.558702223
CGN	10.89514741	0.000138786	0.509457624
CGNL1	50.36265677	0.001770318	0.585135763
CGRRF1	11.41833002	0.000368089	0.567220109
CHAC1	4.571349743	1.12E-06	2.11474794
CHAF1B	3.93310955	4.02E-05	1.853852435
CHAMP1	21.12291704	0.000126707	0.551438851
CHAT	0.524872884	3.15E-10	3.168917801
CHCHD2P1	0.374287948	0.002499422	1.598993932
CHCHD2P6	4.595594849	0.004160595	0.633661959
CHCHD3P3	1.408149622	0.000830861	0.580565351
CHCHD4P2	0.155529702	0.001803708	1.636043568
CHCHD4P3	0	0.004606615	1.557019068
CHD6	17.13163797	1.13E-05	0.494931052
CHD8	28.83538734	0.000184135	0.550207516
CHD9	19.72690915	2.24E-05	0.504833177
CHDH	29.44335131	0.000312658	0.556223546
CHEK1	6.629012963	0.001175826	1.637792961
CHEK2	6.971899719	8.41E-08	2.220847473
CHFR	6.109048884	4.30E-08	2.285680079
CHGA	0.400663352	1.29E-07	2.614216191
CHI3L2	8.432833862	6.00E-05	1.873207883
CHIC1	14.98476056	4.42E-05	0.518878635
CHKB	7.899961794	7.40E-05	1.804326715
CHKB-CPT1	1.052274356	0.001401315	1.622922361
CHM	16.80080912	1.48E-07	0.430595349
CHMP1B	101.1257056	1.22E-05	0.49913382
CHMP1B-A	0	0.004606615	1.557019068
CHMP2B	52.70502242	9.66E-05	0.534305101
CHMP3	53.40425283	4.24E-07	0.449146278
CHMP4BP1	2.298559678	6.68E-10	2.486115379
CHMP4C	29.98516403	0.000772623	0.592674203
CHMP5	147.1386139	4.95E-07	0.451155128
CHPF2	62.0697529	0.003440538	1.557636079
CHRM3	2.788227059	1.75E-07	0.361048753
CHRNA10	1.466173829	0.001559474	1.631412837
CHRNA3	0.107430498	0.00034547	1.803112314
CHRNA5	0.766054204	1.42E-05	1.947495449
CHRNA9	0.079317143	6.31E-05	2.285452218
CHRNB4	0.379220272	3.78E-05	2.079676337
CHRNG	0.100861632	9.92E-05	1.800803148
CHST4	0.186141672	7.27E-07	2.206008109
CHST5	0.134830551	0.000498897	1.691797569
CHST6	0.226576144	3.40E-05	2.041093188
CHTF18	5.948646313	2.11E-09	2.413788885
CHTF8	98.87360668	2.99E-06	0.482035359
CHUK	16.14513103	0.00074593	0.585596151
CHURC1	40.9741059	0.000117362	0.541013338
CHURC1-FN	0.205052801	0.000101798	0.524658649
CIB1	364.023983	0.003207502	1.562540226
CIB3	0.142873191	0.000303599	1.750075057
CICP11	0.0388651	0.0041238	1.593184877
CICP17	0.034625032	0.002636712	1.614606834
CICP5	0.043051234	0.002219598	1.612209411
CIDECP1	7.731463592	1.05E-07	2.211986322
CILP	1.680261511	7.07E-12	3.046752206
CILP2	1.644179461	0.000151385	2.103126512
CIPC	21.33315457	6.02E-05	0.468367003
CIR1P1	0.102400402	0.004679789	1.61327612
CISH	35.95217914	0.004003832	0.580367208
CITED2	214.0203799	0.004467795	0.6156007
CKAP2L	2.633599451	7.96E-07	2.097522669
CKAP4	96.01916037	1.47E-06	2.054132246
CKLF	24.35570068	7.30E-06	1.984121127
CKM	0.455885038	0.000335209	1.745498168
CKS2	72.08608079	0.00220704	1.607519863
CLASP1	33.01153609	0.003272241	0.635169339
CLASP2	11.85728409	0.00011089	0.530093035
CLASRP	25.38779996	1.64E-05	1.905725882
CLCA2	0.089733871	6.34E-06	2.057202396
CLCA3P	0.028719377	3.06E-07	2.143877193
CLCC1	19.53278542	0.003985021	0.637963659
CLCN4	14.56541914	0.002164032	0.605743691
CLCN5	35.17158283	9.28E-06	0.478703402
CLCN7	33.01159621	0.003619903	1.554215603
CLDN10	33.74578305	4.21E-07	0.442291783
CLDN11	1.632703631	0.001381754	1.65616742
CLDN14	1.927292034	0.00243981	1.629919652

CLDN22	0	0.004606615	1.557019068
CLDN4	92.65982161	0.000233181	0.524341035
CLDN9	1.167507762	0.00042006	1.725569352
CLDND2	3.306631578	1.30E-06	2.057942251
CLEC14A	171.6886989	0.000648309	0.571598813
CLEC18B	36.48229608	3.14E-05	0.492928214
CLEC19A	0.035948068	0.000102865	1.855358329
CLEC2B	31.86336262	2.49E-06	2.029081565
CLEC2D	10.222878	0.001177909	1.629390717
CLEC2L	0.090996322	0.000865071	1.774581226
CLEC3B	126.3987495	0.001914326	0.597172692
CLEC4G	0.380049664	9.25E-06	1.952640274
CLEC4GP1	0.633115422	1.04E-05	2.212541899
CLGN	4.073882922	3.49E-05	1.930352857
CLHC1	2.677292298	0.003648059	1.548114443
CLIC2	31.28284802	0.002541523	0.626034365
CLIC3	6.817550659	0.000411779	1.728543284
CLIC4	586.0277084	3.02E-06	0.468257779
CLIC5	7.45836816	6.75E-05	0.478617628
CLINT1	77.61207605	1.78E-05	0.499538879
CLIP3	25.67162569	0.00235106	1.586903446
CLK2	45.70642066	2.50E-05	1.873773553
CLLU1OS	0.449554307	0.001089922	1.780070001
CLMN	16.51800059	2.13E-05	0.473405809
CLMP	5.4446656	4.79E-07	2.331682405
CLN5	24.14195891	3.66E-05	0.518388922
CLOCK	16.42509769	0.000149925	0.542884521
CLP1	15.64886943	0.000397679	0.580163712
CLPB	15.69392057	0.001583745	0.586100475
CLPS	0.110741363	0.004600898	1.651971454
CLPSL1	0.112311197	4.64E-05	1.94010567
CLPX	31.3919911	0.000625754	0.583285389
CLRN3	156.8675881	0.000746559	0.593947387
CLSTN1	217.5253976	3.26E-05	0.506302607
CLTRN	409.8446108	3.28E-07	0.416866239
CLUHP8	0.034258335	0.001843626	1.786377647
CLVS1	0.163995162	0.000166566	2.133416726
CLYBL	13.39468676	0.002329585	0.608308121
CMA1	0.939450722	0.000178204	0.459762503
CMBL	312.350772	1.77E-05	0.492687882
CMTM3	64.08650129	0.001344777	1.628050529
CMTM7	18.44575272	6.06E-05	1.829736415
CMTM8	17.46426516	0.000473381	0.563877802
CMTR2	15.76965292	0.000238842	0.556088454
CNDP2	379.5671337	1.24E-05	0.497235479
CNFN	7.838309685	0.004205873	1.609582349
CNGA1	2.337919652	0.000362415	0.50408018
CNGA3	0.039642375	0.00053693	1.814713825
CNGA4	0.524855715	8.19E-05	1.819887096
CNIH1	66.34980415	0.001283626	0.602915778
CNIH2	1.206559905	1.19E-06	2.063517574
CNKSR3	12.32020847	0.001311067	0.595130286
CNN2P1	0.546496162	0.00225903	1.58917587
CNN2P10	0.036685667	0.000302958	1.850968168
CNN3	452.781684	0.000324391	0.566670428
CNNM2	5.744932381	2.56E-05	0.487141538
CNNM3	42.84565822	0.000157867	0.547052216
CNOT1	64.38678754	0.001360916	0.605174152
CNOT4	18.40795198	1.48E-05	0.506551742
CNOT6	27.92618117	1.59E-05	0.49359867
CNOT6L	32.35748956	2.94E-05	0.512937082
CNOT7	34.36391822	0.000128473	0.552606152
CNOT8	69.90055752	0.000188362	0.559279815
CNPPD1	235.3355279	0.000870805	0.589710122
CNPY1	0.152062836	3.08E-05	1.964592517
CNPY2	34.64021003	4.66E-06	1.985559974
CNPY3	177.0240282	0.000692016	1.676427109
CNST	22.82795496	0.000371148	0.577068962
CNTD1	1.394973617	0.000109104	1.784828201
CNTD2	0.515363559	9.08E-07	2.105553872
CNTFR-AS1	0.071276172	0.001452711	1.663553186
CNTNAP1	10.33717088	7.12E-08	2.22204773
CNTNAP3P	0.182865844	0.002585493	0.59624496
CNTNAP3P	0.986188647	0.000328329	0.543749964
CNTNAP5	2.35281315	0.000350486	0.384049857
COA6-AS1	8.912712869	8.64E-06	1.949179173
COA7	16.34447434	0.003585844	0.632326215
COBL	8.192506051	4.76E-05	0.513617349
COG3	23.29405326	0.002986425	0.632225664
COG5	18.03367847	0.001068676	0.59574657
COG6	15.65062047	4.53E-06	0.490117449
COL11A1	4.943079361	0.000289721	1.943995182
COL18A1-A	0.680944418	6.92E-05	0.393483637
COL20A1	0.088981983	0.002582565	1.68914187
COL21A1	8.900915632	0.000321001	0.538488542
COL22A1	3.209327769	5.59E-08	2.268978379
COL25A1	3.744307414	1.01E-05	0.435869641
COL2A1	0.093405575	0.000212936	1.976794665
COL4A3BP	32.91091967	8.11E-06	0.484346536
COL4A4	10.98637214	9.44E-06	0.44235686
COL4A5	5.399214746	0.002088453	1.613156404
COL5A1-AS	0.211096705	5.35E-08	2.274870461
COL6A1	252.9673984	8.17E-06	1.954410875
COL6A2	387.4876714	6.14E-05	1.825270516
COL6A4P2	0.383384416	6.05E-09	2.368250629
COL7A1	4.04006849	1.46E-12	3.08995005
COL9A2	6.375641565	0.000579177	1.715894288
COLGALT1	124.3744006	3.35E-06	2.000288193
COLGALT2	1.639810835	0.000259697	1.8809027
COLQ	2.432445268	0.000169921	1.755665848
COMMD2	32.7329982	0.000662578	0.585279389
COMMD4	25.03203083	0.000115709	1.788833875
COMMD5	31.40281096	0.000179387	1.766024715
COPS2	51.4678652	0.000361645	0.573163931
COPS4	41.87742294	6.53E-08	0.43112975
COPS7B	18.08129576	1.76E-05	1.908566437
COQ10A	9.913107157	0.00231353	1.588767579
CORO1B	63.75390945	0.000611791	1.68931023
CORO6	1.733088934	3.36E-13	2.935305966
CORO7	6.946260199	7.77E-05	1.812702838
CORT	1.015367116	0.001192738	1.63257256
COX11	31.6358793	3.13E-05	0.51700998
COX15	57.45047489	7.50E-05	0.534583432
COX16	37.44784441	7.85E-06	0.473671085
COX19	8.645999922	1.34E-06	2.061369099
COX20	6.862177859	0.002651073	0.620746903
COX5BP2	0	0.004606615	1.557019068
COX5BP6	3.270197487	4.41E-06	1.982309388
COX5BP7	0	0.004606615	1.557019068
COX6B1P2	0.123530408	8.32E-05	1.934633058
COX6B2	0.208071547	1.65E-07	2.25024776
COX6C	124.5440534	0.004999908	1.538953217
COX6CP2	0.56810863	0.000247755	1.75910241
COX6CP7	0.053858565	0.001682406	1.961198368
COX7B2	0.036945267	0.000346185	1.91498671
CPA2	0.174622451	0.000193354	1.773903093
CPA3	27.65602797	3.79E-05	0.49449425
CPA4	6.048438826	4.45E-05	2.069486059
CPA5	0.173699841	2.34E-07	2.17330245
CPB1	0.507404197	0.000144081	1.861119136
CPEB1	1.066977628	3.40E-06	2.170688987
CPEB1-AS1	0.049079502	6.91E-05	1.941289476
CPEB3	5.454437262	0.004355557	0.620430904
CPEB4	36.54905852	0.000354358	0.54321538
CPHL1P	0.582340338	1.15E-06	2.085720242
CPLANE2	13.06530373	1.16E-07	0.408126478
CPLX1	6.952165336	0.00419132	1.566744854
CPM	46.40088357	0.000250784	0.540078195
CPNE1	105.2340135	2.92E-06	2.025087431
CPNE3	82.34901243	1.77E-09	0.374499317
CPNE7	5.003373501	5.74E-08	2.433358537
CPOX	20.31794193	0.000488608	1.693623709
CPPED1	39.18966297	0.003331413	0.626795452
CPQ	104.197145	0.000240563	0.561110292
CPSF1	43.43886614	1.09E-05	1.928066689
CPSF4	18.85368854	0.000154876	1.766479265
CPSF4L	0.115915087	0.002697194	1.575965645
CPT1A	79.74458584	3.41E-06	0.471551305
CPT1B	2.932024048	7.05E-05	1.815684595
CPT2	44.16976612	2.56E-09	0.364146015
CPXCR1	0.00954462	0.001040057	1.881065682
CPXM1	12.80015964	3.38E-05	1.950489128
CR383656.1	0.002163811	0.001114247	5.411900273
CR391992.1	0.185391884	0.002307839	1.601918273
CR392000.1	0	0.004606615	1.557019068
CR559946.2	0.784682168	1.53E-05	1.901066406
CR769767.2	0.225718067	0.001217016	0.576776379
CRABP2	23.55310917	9.37E-07	2.220258504
CRADD	15.48418351	1.93E-06	0.462667709
CRAT	83.66894587	5.27E-08	0.409193509

CRB3	53.58051115	8.19E-10	0.376011172
CRBN	22.26482474	0.004481969	0.639105403
CREB1	22.7402372	0.000557829	0.58272266
CREB3	124.7469332	0.000806985	0.596843908
CREBBP	31.04450442	0.000105381	0.531002931
CREBL2	143.9707683	4.11E-07	0.456714526
CREBRF	21.99072881	2.76E-09	0.352034103
CRELD2	21.81739697	7.70E-07	2.103802939
CRIM1	224.6220272	2.38E-07	0.436397437
CRIP1P3	0.065703645	0.00214128	1.938170428
CRIPT	31.74075957	4.08E-05	0.516238508
CRISP1	0.014675201	0.000591738	1.783554296
CRNKL1	31.77340395	0.000248549	0.562170742
CROT	15.13234087	0.001605822	0.592249245
CRP	17.54193382	0.000376965	2.524176304
CRTC3	26.79104433	0.000306399	0.569985579
CRX	0.081481287	0.000152232	1.848389851
CRY2	59.03226714	3.47E-05	0.513313161
CRYBA1	0.361173783	2.18E-07	2.149338202
CRYBA4	0.199748394	2.45E-06	2.02908918
CRYBG2	0.667468528	3.77E-05	1.916058052
CRYGN	0.358176161	0.004096947	1.69078823
CRYGS	6.532765061	7.20E-05	1.821887624
CRYL1	277.7718813	3.27E-10	0.369702809
CRYM-AS1	0.50756955	0.001270431	1.632146102
CRYZ	214.2463098	1.27E-05	0.504059906
CSAD	20.58374736	0.000317254	1.713645262
CSAG2	0.068607854	0.000151253	1.899518312
CSAG3	0.129503719	0.000394469	2.024258041
CSDE1	407.2741324	0.000208004	0.560205566
CSF1	61.54283077	0.000636273	1.676096096
CSF2	0.319626856	8.00E-09	2.441599134
CSMD3	0.038229087	0.00019959	2.497928384
CSNK1A1	50.18133062	0.001766048	0.619488291
CSNK1D	52.32262133	0.001858759	1.598683271
CSNK1E	70.17615436	0.001941488	1.593553365
CSNK1G2P	0.100812565	0.002734078	1.586379538
CSNK1G3	28.27308773	0.001996232	0.609054564
CSPG4P10	1.063554391	0.003602516	1.55147047
CSPG4P1Y	0	0.004606615	1.557019068
CSPG4P2Y	0	0.004606615	1.557019068
CSPG4P4Y	0	0.004606615	1.557019068
CSRNP1	84.16318547	7.48E-05	0.487027506
CST2	2.102082558	9.94E-06	2.175608897
CST8	0.066927704	6.81E-07	2.126265861
CSTF1	29.67597074	1.53E-05	0.514174429
CSTF2T	40.6645825	0.000144827	0.552703008
CSTF3-DT	0.104742863	0.00456367	1.544534331
CT45A1	0.068986285	0.003987727	1.846356091
CT45A10	0.194674321	2.80E-05	2.783985536
CT45A11P	0.174060249	0.000809701	1.658614949
CT45A2	0	0.004606615	1.557019068
CT47A1	0	0.004606615	1.557019068
CT47A10	0	0.004606615	1.557019068
CT47A11	0	0.004606615	1.557019068
CT47A12	0	0.004606615	1.557019068
CT47A2	0	0.004606615	1.557019068
CT47A3	0	0.004606615	1.557019068
CT47A4	0	0.004606615	1.557019068
CT47A5	0	0.004606615	1.557019068
CT47A6	0	0.004606615	1.557019068
CT47A7	0	0.004606615	1.557019068
CT47A8	0	0.004606615	1.557019068
CT47A9	0	0.004606615	1.557019068
CT62	0.139667608	0.000408132	1.894220731
CTAGE6	0.090414507	5.49E-07	2.104348547
CTAGE7P	0.79724278	2.01E-06	2.028101226
CTBP1	57.28284608	0.002335978	1.588406689
CTBP1-DT	11.03387286	0.002508549	0.628487392
CTBP2	34.05560759	1.78E-07	0.43942822
CTBP2P2	0.071256905	2.07E-05	1.984114912
CTBP2P7	0.168454688	0.00182383	1.606656539
CTBS	23.57416383	0.002205432	0.613126826
CTCF	61.92774132	0.001815936	0.619972272
CTDSPL	45.99430196	1.94E-05	0.479412234
CTNNA1	245.8955035	3.19E-07	0.440746261
CTNNB1	222.113402	1.25E-05	0.487549196
CTNND1	193.4531795	1.25E-06	0.463084322
CTR9	42.76784216	2.35E-07	0.441320431
CTRB2	0.107456217	4.47E-05	1.87083375
CTRC	0.145778881	4.36E-06	1.998591655
CTRL	0.1221765	0.00147908	1.617410875
CTSA	168.817776	0.000741761	1.671004324
CTSG	3.476131224	0.002058265	0.558957614
CTSO	156.1516539	0.000940975	0.59776154
CTSZ	700.4698466	5.53E-06	1.980343616
CTTNBP2N	31.88377852	1.59E-06	0.46636901
CTU2	12.80327272	0.000111723	1.79380537
CTXN1	9.741966915	4.61E-05	1.871138447
CU634019.4	0	0.004606615	1.557019068
CU634019.5	0	0.004606615	1.557019068
CU638689.2	0	0.004606615	1.557019068
CU638689.3	0	0.004606615	1.557019068
CU639417.3	0	0.004606615	1.557019068
CU639417.4	0.035985312	7.50E-07	2.405907899
CUBN	148.495632	1.32E-06	0.422516708
CUBNP1	0.015153957	0.001732528	0.508528452
CUBNP3	0.364883992	0.000216487	0.393031624
CUL3	22.58560918	3.72E-05	0.52344366
CUL4A	40.5888254	0.000232207	0.566440717
CUL5	29.99099251	0.00016849	0.54696974
CWC22	39.15683107	0.000548679	0.577832859
CWC27	23.80573184	0.000681778	0.579349874
CWF19L2	22.45320409	1.34E-05	0.503722291
CX3CL1	178.6980685	1.05E-06	0.470257468
CXCL1	36.25539068	6.28E-07	2.224746391
CXCL13	20.10746398	0.000306936	1.77674755
CXCL1P1	0.002208932	0.001179146	4.46442171
CXCL2	33.32635834	6.62E-06	2.004421788
CXCL3	2.344210121	0.000204713	1.786445243
CXCL5	22.29594318	6.72E-05	1.980649399
CXCL8	22.05950109	0.001224791	1.731841395
CXorf51B	0.000249754	1.43E-07	29.75548679
CXorf56	32.17897238	0.000808741	0.594460836
CXorf65	1.513288649	0.001093944	1.644379293
CXXC1P1	0.041081839	0.000553847	1.789392944
CYB561D1	14.83727642	0.002777624	0.612602764
CYB5A	151.0550371	0.000922172	0.596283769
CYB5D1	3.994608589	5.98E-05	0.522778781
CYB5D2	55.84149866	1.34E-06	0.459451433
CYB5R2	1.643841027	0.000899983	1.658408195
CYBA	168.7317705	6.49E-05	1.831481987
CYBC1	37.68000094	5.64E-10	2.581996436
CYCSP10	1.140449997	0.004138471	1.541535536
CYCSP27	0.070874087	0.000441051	1.855662903
CYCSP28	0.185796694	0.002113723	1.621732073
CYCSP35	0.070080044	0.002192408	1.724812376
CYCSP40	1.314385646	5.60E-05	1.835298387
CYCSP45	0.371466585	0.000204477	0.518091532
CYFIP1	62.17023759	2.14E-06	0.478794
CYFIP2	79.17435565	3.51E-07	0.407825093
CYHR1	17.12820388	0.000315485	1.719631222
CYLC2	0.029118959	0.000126874	1.824820999
CYP19A1	0.150105817	0.000111511	1.828448082
CYP1B1-AS	0.733230347	5.75E-06	2.079109531
CYP21A1P	5.800282823	4.74E-05	1.843348411
CYP21A2	2.792136195	3.63E-06	2.010751905
CYP26A1	0.392435637	1.24E-07	2.376279137
CYP26C1	0.264408606	0.000392424	1.906443416
CYP2AB1P	0.037314427	0.000486635	1.763912057
CYP2B7P	0.499188375	1.53E-05	2.215990357
CYP2C9	3.161643912	4.08E-06	2.189190958
CYP2D6	2.25112561	0.00275924	1.591813614
CYP2D7	1.579865625	0.000205749	1.741930182
CYP2D8P	1.041928702	1.96E-05	1.886836687
CYP2E1	0.992776001	0.001133783	1.698895696
CYP2G2P	0.052330981	0.00028599	1.832413944
CYP2J2	225.0244935	0.003063602	0.614762993
CYP2U1	11.65826398	0.00498505	0.635775316
CYP3A7	2.538934081	1.67E-07	0.374563716
CYP4A11	92.13501753	0.000296415	0.491096424
CYP4F23P	0.206039933	0.000358421	1.840086982
CYP4F24P	0.24112612	0.000910364	1.699886492
CYP4F26P	0.121048373	2.17E-06	2.263551516
CYP4F29P	1.249102081	0.001350476	1.658787791
CYP4F35P	0.661600024	5.51E-05	1.856404887
CYP4V2	34.96350488	0.000142253	0.533177791
CYP51A1	3.411755444	1.21E-07	0.408887552
CYP7B1	17.81542204	0.000261944	0.538355475
CYS1	132.3104662	5.05E-06	0.481455781

CYSLTR1	4.600057956	0.000122273	0.541683332
CYTOR	21.84822289	1.07E-05	1.935591181
CYYR1	78.7847657	0.000194492	0.552986485
DAAM1	7.095772654	0.002668979	0.608777696
DAB2	271.1130936	0.000379504	0.57372601
DAB2IP	61.33437999	1.03E-07	0.414013711
DAGLB	19.52869152	0.003340078	1.561145786
DAND5	0.684988183	0.002911727	1.591172165
DAOA	0.017185359	0.000146247	1.841374973
DAP3	59.92231412	0.000176155	1.761917427
DAPK1	37.74186914	0.002373737	0.614091081
DARS	219.5589956	2.75E-05	0.521123894
DAW1	0.582009181	8.98E-05	1.939682865
DAZ4	2.29E-05	0	129.4818488
DAZAP1	34.70515701	0.001841012	1.600434032
DAZAP2	239.8900133	5.77E-07	0.463891023
DBF4	5.703359581	2.18E-06	2.054136126
DBF4B	3.256209566	8.86E-05	1.799268273
DBH	1.435466682	0.00015469	1.768591951
DBH-AS1	5.179128309	5.61E-07	2.104557436
DBI	152.1932255	0.004451124	1.542051591
DBN1	50.10307784	0.002262184	1.586696607
DBR1	22.1235297	3.50E-05	0.526797896
DBT	12.21779348	1.60E-07	0.426032439
DBX2	0.087581239	0.00044986	2.052633055
DCAF10	20.75480642	1.30E-05	0.488720774
DCAF11	45.58950057	1.90E-05	0.505404407
DCAF12	46.25971057	4.45E-05	0.527212011
DCAF4L1	0.323647548	5.21E-06	1.970508846
DCAF7	81.67061999	9.61E-05	0.540778034
DCBLD2	26.26161514	1.87E-05	1.997467793
DCD	0.144480364	3.43E-09	2.468179609
DCHS1	22.24949797	0.001643107	0.589202968
DCLRE1A	13.32377153	0.002417291	0.62217917
DCP1A	19.49414631	0.002721449	0.625507498
DCST1	0.398376658	1.04E-10	2.565370647
DCST1-AS1	2.104681087	2.92E-09	2.407631434
DCST2	1.083700791	1.60E-11	2.666640411
DCTN1	64.66072358	1.22E-05	0.496074223
DCTN3	46.59105968	1.13E-08	0.402354809
DCTN6	72.17773959	5.95E-09	0.400048292
DCTPP1	70.64191176	0.000667577	0.57891389
DDA1	29.10118469	0.003191771	1.564233841
DDAH1	122.054093	6.87E-12	0.319549395
DDB1	76.67010193	6.35E-06	0.484873324
DDC	90.7862753	2.17E-05	0.486257892
DDIAS	1.073727248	5.35E-05	1.841954211
DDIT3	90.94206863	6.72E-07	2.122490703
DDIT4-AS1	0	0.004606615	1.557019068
DDO	21.13815629	1.28E-05	0.481600961
DDX1	117.427568	1.22E-05	0.506113801
DDX11	9.392581527	1.60E-05	1.908439179
DDX11-AS1	0.910390796	7.18E-05	1.815518073
DDX12P	3.282768566	3.26E-05	1.862639656
DDX24	67.9298594	4.91E-05	0.520660419
DDX25	0.252392619	0.004962707	0.538474351
DDX39A	50.42228946	4.83E-06	1.989253409
DDX39B	50.13127333	0.001455958	1.610866815
DDX39BP2	0.690171344	0.004102202	1.574957464
DDX3P1	0.32043558	0.004834648	1.571920509
DDX3X	160.9486284	0.002902081	0.618560239
DDX4	0.060809727	0.000182879	1.785452255
DDX43P3	1.578583142	0.003638191	1.64612988
DDX46	26.32137408	0.000373609	0.568567802
DDX47	2.009590073	0.000390152	1.698778274
DDX50	65.9320809	0.000314977	0.573308573
DDX50P1	0.674714899	0.00079235	1.654292894
DDX55	13.34290135	0.001980363	1.596614205
DDX56	73.44262098	1.37E-08	2.332116058
DDX58	41.84176159	0.000318179	0.568108799
DDX59	18.97944884	0.000540355	0.581874876
DDX6	60.84046654	0.004105839	0.641966228
DDX60	31.55070148	0.001042472	0.590121723
DEF6	24.24670855	0.000851618	1.656150578
DEFA1B	0	0.004606615	1.557019068
DEFB108C	0.004861718	0.003571848	3.146883921
DEFB118	0.088653678	3.06E-05	1.946227275
DEFB123	0.036878808	0.002689916	1.798152835
DEFB124	0.29545659	0.002266685	1.602203445
DEFB130D	0.046130572	0.001451096	1.98462107
DEFB131C	0	0.004606615	1.557019068
DENND1A	17.46050611	0.00068499	0.575113696
DENND1C	44.49845472	0.000164682	0.552014026
DENND2C	2.177328672	0.003750211	0.625585423
DENND4B	28.81129305	0.001905067	1.595051067
DENND4C	31.22550238	3.80E-07	0.438439899
DENND5B	11.45716171	0.000100994	0.528008595
DENND6A	24.49031262	0.000474248	0.574328448
DEPDC1	2.064002173	2.30E-06	2.045327697
DEPDC1B	2.278823922	3.79E-05	1.859067664
DEPTOR	45.90605173	0.000754382	0.579141705
DERL3	11.34652498	0.000327515	1.755675866
DESI1	38.94175509	1.77E-06	2.051949706
DET1	10.4865866	0.000487292	0.577263171
DFFA	26.96761006	7.30E-06	0.490902338
DGAT1	31.42701339	0.001432999	1.616425968
DGAT2L7P	0.477291365	0.004745632	1.551544113
DGCR10	8.486899577	0.002206707	1.589445488
DGCR2	108.9505077	3.71E-05	0.524261977
DGKG	1.939557478	6.41E-05	1.826786559
DGKH	8.843940129	0.003320547	0.617431827
DGKK	0.138909645	0.000158571	0.506148651
DGLUCY	33.55010639	5.88E-05	0.509154352
DGUOK	89.22680128	5.08E-05	1.844032922
DGUOK-AS	2.809563702	7.88E-09	2.342088031
DHCR24-DT	0.875928961	0.00058713	1.681501548
DHDDS	31.41173726	0.001953324	0.613329438
DHDH	19.06493806	0.004047021	0.58396551
DHFR2	11.17663055	6.97E-07	0.44854607
DHH	1.098726248	0.000666957	0.553962864
DHRS12	23.63103778	0.000124907	0.548894996
DHRS4-AS1	29.88753631	0.001365981	0.597312537
DHRS4L1	1.78336198	0.002326395	0.603167263
DHTKD1	51.73525804	0.001425952	0.597285239
DHX15	52.92712965	1.59E-05	0.506246591
DHX29	34.60686081	0.000484874	0.574510804
DHX34	14.50707221	0.000708223	1.66453686
DHX40	53.94060999	8.69E-06	0.501342304
DHX58	20.45340218	2.09E-05	1.893855114
DHX9	116.1669771	1.39E-05	0.510037404
DHX9P1	0.061659907	0.001412998	0.586881807
DIAPH1	163.9129552	7.19E-07	0.456439427
DIAPH2-AS	0.281922347	1.33E-05	1.945934679
DICER1-AS	5.305352604	0.001404408	1.621353331
DIMT1	33.62153481	0.000980312	1.667794299
DIP2B	22.77020464	0.000943891	0.588632895
DIP2C	33.21414265	2.32E-07	0.413960717
DIRAS2	31.67526016	0.00068838	0.574344462
DIRC1	0.025904796	0.001316652	1.748291353
DIS3	18.36874408	0.003335857	0.635750402
DIS3L	27.07734062	0.00305073	0.628975531
DISP1	16.97377942	2.45E-06	0.466019772
DIXDC1	31.62649219	1.65E-05	0.481302739
DKKL1	0.565794485	8.37E-05	1.812795851
DLAT	45.74277761	5.84E-06	0.479219074
DLC1	32.19686523	0.000544305	0.572140011
DLEC1	0.402720379	0.00104417	1.647625861
DLEU2	2.943050348	0.00036637	1.704848456
DLG5-AS1	2.223893277	0.000339321	1.722877533
DLGAP1-AS	2.882764686	7.18E-13	2.850280594
DLGAP1-AS	0.29594874	0.000367647	1.815210592
DLGAP1-AS	0.085649099	0.000329704	2.027119614
DLGAP4	47.24116047	0.00387638	1.549328195
DLGAP5	4.164684753	1.77E-07	2.195573128
DLK2	4.322232447	1.33E-05	1.952790178
DLST	105.4992676	2.29E-06	0.461276535
DLX4	1.238062403	1.15E-10	2.579574481
DM1-AS	3.598886609	0.000209788	1.743089812
DMBX1	0.299809716	0.000609928	2.171350463
DMGDH	67.3305654	1.73E-09	0.359699503
DMRT3	0.299249177	2.34E-07	2.275207138
DMRTA1	7.441988598	2.33E-06	0.464108278
DMTN	81.95277891	0.000203583	0.554061285
DMXL1	23.65088771	1.57E-07	0.414522248
DNA2	3.507773555	2.33E-07	2.154772251
DNAAF2	13.45854465	5.90E-05	0.517681433
DNAAF3	1.377477146	4.68E-07	2.123293567
DNAH1	4.740644081	0.000888255	1.646544844
DNAH14	1.000478367	0.000126061	1.779844595
DNAH17	0.961735998	1.47E-06	2.053236078

DNAH17-AS	0.091073492	6.95E-06	2.014023198
DNAH3	0.134478764	0.001019791	1.688446869
DNAJA1	242.5052703	1.94E-05	0.507202389
DNAJA2	74.02126881	0.002351207	0.619002539
DNAJB11	41.09185482	0.00014151	1.785402956
DNAJB14	17.17210869	3.93E-07	0.435087046
DNAJB3	0.389757966	0.002986202	1.594721859
DNAJB4	45.65745742	0.000133817	0.532172834
DNAJB5P1	0.047212863	1.57E-06	2.141406338
DNAJB7	0.217116711	0.003660691	1.554145974
DNAJB9	158.7271836	0.001312837	0.59677344
DNAJC1	91.36869376	6.90E-05	0.512758136
DNAJC11	27.83464412	0.000519419	0.575194588
DNAJC12	7.695629619	5.69E-05	1.915849495
DNAJC13	33.33977451	0.003056557	0.617527959
DNAJC16	19.15070841	6.63E-06	0.48918822
DNAJC18	11.25940236	0.000171414	0.539290157
DNAJC19P2	0	0.004606615	1.557019068
DNAJC19P4	0.008859661	0.001764736	2.557045741
DNAJC19P5	8.045532203	0.000118238	1.782476458
DNAJC19P7	0.138317499	0.001911231	1.627400702
DNAJC2	15.37240447	0.003454735	1.556892417
DNAJC22	47.6633014	0.000151043	0.543246233
DNAJC27	7.419398709	0.001116714	0.593152366
DNAJC28	3.004002482	0.003947922	0.630549296
DNAJC3	99.98236735	0.003694891	0.6371742
DNAL1	11.42332909	2.70E-06	0.459174885
DNASE1	5.237034772	0.001041703	1.735442122
DNASE1L2	1.200871167	0.000505842	1.699491182
DNASE1L3	10.09882185	0.000487692	0.519160963
DNASE2B	1.191564069	0.004151968	0.58145413
DND1	0	0.004606615	1.557019068
DNHD1	2.813006668	1.27E-05	1.914070122
DNLZ	0.236200807	0.002264959	1.59310729
DNM1P24	0	0.004606615	1.557019068
DNM1P48	0	0.004606615	1.557019068
DNM1P51	0.125201905	0.000198215	1.776460042
DNMBP	17.19155312	0.001579806	0.598379887
DNMT3B	4.427668023	1.04E-06	2.071313786
DNMT3L	0.099471834	2.51E-06	2.089013653
DNTTIP1	55.92005364	2.53E-09	2.420233447
DNTTIP2	30.87821948	0.001101087	0.603068518
DOC2GP	1.150582573	1.08E-08	2.324038178
DOCK1	47.55092149	0.000236555	0.552960281
DOCK4	19.84011319	0.000607836	0.569979047
DOCK7	14.31026719	7.21E-05	0.529598794
DOCK8	32.5728298	2.37E-08	0.402313774
DOCK9	31.41942017	0.00015688	0.529330173
DOK3	13.14956546	3.73E-05	1.854590387
DOK7	0.959112265	0.003816754	1.601480865
DONSON	11.17907642	2.39E-12	2.807224898
DPF1	0.249780467	5.34E-09	2.375647115
DPH3P1	0	0.004606615	1.557019068
DPH7	12.73581944	7.10E-05	1.807601358
DPP4	185.4701422	4.06E-07	0.421340382
DPP9-AS1	1.052323669	1.68E-06	2.035893671
DPPA2P2	0.089407645	0.001278638	1.710790686
DPPA2P4	0.109270546	4.31E-05	1.907601504
DPPA3	0.069498872	7.06E-05	1.915486525
DPPA4	0.101857068	0.00065793	1.740998896
DPRX	0.092608104	0.001717972	1.653818879
DPRXP2	0.21190903	2.48E-07	2.160250264
DPRXP4	2.010800726	0.000239781	1.731152542
DPRXP6	0.200552771	0.000111914	1.79538793
DPY19L1P1	5.195388261	4.06E-08	2.243518679
DPY19L2P1	0.618891978	0.000197505	1.78731787
DPY19L4P2	0.049536582	0.000583766	1.770577807
DPYSL2	135.9181739	0.001402005	0.608839001
DPYSL3	57.59620647	0.002468194	1.59370931
DQX1	0.351062907	1.40E-09	2.810358954
DRAP1	136.187153	1.22E-05	1.933089148
DRAXINP1	0.112005525	0.000343564	1.802813448
DRC1	0.227216278	0.001164528	1.651682725
DRC7	0.828986582	0.000455001	1.730035702
DRD4	2.804846577	0.000436325	1.707004808
DRD5	0.078318632	2.32E-05	0.44630601
DRD5P1	0.014963596	0.001292136	1.801052928
DRGX	0.050864568	8.00E-06	2.062603227
DRP2	0.132915058	1.19E-08	2.458308149
DSCAM-AS	0.280211278	0.000827308	1.852983139
DSCR4	0.069175722	0.002577666	1.987239484
DSCR9	0.333310344	5.39E-10	2.479756667
DSEL	9.247292959	0.00073342	0.559016239
DSG3	0.065138209	0.0043364	1.650607621
DSP	43.60829574	0.002134326	0.598438231
DSTN	379.2007114	0.000324426	0.558435866
DSTNP2	24.36203074	0.00280635	1.572043623
DSTYK	17.46967627	3.22E-05	0.507832737
DTWD2	11.11121147	5.25E-06	0.48279855
DTX2	12.09324476	3.79E-07	2.153093771
DTX2P1-UP	1.166031629	0.00144921	1.617662958
DTYMK	29.74044067	0.000153165	1.770820863
DUBR	5.230869358	0.000195302	0.551474225
DUS1L	39.63503497	2.68E-08	2.309936497
DUSP11	29.89322019	0.000476659	0.577804796
DUSP12P1	0.040147218	0.002354613	1.782475077
DUSP13	0.09224489	4.20E-05	2.013107814
DUSP14	38.56004079	0.004419671	1.540595531
DUSP16	34.56867686	0.000537856	0.580847049
DUSP23	290.2790072	0.000128745	1.781270928
DUSP3	127.2254638	0.000182185	0.554907298
DUSP5P1	2.408263065	1.72E-08	2.307364085
DUSP8P3	0.467944237	3.83E-06	1.992036232
DUSP8P4	0.075692476	4.81E-05	1.839040531
DUSP9	1.577010313	0.001296715	1.716276889
DUTP6	3.532559625	0.000663383	1.670195456
DUX4L1	0	0.004606615	1.557019068
DUX4L10	0	0.004606615	1.557019068
DUX4L11	0	0.004606615	1.557019068
DUX4L12	0	0.004606615	1.557019068
DUX4L13	0	0.004606615	1.557019068
DUX4L14	0	0.004606615	1.557019068
DUX4L15	0	0.004606615	1.557019068
DUX4L2	0	0.004606615	1.557019068
DUX4L20	0	0.004606615	1.557019068
DUX4L21	0	0.004606615	1.557019068
DUX4L22	0	0.004606615	1.557019068
DUX4L23	0	0.004606615	1.557019068
DUX4L24	0	0.004606615	1.557019068
DUX4L25	0	0.004606615	1.557019068
DUX4L29	0	0.004606615	1.557019068
DUX4L3	0	0.004606615	1.557019068
DUX4L4	0	0.004606615	1.557019068
DUX4L5	0	0.004606615	1.557019068
DUX4L50	4.634779474	0.001790868	1.606821856
DUX4L51	0	0.004606615	1.557019068
DUX4L6	0	0.004606615	1.557019068
DUX4L7	0	0.004606615	1.557019068
DUX4L9	0.066632688	0.000142761	1.866903247
DUXAP3	0.045815544	1.20E-06	2.219553832
DUXAP8	1.065984378	3.21E-10	2.52449226
DUXAP9	0.428006726	2.92E-05	1.877583279
DVL3	59.46474286	4.22E-05	1.876410146
DXO	25.98054908	0.003077789	1.561147143
DYM	28.14459826	6.03E-06	0.491077933
DYNAP	0.017823852	0.004978887	1.664907467
DYNC1H1	90.16150317	7.15E-06	0.469873129
DYNC1I2	87.66074104	9.94E-07	0.46565547
DYNC1I2P1	1.197967161	0.000960842	0.586516005
DYNC1LI2	52.43878218	0.001488851	0.609567087
DYNC2H1	8.957951461	6.18E-07	0.431596034
DYNLL1P4	0.633188836	0.000267181	1.729226822
DYNLL1P6	0.070669616	3.55E-05	2.218172636
DYNLL2	78.39440965	1.16E-07	0.415544023
DYRK1A	30.0921441	0.000431901	0.570755435
DYRK3	11.1094214	0.000175709	0.541444274
DYRK4	9.814141783	0.000156937	1.77568639
DYSF	84.36817487	0.001146614	0.587916813
E2F1	11.51146228	0.004269833	1.544141143
E2F2	1.701428822	0.00016199	1.765023105
E2F5	5.864917486	0.00020162	1.750702169
E2F7	1.427956393	5.31E-07	2.134948446
E4F1	14.50700132	0.003088327	1.565390984
EAF1-AS1	0.223349754	0.001083957	1.634076012
EAPP	73.89261652	0.000538994	0.578652791
EARS2	24.74316874	0.000176011	0.551892583
EBAG9	40.74539558	0.000163585	0.558747444
EBI3	20.50026027	0.001603794	1.613014862
EBLN3P	49.01437189	0.001226357	0.598132351
ECE2	2.795909976	0.000972049	1.654236769

ECEL1P2	0.01436864	0.001201071	1.785426701
ECHDC1	35.94486109	0.000344583	0.568066437
ECHDC3	88.90688383	2.24E-05	0.510524933
ECI2	62.62093817	4.14E-05	0.522679011
ECPAS	56.92995646	8.01E-06	0.496505195
EDA	6.887516554	2.30E-07	0.41133092
EDAR	1.10006666	3.08E-05	0.428509247
EDEM2	96.2155649	0.00315024	1.564159631
EDNRB	166.4274729	1.19E-05	0.473703981
EEF1A1	4789.026082	0.000218268	0.559126122
EEF1A1P10	1.498115962	6.31E-06	0.45353788
EEF1A1P13	17.2963326	0.001634799	0.564965697
EEF1A1P15	0.109066476	0.000234353	0.511632883
EEF1A1P16	1.327847343	0.001358557	0.572919028
EEF1A1P22	1.663248242	0.00098119	0.559470929
EEF1A1P25	1.155239856	0.001746221	0.582606143
EEF1A1P3	0.860034501	0.000293346	0.526099214
EEF1A1P4	2.25033599	5.05E-05	0.479361839
EEF1A1P6	47.5868555	0.002447947	0.556985566
EEF1A1P7	1.445415381	0.000182605	0.532815297
EEF1B2P7	0.354161002	0.002164782	1.588444668
EEF1DP2	0	0.004606615	1.557019068
EEF1DP8	0.044603731	0.003492584	0.576077889
EEF2	2760.254658	0.000626514	0.579694934
EFCAB10	1.091879229	7.77E-06	1.954959264
EFCAB11	3.3844766	2.62E-06	0.460837486
EFCAB13	2.488949631	0.000486914	1.688885697
EFCAB14	127.3297121	4.86E-06	0.482776589
EFCAB6-AS	0.165582524	0.000104702	1.80362918
EFCAB8	0.158823336	6.85E-07	2.105228284
EFHD1	67.46704861	0.003771603	0.619546078
EFHD2	123.7700493	0.004272781	1.541891894
EFL1	21.97174142	0.000547882	0.586099778
EFNA4	17.23309774	9.57E-05	1.803545629
EFNA5	23.80079966	1.24E-06	2.064751036
EFNB2	107.3722401	0.000118735	0.541150242
EGFEM1P	0.115123063	0.000959881	1.688401452
EGFL8	3.325089996	0.001839747	1.599453522
EGLN1	84.56729847	0.000183384	0.557714429
EGLN1P1	0	0.004606615	1.557019068
EGLN2	11.51364252	0.000308904	1.715264345
EGLN3-AS1	1.169104702	0.002185297	0.570594564
EGOT	21.34167761	0.000977187	0.582100741
EGR1	690.4796984	0.004904051	0.590571346
EHBP1	36.83898277	1.22E-06	0.443779776
EHD3	32.56403283	0.000968426	0.578521225
EHD4	62.46760073	0.00028399	0.546585779
EHHADH	129.7070595	5.59E-08	0.408841253
EID1	410.5679475	7.66E-07	0.467277193
EIF1B	71.54614291	0.000202144	0.52024812
EIF2A	120.5117674	0.000143076	0.551712041
EIF2B3	20.68116936	0.002162939	0.616010393
EIF2S1	47.11007912	0.000459789	0.579177654
EIF3A	121.7221009	2.50E-06	0.479839327
EIF3C	5.34609837	0.003628418	0.632417452
EIF3EP2	0.055189049	2.98E-06	2.104337234
EIF3KP3	0.174690401	6.00E-05	1.914060417
EIF3L	164.9028172	5.93E-07	0.45754155
EIF4A1	8.262288955	4.70E-05	1.837197377
EIF4A1P1	0.059000509	0.003418509	1.570803213
EIF4A1P12	0.080905078	0.001070278	1.955432208
EIF4A2P3	0.172202133	0.000101899	0.508310382
EIF4B	376.0045382	1.43E-05	0.497166249
EIF4BP6	9.062642244	6.34E-05	0.522541505
EIF4E3	17.89685044	4.17E-05	0.517773066
EIF4EBP1	173.006502	9.70E-09	2.342524293
EIF4EBP2	160.287586	3.42E-07	0.433110903
EIF4EP2	1.086742582	0.000559085	0.568045197
EIF4G2	371.0882046	0.001117058	0.605106175
EIF4H	336.8753303	1.18E-05	0.512043363
EIF4HP1	3.225009726	1.28E-07	0.419969487
EIF4HP2	2.628941235	5.69E-05	1.828714365
EIF5	78.4438495	1.98E-06	0.447968118
EIF5A	423.0560133	1.71E-06	2.059373432
EIF5AP2	0.363559121	0.003441311	1.56945033
EIPR1-IT1	0.261803754	0.00022549	1.758480161
ELDR	0.5511865	0.000756038	1.694168893
ELF1	70.13841916	1.00E-05	0.49984548
ELF3-AS1	2.141898356	3.86E-07	2.138847454
ELFN1-AS1	0.43537488	3.19E-05	2.189736695
ELK3	86.51985802	0.002164837	0.604007304
ELK4	25.77208109	0.004229548	0.633830181
ELL2P1	0.561993645	0.000140019	0.536641822
ELL2P2	0.035392821	0.000427666	1.857942874
ELMO2	26.26801025	0.002261103	1.587154865
ELMOD3	9.676355714	0.000207782	1.744763765
ELMSAN1	22.43158631	0.000269558	0.550954031
ELOCP10	0	0.004606615	1.557019068
ELOCP11	0	0.004606615	1.557019068
ELOCP15	0	0.004606615	1.557019068
ELOCP16	0	0.004606615	1.557019068
ELOCP17	0	0.004606615	1.557019068
ELOCP34	0	0.004606615	1.557019068
ELOCP35	0	0.004606615	1.557019068
ELOCP6	0	0.004606615	1.557019068
ELOCP7	0.000470878	3.42E-07	28.06725687
ELOCP8	0	0.004606615	1.557019068
ELOCP9	0	0.004606615	1.557019068
ELOVL7	69.78347331	0.001201268	0.595977575
ELP1	28.80036379	0.001075667	0.599020617
ELP3	31.79859561	0.000503156	0.570294383
ELP4	32.58549096	9.88E-06	0.501843721
EMC3-AS1	1.479069284	1.06E-07	2.186077862
EMC9	30.12819345	4.21E-05	1.850154071
EMCN	63.0300762	2.98E-08	0.390387029
EME1	2.130108388	5.50E-10	2.486125782
EME2	8.739462109	0.000136842	1.771742636
EMG1	12.62462445	4.23E-06	1.995187579
EML1	17.70746819	0.001797036	0.608493365
EML2	14.65747991	0.001236288	1.627537194
EML4	50.08928436	0.003229728	0.628785424
EMP3	165.5489112	0.001182481	1.633417197
EMX2	65.02335347	3.71E-06	0.47711239
EMX2OS	40.64787168	1.05E-05	0.490486891
EN2	0.641921291	6.77E-11	2.83096507
ENAM	5.414305233	0.000412679	0.525269657
ENDOD1	89.80363279	0.000246756	0.553658836
ENGASE	16.40775054	3.90E-06	1.982465524
ENKUR	2.318169534	0.002171493	1.597823429
ENO1	2968.650642	0.004665163	0.644632645
ENO1P1	0	0.004606615	1.557019068
ENO1P4	0	0.004606615	1.557019068
ENO2	204.9484151	0.003557281	1.55488263
ENO3	5.793494795	4.25E-07	2.195196795
ENOX1-AS1	0.032902278	1.52E-05	1.949914146
ENPEP	254.7628925	9.84E-06	0.480787018
ENPP3	337.1165934	0.000454235	0.573362259
ENPP4	42.44564475	0.000121275	0.539568581
ENPP5	29.18401163	4.65E-08	0.37409948
ENPP7P1	0.079687064	0.00142206	1.625194763
ENPP7P11	0.66778076	0.000942147	1.655046967
ENPP7P8	5.271602127	0.002369081	0.589807182
ENPP7P9	0.091325185	0.004991331	1.554897628
ENSAP2	0.751695039	0.003536056	0.622968398
ENTHD1	0.141088273	0.00017377	1.816519189
ENTPD5	28.22911653	0.000728304	0.551139705
ENTPD6	48.54587776	1.52E-05	1.91457062
EP300	51.53655737	6.83E-05	0.525357522
EP400P1	2.958600249	6.87E-06	1.960158441
EPAS1	535.2215762	4.77E-07	0.426212629
EPB41L1	68.65592783	5.41E-06	0.484463814
EPB41L2	37.76294656	0.002111912	0.607890104
EPB41L4A	19.99727753	2.88E-06	0.466747503
EPB41L4A-[illegible]	10.5526276	2.13E-08	0.394722562
EPB41L5	14.73249198	2.50E-06	0.458782577
EPC2	24.46493528	4.56E-05	0.522278855
EPG5	12.96645245	0.00166755	0.595292263
EPHA10	0.803793713	0.003451136	1.605211581
EPHA4	12.27266928	1.66E-06	0.443223848
EPHA5-AS1	0.142055102	0.000808824	2.976212177
EPHA8	0.06824107	4.04E-06	2.137412122
EPHB4	42.31694464	0.001613985	0.601847912
EPHX2	64.06440253	1.80E-05	0.497604056
EPM2A	5.798108361	4.59E-05	0.521044479
EPOP	4.059533429	4.49E-06	1.987244135
EPPIN	0.024870787	1.49E-07	2.348561111
EPS15	48.21665498	0.003639768	0.635172989
EPS8	117.5344187	0.000385889	0.56392378
EPS8L3	6.34779152	0.004531424	1.595396778
ERBB2	55.71070376	3.93E-05	0.503986251

ERBB3	111.2974407	0.001448724	0.605374507
ERBIN	46.83779747	0.002274042	0.617641939
ERC1	22.4364649	0.000490083	0.572366856
ERC2	0.255756862	0.00248944	1.61238811
ERCC1	38.73699211	0.001404787	1.624315604
ERCC4	5.791976557	0.000543071	0.576403138
ERCC6L	1.005773796	1.24E-08	2.332298644
ERCC6L2	6.369587433	0.003042239	0.615715018
EREG	0.833977609	5.87E-05	2.353435508
ERHP1	0.312327372	0.004117787	0.625537704
ERICH5	22.5742896	6.75E-06	0.478179953
ERICH6B	0.452054185	5.53E-05	1.821847066
ERLEC1P1	0	0.004606615	1.557019068
ERLIN2	43.9411357	1.57E-06	0.45550811
ERLNC1	0.424027553	5.97E-05	0.441371338
ERMN	0.618878393	0.002339922	1.592567224
ERMP1	34.36469866	1.22E-06	0.414763734
ERN2	0.123082224	1.41E-07	2.272594667
ERO1B	22.29439336	0.000197974	0.548895138
ERP44	66.82246674	0.004964519	0.644902905
ERVW-1	0.264021979	0.001185122	0.546823628
ESD	154.5182931	3.46E-05	0.530484439
ESR2	0.568929229	0.000164165	1.809897308
ESRRG	5.00203119	0.002264517	0.577488871
ESYT1	129.192433	4.52E-06	0.485163219
ESYT2	120.7767751	0.003821962	0.630647595
ETFA	69.10596472	0.000635531	0.581071665
ETFBKMT	4.332616224	0.001181863	0.600902869
ETFDH	34.52409832	0.001245444	0.599874131
ETNK2	28.23411996	0.000230846	0.528921472
ETS1	199.1397205	0.000176493	0.547966191
ETV3	19.39214827	0.00010599	0.529114977
ETV6	26.34922437	4.21E-05	1.853851419
EVC	43.30788361	3.17E-06	0.447439541
EVL	34.39141523	0.000529299	1.679388173
EVPL	8.978769936	0.004032228	1.545591396
EXD1	0.040716106	0.001344233	1.661323877
EXD2	20.5751933	0.00037908	0.575660863
EXO1	2.172123039	0.003442516	1.567531203
EXO5	7.594490864	0.004982532	0.649166107
EXOC4	37.21794081	2.89E-06	0.477753525
EXOC5	21.47550657	0.002576756	0.619198322
EXOC6B	14.25193486	1.25E-05	0.487556255
EXOC7	36.08186001	0.000427112	0.579965682
EXOC8	18.37089797	2.56E-05	0.509171952
EXOSC1	19.03478456	6.93E-11	2.718832453
EXOSC10-A	1.314342975	9.94E-05	1.797464176
EXOSC3P2	0.126837352	0.001651219	1.641641158
EXTL2P1	0.01170236	0.000343718	2.538834812
EXTL3	16.64546494	6.58E-05	0.517737787
EXTL3-AS1	0.390041453	9.76E-06	1.931150777
EYA1	1.820695249	2.86E-07	2.309820226
EYA3	20.99739063	0.000378461	0.561961544
EZH2	7.85647099	0.00085101	1.655396741
EZR	898.7496804	3.37E-08	0.399722258
F2	10.53944655	0.000735895	1.813204053
F2RL1	77.11786119	3.44E-07	0.434718436
F2RL3	42.82727936	3.47E-06	0.410504965
F3	16.70531387	0.00081137	1.765489283
F7	0.437045221	0.000526449	1.942625132
FAAH	47.55063563	0.004491325	0.636807794
FAAP100	30.89356738	2.49E-06	2.044029462
FAAP24	4.348386177	1.40E-07	2.191527523
FABP12	0.042081942	0.001391198	1.704973837
FABP5	21.71985889	0.002094046	1.601432219
FABP5P3	0.138351716	0.003878711	1.547961055
FADS2P1	0.022552748	0.000583423	1.846375637
FADS3	22.70253471	1.40E-06	2.063871686
FAF2	62.95935125	0.001483408	0.611944265
FAHD1	103.1306642	5.05E-05	0.529497895
FAHD2A	9.811707176	1.56E-06	2.043962905
FAHD2CP	4.575708945	7.55E-09	2.346387492
FALEC	0.757255983	5.50E-06	1.970610084
FAM110C	68.22314089	0.003426711	0.598390845
FAM111A	29.24768131	0.004052134	1.540884384
FAM114A2	20.22597598	0.00105989	0.597614322
FAM117B	14.26371499	0.001846738	0.604222222
FAM120A	116.8893099	1.64E-06	0.46093229
FAM120B	28.22213326	0.000583548	0.583943177
FAM122A	28.99518592	1.06E-06	0.460913853
FAM133A	0.421238407	1.00E-06	3.567785858
FAM13A-AS	5.160100848	0.000616984	1.669873719
FAM13B	23.85517953	4.70E-07	0.427946346
FAM156A	0.15113277	8.30E-05	1.804633411
FAM160A1	4.177204861	1.42E-06	0.423341664
FAM160B2	21.26093341	0.000172694	1.757930678
FAM161B	7.897570673	0.000133956	0.535658214
FAM167A	2.636446804	0.000789511	1.729437676
FAM168A	48.30536046	0.00086238	0.589103722
FAM168B	102.4433966	1.90E-05	0.505025075
FAM171A1	33.33234919	0.004050352	0.623120785
FAM172A	33.67416134	0.000112339	0.538680923
FAM174A	85.22273662	0.000970444	0.593920506
FAM177A1	25.38972951	0.002875871	0.627603657
FAM183BP	0.235490851	0.000369747	1.723251503
FAM184B	0.446302145	0.003741619	1.749616871
FAM186A	0.344317966	0.000341005	1.710220126
FAM187A	0	0.004606615	1.557019068
FAM187B2I	0.810883839	1.70E-05	0.484321927
FAM193B	37.13017305	0.000112412	1.781328773
FAM197Y1	0.00057549	0.001566065	6.900943497
FAM197Y4	0.00315268	0.001359283	3.479128189
FAM197Y8	0.037535627	0.00443889	2.448262323
FAM19A3	0.473794427	2.19E-05	1.915611818
FAM204A	11.35840784	0.001416392	0.610715307
FAM204DP	0.031306111	0.001608225	1.77830158
FAM209A	0.418892035	1.30E-05	1.914078224
FAM209B	0.894936263	5.39E-05	1.831722503
FAM20A	12.82414974	8.66E-08	2.244658372
FAM210A	24.13082203	1.04E-06	0.451910864
FAM210B	77.13040663	0.003496879	0.625518506
FAM216B	0.015330005	0.000122972	1.808452697
FAM218A	2.329886142	0.000178436	1.759583482
FAM219B	15.43874567	2.10E-05	0.511202877
FAM225A	0.370846746	6.50E-08	2.229402092
FAM225B	0.213838641	5.89E-05	1.832905841
FAM227A	0.971717964	1.10E-05	1.925752789
FAM227B	3.716316699	0.001282822	0.601852662
FAM229A	4.222894435	0.00048403	1.688859783
FAM230G	0.009823601	6.07E-07	2.324283823
FAM234A	55.14062645	0.001965896	1.599394948
FAM236A	0	0.004606615	1.557019068
FAM236B	0	0.004606615	1.557019068
FAM238B	0.062002429	4.37E-05	1.873359796
FAM242C	0.300783906	0.003951394	1.548521451
FAM25A	0.176328298	7.17E-07	2.187944424
FAM27E3	1.052361013	0.00103671	1.647827826
FAM30A	0.588889867	0.001006332	1.802382024
FAM30B	0.00261785	0.00045107	3.858236936
FAM47E-ST	0.108727646	4.45E-06	0.452513568
FAM50A	109.7949091	0.002258898	1.588311825
FAM50B	36.19800046	0.00011964	0.545198661
FAM53A	1.067651106	3.52E-05	1.863295557
FAM53B	35.23842337	1.29E-05	0.492014817
FAM53C	25.35125311	0.002409761	0.610576067
FAM71D	0.144073765	1.11E-05	1.940006147
FAM71F1	0.133152612	0.000134127	1.800220899
FAM71F2	1.404714152	0.000146718	1.76947496
FAM72A	0.473684075	0.000589912	1.679844093
FAM72B	0.490395979	2.79E-06	2.020797081
FAM72C	0.21186683	2.40E-08	2.284405734
FAM72D	0.301462929	4.48E-06	1.988205418
FAM78B	3.312016041	1.24E-05	1.940642894
FAM83A	0.190291664	3.25E-06	2.349125519
FAM83A-AS	0.11805577	0.000963119	1.743305499
FAM83C	0.031627304	0.000107921	1.850671154
FAM83E	0.392526853	6.08E-05	2.039615837
FAM84B	61.91515898	0.000126851	0.539515148
FAM85B	0.925417223	0.001544526	0.583411679
FAM86GP	3.053507092	0.000194272	1.752556452
FAM8A1	57.68264633	1.82E-07	0.441357422
FAM90A1	2.068117439	0.000597466	1.680446091
FAM90A13I	0	0.004606615	1.557019068
FAM90A15I	0	0.004606615	1.557019068
FAM90A21I	0	0.004606615	1.557019068
FAM90A26	0.029475493	0.000366681	1.717119507
FAM90A3P	0	0.004606615	1.557019068
FAM90A6P	0	0.004606615	1.557019068
FAM92B	0.321556237	4.06E-06	2.109706403
FAM98B	21.77041076	5.50E-05	0.531484676

FAM9A	0.116316156	0.00058315	1.769669698
FAM9B	0.112751106	0.003431243	1.676903424
FANCA	2.736547099	0.000594365	1.680244156
FANCC	12.54230055	8.57E-06	0.485427876
FANCD2	2.942026538	9.39E-05	1.801295745
FANCD2OS	0.053235182	5.05E-05	1.843757675
FARP1	19.85643774	9.47E-06	0.486747982
FARS2	31.15820252	8.55E-09	0.412110139
FASN	25.51137139	5.91E-05	1.83425774
FASTK	66.11703729	1.42E-07	2.186427976
FASTKD2	22.79771297	1.30E-06	0.462749971
FASTKD3	27.15171871	0.000154174	0.550861438
FASTKD5	27.01001309	1.97E-05	0.500182425
FAT2	0.455940884	0.000571115	1.770145326
FAT4	10.19678252	7.99E-05	0.517632709
FAXDC2	25.54455027	0.000426338	0.555994116
FBF1	2.071693269	0	3.43309656
FBP1	110.4206634	4.75E-05	0.49308203
FBXL14	23.65695651	0.002856098	0.629586293
FBXL16	56.82101664	0.0007354	0.585120026
FBXL17	21.9073425	9.26E-06	0.472373109
FBXL19	16.96337715	1.29E-05	1.939911108
FBXL3	44.20738456	3.84E-07	0.445675656
FBXL4	18.04078463	0.000199219	0.551964265
FBXL5	287.0080423	2.91E-09	0.385879699
FBXL6	15.21040496	2.22E-09	2.436921317
FBXO21	46.09939567	2.10E-05	0.456254911
FBXO24	0.70708444	0.00180571	1.604658444
FBXO28	37.947577	0.000195438	0.559096478
FBXO3	20.93674722	1.13E-07	0.431524962
FBXO30	9.856779115	0.003501447	0.621525938
FBXO34	28.79173271	1.81E-08	0.38807595
FBXO38	21.96397705	1.26E-05	0.493280656
FBXO43	0.603194969	2.84E-11	2.650946231
FBXO46	16.3036593	0.00311371	1.563687949
FBXO48	4.615437144	0.000196493	0.54740002
FBXO7	61.28416326	1.10E-05	0.501774851
FBXO8	36.2055514	4.00E-06	0.480548612
FBXW10	0.094340263	0.00022168	1.762690996
FBXW11	52.47686619	8.73E-06	0.493599992
FBXW11P1	0.601578531	0.004809066	1.532300832
FBXW2	26.25850926	1.32E-05	0.500491716
FBXW4P1	0.735898547	1.64E-05	1.909048816
FCER1A	16.40906601	0.000877911	0.530747052
FCF1	30.44046224	8.05E-05	0.538471885
FCF1P2	22.76084717	0.002011219	0.616204289
FCF1P7	0.748196945	4.07E-06	1.991243623
FCGR1A	12.64970602	0.002902272	1.571404512
FCGR1B	2.111996693	0.001007615	1.644687248
FCGR1CP	3.273767306	0.002146092	1.593473357
FCGRT	224.9703123	5.34E-05	0.537589617
FCHO1	4.665716513	9.27E-07	2.079103333
FCHO2	53.18317472	1.71E-08	0.396528882
FCHSD1	12.36233021	0.001129445	1.633969225
FCRL5	0.917148856	0.000372431	1.796024535
FDFT1	48.39969372	2.95E-07	0.439090345
FDPS	71.85205	0.003041303	0.633482536
FDX2	5.934978824	0.002261347	1.58923451
FECH	27.93600902	1.25E-05	0.496726285
FEM1B	43.34318709	0.00059372	0.578485427
FER	8.683219799	7.45E-06	0.472607451
FER1L4	11.81794605	0.000750302	1.666956246
FERP1	0.877767804	0.003276132	1.557174243
FEZ2	23.32265345	0.003245462	0.636098227
FEZF1	0.055614793	0.000111792	2.074332347
FEZF1-AS1	0.131381027	8.20E-05	1.974396826
FGD5	43.34633179	5.33E-05	0.507421634
FGF12	2.952493793	0.000198067	0.514115247
FGF17	0.354597411	0.000106874	1.812074765
FGF19	0.042719398	0.002383044	1.704113613
FGF21	0.129555725	8.56E-05	1.968708092
FGF23	0.059646448	2.47E-08	2.350688304
FGF7P3	13.16661414	6.66E-05	0.521926729
FGF8	0.111223783	0.000227042	1.767286666
FGGY	17.33407992	0.000233966	0.54058184
FHAD1	0.544951207	0.001391645	1.640226372
FHL5	30.6120156	6.62E-05	0.506988139
FILIP1	11.19166798	0.002408249	0.598479511
FIRRE	0.68284327	0	3.409817533
FITM2	24.1234153	3.51E-06	0.456445062
FKBP10	217.5758109	1.34E-08	2.327712015
FKBP11	21.60062475	3.49E-13	2.891965195
FKBP6	0.056950683	0.000454579	1.728287116
FKBP9P1	8.262698288	1.79E-05	2.222996029
FKTN	14.56040257	0.000134263	0.545146864
FLAD1	25.61737035	2.68E-06	2.02688714
FLJ45513	1.564726358	0.00059489	1.67499315
FLNB	105.5855523	0.002638871	0.579366198
FLNC	10.89685864	0.000488095	1.775295212
FLNC-AS1	0.104058165	0.001724689	1.618374363
FLRT3	31.25964749	7.78E-06	0.446854211
FLT1	197.4420502	0.000164591	0.548110564
FLT3LG	3.568530411	0.000282417	1.731388042
FMNL1	20.93325159	0.002386604	1.58369619
FMO1	77.74821266	1.01E-05	0.473363687
FMO2	33.94076312	0.000987257	0.590294743
FMO4	22.62155753	0.004173484	0.625608181
FMO7P	0.13651684	0.002674061	1.600437043
FMR1-IT1	3.24191065	0.000227986	1.734506775
FMR1NB	0.20207857	1.03E-10	3.329109563
FN3KRP	63.87998649	0.00213905	0.619749523
FNBP1L	74.04524598	9.81E-07	0.45484902
FNBP4	31.24451208	0.001008391	1.638522318
FNDC10	5.568974203	5.40E-05	1.879741311
FNDC3A	56.21260504	1.26E-05	0.49322831
FNDC4	11.58671296	0.000891016	1.709723902
FNDC7	0.039866485	3.29E-05	1.872281899
FNIP1	24.3909543	0.004964778	0.641163136
FNIP2	35.88492883	8.29E-05	0.485835943
FO393401.1	1.672381394	4.92E-05	1.841605217
FO393418.1	0.406856007	0.000580807	1.68133237
FO680682.1	0.203624484	0.000649682	1.674921792
FO681491.1	0.007419663	0.001107253	1.803240822
FOCAD	14.15839323	0.000719615	0.568873798
FOPNL	74.30427501	0.001820084	0.60905687
FOSL1P1	0.588837282	0.003898121	1.578915375
FOXA1	0.909145644	5.16E-08	2.363286632
FOXA2	1.354266852	9.83E-05	1.900907649
FOXB1	0.17922185	2.33E-05	2.151966206
FOXD1	3.60893086	0.000453033	1.736481504
FOXD1-AS1	0	0.004606615	1.557019068
FOXD2-AS1	6.539455382	0.000120936	1.782897356
FOXD3-AS1	0.100133641	9.97E-05	1.901143634
FOXD4L1	0.225840772	3.25E-05	1.867622397
FOXG1	0.153476151	0.001119871	1.794347853
FOXG1-AS1	0.043757826	0.001037307	1.831337321
FOXH1	0.238053028	5.54E-06	1.97426087
FOXI3	0.025113989	0.001607224	1.668810956
FOXK2	14.25180419	0.003262121	1.561404825
FOXL2	0.09574821	0.003070726	1.685790238
FOXL2NB	0.046025651	0.002711737	1.65097804
FOXM1	10.0103481	4.75E-10	2.52881678
FOXN2	21.84022904	0.000601508	0.5774522
FOXN3	32.44254124	1.41E-05	0.491645898
FOXN4	0.151029137	7.84E-07	2.197192536
FOXO1	27.24479009	0.000229024	0.539842296
FOXO3	46.75434592	8.45E-06	0.467583738
FOXO3B	1.583018812	0.00069701	0.569850228
FOXO4	26.79847224	0.000446796	0.572880471
FOXO6	3.020326026	0.000552303	1.68684475
FOXP3	4.93253532	0.001561738	1.620425033
FOXP4-AS1	0.808810801	6.55E-07	2.171372694
FP236240.2	0	0.004606615	1.557019068
FP236240.3	0.000486262	1.79E-08	33.81032893
FP325318.1	0.005735223	0.000532058	2.160539402
FP325331.1	0.215768332	3.75E-07	2.140921647
FP565260.6	1.389325836	0.001480092	1.617492307
FPGT	20.17234492	0.004927042	0.645949011
FRAS1	11.06649172	1.59E-05	0.461761637
FREM1	2.00814127	0.000224559	0.440872254
FREM2	7.099138679	6.75E-07	0.427921775
FRK	12.51396827	9.23E-06	0.463789442
FRMD3	34.87917531	2.48E-05	0.507276394
FRMD4B	20.63255856	0.000982179	0.593759339
FRMD5	0.331948168	7.91E-07	2.282204343
FRMD6	9.604247078	0.003017821	0.614023689
FRMPD2	0.455410465	0.00111865	0.55982764
FRMPD3	0.215928656	0.001332096	1.688705015
FRMPD4	0.136153622	0.000952567	1.890042102
FRS2	18.37803758	0.000654027	0.583445028

FRY	10.37442748	3.03E-05	0.489764629
FRYL	18.15263092	0.002283166	0.612760354
FSCN2	1.134271155	0.001682187	1.683507728
FSD1	0.541516762	1.86E-06	2.068708713
FSIP2-AS1	1.158936186	4.98E-05	1.834366785
FSTL5	0.108763317	0.003867245	1.829965271
FTH1P19	0.290498341	2.92E-07	2.163321214
FUBP3	42.37004085	0.000146458	0.554729913
FUCA1	206.8566964	0.000588787	0.555470319
FUT10	4.8980674	5.67E-06	0.483636368
FUT11	58.88926477	0.001606806	0.615952291
FUT3	2.689146896	0.002118259	0.552732743
FUT6	20.13969894	5.95E-06	0.454375271
FXYD5	125.1118471	0.000579665	1.684319553
FXYD6	30.9286579	0.000289696	0.545268183
FXYD6-FXY	0	0.004606615	1.557019068
FYCO1	24.21775217	9.75E-05	0.507363275
FYN	43.28019724	0.004574883	0.641364022
FZD1	105.1649428	1.68E-07	0.435051128
FZD10	1.155909322	0.003943092	1.565471224
FZD10-AS1	0.234732006	0.000176605	1.765698462
FZD2	7.008601864	5.85E-06	1.97388965
FZD4	113.6980245	9.64E-08	0.417775513
FZD5	25.66254632	0.000246282	0.541457377
G2E3	6.649460358	0.003670288	0.629626315
G3BP2	83.8821083	0.002739852	0.624934245
G6PC	12.51046605	0.00021615	0.452739482
G6PC3	58.26479148	0.000116098	1.787387767
G6PD	43.09820066	0.000529071	1.694829476
GAB1	14.96682922	5.45E-07	0.443217566
GAB2	26.87331321	0.000326425	0.560207247
GAB4	0	0.004606615	1.557019068
GABARAPL	116.0128811	0.000294996	0.563332148
GABARAPL	0	0.004606615	1.557019068
GABBR1	11.11215017	3.24E-08	2.251493353
GABPA	30.14183433	1.49E-05	0.497941838
GABPAP	0.078291229	0.000658289	0.5535732
GABPB1-AS	4.79461384	0.000723152	1.66831242
GABRB3	6.031995481	1.01E-05	0.450884624
GABRG3	0.029761019	0.000336872	1.756220167
GABRG3-AS	0.024632893	0.003383544	1.709494775
GABRQ	2.147087671	0.000227603	0.504425334
GABRR3	0.009830105	0.000632596	1.715382003
GACAT3	0.049515503	1.54E-05	1.971340313
GADD45A	119.2436883	9.55E-06	0.442876769
GAGE10	0.145595314	2.01E-06	2.040391154
GAGE12B	0	0.004606615	1.557019068
GAGE12C	0	0.004606615	1.557019068
GAGE12D	0	0.004606615	1.557019068
GAGE12E	0	0.004606615	1.557019068
GAGE12F	0	0.004606615	1.557019068
GAGE12G	0	0.004606615	1.557019068
GAL3ST2	0.310662481	0.000610616	1.709481519
GALC	36.61104761	3.34E-05	0.515277354
GALK1	31.79183209	0.00270194	1.57580132
GALK2	13.04187307	6.16E-06	0.487069536
GALM	78.87864568	0.000832441	0.587045416
GALNS	16.30004619	6.56E-05	1.821554775
GALNT12	17.21805223	0.000167993	0.531777926
GALNT14	218.5987824	3.29E-05	0.518148562
GALNT18	63.00656508	0.000115956	0.540520434
GALNT2	60.01792502	0.000121009	1.792038594
GALNT4	0.778798405	1.00E-05	0.473044691
GALNTL5	0.039832046	0.000111678	1.867403285
GALR2	0.128342332	0.003201997	1.572724241
GAPDHP14	0.56560824	0.000664848	1.782100547
GAPDHP39	0.196718354	0.004668582	1.557168951
GAPDHP45	0.282480084	0.000423845	1.728748938
GAPDHP50	0.013408084	0.00029542	1.970059575
GAPDHP51	0.164357334	0.001317759	1.687884195
GAPDHP62	0.407808208	0.000255503	1.735250787
GAPLINC	1.543168978	0.004564464	1.542147504
GAPVD1	16.29674239	1.21E-06	0.457426983
GAREM1	33.12708306	7.05E-06	0.489582469
GARNL3	3.830918155	8.65E-05	0.520998255
GARS	86.34945334	9.84E-05	1.806324629
GARS-DT	4.853435634	3.05E-05	1.858735663
GAS2L1P2	0.16144177	0.001382991	1.670648006
GATA4	0.505904047	0.00035399	2.471028867
GATA6-AS1	0.585896764	3.79E-05	1.867072618
GATM	214.3363089	0.000200859	0.539851688
GAU1	0.195341466	0.00321359	1.557904621
GBA3	126.3202698	3.68E-07	0.432529816
GBAP1	3.904373583	3.17E-05	1.862717357
GCC1	22.49950955	0.000610969	0.583239837
GCC2	19.30227324	0.000139221	0.530743945
GCK	0.476480998	0.001180647	1.842266136
GCKR	0.726931887	1.12E-11	3.241818439
GCLC	19.89397154	0.00075173	0.588201345
GCLM	27.40036855	0.001072171	0.592236656
GCNA	1.64068631	6.41E-05	1.817106314
GCNT2	12.37981181	6.39E-05	0.515157928
GCNT4	4.86856984	0.000139173	0.493635086
GCSAML	0.355644205	0.000540036	0.548604983
GCSHP3	0	0.004606615	1.557019068
GCSHP4	0.186212937	0.000326465	1.715174141
GDA	33.94473461	0.000547248	0.502444441
GDF1	0	0.004606615	1.557019068
GDF10	0.705265196	0.003256666	1.61596696
GDF5	0.404620477	1.39E-05	2.016891655
GDF5OS	0.068758201	0.000514355	1.742838697
GDF6	20.06158936	0.004076952	0.614926686
GDF7	3.49711889	0.001622386	0.578086384
GDI2	286.1395181	1.39E-05	0.512616496
GDI2P2	0.390454488	2.88E-05	0.492044742
GDPD2	0.926446897	0.002688716	0.605795933
GDPD3	9.3911631	0.000120455	1.780729829
GEMIN5	25.72790074	0.000421872	0.575331819
GEMIN7	31.12307831	0.000163324	1.767286853
GEMIN8P4	2.441751778	5.43E-05	1.836529501
GEN1	3.825430349	4.44E-05	1.843598798
GFM1	21.75376785	0.000516295	0.575227524
GFM2	30.51069551	0.00022662	0.548968414
GFOD1	8.149136079	1.59E-05	0.467339538
GFOD2	15.91193237	0.000167204	0.550944109
GFPT2	12.53587476	2.54E-06	2.051143998
GFRAL	0.011426981	0.003970874	1.692336447
GGACT	10.10167718	0.000742122	0.489732273
GGN	1.028976699	0.000283802	1.728992404
GGT1	147.4072935	6.28E-05	0.528539718
GGT8P	7.688319944	0.002406938	0.596212146
GGTA2P	0.383845175	0.000802624	1.656972028
GH2	0.051351638	3.29E-05	2.299373731
GHDC	85.76307764	3.40E-05	0.525222837
GHITM	361.4870308	6.74E-05	0.53235491
GHR	13.96018185	1.76E-05	0.46314431
GHRH	0.057757215	0.004586939	1.614467179
GHRLOS	1.39088749	0.001112995	1.633851132
GID4	14.40821223	1.87E-05	0.507753532
GID8	61.04882292	0.000747219	0.587996303
GIGYF1	44.91578509	0.003856965	1.546917895
GIGYF2	28.53858265	3.10E-07	0.437717499
GIHCG	19.82195646	4.44E-06	1.990970452
GIMAP6	83.24786487	0.000245076	0.560774335
GIN1	7.694883558	2.04E-06	0.467632691
GINM1	145.8917832	0.00299323	0.630446723
GINS1	5.935353446	0.002059458	1.59442791
GIP	0.25364029	2.05E-07	2.321053165
GIPC2	50.98135713	2.32E-10	0.361820045
GIPR	1.119529802	0.000256546	1.746525179
GIT2	21.97835526	0.001134675	0.598540805
GJA5	45.85219386	0.0001021	0.518152905
GJB1	32.72991486	2.89E-07	0.429283783
GJB2	51.9775457	0.002179545	1.614775006
GJB3	1.179314533	0.00094903	1.829573071
GJB6	0.585893833	0.000478752	2.024521068
GJD4	0.135196352	1.90E-06	2.043724138
GK	15.94259939	0.003612426	0.618520426
GK-IT1	0.783022718	0.000123905	1.796078399
GLCE	23.97325136	0.000178121	0.543525892
GLDCP1	0.166265826	0.004599258	0.610168804
GLE1	47.08492043	4.06E-05	0.527237644
GLG1	91.91049689	1.27E-05	0.495214391
GLI4	15.0880567	0.000696276	1.66968416
GLIS3	15.80782795	0.00461394	0.638250804
GLIS3-AS1	0.417094754	4.57E-05	1.905011963
GLOD4	48.46923392	4.31E-06	0.489972704
GLRA1	0.056141421	0.00242172	1.613506684
GLRA3	0.032626487	0.000155144	1.850312813
GLRX5	43.01463941	0.000474961	0.555182519

GLT6D1	0.039496226	0.002515719	1.634330977
GLUD1	315.9917994	0.004007096	0.625044338
GLUD1P3	5.526273739	0.001220875	1.625009767
GLUD2	7.772491534	1.15E-08	0.405194328
GLYAT	86.78304541	0.000182594	0.554823174
GLYATL1	49.93005483	3.31E-05	0.507462684
GLYR1	80.922434	0.000131263	0.552484386
GM2AP2	0.089478555	0.00064926	1.67461919
GMCL1P2	0.196207152	0.003962416	1.567013862
GMPPA	33.13124355	0.00070243	1.678216355
GMPPB	11.40022954	0.002215758	1.588943001
GMPR2	83.84407542	0.000238684	0.564258082
GNA11	54.6771826	7.54E-07	0.4344994
GNA14	12.19139596	0.000882074	0.585625318
GNAQ	79.09270396	5.10E-05	0.533154064
GNAQP1	1.052443966	0.002811911	0.606693731
GNAS	339.2479749	9.05E-05	1.803038672
GNAS-AS1	0.28621654	2.31E-05	1.891847291
GNB1	282.5101623	7.32E-05	0.543717317
GNB1L	2.220643897	4.00E-06	2.001803069
GNB3	0.97375936	1.40E-07	2.229722446
GNB5	11.00387722	0.000236715	0.556064665
GNE	38.50166319	7.82E-08	0.40895437
GNG10	27.01262254	0.001103385	0.602907003
GNG12	160.5682528	0.000565245	0.57873363
GNG13	0.078798541	2.04E-05	1.949669387
GNG3	1.17800182	0.000339326	1.712670993
GNG7	13.36741917	3.09E-05	0.488237222
GNL1	33.21446193	0.002124034	0.619339882
GNL3	43.5103336	0.000285293	1.728390439
GNL3L	16.22660777	0.000978228	0.593470968
GNMT	2.665061695	1.41E-07	2.686954381
GNRH1	5.436797772	6.97E-05	1.809779396
GNRH2	0.337351393	0.000468279	1.696388424
GOLGA3	31.13806496	1.49E-05	1.915689713
GOLGA4	27.8538942	0.000817786	0.568278705
GOLGA5	72.28561522	0.001719661	0.610158786
GOLGA6A	0.023346387	0.002361786	1.594003235
GOLGA6C	0.007773057	1.57E-05	2.091720619
GOLGA6L1	0.431674195	0.000193094	1.75110118
GOLGA6L2	0.490578188	5.51E-11	2.945727156
GOLGA6L7	1.127377318	3.94E-06	2.61248684
GOLGA6L9	1.41134959	3.44E-05	1.85310318
GOLGA7	103.9225797	1.59E-06	0.471069785
GOLGA8A	16.28341869	9.47E-05	1.803184375
GOLGA8B	11.10107274	0.000241848	1.736531602
GOLGA8DF	0.016853021	1.93E-06	2.083209244
GOLGA8EP	0.016761861	5.11E-07	2.143363374
GOLGA8UF	0.093252877	0.00295204	1.578656364
GOLGB1	59.94686802	0.003740787	0.632163867
GOLIM4	93.5256204	6.97E-05	0.529165311
GOLPH3L	58.93478816	0.000802158	0.591661679
GOLT1A	22.07828397	0.002107914	1.60258822
GON7	34.89148508	0.004212778	0.63368193
GORAB-AS	0.275301476	0.003914953	1.550530789
GOSR1	25.27412703	2.80E-05	0.521339055
GOT1	175.6798961	0.000454553	0.554124914
GP1BB	0	0.004606615	1.557019068
GPAA1P2	0.329760368	0.000242054	1.949150861
GPALPP1	8.704701049	0.0018454	0.611542134
GPANK1	31.65843689	0.000397881	0.579143567
GPATCH3	24.70819888	2.06E-05	0.522369405
GPBP1	67.62592157	5.40E-05	0.533624939
GPC1	37.78087105	0.000415377	1.704332686
GPC2	0.683184629	2.27E-05	1.882688206
GPC6	46.4315311	0.001408282	0.593515526
GPD1L	27.29874957	0.004269118	0.520224601
GPHN	15.3004064	0.000580922	0.527293354
GPKOW	51.84777619	0.000706086	0.594288766
GPM6BP1	0	0.004606615	1.557019068
GPN3	32.71523703	0.000194823	0.563719343
GPR1-AS	0.038454292	1.44E-05	2.008022159
GPR107	58.59122997	9.79E-05	0.537285269
GPR108	112.2874195	9.29E-07	0.466961161
GPR137	33.19053094	0.000966746	1.648213081
GPR137B	93.05433729	0.001747646	0.606153351
GPR137C	1.270428805	0.000727845	0.553840318
GPR142	0.077423458	0.002150909	1.599314607
GPR149	0.013925108	0.001528902	1.740395356
GPR153	9.223896912	0.001432639	1.628537386
GPR158-AS	0.018186082	0.000336254	1.75167217
GPR160	33.92383157	0.000154361	0.547321824
GPR162	4.372789012	0.000250773	1.732613689
GPR173	3.518227514	2.34E-07	2.194327409
GPR19	0.954941233	0.003607262	1.559719845
GPR25	0.331607089	0.000832457	1.663010576
GPR32	0.060867973	0.002478186	1.685734303
GPR45	0.415731234	0.003657997	1.583478641
GPR61	0.257551925	0.0010017	1.732870102
GPR63	0.604514528	0.001476206	1.619077585
GPR68	4.32282652	0.000185261	1.757810787
GPR78	0.041408993	1.63E-11	2.757536267
GPR83	0.347193279	1.11E-05	1.94273736
GPR84	2.646455023	0.000216083	1.753037427
GPRACR	0.17984529	5.82E-08	2.377563018
GPRASP2	20.17993043	0.000378638	0.565018186
GPRC5A	12.24750628	2.16E-07	2.287801199
GPRC5B	45.19046455	0.004316585	0.625310951
GPRIN1	5.810986652	0.001225595	1.632696895
GPS2	8.054213608	1.46E-05	1.905161209
GPS2P2	0.060518296	0.001491794	1.706738055
GPSM1	9.504880791	1.77E-05	1.903686088
GPSM2	4.884505052	0.000897077	1.691294338
GPX1	665.7762904	0.004006156	1.547267504
GRAMD1A	52.56231996	6.37E-06	1.973066993
GRAMD1B	5.964933063	0.003916364	1.558112763
GRAMD1C	10.33599334	9.30E-05	0.516530815
GRAMD2B	37.25873652	0.000279463	0.556689247
GRAMD4P2	0.087156034	0.000273537	1.77319629
GRB10	82.8473286	0.001844846	0.602568519
GREB1L	0.964322539	0.000121109	1.789664412
GREM1	1.497073243	0.002837938	1.646587389
GREM2	1.651564475	0.001867704	2.168833602
GRHPR	56.23500034	0.000391841	0.571789581
GRIK1-AS1	0.410679655	7.96E-06	1.947942177
GRIK4	1.241728349	7.32E-05	1.880649489
GRIN2D	1.580902187	3.63E-10	2.512980029
GRIN3B	1.088125579	0.00029272	1.744419384
GRK7	0.13817141	0.002198085	1.590533748
GRM4	0.088077649	2.84E-09	2.800743853
GRSF1	49.4937128	0.000990952	0.584066667
GSDMB	5.678666916	7.91E-08	2.213802741
GSDME	12.1758126	0.003354515	1.565177168
GSEC	2.226244123	1.94E-06	2.047772419
GSG1	1.014550095	0.002904377	1.68660609
GSK3B	31.55164257	0.000112685	0.530466911
GSTM3P1	0	0.004606615	1.557019068
GTF2B	41.40919475	0.000189706	0.561666487
GTF2E2	35.04433535	0.004843124	1.537260592
GTF2H4	5.144895336	0.003388617	1.555149962
GTF2I	20.34673455	3.61E-07	0.444966579
GTF2IP11	0.009041347	0.002013355	2.750730586
GTF2IP13	6.859373269	1.51E-06	2.050253396
GTF2IP20	9.57206397	5.47E-06	1.963759936
GTF2IP23	7.90896764	0.002081792	1.59013855
GTF2IP3	0.024427956	0.000393537	2.452590465
GTF2IP7	0.175109945	2.50E-07	2.230711253
GTF3A	137.8732857	0.001069306	1.646354117
GTF3C2-AS	0.436301343	7.66E-06	1.94746583
GTF3C4	16.67529062	7.03E-06	0.489172767
GTPBP10	8.105184467	0.001457777	0.599245555
GTPBP2	44.55689143	1.06E-05	1.938143527
GTPBP3	13.46717619	0.004166686	1.540997532
GTPBP8	9.629013679	3.11E-05	0.507551888
GTSE1	3.759591739	4.96E-10	2.502003077
GUCA1A	0.049706552	0.000654258	1.747577209
GUCA1B	2.390886338	0.001175885	1.631614034
GUCY1A1	68.94399598	3.00E-05	0.504745104
GUCY1A2	8.289309543	0.000669979	0.570918781
GUCY2D	0.561754447	0.001816838	1.617987461
GUCY2EP	0.331674777	1.41E-06	2.263404254
GUCY2F	0.023921935	0.000420749	1.736691602
GUF1	24.80255431	0.003398509	0.620622904
GUSBP10	0.048057814	0.003704791	1.710631803
GUSBP5	1.328102272	0.001326709	0.575084015
GUSBP8	0.100275059	0.000293296	1.782273568
GXYLT1	20.48471152	0.00239794	0.616639167
GYG2	2.324385762	1.13E-07	2.272506007
GYPA	0.763973938	0.001622337	0.540502421
H1FX-AS1	2.000515912	0.003400012	1.563100168

H2AFB2	0	0.004606615	1.557019068
H2AFV	125.4994601	2.59E-05	0.516853063
H2AFVP1	1.56461683	0.004552191	1.540209728
H2AFX	46.85338962	2.03E-08	2.302429588
H2BFWT	0.038962926	0.004314848	1.588094666
HABP4	28.06695305	1.74E-06	0.44618384
HACD1	2.364848128	1.84E-05	1.907763092
HADH	66.60128759	8.49E-06	0.450917837
HADHA	360.8384704	2.78E-05	0.520070355
HADHAP1	0.395890893	6.01E-07	2.170810079
HADHB	193.7657446	1.63E-05	0.503483544
HAGLR	17.02679511	0.000124648	0.515298363
HAMP	2.305686861	7.00E-05	1.861182736
HAO2	54.58473522	3.72E-06	0.41971698
HAPLN3	13.36116663	0.002293024	1.590916077
HAR1A	1.146645035	0.001386919	1.640581274
HAR1B	0.436838238	2.39E-06	2.050337574
HAS2-AS1	0.338184313	4.71E-06	2.084967127
HASPIN	1.193905323	5.12E-05	1.842224386
HAUS2	18.92809661	6.80E-05	0.536495944
HAUS4	39.17483847	0.002209568	0.617803203
HAUS5	13.00284256	3.27E-05	1.864894444
HAUS8	3.295132932	1.63E-05	1.915010421
HAVCR1	114.7010223	0.003337867	0.599773786
HBP1	45.03852187	7.47E-08	0.427651318
HBQ1	0.455421716	0.0002299	1.994673858
HBS1L	14.18351899	0.002903279	0.630057106
HCFC2	14.89583109	0.00134404	0.602826913
HCG11	16.07706101	0.004020508	0.618129076
HCG25	1.655136273	0.001418018	1.614751165
HCN4	0.244226373	0.001248644	1.725058797
HCST	40.95079088	0.000152575	1.770662774
HDAC10	7.562916633	3.60E-06	1.999121726
HDAC4-AS1	2.946897982	6.98E-05	1.818231456
HDAC5	38.73859821	0.000652762	0.586485916
HDHD2	24.31107146	2.04E-05	0.515471238
HEATR5A	6.586693183	0.002531655	0.61328364
HEATR5B	23.03236575	5.46E-05	0.525666517
HEATR9	0.347503739	0.000470644	1.701791736
HECA	33.46761548	0.000878257	0.588688907
HECTD1	43.61778572	9.92E-06	0.483608443
HECW2	17.10330874	4.73E-05	0.48722369
HEG1	70.31961989	1.21E-05	0.465460984
HEIH	94.39541127	0.001109735	0.597838742
HELLPAR	0.022951559	1.58E-06	2.064993752
HELQ	15.41621085	5.66E-05	0.53036567
HEPACAM	0.088139959	0.00058714	1.724562803
HEPH	15.07563256	0.001012791	0.5795693
HEPN1	0	0.004606615	1.557019068
HERC1	18.94074171	7.90E-05	0.512884266
HERC2	13.30328556	0.000962081	0.58055362
HERC2P2	10.87378512	9.40E-07	2.068765541
HERC2P9	3.007435295	0.000200066	1.74459662
HERC3	23.38938463	8.25E-07	0.450392489
HERPUD1	206.7030311	0.001730043	0.61015314
HERPUD2	53.62330336	0.001313131	0.603300061
HES7	0.350586052	0.00033677	1.744214051
HESX1	2.476882	1.93E-05	1.898235504
HEXD	22.28329846	0.003957461	1.547000985
HGF	13.31150826	0.000290039	1.888321592
HGH1	31.4647753	5.74E-08	2.256013502
HHAT	9.204186999	5.87E-05	0.51535105
HHIPL2	0.20474267	5.79E-11	2.659963954
HHLA2	85.16254226	4.13E-06	0.474344787
HHLA3	22.14089938	0.000221119	1.744769826
HIBCH	22.84842952	5.66E-06	0.468948123
HID1-AS1	2.767002705	0.001854539	0.581906642
HIGD1AP11	0	0.004606615	1.557019068
HIGD1AP18	0.308627361	0.001974475	1.599996629
HIGD1AP9	0.180900858	0.004166245	1.560058332
HIGD2B	0.087343587	0.000459388	1.697666122
HIKESHI	21.0111914	1.69E-05	1.931278126
HINT3	47.85759643	3.39E-05	0.508757961
HIP1	44.19127785	0.002000165	0.613907079
HIPK2	133.7235114	3.43E-05	0.509396151
HIPK3	77.06492617	0.000107939	0.536230046
HIRIP3	26.55828219	0.004396076	0.644606105
HIST1H1B	0.57426835	0.003875711	2.310726143
HIST1H2AE	0.306911294	1.72E-05	2.145119178
HIST1H2AJ	0.303554215	2.55E-05	2.024557342
HIST1H2AK	0	0.004606615	1.557019068
HIST1H2AL	0.208970623	0.000124207	1.889205285
HIST1H2BH	2.39434162	3.97E-05	1.881564576
HIST1H2BP	0.12663196	0.001881198	1.619872057
HIST1H3B	0.804027444	1.43E-06	2.511652362
HIST1H3D	4.9663157	0.000901234	1.667949101
HIST1H3J	0.24965687	7.52E-06	1.98793355
HIST2H2AC	3.789144436	2.80E-07	2.369335714
HIST2H2BF	1.644725755	0.001105488	1.703543725
HIST2H3D	0.536727404	0.001234072	1.653336504
HIVEP1	10.21159518	0.004312807	0.632191882
HJURP	4.360618503	4.16E-13	2.873865917
HK3	7.72946247	0.002905468	1.573459077
HLA-E	2105.320615	0.000650116	0.58913726
HLCS-IT1	0.22951401	1.04E-05	1.954809221
HLF	22.0058329	2.67E-06	0.447350753
HLTF-AS1	0.062918667	0.002062893	1.705849537
HM13	68.0883261	6.95E-05	1.820503289
HM13-IT1	5.785287253	2.42E-06	2.01725915
HMBS	12.94727071	1.05E-06	2.082239508
HMCN1	8.847356116	0.001404079	0.586863475
HMG20A	19.00389958	0.001553134	0.615277391
HMGA1	58.79501264	0.003340994	1.605170554
HMGA1P4	3.528754318	0.001135204	1.636268087
HMGA1P7	0.029334522	0.003092092	1.894997126
HMGA2	0.807476166	4.25E-07	2.942469216
HMGA2-AS	0.134492973	0.001891634	1.721066517
HMGB1P16	0.227831974	0.002359464	1.64819707
HMGB1P24	0.243506218	0.00057707	1.697035942
HMGB1P28	0.07822908	1.37E-05	1.940775422
HMGB1P3	0.669888972	2.74E-05	1.869918257
HMGB1P32	0.021351595	0.003379756	1.862272327
HMGB1P41	0.588197296	0.000746218	0.561884364
HMGB1P47	0.069814781	0.003983106	1.660959205
HMGB1P5	30.78819674	0.002119948	0.598345168
HMGB1P50	0.098355532	2.94E-06	2.077759603
HMGB1P51	0.254610543	0.000111755	1.8250732
HMGB2	91.94311923	0.001197304	1.63201009
HMGB2P1	0.80747811	5.32E-07	2.113268286
HMGB3P17	0.093319164	1.91E-05	1.911408262
HMGB3P23	0.02208849	0.000147622	2.144714426
HMGB3P3	0.10900849	0.002023201	1.615408357
HMGB3P5	0.153158378	3.87E-07	2.202085435
HMGB3P7	0.698903603	0.000585073	1.881205308
HMGCL	44.84765404	0.000235009	0.558638711
HMGCLL1	0.651474323	4.97E-06	0.424074193
HMGCR	23.05671448	0.000150435	0.541656179
HMGCS1	37.86269705	0.000117198	0.540856654
HMGCS2	76.17936559	0.000187567	0.450010688
HMGN1	79.07177809	0.000382698	1.710589626
HMGN1P14	0.197995667	4.96E-05	1.911096934
HMGN1P18	0	0.004606615	1.557019068
HMGN1P24	0.462038416	4.90E-07	2.112151185
HMGN1P3	0	0.004606615	1.557019068
HMGN1P6	0	0.004606615	1.557019068
HMGN2P32	0.307900629	0.0015766	1.674701254
HMGN2P40	0.876557819	0.001033691	0.582207199
HMGN2P5	8.421925886	0.00018137	0.551639556
HMGN3	469.7009577	9.13E-05	1.802191732
HMMR	4.479973552	8.94E-06	1.961381504
HMSD	0.16283828	9.46E-05	1.81691298
HNF1A	15.92191363	0.000717672	0.589027241
HNF4GP1	0.018666183	0.001898081	1.782643498
HNMT	88.00167723	1.31E-06	0.466512321
HNRNPA0	70.48781081	1.10E-06	0.444148625
HNRNPA1P	0.910975169	0.003954669	0.636907969
HNRNPA1P	5.2371296	2.09E-07	2.152514226
HNRNPA1P	0.127303126	0.004745562	0.621407742
HNRNPA1P	0.981759617	0.000287178	1.737460971
HNRNPA1P	0.976525075	0.000138637	0.534601758
HNRNPA1P	1.308827714	0.000708878	0.573051335
HNRNPAB	210.169993	0.00087408	1.65791723
HNRNPCP7	2.71034095	0.000128071	1.774947322
HNRNPDLP	0.014937606	0.000265991	3.081783155
HNRNPDLP	0.199349425	0.003269307	0.601410563
HNRNPF	219.4721449	0.004244063	0.647131854
HNRNPH1P	1.055518838	0.000904983	1.653671307
HNRNPH2	172.7726681	2.69E-05	0.508543127
HNRNPK	440.2146761	5.82E-06	0.4983455
HNRNPKP4	1.283218125	9.01E-06	0.469475583

HNRNPKP5	0.28942493	0.004152026	1.566261198
HNRNPLL	21.65152693	0.00386227	0.637796371
HNRNPM	114.591196	0.001251356	0.60570461
HNRNPU	178.1207856	0.000449293	0.581399844
HNRNPUL2	0.786387829	0.00421447	0.632615559
HOMEZ	12.45488354	1.32E-06	0.447062303
HOOK1	46.01979074	2.66E-06	0.463859217
HORMAD1	0.252139041	0.002146252	1.601213927
HOTAIR	1.686134452	6.93E-05	1.823089723
HOTAIRM1	15.30513837	8.92E-10	2.451387523
HOTTIP	0.127075897	1.85E-07	2.478929181
HOXA-AS2	8.504662622	0.000324087	1.717992675
HOXA1	2.341476684	9.94E-05	1.798854759
HOXA11-AS	1.363045253	0.000360172	1.756549165
HOXA13	1.637566959	1.83E-05	2.040038347
HOXA2	2.392729182	9.95E-06	1.942253626
HOXA3	14.10861541	0.000416063	1.706795858
HOXB-AS1	9.027198059	0.000954781	1.644376324
HOXB-AS4	2.654824875	5.33E-07	2.151417853
HOXB13	1.518053054	4.18E-07	2.384125007
HOXB9	10.09204482	0.003799365	1.662503646
HOXC10	91.1233468	0.000851646	0.596842223
HOXC11	2.715838663	0.001937868	1.612452331
HOXC4	12.83250101	0.000728302	1.662179197
HOXD1	1.776735604	0.000225959	0.507584464
HOXD3	3.017527099	0.000127775	0.541154752
HOXD8	50.9578909	0.000990523	0.599477505
HPDL	0.800939315	0.004395739	1.547295832
HPR	2.776189788	0.000274589	2.209409552
HPX	3.271889581	0.003440882	2.115149044
HRH2	30.40254892	3.08E-05	0.470307537
HS1BP3	36.43383593	6.56E-06	0.492457918
HS1BP3-IT1	5.037796448	0.000374337	0.48720626
HS2ST1	34.18789951	0.000799415	0.59541642
HS3ST3A1	2.081602419	9.81E-08	2.720067192
hsa-mir-1253	0	0.004606615	1.557019068
HSD11B1	7.763656528	2.34E-05	1.987279443
HSD11B1-A	4.630527598	0.001336465	1.647139882
HSD17B12	39.43607457	0.000720636	0.576028104
HSD17B3	3.143322812	0.000850318	1.668936608
HSD17B4	61.70618375	2.65E-05	0.497685242
HSD17B7P2	5.941421241	6.58E-05	1.813708565
HSD17B8	67.33332565	1.89E-05	0.511362374
HSD3BP5	3.354234054	0.001254861	0.570975711
HSDL1	22.64490653	0.000366907	0.561358333
HSDL2	107.6923716	1.01E-05	0.481553182
HSF1	59.05868605	0.000203627	1.753813386
HSFY6P	0	0.004606615	1.557019068
HSFY7P	0	0.004606615	1.557019068
HSH2D	4.478808272	2.03E-06	2.048284954
HSP90AA2F	0.506158725	1.39E-05	0.463108892
HSP90AB2F	1.367334605	0.000652774	0.562462992
HSP90AB7F	0.029352979	0.000214209	1.767689273
HSPA12B	22.06361658	0.000956121	0.587624286
HSPA1L	6.080715917	5.36E-06	0.484622968
HSPA4L	15.01903357	2.82E-07	0.428624253
HSPA6	11.69139587	0.00111996	1.660848776
HSPA7	19.49829484	0.000486419	1.700970291
HSPA8P13	0.010875449	0.002628069	2.472369021
HSPA8P14	0.11641146	0.004214244	1.553408838
HSPA8P8	0.293933161	0.00446921	0.569115848
HSPBAP1	7.001450369	0.000420219	1.698593422
HSPD1P4	1.094912431	0.000295943	1.732473838
HSPG2	159.1862629	1.89E-05	0.461213182
HTATSF1	92.76820352	0.000181631	0.550681763
HTATSF1P2	0	0.004606615	1.557019068
HTR1D	0.593803976	2.31E-05	2.096143145
HTR2C	0.026474823	0.000604853	1.893400458
HTR3A	0.341630451	9.92E-07	2.309891907
HTR3C2P	0.05821986	0.000500177	1.706112733
HTRA2	35.58373607	0.000350786	0.579080443
HTT	19.45817944	0.000162245	0.536169142
HUNK	6.217019661	0.002709355	0.609016108
HUS1B	0.627814373	7.34E-05	1.816241173
HUWE1	64.42269519	0.000390767	0.565635015
HYAL1	50.40403867	1.54E-05	0.495075028
HYI-AS1	1.167083761	4.62E-05	1.840595394
HYPK	1.854142627	2.09E-05	1.885960712
HYPM	0.018650229	0.001145307	2.124983422
IARS	57.88989002	0.001385563	0.604172799
IARS2	119.076492	4.85E-05	0.533072445
IBA57	5.960222524	2.04E-07	0.420089508
IBA57-DT	1.426562651	0.003149002	1.573689708
IBSP	3.548939296	0.000626702	1.750592446
ICAM4	0	0.004606615	1.557019068
ICAM5	0.605951111	8.20E-05	1.90576714
ICE1	22.2783319	0.000281266	0.562190932
ICK	19.09661681	0.001418536	0.602031944
IDH3A	13.77110087	0.003108499	0.605920127
IDI1	37.17166076	1.38E-05	0.48315535
IDI2-AS1	0.265480056	0.002236358	1.603374691
IDNK	18.60554168	0.003622252	0.624832675
IDUA	15.35917904	1.04E-08	2.330892139
IFFO1	21.88941739	3.13E-06	1.998214928
IFI16	91.0256263	0.000337676	1.722400784
IFI30	4.139611507	2.28E-10	2.532471041
IFIH1	35.68381248	0.000229872	0.552594139
IFIT1	57.7192329	6.61E-05	0.533032361
IFIT5	42.57431818	9.79E-05	0.546701034
IFITM2	421.7317326	0.002196071	1.592050264
IFITM4P	2.955941678	0.002989662	1.571886083
IFITM9P	1.151051473	0.000806532	1.658770003
IFNA20P	1.282221499	0.001005088	0.584440998
IFNAR1	84.11233319	8.68E-05	0.537796191
IFNB1	0.06242327	0.003650516	1.555023932
IFNE	0.843679474	3.74E-08	2.504922666
IFNL3	0.015809337	0.001644025	1.876288932
IFNL3P1	0.172782169	0.000670531	1.691173646
IFNLR1	5.970604458	0.001250715	0.599814638
IFT20	19.12008661	1.24E-06	2.077040464
IFT27	10.28225616	0.000210245	1.745881889
IFT57	47.42682799	0.002124074	0.623572478
IFT88	17.58683333	0.00014182	0.556806872
IGBP1	233.7694947	2.23E-05	0.503451863
IGBP1-AS1	1.597535056	1.46E-05	1.903011656
IGBP1-AS2	0.261236881	0.000829435	1.65770924
IGDCC4	3.795461981	2.25E-05	1.931273981
IGF1R	38.53331425	3.81E-06	0.455005312
IGF2BP2	5.789954851	7.94E-08	2.311183539
IGF2BP2-AS	0.067838472	2.06E-08	2.306112181
IGF2BP3	1.350625119	2.55E-10	2.637594273
IGF2R	67.54987313	0.000837502	0.590562886
IGFBP1	87.80935291	4.70E-05	2.122923368
IGFL1P1	3.094190236	2.00E-07	3.827872414
IGFL2	0.805356377	0.00010446	1.945114234
IGFL2-AS1	3.573902383	7.12E-09	4.551378642
IGFL3	0.311109477	0.002840152	1.850050992
IGFL4	0.088356	0.004782416	1.544563867
IGFLR1	4.744286671	1.52E-07	2.192867028
IGFN1	3.893033446	4.26E-07	2.987577942
IGHD1-26	0.329688106	0.002646089	2.077099192
IGHD1-7	0.115622065	0.004900976	2.427034027
IGHD1OR1:	0	0.004606615	1.557019068
IGHD1OR1:	0	0.004606615	1.557019068
IGHD2-21	0.596591446	0.003201494	1.872263342
IGHD4OR1:	0	0.004606615	1.557019068
IGHD4OR1:	0	0.004606615	1.557019068
IGHEP2	0.152206412	0.003987406	1.554804725
IGHJ3P	10.62940207	0.000802638	1.822265309
IGHJ4	0	0.004606615	1.557019068
IGHJ5	0	0.004606615	1.557019068
IGHJ6	0	0.004606615	1.557019068
IGHMBP2	9.949013036	0.002325563	1.581275758
IGHV1-18	196.5489362	0.000665934	1.830815872
IGHV1-24	122.5013036	0.000540833	2.033107865
IGHV1-58	11.79374268	0.003435652	1.676906952
IGHV3-30	179.4884286	0.00363115	1.779317692
IGHV4-31	52.09012599	0.00054013	1.960911224
IGHVII-33-1	0.018755236	0.001206511	2.154693506
IGHVII-74-1	0.128447096	0.000461386	1.817859519
IGHVIII-13-	0.019839121	0.0011643	2.238955082
IGIP	28.1045514	0.000260976	0.55807742
IGKC	1486.622142	0.000635054	1.817501984
IGKJ1	0	0.004606615	1.557019068
IGKJ2	0	0.004606615	1.557019068
IGKJ3	0	0.004606615	1.557019068
IGKJ4	0	0.004606615	1.557019068
IGKV2OR22	3.536136811	0.003654872	1.753820112
IGLC1	0	0.004606615	1.557019068
IGLC6	2.118387406	9.38E-05	1.926386988

IGLCOR22-	0.204233785	0.003654543	1.580523595
IGLJ3	0.642878105	0.001076582	1.863895791
IGLJ6	0.123189257	0.00016606	2.470843346
IGLL1	0.644161536	0.000282646	2.02390465
IGLL3P	1.116808234	0.000111072	1.958757897
IGLL5	69.41177944	0.000129728	1.992481211
IGLV3-1	141.6419504	0.003473146	1.736847373
IGLV3-19	408.1301879	0.000529749	1.978931908
IGLV3-21	199.5365104	0.00192714	1.72983493
IGLV5-48	1.717705676	0.002477327	1.770398887
IGSF23	1.820917435	0.002220358	1.717800735
IGSF3	26.19920275	0.00143273	0.581353725
IKBKE	10.92277474	2.16E-07	2.167620398
IKBKGP1	0.141151093	7.79E-05	1.809415335
IKZF5	21.66196754	2.71E-05	0.509805392
IL12A-AS1	0.059382966	0.000584906	1.68402448
IL15RA	24.018371	0.000507752	1.692375251
IL17RD	8.883350544	3.95E-06	0.438412988
IL20RB	42.86966083	4.74E-06	2.048223524
IL20RB-AS1	0.558270384	3.51E-07	2.265029251
IL21R-AS1	0.209357659	3.30E-06	2.003057845
IL22	0.044695937	0.000393339	1.708479016
IL23A	3.160330863	8.78E-10	2.481535034
IL27	0.676450063	0.000208196	1.750261189
IL2RA	6.781773254	3.64E-05	1.916404082
IL34	18.97096938	0.001787006	1.609224807
IL36B	0.054782555	0.003902424	1.689412399
IL36G	0.05253584	0.00037156	1.718381957
IL4	0.273730853	8.54E-07	2.079100046
IL4I1	28.21560706	0.000506646	1.696397811
IL6	20.04565183	4.79E-08	2.492005857
IL6R	36.99843393	0.000110906	0.521940261
IL6ST	175.6060293	0.000121492	0.533891507
ILDR2	3.723201165	0.000144744	0.520819734
ILK	21.64681701	4.24E-05	0.528527394
ILVBL	63.50609524	3.35E-05	0.508922447
IMMP1LP1	0.596988642	1.71E-05	1.906085893
IMMP2L	24.90669952	0.002019712	0.618545967
IMMT	95.74390417	0.001836981	0.608071917
IMPA1P1	0.121379199	0.004601374	1.55852261
IMPA2	186.4661123	3.48E-06	0.473275936
IMPACT	34.05887535	0.000691304	0.592416502
IMPDH1	32.81926858	1.16E-07	2.201015616
IMPDH1P5	0.755281694	0.003191958	1.630113602
IMPDH1P8	0.58417954	0.003653116	1.562421787
INAFM1	33.46361626	1.25E-05	1.927561163
INAFM2	78.27638487	0.004664227	1.533331424
INAVA	13.41958472	0.000858111	0.586940149
INE1	6.378188331	0.0005934	1.674206153
INE2	0	0.004606615	1.557019068
INGX	0.116644389	0.002064961	1.601457404
INHBC	0.786005556	0.000574842	1.716404687
INHBE	4.974537027	1.12E-06	2.228298445
INHCAP	0.40715767	6.76E-05	1.812760078
INIP	25.73917211	9.82E-11	0.352643769
INO80	23.89697239	0.000640703	0.583803262
INPP4B	12.53626608	0.002433833	0.598622881
INPP5A	30.38497416	0.001439553	0.594210896
INPP5F	15.54632484	0.003463792	0.633890935
INPP5K	39.70828983	0.000634135	0.595125857
INSL3	1.810090471	7.82E-08	2.22695964
INSR	164.7225421	0.000424587	0.571709092
INSYN2B	4.054356847	0.000124342	0.507004694
INTS12	17.42102937	0.001628563	0.612518273
INTS14	38.47624409	0.002181741	0.626903961
INTS2	10.7145817	0.001661725	0.608575863
INTS3	32.73129332	6.10E-05	1.826054629
INTS4P1	0.876011523	1.79E-05	1.918192061
INTS5	36.29835042	9.44E-05	0.54206722
INTS6L	8.103787162	0.003922825	1.545314629
INTS8	15.26211491	0.000343875	1.723242308
INVS	11.3009505	0.000104359	0.53387408
IP6K1	41.7158821	0.00127292	0.60263195
IPO11	20.58856429	1.08E-05	0.492436331
IPO5	66.96716909	1.52E-05	0.508374394
IPO8	42.72044138	0.000400235	0.573718482
IPO9-AS1	0.521760598	0.000633843	1.672703714
IPP	14.5881714	1.37E-06	0.44945415
IPPK	2.732072826	4.89E-05	0.522244963
IQCF1	0.0403408	0.002125578	1.651044361
IQGAP1	114.5552429	0.000146748	0.556965845
IQGAP2	27.53175225	1.61E-08	0.381907238
IQGAP3	6.176308141	7.44E-07	2.096054367
IQSEC1	41.19822706	0.001647772	0.594930247
IQSEC3	9.971422474	0.002736379	0.528912664
IQUB	2.778275219	0.004663512	0.632191497
IRAK3	8.046647738	0.004865416	0.627691006
IREB2	25.92017296	6.27E-05	0.53247366
IRF2BP2	165.8268777	0.001024237	0.595673349
IRF3	51.70907415	1.47E-05	1.911665685
IRF5	23.16957518	0.003190623	1.563740916
IRF5P1	0.005256802	0.000663884	2.026997276
IRF6	22.59873565	3.90E-07	0.355794872
IRF7	45.77925495	3.60E-05	1.861856057
IRF9	8.180300137	0.00038414	1.700446372
IRGM	0.224148022	0.00012717	1.783557048
ISCA1	66.65359576	1.30E-05	0.505743432
ISCA2	23.79086087	5.22E-06	0.465199216
ISG15	168.6451443	0.000118003	1.79950613
ISL1	0.056256958	0.003595372	1.754807343
ISL2	0.20345278	2.20E-09	2.458046401
ISLR	42.6945216	0.002163979	1.629767082
ISOC1	51.88610106	0.000175207	0.552873558
ISPD	4.152713784	2.40E-07	0.393072538
ISX	0.042982979	0.001892369	1.805593544
ISX-AS1	0.140051932	0.000145298	1.930671509
ITCH	42.31350494	3.53E-05	0.523152258
ITFG1	48.01250512	3.86E-05	0.514737594
ITGA1	64.02584397	0.003983738	0.634222807
ITGA2	27.20278677	0.000554894	0.556205365
ITGA2B	1.690158424	0.000150708	2.016292698
ITGA6	158.2283863	1.09E-07	0.405099178
ITGA8	21.51514814	4.28E-06	0.459633835
ITGA9	19.31700224	0.000551834	0.560368954
ITGA9-AS1	1.596177674	0.002190592	0.600057394
ITGAE	6.780057857	4.97E-08	2.24792124
ITGB1	328.186824	0.002384734	0.612785901
ITGB1-DT	1.246024616	6.65E-09	2.48962307
ITGB1BP2	1.200338315	0.002579249	1.575000794
ITGB2-AS1	7.018151547	7.16E-06	1.968892809
ITIH4	0.747063735	7.37E-11	2.6662208
ITIH4-AS1	0.226683926	0.001122871	1.651212099
ITIH6	0.042006118	7.12E-06	2.038012956
ITM2B	689.2423821	9.58E-05	0.540602052
ITPA	73.31745861	5.05E-07	2.148952086
ITPKA	5.87559487	1.70E-14	3.145756013
ITPKB	32.33994973	0.000472715	0.564244176
ITPR1-DT	1.968551639	6.05E-06	1.986345916
ITPRID2	53.71763182	1.34E-09	0.367349765
ITPRIP	68.9355152	0.002765418	0.617944873
ITSN2	24.6244243	0.000190462	0.552303518
IVNS1ABP	260.5063825	7.98E-05	0.530458465
IYD	1.064580138	0.000413633	0.499055508
IZUMO2	0.075360571	0.002977608	1.609566139
IZUMO3	0.033400063	0.000327421	1.778580776
IZUMO4	2.664527798	2.17E-08	2.283059718
JADE1	38.43704386	0.00203202	0.60237763
JADE2	34.51825334	0.00146095	0.601651053
JADE3	13.23967712	0.004338632	0.63484803
JAK1	205.6017777	3.16E-07	0.443199893
JAK3	20.79657772	1.13E-06	2.067549898
JAM2	15.95300044	0.000967513	0.58032741
JCAD	50.84917224	0.000622732	0.561300677
JKAMP	41.79063972	0.000650105	0.583170309
JMJD1C	31.32889177	0.000779312	0.576190967
JOSD2	46.19273914	0.000113872	1.790673653
JPH3	0.321198333	0.00017016	2.235677433
JPH4	1.609229953	0.000594776	0.562164506
JPT1	50.49315734	6.05E-08	2.238443459
JPX	13.86267125	0.000162323	1.765466424
JSRP1	2.84776051	0.000369184	1.796536832
JTB	245.7845345	0.002834694	1.572279175
JUP	198.6580621	9.07E-05	0.534550423
KAAG1	1.560076678	0.003508509	1.577069247
KALRN	5.472862739	2.02E-06	0.445699868
KANK3	19.08566231	0.000808387	0.586200069
KANSL1L	26.40210792	0.004168426	0.637512478
KANSL3	31.19974376	0.001710246	1.613395448
KAT14	17.59115794	4.05E-05	0.51548046
KAT2A	51.11012719	7.44E-07	2.073974748

KAT2B	28.28075122	0.002560788	0.620943947
KAT5	40.27028521	2.88E-12	0.322630298
KAT6A	23.73640955	5.36E-06	0.479381939
KAT6B	12.13793383	7.81E-05	0.52973797
KAT7	18.41288151	1.97E-05	0.505732525
KATNA1	28.27736836	0.002670168	0.629939184
KATNAL1	15.04292552	0.001090103	0.594990235
KATNB1	16.85544482	0.000867825	1.65501846
KATNBL1P	0.015719069	0.000348726	1.970772362
KAZN	10.13667962	0.000117228	0.5393151
KBTBD3	6.480063364	1.10E-08	0.385443297
KBTBD4	12.71283247	2.53E-05	0.519177514
KBTBD6	12.6283594	0.000833152	0.591783802
KBTBD7	12.38018797	7.76E-08	0.413151645
KCNA6	0	0.004606615	1.557019068
KCNA7	0.050561721	0.000317142	1.729254863
KCNAB1	10.4210046	0.002634633	0.606584999
KCNAB3	1.578412238	6.98E-05	1.814843846
KCNC1	0.075791668	0.003504753	1.606267894
KCND1	2.335566824	6.00E-07	2.102494234
KCND2	0.609042771	0.001463145	1.745846762
KCNE2	1.553007429	0.002854965	1.573375865
KCNE5	1.875149854	1.66E-11	17.28650822
KCNG1	0.611388126	5.81E-05	1.901687673
KCNH1	0.0912117	1.87E-05	1.970997962
KCNH1-IT1	0.081978801	0.000114709	1.86619187
KCNH3	0.736308731	1.67E-06	2.125341104
KCNH5	0.019809248	0.000205705	1.844024608
KCNIP2	2.618114409	1.40E-05	1.909527742
KCNIP3	7.362518784	0.001314558	1.677126124
KCNJ14	1.714231565	1.93E-05	1.896022789
KCNJ15	62.78742718	1.99E-07	0.427237302
KCNJ16	123.3271528	0.000337461	0.562293527
KCNJ6	0.044043679	4.52E-05	1.950433881
KCNK15-AS	0.454229908	5.76E-05	1.848181488
KCNK17	2.523766796	0.002150358	1.78379268
KCNK5	42.05861803	2.11E-06	0.460756634
KCNMA1	14.74124213	0.001256229	0.583586141
KCNMB2-A	0.479511575	1.28E-12	2.912317617
KCNMB3	0.864699087	0.000261776	1.731385016
KCNN3	11.48821199	1.26E-06	0.42778257
KCNN4	3.848234361	9.42E-07	2.098952318
KCNQ1-AS1	0.134897649	0.002189806	1.591630038
KCNQ4	1.547552595	0.003497268	1.618811409
KCNS1	3.272648625	2.26E-05	2.152465211
KCNT1	0.14256217	0.002751734	1.584802672
KCNT2	2.594414579	0.001175974	1.633160339
KCNV2	0.301254525	0.000519754	1.687041437
KCTD13	12.87312024	6.40E-05	1.825516643
KCTD14	16.30227989	0.002159367	0.621695702
KCTD16	2.413710337	0.002657108	0.573394259
KCTD17	18.19865721	8.02E-05	1.810517123
KCTD18	17.04479882	8.93E-05	0.548974806
KCTD20	62.98864986	0.004652966	0.64699508
KCTD9	45.03427961	0.000948811	0.597721735
KDELR3	48.19088228	1.52E-07	2.187236479
KDF1	8.545509042	0.000123876	0.490882403
KDM1B	23.35908344	0.003337839	0.629818061
KDM3B	60.15762296	2.13E-05	0.503793375
KDM4A-AS	1.506316936	1.23E-06	2.054715144
KDM5B	20.46825592	0.000873273	0.589202444
KDR	187.202119	1.62E-05	0.493945568
KDSR	47.02174239	2.79E-05	0.513718974
KF456478.1	0	0.004606615	1.557019068
KF459542.1	0	0.004606615	1.557019068
KHDC1	1.666122797	0.001622595	1.607871206
KHDC3L	0.070826895	0.002104708	1.615613735
KHDC4	30.71882737	0.000186368	1.74680245
KHDRBS1	198.4208136	9.11E-05	0.545383439
KHDRBS2	0.181389894	0.003035112	1.572591432
KHDRBS3	8.477098695	0.000627036	0.577201472
KHK	196.9713449	5.64E-05	0.516985509
KIAA0232	33.67080084	0.000382519	0.557361885
KIAA0319L	43.28220781	0.000185456	0.553097844
KIAA0355	31.56071625	0.000218768	0.551228518
KIAA0391	3.509884234	0.003025333	0.625825729
KIAA0586	5.887978319	0.001436148	0.601221463
KIAA0825	1.547188201	5.62E-08	0.400288588
KIAA1109	19.05782137	9.34E-05	0.52261408
KIAA1143	21.19051852	1.28E-06	0.446338036
KIAA1147	58.46684718	2.50E-05	0.484050713
KIAA1191	239.2794733	5.35E-06	0.487258919
KIAA1211L	13.33270005	0.000414125	0.542180626
KIAA1217	34.9313465	0.004791566	0.637786803
KIAA1324	1.730881773	5.57E-06	1.992344774
KIAA1549	9.104248788	5.58E-08	0.399738381
KIAA1614	4.768676919	0.000602382	0.579304297
KIAA1671	30.68825405	3.40E-07	0.437404785
KIAA1958	8.842363036	9.86E-06	0.481617871
KIAA2012	0.061169096	0.000749465	1.695176762
KIAA2012-A	0.057164229	0.000554796	1.69893978
KIDINS220	39.08375942	5.53E-07	0.441350422
KIF11	7.967172192	9.75E-05	1.801496351
KIF13A	20.61481855	1.67E-06	0.458286395
KIF13B	25.25747654	4.57E-06	0.400753053
KIF14	1.314490493	1.28E-06	2.074936431
KIF15	1.704338059	2.71E-06	2.026546342
KIF16B	18.27059592	0.000166634	0.549465567
KIF18A	2.505942845	8.53E-07	2.085930423
KIF18B	2.938403801	4.62E-14	2.984942487
KIF1B	19.37775787	2.16E-05	0.496964342
KIF1BP	43.63916102	0.001149958	0.60092519
KIF1C	66.20268867	0.000281985	0.556790608
KIF20A	8.203836173	4.76E-10	2.507764963
KIF21A	22.80844236	0.001952824	0.573151967
KIF23	3.711311672	1.26E-12	2.813554736
KIF26A	8.901987905	0.00123994	0.562918576
KIF2C	6.112245759	2.30E-08	2.309500859
KIF3B	84.18041851	3.27E-07	0.432764892
KIF4A	5.169924894	2.10E-08	2.299339759
KIF4CP	0.056292824	4.15E-07	2.138682358
KIF5A	0.423574372	0.001539904	1.685303547
KIF9-AS1	1.211318851	3.84E-06	2.000437105
KIFAP3	43.94060931	0.003746502	0.635213506
KIFC1	7.630773863	6.71E-08	2.229236306
KIFC2	11.54323195	0.00027937	1.726985572
KIR2DL4	1.628024611	0.001284678	1.631138888
KIR3DX1	0.151369993	0.001684069	1.611669911
KIRREL2	0.296635811	2.10E-05	2.021754649
KIRREL3	0.476301652	8.11E-07	2.46615334
KISS1	2.78903281	0.002651982	1.734834046
KITLG	29.54828576	6.57E-05	0.490775601
KIZ-AS1	0.144583166	0.000118737	1.778023662
KL	81.52214671	2.26E-07	0.381765625
KLC4	36.26817043	0.000356848	0.577063331
KLF11	46.76976032	3.81E-05	0.520692496
KLF12	14.34568437	8.84E-05	0.53375736
KLF13	56.64824729	7.51E-07	0.426383708
KLF17	0.07036244	4.35E-07	2.235591798
KLF2	82.65596376	0.002949342	0.609975872
KLF3	43.57625967	9.55E-05	0.535371499
KLF4	41.91658751	0.000240773	0.512302526
KLF6	324.5933102	6.48E-05	0.504081815
KLF7	29.5156116	2.99E-05	0.496553417
KLF9	130.1749843	5.41E-07	0.422134981
KLHDC2	32.6274702	0.000346003	0.555140209
KLHDC7B	4.565113558	0.001641173	1.692301124
KLHL11	4.363462608	0.00030503	0.550072542
KLHL12	53.36859736	0.004750419	0.644582317
KLHL15	8.726188171	0.000435719	0.56670516
KLHL17	7.646131473	1.51E-07	2.173931333
KLHL20	23.29199655	0.001086392	0.602024512
KLHL22	21.97982235	0.004916539	0.640918709
KLHL26	9.788612888	0.000353863	0.560293005
KLHL28	8.634851536	0.00287548	0.628162179
KLHL32	4.080590994	0.002655946	0.625846846
KLHL33	0.357264283	0.001192563	0.564421276
KLHL36	35.20780014	0.001199963	0.59785675
KLHL5	36.81907189	0.000156028	0.546781507
KLHL7	17.49516842	0.000171646	0.550426951
KLHL8	14.90806923	0.00032132	0.569979513
KLHL9	43.81283841	6.23E-08	0.420785441
KLK10	0.512963644	4.70E-06	2.167511949
KLK11	0.285172283	0.003414414	1.977147979
KLK12	0.058913742	0.000100397	1.914718629
KLK13	0.308581485	0.003585649	1.593178145
KLK14	0.743984025	7.64E-08	2.218768108
KLK8	0.125588702	3.10E-05	1.971352748
KLLN	1.992059707	0.003593397	0.629337794
KLRA1P	4.730895168	0.000252105	1.728062557

KLRC2	0.396405935	0.001080955	1.646539854
KLRC4	0.707349131	0.00279635	1.589414635
KLRK1	1.373856322	0.000408744	1.717612429
KMT2A	18.95367319	0.000742137	0.58255141
KMT2B	19.93995374	0.002718651	1.573884719
KMT2C	17.99799781	0.000352257	0.553433516
KMT2E-AS1	23.5299229	0.004602212	1.545967429
KMT5A	17.814653	0.000252424	1.738067564
KMT5B	26.00318438	0.001670791	0.612684202
KMT5C	6.461920627	7.32E-07	2.081633977
KNL1	1.950348468	0.000750788	1.670704801
KNOP1P1	0.012019791	0.000770574	2.138883622
KNSTRN	8.622882496	0.001243557	1.632413552
KNTC1	6.771202069	2.26E-05	1.883725692
KPNA2	46.21161895	0.000901359	1.662095589
KPNA2P3	0	0.004606615	1.557019068
KPNA6	44.84693411	0.002447867	0.625102191
KPNA7	0.357799286	0.000427151	1.712956239
KPTN	10.94401702	5.49E-05	1.834203798
KREMEN1	0	0.004606615	1.557019068
KREMEN2	0.693198704	6.70E-08	2.320821029
KRT1	0.381902019	0.000287591	0.513458552
KRT10	30.6569034	0.000154628	1.767838354
KRT13	1.920942786	0.000115295	2.197938615
KRT15	0.539955863	1.74E-06	2.130929847
KRT16	0.601149632	0.000487098	1.839333646
KRT17P1	0.032103976	0.000319403	1.795397034
KRT17P3	0.160182656	0.002298064	2.135957055
KRT17P5	0.01199138	0.003031899	1.593458553
KRT17P6	0.016769309	0.004689342	1.685558959
KRT18P12	0.028417241	0.001589086	1.650125853
KRT18P14	0.043204255	0.001315862	0.596128253
KRT18P25	0.123352476	1.64E-05	1.915505824
KRT18P4	0.890655798	2.00E-05	1.892161835
KRT18P40	0.070983387	0.000562338	1.702167902
KRT18P47	0.050773131	0.000414552	1.754729347
KRT18P5	0.565514528	0.001325704	1.617451654
KRT19P2	0.104716546	0.004028065	1.594915556
KRT20	3.687714064	0.004257532	2.862518769
KRT26	0.012391291	3.95E-05	1.981056186
KRT34	0.287541915	1.77E-10	3.67215465
KRT38	0.01108144	8.18E-05	2.053931657
KRT39	0.106523381	0.000111363	1.90990188
KRT4	0.218523878	5.11E-05	1.991765009
KRT42P	0.05175109	0.000504656	1.718200017
KRT5	1.371524052	0.000391217	1.849256226
KRT6A	0.800507644	1.46E-05	2.108670226
KRT6B	0.265667037	3.01E-05	2.063937548
KRT75	0.057714983	0.000430103	1.887896725
KRT78	0.086738721	4.50E-11	2.73534754
KRT79	0.141747382	4.80E-08	2.777502675
KRT89P	0.054559627	0.001206647	1.650058447
KRT8P21	0.072549171	0.003927817	1.605865857
KRT8P23	0.126571081	0.000176319	1.795692189
KRT8P30	0.216695785	0.000741587	1.704955127
KRT8P39	0.623391951	0.004401202	1.539875385
KRT8P40	0.140880963	0.001802146	1.612308398
KRT8P46	0.967679591	0.002516115	0.611670896
KRT8P50	0.085416214	1.47E-08	2.315050255
KRTAP1-1	0.078503961	0.000687416	1.810355341
KRTAP10-1	0.061065648	0.000228934	1.860570511
KRTAP16-1	0.098046749	5.59E-05	1.861993648
KRTAP2-3	0.407149074	5.13E-09	2.586496689
KRTAP4-8	0.005055994	0.002295636	2.179429343
KRTAP5-1	0.805759033	0.000116104	1.878755135
KRTAP5-4	0.075374059	0.001583559	1.705420923
KRTAP5-6	0.143374942	0.00447142	1.581806152
KRTAP8-3P	0.036051169	0.000262579	2.648290683
KRTCAP2	19.49020052	3.65E-07	2.132812729
KSR1	45.60100134	0.002836707	0.62071372
KTN1	43.22649754	4.14E-05	0.504578473
KXD1	63.35085372	0.000681307	1.681501274
KYAT3	36.90729776	0.000260543	0.563198382
L1CAM	9.879692899	1.96E-06	2.436318746
L2HGDH	7.757690124	9.11E-05	0.518025754
L3MBTL1	2.908686854	0.000978625	1.639180842
LACC1	15.79313536	3.77E-05	0.517810674
LAGE3	37.10190325	0.001547331	1.614537285
LAIR2	3.362269098	0.002315354	1.857615775
LAMB2	256.0503596	1.12E-06	0.450699219
LAMP1	210.1211526	4.13E-05	0.515741978
LAMP5-AS1	0.0368537	0.001563839	1.624622185
LAMTOR2	103.0154135	6.65E-05	1.825345348
LAMTOR3	47.34230934	1.76E-08	0.408434074
LAMTOR5-	0.582205075	0.001760517	1.601818052
LANCL1	65.83315744	2.68E-06	0.470714645
LARS2	13.72560252	0.000126143	0.462075869
LARS2-AS1	0.139643311	1.69E-05	1.928220725
LAT	1.171751031	3.36E-06	1.994273165
LATS2	38.58189342	0.000196528	0.556799384
LATS2-AS1	0.144194231	9.86E-05	1.869096127
LBHD1	4.267801428	1.24E-07	2.209065708
LBP	117.9531773	5.86E-06	2.318991144
LBX1	0.030437748	9.89E-05	2.16362671
LBX1-AS1	0.018316188	3.17E-06	2.162760061
LBX2	2.175436607	1.65E-07	2.181420064
LCE3A	0.032766753	9.18E-05	2.274436114
LCLAT1	10.93935312	7.22E-05	0.526713666
LCMT1-AS1	0.711089263	0.000274018	1.72682228
LCMT2	8.011311897	8.12E-06	0.492013226
LCORL	5.035997423	0.000578007	0.570544835
LCTL	0.318006942	5.90E-07	2.119528579
LDAH	17.96655514	0.000240226	0.560220082
LDHAL6EP	0.30221322	0.000338849	1.722488313
LDHAP2	0.665468111	9.45E-05	1.795893388
LDHAP5	2.138816844	0.000354862	0.561201224
LDLRAD1	0.048103253	0.002832895	1.710828473
LDLRAD4	13.01467274	0.000319548	0.559661977
LEF1	12.20409264	0.002909831	1.611809382
LEF1-AS1	0.462355606	0.000794592	1.782146806
LEMD1	0.512033446	0.003620964	1.578467462
LEMD1-AS1	0.081800835	0.000131393	1.786298076
LEMD3	27.36317219	0.001291086	0.605719543
LENG8	105.9594043	0.00033747	1.708899031
LENG8-AS1	14.23056332	0.00102571	1.642516952
LENG9	7.057555631	1.30E-06	2.070786784
LEO1	33.54185455	0.000149347	0.554477915
LEPROT	154.0750704	0.000110378	0.538087034
LEPROTL1	92.11432044	3.03E-06	0.486253381
LGALS1	1061.518185	0.000108936	1.792505045
LGALS14	0.060955489	0.00043466	1.75552916
LGALS2	257.9710583	0.000503871	0.573808915
LGALS3BP	619.7057788	0.000152114	1.771084262
LGALS9	40.42927407	0.003774489	1.551102102
LGALSL	35.02546529	6.29E-05	0.520463785
LGR4	64.36912101	1.92E-05	0.495999081
LHB	0.546949437	1.03E-06	2.082584517
LHFPL5	0.138437894	3.92E-05	1.883563813
LHX2	0.920167737	1.59E-06	2.749451983
LHX3	0.048276434	5.64E-05	2.828456181
LHX5	0.02425299	0.00026091	1.846711708
LIF	39.72434442	5.98E-06	1.997515553
LIF-AS1	0.389489702	2.08E-10	2.548887604
LIFR	37.45083338	3.73E-05	0.511328042
LILRA4	2.896859255	0.001667654	0.568723413
LILRB3	4.464633137	0.000182669	1.755542419
LILRP2	0.2006693	1.51E-07	2.194304921
LIMCH1	36.40342108	1.51E-06	0.433254617
LIMD2	21.32137288	0.000859593	1.657241537
LIMK1	23.15211127	1.56E-05	1.913329773
LIN28AP2	0.021717865	0.000283859	2.176027785
LIN52	11.06313324	0.000350889	0.570873504
LIN54	12.00782895	7.04E-07	0.428725892
LIN7A	33.89791777	0.00018805	0.535546273
LIN7C	36.53090683	0.000847689	0.583117261
LINC-PINT	8.33130659	8.91E-07	2.07414464
LINC00106	11.90980136	2.19E-05	1.885128908
LINC00113	3.672132378	0.002951986	0.559335589
LINC00115	3.160096333	3.05E-06	1.999984921
LINC00174	8.000368019	1.68E-05	1.899876073
LINC00184	0.170007379	0.002495753	1.595294365
LINC00239	2.515788971	0.00088344	1.669930985
LINC00242	0.852960578	6.85E-05	1.840561098
LINC00244	0.391559513	0.001608511	1.621789056
LINC00261	0.16883784	4.27E-06	2.971182964
LINC00265	5.299047779	0.003486083	1.563148795
LINC00266-	0	0.004606615	1.557019068
LINC00271	1.14922921	0.001592316	0.596833362
LINC00280	0	0.004606615	1.557019068
LINC00283	0	0.004606615	1.557019068

LINC00293	0.013636351	0.003303269	1.658097551
LINC00298	0.207362588	0.001229034	1.654456798
LINC00299	0.659872571	1.76E-05	1.908154225
LINC00304	0.214210471	1.07E-05	1.989467323
LINC00313	0.123421749	0.000165936	1.824603787
LINC00326	0.010487048	7.00E-05	1.955413415
LINC00334	0.252369381	0.001083776	1.643874338
LINC00337	0.336535765	4.01E-05	1.855932746
LINC00342	13.55149608	1.84E-05	1.88885999
LINC00348	0.037351663	7.95E-06	2.091834313
LINC00355	0.060625712	0.002615245	1.797594818
LINC00364	0.110576133	0.002591454	1.658201326
LINC00382	0.020533885	0.000580086	1.839409936
LINC00385	0	0.004606615	1.557019068
LINC00388	0.066788743	0.002145446	1.821871774
LINC00460	3.933857229	1.58E-08	2.402194336
LINC00466	0.016085777	0.002170207	1.665974605
LINC00472	4.874594864	0.004733762	0.595497494
LINC00477	0.044736758	0.004851044	1.577983983
LINC00504	0.478999616	0.000829388	0.566565311
LINC00511	1.707793981	0.000241358	1.78456889
LINC00520	0.108090493	5.03E-07	2.236905502
LINC00524	0.822136015	2.22E-07	2.445562307
LINC00525	0.541175076	4.87E-05	1.867107285
LINC00526	8.549453532	0.001914301	0.610793507
LINC00536	0.028203453	5.74E-07	2.142574118
LINC00565	0.511239846	2.78E-06	2.027995227
LINC00566	0.095755691	0.002459258	1.617190478
LINC00592	0.149855088	0.00022356	1.767480461
LINC00601	0.17216408	1.12E-06	2.4614885
LINC00605	2.660448107	4.67E-06	0.441599365
LINC00606	0.014299317	0.004721346	1.602215418
LINC00607	0.398238261	2.93E-05	1.874276833
LINC00608	0.019469507	0.004002895	1.572541037
LINC00618	0.345716938	0.000550785	1.688096107
LINC00623	6.796030095	6.44E-05	1.823355714
LINC00624	0.315461621	0.003019112	1.570232015
LINC00626	8.527610204	0.004794249	1.725262506
LINC00632	0.297004766	0.000257623	1.779443085
LINC00634	0.204494169	9.39E-07	2.10372089
LINC00648	0.206280971	0.001490033	1.769025494
LINC00652	0.235173554	4.57E-06	1.98184173
LINC00653	4.223088884	4.44E-05	1.841253015
LINC00658	0.026932173	0.000608005	1.691888215
LINC00661	0.031463036	0.000121145	1.859311281
LINC00664	0.596070262	1.09E-05	2.026127855
LINC00668	0.139432117	0.002490311	1.964897374
LINC00674	20.74904266	0.00030425	0.567522327
LINC00677	0.687043658	0.00244831	1.578039762
LINC00685	5.758249224	1.82E-06	2.031583856
LINC00691	0.020372567	0.002719584	1.616865444
LINC00705	0.132709792	0.004364166	1.600596
LINC00824	0.061877181	0.000158623	1.789998317
LINC00836	0.131245839	0.001033497	2.106723055
LINC00837	0.028116056	0.000543961	1.738341982
LINC00839	28.47525014	0.00182301	1.603646345
LINC00845	0.282716664	4.72E-05	0.481120942
LINC00862	0.611713017	0.004366447	1.615276003
LINC00868	0.080102326	2.69E-07	2.184547171
LINC00869	2.054884314	1.50E-05	1.923902461
LINC00881	0.86275189	0.002714891	1.617805703
LINC00893	3.433486787	0.000145036	1.764903548
LINC00894	2.209629131	0.000395391	1.702055775
LINC00895	0.05869475	0.000290672	1.785909382
LINC00896	0.419404339	7.89E-10	2.56488532
LINC00900	1.706247878	0.004662792	1.538991988
LINC00909	12.03797237	0.002853203	0.627195954
LINC00922	0.617192106	1.18E-08	4.28991629
LINC00926	2.430824425	9.65E-05	1.796113904
LINC00928	0.137864111	6.00E-05	1.909081503
LINC00930	0.265987692	3.74E-07	2.228332765
LINC00937	0.470097424	0.000136633	1.784741998
LINC00941	1.144787365	5.70E-09	2.489362219
LINC00942	2.317790334	2.28E-05	2.364866149
LINC00943	0.541191489	1.07E-05	1.931000186
LINC00944	4.761371633	0.00019597	1.75304748
LINC00945	0.139017135	0.002613735	1.629408329
LINC00954	0.735172785	0.000969486	1.664803712
LINC00967	0.038371412	0.000347419	1.762466039
LINC00971	0.011656206	7.32E-05	1.87787508
LINC00973	1.373161881	8.05E-11	3.702270797
LINC00993	0.102499026	5.80E-05	1.948659964
LINC00996	0.950980467	0.002021674	1.600586171
LINC00997	8.513883637	3.30E-06	2.006241252
LINC01004	7.213420692	3.38E-07	2.125953057
LINC01011	6.656936102	0.00269917	1.571798306
LINC01016	0.133008388	8.54E-06	1.983673095
LINC01020	0.124547696	0.004449223	0.534296432
LINC01049	0.011821702	0.004225219	1.974705833
LINC01050	0.087523538	0.000296638	1.817548234
LINC01053	0.077448185	0.002058476	1.661890434
LINC01060	2.611367157	9.60E-05	0.489212886
LINC01066	0.016273427	0.000471536	2.243406561
LINC01078	0.051530882	0.000406966	1.940489152
LINC01081	0.190939329	0.004079739	0.584252723
LINC01085	0.175616437	2.52E-05	1.946794055
LINC01089	8.756126883	0.000169391	1.753939056
LINC01093	0.275316946	0.000318241	1.78577622
LINC01098	0.142655	0.000733808	1.802710275
LINC01102	0.15100708	0.003013168	1.593887198
LINC01117	8.051670226	0.001660505	0.613851543
LINC01118	0.176764439	0.002301056	1.586655507
LINC01119	0.687053488	0.000332673	1.7193158
LINC01121	0.109670487	2.77E-07	2.240780159
LINC01132	2.001928537	8.86E-06	0.460436149
LINC01133	0.409260036	0.002836623	1.636051863
LINC01134	0.924095574	0.000871897	1.658838661
LINC01138	5.844092478	7.11E-05	1.817494797
LINC01141	0.343448753	2.51E-05	1.884001568
LINC01151	2.348058397	0.001064031	1.879041417
LINC01153	0.011548695	0.000134501	1.914030463
LINC01160	0.975002067	9.26E-06	1.933216014
LINC01169	0.185033378	0.000264176	1.771022178
LINC01176	9.138054729	0.002086963	1.591606744
LINC01185	0.073057623	6.95E-08	2.237573974
LINC01191	0.477420745	5.88E-09	2.396162172
LINC01192	0.037065388	9.92E-07	2.175948445
LINC01194	0.032498194	0.000224795	1.83297113
LINC01203	0.033503213	0.001419843	1.659687911
LINC01204	0.031269647	1.38E-05	2.161869887
LINC01206	0.011054399	0.000251117	1.783107134
LINC01208	0.089434616	0.001783304	1.655386097
LINC01232	1.703963148	0.000567735	1.685683502
LINC01234	2.095850091	2.13E-08	2.458946244
LINC01238	0.902665668	0.000541397	1.678924581
LINC01241	0.029967419	7.69E-05	1.871103802
LINC01250	0.04335679	4.49E-05	1.855429336
LINC01251	0.346982732	3.26E-07	2.164737566
LINC01267	0.154660819	0.002735406	1.683942416
LINC01270	0.952572684	5.11E-08	2.242381782
LINC01271	0.435233523	3.68E-07	2.125552736
LINC01276	0.031040412	0.000626867	1.727600383
LINC01282	0.032846754	0.000897044	1.707713914
LINC01289	0.015982997	2.18E-05	1.940506555
LINC01293	0.233929186	4.94E-06	1.992456968
LINC01297	0.016104391	0.000823177	1.734456225
LINC01299	0.025768808	0.002430194	1.62686873
LINC01301	0.502982784	5.51E-07	2.131187421
LINC01303	0.396070475	4.58E-07	2.122900499
LINC01305	0.050456286	1.37E-09	2.492037967
LINC01311	2.05704633	2.67E-05	1.870119195
LINC01341	1.054168001	0.003671798	1.561225679
LINC01354	1.359185321	0.003461389	0.568275512
LINC01355	4.706734804	6.10E-06	1.960441309
LINC01389	2.13665756	7.21E-08	2.21785806
LINC01393	0.497348599	0.00063915	1.685565843
LINC01397	0.29863758	4.28E-05	1.871248236
LINC01405	0.10178146	0.000180941	1.803712803
LINC01419	0.129674267	0.000124943	4.301975941
LINC01422	0.406085571	0.00178831	1.599602386
LINC01424	0.571971794	0.000138129	1.77629164
LINC01429	0.063114948	1.40E-06	2.23780793
LINC01431	1.618091305	0.000943547	1.654152518
LINC01432	0.015411602	0.000986439	2.145255634
LINC01436	6.241621506	2.27E-06	2.200359833
LINC01447	0.059590783	0.001448675	1.670097879
LINC01456	0.067202058	6.82E-07	2.255134559
LINC01460	0.169009205	3.58E-06	2.137078441
LINC01470	0.128683502	0.001291504	1.642048203
LINC01480	2.827772022	6.67E-05	1.828574868

LINC01481	0.651056932	0.001450017	1.620954673
LINC01482	0.148755531	1.15E-11	2.692792355
LINC01486	0.089569322	0.003026866	1.598576604
LINC01492	0.02886393	4.60E-06	2.053851104
LINC01496	0.060496859	0.0006697	1.772787812
LINC01507	12.55840772	6.51E-07	0.445498169
LINC01518	0.194758476	8.20E-07	2.22635613
LINC01520	0.028791009	0.002913644	1.659018845
LINC01521	2.641054207	0.002666061	0.610372631
LINC01522	1.315686776	1.40E-05	1.993488686
LINC01527	0.011955184	0.00035807	1.831334485
LINC01531	0.137880083	0.000973034	1.750942938
LINC01548	0.067519629	0.004150865	1.633860258
LINC01549	0.052845265	5.32E-05	1.918923818
LINC01550	4.84803393	1.52E-07	0.430236303
LINC01551	0.084503221	2.14E-07	2.224020673
LINC01563	0.306967739	0.000819852	1.876722927
LINC01582	0.014786664	0.003516592	1.900454809
LINC01583	0.234849499	0.000124126	2.06702288
LINC01585	0.188683397	1.65E-05	1.902179974
LINC01588	0.986798362	2.65E-06	2.033268153
LINC01589	0.797578564	0.002439272	1.617225057
LINC01591	0.024729785	0.003802357	1.647285757
LINC01605	0.889322976	1.04E-14	3.287852079
LINC01615	3.48536422	3.10E-08	2.363153256
LINC01619	1.314866769	0.004655661	1.546941008
LINC01630	0.027107288	0.0001899	1.813087063
LINC01634	0.061113566	2.51E-06	2.06835096
LINC01655	0.483638272	2.78E-08	2.396253988
LINC01664	0.217848464	0.002151478	1.594355712
LINC01667	0.117829873	0.000953428	1.991355981
LINC01671	71.01148143	0.00020265	0.55632118
LINC01673	0.113213469	1.90E-06	3.246722285
LINC01675	0.313452798	0.000482437	1.703919643
LINC01678	0.89803201	0.002157932	1.595247518
LINC01681	0.053406124	0.003515996	1.656058302
LINC01686	0.864251991	7.07E-06	1.957315747
LINC01694	0.212225673	3.28E-07	2.343531095
LINC01705	1.921016897	9.24E-10	2.824848803
LINC01711	1.737665286	9.28E-07	2.389293481
LINC01730	1.105734483	1.73E-07	2.181599221
LINC01731	0.302222332	0.000440812	1.766165779
LINC01735	0.061594886	0.003995098	1.768070347
LINC01748	0.148783756	0.001628517	1.685352758
LINC01776	0.037180469	5.41E-10	3.112583526
LINC01783	0.292794627	0.000424829	0.503816152
LINC01788	1.905827942	0.000553425	0.475799014
LINC01789	9.72255741	0.00184028	0.57942476
LINC01792	0.03984061	0.001359813	1.640712352
LINC01793	0.036046244	3.45E-07	2.202967662
LINC01795	0.072028523	0.003060071	1.782754306
LINC01801	2.495666574	3.32E-05	0.474674606
LINC01804	0.048760383	0.003892972	1.596888255
LINC01807	0.110883064	0.001123856	1.860677075
LINC01812	0.172848578	0.000444976	1.863320989
LINC01822	0.07408156	0.000904633	0.483217817
LINC01828	0.052737458	0.001900657	1.605341327
LINC01829	0.329845402	3.29E-05	1.867794487
LINC01838	0.045541508	0.002263708	1.611166431
LINC01841	0.437562808	0.000996259	1.648005631
LINC01843	12.49461347	4.56E-06	0.460268274
LINC01849	0.036024357	0.00080644	1.864066212
LINC01862	0.09647833	0.000723498	1.753550143
LINC01871	10.05082455	0.001278691	1.628161484
LINC01879	0.032967733	0.00085078	1.703496133
LINC01885	0.599352492	7.30E-05	0.483364516
LINC01886	6.98405521	1.80E-05	0.481501254
LINC01898	0.021479439	6.36E-05	1.96069861
LINC01900	0.066507893	2.04E-05	1.924078866
LINC01910	0.056948664	6.39E-05	1.851166338
LINC01912	0.019667079	0.000626056	2.112440195
LINC01913	0.0245878	0.00025286	1.795126095
LINC01914	1.439580711	3.08E-10	2.607276073
LINC01919	0.074224219	0.001113967	1.666224417
LINC01920	0.030827958	0.000119794	1.942826789
LINC01923	0.011596366	0.002017814	1.673383719
LINC01926	0.079313992	0.000949916	1.666127639
LINC01929	2.121994364	0.00013542	2.014121576
LINC01932	0.075332971	0.004432713	1.548066844
LINC01933	0.181569889	3.46E-05	1.880538335
LINC01938	0.046868849	7.54E-06	2.004518181
LINC01940	0.030239282	0.000483669	1.780995139
LINC01943	3.162793074	0.00019037	1.756188865
LINC01950	0.204779617	0.002071925	1.731248791
LINC01952	0.462162481	0.003189359	1.57413366
LINC01956	0.620285002	1.47E-07	2.332460998
LINC01961	0.036173793	9.05E-05	1.905107399
LINC01970	0.243872976	2.32E-08	2.285032057
LINC01971	0.071106383	0.002195951	1.618257835
LINC01972	0.117261594	0.000814665	1.672204961
LINC01973	0.05541315	5.72E-08	2.250775302
LINC01977	0.311713109	6.26E-08	2.333285794
LINC01989	0.100752788	7.92E-06	1.967296354
LINC01993	0.145442898	6.52E-07	2.154288106
LINC01994	0.016840384	0.001165816	1.706986889
LINC01999	0.15223579	0.004650205	1.559090312
LINC02012	0.370083466	0.004871325	1.575100441
LINC02020	0.018607578	1.18E-06	2.320312253
LINC02026	0.23368857	2.26E-05	1.888711048
LINC02027	6.2147255	5.46E-07	0.416599433
LINC02031	0.060300153	3.08E-05	2.013391414
LINC02043	0.132174365	0.002189212	1.636032148
LINC02057	0.111991535	0.004604284	1.681142331
LINC02061	3.095674335	7.73E-05	0.451604678
LINC02062	3.690629996	7.87E-06	1.947890498
LINC02064	0.128683392	0.000508326	1.788374985
LINC02067	0.165736738	2.88E-05	1.885389537
LINC02073	0.73162262	0.004727807	0.595582558
LINC02075	0.027694999	0.004057162	1.581765582
LINC02076	0.849611647	1.08E-05	1.940670887
LINC02078	0.094083316	0.002111632	1.615511226
LINC02080	0.189289633	1.93E-09	2.411831918
LINC02085	0.013584626	0.002581329	1.655414655
LINC02086	3.996590195	0.00016675	1.771285203
LINC02087	0.045395265	0.002043202	1.632911032
LINC02092	0.026788014	0.000863967	1.723779433
LINC02094	0.126446114	0.003031989	1.598042061
LINC02100	3.426675529	3.11E-07	2.147329015
LINC02102	0.1965619	7.27E-05	1.807437334
LINC02105	0.037292288	0.003549022	1.571901111
LINC02112	0.05846088	1.24E-05	1.943977114
LINC02115	0.663463862	1.42E-07	2.307689215
LINC02117	0.048167398	0.000113206	1.917622507
LINC02126	0.277950046	5.95E-05	1.8625245
LINC02129	0.049456793	0.000257464	1.809442615
LINC02135	0.03924838	4.49E-05	1.935186998
LINC02152	0.14582816	0.001057133	1.755995837
LINC02154	1.447812426	0.000202804	2.074185387
LINC02156	0.069490352	0.0002943	1.751245873
LINC02163	0.153449258	0.00162175	1.801213184
LINC02165	0.068104058	0.000101129	1.929389162
LINC02195	1.21920903	0.00061196	1.69222233
LINC02199	0.031773316	1.58E-06	2.091191325
LINC02208	0.098686248	6.76E-05	1.846810982
LINC02216	0.134370947	2.71E-05	1.951542304
LINC02219	0.137056434	3.34E-06	2.052519852
LINC02252	0.040600337	0.000960721	1.816577317
LINC02257	0.435071733	1.72E-06	2.29748514
LINC02262	0.029075611	1.24E-07	2.456904436
LINC02265	0.097321445	0.000816997	1.682950525
LINC02267	0.020048096	0.000623259	1.712688311
LINC02268	0.370841142	3.82E-09	2.578497298
LINC02269	0.841063331	0.000742411	1.887368398
LINC02275	4.160042627	0.001541485	0.601831043
LINC02278	0.204396832	3.62E-05	0.458522033
LINC02280	0.151712803	0.000860125	1.654294417
LINC02286	0.052185158	1.11E-05	2.328894097
LINC02287	0.047460049	0.001565466	1.623403606
LINC02296	0.128242811	0.00060142	1.858101744
LINC02314	0.062144352	0.001844013	1.731927385
LINC02320	0.171610402	0.000175635	1.792418433
LINC02323	0.151817504	0.00037026	1.80237186
LINC02324	0.033300487	4.37E-05	1.856510784
LINC02328	2.288083495	2.62E-06	2.018158951
LINC02340	0.663374305	0.000787972	1.66466615
LINC02345	0.380525478	0.001233699	1.634212176
LINC02348	34.74900175	0.001950238	0.572940168
LINC02352	1.259673437	2.70E-07	2.141175106
LINC02362	0.955194376	0.000947619	1.714848798
LINC02367	0.29360763	9.59E-05	1.805164455

LINC02368	0.007409725	0.004724301	1.883636756
LINC02373	0.295429688	0.001743993	1.611085522
LINC02376	0.156319547	6.98E-11	3.274979936
LINC02378	0.060652785	4.07E-07	2.152724853
LINC02385	0.084503404	3.66E-05	1.872418247
LINC02388	0.056527367	0.001766699	1.690518731
LINC02389	0.053230109	3.26E-06	2.001415571
LINC02390	0.412581661	0.002387533	1.584359269
LINC02394	0.049045547	0.004909058	1.696719767
LINC02396	0.008931267	0.000828598	1.815776495
LINC02402	0.013486304	0.002324771	1.822777846
LINC02409	0.109488619	0.003221187	0.572996758
LINC02415	0	0.004606615	1.557019068
LINC02418	0.037104649	0.002186852	1.683907075
LINC02421	0.149956829	2.44E-05	1.875715511
LINC02422	0.570524095	3.62E-06	2.01498601
LINC02426	0.037886564	0.000411626	1.767537797
LINC02428	0.128672994	0.004144781	1.838230223
LINC02438	0.161418229	0.00014985	1.897852282
LINC02441	0.198759411	7.44E-05	1.896021304
LINC02444	0.028177825	0.000741127	1.718296435
LINC02446	2.663301673	0.000147457	1.805266273
LINC02450	0.549104049	0.000118292	1.864081918
LINC02454	0.386232551	1.14E-07	2.312079272
LINC02455	0.114889151	0.000440718	1.717829018
LINC02458	0.141687896	0.002341006	1.596402172
LINC02461	0.243116435	2.60E-08	2.526958849
LINC02464	0.057191222	3.23E-05	2.119325853
LINC02475	0.267625439	0.003319255	1.801762696
LINC02476	0.044648188	6.00E-05	1.975666022
LINC02485	0.096792812	0.000370753	1.761659027
LINC02507	0.176007628	3.37E-05	1.867809021
LINC02511	2.591974292	0.003579004	1.840757398
LINC02513	0.363913548	4.97E-07	2.218997176
LINC02518	0.106911014	0.000463042	1.795061645
LINC02526	4.511678592	6.64E-06	1.977032544
LINC02538	0.718890756	0.004476876	0.593667125
LINC02539	0.175046972	0.000165933	1.80958548
LINC02541	1.070439567	0.002744355	1.595617733
LINC02544	1.315294285	2.66E-06	2.058728568
LINC02551	0.143219948	5.18E-09	2.364699876
LINC02560	0.291058392	7.45E-05	1.834766898
LINC02565	0.037291691	7.57E-05	2.325901296
LINC02569	0.950945614	0.001947488	0.608374479
LINC02570	0.516161818	1.58E-05	2.056131616
LINC02577	2.052636319	0.002190808	1.685442872
LINC02601	0.501673664	0.003014008	1.572810515
LINC02603	0.449898912	0.003765924	1.554284848
LINC02604	11.41525815	2.70E-10	2.523682307
LINC02605	0.941986552	0.000462842	1.698133356
LINC02609	3.768509442	2.27E-08	2.304884823
LINC02611	2.751971835	0.001609611	1.611798239
LINC02612	0.018239222	2.43E-06	2.211862456
LINC02614	1.528472807	0.00291881	1.566468368
LINC02615	6.390649487	0.001984485	1.603975912
LINC02624	0.02245713	0.000375979	1.762511912
LINC02625	1.23919074	0.001472252	0.562004681
LINC02626	0.507952985	0.000973856	1.655526015
LINC02628	0.047088006	0.001157715	1.727176814
LINC02631	0.213506629	0.003211235	1.569276611
LINC02641	0.402903226	7.44E-06	1.987175772
LINC02655	0.128630368	0.000970824	1.686692101
LINC02657	1.301828457	2.40E-07	2.20597488
LINC02662	0.027956579	0.001453916	1.889135111
LINC02663	0.206350314	8.46E-05	1.858404515
LINC02674	0.6612128	3.54E-05	0.45389212
LIPA	191.0220172	0.00334613	0.620184538
LIPE	4.882856172	0.00263766	0.6098928
LIPT2	9.146258906	0.0044337	0.646604369
LIX1-AS1	0.170349084	0.000990288	1.656491002
LIX1L-AS1	1.016881149	0.000953631	1.641698346
LMBR1L	23.96483963	0.000987491	1.635518862
LMBRD1	186.3330656	1.05E-06	0.464099189
LMBRD2	18.32722497	1.01E-05	0.484209743
LMNB1	20.69594044	0.002188626	1.590532203
LMNTD1	0.02888665	0.000440063	1.767948673
LMNTD2	10.5404376	0.000420863	1.701383756
LMO7	20.05308471	3.82E-05	0.498749121
LMO7DN	0.106311816	0.001095551	1.664140612
LMTK2	27.01994887	0.000113397	0.541675679
LMX1B	0.423527524	0.002423339	1.701729503
LNCOG	0.342655747	6.68E-07	2.133336431
LNX1	10.50005767	0.000592358	0.560522381
LNX2	22.16201578	4.36E-07	0.447836007
LONP2	39.25991922	0.001069065	0.593198394
LONRF2P2	0.009388356	0.004770245	2.152669928
LONRF3	4.786877467	2.39E-05	0.474865899
LOXL1	16.27819743	5.87E-05	1.852856021
LOXL1-AS1	4.705834781	0.000691784	1.670876133
LPA	0.168806161	0.004158858	0.488671062
LPAL2	1.18687207	0.00017767	1.758694784
LPAR2	15.49572446	1.85E-07	2.187803685
LPGAT1-AS	0.040459285	0.001135207	1.641547933
LPIN2	43.70884022	0.002561126	0.612132317
LPIN3	43.3580012	0.002551816	1.57690153
LPO	0.064182788	1.16E-12	2.835853111
LPP	32.67275188	0.0001474	0.544087927
LPP-AS1	0.15106161	0.002278861	1.65975284
LRBA	24.95407992	1.58E-07	0.419121194
LRCH1	28.41699328	0.000863983	0.594394472
LRFN1	2.57461008	1.67E-08	2.343939138
LRP1-AS	0.243113813	2.11E-06	2.039418808
LRP2	150.5891333	1.80E-06	0.433553869
LRP4	8.042751397	0.000685102	0.552081373
LRP5	63.76764619	5.42E-11	0.322083135
LRP6	20.05046206	0.001078856	0.58624375
LRP8	1.705936811	2.69E-10	2.53086162
LRPPRC	71.41707123	4.47E-07	0.440476011
LRRC1	13.5251904	0.000293737	0.549307481
LRRC15	3.054985793	0.002893593	1.832146322
LRRC19	19.71058081	3.41E-05	0.475922159
LRRC28	8.328007139	7.00E-05	0.532177597
LRRC3-DT	0.103490494	5.18E-05	1.889337357
LRRC31	1.319810329	0.004022806	0.542599229
LRRC34P1	0.038898963	0.000256588	1.820160583
LRRC37A1[illegible]	0.107681264	0.00337114	1.602121334
LRRC37A1[illegible]	10.81571102	0.000298808	0.563538001
LRRC37A1[illegible]	6.406840867	0.003345761	0.625595379
LRRC37A3	4.385350626	0.004808051	1.531983667
LRRC37A7[illegible]	10.86730081	4.53E-05	0.492654549
LRRC45	17.95935276	0.001638658	1.612008562
LRRC46	1.984914817	0.001273499	1.638268782
LRRC47	46.41363298	0.000215761	0.564835229
LRRC49	3.751554484	0.003534361	0.631531406
LRRC55	0.649131996	0.001226095	0.576863793
LRRC58	34.90799689	0.004428606	0.637890166
LRRC69	0.86409001	3.59E-07	2.122510702
LRRC73	4.168975687	0.001625066	1.618497267
LRRC74B	0.042320277	9.14E-07	2.14833404
LRRC77P	0.60999573	0.001850866	0.565868245
LRRC8A	78.10289499	3.03E-07	0.430224882
LRRC8C	14.42939507	0.002605983	0.617982676
LRRD1	0.05181401	0.001109027	1.64036407
LRRK2	55.74884761	0.001650922	0.602551134
LRTM1	0.070081395	6.52E-05	1.916068942
LRTM2	0.020134165	2.90E-05	1.907189933
LSAMP-AS1	0.090960627	0.000106152	1.975281853
LSM10	47.82509085	0.00020781	1.75710465
LSM14A	134.2579249	0.000154028	0.554892081
LSM2	93.60656927	0.001565361	1.624571848
LSM5	24.1778479	0.00475283	1.534048427
LSM7	53.42470399	4.86E-07	2.119252462
LSM8	12.36205133	0.001271397	1.626431517
LSMEM1	3.795347558	0.002646125	1.580297412
LTA4H	70.0069241	5.23E-05	0.535672661
LTB4R	13.1573565	1.13E-06	2.065147091
LTB4R2	3.45032272	0.000175588	1.755643395
LUARIS	0.141050736	1.30E-05	1.990044313
LUC7L	23.85899599	3.23E-06	1.998647167
LUC7L3	54.79676868	0.000552391	1.678348372
LUCAT1	6.063964246	6.91E-05	1.865182916
LURAP1L	34.47007224	0.002135739	0.600241786
LUZP1	27.0595848	7.62E-06	0.479280922
LUZP4	0.022749834	7.73E-05	1.932592264
LUZP4P1	0.102710987	1.05E-05	1.998253042
LUZP6	0	0.004606615	1.557019068
LY6D	0.224541744	0.004171762	1.65409304
LY6G5B	4.17638933	0.001505631	1.610113274
LY6G6C	0.232981084	0.000303503	1.73938206
LY96	55.05252383	5.39E-05	1.844540045

LYG2	0.674282906	8.26E-10	2.466180133
LYPD3	1.915429804	0.00109561	1.66406325
LYPD4	0.123666172	0.0001025	1.905271402
LYPD5	1.882220575	0.004909598	0.640268898
LYPD6	0.713493948	8.95E-05	2.07998173
LYPLAL1-D	0.973158686	0.001303808	0.591352538
LYRM7	19.8465349	6.50E-07	0.4492437
LYSMD3	38.46871733	1.22E-05	0.496635932
LZIC	31.01538818	4.82E-07	0.445204974
LZTFL1	13.92782897	0.003071591	0.627358068
LZTS2	42.81522878	0.000136874	1.777060992
MACF1	27.80850526	2.16E-05	0.483693722
MACO1	43.38449646	4.16E-07	0.444917247
MACROD2	3.862079187	0.004429422	1.641898877
MAD1L1	13.52692501	0.002602433	1.579349134
MAD2L2	21.79225333	0.001103445	1.63921167
MADCAM1	0.710837431	1.49E-07	2.183093652
MAF	122.4806444	0.001766093	0.598429092
MAFA-AS1	1.156583961	0.000521387	1.807869997
MAFG-DT	3.848202206	3.31E-05	1.874513643
MAGEA10	0.359065664	0.00239628	2.589513677
MAGEA11	0.119703244	0.000267232	3.697345601
MAGEA12	0.529603894	0.000725363	2.31143558
MAGEA4	0.192424869	0.000368239	2.883526883
MAGEA4-A	0.036885774	0.004466486	2.074507541
MAGEA5	0.063409655	0.000518078	1.70708604
MAGEB1	0.276229233	0.000158976	5.455953607
MAGEB10	0.036769049	7.78E-05	2.094149214
MAGEB16	0.019826681	0.002988593	1.656615923
MAGEB18	0.028672547	0.003500974	1.770169119
MAGEB2	0.858425753	2.13E-05	4.117721998
MAGEB6	0.006383743	2.57E-05	2.724431779
MAGEC1	0.069317265	0.003434397	2.334682917
MAGEC2	1.958765536	1.96E-06	4.744961508
MAGEC3	0.406434728	9.70E-06	2.549460962
MAGEE1	6.49396483	9.86E-05	0.532835207
MAGEH1	77.27984529	0.003628027	0.636115234
MAGEL2	0.351725756	0.000187234	1.906804105
MAGI1	15.62498659	0.000311036	0.537871324
MAGI1-IT1	0.270349408	0.00059782	1.864883468
MAGI3	6.310002499	1.51E-07	0.372182927
MAGOHB	10.66807354	2.59E-05	1.884272847
MAGT1	117.6708634	1.12E-05	0.500957649
MAIP1	38.54835146	0.001173702	0.603581974
MAK	0.900253817	0.002446622	1.579023835
MALL	6.844466898	0.000402075	0.546153702
MAMDC4	12.8398404	0.002709054	1.575975293
MAML2	42.53370755	7.37E-09	0.389969156
MAML3	11.74210248	6.50E-05	0.517463352
MAN2A1	34.10936865	0.000910392	0.580721998
MAN2C1	34.31498281	0.001691929	1.60377911
MANCR	0.844775285	1.26E-05	2.041558305
MANEA	31.3154906	0.000388686	0.567583238
MANEA-DT	1.414134473	0.000169432	1.759315562
MANF	78.05490581	0.001750985	1.60762957
MANSC1	17.50036505	0.000313298	0.563453009
MAOB	351.7254827	0.000623313	0.5897948
MAP2K2	69.40660133	0.000818597	1.659191198
MAP2K4	26.92209955	0.000179105	0.549871907
MAP2K5	15.6059829	0.000474579	0.576601017
MAP3K1	27.53268893	0.001173839	0.595657283
MAP3K12	9.778925242	2.48E-05	1.880337802
MAP3K13	17.57164732	0.000809983	0.5938704
MAP3K14-A	1.937707649	0.000917678	1.647434643
MAP3K19	0.053624666	0.002996699	1.574133343
MAP3K20-A	0.356460583	6.76E-07	2.096769519
MAP3K8	12.6786648	0.000366829	1.704636986
MAP6D1	3.641685577	3.13E-10	2.523721154
MAP7	45.51436932	3.06E-08	0.38546346
MAPK1	89.32763689	9.51E-07	0.461672518
MAPK1IP1L	61.79643446	0.000291763	0.560406605
MAPK3	103.0752044	0.000273011	0.570895159
MAPK8	13.89853276	0.001328314	0.60373852
MAPK8IP1	18.86476957	0.003690946	0.622163978
MAPK8IP2	1.689200037	0.004939216	1.632819889
MAPK8IP3	31.13845362	0.000211079	1.741379497
MAPK9	26.44585436	0.00025644	0.562735656
MAPKAPK3	57.63539138	0.00115422	0.576627956
MAPKAPK5	22.02903821	0.004200785	1.542056724
MAPRE2	91.25402013	0.001000594	0.581447216
MAPRE3	23.9450734	0.001118976	1.661274614
MAPT	26.23542305	3.15E-06	0.463929486
MAPT-AS1	1.151716949	9.34E-05	0.506926366
MAPT-IT1	0.218796123	0.000639404	0.529766363
2-Mar	76.32080201	0.000417632	0.575239691
4-Mar	1.588690749	0.001028151	0.57641256
6-Mar	79.07817459	0.002438115	0.620444038
7-Mar	48.85846848	0.001324624	0.602404819
8-Mar	32.10060665	2.81E-07	0.439031788
MARF1	41.12208084	0.002300474	0.617733117
MARK2P10	0.041748062	8.66E-05	1.902829248
MARK2P11	0.02737507	0.000520797	1.742892305
MARK2P16	0	0.004606615	1.557019068
MARS	37.4467221	7.96E-09	2.367995619
MARVELD2	9.520041061	0.000205227	0.511968508
MARVELD3	5.502602365	0.003655883	0.624341895
MAST1	0.383160087	3.97E-06	2.109531181
MAST4	20.88211133	1.78E-05	0.500866314
MAT1A	4.56934133	6.99E-09	2.54769133
MAT2A	185.7595326	0.000365957	0.566997989
MAT2B	99.49079043	8.41E-09	0.384875795
MATN1	0.335044473	2.49E-05	1.880424376
MATN1-AS	2.207980859	0.00107792	1.633473909
MATN4	0.41709728	2.08E-07	2.422850115
MB21D2	16.93944094	0.000882613	0.58570763
MBD2	62.29246655	0.001998106	0.621881312
MBD3	31.77232163	0.000702767	1.66858096
MBD3L4	0	0.004606615	1.557019068
MBD5	7.704802135	0.001359787	0.600835835
MBIP	18.08322384	0.004146394	0.629571093
MBLAC2	8.431713572	0.000204417	0.554252951
MBNL1-AS	3.110232456	0.004824059	0.620048558
MBNL2	99.25275079	5.42E-05	0.527303711
MBOAT7	51.48718374	1.34E-08	2.330816446
MBP	14.2737055	0.000123254	0.519742229
MBTPS1	57.43172193	0.000112474	0.546231608
MBTPS2	16.19936721	0.001221693	0.602691616
MC1R	3.431467861	2.34E-05	1.883828991
MC3R	0.039568981	0.004379629	1.746874915
MCC	12.56877601	0.001372173	0.588196733
MCCC1-AS	2.881626838	9.33E-06	1.933763488
MCCC2	59.96670812	0.002857323	0.617042986
MCEMP1	1.397048118	0.0014579	1.696379735
MCF2L2	0.221781001	4.06E-06	2.006705173
MCFD2	142.9049597	0.004084723	0.644350793
MCIDAS	0.15845405	0.002835797	1.747219716
MCM10	1.703642677	0.000565354	1.690003014
MCM3AP-A	1.96101765	0.001638877	1.603953723
MCRIP1	57.74090671	0.004968567	1.531698224
MCRIP2P1	0.557620433	6.55E-15	3.143791664
MCTS1	35.7243124	0.000411827	1.706146636
MCU	27.09479216	0.004888688	0.637967312
MCUR1	24.28584996	0.001773933	0.614002835
MDGA2	0.208915082	0.004586411	0.573932056
MDK	101.0519255	4.31E-07	2.142146162
MDM4	16.73839099	0.00484679	1.527271598
MDN1	10.2864401	0.000972246	0.585173206
MECOM	18.585627	0.00404643	0.583940837
MECP2	18.74162961	0.00031866	0.554744706
MED13	27.06144674	0.000652577	0.578106974
MED13L	33.16867813	0.00123779	0.59581433
MED15P4	0.0251922	0.000342273	2.180110024
MED17	11.61555033	0.000556635	0.581458964
MED21	33.70603643	5.08E-05	0.523199194
MED22	17.73236069	0.002950541	0.622800231
MED25	26.64380162	0.000633469	1.675025634
MED28P3	0.820120161	0.000144864	0.531944902
MED28P4	0.041613349	3.15E-05	2.068477716
MED6P1	0.067903448	0.001342197	1.671697958
MED7	21.47355385	2.11E-06	0.469733152
MED9	15.3551354	0.003521395	0.623399224
MEF2A	64.83233004	0.000849523	0.593049494
MEF2B	1.179227966	4.52E-06	1.978890311
MEF2D	65.94186713	0.00454773	0.637938345
MEG9	0.341110593	0.000404245	1.793568615
MEGF9	30.31316148	7.96E-07	0.432962092
MEI1	2.108775495	0.001510541	1.63671321
MEI4	5.828034654	0.002781789	0.60066859
MEIG1	0.255524649	0.002579659	1.578556732
MEIS1-AS2	0.288769865	0.001371729	1.634149655

MEIS3P1	15.83350358	0.002431556	0.610738539
MEIS3P2	1.678773956	2.71E-05	0.444933373
MELK	5.570740231	4.24E-07	2.142683058
MELTF	4.917302229	5.84E-11	2.741360144
MELTF-AS1	6.022002019	3.33E-16	3.218692922
MEMO1	2.046122085	0.00048583	1.688714226
MEPCE	89.92041057	9.35E-05	0.530417624
MERTK	24.73295084	0.00145784	0.596203008
METAP1	43.90887503	5.37E-05	0.517335388
METAP2	67.30761716	4.91E-05	0.532526745
METTL1	30.10412748	0.000748951	1.665102813
METTL11B	0.058568794	0.0013709	1.659210642
METTL14	15.82378146	3.06E-05	0.516415773
METTL15	11.58267967	1.21E-05	0.494683808
METTL16	17.70479078	0.000851922	0.59422461
METTL21EP	0.35062298	0.004611998	1.537482482
METTL22	5.786465723	7.74E-05	1.809055323
METTL24	5.204826511	5.48E-08	0.379148425
METTL25	6.742631214	0.002757851	0.623153336
METTL26	167.1642674	0.003688589	1.553249963
METTL7A	198.8602821	2.25E-06	0.46258816
METTL9	112.0352766	0.000420666	0.57733508
MFAP1	63.68766299	3.11E-08	0.420266779
MFAP2	6.159685948	0.0001794	1.861755695
MFAP3	27.2238819	0.000164897	0.549455294
MFAP3L	22.53767419	4.91E-05	0.508175019
MFHAS1	17.56512701	3.37E-05	0.491705283
MFN2	92.77677215	1.09E-05	0.495833066
MFSD1	64.02824516	0.003503832	0.628068243
MFSD10	91.66747789	2.22E-07	2.187089552
MFSD14A	80.51917168	0.00099233	0.598965109
MFSD2A	6.60321399	3.17E-05	1.920038335
MFSD2B	0.19391311	0.004193523	1.556328514
MFSD4A	8.711640178	4.39E-05	0.501438965
MFSD4BP1	0.053380114	7.93E-05	1.88302174
MFSD6	28.84122774	0.000203537	0.548065037
MFSD8	17.90952822	0.002919774	0.623636992
MFSD9	12.71639605	0.001563118	0.606952951
MGA	9.765970488	0.003568929	0.619064759
MGAM	30.00968275	6.50E-07	0.409564691
MGAT2P2	0	0.004606615	1.557019068
MGAT4EP	0.086479352	2.88E-05	1.907949814
MGAT5	40.3102544	0.000937613	0.591008983
MHENCR	21.02994122	4.85E-06	1.975307765
MIAT	3.021241616	6.98E-07	2.128300053
MIATNB	3.243747052	0.00446112	1.535726566
MIB1	23.06045084	0.000988078	0.584077803
MICAL1	27.83824476	8.68E-05	1.799847196
MICAL3	10.84511309	0.000149758	0.538006239
MICALL2	14.25025781	6.12E-07	2.092311302
MICU1	79.68050327	1.33E-06	0.454607303
MICU2	50.12505598	2.40E-06	0.470528419
MID2	12.88299408	0.000138396	0.549413945
MIEF1	33.19910928	2.02E-05	0.513282881
MIEN1	61.96106414	6.62E-05	1.823255427
MIER1	26.81249301	0.000238438	0.562419466
MIGA1	22.22834979	4.96E-08	0.41065268
MIIP	18.46989077	1.61E-07	2.182674919
MINAR1	0.87628863	4.49E-07	2.117862277
MINAR2	0.027069773	0.004320344	1.58753932
MINCR	6.078927741	1.05E-06	2.070043393
MINDY1	40.67358158	0.000103587	0.513531257
MINDY2	24.33145504	0.000161768	0.529986456
MIOX	190.533205	0.00288113	0.607783081
MIP	0.148611075	0.004490379	1.538055234
MIPEP	29.59091324	0.000192672	0.543084394
MIPEPP2	0.011230349	0.004612016	1.727425006
MIR1-1HG-A	0.143629093	0.00461779	1.588337676
MIR1183	0.241192596	0.002737113	1.712034334
MIR1184-1	0	0.004606615	1.557019068
MIR1184-2	0	0.004606615	1.557019068
MIR1184-3	0	0.004606615	1.557019068
MIR1205	0.902761904	0.002106328	1.608725982
MIR1233-1	0	0.004606615	1.557019068
MIR1233-2	0	0.004606615	1.557019068
MIR1249	3.120116733	1.98E-06	2.028671725
MIR1253	0	0.004606615	1.557019068
MIR1258	0.003209285	0.000550753	7.899676717
MIR125A	0	0.004606615	1.557019068
MIR1260A	0	0.004606615	1.557019068
MIR1260B	0	0.004606615	1.557019068
MIR127	0	0.004606615	1.557019068
MIR1282	0	0.004606615	1.557019068
MIR1289-1	0.189746353	7.78E-05	1.86329678
MIR135A2	0	0.004606615	1.557019068
MIR136	0	0.004606615	1.557019068
MIR137HG	0.065676055	3.95E-11	4.783510665
MIR138-1	0.066840987	0.000445341	2.560224826
MIR140	24.44261851	0.003676281	0.616832096
MIR145	0	0.004606615	1.557019068
MIR1470	0	0.004606615	1.557019068
MIR152	0.302249881	0.002078039	1.613585659
MIR1538	0	0.004606615	1.557019068
MIR155HG	11.1689166	0.000834347	1.67527681
MIR1587	0	0.004606615	1.557019068
MIR181A1H	0.13791535	0.00183382	1.661985421
MIR193BHC	2.285813445	6.77E-06	1.993972086
MIR196A1	4.327846958	0.000139826	1.817657575
MIR205HG	0.271379728	0.00039743	1.839436356
MIR2117HC	0.164562446	1.87E-06	2.104504586
MIR221	3.627278314	0.003131315	1.619812219
MIR222	0.852585406	0.002186878	1.628702765
MIR222HG	3.406212417	0.000100032	1.918804987
MIR297	0	0.004606615	1.557019068
MIR3074	0	0.004606615	1.557019068
MIR3118-1	0.008401198	0.000222384	6.458082903
MIR3118-2	0	0.004606615	1.557019068
MIR3118-3	0	0.004606615	1.557019068
MIR3118-4	0	0.004606615	1.557019068
MIR3124	0	0.004606615	1.557019068
MIR3135A	0.249070163	3.93E-05	1.953603956
MIR3140	0.266174925	0.002129125	1.918584112
MIR3152	0.029758185	0.003236736	2.951179453
MIR3179-3	0	0.004606615	1.557019068
MIR3180-1	0	0.004606615	1.557019068
MIR3180-2	0	0.004606615	1.557019068
MIR3180-3	0	0.004606615	1.557019068
MIR3180-4	0.009957473	0.001375178	3.008070179
MIR3185	0	0.004606615	1.557019068
MIR3187	0	0.004606615	1.557019068
MIR3190	0	0.004606615	1.557019068
MIR3193	0.620591403	0.000551383	0.525662493
MIR3195	0	0.004606615	1.557019068
MIR324	3.404567928	8.25E-05	1.807607109
MIR335	0	0.004606615	1.557019068
MIR3591	0	0.004606615	1.557019068
MIR3621	0	0.004606615	1.557019068
MIR3622B	0	0.004606615	1.557019068
MIR3650	0	0.004606615	1.557019068
MIR3651	0.22107229	1.26E-05	2.089984737
MIR3660	0.14933837	0.00345054	1.770841674
MIR3663	0.018749156	0.003825071	2.72941406
MIR3665	0	0.004606615	1.557019068
MIR3666	0.305694844	0.000452344	1.806161351
MIR3670-1	0	0.004606615	1.557019068
MIR3670-3	0	0.004606615	1.557019068
MIR3670-4	0	0.004606615	1.557019068
MIR3682	11.0672873	7.31E-05	1.814551172
MIR3685	19.13103368	0.000154114	1.766695363
MIR3687-2	0	0.004606615	1.557019068
MIR3689A	0	0.004606615	1.557019068
MIR371A	0	0.004606615	1.557019068
MIR372	0	0.004606615	1.557019068
MIR378J	3.419604185	0.001041125	1.633031161
MIR3918	0	0.004606615	1.557019068
MIR3934	0	0.004606615	1.557019068
MIR3945HC	0.542405074	0.003145996	1.569874053
MIR4259	0.122851525	4.41E-05	2.382774146
MIR4271	0	0.004606615	1.557019068
MIR4273	0	0.004606615	1.557019068
MIR4292	10.9743846	0.000792089	1.656939868
MIR4300HC	0.049742578	0.002391903	1.688885977
MIR431	0	0.004606615	1.557019068
MIR432	0	0.004606615	1.557019068
MIR4323	0.165847413	0.000819843	2.006828915
MIR4326	2.249633332	0.000486782	1.691990261
MIR433	0	0.004606615	1.557019068
MIR4451	0.205435511	0.000343535	1.887415575
MIR4464	0.007336754	0.00130678	7.067688825
MIR4477B	7.152144235	0.001013075	1.643993391

MIR4479	0.20693738	0.001517732	1.784750335
MIR4489	4.084056396	6.01E-07	2.092218793
MIR4492	0	0.004606615	1.557019068
MIR4508	0.003885574	0.000845965	7.487844431
MIR4509-1	0	0.004606615	1.557019068
MIR4509-2	0	0.004606615	1.557019068
MIR4509-3	0	0.004606615	1.557019068
MIR4510	0.095326696	0.000225435	2.170626844
MIR4519	0	0.004606615	1.557019068
MIR4527HG	0.082618812	1.72E-07	2.228099677
MIR4528	0.009983796	4.06E-06	7.277523835
MIR4538	1.806491576	0.004537513	1.610924681
MIR4539	1.327109319	9.99E-05	1.863312512
MIR4638	0	0.004606615	1.557019068
MIR4641	0.168436721	3.98E-05	2.151456161
MIR4642	0.408719425	0.001302113	1.654649874
MIR4644	0.843165262	0.001574056	1.624253396
MIR4656	0	0.004606615	1.557019068
MIR4664	18.15980766	0.000618645	0.554411301
MIR4668	4.92686324	6.18E-05	1.826667584
MIR4685	1.396834276	1.66E-05	1.905221802
MIR4690	0.222295395	0.004438584	1.710799718
MIR4728	1.263789005	6.38E-05	1.841264878
MIR4746	0	0.004606615	1.557019068
MIR4755	0.688315554	0.000823189	1.657530826
MIR4757	0.025937163	0.001714225	2.792785593
MIR4768	25.69377755	3.45E-05	1.872207724
MIR4771-1	0.01033082	0.004002519	4.585758292
MIR4777	0.558132459	2.66E-05	1.874795355
MIR490	0	0.004606615	1.557019068
MIR499A	1.680671631	1.96E-07	2.164081286
MIR503HG	14.12444841	0.000104465	1.809028337
MIR519A1	0.160605352	0.001524986	1.956305338
MIR519D	0.236406198	5.98E-05	2.030274799
MIR548AA2	6.319033134	0.000255374	1.743341713
MIR548BB	0	0.004606615	1.557019068
MIR548H3	0.066911443	0.000230917	2.377049933
MIR548X	0.029899148	0.002460456	3.033015781
MIR548XHG	0.043821828	0.000412368	1.788107729
MIR5584	0.087764325	0.004700766	2.106949402
MIR5585	0.341594734	0.003171046	1.647207227
MIR567	0.257515998	6.69E-05	1.948798268
MIR5689HG	0.055171131	0.000159935	1.78090578
MIR5701-1	0	0.004606615	1.557019068
MIR5701-2	0	0.004606615	1.557019068
MIR5701-3	0	0.004606615	1.557019068
MIR581	1.591573189	0.000380055	1.763700105
MIR582	0.197783178	0.000644618	1.885034377
MIR583HG	0.616343343	0.00024247	1.742965637
MIR606	0.51657182	0.002534808	1.631257548
MIR6077	0.337865606	6.78E-07	2.171311622
MIR6080	0	0.004606615	1.557019068
MIR6085	0.471106342	0.003979119	1.572886586
MIR6126	0	0.004606615	1.557019068
MIR615	0.384264916	0.000742934	1.679642837
MIR633	0.035447932	0.000207044	3.512667908
MIR642A	0.401967538	0.004736072	1.548946442
MIR647	29.23394376	0.003570045	1.552160787
MIR6511A2	0	0.004606615	1.557019068
MIR662	0	0.004606615	1.557019068
MIR663AHG	0.101049621	0.000484389	2.822658828
MIR6719	1.685037822	0.000389837	1.709526054
MIR6720	0	0.004606615	1.557019068
MIR6724-1	0	0.004606615	1.557019068
MIR6724-2	0	0.004606615	1.557019068
MIR6728	2.361330573	7.46E-05	1.815463699
MIR6735	0	0.004606615	1.557019068
MIR6745	0.153531445	0.000920718	1.77884107
MIR6750	0.555799133	0.000185922	1.755244375
MIR6753	1.645262621	4.64E-07	2.110017636
MIR6768	0.139411919	0.000885299	1.909649032
MIR6770-1	0	0.004606615	1.557019068
MIR6770-2	0	0.004606615	1.557019068
MIR6770-3	0	0.004606615	1.557019068
MIR6775	0.787632323	6.82E-06	1.967629265
MIR6793	1.568753049	0.004946466	1.534844909
MIR6797	3.217552831	1.73E-07	2.169936034
MIR6804	0.464518051	0.000165587	1.796864259
MIR6818	0	0.004606615	1.557019068
MIR6819	4.685108843	1.05E-05	1.928271096
MIR6832	1.169010137	6.56E-08	2.224003346
MIR6855	0.250598954	0.004298794	1.643675274
MIR6859-4	1.066826582	0.000163914	1.777704415
MIR6862-1	0	0.004606615	1.557019068
MIR6877	0.114401579	0.003684403	1.940511379
MIR7106	0	0.004606615	1.557019068
MIR7111	3.376864477	2.52E-07	2.1447418
MIR7161	0	0.004606615	1.557019068
MIR7515HG	0.007305154	0.001128678	1.67821561
MIR762	0	0.004606615	1.557019068
MIR762HG	2.165442311	6.27E-07	2.097882343
MIR765	1.590447857	0.000710681	1.663243471
MIR766	0.352622746	0.001627414	1.631960477
MIR7851	0.772072942	4.53E-05	1.838194685
MIR8079	0	0.004606615	1.557019068
MIR8086	0.069375058	2.82E-05	2.474004991
MIR891A	0	0.004606615	1.557019068
MIR941-2	0	0.004606615	1.557019068
MIRLET7A1	5.401535954	0.000460973	1.698864489
MIRLET7BI	1.689282087	0.003483614	1.568310477
MIRLET7E	0	0.004606615	1.557019068
MITD1	17.22690501	0.001703385	1.605694094
MITF	29.92395195	0.001095308	0.569337101
MIXL1	0.433566677	4.65E-06	2.028885659
MKI67	8.002704373	3.89E-06	2.005585989
MKKS	25.97372795	0.000278631	0.535687012
MKLN1	17.43256502	0.000100619	0.534708415
MKLN1-AS	5.414079729	0.00098547	0.594782016
MKRN1	77.04305886	0.000617162	0.590713953
MKRN5P	0.248663081	0.004145371	1.556478445
MKRN9P	0.138793561	0.000372056	1.790544749
MLF2	186.5737651	0.001169946	1.634531555
MLLT11	7.027796099	5.81E-06	2.042204202
MLLT3	12.1056472	0.001293207	0.600697167
MLPH	3.667108304	3.82E-05	1.935969948
MMAA	11.99670107	8.96E-06	0.486123835
MMADHC	146.3720019	9.29E-05	0.545068466
MMGT1	54.32399175	7.24E-06	0.49486699
MMP12	4.778619625	0.000252646	2.248827746
MMP17	1.69345456	3.16E-05	1.965706798
MMP19	9.260914774	0.000160326	1.776517247
MMP23B	0.15829562	0.000655431	1.679049279
MMP24	32.85494014	0.001091537	0.56189983
MMP25-AS1	7.279760888	0.001319789	1.621517998
MMP3	0.54520842	6.60E-05	2.012460343
MMP9	59.08887448	0.001448562	1.707587749
MMRN2	103.0917423	0.000296526	0.559501834
MMUT	57.52262301	6.83E-09	0.373566019
MND1	3.049988056	0.001998555	1.599221288
MNX1	0.603437004	3.75E-06	2.028626793
MNX1-AS1	0.305698239	6.13E-07	2.191515387
MNX1-AS2	0.434920338	4.09E-09	2.422395648
MOAP1	88.58143275	2.56E-06	0.466445552
MOB3B	28.50255217	8.88E-11	0.338922008
MOB4	26.70358394	0.000793528	0.58464143
MOBP	0.040859363	4.93E-05	1.840970513
MOCOS	5.469140566	1.73E-10	2.564218855
MOCS2	39.32853705	0.002674159	0.618185717
MOCS3	8.772546941	0.004296471	0.643459421
MOGS	79.63589001	0.003314466	1.563482659
MORF4L1	139.271047	3.41E-05	0.523500269
MORF4L1P	33.59782184	5.39E-08	0.423988793
MOSMO	12.54695801	6.94E-05	0.527325236
MOV10	35.97521877	4.48E-07	2.14449795
MPG	56.28015483	0.001899621	1.600579102
MPI	39.15421738	8.81E-05	0.544097282
MPL	0.647446243	0.00073045	0.573396516
MPP1	42.50972694	0.001221671	0.597996983
MPP2	1.149208843	0.000746879	1.718901953
MPP3	3.195546	0.000681048	1.668782477
MPP4	0.158082586	2.61E-07	2.152098181
MPP5	20.24149977	8.88E-08	0.399865617
MPP6	13.17281668	2.52E-05	0.492676711
MPP7	5.738229278	2.97E-05	0.428428731
MPPED2	2.364952512	0.000912337	0.519706902
MPRIP-AS1	0.249322332	0.001616689	1.629350583
MPZL2	71.83965731	8.84E-05	0.527091527
MRAP	0.099716237	0.000808564	1.709286782
MRFAP1	441.1221432	9.48E-06	0.509424313
MRFAP1L1	123.0731635	0.004119644	0.644936492

MRGBP	28.19736293	0.000939056	1.651447872
MRGPRD	0.068641067	0.001186516	1.651603751
MRGPRX3	0.066514702	0.000470264	1.927922782
MROH3P	0.382282513	0.000256496	1.766460363
MROH5	0.176473535	0.001378261	1.637734772
MROH6	4.897406925	0.000702248	1.66902694
MROH7-TT	0.092530048	0.001704155	1.610452778
MROH9	0.053186056	0.002395821	1.627877946
MRPL12	63.08166006	3.58E-05	1.862375072
MRPL17	102.6098103	5.98E-05	1.833543335
MRPL30	41.10852926	1.84E-05	0.510316622
MRPL37P1	0.484651019	0.000122341	1.779219788
MRPL38	7.410983087	0.000152401	1.763616094
MRPL40	88.06792001	0.001338061	1.625462024
MRPL40P1	0.543951797	8.86E-07	2.084827858
MRPL44	82.33198194	0.000181173	0.5595829
MRPL45	95.93947255	0.000387662	0.572094448
MRPL45P1	0.036317785	1.07E-05	2.213497175
MRPL49	88.099721	1.96E-10	0.364764233
MRPL50	33.6770491	2.62E-08	0.404525519
MRPL52	36.068331	6.63E-05	1.822770025
MRPL53	9.565182169	0.000138706	1.773740532
MRPL57P7	0.05393811	3.69E-05	2.218671378
MRPL58	66.65573736	0.001500494	1.618973983
MRPL9P1	0.047417295	0.004004045	1.744194824
MRPS12	47.32679985	0.000215865	1.748672288
MRPS14	37.4098876	4.73E-05	0.531636688
MRPS15	70.89402742	0.00027594	1.731691328
MRPS18A	58.00864183	0.000653641	0.592429994
MRPS18AP1	0.857859947	6.62E-05	1.81746618
MRPS18B	166.612502	4.44E-05	0.532880587
MRPS18BP2	0.027666641	0.001393119	1.792152152
MRPS18C	10.15652037	5.52E-06	0.49310168
MRPS18CP6	0.476000322	7.14E-06	1.97002826
MRPS31P1	0.043866092	1.26E-05	2.818588302
MRPS36	69.91364911	0.001440893	0.603982168
MRPS36P5	0.35996224	0.001033262	1.712394733
MRTFA-AS1	0.313316203	3.33E-05	1.881974947
MRTFB	13.85990112	0.000159458	0.537617146
MRVI1	26.51657282	0.002302885	0.609079267
MS4A14	4.759384748	0.000813142	1.658159766
MS4A2	2.329386063	0.002736928	0.606064748
MS4A8	0.270338375	6.40E-05	2.230171906
MSANTD1	0.186452996	0.000199169	1.745441725
MSANTD2P	0.046273945	0.003351779	1.619433562
MSANTD4	16.16837452	0.000121914	0.540279963
MSC	83.09845958	1.10E-06	2.102993572
MSH2	23.30763591	0.000330009	0.570343794
MSH3	20.72514311	5.04E-08	0.403789202
MSH5	3.324396963	7.46E-05	1.80637991
MSH5-SAPC	1.121072247	0.000264445	1.73182801
MSL2	30.00412666	0.002217608	0.622237762
MSMB	0.477304502	7.92E-05	1.911132086
MSMP	0	0.004606615	1.557019068
MSN	594.8429047	0.00015691	0.558996639
MSNP1	0.644569899	1.11E-07	0.406107208
MSRA	35.67423817	2.22E-05	0.48726634
MSS51	2.59650528	1.91E-05	1.890608396
MSTO1	9.621803505	9.55E-08	2.221343549
MSTO2P	6.688624643	1.80E-05	1.893841983
MSX2	1.44742292	1.98E-05	1.916061822
MT1E	178.6837369	0.000128024	1.824095058
MT1F	45.42560809	0.000494595	1.703919021
MT1HL1	0.174062453	0.002569044	1.582507118
MT1X	137.9281127	0.000146771	1.823864025
MT1XP1	2.881244008	0.000181497	1.79073128
MT2A	580.662284	5.01E-05	1.866749314
MTAP	9.505728424	0.000662822	0.586953697
MTATP6P21	0.154563735	0.002123454	1.691976544
MTATP6P26	0.342513036	4.47E-05	1.846081624
MTATP6P27	0.507627579	1.54E-05	1.910424052
MTCH1	256.7251271	2.73E-06	0.472637074
MTCO1P1	0.007159383	0.004010474	1.981921266
MTCO1P39	0.121473193	0.003067483	1.573896842
MTCO1P4	0.03143259	0.001946475	1.60836049
MTCO1P49	0.064712802	0.000345255	1.743422418
MTCO3P27	0.034795567	0.00179553	0.268396382
MTCO3P41	0.085576789	0.000112063	1.898751213
MTCO3P43	0.29900504	9.43E-05	1.795528471
MTCO3P5	0.098292393	0.003708881	1.582127613
MTCP1	13.72371876	0.003761199	1.558120268
MTCYBP21	0.170452684	9.38E-05	1.816558528
MTCYBP27	0.03160427	0.000247791	1.973088921
MTCYBP45	0.048803144	0.003401825	1.669852319
MTDHP1	0.492938591	1.11E-06	2.06157948
MTERF3	31.01914586	0.00055465	1.684725867
MTERF4	11.39285203	0.003183041	0.625297607
MTF1	11.75734654	0.000150476	0.545980944
MTFMT	12.91936565	0.001417449	0.606488175
MTFR1L	44.21017301	0.002478994	0.626857601
MTFR2	2.051601084	1.52E-07	2.184044349
MTG1	6.415912818	5.83E-05	1.821096936
MTG2	18.52074512	0.001040106	1.646371102
MTHFD1	40.16343256	5.77E-06	0.477640219
MTHFD2	25.80160821	3.78E-07	2.133531446
MTHFR	18.90818374	0.001879592	0.607765634
MTHFS	19.27071112	1.08E-05	0.487415519
MTM1	19.9106722	1.39E-05	0.508850876
MTMR12	44.5426061	8.59E-06	0.492050618
MTMR6	37.62171356	0.000273167	0.564281572
MTMR9	9.805802855	0.000215991	0.552270333
MTND1P6	0.02040835	0.002555932	1.745951648
MTND2P3	0	0.004606615	1.557019068
MTND2P40	0.452063613	0.001419797	1.622576358
MTND4P20	1.21405253	4.42E-05	1.950685369
MTND4P23	0.30075543	0.003765602	1.557063322
MTND4P31	0.042911025	8.81E-05	1.851864267
MTND5P1	0.183853833	0.002071298	1.633378772
MTND5P25	0.277661815	5.16E-05	1.840856653
MTND5P28	0.203523716	0.003160282	1.56199123
MTND5P34	0.008940547	0.000822846	2.715663133
MTND6P5	0.168683713	0.004974848	1.548379531
MTNR1B	0.039970844	1.18E-05	2.343830529
MTO1	8.315775962	0.003500666	0.638700179
MTPN	228.7190852	0.001611509	0.61347394
MTREX	43.7579655	9.01E-05	0.543487587
MTSS1	42.0033878	0.000606543	0.560735965
MTURN	22.61056852	1.39E-05	0.433034766
MTUS1	26.15512697	0.00097254	0.57481751
MTUS2-AS1	0.072386469	2.37E-08	2.346276536
MUC12	0.676697236	1.29E-05	1.986563598
MUC5AC	0.008206987	0.002441671	1.622294267
MUC5B	0.188740348	3.42E-05	2.105347028
MUC5B-AS1	0	0.004606615	1.557019068
MUL1	77.12976429	4.70E-07	0.449628498
MUSK	0.052766001	0.0008402	1.685340482
MUTYH	9.802623712	0.001684116	1.606465823
MX2	5.191117748	9.71E-05	1.800042319
MXD3	4.224741447	6.20E-11	2.597870597
MXI1	115.8856241	3.61E-05	0.518077493
MXRA8	89.33635316	0.001785026	1.612635712
MYADML	0.123964235	0.001796248	1.706078024
MYADML2	0.035525002	3.25E-06	2.023996276
MYB	0.719160751	5.59E-05	1.85013563
MYBL2	10.80949223	1.40E-09	2.473983009
MYBPC2	1.070525309	0.000823605	1.68287879
MYBPC3	0.261526989	3.34E-06	1.994853979
MYBPH	0.333198841	0.003129484	1.602613941
MYBPHL	0.101475736	2.14E-05	1.898919597
MYCBP2	18.34960385	1.36E-07	0.404676088
MYCBPAP	0.412470467	1.27E-07	2.196052198
MYCN	1.616503929	7.78E-05	0.491235783
MYCT1	45.96279869	0.004481054	0.636838174
MYDGF	224.6244592	0.003398172	1.559727798
MYF6	0.063327195	0.000192807	1.8641005
MYH10	40.89071504	0.000508218	0.551792131
MYH16	0.218486755	2.21E-10	2.54753553
MYH3	1.977218529	0.004118169	1.545676634
MYH6	0.01364831	0.004818996	1.574586109
MYL3	16.84246988	7.96E-05	0.505965396
MYL7	0.063210768	0.000194742	1.827139334
MYLIP	76.51093003	8.65E-06	0.493735189
MYLK	48.84252016	3.33E-05	0.515231892
MYLK-AS1	1.656364574	0.001665133	1.606733762
MYLK2	0.452836353	1.20E-05	1.936012079
MYNN	14.51660478	0.0036473	0.634728288
MYO16-AS1	0.19648878	0.000126602	1.909477292
MYO18A	24.97814248	0.004924107	0.643805014
MYO19	9.923784527	0.001927489	1.597914471
MYO5B	19.11010796	0.003761354	0.612336178

MYO6	50.54253468	2.28E-08	0.390200296
MYO9A	21.6174606	2.10E-06	0.459659873
MYOG	0.122961262	6.48E-05	1.846953874
MYOM3	23.86545379	0.001514547	0.597042316
MYORG	13.92404314	1.16E-05	0.467855522
MYOSLID	1.203736436	4.40E-05	2.033766095
MYPN	0.098198599	0.002021939	1.702882289
MYPOP	13.17225513	3.51E-05	1.875387336
MZB1	17.39015916	0.003285822	1.675511092
MZF1	13.82950092	1.37E-06	2.046238605
MZT1P2	0.570825739	1.64E-06	2.044626523
MZT2A	41.82748974	0.000239588	1.740773823
MZT2B	134.7010508	0.001045919	1.642398727
N4BP2L2-IT	2.880137536	5.31E-05	1.824681974
NAA10	24.41793519	8.09E-07	2.086696281
NAA30	15.98300013	4.45E-05	0.503966678
NAA35	17.87569312	0.001285842	0.598111165
NAA38	63.1796358	0.004951723	1.534548495
NAA40	16.67667561	5.98E-06	1.97392279
NAA50	58.69878111	0.000358792	0.569731324
NABP1	7.41796681	0.000112392	1.781036845
NACC1	43.87642915	0.000396224	1.707167252
NADK2	32.61485475	1.27E-05	0.498689553
NADSYN1	18.04937729	0.002377731	1.581208828
NAF1	9.118952351	0.00022938	0.557649483
NAGPA	12.96239827	3.80E-06	2.010908149
NAIF1	9.638996614	0.001506632	0.609883919
NALCN-AS	0.132800299	2.46E-06	2.082461461
NALT1	2.732701312	9.69E-07	2.085511326
NAMPT	63.1925073	0.003918251	1.563239186
NANOG	0.048118788	0.00148399	1.658963172
NANOGNB	0.544796492	0.00015291	1.768941531
NAP1L4P2	0.012652197	8.23E-05	2.424184313
NAPA	52.94316647	0.001383429	0.607913129
NAPEPLD	15.86281572	0.000665273	0.577139355
NAPSA	32.58624673	0.001829712	0.558651411
NARF	22.23826554	1.41E-07	2.219263229
NARF-AS1	0.388388288	0.001729602	1.60202748
NARF-IT1	1.774944381	0.000277378	1.722922139
NARS2	21.49083784	6.13E-05	0.535033476
NAT16	0.215711665	4.31E-05	2.004180259
NAT8	1204.424561	0.001399685	0.601825408
NAT8B	52.79188874	5.41E-06	0.433980842
NAT9	23.29854338	2.68E-07	2.152534083
NATD1	23.3146555	0.001325158	0.601419906
NAV2-AS1	0	0.004606615	1.557019068
NAV2-AS3	0.215508793	5.53E-05	1.944949964
NAV3	0.982555045	1.04E-05	1.977377045
NBAS	28.68609469	7.96E-06	0.485680074
NBAT1	0.920472242	0.002437811	0.590112184
NBEA	8.38643882	1.37E-08	0.333071194
NBEAL1	5.088514181	9.08E-05	0.513249609
NBEAP5	0.020480342	0.000485698	2.627503644
NBL1	48.67281451	0.004863702	1.550218331
NBPF1	4.966716282	0.000192763	0.541577261
NBPF3	4.403634718	0.000122968	0.532033175
NBPF8	5.498690048	0.000210497	1.743784774
NBR1	105.5095964	2.95E-06	0.478526413
NCAM1-AS	0.198275306	5.69E-05	1.924474613
NCAM2	0.280314919	8.06E-05	1.860229244
NCAPG	3.938553501	2.43E-09	2.41407293
NCAPGP1	0.100631651	4.98E-07	2.119721432
NCAPH	4.16632176	5.21E-09	2.387555309
NCBP2-AS1	0.968868185	0.004646795	1.53502512
NCF4	29.47078112	8.63E-05	1.805182146
NCF4-AS1	0.073162294	0.002067156	1.593887749
NCK1	45.53493601	0.001692111	0.613347327
NCKAP1	38.35193872	8.31E-07	0.458977035
NCKAP5	3.218449046	8.20E-05	0.507043733
NCKAP5L	13.84151055	6.98E-11	2.663867887
NCKIPSD	48.66423407	0.003940292	0.625483417
NCL	265.956725	0.001585065	0.612853146
NCLN	66.29034538	1.48E-06	2.073819157
NCOA1	35.06239484	8.75E-05	0.531859717
NCOA2	26.6394184	0.000846768	0.583101368
NCOA3	32.87472988	0.002192646	0.611852579
NCOA4	301.3130881	5.00E-07	0.455332941
NCOA4P3	0.094679634	0.000230931	0.511945377
NCOA4P4	0.064596881	0.003908369	0.625074695
NCOA7	42.55049243	1.80E-06	0.429883755
NCOR1	36.00026389	4.69E-05	0.520316103
NCR3LG1	7.89915963	6.82E-08	0.367638108
NDC80	5.501953338	5.18E-07	2.113674496
NDFIP1	130.3288904	2.92E-07	0.420121397
NDFIP2	65.42574006	3.40E-06	0.471240296
NDOR1	12.5091422	0.000255434	1.734653817
NDRG1	1026.35471	0.002276893	0.617498504
NDRG2	57.32359781	0.000375773	0.558478628
NDRG4	3.171084949	0.001746205	1.921838464
NDUFA10	40.6826412	0.003507363	0.633615204
NDUFA12P	0.065285629	8.53E-05	2.035560758
NDUFA3P3	0.093437961	0.001054695	1.924300875
NDUFA3P4	0.455908053	0.00205195	1.595783208
NDUFA5P6	0.080131493	0.002309299	1.737381295
NDUFA5P8	0.001440039	0.000342162	8.344938168
NDUFA9P1	0.223441488	8.62E-05	1.818475762
NDUFAB1P	0.388287843	0.000837998	1.668277293
NDUFAF3	79.74250993	0.001352221	1.624389208
NDUFAF4P	0.119529243	4.57E-06	1.979094639
NDUFAF5	6.176034297	0.000607924	1.681595575
NDUFAF8	42.75448218	1.04E-06	2.078891403
NDUFB10P	0.121317034	0.002505466	1.593612486
NDUFB2-AS	1.873911539	1.71E-06	2.04312771
NDUFB4P1	0.265119538	0.00075491	1.794635322
NDUFB4P3	0.293031181	0.001585085	1.670268161
NDUFB4P5	0.00917678	0.000535944	2.608120147
NDUFB6	109.7978163	3.43E-05	0.507561935
NDUFB9P2	0.336263951	0.001011221	1.678877993
NDUFC2-K	0	0.004606615	1.557019068
NDUFS1	38.51700873	7.72E-06	0.459078215
NDUFS4	159.9432371	0.001273507	0.601607615
NDUFS8	51.79263116	0.001525323	1.618773486
NEBL	20.4271304	2.03E-05	0.491979962
NECAP2	61.20814692	0.002455976	0.631677654
NECTIN3	15.13097304	6.04E-07	0.430011583
NEDD9	66.32712707	1.69E-05	0.481883942
NEIL3	1.703989672	4.19E-10	2.513556852
NEK1	10.8670372	0.002357638	0.614978027
NEK11	11.08453169	0.000256228	0.568605857
NEK2	3.534035119	1.49E-07	2.191110741
NEK4	9.09644266	0.00053634	0.574552954
NEK4P1	0.003057675	0.001936193	2.909679419
NEK4P3	0.062295173	0.001481143	1.734957361
NEK6	131.812675	4.47E-05	0.512529769
NEK7	59.93691278	0.000856432	0.583463971
NELFB	104.2918037	0.000696336	0.590900024
NEMP2	7.383556157	0.000306629	0.565551432
NEO1	52.00207662	0.000453462	0.566283268
NES	93.1827753	0.001021697	0.584951095
NEUROD1	0.052086387	3.03E-05	2.00919506
NEUROD2	0.064839564	7.43E-05	1.840550793
NEUROD4	0.161066203	0.000379152	2.205094681
NEXN-AS1	0.489800036	0.003421971	1.562505336
NFAT5	16.56197139	0.002551725	0.616020106
NFATC3	13.81476264	0.000163814	0.558716798
NFE2L1	397.3531457	0.002405447	0.620339505
NFE2L3	40.17762972	0.000130226	1.78534685
NFE4	2.091990087	7.22E-11	5.9452635
NFIA	34.20268665	4.38E-05	0.536597977
NFIA-AS2	0.411093919	0.000265017	1.768475997
NFIB	57.10595486	6.39E-06	0.493564345
NFKB1	48.63221295	2.28E-05	0.514753755
NFKB2	76.09632864	0.00339124	1.559367559
NFKBIZ	18.36580586	3.47E-08	2.264326277
NFU1	67.85993785	0.001234647	0.605238651
NFX1	32.41510544	2.11E-05	0.513744014
NFXL1	9.909843842	0.004862993	0.637281321
NHEJ1	3.023460076	0.000610826	0.563114624
NHSL2	3.249053862	0.000599927	0.553648059
NIFKP7	0.029439225	0.000786324	1.770327967
NIPAL1	2.141656915	0.001614967	0.494281286
NIPAL3	27.21337945	1.96E-07	0.406522193
NIPAL4	0.619811412	6.03E-12	3.11023975
NIPBL	24.71586767	0.003353379	0.626627355
NIPSNAP1	143.6707972	0.000563129	0.581784602
NKAIN4	20.58417853	0.001305108	1.721163841
NKAPD1	31.52793419	0.000316636	0.573441665
NKIRAS1	9.715269697	3.92E-05	0.510173496
NKTR	15.06400387	0.004176955	1.537479687
NKX2-1	0.01645049	0.000781715	1.722153159

NKX2-2	0.246876095	0.000558144	2.155790324
NKX2-2-AS	0	0.004606615	1.557019068
NKX2-5	0.27826925	2.06E-07	2.375634206
NKX2-8	0.147580898	4.13E-06	2.260402493
NKX3-2	0.192359271	0.001051878	1.726831351
NKX6-3	0.063765656	0.000260414	1.846837506
NLGN2	18.19639203	0.002063303	1.592314807
NLGN3	1.110988388	0.004379634	1.535710525
NLGN4X	2.473986254	0.002688276	0.576857328
NLRP14	0.48000388	0.001869987	0.584362132
NLRP9P1	0.043143474	0.000495569	1.72316061
NMD3	56.90066568	4.97E-05	0.522929526
NME1	37.04496723	1.40E-06	2.05751288
NME1-NME	1.711362352	3.56E-05	1.861815566
NME2	79.52971305	1.10E-05	1.935467764
NME2P1	2.120183721	0.000954779	1.65101676
NME3	113.10868	0.001467126	1.619473057
NME4	92.82307109	0.000190288	1.756178569
NME8	1.21729756	0.003981084	1.542169152
NMNAT1	10.46116325	7.68E-07	0.439316604
NMNAT1P3	0.18072565	0.000561315	1.722904964
NMNAT1P5	0.014381	0.004859323	2.02215414
NMRAL2P	1.560171093	0.000601799	1.838881639
NMT2	22.27356736	9.33E-05	0.533210462
NMTRS-TG	0	0.004606615	1.557019068
NMU	4.0994353	0.000257803	1.829264174
NNT	52.21687359	8.82E-05	0.468363809
NNT-AS1	13.66745625	0.002862447	0.613923331
NOA1	31.64652046	1.15E-05	0.505060887
NOC4L	31.12472825	4.11E-05	1.861144478
NOD2	3.872271104	0.003637466	1.560294933
NOL12	5.208715797	7.11E-06	1.94980731
NOP14	40.11482228	0.001439513	0.6085497
NOP2	26.09698693	1.88E-09	2.439622976
NOP53-AS1	1.595672479	0.002679094	0.617656093
NOP56	53.44665475	0.000240366	1.744392682
NOP9	28.18423291	2.34E-05	0.508909708
NORAD	297.5624421	0.001118553	0.595590266
NOS1AP	0.448673803	0.000684566	0.546883794
NOS2P2	0.051876474	0.001169185	1.81932236
NOS2P3	0.030169581	7.66E-07	2.097532251
NOS2P4	0	0.004606615	1.557019068
NOSTRIN	33.94670593	0.001702569	0.612233752
NOTCH1	38.30626948	0.002700973	0.61512521
NOTUM	0.347343655	9.95E-05	2.262337164
NOX1	1.285066103	0.002653432	1.57787041
NPAS2	9.128840361	0.000289991	1.726331795
NPAT	12.36063293	6.59E-05	0.529465126
NPC1	30.52225666	0.004054977	1.567854161
NPEPL1	13.53591821	1.11E-16	3.267486352
NPFF	0	0.004606615	1.557019068
NPFFR1	0.363840496	0.000825818	1.725649621
NPFFR2	0.153562255	4.50E-07	2.224437955
NPHP3	5.30460843	0.00014346	1.769240059
NPIPA1	2.484305421	4.15E-05	1.845997136
NPIPA7	0.064919903	0.002854175	1.849083427
NPIPA9	0.13359818	0.000758231	1.671366682
NPIPB11	1.474967051	4.15E-06	2.007528255
NPIPB12	0.388938817	1.65E-05	1.901079403
NPIPB13	0.239369283	0.000591676	1.75525201
NPIPB14P	0.974136789	0.000215877	1.739860205
NPIPB3	1.978411791	0.000116466	1.776807542
NPIPB4	1.782007512	0.000933535	1.645002016
NPIPB5	2.282243197	9.57E-08	2.202664804
NPIPB6	0.231849546	8.74E-08	2.232581189
NPIPB9	0.06795203	0.001928189	1.601832846
NPIPP1	9.856905099	1.75E-05	1.899277975
NPLOC4	50.31527549	0.000122234	1.790252982
NPM1	739.4235398	0.003394691	0.624763558
NPM1P10	0.067140986	0.000366233	1.766525484
NPM1P11	0.158712626	0.000249325	1.816088245
NPM1P18	0.237274789	0.000206141	0.531634106
NPM1P21	0.457987676	0.00382256	0.616175853
NPM1P23	0	0.004606615	1.557019068
NPM1P24	0.604888718	0.003566569	0.612268488
NPM1P27	16.57060041	0.000117447	0.518789766
NPM1P28	0.032621223	0.000141054	1.894571859
NPM1P43	0.114243375	0.003124557	1.593596495
NPM1P9	0.356706242	0.000506458	1.688561442
NPM3	36.62994293	6.46E-06	1.96950327
NPNT	39.92348347	0.00025381	0.519930601
NPR3	115.2251148	1.06E-06	0.434424785
NPTX2	292.3679487	0.002303143	1.610591942
NPW	0.350764834	7.92E-09	2.400272291
NPY4R2	0.062974012	0.000217002	1.818521534
NPY6R	35.93873394	2.24E-06	0.454726001
NR1H5P	0.013918335	0.001267553	1.790317907
NR1I2	0.439566216	0.000838033	1.683537801
NR2E3	0.420592759	0.000746609	1.672700298
NR3C2	11.61906582	8.67E-07	0.398489416
NRAS	85.79680049	0.002826674	0.630144901
NRBF2P3	0.099581252	0.001560985	1.632215732
NRBF2P5	1.131038633	0.002912635	1.568085838
NRBP2	48.36154349	0.001426291	1.620642908
NREP-AS1	0.062121886	0.004421026	1.59662813
NRG4	0.894710465	0.000468314	1.868942635
NRIP1	37.4403383	4.51E-05	0.512602783
NRIR	1.314067297	0.000287514	1.722864354
NRN1	42.89266511	0.004933593	1.620692928
NRP1	178.8868561	0.001209646	0.603162096
NRSN1	0.097218005	0.0002947	1.783728391
NRSN2	75.37920084	0.001594379	1.612801912
NRXN2	14.69316839	0.003062795	0.60956609
NRXN3	3.5387871	0.001823493	0.54407414
NSA2	94.05435452	0.003143813	0.615504581
NSD1	24.21128524	0.000181384	0.546963694
NSL1	28.12793706	0.001389305	0.610567157
NSMCE1-D'	0.518406851	3.46E-05	1.855449086
NSUN4	13.28547958	0.003325747	0.631493277
NSUN5	20.58474382	1.99E-06	2.041206686
NSUN5P1	9.134760993	2.52E-07	2.149217338
NSUN5P2	2.04154652	2.53E-05	1.879974132
NSUN6	10.61915814	0.004171518	1.541086758
NT5DC1	26.22043075	2.61E-06	0.468953251
NT5DC4	0.175243533	5.41E-05	1.901267312
NTF4	0.076051028	8.67E-05	1.999234039
NTF6A	0.012812479	0.002055433	2.11560495
NTF6G	0.033747077	0.002547269	1.767120882
NTM	9.495225816	0.00373826	1.558867754
NTN4	125.2319731	7.55E-05	0.514745779
NTN5	0.705849361	0.002661422	1.579212612
NTNG1	0.353497986	0.004607178	1.709224364
NTNG2	1.84690979	8.96E-05	1.829199133
NTRK1	0.475155712	0.001190804	1.654429874
NUBP1	47.98465638	0.001388761	0.614077186
NUBP2	32.97442421	0.004136688	1.544716437
NUBPL	7.188970712	1.98E-06	0.458760835
NUCB2	37.18873025	0.002315048	1.586172691
NUCKS1	218.9783731	9.70E-05	0.541869308
NUDT1	18.74824221	1.19E-05	1.93150933
NUDT11	1.266950237	0.000115159	1.881596935
NUDT12	30.54256823	2.06E-06	0.461627352
NUDT17	8.580882994	3.14E-05	1.868876173
NUDT4	17.86319909	0.003409993	0.605140646
NUDT5	41.81396149	0.000694961	1.670034632
NUDT7	10.52669944	0.000356781	0.564055965
NUDT9	39.65010418	3.95E-05	0.492706549
NUF2	3.126369757	5.22E-15	3.097686992
NUFIP1	10.19976464	5.58E-05	0.527717996
NUMB	42.62699352	2.49E-06	0.460309352
NUMBL	9.089463969	3.50E-11	2.639898951
NUP133	30.96942345	0.000244704	0.564717263
NUP153	41.09522723	0.004748763	0.641612144
NUP160	30.04371774	0.000528646	0.580461225
NUP205	28.54311156	0.001406962	0.600541137
NUP210L	0.1919204	5.29E-05	1.841159622
NUP214	25.68121675	0.002768419	0.631295702
NUP50-DT	6.872520735	7.40E-06	1.947902454
NUP98	39.58905271	0.000937874	0.593503923
NUPR1	119.4509734	0.001294405	1.65317516
NUS1	34.39538514	0.004601933	0.644034463
NUS1P1	5.716075614	0.00296597	0.627214067
NUTF2	85.87397707	3.98E-09	2.411378483
NXF3	0.267009868	6.71E-05	1.822218459
NXF4	0.024995092	0.002440768	1.652846187
NXNL2	11.84116317	0.001253543	0.592389267
NXT1	47.48052179	0.000292325	1.727581219
NYAP2	0.043051799	0.000133381	1.793770544
NYNRIN	14.6912407	0.000607693	0.554277286
OASL	13.27678458	0.002563081	1.581545617

OBP2A	0.413922057	1.84E-06	2.153546257
OBSCN-AS1	1.209127762	0.001054882	1.636511701
OCIAD1-AS	3.664329727	6.66E-08	2.222167948
OCIAD2	199.1914604	0.001777087	0.62012271
OCLN	5.145110804	0.000939208	0.546166837
OCM	0.476744115	0.00053393	1.68620052
ODAPH	0.354673153	0.000164255	1.870353958
ODC1-DT	2.530665836	2.18E-05	1.891410367
ODCP	0.422146652	0.00122921	1.63203401
ODF2-AS1	0.912471748	1.27E-06	2.055047259
ODF2L	8.265370499	0.004579807	1.533471514
ODF3B	49.37502844	0.002867397	1.572751578
ODF3L1	2.096634557	0.001223885	1.643058997
OFD1P13Y	0	0.004606615	1.557019068
OFD1P17	0.105216662	0.003995229	1.564045838
OGDH	209.6254803	0.000436398	0.550591047
OGFOD2	1.895668853	0.00195772	1.594483298
OGFR-AS1	1.649083109	0.000347573	1.712789837
OIP5	3.605263963	9.22E-05	1.802342898
OIT3	7.037619177	0.000165757	0.523926163
OLA1P3	0.045558732	6.92E-05	1.834156535
OLFML2A	109.8521419	0.000411837	0.555158196
ONECUT2	0.449284339	0.000371258	1.981803438
ONECUT3	0.058574001	0.000825005	1.738217881
OOSP1P1	0.055848038	0.001369889	1.969542404
OOSP4B	0.032494696	0.003179646	1.936188372
OPA1	34.78946773	0.000654983	0.581169288
OPA3	17.91781041	5.50E-05	0.522892915
OPCML	5.608657382	0.001035491	0.518994299
OPHN1	4.547116265	5.06E-05	0.50858767
OPN1MW2	0	0.004606615	1.557019068
OPN1MW3	0	0.004606615	1.557019068
OPN1SW	8.615223704	2.33E-05	1.886370122
OPRD1	0.281460856	4.28E-07	2.119222708
OPTC	0.040273227	0.00031395	1.756839235
OPTN	141.9939747	0.000506381	0.582717514
OR10A6	0.00147068	0.002106113	3.670113532
OR10T1P	0.0149494	0.000147285	2.101156658
OR10X1	0	0.004606615	1.557019068
OR11A1	0.032939626	1.88E-05	1.993986005
OR11H13P	0.044519865	0.002114605	1.633357906
OR11H7	0.390832573	0.000801632	1.663088734
OR11Q1P	0.014662595	0.000462054	2.115847935
OR13A1	0.258339349	3.22E-11	2.749502834
OR13E1P	0	0.004606615	1.557019068
OR13J1	0.12094477	0.003476888	1.570156359
OR1E3	0.007102363	0.001658702	2.184492807
OR1J1	0.065132399	0.000454489	1.71313473
OR2B6	0.263429881	4.97E-06	1.991112451
OR2H1	0.059884453	7.27E-05	1.972644836
OR2H2	0.060701349	1.89E-09	2.430304121
OR2K2	0.024737068	0.001020484	1.731308399
OR2L8	0	0.004606615	1.557019068
OR2U2P	0.030540973	1.02E-05	2.155840669
OR3A4P	0	0.004606615	1.557019068
OR4A47	0.029128103	0.001496385	1.939680794
OR4C16	0	0.004606615	1.557019068
OR4C6	0.122337121	0.00046079	1.994061684
OR4D1	0.020395556	0.000234465	1.907085981
OR4K2	0.055316862	9.03E-06	2.021926595
OR4N4	0	0.004606615	1.557019068
OR4X1	0	0.004606615	1.557019068
OR51J1	0.047711949	0.001098644	1.654251622
OR51N1P	0.051313486	0.002031321	1.660806879
OR52B3P	0.263721362	2.45E-06	2.022390795
OR52K3P	0.805990836	0.003423827	1.571337702
OR52R1	0	0.004606615	1.557019068
OR5A1	0.05715992	1.15E-05	2.015470832
OR5AL1	0.000255056	5.10E-06	22.83636517
OR5AP2	0.025004162	0.004935109	1.701133102
OR5C1	0.10758918	0.002183161	1.681593046
OR5D13	0	0.004606615	1.557019068
OR5F1	0.010517585	0.000157045	2.186362741
OR5H6	0	0.004606615	1.557019068
OR5J1P	0.036855038	0.000103862	1.905027513
OR5J7P	0.007284092	0.001771778	2.782692875
OR5R1	0	0.004606615	1.557019068
OR5W2	0.02371434	7.35E-05	2.077345774
OR6S1	0.058055787	0.001493375	1.691024336
OR6W1P	0.07380473	0.000239966	1.789450579
OR7E100P	0.240054603	0.000329898	1.78409406
OR7E101P	0.026033126	0.001090339	1.887449859
OR7E102P	0.24030697	0.001620202	1.627256605
OR7E140P	0.029707639	0.000387736	1.789095522
OR7E161P	0.156437274	0.000570092	1.687537891
OR7E26P	0.024610795	0.001783174	1.756811705
OR7E38P	14.09438377	1.30E-06	2.062447465
OR7E39P	0.066083498	0.000421469	1.888599077
OR7E55P	0.055369372	0.001189871	1.635072989
OR7E62P	0.165108709	0.001476972	1.652422354
OR7G2	0.021035361	0.003419925	1.736452627
OR7M1P	0.367405802	0.001922633	1.609423968
OR8A3P	0.070335347	0.00163016	1.848890003
OR8B5P	0.065584614	3.80E-05	1.92771599
OR8B7P	0.091231818	5.52E-05	1.898503423
OR8H2	0.00819931	0.000125028	2.300527361
OR8K3	0	0.004606615	1.557019068
OR9K1P	0.011323418	0.002813155	2.034006548
OR9N1P	0.231855788	0.004114627	1.56620406
OR9S24P	0.071452604	0.001636995	1.787791924
ORAI3	55.4005732	0.003006151	1.567418571
ORAOV1P1	11.39374666	1.41E-07	2.177673275
ORC1	2.895605856	0.002440475	1.584738297
ORC4	17.2926909	6.89E-08	0.414371812
ORC6	2.178009105	8.82E-10	2.472445592
ORM1	17.18399379	0.000596873	4.800716984
ORM2	12.10635256	0.001062078	5.444564488
ORMDL1	37.187594	0.000145591	1.768511108
OSBP	79.96488374	1.23E-08	0.401311557
OSBPL10-A	0.149283603	0.000295234	1.762104991
OSBPL1A	42.72420112	1.99E-06	0.469847636
OSBPL8	43.56436891	0.002586097	0.624194362
OSCAR	10.702677	0.000307896	1.724259748
OSCP1	16.81142656	0.001085336	0.599643016
OSMR-AS1	1.945806417	0.000144936	1.767121229
OSR2	2.367512646	0.001310993	1.644434294
OTOA	0.519623488	0.000320785	0.543033927
OTOF	0.213097049	4.24E-08	2.259764224
OTOG	0.166751465	0.000969296	1.653825729
OTOGL	1.078547609	0.000383216	0.520026756
OTP	0.044291348	1.31E-07	2.221850712
OTUD1	46.4894073	1.80E-05	0.460297597
OTUD6B-AS	19.47958894	0.000128557	0.534748869
OTUD7A	1.960255862	1.92E-05	0.471499581
OTUD7B	12.42943182	0.0040368	0.633516698
OTX1	0.189238315	4.56E-09	2.675251773
OTX2	0.022674692	0.002506632	1.67342785
OVCA2	0	0.004606615	1.557019068
OVGP1	6.065083345	6.59E-05	1.924976407
OVOL1	2.96851388	0.00208038	0.572011092
OXLD1	35.59425861	0.000131392	1.780402997
OXR1	33.45591281	0.00325316	0.613319065
OXSR1	38.74969302	0.000617899	0.583164201
P2RX5	0.785488804	0.000208267	1.776989912
P2RX6	0.361188753	6.71E-06	1.999334803
P2RX6P	0.178500233	4.91E-06	2.069316709
P2RY8	33.13548547	0.000709558	0.574164384
P3H1	27.31688622	2.22E-12	2.800915783
P3H2-AS1	5.923345831	0.000605901	1.692683274
P3H3	13.97426616	0.00067554	1.739384118
P3H4	38.29792832	0.000248853	1.736443484
P4HA3	8.894797597	0.000302649	1.800333079
PA2G4	128.960999	0.00048774	1.696757242
PA2G4P4	1.066889032	0.000120906	1.794785956
PABPC1L	18.57142662	2.55E-11	2.643603096
PABPC1P3	2.854803	2.86E-08	2.277511016
PABPC1P8	0.006030693	0.004780383	1.748616008
PABPC4L	9.320468182	0.000740741	0.576643257
PABPN1	71.98436597	0.000134167	1.768444467
PACERR	0.707078542	3.16E-08	2.311591396
PACSIN2	105.0957643	3.74E-06	0.457243678
PADI3	4.370832021	8.54E-06	2.609926215
PAEP	21.83024916	3.32E-07	2.871047683
PAFAH1B1	77.89456143	0.00346029	0.635908567
PAFAH2	28.36832366	1.22E-07	0.420398824
PAGE1	0.862498948	5.17E-06	4.881757189
PAGE5	4.251218427	0.002660494	4.849953733
PAICSP2	0.020474983	0.003416007	1.709743671
PAIP1	61.22455197	0.001116492	0.603427407
PAIP1P1	1.435545997	9.09E-05	1.795258953

PAIP2	107.039725	6.17E-07	0.456519397
PAIP2B	12.0235399	1.65E-06	0.400072003
PALM2-AK.	0.515073945	0.000858791	0.577025295
PAM	169.2325857	5.08E-05	0.524278257
PAM16	12.74489341	3.03E-05	1.869758763
PANK1	12.87997514	3.30E-10	0.320968325
PANK3	27.15564735	0.000923758	0.589444646
PANO1	1.126234868	0.0001341	1.777035072
PANX2	2.651120912	1.58E-11	2.77792337
PANX3	0.048844168	0.000771746	1.704885518
PAPPA-AS1	0	0.004606615	1.557019068
PAQR4	10.31953239	0.003259487	1.566428659
PAQR5	31.3408788	4.85E-09	0.366595527
PAQR6	6.369027574	0.000321471	1.727278272
PAQR7	34.37414384	0.00259812	0.620174349
PARAL1	0.173659873	4.75E-05	1.870559809
PARD3-AS1	4.917754857	0.000451565	1.705072759
PARD3B	7.98521239	6.57E-05	0.515578623
PARP15	2.658563952	0.001604321	1.612430025
PARP4	75.2242791	3.54E-05	0.520180803
PARP6	26.71059631	0.000761023	1.658831564
PARPBP	2.379459914	5.71E-05	1.834932059
PARS2	11.56288588	0.000342998	0.561602061
PATJ	35.87175477	4.12E-07	0.41856294
PATZ1	32.81618011	0.000204444	0.558192809
PAUPAR	0.014162555	0.000427751	1.839951161
PAX1	0.025583573	0.001786747	1.656849813
PAX3	0.03578871	1.13E-06	2.55577797
PAX9	0.849301115	0.003282957	2.768205791
PAXBP1	17.00218586	0.002429879	1.579017786
PAXX	54.95545451	2.45E-05	1.885407146
PBK	5.570718424	0.004288094	1.551361157
PBLD	73.16466996	2.75E-05	0.501419494
PBOV1	0.041040396	0.004432588	1.605221706
PBRM1	16.2115275	0.000778257	0.577894836
PBX1	11.69277538	0.000705764	0.566410758
PBX2P1	0.387131936	0.001991651	0.606339078
PBX4	4.071727783	9.05E-08	2.205996109
PBXIP1	207.8735946	1.65E-06	0.45632174
PCAT5	0.222979565	0.000210197	2.071965547
PCAT6	10.54206221	0.000153705	1.778373856
PCBP1	518.9610739	9.37E-12	0.331087504
PCBP3	1.602199722	0.000262018	1.881179341
PCBP4	15.6797274	0.000269118	1.733063248
PCCA	31.24588071	3.11E-09	0.368000668
PCDH1	41.9720156	0.000969898	0.570629221
PCDH11Y	0.062922018	5.34E-05	0.473563461
PCDH12	51.19018834	0.002971329	0.616573607
PCDH17	28.02340625	0.00289606	0.614224343
PCDH19	0.473831012	0.000478122	0.510957606
PCDH8P1	0.011116152	0.000164218	1.894141966
PCDHA14	0	0.004606615	1.557019068
PCDHAC1	0.385684182	0.000135308	0.521767147
PCDHAC2	1.056816246	0.003567855	0.623627717
PCDHGB8P	0.393731367	0.002540737	1.582642218
PCDHGC3	26.82096606	0.000896907	0.59433631
PCDHGC5	0.611237432	1.11E-05	1.930194736
PCED1A	84.42575551	0.000140308	1.767095277
PCF11-AS1	0	0.004606615	1.557019068
PCGF5	50.55997987	6.03E-08	0.412921064
PCK1	96.8318531	0.000918638	0.532301562
PCK2	47.23381613	0.001588411	0.592088522
PCLAF	6.522340334	0.000355344	1.715502638
PCM1	34.86179099	0.000274295	0.557509077
PCMT1	73.72139129	0.004500425	0.644845077
PCMTD2	56.16123922	0.001026503	0.592729712
PCNAP4	0.024894081	0.002876636	1.724620929
PCNP	190.1172085	0.000720002	0.586956904
PCNPP1	1.281692732	0.001345861	1.623578619
PCNPP3	0.208618559	2.35E-05	2.234686523
PCNX1	17.15263019	3.44E-06	0.458297279
PCNX2	5.063668581	0.000733987	1.659364299
PCSK1	1.477589434	0.000466194	1.928430387
PCSK4	4.752589514	0.002505585	1.580342292
PCSK5	8.233486627	1.13E-05	0.477292406
PCYOX1	130.2974254	4.01E-07	0.443128551
PDAP1	137.5999547	0.002652742	1.578855452
PDCD2L	9.480071769	0.003892778	1.549106246
PDCD4	67.0734591	0.000258407	0.553482742
PDCD5	83.74044513	5.65E-10	2.537010851
PDCD6IP	54.6718063	4.74E-06	0.455940392
PDCL	34.48656019	3.49E-05	0.507482795
PDCL3P4	8.17398775	0.000438024	1.704676309
PDCL3P6	0.101581623	0.001492557	1.617429247
PDE12	11.91369354	0.001263692	0.58381054
PDE2A	23.54534909	0.000190393	0.531556493
PDE4D	14.50435249	0.001797064	0.603413156
PDE6C	0.734529353	0.000308619	1.716059509
PDE7B	11.43526545	0.001416045	0.593796232
PDGFB	77.8600543	0.000537117	0.579776573
PDGFC	33.96850049	0.000757376	0.574020983
PDGFD	104.62853	1.85E-05	0.498493972
PDGFRL	7.451007914	1.75E-10	2.640862216
PDIA2	1.113411085	2.86E-05	2.434171386
PDK2	42.69585517	0.000123422	0.544748104
PDK4	734.7666324	0.000917133	0.524118307
PDLIM7	42.55260326	0.000408073	1.703278705
PDP1	22.63879779	0.000732944	0.546362031
PDPK1	14.75271447	2.18E-07	0.431400875
PDPN	5.405385626	0.002906755	1.708209576
PDS5B	12.61954184	6.27E-05	0.523763786
PDSS2	25.38368103	0.001721344	0.606176637
PDZD2	17.55798105	0.000277762	0.535117
PDZD7	1.519777078	0.000764411	1.667938966
PDZD8	26.2801685	0.000206177	0.539356566
PDZK1	180.3014753	3.63E-06	0.473999538
PDZRN3	7.531472076	1.88E-05	0.490651772
PEAK1	14.75176096	0.00109461	0.586848574
PEBP1P2	0.969620488	0.000166089	0.524647629
PEBP1P3	0.326239679	0.000141655	1.792069297
PECR	43.67869397	1.04E-07	0.423188883
PEG10	31.28543462	0.002656722	0.608610026
PEG3	2.385675893	4.50E-06	0.395413251
PELI2	9.487072508	4.01E-05	0.504201935
PEPD	103.0365605	0.00182536	0.59044457
PER2	30.90073148	0.002229316	0.60318798
PER3	20.8185015	0.002890322	0.611017948
PEX10	23.86929513	0.004139017	0.643309538
PEX11A	24.66720677	0.000145818	0.545565711
PEX12	18.00186955	3.70E-07	0.44570809
PEX12P1	0.030723188	0.001479223	1.797352057
PEX19	73.83298025	5.03E-06	0.491497039
PEX2	33.43173213	0.001759648	0.60790052
PEX3	32.47435925	0.000181893	0.546448024
PEX5	42.8548564	0.000661248	0.583558018
PEX5L	0.460398785	0.000257932	1.889729433
PEX7	20.76865866	2.57E-05	0.494552625
PF4V1	12.80908991	0.001209985	1.672036536
PFDN2	167.9331374	3.96E-06	1.995963752
PFDN4	32.36013236	0.000377083	1.711075556
PFDN6	37.92362082	0.002903091	1.57178612
PFKFB1	0.599242332	0.000198051	1.78233496
PFKFB3	177.7752168	0.004430836	0.628983399
PFKP	472.8747744	2.71E-05	0.515537296
PFN1P2	1.195980647	0.000476799	1.690064854
PFN4	2.651735598	1.35E-05	1.920543379
PGA4	0.019560022	0.003190426	0.548846583
PGAM1	127.8803989	0.004902407	0.646209605
PGAM1P7	0.301652474	1.92E-05	1.904695632
PGAM2	0	0.004606615	1.557019068
PGAM4P2	0.051936816	0.002355951	1.683082273
PGAP1	6.044746956	0.002157111	0.611207088
PGAP3	47.78847614	0.003458098	0.628782864
PGBD4P3	0.951510911	0.000585414	0.575253251
PGGHG	104.2088766	0.00018949	1.778630371
PGGT1B	14.07629025	0.001704221	0.609520719
PGK1	812.3131491	0.004648806	0.645345453
PGLYRP2	0.688802127	0.0009558	1.753261974
PGLYRP3	0.021178746	0.004447061	1.624317374
PGM2	34.09320328	0.000203689	0.557481386
PGP	17.24582791	0.000689525	1.671095874
PGPEP1	51.16571193	0.000103476	0.536855829
PGRMC1	642.3361872	0.001195719	0.597849285
PGRMC2	128.9829831	0.001918008	0.615641757
PGS1	23.49944961	6.40E-05	1.828067981
PHACTR2	18.4196555	4.56E-05	0.49995948
PHAX	40.37366797	1.07E-05	0.500598565
PHBP19	0.661559614	2.66E-05	1.870447553
PHBP20	0.232285231	0.000458572	1.695758578
PHETA1	18.93708912	0.001029796	1.64348504

PHEX-AS1	0.161141554	0.001558852	1.657680862
PHF2	35.52881547	0.001436869	0.604724723
PHF21A	17.08225124	0.000898469	1.65383405
PHF3	39.22113997	3.15E-05	0.513974304
PHKA1-AS1	0.154509342	0.002220136	1.63994417
PHKB	30.52958714	8.35E-06	0.482780788
PHKG1	2.034959266	8.48E-05	1.803578245
PHKG2	13.05157258	7.65E-05	1.811376698
PHLDA2	18.12302517	0.003002171	1.569179965
PHLDB2	35.95789564	2.63E-06	0.465347387
PHLPP1	6.777806556	0.000505078	0.554028962
PHOSPHO2	7.196654345	6.46E-06	0.468034725
PHOX2B	0.025431244	0.000179448	1.875516352
PHTF1	10.40051405	0.002337491	1.58286274
PHYH	71.41677124	5.51E-05	0.511408685
PHYHIP	1.365381842	0.000193581	1.767074176
PHYHIPL	22.47038056	2.31E-05	0.485788605
PI3	15.44554039	3.39E-06	2.29241903
PIAS1	17.94495535	5.45E-05	0.529718937
PICALM	225.7894212	0.004119938	0.635223131
PICART1	1.615323427	0.000801041	1.667955948
PIDD1	10.2039424	0.000148489	1.762851467
PIEZO1P1	0.016336239	0.002409364	2.583527251
PIEZO2	10.13411871	0.001098446	0.579940403
PIF1	1.347454643	1.40E-07	2.178254464
PIGCP1	18.11936703	0.001660647	0.60534893
PIGH	24.01801382	9.88E-05	0.53449506
PIGK	36.28655726	0.004782102	0.644880157
PIGL	6.179050471	0.000417915	1.693909695
PIGO	24.94258654	2.77E-06	0.467655413
PIGP	12.10707581	0.000465831	0.571632399
PIGU	36.70340907	0.000872642	1.65476541
PIGV	25.15234091	1.68E-07	0.428190331
PIGY	0	0.004606615	1.557019068
PIGZ	4.389212905	0.00032827	1.732825393
PIH1D2	6.206298989	0.004052652	0.63934798
PIK3C2A	41.77243192	2.72E-07	0.415948857
PIK3C2B	18.4381393	0.00163605	0.601112164
PIK3C3	13.25826286	0.001367958	0.602187693
PIK3CA	15.99483871	0.000115453	0.535895585
PIK3CB	39.09712052	2.68E-05	0.510130755
PIK3IP1-AS	1.886764712	0.001050025	1.644735796
PIK3R1	44.05970024	0.002528644	0.607160651
PIK3R2	0.928105527	0.000251321	1.745675232
PIK3R3	44.90699628	0.000421723	0.559405301
PIK3R4	28.00845522	1.56E-06	0.467131227
PIK3R6	6.164262277	0.001201416	1.658702792
PIKFYVE	17.07853538	0.00066857	0.577135276
PIM1	64.44843228	0.000375836	1.713901399
PIM2	44.80884584	9.90E-05	1.824781374
PIMREG	3.278896916	1.97E-08	2.299381679
PINK1	40.55915814	1.71E-10	0.354190225
PINK1-AS	7.111136666	0.001797481	0.613786188
PIP5KL1	0.769919944	1.70E-10	2.581866181
PISD	32.38160981	1.78E-05	1.90076471
PITPNA	89.35198797	0.002635657	0.623740686
PITPNC1	29.94572009	0.0006951	0.589901375
PITX1	1.910805332	1.07E-07	2.51886398
PITX2	2.041819137	0.003240918	1.631924807
PJA1	30.69458123	0.004688837	0.643040773
PJA2	176.5516402	4.18E-05	0.523756287
PJVK	3.05098344	2.52E-05	1.86941407
PKD1L3	0.178357157	0.001871015	1.604331242
PKD2	93.36855138	0.000232793	0.556894382
PKD2L2	0.192973761	1.16E-06	2.059955178
PKHD1	16.96382485	8.78E-05	0.52712268
PKIA-AS1	0.16623915	5.26E-05	2.11170343
PKLR	33.58950418	0.000747452	0.564179636
PKMP1	0.58339745	0.001796719	0.596816655
PKMP2	0.070329949	0.004795264	0.62368317
PKMYT1	2.22004915	3.67E-05	1.864328253
PKN2	36.89310325	0.00186438	0.617334927
PKP3	4.935123759	4.36E-05	2.03556952
PKP4	65.76098379	0.00013487	0.537471157
PLA2G12A	38.2968196	0.00221567	0.599615032
PLA2G2A	4.569025259	7.45E-05	2.131723662
PLA2G2F	0.02853054	0.004519587	1.659944789
PLA2G4B	0.563285868	0.001040963	1.638006444
PLA2G4C-A	0.718813314	2.00E-05	1.89191387
PLA2G4E	0.044700312	0.002785757	1.58852143
PLA2G6	10.12743417	0.001403299	1.616465862
PLAA	30.12297813	0.001914998	0.616666729
PLAC1	0.160041369	7.85E-12	2.767490071
PLAUR	22.90269526	1.08E-07	2.212663416
PLB1	2.689097858	0.000574875	1.682809004
PLBD1-AS1	6.271565724	0.000329879	0.541760993
PLCB1	17.0998808	2.16E-06	0.462092529
PLCB2	15.32843722	0.00331799	1.558869371
PLCG1-AS1	1.326922293	0.004014895	1.545452537
PLCL1	11.49125843	5.75E-05	0.46739316
PLCL2	19.50372061	3.47E-08	0.383808496
PLCXD1	7.063123587	3.34E-06	1.994923702
PLEKHA3	7.421495695	0.000928143	0.590974328
PLEKHA7	19.27791851	9.73E-06	0.479248612
PLEKHA8P	6.029019393	6.02E-05	1.830212571
PLEKHB2	116.6262797	0.004067713	0.622174656
PLEKHF1	11.8455074	0.000276056	1.733819954
PLEKHF2	39.29984727	3.84E-06	0.474988651
PLEKHG4	10.38958864	0.001302872	1.641705607
PLEKHG4B	0.800244255	5.85E-09	2.631929439
PLEKHM1P	5.359798799	0.002332124	1.58048557
PLEKHM3	3.613324667	3.45E-05	0.492125194
PLEKHN1	5.027014362	0.002398697	1.58545875
PLEKHO1	34.85995099	8.46E-05	1.809770937
PLEKHS1	0.857191537	5.97E-07	2.408872119
PLG	14.67415755	0.000581314	0.426365339
PLIN2	888.3129535	0.000187601	0.550190843
PLIN3	87.81586568	0.000672397	1.672894824
PLIN5	2.092997094	0.000226332	1.800981804
PLK1	5.248582761	2.19E-14	3.036099039
PLOD2	187.7689471	0.0002267	1.751558086
PLP2	299.2656519	0.000741608	1.665209615
PLPBP	80.52105599	0.000137176	0.555995526
PLPP1	360.5795054	0.000470491	0.574784758
PLPP3	264.7878809	3.46E-08	0.3998106
PLPP5	31.91225129	4.08E-05	1.86156281
PLPPR3	0.24018184	6.07E-08	2.333888094
PLRG1	38.80676935	2.43E-06	0.474057777
PLS1	31.28018227	1.70E-06	0.456993438
PLSCR5	0.015079884	1.54E-06	2.126738891
PLTP	208.4992421	5.59E-07	2.139080061
PLVAP	1568.699773	0.000888837	0.581233876
PLXNA2	23.45079479	0.00013259	0.529895943
PLXNA4	0.776529994	0.000190081	1.905083066
PLXNB3	2.0869937	2.06E-09	2.489278258
PM20D2	24.7098072	0.001241283	0.594518422
PMAIP1	7.487806722	8.99E-05	1.862775569
PMF1-BGLA	4.346400254	0.001682553	1.610430413
PMFBP1	0.801466976	0.001246615	1.646239167
PMM1	50.90429359	0.001469775	0.59905997
PMS1	10.28103925	0.003536843	1.554925151
PMS2P3	6.093654611	0.00081087	1.654445532
PNKD	48.60575665	5.82E-05	1.83205145
PNKP	27.22440196	1.72E-07	2.182772426
PNLIP	0.103311676	0.000975302	1.826662433
PNMA1	87.2304907	0.00102875	0.596934081
PNMA2	66.54137356	0.000273872	0.561308559
PNMA5	0.192644103	0.000778883	1.698634905
PNPLA4	30.31160518	0.001481042	0.591764211
PNPLA8	31.9266457	9.56E-05	0.498512607
PNPT1P2	0.016418433	0.00213165	1.693045496
PNRC2	113.9346829	0.001071431	0.597930875
POC1A	4.849166874	2.20E-05	1.904694681
POC1B	10.84059986	1.41E-06	0.466329269
POC5	10.91330025	0.003219039	1.562746099
PODNL1	3.137569044	1.55E-06	2.107306776
PODXL	211.8933031	5.88E-07	0.435681952
POFUT2	28.45245362	2.94E-07	2.149147779
POLA1	11.51775127	0.000199044	0.554600516
POLD1	13.76006545	0.000731541	1.664695314
POLD4	26.82780779	2.49E-05	1.883767925
POLDIP3	106.2691224	0.000867983	0.594551026
POLE3	88.16387437	8.81E-05	0.546692151
POLG2	12.27811172	0.003305154	1.55599728
POLK	16.4623796	8.49E-06	0.483155968
POLM	23.6088193	0.000650325	1.671874594
POLQ	0.88255382	6.87E-07	2.100111083
POLR1B	18.98522182	0.001786622	0.613710719
POLR1D	56.30764316	0.001916965	0.614983587
POLR1E	37.11856483	2.03E-06	0.455313966

POLR2A	118.5624805	4.17E-06	0.482026788
POLR2B	55.75202445	0.000631418	0.588483389
POLR2C	100.3087271	4.27E-06	0.494305404
POLR2G	93.0707827	9.98E-06	1.963770438
POLR2H	58.64821077	7.16E-05	1.822755456
POLR2J	88.61413662	0.000509642	1.691135148
POLR2J3	1.501157439	0.001978419	1.598576518
POLR3DP1	0.054125086	7.53E-05	1.81484858
POLR3E	13.02615989	0.0040766	1.548357391
POLR3G	2.286536403	0.000503239	1.691602969
POM121L2	0.056662242	0.000544874	1.714840282
POM121L9P	0.550133019	0.001188136	1.657831061
POMK	3.964663015	0.001643574	0.591021904
PON2	101.8159418	7.54E-06	1.966171947
POP5	30.70366343	5.59E-07	2.12555887
POP7	86.17858939	5.66E-05	1.841695215
POPDC3	0.704500959	1.67E-07	3.49037423
POU2AF1	2.763549173	0.00173594	1.692393032
POU3F2	0.062727566	1.15E-06	2.099702165
POU5F1P3	1.4273507	0.002218616	1.582055144
POU5F1P4	0.204691037	0.000473106	1.706413226
POU5F1P6	0.434630731	0.001205974	1.636003662
PPAN	7.718734172	1.08E-05	1.931530249
PPARA	18.42473366	3.13E-06	0.456991126
PPARG	25.05049349	0.000388109	0.565817451
PPEF1	1.195414898	0.003981207	1.59042431
PPEF2	0.079148116	0.000529275	1.700476693
PPFIA1	22.13700577	0.000318489	0.571659089
PPFIBP1	57.18795949	5.61E-05	0.518225486
PPFIBP2	17.93802096	0.003648458	0.604898732
PPIAP27	0.168867048	0.000449408	1.778757311
PPIAP40	0.734839378	3.54E-08	2.267435432
PPIAP72	0.903020504	0.001628044	1.613098167
PPIAP83	0.054777783	0.00212264	1.633728993
PPIAP85	0.127920364	0.001952185	1.621441889
PPIAP90	0.650400716	0.000405553	1.706128
PPIB	479.3550504	4.09E-06	2.006363294
PPIF	87.73407119	0.001380915	1.627401092
PPIH	42.46010088	4.96E-05	1.849310363
PPM1A	28.05078214	2.27E-06	0.452436676
PPM1D	22.82325304	9.54E-07	0.456847914
PPM1J	1.261064349	0.000589839	1.698469802
PPM1M	30.75906505	0.000379811	1.712205248
PPM1N	1.663556918	1.36E-07	2.192207284
PPOX	15.35939605	0.000314631	1.720535
PPP1CB	200.7926238	6.21E-05	0.523649259
PPP1R11	122.5453246	0.000510065	0.586832538
PPP1R12B	15.58518168	0.001783519	0.580943166
PPP1R13B	14.70943562	0.003537248	0.629438472
PPP1R14B	111.3146326	9.92E-07	2.082855351
PPP1R14BP	71.61696948	2.54E-06	2.023120462
PPP1R15B	80.59776522	1.46E-05	0.453363217
PPP1R16B	23.49654629	0.000303248	0.539877926
PPP1R18	81.88717364	3.25E-05	1.889004593
PPP1R1A	29.17304647	7.05E-07	2.203248538
PPP1R1AP2	0.08871548	0.001038207	2.357476737
PPP1R21	40.80408472	5.12E-06	0.480435039
PPP1R27	0.177066828	0.001413889	1.667354188
PPP1R2P4	0	0.004606615	1.557019068
PPP1R32	4.053822786	8.27E-07	2.074026243
PPP1R35	42.90179125	5.44E-06	1.975327704
PPP1R3D	12.65896607	3.39E-05	0.523561638
PPP1R8	52.59069996	0.003904328	0.640877888
PPP1R8P1	0.276260284	0.001557588	1.628535319
PPP2CA	108.7927347	0.00066141	0.584831596
PPP2CB	104.5581287	2.39E-06	0.469611705
PPP2R2A	26.68554849	0.001908982	0.615500075
PPP2R2C	1.446791644	6.79E-09	2.55223781
PPP2R3A	42.56549971	0.00032864	0.560198018
PPP2R3B	4.104930072	3.49E-06	1.993660378
PPP2R5A	69.87332928	0.001198686	0.572227918
PPP2R5C	26.07827627	0.001175195	0.602577021
PPP4C	112.6078801	0.002681727	1.580848008
PPP4R3B	65.80666782	1.25E-06	0.469267153
PPP4R3C	0.022164248	0.002634685	1.734336036
PPP6C	74.06733922	4.72E-07	0.455076973
PPT1	267.9482233	0.000142404	0.557959756
PPT2-EGFL	0.991588495	0.004786712	1.52966262
PQBP1	107.1959229	0.004853911	1.53358438
PQLC2	22.51194941	0.002868581	1.570495462
PQLC2L	2.321552063	3.23E-05	1.921171893
PQLC3	68.35254594	0.000540755	0.580883933
PRAC1	0.050543373	0.0007961	2.03326902
PRAC2	0.433501504	0.001440811	1.77165189
PRAG1	17.37196343	0.000186145	0.549419123
PRAL	0.329347829	2.68E-05	1.878935315
PRAM1	5.388961377	0.002337741	1.585471127
PRAME	16.57494582	1.27E-05	1.934756138
PRAMEF17	0.035403396	0.000860456	1.707142543
PRAMEF30P	0.02572401	0.004266198	1.63162024
PRC1	10.7501092	0.000236796	1.743338667
PRCP	58.17058587	0.000170196	0.553657468
PRDM12	0.093957636	0.000182754	1.766350844
PRDM16	1.481189802	0.003219132	0.553613915
PRDM2	13.64207697	0.003432753	0.626798106
PRDM6	0.811858385	0.001060119	1.646568984
PRDM8	2.610056381	0.001436992	1.639875557
PRDX3	210.8157652	0.00037373	0.575762283
PRELID1P4	1.629276128	0.000121716	1.782068707
PRELID2	7.282349559	0.001491988	0.605871779
PRELID2P1	0.474563042	0.000659754	1.674609257
PRELID3A	3.14072499	1.80E-08	2.300114508
PRELID3BP	0.005559045	0.001033313	3.958655738
PRELID3BP	0.059323191	0.003392082	1.786093705
PREPL	47.36204188	0.000255412	0.561265378
PREX1	59.98619318	0.000198897	0.550550737
PRG2	0.273352057	9.01E-05	1.805977201
PRG4	1.612510744	0.002575433	2.259107793
PRH2	0.42063996	1.90E-05	1.902439275
PRICKLE2	9.322186852	0.004893426	0.629123926
PRICKLE2-A	0	0.004606615	1.557019068
PRICKLE3	10.55531478	0.004073806	1.544095243
PRICKLE4	0.880850868	0.001425865	1.617838166
PRKAA1	62.1781754	1.92E-05	0.512396117
PRKAA2	30.38533763	1.11E-07	0.412977848
PRKAG2-AS	13.22888204	5.13E-05	1.837643571
PRKAR1A	193.8061441	0.000106736	0.543252846
PRKAR2A	30.296128	0.001631378	0.605789867
PRKCE	9.162619474	1.45E-06	0.441283257
PRKCG	0.161968155	6.06E-08	2.325692035
PRKCZ	8.492103156	2.91E-07	0.429919793
PRKD1	18.03168061	1.13E-06	0.448981481
PRKG2	2.48204723	0.004364255	0.595552008
PRKN	16.42190476	0.000453829	0.542431763
PRKRIP1	35.02106771	0.000344192	1.711893431
PRKX	24.74954524	0.000781281	0.562814436
PRLH	0.030918563	0.004879126	1.928218017
PRMT5	54.40805809	6.10E-05	0.533204527
PRMT5-AS1	0.391832594	0.000178977	1.751569633
PRMT9	11.88906806	1.04E-06	0.443531295
PROCA1	2.329512051	0.000945012	1.647177506
PROP1	0.1010919	0.001737728	1.65808442
PRORSD1P	4.292915008	0.000479017	1.690322962
PRORY	0.033089808	0.002612104	1.785244583
PROSER3	6.885537446	0.001365326	1.619146842
PROX1-AS1	0.049502995	1.99E-09	2.768214102
PRPF18	19.70144006	0.000126269	0.55542588
PRPF38A	33.88456362	0.003892324	0.638019716
PRPF4	40.56024853	0.000111281	0.545425396
PRPF6	186.5045893	0.00010873	0.548627329
PRPF8	116.4686519	0.000257373	0.561794495
PRPSAP2	23.90444846	0.00299977	0.630497155
PRR11	6.715029756	7.63E-06	1.960965945
PRR14L	15.12307071	0.000437315	0.569782622
PRR15L	20.68636631	0.000704347	0.431548657
PRR19	0.842282488	0.000771513	1.677316051
PRR20A	0	0.004606615	1.557019068
PRR20B	0	0.004606615	1.557019068
PRR20C	0	0.004606615	1.557019068
PRR20D	0	0.004606615	1.557019068
PRR23B	0.013627999	0.000139093	2.153431166
PRR26	5.221492732	0.001968586	0.601301528
PRR4	1.614371147	0.001587269	1.610000274
PRR7-AS1	1.455094644	0.000103051	1.794637974
PRRC1	41.18798549	0.001296438	0.604527974
PRRC2B	54.59758524	6.95E-06	0.48164566
PRRG1	13.51308291	2.97E-05	0.484230997
PRRG4	38.60281038	0.000893888	0.586761842
PRRT2	5.079917343	0.001764011	1.601140986
PRSS23	94.13886424	0.001312194	0.601051289

PRSS27	1.140088183	0.004582521	1.538631966
PRSS36	2.345767963	0.000366368	1.711207911
PRSS3P4	0.055452399	0.000766832	2.117568325
PRSS41	0.088946325	0.000207997	1.872423689
PRSS46P	0.316562473	0.002129284	1.605703854
PRSS50	0.874483955	1.13E-05	1.953967822
PRSS53	2.792226423	1.31E-06	2.044419074
PRSS54	0.042411091	0.001851746	1.63894047
PRUNE2	76.17528766	3.50E-07	0.422316133
PRX	7.488189307	1.59E-05	0.491614239
PRXL2A	87.7165147	7.37E-05	0.531487544
PRXL2B	52.27219642	0.000762639	0.589567903
PRXL2C	32.32473755	0.001235311	0.599467752
PRYP1	0	0.004606615	1.557019068
PRYP3	0	0.004606615	1.557019068
PRYP4	0	0.004606615	1.557019068
PSAT1	33.88478467	0.001074859	1.685049851
PSAT1P1	0.052631344	0.000688974	1.701636566
PSEN1	33.7084929	1.57E-07	0.421476908
PSENEN	78.09490083	7.77E-06	1.956570924
PSG1	0.044321902	0.000217269	1.848079635
PSG10P	0.033531687	0.000502973	1.785630896
PSG2	0.031810599	1.31E-05	1.990832325
PSG6	0.021558614	0.001701298	1.702732686
PSG7	0.009562614	0.004800556	1.592435597
PSG8-AS1	0.067333107	3.96E-06	2.119645876
PSIP1	46.76696308	0.000176002	0.554675799
PSKH1	40.93669167	0.000423514	0.563017088
PSMA1	73.5902086	0.000203714	1.759648682
PSMA4	52.26149912	0.000812047	1.663262654
PSMA5	44.08167932	0.000664425	1.673372228
PSMB3	270.0209652	1.53E-05	1.914652627
PSMB4	350.0234072	0.00070973	1.674752458
PSMB7	277.6316461	0.002167815	0.627700891
PSMC1P4	0.05541369	0.004269563	1.56561676
PSMC5	103.474315	0.000709362	1.675839579
PSMD10P2	0.135729525	0.000268066	1.763222098
PSMD13	85.4562856	2.78E-08	2.336225684
PSMD14	25.62960235	2.58E-05	1.882500129
PSMD4	148.9210244	0.000981441	1.656630986
PSMD4P1	0	0.004606615	1.557019068
PSMD5	32.57840231	0.004038205	0.641260295
PSMD6-AS1	0	0.004606615	1.557019068
PSME2	121.4867571	3.01E-05	1.875812731
PSME2P2	4.309969387	7.16E-06	1.960510945
PSMG3	45.82690052	0.000634599	1.676519769
PSORS1C2	1.699835408	3.96E-05	1.856881792
PSPC1-AS2	3.424152594	0.000274989	1.725875139
PSRC1	5.675366551	2.72E-07	2.145021417
PTAR1	25.63792322	1.25E-05	0.487454292
PTBP1P	0.143229246	0.001335334	1.637793568
PTBP3	55.40243361	5.86E-05	0.532702263
PTCD1	4.16290112	8.13E-06	1.961311467
PTCHD3P1	0	0.004606615	1.557019068
PTCSC2	0.127701252	0.000870738	1.781986194
PTDSS2	24.34027135	0.003603084	1.554931146
PTEN	44.89751566	0.000414674	0.569976032
PTENP1	1.333129415	0.000801566	0.587009864
PTGDR2	0.741125526	0.001815465	0.585732392
PTGER1	1.024451446	0.002618674	1.670231485
PTGER2	9.996658588	0.000680959	1.705180282
PTGES	19.51320384	0.000472005	1.760841855
PTGES3L	0.396931224	0.000492396	1.69013909
PTGR1	89.19688367	0.002734182	0.61252806
PTGR2	9.694736451	0.003075715	0.622889017
PTH	0.086736242	2.59E-05	1.99029423
PTH1R	34.85813419	0.003647069	0.602544252
PTHLH	61.94214653	0.004754449	1.595345465
PTK2	41.71749088	0.000347984	0.571947207
PTK6	3.808199071	3.25E-07	2.239108411
PTMAP15	0.138125216	0.000959531	1.71118967
PTOV1	67.42295186	0.002954615	1.569999299
PTOV1-AS1	9.535715311	0.000269172	1.731742343
PTOV1-AS2	12.02219717	1.08E-05	1.924695354
PTP4A2P1	1.620478221	0.002994097	1.565269502
PTP4A2P2	9.414966307	0.00157697	0.598260704
PTPN11	78.27129569	0.003146011	0.628039045
PTPN13	20.55491796	0.000208289	0.543324827
PTPN2	15.97675163	0.000530182	1.686561499
PTPN3	24.12518734	3.03E-05	0.48527902
PTPN4	7.739770322	0.000376701	0.561783597
PTPN9	30.16857139	5.92E-05	0.529962212
PTPRA	89.54184376	0.000562647	0.58074559
PTPRB	47.78845348	4.84E-07	0.433399754
PTPRCAP	0	0.004606615	1.557019068
PTPRG	37.66813238	8.48E-08	0.393512454
PTPRH	3.282146814	2.83E-10	2.715843754
PTPRJ-AS1	0.774173147	0.004834592	1.709106714
PTPRK	53.86449882	1.57E-05	0.495089071
PTPRM	88.47811546	3.41E-05	0.505091673
PTPRN	4.999934137	0.000680707	1.87029976
PTPRN2	14.51553767	0.001055932	0.573243241
PTPRVP	0.271973731	0.000307069	1.724382913
PTRH2	13.45708407	0.001709646	1.608160856
PTRHD1	26.51782812	0.000389058	1.708860586
PTTG1	15.56976517	1.45E-10	2.567667385
PTTG4P	0.475945529	0.003984969	1.551912447
PTX3	5.05580594	7.24E-07	2.25495548
PUM1	65.25780019	8.87E-06	0.492661446
PUM2	80.35811413	0.000472939	0.580724379
PURA	25.97560858	2.48E-07	0.412073772
PURB	41.17898504	0.001729025	0.602205528
PUS1	11.85642789	2.13E-11	2.716685855
PUSL1	9.646654038	1.32E-09	2.489749059
PVRIG2P	0	0.004606615	1.557019068
PVT1	13.02691844	1.42E-05	1.910604476
PWAR5	2.614987148	5.64E-05	0.467051346
PXMP2	45.24243965	0.000787562	0.586552638
PXN-AS1	4.521003374	0.001206253	1.628133316
PXT1	0.226067401	0.001840735	1.603379771
PYCARD	41.52927778	3.19E-05	1.869024844
PYCR1	14.01878337	4.45E-08	2.272179305
PYCR2	59.3903334	0.000699856	1.676386562
PYCR3	17.38405795	0.003268859	1.562406125
PYGB	120.4141579	4.17E-05	1.873504206
PYGO2	47.84972299	0.003721459	1.558809383
PYHIN5P	0.008826188	0.001333152	2.141256428
PYROXD1	14.16261409	0.002577567	0.625502296
PYY2	0.982157898	2.53E-05	1.878876952
QPCTL	21.41516748	0.00012998	1.776010667
QRFPR	29.18318675	1.20E-06	0.448359472
QSOX1	96.41552419	0.000108452	1.801929269
QTRT1	52.34243069	0.000335358	1.714458068
R3HCC1	57.22066603	0.000242863	0.567577959
R3HDM4	68.81070642	3.32E-08	2.313863064
R3HDML	0.069825723	0.000473872	1.865570891
RAB11A	112.3632425	6.30E-05	0.527894666
RAB11FIP1	15.00573982	0.002768993	0.596066448
RAB11FIP2	22.54867228	5.01E-05	0.517834135
RAB11FIP5	80.07778779	0.000107799	0.539682704
RAB14	119.7798448	2.82E-06	0.479413407
RAB17	50.53402398	0.000210458	0.549106102
RAB18	59.63115618	0.002629991	0.627670943
RAB1B	344.6291912	0.001170723	0.606811365
RAB1C	0.493932109	0.000513661	0.562721699
RAB21	33.21903456	1.36E-05	0.49988961
RAB26	1.628463828	0.004072443	1.572121153
RAB27B	2.852044866	0.003088483	1.611514821
RAB33B	14.87810366	0.000106635	0.541741597
RAB3D	27.53328809	0.002474337	0.623716671
RAB3GAP1	51.15685587	9.98E-06	0.501316353
RAB3GAP2	22.35316936	0.000217358	0.556595354
RAB3IP	24.43600278	8.66E-08	0.410344592
RAB40A	1.220550897	0.003755479	1.567092319
RAB40AL	0.169949524	9.18E-05	1.802322428
RAB4A	45.42408889	4.89E-05	0.536037827
RAB4B	12.31852318	3.13E-06	2.006828353
RAB5B	120.117921	0.00028821	0.575315603
RAB5C	144.5511811	0.002586776	0.628057681
RAB5IF	38.8227059	0.00120225	1.635116259
RAB6A	138.5056774	0.000279195	0.570706821
RAB6B	19.14615062	0.004814839	1.539570393
RAB8B	44.58368046	0.001242133	0.60189113
RAB9A	60.01550127	0.002115136	0.615763035
RAB9AP1	0	0.004606615	1.557019068
RAB9AP2	0	0.004606615	1.557019068
RAB9AP3	0	0.004606615	1.557019068
RABEP2	21.07001313	9.94E-05	1.795066273
RABGAP1	29.10009243	4.53E-06	0.476826839
RABGAP1L	10.45532262	0.002451776	0.606539798

RABGAP1L	0.096226399	4.95E-05	1.856272454
RABL2A	6.273000006	0.001496645	1.611141756
RABL3	26.07688057	3.00E-05	0.521127906
RAC3	7.576709919	4.02E-05	1.865433717
RAD17	32.97067612	8.30E-06	0.490455075
RAD1P2	0.321198073	0.004775715	1.551696484
RAD21	97.04350992	0.001871226	0.61716949
RAD21L1	0.048516764	0.001397655	1.633386625
RAD23B	126.0352692	1.53E-05	0.511290623
RAD23BP1	0.082554046	0.003477295	0.622965577
RAD50	38.05920567	8.38E-07	0.442652775
RAD51	3.488809997	0.000696216	1.67389464
RAD51AP1	6.454320384	0.001234244	1.633418022
RAD51AP1I	0.120684689	0.000363719	1.814358794
RAD51AP2	0.023961352	0.001891654	1.772411879
RAD52	10.23769407	0.002429019	1.577412668
RAD54B	2.135045989	0.000168677	1.769245219
RAD54L	4.09146755	0.000111403	1.810549193
RAD9A	14.73092255	2.09E-05	1.890073054
RAET1K	0.39450506	7.32E-05	1.816965204
RAG1	2.440832692	1.42E-06	0.440895878
RAI2	43.99437542	7.13E-06	0.490323843
RALB	179.6829971	1.77E-06	0.476207476
RALBP1	61.53729564	0.000663146	0.590528425
RALGAPA1	6.436436385	5.75E-06	0.472838362
RALGAPA1	1.092803444	0.000196073	0.548720657
RAMP2	181.100699	0.001584258	0.602541958
RAMP3	221.6871869	5.47E-05	0.527808627
RAN	152.6207181	0.002120085	1.593822414
RANBP1	33.773179	0.000979456	1.647391649
RANBP2	34.96895772	0.001192097	0.594732744
RANBP6	25.86134006	1.27E-05	0.490557447
RANGAP1	43.37603286	0.000250926	1.737486763
RANGRF	19.16827512	0.002071415	1.595332438
RANP5	0.066868651	8.01E-05	1.852047447
RAP1A	66.57017141	0.000250261	0.568826606
RAP1GDS1	27.38134095	0.002741162	0.619434664
RAP2A	53.42613387	1.48E-05	0.499364235
RAP2C-AS1	2.354738608	0.000637163	0.577410247
RAPGEF1	89.91336448	0.000648748	0.577950603
RAPGEF2	30.91068931	1.22E-06	0.438251193
RAPGEF4	15.63851809	4.82E-06	0.465751849
RAPGEF5	34.15594084	0.001222516	0.596812249
RAPGEF6	5.495799339	0.003230817	0.630963514
RAPGEFL1	4.726569031	2.60E-07	2.135654879
RAPH1	2.072753986	5.08E-06	0.456873401
RAPSN	0.580362841	0.000112201	1.80627901
RARB	21.55742473	0.000304176	0.548236971
RARRES2	538.1734722	7.81E-05	1.813799451
RARRES2P3	0.131843419	0.000860089	1.764019641
RASAL2	13.88715549	0.001835675	0.601031685
RASAL2-AS	2.140459804	0.000782299	0.582599755
RASEF	15.06221432	2.68E-06	0.465661318
RASL10B	4.348020551	0.002542249	1.594282953
RAVER2	19.94372017	2.75E-06	0.474334899
RB1	72.64360372	0.000299368	0.573675937
RBAKDN	0.206585239	0.000345262	1.738031063
RBBP5	27.11577431	0.003405625	0.634280372
RBBP9	51.38562778	0.000176757	0.554933686
RBCK1	136.7445255	4.13E-08	2.260516251
RBFOX3	0.090118489	1.11E-07	2.195264773
RBL2	54.10220033	0.00391758	0.638187426
RBM14-RBI	1.61267876	0.00051662	1.684997637
RBM18	27.88548635	4.36E-08	0.414652459
RBM22	70.23417899	0.000128267	0.55018952
RBM22P2	0.813188723	0.000571976	1.683052949
RBM22P4	0.058521306	0.004658352	1.618498686
RBM23	19.66954616	0.000162646	0.546114856
RBM27	17.10047301	0.004827533	0.645183653
RBM3	271.7705051	0.000991084	0.589988864
RBM38	29.13051412	0.003510354	1.556948772
RBM43	25.73092479	0.000215215	0.544101808
RBM47	65.45170211	1.95E-06	0.45839775
RBM6	34.53562714	0.000455753	1.690890439
RBM7	14.95838867	0.000184384	0.553278508
RBMS2	46.26690777	0.000192548	0.555613783
RBMS2P1	2.490653012	0.001916925	0.606556782
RBMXL1	15.23975573	0.001281456	0.60375837
RBMXP2	0.722175018	0.000311765	0.546723636
RBMY1A1	0.001318266	7.26E-05	5.845461273
RBMY1B	0	0.004606615	1.557019068
RBMY1D	0	0.004606615	1.557019068
RBMY1HP	0	0.004606615	1.557019068
RBMY2BP	0	0.004606615	1.557019068
RBMY2CP	0	0.004606615	1.557019068
RBMY2UP	0	0.004606615	1.557019068
RBMY2YP	0	0.004606615	1.557019068
RBP1	12.47382529	0.004107634	1.558902147
RBPJL	0.132277045	1.51E-05	1.999427034
RBSN	13.52796335	4.29E-05	0.514992386
RC3H2	21.53806414	6.36E-06	0.478533474
RCAN1	89.91586042	0.001861678	0.54091825
RCAN2	119.8525973	0.000157123	0.514742565
RCBTB1	31.9467952	0.00231426	0.610596377
RCBTB2	73.23860534	0.00037659	0.571474867
RCC1	27.43844465	0.004822431	1.534894269
RCC2	97.00140367	0.003376903	1.571693928
RCC2-AS1	0	0.004606615	1.557019068
RCCD1	10.59617855	0.000348944	1.710661044
RCHY1	18.0434733	4.94E-06	0.483276451
RCL1	10.87067674	0.001643789	0.589142843
RCN3	52.41100464	7.75E-06	1.953846392
RCOR1	23.93250498	0.001333648	0.602652413
RCOR2	1.222509077	0.000100467	1.934899257
RDH10-AS1	0.487706385	0.002598566	1.607091386
RDH11	71.77162325	0.003990436	0.605619304
RDH14	48.83161416	0.00156979	0.607161709
RDH16	0.925518839	7.50E-09	2.683167352
RDM1	0.78201984	9.29E-05	1.803957411
RDX	87.34279081	5.58E-06	0.481776502
REC114	0.192809027	2.46E-07	2.151340445
REC8	8.735692465	0.000155019	1.763183215
RECK	13.53213087	0.000674885	0.575796322
RECQL4	5.925917378	6.74E-09	2.359205983
REEP4	28.47874847	3.93E-08	2.277512015
REEP5	254.7109701	0.002360447	0.616758315
REG3A	0.21910669	5.07E-05	2.186575651
RELL1	20.12650313	0.00107599	0.592215491
RELL2	2.024498666	0.000615789	1.67656208
RELT	5.964678525	0.001105767	1.637371204
REM2	0.648559682	3.94E-08	2.251757093
REPS2	20.47364301	0.000536361	0.56222686
RERE	65.72093784	0.001457174	0.607051894
RERGL	8.585159851	0.000507242	0.536650729
REST	23.88512914	2.55E-05	0.50176136
RET	1.017377401	0.000474704	1.744216807
RETNLB	0.055508709	0.003055135	1.660152441
RETREG2	139.4711114	6.99E-05	0.542453739
RETSAT	101.98211	3.41E-09	0.366139197
REX1BD	23.65744512	7.34E-06	1.955909752
REXO1L10I	0	0.004606615	1.557019068
REXO1L11I	0	0.004606615	1.557019068
REXO1L12I	0	0.004606615	1.557019068
REXO1L2P	0	0.004606615	1.557019068
REXO1L4P	0	0.004606615	1.557019068
REXO1L5P	0	0.004606615	1.557019068
REXO1L6P	0.00026397	0	261.7554331
RF00003	0.635762009	3.28E-05	1.891751049
RF00017	0.263261231	0.000165528	1.755909254
RF00019	0.686596521	0.00018175	1.751259416
RF00024	0	0.004606615	1.557019068
RF00099	0	0.004606615	1.557019068
RF00108	0.175455272	0.002921592	1.620994634
RF00134	2.697403022	0.000322878	1.716920046
RF00139	0.28594494	0.001126974	1.643914881
RF00156	0.164929417	0.000442659	1.708379563
RF00181	0.049263225	0.002842152	1.725537832
RF00201	0	0.004606615	1.557019068
RF00263	0.126917731	0.002798983	1.613914175
RF00275	1.425217373	9.90E-05	1.788059353
RF00279	0.219729259	5.11E-05	1.895708716
RF00345	0	0.004606615	1.557019068
RF00393	0.082382763	0.002954739	1.57869328
RF00394	0.301709172	0.004473643	1.538946626
RF00397	0.707603728	0.000380643	1.751019336
RF00406	0.097742056	0.003686254	1.658550906
RF00409	1.207770511	8.75E-05	1.796616513
RF00416	0.103652136	0.002964616	1.586152341
RF00421	0.092234743	0.000691589	1.731929807
RF00422	1.073950981	0.000343651	1.716488276

RF00425	0.086915096	0.000298325	1.784083922
RF00426	0.176386268	0.001561449	1.618418251
RF00432	0.676136278	8.18E-06	1.955570854
RF00478	0.085783423	3.00E-06	2.196544317
RF00561	0.48358597	0.003177917	1.582143638
RF00569	0.007584071	0.004148371	4.558639834
RF00572	0.353614227	0.003340231	1.64719401
RF00573	0.389855057	0.004473947	1.54501571
RF00599	0.811213534	8.60E-09	2.36049397
RF00603	0.027904128	0.001874629	2.542987463
RF00604	1.190078148	0.000174288	1.776907838
RF00635	0	0.004606615	1.557019068
RF01159	0	0.004606615	1.557019068
RF01293	1.26949605	0.002851406	1.569479772
RF01518	0.147662275	0.000116646	1.848172951
RF01871	0	0.004606615	1.557019068
RF01872	0	0.004606615	1.557019068
RF01873	0	0.004606615	1.557019068
RF01875	0	0.004606615	1.557019068
RF01876	0	0.004606615	1.557019068
RF01877	0	0.004606615	1.557019068
RF01879	0	0.004606615	1.557019068
RF01880	0	0.004606615	1.557019068
RF01881	0	0.004606615	1.557019068
RF01882	0	0.004606615	1.557019068
RF01883	0	0.004606615	1.557019068
RF01887	0	0.004606615	1.557019068
RF01888	0	0.004606615	1.557019068
RF01891	0	0.004606615	1.557019068
RF01892	0	0.004606615	1.557019068
RF01894	0	0.004606615	1.557019068
RF01905	0	0.004606615	1.557019068
RF01906	0	0.004606615	1.557019068
RF01909	0.753949347	0.000253016	1.742430833
RF01946	0	0.004606615	1.557019068
RF01947	0	0.004606615	1.557019068
RF01948	0	0.004606615	1.557019068
RF01950	0	0.004606615	1.557019068
RF01951	0	0.004606615	1.557019068
RF01953	0	0.004606615	1.557019068
RF01954	0.078249268	0.00089898	2.096670173
RF01955	0	0.004606615	1.557019068
RF01956	0	0.004606615	1.557019068
RF01957	0	0.004606615	1.557019068
RF01963	0	0.004606615	1.557019068
RF01967	0	0.004606615	1.557019068
RF01968	0	0.004606615	1.557019068
RF01971	0	0.004606615	1.557019068
RF01972	0	0.004606615	1.557019068
RF01973	0	0.004606615	1.557019068
RF01975	0	0.004606615	1.557019068
RF01976	0	0.004606615	1.557019068
RF01977	0	0.004606615	1.557019068
RF01978	0	0.004606615	1.557019068
RF01979	0	0.004606615	1.557019068
RF01981	0	0.004606615	1.557019068
RF01984	0	0.004606615	1.557019068
RF01986	0	0.004606615	1.557019068
RF01987	0	0.004606615	1.557019068
RF02038	0	0.004606615	1.557019068
RF02039	0	0.004606615	1.557019068
RF02040	0	0.004606615	1.557019068
RF02041	0	0.004606615	1.557019068
RF02042	0	0.004606615	1.557019068
RF02043	0	0.004606615	1.557019068
RF02045	0.019600637	0.001373757	2.713494235
RF02089	0	0.004606615	1.557019068
RF02090	0	0.004606615	1.557019068
RF02101	0	0.004606615	1.557019068
RF02103	0	0.004606615	1.557019068
RF02104	0	0.004606615	1.557019068
RF02105	0	0.004606615	1.557019068
RF02106	0	0.004606615	1.557019068
RF02107	0	0.004606615	1.557019068
RF02109	0	0.004606615	1.557019068
RF02114	0	0.004606615	1.557019068
RF02121	0	0.004606615	1.557019068
RF02122	0	0.004606615	1.557019068
RF02124	0	0.004606615	1.557019068
RF02125	0	0.004606615	1.557019068
RF02126	1.189410062	0.003714342	1.554837972
RF02127	0	0.004606615	1.557019068
RF02130	0	0.004606615	1.557019068
RF02131	0	0.004606615	1.557019068
RF02137	0	0.004606615	1.557019068
RF02138	0	0.004606615	1.557019068
RF02139	0	0.004606615	1.557019068
RF02141	0.56093402	0.000593259	1.701033563
RF02142	0	0.004606615	1.557019068
RF02143	0	0.004606615	1.557019068
RF02145	0	0.004606615	1.557019068
RF02146	0	0.004606615	1.557019068
RF02148	0	0.004606615	1.557019068
RF02149	0	0.004606615	1.557019068
RF02150	0	0.004606615	1.557019068
RF02156	0	0.004606615	1.557019068
RF02157	0	0.004606615	1.557019068
RF02158	0	0.004606615	1.557019068
RF02159	0	0.004606615	1.557019068
RF02160	0	0.004606615	1.557019068
RF02161	0	0.004606615	1.557019068
RF02166	0	0.004606615	1.557019068
RF02172	0	0.004606615	1.557019068
RF02173	0	0.004606615	1.557019068
RF02174	0	0.004606615	1.557019068
RF02175	0	0.004606615	1.557019068
RF02176	0	0.004606615	1.557019068
RF02177	0	0.004606615	1.557019068
RF02178	0	0.004606615	1.557019068
RF02179	0	0.004606615	1.557019068
RF02180	0	0.004606615	1.557019068
RF02181	0	0.004606615	1.557019068
RF02183	0	0.004606615	1.557019068
RF02185	0	0.004606615	1.557019068
RF02187	0	0.004606615	1.557019068
RF02189	0	0.004606615	1.557019068
RF02190	0	0.004606615	1.557019068
RF02192	0	0.004606615	1.557019068
RF02193	0	0.004606615	1.557019068
RF02195	0	0.004606615	1.557019068
RF02196	0	0.004606615	1.557019068
RF02197	0	0.004606615	1.557019068
RF02198	0	0.004606615	1.557019068
RF02199	0	0.004606615	1.557019068
RF02201	0	0.004606615	1.557019068
RF02202	0	0.004606615	1.557019068
RF02203	0	0.004606615	1.557019068
RF02204	0.273636665	0.000720647	1.775978519
RF02205	0	0.004606615	1.557019068
RF02208	0	0.004606615	1.557019068
RF02209	0	0.004606615	1.557019068
RF02210	0	0.004606615	1.557019068
RF02211	0	0.004606615	1.557019068
RF02212	0	0.004606615	1.557019068
RF02213	0	0.004606615	1.557019068
RF02215	0	0.004606615	1.557019068
RF02216	0	0.004606615	1.557019068
RF02217	0	0.004606615	1.557019068
RF02218	0	0.004606615	1.557019068
RF02220	0	0.004606615	1.557019068
RF02248	0	0.004606615	1.557019068
RF02251	0	0.004606615	1.557019068
RF02252	0.045496514	7.86E-07	2.729659817
RF02266	0	0.004606615	1.557019068
RF02271	0.600690342	0.001233258	1.624275718
RFC1	39.27615749	7.08E-05	0.530772132
RFC4	15.25520953	1.14E-05	1.929664576
RFLNA	6.211422723	1.47E-05	2.075374622
RFPL3S	0.667787405	7.67E-07	2.079464209
RFTN2	5.418766043	0.004408023	0.622547549
RGCC	374.2095076	0.000579738	0.575261643
RGL1	79.9964192	2.74E-07	0.443186288
RGMB-AS1	0.728784025	0.000205447	1.764438592
RGP1	30.3608496	6.20E-08	0.400954737
RGPD1	0.038200005	0.000789751	1.688256556
RGR	0.019589966	0.003235262	1.603542979
RGS10	77.85764774	0.000260438	1.739615034
RGS11	1.243518102	0.002713996	1.62803403
RGS17	1.000011419	1.53E-10	2.583014072
RGS19	33.86833154	0.001725411	1.613063487

RGS2	70.00519322	0.000528658	1.701892778
RGS20	1.056153087	1.35E-06	2.142710876
RHBDD1	22.68506313	0.001420099	0.613141531
RHBDD3	18.41422062	1.01E-09	2.508748047
RHBDF1	21.68045736	2.23E-05	1.887611052
RHBDF2	34.54124838	8.23E-05	1.815794737
RHBDL2	3.27215647	2.21E-07	2.160140737
RHEBL1	3.447768764	0.000600346	1.680177858
RHNO1	37.21952272	4.37E-06	2.002762593
RHO	0.09544313	0.000286733	1.743129293
RHOA	690.8486486	0.000794013	0.590359613
RHOB	1527.206177	4.47E-05	0.502029633
RHOBTB1	73.95709651	0.001620205	0.606844409
RHOG	130.9847813	8.40E-05	1.820691472
RHOQ	60.6032743	0.000111703	1.790489878
RHOT2	52.876096	0.003834353	1.548411272
RHOU	38.23170034	0.000911751	0.578583336
RHOXF1	1.851973036	0.002949636	1.568443176
RHPN1	17.76177544	3.45E-06	1.999353097
RHPN2	42.57811003	0.000100126	0.543760021
RIF1	16.01275738	0.000779626	0.579660051
RILPL1	10.29289189	3.33E-06	2.000483443
RILPL2	26.81403728	0.000952372	0.600191042
RIMKLA	16.11802231	0.000146364	0.53758315
RIN1	7.959992683	3.34E-07	2.139170966
RIN2	76.07623423	9.29E-05	0.538902139
RIOK2	18.45285487	0.000612813	0.5875388
RIOK3	76.08163237	8.31E-05	0.537953358
RIPK2	28.12284849	0.000832588	1.661709088
RIPOR1	73.81639492	0.001207865	0.609130142
RIPOR2	10.5241557	0.001416006	0.60189378
RIT1	91.92761394	1.10E-05	0.492988197
RLBP1	0.093952028	0.000142379	2.050649057
RLIMP2	0	0.004606615	1.557019068
RLN2	1.384475343	0.003985408	0.609788602
RMDN1	33.18564975	2.64E-05	0.510850504
RMDN2	4.383837793	0.002118341	0.598163427
RMDN2-AS	0.09847581	0.000737269	1.670531521
RMDN3	39.23550122	2.22E-05	0.51105237
RMND1	56.95390518	0.00133151	0.59538039
RMND5A	44.3601044	1.49E-07	0.420720264
RMND5B	23.02547757	0.001855552	0.615825266
RN7SKP106	0.121478736	0.000556217	1.714769272
RN7SKP109	0.21165349	1.50E-05	2.000713479
RN7SKP11	0.084307457	0.000103936	1.942717361
RN7SKP131	0	0.004606615	1.557019068
RN7SKP132	0.013343505	0.004385471	2.685780196
RN7SKP147	0.006076415	0.004649019	3.054056453
RN7SKP17	0.084413133	0.000182099	1.907047658
RN7SKP172	0.034715819	0.002254664	2.066501891
RN7SKP175	0	0.004606615	1.557019068
RN7SKP176	0	0.004606615	1.557019068
RN7SKP177	0.056183375	0.001595746	1.863837561
RN7SKP18	0.028321387	1.67E-05	2.621934728
RN7SKP181	0.058847531	0.000574764	1.94994719
RN7SKP185	0.340325704	0.00226404	1.701120227
RN7SKP187	0	0.004606615	1.557019068
RN7SKP198	0.418100776	0.000757001	1.665190617
RN7SKP202	0.107139044	0.002439969	1.653120402
RN7SKP212	0.006921902	0.000257282	3.704592444
RN7SKP241	0.125882343	0.000284138	1.751928904
RN7SKP247	0.135072666	0.000160223	1.876592247
RN7SKP287	0.847494683	6.23E-06	1.966273428
RN7SKP295	0.148436089	0.001329004	1.80644084
RN7SKP296	0.671929395	0.003290208	1.564190847
RN7SKP38	0.331248118	0.000310751	1.779550577
RN7SKP51	0.036420471	3.18E-05	2.766094755
RN7SKP54	0	0.004606615	1.557019068
RN7SKP63	0	0.004606615	1.557019068
RN7SKP7	0	0.004606615	1.557019068
RN7SKP80	2.881660649	0.003222816	1.573383235
RN7SKP88	0	0.004606615	1.557019068
RN7SKP90	0.30850008	0.001779642	1.677235866
RN7SKP97	0.739420913	0.000244838	1.736457782
RN7SKP98	0.056981154	0.002248277	1.891049409
RN7SL1	0	0.004606615	1.557019068
RN7SL107P	0.079043972	0.000483655	1.938288817
RN7SL125P	0.385512797	0.001322315	1.667524842
RN7SL144P	0.275078725	0.002179807	1.662336563
RN7SL154P	0.192650805	0.000197243	1.749862534
RN7SL170P	0.191182967	0.000346139	1.769200564
RN7SL174P	0.270163522	0.00199407	1.631076151
RN7SL176P	0	0.004606615	1.557019068
RN7SL181P	1.992504577	0.001354157	0.581763349
RN7SL200P	0.392827741	4.42E-06	2.053936809
RN7SL203P	0	0.004606615	1.557019068
RN7SL233P	0.82263492	0.002257952	1.60386656
RN7SL236P	0.507031741	1.43E-11	2.688749645
RN7SL239P	0.641725024	2.08E-05	1.897208236
RN7SL244P	0	0.004606615	1.557019068
RN7SL259P	0.113134694	6.28E-06	2.08189535
RN7SL277P	0.476360992	0.003328298	1.667135108
RN7SL278P	0.151807086	0.001048392	1.707424135
RN7SL309P	0.047744546	0.001541568	1.935850715
RN7SL316P	0.20424505	0.000214431	1.744552558
RN7SL327P	0	0.004606615	1.557019068
RN7SL33P	0.083881254	0.001110084	1.784986106
RN7SL344P	0.199723305	0.000313266	1.733802868
RN7SL364P	1.592998787	0.001047571	1.644149245
RN7SL368P	1.512917491	3.82E-06	2.001415599
RN7SL376P	0.109542742	2.53E-05	1.934804774
RN7SL37P	0.173576911	0.001974432	1.610860925
RN7SL380P	0.141625692	0.000194684	1.809903135
RN7SL390P	0.250530355	0.001080823	1.718104399
RN7SL39P	0.042561808	0.002072769	2.151615718
RN7SL404P	0.262863689	0.00384811	1.563182121
RN7SL415P	0.051047544	0.000364557	2.142061532
RN7SL416P	0.099309846	0.001718535	1.7638131
RN7SL417P	1.548138073	3.90E-05	1.886127886
RN7SL428P	0	0.004606615	1.557019068
RN7SL448P	0.241823962	0.002724725	1.628568177
RN7SL456P	0.105249396	0.002796663	1.672996807
RN7SL477P	0.682484152	0.004138097	1.554918943
RN7SL484P	0	0.004606615	1.557019068
RN7SL491P	0.135274368	1.95E-05	1.921152797
RN7SL492P	0.066034253	0.003365485	1.784950372
RN7SL513P	0.302563211	0.000224556	1.762554714
RN7SL521P	1.27788086	8.87E-05	1.815988954
RN7SL539P	0	0.004606615	1.557019068
RN7SL547P	0.23399117	0.002854368	1.622167364
RN7SL549P	0.101487779	7.01E-06	2.065545149
RN7SL558P	1.231324971	1.29E-05	1.927285192
RN7SL569P	0.144632198	0.002836137	1.596691656
RN7SL573P	0	0.004606615	1.557019068
RN7SL607P	0.172344706	0.001447452	1.674913819
RN7SL608P	5.250254987	6.78E-05	1.812799496
RN7SL610P	0.268092295	1.32E-05	1.930065718
RN7SL633P	0	0.004606615	1.557019068
RN7SL636P	0.25607304	0.003724628	1.627140654
RN7SL640P	0	0.004606615	1.557019068
RN7SL657P	0	0.004606615	1.557019068
RN7SL663P	1.365404057	3.92E-05	1.852686333
RN7SL672P	0.354367478	0.001390169	1.682538691
RN7SL67P	1.556940977	6.99E-06	2.015451125
RN7SL692P	0.070486568	0.002307355	1.787783002
RN7SL703P	0.034095907	0.00304616	1.965548239
RN7SL725P	0	0.004606615	1.557019068
RN7SL726P	0.821497584	7.26E-06	0.434258384
RN7SL731P	0	0.004606615	1.557019068
RN7SL737P	0	0.004606615	1.557019068
RN7SL738P	1.141226883	0.00030062	0.551655014
RN7SL748P	0.252085591	0.000674235	1.681454844
RN7SL771P	0.15589389	0.003361036	1.570892182
RN7SL77P	0	0.004606615	1.557019068
RN7SL785P	0.073644813	0.0032051	1.714643268
RN7SL793P	0	0.004606615	1.557019068
RN7SL794P	0.277002355	0.004027464	1.60308926
RN7SL799P	0.090793101	0.00178895	1.664641141
RN7SL800P	0	0.004606615	1.557019068
RN7SL818P	0	0.004606615	1.557019068
RN7SL825P	0.285802917	1.54E-05	1.936734977
RN7SL836P	0.215658086	0.002869347	1.693285348
RN7SL845P	0.055910842	0.000860606	1.924264379
RN7SL851P	0.244662635	1.48E-06	2.057087569
RN7SL860P	0	0.004606615	1.557019068
RN7SL88P	0.129518621	0.002013976	1.68243135
RNA5S1	0	0.004606615	1.557019068
RNA5S10	0	0.004606615	1.557019068
RNA5S11	0	0.004606615	1.557019068
RNA5S12	0	0.004606615	1.557019068

RNA5S13	0	0.004606615	1.557019068
RNA5S14	0	0.004606615	1.557019068
RNA5S15	0	0.004606615	1.557019068
RNA5S16	0	0.004606615	1.557019068
RNA5S17	0	0.004606615	1.557019068
RNA5S2	0	0.004606615	1.557019068
RNA5S3	0	0.004606615	1.557019068
RNA5S4	0	0.004606615	1.557019068
RNA5S5	0	0.004606615	1.557019068
RNA5S6	0	0.004606615	1.557019068
RNA5S7	0	0.004606615	1.557019068
RNA5S8	0	0.004606615	1.557019068
RNA5SP100	0.030577071	0.000617524	2.583762649
RNA5SP108	1.599411616	1.12E-08	2.32705858
RNA5SP122	2.984435372	0.000977343	1.654422624
RNA5SP130	0.080702237	0.00215398	2.012752777
RNA5SP151	1.143087552	0.001750161	1.609314215
RNA5SP153	0	0.004606615	1.557019068
RNA5SP18	7.55938714	3.32E-05	1.857449394
RNA5SP181	0.007477983	0.000801105	5.614012615
RNA5SP19	0.086722677	8.07E-05	2.283764537
RNA5SP196	0	0.004606615	1.557019068
RNA5SP204	0.118091852	0.001059095	1.893492745
RNA5SP206	0.21718595	3.95E-05	1.938775805
RNA5SP207	0.382103594	0.001694613	1.638515768
RNA5SP21	0.566460659	7.02E-07	0.436898424
RNA5SP216	0	0.004606615	1.557019068
RNA5SP226	0.17667759	0.004663135	1.66403491
RNA5SP227	0.198533059	0.000255718	1.899913513
RNA5SP236	0	0.004606615	1.557019068
RNA5SP244	0	0.004606615	1.557019068
RNA5SP253	0	0.004606615	1.557019068
RNA5SP254	0	0.004606615	1.557019068
RNA5SP257	0	0.004606615	1.557019068
RNA5SP265	0	0.004606615	1.557019068
RNA5SP274	0.05569157	0.000522785	2.2323002
RNA5SP276	0	0.004606615	1.557019068
RNA5SP319	0.111190071	0.002597041	1.814936266
RNA5SP327	0.201208859	0.00034957	1.954161051
RNA5SP329	0.163234183	0.004771572	1.748347579
RNA5SP334	0.180600575	5.92E-05	2.133586138
RNA5SP344	0.098547682	0.001916714	1.973406378
RNA5SP352	0	0.004606615	1.557019068
RNA5SP357	0.443988675	0.004998805	0.605764299
RNA5SP364	1.036795838	0.000728623	1.670817543
RNA5SP368	0	0.004606615	1.557019068
RNA5SP371	0.172378162	0.002000272	1.708452116
RNA5SP378	0.364536303	0.00105194	1.718673435
RNA5SP38	0.082859933	0.00234668	1.973585803
RNA5SP383	7.607408775	6.09E-05	1.819093003
RNA5SP384	0.11697477	0.000789628	1.970695479
RNA5SP392	0	0.004606615	1.557019068
RNA5SP410	0.018758632	3.36E-06	4.289158694
RNA5SP415	0.04059205	0.003692388	2.601125499
RNA5SP420	0.024872105	8.46E-05	4.039683882
RNA5SP422	0.034434408	9.69E-05	3.307737878
RNA5SP425	1.184379862	0.002066642	1.616093105
RNA5SP427	0.165214239	4.35E-05	2.083955299
RNA5SP432	0.297331774	1.48E-05	2.058801244
RNA5SP436	0.047535346	2.94E-06	3.281890873
RNA5SP439	0	0.004606615	1.557019068
RNA5SP445	0.1544621	0.002020158	1.747063146
RNA5SP447	0.17563556	0.000242659	1.864368805
RNA5SP449	0.987305429	5.22E-05	1.833295761
RNA5SP469	0.403204097	0.001134629	1.666508171
RNA5SP477	0.707999553	0.00074241	1.676535836
RNA5SP478	0.029367376	1.55E-06	3.781998127
RNA5SP483	0.550922918	0.002404774	1.588692312
RNA5SP498	3.357651058	0.001299979	1.63183584
RNA5SP503	0.057490816	0.001523719	2.087148378
RNA5SP510	0.004744932	1.11E-06	9.930086783
RNA5SP522	0	0.004606615	1.557019068
RNA5SP91	0.154220865	0.000248571	1.95828807
RNASE2	8.476008247	6.05E-06	1.971566689
RNASE9	0.017075394	0.001851696	1.624815301
RNASEH1P	0.026094711	0.000465953	1.882455524
RNASEH2B	9.280239307	0.00260513	1.582373157
RNASEK	18.50014929	6.01E-05	1.824636187
RNASEK-C1	1.127776483	0.000401606	1.703688337
RNASET2	141.3092885	3.42E-07	2.149829452
RND3	53.19871684	0.003101224	1.570176783
RNF10P1	0.046201515	8.20E-07	2.506887331
RNF11	166.6317075	3.98E-06	0.487752359
RNF111	22.12627772	3.37E-05	0.524375219
RNF121	41.03736974	0.000177142	0.56075126
RNF13	109.6563212	1.47E-08	0.408003419
RNF139-AS	1.083112634	1.76E-06	2.03779313
RNF141	27.3226788	0.000465556	0.575098501
RNF144A-A	0.710097481	0.000292853	1.763913533
RNF146	43.45504779	0.001857416	0.613739123
RNF152	38.02394207	0.001303817	0.567528997
RNF166	21.50782363	0.000829383	1.652460863
RNF170	21.68222307	0.002602222	0.620362739
RNF175	1.796130285	0.0007829	1.672060295
RNF183	6.340711378	0.000850166	0.543689464
RNF185	67.49786603	5.78E-09	0.398459371
RNF185-AS	0	0.004606615	1.557019068
RNF19B	68.8805201	1.15E-06	0.457008083
RNF20	50.20724927	1.58E-06	0.464966665
RNF207	8.190794896	2.79E-07	2.135785681
RNF215	8.77416782	1.53E-06	2.061468578
RNF216-IT1	0.488272758	0.001458296	1.617554328
RNF216P1	9.861641373	0.001205388	1.636145788
RNF34	21.47061143	0.003528603	1.557986609
RNF38	40.56447438	4.60E-06	0.478705372
RNF40	43.32634065	3.47E-05	0.527677309
RNF41	32.12890739	1.28E-07	0.437768142
RNF5	307.0132353	0.00397965	0.645392314
RNF6	34.47931697	0.000484404	0.583394739
RNF6P1	0.063589176	0.000911678	1.714327877
RNF8	9.322033404	2.52E-05	0.513138561
RNFT1P2	0.496860864	7.39E-05	0.514088779
RNFT2	1.728591892	0.001638414	1.63444978
RNU1-103P	2.817963411	9.90E-05	1.787391914
RNU1-106P	5.60442928	0.000566169	1.678744728
RNU1-107P	0	0.004606615	1.557019068
RNU1-108P	0.420241821	1.61E-05	1.970576892
RNU1-112P	0.146495839	5.05E-05	2.070011039
RNU1-11P	1.075232417	0.0047503	1.776503955
RNU1-122P	1.55493375	5.50E-05	1.834099126
RNU1-123P	0	0.004606615	1.557019068
RNU1-125P	0	0.004606615	1.557019068
RNU1-128P	0	0.004606615	1.557019068
RNU1-132P	0.204746679	0.001049683	1.680214395
RNU1-134P	0.498636788	0.001468445	1.639604432
RNU1-142P	0.006247785	0.000807996	4.651555226
RNU1-149P	0	0.004606615	1.557019068
RNU1-36P	1.126061429	4.91E-05	1.843914735
RNU1-39P	0	0.004606615	1.557019068
RNU1-42P	0.16093523	0.001899386	1.736762238
RNU1-47P	3.19278909	0.004571669	1.597489572
RNU1-56P	0.511137779	0.00047623	1.705647544
RNU1-5P	0	0.004606615	1.557019068
RNU1-67P	2.068231434	3.39E-06	2.033940172
RNU1-86P	0	0.004606615	1.557019068
RNU1-8P	0.251646745	0.000648355	1.759105494
RNU1-95P	0	0.004606615	1.557019068
RNU1-97P	0	0.004606615	1.557019068
RNU12	0	0.004606615	1.557019068
RNU2-11P	5.82498707	1.01E-05	1.941906355
RNU2-22P	1.272002385	0.0005702	1.683852376
RNU2-2P	0	0.004606615	1.557019068
RNU2-34P	0.035961542	0.00196046	2.441956205
RNU2-36P	0.184427503	0.002800722	1.735887546
RNU2-4P	0	0.004606615	1.557019068
RNU2-63P	0.13511138	0.004796845	1.627604724
RNU2-68P	0.383513246	0.004000853	1.557703641
RNU4-16P	0.134784939	0.002174906	1.819389983
RNU4-32P	0	0.004606615	1.557019068
RNU4-39P	1.503684157	4.84E-05	1.834685895
RNU4-40P	0.531426155	4.02E-05	1.886183344
RNU4-42P	0.182245831	9.21E-05	1.96442698
RNU4-46P	0	0.004606615	1.557019068
RNU4-52P	0.521369665	0.0013447	1.628554235
RNU4-61P	0.221450333	5.06E-05	2.005312398
RNU4-62P	6.770428272	0.000338739	1.751850073
RNU4-65P	0.03327603	0.004426242	2.282985565
RNU4-90P	0.868559223	3.51E-05	1.938885291
RNU4-92P	0	0.004606615	1.557019068
RNU4ATAC	2.189823119	5.71E-06	2.199843708

RNU4ATAC	6.623277719	4.95E-06	1.974762426
RNU5B-2P	6.976836198	0.000293854	1.726042668
RNU5E-6P	0	0.004606615	1.557019068
RNU5F-1	0.644696361	0.003270486	1.575228698
RNU5F-6P	0.118514323	0.001410163	1.947016808
RNU6-1004	0.468851405	1.78E-06	2.041746355
RNU6-1010	2.697719356	2.16E-06	2.059744356
RNU6-1011	2.157271674	0.004339283	1.538580269
RNU6-1024	1.483860216	0.001408306	1.615892072
RNU6-1038	0.063403082	0.000494522	2.277549052
RNU6-1046	0.324576868	0.00455577	1.574732923
RNU6-1049	0	0.004606615	1.557019068
RNU6-105P	0	0.004606615	1.557019068
RNU6-1060	0.104366173	0.004266522	1.918557399
RNU6-1064	0.332280942	0.003007874	1.639772933
RNU6-1066	0.10041955	0.00380009	1.850161211
RNU6-1069	0.498130847	0.002694107	1.591075174
RNU6-106P	0.259539653	0.002912475	1.633059103
RNU6-1071	0	0.004606615	1.557019068
RNU6-1076	0	0.004606615	1.557019068
RNU6-1077	0.371114022	1.71E-06	2.125574758
RNU6-1083	0	0.004606615	1.557019068
RNU6-109P	0.001932375	2.94E-11	46.37137314
RNU6-10P	1.164655862	0.00015438	1.789440867
RNU6-1101	0.336864449	0.000768193	1.739693577
RNU6-1109	0.391176394	0.00085196	1.702445483
RNU6-1114	0.160442005	0.001261479	1.801950332
RNU6-112P	0	0.004606615	1.557019068
RNU6-1132	0	0.004606615	1.557019068
RNU6-1136	2.187174441	0.003297388	1.557508542
RNU6-1157	2.426996551	0.00223504	1.583197817
RNU6-1169	0.128698338	0.003786799	1.813532697
RNU6-1171	0	0.004606615	1.557019068
RNU6-1183	0.26800669	0.004882903	1.733202755
RNU6-1186	0.067187871	0.003687177	2.065118863
RNU6-118P	3.239489942	6.06E-06	1.957367182
RNU6-1193	0	0.004606615	1.557019068
RNU6-1219	0	0.004606615	1.557019068
RNU6-121P	0.050690336	0.004011543	2.133460551
RNU6-1235	0	0.004606615	1.557019068
RNU6-1239	0	0.004606615	1.557019068
RNU6-1240	0	0.004606615	1.557019068
RNU6-1241	0.093160928	0.002510045	1.92002016
RNU6-1247	0.364798466	0.001368735	1.682358344
RNU6-1262	1.323054783	0.002425209	1.579267318
RNU6-1266	0.771290247	0.001040871	1.667731717
RNU6-1268	0	0.004606615	1.557019068
RNU6-1269	0	0.004606615	1.557019068
RNU6-1280	3.716877884	0.000593875	1.70200672
RNU6-1292	0.16435985	2.71E-05	2.308794397
RNU6-1293	0	0.004606615	1.557019068
RNU6-1299	0.112070136	0.000907678	1.92324432
RNU6-1300	0.368362408	0.000229732	1.787858088
RNU6-1315	0.565816517	0.000211842	1.754441086
RNU6-1324	0.156204067	0.002677972	1.706771619
RNU6-1330	0	0.004606615	1.557019068
RNU6-1340	0.091975005	4.32E-05	2.322321774
RNU6-140P	0.253918239	0.000205705	1.866466939
RNU6-146P	0	0.004606615	1.557019068
RNU6-170P	0.032335388	0.002764882	2.311774349
RNU6-174P	0	0.004606615	1.557019068
RNU6-184P	0.001977305	0	261.7554331
RNU6-189P	0.052490008	0.000809377	2.399531342
RNU6-190P	1.454959978	8.29E-05	1.802864533
RNU6-200P	0.192958128	0.001447462	1.833107991
RNU6-201P	0	0.004606615	1.557019068
RNU6-206P	0.035817786	0.00148124	2.587625576
RNU6-218P	0.516377357	0.001155726	1.635784449
RNU6-238P	0.535484615	9.71E-06	1.948681417
RNU6-244P	0	0.004606615	1.557019068
RNU6-249P	0.223400762	0.001898423	1.713511166
RNU6-259P	0.01510596	0.00101723	3.57874049
RNU6-26P	2.866428113	7.85E-07	2.089272459
RNU6-286P	0	0.004606615	1.557019068
RNU6-306P	0.268536045	0.001045765	1.788220968
RNU6-323P	1.124477929	3.99E-06	1.985339184
RNU6-327P	0	0.004606615	1.557019068
RNU6-341P	1.815400882	1.59E-05	1.92071669
RNU6-342P	0.211138465	0.000383223	1.956615028
RNU6-344P	0.154432908	0.002535597	1.774901126
RNU6-353P	1.026122647	1.13E-08	2.317843582
RNU6-363P	0.002014968	0	261.7554331
RNU6-364P	0.059648285	0.003947264	2.245194997
RNU6-368P	0	0.004606615	1.557019068
RNU6-370P	0.158655453	0.001607983	1.835261085
RNU6-375P	0.517297771	0.000927908	1.648134143
RNU6-37P	1.661714707	2.98E-08	2.290274528
RNU6-384P	0	0.004606615	1.557019068
RNU6-394P	0	0.004606615	1.557019068
RNU6-395P	0	0.004606615	1.557019068
RNU6-398P	0	0.004606615	1.557019068
RNU6-403P	1.83823197	0.002414102	1.796330971
RNU6-424P	0.037662203	0.001105831	2.562143462
RNU6-44P	2.922085673	0.002466258	0.55744107
RNU6-450P	0.432380791	0.003064483	1.576493761
RNU6-451P	0.134789462	0.002264197	1.87303202
RNU6-458P	0	0.004606615	1.557019068
RNU6-45P	0.938971377	2.52E-06	2.017738262
RNU6-46P	1.675002729	0.000232086	0.520626851
RNU6-473P	0	0.004606615	1.557019068
RNU6-482P	0.217668623	0.000435252	1.847279745
RNU6-497P	0.203322833	0.00055541	1.864790676
RNU6-498P	0	0.004606615	1.557019068
RNU6-501P	0.391925438	0.000636473	1.700172284
RNU6-507P	0	0.004606615	1.557019068
RNU6-513P	0	0.004606615	1.557019068
RNU6-529P	6.546503551	0.001530328	1.616151021
RNU6-531P	5.334928164	3.66E-05	0.504501172
RNU6-558P	0.328998574	4.32E-05	1.891095742
RNU6-574P	2.856376121	6.98E-05	1.813723884
RNU6-595P	1.772755749	0.003789614	1.560532685
RNU6-605P	0.061461176	0.003074039	1.990854735
RNU6-610P	2.978645209	0.000898427	1.650653743
RNU6-623P	1.513799417	0.000285339	1.721965894
RNU6-627P	0.007960401	0.004663476	3.348524836
RNU6-62P	2.159893383	0.000618384	0.547027862
RNU6-631P	0	0.004606615	1.557019068
RNU6-636P	0.172187506	0.000682539	1.987328137
RNU6-650P	0.159601797	2.92E-05	2.146983535
RNU6-665P	0.558392592	0.003961991	1.548281073
RNU6-678P	1.675238741	0.00030492	1.723422701
RNU6-67P	0.108719395	0.00063607	2.205061638
RNU6-685P	0	0.004606615	1.557019068
RNU6-702P	0.417285038	0.000446433	1.713465605
RNU6-710P	0.051468334	0.0048721	2.100760554
RNU6-712P	0.001256151	5.10E-06	22.83636517
RNU6-722P	6.996958615	0.000278106	1.731562222
RNU6-725P	0.895063326	0.002628799	1.626900685
RNU6-731P	0.394022151	0.004558433	1.54841245
RNU6-758P	0.860197817	0.001409692	1.638636127
RNU6-762P	3.145977829	3.61E-06	1.991181768
RNU6-777P	0.20918663	0.001211762	1.769237234
RNU6-783P	0.214574754	0.00127763	1.842795919
RNU6-785P	0	0.004606615	1.557019068
RNU6-788P	0.024978878	0.00123233	2.875417482
RNU6-799P	0.078132148	0.000497828	2.086308655
RNU6-79P	0.242288458	0.000411492	1.831361648
RNU6-810P	0	0.004606615	1.557019068
RNU6-816P	0	0.004606615	1.557019068
RNU6-826P	0.235517665	0.000134984	1.922507594
RNU6-831P	0.189005	0.000399152	1.915534709
RNU6-853P	1.446680808	0.001147804	1.635524002
RNU6-858P	0.344778456	0.000727675	1.674656975
RNU6-871P	0.113606067	0.000191108	2.140946446
RNU6-877P	0.294553061	0.001995196	1.648238397
RNU6-87P	0.361684132	0.004589105	1.551568733
RNU6-885P	0	0.004606615	1.557019068
RNU6-887P	0.079562036	0.001201987	2.120084886
RNU6-888P	0.725592908	0.001404994	1.626105969
RNU6-900P	0	0.004606615	1.557019068
RNU6-911P	0.094672602	0.000846331	2.202019231
RNU6-914P	0	0.004606615	1.557019068
RNU6-918P	0.181655255	0.002182117	1.7177603
RNU6-919P	0.23550976	0.001301051	1.779037499
RNU6-928P	0.25475312	0.004385307	1.641319119
RNU6-933P	0.349232241	0.00199311	1.63858895
RNU6-941P	0	0.004606615	1.557019068
RNU6-94P	2.65216142	5.95E-06	1.977018717
RNU6-968P	0.08469836	0.000936084	2.0894614
RNU6-97P	1.036829887	0.001204624	1.639435113

RNU6ATAC	0.280266389	0.002139703	1.653379037
RNU6ATAC	0.938982282	0.00218602	1.597035547
RNU6ATAC	0.240513655	0.000353587	1.818018268
RNU6ATAC	0.442716219	0.000703373	1.68882985
RNU6ATAC	0.315980671	0.003071511	1.623139983
RNU7-1	0	0.004606615	1.557019068
RNU7-104P	0	0.004606615	1.557019068
RNU7-10P	0.415692416	0.001353412	1.759973893
RNU7-111P	0	0.004606615	1.557019068
RNU7-115P	0.323163395	4.62E-05	2.030130848
RNU7-123P	2.574283308	0.004977633	1.532511921
RNU7-128P	0.579050079	0.000281415	1.797376991
RNU7-140P	4.034779517	0.000870133	1.652159796
RNU7-143P	0.251213223	1.24E-08	2.719506438
RNU7-14P	0.239375572	0.000201821	2.020652038
RNU7-151P	1.289908822	0.000983669	1.655642452
RNU7-174P	0.368336832	9.36E-06	2.163372674
RNU7-186P	3.539566731	0.000142749	1.781042614
RNU7-193P	0.374165084	5.97E-06	2.109604396
RNU7-197P	0.542136761	0.002756594	1.661773131
RNU7-37P	0.005106879	0.002020308	5.028897042
RNU7-40P	5.622383611	0.000166043	1.761532753
RNU7-41P	0.450684305	0.002442359	1.672767293
RNU7-49P	8.002084299	0.001275684	1.62402868
RNU7-4P	0.552128222	0.001526017	0.435595581
RNU7-50P	0.421207582	8.41E-05	1.942548226
RNU7-51P	0.00202605	5.10E-06	22.83636517
RNU7-77P	0.99696341	1.13E-05	1.936136652
RNU7-84P	2.844918697	0.001189642	0.509018896
RNVU1-14	0	0.004606615	1.557019068
RNVU1-3	3.089057405	5.57E-05	1.841113954
RNY1P10	0.988139093	1.51E-05	1.985352083
RNY1P6	0	0.004606615	1.557019068
RNY3P15	0	0.004606615	1.557019068
RNY3P16	5.797866406	8.97E-05	1.81036324
RNY4P34	0.92134512	0.00014915	1.82852061
RO60	21.27505399	0.002100864	0.619047381
ROBO3	4.292299705	4.30E-07	2.124413667
ROCK1	28.45019018	1.87E-05	0.49808469
ROCK2	30.68037103	0.000132635	0.537174894
ROMO1	226.1152343	0.002143303	1.599843638
ROR1	5.6089944	0.000210171	0.543842403
ROR1-AS1	0.245052527	5.38E-13	3.027842056
RORA	14.75010706	8.45E-07	0.440464288
RORC	44.47179387	0.001715872	0.602073323
RP1	0.162940969	3.20E-05	1.894809957
RP2	35.5486349	0.00054168	0.585783252
RP9	15.25671121	0.000312442	1.725995917
RPA1	63.26304048	0.002971389	0.636788397
RPA4	0.35902704	0.000699408	1.670659498
RPH3AL	11.80857044	0.000827434	0.577732934
RPL10P2	0.302107012	0.000267755	1.747315542
RPL12P16	0	0.004606615	1.557019068
RPL12P21	0.347243528	4.59E-08	2.247049152
RPL12P28	0	0.004606615	1.557019068
RPL12P32	0.351779328	0.001482257	1.617047589
RPL12P45	0.120411308	0.001122241	1.713778032
RPL13	686.4095861	0.000639775	1.676946924
RPL17P40	0.305752026	0.002649878	1.62371865
RPL17P50	14.8034261	1.07E-06	2.076797671
RPL18P10	0.924240139	0.000939105	1.655341441
RPL19P20	1.140679415	0.00468998	1.53584965
RPL21	409.3967831	0.000533307	0.570281147
RPL21P1	0.438204892	0.002903679	1.58228478
RPL21P106	0.206512011	0.000521826	1.718416294
RPL21P126	0.071556383	0.001280766	1.689040351
RPL21P33	0.152735166	0.002280715	1.601413551
RPL21P6	0	0.004606615	1.557019068
RPL21P89	1.171292607	0.001723825	1.606208748
RPL21P93	0.748267737	0.004060414	0.619875496
RPL22	240.6352891	0.000195499	0.557767528
RPL22L1	38.68923671	9.90E-09	2.34036075
RPL22P19	1.065083321	0.003666403	1.555414592
RPL23AP1	0	0.004606615	1.557019068
RPL23AP34	0	0.004606615	1.557019068
RPL23AP42	16.63907079	0.001129758	0.554311427
RPL23AP50	0.194432036	0.000208573	1.761231504
RPL23AP59	0.173599649	0.002772494	1.573847643
RPL23AP67	0.484742498	0.001491579	1.61706112
RPL23AP71	0.111578591	0.000574053	1.694457243
RPL23AP8	0.108027391	0.000940269	1.649522558
RPL23AP82	7.626699174	0.000576567	0.583495553
RPL23AP91	0.133787932	0.00498981	1.563334475
RPL23AP93	0.424164625	0.002056101	1.634810699
RPL23P2	4.401947735	1.10E-07	2.191754911
RPL26P29	0.299222008	8.91E-05	1.824944061
RPL27A	386.0524846	0.000315715	1.723437855
RPL27AP	0.321687274	0.00097682	1.646946185
RPL28	324.9671856	4.32E-05	1.851607523
RPL29	808.5220161	0.000532654	1.687548051
RPL29P14	1.947586947	6.89E-12	2.750967643
RPL29P19	2.539815843	7.42E-08	2.304136016
RPL29P23	0	0.004606615	1.557019068
RPL31P15	0	0.004606615	1.557019068
RPL31P52	1.097650648	2.51E-05	1.87602174
RPL32P26	0.359341072	0.000304218	1.776347842
RPL32P28	0.231783959	2.04E-05	1.996553406
RPL32P29	0	0.004606615	1.557019068
RPL32P3	6.937012834	0.000253713	1.733546738
RPL35	1011.015777	0.00036289	1.715593539
RPL35AP	0.082064676	0.001212188	1.72216089
RPL35P9	0.289248587	0.000313012	1.779273574
RPL36A	73.27611198	3.62E-05	1.861381436
RPL36P4	0	0.004606615	1.557019068
RPL37P1	0.837204202	0.0025355	1.582881143
RPL38	351.1296779	0.002118887	1.592872866
RPL39P	0	0.004606615	1.557019068
RPL39P28	0.064685115	0.002891314	1.944964295
RPL39P34	0	0.004606615	1.557019068
RPL4P6	0.941434483	0.000718567	1.666533343
RPL5P13	0.108493783	0.001085799	1.814543865
RPL5P14	0.215442944	0.00046702	0.510488825
RPL6P7	0.189983164	0.000101277	1.81013422
RPL6P9	0.085010741	0.000299529	1.737629372
RPL7AP52	0.058024042	2.37E-05	1.927188031
RPL7AP69	0.055317596	0.000453078	1.757706871
RPL7L1P9	0.999948925	0.004213865	1.579198123
RPL7P18	0.400907006	0.000187192	1.770212537
RPL9P25	0	0.004606615	1.557019068
RPL9P29	4.509975375	0.000310779	1.721961289
RPLP0P1	0.072373126	0.000770384	1.705350363
RPLP0P2	0.655999255	3.76E-09	2.614968291
RPLP1	1202.5814	2.24E-05	1.896652577
RPLP1P11	0	0.004606615	1.557019068
RPLP1P6	9.998317056	0.00025063	1.737984288
RPLP2	1188.46341	5.32E-05	1.838104318
RPP14	7.919633868	0.000184906	0.533006493
RPP40	6.974695487	0.000300917	1.728648473
RPPH1	0	0.004606615	1.557019068
RPPH1-2P	0.01326835	0.002806243	2.460172417
RPRD1A	26.36933049	0.000695031	0.588030769
RPRD2	38.28314248	0.000123458	0.544600222
RPS10-NUD	0.192976271	0.004454092	1.541683797
RPS10P18	0.183901894	0.000480712	1.70591261
RPS10P20	0	0.004606615	1.557019068
RPS10P21	0.154776003	2.40E-05	1.973533731
RPS10P7	1.753577804	0.001449958	1.625065655
RPS12P16	0.527504128	2.00E-05	1.933204976
RPS12P21	0	0.004606615	1.557019068
RPS12P28	0	0.004606615	1.557019068
RPS12P31	0.456061717	0.001835186	1.613176193
RPS14P4	0.369591284	0.000230278	1.756669972
RPS15AP30	0.685776995	6.32E-08	2.261369052
RPS15AP6	0.695608104	6.31E-07	2.10733651
RPS17	604.6584589	0.000169354	1.763892797
RPS19	815.9291697	0.000453429	1.698726628
RPS19P3	2.838837391	0.001028248	1.638537794
RPS2	1333.188545	0.001226551	1.633084984
RPS20	1135.038721	0.000199627	1.754023978
RPS20P20	0.134299608	0.003066779	1.601931259
RPS20P21	0	0.004606615	1.557019068
RPS21P4	0	0.004606615	1.557019068
RPS23P3	0.167043856	0.000454165	1.845787696
RPS23P6	3.104434647	0.003243211	1.599012375
RPS23P7	0.162890472	0.002953103	1.592089689
RPS24	708.684616	0.001732422	1.609784711
RPS24P18	0.128167203	0.004396773	1.552903322
RPS26P45	0.403672826	0.000723997	1.668291865
RPS26P56	0.364203757	0.000530889	1.730813959
RPS27AP12	0.77411248	4.57E-05	1.843445139

RPS27AP13	0.164266145	8.13E-06	2.029057376
RPS27AP18	0.10302221	0.000105949	1.830402982
RPS27AP3	0.21825063	0.002928876	1.577868879
RPS27AP8	0.199086428	0.00064561	1.72736406
RPS29P24	0.293384777	0.000351452	1.785961068
RPS2P24	0.182301874	0.001020313	1.649582782
RPS3AP38	1.138202388	0.000517711	1.684678172
RPS3AP6	6.952250802	0.003019	0.583661626
RPS4XP5	0.107345045	0.004242764	1.575767527
RPS5P7	0.117115289	4.36E-05	1.90393592
RPS6KA2	39.75125267	1.48E-05	0.47957167
RPS6KA4	36.58567177	0.001749398	1.607248799
RPS6KA5	1.474892403	0.002460984	0.616371131
RPS6KA6	2.69292374	0.000678511	0.53764577
RPS6KB2	22.84218665	0.001226272	1.634247265
RPS6KB2-A	0	0.004606615	1.557019068
RPS6KL1	2.988903463	0.001234079	1.643646802
RPS6P12	0.037572126	0.001482681	1.752020062
RPS6P20	0.070629309	0.000283634	1.78425105
RPS6P21	0.150968642	5.05E-05	1.911699759
RPS6P4	0.010173348	0.002972543	2.183698475
RPS7P3	0.869887903	1.89E-05	1.901281703
RPS7P7	0.141148639	0.000789859	1.680335542
RPS9P1	0	0.004606615	1.557019068
RPSA	374.6294711	4.66E-05	1.845488153
RPSAP11	0.268063459	0.004962771	1.533577147
RPSAP16	0.205014597	8.31E-07	2.096537234
RPSAP26	0	0.004606615	1.557019068
RPSAP36	0.364243665	0.000513824	1.702780821
RPSAP51	0.116236586	0.00401857	1.571984495
RPSAP52	0.230217814	1.10E-10	3.021763671
RPSAP69	0.179651385	0.001629512	1.637604925
RPSAP71	0.191861132	6.52E-05	1.998259517
RPUSD3	11.55265305	0.001562602	1.613196665
RRAGA	312.0405839	0.000471623	0.583107333
RRAGD	62.17803385	1.90E-05	0.479161746
RRAS	224.7832988	1.13E-05	1.937644067
RRM1-AS1	0.196345469	0.000227439	1.738485347
RRM2	12.21839944	1.32E-06	2.075228538
RRM2B	62.17391263	0.001802846	0.606357706
RRN3P2	2.173408633	0.000260276	1.73501445
RRP12	13.58678238	8.08E-06	1.954067139
RRP7BP	3.140004205	1.88E-05	1.895096711
RSBN1	19.98463875	0.001081518	0.600177214
RSC1A1	0	0.004606615	1.557019068
RSF1	15.82017178	0.000172935	0.550228806
RSKR	2.730965958	0.000318142	1.716238551
RSL1D1	121.7264758	0.001465713	0.610771481
RSL24D1	143.3524372	0.000251197	0.544202727
RSL24D1P1	0.704056121	0.001812601	1.605213281
RSL24D1P9	0.051199717	3.70E-05	1.958007247
RSPH10B	0.06913833	0.000116739	1.788363231
RSPH10B2	0.035169543	2.00E-05	1.907943907
RSPH3	18.96541918	0.000295269	0.556453499
RSPH6A	0.115624728	0.002036901	1.678441951
RSPO4	1.44106944	1.69E-08	2.560715729
RSRP1	28.4813468	0.002500344	1.574018358
RTBDN	0.139372598	0.000136628	2.014221607
RTCA-AS1	7.183604154	0.000444199	0.565636461
RTEL1-TNF	1.894828701	0.001427485	1.616854349
RTEL1P1	0.077665701	0.00444569	1.543037898
RTKN2	0.969495225	2.35E-05	1.887301794
RTL1	1.798905619	5.10E-07	4.743783666
RTL6	37.17231596	0.000237528	0.559742133
RTL9	0.173074092	0.002789901	1.738817807
RTN4	256.1122367	0.002689046	0.606398845
RTN4R	4.139498678	0.004739272	1.553711484
RTP1	0.038873199	0.000650453	1.70143391
RTP5	0.355845891	0.001464136	1.646877726
RUFY4	1.154572172	2.66E-07	2.200204599
RUNDC1	21.08240969	0.00094316	0.597252143
RUNDC3B	4.663793023	3.78E-05	0.505770506
RUNX1	18.87934958	1.53E-07	2.180360048
RUNX1T1	2.199957856	0.002842248	0.603517229
RUNX2	5.84067477	0.000636191	1.67528978
RUSC1-AS1	6.261726845	0.00023635	1.732570013
RXRA	37.87474083	0.000110091	0.525917104
RYBP	33.09105359	2.38E-05	0.496669411
RYKP1	0.228458417	5.97E-05	1.823953278
RYR2	0.961492263	1.78E-06	2.095539964
S100A11	1796.127903	1.15E-07	2.205061528
S100A13	112.1850532	0.000136465	1.775986603
S100A16	269.9991215	0.00109817	1.638766533
S100A5	1.321036238	0.001230211	1.750202449
S100A6	1641.831709	0.000778694	1.671005802
S100A7	0.442036938	1.69E-05	2.146550771
S1PR1	142.5873673	4.36E-06	0.468686389
S1PR2	7.17444539	0.002481999	1.580494147
SAA1	386.1408916	3.15E-06	2.371502067
SAA2	30.8130838	0.004035399	1.843579021
SAA3P	0.025902107	0.002467987	1.878625648
SAA4	2.745956283	0.000591408	1.983522438
SAAL1	10.92455978	0.001355858	1.62259141
SAC3D1	12.16716222	6.54E-07	2.109305854
SACS-AS1	0.072850437	3.48E-05	1.931715918
SAG	0.036281293	2.31E-05	1.917074811
SAGE1	0.106975067	0.002590838	1.659791465
SALL1	57.27155639	4.47E-08	0.386422858
SALL4	0.769190759	0.000495869	1.762576049
SALL4P5	0.03329977	0.003226837	1.586366935
SAMD1	47.17214344	2.36E-07	2.181329346
SAMD15	3.878876442	0.000530206	0.569833736
SAMD5	11.39665379	0.000420602	0.559431946
SAMD8	20.62017248	0.001173625	0.595721603
SAMM50	34.68767893	1.27E-06	0.466973273
SAP25	0.576865675	1.50E-06	2.03940882
SAP30L	36.13941865	1.54E-06	0.457197166
SAPCD1	2.615294536	0.000108125	1.786778004
SAPCD2	2.940219389	3.40E-07	2.166553817
SAPCD2P3	0.160110312	0.004982718	1.533882659
SARAF	724.535355	1.03E-06	0.459235612
SARNP	2.903847778	0.002912188	1.566064805
SARS2	6.181121611	9.15E-10	2.478894572
SASH1	40.11205657	9.17E-06	0.4802927
SATB1	8.328553441	0.000142942	0.515396458
SATB2	8.673396856	0.000135802	0.526448846
SAV1	28.49244925	6.31E-06	0.482189229
SBDS	256.881707	9.23E-05	0.541082845
SBF2	15.57001995	6.70E-05	0.5281977
SBF2-AS1	9.956271501	0.000189762	0.510748781
SBK2	0.221962389	0.000703463	1.748432948
SBNO2	44.39594896	2.17E-07	2.18058353
SBSN	1.367273636	5.78E-05	2.565889363
SC5D	25.73464058	0.003392687	0.541290394
SCAF11	40.35498338	0.000271708	0.562800779
SCAF8	31.27412108	0.000570832	0.582067186
SCAMP1	65.27442116	0.000906105	0.57100157
SCAMP2	108.9442198	0.002243852	0.62473168
SCAMP4	33.87207323	0.003391802	1.558344039
SCAND1	82.66534986	0.002344386	1.587177654
SCAPER	7.36748518	5.68E-06	0.480446195
SCARB2	240.1879258	0.001840955	0.619193221
SCARNA12	4.456075179	0.000142304	2.125265124
SCARNA15	0	0.004606615	1.557019068
SCARNA16	0	0.004606615	1.557019068
SCARNA17	0	0.004606615	1.557019068
SCARNA2	0	0.004606615	1.557019068
SCARNA21	8.200735662	3.90E-08	2.712383518
SCARNA23	0.243564599	0.000281366	2.059722461
SCARNA8	1.44718139	0.001732635	1.620539833
SCART1	1.487319831	0.0038933	1.550521756
SCAT1	0.676003498	6.60E-07	2.184754532
SCAT2	2.499962532	5.84E-10	2.482703396
SCAT8	0.400032974	8.26E-10	2.485955877
SCD5	40.77480059	0.000829235	0.498144157
SCFD1	28.10969587	0.002036704	0.615413062
SCFD2	24.12076144	0.000438243	0.576264318
SCGB1A1	0.177482573	2.42E-05	1.903792913
SCGB1B2P	4.457711999	1.46E-06	2.054176497
SCGB1D2	11.16176398	0.000625814	0.536202569
SCGB2A2	0.311922065	5.69E-05	1.903766394
SCGB2B3P	0	0.004606615	1.557019068
SCN4A	4.165806315	0.000141329	0.497552302
SCN4B	30.40531462	0.001058701	0.570665971
SCNM1	30.00015407	9.93E-06	1.951856224
SCNN1D	4.823458673	0.00122867	1.630053059
SCO2	55.42910847	0.00032119	1.721100589
SCOC	40.90975338	0.000123715	0.50390595
SCP2	83.1340593	8.80E-09	0.407899551
SCRIB	31.00772153	0.003534942	1.555579717

SCRN3	19.84832841	8.94E-07	0.443500362
SCX	2.878178128	2.51E-06	2.109791434
SDAD1P1	6.850158227	0.002736291	0.622863035
SDE2	32.92377728	0.000209637	0.55328546
SDHAP2	0	0.004606615	1.557019068
SDHB	116.8580198	1.62E-05	0.503217531
SDHC	34.16920708	0.000194901	0.553224331
SDHD	141.2405878	2.89E-05	0.503769125
SDR42E1P1	0	0.004606615	1.557019068
SDR42E2	0.332299541	0.002426429	1.58513639
SDR9C7	0.076830152	1.47E-09	2.431535564
SEC14L1	80.02353383	0.002065526	0.608837481
SEC14L3	0.110703056	0.000157734	1.793996615
SEC24B	47.07908691	2.10E-07	0.431136162
SEC24C	39.91815189	0.002792107	0.629531867
SEC31A	118.937181	0.000114972	0.5444478
SEC31B	4.60982736	0.003515752	1.550273824
SEC61A2	6.436246715	1.52E-06	2.052241252
SEC62	120.0808465	0.000125311	0.553983007
SEC63P2	0.026727486	1.15E-06	2.151949976
SECISBP2L	43.82436451	3.91E-07	0.425754642
SEL1L	75.11216095	0.004691899	0.642298319
SELENOKP	0.070806036	0.000588661	1.687655093
SELENOM	74.78240406	0.000773389	1.662658425
SELENOP	141.7782406	1.49E-07	0.422722416
SELENOWI	0.185722212	0.003252027	0.628585339
SEM1	24.33632877	0.000142488	1.774399633
SEMA3A	1.731761453	6.16E-07	2.219294198
SEMA3C	31.32065966	0.000404349	1.779502904
SEMA3G	26.06863721	6.55E-08	0.381182305
SEMA4B	71.49505738	0.002481214	1.609848071
SEMA5A	24.04923126	2.03E-06	0.452937811
SEMA6A-AS	1.811606613	0.00021797	0.548637022
SEMA6D	5.525436847	0.000876016	0.544594556
SEMA7A	7.737757518	0.000365615	1.723618185
SENP8	4.601750655	5.87E-09	0.386074906
SEPHS2	223.3903228	0.000635458	0.542968348
SEPSECS	21.41772528	0.001026444	0.589492225
10-Sep	80.79905543	3.10E-05	0.51534003
SEPT10P1	0.424213224	0.001228214	0.591284838
11-Sep	77.07360546	2.04E-07	0.430971727
12-Sep	0.094094162	0.000243671	1.795974261
SEPT14P17	0.043758997	0.001876205	2.258386749
SEPT4-AS1	1.070155674	1.01E-05	1.932741176
SEPT7-AS1	2.388873662	0.00141938	0.590490382
SEPT7P9	0.834987103	0.001861458	1.600377323
SERBP1	128.6644121	0.000192878	0.562825191
SERBP1P3	0.453557446	1.38E-08	2.322712835
SERBP1P5	0.706828759	0.000230972	0.548032513
SERF1B	0.726537271	0.002369424	0.606807461
SERF2	199.6257507	0.000243111	1.740519508
SERINC1	489.8756443	6.97E-06	0.485304209
SERP2	8.710832167	0.000528419	0.584169594
SERPINA3	0.531254358	0.000135068	1.915305984
SERPINB2	0.198809894	0.000365333	1.98290112
SERPINB3	0.079712435	0.001742047	1.872018812
SERPINB4	0.074323939	2.38E-05	2.303900936
SERPINE1	517.8422581	0.002288071	1.642567737
SERPINF1	79.70390209	0.000240296	1.774515255
SERPINH1	216.7033662	0.000123452	1.782408463
SERPINI1	50.18096357	0.00247859	0.60807185
SERTAD2	43.46614428	3.47E-05	0.523518494
SERTAD4	9.749554927	0.002766824	0.616082684
SERTM1	0.176414677	1.10E-05	2.20588857
SESN1	28.44380583	0.001676154	0.588202343
SESTD1	17.79305479	3.11E-09	0.362033525
SET	245.340826	0.000543611	0.589596104
SETBP1	12.69782371	0.00021979	0.548308129
SETD2	23.65125704	6.07E-05	0.513968329
SETD3	92.09669018	6.54E-06	0.484927095
SETD4	9.320682893	4.47E-05	1.842008944
SETP12	0.266712796	0.003951444	1.546997761
SETX	35.46874372	2.21E-05	0.504587431
SF1	139.6245203	0.003065007	0.62618985
SF3A1	87.35332351	4.56E-07	0.457046579
SF3B2	77.88996954	0.000160171	0.561814654
SF3B4	110.1593893	0.000154901	1.779546657
SFI1	7.62342071	0.00044375	1.691555747
SFR1	21.4155153	0.00090982	0.59158283
SFRP5	0.378902714	3.10E-06	2.673944405
SFSWAP	18.56905473	0.000255819	1.730752706
SFT2D2	43.15641423	0.003622506	0.635112332
SFT2D3	0	0.004606615	1.557019068
SFTA3	0.020423745	0.000179564	1.831944323
SFTPA1	0.051341095	0.000139638	1.874304313
SFXN1	44.20726603	0.000742441	0.581144085
SFXN2	7.890313401	0.00265119	0.599287826
SGCB	147.2191713	2.93E-12	0.323968002
SGCZ	0.036374138	0.003585494	1.71414901
SGK1	87.76619787	8.55E-05	0.516644501
SGMS1	25.49230947	5.86E-08	0.405351157
SGMS1-AS1	3.090390454	6.44E-05	0.524254214
SGMS2	28.92310626	1.89E-05	0.49303551
SGO1	1.124284075	1.97E-06	2.043227239
SGO1-AS1	0.041945549	0.000107177	1.810378931
SGPP2	67.34398959	0.001594768	0.609620513
SGSH	26.35875121	6.01E-05	1.844429451
SGSM1	5.02055007	0.000333978	0.557489345
SGSM3	39.95236245	0.000748035	1.655412179
SGTB	11.13442249	0.000118015	0.533153482
SH2D1B	2.39796436	0.002669542	0.610231236
SH2D2A	10.88253667	0.000214401	1.753948578
SH2D4A	16.68330251	0.000876901	0.565676578
SH2D6	0.287938573	1.54E-08	2.384841329
SH3BGRL2	36.11483383	9.32E-08	0.413118286
SH3BGRL3	385.3778598	4.61E-08	2.262535682
SH3BP4	48.30549455	2.50E-05	0.493998417
SH3BP5	19.29204675	6.23E-05	0.514705835
SH3BP5-AS	7.054211822	0.001365595	1.616470877
SH3D19	32.29898105	7.55E-08	0.420734948
SH3D21	15.81018606	0.000638365	1.667333124
SH3GL1P1	1.327728157	0.000345418	1.709270023
SH3GL1P3	0	0.004606615	1.557019068
SH3GL2	4.296313325	0.002553368	0.417174429
SH3GL3	0.153561794	0.000327288	1.779845234
SH3GLB1	95.36272882	0.003791532	0.640246318
SH3RF1	25.94047194	9.40E-08	0.421321499
SH3TC2	0.375512725	0.001387645	0.578102461
SH3YL1	25.62377601	0.00023268	0.550930484
SHANK2	8.917551322	4.63E-07	0.427942627
SHANK3	53.186168	0.003114549	0.616493056
SHARPIN	83.76119824	0.000125829	1.78595837
SHC1	158.5314763	2.53E-05	1.884474601
SHCBP1	4.387605701	1.24E-05	1.934060018
SHCBP1L	0.023923572	0.003444378	1.581499715
SHE	13.43528836	0.002124468	0.609458713
SHISA4	19.60952272	0.002963156	1.568235459
SHISA5	146.2958819	0.000290236	1.745142543
SHISA6	1.135806015	0.000462667	0.510350392
SHISA7	0.052641429	6.76E-07	2.094366668
SHISA9	8.737174659	0.000439818	0.550590864
SHISAL1	2.199515617	0.004658339	1.656685488
SHLD2	37.84498975	6.24E-05	0.528850876
SHMT1	93.54497414	0.000367886	0.574147911
SHOC2	56.00100228	0.000104118	0.545512395
SHOX	0.065523857	0.000794473	1.707339783
SHOX2	0.563551318	1.44E-06	2.199850678
SHPRH	3.142745483	0.002654051	0.613393075
SHROOM3	8.507854667	0.000315486	0.487650713
SHROOM4	19.28767272	1.60E-06	0.444817393
SI	0.011285265	6.76E-05	1.883890717
SIAE	43.83163766	0.000672022	0.578716792
SIAH1P1	0.036942268	0.001080296	1.747992008
SIAH2-AS1	1.277580925	0.003420139	0.61650638
SIGLECL1	0.067307615	0.00010707	1.942083061
SIK2	40.90320396	9.22E-08	0.400187725
SIK3	17.44769967	2.20E-05	0.502833378
SILC1	0.029296107	0.002192404	2.01057267
SIM2	0.98382457	5.57E-07	2.195620444
SIN3A	23.12443388	0.00194334	0.617624984
SINHCAFP3	0.091381694	0.00168817	1.648704807
SIPA1L1	29.24135829	4.25E-06	0.461930181
SIRLNT	0.158604664	6.02E-05	2.214215262
SIRPB3P	0.467806929	0.000306377	1.739012765
SIRPD	0.332932629	0.001946394	1.598308901
SIRT1	30.38826914	6.63E-05	0.529541211
SIRT3	24.88458463	0.00072024	0.589821132
SIRT5	14.59279261	0.000468142	0.573992844
SIRT7	11.29991586	3.91E-08	2.274468105
SIX2	1.76377967	3.20E-06	2.127658501

SIX3-AS1	0.062362629	0.004465036	1.722610083
SIX5	7.479099504	0.000495546	1.698358371
SKA1	2.35796413	4.23E-08	2.266220571
SKA3	3.382602703	1.14E-05	1.931642514
SKAP1-AS1	0.530109248	0.000203345	1.766564841
SKAP2	77.63205798	0.002189914	0.620743153
SKI	77.68156546	0.001131382	0.603339425
SKIDA1	1.112929299	0.001167822	0.577765904
SKINT1L	0.665666401	2.39E-05	1.884401307
SKIV2L	36.29418765	0.000900836	1.6544981
SKP1	90.42167566	5.80E-05	0.533670852
SKP2	11.09011439	3.20E-05	1.862634961
SLAIN2	38.22783717	0.004057285	0.644580629
SLAMF9	0.496240171	3.81E-07	2.424884159
SLC10A2	24.84523279	4.53E-08	0.346614297
SLC10A5	1.825752773	0.002654495	0.58513246
SLC10A6	3.902332187	0.000196352	0.504235291
SLC10A7	6.683638708	0.003617281	0.636594535
SLC11A1	6.752117618	5.32E-06	1.988487205
SLC12A2	14.76150433	0.001176711	0.575855737
SLC12A4	48.85245257	5.91E-05	0.529941076
SLC12A5	0.632180605	0.001229597	1.652926208
SLC12A5-A	0.406877634	3.77E-08	2.27779814
SLC12A6	16.90972132	1.33E-06	0.431447874
SLC12A7	155.1015477	4.26E-05	0.498834741
SLC12A8	6.204833834	7.23E-09	2.355030734
SLC12A9	19.33928202	0.002877516	1.569696439
SLC13A1	37.33424266	0.001846494	0.556109246
SLC13A4	0.951715976	0.000610115	1.677802333
SLC16A1-A	2.479272634	2.20E-06	2.026686059
SLC16A12	93.61048064	1.70E-09	0.347688356
SLC16A4	159.3307113	1.38E-05	0.500126747
SLC16A8	1.835332041	0.000889919	1.65256336
SLC16A9	90.642095	4.51E-07	0.382554362
SLC17A5	55.12166904	0.004774364	0.64448659
SLC17A9	8.50262928	5.31E-08	2.2731325
SLC18A3	5.78036707	1.73E-06	2.449104036
SLC19A1	6.144764647	4.09E-08	2.261486477
SLC1A1	75.56163207	2.25E-08	0.391507047
SLC1A5	88.34654159	9.64E-06	1.944106998
SLC20A1	33.73264795	0.000124329	1.788014722
SLC20A2	19.92086376	0.003386641	0.625881483
SLC22A1	1.284399762	6.08E-05	1.89991708
SLC22A11	72.19206564	0.001892189	0.59542745
SLC22A12	70.41670459	0.000184626	0.442775885
SLC22A13	2.767058506	0.000903089	0.527942563
SLC22A2	147.5334583	6.47E-07	0.446261191
SLC22A20P	0.461975023	6.70E-06	2.000724602
SLC22A23	9.429639504	0.002892369	0.601857488
SLC22A24	2.014824603	0.000637709	0.54545304
SLC22A31	0.240367307	6.25E-06	2.158992154
SLC22A4	24.58410303	7.10E-05	0.5018115
SLC22A6	55.9165142	3.46E-05	0.430980612
SLC24A1	7.781790601	0.001808266	0.607598074
SLC24A5	0.044854981	0.002606843	1.582962256
SLC25A13	47.23583712	1.91E-05	0.498927003
SLC25A19	8.515084523	0.003001929	1.56904772
SLC25A1P1	0.25782295	0.001225407	1.631761138
SLC25A1P2	0	0.004606615	1.557019068
SLC25A1P5	0.480435945	0.00411265	1.541123196
SLC25A2	0.21106686	1.60E-06	2.05487434
SLC25A22	14.26427898	0.000814577	1.66023573
SLC25A23	95.05138487	0.003714743	0.637628204
SLC25A24	30.05116773	4.54E-07	0.456047592
SLC25A24P	0.093832	0.002642717	1.63892233
SLC25A25	14.53154404	0.003686597	0.60508933
SLC25A25-A	5.724845319	0.003218422	1.56451054
SLC25A30	30.92401604	0.00013339	0.539540754
SLC25A31	0.042207299	0.001867454	1.620536814
SLC25A35	4.867219897	6.90E-05	1.834099947
SLC25A37	29.46387572	1.49E-05	1.908233704
SLC25A39	131.4797324	0.000337679	1.72503579
SLC25A3P1	0.014780812	0.001721059	1.632597565
SLC25A4	51.95385401	3.48E-06	0.440020759
SLC25A42	37.47210073	2.83E-05	0.500337923
SLC25A46	33.2581868	3.40E-05	0.510631266
SLC25A48	3.580702396	0.000289392	0.523281451
SLC25A51	10.4266995	0.000540156	0.576198939
SLC26A10	0.61016002	3.31E-05	1.865662396
SLC26A5	0.285831192	0.000398695	1.701334138
SLC26A6	5.767667436	7.81E-07	2.078317765
SLC26A8	0.130129061	0.003928771	1.549915673
SLC27A2	68.32959679	3.06E-11	0.31292952
SLC28A1	191.3566526	0.001493084	0.612981223
SLC2A13	13.55960632	0.004938327	0.622727124
SLC2A3P1	0	0.004606615	1.557019068
SLC2A6	7.604438018	1.39E-07	2.188150867
SLC2A9	19.63065887	1.94E-10	0.308498461
SLC30A1	56.70190367	0.003196745	0.622800285
SLC30A4	8.174419745	0.000181424	0.55258684
SLC30A9	51.08519992	4.02E-07	0.435119843
SLC31A1P1	0.977808433	0.003574503	1.569882425
SLC31A2	5.520721001	2.81E-05	0.483276731
SLC34A2	63.76663509	0.001274718	1.672478242
SLC35A3	26.26858497	1.52E-05	0.505818387
SLC35A4	132.1426132	0.000205832	0.559994166
SLC35A5	36.77585852	6.46E-06	0.492223411
SLC35B4	23.95424122	0.003733472	0.627774811
SLC35C2	29.19116582	1.08E-06	2.073256509
SLC35D2	61.58770435	0.001130221	0.607809161
SLC35F1	3.643510246	0.00315365	0.618753451
SLC35F3	3.2048626	0.00231266	1.592431201
SLC35F5	42.67737738	7.41E-06	0.480234544
SLC35G3	0.156615399	6.06E-07	2.206784952
SLC35G4	0.076030408	5.22E-05	1.978663634
SLC35G5	0.266822928	8.10E-06	1.950684074
SLC38A5	15.49215846	4.47E-09	2.621292719
SLC38A7	13.26918804	0.003667451	1.554256756
SLC39A11	20.42298483	0.000159978	1.767954346
SLC39A12-A	0.015655245	0.000416804	1.764786964
SLC39A5	78.03134951	0.003529069	0.632111346
SLC39A8	31.78891146	0.000548456	0.562037501
SLC39A9	57.07822239	2.73E-06	0.474402344
SLC3A1	290.1723049	1.57E-08	0.399731976
SLC40A1	221.3821958	3.98E-05	0.506115576
SLC41A1	41.22059357	0.000167421	0.549820456
SLC43A3	33.43755823	0.000127166	1.778132946
SLC44A1	32.17767107	2.58E-07	0.437378754
SLC44A2	181.8601646	0.001276483	0.597955206
SLC44A3	63.2629151	1.00E-04	0.535060077
SLC44A4	34.79314701	0.000348272	0.490362633
SLC45A4	19.49174622	0.001973845	0.584209734
SLC46A1	7.659524823	4.44E-05	0.517371023
SLC47A1	180.5123448	3.60E-05	0.513737436
SLC49A4	17.10252668	0.002615069	0.612441806
SLC4A3	3.680773008	0.00012569	1.830983757
SLC4A4	98.82304002	9.48E-07	0.430308413
SLC4A8	0.520420441	0.003572417	1.551629149
SLC50A1	59.35340194	2.18E-07	2.185910578
SLC52A1	0.878869814	0.000534445	1.763632431
SLC52A2	43.40959872	7.01E-08	2.2467911
SLC52A3	3.98812711	0.000597903	1.727943797
SLC5A1	49.27116596	0.00472689	0.611066127
SLC5A10	36.48178139	0.001875277	0.605180744
SLC5A12	59.77086241	1.30E-05	0.47561913
SLC5A5	0.204899488	0.002914713	1.612308453
SLC5A6	17.02687908	2.17E-06	2.031008385
SLC5A8	42.45708382	0.000605034	0.565181859
SLC6A13	174.5132134	9.73E-05	0.523662421
SLC6A14	0.052188202	5.72E-05	2.03611474
SLC6A17	0.766985736	0.001053713	1.69268226
SLC6A19	46.45275384	4.69E-06	0.338177544
SLC6A5	0.016450275	0.002268086	1.712971953
SLC6A7	0.087481511	0.000935598	1.664308558
SLC7A15P	0.034245954	0.000168942	1.807548609
SLC7A5	66.59241221	8.99E-08	2.227959769
SLC7A5P2	0	0.004606615	1.557019068
SLC7A9	28.51709506	0.000323923	0.557021918
SLC8A2	0.176932049	0.000203131	1.931835749
SLC9A3R2	138.1454412	9.05E-06	0.486767914
SLC9A5	1.711443345	0.0009804	1.639695829
SLC9A6	20.76082176	6.34E-05	0.53308462
SLC9B1P4	0.017555574	0.004231275	1.705816519
SLC9C2	0.357428128	0.000283329	1.872401426
SLCO1B7	0.013459634	0.003337775	1.659665433
SLCO2A1	136.8864641	0.000567682	0.568449493
SLCO4C1	89.1598516	4.40E-06	0.473836361
SLCO5A1	0.304831114	0.000769413	1.680941857
SLIT1	0.32066784	0.001062786	1.677421062
SLITRK2	3.269667279	0.000317972	1.753075634

SLK	53.3597732	7.93E-06	0.479993941
SLMAP	12.26200537	4.14E-05	0.510820065
SLN	7.010241438	0.000250159	2.051882654
SLPI	191.6655949	5.88E-05	2.045827605
SLU7	51.01045772	2.12E-09	0.378964101
SLX1A	0.064997059	8.64E-06	1.947484189
SLX1A-SUL	0.072297153	0.003913827	1.546548133
SLX1B	0.080640238	0.000345789	1.713460428
SLX1B-SUL	0.009493147	0.000235538	1.750588158
SLX4	4.645234402	0.004794473	1.539007074
SMAD4	24.6573716	0.000315586	0.566073152
SMAD5	37.44307834	0.000332289	0.558822216
SMAD6	7.398640217	0.00032201	0.56814896
SMARCA1	52.67717031	0.001305791	0.598482263
SMARCA2	64.11251455	0.00018171	0.552709697
SMARCA5	50.61181512	0.000446035	0.574022938
SMARCAL1	23.18087436	0.001129176	0.608416433
SMARCB1	114.1364773	0.000475406	0.582499388
SMARCD1	52.50204425	0.001394678	1.626619328
SMARCD3	12.68817415	0.003242824	1.569576836
SMARCE1P	0.302807506	0.00366803	1.556632998
SMC2	15.89282166	2.44E-05	0.508401294
SMC3	56.9318977	0.003276732	0.633521558
SMC5	21.52706288	0.001337225	0.59203543
SMCO1	0.040001529	0.000189809	1.764830046
SMCO3	2.909575781	0.000729271	1.86174687
SMCR2	0.400138939	8.25E-05	1.815068294
SMCR8	24.51415666	0.000532555	0.574653364
SMG1P7	2.008490351	3.19E-06	1.990331325
SMG6	11.20816242	6.41E-06	0.483935514
SMG7	53.90905675	0.001298054	0.605018298
SMG8	22.12371104	0.000214706	0.568956537
SMG9	12.24651458	2.23E-07	2.174258147
SMILR	0.415714142	0.000604185	1.859537223
SMIM10L1	24.27142545	0.000478592	1.697329255
SMIM12	18.09271117	0.000598843	0.585868511
SMIM23	0.344686556	1.82E-05	1.91768745
SMIM24	311.4674032	0.000114565	0.525070785
SMIM28	0.078698883	0.001553138	1.670251337
SMIM29	30.08945595	3.96E-06	1.995772806
SMIM3	131.6046372	0.003023587	1.57304169
SMKR1	2.816589183	0.00041492	1.711099203
SMN1	5.199684837	0.000654091	1.676828308
SMPD4	28.3360078	0.004046077	1.551700567
SMPD4P1	0.052329754	0.000207697	1.835909248
SMPX	0.462158818	1.42E-06	2.674056008
SMTNL1	1.580779671	0.000430392	1.734479835
SMU1	40.29922324	3.29E-06	0.487543561
SMUG1	21.67505823	5.54E-05	1.834177597
SMYD3-IT1	0.185678803	0.003203878	1.578093237
SNAP29	35.87705591	9.56E-05	0.544438396
SNAPC3	23.56896	6.72E-05	0.529861731
SNAPC5P1	0	0.004606615	1.557019068
SNCG	81.10453469	0.000665136	1.716426463
SNED1	8.325575523	0.000117252	0.525286683
SNF8	43.66695807	1.98E-06	2.063025542
SNHG10	5.128924444	5.76E-06	1.963263072
SNHG11	11.09816195	0.000314055	1.721476906
SNHG12	20.15748645	5.47E-05	1.831811333
SNHG15	24.94947487	1.05E-05	1.936240909
SNHG17	14.23018414	1.17E-11	2.69670667
SNHG19	75.73264646	0.000914357	1.657485685
SNHG21	3.03282271	9.39E-05	1.79201765
SNHG25	5.208181637	2.60E-07	2.185176358
SNHG28	2.501443787	4.69E-05	1.838980699
SNHG3	11.93176794	1.55E-08	2.29350152
SNHG4	2.121421881	6.90E-05	1.815302461
SNHG8	191.9125786	0.000970705	0.578492024
SNHG9	13.9042751	0.000747756	1.672445951
SNN	45.94339309	0.000986886	0.594965704
SNORA1	0	0.004606615	1.557019068
SNORA10	0	0.004606615	1.557019068
SNORA10B	0.160078162	0.000119026	1.933243589
SNORA11	4.317626534	0.003195088	1.579024042
SNORA11D	0	0.004606615	1.557019068
SNORA11E	0	0.004606615	1.557019068
SNORA11F	5.879108036	8.29E-05	1.801157458
SNORA11G	0	0.004606615	1.557019068
SNORA16A	0	0.004606615	1.557019068
SNORA17A	0	0.004606615	1.557019068
SNORA17B	0	0.004606615	1.557019068
SNORA18	0	0.004606615	1.557019068
SNORA21	0	0.004606615	1.557019068
SNORA21B	0	0.004606615	1.557019068
SNORA24	0	0.004606615	1.557019068
SNORA25	0	0.004606615	1.557019068
SNORA26	4.64981619	9.54E-05	1.813703813
SNORA27	0	0.004606615	1.557019068
SNORA29	0	0.004606615	1.557019068
SNORA31	9.82174366	3.07E-06	2.008966753
SNORA32	0	0.004606615	1.557019068
SNORA33	14.69707857	2.80E-11	2.641193197
SNORA3A	0	0.004606615	1.557019068
SNORA3B	2.003847654	5.32E-06	1.973548803
SNORA4	0	0.004606615	1.557019068
SNORA40	0	0.004606615	1.557019068
SNORA41	0	0.004606615	1.557019068
SNORA44	0	0.004606615	1.557019068
SNORA48	0	0.004606615	1.557019068
SNORA50B	0	0.004606615	1.557019068
SNORA50C	0	0.004606615	1.557019068
SNORA50D	0	0.004606615	1.557019068
SNORA52	0	0.004606615	1.557019068
SNORA56	0	0.004606615	1.557019068
SNORA57	0	0.004606615	1.557019068
SNORA58B	0.790212383	0.00014378	1.777471071
SNORA5B	0	0.004606615	1.557019068
SNORA5C	8.117863978	0.000299863	1.72275589
SNORA6	0	0.004606615	1.557019068
SNORA60	5.423588096	8.02E-05	1.807396046
SNORA61	0	0.004606615	1.557019068
SNORA62	0	0.004606615	1.557019068
SNORA63	0	0.004606615	1.557019068
SNORA63B	0	0.004606615	1.557019068
SNORA63E	0	0.004606615	1.557019068
SNORA64	0	0.004606615	1.557019068
SNORA65	9.218438942	0.001163727	1.654684309
SNORA66	5.983250732	9.34E-06	1.928965786
SNORA67	0	0.004606615	1.557019068
SNORA68	0	0.004606615	1.557019068
SNORA69	0.936290758	9.37E-07	2.073812581
SNORA70	0	0.004606615	1.557019068
SNORA70B	0.81238413	4.60E-07	2.121522063
SNORA70C	0.019984429	9.84E-05	3.713185555
SNORA70J	0.062524938	4.59E-06	2.775003605
SNORA71A	5.429498536	4.35E-06	2.014296023
SNORA71E	0	0.004606615	1.557019068
SNORA72	0	0.004606615	1.557019068
SNORA73A	0	0.004606615	1.557019068
SNORA75	3.623909727	4.89E-05	1.833108556
SNORA77	2.598497512	0.000916692	1.645481544
SNORA77B	0	0.004606615	1.557019068
SNORA78	0	0.004606615	1.557019068
SNORA7A	0	0.004606615	1.557019068
SNORA8	0	0.004606615	1.557019068
SNORA81	0	0.004606615	1.557019068
SNORA9	0	0.004606615	1.557019068
SNORA9B	1.774460445	6.44E-07	2.094058084
SNORC	2.912958549	1.24E-06	2.075659466
SNORD10	0	0.004606615	1.557019068
SNORD100	8.537413966	1.18E-10	2.565539115
SNORD101	5.707021262	0.000846769	1.651641572
SNORD102	0	0.004606615	1.557019068
SNORD104	34.54028271	1.32E-05	1.939380257
SNORD107	0	0.004606615	1.557019068
SNORD108	0	0.004606615	1.557019068
SNORD109A	0	0.004606615	1.557019068
SNORD110	0	0.004606615	1.557019068
SNORD113-	0.024473442	0.000297539	3.995059055
SNORD114-	0.017921917	0.000534534	4.847770351
SNORD114-	0.124362865	0.004343256	1.972345014
SNORD114-	0	0.004606615	1.557019068
SNORD115-	0	0.004606615	1.557019068
SNORD115-	0	0.004606615	1.557019068
SNORD115-	0	0.004606615	1.557019068
SNORD116-	0.127463529	0.000231347	2.058302499
SNORD116-	1.110979247	0.002803353	0.600757856
SNORD116-	0	0.004606615	1.557019068
SNORD116-	0	0.004606615	1.557019068
SNORD116-	0	0.004606615	1.557019068

SNORD118	0	0.004606615	1.557019068
SNORD12	0	0.004606615	1.557019068
SNORD123	4.525946587	0.000164314	1.767705808
SNORD124	2.439884809	3.25E-05	1.858535146
SNORD125	0	0.004606615	1.557019068
SNORD12B	4.974429613	0.000109542	1.784287166
SNORD13	0	0.004606615	1.557019068
SNORD14A	15.72720554	1.50E-05	1.905944625
SNORD14B	0	0.004606615	1.557019068
SNORD14C	0	0.004606615	1.557019068
SNORD14D	0	0.004606615	1.557019068
SNORD14E	13.79259517	0.002839298	1.63261109
SNORD15A	0.370844623	0.000119672	1.780700585
SNORD16	0	0.004606615	1.557019068
SNORD18A	0	0.004606615	1.557019068
SNORD18B	0	0.004606615	1.557019068
SNORD18C	0	0.004606615	1.557019068
SNORD19B	0	0.004606615	1.557019068
SNORD19C	5.978955659	9.11E-07	2.071239371
SNORD1A	0	0.004606615	1.557019068
SNORD1C	0	0.004606615	1.557019068
SNORD2	0	0.004606615	1.557019068
SNORD20	0	0.004606615	1.557019068
SNORD21	0	0.004606615	1.557019068
SNORD22	0	0.004606615	1.557019068
SNORD23	0	0.004606615	1.557019068
SNORD24	0	0.004606615	1.557019068
SNORD25	0	0.004606615	1.557019068
SNORD26	0	0.004606615	1.557019068
SNORD27	0	0.004606615	1.557019068
SNORD28	0	0.004606615	1.557019068
SNORD32A	0	0.004606615	1.557019068
SNORD33	0	0.004606615	1.557019068
SNORD34	0	0.004606615	1.557019068
SNORD35A	0	0.004606615	1.557019068
SNORD35B	0	0.004606615	1.557019068
SNORD36A	0	0.004606615	1.557019068
SNORD36B	0	0.004606615	1.557019068
SNORD38B	0	0.004606615	1.557019068
SNORD3B-1	0.088019595	0.001245898	1.66156799
SNORD3D	0	0.004606615	1.557019068
SNORD3E	0	0.004606615	1.557019068
SNORD42A	0	0.004606615	1.557019068
SNORD42B	0.515161578	0.000457124	1.721522628
SNORD43	0	0.004606615	1.557019068
SNORD45A	0	0.004606615	1.557019068
SNORD45B	0	0.004606615	1.557019068
SNORD45C	0	0.004606615	1.557019068
SNORD46	4.406620119	0.002085732	1.593687639
SNORD49A	0	0.004606615	1.557019068
SNORD49B	0	0.004606615	1.557019068
SNORD4A	0	0.004606615	1.557019068
SNORD4B	0	0.004606615	1.557019068
SNORD5	0	0.004606615	1.557019068
SNORD50B	0	0.004606615	1.557019068
SNORD51	1.785519631	2.98E-07	2.14053669
SNORD52	0	0.004606615	1.557019068
SNORD54	0	0.004606615	1.557019068
SNORD55	0	0.004606615	1.557019068
SNORD56	0	0.004606615	1.557019068
SNORD57	0	0.004606615	1.557019068
SNORD58A	0	0.004606615	1.557019068
SNORD58B	0	0.004606615	1.557019068
SNORD58C	0	0.004606615	1.557019068
SNORD6	12.07861626	0.000470051	1.691617391
SNORD60	4.5421528	4.45E-07	2.117852005
SNORD61	0	0.004606615	1.557019068
SNORD64	0	0.004606615	1.557019068
SNORD65	0	0.004606615	1.557019068
SNORD68	0	0.004606615	1.557019068
SNORD69	3.387111731	3.27E-05	1.85613229
SNORD7	3.036296466	1.91E-05	1.893579949
SNORD73A	0	0.004606615	1.557019068
SNORD73B	1.862913862	0.001916199	1.609241814
SNORD83B	0	0.004606615	1.557019068
SNORD84	0	0.004606615	1.557019068
SNORD86	0	0.004606615	1.557019068
SNORD87	0	0.004606615	1.557019068
SNORD88A	0.896481364	0.002518351	1.603144963
SNORD88B	0	0.004606615	1.557019068
SNORD88C	0	0.004606615	1.557019068
SNORD89	12.26124023	0.000142379	1.799239764
SNORD9	1.648284827	0.000219044	1.792200621
SNORD91A	0.62652666	0.000255254	1.740002052
SNORD93	2.493686388	1.61E-05	1.907908704
SNORD94	16.80070914	2.27E-05	2.14826066
SNORD95	0	0.004606615	1.557019068
SNORD96A	0	0.004606615	1.557019068
SNORD97	0	0.004606615	1.557019068
SNORD99	25.70831761	1.28E-09	2.453026146
SNRK	69.02205016	0.000684778	0.574635005
SNRNP200	102.3815758	0.000418491	0.570132226
SNRNP70	126.3565529	0.000117024	1.777704394
SNRPA	78.38647194	3.76E-05	1.862580151
SNRPA1	23.62346066	5.10E-06	1.980265734
SNRPD2	275.5410161	0.000175501	1.762354175
SNRPF	36.93075084	0.000121417	1.783668345
SNRPFP4	0.129906133	1.50E-05	1.959433754
SNRPG	66.74042403	0.002631907	1.577254265
SNRPGP18	0.542966878	0.001442631	1.861114073
SNRPGP20	0.098999248	0.000106497	1.969829651
SNRPGP3	0.113163985	0.0047553	1.59944031
SNTG1	0.131836564	0.002253536	1.843694659
SNTG2-AS1	5.470404076	2.60E-05	2.041822924
SNW1	92.51214337	0.000126044	0.549814305
SNX1	52.20677032	0.000104307	0.545779708
SNX12	94.0674795	9.48E-07	0.456104448
SNX13	15.52576671	0.000230715	0.541905363
SNX18	31.69758345	4.80E-07	0.439749185
SNX18P1Y	0.154468892	0.002534074	1.615511383
SNX18P7	0.126238207	1.06E-08	2.538590787
SNX2	95.29431305	1.26E-08	0.393323075
SNX21	16.47861183	0.002546411	1.580046147
SNX22	3.087642188	0.000372831	1.719488593
SNX29	37.94513331	1.10E-05	0.481584364
SNX30	21.431843	1.70E-09	0.33009218
SNX32	1.016552695	0.000608371	1.712648301
SNX33	60.26793126	0.002999143	0.629689023
SNX4	72.99880397	2.42E-07	0.439255992
SNX7	77.57022124	0.000141207	0.558815635
SNX9	76.8526491	9.32E-05	0.547629858
SOCS1	24.45099175	5.51E-05	1.859192881
SOCS2	13.64553212	4.43E-05	0.491612515
SOCS3	212.6753022	0.002053672	1.600094981
SOCS6	23.28239459	1.85E-05	0.503215533
SOCS7	13.59123527	0.000479112	0.550981319
SORBS2	25.08483578	6.16E-10	0.367557875
SOS1	32.8271159	0.00012212	0.537192113
SOS2	33.84631495	1.99E-06	0.452564984
SOWAHB	12.17248335	1.69E-11	0.320030309
SOWAHC	29.34444821	1.91E-06	0.427663139
SOWAHD	4.657442943	0.002363248	1.584974194
SOX1	0.168191402	7.55E-08	2.526835769
SOX10	0	0.004606615	1.557019068
SOX12	28.0770815	1.39E-05	1.919451639
SOX13	29.73846424	0.00019498	0.547334125
SOX2	0.254218536	0.000609792	2.063832017
SOX6	6.584701974	6.77E-07	0.406387175
SP140L	14.52434028	0.001018029	1.645915971
SP2	35.98408864	8.77E-05	0.539163634
SP4	8.525551492	0.000249518	0.559786675
SP5	2.496215968	0.000229503	1.813940045
SP9	0.035119872	5.34E-09	3.226154315
SPACA3	0.17719395	0.000388017	1.818924427
SPACA4	0.13661692	0.001378138	1.686725139
SPACA6	5.902880698	8.39E-07	2.072054781
SPACA6P-A	0.320585463	0.000335218	1.718633984
SPAG11A	0.021833441	0.003384277	1.652402
SPAG16	7.621651396	0.000117411	0.539778307
SPAG17	3.934629323	7.11E-05	1.81368064
SPAG9	58.35270213	0.000368261	0.557972806
SPAM1	0.020327944	8.79E-06	2.003480902
SPANXA1	0.001175949	0.000358962	5.055029694
SPANXB1	1.458348633	1.88E-07	3.387651554
SPANXC	0.091446162	5.74E-08	3.044177807
SPANXD	0.021413364	0.000380704	2.87063673
SPANXN1	0.016914904	0.000206822	1.97603094
SPART	69.30097925	3.05E-05	0.521835919
SPART-AS1	2.449497091	1.01E-05	1.942396284
SPATA1	0.693908135	1.44E-07	2.177558094

SPATA18	44.28947164	2.41E-09	0.368383866
SPATA20	71.1728958	0.003624139	1.556960208
SPATA21	0.165857576	1.27E-06	2.09212485
SPATA3-AS	0.049778534	0.003627879	1.566077563
SPATA31A5	0	0.004606615	1.557019068
SPATA31C2	0.017499236	0.002504912	1.591096237
SPATA31E3	0.000444741	5.96E-05	3.60375558
SPATA41	0.545529076	0.002831861	1.5726209
SPATA42	0.11653302	0.000657528	1.693412168
SPATA6	12.35204145	0.000358332	0.573280911
SPATA7	7.870567891	4.86E-05	0.522503088
SPATA9	0.563189212	0.0001448	1.767398435
SPATC1L	8.960378811	0.000971185	1.645311605
SPC24	5.838218475	7.75E-11	2.592678432
SPC25	5.135941623	0.004287755	1.543168883
SPDEF	1.423497836	5.99E-07	2.21494748
SPDYA	0.983279535	2.64E-08	2.264453973
SPDYE1	0.5180968	0.001605323	1.611004996
SPDYE20P	0.142853045	0.001092569	1.64926735
SPDYE22P	0.114505557	4.82E-05	1.983234031
SPDYE5	0.787878003	0.000256354	1.732222752
SPDYE8P	0	0.004606615	1.557019068
SPEG	0.923971748	1.71E-06	2.09808586
SPEN	29.11482948	0.001750314	0.601091159
SPG11	30.49327281	0.001472211	0.60418099
SPHAR	0	0.004606615	1.557019068
SPHK1	8.104929625	1.68E-06	2.066558227
SPIN1	108.5814995	9.03E-08	0.430214261
SPIN4-AS1	0.356684271	0.001034845	1.645940254
SPIRE1	39.03774461	0.004013748	0.631143312
SPNS1	3.532290156	0.002362464	1.582494482
SPNS3	6.849213331	0.003288173	1.563641788
SPOCD1	0.736333945	1.68E-10	2.602643919
SPON2	62.33309271	0.000106232	1.80764652
SPOPL	26.02731256	1.51E-06	0.465977949
SPPL2B	30.00517879	0.000898488	1.648399392
SPRED2	43.2980584	6.66E-05	0.529609485
SPRED3	1.884978859	7.81E-05	1.822318586
SPRN	4.243398686	3.84E-05	1.855238856
SPRR2B	0.033233087	0.000239089	2.528980838
SPRR2D	0.074327692	1.90E-05	2.138680674
SPRR2E	0.104245722	0.002097029	1.797849757
SPRR3	0.174283117	0.00462435	1.665720586
SPRY2	43.81785955	0.002017595	0.60203185
SPRYD3	81.93964776	8.54E-05	0.540759758
SPRYD7	25.00809678	0.000355482	0.574858805
SPSB2	16.07857578	0.001854601	1.602127811
SPTAN1	87.99498252	2.44E-05	0.502944419
SPTBN1	159.7364123	1.91E-09	0.354557335
SPTBN5	1.999103407	0.004202379	1.565528066
SPTLC1	63.91487254	9.28E-05	0.548350605
SPTLC1P4	0.125472255	0.001605584	1.629804262
SPTLC2	50.39668063	1.72E-05	0.493627094
SPTLC3	23.28367375	0.000885747	0.577878747
SPTY2D1	25.96680658	0.000288754	0.565123722
SPTY2D1OS	1.595102229	5.00E-05	1.836744353
SRA1	42.25221208	0.003898755	1.549177716
SRBD1	16.98951297	8.47E-06	0.496329539
SRC	37.73691058	1.77E-05	1.922761062
SRCIN1	1.237719482	1.68E-05	1.915987346
SRD5A2	0.177090036	0.001887926	1.690537413
SRD5A3-AS	1.019877547	4.75E-07	2.130241759
SRFBP1	17.47758072	1.88E-06	0.464274198
SRGAP2B	2.076308751	1.10E-05	0.489244866
SRGAP2D	2.924553565	2.19E-05	0.497358617
SRM	90.88056529	7.20E-08	2.236019901
SRMS	0.731329264	0.001558494	1.629485843
SRP14P4	0	0.004606615	1.557019068
SRP19	18.24683482	2.54E-05	1.899480994
SRP54	55.65052744	0.001131697	0.593682364
SRP72	91.240848	0.000547636	0.587481427
SRP9P1	2.181395243	0.002840098	0.594615212
SRPK3	0.75593038	3.31E-06	2.012145664
SRPX	15.97643238	0.00242365	1.640450432
SRPX2	14.47152583	4.32E-07	2.213500377
SRR	7.405408445	0.001896198	0.60331
SRRM1P2	0.019702065	0.000210663	1.89747222
SRRM1P3	0.129792471	0.002111765	1.594356554
SRRM2-AS1	0.782914633	0.000100109	1.790805522
SRRM5	1.20782619	4.66E-05	1.838908092
SRSF10P1	0.129287569	0.004603252	1.568748131
SRSF2	136.8807629	0.002232095	1.586238303
SRSF8	68.14510456	6.40E-05	0.540681366
SS18L2	26.67109437	0.003799291	1.55167561
SSBP4	51.47003974	6.78E-07	2.118767938
SSC4D	2.607127194	0.00150395	1.650391532
SSMEM1	0.064202816	0.003811083	1.558494745
SSPO	0.45668652	4.13E-06	1.988665905
SSTR1	5.416813108	0.001119	0.577484471
SSX1	2.339190028	0.000254839	3.720788875
SSX6P	0.048446689	0.002020417	1.674104419
SSX8P	0.273837708	0.000831039	2.027226141
ST13	357.8492671	1.95E-08	0.409926497
ST13P3	0.683793514	0.000994273	0.593235397
ST13P4	1.039436785	0.000472273	0.568722721
ST13P5	2.839486446	0.0011114	0.599812014
ST13P6	1.550602272	8.76E-06	0.473324742
ST20	8.297626894	0.001006984	1.645371116
ST3GAL3	15.30579141	0.004428971	1.550440621
ST3GAL5	13.08234314	0.000203075	1.752632888
ST6GALNA	7.016516278	0.000562372	0.568238687
ST6GALNA	2.168205386	0.000339114	1.717224432
ST7	25.66945101	0.001058787	0.588881653
ST8SIA4	35.8139683	0.000209526	0.526366465
STAC3	9.116162705	0.000140802	1.778126105
STAG1	20.98649239	0.001270288	0.605316066
STAG2	62.94578381	4.83E-06	0.48293417
STAG3L1	0.724657926	0.003198869	1.564499959
STAG3L5P-	4.284558199	0.001190253	1.633844602
STAM	33.36048372	7.43E-05	0.539339945
STAM-AS1	0.535900414	4.40E-06	1.981569655
STAM2	26.48916928	8.52E-07	0.454522142
STAR	0.189825207	1.32E-05	1.960110579
STARD13	22.23362565	0.001179085	0.593388524
STARD3NL	54.83159126	0.002885206	0.626131732
STARD8	36.53099329	2.94E-05	0.500444362
STAT2	80.37984419	2.69E-08	2.286076375
STAT5B	73.10568746	1.64E-05	0.511714593
STAU2-AS1	0.213521259	0.002776708	1.580703012
STBD1	5.758266047	0.001405014	0.596675721
STEAP3	41.68240423	4.35E-09	2.396470358
STEAP3-AS	0.512489546	8.10E-09	2.427544226
STIL	2.592274538	3.37E-05	1.86542407
STK19B	7.483401608	0.00103524	1.66974773
STK24	23.22396592	0.000250887	0.569590825
STK31	0.222058038	0.000452415	1.809412107
STK32B	33.82081282	4.30E-07	0.445617185
STK32C	9.882198493	1.18E-05	1.929136238
STK33P1	0.045205913	0.000236761	1.820563691
STK36	22.79881111	0.004250587	1.540760367
STMN1P1	0.512806365	4.29E-05	1.846542371
STMND1	0.05078002	0.003846808	1.596757303
STMP1	40.76099257	0.000386801	1.710908312
STN1	55.69364557	0.000416867	0.57700587
STOM	599.2954225	0.000569466	0.588301003
STON1	12.76335551	0.000106137	0.534523729
STPG1	4.910621922	0.000129185	0.550689523
STPG2	0.342732129	0.00384633	1.559207301
STPG4	0.118437175	0.003097515	1.588589098
STRA6	0.633338626	1.05E-05	2.041742878
STRADA	5.525462192	0.000263431	1.724459294
STRBP	9.134502875	2.95E-05	0.484633381
STRC	0.079838267	6.11E-06	1.981390234
STRCP1	0.179058024	3.54E-08	2.27665742
STRIP2	4.212015831	0.001985012	1.687635369
STRN	20.3096518	7.35E-07	0.443159396
STRN3	29.46288181	0.000663483	0.581309748
STS	27.33666991	0.000910586	0.570901744
STX10	73.12548949	0.000278548	1.735165909
STX12	82.97928586	3.77E-05	0.527419094
STX16	61.95487172	4.08E-08	2.263067785
STX16-NPE	1.397480031	0.000153705	1.761703401
STX17	15.35928692	1.18E-05	0.493773999
STX17-AS1	4.295699146	0.003129577	0.623825337
STX1A	3.185613012	3.00E-08	2.285241812
STX3	66.05875476	0.000155071	0.54245332
STX4	63.15247586	4.36E-05	1.864501329
STX7	27.95920765	0.002446659	0.619595452
STX8P1	0.202289229	0.001556834	1.621062498
STXBP1	36.47739698	0.002227133	0.597954449

STXBP3	37.700436	0.002983542	0.628366711
STYK1	0.631123297	4.74E-08	2.261308242
STYXL1	44.31611728	0.004604941	1.537196297
SUB1P4	0.237462591	0.004517219	1.565116035
SUCLA2	56.92177336	3.27E-10	0.357926402
SUCLG1	83.06858254	0.00374232	0.58154935
SUCLG2-AS	1.451230397	0.000460995	0.517498297
SUCLG2P2	0.721215535	0.001204339	0.587960512
SUFU	25.00079739	0.003392808	0.633287094
SUGCT	17.82611739	9.46E-07	0.427491268
SUGT1	16.36744591	7.18E-05	0.543528169
SUGT1P3	1.336468494	0.000217946	0.534369326
SULF2	114.028781	0.00035295	1.714613028
SULT1A1	11.24368585	0.001306483	1.630059628
SULT1A3	0.126152525	0.000151742	1.76613618
SULT1B1	1.489621776	1.66E-05	0.412005538
SULT1C4	59.28827659	1.85E-05	0.472796395
SULT2B1	1.280930635	0.000182874	1.808824071
SUMO1P1	0.289713216	0.003016363	1.585495384
SUMO1P3	2.369995309	0.004257634	0.625132585
SUMO2P18	0.135120147	0.003860486	1.609877681
SUMO2P19	0.725494013	0.001012291	1.642612146
SUMO2P7	0.22326015	0.003791581	1.57204403
SUMO4	0.569834111	7.94E-07	2.095148779
SUN3	0.180638899	0.000783783	1.680435542
SUPT16H	55.25405429	0.002436911	0.620421733
SUPT4H1P1	0.236596885	0.001296563	1.634802065
SUPT6H	55.50801792	0.000972286	0.596511631
SUZ12	24.86809586	0.000467444	0.572647964
SUZ12P1	6.902615448	3.56E-05	1.852169551
SV2C	0.236628337	0.000151208	0.455700396
SWAP70	60.38642603	1.57E-06	0.45940637
SWT1	11.03086143	3.04E-07	0.439379736
SYCE1L	7.108797988	1.93E-08	2.30087882
SYCE2	1.987039239	3.63E-08	2.255902206
SYCE3	3.898000164	0.002800559	1.626785381
SYCP1	0.016641525	4.76E-05	1.901550097
SYCP2L	0.58244939	0.002071502	1.6072408
SYDE2	3.546068143	3.97E-05	0.508168483
SYF2	74.7936897	7.06E-05	0.533468604
SYN3	0.563513777	0.000126344	0.534807793
SYNE1	12.53108488	0.000329573	0.553764641
SYNE2	27.84035889	9.78E-06	0.481423984
SYNGR4	0.479624456	0.000761323	1.70013935
SYNJ2-IT1	0.138207871	9.44E-05	1.857854284
SYNJ2BP	30.15803705	1.73E-06	0.415867763
SYNM	25.99900377	0.00157388	0.604973724
SYNPO2	25.10477267	0.000411833	0.521961326
SYNRG	18.36809819	0.001285461	0.600549369
SYP-AS1	0.038821296	0.001420541	1.731440884
SYPL1	240.1757987	2.83E-06	0.479614815
SYPL1P1	0.016148547	0.003199538	1.804695981
SYPL1P2	0.975672944	0.000119533	1.781867123
SYT11	33.17078679	0.003094116	0.617788437
SYT3	0.542697368	2.55E-05	2.055004338
SYT5	0.2300524	0.001208807	1.722071857
SYT9	8.296518457	0.000868469	0.569797491
SYTL1	4.412645062	0.000757869	1.682248096
SZT2-AS1	0.32385998	0.000792811	1.666376478
TAAR1	0.221774982	0.004640959	0.610399425
TAAR7P	0.010301165	0.000767188	3.158530657
TAB3	12.87765345	0.001618213	0.597298506
TAB3-AS1	0	0.004606615	1.557019068
TAB3-AS2	0	0.004606615	1.557019068
TAC3	0.210806058	3.42E-09	2.452624937
TACC1	65.69534648	2.47E-06	0.44314098
TACC2	10.07337802	0.000476446	0.550574833
TACC3	12.22194572	3.27E-08	2.275226407
TACR1	1.328767866	0.001497843	0.570946551
TADA2B	29.53203971	5.28E-05	0.527924361
TAF10	31.28316282	2.88E-10	2.549410298
TAF1B	16.12080507	5.24E-06	0.475394307
TAF2	19.69264566	0.000686803	0.590636161
TAF3	14.5539854	0.000573796	0.581249047
TAGLN2	830.8932544	0.000224349	1.756608335
TAGLN3	1.129584067	0.004541305	1.781933783
TAL2	10.59415765	3.60E-07	0.426503208
TANC1	15.32320783	0.004407001	0.637322904
TANGO6	12.07915023	3.21E-05	0.523576433
TAOK3	21.435172	0.000594905	0.584266838
TAPT1	20.34515591	0.004460885	0.640545677
TARBP1	15.43577579	0.0003057	1.716675558
TARBP2	17.71922124	2.06E-10	2.565958435
TAS2R14	1.920450335	0.002571496	1.577772996
TAS2R15P	1.367812093	0.003991352	1.550486044
TAS2R19	0.855146777	2.84E-05	1.875551028
TAS2R20	1.565007289	8.32E-07	2.077120264
TAS2R42	0.071256311	0.002657509	1.586687681
TAS2R6P	1.09711846	0.003038451	1.564735062
TATDN1	14.54201124	0.001370722	0.603398909
TAX1BP1	69.00444325	0.004698324	0.635632073
TAX1BP3	81.82964249	0.000117428	1.786189321
TAZ	23.14791863	6.08E-05	1.820248115
TBC1D1	56.57328173	1.37E-05	0.362909698
TBC1D12	11.82546893	0.001779855	0.607179918
TBC1D13	53.92710991	0.000778001	0.591865937
TBC1D14	28.20997285	0.000498472	0.467214169
TBC1D15	32.09557848	4.23E-06	0.479092834
TBC1D19	13.00297793	1.24E-07	0.411462638
TBC1D21	0.053308448	0.001315833	1.650870075
TBC1D27P	0.149380736	0.000287355	1.771051397
TBC1D31	2.780936385	0.004261275	1.541028817
TBC1D3L	1.049496484	0.000113689	1.796116292
TBC1D3P1	0.03037714	0.000211972	1.790939955
TBC1D3P5	0.016621648	2.61E-05	1.899890077
TBC1D5	25.36156575	7.38E-05	0.530056516
TBC1D7	6.577284662	0.000379351	1.712289348
TBC1D8B	12.08479476	6.71E-05	0.502333101
TBCCD1	15.61759516	0.003912406	0.638194518
TBL1X	57.38221421	1.60E-06	0.461558363
TBR1	0.017049003	0.000865422	1.666026951
TBRG4	41.36154285	0.002736897	1.574735612
TBX10	0.220646647	3.22E-08	2.268020747
TBX15	7.053698799	0.001148481	1.658707534
TBX18-AS1	0.106121671	0.000255871	1.758486894
TBX19	6.313851991	0.000538148	1.681908763
TBXA2R	15.08010777	0.000656363	0.573617419
TCAF1	15.25451101	3.79E-05	0.509293215
TCAIM	14.02882394	3.59E-05	0.502634295
TCEAL1	46.95733936	5.33E-06	0.48433596
TCEAL8	196.5018512	8.32E-07	0.455322714
TCERG1	23.03350447	0.00013761	1.771104366
TCF19	35.60390725	0.002566717	1.588411477
TCF20	26.93477513	0.000738515	0.586518254
TCF3	30.38262752	0.002263735	1.589049841
TCF4	24.03062641	0.001253195	0.590363825
TCF4-AS2	0.202059701	0.004790108	1.62071212
TCF7L1-IT1	0.161677863	0.003484003	1.592590058
TCF7L2	28.21033083	0.000136697	0.542612528
TCHHL1	0.007540696	0.001239303	1.814827415
TCIRG1	56.21088886	6.58E-09	2.361220956
TCL6	1.039798537	0.000551752	0.492321061
TCN1	2.881366029	0.003128351	1.938925728
TCN2	295.2560121	0.001290578	0.597021607
TCP11L1	14.47167176	0.000406091	0.569337426
TCP11L2	7.34684771	0.000474392	0.572195221
TCTA	95.31880908	0.000400293	0.56511433
TCTE3	3.352337062	1.78E-06	2.034347671
TCTEX1D2	8.455299954	0.004460894	1.54973571
TCTN3	94.01900939	0.000258653	0.568389459
TDGF1P5	0.327238592	0.000565291	1.690456996
TDO2	2.634882854	0.001403904	1.671842937
TDP2	82.56090946	0.000291556	0.54582871
TDRD7	25.19720278	4.41E-08	0.429092131
TDRG1	0.05194189	4.93E-05	1.936525804
TEAD1	40.92237005	9.82E-05	0.53856874
TEAD3	25.85421343	0.002043167	1.592900942
TEAD4	35.97376214	4.38E-05	1.852787695
TECPR2	13.30098965	0.000334096	0.566124379
TEDC2	1.814593496	7.12E-08	2.233393691
TEF	47.14570182	5.54E-05	0.518504211
TEK	37.30003391	1.35E-06	0.440079013
TEKT4	0.184101033	0.003589587	1.574806714
TEKT5	0.171081276	8.46E-08	2.215720864
TELO2	29.03376067	5.68E-05	1.837267442
TEN1-CDK3	1.953647985	0.003082526	1.5609857
TENT4B	11.75864535	0.003932915	0.636643242
TEPSIN	9.216034726	2.00E-06	2.028813556
TERB1	0.095223887	0.003063461	1.572399517
TERF2	25.68684438	0.002012843	0.625186212

TESK2	5.950944814	0.000272386	0.557689158
TET2	8.370519261	0.001906295	0.609293789
TEX101	0.277857523	0.002182723	1.760940893
TEX14	0.455147144	9.27E-05	1.794855476
TEX19	0.206169579	0.000195558	1.970423077
TEX28P1	0	0.004606615	1.557019068
TEX28P2	0	0.004606615	1.557019068
TEX29	1.502430208	3.87E-06	2.017209044
TEX44	0.034686535	0.003903465	1.598410982
TEX49	0.090015388	0.002163179	1.601128229
TEX53	0.256843872	0.000271036	1.729385209
TEX9	6.174797682	0.000686821	0.586044132
TF	18.17181903	0.002646493	1.934889817
TFAM	28.57525574	5.55E-05	0.533670271
TFAMP1	0.061427958	0.00235858	1.598695678
TFAP2A	2.639463543	0.000815395	1.742537455
TFAP2A-AS	0.885522226	3.68E-08	2.278561146
TFAP2D	0.057935543	0.001150816	1.758292447
TFAP2E	1.243423026	1.65E-06	2.047443135
TFDP1	58.65282945	0.000152967	0.556653766
TFEC	25.83398286	1.83E-05	0.501607083
TFF1	2.147100755	5.96E-06	2.120841185
TFPI2	101.5392192	0.000959379	1.747141375
TFPT	37.23126112	0.00190628	1.599945381
TGDS	20.71311565	0.000427436	0.579388155
TGFA	141.4508248	0.004049095	0.626177029
TGFB2-OT1	0	0.004606615	1.557019068
TGFBR2	280.8609449	4.27E-05	0.511247579
TGFBR3	23.03925719	9.07E-05	0.526131801
TGM3	0.378053943	2.72E-05	1.896538836
TGM4	0.062774814	4.08E-07	2.140205735
TGOLN2	285.4074976	0.002271039	0.624272958
TGS1	21.36189651	0.003022269	0.62890428
TH	0.051461748	0.001954977	1.734624675
THAP1	18.39014417	0.002609004	0.628517746
THAP10	6.507490502	9.05E-06	0.503450198
THAP12P1	0.051057107	0.004817137	1.536646316
THAP12P7	0.394728974	0.001377033	0.591500572
THAP5	27.37714465	6.20E-07	0.443108543
THAP9	6.395013626	3.83E-05	0.494391187
THBS3	28.88695857	1.47E-05	1.908397638
THEG	0.12145726	0.004364977	1.621422994
THEGL	0.050486681	9.02E-06	2.035045621
THEM6	52.84121986	0.000295778	0.569883111
THNSL1	17.73541694	3.76E-05	0.521927333
THOC6	61.46550487	0.001000298	1.645271529
THRA	46.78261222	6.44E-05	0.530239676
THRB	6.860938603	7.82E-06	0.443117291
THRIL	0	0.004606615	1.557019068
THSD7A	12.79702281	0.000437826	0.491045235
THSD7B	3.073598327	4.09E-05	0.484571017
THTPA	11.37583611	2.45E-06	0.470938955
THUMPD1	53.20800413	2.82E-05	0.519411033
THUMPD1F	0.036557578	0.002836601	1.610938666
THUMPD3-	5.126320721	0.001308275	1.61798744
TIAF1	2.530945451	0.002495336	1.576246745
TICAM1	58.30485036	0.00154192	0.609072427
TICRR	1.04957388	1.38E-11	2.683449603
TIGD1	8.692345315	0.000302984	1.721084305
TIGD6	8.027935564	0.001163	0.60020846
TIMELESS	13.73121592	0.002117659	1.591623298
TIMM10B	42.14093386	0.000305193	0.546334652
TIMM21	16.26778069	0.000430792	0.579524894
TIMM8AP1	2.509106176	0.002042354	1.746621956
TIMP1	1225.392744	3.93E-07	2.130926128
TIMP3	17.7972771	1.40E-08	0.368805956
TINAG	64.83581728	8.93E-05	0.526718138
TINCR	0.441128036	5.99E-11	2.611861333
TINF2	74.71816861	0.001769559	0.618677011
TIRAP	9.936454201	0.002702719	0.62353962
TJP1	43.12564067	0.000461242	0.568110263
TJP2	47.67413933	1.88E-08	0.395606299
TK1	51.25180077	1.93E-06	2.038821831
TLCD1	10.99621745	9.16E-08	2.217673691
TLCD2	7.29807523	0.000148327	1.773666249
TLE1P1	2.892379762	0.003863289	0.621584518
TLE6	1.681644828	0.002173623	1.605927294
TLK2P1	0.332717328	0.00173926	0.606354975
TLL1	10.78313873	0.000290884	0.538470811
TLL2	0.369176507	0.000475454	1.740841356
TLN1	236.6501012	3.80E-06	0.478042748
TLN2	24.13756035	1.28E-07	0.414164919
TLR3	68.46711072	4.35E-06	0.48466755
TLR4	34.09418062	0.000698059	0.57831848
TLX1	0.055296313	0.003918483	1.635753518
TLX2	0.042710519	2.01E-06	2.107296848
TM4SF18	103.5809336	0.002421548	0.620529363
TM4SF19	1.224280562	7.12E-05	1.965227491
TM4SF19-A	1.256952196	0.000364386	1.715177264
TM4SF19-T	0.126887514	0.000301915	1.734576762
TM6SF1	8.737262441	0.003876881	0.62656145
TM7SF3	153.3254851	6.32E-07	0.449061988
TM9SF2	234.8006551	4.55E-05	0.533335629
TMA7	147.9058701	0.00015866	1.768977804
TMBIM1	206.4725602	2.53E-07	0.449553423
TMC2	0.089343886	0.002537937	1.591562873
TMC3	0.211981766	2.14E-05	2.223536517
TMC8	16.19731764	0.004992934	1.533488791
TMCC3	39.54304666	0.000282273	0.548312518
TMED10	267.151041	0.000456791	0.578406561
TMED10P1	0	0.004606615	1.557019068
TMED10P2	1.250711031	0.001007367	0.567141436
TMED3	14.47851844	0.001542519	1.616955776
TMED5	60.48876902	0.003068168	0.625210639
TMED7	173.4344456	0.000109853	0.541976541
TMED8	28.92639321	2.43E-05	0.499094871
TMEFF1	0.073875383	6.51E-05	1.954561725
TMEM106B	31.28619871	0.000229443	0.546031277
TMEM109	268.3726725	3.36E-05	0.52911642
TMEM110-I	0.315014147	0.000219313	1.737484398
TMEM114	0.07879029	0.003088304	1.771132009
TMEM125	21.3170331	1.40E-06	0.447475763
TMEM127	103.4433943	4.56E-05	0.532474507
TMEM131L	15.61140551	0.001239565	0.598784435
TMEM132A	21.31299353	1.81E-06	2.04354679
TMEM135	37.44166015	7.85E-07	0.45059854
TMEM138	13.41312686	0.000486907	1.697141913
TMEM140	226.0597019	0.001927338	0.619547864
TMEM145	3.670433424	0.000145773	1.979370108
TMEM147-/	4.665123205	3.11E-06	1.997088286
TMEM14A	112.5938718	0.002434171	0.621008149
TMEM14EP	0.473064052	0.004642402	1.716489511
TMEM150C	48.09008948	2.75E-07	0.432369446
TMEM151B	0.09122449	0.000262592	1.754232322
TMEM158	5.671461638	0.00012439	1.931380036
TMEM165	39.07577011	5.87E-07	2.112088525
TMEM167B	54.7329123	0.002730282	0.628863817
TMEM170A	22.89092283	1.98E-06	0.466393581
TMEM171	37.75274421	0.000151979	0.534348978
TMEM174	41.4923069	1.17E-05	0.411975496
TMEM175	22.12593659	1.57E-05	1.905615868
TMEM176B	1188.443444	0.003164501	0.635601746
TMEM18	29.82670072	0.003411274	0.629610057
TMEM183A	0.068685964	0.001722294	1.709296676
TMEM184A	5.841095977	0.00121977	1.634148649
TMEM184C	35.64867079	0.000276538	0.559171207
TMEM186	21.09484886	0.001718514	0.61342261
TMEM189	16.78639478	1.97E-11	2.697641859
TMEM190	0.130708945	7.02E-05	1.839803363
TMEM192	19.06492606	2.49E-07	0.422468048
TMEM198B	17.44294893	0.000727563	1.658057781
TMEM200A	36.79430862	0.000235953	0.558987601
TMEM204	150.2058912	0.002917988	0.621385579
TMEM212-I	0.007983859	0.002479786	2.581335135
TMEM214	108.0805738	0.000242652	1.751446154
TMEM219	203.3549792	0.003091317	1.565903438
TMEM220	20.85506519	3.88E-05	0.512966699
TMEM220-/	3.087805247	0.000585602	0.561795367
TMEM225	0.071232426	0.00068841	1.741983072
TMEM232	1.594090542	1.93E-05	0.495899501
TMEM233	10.89940837	0.003306731	0.618338617
TMEM234	17.57734201	0.001438303	1.617598461
TMEM239	0.016704211	3.54E-08	2.315232483
TMEM241	5.071506705	0.00105187	0.595459276
TMEM242	17.3346355	0.001625359	0.617010208
TMEM245	56.84405977	1.79E-07	0.419132564
TMEM246	24.41663093	9.88E-07	0.457647725
TMEM249	0.171311093	1.66E-05	1.928196185
TMEM25	13.96880929	2.24E-08	0.387151075
TMEM252	50.04736622	5.89E-06	0.388970067

TMEM262	1.278742553	4.57E-05	1.833821091
TMEM269	0.290502099	0.00074903	1.662000249
TMEM271	0.127752345	1.42E-07	2.27694009
TMEM35B	32.07881329	0.002435874	0.629298374
TMEM37	343.6549824	0.000222202	0.551835623
TMEM38B	48.83685701	1.54E-06	0.450421438
TMEM40	0.111684042	0.001719427	1.747184071
TMEM42	14.27563222	0.002844318	1.571826326
TMEM44	22.03885477	2.81E-08	2.289026914
TMEM45A	33.50108173	0.000463872	1.719812741
TMEM47	106.5206337	5.12E-08	0.387936535
TMEM59	150.9777372	0.000271892	0.56700011
TMEM63C	0.622720586	7.08E-05	1.940515149
TMEM72-A	0.554633557	0.003106501	1.564520495
TMEM74	0.159379793	0.000906296	1.750069687
TMEM74B	12.66709851	6.85E-05	1.824706419
TMEM75	0.322117095	0.001670404	1.671313588
TMEM79	8.095186496	0.000677644	1.67809324
TMEM81	8.065877992	6.21E-06	1.974032428
TMEM82	12.86750133	7.57E-05	0.497243416
TMEM86B	2.96843322	3.37E-11	2.64431625
TMEM8B	17.00440265	2.16E-06	0.429396411
TMEM92-A	2.026788389	1.82E-13	2.901471026
TMIGD1	1.661796982	0.00492179	0.624667426
TMLHE	13.32355072	0.000109856	0.543478381
TMOD2	12.86791293	2.73E-05	0.467919275
TMOD3	36.96292304	1.49E-05	0.502649182
TMPO-AS1	2.437204521	3.13E-07	2.140598068
TMPOP1	0.090633502	0.002152526	1.631002876
TMPPE	2.032416154	0.00049837	0.553677143
TMPRSS11	0.074550761	3.30E-08	2.325124877
TMPRSS13	0.72582774	0.003834536	1.594182944
TMPRSS6	1.179072966	0.001274436	1.76375877
TMPRSS9	0.60179014	0.000269203	1.726982552
TMSB10	10701.87631	0.00125576	1.628072092
TMSB4XP6	0	0.004606615	1.557019068
TMTC1	19.98024363	0.000148484	0.536070639
TMTC2	17.29313339	7.48E-06	0.483697612
TMTC3	30.83531945	0.00031729	0.564043222
TMUB1	64.06050552	0.000359031	1.714737565
TMX1	61.70830859	0.004339988	0.641606399
TMX2P1	14.74457911	0.00021563	0.556598866
TNFAIP2	87.44683478	0.002854909	1.569860049
TNFRSF10A	19.58632857	5.67E-08	0.417501183
TNFRSF10A	9.630596925	0.000444029	0.567722155
TNFRSF12A	212.0207648	0.002275547	1.58991194
TNFRSF13C	1.306134859	7.73E-05	1.850419482
TNFRSF18	4.803732069	0.000133735	1.780416773
TNFRSF19	29.61942263	1.68E-05	0.494603048
TNFRSF21	156.5858461	2.38E-06	0.446922467
TNFRSF25	10.53582101	0.00119856	1.626002837
TNFRSF6B	0	0.004606615	1.557019068
TNFSF13	42.44135075	0.001718991	0.595864954
TNFSF13B	23.77264322	0.000400374	1.706099709
TNFSF14	6.724892041	1.47E-09	2.483497597
TNIP1	325.0371326	0.004797754	0.643808267
TNIP2	51.11940515	3.52E-05	1.872799264
TNIP3	1.820714445	0.002947663	1.608833645
TNK1	12.8596478	0.00195259	0.621454376
TNKS2	49.38610773	6.23E-05	0.533627619
TNN	1.624990281	0.003422436	0.568005787
TNNT1	4.82530571	5.80E-09	2.736658212
TNRC6C	7.320089657	0.001654302	0.602616233
TNS1	259.160055	5.12E-05	0.519978819
TNS3	105.1244872	2.08E-05	0.506615831
TNXA	2.248060409	3.01E-05	1.951645613
TNXB	4.530093458	0.00321078	0.58551384
TOB2	90.14320162	6.01E-08	0.386590935
TOGARAM	9.600642269	3.89E-05	0.511331167
TOGARAM	0.176586467	0.000266483	1.737909212
TOLLIP	65.00876568	8.10E-08	0.422332085
TOM1L1	28.01188235	5.01E-07	0.450760924
TOM1L2	35.45140769	0.001158734	0.594133502
TOMM20	364.0731312	3.54E-08	0.408501614
TOMM20L	1.238578949	0.000474549	0.566046135
TOMM6	0	0.004606615	1.557019068
TONSL	4.326813751	0.000223125	1.745415195
TONSL-AS1	1.362216636	0.002305047	1.586774942
TOP2A	16.26899239	1.84E-07	2.179848744
TOPORS	26.47049471	1.56E-08	0.396171331
TOR1AIP1	43.07787698	2.50E-06	0.471719658
TOR1B	42.83684834	0.004310454	0.645409227
TOX3	6.257027936	9.31E-07	0.356284075
TOX4	42.49353226	0.000102453	0.538058751
TP53I13	35.52076227	5.59E-06	1.979751246
TP53INP2	116.2390466	1.16E-05	0.488200493
TP53TG3B	0	0.004606615	1.557019068
TP53TG3C	0	0.004606615	1.557019068
TP53TG3E	0	0.004606615	1.557019068
TP53TG3F	0	0.004606615	1.557019068
TPBGL	2.481849596	6.86E-05	1.891034493
TPD52L2	107.3490394	1.50E-06	2.056296236
TPH2	0.02061896	1.52E-06	2.073938953
TPM3P2	0.081946953	0.000543347	1.74541963
TPM3P8	0.236441236	0.000278057	0.547028557
TPMT	87.94697366	1.43E-05	0.506787688
TPMTP4	0.023556066	0.000190971	2.059048863
TPR	42.78007465	0.000504845	0.57913369
TPRA1	34.71677821	0.001662709	1.610750653
TPRG1L	137.5486361	6.02E-13	0.314631365
TPSB2	23.13424028	0.0046421	0.619048598
TPSD1	2.756290692	0.002797822	0.601339335
TPSG1	12.52848932	0.000109597	1.933254327
TPST2	21.59093984	3.19E-05	1.874444258
TPT1P5	3.716287906	0.000799931	1.699946573
TPTE	0.015056587	0.001443761	1.674604228
TPTE2	0.229378251	3.41E-06	2.03926302
TPTEP2	2.848498483	0.000981046	1.639933511
TPX2	18.33257987	3.55E-10	2.522089997
TRA2A	71.35565907	0.003114603	1.560540226
TRABD	40.74820251	2.90E-09	2.415206474
TRAF3IP1	20.27574765	0.000852652	0.586111378
TRAF5	9.931561769	0.002662773	1.573973366
TRAF6	16.41990233	2.94E-07	0.435111643
TRAF6P1	0.124966074	5.69E-05	0.463545769
TRAF7	48.95004624	0.001234974	1.630314889
TRAIP	2.429382661	1.63E-05	1.911078844
TRAJ31	1.04241897	0.001692604	1.62737467
TRAJ32	0	0.004606615	1.557019068
TRAK2	43.48540608	5.74E-06	0.463784718
TRAM1L1	10.67944421	0.001431878	0.590707806
TRAM2-AS1	17.53755162	0.000442808	0.575381094
TRAPPC1	258.4252443	0.003278229	1.560946434
TRAPPC12	19.70203731	0.001661536	1.607436881
TRAPPC12-	4.439484048	0.002229564	1.584182278
TRAPPC13P	0.078421445	0.002994029	1.609691926
TRAPPC2P1	0	0.004606615	1.557019068
TRAPPC2P5	0	0.004606615	1.557019068
TRAPPC2P9	0	0.004606615	1.557019068
TRAPPC3L	0.395233401	0.003754598	0.626674148
TRAPPC5	0.967262173	6.85E-05	1.832695787
TRAPPC8	21.47582897	5.39E-06	0.481228005
TRAV38-2D	0	0.004606615	1.557019068
TRBJ2-5	0	0.004606615	1.557019068
TRBJ2-6	0	0.004606615	1.557019068
TRDD3	0	0.004606615	1.557019068
TRDJ1	0	0.004606615	1.557019068
TREM1	4.344399922	0.001683166	1.713094414
TREML3P	0.361009112	1.34E-09	2.504302186
TREX1	0	0.004606615	1.557019068
TRHDE	7.985818104	2.53E-06	0.474088055
TRIB3	76.50648824	2.50E-06	2.026331559
TRIM10	6.030260878	0.00349835	0.629170762
TRIM11	8.796128626	0.003222954	1.562486338
TRIM13	20.73875201	5.89E-05	0.528689279
TRIM15	20.55161793	0.003450546	0.628890019
TRIM2	30.95370699	4.84E-05	0.386063897
TRIM21	72.79535347	0.000774679	0.595158733
TRIM23	19.95556182	0.000108332	0.539169725
TRIM26	61.33140214	0.00210237	0.619088832
TRIM32	19.84045681	0.000104248	0.541325979
TRIM36	1.62247435	6.72E-05	1.822886541
TRIM36-IT1	0.110499107	5.33E-06	2.063902111
TRIM4	42.8208912	1.02E-05	0.50065007
TRIM44	44.46503632	0.001579291	0.610204637
TRIM46	2.128397895	0.000198048	1.799020519
TRIM60P10	0	0.004606615	1.557019068
TRIM60P11	0	0.004606615	1.557019068
TRIM60P18	8.240601344	0.000729702	1.662396768
TRIM60P5Y	0	0.004606615	1.557019068

TRIM60P9Y	0	0.004606615	1.557019068
TRIM65	23.52366703	0.000265502	1.734376874
TRIM66	6.825749903	0.001679161	1.603411094
TRIM72	0.341549688	9.25E-05	2.924494407
TRIM73	0.213153943	0.000110365	1.787712264
TRIM74	0.416396009	0.001954065	1.605142265
TRIML2	0.214929984	0.00063912	1.97625105
TRIP11	18.36567213	1.21E-06	0.443108758
TRIP12	62.69641963	0.000532161	0.583434195
TRIP13	6.676873182	1.21E-07	2.198321312
TRIP4	38.94467013	1.54E-06	0.47325455
TRIQK	31.03361948	0.00266974	0.622327172
TRMO	11.49080094	4.27E-07	0.440202551
TRMT1	38.43426254	0.000230299	1.739189519
TRMT112P1	0.023665595	0.002964292	1.922026807
TRMT12	18.14610531	1.15E-05	0.49678269
TRMT1L	30.97612764	2.71E-07	0.436388799
TRMT2B	20.9277443	0.002695447	0.617678599
TRMT5	12.77493643	9.86E-06	0.489174579
TRMT61B	20.76514083	1.99E-08	0.402135884
TRMU	11.56163886	1.37E-06	2.048099166
TRNP1	10.69622742	2.92E-05	1.956088714
TROAP	3.57931625	3.60E-14	2.99820164
TRPA1	5.001039918	0.000957921	0.572365001
TRPC7-AS1	2.146599737	0.001901212	0.575200261
TRPM3	4.310518593	9.63E-06	0.47720222
TRPM7	18.67146956	0.000362398	0.555806012
TRPM8	0.583903406	0.002854964	1.77998093
TRPV3	0.416031406	1.08E-11	2.700326507
TRRAP	25.74782408	0.001510181	0.599910346
TRUB2	25.54778061	0.000387223	0.576556231
TSACC	0.690673572	5.15E-08	2.246003435
TSC22D1	207.0008003	3.42E-06	0.478595899
TSEN15	30.27916417	0.003421508	1.558249037
TSEN54	24.69845567	0.003886041	1.547032309
TSG101	72.1346278	0.000127034	0.554475356
TSHZ1	28.95956982	9.87E-06	0.485590154
TSKU	47.82997518	0.00011083	1.831719044
TSN	74.90291221	0.000944177	0.600016199
TSNAX	23.73106023	0.000593435	0.583182232
TSPAN12	226.5146126	0.001452348	0.606370591
TSPAN14	39.55471099	0.004723636	0.638544268
TSPAN16	0.207932321	0.002651743	1.586303741
TSPAN17	34.12253651	0.00385608	1.550298833
TSPAN18	92.746856	2.73E-06	0.476403993
TSPAN32	2.717307906	0.000886722	1.652035869
TSPAN7	67.38357531	0.000215071	0.524415748
TSPEAR-AS	0.547656597	7.51E-05	1.886211167
TSPO	204.0254731	0.000910093	1.650092674
TSPY18P	0	0.004606615	1.557019068
TSPY21P	0	0.004606615	1.557019068
TSPY23P	0	0.004606615	1.557019068
TSPY7P	0	0.004606615	1.557019068
TSPYL1	160.2875165	1.88E-08	0.3928777
TSPYL2	40.48798105	0.001789367	1.608703526
TSPYL4	34.27649962	0.000496563	0.566793185
TSR2	138.5207915	0.000436525	0.565273507
TSSC2	2.108512335	8.08E-05	1.809359375
TSSK2	0	0.004606615	1.557019068
TSTA3	43.44477897	4.50E-08	2.283325278
TSTD2	7.202422518	0.003638285	0.632363304
TTC1	147.1346473	1.08E-05	0.497725437
TTC12	12.05917977	0.001754377	0.599999024
TTC19	34.59597451	0.002750391	0.626817898
TTC24	0.670179268	0.003031298	1.600580502
TTC27	27.98311626	0.000150342	0.555424649
TTC28	24.70703358	1.04E-06	0.451971622
TTC33	28.74690049	8.45E-07	0.455019433
TTC37	54.3715516	1.14E-06	0.456780808
TTC39B	11.02495153	0.001030206	0.567753855
TTC39C	5.78113681	0.001270588	1.626931827
TTC4	3.268260522	0.003979099	0.638907992
TTC5	7.940674065	3.56E-05	0.517853845
TTC6	0.039965124	1.91E-05	2.004756498
TTC7B	6.568752331	0.000127151	0.527078813
TTC9	6.751161419	6.60E-05	0.477764304
TTC9B	0.358652516	6.86E-05	1.81672694
TTF1	18.04130338	0.002387027	0.62174395
TTI2	8.928134116	6.26E-05	0.542500124
TTK	2.507144139	1.49E-06	2.063986905
TTLL11	4.392603708	0.000131403	0.545825243
TTLL13P	0.225956076	7.61E-06	1.949703707
TTLL3	7.907849352	2.90E-05	1.863808704
TTLL4	9.333795263	0.00035004	1.710815828
TTLL5	7.612090489	0.001578798	0.602508616
TTLL9	0.409995178	1.72E-05	1.899596224
TTTY17A	0	0.004606615	1.557019068
TTTY21	0.000374766	0	73.43182481
TTTY21B	0	0.004606615	1.557019068
TTTY22	0.00060443	1.53E-08	18.09498863
TTTY23B	0	0.004606615	1.557019068
TTTY3	0	0.004606615	1.557019068
TTTY3B	0	0.004606615	1.557019068
TTTY8B	0	0.004606615	1.557019068
TTYH1	0.673479801	3.06E-12	2.763072277
TUB	13.90700629	0.00237075	0.601728518
TUBA1C	72.72594504	9.99E-05	1.797294171
TUBA8	0.384639044	0.001803919	1.603480091
TUBB2B	4.508553716	0.00492702	1.674833638
TUBB3	1.048310137	9.01E-06	2.016063943
TUBB3P1	0.138861165	0.00065616	1.798173893
TUBB6	73.14307189	0.00395048	1.548566873
TUBB8	0.116968339	3.57E-05	1.882402085
TUBB8P2	0.246922489	0.004804482	1.54074453
TUBBP11	0.118720951	0.000323075	1.871987062
TUBBP6	0.492427634	2.72E-05	2.223192483
TUBGCP3	16.03659066	0.004975852	0.647910339
TUBGCP6	29.23687584	0.000303329	1.717171467
TULP1	0.045683601	0.002421219	1.586268646
TUNAR	1.905784861	0.003323091	0.461318352
TUSC1	54.8978837	0.002171447	0.625221821
TVP23C	1.63971159	0.002663307	1.572205072
TWF2	84.00983271	0.000296756	1.7445103
TWIST2	1.567425407	0.000514195	1.765891341
TXLNA	66.9874731	0.001104403	1.641589271
TXNDC15	70.44291435	1.92E-05	0.512729442
TXNDC16	15.55446147	6.36E-05	0.524026438
TXNDC17	32.88580887	0.000932894	1.65064112
TXNIP	1251.421456	3.62E-05	0.513553153
TXNL1	27.38191928	0.003398705	0.637043327
TYMP	138.609168	0.000155282	1.770607718
TYW3	22.43519149	0.000118913	0.545276515
U2AF1	0.461100935	0.001098939	1.636848765
U2AF1L4	12.36660366	0.000252703	1.727318553
U47924.2	4.953301313	5.11E-05	1.840036574
U47924.3	1.872407609	6.70E-10	2.463205153
U52111.1	0.720485523	8.90E-08	2.199420614
U52112.1	0.045225629	0.00285446	1.610180015
U62317.1	4.468185233	9.86E-10	2.465623751
U62317.2	29.09536498	4.61E-08	2.238613905
U62317.4	1.438499474	1.11E-06	2.095345858
U73166.1	0.857496347	7.26E-05	1.814532541
U82695.1	0.220202424	0.000384728	1.756418625
U85056.1	0	0.004606615	1.557019068
U91319.1	1.884834095	0.000390402	0.554379556
U91328.1	6.090422378	1.28E-06	0.450917782
U91328.3	1.836863304	0.002298118	1.591800734
UBAP1	96.5766632	0.000955429	0.600916519
UBAP1L	3.135646346	0.000559566	1.675740488
UBAP2	13.35183651	0.001551873	0.609766607
UBB	1189.656922	0.002606412	0.627173439
UBE2B	98.27168183	0.000449232	0.579127462
UBE2C	24.91308963	3.44E-15	3.200290554
UBE2CP2	1.449789	1.65E-05	1.896487774
UBE2D3	118.6567934	5.32E-06	0.488520932
UBE2D3P1	2.012693992	7.61E-06	0.478003333
UBE2L4	0.77591176	0.003895556	1.553005152
UBE2Q1-AS	1.81715155	3.97E-05	1.847600876
UBE2Q2P4Y	0	0.004606615	1.557019068
UBE2Q2P5Y	0	0.004606615	1.557019068
UBE2Q2P8	0	0.004606615	1.557019068
UBE2QL1	7.798836272	0.001572014	0.479536432
UBE2R2	89.76764026	3.56E-06	0.492712605
UBE2R2-AS	0.483646329	4.07E-07	2.1252007
UBE2S	10.81565865	4.06E-08	2.264936001
UBE2SP1	1.216697418	2.94E-05	1.873434666
UBE2T	13.2376695	2.97E-07	2.14582285
UBE2V1P1	0.498660007	0.000927834	1.650928964
UBE2V1P7	0.008479713	0.001077438	2.906369861
UBE2V2P1	0.100404358	0.00155264	1.670658745

UBE3C	38.01567419	0.003830368	0.63595434
UBE4A	32.12914875	0.001526083	0.607441885
UBE4B	24.18517057	1.76E-05	0.495456871
UBIAD1	18.00660872	0.000269943	0.566188329
UBL4B	0.157401756	0.001242085	1.643327758
UBL7-AS1	3.799566447	0.001572479	0.611705827
UBN1	25.41276129	0.003624039	0.633280227
UBOX5-AS1	0.669979529	0.000845835	1.647482052
UBQLN1	70.10439486	0.000224279	0.562155429
UBQLN2	58.82476244	0.000793941	0.595277414
UBQLNL	1.206459294	4.35E-06	1.985409258
UBR1	16.44757263	8.93E-05	0.538074049
UBR2	31.26632037	0.000329544	0.567014492
UBR3	23.99677363	1.61E-05	0.48970205
UBR5-AS1	7.901865111	7.04E-06	1.958964075
UBR7	61.43377951	0.000462363	0.577169936
UBTD1	32.57977202	0.002721258	1.5754899
UBTD2	40.01402423	0.004420331	0.640330059
UBTFL11	0.020141267	0.001605117	1.657498133
UBTFL6	2.542314093	4.67E-05	0.487449389
UBTFL8	0.038900847	0.000161347	1.820246511
UBXN10	14.10787555	0.001437217	0.594067132
UBXN2B	34.6685584	2.71E-05	0.511411657
UCHL1	35.1528918	4.69E-06	2.035719308
UCHL5	19.74552889	0.0001625	0.550794081
UCK1	66.97626514	0.003874568	0.639616227
UCK2	13.53735422	0.000422402	1.700839028
UCN	5.649583542	2.10E-07	2.161889357
UCN2	0.398260497	1.52E-13	3.250010185
UEVLD	20.42163178	0.0005024	0.58122705
UFL1	53.41077683	0.001818053	0.614940771
UFM1	99.35681321	5.87E-05	0.532372507
UFSP2	54.74177976	2.10E-07	0.441969784
UGDH-AS1	4.17637176	0.00125974	0.59206535
UGT1A10	1.90634257	1.03E-07	2.489466474
UGT1A11P	0.063714102	0.000726647	0.565167972
UGT1A12P	0.048527811	0.001975485	1.704589106
UGT1A13P	0.07217884	0.003196458	1.614503481
UGT2A3	214.6823028	4.51E-05	0.497379128
UGT2B7	442.858514	2.34E-05	0.471319514
UGT3A1	21.58359654	0.000669927	0.546256661
UHRF1	4.108518804	1.05E-06	2.079824155
UHRF1BP1l	20.23078499	0.00262017	0.622758126
ULBP1	0.851110146	8.23E-06	1.988586177
ULK1	36.83225776	0.004092614	1.550931539
ULK3	52.60596653	0.003962658	1.545703849
UMAD1	29.7317699	0.000575856	0.577100732
UNC119	14.21524944	6.69E-08	2.230153693
UNC119B	68.04026825	2.96E-06	0.462875574
UNC13B	42.75613532	9.14E-06	0.474119
UNC13D	8.601408164	3.22E-07	2.144290743
UNC45A	47.42785508	0.001493698	0.612784917
UNC5B	77.74440419	0.004759937	0.625547903
UPB1	20.59003345	6.69E-05	0.521592461
UPF1	49.02549618	3.99E-05	0.522276263
UPF2	26.24417279	0.001695546	0.609290856
UPK1A	0.773691531	0.000214336	2.181234438
UPK1A-AS1	0.172253194	4.42E-05	1.894498979
UPK2	1.011179873	0.00046379	1.737952441
UPK3B	1.598201212	7.54E-11	2.645107199
UPP1	36.27645221	0.004997965	1.586691277
UPRT	21.17852925	0.00048881	0.57961445
UQCC2	14.5415396	0.000300009	1.725375332
UQCRC2	148.9094686	0.003018798	0.616265386
URB1-AS1	16.3058412	0.004258597	1.542677634
URI1	48.63324592	7.45E-06	0.500222793
UROC1	0.192509534	1.58E-06	2.061877041
USB1	18.06143079	9.64E-06	1.945755707
USF1	78.74208927	2.39E-05	1.88477717
USF2	93.25852424	0.003254879	1.563508947
USF3	8.80298216	1.27E-05	0.474325797
USH1C	132.8873053	9.10E-05	0.548478592
USH2A	0.136193281	0.000571014	1.715550619
USO1	111.9739096	0.000271894	0.569897375
USP10	62.912578	2.09E-05	0.522054257
USP12	43.59739285	6.12E-05	0.530546964
USP12PX	0.034376099	0.002362329	0.589020642
USP17L11	0.000125935	0	261.7554331
USP17L12	0	0.004606615	1.557019068
USP17L13	0	0.004606615	1.557019068
USP17L17	0	0.004606615	1.557019068
USP17L18	7.48E-05	5.10E-06	22.83636517
USP17L19	0	0.004606615	1.557019068
USP17L20	0	0.004606615	1.557019068
USP17L21	0	0.004606615	1.557019068
USP17L22	0	0.004606615	1.557019068
USP17L24	0	0.004606615	1.557019068
USP17L25	0	0.004606615	1.557019068
USP17L26	0	0.004606615	1.557019068
USP17L27	0	0.004606615	1.557019068
USP17L28	0	0.004606615	1.557019068
USP17L29	0	0.004606615	1.557019068
USP17L30	0	0.004606615	1.557019068
USP17L5	0	0.004606615	1.557019068
USP17L9P	0	0.004606615	1.557019068
USP19	35.28812996	0.00110886	0.591524424
USP21	24.20907675	0.00374732	1.549867734
USP24	27.86533778	0.000214686	0.558910227
USP26	0.009858904	0.002051262	1.645395339
USP27X	11.81096547	9.87E-06	0.494132129
USP27X-AS	4.479754391	2.01E-06	0.466424756
USP29	0.018249643	0.00016731	1.816855541
USP30	18.34243208	0.001689926	0.61141003
USP32P1	0.737863969	0.004990802	1.569761401
USP32P3	0.849434007	0.000332518	1.720488018
USP33	41.22725795	0.001226269	0.605279453
USP34	16.58672045	0.000434233	0.566604385
USP35	6.309933985	0.000466366	1.688473522
USP37	5.042819174	7.51E-05	0.53040073
USP38	21.42297818	7.95E-05	0.534610138
USP46	7.493546921	0.004158133	0.633456128
USP51	8.748283357	7.63E-06	0.479497714
USP53	12.12933709	0.000191315	0.516136334
USP8	12.46179688	1.05E-05	0.49355572
USP9YP11	0	0.004606615	1.557019068
USP9YP12	0	0.004606615	1.557019068
USP9YP13	0	0.004606615	1.557019068
USP9YP16	0	0.004606615	1.557019068
USP9YP17	0	0.004606615	1.557019068
USP9YP18	0	0.004606615	1.557019068
USP9YP19	0	0.004606615	1.557019068
USP9YP21	0	0.004606615	1.557019068
USP9YP23	0	0.004606615	1.557019068
USP9YP24	0	0.004606615	1.557019068
USP9YP26	0	0.004606615	1.557019068
USP9YP27	0	0.004606615	1.557019068
USP9YP30	0	0.004606615	1.557019068
USP9YP33	0	0.004606615	1.557019068
USP9YP35	0	0.004606615	1.557019068
USP9YP36	0	0.004606615	1.557019068
USP9YP7	0	0.004606615	1.557019068
USP9YP9	0	0.004606615	1.557019068
UTP14C	26.86249215	8.02E-07	0.45694141
UTP18	42.459158	0.003159252	0.631709739
UTP20	14.23234684	0.001057232	0.57890108
UTP23	14.61258876	0.002638967	0.624640371
UTP25	12.55170797	0.000412042	0.576589194
UTP3	62.80346424	0.000645465	0.574888589
UTRN	53.27739048	3.43E-06	0.450847977
UVRAG	20.53938583	0.001662775	0.614249545
UXS1	31.13288529	0.002582322	0.613714805
VAC14-AS1	0.420681475	2.70E-10	2.55914847
VAMP1	10.43348603	4.96E-06	1.972824951
VAMP3	196.5018328	1.06E-08	0.407834693
VANGL1	17.63308886	2.67E-05	0.51135678
VAPB	31.55939964	7.85E-05	0.52973084
VAV2	42.07086185	0.002327272	0.612146266
VAV3	44.59772642	1.88E-06	0.466667717
VAX1	0.023016593	0.002994298	1.704321376
VCP	213.7291943	2.22E-05	0.514720131
VCPIP1	17.00245474	0.003931364	0.635032013
VCX	0.129090788	0.003934619	1.56019163
VCX3A	0.075438396	0.004172099	1.612039832
VDAC1P1	0.924641592	0.000478497	0.561186122
VDAC1P9	0.057408306	1.19E-05	1.979869947
VENTX	2.88309329	0.003469118	1.565096738
VENTXP7	0.159974716	0.000152888	1.881833327
VEZF1	62.33770452	0.000390048	0.57106731
VGF	0.98769854	0.002832009	1.806150363
VIL1	16.62810048	7.74E-05	0.488528993

VIPAS39	28.41922275	0.000670313	0.578940209
VIRMA	25.12683124	1.34E-05	0.503746623
VKORC1	122.7455134	2.70E-05	1.881783923
VMAC	6.953606058	0.002496298	1.574707088
VMO1	15.27235684	1.08E-06	2.071774761
VMP1	136.5802586	4.61E-05	1.842122008
VN1R110P	0.072965632	0.000342213	1.73275794
VN1R51P	0.376584704	0.000511477	1.711009387
VN1R76P	0.063781076	0.000841924	1.748346605
VN1R7P	0.123338421	0.002987312	1.646535831
VN1R82P	0	0.004606615	1.557019068
VN1R8P	0.05278982	0.000113158	1.855964491
VN1R96P	0.00960433	1.50E-08	3.026599869
VN1R9P	0	0.004606615	1.557019068
VN2R1P	0.01944506	0.00141091	1.710557843
VNN3	0.443645362	3.66E-05	1.937225426
VPS11	59.4280227	9.69E-06	0.507178079
VPS13A	16.18233735	0.000588461	0.569416924
VPS13B	12.38245986	0.000146126	0.533827968
VPS13C	21.33127238	0.000183393	0.540583563
VPS13D	14.59279856	9.66E-06	0.458910341
VPS26A	74.42671226	3.38E-05	0.518212668
VPS26C	31.41697074	3.22E-06	0.475699531
VPS33B-DT	0.265035469	5.34E-05	1.83507704
VPS35	64.15341475	4.80E-05	0.534518747
VPS35L	27.83811928	5.83E-06	0.496222337
VPS37C	31.03095071	0.004445246	0.6407764
VPS37D	22.41893147	4.26E-05	0.503056543
VPS41	38.48677517	0.000807888	0.584751975
VPS4B	57.81305288	0.000172433	0.557249533
VPS50	11.02416499	0.000895063	0.588921734
VPS51	69.02446858	0.000645074	0.583198883
VPS9D1-AS	3.209493092	1.07E-06	2.062082523
VRK3	17.53694341	0.001061895	0.600713692
VSIG10L	3.211532545	1.58E-05	1.910995409
VSIG4	76.17142406	0.003136684	1.56677419
VSIG8	0.64792167	0.000621069	1.680855982
VSTM4	21.82492178	7.62E-05	0.516699297
VSX1	0.612041814	0.000138849	1.837122518
VSX2	0.025676445	0.002872231	1.607778322
VTA1	22.47508662	0.002454018	0.624365017
VTA1P1	0.131208439	0.000197042	1.79574018
VTI1A	8.117289495	0.001145301	0.59778821
VTI1B	63.45247754	2.87E-05	0.516149251
VTI1BP2	0.186038551	0.003329958	1.567591843
VTRNA1-2	0.637470318	0.000203956	1.767118231
VTRNA2-2I	0.50470835	9.55E-05	1.873197152
VWA3A	0.554400206	4.42E-05	1.996246233
VWA3B	0.070160229	3.42E-05	1.869695647
VWA5B2	0.295197181	1.46E-06	2.071580271
VWA8	17.76513135	7.41E-08	0.400446236
VWDE	0.558956185	0.003453735	1.630883591
VWFP1	3.667724689	0.000596661	0.527824269
WAC	55.3304192	8.33E-05	0.536702069
WAPL	39.211361	0.000558443	0.58055226
WARS2	23.61307158	2.28E-05	0.503332032
WASF2	160.6620709	0.000214708	0.553580439
WASF3-AS1	0.066152043	0.000362663	1.778223498
WASF4P	0.154993805	0.001995794	0.594736143
WASH4P	2.48252739	0.001064487	1.64244141
WASH7P	3.819225136	0.000343704	1.714155323
WASH9P	5.577323151	6.19E-07	2.103122209
WASHC1	11.93626345	3.77E-06	1.993451577
WASHC4	47.31271804	0.001273555	0.59779201
WASHC5	49.04586874	0.003269768	0.632720034
WASIR2	0.330707665	0.002128068	1.626960573
WASL	99.37697053	3.77E-05	0.514923646
WBP1L	87.29366536	0.000877238	0.589349554
WBP1LP8	0.000467576	0.000520006	14.02082721
WBP4	27.81271617	0.000574536	0.581314385
WDFY3	29.46882643	1.07E-07	0.404822304
WDFY3-AS2	9.797624492	0.000292517	0.561765873
WDR11	25.17672804	0.001896487	0.611174407
WDR20	11.39033922	0.000173601	0.551635043
WDR25	8.907514226	0.003537694	0.637602933
WDR27	6.015469723	0.000821941	1.65140232
WDR3	13.47897815	0.000600235	0.586380188
WDR31	6.612724635	2.24E-07	0.407975927
WDR35	17.9964823	0.003862602	0.63106324
WDR44	19.47489597	0.00189109	0.610008759
WDR45	44.22829853	0.001562004	1.614597088
WDR47	19.94246069	0.000500418	0.582856963
WDR48	21.48418924	0.000289249	0.548838038
WDR49	0.098301371	2.29E-05	1.911191866
WDR61	23.37415216	0.000815144	0.592388419
WDR62	1.313168337	5.55E-08	2.239949532
WDR7	7.469575362	1.05E-06	0.45381575
WDR72	31.14217659	2.59E-09	0.341536358
WDR87	0.016881205	0.003768144	1.578175133
WDR92	1.43722088	0.002189446	1.58949755
WDR97	0.834977698	3.64E-05	1.851781972
WDSUB1	30.878174	8.77E-06	0.475358073
WFDC10B	0.956128772	3.69E-11	2.718783168
WFDC11	0.053016174	3.31E-07	2.220833556
WFDC13	0.185963179	2.66E-05	1.939364799
WFDC3	1.603070974	5.09E-09	2.422720334
WFDC5	4.296765265	1.52E-05	2.406509318
WFDC6	0.074649447	0.003413272	1.645516401
WFDC8	0.057594513	0.00083266	1.73259367
WHRN	9.235682382	0.000163589	1.7642941
WIPF2	42.73739019	0.000168754	0.556751386
WIPF3	7.386704327	0.004959006	1.534719593
WLS	69.19594844	0.00013661	0.539393324
WNT10B	0.68441593	2.20E-06	2.052907447
WNT16	0.151678303	6.34E-10	2.487490671
WNT3	3.337585323	5.75E-06	1.97135514
WNT5A	4.49798036	0.000536517	1.706066837
WNT7B	1.000607987	6.77E-05	2.226433293
WNT9B	0.653873593	0.001704482	0.553360659
WSB1	72.51234552	0.001393106	1.613878163
WT1	4.22878401	0.000175568	1.787544973
WT1-AS	0.271366876	1.61E-09	2.508535816
WWC2	25.24314526	3.50E-05	0.511273919
WWOX	14.01785346	0.000462771	0.558916806
WWP1	52.50004596	0.000600277	0.564271003
WWP2	36.82813509	9.94E-05	0.525910104
WWTR1	129.362552	0.000615223	0.579389139
XAF1	10.56548	0.002793283	1.572758025
XBP1	0	0.004606615	1.557019068
XCL1	4.235114178	0.002107865	1.599716042
XIAP	32.45642625	0.000106403	0.536198178
XKRYP4	0	0.004606615	1.557019068
XKRYP5	0	0.004606615	1.557019068
XPA	27.5124375	0.002154991	0.619931843
XPC	39.94504729	2.88E-06	0.47011977
XPOT	46.50516883	0.00369275	1.553507097
XPR1	34.40761798	0.000192893	0.557883259
XRCC2	1.837463923	4.81E-06	1.983257053
XRCC3	4.310967789	8.52E-05	1.79719794
XRCC5	204.5714391	0.001631312	0.61131901
XRCC6	309.3620258	0.000865624	0.603859485
XRCC6P1	0.10358199	0.00451951	1.550325549
XRCC6P4	0.055481441	0.000307742	1.766506674
XRN2	109.7985333	0.000554421	0.589670233
XYLB	6.178742447	1.22E-05	0.471335735
YARS	33.17430099	0.003675952	1.554095765
YBX2P1	0.199861374	0.003360094	1.578291093
YBX3	150.9322927	0.00029644	1.73207254
YDJC	43.57606139	1.14E-05	1.93872604
YEATS2-AS	1.923061123	6.08E-07	2.10067832
YEATS4	27.45303562	0.004569409	0.639827557
YES1	59.5681138	0.000265532	0.558237598
YES1P1	1.052015287	3.72E-07	2.128787953
YIF1B	26.20729789	2.34E-05	1.890282835
YIPF6	36.17666868	2.03E-05	0.509544759
YJEFN3	5.246832065	0.000497583	1.688012646
YKT6	89.50911761	0.003689017	1.554545984
YLPM1	19.63528037	9.56E-06	0.483150432
YME1L1	85.07687586	0.001178051	0.608990819
YPEL5	135.643964	8.78E-06	0.482067704
YTHDC1	52.38512466	0.002988393	0.627410428
YTHDF1P1	0.050003359	0.000347861	1.743047219
YTHDF3	62.16841484	0.001101592	0.597627771
YWHAE	578.0805714	0.000699446	0.597191854
YWHAEP2	0	0.004606615	1.557019068
YWHAEP5	0.800176474	0.003194773	0.615825751
YWHAZP4	4.132834389	0.001219006	0.596730396
Z69720.1	0.262311418	0.004999449	1.523428858
Z73965.1	0.830765457	1.88E-06	2.033810662
Z82188.2	0.507716246	0.000239857	1.741505343

Z82198.2	0	0.004606615	1.557019068
Z82206.1	0.474377898	4.33E-05	1.849194139
Z83836.1	0.035246557	0.000318321	1.746028151
Z83844.2	0.168741632	1.52E-05	1.909205169
Z84485.1	2.043014694	4.22E-08	2.245608539
Z84488.1	0.120510128	1.89E-05	1.898822422
Z84492.1	4.592068915	0.003494852	0.624476242
Z84492.2	0.030159968	6.19E-05	2.054731349
Z85994.1	0.069681545	8.05E-05	1.848251129
Z92544.3	0	0.004606615	1.557019068
Z93241.1	1.013211092	4.03E-06	2.052310711
Z94160.1	0.086517762	0.000348178	1.807259301
Z94160.2	0	0.004606615	1.557019068
Z95114.2	0.076801971	5.26E-05	1.883022954
Z95115.1	6.090568868	0.000883378	1.659964087
Z95331.1	0.144900406	2.78E-06	2.005768083
Z97192.3	0.333178297	0.00010646	1.817882639
Z97200.1	0.530470189	1.76E-09	2.446325013
Z97205.2	0.08668585	0.001549292	1.714176249
Z97353.2	0.434906717	0.000368218	1.70826714
Z97832.2	1.580637127	0.00031135	1.716772334
Z97986.1	0	0.004606615	1.557019068
Z98200.1	0.897563625	3.83E-05	1.850590685
Z98257.1	0.945870433	1.26E-06	2.497266345
Z98752.1	0	0.004606615	1.557019068
Z98949.1	0.010971111	0.00101964	1.70730116
Z98949.3	0.058971025	5.23E-05	1.945956415
Z99127.3	0.191918678	0.002269338	1.658719033
Z99289.1	1.261926269	0.00018178	1.760375651
Z99289.2	0.455363304	1.68E-10	2.561912301
Z99755.1	0	0.004606615	1.557019068
Z99916.3	0.072185785	4.04E-06	2.042306299
ZACN	0.39081258	2.60E-05	1.880355721
ZAN	0.138589738	0.000273348	1.844795678
ZAP70	7.848595158	0.001672657	1.609391044
ZBED6CL	24.73570841	9.06E-05	1.80576485
ZBED9	3.694151949	0.00171322	0.606069457
ZBP1	2.094983509	7.19E-05	1.827494822
ZBTB10	20.1271293	0.001057537	0.577105775
ZBTB16	10.94275902	0.002999295	0.57725494
ZBTB20	2.146559754	0.000770633	0.54728458
ZBTB20-AS	0.41522897	0.001000456	1.677529427
ZBTB20-AS	0.523110978	0.001521273	0.576394647
ZBTB21	14.44783601	0.001934183	0.593897167
ZBTB33	23.4463854	0.000358044	0.567995019
ZBTB34	9.592521463	0.002542248	0.62057668
ZBTB4	90.99166679	2.93E-06	0.468369788
ZBTB41	16.33923202	0.002372276	0.616504591
ZBTB44	35.66723145	0.000185946	0.551845144
ZBTB5	19.33983965	0.000870364	0.597211996
ZBTB6	13.1339419	0.000101208	0.534817288
ZBTB7C	4.566552505	0.004895649	1.610791687
ZBTB8OS	18.69324329	0.000111717	1.799123119
ZC2HC1C	5.731567745	0.001158419	0.594736013
ZC3H11B	0.081220978	0.003516196	0.617043307
ZC3H12C	9.476146893	4.47E-07	0.42741526
ZC3H13	30.99035869	2.80E-06	0.469557517
ZC3H14	7.873039304	0.002362279	0.617414076
ZC3H3	28.10636665	4.09E-07	2.152442471
ZC3H7B	54.47167276	0.00079659	0.583128175
ZCCHC10	26.40212455	0.000636065	0.582725509
ZCCHC12	0.384691141	0.002403438	1.601168693
ZCCHC14	30.56402461	5.37E-06	0.467649434
ZCCHC18	0.773584208	0.000948555	1.642404597
ZCRB1	78.23929652	0.002147515	1.593240666
ZCWPW1	7.114357461	9.49E-05	1.797333512
ZDHHC16	36.90888369	0.003330713	1.562315248
ZDHHC19	0.377129441	0.000195967	1.768033221
ZDHHC20P	0.276896332	0.001538884	0.59896305
ZDHHC21	10.53674329	0.002970872	0.617925732
ZDHHC6	49.34716482	0.000435329	0.578715717
ZEB1	31.30244908	0.001651661	0.59959182
ZER1	61.99561748	0.000172697	0.555229761
ZFAND1	33.5913258	9.91E-05	0.532968591
ZFAND2A	31.28749453	0.000889199	1.655589891
ZFAND6P1	0	0.004606615	1.557019068
ZFAT	10.66983366	1.42E-05	0.465340342
ZFHX2-AS1	1.038408353	3.15E-05	1.860957057
ZFHX3	11.16373878	3.99E-05	0.502022205
ZFHX4	1.147230647	4.26E-06	2.069905054
ZFHX4-AS1	0.216516553	0.000309322	2.014498267
ZFP1	14.66039388	0.000594189	0.584263813
ZFP2	6.281438791	0.004155825	0.637868738
ZFP3	10.0709574	0.00025892	0.562802234
ZFP30	5.676363665	0.000124003	0.53258256
ZFP36L2	198.2953283	0.000120578	0.533524822
ZFP64	9.788337062	0.003215595	0.633043288
ZFP64P1	0.016172692	1.60E-05	2.198397154
ZFP91	64.59002716	0.002108156	0.623497558
ZFPM1	2.986942855	0.000851306	1.655142627
ZFPM2-AS1	6.969154855	6.51E-07	2.114564143
ZFR	65.71524659	1.30E-05	0.512849718
ZFYVE1	25.8788408	0.000186658	0.531555111
ZFYVE26	11.1836066	0.001139642	0.593784697
ZFYVE9	22.37749506	1.90E-07	0.429653448
ZGLP1	1.516884679	5.34E-08	2.236161696
ZGRF1	1.622802616	0.000620656	1.671411937
ZHX1	26.15041072	0.000673651	0.583998189
ZHX1-C8orf	1.587639095	3.69E-08	2.265889705
ZHX3	25.37701752	0.001361847	0.594287041
ZIC1	0.112429819	0.004419142	1.712823716
ZIC2	0.475466671	3.75E-14	3.425835738
ZIC4	0.060763828	0.004003578	1.686081472
ZIC4-AS1	0	0.004606615	1.557019068
ZIC5	0.180024914	1.47E-14	3.477697964
ZIK1	4.980708999	1.26E-06	0.441258516
ZKSCAN2-I	3.612908448	1.62E-06	2.035844649
ZKSCAN4	10.33839694	0.000582186	0.584015877
ZMAT2	226.4798149	0.000238062	0.562147115
ZMIZ1-AS1	1.197841302	1.53E-06	2.083059901
ZMIZ2	69.97192936	0.001692912	1.606587481
ZMPSTE24	96.01748397	0.000241255	0.556930148
ZMYND11	71.91091222	0.001049293	0.600206797
ZMYND12	7.1632618	0.000358495	0.541416049
ZMYND8	29.75146787	0.003907372	1.560648077
ZNF101P1	0.039902528	0.000777862	1.684049711
ZNF101P2	0.112656881	0.000346979	1.723065059
ZNF106	30.33676139	1.81E-05	0.49190008
ZNF112	5.650329029	2.84E-06	0.464201932
ZNF114	5.683390846	0.004856959	1.661016741
ZNF114-AS	0.631598257	3.89E-05	1.960667555
ZNF117	7.526944226	2.05E-06	2.024614696
ZNF134	17.32921413	0.000288043	0.562859336
ZNF136	10.22221482	1.62E-06	0.463287704
ZNF14	14.06046566	1.21E-08	0.402841583
ZNF143	16.35457488	0.003227113	0.63645171
ZNF148	26.77089624	2.69E-05	0.511712886
ZNF155	4.076852521	0.001797546	0.603821108
ZNF17	6.623080906	0.002129473	0.617899731
ZNF18	13.33158171	6.31E-06	1.972295169
ZNF180	7.588086393	0.003606297	0.626462959
ZNF181	8.740551252	0.003338502	0.633386217
ZNF184	15.49039397	6.60E-06	0.47314376
ZNF189	72.92510363	1.66E-06	0.395242593
ZNF2	6.590179449	0.000133653	0.548831211
ZNF202	10.41480085	0.001741462	1.602215907
ZNF214	5.419495603	0.000713057	0.57880026
ZNF222	5.998836874	0.000611232	0.58032377
ZNF23	0.869357456	2.78E-05	1.870665598
ZNF230	4.385051607	0.001791523	0.608438718
ZNF24	46.231672	0.000166982	0.550858328
ZNF251	14.43690435	0.00059569	1.674858188
ZNF252P	11.80815491	8.74E-05	0.523218451
ZNF252P-AS	0.584599258	0.00194675	1.604033619
ZNF253	13.36950827	5.12E-06	0.475064429
ZNF26	3.405279535	1.54E-06	2.046023882
ZNF260	17.56970737	0.000217553	0.556652767
ZNF263	18.73717676	0.004501321	0.645945641
ZNF273	1.802796007	0.004005412	1.546765228
ZNF276	10.91746016	4.44E-05	1.840904979
ZNF28	12.84280577	0.000122201	0.550352522
ZNF280A	0.067906007	8.26E-05	2.073145038
ZNF287	6.207495106	0.00109255	0.591097068
ZNF296	3.938714946	0.00135563	1.629258424
ZNF304	15.4422778	2.43E-09	0.375047533
ZNF311	2.83190743	0.004148826	1.54366842
ZNF317	27.49204657	0.002768148	0.631881335
ZNF32-AS1	2.28548052	0.000225188	1.742538874
ZNF32-AS2	4.016898777	0.00081523	1.651813653
ZNF322	5.122943583	0.003725154	0.636102911

ZNF329	13.62384856	3.78E-08	0.404775505
ZNF337	4.73278739	0.00394069	1.543299224
ZNF33B	33.81091783	0.001206004	0.601141837
ZNF33BP1	0.443412135	2.62E-05	1.898741114
ZNF341	3.726104	0.001337281	1.62534276
ZNF346	12.56976828	0.004699707	0.638566804
ZNF347	6.264284473	0.002553728	0.616289682
ZNF365	1.408647965	0.000192549	1.833570418
ZNF366	9.89289333	3.01E-07	0.414651193
ZNF367	8.574655176	0.000953872	0.582869091
ZNF37BP	7.431296922	0.003020628	1.561995481
ZNF385A	36.58089552	0.000112959	1.789630889
ZNF396	3.492656397	1.97E-05	0.504464728
ZNF398	11.79683987	0.001928813	0.610619082
ZNF407	5.263505142	0.000288753	0.555600635
ZNF41	12.83393659	0.00304141	0.62705811
ZNF415P1	0.085012954	0.000237672	1.760998787
ZNF416	9.155204481	1.82E-07	0.431212072
ZNF420	10.08507458	2.15E-07	0.433242612
ZNF426	9.894195446	8.05E-05	0.5200163
ZNF428	50.67074129	0.003172548	1.564273409
ZNF429	10.45170472	0.004322682	0.63771831
ZNF433-AS	1.536843098	4.45E-05	1.836325413
ZNF436-AS	5.513551966	1.65E-05	1.903272548
ZNF440	8.001675279	0.000452091	0.567886519
ZNF444	13.69335773	9.92E-05	1.794984446
ZNF45	14.05069086	9.29E-06	0.493137911
ZNF451-AS	0.487613928	0.000155607	1.75758627
ZNF460-AS	3.051702656	0.000426612	1.697732579
ZNF462	12.60074656	1.79E-05	0.502647748
ZNF470	4.834951906	0.003169325	0.623848988
ZNF473	6.601546035	0.000152001	0.552542816
ZNF474	0.564032397	8.50E-05	1.856441914
ZNF480	17.62170952	0.003439686	0.630798334
ZNF483	1.307274508	0.002283541	0.592819894
ZNF484	6.273018435	0.003893095	0.633567549
ZNF487	4.194325756	0.000289247	1.729771696
ZNF503-AS	2.765219106	8.45E-05	0.535803946
ZNF503-AS2	3.557129444	0.002015423	0.576959297
ZNF510	9.802532067	0.000111589	0.541302224
ZNF513	27.48737933	0.000642337	1.672237051
ZNF517	8.434106787	0.000957927	1.64397343
ZNF518B	5.62763823	0.000974745	0.583757175
ZNF521	11.74947962	0.000668131	0.558714932
ZNF528-AS	6.655497335	0.002446727	1.579030234
ZNF529-AS	6.912031739	0.000540447	1.685353877
ZNF542P	8.301355076	1.97E-05	0.491232314
ZNF543	6.836406098	1.75E-06	0.457119454
ZNF549	3.776758679	1.76E-07	0.424960468
ZNF551	6.249814693	4.27E-05	0.516734429
ZNF555	3.463592227	6.03E-05	0.51395175
ZNF561	22.46207763	8.25E-06	0.495649311
ZNF564	1.06098496	0.000835387	0.584289619
ZNF568	4.283017933	0.000289107	0.564345644
ZNF569	6.598199336	0.000876242	0.593945001
ZNF570	6.730705228	3.95E-06	0.472917019
ZNF572	3.455112322	0.004186909	0.635115178
ZNF573	2.329607598	0.003622234	0.621883607
ZNF576	13.92740954	0.000481502	0.584968371
ZNF579	14.35829762	6.79E-05	1.820206265
ZNF580	30.7867672	2.98E-05	1.868769285
ZNF582	2.984150795	0.001956371	0.604271746
ZNF583	2.634419329	1.17E-05	0.493634008
ZNF587P1	0.162387998	0.000178002	1.782024998
ZNF592	29.90401261	0.000881557	0.594916777
ZNF593	4.824595392	0.001394758	1.625090364
ZNF595	8.852539507	0.00102403	0.600940618
ZNF598	21.79522784	0.001315888	1.627771402
ZNF603P	0.673855989	0.000288758	1.738261145
ZNF609	16.51926139	0.002151604	0.611207152
ZNF610	4.264007812	0.00091908	0.58664747
ZNF611	16.45695647	9.98E-05	0.513397883
ZNF613	6.847982555	6.78E-05	0.538563132
ZNF614	11.41560933	9.14E-05	0.529585595
ZNF616	11.00319669	0.004857197	0.639650529
ZNF618	17.47994701	0.001333241	0.588879544
ZNF619	4.235559951	5.42E-06	0.465601062
ZNF625-ZN	0.094089548	0.003640613	0.631407139
ZNF627	20.48865398	8.81E-06	0.495617299
ZNF644	21.21750251	0.002649015	0.625820647
ZNF649	9.147376496	5.48E-06	0.483304537
ZNF654	17.21747622	0.000728116	0.572821992
ZNF658	2.776011274	5.64E-09	0.38356313
ZNF658B	0.940253897	6.91E-07	0.437829948
ZNF669	6.789294001	0.000251743	0.544284023
ZNF670	5.342828111	0.000175799	0.544787332
ZNF677	3.90346286	0.000579254	0.561644368
ZNF680P1	0.128187682	0.001901597	1.617105272
ZNF682	3.502464205	0.000294727	1.719082424
ZNF683	5.12117219	0.001944992	1.605567873
ZNF684	6.3945864	0.00128759	0.580474977
ZNF687	23.04323872	0.000239912	0.553176838
ZNF689	11.99847134	0.000832153	0.593196598
ZNF692	23.17702634	7.97E-07	2.071013667
ZNF695	0.200100741	0.002241193	1.600397284
ZNF697	14.77240187	4.07E-05	0.506562801
ZNF699	4.2776591	8.53E-06	0.474860073
ZNF703	15.41195274	0.004677798	1.538332465
ZNF704	20.83102763	3.31E-08	0.399299804
ZNF705A	0.055639503	0.000957026	1.687351467
ZNF706	27.47050353	6.96E-05	1.822749641
ZNF710	17.70239121	0.001450202	0.596105461
ZNF716	0.019928973	0.000277946	1.791163651
ZNF718	7.082861232	0.000274168	0.553339052
ZNF726	0.690998859	0.001427544	1.631413447
ZNF732	0.412157717	8.63E-05	1.80826076
ZNF736P1Y	0	0.004606615	1.557019068
ZNF736P2Y	0	0.004606615	1.557019068
ZNF736P5Y	0	0.004606615	1.557019068
ZNF738	4.376743125	0.001495536	1.609425573
ZNF750	0.528934697	0.000282438	0.475666711
ZNF765	3.673683792	0.004224673	0.632107668
ZNF767P	11.0529211	4.96E-05	1.833744253
ZNF770	29.24320612	1.26E-05	0.496523957
ZNF772	5.99580785	0.000173296	0.533707232
ZNF775	7.57626138	1.32E-09	2.442993271
ZNF776	15.82426262	1.47E-05	0.506314815
ZNF778	4.330071622	0.000327731	0.560188331
ZNF780A	7.351225054	0.000212473	0.54758629
ZNF783	8.301882495	2.49E-05	1.871365737
ZNF788P	3.663705624	0.003303109	0.630397278
ZNF789	6.722230148	0.001592026	1.605363596
ZNF79	9.96535938	8.68E-06	0.486985205
ZNF790	8.041348442	0.004420639	0.637505141
ZNF792	7.149230016	1.34E-05	0.496229156
ZNF80	0.353660613	6.78E-05	1.828550486
ZNF805	4.670486982	0.00064193	0.580258967
ZNF812P	3.340354963	0.003057706	0.564817643
ZNF816	11.47298964	0.001089238	0.592815541
ZNF821	6.555043413	5.85E-05	0.527163956
ZNF829	2.241572341	2.90E-06	0.454015323
ZNF83	43.6633985	3.93E-07	2.114337783
ZNF830	29.0508872	4.77E-06	0.472004077
ZNF835	2.510838566	0.00202692	0.602098124
ZNF836	8.508238672	1.46E-05	0.492063698
ZNF844	13.21286361	8.48E-07	0.430741825
ZNF845	8.142321685	7.10E-06	0.490523603
ZNF847P	0.131278329	1.14E-06	2.06705486
ZNF863P	0.08425549	0.001922686	1.683486847
ZNF880	9.74687097	7.44E-05	0.513861471
ZNF962P	0.010660204	0.002848798	1.833380899
ZNF970P	0.059351277	0.001524511	1.785480971
ZNFX1	45.13083439	0.00146803	0.606656384
ZNHIT1	58.90701393	0.001019596	1.646858573
ZNRF2	30.13529547	0.000109286	0.529003248
ZNRF3	0.227488084	0.001112391	0.58027822
ZNRF3-AS1	0	0.004606615	1.557019068
ZNRF3-IT1	0	0.004606615	1.557019068
ZNRF4	0.02951641	0.00318623	1.670827018
ZP1	1.369325804	1.61E-05	2.037101869
ZP3	8.234831238	7.45E-05	1.815151364
ZPBP2	0.031268795	0.00074321	1.704935055
ZPLD1	1.076238396	0.002003475	1.948154062
ZRANB1	11.89496665	1.59E-05	0.504982784
ZSCAN10	0.047770442	8.63E-05	1.85581247
ZSCAN2	7.357361644	1.81E-06	0.4560091
ZSCAN31	11.90467463	0.000627438	0.564493919
ZSCAN5DP	0.227161781	5.40E-05	1.85044795
ZSWIM5	12.33040897	0.000763477	0.580589317
ZSWIM8-AS	0.230641136	0.001209709	1.627335649

ZW10	22.62541009	0.000313429	0.573895477
ZXDA	8.125100098	2.43E-08	0.406212388
ZXDB	11.51002478	0.000233763	0.559348638
ZYG11B	28.84583134	2.93E-07	0.42678569
ZZEF1	16.48811586	0.002516792	0.621549034
