# Supplementary material for: N6-methyladenosine-modified ALDH9A1 modulates lipid accumulation and tumor progression in clear cell renal cell carcinoma through the NPM1/IQGAP2/AKT signaling pathway
Source: Cell Death Dis. 2024 Jul 22;15(7):520. doi: 10.1038/s41419-024-06896-z (PMC11263707; doi:10.1038/s41419-024-06896-z)
Supplement: Supplementary file 7 — Original data [file 41419_2024_6896_MOESM7_ESM.pptx]

## Slide 1
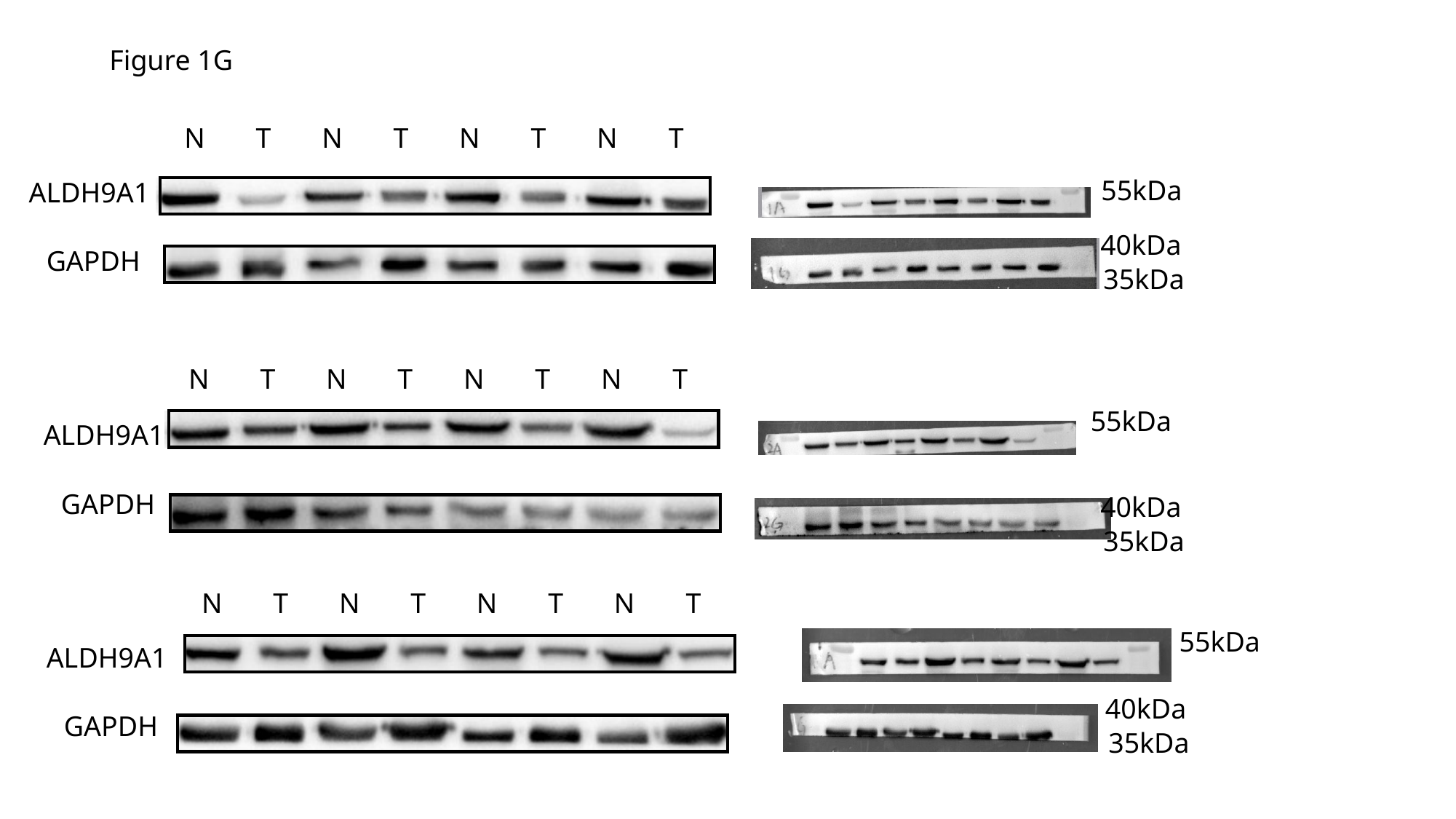

Figure 1G
| N | T | N | T | N | T | N | T |
| --- | --- | --- | --- | --- | --- | --- | --- |
55kDa
ALDH9A1
40kDa
GAPDH
35kDa
| N | T | N | T | N | T | N | T |
| --- | --- | --- | --- | --- | --- | --- | --- |
55kDa
ALDH9A1
GAPDH
40kDa
35kDa
| N | T | N | T | N | T | N | T |
| --- | --- | --- | --- | --- | --- | --- | --- |
55kDa
ALDH9A1
40kDa
GAPDH
35kDa

## Slide 2
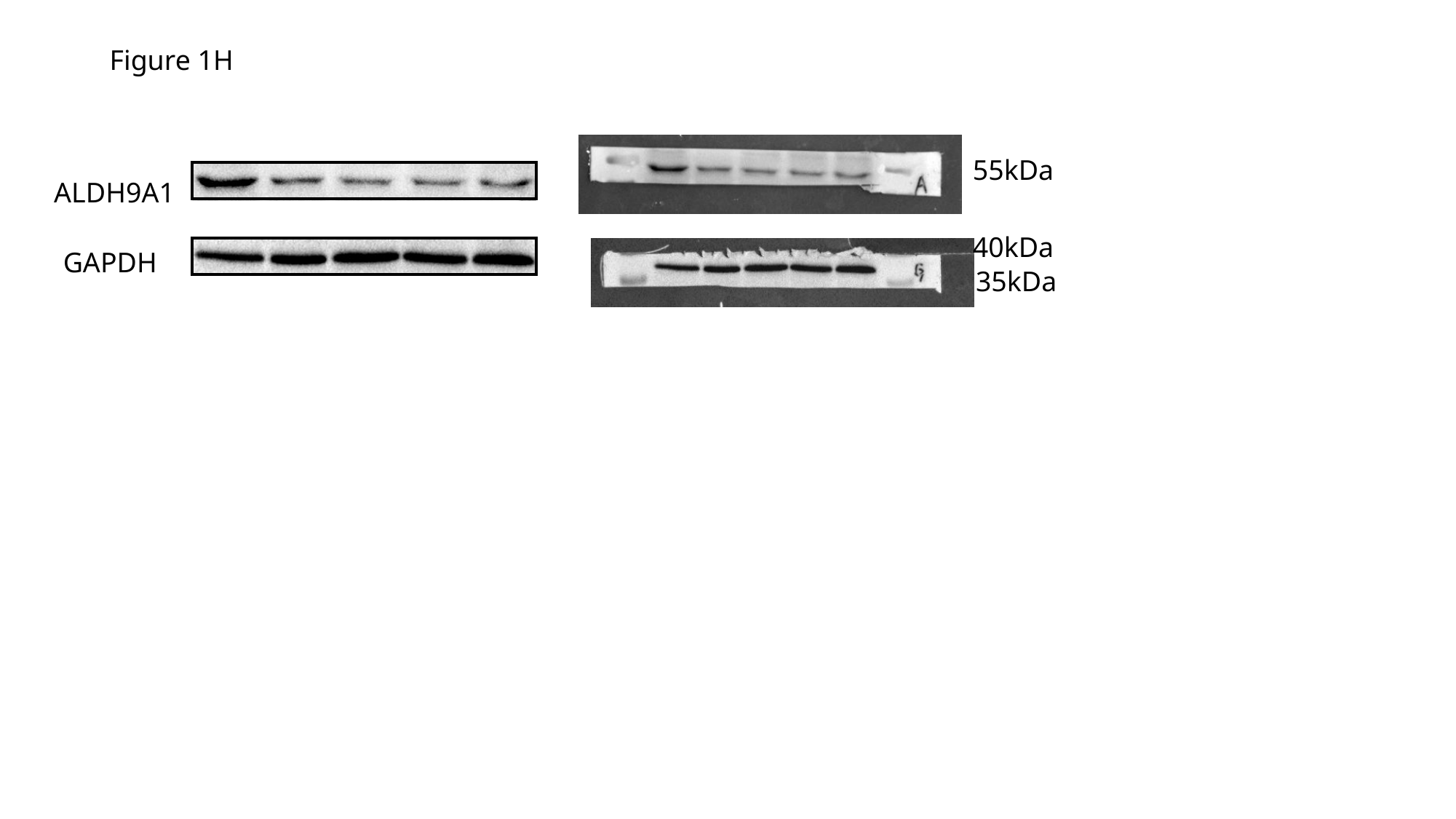

Figure 1H
55kDa
ALDH9A1
40kDa
GAPDH
35kDa

## Slide 3
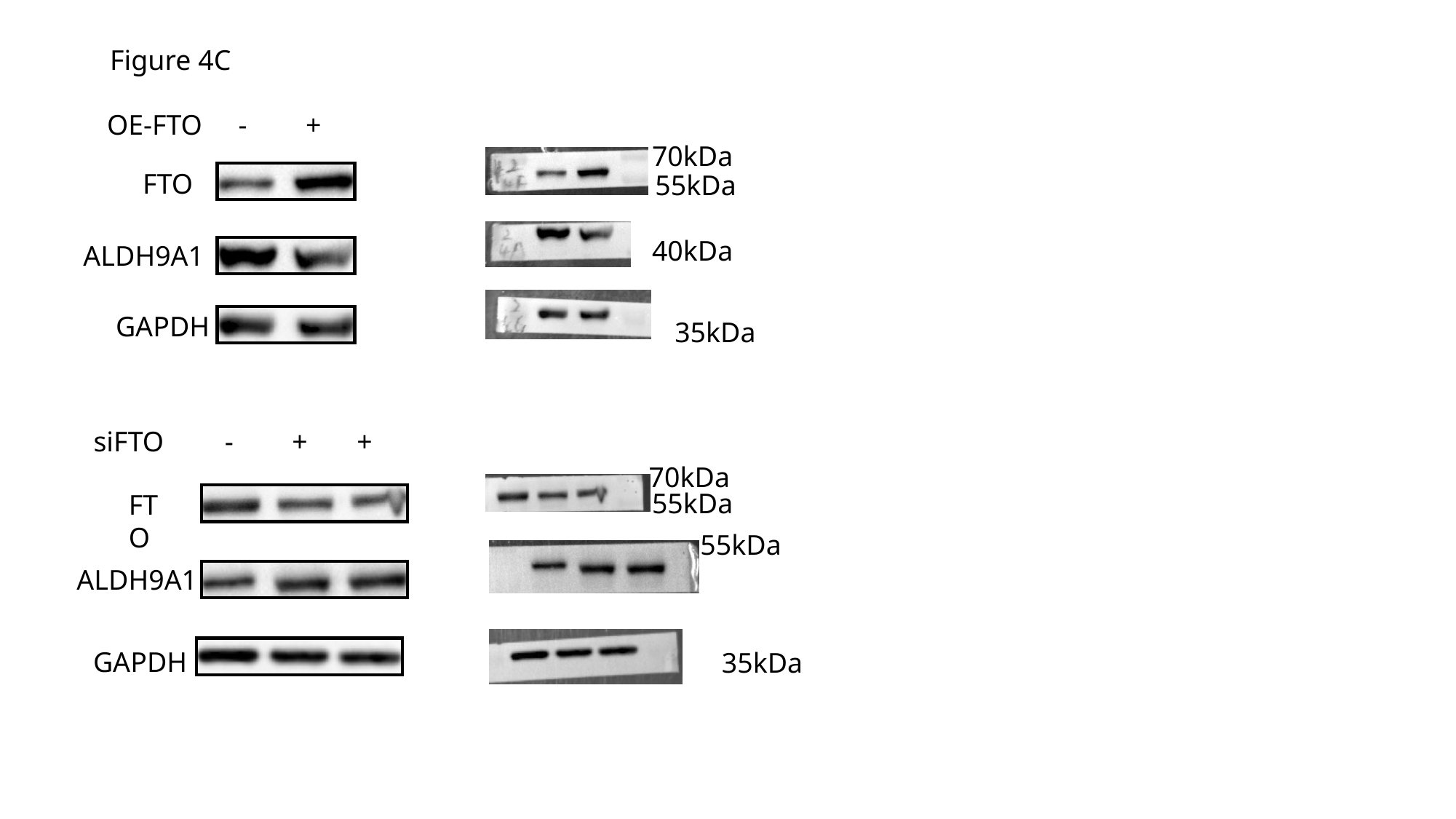

Figure 4C
| OE-FTO | - | + | |
| --- | --- | --- | --- |
70kDa
FTO
55kDa
40kDa
ALDH9A1
GAPDH
35kDa
| siFTO | - | + | + |
| --- | --- | --- | --- |
70kDa
55kDa
FTO
55kDa
ALDH9A1
GAPDH
35kDa

## Slide 4
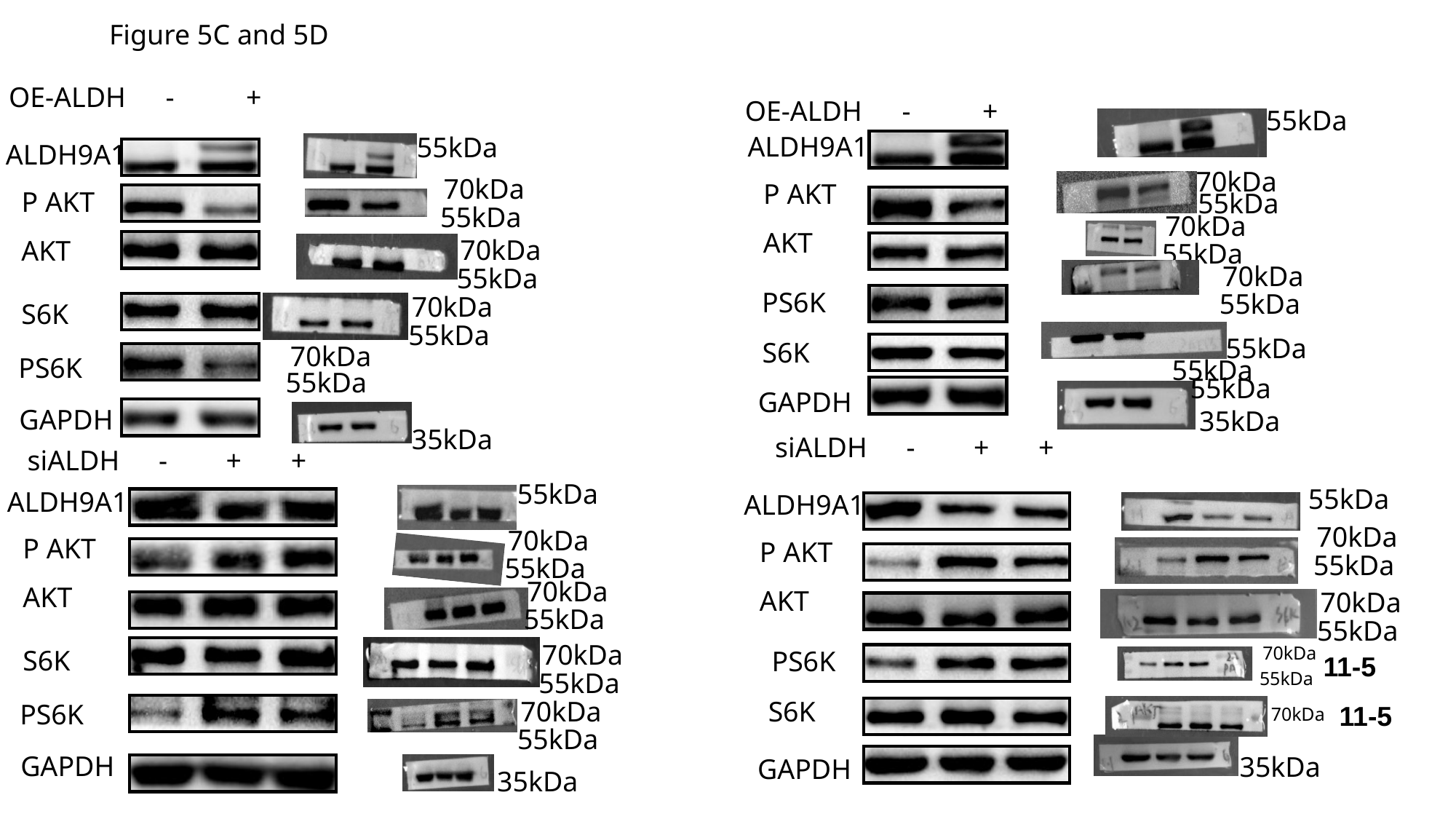

Figure 5C and 5D
| OE-ALDH | - | + | |
| --- | --- | --- | --- |
| OE-ALDH | - | + | |
| --- | --- | --- | --- |
55kDa
ALDH9A1
55kDa
ALDH9A1
70kDa
70kDa
P AKT
P AKT
55kDa
55kDa
70kDa
AKT
70kDa
AKT
55kDa
70kDa
55kDa
PS6K
55kDa
70kDa
S6K
55kDa
55kDa
S6K
70kDa
PS6K
55kDa
55kDa
55kDa
GAPDH
GAPDH
35kDa
35kDa
| siALDH | - | + | + |
| --- | --- | --- | --- |
| siALDH | - | + | + |
| --- | --- | --- | --- |
55kDa
55kDa
ALDH9A1
ALDH9A1
70kDa
70kDa
P AKT
P AKT
55kDa
55kDa
70kDa
AKT
AKT
70kDa
55kDa
55kDa
70kDa
70kDa
S6K
PS6K
11-5
55kDa
55kDa
70kDa
S6K
PS6K
11-5
70kDa
55kDa
GAPDH
35kDa
GAPDH
35kDa

## Slide 5
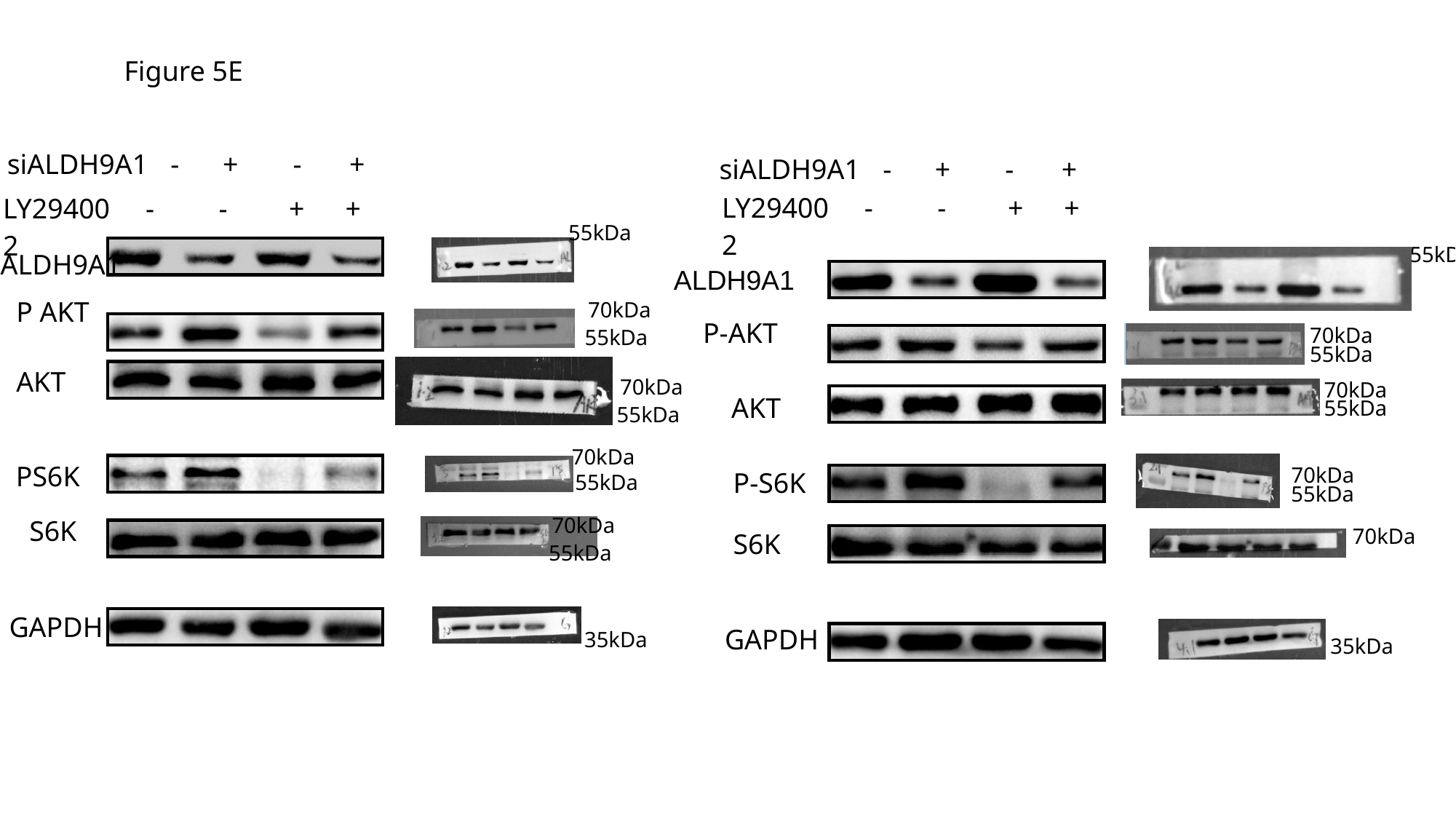

Figure 5E
| siALDH9A1 | - | + | - | + | |
| --- | --- | --- | --- | --- | --- |
| siALDH9A1 | - | + | - | + | |
| --- | --- | --- | --- | --- | --- |
| LY294002 | - | - | + | + | |
| --- | --- | --- | --- | --- | --- |
| LY294002 | - | - | + | + | |
| --- | --- | --- | --- | --- | --- |
55kDa
55kDa
ALDH9A1
ALDH9A1
P AKT
70kDa
P-AKT
70kDa
55kDa
55kDa
AKT
70kDa
70kDa
AKT
55kDa
55kDa
70kDa
PS6K
70kDa
P-S6K
55kDa
55kDa
70kDa
S6K
70kDa
S6K
55kDa
GAPDH
GAPDH
35kDa
35kDa

## Slide 6
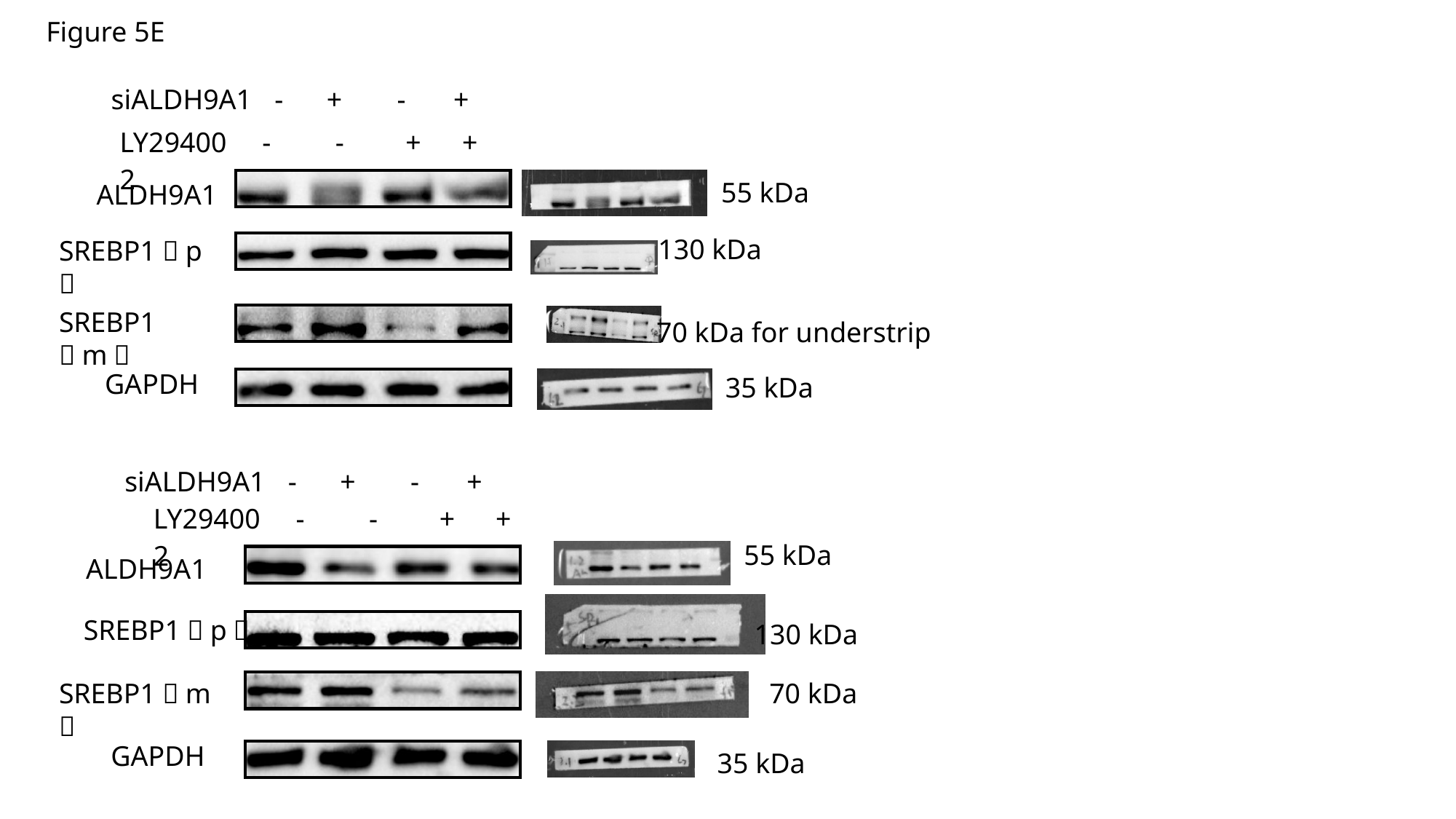

Figure 5E
| siALDH9A1 | - | + | - | + | |
| --- | --- | --- | --- | --- | --- |
| LY294002 | - | - | + | + | |
| --- | --- | --- | --- | --- | --- |
55 kDa
ALDH9A1
130 kDa
SREBP1（p）
SREBP1（m）
70 kDa for understrip
GAPDH
35 kDa
| siALDH9A1 | - | + | - | + | |
| --- | --- | --- | --- | --- | --- |
| LY294002 | - | - | + | + | |
| --- | --- | --- | --- | --- | --- |
55 kDa
ALDH9A1
SREBP1（p）
130 kDa
70 kDa
SREBP1（m）
GAPDH
35 kDa

## Slide 7
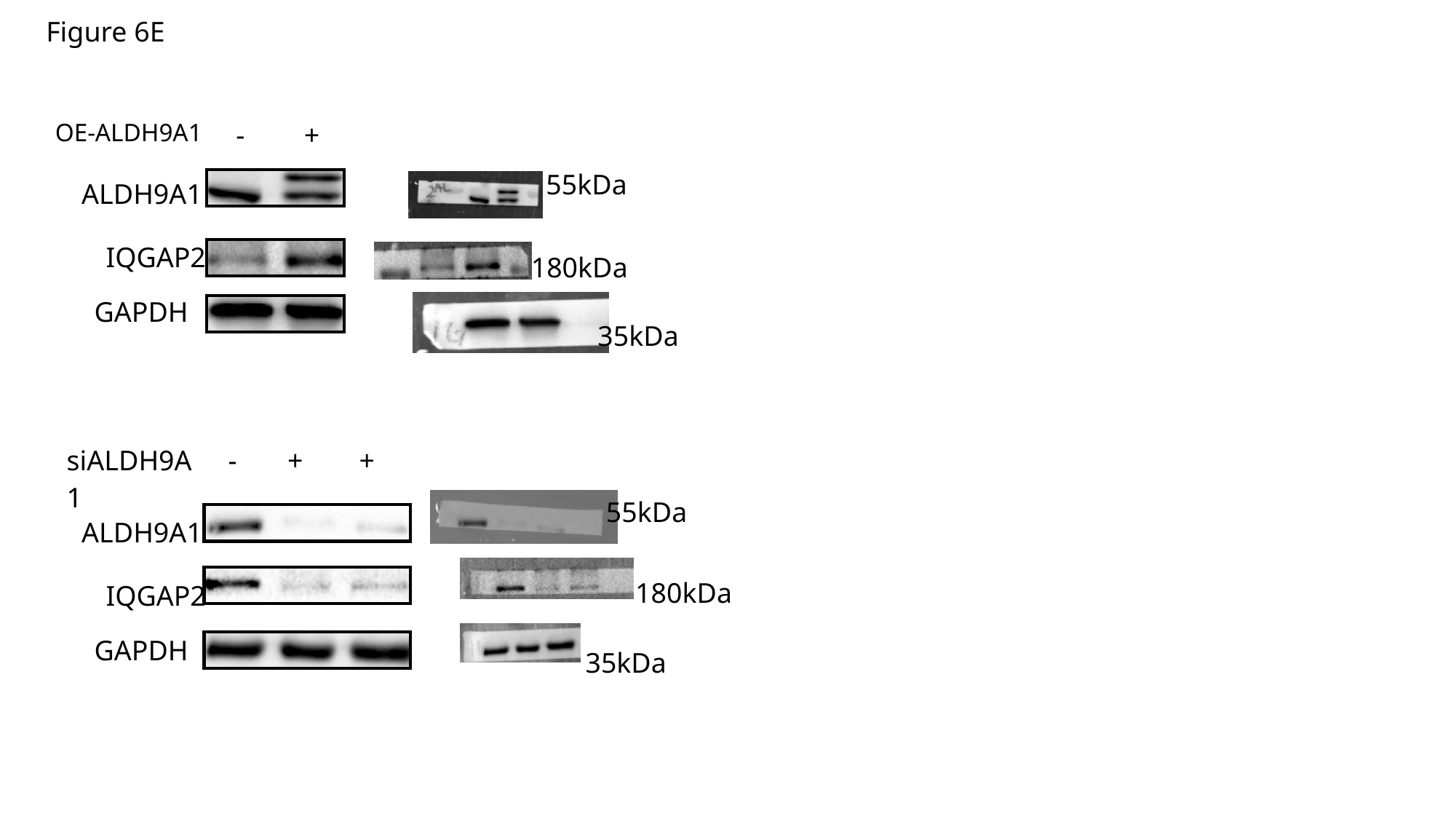

Figure 6E
| OE-ALDH9A1 | - | + | |
| --- | --- | --- | --- |
55kDa
ALDH9A1
IQGAP2
180kDa
GAPDH
35kDa
| siALDH9A1 | - | + | + |
| --- | --- | --- | --- |
55kDa
ALDH9A1
180kDa
IQGAP2
GAPDH
35kDa

## Slide 8
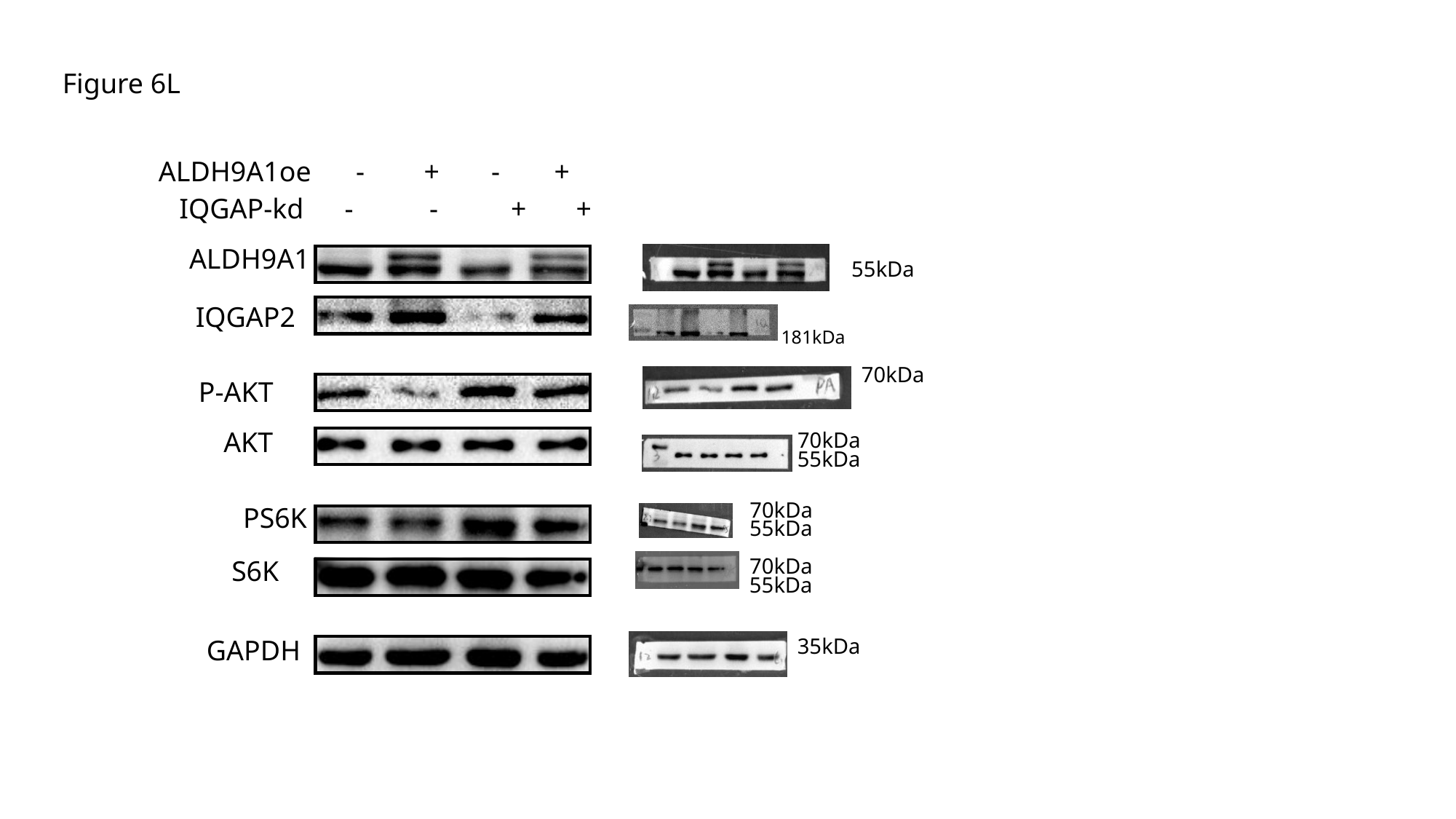

Figure 6L
| ALDH9A1oe | - | + | - | + | |
| --- | --- | --- | --- | --- | --- |
| IQGAP-kd | - | - | + | + | |
| --- | --- | --- | --- | --- | --- |
ALDH9A1
55kDa
IQGAP2
181kDa
70kDa
P-AKT
AKT
70kDa
55kDa
70kDa
PS6K
55kDa
70kDa
S6K
55kDa
35kDa
GAPDH

## Slide 9
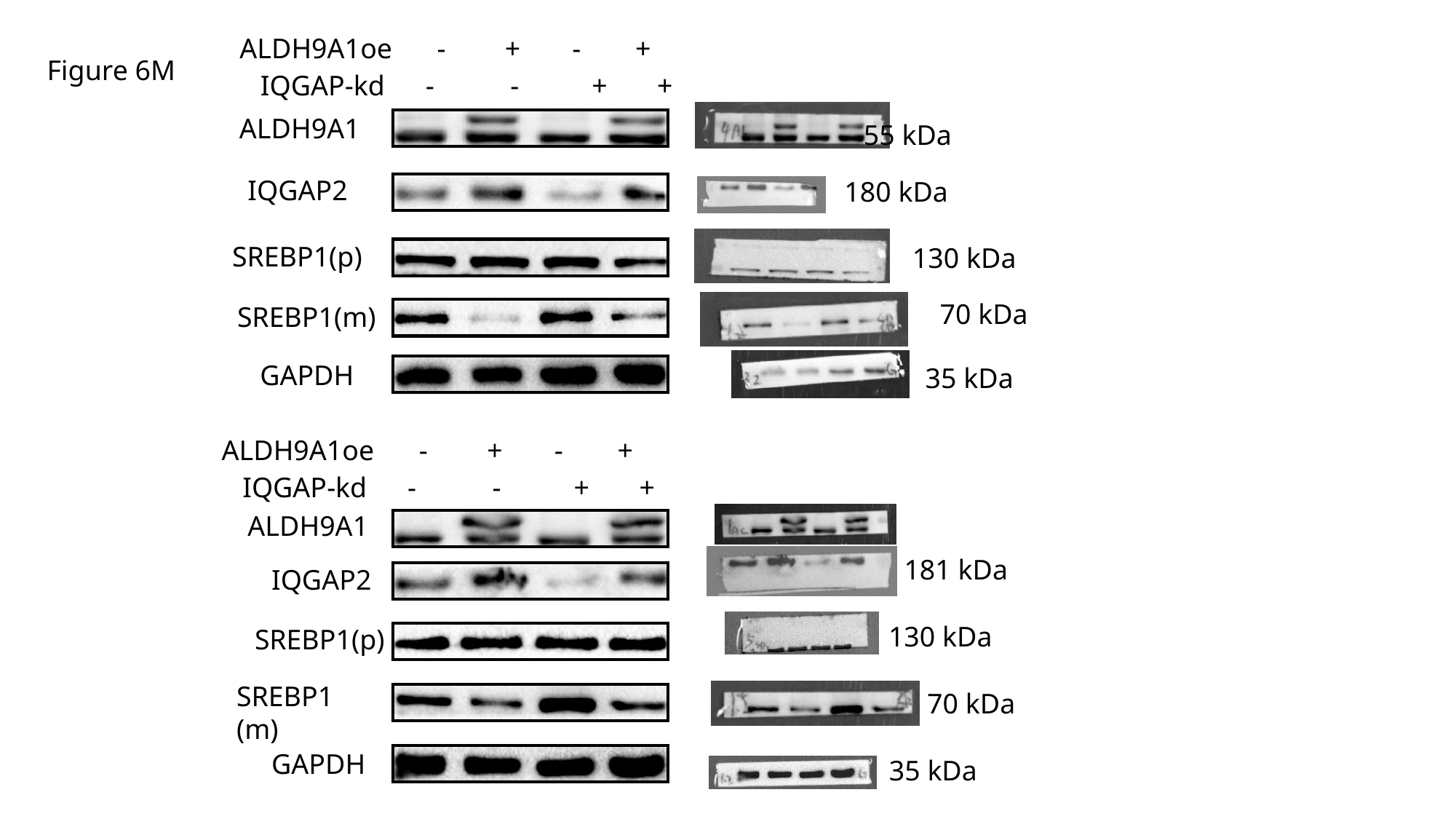

| ALDH9A1oe | - | + | - | + | |
| --- | --- | --- | --- | --- | --- |
Figure 6M
| IQGAP-kd | - | - | + | + | |
| --- | --- | --- | --- | --- | --- |
ALDH9A1
55 kDa
IQGAP2
180 kDa
SREBP1(p)
130 kDa
70 kDa
SREBP1(m)
GAPDH
35 kDa
| ALDH9A1oe | - | + | - | + | |
| --- | --- | --- | --- | --- | --- |
| IQGAP-kd | - | - | + | + | |
| --- | --- | --- | --- | --- | --- |
ALDH9A1
181 kDa
IQGAP2
130 kDa
SREBP1(p)
SREBP1 (m)
70 kDa
GAPDH
35 kDa

## Slide 10
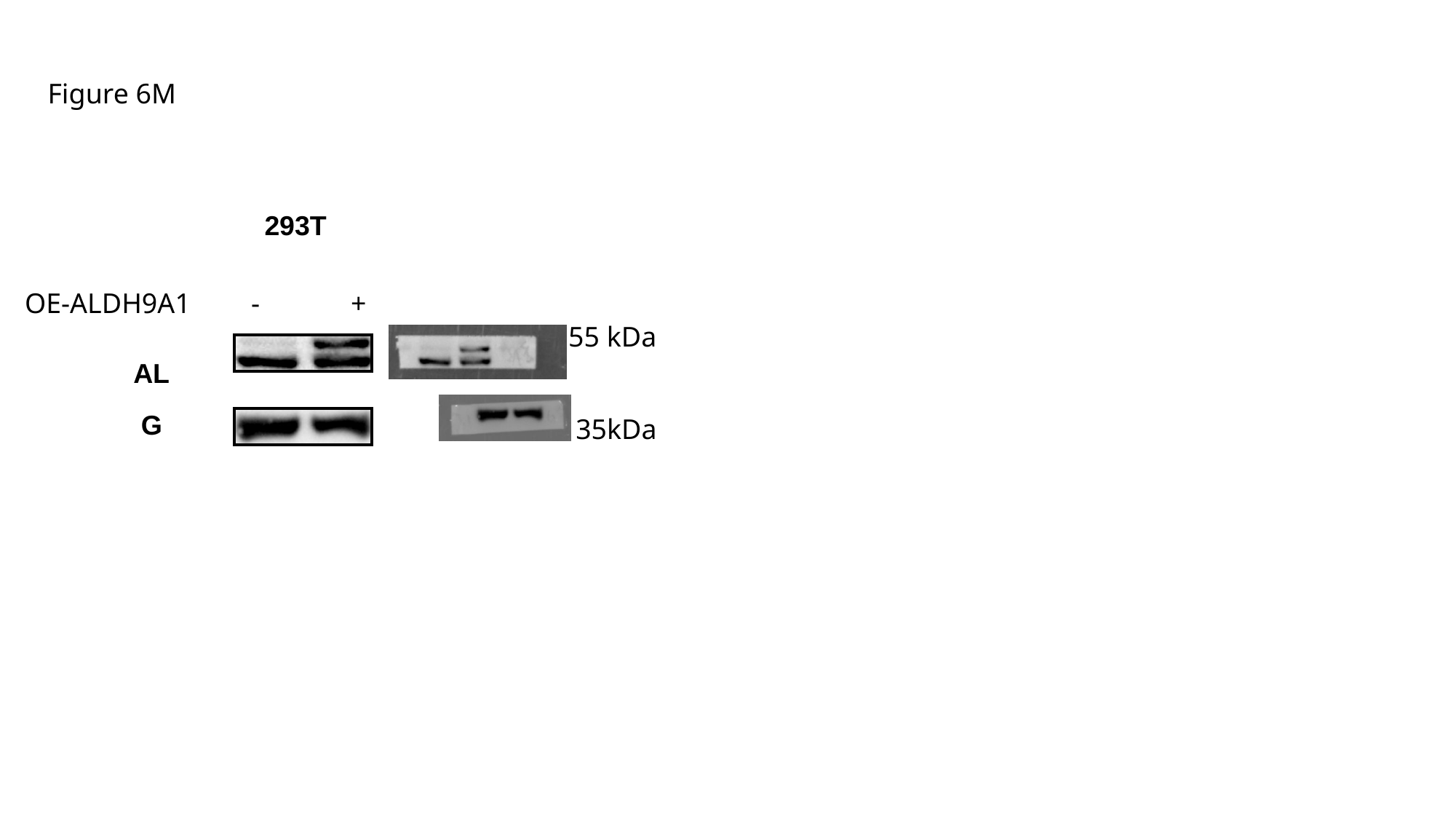

Figure 6M
293T
| OE-ALDH9A1 | - | + | |
| --- | --- | --- | --- |
55 kDa
AL
G
35kDa

## Slide 11
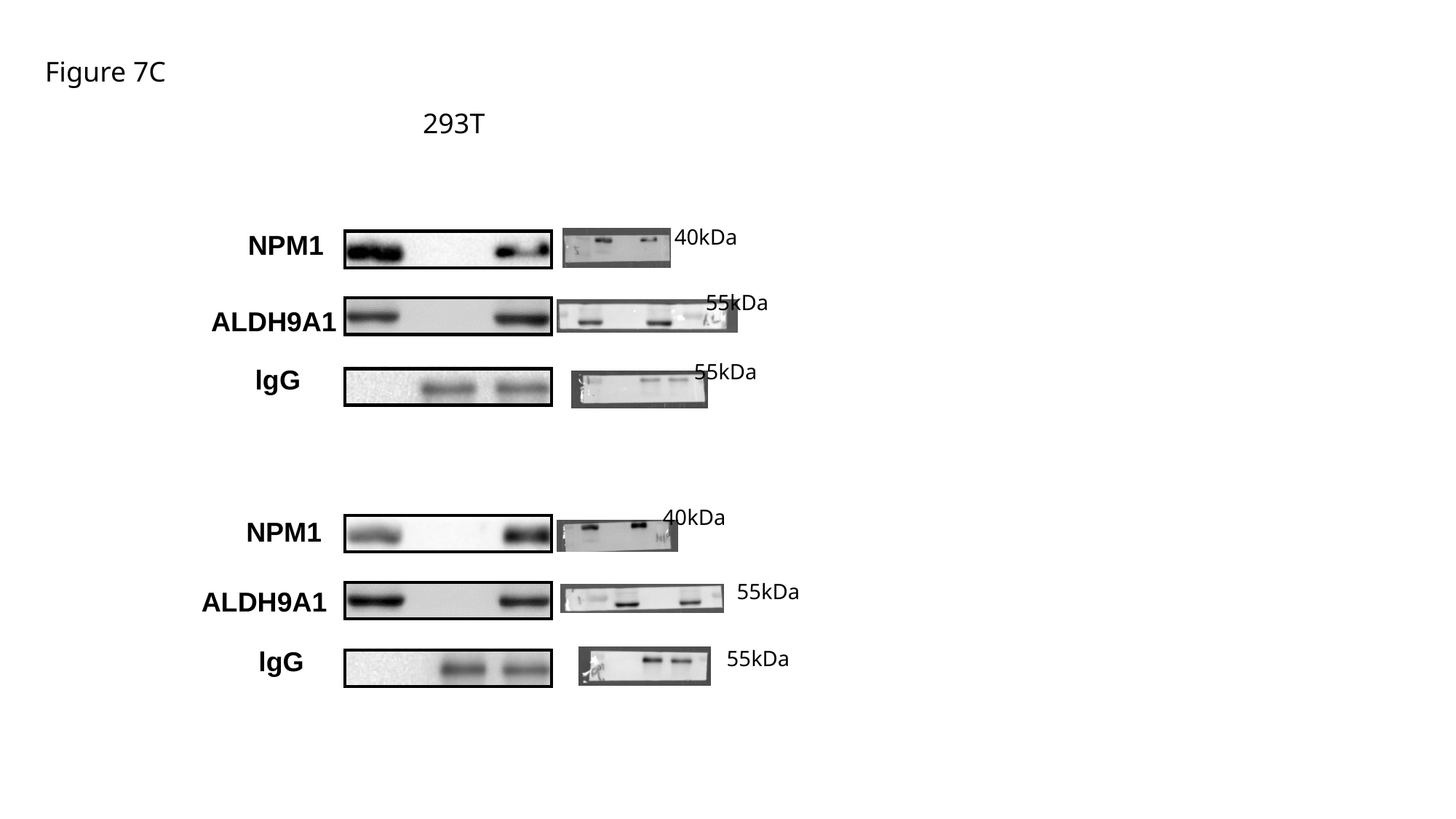

Figure 7C
293T
40kDa
NPM1
55kDa
ALDH9A1
55kDa
lgG
40kDa
NPM1
55kDa
ALDH9A1
lgG
55kDa

## Slide 12
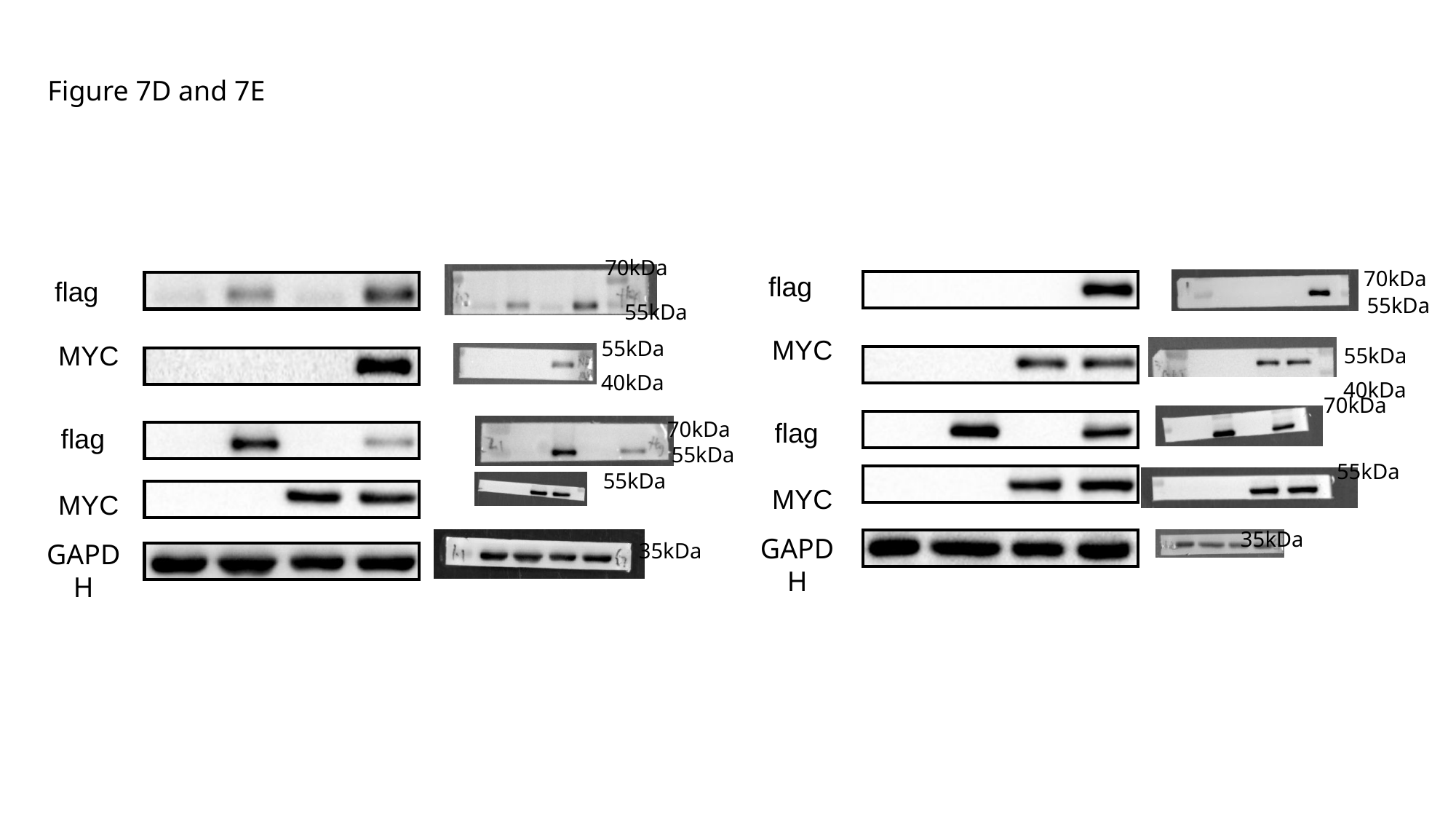

Figure 7D and 7E
70kDa
70kDa
flag
flag
55kDa
55kDa
MYC
55kDa
MYC
55kDa
40kDa
40kDa
70kDa
70kDa
flag
flag
55kDa
55kDa
55kDa
MYC
MYC
35kDa
GAPDH
35kDa
GAPDH

## Slide 13
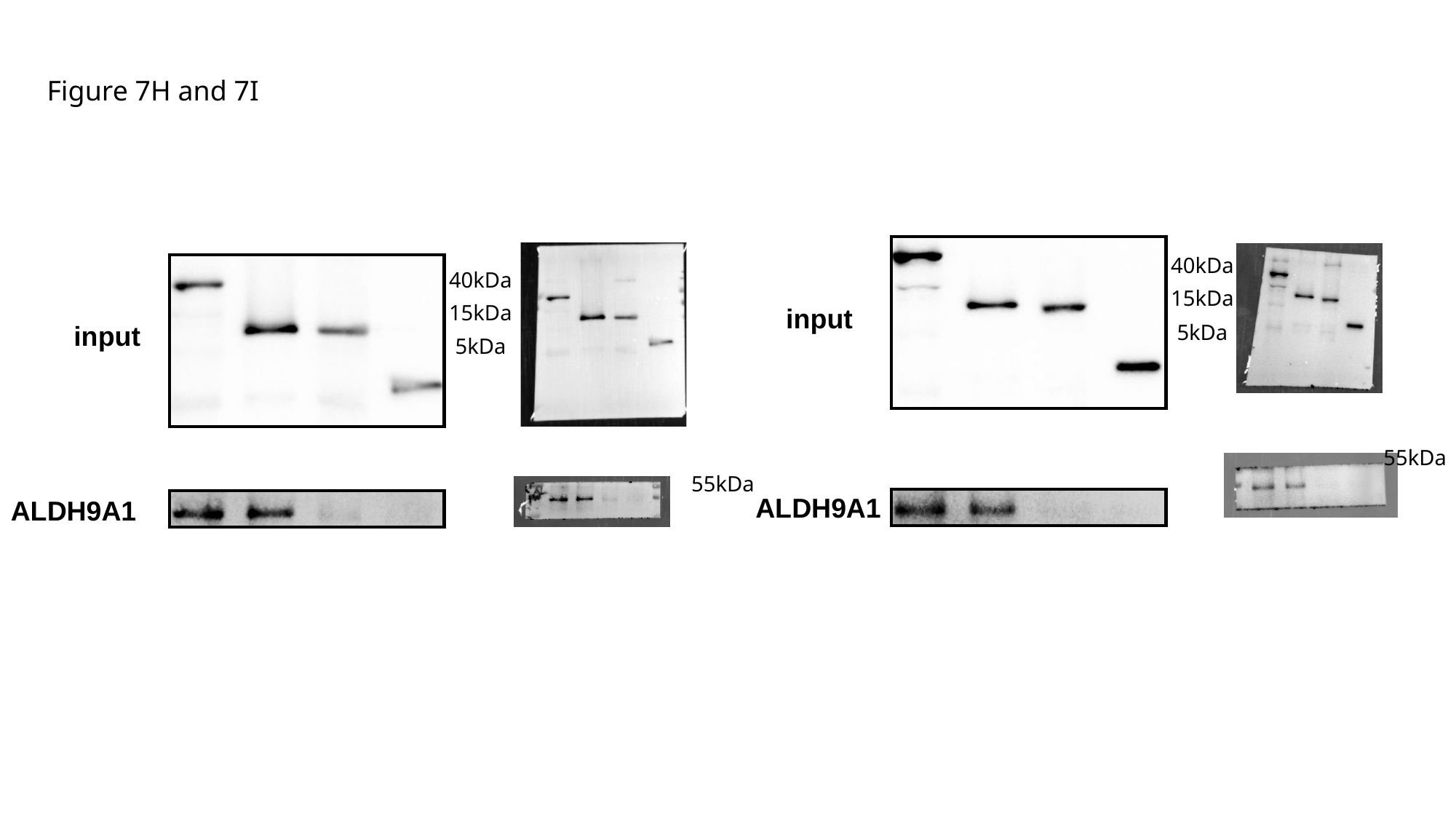

Figure 7H and 7I
40kDa
40kDa
15kDa
15kDa
input
input
5kDa
5kDa
55kDa
55kDa
ALDH9A1
ALDH9A1

## Slide 14
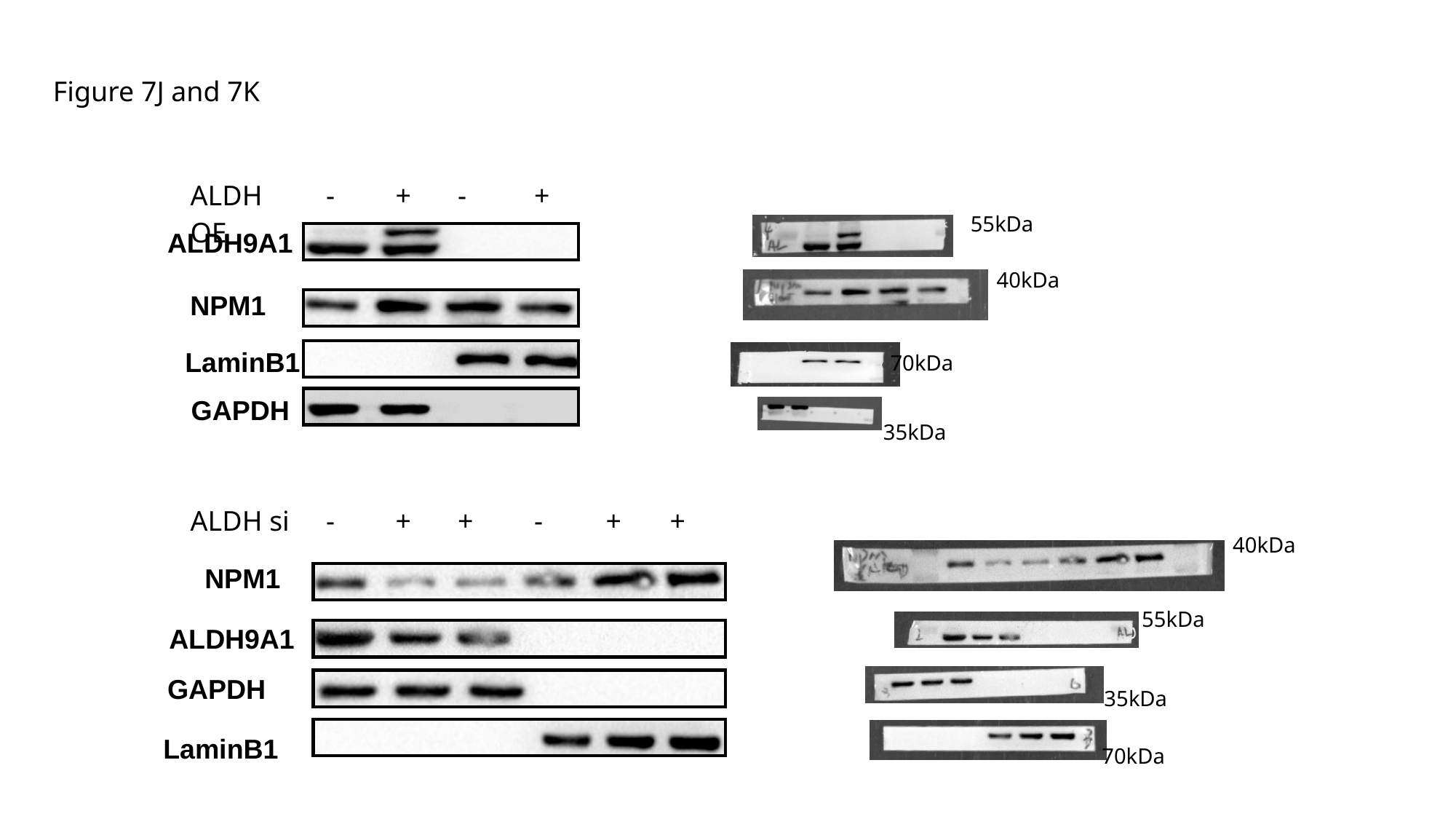

Figure 7J and 7K
| ALDH OE | - | + | - | + | | | | |
| --- | --- | --- | --- | --- | --- | --- | --- | --- |
55kDa
ALDH9A1
40kDa
NPM1
LaminB1
70kDa
GAPDH
35kDa
| ALDH si | - | + | + | - | + | + | | |
| --- | --- | --- | --- | --- | --- | --- | --- | --- |
40kDa
NPM1
55kDa
ALDH9A1
GAPDH
35kDa
LaminB1
70kDa

## Slide 15
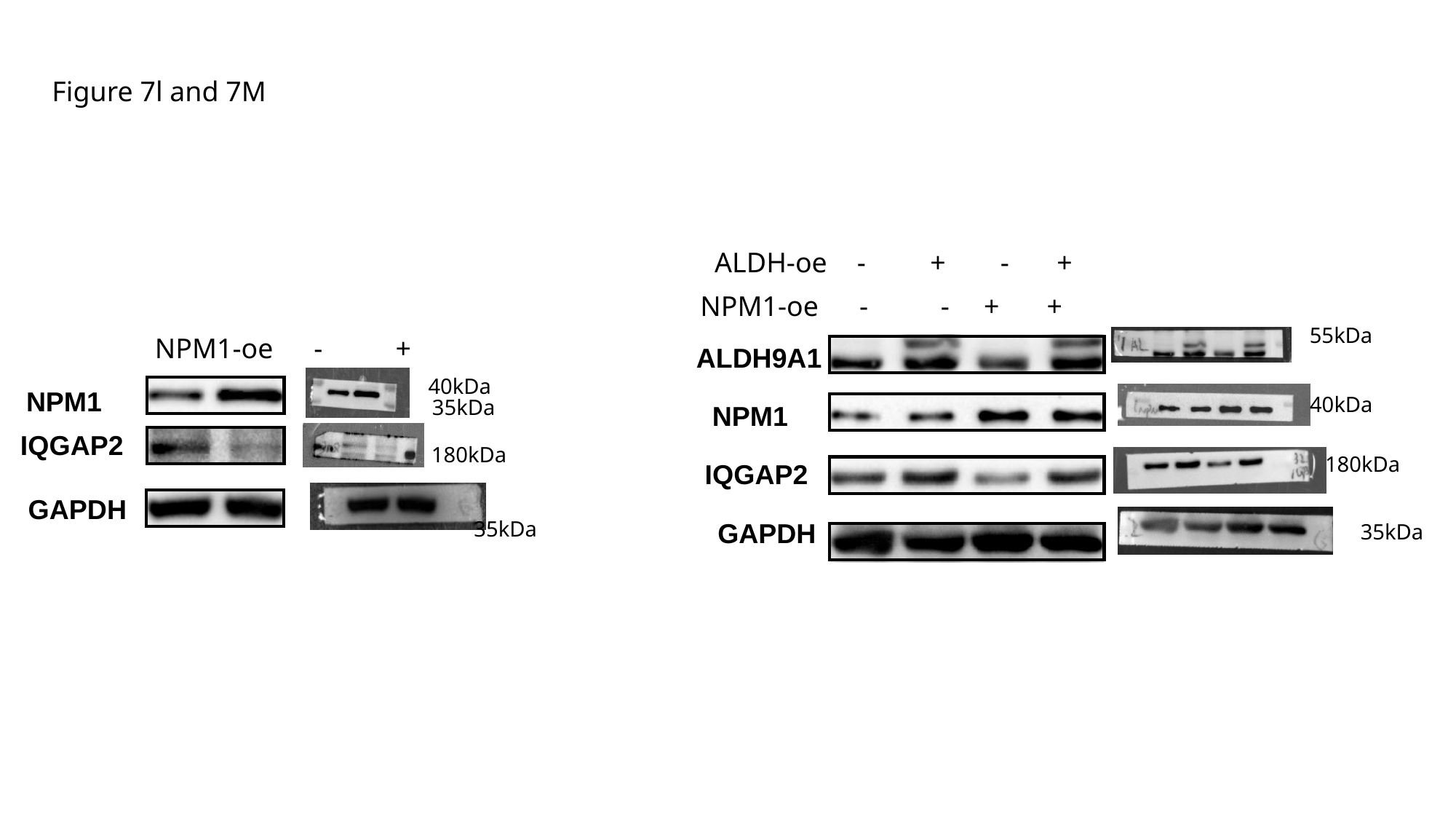

Figure 7l and 7M
| ALDH-oe | - | + | - | + | |
| --- | --- | --- | --- | --- | --- |
| NPM1-oe | - | - | + | + | |
| --- | --- | --- | --- | --- | --- |
55kDa
| NPM1-oe | - | + | | | |
| --- | --- | --- | --- | --- | --- |
ALDH9A1
40kDa
NPM1
40kDa
35kDa
NPM1
IQGAP2
180kDa
180kDa
IQGAP2
GAPDH
35kDa
GAPDH
35kDa

## Slide 16
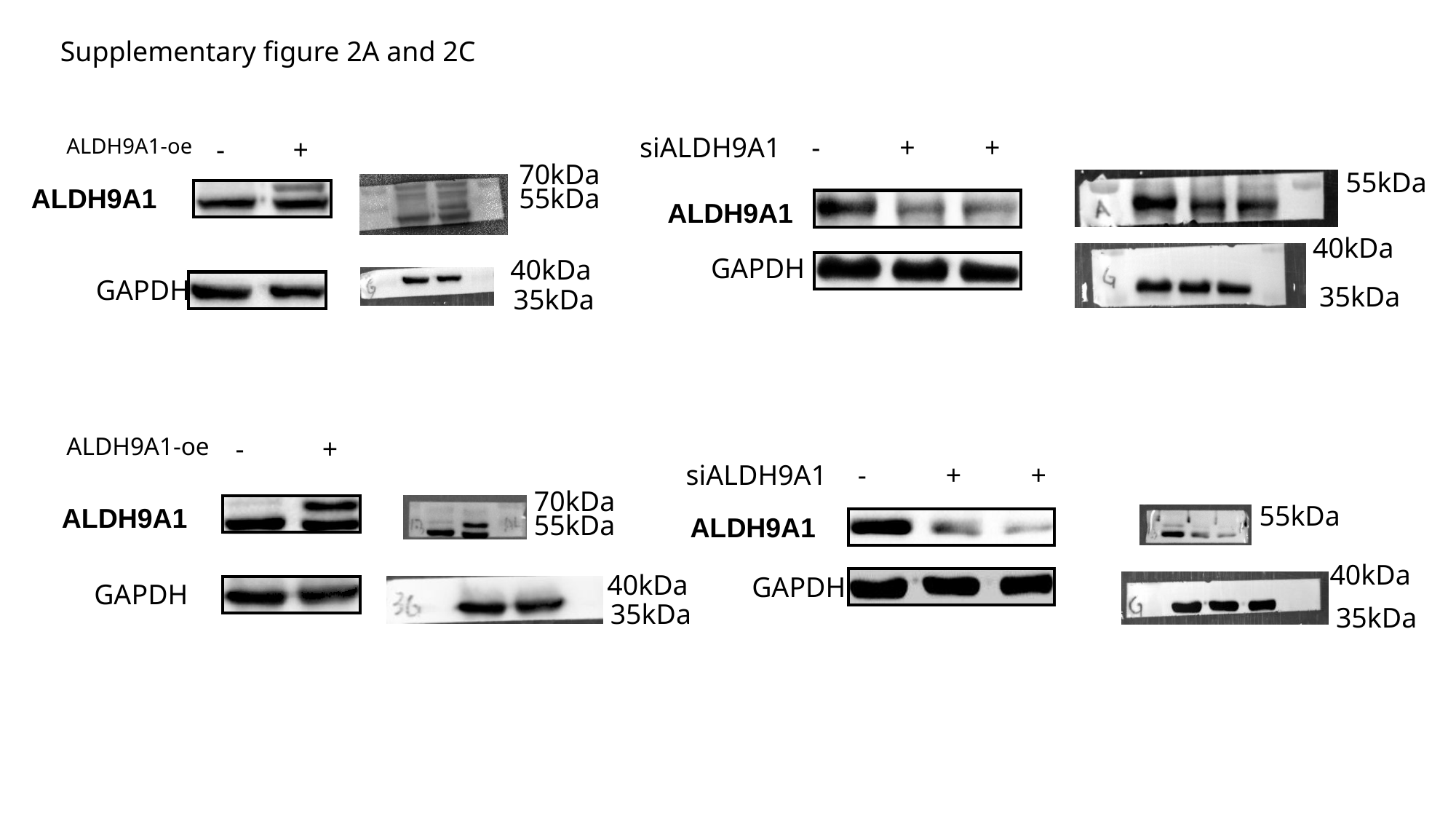

Supplementary figure 2A and 2C
| siALDH9A1 | - | + | + |
| --- | --- | --- | --- |
| ALDH9A1-oe | - | + | |
| --- | --- | --- | --- |
70kDa
55kDa
ALDH9A1
55kDa
ALDH9A1
40kDa
GAPDH
40kDa
GAPDH
35kDa
35kDa
| ALDH9A1-oe | - | + | |
| --- | --- | --- | --- |
| siALDH9A1 | - | + | + |
| --- | --- | --- | --- |
70kDa
55kDa
ALDH9A1
55kDa
ALDH9A1
40kDa
40kDa
GAPDH
GAPDH
35kDa
35kDa

## Slide 17
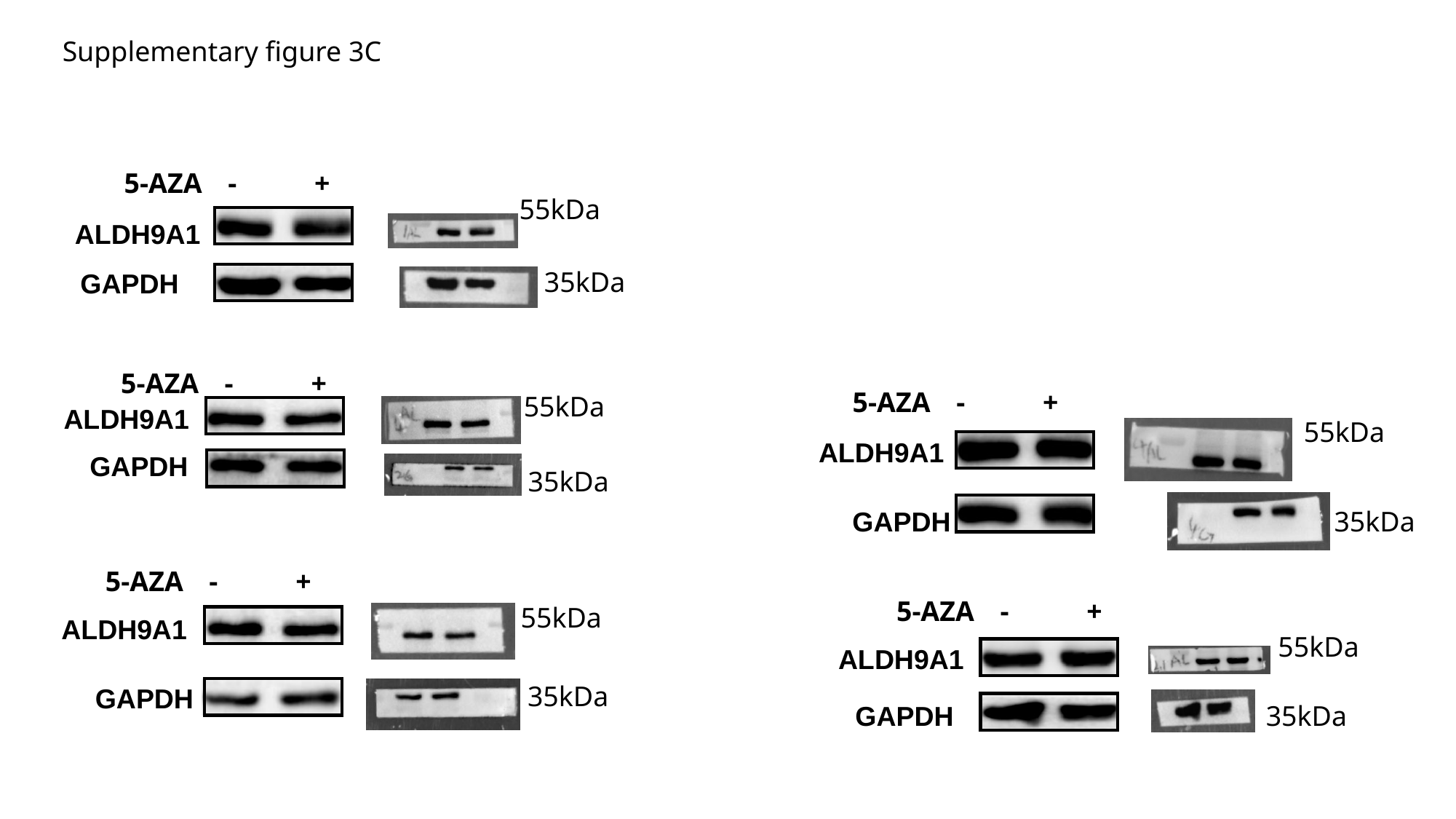

Supplementary figure 3C
5-AZA
- +
55kDa
ALDH9A1
35kDa
GAPDH
5-AZA
- +
5-AZA
- +
55kDa
ALDH9A1
55kDa
ALDH9A1
GAPDH
35kDa
GAPDH
35kDa
5-AZA
- +
5-AZA
- +
55kDa
ALDH9A1
55kDa
ALDH9A1
35kDa
GAPDH
GAPDH
35kDa

## Slide 18
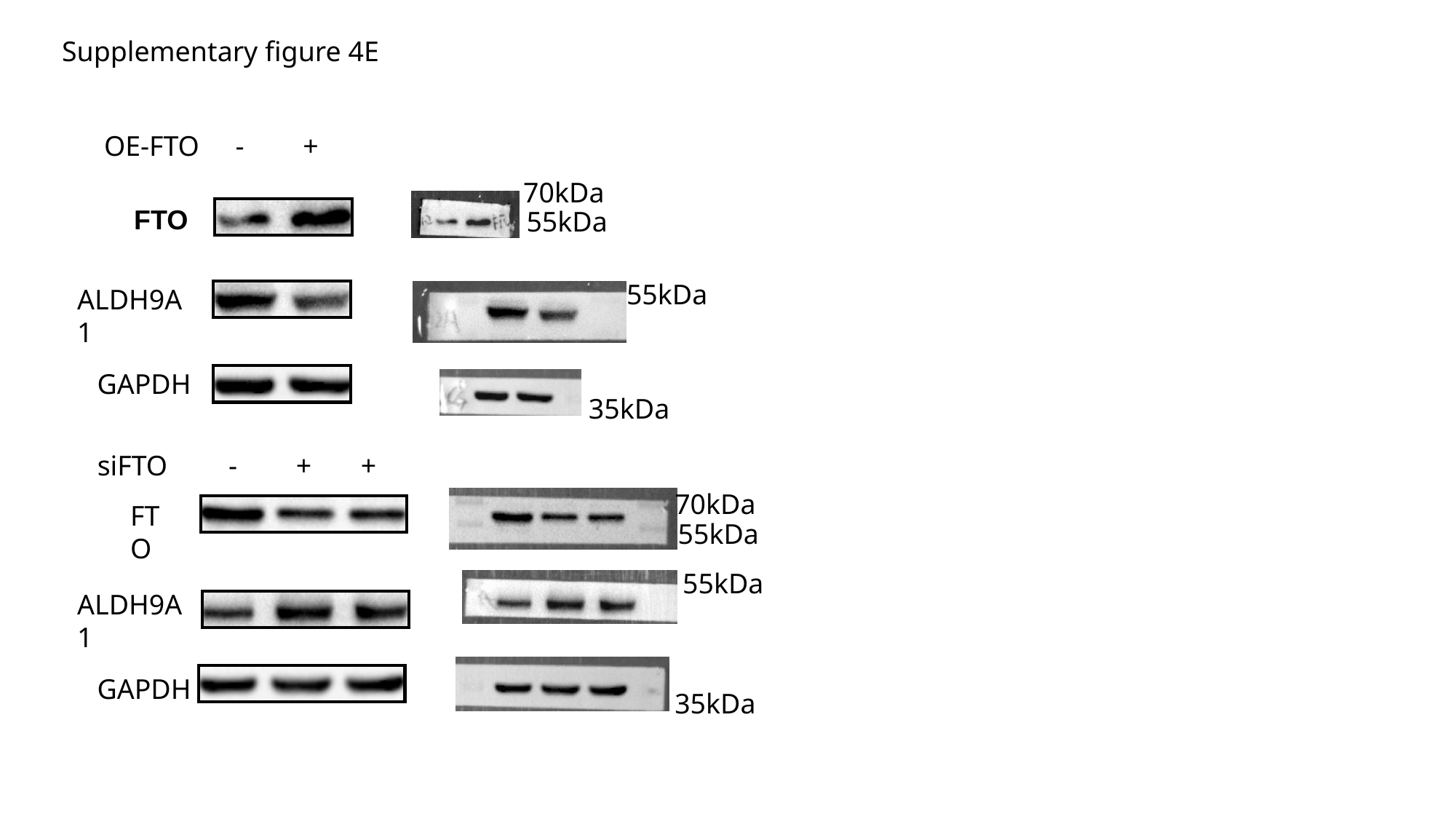

Supplementary figure 4E
| OE-FTO | - | + | |
| --- | --- | --- | --- |
70kDa
FTO
55kDa
55kDa
ALDH9A1
GAPDH
35kDa
| siFTO | - | + | + |
| --- | --- | --- | --- |
70kDa
FTO
55kDa
55kDa
ALDH9A1
GAPDH
35kDa

## Slide 19
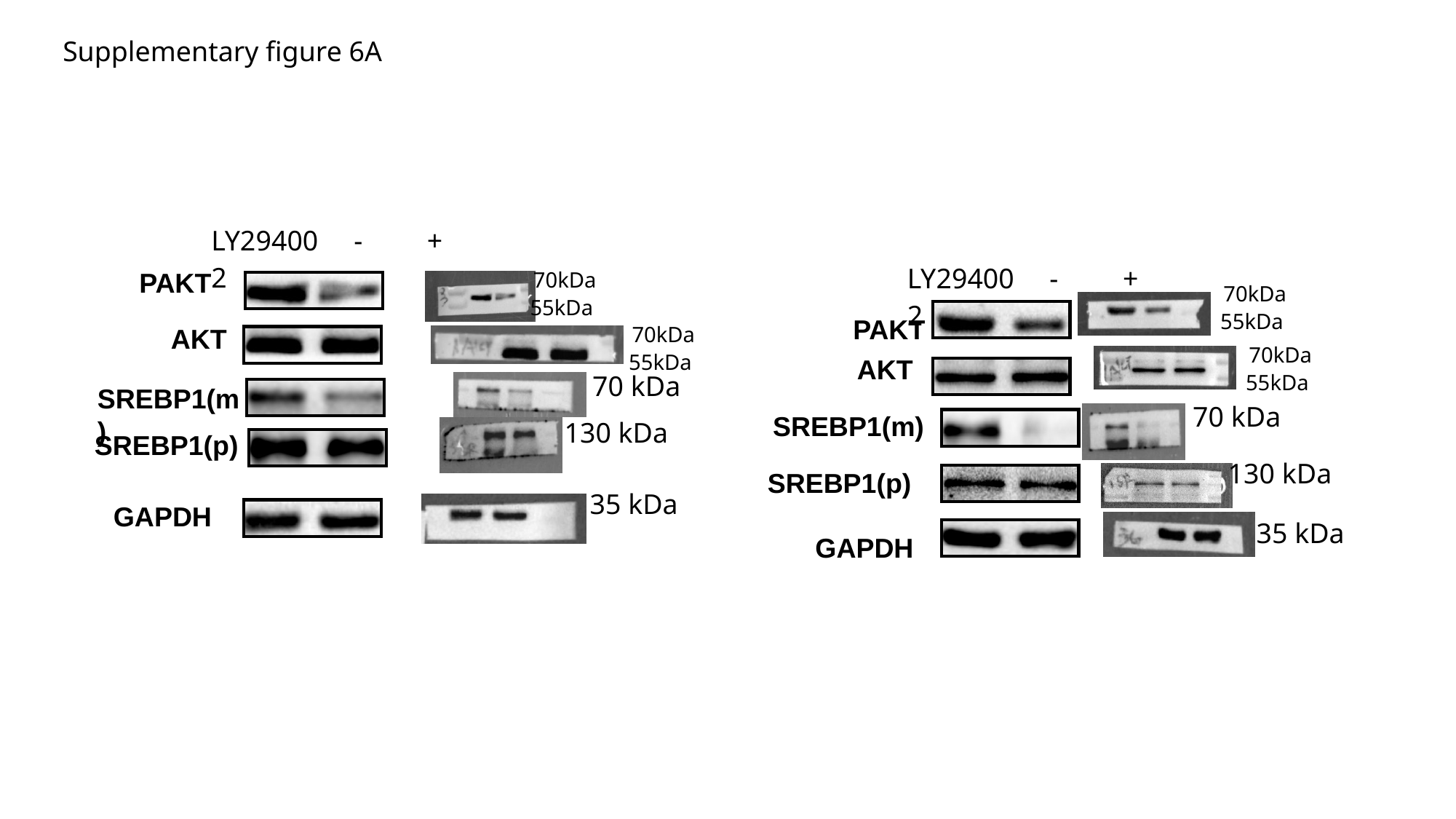

Supplementary figure 6A
| LY294002 | - | + | | | |
| --- | --- | --- | --- | --- | --- |
| LY294002 | - | + | | | |
| --- | --- | --- | --- | --- | --- |
PAKT
70kDa
70kDa
55kDa
55kDa
PAKT
70kDa
AKT
70kDa
55kDa
AKT
70 kDa
55kDa
SREBP1(m)
70 kDa
SREBP1(m)
130 kDa
SREBP1(p)
130 kDa
SREBP1(p)
35 kDa
GAPDH
35 kDa
GAPDH

## Slide 20
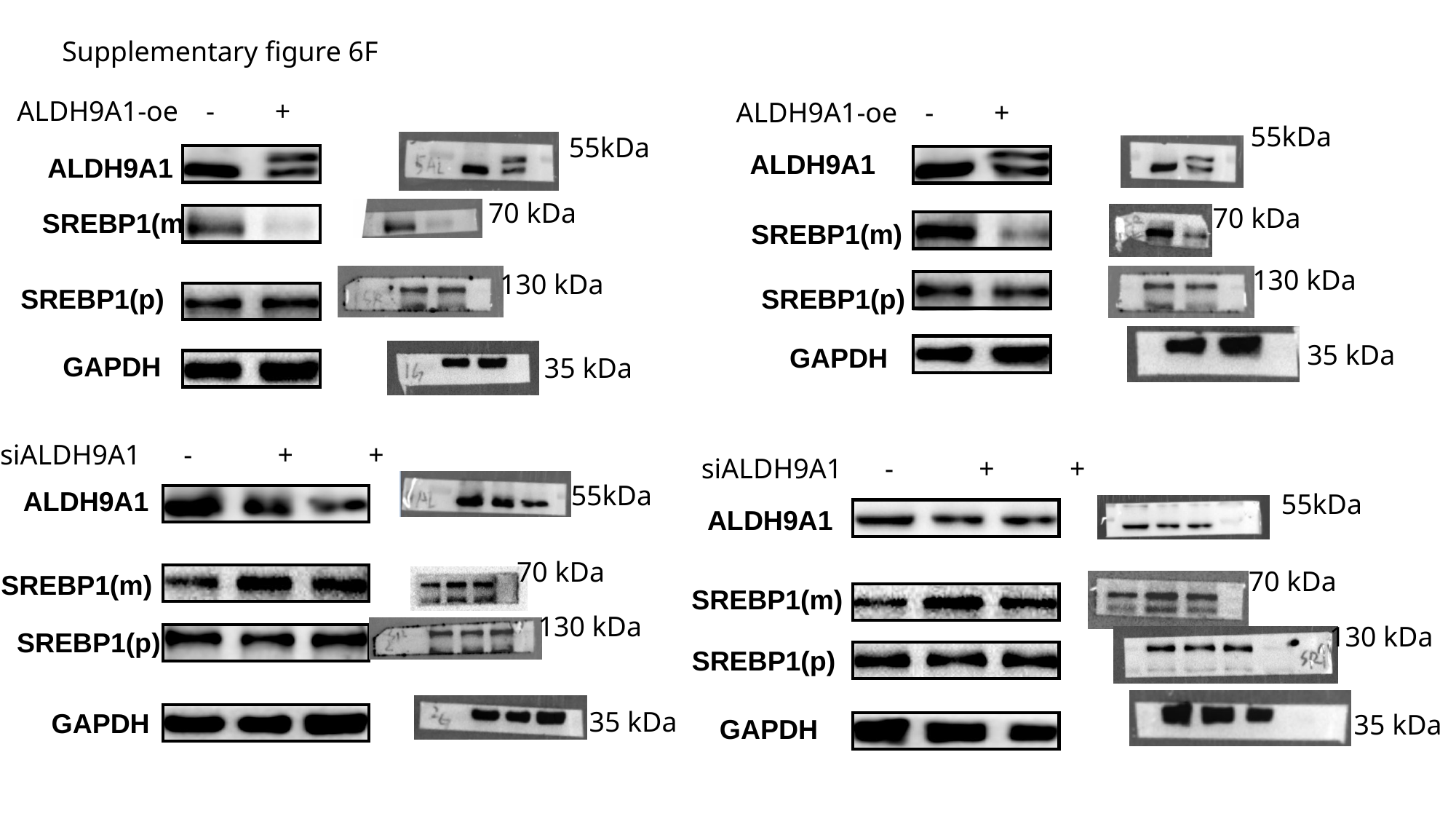

Supplementary figure 6F
| ALDH9A1-oe | - | + | | | |
| --- | --- | --- | --- | --- | --- |
| ALDH9A1-oe | - | + | | | |
| --- | --- | --- | --- | --- | --- |
55kDa
55kDa
ALDH9A1
ALDH9A1
70 kDa
70 kDa
SREBP1(m)
SREBP1(m)
130 kDa
130 kDa
SREBP1(p)
SREBP1(p)
35 kDa
GAPDH
GAPDH
35 kDa
| siALDH9A1 | - | + | + | | |
| --- | --- | --- | --- | --- | --- |
| siALDH9A1 | - | + | + | | |
| --- | --- | --- | --- | --- | --- |
55kDa
ALDH9A1
55kDa
ALDH9A1
70 kDa
70 kDa
SREBP1(m)
SREBP1(m)
130 kDa
130 kDa
SREBP1(p)
SREBP1(p)
35 kDa
GAPDH
35 kDa
GAPDH

## Slide 21
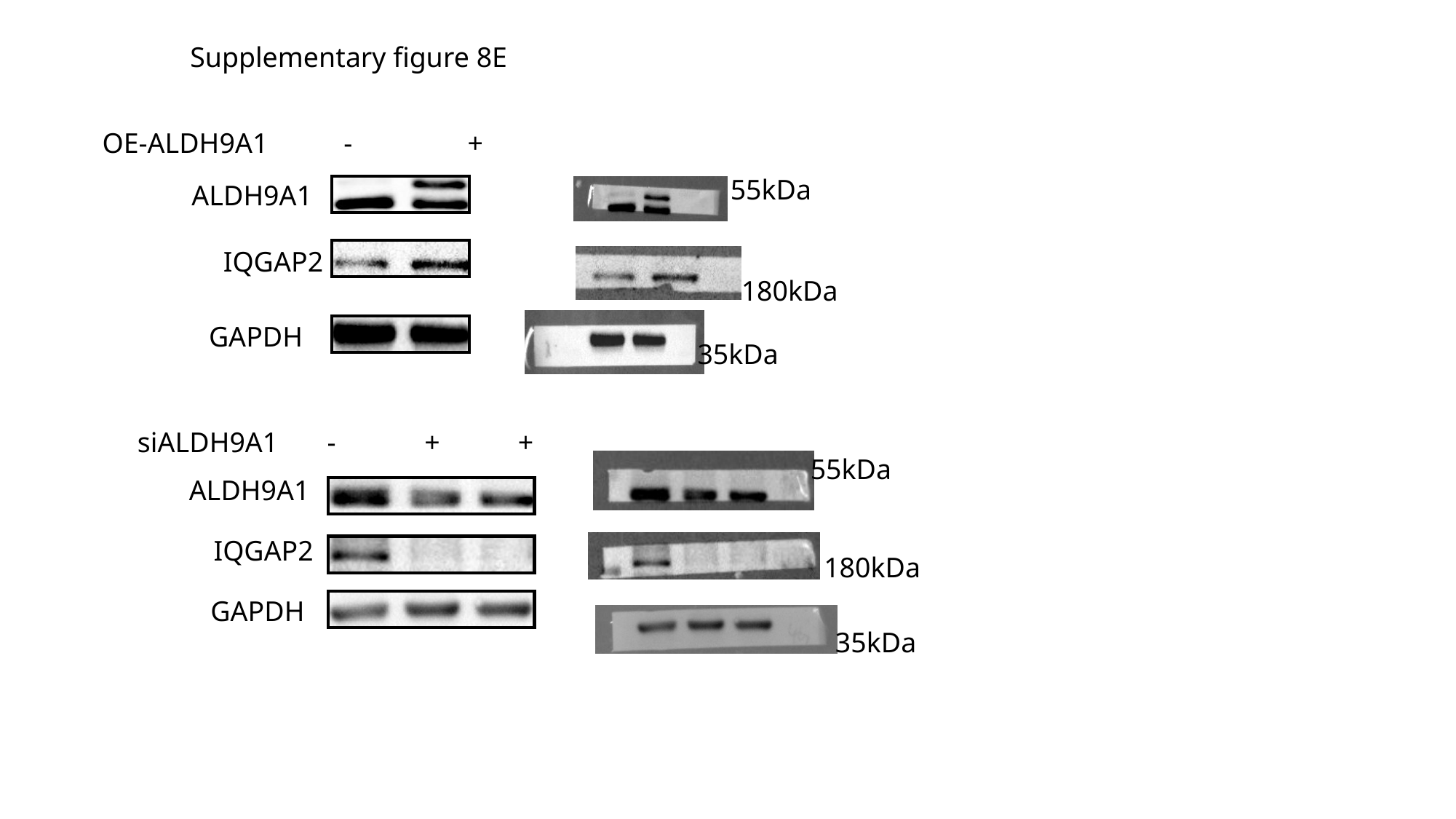

Supplementary figure 8E
| OE-ALDH9A1 | - | + | |
| --- | --- | --- | --- |
55kDa
ALDH9A1
IQGAP2
180kDa
GAPDH
35kDa
| siALDH9A1 | - | + | + |
| --- | --- | --- | --- |
55kDa
ALDH9A1
IQGAP2
180kDa
GAPDH
35kDa

## Slide 22
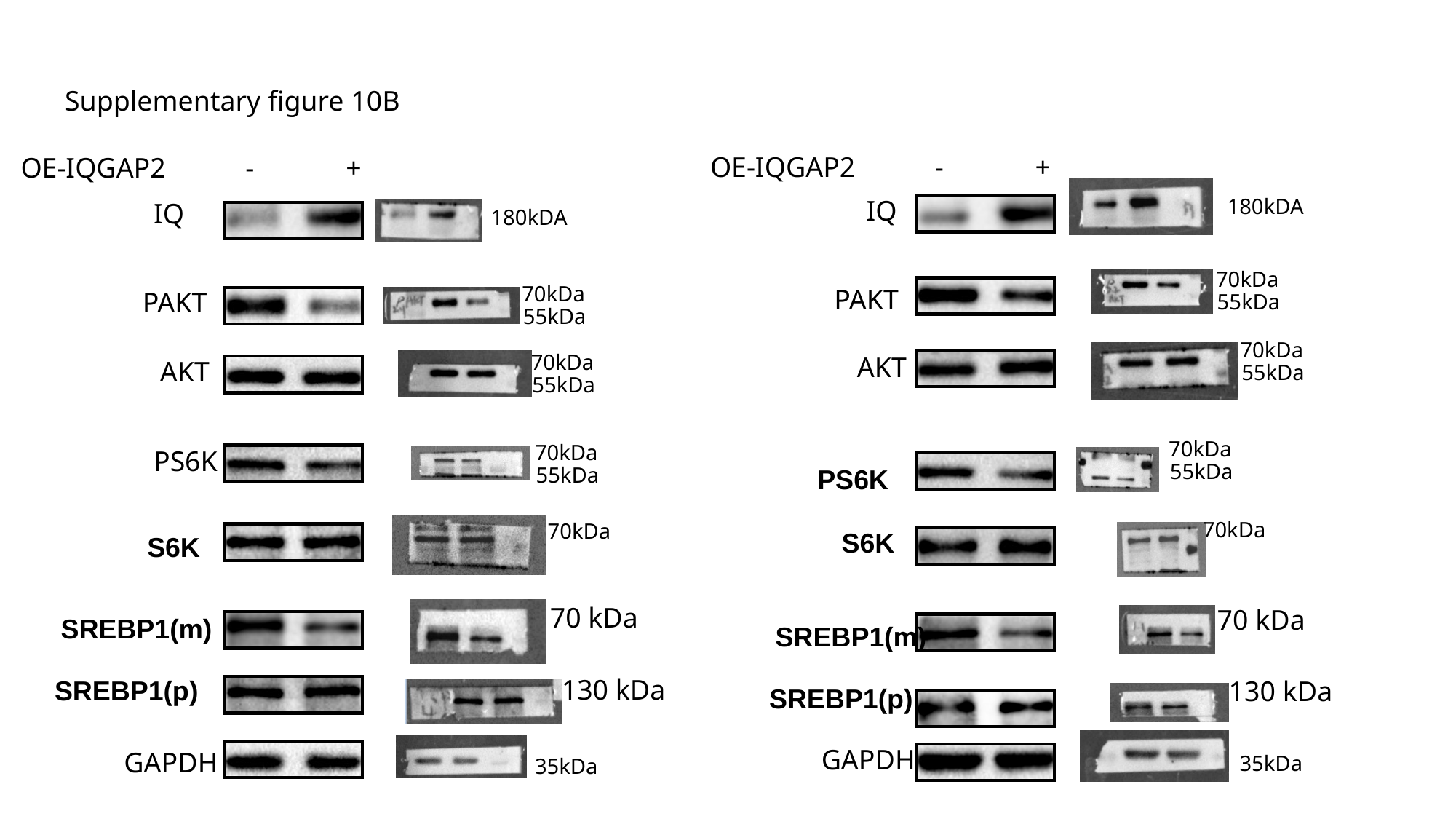

Supplementary figure 10B
| OE-IQGAP2 | - | + | |
| --- | --- | --- | --- |
| OE-IQGAP2 | - | + | |
| --- | --- | --- | --- |
IQ
180kDA
IQ
180kDA
70kDa
70kDa
PAKT
PAKT
55kDa
55kDa
70kDa
70kDa
AKT
AKT
55kDa
55kDa
70kDa
70kDa
PS6K
55kDa
55kDa
PS6K
70kDa
70kDa
S6K
S6K
70 kDa
70 kDa
SREBP1(m)
SREBP1(m)
130 kDa
SREBP1(p)
130 kDa
SREBP1(p)
GAPDH
GAPDH
35kDa
35kDa

## Slide 23
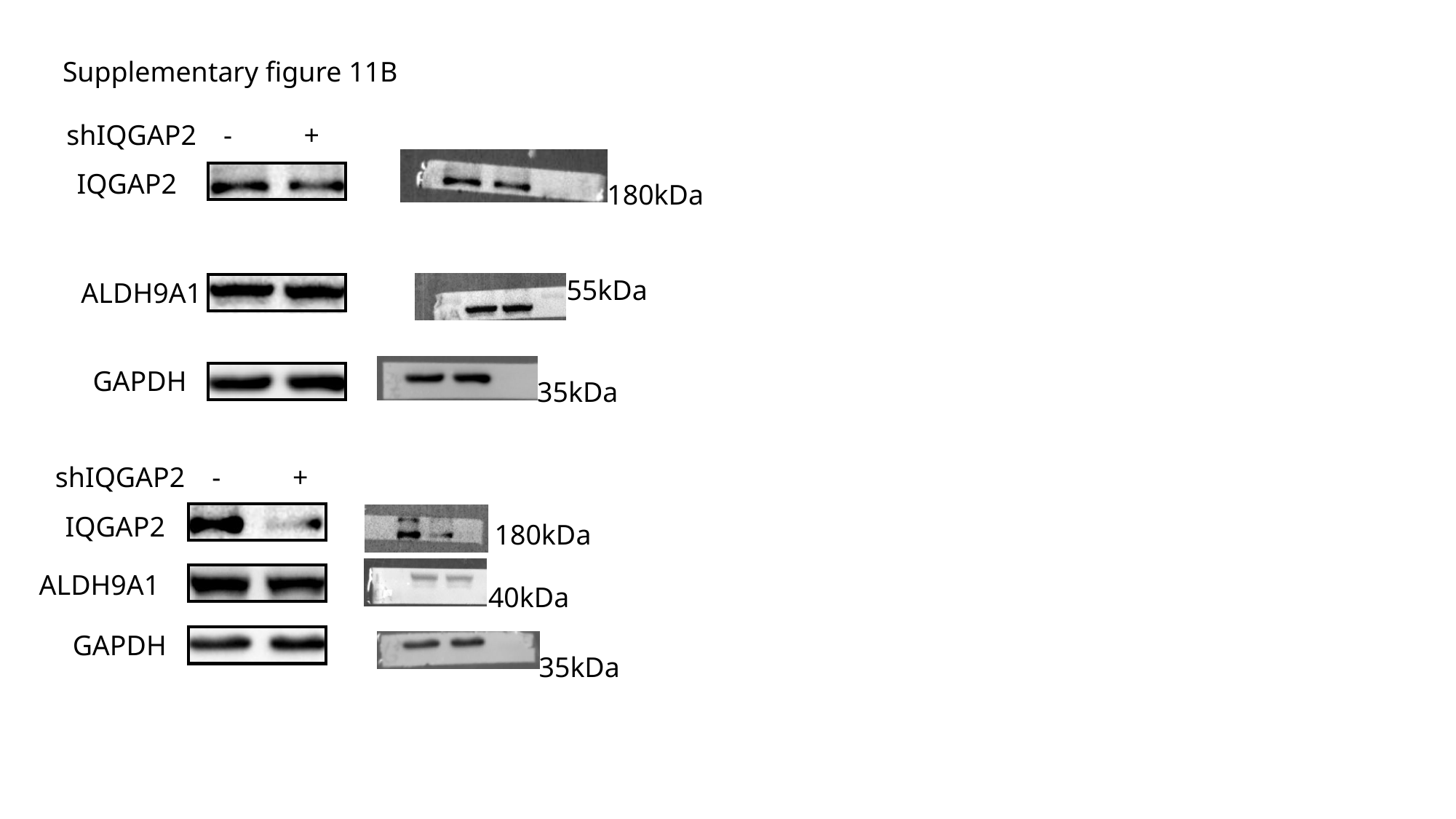

Supplementary figure 11B
| shIQGAP2 | - | + | |
| --- | --- | --- | --- |
IQGAP2
180kDa
55kDa
ALDH9A1
GAPDH
35kDa
| shIQGAP2 | - | + | |
| --- | --- | --- | --- |
IQGAP2
180kDa
ALDH9A1
40kDa
GAPDH
35kDa

## Slide 24
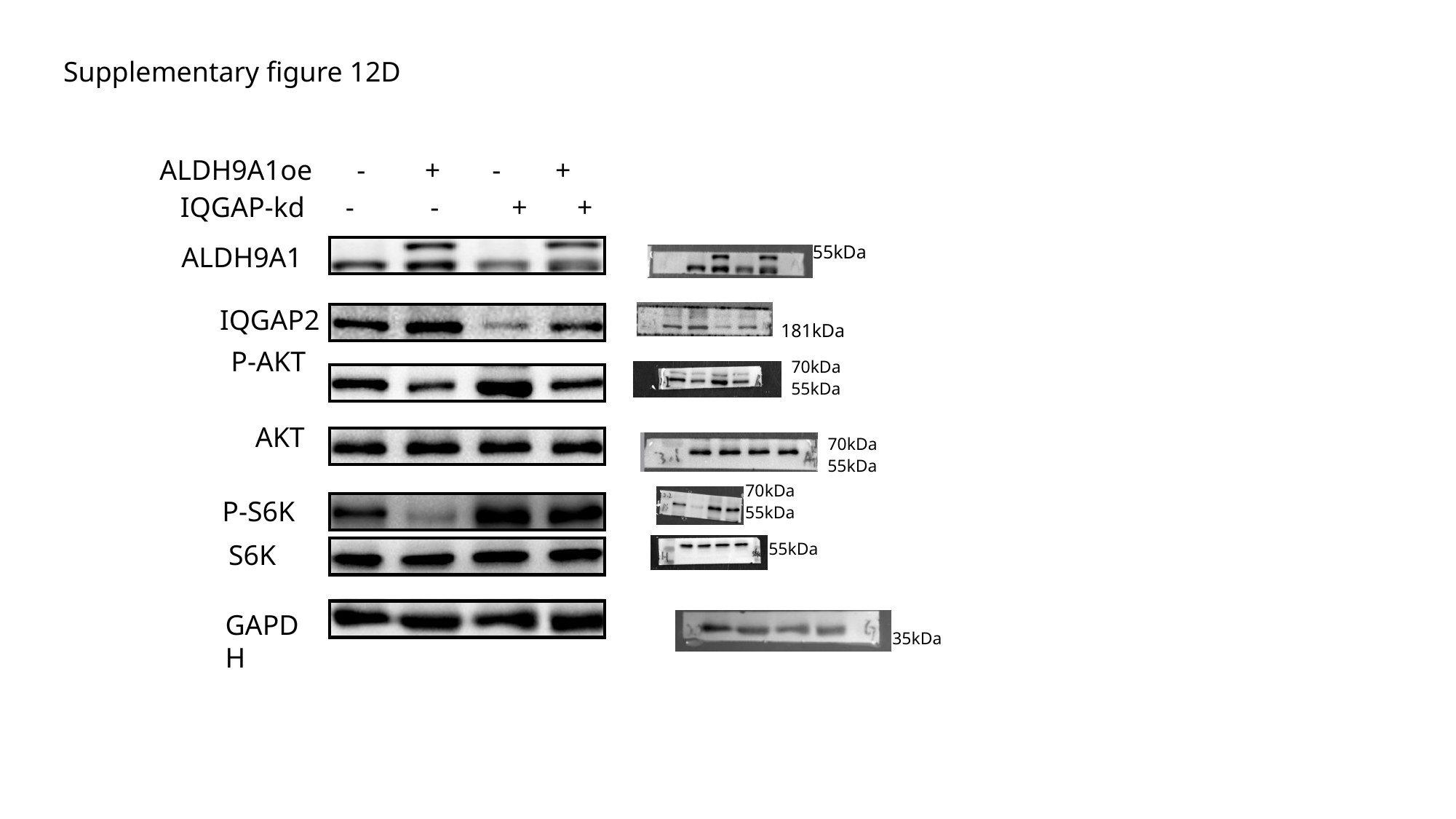

Supplementary figure 12D
| ALDH9A1oe | - | + | - | + | |
| --- | --- | --- | --- | --- | --- |
| IQGAP-kd | - | - | + | + | |
| --- | --- | --- | --- | --- | --- |
ALDH9A1
55kDa
IQGAP2
181kDa
P-AKT
70kDa
55kDa
AKT
70kDa
55kDa
70kDa
P-S6K
55kDa
S6K
55kDa
GAPDH
35kDa

## Slide 25
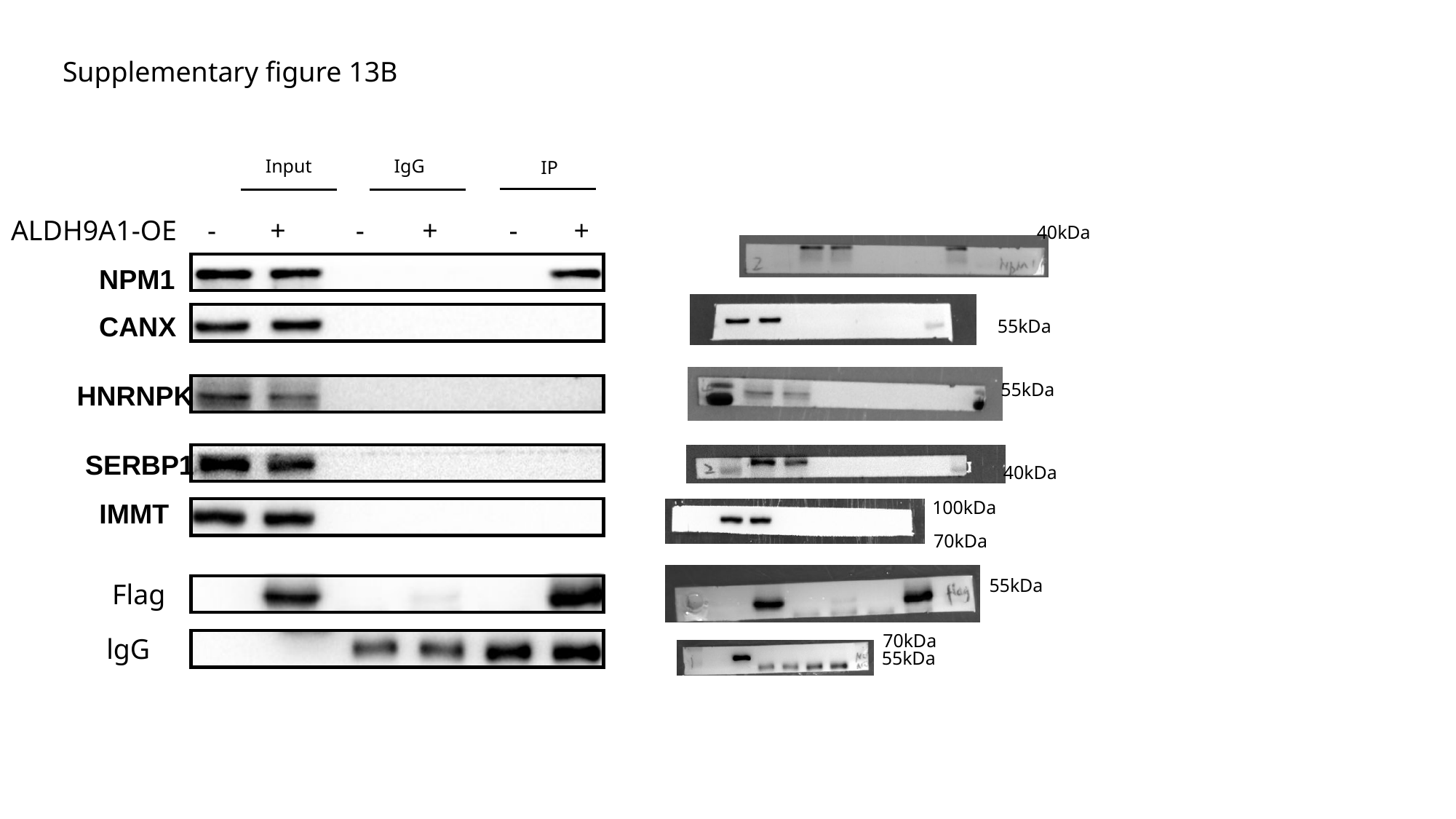

Supplementary figure 13B
Input
IgG
IP
| ALDH9A1-OE | - | + | - | + | - | + |
| --- | --- | --- | --- | --- | --- | --- |
40kDa
NPM1
CANX
55kDa
55kDa
HNRNPK
SERBP1
40kDa
100kDa
IMMT
70kDa
55kDa
Flag
70kDa
lgG
55kDa

## Slide 26
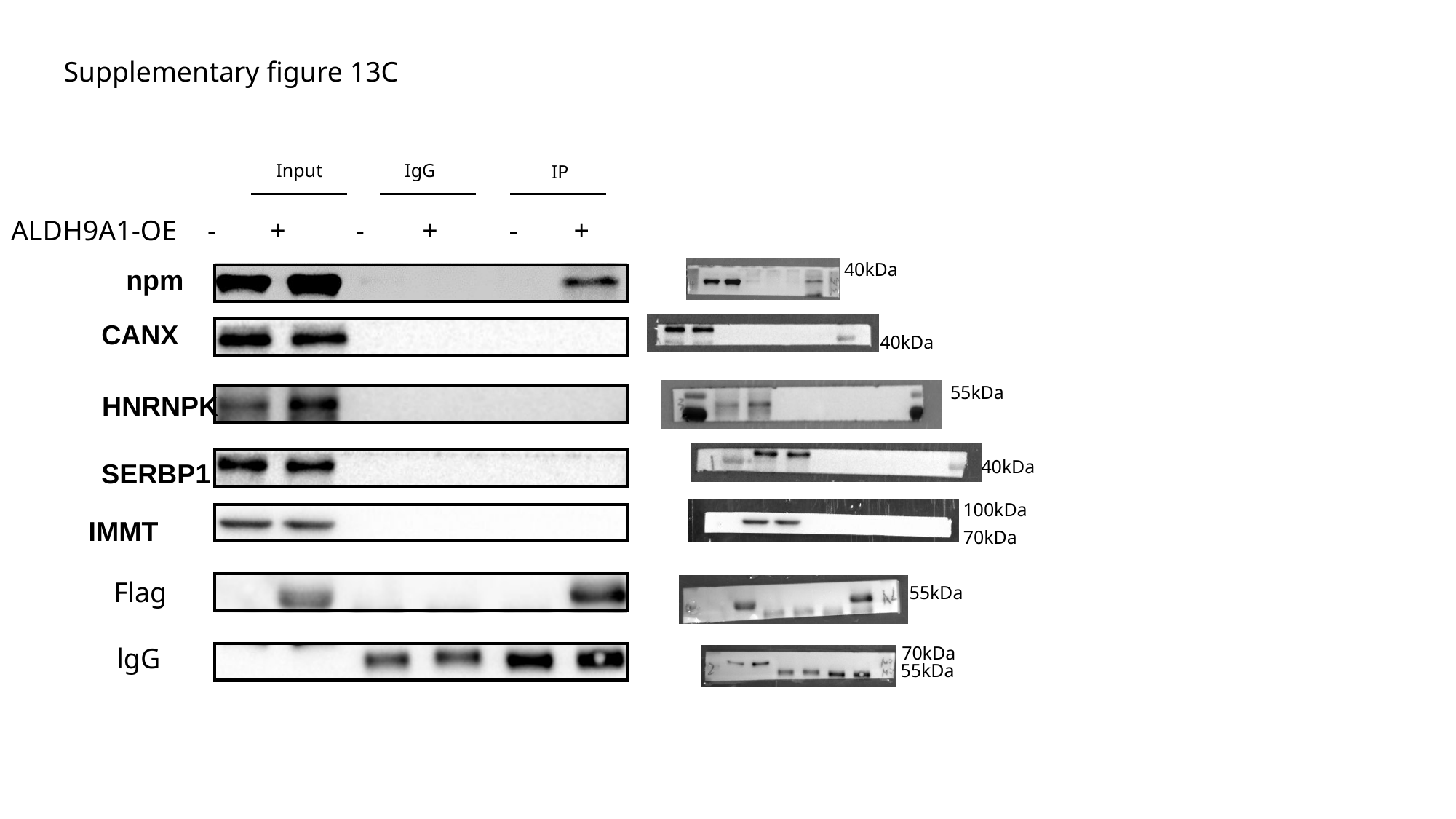

Supplementary figure 13C
Input
IgG
IP
| ALDH9A1-OE | - | + | - | + | - | + |
| --- | --- | --- | --- | --- | --- | --- |
40kDa
npm
CANX
40kDa
55kDa
HNRNPK
40kDa
SERBP1
100kDa
IMMT
70kDa
Flag
55kDa
70kDa
lgG
55kDa

## Slide 27
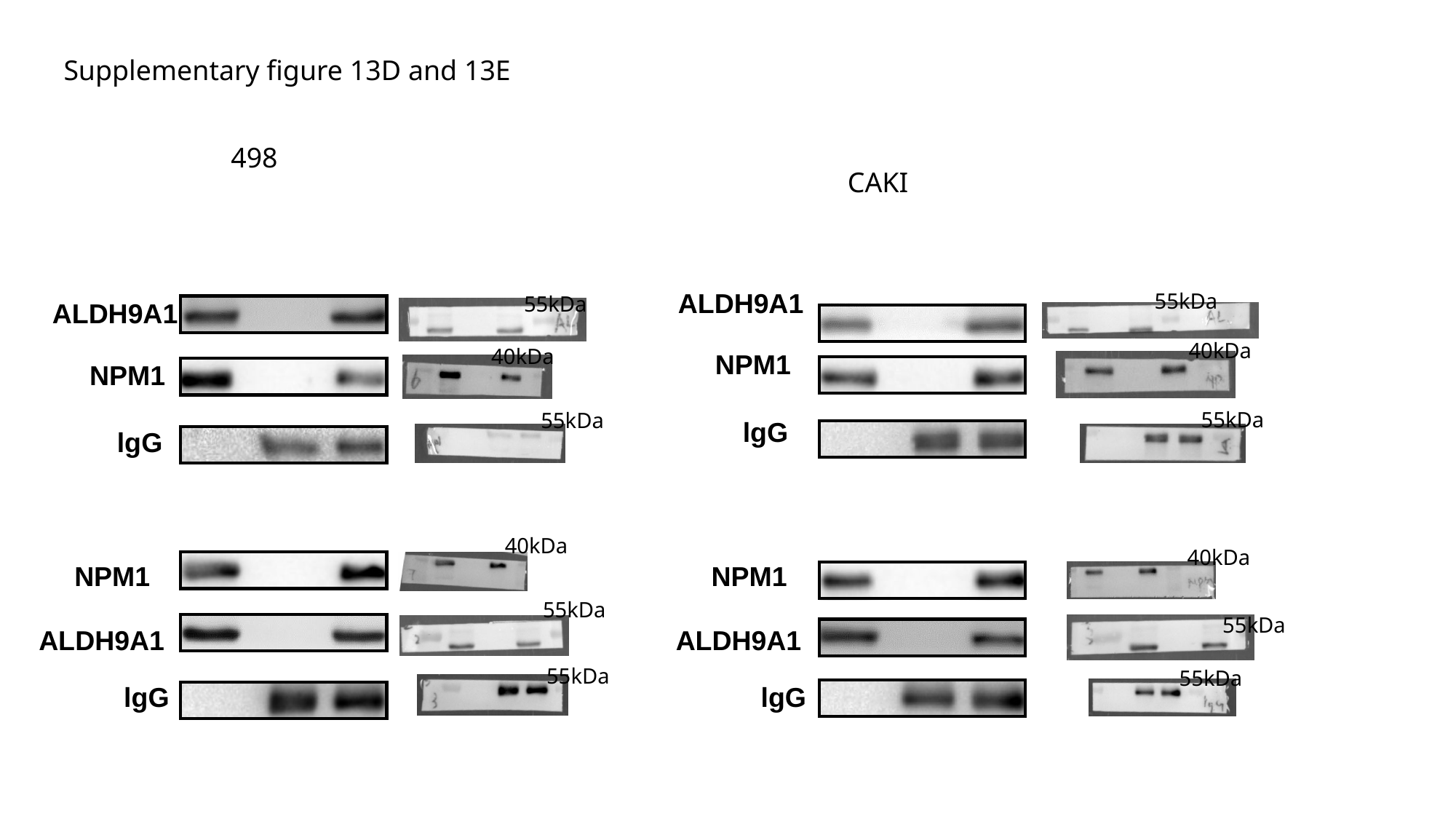

Supplementary figure 13D and 13E
498
CAKI
ALDH9A1
55kDa
55kDa
ALDH9A1
40kDa
40kDa
NPM1
NPM1
55kDa
55kDa
lgG
lgG
40kDa
40kDa
NPM1
NPM1
55kDa
55kDa
ALDH9A1
ALDH9A1
55kDa
55kDa
lgG
lgG

## Slide 28
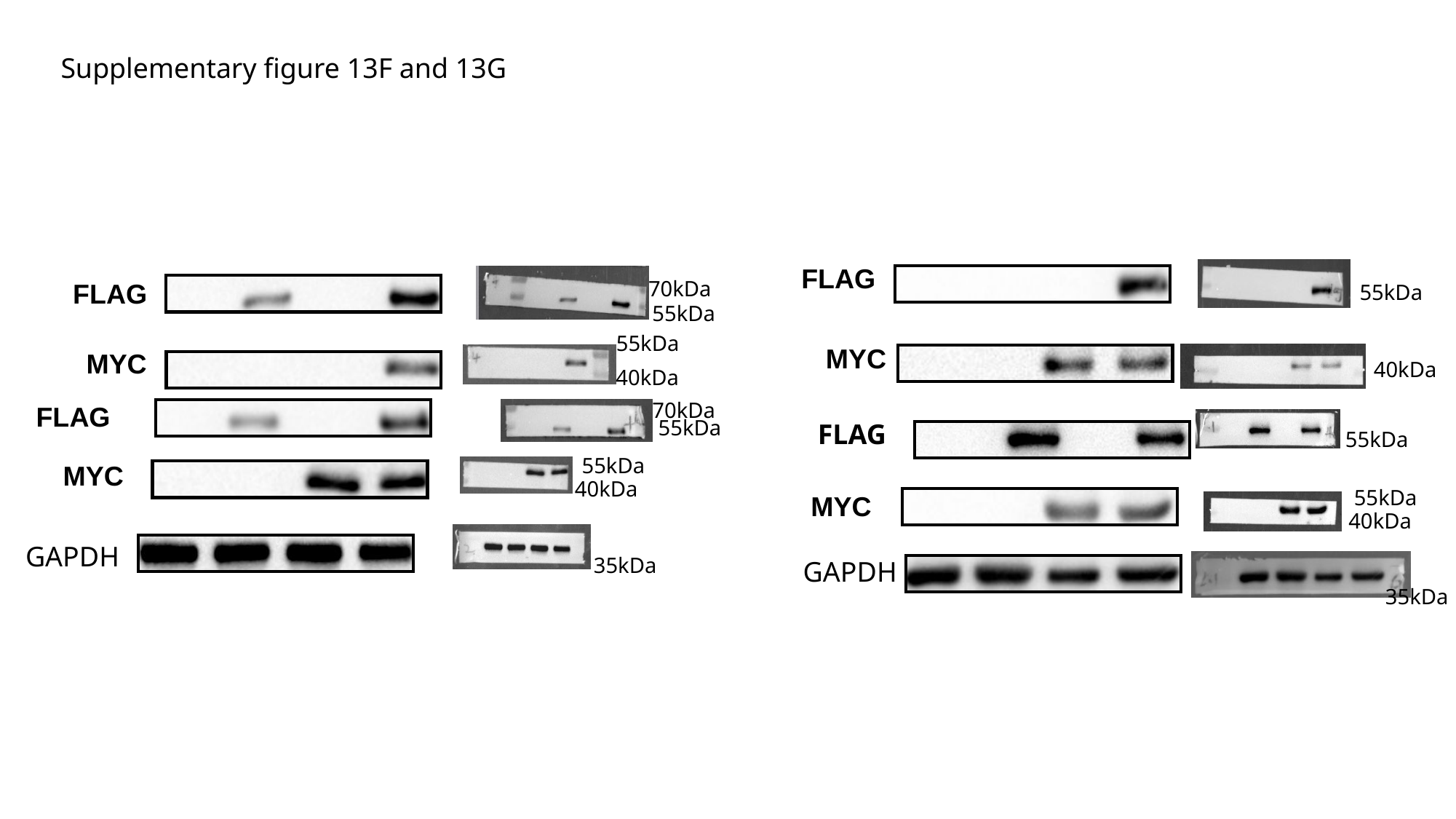

Supplementary figure 13F and 13G
FLAG
70kDa
FLAG
55kDa
55kDa
55kDa
MYC
MYC
40kDa
40kDa
70kDa
FLAG
55kDa
FLAG
55kDa
55kDa
MYC
40kDa
55kDa
MYC
40kDa
GAPDH
35kDa
GAPDH
35kDa

## Slide 29
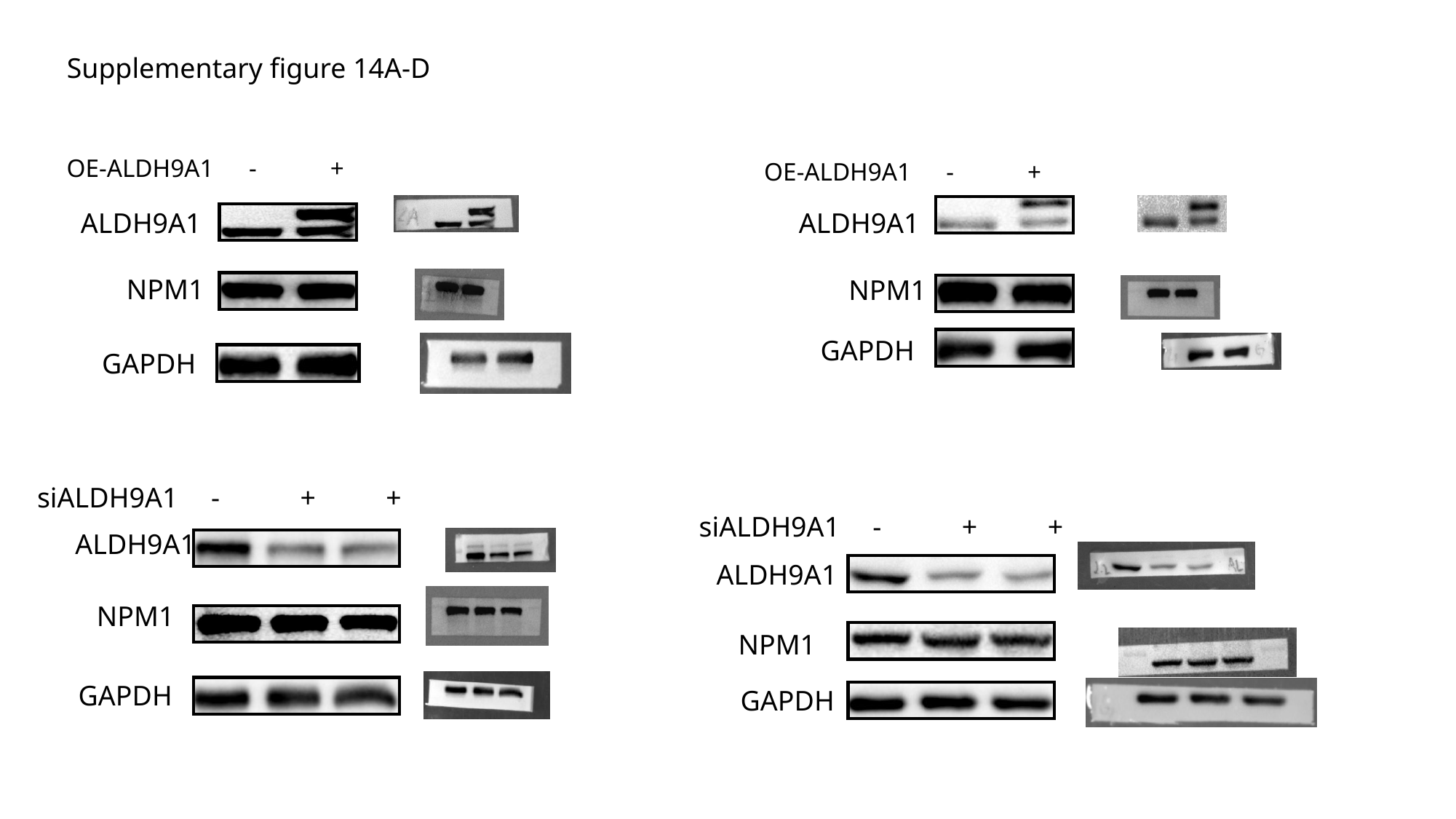

Supplementary figure 14A-D
| OE-ALDH9A1 | - | + | |
| --- | --- | --- | --- |
| OE-ALDH9A1 | - | + | |
| --- | --- | --- | --- |
ALDH9A1
ALDH9A1
NPM1
NPM1
GAPDH
GAPDH
| siALDH9A1 | - | + | + |
| --- | --- | --- | --- |
| siALDH9A1 | - | + | + |
| --- | --- | --- | --- |
ALDH9A1
ALDH9A1
NPM1
NPM1
GAPDH
GAPDH

## Slide 30
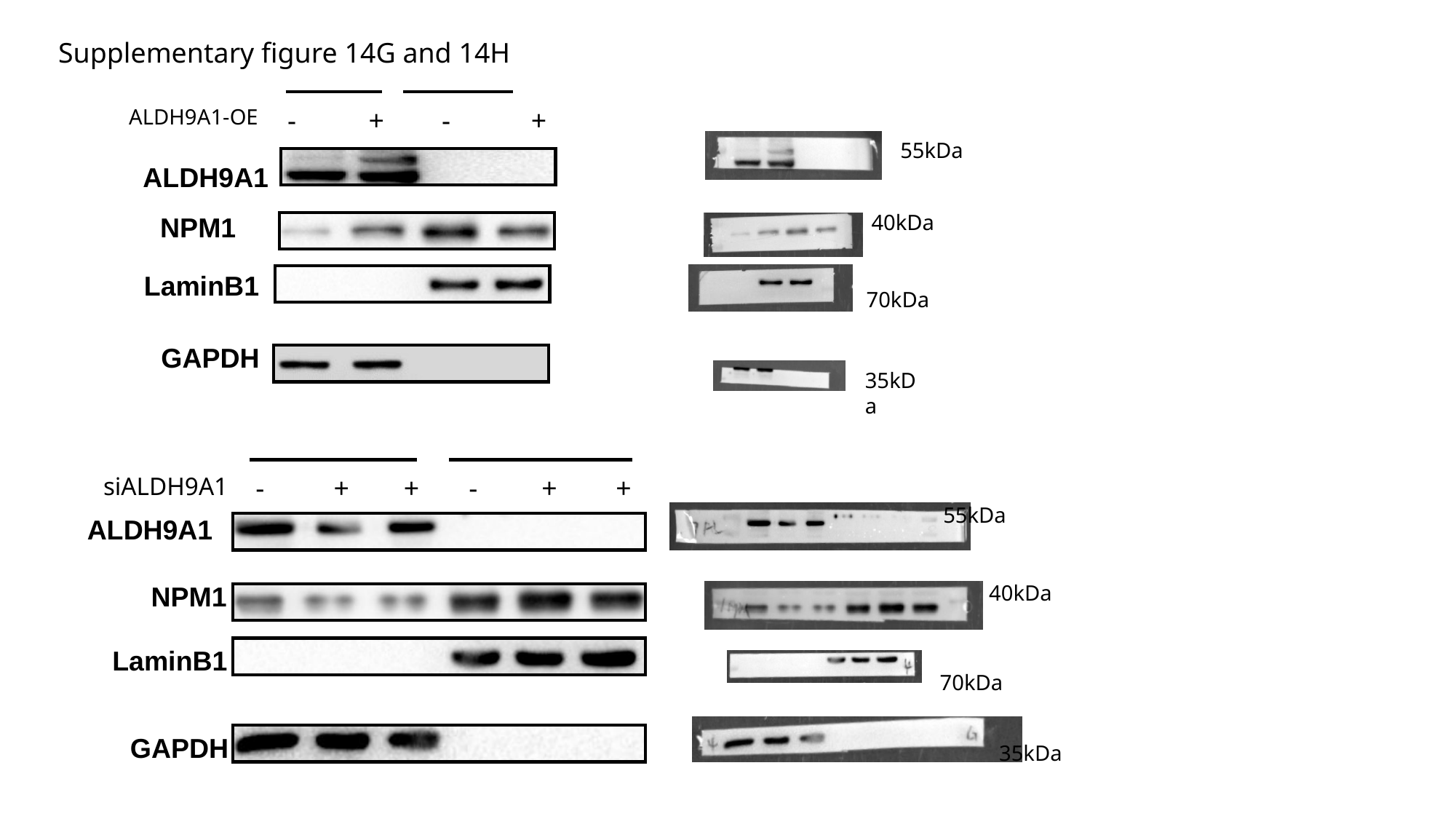

Supplementary figure 14G and 14H
| ALDH9A1-OE | - | + | - | + | | | | |
| --- | --- | --- | --- | --- | --- | --- | --- | --- |
55kDa
ALDH9A1
40kDa
NPM1
LaminB1
70kDa
GAPDH
35kDa
| siALDH9A1 | - | + | + | - | + | + | | |
| --- | --- | --- | --- | --- | --- | --- | --- | --- |
55kDa
ALDH9A1
NPM1
40kDa
LaminB1
70kDa
GAPDH
35kDa

## Slide 31
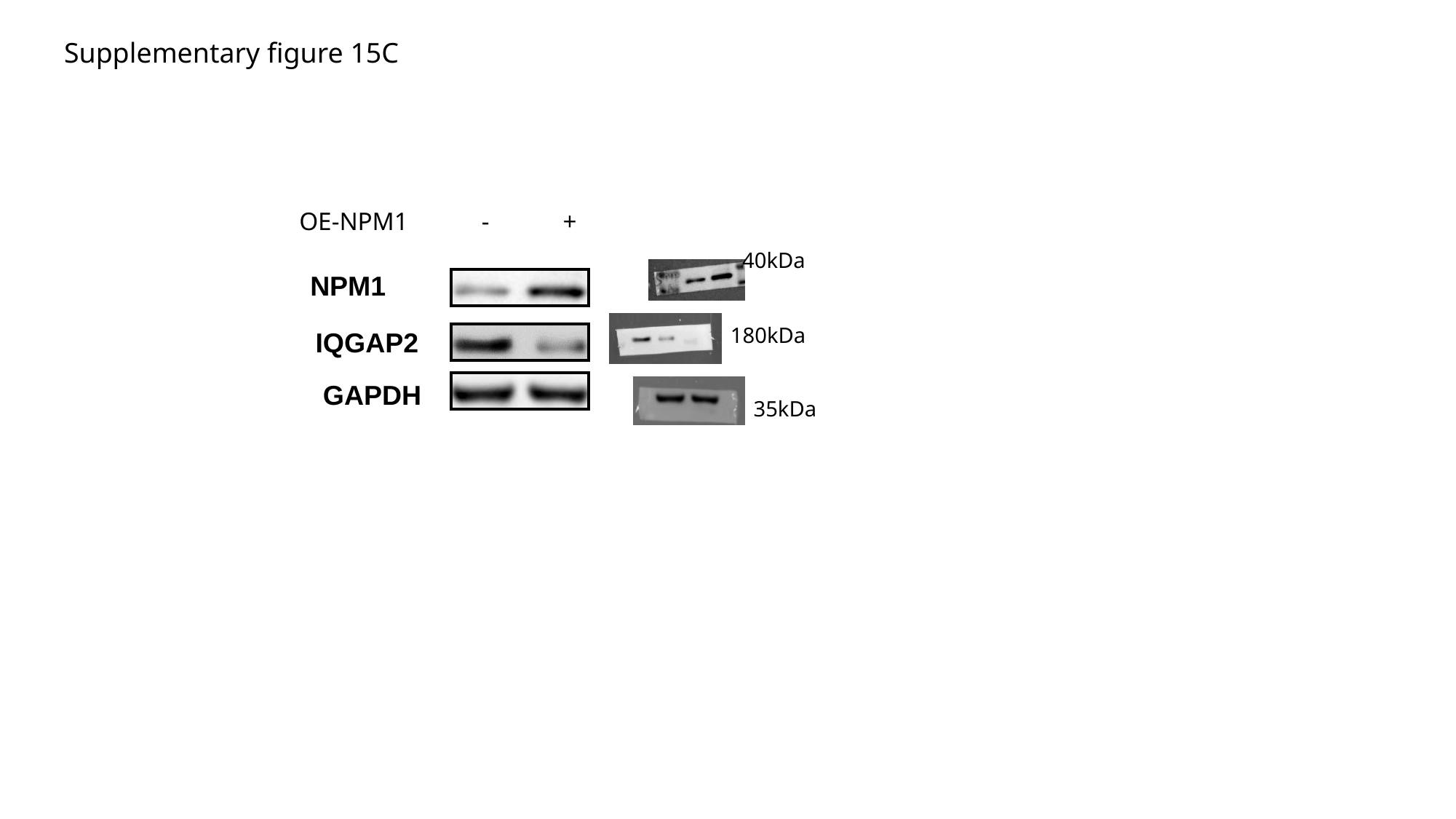

Supplementary figure 15C
| OE-NPM1 | - | + | |
| --- | --- | --- | --- |
40kDa
NPM1
180kDa
IQGAP2
GAPDH
35kDa

## Slide 32
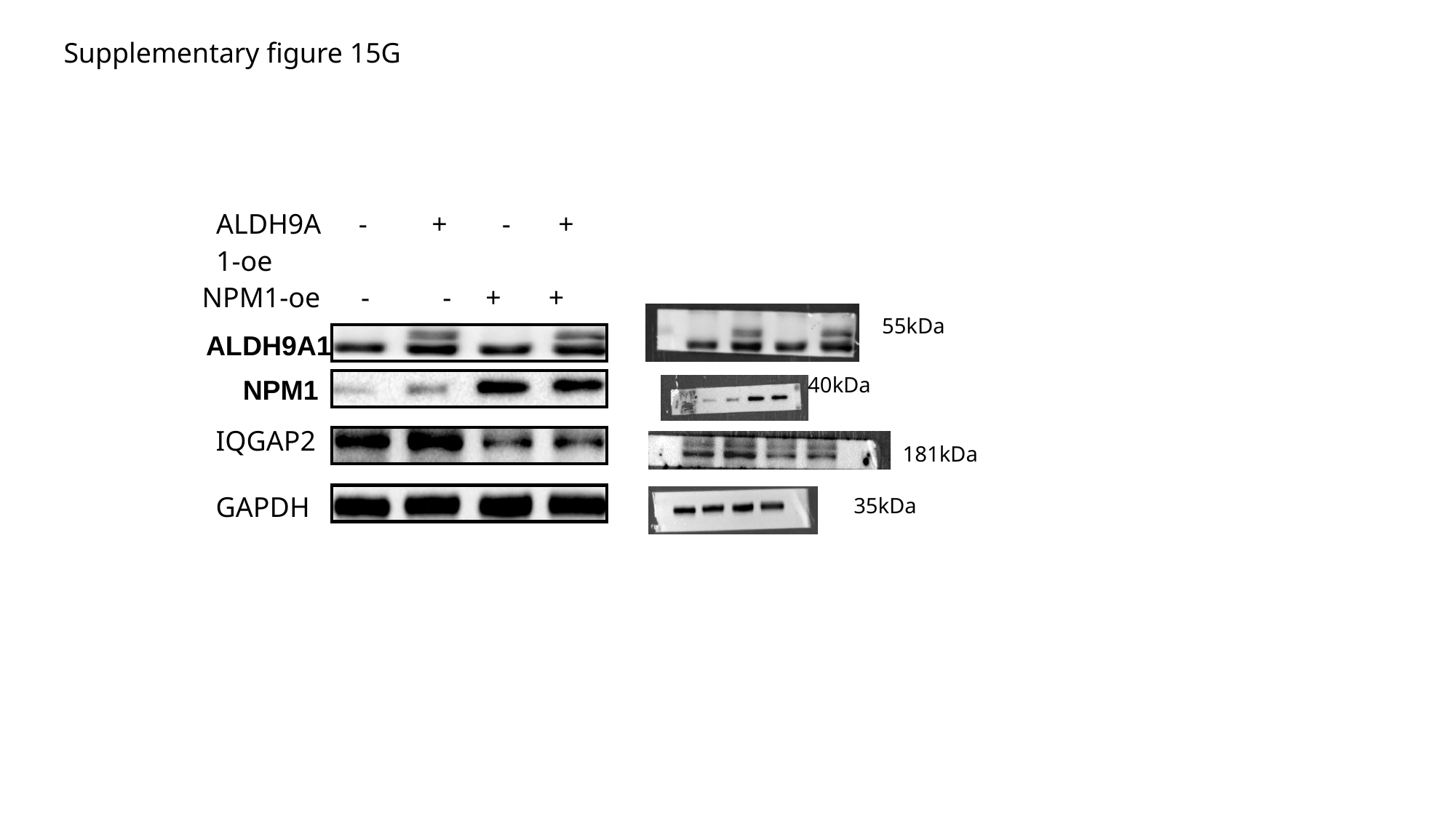

Supplementary figure 15G
| ALDH9A1-oe | - | + | - | + | |
| --- | --- | --- | --- | --- | --- |
| NPM1-oe | - | - | + | + | |
| --- | --- | --- | --- | --- | --- |
55kDa
ALDH9A1
40kDa
NPM1
IQGAP2
181kDa
GAPDH
35kDa
